# Streamlined Synthesis and Immunomodulatory Effects of Ulvan Oligosaccharides from Marine Green Algae

**DOI:** 10.1021/jacs.5c09759

**Published:** 2025-08-28

**Authors:** Junpei Shimabukuro, Ci-Hua Shen, Tsung-Kai Hsueh, Ting-Wen Chen, Yin-Chen Tseng, Veeranjaneyulu Gannedi, Jasper S. Dumalaog, Cheng-Hsiu Chang, Ting-Jen Rachel Cheng, Chung-Shan Yu, Shang-Cheng Hung

**Affiliations:** † 38017 Genomics Research Center, Academia Sinica, 128, Section 2, Academia Road, Taipei 11529, Taiwan; ‡ Institute of Nuclear Engineering Science, 34881 National Tsing Hua University, 101, Section 2, Kuang-Fu Road, Hsinchu 30013, Taiwan; § Department of Chemistry, National Tsing Hua University, 101, Section 2, Kuang-Fu Road, Hsinchu 30013, Taiwan; ∥ Biomedical Engineering and Environmental Sciences, National Tsing Hua University, 101, Section 2, Kuang-Fu Road, Hsinchu 30013, Taiwan; ⊥ Department of Chemistry, National Cheng Kung University, 1, University Road, Tainan 70101, Taiwan; # Department of Applied Science, National Taitung University, 369, Section 2, University Road, Taitung 95092, Taiwan

## Abstract

Green algae, commonly used as a source of food nutrients, contain ulvan, which is a water-soluble sulfated heteropolysaccharide isolated from *Ulva*. Ulvan exhibits a range of biological properties, including antitumor, antiviral, anticoagulant, and immunomodulatory activities. Its sugar backbone consists of 1→4-linked disaccharide repeating units, which are categorized as ulvanobiuronic acids (types A_3′S_ and B_3′S_) and ulvanobioses (types U_3′S_ and U_2S3′S_). Despite its promising therapeutic potential, the detailed structure–activity relationship of ulvan remains unclear. In this study, an efficient strategy was developed, utilizing orthogonally protected α1→4-linked l-Rha-d-Glc, l-Rha-l-Ido, and l-Rha-d-Xyl derivatives as disaccharide building blocks to synthesize di-, tetra-, hexa-, and octasaccharides of types A_3′S_, B_3′S_, and U_3′S_. The immunomodulatory effects of these compounds were evaluated through cytokine induction experiments. The results revealed that only the B_3′S_ and U_3′S_ octasaccharides had a significant influence on the gene expression of IL-1β, IL-6, IL-10, GM-CSF, and G-CSF. This chemical glycobiology approach offers valuable insights into the biomedical applications of marine ulvan oligosaccharides.

## Graphical abstract

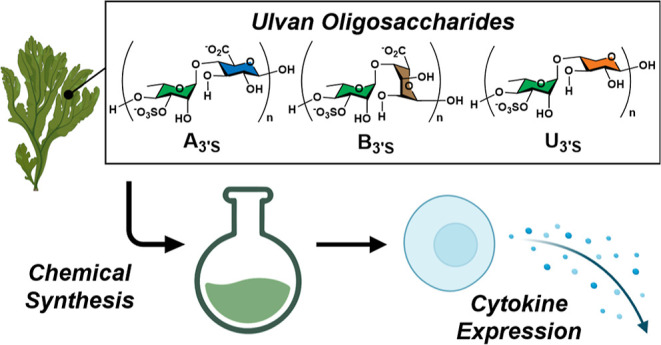

## Introduction

Polysulfated polysaccharides are crucial bioactive macromolecules found in mammals^1–4^ and marine seaweeds.^5,6^ Glycosaminoglycans (GAGs), such as heparan sulfate, chondroitin sulfate, dermatan sulfate, and keratan sulfate, are linear polysulfated polysaccharides located on the cell surface. These molecules play vital roles in numerous physiological processes, including viral and bacterial infections, tumor progression and angiogenesis, inflammation, the immune response, and neurodegenerative diseases. Ulvan, a linear sulfated polysaccharide isolated from the cell walls of marine green algae from *Ulva* and *Enteromorpha,* ^7,8^ shares structural similarities with GAGs. As a result, ulvan has gained considerable attention for its antitumor, antiviral, anticoagulant, and immunomodulatory properties, and has been widely used in tissue engineering, nutraceuticals, and biomaterial science.^9–15^ The sugar backbone of ulvan primarily consists of four monosaccharides: l-rhamnose (l-Rha), d-glucuronic acid (d-GlcA), l-iduronic acid (l-IdoA), and d-xylose (d-Xyl). These monosaccharides are linked in 1→4 disaccharide repeating units, known as ulvanobiuronic acids and ulvanobioses (Figure 1). Ulvanobiuronic acids include type A_3′S_ [→4)-α-l-Rha3S-(1→4)-β-d-GlcA-(1→] and type B_3′S_ [→4)-α-l-Rha3S-(1→4)-α-l-IdoA­(1→], while ulvanobioses include type U_3′S_ [→4)-α-l-Rha3S-(1→4)-β-d-Xyl-(1→] and type U_2S3′S_ [→4)-α-l-Rha3S-(1→4)-β-d-Xyl2S-(1→].^16^ All disaccharide repeats feature 3-*O*-sulfated l-rhamnose as a common sugar unit. However, due to the challenges in obtaining homogeneously pure and structurally well-defined saccharides from natural sources, further exploration of their physicochemical and biological properties is necessary.

**Figure 1:**
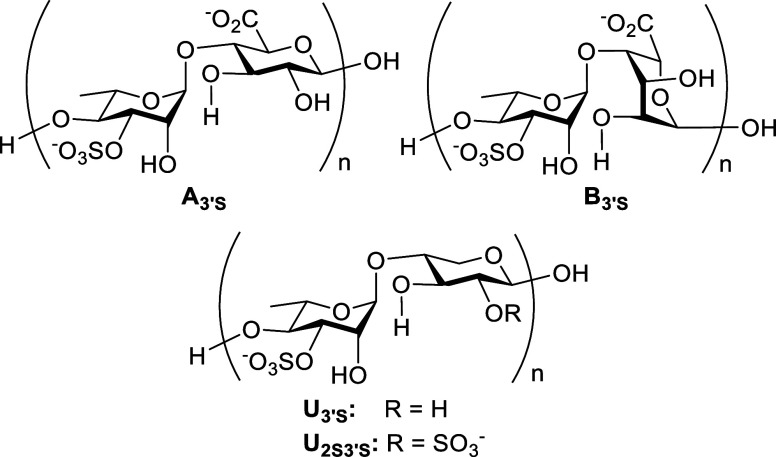
Disaccharide repeating units of ulvan.

Chemical synthesis provides a viable and precise approach for producing ulvan oligosaccharides with definite chain lengths and well-defined sulfation patterns, which are essential for an in-depth investigation of their structure–activity relationships (SARs) in biological systems. To date, only a synthetic ulvan type B-related tetrasaccharide, [α-l-IdoA-(1→4)-α-l-Rha3S-(1→4)-α-l-IdoA­(1→4)-α-l-Rha3S-1→*n*-C_5_H_10_NH_2_], has been reported,^17^ along with its effects on phagocytosis in macrophage RAW264.7 cells. In continuation of our ongoing interest in the chemistry and biology of sulfated GAGs and the development of ulvan-based oligosaccharides for drug discovery,^2,18–20^ we not only present a systematic approach for the synthesis of di-, tetra-, hexa-, and octasaccharides of ulvan types A_3′S_, B_3′S_, and U_3′S_ but also evaluate their immunomodulatory activities through experimental measurement of cytokine expression. In contrast to our research on GAGs, here we focus on marine ulvans, structurally distinct polysaccharides that lack amino sugars and originate from algae rather than animals, highlighting their unique biological roles and therapeutic potential.

The synthesis of various ulvan oligosaccharide series with such complexity presents significant challenges, including the installation of appropriate protecting groups on each monosaccharide building block, stereocontrol of all 1,2-*trans*-glycosidic linkages in the l-Rha, d-GlcA, l-IdoA, and d-Xyl units, elongation of sugar backbones, regioselective sulfation at the O3 position of all l-Rha units, multifunctional group transformations, and global deprotections. The retrosynthetic strategy for the target molecules **1–4** (A_3′S_ series), **5**–**8** (B_3′S_ series), and **9**–**12** (U_3′S_ series), as depicted in Scheme 1, addresses the key synthetic challenges, with pivotal disaccharide building blocks **13**, **14**, and **15** serving as key intermediates in the chain elongation of each respective series. In the synthesis of the target molecules, late-stage oxidation was employed to circumvent the inherently low reactivity of uronic acid building blocks during glycosylation.^21^ The benzoyl (Bz) groups at the O2 position of the glycosyl donors ensure stereochemical control in all 1,2-*trans*-glycosidic bonds during acid-promoted coupling through neighboring group participation. However, the migration of this Bz group from the axial O2 position to the equatorial O3 position of the l-Rha unit needs to be avoided during deprotection at O3 and the subsequent late-stage *O*-sulfation. The *tert*-butyldimethylsilyl (TBS) group, which could be selectively cleaved by various fluoride reagents, is designed for this purpose. Orthogonal protecting groups, such as acetyl (Ac), are introduced at the O6 position as sites for functional group transformations. The 2-naphthylmethyl (2-NAP) group blocks the C4-hydroxy group of l-rhamnose, enabling chemoselective deprotection with 2,3-dichloro-5,6-dicyano-1,4-benzoquinone (DDQ) during chain elongation and its simultaneous removal in the final deprotection. The judicious choice of protecting groups enables the synthesis of key intermediates **13**, **14**, and **15** through stereoselective glycosylation, with l-rhamnothioglycoside **16** as the common glycosyl donor, coupled with glycosyl acceptors derived from d-glucose (**17**), l-idose (**18**), and d-xylose (**19**), respectively. These monosaccharides are readily available from commercially sourced starting materials in suitably protected forms.

**Scheme 1:**
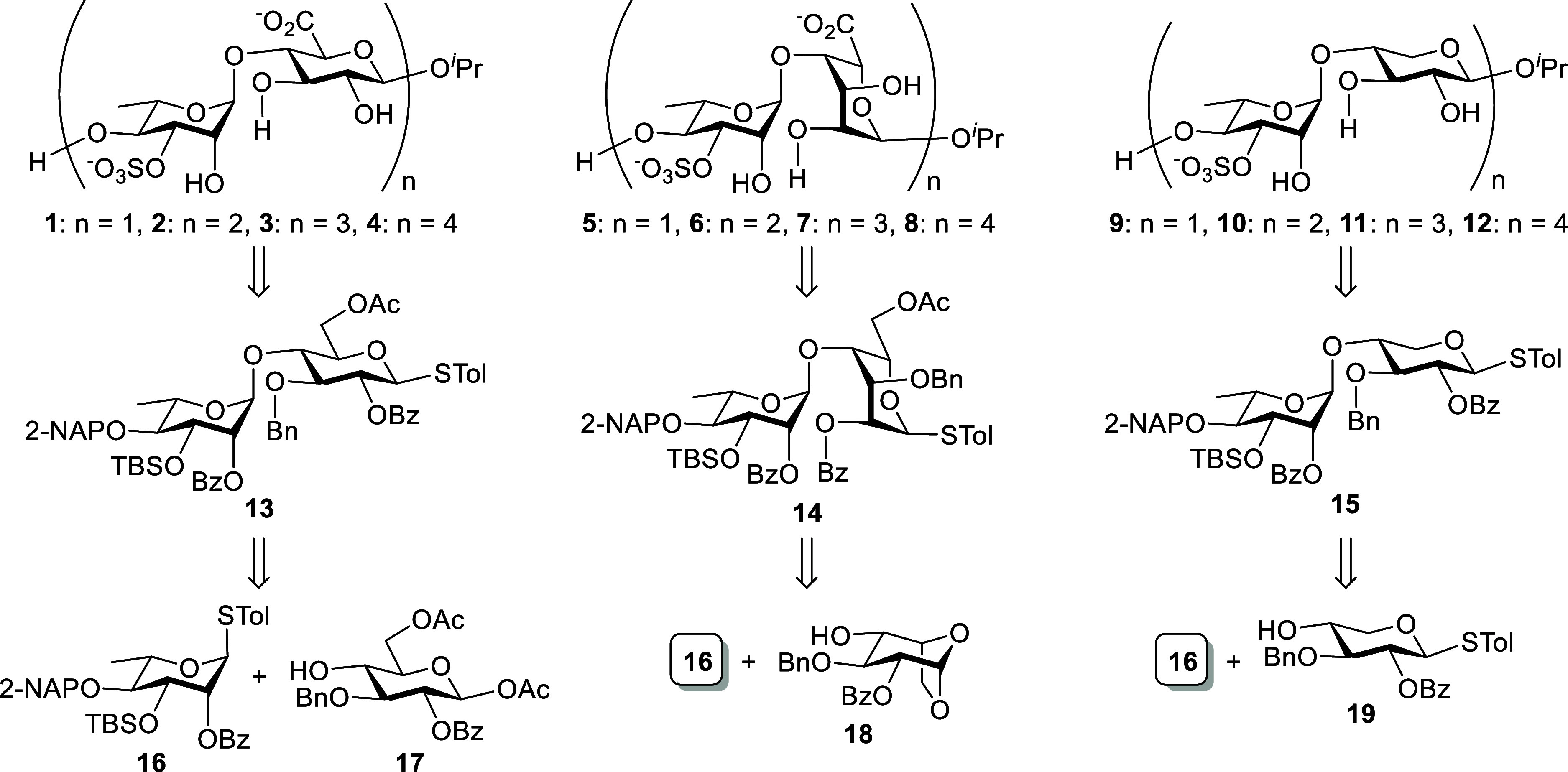
Retrosynthetic Plan of Ulvan Types A_3′S_, B_3′S_, and U_3′S_ Oligosaccharides

## Results and Discussion

### Synthesis of the Common l-Rhamnose-Derived Donor 16

A synthetic route to the key l-rhamnothioglycoside **16** is outlined in Scheme 2. Starting from l-rhamnose monohydrate, the intermediate 2,3,4-tri-*O*-acetylated thioglycoside **20** was prepared via two established steps.^22^ This compound was then subjected to Zemplén deacetylation, followed by 2,3-*O*-isopropylidenation, yielding 4-alcohol **21**. The hydroxy group at the O4 position of **21** was subsequently protected with a 2-NAP group via Williamson etherification, affording ether **22** with an overall yield of 81% over three steps. Hydrolysis of the isopropylidene ketal in **22** using 2 N HCl in an H_2_O/MeOH mixture furnished 2,3-diol **23** in 92% yield. Regioselective silylation of **23** with TBSCl and imidazole in *N*,*N*-dimethylformamide (DMF) at 0 °C resulted in three products: 3-*O*-TBS **24** (66%), 2-*O*-TBS **25** (22%), and 2,3-di-*O*-TBS **26** (7%). The undesired byproducts **25** and **26** were converted back to **23** by treatment with tetra-*n*-butylammonium fluoride (TBAF), achieving a 95% yield. Finally, the benzoylation of alcohol **24** at the O2 position with BzCl and pyridine led to the target glycosyl donor **16** in 93% yield.

**Scheme 2:**
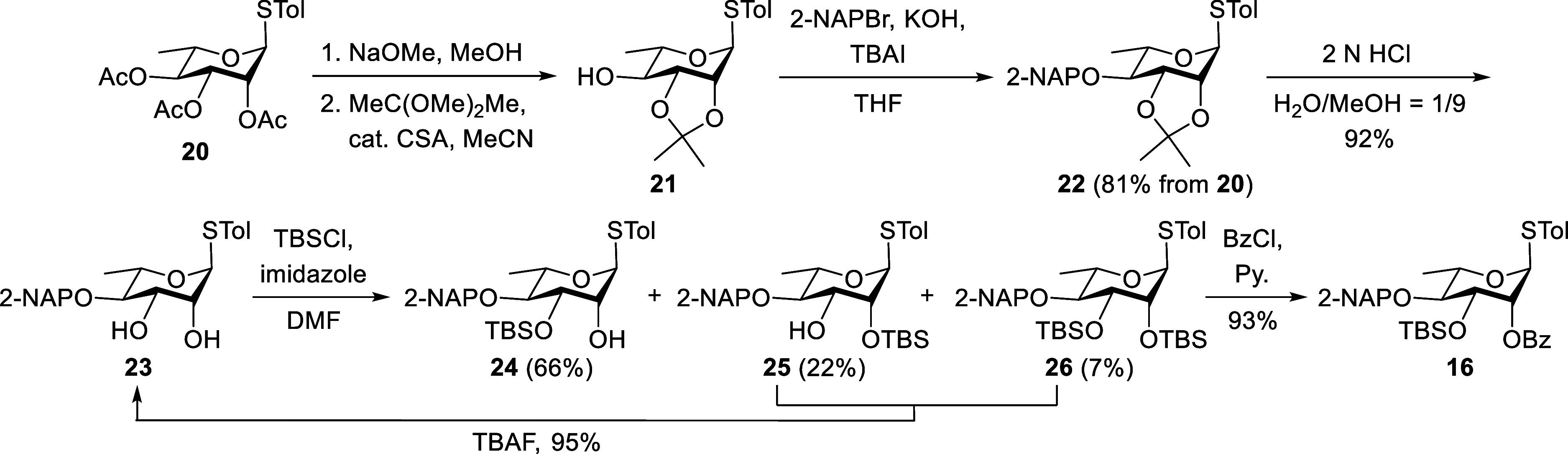
Synthesis of the l-Rhamnothioglycoside Donor 16

### Synthesis of Ulvan A_3′S_ Oligosaccharides

The target A_3′S_ sugar series contains an α-l-Rha*p*-(3S)-(1→4)-β-d-Glc*p*A repeating unit. Our initial goal was to synthesize A_3′S_ di- (**1**), tetra- (**2**), hexa- (**3**), and octasaccharide (**4**). Scheme 3 depicts a streamlined synthesis of suitably protected compounds **31**, **33**, **35**, and **37**, each containing an even number of sugar units. 3-*O*-Benzylation of readily available diacetone α-d-glucose **27** followed by hydrolysis in the presence of Dowex 50 × 4 acidic ion-exchange resin gave the 1,2,4,6-tetrol **28**.^23^ Subsequent 1,6-di-*O*-acetylation with acetic anhydride and triethylamine in DMF at −10 °C led to the desired β-acetate **29** in 53% yield over three steps. The β-configuration was confirmed by the coupling constant of H1 (*J*_H1_ = 8.2 Hz) from its ^1^H NMR spectrum. The high regioselectivity of the acetylation was driven by the low steric hindrance at the primary C6-hydroxy group and the higher acidity of the C1-hydroxy proton compared to those at C2 and C4. Regioselective benzoylation of 2,4-diol **29** with BzCl and pyridine at 0 °C afforded 4-alcohol **17** (61%), which was then coupled with the l-rhamnothioglycoside donor **16** using *N*-iodosuccinimide/trifluoromethanesulfonic acid (NIS/TfOH) in dichloromethane at −78 °C to −60 °C to furnish the exclusive α-disaccharide **30** in 82% yield. The α-selectivity was attributed to the anchimeric assistance of the 2-*O*-Bz group, as evidenced by a one-bond ^13^C–^1^H coupling constant constant (*J*_C1’–H1’_) of 171 Hz observed in the NMR analysis of compound **30**. Nondecoupled HSQC spectra enabled the identification and differentiation of α- and β-anomers, with α-isomers exhibiting a constant *J*_C1’–H1’_ value of ∼170 Hz, compared to ∼160 Hz for β-isomers.^24^ This trend in *J* values was also observed in l-rhamnosides, which served as a basis for confirming the anomeric configurations in this study.^25^ Anomeric replacement of **30** with trimethylsilyl *p*-toluenyl thioether (TMSSTol) and zinc iodide (ZnI_2_) provided the expected β-thioglycoside **13** (93%), which underwent coupling with isopropanol (^*i*^PrOH) employing NIS/TfOH to generate the exclusive β-diastereoisomer **31** in 82% yield. Chain elongation was achieved via a two-step sequence comprising 2-NAP deprotection, followed by glycosylation. Treatment of compound **31** with DDQ in wet CH_2_Cl_2_ gave 4′-alcohol **32** (92%), which was then coupled with disaccharide donor **13** to afford the target tetrasaccharide **33** (80%). The same protocol was repeated twice with compounds **34** and **36** as glycosyl acceptors, generating fully protected hexasaccharide **35** (79%) and octasaccharide **37** (83%).

**Scheme 3:**
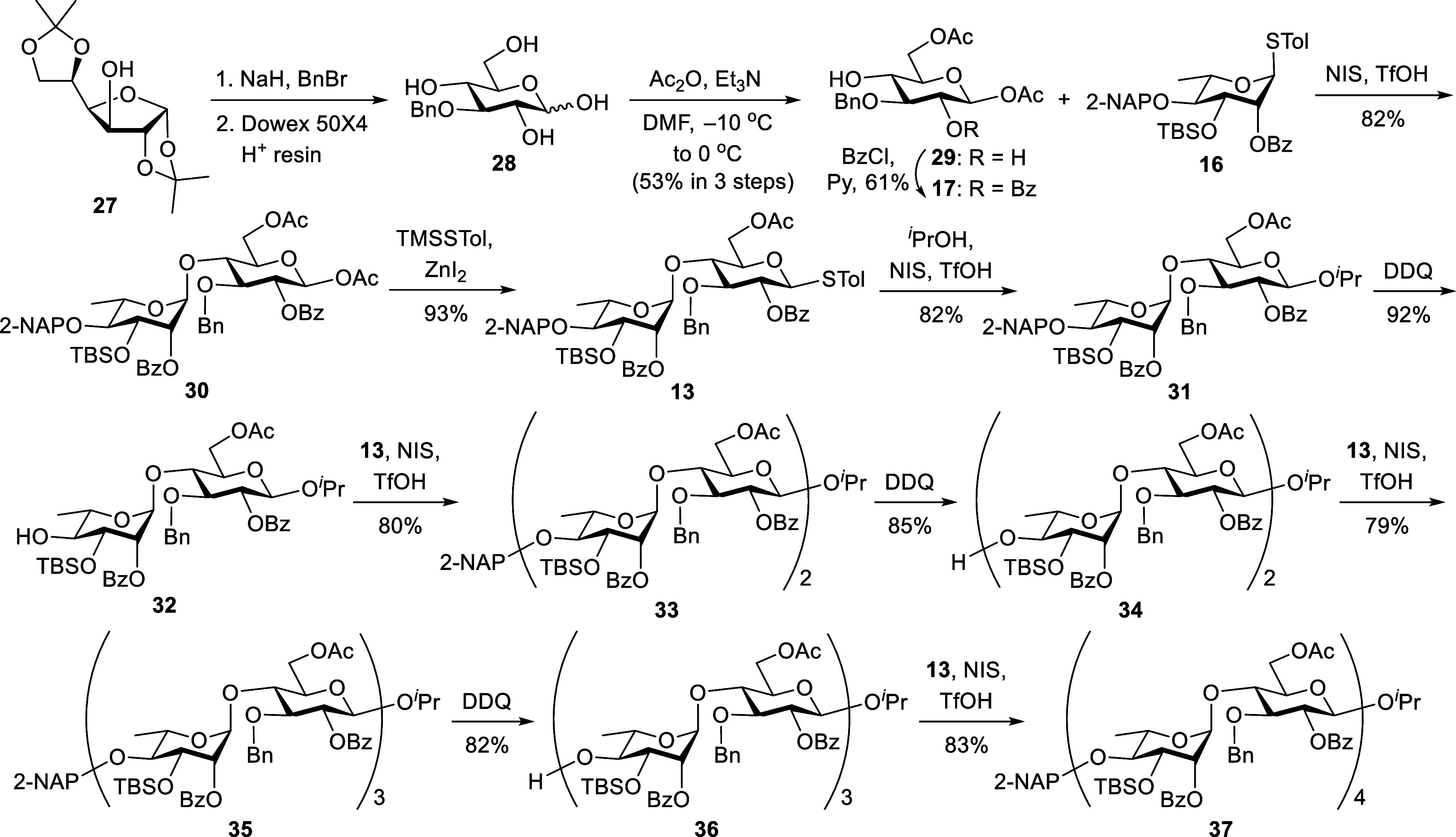
Preparation of Ulvan A_3′S_ Oligosaccharide Skeletons

The protons of octasaccharide **37** were assigned using ^1^H, ^13^C, 1D-TOCSY, and 2D-NMR analysis. Anomeric carbons and protons were first assigned by HSQC, and the complex ^1^H NMR spectrum was then resolved into six distinct patterns by selectively exciting anomeric ^1^H nuclei at a given frequency using 1D-TOCSY NMR (Figure 2). Both 1D-TOCSY and 2D-COSY NMR analyses confirmed proton connectivity within each sugar ring. The correlation of ^3^ *J*_C–H_ coupling between the anomeric glucose reducing end and the C–H of the isopropyl group was identified by the 2D-HMBC spectrum. The connectivity of sugar rings in A_3′S_ octasaccharide **37** was elucidated using 1D-TOCSY, 2D-COSY, HSQC, and HMBC.

**Figure 2:**
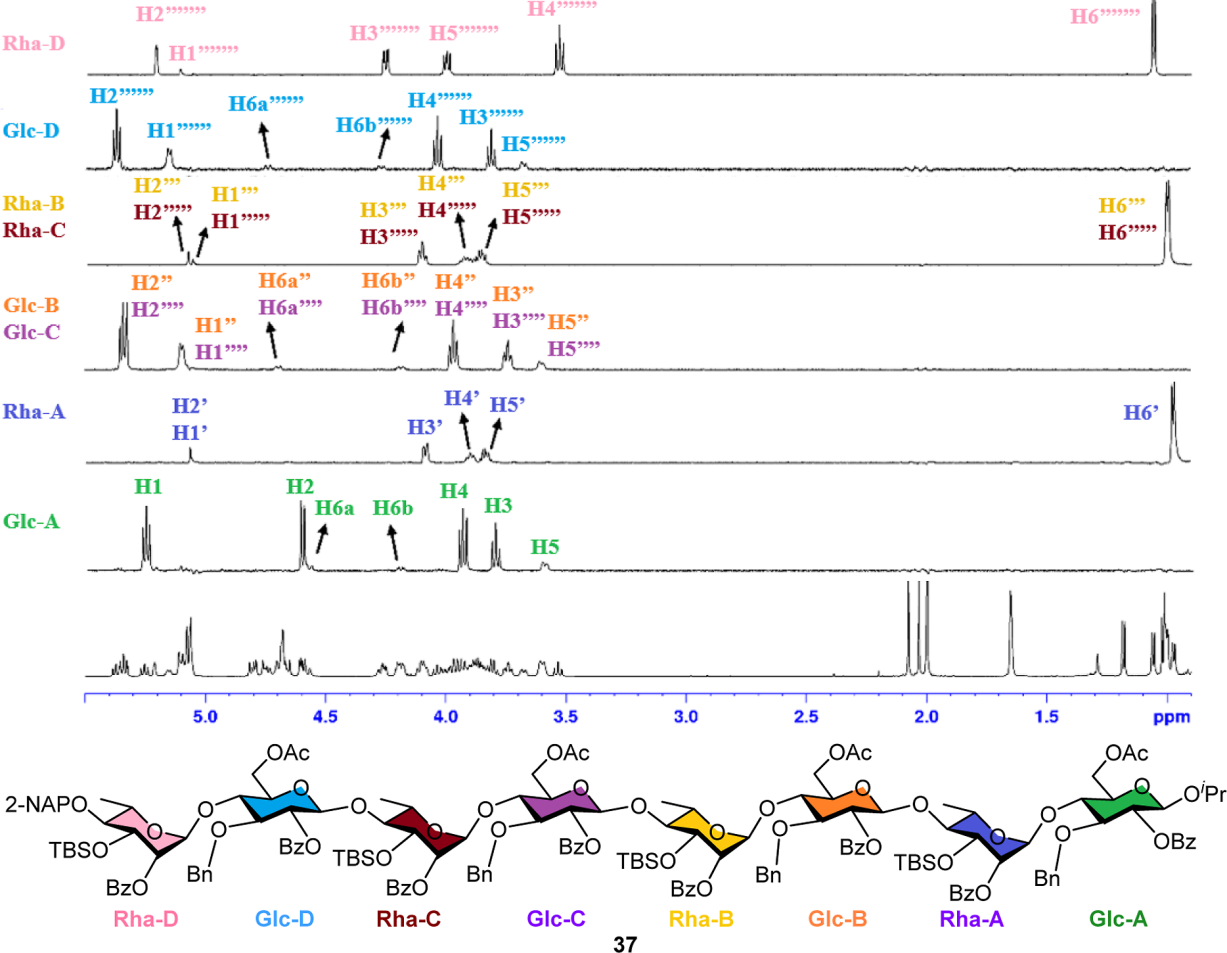
1D-TOCSY spectrum of ulvan A_3′S_ octasaccharide skeleton **37**.

Having all four desired A_3′S_ skeletons in hand, we next focused on the final functionalizations (Scheme 4). Chemoselective deacetylation of the 6-*O*-Ac groups in skeletons **31**, **33**, **35**, and **37** with Mg­(OMe)_2_ in MeOH provided the primary alcohols **38** (74%), **39** (86%), **40** (90%), and **41** (85%), respectively. TEMPO-catalyzed oxidation of compounds **38–41**, followed by CH_2_N_2_-mediated methyl esterification, furnished the methyl esters **42**, **43**, **44**, and **45** in 81%, 83%, 81%, and 78% yields, respectively. Initial efforts to remove the 3-*O*-TBS group in the disaccharide **42** using tris­(dimethylamino) sulfonium difluorotrimethylsilicate (TASF) produced the desired compound **46**, accompanied by a minor amount of a 2-*O*-Bz migrated byproduct. However, when the same conditions were applied to longer oligosaccharides, benzoyl migration became more pronounced. In particular, desilylation of tetrasaccharide **43** resulted predominantly in the formation of 2-*O*-Bz-migrated compound **47**, as unambiguously confirmed by a 2D-COSY NMR analysis (Figure 3). These results highlight the instability of Bz groups under desilylation in higher-order oligosaccharides for this series, posing a major synthetic challenge.

**Scheme 4:**
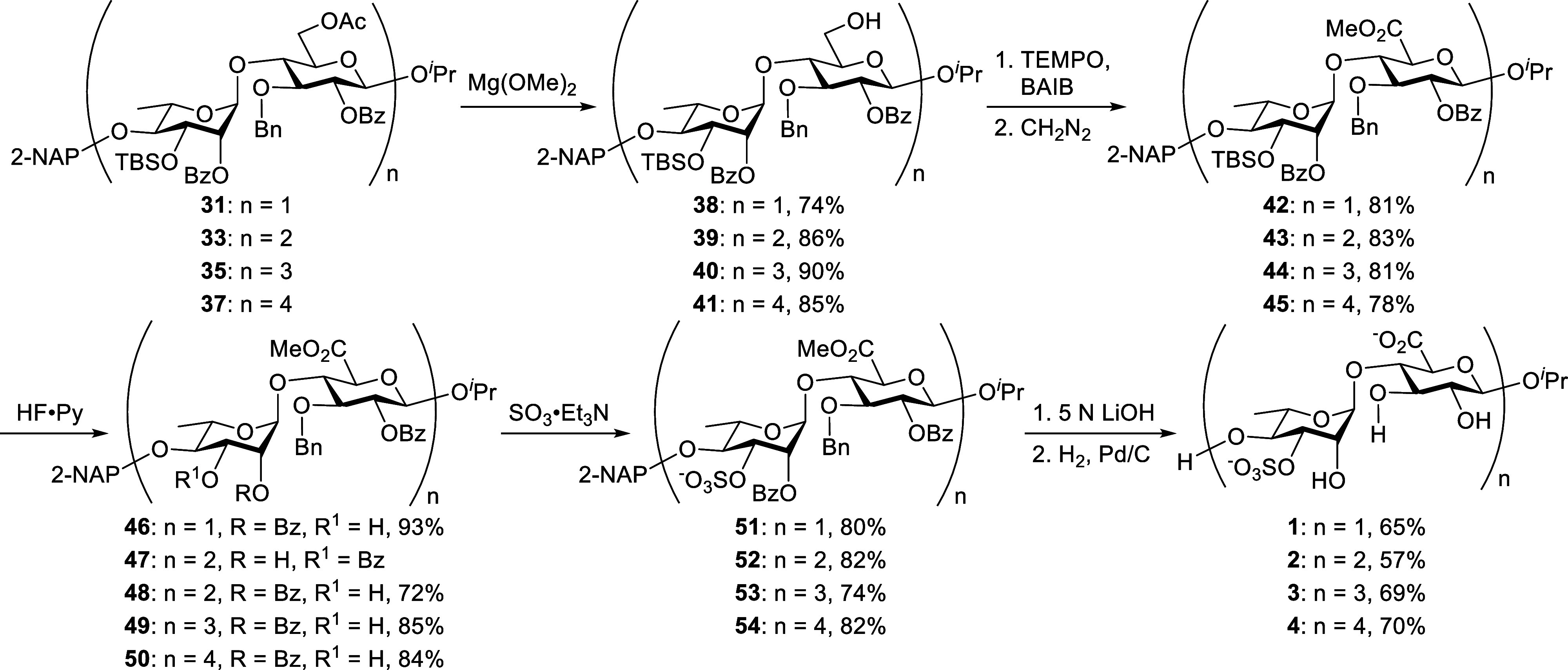
Final Functionalization of Ulvan A_3′S_ Oligosaccharides

**Figure 3:**
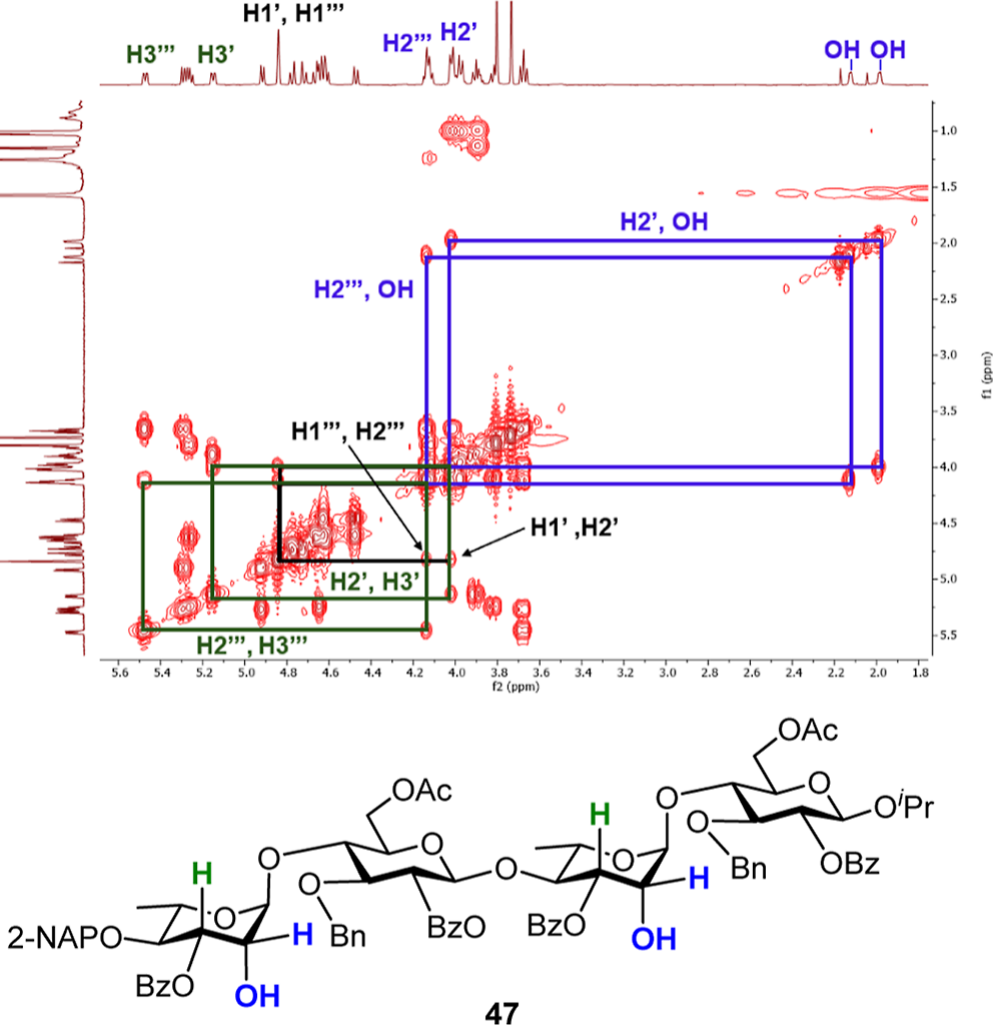
2D-COSY NMR characterization of compound **47**.

To ensure efficient desilylation and circumvent benzoyl migration, the 3-*O*-TBS groups in **42**, **43**, **44**, and **45** were effectively removed by employing HF·pyridine, providing **46** (93%), **48** (72%), **49** (85%), and **50** (84%), respectively. Subsequent 3-*O*-sulfation was successfully accomplished using SO_3_·Et_3_N, and the sulfated derivatives **51**, **52**, **53**, and **54** were obtained in 80%, 82%, 74%, and 82%, respectively. Treatment of compounds **51–54** with 5 N LiOH to remove all ester-type protecting groups, followed by global deprotection of *O-*Bn and *O*-(2-NAP) groups via hydrogenolysis, furnished the fully deprotected A_3′S_ analogues **1**, **2**, **3**, and **4** in 65%, 57%, 69%, and 70% yields, respectively. The final compound structures were rigorously confirmed by NMR and mass spectrometry (Supporting Information).

### Synthesis of Ulvan B_3′S_ Oligosaccharides

The target ulvanobiuronic acid B_3′S_ is a repeating disaccharide unit composed of α-l-Rha*p*-(3S)-(1→4)-α-l-Ido*p*A. We aimed to prepare B_3′S_ di- (**5**), tetra- (**6**), hexa- (**7**), and octasaccharide (**8**) forms of B_3′S_, which present several key synthetic challenges, including the incorporation of l-IdoA, α-glycosidic bonds, and *O*-sulfations. The synthesis of the target molecules commenced with orthogonally protected key disaccharide **14**, which was prepared efficiently as a key intermediate. The l-Ido-derived acceptor **18** was generated from commercially available diacetone α-d-glucose **27**, following previous reported procedures.^26,27^ With the appropriately protected l-Rha donor **16** and l-Ido acceptor **18** in hand, we proceeded with the construction of the B_3′S_ oligosaccharide backbones (Scheme 5). Coupling of **16** with **18** gave α-linked disaccharide **55** in 81% yield. Cu­(OTf)_2_-catalyzed acetolysis of compound **55** with acetic anhydride led to the diacetate **56**, which upon ZnI_2_-promoted anomeric substitution with TMSSTol, furnished the thioglycoside **14** in 77% yield in two steps. Compound **14** was coupled with isopropanol (^*i*^PrOH) in the presence of NIS/TfOH, affording the desired product **57** (73%) with an exclusive α-selectivity. Treatment of compound **57** with DDQ to cleave the 2-NAP group provided the corresponding 4′-alcohol **58** (78%), which underwent glycosylation with the disaccharide donor **14** to yield the tetrasaccharide **59** (81%). The same de-NAP-glycosylation protocol was carried out twice employing alcohols **60** and **62** as glycosyl acceptors, and fully protected hexasaccharide **61** and octasaccharide **63** were isolated in 92% and 86% yields, respectively. The structures of the oligosaccharide skeletons were confirmed by NMR spectroscopy and mass spectrometric analyses (see Supporting Information). The protons of octasaccharide skeleton **63** were identified using ^1^H, ^13^C, 1D-TOCSY, and 2D-NMR analysis. The complex ^1^H NMR spectrum was resolved into five ^1^H spectroscopic patterns by using 1D-TOCSY NMR (Figure 4). Both 1D-TOCSY NMR and 2D-COSY reveal proton connectivity within each ring of octasaccharide **63**.

**Scheme 5:**
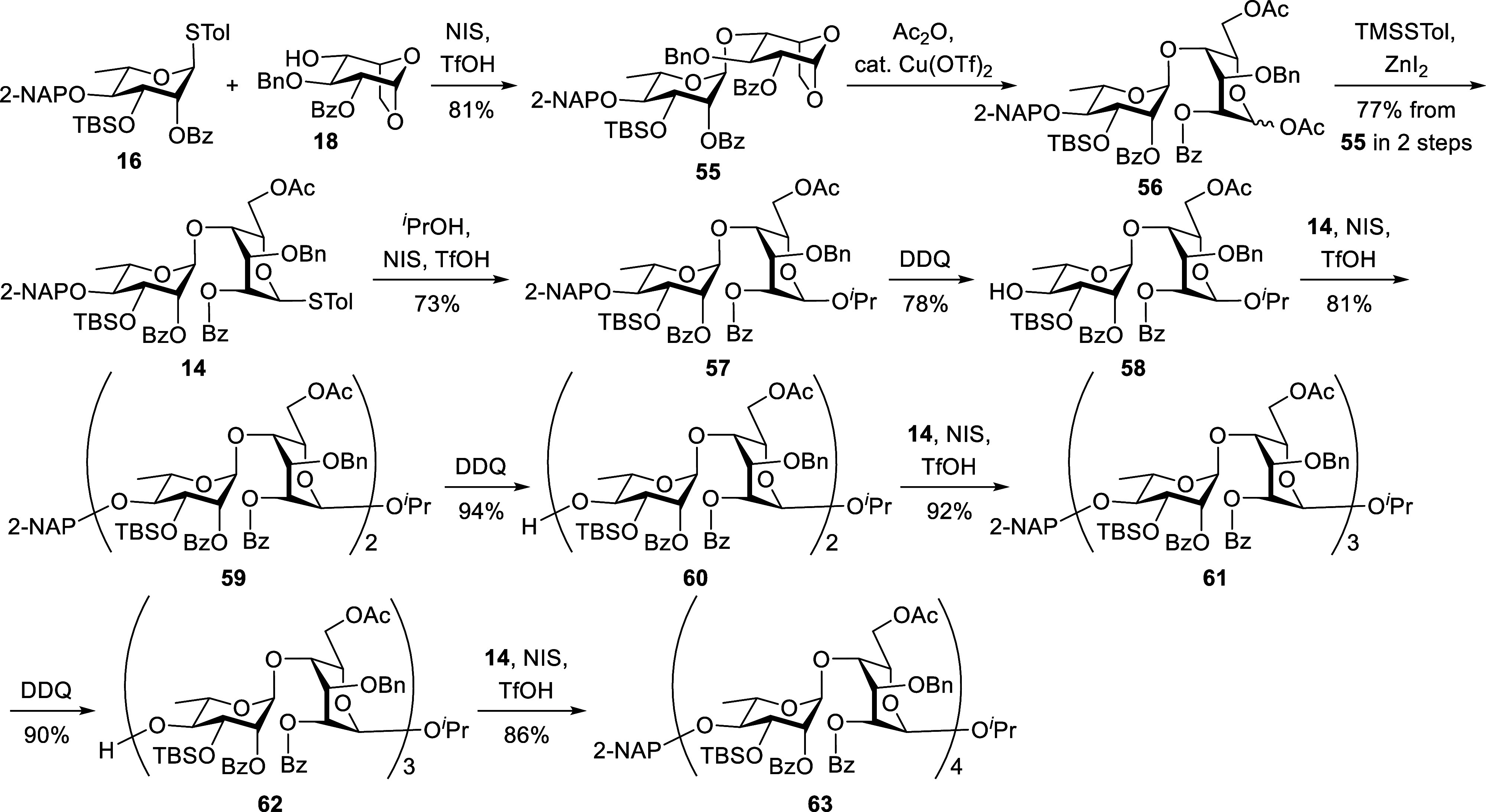
Preparation of Ulvan B_3′S_ Oligosaccharide Skeletons

**Figure 4:**
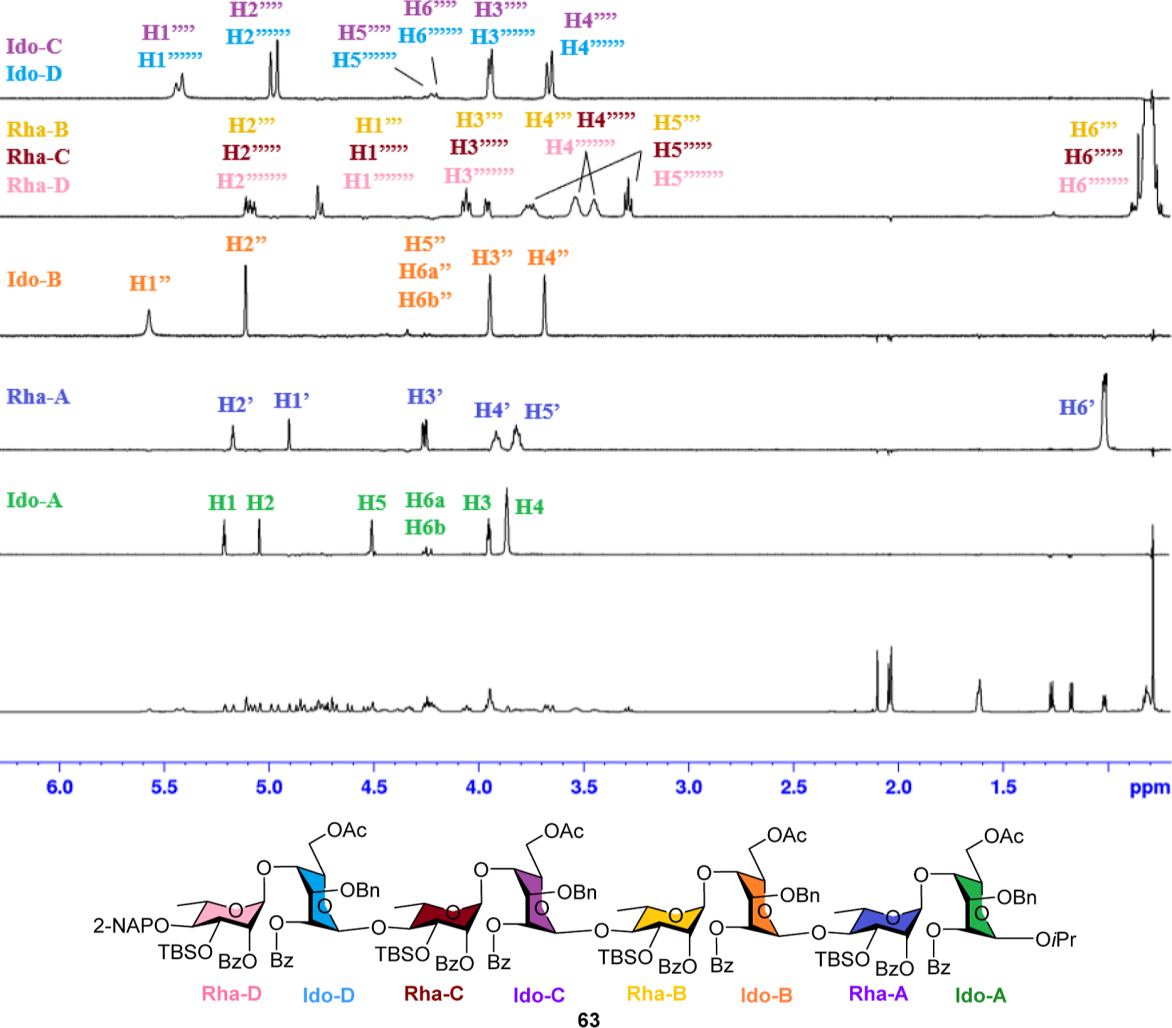
1D-TOCSY spectrum of ulvan B_3′S_ octasaccharide skeleton **63**.

After obtaining all four skeletons, we proceeded to the final functionalizations, as detailed in Scheme 6. Chemoselective cleavage of the 6-*O-*Ac groups in **57**, **59**, **61**, and **63** with Mg­(OMe)_2_ in MeOH afforded compounds **64, 65**, **66**, and **67** in 94%, 94%, 93%, and 88%, respectively. TEMPO-catalyzed oxidation of alcohols **64–67**, followed by CH_2_N_2_-mediated methyl esterification, furnished methyl esters **68** (79%), **69** (76%), **70** (87%), and **71** (78%), respectively. This late-stage oxidation strategy effectively generated the desired oligosaccharide backbone and enabled the successful installation of essential functional groups after glycosidic bond formation. Similar to the A_3′S_ oligosaccharides, removal of the 3-*O*-TBS group from disaccharide **68** proved challenging, as nonacidic conditions consistently triggered the undesired migration of the benzoyl group from the O2 to the O3 position of the rhamnose moiety. As illustrated in Table 1, basic (TBAF, entry 1) and neutral (TASF, entry 2) treatments both led to benzoyl group migration. Acidic desilylation using HF·pyridine (entry 3) was also investigated but failed to produce the target compound. Phosphomolybdic acid^28^ (PMA, entry 4) in THF was also ineffective, yielding only trace amounts of product after 3 days. In contrast, PMA in acetonitrile (entry 5) overcame the benzoyl group migration and provided efficient cleavage of the 3-*O*-TBS group, affording the desired product **72** in 92% yield within 3 h at room temperature. This optimized PMA-mediated 3-*O*-TBS deprotection protocol was successfully extended to compounds **69**, **70**, and **71**, giving the corresponding desilylated intermediates **73**, **74**, and **75** in 86%, 84%, and 61% yields, respectively. Subsequent regioselective *O*-sulfation of the alcohols **72–75** using the SO_3_·Et_3_N complex led to the sulfated derivatives **76** (86%), **77** (86%), **78** (90%), and **79** (71%), respectively. These compounds then underwent deacylation under basic conditions (5 N LiOH), followed by global deprotection via hydrogenolysis, ultimately providing the B_3′S_ oligosaccharides **5**, **6**, **7**, and **8** in 48%, 74%, 71%, and 60% yields, respectively. The structures of all final B_3′S_ oligosaccharides were definitively confirmed by comprehensive NMR spectroscopy and mass spectrometry analyses (see Supporting Information).

**Scheme 6:**
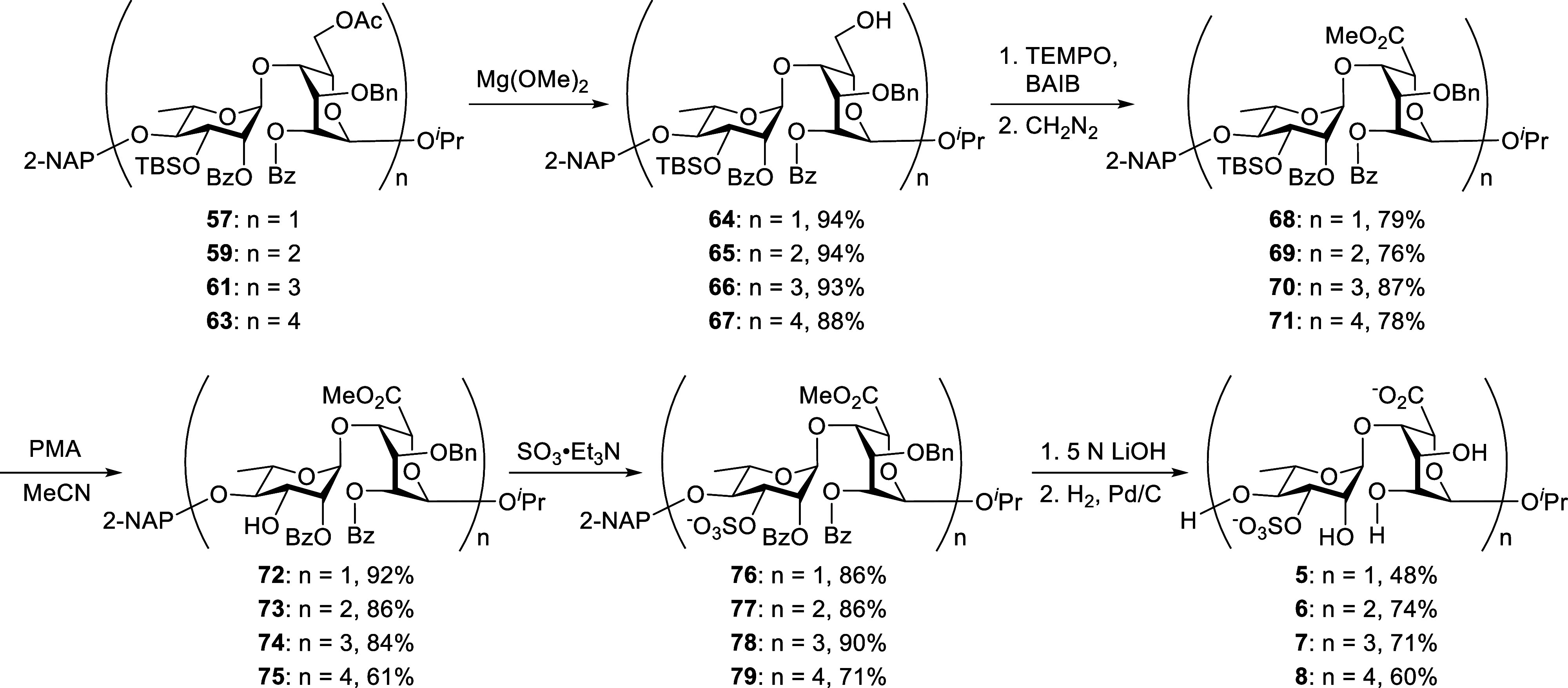
Final Functionalization of Ulvan B_3′S_ Oligosaccharides

**Table 1:**
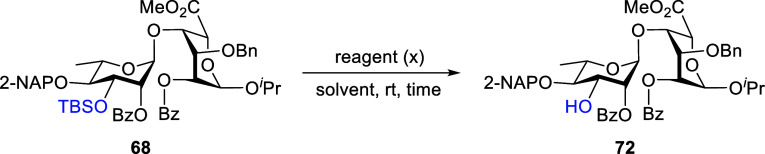
Optimization of the Desilylation Reactions

entry	reagent	x	solvent	time	yield
1	TBAF	3.0 equiv	THF		Bz migration
2	TASF	10 mL/g	DMF		Bz migration
3	HF·pyridine	4 mL/g	THF		Bz migration
4	PMA	0.5 mol %	THF	3 d	trace amount
5	PMA	0.5 mol %	MeCN	3 h	92%

### Synthesis of Ulvanobioses U_3′S_

The target U_3′S_ oligosaccharides—di- (**9**), tetra- (**10**), hexa- (**11**), and octasaccharide (**12**)—are composed of repeating disaccharide units of α-l-Rha*p*-(3S)-(1→4)-β-d-Xyl*p*. The key synthetic challenges include the incorporation of d-xylose residues, formation of β-glycosidic bonds, and precise *O*-sulfation patterns. The synthesis of d-xylose-derived acceptor **19** is outlined in Scheme 7. Triol **80**, prepared from d-xylose according to our previous reports,^29,30^ was subjected to trimethylsilyl (TMS) etherification using TMSCl and Et_3_N, furnishing the tri-*O*-TMS ether **81** in 97% yield. Subsequent treatment of compound **81** with benzaldehyde (PhCHO) and TMSOTf at 0 °C, followed by the addition of TBAF at room temperature in a one-pot manner, afforded 2,4-*O*-benzylidene acetal **82** in 81% yield. The structure of **82** was confirmed by NMR analysis. 2D-COSY W-coupling (see Supporting Information) indicated that **82** adopts a ^1^ *C*_4_ conformation as the ^4^ *C*_1_ conformer did not exhibit W-coupling. Furthermore, the absolute configuration of **82** was established by the X-ray crystallography of single crystals (Scheme 7, CCDC 2363767). Interestingly, the anomeric STol group was epimerized to an **α-configuration**, as confirmed by both the X-ray and NMR analysis of **82**. Subsequent 3-*O*-benzylation of **82** using BnBr and NaH, followed by benzylidene deprotection with CSA in MeOH, furnished 2,4-diol **83** in 83% yield. NMR analysis revealed that the anomeric STol group had reverted to the **β-configuration**. Finally, regioselective 2-*O*-benzoylation of **83** with benzoic anhydride in the presence of Yb­(OTf)_3_ produced the desired 4-alcohol **19** in 81% yield. The NMR spectrum of **19** was consistent with previously published data.^29,30^

**Scheme 7:**
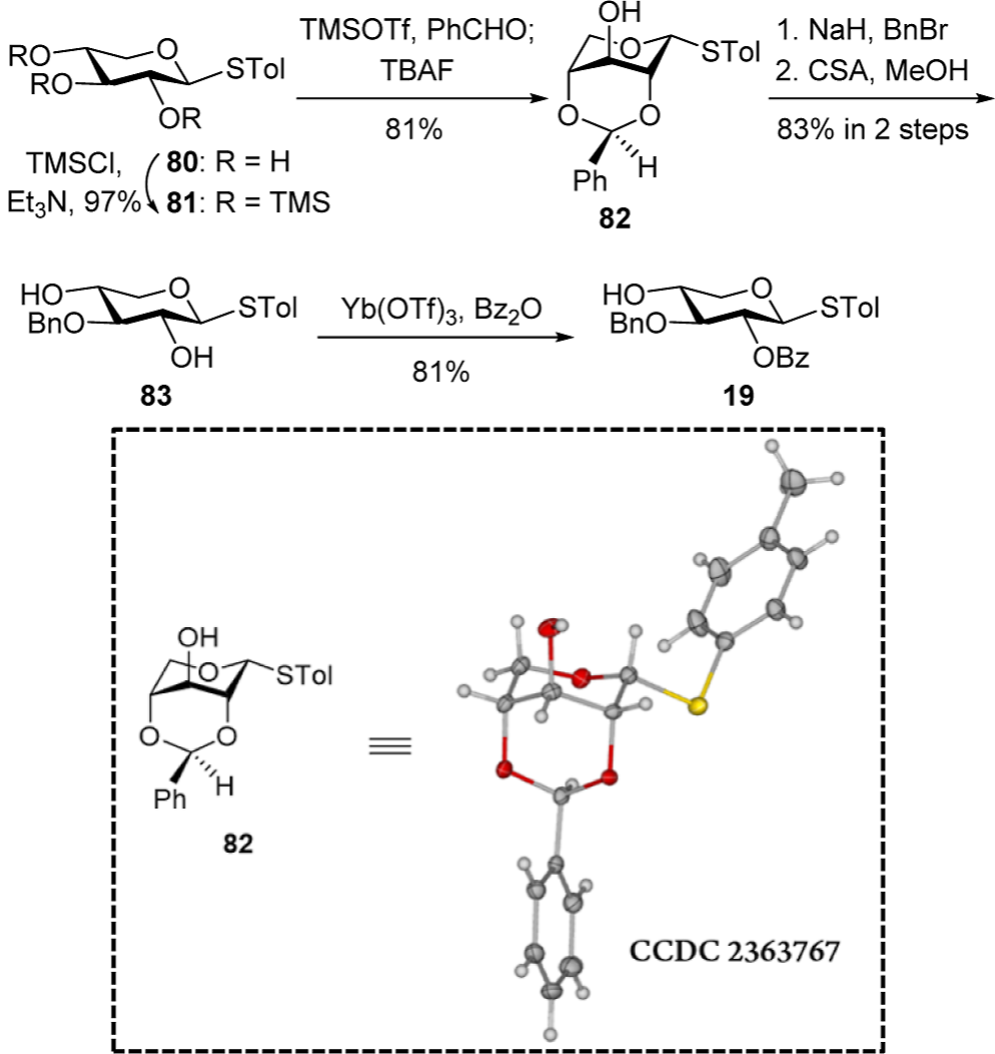
Preparation of d-Xylose-Derived Thioglycoside 19

With the l-rhamnothioglycoside **16** and the d-xylose-derived acceptor **19** in hand, we proceeded to investigate the stereoselective coupling reaction (Scheme 8). In our initial attempt, a preactivation-based glycosylation was performed using 1-benzenesulfinyl piperidine (BSP) and trifluoromethanesulfonic anhydride (Tf_2_O) as promoters, in the presence of 2,4,6-tri-*tert*-butylpyrimidine (TTBP).^31^ This reaction furnished the desired α-disaccharide **15** in 60% yield along with various side products. To improve the yield of **15**, compound **16** was first oxidized to the corresponding anomeric lactol **84** in 96% yield by using *N*-bromosuccinimide (NBS) in wet acetone. A subsequent cesium carbonate-promoted reaction of **84** with trichloroacetonitrile and 2,2,2-trifluoro-*N*-phenylacetimidoyl chloride (**86**) yielded two distinct glycosyl imidate donors, **85** and **87**. These intermediates were used directly in the glycosylation step without further purification. TfOH-catalyzed coupling of the trichloroacetimidate **85** with **19** predominantly resulted in the formation of a rearranged anomeric amide byproduct, rather than the desired glycoside. In contrast, glycosylation of *N*-phenyltrifluoroacetimidate **87** with **19** proceeded efficiently, affording the disaccharide **15** in an excellent yield of 92%.

**Scheme 8:**
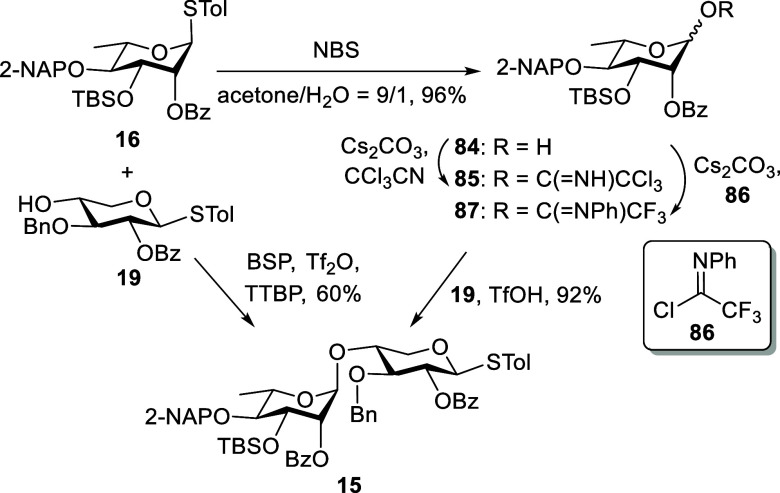
Synthesis of the Ulvan U_3′S_ Disaccharide Skeleton

Next, we focused on the preparation of the U_3′S_ oligosaccharide skeletons **88**, **90**, **92**, and **94**, as illustrated in Scheme 9. Treatment of disaccharide **15** with ^*i*^PrOH in the presence of NIS/TfOH as the promoter led to compound **88** in 85% yield. The β-stereochemistry at the newly formed anomeric center was confirmed by the coupling constant of the anomeric proton observed at 4.55 ppm in the ^1^H NMR spectrum (d, *J* = 7.5 Hz, H1). Subsequent DDQ-mediated oxidative cleavage of the 2-NAP group in **88** provided the corresponding 4′-hydroxy compound **89** (86%), which was coupled with donor **15** to furnish the tetrasaccharide **90** (87%). This de-NAP-glycosylation sequence was repeated twice to elongate the chain, affording alcohol **91**, hexasaccharide **92**, alcohol **93**, and octasaccharide **94** in yields of 85%, 86%, 80%, and 87%, respectively. The structures of the U_3′S_ oligosaccharide skeletons were unambiguously confirmed by NMR spectroscopy (see Supporting Information). For octasaccharide **94**, all proton resonances along the backbone were assigned based on ^1^H, ^13^C, 1D-TOCSY, and 2D-NMR experiments. Notably, the complex ^1^H NMR spectrum of **94** was successfully deconvoluted into seven distinct ^1^H spectroscopic patterns using 1D-TOCSY (Figure 5). Both 1D-TOCSY and 2D-COSY analyses clearly revealed the intraring proton connectivities, confirming the integrity and sequence of the oligosaccharide.

**Scheme 9:**
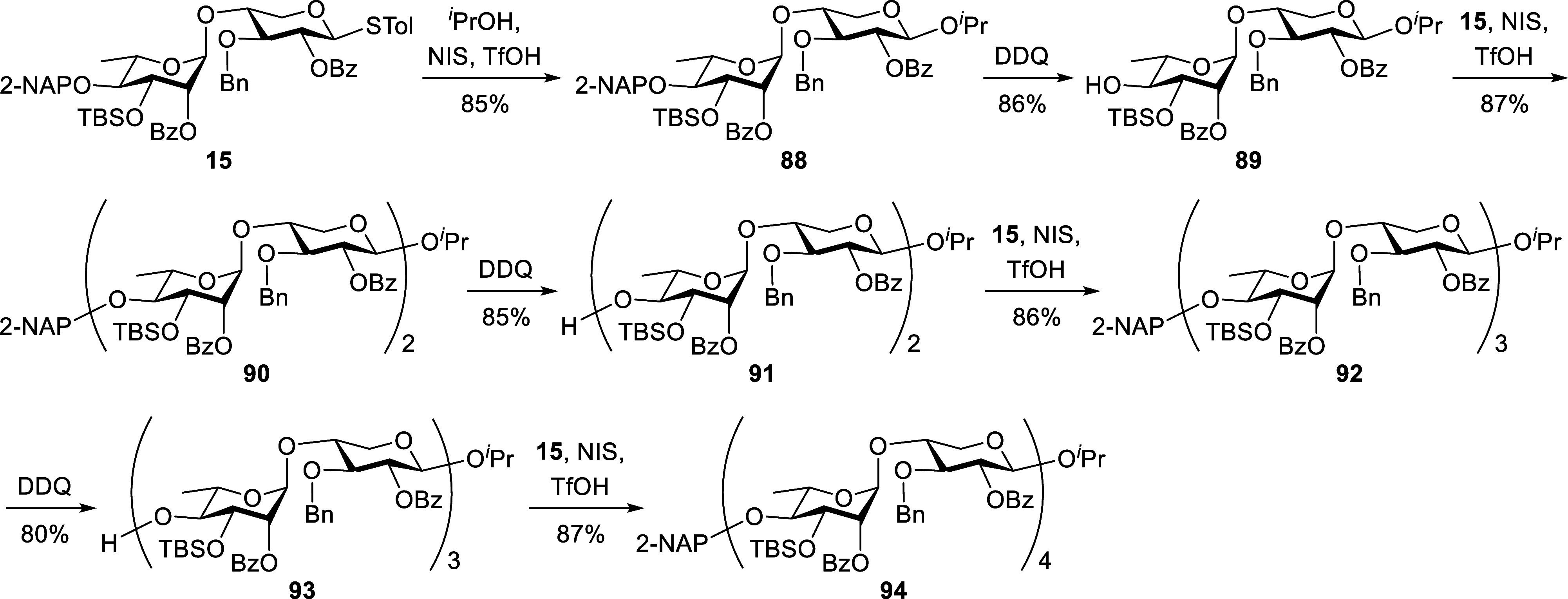
Preparation of Ulvan U_3′S_ Oligosaccharide Skeletons

**Figure 5:**
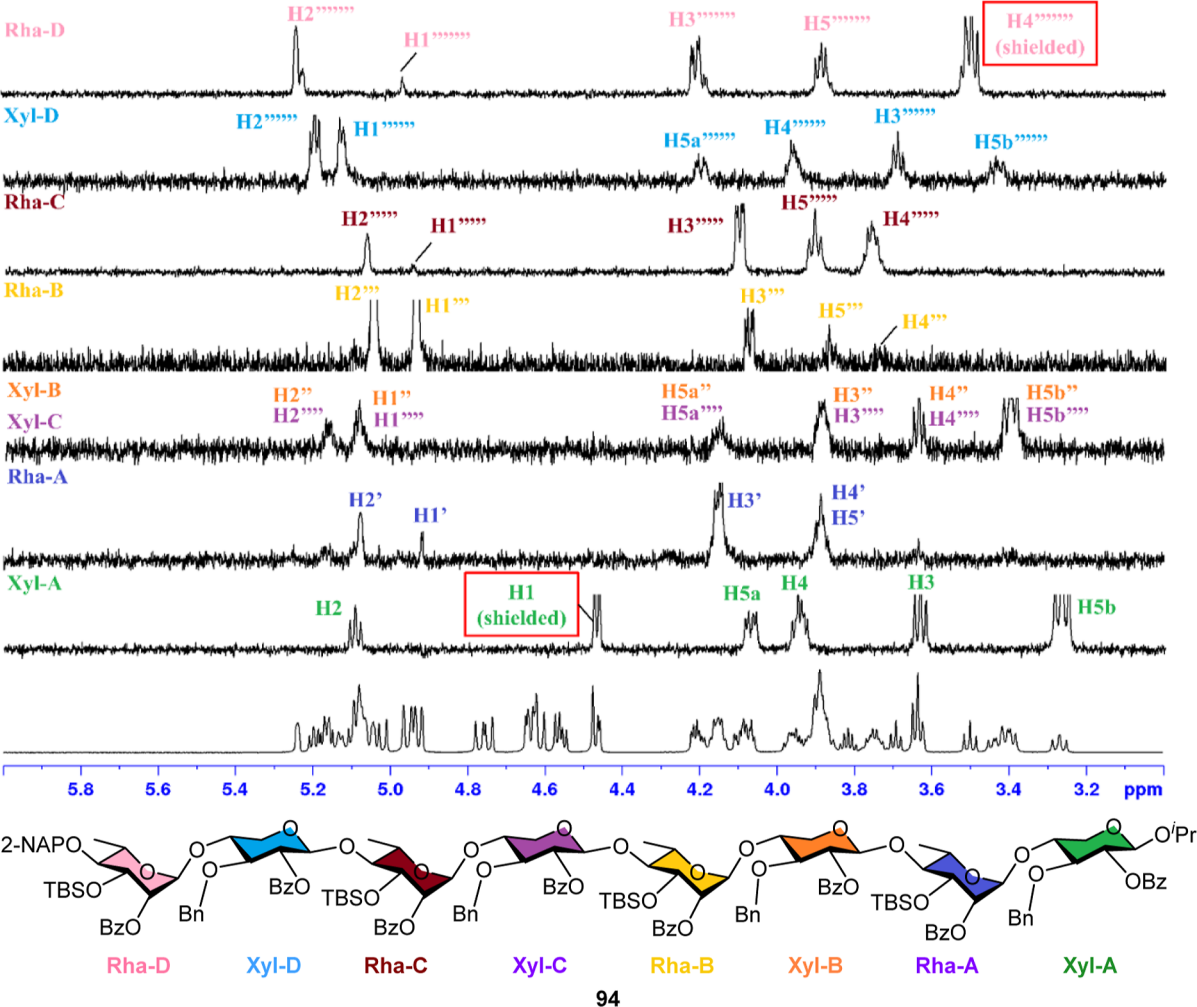
1D-TOCSY spectrum of ulvan U_3′S_ octasaccharide skeleton **94**.

With the U_3′S_ skeletons in hand, we proceeded to the final functionalization, as outlined in Scheme 10. Initial attempts to deprotect the 3-*O*-TBS group using HF·pyridine or TBAF consistently resulted in undesired migration of the 2-*O*-Bz group within the l-Rha units. This differential reactivity of individual sugar residues toward desilylation highlighted a key synthetic challenge that required careful resolution. This obstacle was successfully overcome by employing boron trifluoride diethyl etherate (BF_3_·Et_2_O),^27^ which cleanly effected 3-*O*-TBS deprotection and furnished the corresponding alcohols **95**, **96**, **97**, and **98** in 90%, 88%, 83%, and 80% yields, respectively. Subsequent *O*-sulfonation was carried out using SO_3_·Et_3_N, yielding individual sulfated derivatives **99** (89%), **100** (75%), **101** (81%), and **102** (80%) following purification by column chromatography. The benzoyl protecting groups of **99–102** were then removed under basic conditions (5 N NaOH), affording the corresponding free alcohols **103** (70%), **104** (88%), **105** (91%), and **106** (85%). These intermediates were finally subjected to global deprotection and purification to deliver the fully functionalized U_3′S_ oligosaccharides **9**, **10**, **11**, and **12** in 72%, 70%, 74%, and 68% yields, respectively. The structures of the final compounds were unequivocally confirmed by NMR spectroscopy and mass spectrometric analyses (see Supporting Information).

**Scheme 10:**
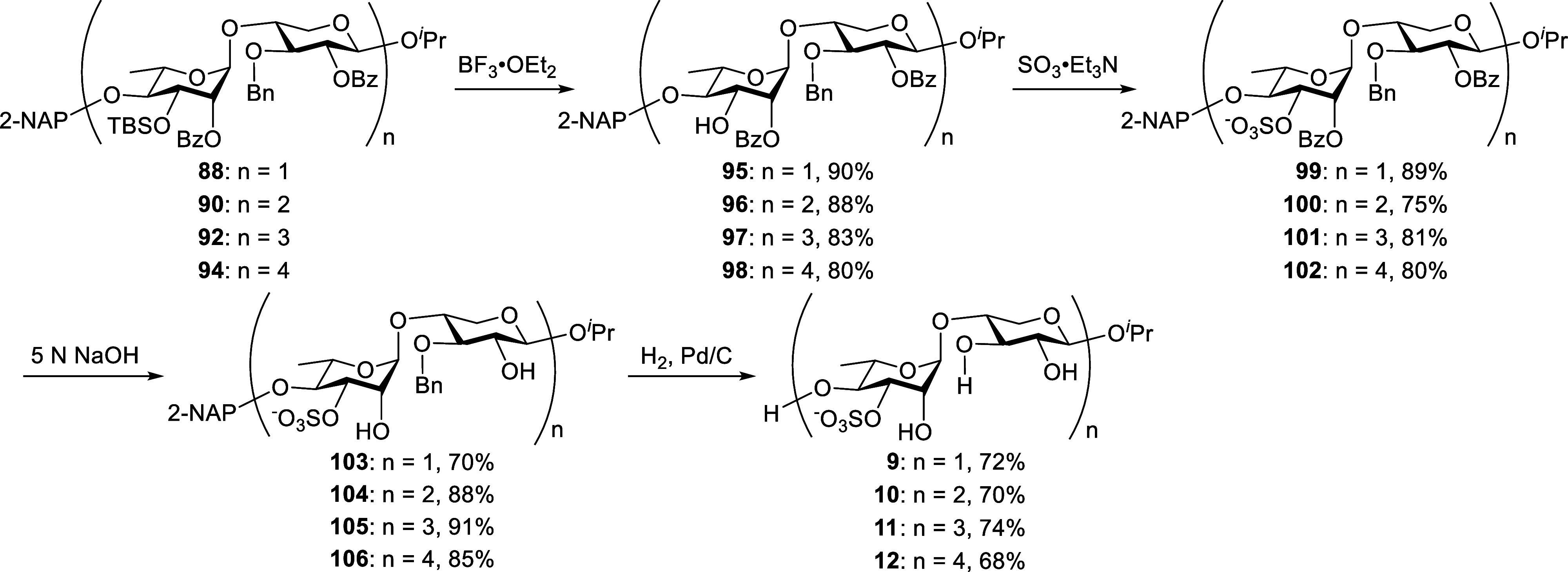
Final Functionalization of Ulvan U_3′S_ Oligosaccharides

### Immunomodulatory Effects

The synthesized compounds were evaluated for their immunomodulatory activities by assessing the cytokine gene expression. RAW264.7 cells were treated with each compound at a concentration of 250 μM for 24 h, followed by RT-PCR analysis to monitor changes in cytokine expression. Untreated cells served as the control group. We first monitored the expression of IL-1β, an important upstream regulator for immunomodulation (Figure S3A). Once the activities were confirmed, transcriptional regulation of other cytokines was further measured (Figures 6 and S3B). Notably, both the B_3′S_-octasaccharide **8** and the U_3′S_-octasaccharide **12**, at a concentration of 250 μM, induced the upregulation of IL-1β, IL-6, IL-10, GM-CSF, and G-CSF genes, indicating transcriptional activation of both pro- and anti-inflammatory pathways. The immunomodulatory effects of octasaccharides **8** and **12** were further validated at the protein expression level via ELISA, which quantified the secretion of selected cytokines. Consistent with gene expression data, both compounds significantly enhanced the secretion of GM-CSF and G-CSF (Figure 7). Interestingly, U_3′S_-oligosaccharides **9**, **10**, and **11** elicited only a modest induction of **G-CSF** expression. These findings indicate that the immunomodulatory activity is strongly dependent on oligosaccharide chain length, with the octasaccharides **8** (B_3′S_) and **12** (U_3′S_) exhibiting markedly higher activity compared to their hexameric or tetrameric counterparts. Among the active octamers, B_3′S_-octasaccharide **8** demonstrated superior immunomodulatory potency relative to U_3′S_-octasaccharide **12**. In contrast, the A_3′S_-oligosaccharides did not display any detectable immunomodulatory activity under the conditions tested.

**Figure 6:**
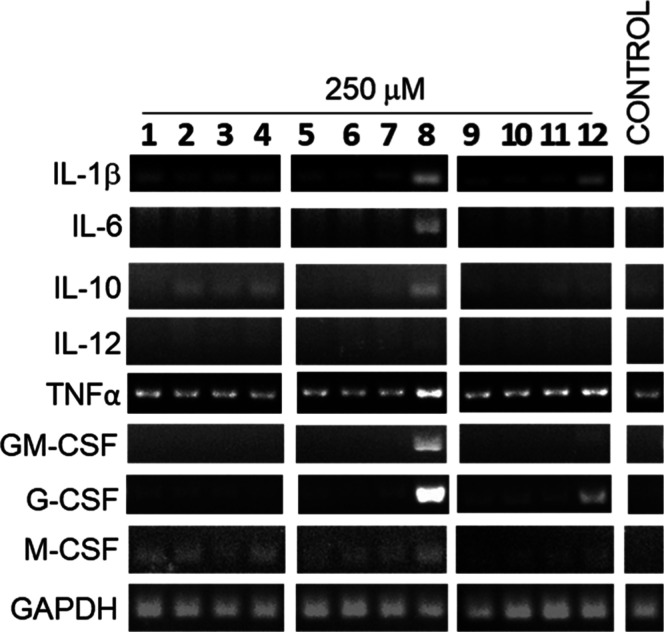
Gene expression of cytokines induced by **1**–**12**.

**Figure 7:**
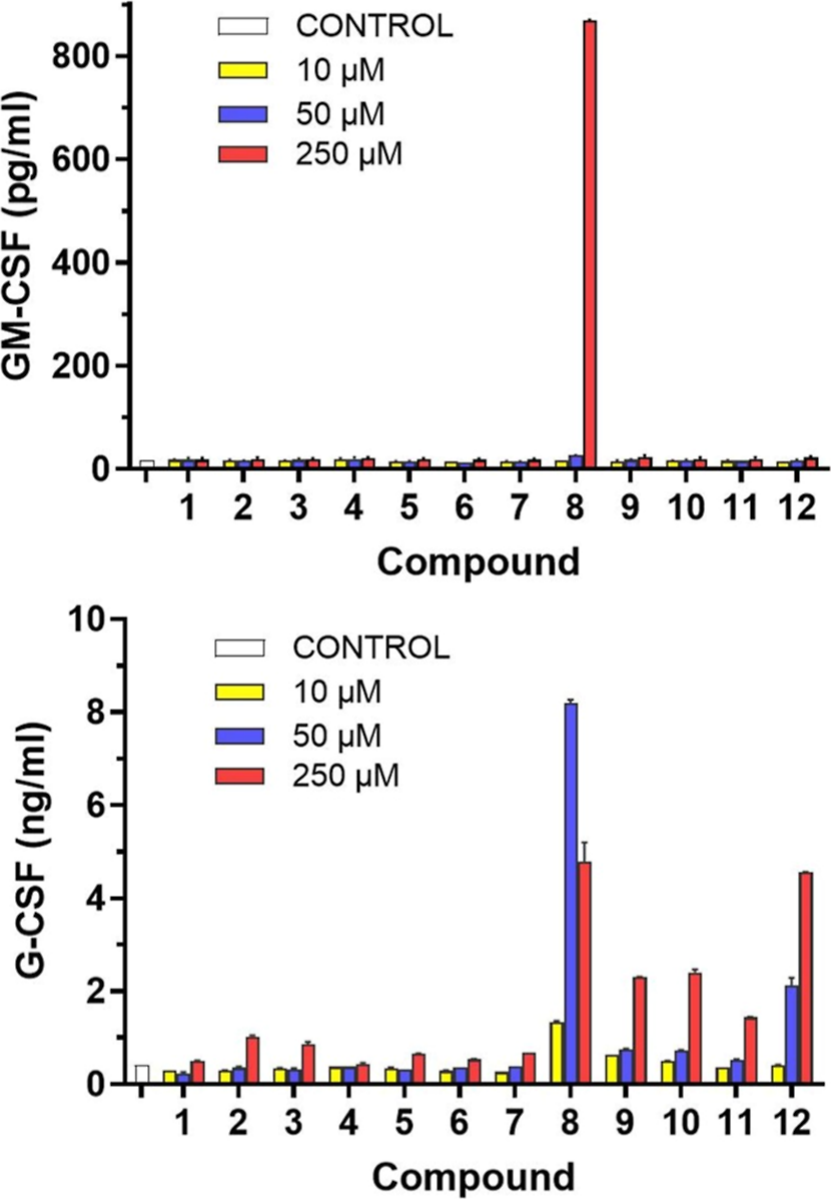
Protein expression of cytokines induced by **1**–**12**. RAW264.7 cells were treated with compounds at indicated concentrations for 24 h and the medium of the treated cells was processed for ELISA to determine the concentration of the released cytokines. The untreated samples were marked as control. Shown values are the mean values ± standard deviations from three independent experiments.

## Conclusion

We developed a systematic strategy for the synthesis of sulfated ulvan oligosaccharide libraries, beginning with orthogonally protected key disaccharides **13**, **14**, and **15**. This approach employs streamlined glycosylation reactions and effectively overcomes multiple synthetic challenges. All glycosidic linkages were constructed with high stereoselectivity, facilitated by anchimeric assistance. The issue of desilylation without benzoyl group migration was successfully addressed by using appropriate reagents. Sulfation, followed by global deprotection, reliably yielded the complex target oligosaccharides, whose structures were thoroughly characterized using NMR and mass spectrometry. These versatile synthetic methodologies are expected to be broadly applicable in the construction of structurally defined sulfated glycans. Immunomodulatory assays revealed that the B_3′S_-octasaccharide **8** induced significantly stronger expression of IL-1β, IL-6, IL-10, GM-CSF, and G-CSF genes compared to the U_3′S_-octasaccharide **12**.

## Supplementary Material



## Notes

Synthetic procedures, characterization data, crystal structure (CCDC number 2363767), NMR spectra, structural characterization data, and immunomodulatory activity assays (PDF)
