# Supplementary material for: Streamlined Synthesis and Immunomodulatory Effects of Ulvan Oligosaccharides from Marine Green Algae
Source: J Am Chem Soc. 2025 Aug 28;147(36):32951–62. doi: 10.1021/jacs.5c09759 (PMC12426931; doi:10.1021/jacs.5c09759)
Supplement: Supplementary file 1 [file ja5c09759_si_001.pdf]

Supporting Information

Streamlined Synthesis and Immunomodulatory Effects of Ulvan Oligosaccharides from Marine Green Algae

Junpei Shimabukuro,[‡1] Ci-Hua Shen,[‡1,2] Tsung-Kai Hsueh,[‡1,2] Ting-Wen Chen,[‡1,2] Yin-Chen Tseng,[1] Veeranjaneyulu Gannedi,[1] Jasper S. Dumalaog,[1,3] Cheng-Hsiu Chang,*[1] Ting-Jen Rachel Cheng,*[1] Chung-Shan Yu,*[2,4] Shang-Cheng Hung*[1,3,5,6]

[1]Genomics Research Center, Academia Sinica, 128, Section 2, Academia Road, Taipei 11529, Taiwan.

[2]Institute of Nuclear Engineering Science, National Tsing Hua University, 101, Section 2, Kuang-Fu Road, Hsinchu 30013, Taiwan.

[3]Department of Chemistry, National Tsing Hua University, 101, Section 2, Kuang-Fu Road, Hsinchu 30013, Taiwan.

[4]Department of Biomedical Engineering and Environmental Sciences, National Tsing Hua University, 101, Section 2, Kuang-Fu Road, Hsinchu 30013, Taiwan.

[5]Department of Chemistry, National Cheng Kung University, 1, University Road, Tainan 70101, Taiwan.

[6]Department of Applied Science, National Taitung University, 369, Section 2, University Road, Taitung 95092, Taiwan.

*E-mails: dp33426@as.edu.tw, tingjenc@gate.sinica.edu.tw, csyu@mx.nthu.edu.tw, schung@gate.sinica.edu.tw

‡These authors contributed equally to this work.

<u>**Table of Contents**</u>

Experimental Section S3

1. General Procedures S3
2. Synthetic Procedures and Characterization data for A3's S4
3. Synthetic Procedures and Characterization data for B3's S40
4. Synthetic Procedures and Characterization data for U3's S70
5. Crystallographic Data for Compound **82** S96
6. Biological Procedures S98
7. NMR Spectra for the Synthesis of A3's Libraries S100
8. NMR Spectra for the Synthesis of B3's Libraries S200
9. NMR Spectra for the Synthesis of U3's Libraries S290

Experimental Section

1. General Procedures

All reactions were performed under an atmosphere of nitrogen/argon in flame-dried glassware. Solvents were distilled in the standard way, and commercial reagents were used without any purification, unless otherwise stated. Anhydrous solvents like CH_2Cl_2, Et_2O, DMF, and Et_3N were dried in a standard way. TLC was performed on glass plates pre-coated with Silica Gel 60 F_{254} (0.25 mm, E. Merck). TLC plates were visualized by exposure to ultraviolet light (UV-254 nm) and/or detection was executed by spraying with a solution of $Ce(NH_4)_2(NO_3)_6$, $(NH_4)_6Mo_7O_{24}$, and H_2SO_4 in water and subsequent heating on a hot plate. Specific rotations were taken at ambient conditions and reported in $10^{-1}\cdot deg\cdot cm^2\cdot g^{-1}$; the sample concentrations are in $g\cdot dL^{-1}$. Flash column chromatography was carried out on Silica Gel 60 (230–400 mesh, E. Merck) or MP. ^1H NMR spectra were recorded on 600 MHz instrument at ambient temperature. Data were recorded as follows: chemical shift in ppm from the solvent resonance employed as the internal standard ($CDCl_3$ at 7.26 ppm, CD_3OD at 3.31 ppm and 4.78 ppm, and D_2O at 4.80 ppm), multiplicity (s = singlet, d = doublet; t = triplet; q = quartet; st = septet; m = multiplet; br = broad), coupling constant (in Hz), integration. ^{13}C NMR spectra were measured on 150 MHz spectrometer. Chemical shifts were recorded in ppm from the solvent resonance employed as the internal standard ($CDCl_3$ at 77.2 ppm and CD_3OD at 49.0 ppm). Mass spectra were obtained with ESI Finnigan LCQ mass spectrometer (Thermo Finnigan), performed at the Genomics Research Center. Prior to all the glycosylations, the starting materials were dried under high vacuum overnight in a desiccator.

2. Synthetic Procedures and Characterization data for A3's

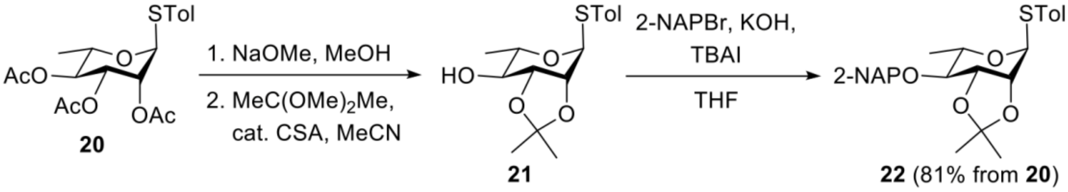

Compound 22. To a solution of compound **20** (50.2 g, 126.0 mmol) in MeOH (250.0 mL), $NaOCH_3$ (340 mg, 6.3 mmol) was added under nitrogen atmosphere. The reaction mixture was allowed to stir at room temperature for 1 h, and TLC analysis indicated the complete conversion of starting materials into products. DOWEX 50 (H^+ exchange resin) was added to quench the reaction, the whole mixture was filtered through a pad of Celite® and the solids were washed with MeOH. The filtrate was concentrated *in vacuo* to afford the crude product, which was directly used for the next reaction without further purification.

The crude residue was dissolved in anhydrous CH_3CN (500 mL), then 2,2-DMP (23.3 mL, 189.0 mmol) and CSA (740 mg, 3.2 mmol) were added under nitrogen atmosphere. The resulting reaction mixture was allowed to stir at room temperature for 2 h, and then, the reaction mixture was poured into the sat. $NaHCO_{3(aq)}$. The crude compound was extracted with EtOAc (200 mL × 2), and the combined organic layers were washed with brine, dried over anhydrous $MgSO_4$, filtered, and concentrated *in vacuo* to afford **21**. The obtained crude residue was dried on high vacuum for 1 h, which was directly used for next reaction without further purification.

The obtained residue **21** was dissolved in anhydrous THF (500 mL), KOH (11.4 g, 202.0 mmol), TBAI (11.7 g, 31.7 mmol), and 2-NAPBr (33.6 g, 152.0 mmol) were added at 85 °C under nitrogen atmosphere. The reaction mixture was allowed to stir at same temperature for 24 h and then cooled to room temperature, filtered through a pad of Celite® and the filtrate was concentrated *in vacuo*. The crude residue was dissolved in EtOAc and washed with H_2O, organic layer was dried over anhydrous $MgSO_4$, filtered, and concentrated *in vacuo*. The crude residue was purified by flash column chromatography (EtOAc/Hex =1/7) on silica gel to afford the desired compound **22** (46.2 g, 81%, over 3 steps) as a white solid. $[\alpha]_D^{20}$ = -255.4 (*c* = 0.3, $CHCl_3$); IR (thin film in KBr) *v* 2985, 2933, 2893, 1107, 1089, 1074, 809 cm^{-1}; ($CDCl_3$, 600 MHz) δ = 7.82-7.79 (m, 4H, Ar-H), 7.48-7.43 (m, 3H, Ar-H), 7.33 (d, *J* = 7.8 Hz, 2H, Ar-H), 7.09 (d, *J* = 7.7 Hz, 2H, Ar-H), 5.64 (s, 1H, H-1), 5.04-4.77 (ABq, *J* = 11.7 Hz, 2H, $ArCH_2$), 4.35-4.32 (m, 2H, H-2, H-3), 4.19-4.14 (m, 1H, H-5), 3.31 (dd, *J* = 5.7 Hz, *J* = 9.7 Hz, H-4), 2.31 (s, 3H, Ar-CH_3), 1.49 (s, 3H, CCH_3), 1.37 (s, 3H, CCH_3), 1.23 (d, *J* = 6.2 Hz, 3H, H-6); ^{13}C NMR ($CDCl_3$, 150 MHz) δ = 138.0 (C), 135.8 (C), 133.4 (C), 133.2 (C), 132.7 (2C, CH),

130.0 (2C, CH), 129.9 (C), 128.2 (CH), 128.1 (CH), 127.9 (CH), 127.0 (CH), 126.3 (2C, CH), 126.1 (CH), 109.7 (C), 84.4 (CH), 81.6 (CH), 78.7 (CH), 76.8 (CH), 73.4 (CH_2), 66.3 (CH), 28.2 (CH_3), 26.7 (CH_3), 21.3 (CH_3), 17.9 (CH_3); HRMS *m/z* $(M+Na)^+$ calcd for $C_{27}H_{30}O_4SNa^+$ 473.1762, found 473.1762.

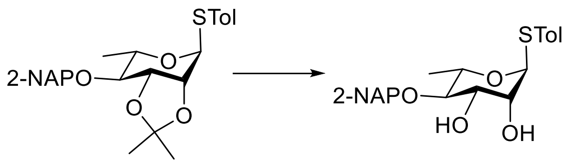

22 **23**

Compound 23. To a solution of compound **22** (39.3 g, 87.1 mmol) in H_2O/MeOH (400.0 mL, 1/9), 2 N HCl solution was added, and the pH was adjusted to 2. The resulting reaction mixture was allowed to stir at room temperature for 8 h. Then, the reaction was diluted with CH_2Cl_2, washed with sat. $NaHCO_{3(aq)}$, and H_2O, organic layer was dried over anhydrous $MgSO_4$, filtered, and concentrated *in vacuo*. The crude residue was purified by flash column chromatography (EtOAc/Hex =1/2) on silica gel to afford the desired product **23** (32.9 g, 92%) as a white solid. $[\alpha]_D^{20}$ = -221.8 (c = 0.5, $CHCl_3$); IR (thin film in KBr) v 3303, 2900, 1099, 1083, 1057, 810, 790 cm^{-1}; ^1H NMR ($CDCl_3$, 600 MHz) δ = 7.85-7.79 (m, 4 H, Ar-H), 7.47-7.46 (m, 3H, Ar-H), 7.32 (d, J = 7.3 Hz, 2H, Ar-H), 7.09 (d, J = 7.6 Hz, 2H, Ar-H), 5.38 (s, 1H, H-1), 4.92, 4.88 (ABq, J = 11.7 Hz, 2H, $ArCH_2$), 4.27-4.22 (m, 1H, H-5), 4.16 (s, 1H, H-2), 3.96-3.93 (m, 1H, H-3), 3.45 (t, J = 9.2 Hz, H-4), 2.46 (d, J = 3.6 Hz, 1H, OH), 2.36 (d, J = 5.1 Hz, 1H, OH), 2.30 (s, 3H, Ar-CH_3), 1.36 (d, J = 6.2 Hz, 3H, H-6); ^{13}C NMR ($CDCl_3$, 150 MHz) δ = 137.8 (C), 135.7 (C), 133.4 (C), 133.2 (C), 132.2 (2C, CH), 130.4 (C), 130.0 (2C, CH), 128.6 (CH), 128.1 (CH), 127.9 (CH), 126.9 (CH), 126.4 (CH), 126.2 (CH), 126.0 (CH), 88.0 (CH), 82.0 (CH), 75.3 (CH_2), 72.8 (CH), 72.1 (CH), 68.7 (CH), 21.3 (CH_3), 18.1 (CH_3); HRMS *m/z* $(M+Na)^+$ calcd for $C_{24}H_{26}O_4SNa^+$ 433.1444, found 433.1454.

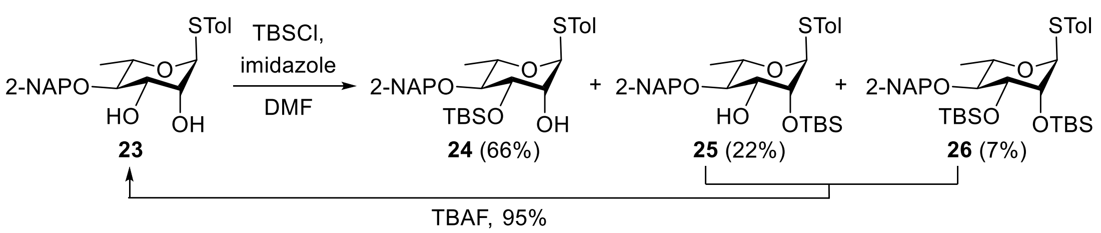

Compound 24. To a solution of compound **23** (20.2 g, 49.2 mmol) in DMF (160.0 mL), imidazole (6.0 g, 88.6 mmol) and TBSCl (8.9 g, 59.1 mmol) were added at 0 °C. The reaction

was allowed to stir at the same temperature for 16 h, then MeOH was added to quench the reaction, and concentrated under reduced pressure. The resulting residue was dissolved in EtOAc and H_2O, the aqueous layer was extracted twice with EtOAc, after which the combined organic layers were washed with brine, dried over anhydrous $MgSO_4$, filtered, and concentrated *in vacuo*. The crude residue was purified by flash column chromatography (EtOAc/n-Hexane =1/9) on silica gel to afford the desired compound **24** (66%) as a colorless syrup, compound **25** (22%) and compound **26** (7%). The undesired compounds **25** and **26** were further converted back into starting material **23** (To a solution of compounds **25** and **26** in THF (10 mL/g), TBAF was added, and allowed to stir for 1 h at RT. Then, the reaction was quenched with sat. $NaHCO_{3(aq)}$ and extracted with EtOAc (100 mL × 2). The combined organic layers were washed with brine, dried over $MgSO_4$, filtered, and concentrated *in vacuo*. The crude residue was purified by flash column chromatography (EtOAc/Hex =1/9) on silica gel to get the compound **23** (95%)**)**. $[\alpha]_D^{22}$ = -147.7 (c = 1.0, $CHCl_3$); IR (thin film in KBr) ν 3557, 3055, 3021, 2953, 2928, 2856, 1602, 1492, 1462, 1380, 1254, 1099, 1088, 866, 838, 809, 778 cm^{-1}; ^1H NMR ($CDCl_3$, 600 MHz) δ = 7.82-7.77 (m,filtered,,,-H), 7.77 (s, 1 H, Ar-H), 7.48-7.42 (m, 3 H, Ar-H), 7.33 (d, J = 7.5 Hz, 2 H, Ar-H), 7.09 (d, J = 7.7 Hz, 2 H, Ar-H), 5.45 (s, 1 H, H-1), 4.98-4.75 (ABq, J = 11.4 Hz, 2 H, $ArCH_2$), 4.24-4.19 (m, 1 H, H-5), 4.06-4.04 (m, 2 H, H-2, H-3), 3.47 (t, J = 8.7 Hz, H-4), 2.79 (s, 1 H, OH), 2.30 (s, 3H, Ar-CH_3), 1.27 (d, J = 6.2 Hz, 3 H, H-6), 0.94 (s, 9 H, TBS-*t*Bu), 0.15 (s, 3H, TBS-CH_3), 0.13 (s, 3H, TBS-CH_3); ^{13}C NMR ($CDCl_3$, 150 MHz) δ = 137.7 (C), 135.9 (C), 133.4 (C), 133.1 (C), 132.2 (2C, CH), 130.4 (C), 130.0 (2C, CH), 128.2 (CH), 128.1 (CH), 127.8 (CH), 126.4 (CH), 126.2 (CH), 126.0 (CH), 125.8 (CH), 87.3 (CH), 81.6 (CH), 75.6 (CH_2), 73.7 (CH), 73.6 (CH), 68.9 (CH), 26.0 (3C, CH_3), 21.3 (CH_3), 18.1 (C), 17.9 (CH_3), -4.3 (CH_3), -4.4 (CH_3); HRMS *m/z* $(M+Na)^+$ calcd for $C_{30}H_{40}O_4SSiNa^+$ 547.2309, found 547.2303.

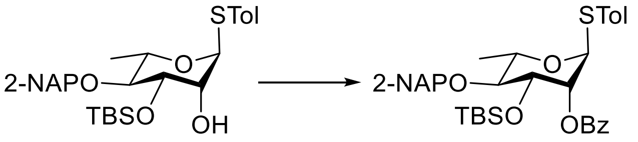

24 **16**

Compound 16. To a solution of compound **24** (45.1 g, 85.8 mmol) in a mixed solvent of CH_2Cl_2/pyridine (250 mL, 1/3), BzCl (14.9 mL, 128.7 mmol) was added. The reaction mixture was allowed to stir at room temperature for 6 h, TLC analysis indicates the complete conversion

of the starting material into products. The solvents were then removed under reduced pressure, and the crude residue was dissolved in EtOAc and washed with H_2O. The organic layer was dried over anhydrous $MgSO_4$, filtered, and concentrated *in vacuo*. The crude residue was purified by flash column chromatography (EtOAc/Hex = 1/8) on silica gel to afford the desired compound **16** (50.2 g, 93%) as a yellow syrup. $[\alpha]_D^{20}$ = -160.4 (c = 0.4, $CHCl_3$); IR (thin film in KBr) ν 2953, 2929, 2857, 1723, 1267, 1091, 867, 838, 710 cm^{-1}; ^1H NMR ($CDCl_3$, 600 MHz) δ = 8.03 (d, J = 7.6 Hz, 2 H, Ar-H), 7.83-7.79 (m, 3H, Ar-H), 7.79 (s, 1H, Ar-H), 7.58-7.55 (m, 1H, Ar-H), 7.49-7.42 (m, 5H, Ar-H), 7.35 (d, J = 8.0 Hz, 2 H, Ar-H), 7.08 (d, J = 7.9 Hz, 2 H, Ar-H), 5.52 (s, 1 H, H-2), 5.41 (s, 1 H, H-1), 5.08-4.83 (ABq, J = 11.5 Hz, 2 H, $ArCH_2$), 4.31-4.26 (m, 1 H, H-5), 4.23 (dd, J = 3.2 Hz, J = 9.0 Hz, H-3), 3.60 (t, 1 H, J = 9.2 Hz, H-4), 2.29 (s, 3 H, Ar-CH_3), 1.31 (d, J = 6.2 Hz, 3 H, H-6), 0.82 (s, 9 H, *t*Bu), 0.15 (s, 3 H, CH_3), 0.11 (s, 3 H, CH_3)；^{13}C NMR ($CDCl_3$, 150 MHz) δ = 166.0 (C), 138.0 (C), 136.0 (C), 133.5 (C), 133.3 (CH), 133.2 (C), 132.5 (2C, CH), 130.4 (C), 130.3 (C), 130.1 (2C, CH), 130.0 (2C, CH), 128.6 (2C, CH), 128.3 (CH), 128.2 (CH), 127.9 (CH), 126.7 (CH), 126.3 (CH), 126.11 (CH), 126.09 (CH), 86.7 (CH), 81.7 (CH), 75.7 (CH_2), 75.4 (CH), 72.1 (CH), 69.4 (CH), 26.0 (3C, CH_3), 21.3 (CH_3), 18.2 (CH_3), 18.0 (C), -4.45 (CH_3), -4.51 (CH_3); HRMS m/z $(M+Na)^+$ calcd for $C_{37}H_{44}O_5SSiNa^+$ 651.2571 found 651.2583.

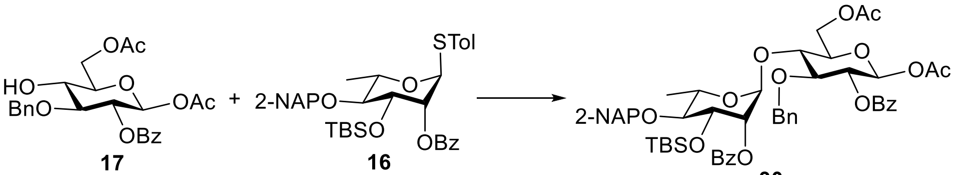

Compound 30. To a solution of donor **16** (1.0 g, 1.6 mmol), and acceptor **17** (802 mg, 1.8 mmol) in dry CH_2Cl_2 (16.0 mL), freshly activated AW-500 MS (1.0 g) were added under nitrogen, and the reaction was allowed to stir at room temperature for 1 h. The reaction flask was cooled to –78 °C, then NIS (393 mg, 1.8 mmol) and TfOH (30 μL, 300.0 μmol) were added. The reaction flask was gradually warmed up to –60 °C for 3 h. When TLC (EtOAc/Hexane, 1/2.3, Rf = 0.39) analysis indicated the products formation, Et_3N was added to quench the reaction. The whole mixture was filtered through a pad of Celite®, solids were washed with CH_2Cl_2, and the filtrate was washed with 10% $Na_2S_2O_3$ solution and water. The organic layer was dried over anhydrous $MgSO_4$, filtered, and concentrated *in vacuo*. The resulting crude residue was purified by column chromatography (EtOAc/Hexane, 1/4) on silica

gel to afford the desired product **30** (126 mg, 82%) as a white solid. $[\alpha]_D^{24}$ = +10.5 (*c* = 1.0, $CHCl_3$); IR (thin film in KBr) ν 2930, 2857, 1729, 1602, 1451, 1365, 1316, 1266, 1228, 1176, 1106, 1069, 1028, 870, 838, 777, 754, 711, 600, 477 cm^{-1}; ^1H NMR (600 MHz, $CDCl_3$) δ = 8.04 (d, *J* = 7.6 Hz, 2H, Ar-H), 7.98 (d, *J* = 7.7 Hz, 2H, Ar-H), 7.82-7.79 (m, 3H, Ar-H), 7.76 (s, 1H, Ar-H), 7.59-7.53 (m, 2H, Ar-H), 7.46-7.40 (m, 7H, Ar-H), 7.15 (d, *J* = 5.9 Hz, 2H, Ar-H), 7.04-7.02 (m, 3H, Ar-H), 5.85 (d, *J* = 7.8 Hz, 1H, H-1), 5.42 (t, *J* = 8.4 Hz, 1H, H-2), 5.16 (bs, 1H, H-2'), 5.06-5.04 (m, 2H, H-1', Ar-CH_2), 4.78-4.75 (m, 2H, Ar-CH_2), 4.66 (d, *J* = 10.3 Hz, 1H, Ar-CH_2), 4.59 (d, *J* = 12.2 Hz, 1H, H-6), 4.26-4.22 (m, 2H, H-3', H-6), 4.03 (t, *J* = 9.1 Hz, 1H, H-4), 4.00-3.97 (m, 1H, H-5'), 3.89 (t, *J* = 8.5 Hz, 1H, H-3), 3.82-3.80 (m, 1H, H-5), 3.49 (t, *J* = 9.1 Hz, 1H, H-4'), 2.08 (s, 3H, CH_3), 1.98 (s, 3H, CH_3), 1.05 (d, *J* = 5.3 Hz, 3H, H-6'), 0.81 (s, 9H, TBS), 0.09 (s, 3H, TBS), 0.08 (s, 3H, TBS); ^{13}C NMR (150 MHz, CDCl3) δ = 170.7 (C), 169.5 (C), 166.2 (C), 165.2 (C), 137.3 (C), 136.1 (C), 133.7 (CH), 133.44 (C), 133.38 (CH), 133.1 (C), 130.1 (C), 130.00 (CH), 129.98 (CH), 129.4 (C), 128.8 (CH), 128.6 (CH), 128.5 (CH), 128.4 (CH), 128.3 (CH), 128.2 (CH), 128.1 (CH), 127.9 (CH), 127.8 (CH), 126.6 (CH), 126.3 (CH), 126.2 (CH), 126.10 (CH), 126.06 (CH), 98.2 (CH), 92.2 (CH), 81.4 (CH), 81.3 (CH), 77.4 (CH), 75.5 (CH_2), 75.4 (CH), 75.2 (CH_2), 74.3 (CH), 73.9 (CH), 72.8 (CH), 71.2 (CH), 69.0 (CH), 62.6 (CH_2), 25.9 (CH_3), 21.1 (CH_3), 21.0 (CH_3), 18.1 (CH_3), 18.0 (C), -4.4 (CH_3), -4.5 (CH_3); HRMS m/z $(M+Na)^+$ calcd for $C_{54}H_{62}O_{14}Si_1Na^+$ 985.3801 found 985.3821.

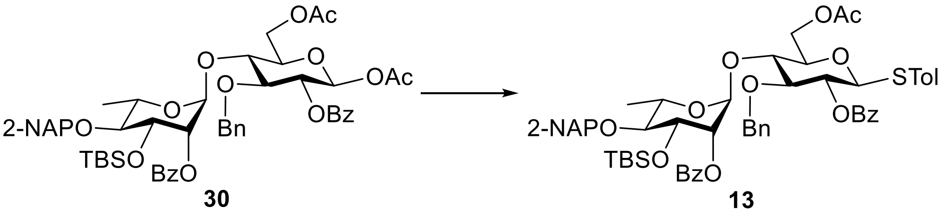

30 **13**

Compound 13. To a solution of compound **30** (300 mg, 310.0 μmol) in anhydrous CH_2Cl_2 (3.1 mL), TMSSTol (290 μL, 1.2 mmol) and ZnI_2 (179 mg, 560.0 μmol) were added at 0 °C under nitrogen atmosphere, and the reaction mixture was allowed to stir at room temperature for 3 h. TLC (EtOAc/Hexane = 1/4, Rf = 0.48) analysis indicated the complete conversion of starting materials into products. The reaction mixture was filtered through a pad of Celite® and the solids were washed with CH_2Cl_2. The filtrate was washed with sat. $NaHCO_{3(aq)}$ and H_2O. Organic layer was dried over anhydrous $MgSO_4$, filtered, and concentrated *in vacuo*. The crude residue was purified by column chromatography (EtOAc/Hexane = 1/5) on silica gel to afford

the desired product **13** (298 mg, 93%) as a white solid. $[\alpha]_D^{28}$ = +1.1 (*c* = 1.0, $CHCl_3$); IR (thin film in KBr) ν 2929, 2857, 1727, 1601, 1492, 1451, 1362, 1316, 1265, 1096, 1069, 1028, 870, 838, 811, 777, 753, 710, 477 cm^{-1}; ^1H NMR (600 MHz, $CDCl_3$) δ = 8.05 (d, *J* = 7.7 Hz, 4H, Ar-H), 7.84-7.79 (m, 3H, Ar-H), 7.76 (s, 1H, Ar-H), 7.60-7.55 (m, 2H, Ar-H), 7.48-7.42 (m, 7H, Ar-H), 7.36 (d, *J* = 7.9 Hz, 2H, Ar-H), 7.15 (d, *J* = 6.5 Hz, 2H, Ar-H), 7.07 (d, *J* = 7.9 Hz, 2H, Ar-H), 7.04-7.01 (m, 3H, Ar-H), 5.27 (t, J = 9.4 Hz, 1H, H-2), 5.17 (bs, 1H, H-2'), 5.06-5.04 (m, 2H, H-1', Ar-CH_2), 4.78 (d, *J* = 11.5 Hz, 1H, Ar-CH_2), 4.74 (d, *J* = 10.1 Hz, 2H, H-1, Ar-CH_2), 4.71 (d, *J* = 12.0 Hz, 1H, Ar-CH_2), 4.64 (d, *J* = 10.3 Hz, 1H, Ar-CH_2), 4.24-4.21 (m, 2H, H-3', Ar-CH_2), 4.00-3.96 (m, 1H, H-5'), 3.92 (t, *J* = 9.3 Hz, 1H, H-4), 3.85 (t, *J* = 8.8 Hz, 1H, H-3), 3.67 (m, 1H, H-5), 3.49 (t, *J* = 9.2 Hz, 1H, H-4'), 2.31 (s, 3H, CH_3), 2.08 (s, 3H, CH_3), 1.01 (d, *J* = 6.1 Hz, 3H, H-6'), 0.82 (s, 9H, TBS), 0.1 (s, 3H, TBS), 0.09 (s, 3H, TBS); ^{13}C NMR (150 MHz, $CDCl_3$) δ = 170.6 (C), 166.1 (C), 165.2 (C), 138.5 (C), 137.3 (C), 136.1 (C), 133.9 (CH), 133.5 (CH), 133.4 (C), 133.3 (CH), 133.0 (C), 130.1 (C), 130.0 (CH), 129.99 (CH), 129.95 (C), 129.7 (CH), 128.6 (CH), 128.5 (CH), 128.4 (C), 128.32 (CH), 128.3 (CH), 128.1 (CH), 128.0 (CH), 127.8 (CH), 127.7 (CH), 126.4 (CH), 126.2 (CH), 126.1 (CH), 126.0 (CH), 98.0 (CH), 86.4 (CH), 82.8 (CH), 81.4 (CH), 77.6 (CH), 75.5 (CH), 75.4 (CH_2), 73.9 (CH), 72.9 (CH), 71.1 (CH), 68.9 (CH), 62.8 (CH_2), 25.7 (CH), 21.3 (CH_3), 21.0 (CH_3), 18.02 (CH_3), 18.01 (CH_3), -4.4 (CH_3), -4.5 (CH_3); HRMS m/z $(M+H)^+$ calcd for $C_{59}H_{67}O_{12}SSi^+$ 1027.4117 found 1027.4137.

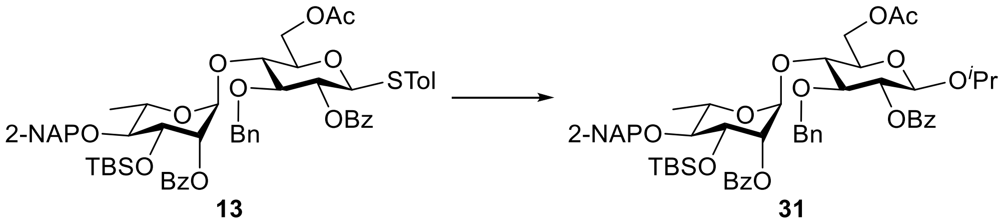

13 **31**

Compound 31. To a solution of donor **13** (2.2 g, 2.1 mmol), and acceptor (^{i}PrOH, 410 µL, 5.4 mmol) in dry CH_2Cl_2 (21.4 mL), freshly activated AW-500 MS (1.0 g) were added under N_2 atmosphere, and the reaction was allowed to stir at room temperature for 0.5 h. Then, the reaction flask was cooled to –40 °C, NIS (39.3 mg, 180.0 µmol) and TfOH (3 µL, 30.0 µmmol) were added. The flask was gradually warmed up to –20 °C for 3 h and TLC (EtOAc/Hexane = 1/4, Rf = 0.36) analysis indicated the product formation. Afterwards, Et_3N was added to quench the reaction. The whole mixture was filtered through a pad of Celite®, solids were washed with CH_2Cl_2, and the filtrate was washed with 10% $Na_2S_2O_3$ solution and water. The organic layer

was dried over anhydrous $MgSO_4$, filtered, and concentrated *in vacuo*. The crude residue was purified by column chromatography (EtOAc/Hexane, 1/1.3) on silica gel to afford the desired product **31** (1.69 g, 82%) as a white solid. $[\alpha]_D^{28}$ = +3.1 (*c* = 1.0, $CHCl_3$); IR (thin film in KBr) ν 2927, 2855, 1727, 1452, 1363, 1266, 1107, 1070, 838, 754, 711 cm^{-1}; ^1H NMR (600 MHz, $CDCl_3$) δ = 8.04 (d, *J* = 7.7 Hz, 2H, Ar-H), 7.99 (d, *J* = 7.7 Hz, 2H, Ar-H), 7.82-7.78 (m, 3H, Ar-H), 7.75 (s, 1H, Ar-H), 7.57 (t, *J* = 7.3 Hz, 1H, Ar-H), 7.52 (t, *J* = 7.3 Hz, 1H, Ar-H), 7.47-7.39 (m, 7H, Ar-H), 7.17 (m, 2H, Ar-H), 7.02 (m, 3H, Ar-H), 5.24 (t, *J* = 8.3 Hz, 1H, H-2), 5.16 (bs, 1H, H-2'), 5.05 (m, 2H, H-1', $ArCH_2$), 4.77 (d, *J* = 11.5 Hz, 1H, Ar-CH_2), 4.73 (d, *J* = 10.4 Hz, 1H, Ar-CH_2), 4.65 (d, *J* = 10.5 Hz, 1H, Ar-CH_2), 4.62 (d, *J* = 7.8 Hz, 1H, H-1), 4.60-4.58 (m, 1H, H-6), 4.24-4.21 (m, 2H, H-3', H-6), 4.01-3.95 (m, 2H, H-5', H-4), 3.88-3.81 (m, 2H, ^{i}Pr), 3.63-3.61 (m, 1H, H-5), 3.49 (t, *J* = 9.2 Hz, 1H, H-4') , 2.06 (s, 3H, CH_3), 1.16 (d, *J* = 6.2 Hz, 3H, ^{i}Pr), 1.02 (d, *J* = 6.2 Hz, 3H, H-6'), 0.99 (d, *J* = 6.1 Hz, 3H, ^{i}Pr), 0.81 (s, 9H, TBS), 0.09 (s, 3H, TBS), 0.07 (s, 3H, TBS); ^{13}C NMR (150 MHz, $CDCl_3$) δ = 207.2 (C), 170.8 (C), 166.1 (C), 165.2 (C), 137.6 (C), 136.1 (C), 133.4 (C), 133.3 (CH), 133.2 (CH), 133.1 (C), 130.1 (C), 130.0 (CH), 129.8 (CH), 128.6 (CH), 128.3 (CH), 128.1 (CH), 128 (CH), 127.9 (CH), 127.6 (CH), 126.5 (CH), 126.2 (CH), 126.1 (CH), 126.0 (CH), 100.0 (CH), 98.0 (CH), 81.5 (CH), 81.4 (CH), 77.4 (CH), 75.7 (CH), 75.4 (CH_2), 74.7 (CH_2), 74.3 (CH), 73.9 (CH), 73.5 (CH), 72.9 (CH), 71.2 (CH), 68.8 (CH), 63.0 (CH_2), 31.1 (CH_3), 25.9 (CH_3), 23.4 (CH_3), 22.1 (CH_3), 21.1 (CH_3), 18.1 (CH_3), 18.0 (CH_3), -4.4 (CH_3); HRMS m/z $(M+Na)^+$ calcd for $C_{55}H_{66}O_{13}SiNa^+$ 985.4165 found 985.4163.

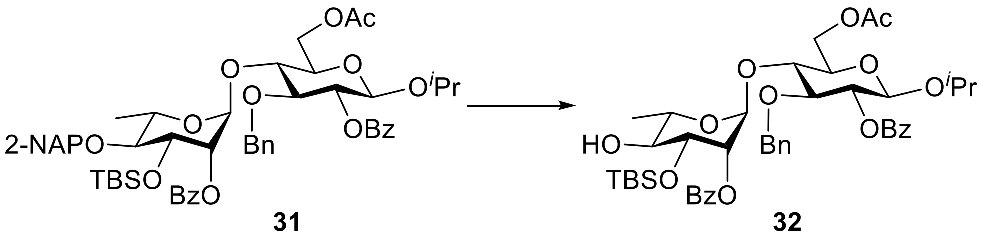

Compound 32. To a solution of compound **31** (300 mg, 310.0 μmol) in a mixed solvent CH_2Cl_2/H_2O (21.0 mL, 19/1), DDQ (212 mg, 940.0 μmol) was added at 0 °C. The reaction temperature was gradually warmed up to 25 °C for 3 hours, TLC (EtOAc/Hexane = 1/4, Rf = 0.33) analysis indicated the complete conversion of starting materials into products. The reaction mixture was filtered through a pad of Celite®, solids were washed with CH_2Cl_2, and the filtrate was washed with sat. $NaHCO_{3(aq)}$. The organic layer was dried over anhydrous $MgSO_4$, filtered, and concentrated *in vacuo*. The resulting crude residue was purified by flash

column chromatography (EtOAc/Hex = 1/5) on silica gel to afford the desired product **32** (251 mg, 92%) as a white solid. $[\alpha]_D^{27}$ = +14.1 (*c* = 1.0, $CHCl_3$); IR (thin film in KBr) ν 3536, 2927, 2855, 1723, 1452, 1370, 1315, 1269, 1110, 1070, 872, 838, 779, 711 cm^{-1}; ^1H NMR (600 MHz, $CDCl_3$) δ = 8.00-7.98 (m, 4H, Ar-H), 7.55-7.52 (m, 2H, Ar-H), 7.43-7.39 (m, 4H, Ar-H), 7.19-7.17 (m, 2H, Ar-H), 7.13-7.09 (m, 3H, Ar-H), 5.25 (t, *J* = 8.3 Hz, 1H, H-2), 5.12 (t, *J* = 1.7 Hz, 1H, H-2'), 5.06 (bs, 1H, H-1'), 4.72 (d, *J* = 10.6 Hz, 1H, Ar-CH_2), 4.67 (d, *J* = 10.6 Hz, 1H, Ar-CH_2) , 4.63 (d, *J* = 7.8 Hz, 1H, H-1), 4.58 (dd, *J* = 1.9, 12.1 Hz, 1H, H-6), 4.23 (dd, *J* = 4.3, 12.1 Hz, 1H, H-6), 3.98-3.95 (m, 2H, H-3', H-4), 3.94-3.89 (m, 1H, H-5'), 3.88-3.83 (m, 2H, H-3, ^{i}Pr-CH), 3.65-3.63 (m, 1H, H-5), 3.59 (td, *J* = 2.6, 9.3 Hz, 1H, H-4'), 2.07 (s, 3H, CH_3), 1.94 (d, *J* = 2.8 Hz, 1H, OH), 1.16 (d, *J* = 6.2 Hz, 3H, ^{i}Pr-CH_3), 1.06 (d, *J* = 6.1 Hz, 3H, H-6'), 1.00 (d, *J* = 6.1 Hz, 3H, ^{i}Pr-CH_3), 0.79 (s, 9H, TBS), 0.11 (s, 3H, TBS), 0.05 (s, 3H, TBS); ^{13}C NMR (150 MHz, $CDCl_3$) δ = 170.8 (C), 166.1 (C), 165.2 (C), 137.7 (C), 133.3 (CH), 133.2 (CH), 130.1 (C), 129.9 (CH), 129.8 (CH), 128.6 (CH), 128.5 (CH), 128.3 (CH), 128.2 (CH), 127.7 (CH), 100.0 (CH), 98.2 (CH), 81.4 (CH), 75.6 (CH), 74.6 (CH_2), 74.3 (CH), 73.6 (CH), 73.4 (CH), 73.1 (CH), 72.9 (CH), 71.3 (CH), 68.9 (CH), 62.9 (CH_2), 25.7 (CH_3), 23.4 (CH_3), 22.1 (CH_3), 21.1 (CH_2), 18.1 (C), 17.7 (CH_3), -4.4 (CH_3), -4.5 (CH_3); HRMS m/z $(M+Na)^+$ calcd for $C_{44}H_{58}O_{13}SiNa^+$ 845.3539 found 845.3536.

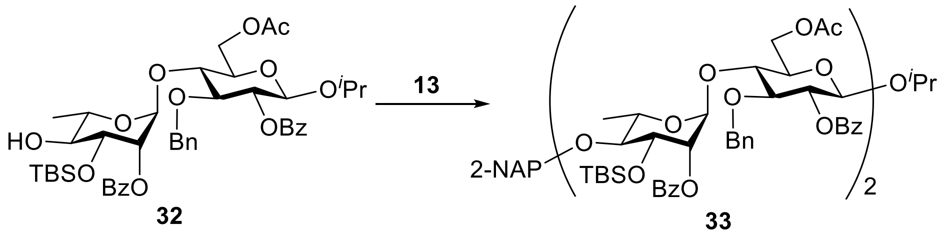

32 **33**

Compound 33. A mixture of donor **13** (674 mg, 0.6 mmol), acceptor **32** (450 mg, 0.5 mmol) and freshly activated AW-500 MS (100 mg) in dry CH_2Cl_2 (5.4 mL) was stirred at room temperature for 1 h under nitrogen atmosphere, and then cooled to –78 °C. NIS (135 mg, 0.60 mmol) and TfOH (14 μL, 0.16 mmol) were added to the reaction mixture, and the reaction temperature was gradually warmed up to –60 °C for 3 hours and TLC (EtOAc/Hexane, 1/4, Rf =0.18) analysis indicates the products formation, then Et_3N was added to quench the reaction. The mixture was filtered through a pad of Celite®, solids were washed with CH_2Cl_2, and the filtrate was washed with 20% $Na_2S_2O_3$ solution and water. The organic layer was dried over anhydrous $MgSO_4$, filtered, and concentrated *in vacuo*. The crude residue was purified by column chromatography (EtOAc/Hexane, 1/5) on silica gel to afford the desired product **33**

(751 mg, 80%) as a white solid $[\alpha]_D^{29}$ = +8.1 (*c* = 1.0, $CHCl_3$); IR (thin film in KBr) ν 2931, 2886, 2858, 1729, 1602, 1492, 1452, 1384, 1362, 1316, 1226, 1107, 1069, 1028, 935, 869, 838, 778, 754, 711 cm^{-1}; ^1H NMR (600 MHz, $CDCl_3$) δ = 8.04 (d, *J* = 7.9 Hz, 2H, Ar-H), 7.99-7.96 (m, 6H, Ar-H), 7.82-7.78 (m, 3H, Ar-H), 7.74 (s, 1H, Ar-H), 7.59-7.55 (m, 2H, Ar-H), 7.54-7.50 (m, 2H, Ar-H), 7.46-7.43 (m, 6H, Ar-H), 7.41-7.35 (m, 5H, Ar-H), 7.13-7.09 (m, 6H, Ar-H), 7.05-6.98 (m, 4H, Ar-H), 5.32 (t, *J* = 7.7 Hz, 1H, H2''), 5.19 (t, *J* = 8.4 Hz, 1H, H2), 5.16 (bs, 1H, H2'''), 5.11 (d, *J* = 6.8 Hz, 1H, H1''), 5.06 (s, 1H, H1'), 5.04-5.02 (m, 3H, H2', H1''', CH_2), 4.76-4.73 (m, 2H, CH_2, CH_2), 4.71-4.68 (m, 2H, H6''', CH_2), 4.62 (d, *J* = 10.3 Hz, 1H, CH_2, CH_2), 4.56-4.52 (m, 3H, H6, H1, CH_2), 4.23-4.19 (m, 2H, H6'', H3'''), 4.15 (dd, *J* = 3.8, 12.1 Hz, 1H, H6), 4.08 (d, *J* = 8.3 Hz, 1H, H3'), 3.99 (t, *J* = 9.2 Hz, 1H, H4''), 3.96-3.92 (m, 1H, H5'''), 3.91-3.87 (m, 2H, H4, H4'), 3.86-3.79 (m, 2H, H5', ^{i}Pr-CH), 3.77-3.74 (m, 2H, H3, H3''), 3.63 (d, *J* = 9.6 Hz, 1H, H5''), 3.54 (d, *J* = 8.6 Hz, 1H, H5), 3.48 (t, *J* = 9.2 Hz, 1H, H4'''') 2.03 (s, 3H, CH_3), 1.99 (s, 3H, CH_3), 1.92 (d, *J* = 2.6 Hz, 1H, OH), 1.13 (d, *J* = 6.2 Hz, 3H, ^{i}Pr-CH_3), 1.04 (d, *J* = 6.1 Hz, 3H, H6'), 0.99 (d, *J* = 5.9 Hz, 3H, H6'''), 0.97 (d, *J* = 6.1 Hz, 3H, ^{i}Pr-CH_3), 0.78 (s, 18H, TBS), 0.1 (s, 3H, TBS), 0.04 (s, 3H, TBS), -0.03 (s, 3H, TBS), -0.06 (s, 3H, TBS); ^{13}C NMR (150 MHz, $CDCl_3$) δ = 170.8 (C), 170.6 (C), 166.2 (C), 166.0 (C), 165.2 (C), 165.1 (C), 137.6 (CH), 136.1 (CH), 133.44 (CH), 133.43 (CH), 133.39 (CH), 133.3 (CH), 133.2 (C), 133.1 (CH), 130.2 (C), 130.1 (C), 130.02 (CH), 130.0 (CH), 129.97 (CH), 129.9 (CH), 129.8 (CH), 128.7 (CH), 128.6 (CH), 128.57 (CH), 128.5 (CH), 128.49 (CH), 128.4 (CH), 128.3 (CH), 128.2 (CH), 128.16 (CH), 128.1 (CH), 128.07 (CH), 127.9 (CH), 127.7 (CH), 127.6 (CH), 126.5 (CH), 126.2 (CH), 126.1 (CH), 126.04 (CH), 100.0 (CH), 99.5 (CH), 98.1 (CH), 98.0 (CH), 81.8 (CH), 81.5 (CH), 81.2 (CH), 77.4 (CH), 76.3 (CH), 76.0 (CH), 75.4 (CH_2), 74.87 (CH_2), 74.83 (CH_2), 74.5 (CH), 74.4 (CH), 73.9 (CH), 73.6 (CH), 73.3 (CH), 73.0 (CH), 72.9 (CH), 71.1 (CH), 70.9 (CH), 68.8 (CH), 68.3 (CH), 62.8 (CH_2), 62.5 (CH_2), 26.0 (CH), 25.9 (CH), 23.3 (CH), 22.1 (CH), 21.0 (CH), 20.9 (CH), 18.06 (CH), 18.02 (C), 17.96 (CH), 17.9 (CH), -4.2 (CH_3), -4.38 (CH_3), -4.40 (CH_3), -4.5 (CH_3); HRMS m/z $(M+H)^+$ calcd for $C_{95}H_{113}O_{26}Si_2^+$ 1726.784 found 1726.7461.

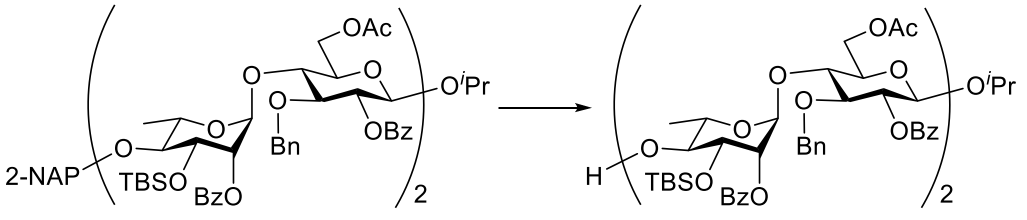

33 **34**

Compound 34. To a solution of compound **33** (600 mg, 650.0 μmol) in CH_2Cl_2/H_2O (42 mL,

CH_2Cl_2/H_2O = 30/1), DDQ (236 mg, 1.1 mmol) was added at 0 °C. The temperature of the reaction was gradually increased to room temperature and allowed to stir for 3 h. TLC (EtOAc/Hexane = 1/2.3, Rf = 0.42) analysis indicates the complete conversion of starting materials into products. The whole mixture was filtered through a pad of Celite® and washed with CH_2Cl_2, and the filtrate was washed with 10% $Na_2S_2O_3$ solution and saturated $NaHCO_{3(aq)}$. The organic layer was dried over anhydrous $MgSO_4$, filtered, and concentrated *in vacuo*. The crude residue was purified by column chromatography (EtOAc/Hexane = 1/2) on silica gel to afford the desired compound **34** (326 mg, 85%) as a white solid. $[\alpha]_D^{28.8}$ = +6.0 (c = 1.0, $CHCl_3$); IR (thin film in KBr) ν 3536, 2931, 2858, 1726, 1602, 1452, 1362, 1316, 1266, 1177, 1095, 1069, 1028, 925, 870, 838, 778, 754, 711 cm^{-1}; ^1H NMR (600 MHz, $CDCl_3$) δ = 8.00-7.96 (m, 8H, Ar-H), 7.57-7.51 (m, 4H, Ar-H), 7.46-7.36 (m, 8H, Ar-H), 7.15-7.09 (m, 9H, Ar-H), 7.05-7.03 (m, 1H, Ar-H), 5.33 (t, *J* = 8.1 Hz, 1H, H2''), 5.20 (t, *J* = 8.5 Hz, 1H, H2), 5.12-5.11 (m, 2H, H1'', H2'''), 5.07 (bs, 1H, H1'), 5.02 (bs, 2H, H2', H1'''), 4.73 (m, 2H, CH_2, CH_2), 4.68 (d, *J* = 10.6 Hz, 1H, H6''), 4.62 (d, *J* = 10.3 Hz, 1H, CH_2, CH_2), 4.56-4.52 (m, 3H, CH_2, H1, H6), 4.22 (dd, *J* = 4.0, 12.1 Hz, 1H, H6''), 4.15 (dd, J = 4.0, 12.1 Hz, 1H, H6), 4.08 (d, *J* = 8.3 Hz, 1H, H3'), 4.00 (t, *J* = 9.2 Hz, 1H, H4''), 3.95 (dd, *J* = 3.3, 9.0 Hz, 1H, H3'''), 3.91-3.80 (m, 5H, H4'', H4', H5', H5''', ^{i}Pr-CH), 3.79-3.74 (m, 2H, H3'', H3), 3.65-3.63 (m, 1H, H5''), 3.60-3.54 (m, 2H, H4''', H5), 2.03 (s, 3H, CH_3), 1.99 (s, 3H, CH_3), 1.92 (d, *J* = 2.6 Hz, 1H, OH), 1.13 (d, *J* = 6.2 Hz, 3H, ^{i}Pr-CH_3), 1.04 (d, *J* = 6.1 Hz, 3H, H6'), 0.99 (d, *J* = 5.9 Hz, 3H, H6'''), 0.97 (d, *J* = 6.1 Hz, 3H, ^{i}Pr-CH_3), 0.78 (s, 18H, TBS), 0.1 (s, 3H, TBS), 0.04 (s, 3H, TBS), -0.03 (s, 3H, TBS), -0.06 (s, 3H, TBS); ^{13}C NMR (150 MHz, $CDCl_3$) δ = 170.9 (C), 170.6 (C), 166.2 (C), 166.0 (C), 165.3 (C), 165.2 (C), 137.7 (C), 137.6 (C), 133.4 (CH), 133.3 (CH), 133.2 (CH), 130.2 (C), 130.1 (CH), 130.0 (CH), 129.96 (CH), 129.9 (C), 129.8 (CH), 128.6 (CH), 128.57 (CH), 128.5 (CH), 128.48 (CH), 128.4 (CH), 128.3 (CH), 128.0 (CH), 127.7 (CH), 127.68 (CH), 100.0 (CH), 99.6 (CH), 98.3 (CH), 97.9 (CH), 81.8 (CH), 81.2 (CH), 77.4 (CH), 76.3 (CH), 75.9 (CH), 74.8 (CH_2), 74.7 (CH_2), 74.5 (CH), 74.4 (CH), 73.7 (CH), 73.6 (CH), 73.3 (CH), 73.1 (CH), 73.0 (CH), 72.9 (CH), 71.3 (CH), 71.0 (CH), 69.0 (CH), 68.3 (CH), 62.8 (CH_2), 62.5 (CH_2), 26.0 (CH), 25.8 (CH), 23.4 (CH_3), 22.1 (CH_3), 21.1 (CH_3), 20.9 (CH_3), 18.1 (C), 17.9 (C), 17.9 (CH_3), 17.7 (CH_3), -4.2 (CH_3), -4.4 (CH_3), -4.42 (CH_3), -4.6 (CH_3); HRMS m/z $(M+Na)^+$ calcd for $C_{85}H_{108}O_{25}Si_2Na^+$ 1608.6641 found 1608.6637.

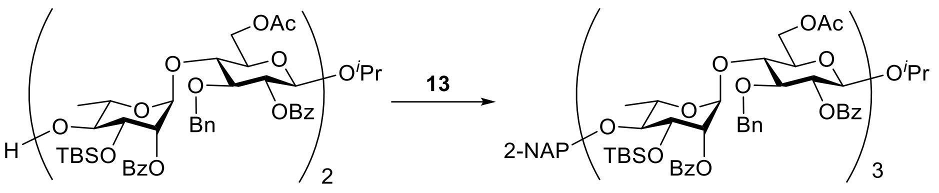

34 **35**

Compound 35. To a solution of donor **13** (272 mg, 260.0 μmol) and acceptor **34** (337 mg, 210.0 μmol) in anhydrous CH_2Cl_2 (4.8 mL), freshly activated AW-500 molecular sieves (0.6 g) was added under nitrogen atmosphere, and the reaction mixture was allowed to stir at room temperature for 1 h. Afterwards, the reaction flask was cooled to –78 °C, NIS (71.7 mg, 320.0 μmol) and TfOH (7 μL, 80.0 μmol) were added, and the reaction temperature was gradually raised to –60 °C for 3 h. TLC (EtOAc/Hexane, 1/2.3, Rf =0.37) analysis indicated the products formation, Et_3N was added to quench the reaction. The whole mixture was filtered through a pad of Celite®, the solids were washed with CH_2Cl_2, and the filtrate was washed with 10% $Na_2S_2O_3$ solution and water. The organic layer was dried over anhydrous $MgSO_4$, filtered, and concentrated *in vacuo*. The resulting crude residue was purified by column chromatography (EtOAc/Hexane, 1/2.3) on silica gel to afford the desired product **35** (421 mg, 79%) as a white solid. $[\alpha]_D^{27}$ = -9.55 (c = 2.0, $CHCl_3$); IR (thin film in KBr) ν 2927, 2855, 1728, 1452, 1363, 1265, 1096, 1069, 838, 778, 711 cm^{-1}; ^{1}H NMR (600 MHz, $CDCl_3$) δ = 8.06-7.97 (m, 12H, Ar-H), 7.83-7.76 (m, 4H, Ar-H), 7.60-7.51 (m, 6H, Ar-H), 7.48-7.36 (m, 15H, Ar-H), 7.15-7.10 (m, 10H, Ar-H), 7.08-7.00 (m, 5H, Ar-H), 5.35-5.30 (m, 2H, H2'''', H2''), 5.22 (t, *J* = 8.5 Hz, 1H, H2), 5.18 (bs, 1H, H2'''''), 5.13 (d, *J* = 7.1 Hz, 1H, H1''''), 5.08-5.03 (m, 7H, H1'', H1''''', H1''', H2''', H2', H1', CH_2), 4.78-4.76 (m, 2H, CH_2, CH_2), 4.73-4.62 (m, 6H, H6'''', H6'', CH_2, CH_2, CH_2, CH_2, CH_2, CH_2), 4.57-4.53 (m, 3H, H1, H6, CH_2), 4.25-4.21 (m, 2H, H6'''', H3'''''), 4.17-4.15 (m, 2H, H6'', H6), 4.09-4.06 (m, 2H, H3', H3'''), 4.02-3.83 (m, 9H, H4'''', H4'', H4, H5''''', H5', H4', H4''', H5''', ^{i}Pr-CH), 3.78-3.75 (m, 2H, H3'''', H3), 3.71 (t, *J* = 8.8 Hz, 1H, H3''), 3.65 (d, *J* = 9.2 Hz, 1H, H5''''), 3.57 (m, 2H, H5'', H5), 3.5 (t, *J* = 9.2 Hz, 1H, H4'''''), 2.03 (s, 3H, CH_3), 1.99 (s, 3H, CH_3), 1.97 (s, 3H, CH_3), 1.14 (d, *J* = 6.1 Hz, 3H, ^{i}Pr-CH_3), 1.02 (d, *J* = 6.1 Hz, 3H, H6'''''), 0.98 (m, 9H, H6''', H6', ^{i}Pr-CH_3), 0.82 (s, 9H, TBS), 0.78 (s, 9H, TBS), 0.78 (s, 9H, TBS), 0.1 (s, 3H, TBS), 0.08 (s, 3H, TBS), -0.02 (s, 3H, TBS), -0.03 (s, 3H, TBS), -0.05 (s, 3H, TBS), -0.07 (s, 3H, TBS); ^{13}C NMR (150 MHz, $CDCl_3$) δ = 170.8 (C), 170.6 (C), 170.5 (C), 166.1 (C), 166.0 (C), 165.9 (C), 165.3 (C), 165.2 (C), 165.1 (C), 137.6 (C), 137.5 (C), 136.1 (C), 133.44 (CH), 133.4 (CH), 133.3 (CH), 133.29 (CH), 133.2 (CH), 133.0 (C), 130.2 (CH), 130.1 (CH), 130.0 (CH), 129.94 (CH), 129.90 (CH), 129.8 (CH), 128.64 (CH), 128.60 (CH),

128.55 (CH), 128.5 (CH), 128.4 (CH), 128.36 (CH), 128.3 (CH), 128.26 (CH), 128.2 (CH), 128.1 (CH), 128.1 (CH), 128.0 (CH), 127.8 (CH), 127.65 (CH), 127.63 (CH), 127.6 (CH), 126.4 (CH), 126.2 (CH), 126.0 (CH), 100.0 (CH), 99.56 (CH), 99.5 (CH), 98.2 (CH), 98.1 (CH), 97.9 (CH), 81.8 (CH), 81.5 (CH), 81.4 (CH), 81.2 (CH), 76.6 (CH), 76.3 (CH), 76.0 (CH), 75.4 (CH2), 74.95 (CH_2), 74.9 (CH_2), 74.8 (CH_2), 74.6 (CH), 74.5 (CH), 74.4 (CH), 73.8 (CH), 73.65 (CH), 73.6 (CH), 73.2 (CH), 73.0 (CH), 71.1 (CH), 70.9 (CH), 68.8 (CH), 68.3 (CH), 68.2 (CH), 62.7 (CH_2), 62.4 (CH_2), 62.2 (CH_2), 25.9 (CH_3), 25.8 (CH_3), 23.3 (CH_3), 22.1 (CH_3), 21.0 (CH_3), 20.9 (CH_3), 20.8 (CH_3), 18.0 (CH_3), 17.98 (C), 17.9 (C), 17.87 (CH_3), 17.8 (CH_3), -4.25 (C), -4.30 (C), -4.40 (C), -4.44 (C), -4.5 (C); HRMS m/z $(M+H)^+$ calcd for $C_{137}H_{167}O_{37}Si_3^+$ 2489.0519 found 2489.0511.

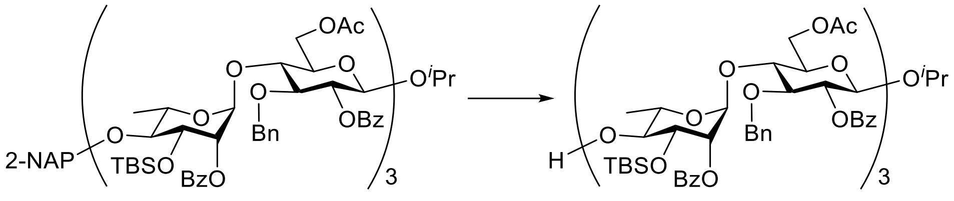

35 **36**

Compound 36. To a solution of compound **35** (150 mg, 60.0 μmol) in a mixed solvent CH_2Cl_2/H_2O (7.5 mL, 30/1), DDQ (41 mg, 180.0 μmol) was added at 0 °C. Then, the reaction mixture was warmed up to room temperature and allowed to stir for 3 h. TLC (EtOAc/Hexane = 1/2.3, Rf = 0.36) analysis indicates the complete conversion of starting materials into products. The whole mixture was filtered through a pad of Celite®, solids were washed with CH_2Cl_2, and the combined filtrates were washed with sat. $NaHCO_3$, 10% $Na_2S_2O_3$ and H_2O. The organic layer was dried over anhydrous $MgSO_4$, filtered, and concentrated *in vacuo*. The resulting crude residue was purified by column chromatography (EtOAc/Hexane = 1/2) on silica gel to afford the desired compound **36** (100 mg, 82%) as a white solid. $[\alpha]_D^{27}$ = +2.00 (*c* = 1.0, $CHCl_3$); IR (thin film in KBr) ν 3736, 2956, 2926, 1728, 1453, 1378, 1266, 1096, 1069, 838, 778, 711 cm^{-1}; ^1H NMR (600 MHz, $CDCl_3$) δ = 8.01-7.96 (m, 12H, Ar-H), 7.58-7.50 (m, 6H, Ar-H), 7.47-7.35 (m, 12H, Ar-H), 7.15-7.03 (m, 15H, Ar-H), 5.33 (t, *J* = 7.9 Hz, 1H, H2''''), 5.3 (t, *J* = 7.9 Hz, 1H, H2''), 5.21 (t, *J* = 8.5 Hz, 1H, H2), 5.12 (m, 2H, H2''''', H1''''), 5.07-5.01 (m, 6H, H1''''', H1'', H2', H2''', H1''', H1'), 4.73-4.69 (m, 3H, CH_2, CH_2, H6a''''), 4.66-4.60 (m, 4H, H6a'', CH_2, CH_2, CH2), 4.56-4.52 (m, 3H, H1, H6a, CH_2), 4.24-4.21 (m, 1H, H6b''''), 4.16-4.13 (m, 2H, H6b'', H6b), 4.08-4.04 (m, 2H, H3', H3'''), 4 (t, J = 9.1 Hz, 1H, H4''''), 3.97-3.93 (m, 2H, H3''''', H4''), 3.92-3.69 (m, 10H, ^{i}Pr-CH, H5''''', H5''', H5', H3'''', H3'', H3, H4''', H4',

H4), 3.65-3.63 (m, 1H, H5'''', H4'''''), 3.61-3.56 (m, 3H, H5, H5''), 2.02 (s, 3H, CH_3), 1.99 (s, 3H, CH_3), 1.96 (s, 3H, CH_3), 1.14 (d, J = 6.2 Hz, 3H, ^{i}Pr-CH_3), 1.05 (d, J = 6.1 Hz, 3H, H6'''''), 0.97-0.96 (d, 9H, ^{i}Pr-CH_3, H6', H6'''), 0.79-0.76 (m, 27H, TBS), 0.1 (s, 3H, TBS), 0.05 (s, 3H, TBS), -0.02 (s, 3H, TBS), -0.04 (s, 3H, TBS), -0.06 (s, 3H, TBS), -0.08 (s, 3H, TBS); ^{13}C NMR (150 MHz, $CDCl_3$) δ = 170.8 (C), 170.6 (C), 166.1 (C), 166.0 (C), 165.9 (C), 165.26 (C), 165.2 (C), 165.1 (C), 137.62 (C), 137.6 (C), 137.5 (C), 133.45 (CH), 133.41 (CH), 133.35 (CH), 133.3 (CH), 133.2 (CH), 130.2 (CH), 130.1 (CH), 130.0 (C), 129.99 (CH), 129.96 (CH), 129.9 (C), 129.8 (CH), 128.66 (CH), 128.6 (CH), 128.57 (CH), 128.5 (CH), 128.4 (CH), 128.38 (CH), 128.3 (CH), 128.2 (CH), 128.0 (CH), 127.7 (CH), 127.67 (CH), 127.65 (CH), 100.0 (CH), 99.58 (CH), 99.5 (CH), 98.3 (CH), 98.2 (CH), 98.0 (CH), 81.8 (CH), 81.6 (CH), 81.2 (CH), 76.7 (CH), 76.3 (CH), 75.9 (CH), 74.9 (CH_2), 74.88 (CH_2), 74.8 (CH_2), 74.6 (CH), 74.5 (CH), 74.4 (CH), 73.7 (CH), 73.6 (CH), 73.3 (CH), 73.0 (CH), 72.9 (CH), 72.8 (CH), 71.3 (CH), 70.9 (CH), 69.0 (CH), 68.4 (CH), 68.3 (CH), 62.8 (CH_2), 62.4 (CH_2), 62.2 (CH_2), 25.9 (CH_3), 25.7 (CH_3), 23.3 (CH_3), 22.1 (CH_3), 21.0 (CH_3), 20.9 (CH_3), 20.8 (CH_3), 18.1 (C), 17.9 (C), 17.89 (CH_3), 17.87 (CH_3), 17.7 (CH_3), -4.2 (CH_3), -4.3 (CH_3), -4.38 (CH_3), -4.43 (CH_3), -4.6 (CH_3); HRMS m/z $(M+Na)^+$ calcd for 2370.9716 $C_{126}H_{158}O_{37}Si_3Na^+$ found 2370.9661.

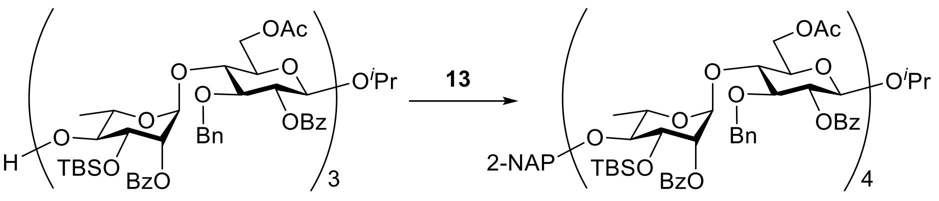

36 **37**

Compound 37. To a solution of donor **13** (350 mg, 340.0 μmol) and acceptor **36** (640 mg, 270.0 μmol) in dry CH_2Cl_2 (6.1 mL), freshly activated AW-500 MS (1.0 g) were added and allowed to stir at room temperature for 1 h under nitrogen atmosphere. Then, the reaction mixture was cooled to –78 °C, NIS (92.0 mg, 410.0 μmol) and TfOH (9 μL, 100 μmol) were added, and the reaction mixture was gradually warmed up to –60 °C for 3 h. TLC (EtOAc/Hexane, 1/4, Rf =0.21) analysis indicates the products formation, Et_3N was added to quench the reaction. The whole mixture was filtered through a pad of Celite®, the solids were washed with CH_2Cl_2, and the filtrate was washed with 10% $Na_2S_2O_3$ solution and water. The organic layer was dried over anhydrous $MgSO_4$, filtered, and concentrated *in vacuo*. The resulting crude residue was purified by column chromatography (EtOAc/Hexane, 1/4) on silica gel to afford the desired compound **37** (651 mg, 83%) as white solid. $[\alpha]_D^{27}$ = -12.1 (*c* = 1.0,

$CHCl_3$); IR (thin film in KBr) ν 2931, 2858, 1728, 1602, 1452, 1363, 1316, 1266, 1176, 1096, 1069, 1028, 936, 860, 838, 778, 754, 711 cm^{-1}; ^1H NMR (600 MHz, $CDCl_3$) δ = 8.04 (d, *J* = 7.6 Hz, 2H, Ar-H), 8.00-7.96 (m, 14H, Ar-H), 7.82-7.78 (m, 3H, Ar-H), 7.74 (s, 1H, Ar-H), 7.59-7.50 (m, 8H, Ar-H), 7.47-7.35 (m, 19H, Ar-H), 7.13-6.98 (m, 20H, Ar-H), 5.33-5.27 (m, 3H, H2'', H2'''', H2'''''), 5.22 (t, *J* = 8.4 Hz, 1H, H2), 5.16 (bs, 1H, H2''''''), 5.11 (d, *J* = 6.6 Hz, 1H, H1'''''), 5.06-5.01 (m, 10H, H1', H1'', H1''', H1'''', H1''''', H1'''''', H2', H2''', H2'''', CH_2), 4.76-4.74 (m, 2H, CH_2, CH_2), 4.71-4.68 (m, 2H, H6a''''', CH_2), 4.65-4.60 (m, 7H, H6a'', H6a'''', CH_2, CH_2, CH_2, CH_2, CH_2), 4.56-4.51 (m, 3H, H1, H6, CH_2), 4.23-4.19 (m, 2H, H6b''''', H3''''''), 4.16-4.12 (m, 3H, H6b, H6b'', H6b''''), 4.07-4.03 (m, 3H, H3', H3''', H3'''''), 4.00-3.73 (m, 14H, ^{i}Pr-CH, H4, H4', H4'', H4''', H4'''', H4''''', H4'''''', H3, H3'''''', H5', H5''', H5''''', H5''''''), 3.70-3.67 (m, 2H, H3'', H3''''), 3.63-3.62 (m, 1H, H5''''''), 3.55-3.54 (m, 3H, H5, H5'', H5''''), 3.48 (t, *J* = 9.2 Hz, 1H, H4''''''), 2.02 (s, 3H, CH_3), 1.98 (s, 3H, CH_3), 1.95 (s, 3H, CH_3), 1.13 (d, *J* = 6.2 Hz, 3H, H6''''''), 1.01 (d, *J* = 6.2 Hz, 3H, H6'''''), 0.97-0.91 (m, 12H, H6''', ^{i}Pr-CH_3), 0.8 (s, 9H, TBS), 0.78 (s, 9H, TBS), 0.76 (s, 18H, TBS), 0.08 (s, 3H, TBS), 0.07 (s, 3H, TBS), -0.03 (s, 3H, TBS), -0.05 (s, 6H, TBS), -0.07 (s, 3H, TBS); ^{13}C NMR (150 MHz, $CDCl_3$) δ = 170.8 (C), 170.6 (C), 166.2 (C), 166.0 (C), 165.3 (C), 165.26 (C), 165.1 (C), 137.6 (C), 137.58 (C), 137.5 (C), 136.1 (CH), 133.5 (C), 133.4 (CH), 133.3 (CH), 133.2 (CH), 133.1 (CH), 130.2 (C), 130.1 (CH), 130.0 (CH), 129.9 (CH), 129.8 (CH), 128.7 (CH), 128.6 (CH), 128.51 (CH), 128.5 (CH), 128.4 (CH), 128.3 (CH), 128.2 (CH), 128.17 (CH), 128.1 (CH), 128.0 (CH), 127.9 (CH), 127.6 (CH), 126.5 (CH), 126.2 (CH), 126.0 (CH), 100.0 (CH), 99.6 (CH), 99.5 (CH), 98.2 (CH), 98.1 (CH), 98.0 (CH), 81.8 (CH), 81.6 (CH), 81.5 (CH), 81.2 (CH), 76.74 (CH), 76.7 (CH), 76.3 (CH), 76.0 (CH), 75.4 (CH_2), 75.0 (CH_2), 74.9 (CH_2), 74.8 (CH_2), 74.6 (CH), 74.5 (CH), 74.4 (CH), 73.9 (CH), 73.6 (CH), 73.6 (CH), 73.3 (CH), 73.1 (CH), 71.1 (CH), 70.9 (CH), 68.9 (CH), 68.4 (CH), 68.3 (CH), 62.8 (CH_2), 62.5 (CH_2), 62.2 (CH_2), 26.0 (CH_3), 25.9 (CH_3), 22.1 (CH_3), 21.0 (CH_3), 20.94 (CH_3), 20.9 (CH_3), 18.05 (CH_3), 18.0 (C), 17.9 (C), 17.89 (CH_3), 17.86 (CH_3), 17.8 (CH_3), -4.2 (CH_3), -4.3 (CH_3), -4.37 (CH_3), -4.41 (CH_3), -4.5 (CH_3).

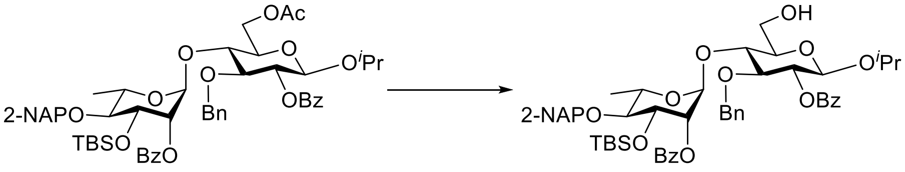

31 38

Compound 38. To a solution of compound **31** (175 mg, 180 μmol) in CH_2Cl_2 (1.8 mL), $Mg(OMe)_2$ (0.91 mL, 7-8% in MeOH) was added at room temperature under N_2 atmosphere.

Then, the reaction mixture was allowed to stir for 5 h. TLC (EtOAc/Hexane = 1/4, Rf = 0.21) analysis indicated the complete conversion of starting material into products, the reaction mixture was neutralized with 1 N HCl, diluted with CH_2Cl_2, and washed with H_2O. Organic layer was dried over anhydrous $MgSO_4$, filtered, and concentrated *in vacuo*. The resulting crude residue was purified by flash column chromatography (EtOAc/Hexane = 1/4) to afford the desired product **38** (123 mg, 74%) as a white solid. $[\alpha]_D^{28}$ = -5.55 (*c* = 1.0, $CHCl_3$); IR (thin film in KBr) ν 3521, 2927, 2855, 1726, 1602, 1451, 1383, 1267, 1107, 1069, 1028, 871, 837, 777, 752, 710, 476 cm^{-1}; ^{1}H NMR (600 MHz, $CDCl_3$) δ = 8.05 (d, *J* = 7.8 Hz, 2H, Ar-H), 7.99 (d, *J* = 7.8 Hz, 2H, Ar-H), 7.83-7.78 (m, 3H, Ar-H), 7.75 (s, 1H, Ar-H), 7.57 (t, J = 7.40 Hz, 1H, Ar-H), 7.53 (t, *J* = 7.4 Hz, 1H, Ar-H), 7.47-7.39 (m, 7H, Ar-H), 7.18 (d, *J* = 12.4 Hz, 2H, Ar-H), 7.04-6.99 (m, 3H, Ar-H), 5.25 (t, *J* = 8.6 Hz, 1H, H-2), 5.21 (bs, 1H, H-2'), 5.08 (bs, 1H, H-1'), 5.05 (d, *J* = 11.5 Hz, 1H, Ar-CH_2), 4.77 (d, *J* = 11.5 Hz, 1H, Ar-CH_2), 4.73 (d, *J* = 10.5 Hz, 1H, Ar-CH_2), 4.67-4.65 (m, 2H, H-1, Ar-CH_2), 4.22 (dd, *J* = 3.3, 5.8 Hz, 1H, H-3'), 4.04-3.96 (m, 3H, H-5', H-4, H-6), 3.92-3.83 (m, 3H, ^{i}Pr -CH, H-3, H-6), 3.49 (t, *J* = 9.3 Hz, 2H, H-4', H-5), 2.12-2.10 (m, 1H, OH), 1.17 (d, *J* = 6.2 Hz, 3H, ^{i}Pr-CH_3), 1.01-0.98 (m, 6H, ^{i}Pr-CH_3, H-6'), 0.82 (s, 9H, TBS), 0.11 (s, 3H, TBS), 0.08 (s, 3H, TBS); ^{13}C NMR (150 MHz, $CDCl_3$) δ = 166.4 (C), 165.2 (C), 137.6 (C), 136.2 (C), 133.4 (C), 133.3 (CH), 133.2 (CH), 133.0 (C), 130.1 (C), 130.0 (CH), 129.8 (CH), 128.6 (CH), 128.5 (CH), 128.3 (CH), 128.3 (CH), 128.1 (CH), 128.0 (CH), 127.9 (CH), 127.6 (CH), 126.4 (CH), 126.2 (CH), 126 .0(CH), 125.9 (CH), 100.1 (CH), 97.7 (CH), 81.5 (CH), 81.2 (CH), 77.4 (CH), 76.1 (CH), 75.4 (CH_2), 74.8 (CH_2), 74.6 (CH), 74.4 (CH), 73.9 (CH), 72.9 (CH), 71.3 (CH), 68.7 (CH), 62.0 (CH_2), 25.9 (CH_3), 23.4 (CH_3), 22.1 (CH_3), 18.0 (CH_3), 17.9 (CH_3), -4.3 (CH_3), -4.4 (CH_3); HRMS m/z $(M+H)^+$ calcd for $C_{55}H_{67}O_{13}Si^+$ 963.4345 found 963.4355.

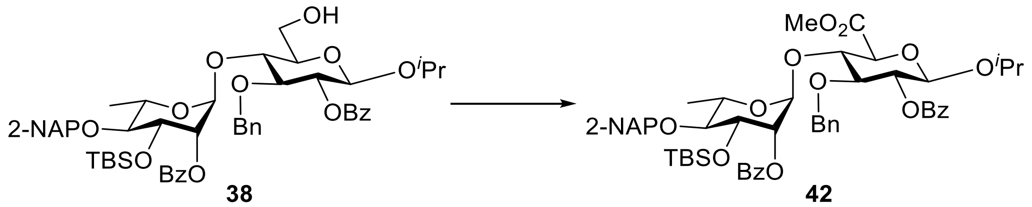

38 **42**

Compound 42. To a solution of compound **38** (119 mg, 130 μmol) in a mixed solvent CH_2Cl_2/H_2O (3.6 mL, 1/2), TEMPO (6.1 mg, 40.0 μmol) and BAIB (166 mg, 520 μmol) were added at 0 °C. Then, the reaction mixture was allowed to stir for 5 h at room temperature, diluted with EtOAc, and washed with 10% $Na_2S_2O_3$ and brine. The organic layer was dried

over anhydrous $MgSO_4$, filtered, and concentrated *in vacuo* to afford the crude acid, which was directly used for the next reaction without any purification. Freshly prepared CH_2N_2 (0.77 mmol in Et_2O) was added to a solution of crude acid (58.0 μmol) in THF (2.6 mL) at 0 °C. The reaction mixture was allowed to stir at room temperature for 0.5 h. TLC (EtOAc/Hexane = 1/9, Rf = 0.24) analysis indicates the complete conversion of starting materials into products. The excess CH_2N_2 was quenched with AcOH, then diluted with EtOAc, washed with sat. $NaHCO_{3(aq)}$ and brine. The organic layer was dried over anhydrous $MgSO_4$, filtered, and concentrated *in vacuo*. The crude residue was purified by column chromatography (EtOAc/Hexane = 1/9) to afford the desired product **42** (85 mg, 81%) as a white solid. $[\alpha]_D^{20}$ = -6.95 (*c* = 1.0, $CHCl_3$); IR (thin film in KBr) ν 2957, 2926, 2855, 1750, 1726, 1602, 1452, 1383, 1315, 1266, 1221, 1175, 1106, 1070, 931, 871, 838, 817, 777, 755, 710, 477 cm^{-1}; ^1H NMR (600 MHz, $CDCl_3$) δ = 8.03 (d, J = 7.7 Hz, 2H, Ar-H), 7.98 (d, J = 7.8 Hz, 2H, Ar-H), 7.82-7.76 (m, 3H, Ar-H), 7.72 (s, 1H, Ar-H), 7.57 (t, J = 7.4 Hz, 1H, Ar-H), 7.52 (t, J = 7.4 Hz, 1H, Ar-H), 7.46-7.43 (m, 4H, Ar-H), 7.41-7.38 (m, 3H, Ar-H), 7.18-7.16 (m, 2H, Ar-H), 7.03 (m, 3H, Ar-H), 5.27 (t, J = 7.8 Hz, 1H, H-2), 5.2 (bs, 1H, H-2'), 5.02 (d, J = 11.5 Hz, 1H, Ar-CH_2), 4.88 (bs, 1H, H-1'), 4.74-4.70 (m, 3H, H-1, Ar-CH_2, Ar-CH_2), 4.66 (d, J = 12.0 Hz, 1H, Ar-CH_2), 4.24 (t, J = 8.9 Hz, 1H, H-4), 4.19 (dd, J = 3.2, 9.1 Hz, 1H, H-3'), 4.06 (d, J = 9.2 Hz, 1H, H-5), 4.00-3.95 (m, 1H, H-5'), 3.90 (m, 1H, ^{i}Pr-CH), 3.86-3.84 (m, 4H, H-3, CH_3), 3.46 (t, J = 9.4 Hz, 1H, H-4'), 1.14 (d, J = 6.2 Hz, 3H, ^{i}Pr-CH_3), 1.01 (d, J = 6.2 Hz, 3H, H-6'), 0.99 (d, J = 6.2 Hz, 3H, ^{i}Pr-CH_3), 0.8 (s, 9H, TBS), 0.13 (s, 3H, TBS), 0.07 (s, 3H, TBS), ^{13}C NMR (150 MHz, $CDCl_3$) δ = 169.0 (C), 165.8 (C), 165.1 (C), 137.5 (C), 136.1 (C), 133.4 (C), 133.3 (CH), 133.2 (CH), 133.0 (C), 130.3 (C), 130.0 (CH), 129.9 (CH), 129.8 (CH), 128.6 (CH), 128.3 (CH), 128.2 (CH), 128.1 (CH), 128.0 (CH), 127.9 (CH), 127.6 (CH), 126.5 (CH), 126.2 (CH), 126.09 (CH), 126.01 (CH), 99.8 (CH), 98.2 (CH), 81.6 (CH), 80.8 (CH), 77.4 (CH), 76.8 (CH), 75.5 (CH_2), 75.1 (CH), 74.3 (CH_2), 74.0 (CH), 73.6 (CH), 72.6 (CH), 71.3 (CH), 68.9 (CH), 53.3 (CH_3), 26.0 (CH_3), 23.4 (CH_3), 21.9 (CH_3), 18.1 (CH_3), 18.0 (C), -4.4 (CH_3), -4.5 (CH_3); HRMS m/z $(M+Na)^+$ calcd for $C_{54}H_{64}O_{13}SiNa^+$ 971.4007 found 971.4008.

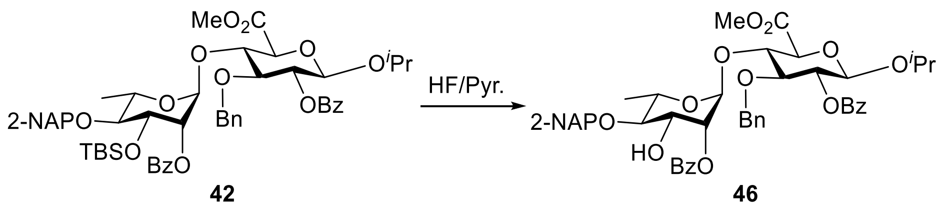

Compound 46. To a solution of compound **42** (502 mg, 530.0 μmol) in THF (5.0 mL), HF/pyridine (2.0 mL, 420 μmol) was added dropwise at 0 °C, After 10 minutes, reaction mixture was allowed to stir at room temperature for 5 h. TLC (EtOAc/Hexane = 1/4, Rf = 0.18) analysis indicates the complete conversion of starting materials into products. The excess HF/pyridine was quenched with silica gel, diluted with CH_2Cl_2/MeOH (1/1), filtered through a pad of Celite® and washed with CH_2Cl_2/MeOH (1/1). Filtrate was concentrated *in vacuo*, crude residue was dissolved in CH_2Cl_2 and washed with sat. $NaHCO_{3(aq)}$ and H_2O. The organic layer was dried over anhydrous $MgSO_4$, filtered, and concentrated *in vacuo*. The crude residue was purified by column chromatography (EtOAc/Hexane = 1/4) on silica gel to afford the desired compound **46** (422 mg, 93%) as a white solid. $[\alpha]_D^{20}$ = +13.4 (c = 0.5, $CHCl_3$); IR (thin film in KBr) ν 3508, 2972, 2926, 1725, 1601, 1451, 1371, 1315, 1266, 1223, 1176, 1145, 1095, 1069, 1027, 910, 856, 819, 754, 711, 476 cm^{-1}; ^1H NMR (600 MHz, $CDCl_3$) δ = 7.98-7.96 (m, 4H, Ar-H), 7.81 (d, *J* = 8.4 Hz, 2H, Ar-H), 7.78-7.77 (m, 1H, Ar-H), 7.74 (s, 1H, Ar-H), 7.55 (t, *J* = 7.3 Hz, 2H, Ar-H), 7.52 (t, *J* = 7.3 Hz, 2H, Ar-H), 7.46-7.38 (m, 7H, Ar-H), 7.17-7.16 (m, 2H, Ar-H), 7.06 (m, 3H, Ar-H), 5.29-5.27 (m, 2H, H-2, H-2'), 4.92 (s, 1H, H-1'), 4.91 (d, *J* = 11.4 Hz, 1H, Ar-CH_2), 4.82 (d, *J* = 11.4 Hz, 1H, Ar-CH_2), 4.68-4.63 (m, 3H, H-1), 4.19 (t, *J* = 9.2 Hz, 1H, H-4), 4.18-4.16 (m, 1H, H-3'), 4.02 (d, *J* = 9.2 Hz, 1H, H-5), 4.00-3.97 (m, 1H, H-5'), 3.91-3.87 (m, 1H, ^{i}Pr-CH), 3.82 (s, 3H, CH_3), 3.79 (t, *J* = 2.7 Hz, 1H, H-3), 3.43 (t, *J* = 9.5 Hz, 1H, H4'), 2.21 (bs, 1H, OH), 1.15 (d, *J* = 6.2 Hz, 3H, ^{i}Pr-CH_3), 1.05 (d, *J* = 6.1 Hz, 3H, H-6'), 0.99 (d, *J* = 6.2 Hz, 3H, ^{i}Pr-CH_3); ^{13}C NMR (150 MHz, $CDCl_3$) δ = 169.1 (C), 166.2 (C), 165.1 (C), 137.4 (C), 135.8 (C), 133.5 (CH), 133.4 (C), 133.3 (CH), 133.2 (C), 130.0 (CH), 129.9 (C), 129.8 (CH), 129.7 (C), 128.61 (CH), 128.6 (CH), 128.5 (CH), 128.3 (CH), 128.0 (CH), 127.9 (CH), 127.7 (CH), 126.9 (CH), 126.4 (CH), 126.2 (CH), 126.1 (CH), 99.9 (CH), 97.8 (CH), 81.7 (CH), 80.8 (CH), 76.3 (CH), 75.3 (CH_2), 74.9 (CH), 74.6 (CH_2), 74.0 (CH), 73.1 (CH), 72.6 (CH), 70.5 (CH), 68.6 (CH), 53.2 (CH_3), 23.3 (CH_3), 21.9 (CH_3), 18.1 (CH_3); HRMS m/z $(M+Na)^+$ calcd for $C_{48}H_{50}O_{13}Na^+$ 857.3138 found 857.3144.

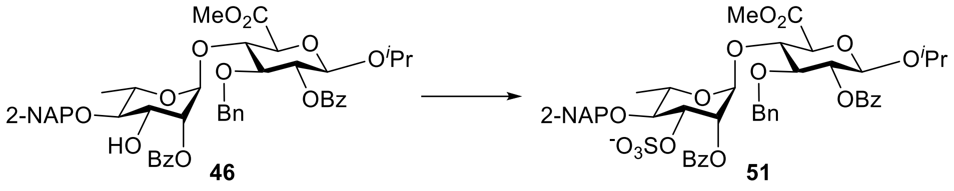

Compound 51. To a solution of compound **46** (113 mg, 140.0 μmol) in anhydrous DMF (6.8 mL), $SO_3 \cdot Et_3N$ (2.5 mg, 1.4 mmol) was added at room temperature under an argon atmosphere. Then, the reaction mixture was heated to 60 °C and was allowed to stir at same temperature for 3 h. The reaction mixture was cooled to room temperature, then sat. $NaHCO_{3(aq)}$ was added to quench the reaction and allowed to stir at room temperature for 3 h. The resulting mixture was extracted with THF, and the combined organic layers were washed with brine, dried over anhydrous $MgSO_4$, filtered, and concentrated *in vacuo*. The crude residue was subjected to AG 50W-X8 cation exchange column (Na^+ form) followed by LH-20 column using MeOH as an eluent to afford the desired product **51** (106 mg, 80%) as a white solid. ^1H NMR (600 MHz, CD_3OD) δ = 8.07 (dd, *J* = 1.4, 8.0 Hz, 2H, Ar-H), 8.01 (dd, *J* = 1.4, 8.0 Hz, 2H, Ar-H), 7.92 (s, 1H, Ar-H), 7.88-7.85 (m, 3H, Ar-H), 7.63-7.60 (m, 3H, Ar-H), 7.50-7.44 (m, 6H, Ar-H), 7.12 (d, *J* = 7.3 Hz, 2H, Ar-H), 6.97 (t, *J* = 7.4 Hz, 1H, Ar-H), 6.88 (t, *J* = 7.6 Hz, 2H, Ar-H), 5.64 (dd, J = 2.1, 3.1 Hz, 1H, H-2'), 5.25 (d, *J* = 11.0 Hz, 1H, Ar-CH_2), 5.18 (m, 1H, H-2), 5.04 (dd, *J* = 3.2, .9.2 Hz, 1H, H-3'), 4.93 (d, *J* = 1.9 Hz, 1H, H-1'), 4.91 (d, *J* = 8.0 Hz, 1H, H-1), 4.85 (d, *J* = 11.0 Hz, 1H, Ar-CH_2), 4.79 (d, *J* = 10.4 Hz, 1H, Ar-CH_2), 4.65 (d, *J* = 10.4 Hz, 1H, Ar-CH_2), 4.27-4.25 (m, 1H, H-4), 4.17-4.12 (m, 1H, H-5'), 4.07-4.02 (m, 2H, H-3, H-5), 3.96-3.94 (m, 1H, ^{i}Pr-CH), 3.92 (s, 1H, CH_3), 3.69 (t, *J* = 9.4 Hz, 1H, H-4'), 1.15 (d, *J* = 6.2 Hz, 3H, ^{i}Pr-CH_3), 1.02 (d, *J* = 6.2 Hz, 3H, ^{i}Pr-CH_3), 0.96 (d, *J* = 6.2 Hz, 3H, H-6'); ^{13}C NMR (150 MHz, CD_3OD) δ = 171.0 (C), 166.9 (C), 166.7 (C), 138.5 (C), 137.4 (C), 134.7 (C), 134.6 (CH), 134.5 (C), 134.3 (CH), 131.2 (C), 131.0 (C), 130.7 (CH), 130.6 (CH), 129.7 (CH), 129.5 (CH), 129.4 (CH), 129.0 (CH), 128.9 (CH), 128.8 (CH), 128.6 (CH), 128.4 (CH), 128.2 (CH), 126.9 (CH), 126.8 (CH), 101.0 (CH), 97.8 (CH), 81.6 (CH), 79.5 (CH), 77.6 (CH), 77.1 (CH), 76.6 (CH_2), 75.8 (CH_2), 75.4 (CH), 74.0 (CH), 73.2 (CH), 69.5 (CH), 53.8 (CH_3), 23.5 (CH_3), 22.2 (CH_3), 18.3 (CH_3); HRMS m/z $(M+Na)^+$ calcd for $C_{48}H_{49}Na_2O_{16}S_1Na^+$ 959.2531 found 959.2518.

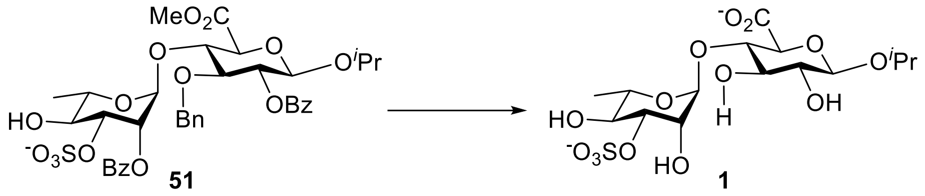

Compound 1. To a solution of compound **51** (60 mg, 60.0 μmol) in $MeOH/CHCl_3/H_2O$ (10.1 mL, 7.3/1.6/1.2), LiOH (980 μL, 5.0 M) was added. Then, the reaction mixture was allowed to stir at 35 °C for 72 h, cation exchange resin (H^+ form) was added to neutralize the reaction, and the pH was adjusted to 7. The resulting mixture was filtered and concentrated *in vacuo*. The crude residue was subjected to AG 50W-X8 cation exchange column (Na^+ form) followed by LH-20 column using MeOH as an eluent to afford the deacylated product. The obtained product was dissolved in MeOH/ H_2O (2.0 mL, 1/1), $Pd(OH)_2$ on activated charcoal (20% Pd, wet, Degussa type, 360 mg) was added. The reaction mixture was degassed several times with hydrogen gas, and then allowed to stir under hydrogen atmosphere. After 72 h, the reaction mixture was filtered off through a pad of Celite®, then the filtrate was concentrated under reduced pressure. The crude residue was subjected to Sephadex G-10 column followed by AG 50W-X8 cation exchange column (Na^+ form) using water as an eluent to obtain the desired compound **1** (21 mg, 65%) as a white solid. ^1H NMR (600 MHz, D_2O) δ = 4.76 (d, J = 8.3 Hz, 1H, H-1'), 4.54 (d, J = 8.0 Hz, 1H, H-1), 4.44 (dd, J = 3.2, 9.7 Hz, 1H, H-3'), 4.24 (m, 1H, H-2'), 4.16-4.11 (m, 1H, H-5'), 4.09-4.03 (m, 1H, ^{i}Pr-CH), 3.76-3.73 (m, 1H, H-4), 3.58-3.54 (m, 3H, H-3, H-5, H-4'), 3.27-3.24 (m, 1H, H-2), 1.25 (d, J = 6.3 Hz, 3H, H-6'), 1.21 (d, J = 6.2 Hz, 3H, ^{i}Pr-CH_3), 1.18 (d, J = 6.1 Hz, 3H, ^{i}Pr-CH_3); ^{13}C NMR (150 MHz, D_2O) δ = 175.4 (C), 100.2 (CH), 100.1 (CH), 78.7 (CH), 78.5 (CH), 76.2 (CH), 74.2 (CH), 73.4 (CH), 73.1 (CH), 69.8 (CH), 68.9 (CH), 68.7 (CH), 22.3 (CH), 20.9 (CH), 16.5 (CH); HRMS m/z $(M+H)^+$ calcd for $C_{15}H_{25}Na_2O_{14}S^+$ 507.0755 found 507.0751.

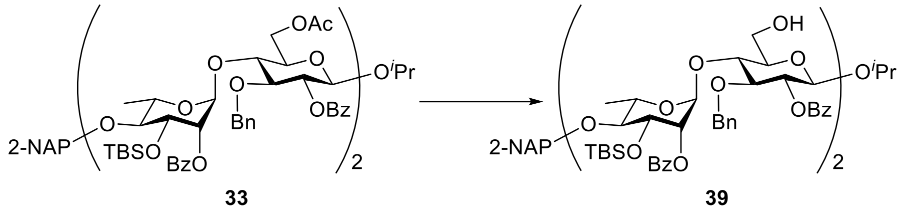

Compound 39. To a solution of compound **33** (510 mg, 0.3 mmol) in CH_2Cl_2 (5.1 mL), $Mg(OMe)_2$ (5.0 mL, 7-8% in MeOH) was added at 25°C under N_2, and the reaction mixture was allowed to stir at room temperature for 5 h. TLC (EtOAc/Hexane = 1/2.3, Rf = 0.24)

analysis indicates the complete convertion of starting materails into products. The reaction mixture was diluted with CH_2Cl_2, neutralized with 2 N HCl and washed with H_2O. Organic layer was dried over anhydrous $MgSO_4$, filtered, and concentrated *in vacuo*. The resulting crude residue was purified by flash column chromatography (EtOAc/Hexane = 1/2.3) to afford the desired product **39** (422 mg, 86%) as a white solid. $[\alpha]_D^{20}$ = -19.0 (*c* = 1.0, $CHCl_3$); IR (thin film in KBr) ν 3509, 2930, 2885, 2857, 1728, 1602, 1584, 1492, 1452, 1360, 1316, 1266, 1107, 1069, 932, 870, 838, 778, 754, 711, 591, 477 cm^{-1}; ^1H NMR (600 MHz, CDCl3) δ = 8.06 (d, *J* = 7.9 Hz, 2H, Ar-H), 7.99-7.96 (m, 6H, Ar-H), 7.83-7.78 (m, 3H, Ar-H), 7.74 (s, 1H, Ar-H), 7.59-7.49 (m, 4H, Ar-H), 7.46-7.43 (m, 6H, Ar-H), 7.41-7.39 (m, 3H, Ar-H), 7.35 (t, *J* = 7.6 Hz, 2H, Ar-H), 7.17-7.08 (m, 7H, Ar-H), 7.02-6.97 (m, 3H, Ar-H), 5.34 (t, *J* = 8.2 Hz, 1H, H2''), 5.23-5.16 (m, 3H, H2, H2'''‘, H1''), 5.08-5.03 (m, 4H, H1''', H1', H2', CH_2), 4.76-4.71 (m, 3H, CH_2, CH_2, CH_2), 4.60-4.58 (m, 3H, CH_2, CH_2, H1), 4.22 (dd, *J* = 2.9, 8.9 Hz, 1H, H3'''), 4.12-4.10 (m, 1H, H3'), 4.04-3.76 (m, 12H, H6'', H4'', H5''', H6, H4', H6'', H5', H4, H6, ^{i}Pr-CH, H3'', H3), 3.51-3.47 (m, 2H, H5'', H4'''), 3.43 (d, *J* = 9.2 Hz, 1H, H5), 2.14 (m, 1H, OH), 2.05 (m, 1H, OH), 1.15 (d, *J* = 6.2 Hz, 3H, ^{i}Pr-CH_3), 0.98 (d, *J* = 6.2 Hz, 6H, ^{i}Pr-CH_3, H6'''), 0.95 (d, *J* = 5.6 Hz, 3H, H6'), 0.81 (s, 9H, TBS), 0.8 (s, 9H, TBS), 0.11 (s, 3H, TBS), 0.08 (s, 3H, TBS), -0.01 (s, 3H, TBS), -0.04 (s, 3H, TBS); ^{13}C NMR (150 MHz, $CDCl_3$) δ = 166.4 (C), 166.27 (C), 165.2 (C), 137.5 (C), 137.4 (C), 136.2 (C), 133.5 (CH), 133.42 (C), 133.4 (C), 133.3 (CH), 133.2 (CH), 133.0 (C), 130.2 (C), 130.1 (C), 130.09 (CH), 130.0 (CH), 129.99 (C), 129.9 (CH), 129.8 (CH), 128.6 (CH), 128.59 (CH), 128.56 (CH), 128.5 (CH), 128.4 (CH), 128.2 (CH), 128.1 (CH), 128.0 (CH), 127.9 (CH), 127.7 (CH), 127.5 (CH), 126.3 (CH), 126.2 (CH), 126.0 (CH), 125.9 (CH), 100.1 (CH), 99.7 (CH), 97.9 (CH), 97.6 (CH), 81.6 (CH), 81.5 (CH), 80.8 (CH), 75.9 (CH), 75.8 (CH), 75.4 (CH_2), 75.3 (CH), 75.0 (CH), 74.94 (CH_2), 74.9 (CH_2), 74.5 (CH), 73.9 (CH), 73.8 (CH), 72.9 (CH), 71.2 (CH), 71.1 (CH), 68.7 (CH), 68.0 (CH), 62.1 (CH_2), 61.8 (CH_2), 26.0 (CH_3), 25.9 (CH_3), 23.4 (CH_3), 22.1 (CH_3), 18.0 (C), 17.98 (CH_3), 17.9 (CH_3), -4.1 (CH_3), -4.3 (CH_3), -4.4 (CH_3), -4.41 (CH_3); HRMS m/z (ESI, M+Na^+) calcd for $(C_{92}H_{112}O_{23}Si_2+Na)^+$ 1664.7059 found 1664.7059.

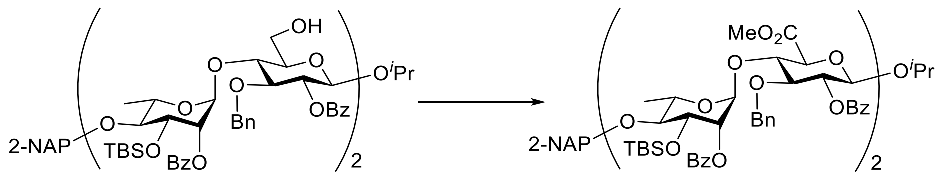

Compound 43. To a solution of compound **39** (118 mg, 70.0 μmol) in a co-solvent CH_2Cl_2/H_2O (3.6 mL, 2/1), TEMPO (3.4 mg, 20.0 μmol) and BAIB (116 mg, 0.4 mmol) were added at 0 °C. Then, the reaction mixture was allowed to stir for 24 h at ambient temperature, reaction was diluted with EtOAc, and washed with 10% $Na_2S_2O_3$ and brine. The organic layer was dried over anhydrous $MgSO_4$, filtered, and concentrated *in vacuo*. The resulting crude residue was further used in the next step without any purification. Freshly prepared CH_2N_2 (0.9 mmol in Et_2O) was added to solution of acid compound (0.5 mmol) in THF (1.5 mL) at 0 °C. The reaction mixture was allowed to stir at room temperature for 0.5 h. TLC (EtOAc/Hexane = 1/2.3, Rf = 0.21) analysis indicates the complete conversion of starting materails into products. AcOH was added to quench the excess CH_2N_2, diluted with EtOAc, washed with sat. $NaHCO_{3(aq)}$ and brine. The organic layer was dried over anhydrous $MgSO_4$, filtered, and concentrated *in vacuo*. The crude residue was purified by flash column chromatography (EtOAc/Hexane = 1/2.3) to afford the desired product **43** (102 mg, 83%) as a white solid. $[\alpha]_D^{29}$ = +22.0 (c = 1.0, $CHCl_3$); IR (thin film in KBr) ν 2930, 2857, 1726, 1602, 1384, 1315, 1265, 1176, 1095, 1069, 1027, 870, 838, 778, 754, 710 cm^{-1}; ^1H NMR (600 MHz, $CDCl_3$) δ = 8.04 (d, J = 7.6 Hz, 2H, Ar-H), 7.98 (d, J = 7.7 Hz, 2H, Ar-H), 7.96 (d, J = 7.6 Hz, 4H, Ar-H), 7.81-7.76 (m, 3H, Ar-H), 7.71 (s, 1H, Ar-H), 7.59-7.53 (m, 4H, Ar-H), 7.46-7.35 (m, 11H, Ar-H), 7.16-7.10 (m, 6H, Ar-H), 7.05-7.02 (m, 4H, Ar-H), 5.36 (t, J = 7.0 Hz, 1H, H2''), 5.24 (t, J = 7.9 Hz, 1H, H2), 5.21 (d, J = 6.5 Hz, 1H, H1''), 5.18 (bs, 1H, H2'''), 5.04 (bs, 1H, H2'), 5.01 (d, J = 11.5 Hz, 1H, CH_2), 4.90 (bs, 2H, H1''', H1'), 4.77-4.72 (m, 3H, CH_2, CH_2, CH_2), 4.65 (d, J = 7.3 Hz, 1H, H1), 4.62 (d, J = 10.7 Hz, 1H, CH_2), 4.57 (d, J = 10.6 Hz, 1H, CH_2), 4.26 (t, J = 8.8 Hz, 1H, H4''), 4.17-4.15 (m, 2H, H4, H3'''), 4.08 (dd, J = 2.9 Hz, 1H, H3'), 4.05 (d, J = 9.8 Hz, 1H, H5''), 3.98 (d, J = 9.4 Hz, 1H, H5), 3.95-3.84 (m, 7H, H5''', H5', H4', CO_2Me, ^{i}Pr-CH), 3.82-3.79 (m, 4H, CO_2Me, H3), 3.76 (t, J = 7.9 Hz, 1H, H3''), 3.46 (t, J = 9.3 Hz, 1H, H4'''), 1.13 (d, J = 6.2 Hz, 3H, ^{i}Pr-CH_3), 1.03 (m, 6H, H6''', H6'), 0.98 (d, J = 6.0 Hz, 3H, ^{i}Pr-CH_3), 0.79 (s, 9H, TBS), 0.75 (s, 9H, TBS), 0.11 (s, 3H, TBS), 0.05 (s, 3H, TBS), 0.00 (s, 3H, TBS), -0.04 (s, 3H, TBS); ^{13}C NMR (150 MHz, $CDCl_3$) δ = 168.8 (C), 165.9 (C), 165.2 (C), 165.1 (C), 137.6 (C), 137.5 (C), 136.1 (C), 133.4 (CH), 133.3 (CH), 133.2 (CH), 133.0 (C), 130.2 (C), 130.1 (CH), 130.0 (CH), 129.8 (CH), 129.7 (C), 128.6 (CH), 128.56 (CH), 128.5 (CH),

128.4 (CH), 128.3 (CH), 128.2 (CH), 128.1 (CH), 128.0 (CH), 127.9 (CH), 127.8 (CH), 127.7 (CH), 127.5 (CH), 126.4 (CH), 126.2 (CH), 126.1 (CH), 126.0 (CH), 100.0 (CH), 99.8 (CH), 98.4 (CH), 98.1 (CH), 81.5 (CH), 81.4 (CH), 80.5 (CH), 77.5 (CH), 77.4 (CH), 77.2 (CH), 75.5 (CH_2), 75.0 (CH), 74.3 (CH), 74.2 (CH_2), 74.1 (CH), 74.0 (CH_2), 73.5 (CH), 73.43 (CH), 73.4 (CH), 72.6 (CH), 71.2 (CH), 71.0 (CH), 68.8 (CH), 68.3 (CH), 53.2 (CH_3), 25.9 (CH_3), 23.3 (CH_3), 21.8 (CH_3), 18.1 (CH_3), 18.0 (CH_3), 17.9 (C), 17.8 (C), -4.2 (CH_3), -4.4 (CH_3), -4.5 (CH_3); HRMS m/z $(M+Na)^+$ calcd for $C_{94}H_{112}O_{25}Si_2Na^+$ 1720.6954 found 1720.6957.

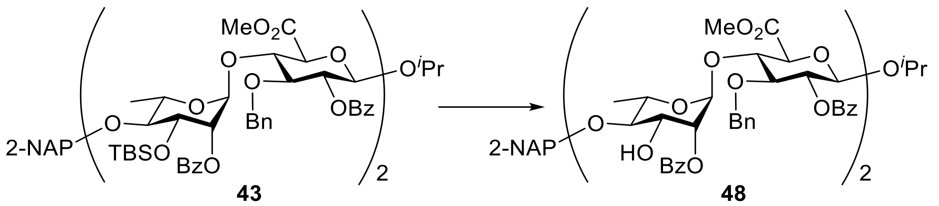

Compound 48. To a solution of compound **43** (337 mg, 0.2 mmol) in THF/pyridine (10.8 mL, 1/1), HF/pyridine (2.0 mL, 0.4 mmol) was added dropwise at 0 °C. The reaction mixture was gradually warmed up to room temperature and allowed to stir for 16 h. TLC (EtOAc/Hexane = 1/2, Rf = 0.21) analysis indicates the complete conversion of starting materials into products, silica gel was added to quench the excess HF/pyridine. The whole mixture was filtered and washed with CH_2Cl_2, filtrate was washed with sat. $NaHCO_{3(aq)}$ and H_2O. The organic layer was dried over anhydrous $MgSO_4$, filtered, and concentrated *in vacuo*. The crude residue was purified by column chromatography (EtOAc/Hexane = 1/2) on silica gel to afford the compound **48** (218 mg, 72%) as a white solid. $[\alpha]_D^{26}$ = +11.0 (c = 1.0, $CHCl_3$); IR (thin film in KBr) ν 3510, 2958, 2924, 2854, 1724, 1601, 1452, 1315, 1268, 1177, 1093, 1069, 1027, 812, 753, 711 cm^{-1}; ^1H NMR (600 MHz, $CDCl_3$) δ = 8.01 (d, *J* = 7.8 Hz, 2H, Ar-H), 7.98-7.94 (m, 6H, Ar-H), 7.78 (d, *J* = 8.0 Hz, 2H, Ar-H), 7.77-7.75 (m, 1H, Ar-H), 7.73 (s, 1H, Ar-H), 7.59-7.52 (m, 4H, Ar-H), 7.43-7.44 (m, 4H, Ar-H), 7.42-7.37 (m, 7H, Ar-H), 7.15-7.08 (m, 6H, Ar-H), 7.05-7.01 (m, 4H, Ar-H), 5.32 (t, *J* = 8.2 Hz, 1H, H2''), 5.25 (bs, 1H, H2'''), 5.22 (t, *J* = 8.2 Hz, 1H, H2), 5.12 (bs, 1H, H2'), 4.98 (d, *J* = 7.6 Hz, 1H, H1''), 4.89-4.88 (m, 3H, H1''', H1', CH_2), 4.82 (d, *J* = 11.4 Hz, 1H, CH_2), 4.67 (d, J = 10.8 Hz, 1H, CH_2), 4.62-4.60 (m, 3H, H1, CH_2, CH_2), 4.58 (d, *J* = 10.8 Hz, 1H, CH_2), 4.17-4.14 (m, 2H, H3''', H4''), 4.11 (t, *J* = 9.2 Hz, 1H, H4), 4.01 (d, *J* = 9.3 Hz, 1H, H5''), 3.98-3.94 (m, 1H, H5'''), 3.93 (d, *J* = 9.5 Hz, 1H, H5), 3.90-3.84 (m, 3H, H3', H5', ^{i}Pr-CH), 3.8 (s, 3H, CO_2Me), 3.75 (t, *J* = 8.8 Hz, 1H, H3), 3.74 (t, *J* = 8.5 Hz, 1H, H3''), 3.68 (s, 3H, CO_2Me), 3.53 (t, *J* = 9.5 Hz, 1H, H4'), 3.43 (t, *J* = 9.5 Hz,

1H, H4'''), 2.29 (m, 1H, OH), 2.17 (m, 1H, OH), 1.12 (d, J = 6.2 Hz, 3H, ^{i}Pr-CH_3), 1.06 (d, J = 6.1 Hz, 3H, H6'''), 1.01 (d, J = 6.1 Hz, 3H, H6'), 0.98 (d, J = 6.2 Hz, 3H, ^{i}Pr-CH_3); ^{13}C NMR (150 MHz, $CDCl_3$) δ = 169.0 (C), 168.8 (C), 166.4 (C), 166.2 (C), 165.2 (C), 165.1 (C), 137.4 (C), 137.3 (C), 135.7 (C), 133.7 (CH), 133.6 (CH), 133.5 (CH), 133.4 (C), 133.3 (CH), 133.2 (C), 130.1 (CH), 130.0 (CH), 129.9 (C), 129.89 (CH), 129.88 (CH), 129.72 (C), 129.7 (C), 129.6 (C), 128.8 (CH), 128.7 (CH), 128.67 (CH), 128.6 (CH), 128.5 (CH), 128.4 (CH), 128.34 (CH), 128.3 (CH), 128.0 (CH), 127.9 (CH), 127.7 (CH), 126.9 (CH), 126.4 (CH), 126.2 (CH), 126.1 (CH), 101.3 (CH), 99.9 (CH), 98.1 (CH), 97.7 (CH), 81.6 (CH), 81.1 (CH), 80.8 (CH), 80.6 (CH), 76.9 (CH), 76.3 (CH), 75.3 (CH_2), 74.9 (CH_2), 74.7 (CH), 74.5 (CH), 74.4 (CH_2), 73.9 (CH), 73.7 (CH), 73.0 (CH), 72.8 (CH), 72.6 (CH), 70.5 (CH), 70.1 (CH), 68.6 (CH), 67.6 (CH), 53.2 (CH_3), 53.0 (CH_3), 23.3 (CH_3), 21.8 (CH_3), 18.1 (CH_3), 17.5 (CH_3); HR HRMS m/z $(M+Na)^+$ calcd for $C_{82}H_{84}O_{25}Na^+$ 1491.5204 found 1491.5194.

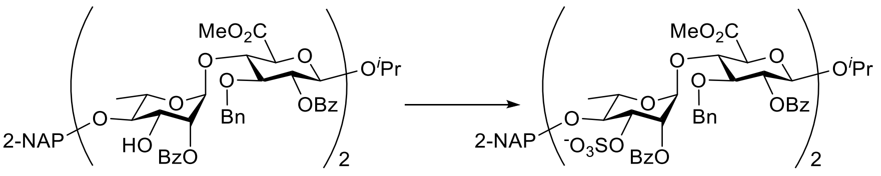

48 **52**

Compound 52. Compound **48** (60 mg, 20.0 µmol) and $SO_3 \cdot Et_3N$ (79.5 mg, 440.0 µmol) were dried on high vacuum for overnight, and then dissolved in anhydrous DMF (3.0 mL) under argon atmosphere. The reaction mixture was allowed to stir at 60 °C for 3 h. The reaction mixture was cooled to room temperature, sat. $NaHCO_{3\ (aq)}$ was added to quench the reaction and allowed to stir at room temperature for 3 h. The resulting mixture was extracted with THF, and the combined organic layers were washed with brine, dried over anhydrous $MgSO_4$, filtered, and concentrated *in vacuo*. The crude residue was subjected to AG 50W-X8 cation exchange column (Na^+ form) and LH-20 column by using MeOH as an eluent to afford the desired product **52** (30 mg, 82%) as white solid. ^{1}H NMR (600 MHz, CD_3OD) δ = 8.11 (d, J = 7.4 Hz, 2H, Ar-H), 8.00-7.96 (m, 6H, Ar-H), 7.89 (s, 1H, Ar-H), 7.84-7.82 (m, 3H, Ar-H), 7.64-7.58 (m, 4H, Ar-H), 7.50-7.42 (m, 9H, Ar-H), 7.37 (t, J = 7.7 Hz, 2H, Ar-H), 7.11-7.03 (m, 7H, Ar-H), 6.95 (t, J = 7.4 Hz, 1H, Ar-H), 6.85 (t, J = 7.5 Hz, 2H, Ar-H), 5.63 (bs, 1H, H2'''), 5.53 (bs, 1H, H2'), 5.49 (d, J = 7.7 Hz, 1H, H1''), 5.24 (t, J = 8.2 Hz, 1H, H2''), 5.21 (d, J = 11.1 Hz, 1H, CH_2), 5.08 (t, J = 9.1 Hz, 1H, H2), 4.99 (dd, J = 3.2, 9.2 Hz, 1H, H3'''), 4.95 (s, 1H, H1'''), 4.88 (s, 1H, H1'), 4.83 (dd, J = 3.1, 8.6 Hz, 1H, H3'), 4.82 (d, J = 11.0 Hz, 1H, CH_2), 4.78 (d, J =

5.3 Hz, 1H, H1), 4.73 (d, J = 10.1 Hz, 1H, CH_2), 4.59 (d, J = 10.1 Hz, 1H, CH_2), 4.47-4.46 (m, 2H, CH_2, CH_2), 4.31 (d, J = 9.2 Hz, 1H, H5''), 4.13-4.02 (m, 5H, H5, H5''', H3'', H4'', H4'), 3.97-3.83 (m, 5H, CO_2Me, H5', H4), 3.89-3.81 (m, 5H, CO_2Me, H3, ^{i}Pr-CH), 3.65 (t, J = 9.4 Hz, 1H, H4'''), 1.09 (d, J = 6.2 Hz, 3H, ^{i}Pr-CH_3), 0.95 (d, J = 6.1 Hz, 3H, ^{i}Pr-CH_3), 0.94 (d, J = 6.2 Hz, 3H, H6'''), 0.91 (d, J = 6.1 Hz, 3H, H6'); ^{13}C NMR (150 MHz, CD_3OD) δ = 170.96 (C), 170.9 (C), 167.4 (C), 167.0 (C), 166.8 (C), 166.7 (C), 138.6 (C), 138.5 (C), 137.4 (C), 134.7 (C), 134.6 (CH), 134.5 (C), 134.3 (CH), 134.1 (CH) , 131.4 (C), 131.2 (C), 131.0 (C), 130.8 (CH), 130.7 (CH), 130.6 (CH), 129.6 (CH), 129.5 (CH), 129.5 (CH), 129.45 (CH), 129.4 (CH), 129.1 (CH), 129.0 (CH), 128.9 (CH), 128.8 (CH), 128.65 (CH), 128.6 (CH), 128.5 (CH), 128.3 (CH), 128.1 (CH), 126.9 (CH), 126.8 (CH), 101.0 (CH), 100.6 (CH), 98.04 (CH), 98.0 (CH), 81.9 (CH), 81.1 (CH), 79.5 (CH), 77.6 (CH), 77.1 (CH), 76.7 (CH_2), 76.4 (CH_2), 75.7 (CH_2), 75.4 (CH), 75.31 (CH), 75.3 (CH), 74.0 (CH), 73.2 (CH), 72.5 (CH), 69.5 (CH), 68.9 (CH), 53.9 (CH_3), 53.8 (CH_3), 23.5 (CH_3), 22.2 (CH_3), 18.3 (CH_3), 18.1 (CH_3); HRMS m/z $(M+Na)^+$ calcd for $C_{82}H_{82}Na_2O_{31}S_2Na^+$ 1696.4002 found 1696.4001.

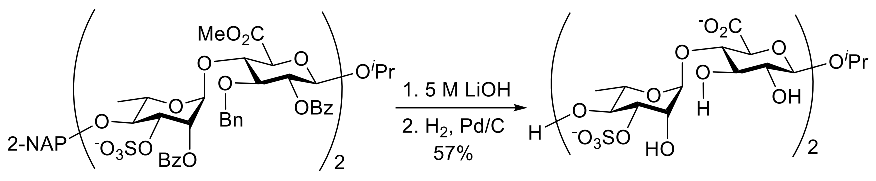

52 **2**

Compound 2. LiOH (5.0 M solution in water, 630 μL) was added to a solution of compound **52** (58 mg, 40.0 μmol) in $MeOH/CHCl_3/H_2O$ (4.0/0.9/0.6 mL). Then, the reaction mixture was allowed to stir at room temperature for 72 h. AG 50W-X8 cation exchange resin (H^+ form) was added to quench the reaction, and pH was adjusted to 7. The whole mixture was filtered and concentrated *in vacuo*. The crude residue was subjected to Sephadex LH-20 column and AG 50W-X8 cation exchange column (Na^+ form) by using MeOH as an eluent to afford the desired compound.

The obtained compound was dissolved in $MeOH/H_2O$ (2.0 mL, 1/1), $Pd(OH)_2$ on activated charcoal (20% Pd, wet, Degussa type, 348 mg) was added. The reaction was purged with hydrogen and then equipped with a hydrogen balloon at room temperature. After 72 h, the reaction mixture was filtered through a pad Celite® and the filtrate was concentrated *in vacuo*. The crude residue was purified by using a Sephadex G-10 column and AG 50W-X8 cation exchange column (Na^+ form) with water as eluent to obtain the desired product **2** (19 mg, 57%)

as a white solid. ^{1}H NMR (600 MHz, D_2O) δ = 4.75 (d, *J* = 1.3 Hz, 1H, H1'), 4.74 (d, *J* = 1.3 Hz, 1H, H1'''), 4.63 (d, *J* = 7.9 Hz, 1H, H1''), 4.59 (dd, *J* = 3.2, 9.6 Hz, 1H, H3'), 4.55 (d, *J* = 8.1 Hz, 1H, H1), 4.44 (dd, J = 3.2, 9.7 Hz, 1H, H3'''), 4.24-4.22 (m, 2H, H2', H2'''), 4.20-4.12 (m, 2H, H5', H5'''), 4.10-4.04 (m, 1H, ^{i}Pr-CH), 3.77-3.73 (m, 3H, H4', H4'', H4), 3.64 (t, *J* = 9.2 Hz, 1H, H3''), 3.60-3.53 (m, 4H, H4''', H5'', H3, H5), 3.35 (t, *J* = 8.9 Hz, 1H, H2''), 3.25 (t, *J* = 8.6 Hz, 1H, H2), 1.31 (d, *J* = 6.3 Hz, 3H, H6'), 1.27 (d, *J* = 6.3 Hz, 3H, H6'''), 1.22 (d, *J* = 6.2 Hz, 3H, ^{i}Pr-CH_3), 1.19 (d, *J* = 6.1 Hz, 3H, ^{i}Pr-CH_3); ^{13}C NMR (150 MHz, D_2O) δ = 175.4 (C), 175.3 (C), 103.2 (CH), 100.1 (CH), 100.0 (CH), 99.8 (CH), 78.9 (CH), 78.6 (CH), 78.4 (CH), 78.2 (CH), 78.0 (CH), 76.1 (CH), 76.0 (CH), 74.1 (CH), 73.8 (CH), 73.7 (CH), 73.3 (CH), 73.0 (CH), 69.9 (CH), 68.9 (CH), 68.8 (CH), 68.7 (CH), 68.0 (CH) , 22.2 (CH_3), 20.9 (CH_3), 16.8 (CH_3), 16.5 (CH_3); HRMS m/z $(M+H)^+$ calcd for $C_{27}H_{41}Na_4O_{27}S_2^+$ 953.0862 found 953.0867.

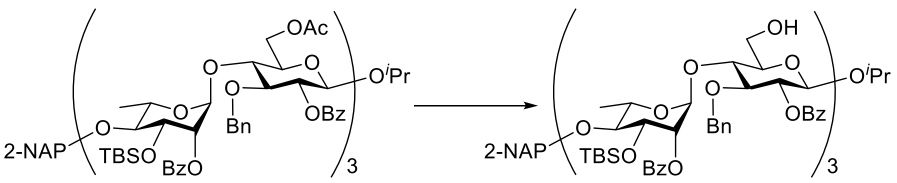

35 **40**

Compound 40. To a solution of compound **35** (300 mg, 120 μmol) in CH_2Cl_2 (3.0 mL), $Mg(OMe)_2$ (15.0 mL, 7-8% in MeOH) was added, and the reaction mixture was allowed to stir at room temperature for 10 h. TLC (EtOAc/Hexane = 1/1.5, Rf = 0.33) analysis indicates the complete conversion of starting materials into products. The reaction mixture was neutralized with 1 N HCl and washed with H_2O. The organic layer was dried over anhydrous $MgSO_4$, filtered, and concentrated *in vacuo*. The resulting crude residue was purified by flash column chromatography (EtOAc/Hexane = 1/1.5) to afford the desired product **40** (256 mg, 90%) as a white solid. $[\alpha]_D^{25}$ = -29.1 (*c* = 1.0, $CHCl_3$); IR (thin film in KBr) ν 3492, 2930, 1727, 1602, 1452, 1266, 1106, 1068, 837, 778, 752, 711 cm^{-1}; ^{1}H NMR (600 MHz, $CDCl_3$) δ = 8.06-8.04 (m, 2H, Ar-H), 7.99-7.94 (m, 10H, Ar-H), 7.83-7.78 (m, 3H, Ar-H), 7.74 (s, 1H, Ar-H), 7.59-7.50 (m, 5H, Ar-H), 7.46-7.33 (m, 15H, Ar-H), 7.17-7.08 (m, 13H, Ar-H), 7.02-6.96 (m, 3H, Ar-H), 5.35-5.29 (m, 2H, H2', H2''), 5.22-5.19 (m, 2H, H2, H2'''''), 5.16 (d, *J* = 7.1 Hz, 1H, H1''), 5.11 (d, *J* = 7.4 Hz, 1H, H1'), 5.08-5.02 (m, 6H, H2''', H1''', H2'''', H1'''', H1'''''), 4.76-4.70 (m, 3H, CH_2, CH_2, CH_2), 4.66-4.64 (d, 1H, CH_2), 4.61-4.55 (m, 4H, CH_2, CH_2, CH_2, H1), 4.22-4.20 (m, 1H, H3'''''), 4.10-4.07 (m, 2H, H3'''', H3'''), 4.04-3.91 (m, 6H, H5''''', H4', H4'', CH_2, CH_2, CH_2), 3.90-3.75 (m, 9H, CH_2, CH_2, CH_2, ^{i}Pr-CH, H3, H3'', H4''', H4'''', H5''''), 3.71 (t, *J* =

8.9 Hz, 1H, H3'), 3.51-3.46 (m, 2H, H5'', H4'''''), 3.43-3.41 (m, 2H, H5, H5'), 1.15 (d, J = 6.2 Hz, 3H, ^{i}Pr-CH_3), 0.98 (m, 6H, ^{i}Pr-CH_3, H6'''''), 0.91 (m, 6H, H6''', H6''''), 0.81 (s, 9H, TBS), 0.8 (s, 9H, TBS), 0.78 (s, 9H, TBS), 0.1 (s, 3H, TBS), 0.08 (s, 3H, TBS), -0.02 (s, 3H, TBS), -0.04 (s, 3H, TBS), -0.05 (s, 3H, TBS), -0.07 (s, 3H, TBS); ^{13}C NMR (150 MHz, $CDCl_3$) δ = 166.4 (C), 166.3 (C), 166.2 (C), 165.2 (C), 165.1 (C), 137.5 (C), 137.47 (C), 137.4 (C), 136.2 (C), 133.6 (CH), 133.5 (CH), 133.4 (CH), 133.3 (CH), 133.2 (CH), 133.0 (CH), 130.2 (C), 130.16 (CH), 130.1 (C), 130.05 (CH), 130.0 (C), 129.97 (CH), 129.9 (CH), 129.8 (CH), 128.6 (CH), 128.59 (CH), 128.5 (CH), 128.47 (CH), 128.4 (CH), 128.3 (CH), 128.2 (CH), 128.1 (CH), 128.0 (CH), 127.9 (CH), 127.8 (CH), 127.7 (CH), 127.5 (CH), 126.3 (CH), 126.2 (CH), 126.0 (CH), 125.9 (CH), 100.1 (CH), 99.8 (CH), 99.7 (CH), 97.9 (CH), 97.6 (CH), 81.6 (CH), 81.5 (CH), 81.2 (CH), 80.8 (CH) , 75.9 (CH), 75.86 (CH), 75.8 (CH), 75.5 (CH), 75.45 (CH2), 75.4 (CH), 75.0 (CH_2), 74.99 (CH_2), 74.9 (CH_2), 74.6 (CH), 74.5 (CH), 73.9 (CH), 73.7 (CH), 72.9 (CH), 71.2 (CH), 71.1 (CH), 71.0 (CH), 68.7 (CH), 68.0 (CH), 62.0 (CH_2), 61.9 (CH_2), 61.8 (CH_2), 26.0 (CH_3), 25.9 (CH_3), 23.4 (CH_3), 22.1 (CH_3), 18.0 (CH_3), 17.96 (CH_3), 17.93 (CH_3), -4.1 (CH_3), -4.2 (CH_3); HRMS m/z $(M+Na)^+$ calcd for $C_{133}H_{162}O_{35}Si_3Na^+$ 2427.0127 found 2427.0014.

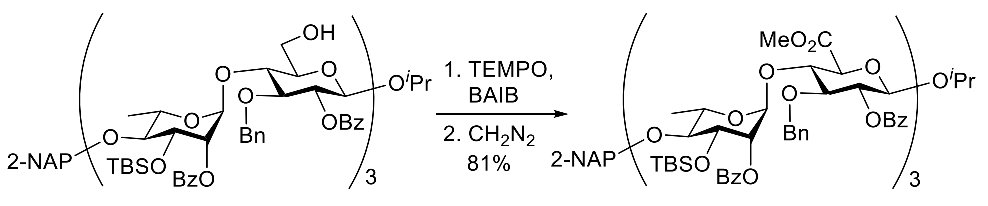

40 **44**

Compound 44. To a solution of compound **40** (90 mg, 40.0 μmol) in a mixed solvent CH_2Cl_2/H_2O (2.7 mL, 2/1), BAIB (92 mg, 290 μmol) and TEMPO (2.7 mg, 20.0 μmol) were added at 0 °C. Then, the reaction mixture was allowed to stir for 15 h at room temperature. EtOAc was added to dilute the reaction, and then the reaction mixture was washed with 10% $Na_2S_2O_3$ and brine. The organic layer was dried over anhydrous $MgSO_4$, filtered, and concentrated *in vacuo* to afford the crude acid, which was used for next step without doing any purification. Freshly prepared CH_2N_2 (1.03 mmol in Et_2O) was added to a solution of the acid compound (58.0 μmol) in THF (1.8 mL) at 0 °C. The reaction mixture was allowed to stir at room temperature for 0.5 h. TLC (EtOAc/Hexane = 1/2.6, Rf = 0.22) indicates the products formation, AcOH was added to quench the excess CH_2N_2, diluted with EtOAc, washed with sat. $NaHCO_{3(aq)}$ and brine. The organic layer was dried over anhydrous $MgSO_4$, filtered, and

concentrated *in vacuo*. The crude residue was purified by flash column chromatography (EtOAc/Hexane = 1/2.6) to afford the desired product **44** (81 mg, 81%) as a white solid. $[\alpha]_D^{27}$ = -8.25 (*c* = 1.0, $CHCl_3$); IR (thin film in KBr) ν 2928, 1732, 1452, 1373, 1264, 1095, 1069, 839, 755, 711 cm^{-1}; ^1H NMR (600 MHz, $CDCl_3$) δ = 8.03-8.02 (d, *J* = 7.7 Hz, 2H, Ar-H), 7.99-7.92 (m, 10H, Ar-H), 7.81-7.76 (m, 3H, Ar-H), 7.71 (s, 1H, Ar-H), 7.58-7.49 (m, 6H, Ar-H), 7.45-7.34 (m, 15H, Ar-H), 7.15-7.09 (m, 10H, Ar-H), 7.05-7.01 (m, 5H, Ar-H), 5.36-5.32 (m, 2H, H2'', H2''''), 5.24 (t, *J* = 7.8 Hz, 1H, H2), 5.19 (m, 2H, H2''''', H1''''), 5.15 (d, *J* = 6.5 Hz, 1H, H1''), 5.03 (m, 2H, H2', H2'''), 5.01 (d, *J* = 11.5 Hz, 1H, CH_2), 4.91 (m, 3H, H1', H1''', H1'''''), 4.76-4.72 (m, 3H, CH_2, CH_2, CH_2), 4.69 (d, *J* = 10.6 Hz, 1H, CH_2), 4.64 (m, 2H, H1, CH_2), 4.62 (d, *J* = 10.7 Hz, 1H, CH_2), 4.55 (d, *J* = 10.7 Hz, 1H, CH_2), 4.24 (t, *J* = 8.7 Hz, 1H, H4''''), 4.19 (t, *J* = 8.8 Hz, 1H, H4''''), 4.17-4.13 (m, 2H, H3''''', H4), 4.07-4.02 (m, 3H, H3', H3''', H3''''), 3.93 (m, 2H, H3, H3''), 3.94-3.78 (m, 16H, ^{i}Pr-CH, H5, H5', H5''', H5''''', H4', H4''', CH_3, CH_3, CH_3), 3.76 (t, *J* = 7.9 Hz, 1H, H5''''), 3.71 (t, *J* = 8.0 Hz, 1H, H5''), 3.45 (t, *J* = 9.3 Hz, 1H, H5'''''), 1.13 (d, *J* = 6.2 Hz, 3H, ^{i}Pr-CH_3), 1.02 (d, *J* = 6.1 Hz, 6H, H6''', H6'''''), 0.99 (d, *J* = 5.8 Hz, 3H, H6'), 0.97 (d, *J* = 6.1 Hz, 3H, ^{i}Pr-CH_3), 0.79 (s, 9H, TBS), 0.74 (s, 9H, TBS), 0.73 (s, 9H, TBS), 0.1 (s, 3H, TBS), 0.05 (s, 3H, TBS); ^{13}C NMR (150 MHz, CDCl3) δ = 168.9 (C), 168.8 (C), 168.6 (C), 165.8 (C), 165.2 (C), 165.1 (C), 137.7 (C), 137.6 (C), 137.5 (C), 136.1 (C), 133.5 (CH), 133.4 (C), 133.37 (CH), 133.3 (CH), 133.2 (CH), 133.0 (C), 130.3 (C), 130.1 (CH), 130.02 (CH), 130.0 (CH), 129.8 (CH), 129.78 (C), 129.7 (C), 128.6 (CH), 128.57 (CH), 128.55 (CH), 128.5 (CH), 128.4 (CH), 128.35 (CH), 128.3 (CH), 128.2 (CH), 128.1 (CH), 128.07 (CH), 128.0 (CH), 127.91 (CH), 127.9 (CH), 127.7 (CH), 127.6 (CH), 127.59 (CH), 126.5 (CH), 126.2 (CH), 126.1 (CH), 126.0 (CH), 100.05 (CH), 100.0 (CH), 99.8 (CH), 98.5 (CH), 98.4 (CH), 98.1 (CH), 81.5 (CH), 81.4 (CH), 81.1 (CH), 80.5 (CH), 78.0 (CH), 77.2 (CH), 75.5 (CH), 75.0 (CH), 74.3 (CH2), 75.0 (CH), 74.4 (CH), 74.3 (CH2), 74.1 (CH), 74.0 (CH2), 73.5 (CH), 73.49 (CH), 73.44 (CH), 73.4 (CH), 72.6 (CH), 71.2 (CH), 71.0 (CH), 70.9 (CH), 68.9 (CH), 68.4 (CH), 68.3 (CH), 53.2 (CH_3), 26.0 (CH_3), 25.9 (CH_3), 23.3 (CH_3), 21.8 (CH_3), 18.1 (CH_3), 18.06 (CH_3), 18.0 (CH_3), 17.9 (C), 17.8 (C), -4.1 (C), -4.2 (C), -4.4 (C), -4.51 (C), -4.55 (C); HRMS m/z $(M+H)^+$ calcd for $C_{134}H_{161}O_{37}Si_3^+$ 2447.0053 found 2447.0061.

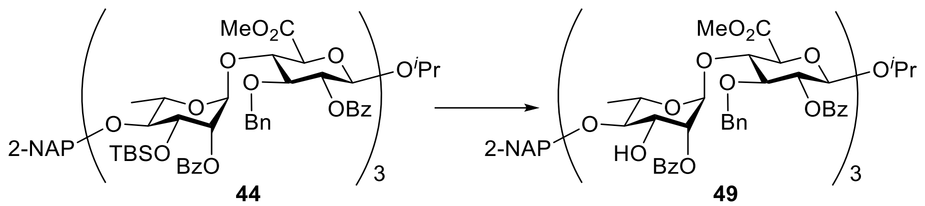

44 **49**

Compound 49. To a solution of compound **44** (255 mg, 0.1 mmol) in THF/pyridine (8.2 mL, 1/1), HF/pyridine (1.02 mL, 0.4 mmol) was added dropwise at 0 °C. The reaction mixture was gradually warmed up to room temperature and allowed to stir for 16 h. TLC (EtOAc/Hexane = 1/1.5, Rf = 0.39) analysis indicates the complete conversion of starting materials into products, silica gel was added to quench the excess HF/pyridine. The whole mixture was filtered and washed with CH_2Cl_2/MeOH (9/1), filtrate was washed with sat. $NaHCO_{3(aq)}$ and H_2O. The organic layer was dried over anhydrous $MgSO_4$, filtered, and concentrated *in vacuo*. The resulting crude residue was purified by column chromatography (EtOAc/Hexane = 1/2) on silica gel to afford the desired compound **49** (245 mg, 85%) as a white solid. $[\alpha]_D^{24}$ = +13.9 (*c* = 1.0, $CHCl_3$); IR (thin film in KBr) ν 3469, 2958, 2925, 2854, 1725, 1601, 1451, 1376, 1265, 1094, 1027, 723, 711 cm^{-1}; ^1H NMR (600 MHz, $CDCl_3$) δ = 8.01-7.99 (m, 4H, Ar-H), 7.96-7.94 (m, 8H, Ar-H), 7.80-7.73 (m, 2H, Ar-H), 7.77-7.75 (m, 1H, Ar-H), 7.73 (s, 1H, Ar-H), 7.59-7.50 (m, 6H, Ar-H), 7.46-7.35 (m, 15H, Ar-H), 7.14-7.06 (m, 12H, Ar-H), 7.04-7.01 (m, 3H, Ar-H), 5.31 (t, *J* = 8.3 Hz, 1H, H2"), 5.27 (t, *J* = 8.8 Hz, 1H, H2''''), 5.25 (bs, 1H, H2'''''), 5.21 (t, *J* = 8.2 Hz, 1H, H2), 5.11 (bs, 2H, H2', H2'''), 4.96 (d, *J* = 7.6 Hz, 1H, H1"), 4.91 (d, *J* = 7.6 Hz, 1H, H1''''), 4.90-4.88 (m, 3H, CH_2, H1', H1'''''), 4.84 (s, 1H, H1'''), 4.82 (d, *J* = 11.4 Hz, 1H, CH_2), 4.66 (d, *J* = 10.6 Hz, 1H, CH_2), 4.62-4.55 (m, 5H, H1, CH_2, CH_2, CH_2, CH_2), 4.54 (d, *J* = 10.7 Hz, 1H, CH_2), 4.16-4.13 (m, 2H, H3''''', H4"), 4.10-4.04 (m, 2H, H4, H4''''), 3.98 (d, *J* = 9.4 Hz, 1H, H5"), 3.97-3.93 (m, 1H, H5'''''), 3.9 (m, 2H, H5, H5''''), 3.89-3.83 (m, 5H, ^{i}Pr-CH, H3', H3''', H5', H5'''), 3.78 (s, 3H, CH_3), 3.74 (m, 2H, H3, H3"), 3.70-3.66 (m, 7H, H3'''', CH_3, CH_3), 3.52 (t, *J* = 9.4 Hz, 1H, H4'''), 3.5 (t, *J* = 9.5 Hz, 1H, H4'), 3.42 (t, *J* = 9.5 Hz, 1H, H4'''''), 223-2.22 (m, 2H, OH), 2.16 (d, *J* = 5.0 Hz, 1H, OH), 1.12 (d, *J* = 6.2 Hz, 3H, ^{i}Pr-CH_3), 1.05 (d, *J* = 6.1 Hz, 3H, H6'''''), 0.99 (d, *J* = 6.1 Hz, 3H, H6'''), 0.98 (d, *J* = 6.1 Hz, 6H, ^{i}Pr-CH_3, H6'); ^{13}C NMR (150 MHz, $CDCl_3$) δ = 169.0 (C), 168.8 (C), 168.7 (C), 166.43 (C), 166.4 (C), 166.2 (C), 165.18 (C), 165.16 (C), 165.1 (C), 137.3 (C), 137.2 (C), 137.1 (C), 135.7 (C), 133.7 (CH), 133.6 (CH), 133.5 (CH), 133.4 (C), 133.3 (CH), 133.2 (C), 130.1 (CH), 130.0 (CH), 129.9 (C), 129.89 (CH), 129.8 (CH), 129.7 (C), 129.67 (C), 129.6 (C), 129.5 (C), 128.8 (CH), 128.7 (CH), 128.6 (CH), 128.59 (CH), 128.5 (CH), 128.4 (CH), 128.35 (CH), 128.33

(CH), 128.3 (CH), 128.0 (CH), 127.9 (CH), 127.8 (CH), 127.79 (CH), 127.7 (CH), 126.9 (CH), 126.8 (CH), 126.4 (CH), 126.2 (CH), 126.1 (CH), 101.4 (CH), 101.3 (CH), 99.9 (CH), 98.1 (CH), 98.0 (CH), 97.7 (CH), 81.6 (CH), 81.2 (CH), 81.1 (CH), 80.8 (CH), 80.7 (CH), 80.6 (CH), 77.4 (CH), 76.9 (CH), 76.8 (CH), 76.3 (CH), 75.3 (CH_2), 74.9 (CH_2), 74.8 (CH_2), 74.7 (CH), 74.5 (CH), 74.4 (CH_2), 74.3 (CH), 74.0 (CH), 73.8 (CH), 73.7 (CH), 73.0 (CH), 72.8 (CH), 72.7 (CH), 72.6 (CH), 70.4 (CH), 70.0 (CH), 68.6 (CH), 67.7 (CH), 67.6 (CH), 53.2 (CH_3), 53.1 (CH_3), 53.0 (CH_3), 52.9 (CH_3), 29.9 (CH_2), 23.3 (CH_3), 21.8 (CH_3), 18.1 (CH_3), 17.5 (CH_3); HRMS m/z $(M+Na)^+$ calcd for $C_{116}H_{118}O_{37}Na^+$ 2104.7458 found 2104.7472.

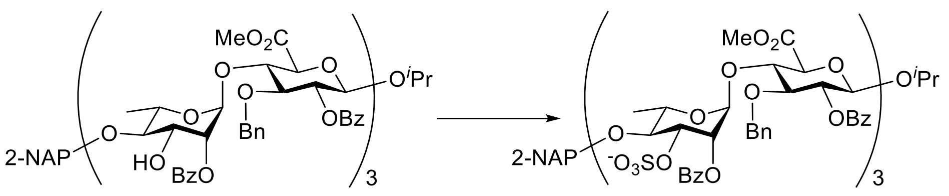

49 **53**

Compound 53. To a solution of compound **49** (60 mg, 20.0 μmol) in anhydrous DMF (3.0 mL), $SO_3 \cdot Et_3N$ (154 mg, 850.0 μmol) was added under argon atmosphere at room temperature. The reaction mixture was heated to 60 °C and allowed to stir at same temperature for 16 h. The reaction mixture was cooled to room temperature, sat. $NaHCO_3$ (aq) was added to quench the reaction and allowed to stir at room temperature for 3 h. The resulting mixture was extracted with THF, and the combined organic layers were washed with brine, dried over anhydrous $MgSO_4$, filtered, and concentrated *in vacuo*. The crude residue was subjected to AG 50W-X8 cation exchange column (Na^+ form) followed by LH-20 column using MeOH as an eluent to afford the desired product **53** (132 mg, 74%) as a white solid. ^{1}H NMR (600 MHz, CD_3OD) δ = 8.12 (d, *J* = 7.3 Hz, 2H, Ar-H), 8.07 (d, *J* = 7.2 Hz, 2H, Ar-H), 7.99-7.96 (m, 8H, Ar-H), 7.89 (s, 1H, Ar-H), 7.85-7.82 (m, 3H, Ar-H), 7.63-7.57 (m, 5H, Ar-H), 7.52-7.35 (m, 16H, Ar-H), 7.11-7.02 (m, 12H, Ar-H), 6.94 (t, *J* = 7.3 Hz, 3H, Ar-H), 6.85 (t, *J* = 7.6 Hz, 3H, Ar-H), 5.61 (m, 1H, H2'''''), 5.48 (m, 3H, H2', H2''', H1''''), 5.38 (d, *J* = 7.7 Hz, 1H, H1''), 5.23-5.16 (m, 3H, H2'', H2'''', CH_2), 5.08-5.06 (m, 1H, H2), 5.00-4.98 (m, 1H, H3'''''), 4.94 (d, *J* = 1.9 Hz, 1H, H1'''''), 4.91 (d, *J* = 2.5 Hz, 1H, H1'''), 4.87 (m, 1H, H1'), 4.82-4.77 (m, 4H, CH_2, H3', H3''', H1), 4.72 (d, *J* = 10.3 Hz, 1H, CH_2), 4.61 (d, *J* = 10.2 Hz, 1H, CH_2), 4.46-4.38 (m, 3H, CH_2, CH_2, CH_2), 4.28 (d, *J* = 9.1 Hz, 1H, H5''''), 4.17 (d, *J* = 9.7 Hz, 1H, H5''), 4.12-4.08 (m, 2H, H5, H5'''''), 4.07-4.04 (m, 2H, H4'''', H3''''), 4.01-3.97 (m, 3H, H4', H4'', H4'''), 3.95-3.78 (m, 15H, ^{i}Pr-CH, H5', H5''', H3, H3'', H4, CH_3, CH_3, CH_3), 3.64 (t, *J* = 9.4 Hz, 1H, H4'''''), 1.10 (d,

J = 6.2 Hz, 3H, ^{i}Pr-CH$_3$), 0.95 (d, J = 6.1 Hz, 3H, ^{i}Pr-CH$_3$), 0.93 (d, J = 6.1 Hz, 3H, H6'''''), 0.87-0.85 (m, 6H, H6', H6'''); ^{13}C NMR (150 MHz, CD$_3$OD) δ = 171.0 (C), 170.9 (C), 170.8 (C), 167.5 (C), 167.4 (C), 166.9 (C), 166.7 (C), 138.6 (C), 138.6 (C), 138.5 (C), 137.4 (C), 134.7 (C), 134.5 (CH), 134.3 (CH), 134.3 (CH), 134.3 (CH), 134.1 (CH), 131.4 (CH), 131.3 (C), 131.2 (C), 131.0 (C), 131.0 (C), 131.0 (C), 130.8 (CH), 130.8 (CH), 130.7 (CH), 130.6 (CH), 129.6 (CH), 129.6 (CH), 129.5 (CH), 129.4 (CH), 129.4 (CH), 129.1 (CH), 129.0 (CH), 128.8 (CH), 128.7 (CH), 128.6 (CH), 128.6 (CH), 128.5 (CH), 128.4 (CH), 128.3 (CH), 128.2 (CH), 126.9 (CH), 126.8 (CH), 101.0 (CH), 100.6 (CH), 100.4 (CH), 98.2 (CH), 97.9 (CH), 97.9 (CH), 81.8 (CH), 81.4 (CH), 81.1 (CH), 79.5 (CH), 77.6 (CH), 77.5 (CH), 77.0 (CH), 76.7 (CH$_2$), 76.4 (CH$_2$), 76.4 (CH$_2$), 75.6 (CH$_2$), 75.4 (CH), 75.4 (CH), 75.3 (CH), 75.2 (CH), 73.9 (CH), 73.2 (CH), 72.5 (CH), 72.4 (CH), 69.4 (CH), 68.9 (CH), 53.8 (CH$_3$), 53.8 (CH$_3$), 30.7 (CH$_3$), 23.5 (CH$_3$), 22.2 (CH$_3$), 18.3 (CH$_3$), 18.2 (CH$_3$), 18.1 (CH$_3$); HRMS m/z (M+H)$^{3-}$ calcd for $C_{116}H_{116}O_{46}S_3H_3^{3-}$ 780.1910 found 780.1957.

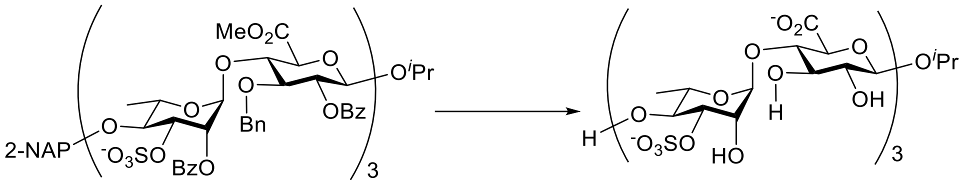

53 **3**

Compound 3. LiOH (2.0 mL, 5.0 M) was added to a solution of compound **53** (64.6 mg, 30.0 μmol) in MeOH/ CHCl$_3$/H$_2$O (7.3 mL, 4.6/1.0/0.7). Then, the reaction mixture was allowed to stir at room temperature for 72 h. After that AG 50W-X8 cation exchange resin was added to quench the reaction, and pH was adjusted to 7. Then, the whole mixture was filtered through a pad of Celite® and concentrated *in vacuo*. The crude residue was subjected to Sephadex LH-20 column followed by AG 50W-X8 cation exchange column (Na$^+$ form) using MeOH as an eluent to afford the desired compound. The obtained compound was dissolved in MeOH/ H$_2$O (2.0 mL, 1/1), Pd(OH)$_2$ on activated charcoal (20% Pd, wet, Degussa type, 384 mg) was added, and the reaction mixture was purged with hydrogen. Then, the reaction mixture was allowed to stir under hydrogen atmosphere at room temperature. After 72 h, the reaction mixture was filtered through a pad of Celite® and the filtrate was concentrated *in vacuo*. The crude residue was purified by using a Sephadex G-10 column and AG 50W-X8 cation exchange column (Na$^+$ form) by using water as eluent to obtain the desired product **3** (26 mg, 69%) as a white solid. ^{1}H NMR (600 MHz, D$_2$O) δ = 4.76 (s, 1H, H1'''''), 4.75 (bs, 2H, H1', H1'''), 4.64 (d, J = 7.9 Hz,

2H, H1'', H1''''), 4.61-4.58 (m, 2H, H3', H3'''), 4.55 (d, J = 8.0 Hz, 1H, H1), 4.46-4.43 (m, 1H, H3'''''), 4.24-4.23 (m, 3H, H2''''', H2', H2'''), 4.20-4.14 (m, 3H, H5', H5''', H5'''''), 4.09-4.05 (m, 1H, ^{i}Pr-CH), 3.77-3.73 (m, 5H, H4, H4', H4'', H4''', H4''''), 3.64 (m, 2H, H3'', H3''''), 3.60-3.54 (m, 5H, H3, H4''''', H5, H5'', H5''''), 3.37-3.33 (m, 2H, H2'', H2''''), 3.26 (t, J = 8.4 Hz, 1H, H2), 1.31 (d, J = 6.2 Hz, 6H, H6', H6'''), 1.28 (d, J = 6.2 Hz, 3H, H6'''''), 1.23 (d, J = 6.2 Hz, 3H, ^{i}Pr-CH_3), 1.19 (d, J = 6.2 Hz, 3H, ^{i}Pr-CH_3); ^{13}C NMR (150 MHz, D_2O) δ = 175.46 (C), 175.4 (C), 175.3 (C), 103.2 (CH), 100.1 (CH), 100.0 (CH), 99.85 (CH), 99.8 (CH), 78.9 (CH), 78.8 (CH), 78.7 (CH), 78.4 (CH), 78.1 (CH), 78.09 (CH), 78.0 (CH), 76.2 (CH), 76.1 (CH), 76.06 (CH), 74.1 (CH), 73.8 (CH), 73.3 (CH), 73.1 (CH), 69.8 (CH), 68.9 (CH), 68.8 (CH), 68.7 (CH), 68.1 (CH), 68.0 (CH), 22.2 (CH_3), 20.9 (CH_3), 16.8 (CH_3), 16.5 (CH_3); HRMS m/z $(M+4Na+H)^{2-}$ calcd for $C_{39}H_{57}O_{40}S_3H_2Na_4^{2-}$ 676.0551 found 676.0556.

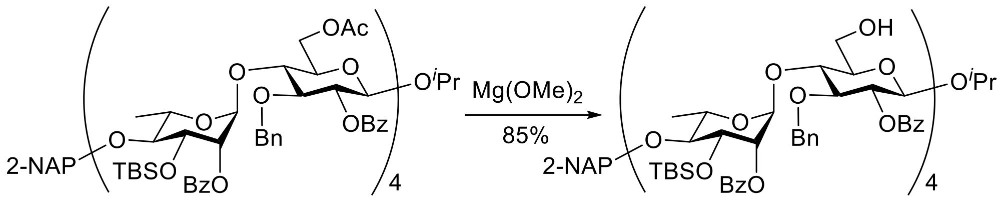

37 **41**

Compound 41. To a solution of compound **37** (100 mg, 310.0 μmol) in CH_2Cl_2 (1.0 mL), $Mg(OMe)_2$ (0.5 mL, 7-8% in MeOH) was added under nitrogen atmosphere, and the reaction mixture was allowed to stir for 12 h. TLC (EtOAc/Hexane = 1/1.5, Rf = 0.50) analysis indicates the complete conversion of starting materials into products. The reaction mixture was neutralized with 1 N HCl and washed with H_2O. The organic layer was dried over anhydrous $MgSO_4$, filtered, and concentrated *in vacuo*. The resulting crude residue was purified by column chromatography (EtOAc/Hexane = 1/3) on silica gel to afford the desired compound **41** (62 mg, 85%) as white solid. $[\alpha]_D^{27}$ = -20.9 (c = 1.0, $CHCl_3$); IR (thin film in KBr) ν 3503, 2930, 2858, 1727, 1602, 1452, 1266, 1107, 1069, 1028, 860, 837, 778, 753, 711 cm^{-1}; ^{1}H NMR (600 MHz, $CDCl_3$) δ = 8.06 (d, J = 7.5 Hz, 2H, Ar-H), 8.00-7.95 (m, 14H, Ar-H), 7.83-7.78 (m, 3H, Ar-H), 7.75 (s, 1H, Ar-H), 7.59-7.49 (m, 8H, Ar-H), 7.48-7.33 (m, 19H, Ar-H), 7.18-7.09 (m, 17H, Ar-H), 7.03-6.97 (m, 3H, Ar-H), 5.35-5.29 (m, 3H, H2'', H2'''', H2''''''), 5.23-5.20 (m, 2H, H2, H2'''''''), 5.17 (d, J = 7.3 Hz, 1H, H1'''''''), 5.11-5.03 (m, 10H, H1'', H1'''', H2', H2''', H2''''', H1', H1''', H1''''', H1''''''', CH_2), 4.77-4.71 (m, 3H, CH_2, CH_2, CH_2), 4.66-4.56 (m, 7H, H1, CH_2, CH_2, CH_2, CH_2, CH_2, CH_2), 4.23-4.21 (m, 1H, H3'''''''), 4.11-4.07 (m, 3H, H3', H3''', H3'''''), 4.05-3.75 (m, 22H, H4'', H4'''', H4'''''', H5''''''', H6a, H6a'', H6a'''', H6a'''''', ^{i}Pr-CH, H4, H4', H4''',

H4'''', H3, H3''''', H6b, H6b'', H6b'''', H6b''''', H5', H5''', H5'''''), 3.73-3.70 (m, 2H, H3'', H3''''), 3.51-3.47 (m, 2H, H4'''''', H5''''''), 3.44-3.42 (m, 3H, H5, H5'', H5''''), 2.10-2.08 (m, 1H, OH), 2.02-1.98 (m, 3H, OH, OH, OH), 1.16 (d, J = 6.2 Hz, 3H, IPA-CH_3), 0.98 (d, J = 6.1 Hz, 6H, ^{i}Pr-CH_3, H6''''''), 0.92 (d, J = 5.8 Hz, 6H, H6'''', H6'''), 0.89 (d, J = 6.0 Hz, 3H, H6'), 0.82 (s, 9H, TBS), 0.81 (s, 9H, TBS), 0.79 (s, 18H, TBS), 0.11 (s, 3H, TBS), 0.09 (s, 3H, TBS), -0.01 (s, 3H, TBS), -0.03 (s, 6H, TBS), -0.04 (s, 3H, TBS), -0.06 (s, 6H, TBS); ^{13}C NMR (150 MHz, $CDCl_3$) δ = 166.4 (C), 166.21 (C), 166.2 (C), 165.1 (C), 137.5 (C), 137.41 (C), 137.4 (C), 136.2 (C), 133.5 (CH), 133.4 (CH), 133.4 (CH), 133.2 (CH), 133.2 (CH), 133.2 (CH), 133 (C), 130.1 (CH), 130.1 (CH), 130.0 (CH), 129.93 (CH), 129.92 (CH), 129.9 (CH), 129.8 (CH), 128.6 (CH), 128.51 (CH), 128.5 (CH), 128.41 (CH), 128.4 (CH), 128.3 (CH), 128.2 (CH), 128.01 (CH), 128.0 (CH), 127.8 (CH), 127.71 (CH), 127.7 (CH), 127.5 (CH), 126.3 (CH), 126.2 (CH), 126.0 (CH), 125.9 (CH), 100.1 (CH), 99.7 (CH), 99.7 (CH), 99.7 (CH), 97.8 (CH), 97.6 (CH), 81.6 (CH), 81.5 (CH), 81.2 (CH), 81.2 (CH), 80.8 (CH), 77.4 (CH), 75.9 (CH), 75.8 (CH), 75.8 (CH), 75.5 (CH), 75.4 (CH_2), 75.4 (CH), 75.1 (CH_2), 75.0 (CH2), 74.9 (CH_2), 74.9 (CH), 74.9 (CH_2), 74.6 (CH), 74.5 (CH), 73.9 (CH), 73.7 (CH), 73.7 (CH), 72.9 (CH), 71.2 (CH), 71.01 (CH), 71.0 (CH), 68.7 (CH), 68.1 (CH), 68.0 (CH), 62.0 (CH_2), 61.9 (CH_2), 61.8 (CH_2), 26.0 (CH), 25.9 (CH), 23.4 (CH), 22.1 (CH), 18.0 (CH_3), 17.9 (CH_3), 17.8 (CH_3), -4.1 (CH_3), -4.12 (CH_3), -4.2 (CH_3), -4.3 (CH_3), -4.31 (CH_3), -4.4 (CH_3); HRMS m/z $(M+Na)^+$ calcd for $C_{170}H_{208}O_{45}Si_4Na^+$ 3106.3012 found 3106.2884.

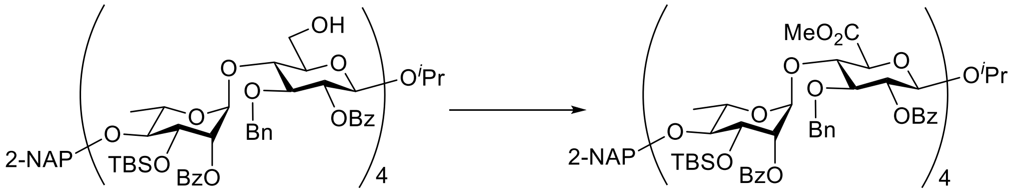

41 **45**

Compound 45. To a solution of compound **41** (243 mg, 80.0 μmol) in CH_2Cl_2/H_2O (7.3 mL, 2/1), BAIB (191 mg, 590.0 mol) and TEMPO (5.6 mg, 40.0 μmol) were added at 0°C. Then, the reaction mixture was allowed to stir at room temperature for 19 h. The reaction mixture was diluted with CH_2Cl_2, washed with 10 % $Na_2S_2O_3$ solution and water. The organic layer was dried over anhydrous $MgSO_4$, filtered, and concentrated *in vacuo* to afford the crude acid, which was used for next step without doing any purification. Freshly prepared CH_2N_2 (2.1 mmol in Et_2O) was added to a solution of crude acid (80.0 μmol) in THF (4.9 mL) at 25 °C. The reaction mixture was allowed to stir at room temperature for 0.5 h. TLC (EtOAc/Hexane

= 1/3.3, Rf = 0.18) indicates the products formation, AcOH was added to quench the excess CH_2N_2, diluted with EtOAc, washed with sat. $NaHCO_{3(aq)}$ and brine. The organic layer was dried over anhydrous $MgSO_4$, filtered, and concentrated *in vacuo*. The crude residue was purified by column chromatography (EtOAc/Hexane = 1/3) to afford the desired compound **45** (198 mg, 78%) as a white solid. $[\alpha]_D^{20}$ = -12.3 (c = 1.0, $CHCl_3$); IR (thin film in KBr) ν 3737, 2954, 2858, 1727, 1602, 1452, 1265, 1095, 1069, 838, 778, 754, 711 cm^{-1}; ^{1}H NMR (600 MHz, $CDCl_3$) δ = 8.03 (d, *J* = 7.7 Hz, 2H, Ar-H), 7.99-7.92 (m, 14H, Ar-H), 7.81-7.76 (m, 3H, Ar-H), 7.71 (s, 1H, Ar-H), 7.58-7.49 (m, 8H, Ar-H), 7.45-7.33 (m, 20H, Ar-H), 7.15-7.09 (m, 14H, Ar-H), 7.05-7.01 (m, 6H, Ar-H), 5.36-5.31 (m, 3H, H2'', H2'''', H2''''''), 5.23 (t, *J* = 8.0 Hz, 1H, H2), 5.19 (m, 2H, H2''''''', H1''''''), 5.13 (t, *J* = 5.6 Hz, 2H, H1'', H1''''), 5.03-5.02 (m, 3H, H2', H2''', H2'''''), 5.01 (t, *J* = 11.5 Hz, 1H, CH_2), 4.90-4.89 (m, 4H, H1', H1''', H1''''', H1'''''''), 4.74-4.72 (m, 3H, CH_2, CH_2, CH_2), 4.69 (d, *J* = 10.8 Hz, 2H, CH_2, CH_2), 4.64-4.61 (m, 3H, H1, CH_2, CH_2), 4.61 (d, *J* = 10.7 Hz, 1H, CH_2), 4.56 (d, *J* = 10.6 Hz, 1H, CH_2), 4.24 (t, *J* = 9.0 Hz, 1H, H4''''''), 4.19-4.13 (m, 4H, H4, H4'', H4'''', H3'''''''), 4.06-4.02 (m, 4H, H3', H3''', H3''''', H3''''''), 3.99 (m, 3H, H3, H3'', H3''''), 3.93-3.74 (m, 23H, CH_3, CH_3, CH_3, CH_3, ^{i}Pr-CH, H5, H5', H5''', H5''''', H5'''''', H5''''''', H4', H4''', H4''''', H4'''''''), 3.71 (t, *J* = 8.0 Hz, 2H, H5'', H5''''), 3.45 (t, *J* = 9.3 Hz, 1H, H5''''''), 1.13 (d, *J* = 6.2 Hz, 3H, ^{i}Pr-CH_3), 1.02 (d, *J* = 6.1 Hz, 6H, H6''''', H6'''''''), 1.00-0.96 (m, 9H, H6', H6''', ^{i}Pr-CH_3), 0.79 (s, 9H, TBS), 0.74 (s, 9H, TBS), 0.73 (s, 9H, TBS), 0.72 (s, 9H, TBS), 0.1 (s, 3H, TBS), 0.05 (s, 3H, TBS), -0.03 (s, 6H, TBS), -0.04 (s, 3H, TBS); ^{13}C NMR (150 MHz, $CDCl_3$) δ = 168.9 (C), 168.8 (C), 168.6 (C), 165.8 (C), 165.2 (C), 165.15 (C), 165.1 (C), 137.6 (C), 137.59 (C), 137.5 (C), 136.1 (C), 133.45 (CH), 133.4 (C), 133.33 (CH), 133.3 (CH), 133.2 (CH), 133.0 (C), 130.2 (CH), 130.1 (CH), 130.0 (CH), 129.8 (CH), 129.7 (CH), 128.6 (CH), 128.56 (CH), 128.54 (CH), 128.5 (CH), 128.4 (CH), 128.34 (CH), 128.3 (CH), 128.2 (CH), 128.1 (CH), 128.06 (CH), 128.0 (CH), 127.9 (CH), 127.7 (CH), 127.6 (CH), 126.4 (CH), 126.2 (CH), 126.1 (CH), 126.0 (CH), 100.0 (CH), 99.9 (CH), 99.8 (CH), 98.5 (CH), 98.3 (CH), 98.1 (CH), 81.5 (CH), 81.4 (CH), 81.1 (CH), 80.5 (CH), 78.0 (CH), 77.9 (CH), 77.5 (CH), 77.4 (CH), 77.2 (CH), 75.5 (CH), 75.0 (CH2), 74.33 (CH), 74.3 (CH_2), 74.1 (CH), 74.0 (CH_2), 73.5 (CH), 73.48 (CH), 73.43 (CH), 73.4 (CH), 72.6 (CH), 71.2 (CH), 71.0 (CH), 70.9 (CH), 70.8 (CH), 68.8 (CH), 68.31 (CH), 68.3 (CH), 53.2 (CH), 53.1 (CH), 25.9 (CH), 23.3 (CH), 21.8 (CH), 18.1 (CH_3), 18.04 (CH_3), 18.0 (CH_3), 17.9 (C), 17.8 (C), -4.2 (CH_3), -4.24 (CH_3), -4.4 (CH_3), -4.51 (CH_3), -4.5 (CH_3); HRMS m/z $(M+H)^+$ calcd for $C_{174}H_{209}O_{49}Si_4^+$ 3196.3001 found 3196.2994.

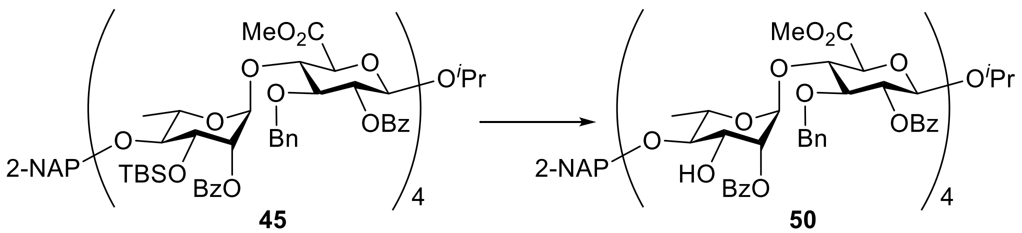

45 **50**

Compound 50. To a solution of compound **45** (439 mg, 140 μmol) in THF/pyridine (14.0 mL, 1/1), HF/pyridine (1.76 mL, 40.0 μmol) was added dropwise at 0 °C. The reaction mixture was gradually warmed up to room temperature and allowed to stir for 16 h. TLC (EtOAc/Hexane = 1/1, Rf = 0.30) analysis indicates the complete conversion of starting materials into products, silica gel was added to quench the excess HF/pyridine. The whole mixture was filtered, solids were washed with CH_2Cl_2/MeOH (9/1), and filtrate was washed with sat. $NaHCO_{3(aq)}$ and H_2O. The organic layer was dried over anhydrous $MgSO_4$, filtered, and concentrated *in vacuo*. The resulting crude residue was purified by column chromatography (EtOAc/Hexane = 1/1) on silica gel to afford the desired compound **50** (316 mg, 84%) as a white solid. $[\alpha]_D^{26.3}$ = +8.05 (c = 1.0, $CHCl_3$); IR (thin film in KBr) ν 2566, 2935, 1727, 1602, 1451, 1266, 1094, 1069, 1028, 753, 711 cm^{-1}; ^1H NMR (600 MHz, $CDCl_3$) δ = 8.00-7.97 (m, 6H, Ar-H), 7.96-7.91 (m, 10H, Ar-H), 7.81-7.78 (m, 2H, Ar-H), 7.77-7.74 (m, 1H, Ar-H), 7.73 (s, 1H, Ar-H), 7.58-7.49 (m, 8H, Ar-H), 7.46-7.34 (m, 19H, Ar-H), 7.13-7.00 (m, 20H, Ar-H), 5.32 (m, 1H, H2''), 5.27-5.24 (m, 3H, H2'''', H2'''''', H2'''''''), 5.21 (m, 1H, H2), 5.10-5.08 (m, 3H, H2'. H2''', H2'''''), 4.96 (d, 1H, H1''), 4.90-4.87 (m, 5H, H1', H1'''', H1'''''', H1''''''', CH_2), 4.84 (d, *J* = 6.1 Hz, 2H, H1''', H1'''''), 4.81 (d, *J* = 11.4 Hz, 1H, CH_2), 4.66 (d, *J* = 10.6 Hz, 1H, CH_2), 4.61 (d, *J* = 7.3 Hz, 2H, CH_2, CH_2), 4.58-4.55 (m, 4H, H1, CH_2, CH_2, CH_2), 4.53 (d, *J* = 10.7 Hz, 2H, CH_2, CH_2), 4.16-4.12 (m, 2H, H3'''''', H4''), 4.09 (t, *J* = 9.2 Hz, 1H, H4), 4.04 (t, *J* = 9.1 Hz, 2H, H4'''', H4''''''), 3.99 (d, *J* = 9.3 Hz, 1H, H5''), 3.96-3.93 (m, 1H, H5''''''), 3.91 (d, *J* = 9.4 Hz, 1H, H5), 3.89-3.81 (m, 9H, H5'''', H5''''''', ^{i}Pr-CH, H3', H3''', H3''''', H5', H5''', H5'''''), 3.79 (s, 3H, CH_3), 3.73 (m, 2H, H3, H3''), 3.69-3.66 (m, 11H, H3'''', H3''''''', CH_3, CH_3, CH_3), 3.53-3.47 (m, 3H, H4', H4''', H4'''''), 3.42 (t, *J* = 9.4 Hz, 1H, H4'''''''), 2.21-2.18 (m, 3H, OH, OH, OH), 2.12 (m, 1H, OH), 1.12 (d, *J* = 6.2 Hz, 3H, ^{i}Pr-CH_3), 1.05 (d, *J* = 6.2 Hz, 3H, H6'''''''), 0.99-0.95 (m, 9H, ^{i}Pr-CH_3, H6', H6''', H6'''''); ^{13}C NMR (150 MHz, $CDCl_3$) δ = 207.2 (C), 169.0 (C), 168.8 (C), 168.7 (C), 166.4 (C), 166.2 (C), 165.2 (C), 165.16 (C), 165.1 (C), 137.3 (C), 137.2 (C), 137.1 (C), 135.7 (C), 133.7 (CH), 133.68 (CH), 133.65 (CH), 133.6 (CH), 133.5 (CH), 133.4 (C), 133.3 (CH), 133.2 (C), 130.1 (CH), 130.0 (CH), 129.9 (CH), 129.8 (CH), 129.7 (CH), 129.5 (CH), 128.8 (CH), 128.68 (CH), 128.6 (CH), 128.5 (CH), 128.4 (CH), 128.34 (CH), 128.3 (CH),

128.0 (CH), 127.9 (CH), 127.83 (CH), 127.8 (CH), 127.7 (CH), 126.9 (CH), 126.4 (CH), 126.2 (CH), 126.1 (CH), 101.3 (CH), 99.9 (CH), 98.1 (CH), 98.0 (CH), 97.7 (CH), 81.6 (CH), 81.2 (CH), 81.1 (CH), 80.8 (CH), 80.6 (CH), 77.4 (CH), 76.9 (CH), 76.8 (CH), 76.3 (CH), 75.3 (CH_2), 74.9 (CH_2), 74.8 (CH_2), 74.7 (CH), 74.5 (CH), 74.4 (CH_2), 74.3 (CH), 74.0 (CH), 73.7 (CH), 73.0 (CH), 72.8 (CH), 72.7 (CH), 72.6 (CH), 70.5 (CH), 70.0 (CH), 68.6 (CH), 67.7 (CH), 67.6 (CH), 53.2 (CH_3), 53.0 (CH_3), 31.1 (CH_3), 23.3 (CH_3), 21.8 (CH_3), 18.1 (CH_3), 17.5 (CH_3); HRMS m/z $(M+2Na)^{2-}$ calcd for $C_{150}H_{152}O_{49}Na_2^{2-}$ 1391.9610 found 1391.9574.

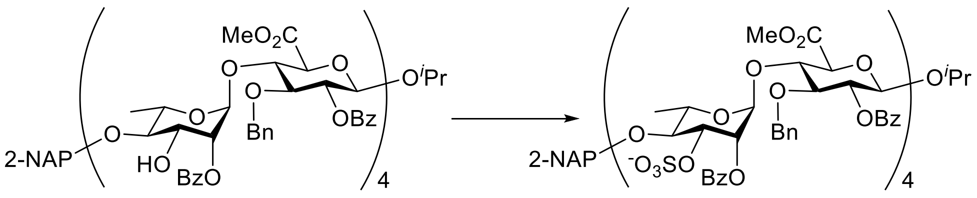

50 **54**

Compound 54. To a solution of compound **50** (63 mg, 20.0 μmol) in anhydrous DMF (3.1 mL), SO_3•Et_3N (125 mg, 690 μmol) was added under argon atmosphere at room temperature. Then, the reaction mixture was heated to 60 °C and allowed to stir at same temperature for 16 h. After that the reaction mixture was cooled to room temperature, sat. $NaHCO_{3\ (aq)}$ was added to quench the reaction and allowed to stir at room temperature for 3 h. The resulting mixture was extracted with THF, and the combined organic layers were washed with brine, dried over anhydrous $MgSO_4$, filtered, and concentrated *in vacuo*. The crude residue was subjected to AG 50W-X8 cation exchange column (Na^+ form) followed by LH-20 column using MeOH as an eluent to afford the desired product **54** (59 mg, 82%) as a white solid. ^{1}H NMR (600 MHz, CD_3OD) δ = 8.12 (m, 2H, Ar-H), 8.07-8.05 (m, 4H, Ar-H), 7.99-7.96 (m, 10H, Ar-H), 7.89 (m, 2H, Ar-H), 7.84-7.82 (m, 3H, Ar-H), 7.63-7.57 (m, 7H, Ar-H), 7.52-7.42 (m, 15H, Ar-H), 7.39-7.35 (m, 6H, Ar-H), 7.10-7.02 (m, 17H, Ar-H), 6.94 (t, *J* = 7.4 Hz, 1H, Ar-H), 6.85 (t, *J* = 7.6 Hz, 2H, Ar-H), 5.61 (bs, 1H, H2'''''''), 5.50-5.49 (m, 4H, H2', H2''', H2''''', H1'''''''), 5.39-5.37 (m, 2H, H1'', H1''''), 5.23-5.14 (m, 4H, H2'', H2'''', H2'''''', CH_2), 5.07 (t, J = 8.6 Hz, 1H, H2), 4.99-4.97 (m, 1H, H3'''''''), 4.94 (s, 1H, H1''''''), 4.93-4.77 (m, 7H, H1', H1''', H1''''', H3', H3''', H3''''', H1), 4.72 (d, *J* = 10.1 Hz, 1H, CH_2), 4.61 (d, J = 10.1 Hz, 1H, CH_2), 4.45-4.37 (m, 6H, CH_2, CH_2, CH_2, CH_2, CH_2, CH_2), 4.29 (d, *J* = 8.8 Hz, 1H, H5'''''), 4.16-4.14 (m, 2H, H5'', H5''''), 4.12-4.08 (m, 2H, H5, H5''''''), 4.07-4.04 (m, 2H, H4''''''', H3''''''), 4.00-3.77 (m, 25H, ^{i}Pr-CH, H5', H5''', H5''''', H3, H3'', H3'''', H4, H4', H4'', H4''', H4'''', H4''''', CH_3, CH_3, CH_3, CH_3), 3.64 (t, *J* = 9.3 Hz, 1H, H4''''''), 1.09 (d, *J* = 6.1 Hz, 3H, ^{i}Pr-CH_3), 0.95 (d, *J* = 6.1 Hz, 3H, ^{i}Pr-CH_3),

0.92 (d, *J* = 5.9 Hz, 3H, H6'''''''), 0.87 (d, *J* = 5.8 Hz, 6H, H6', H6'''), 0.84 (d, *J* = 5.9 Hz, 3H, H6'''''); ^{13}C NMR (150 MHz, CD_3OD) δ = 170.95 (C), 170.9 (C), 170.7 (C), 167.5 (C), 167.4 (C), 166.8 (C), 166.73 (C), 166.7 (C), 138.7 (C), 138.6 (C), 138.5 (C), 137.4 (C), 134.7 (C), 134.5 (CH), 134.4 (C), 134.3 (C), 134.1 (C), 131.4 (CH), 131.35 (CH), 131.3 (CH), 131.0 (CH), 130.8 (CH), 130.7 (CH), 130.6 (CH), 129.7 (CH), 129.6 (CH), 129.5 (CH), 129.41 (CH), 129.4 (CH), 129.1 (CH), 129.0 (CH), 128.9 (CH), 128.7 (CH), 128.65 (CH), 128.6 (CH), 128.5 (CH), 128.4 (CH), 128.3 (CH), 128.2 (CH), 126.9 (CH), 126.8 (CH), 101.0 (CH), 100.6 (CH), 100.5 (CH), 100.4 (CH), 98.1 (CH), 97.9 (CH), 97.8 (CH), 81.8 (CH), 81.4 (CH), 81.2 (CH), 77.5 (CH), 77.1 (CH), 76.7 (CH_2), 76.5 (CH_2), 76.4 (CH_2), 75.7 (CH_2), 75.5 (CH), 75.4 (CH), 75.3 (CH), 73.9 (CH), 73.2 (CH), 72.5 (CH), 69.4 (CH), 68.9 (CH), 53.8 (CH), 30.7 (CH), 23.5 (CH_3), 22.2 (CH_3), 18.3 (CH_3), 18.2 (CH_3); HRMS m/z $(M+H)^{3-}$ calcd for $C_{150}H_{149}H_4O_{61}S_4^{3-}$ 1018.2497 found 1018.2438.

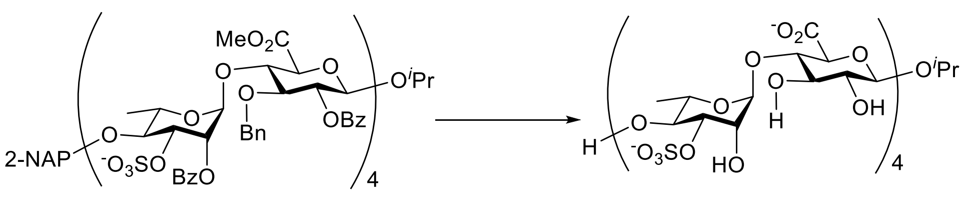

54 **4**

Compound 4. LiOH (5.0 M solution in water, 2.0 mL) was added to a solution of compound **54** in MeOH/$CHCl_3$/H_2O (4.6/1.0/0.7). Then, the reaction mixture was allowed to stir at room temperature for 72 h. Afterwards, AG 50W-X8 cation exchange resin was added to quench the reaction, and pH was adjusted to 7. The whole mixture was then filtered through a pad of Celite® and concentrated *in vacuo*. The crude residue was subjected to Sephadex LH-20 column followed by AG 50W-X8 cation exchange column (Na^+ form) using MeOH as an eluent to afford the desired deacylated compound. The obtained compound was dissolved in MeOH/ H_2O (2.0 mL, 1/1), $Pd(OH)_2$ on activated charcoal (20% Pd, wet, Degussa type, 300 mg) was added, and the reaction mixture was purged with hydrogen. Then, the reaction mixture was allowed to stir under hydrogen atmosphere at room temperature. After 72 h, the reaction mixture was filtered through a pad of Celite® and the filtrate was concentrated *in vacuo*. The crude residue was purified by using a Sephadex G-10 column and AG 50W-X8 cation exchange column (Na^+ form) by using water as an eluent to afford the desired product **4** (21 mg, 70%) as a white solid. ^{1}H NMR (600 MHz, D_2O) δ = 4.76 (s, 1H, H1'''''''), 4.74 (s, 3H, H1', H1''', H1'''''), 4.63 (d, *J* = 6.1 Hz, 3H, H1'', H1'''', H1''''''), 4.60-4.58 (dt, *J* = 9.7, 2.7 Hz, 3H, H3', H3''', H3'''''),

4.55 (d, J = 8.0 Hz, 1H, H1), 4.45 (dd, J = 3.3, 9.7 Hz, 1H, H3'''''''), 4.24-4.23 (m, 4H, H2', H2''', H2''''', H2'''''''), 4.20-4.13 (m, 4H, H5', H5''', H5''''', H5'''''''), 4.09-4.05 (m, 1H, ^{i}Pr-CH), 3.77-3.73 (m, 7H, H4, H4', H4'', H4''', H4'''', H4''''', H4''''''), 3.66-3.62 (m, 3H, H3'', H3'''', H3''''''), 3.60-3.53 (m, 6H, H3, H4''''''', H5, H5'', H5'''', H5''''''), 3.37-3.33 (m, 3H, H2'', H2'''', H2''''''), 3.26 (t, J = 8.6 Hz, 1H, H2), 1.31-1.30 (m, 9H, H6', H6''', H6'''''), 1.27 (d, J = 6.2 Hz, 3H, H6'''''''), 1.22 (d, J = 6.2 Hz, 3H, ^{i}Pr-CH_3), 1.18 (d, J = 6.2 Hz, 3H, ^{i}Pr-CH_3); ^{13}C NMR (150 MHz, D_2O) δ = 175.5 (C), 175.4 (C), 175.3 (C), 103.2 (C), 100.1 (CH), 100.0 (CH), 99.84 (CH), 99.8 (CH), 78.9 (CH), 78.8 (CH), 78.7 (CH), 78.4 (CH), 78.1 (CH), 78.08 (CH), 78.0 (CH), 76.2 (CH), 76.1 (CH), 76.07 (CH), 76.0 (CH), 74.1 (CH), 73.8 (CH), 73.7 (CH), 73.3 (CH), 73.0 (CH), 69.8 (CH), 68.9 (CH), 68.8 (CH), 68.7 (CH), 68.1 (CH), 68.04 (CH), 68.0 (CH), 22.2 (CH_3), 20.9 (CH_3), 16.8 (CH_3), 16.5 (CH_3); HRMS m/z $(M+H+4Na)^{2+}$ calcd for $C_{51}H_{74}O_{53}S_4H_4Na_4^{2+}$ 877.0790 found 877.0798.

3. Synthetic Procedures and Characterization data for B_3's.

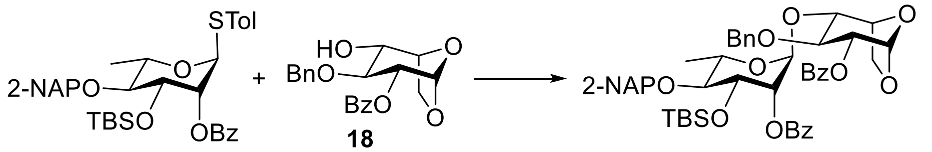

16 18 55

Compound 55. To a solution of donor **16** (30.3 g, 48.2 mmol) and acceptor **18** (18.0 g, 50.6 mmol) in anhydrous CH_2Cl_2 (250 mL), freshly activated AW-500 molecular sieves (6.0 g) were added under nitrogen atmosphere, and the reaction mixture was allowed to stir at room temperature for 0.5 h. Then, the reaction flask was cooled to -40 °C, NIS (12.9 g, 57.8 mmol) and TfOH (428 μL, 4.82 mmol) were added. Further, the reaction was allowed to stir at same temperature for 1 h, and Et_3N was added to quench the reaction. The whole reaction mixture was filtered through a pad of Celite®, and solids were washed with CH_2Cl_2. The filtrate was washed with 10% $Na_2S_2O_3$ and sat. $NaHCO_{3(aq)}$. The organic layer was dried over anhydrous $MgSO_4$, filtered, and concentrated *in vacuo*. The resulting crude residue was purified by silica gel column chromatography to give the desired product **55** (33.8 g, 81%) as a white solid. $[\alpha]_D^{20}$ = +38.9 (c = 0.5, $CHCl_3$); IR (thin film in KBr) ν 2951, 2929, 2898, 2855, 1723, 1268, 1096, 1034, 710 cm^{-1}; ^{1}H NMR (600 MHz, $CDCl_3$) δ = 8.01-7.04 (m, 20 H, Ar-H), 5.56 (d, J = 1.3 Hz, 1 H, H1), 5.25 (dd, J = 1.9 Hz, J = 3.1 Hz, 1 H, H2'), 5.11-5.08 (m, 2 H, H2, CH_2Ph), 5.00 (d, J = 1.0 Hz, 1 H, H1'), 4.83-4.79 (m, 2 H, CH_2Ph), 4.72 (ABq, J = 11 Hz, CH_2Ph), 4.66 (t, J

= 4.4 Hz, 1 H, H5), 4.33 (dd, J = 3.3 Hz, J = 9.1 Hz, 1 H, H3'), 4.22 (d, J = 7.7 Hz, H6a), 4.15 (dd, J = 3.9 Hz, J = 8.3 Hz, 1 H, H3), 4.08-4.05 (m, 1 H, H5'), 4.01 (t, J = 8.3 Hz, 1 H, H4), 3.85 (dd, J = 5.6 Hz, J = 6.8 Hz, 1 H, H6b), 3.57 (t, J = 9.3 Hz, 1 H, H4'), 1.20 (d, J = 6.2 Hz, 3 H, H6'), 0.90 (s, 9 H, ^{t}Bu), 0.15 (s, 3 H, CH_3), 0.11 (s, 3 H, CH_3) ; ^{13}C NMR (150 MHz, $CDCl_3$) δ = 166.2 (C), 165.8 (C), 137.4 (C), 136.0 (C), 133.5 (C), 133.4 (CH), 133.3 (CH), 133.0 (CH), 130.0 (CH), 129.9 (CH), 129.5 (CH), 128.6 (CH), 128.6 (CH), 128.5 (CH), 128.3 (CH), 128.1 (CH), 128.0 (CH), 127.9 (CH), 127.9 (CH), 126.4 (CH), 126.2 (CH), 126.0 (CH), 125.9 (CH), 99.4 (CH), 95.1 (CH), 81.3 (CH), 78.4 (CH), 77.0 (CH), 75.7 (CH_2), 75.5 (CH), 74.2 (CH), 73.7 (CH), 72.1 (CH), 71.3 (CH), 68.7 (CH), 65.5 (CH_2), 25.8 (CH_3), 18.1 (CH_3), 18.0 (CH_3); HRMS m/z $(M+Na)^+$ calcd for $C_{50}H_{56}NaO_{11}Si^+$ 833.3484, found 833.3484.

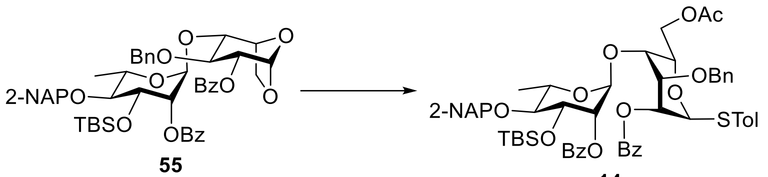

55 **14**

Compound 14. To a solution of compound **55** (31.5 g, 36.0 mmol) in Ac_2O/CH_2Cl_2 (620 mL, 1/1), $Cu(OTf)_2$ (1.3 g, 3.60 mmol) was added at 0 °C under nitrogen atmosphere. After stirring for 24 h, sat. $NaHCO_{3(aq)}$ was added to quench the reaction, and the mixture was extracted with EtOAc (3 x 250 mL). The combined organic layers were washed with brine, dried over anhydrous $MgSO_4$, filtered, and concentrated *in vacuo*. The residue was purified by flash column chromatography on silica gel (EtOAc/Hex = 1/4) to afford the desired 1,6-diacetate **56**. The acetylated compound **56** was dissolved in CH_2Cl_2 (120 mL), TMSSTol (30.8 mL, 157 mmol) and ZnI_2 (20.1 g, 62.9 mmol) were added at 0 °C. After stirring the reaction at room temperature for 24 h, sat. $NaHCO_{3(aq)}$ was added to quench the reaction, and the mixture was extracted with EtOAc (3 x 250 mL). The combined organic layers were washed with brine, dried over anhydrous $MgSO_4$, filtered, and concentrated *in vacuo*. The resulting crude residue was purified by silica gel column chromatography to afford the desired product **14** (31.1 g, 77%, over 2 steps) as a colorless syrup. $[\alpha]_D^{20}$ = -111.7 (*c* = 0.5, $CHCl_3$); IR (thin film in KBr) ν 2951, 2929, 2857, 1748, 1721, 1267, 1105, 1096, 1070, 1045, 710 cm^{-1}; ^{1}H NMR (600 MHz, $CDCl_3$) δ = 8.10-8.09 (m, 4 H, Ar-H), 7.92-7.84 (m, 3 H, Ar-H), 7.67-7.62 (m, 2 H, Ar-H), 7.56-7.48 (m, 8 H, Ar-H), 7.40-7.16 (m, 9 H, Ar-H), 5.57 (s, 1 H, H1), 5.48 (s, 1 H, H2), 5.24 (s, 1 H, H2'), 5.03 (t, *J* =5.6 Hz, 1 H, H5), 4.98-4.92 (m, 3 H, H1', 2CH_2Ph), 4.78 (ABq, *J* = 12.0 Hz, CH_2Ph), 4.69 (ABq, *J* = 11.0 Hz, CH_2Ph), 4.54 (dd, *J* =7.8 Hz, *J* = 11.0 Hz, 1 H, $H6_a$), 4.38

(dd, J =5.9 Hz, J = 11.1 Hz, 1 H, $H6_b$), 4.14 (dd, J =2.6 Hz, J = 8.3 Hz, 1 H, H3'), 4.06 (s, 1 H, H3), 3.85 (s, 1 H, H4), 3.76-3.73 (m, 1 H, H5'), 3.45 (t, J = 8.8 Hz, 1 H, H4'), 2.37 (s, 3 H, $PhCH_3$), 2.16 (s, 3 H, Ac), 1.00 (d, J = 6.1 Hz, 1 H, H6'), 0.86 (s, 9 H, ^{t}Bu), 0.11-0.05 (2s, 6 H, CH_3); ^{13}C NMR (150 MHz, $CDCl_3$) δ = 170.6 (C), 165.9 (C), 165.9 (C), 137.9 (C), 137.3 (C), 135.9 (CH), 133.4 (CH), 133.2 (CH), 133.0 (CH), 132.7 (CH), 131.8 (CH), 130.0 (CH), 129.8 (CH), 129.8 (CH), 129.3 (CH), 128.6 (CH), 128.6 (CH), 128.5 (CH), 128.0 (CH), 127.92 (CH), 127.91 (CH), 127.9 (CH), 127.8 (CH), 126.5 (CH), 126.2 (CH), 126.1 (CH), 125.9 (CH), 101.1 (CH), 86.4 (CH), 81.2 (CH), 76.2 (CH), 75.0 (CH_2), 73.7 (CH), 73.5 (CH), 72.7 (CH_2), 71.0 (CH), 69.6 (CH), 68.6 (CH), 65.7 (CH), 62.5 (CH_2), 25.7 (CH_3), 21.2 (CH_3), 20.9 (CH_3), 17.8 (CH_3), 17.7 (CH_3); HRMS m/z $(M+Na)^+$ calcd for $C_{59}H_{66}NaO_{12}SSi^+$ 1049.3936, found 1049.3950.

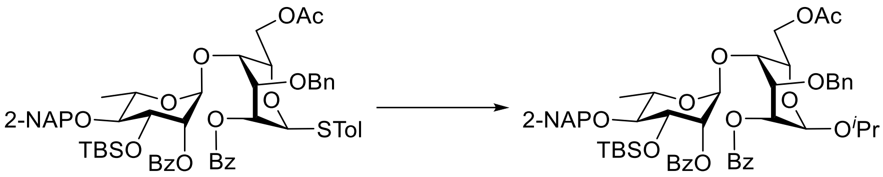

14 **57**

Compound 57. To a solution of donor **14** (14.5 g, 14.1 mmol), and ^{i}PrOH (3.5 mL, 42.3 mmol) in anhydrous CH_2Cl_2 (145.0 mL), freshly activated AW-500 molecular sieves (3.0 g) were added under nitrogen atmosphere, and reaction mixture was allowed to stir at room temperature for 0.5 h. Then, the reaction flask was cooled to 0 °C, NIS (3.5 g, 15.5 mmol) and TfOH (125 µL, 1.41 mmol) were added. The reaction mixture was allowed to stir at same temperature for 1 h. Et_3N was added to quench the reaction, the whole mixture was filtered through a pad of Celite®, and solids were washed with CH_2Cl_2. The filtrate was washed with 10% $Na_2S_2O_3$ and sat. $NaHCO_{3(aq)}$, then, the organic layer was dried over anhydrous $MgSO_4$, filtered, and concentrated *in vacuo*. The obtained crude residue was purified by silica gel column chromatography to afford the desired product **57** (9.95 g, 73%) as a white solid. $[\alpha]_D^{20}$ = -39.6 (c = 1.0, $CHCl_3$); IR (thin film in KBr) ν 2956, 2930, 2855, 1747, 1722, 1267, 1107, 1070, 1034, 711 cm^{-1}; ^{1}H NMR (600 MHz, $CDCl_3$) δ = 8.10-8.08 (m, 4 H, Ar-H), 7.91-7.84 (m, 3 H, Ar-H), 7.69-7.61 (m, 2 H, Ar-H), 7.55-7.49 (m, 4 H, Ar-H), 7.40-7.24 (m, 9 H, Ar-H), 5.25 (t, J =4.9 Hz, 1 H, H2'), 5.21 (t, J =2.3 Hz, 1 H, H2), 5.09 (d, J = 1.5 Hz, H1), 4.90 (d, 1 H, J = 11.7 Hz, CH_2Ph), 4.93 (s, 1 H, H1'), 4.86 (d, 1 H, J = 11.3 Hz, CH_2Ph), 4.75-4.71 (m, 2 H, 2 CH_2Ph), 4.55-4.51 (m, 2 H, H5, H6a), 4.30 (q, J =9.7 Hz, J = 5.2 Hz, 1 H, $H6_b$), 4.24 (dd, J =3.2 Hz, J = 8.7 Hz, 1 H, H3'), 4.01-3.97 (m, 2 H, H3, CH(CH_3)$_2$), 3.87 (s, 1 H, H4), 3.85-3.82

(m, 1 H, H5'), 3.47 (t, J = 9.1 Hz, 1 H, H4'), 2.14 (s, 3 H, Ac), 1.30 (d, J = 6.2 Hz, 3 H, ^{i}Pr), 1.21 (d, J = 6.1 Hz, 3 H, ^{i}Pr), 1.02 (d, J = 6.2 Hz, 3 H, H-6'), 0.86 (s, 9 H, ^{t}Bu), 0.14 (s, 3 H, CH_3), 0.09 (s, 3 H, CH_3); ^{13}C NMR (150 MHz, $CDCl_3$) δ = 170.7 (C), 165.9 (C), 165.9 (C), 137.9 (C), 136.0 (C), 133.3 (C), 133.3 (C), 133.0 (CH), 130.1 (CH), 129.8 (CH), 129.8 (CH), 129.6 (CH), 128.6 (CH), 128.5 (CH), 128.4 (CH), 128.0 (CH), 127.9 (CH), 127.8 (CH), 127.8 (CH), 127.7 (CH), 126.6 (CH), 126.2 (CH), 126.1 (CH), 125.9 (CH), 100.3 (CH), 96.5 (CH), 81.3 (CH), 76.0 (CH), 75.2 (CH_2), 75.1 (CH), 73.6 (CH), 72.5 (CH_2), 71.1 (CH), 70.4 (CH), 69.6 (CH), 68.6 (CH), 65.4 (CH), 62.3 (CH_2), 25.8 (CH_3), 23.3 (CH_3), 21.6 (CH_3), 20.9 (CH_3), 17.9 (CH_3), 17.8 (CH_3); HRMS m/z $(M+Na)^+$ calcd for $C_{55}H_{66}NaO_{13}Si^+$ 985.4165, found 985.4192.

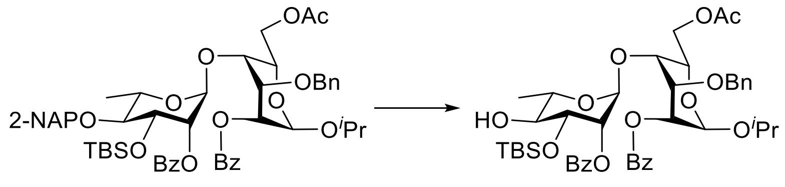

Compound 58. To a solution of compound **57** (1.6 g, 1.70 mmol) in a mixed solvent CH_2Cl_2/H_2O (15.7 mL, 1:9), DDQ (1.2 g, 5.10 mmol) was added at 0 °C, and the reaction mixture was allowed to stir at same temperature for 16 h. TLC analysis indicates the complete conversion of starting materials into products, the whole reaction mixture was filtered through a pad of Celite® and solids were washed with CH_2Cl_2, the filtrate was washed with sat. $NaHCO_{3(aq)}$ and brine. Organic layer was dried over anhydrous $MgSO_4$, filtered, and concentrated *in vacuo*. The resulting crude residue was purified by silica gel column chromatography to afford the desired product **58** (1.07 g, 78%) as a colorless syrup. $[\alpha]_D^{20}$ = -14.4° (c = 1.3, $CHCl_3$); IR (thin film in KBr) ν 3546, 2930, 2855, 1720, 1267, 1112, 1070, 1030, 712 cm^{-1}; ^{1}H NMR (600 MHz, $CDCl_3$) δ = 8.12-8.11 (m, 2 H, Ar-H), 8.04-8.02 (m, 2 H, Ar-H), 7.62-7.58 (m, 2 H, Ar-H), 7.50-7.44 (m, 4 H, Ar-H), 7.40-7.38 (m, 2 H, Ar-H), 7.32-7.25 (m, 3 H, Ar-H), 5.19-5.17 (m, 2 H, H2', H2), 5.10 (d, J = 1.5 Hz,1 H, H1), 4.94-4.89 (m, 2 H, H1', CH_2Ph), 4.73 (d, J = 11.7 Hz, 1 H, CH_2Ph), 4.55-4.50 (m, 2 H, H5, H6a), 4.29 (q, J = 7.6 Hz, J = 2.5 Hz,1 H, H6b), 4.01-3.96 (m, 2 H, H-3, $CH(CH_3)_2$), 3.92 (dd, J = 3.4, J = 9.1, 1 H, H3'), 3.85 (s, 1 H, H4), 3.69-3.66 (m, 1 H, H5'), 3.55 (td, J = 3.0, J = 9.2, 1 H, H4'), 2.13 (s, 3 H, Ac), 1.58 (d, J = 3.1, 1 H, 4OH), 1.30 (d, J = 6.2 Hz, 3 H, ^{i}Pr), 1.21 (d, J = 5.9 Hz, 3 H, ^{i}Pr), 1.02 (d, J = 6.2 Hz, 3 H, H6'), 0.82 (s, 9 H, ^{t}Bu), 0.10 – 0.09 (2s, 6 H, $2CH_3$); ^{13}C NMR (150

MHz, $CDCl_3$) δ = 170.7 (C), 165.9 (C), 165.7 (C), 137.9 (C), 133.5 (CH), 133.3 (CH), 129.9 (CH), 129.8 (CH), 129.8 (CH), 128.7 (CH), 128.5 (CH), 128.4 (CH), 127.8 (CH), 127.8 (CH), 100.4 (CH), 96.5 (CH), 76.0 (CH), 74.8 (CH), 73.3 (CH), 72.7 (CH), 72.5 (CH_2), 71.3 (CH), 70.4 (CH), 69.6 (CH), 68.8 (CH), 65.3 (CH), 62.3 (CH_2), 25.7 (CH_3), 23.3 (CH_3), 21.6 (CH_3), 20.9 (CH_3), 18.0 (CH_3), 17.3 (CH_3); HRMS m/z $(M+Na)^+$ calcd for $C_{44}H_{58}NaO_{13}Si^+$ 845.3539, found 845.3558.

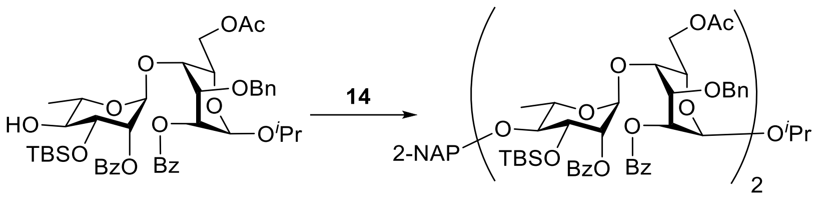

58 **59**

Compound 59. To a solution of donor **14** (1.0 g, 1.21 mmol), and acceptor **58** (1.4 g, 1.33 mmol) in anhydrous CH_2Cl_2 (12.5 mL), freshly activated AW-500 molecular sieves (0.6 g) was added under nitrogen atmosphere, and the reaction mixture was allowed to stir at room temperature for 0.5 h. Then, the reaction flask was cooled to -40 °C, NIS (326 mg, 1.45 mmol) and TfOH (10.75 μL, 0.12 mmol) were added to the reaction mixture, Further, the reaction mixture was allowed to stir at the same temperature for 1 h. Et_3N was added to quench the reaction, the whole mixture was filtered through a pad of Celite®, and solids were washed with CH_2Cl_2. The filtrate was washed with 0.1 N HCl, sat. $NaHCO_{3(aq)}$, 10% $Na_2S_2O_3$, and brine solution. The organic layer was dried over anhydrous $MgSO_4$, filtered, and concentrated *in vacuo*. The resulting crude residue was purified by flash column chromatography to afford the desired product **59** (1.71 g, 81%) as a white solid. $[\alpha]_D^{20}$ = -35.0 (*c* = 0.6, $CHCl_3$); IR (thin film in KBr) ν 2929, 2855, 1744, 1720, 1267, 1107, 1070, 1029, 712 cm^{-1}; ^1H NMR (600 MHz, $CDCl_3$) δ = 8.01-8.00 (m, 8 H, Ar-H), 7.90-7.82 (m, 3 H, Ar-H), 7.62-7.58 (m, 4 H, Ar-H), 7.55-7.46 (m, 7 H, Ar-H), 7.44-7.41 (m, 2 H, Ar-H), 7.38-7.21 (m, 13 H, Ar-H), 5.57 (s, 1 H, H1”), 5.23 (s, 1 H, H2), 5.20 (s, 1 H, H2’), 5.15 (s, 1 H, H2’”), 5.11 (s, 1 H, H2”), 5.07 (s, 1 H, H1), 4.92 (s, 1 H, H1’), 4.89 (d, *J* = 11.8 Hz, 1 H, Ar-CH_2), 4.83-4.80 (m, 2 H, Ar-CH_2), 4.76-4.72 (m, 2 H, Ar-CH_2, H1’”), 4.59 (d, *J* = 11.7 Hz, 1 H, Ar-CH_2), 4.58-4.52 (m, 3 H, Ar-CH_2, H5, H6), 4.42 (dd, *J* =7.0 Hz, *J* = 10.6 Hz, 1 H, H6”), 4.34 (t, *J* = 5.8 Hz, 1 H, H5”), 4.35-4.24 (m, 3 H, H3’, H6, H6”), 4.01-3.90 (m, 4 H, ^{i}Pr, H3, H3”, H3’”), 3.90-3.89 (m, 2 H, H4, H4’), 3.84-3.82 (m, 1 H, H5’), 3.68 (s, 1 H, H4”), 3.50-3.47 (m, 1 H, H4’”), 3.31 (t, *J* = 8.3 Hz, 1 H, H5’”), 2.12 (s, 3 H, Ac), 2.07 (s, 3 H, Ac), 1.20 (d, *J* = 6.1 Hz, 3 H, ^{i}Pr), 1.30 (d, *J* =

6.1 Hz, 3 H, ^{i}Pr), 1.02 (d, J = 6.1 Hz, 3 H, H6'), 0.83 (d, J = 6.2 Hz, 3 H, H6'''), 0.81 (s, 9 H, ^{t}Bu), 0.62 (s, 9 H, ^{t}Bu), 0.10 – (-0.07) (4s, 12 H, 4CH3); ^{13}C NMR (150 MHz, $CDCl_3$) δ = 170.6 (C), 170.5 (C), 165.9 (C), 165.8 (C), 165.7 (C), 165.6 (C), 137.8 (C), 137.6 (C), 135.9 (C), 133.4 (CH), 133.3 (CH), 133.2 (CH), 132.9 (CH), 130.0 (CH), 129.8 (CH), 129.7 (CH), 129.7 (CH), 129.5 (CH), 129.3 (CH), 128.6 (CH), 128.5 (CH), 128.4 (CH), 128.4 (CH), 128.4 (CH), 128.3 (CH), 127.9 (CH), 127.8 (CH), 127.8 (CH), 127.8 (CH), 127.7 (CH), 127.7 (CH), 126.4 (CH), 126.1 (CH), 126.1 (CH), 125.8 (CH), 101.0 (CH), 99.8 (CH), 98.6 (CH), 96.5 (CH), 81.1 (CH), 76.0 (CH), 75.8 (CH), 74.9 (CH_2), 74.8 (CH), 73.5 (CH), 73.5 (CH), 73.1 (CH), 72.4 (CH_2), 72.1 (CH_2), 71.8 (CH), 70.8 (CH), 70.4 (CH), 69.5 (CH), 68.5 (CH), 68.2 (CH), 67.7 (CH), 65.3 (CH), 64.4 (CH), 63.0 (CH_2), 62.2 (CH_2), 25.7 (CH_3), 25.5 (CH_3), 23.3 (CH_3), 21.5 (CH_3), 20.8 (CH_3), 18.2 (CH_3), 17.7 (CH_3), 17.5 (CH_3), 17.5 (CH_3); HRMS m/z $(M+Na)^+$ calcd for $C_{96}H_{116}NaO_{25}Si_2^+$ 1748.7267, found 1748.7252.

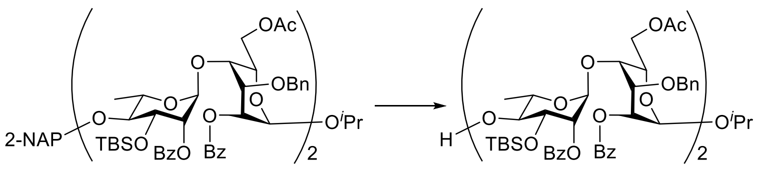

59 **60**

Compound 60. To a solution of compound **59** (8.9 g, 5.15 mmol) in CH_2Cl_2/H_2O (90 mL, 1/9), DDQ (3.50 g, 15.46 mmol) was added at 0 °C. Then, the reaction mixture was allowed to stir at the same temperature for 16 h. TLC analysis indicates the complete conversion of starting materials into products. The whole reaction mixture was filtered through a pad of Celite® and solids were washed with CH_2Cl_2, then the filtrate was washed with sat. $NaHCO_{3(aq)}$ and brine. Organic layer was dried over anhydrous $MgSO_4$, filtered, and concentrated *in vacuo*. The resulting crude residue was purified by silica gel column chromatography to afford the desired product **60** (7.60 g, 94%) as a colorless syrup. $[\alpha]_D^{27}$ = -31.0 (c = 1.0, $CHCl_3$); IR (thin film in KBr) ν 3558, 3064, 3032, 2954, 2930, 2857, 1747, 1720, 1602, 1584, 1493, 1452, 1369, 1316, 1302, 1177, 1146, 1110, 1070, 1028, 980, 938, 858, 838, 778, 754, 687, 618 cm^{-1}; ^{1}H NMR (600 MHz, $CDCl_3$): δ = 8.06-8.05 (m, 2 H, Ar-H), 8.02-8.01 (m, 2 H, Ar-H), 7.97-7.95 (m, 4 H, Ar-H), 7.58-7.52 (m, 4 H, Ar-H), 7.47-7.36 (m, 8 H, Ar-H), 7.33-7.27 (m, 6 H, Ar-H), 7.24-7.16 (m, 4 H, Ar-H), 5.54 (s, 1 H, H1''), 5.18 (s, 1 H, H2), 5.15 (s, 1 H, H2'), 5.05 (m, 2 H, H2'', H2'''), 5.02 (s, 1 H, H1), 4.86 (s, 1 H, H1'), 4.83 (d, J = 11.5 Hz, 1 H, Ar-CH_2), 4.74 (s, 1 H, H1'''), 4.71-4.67 (m, 2 H, Ar-CH_2), 4.58 (d, J = 10.6 Hz, 1 H, Ar-CH_2), 4.48 (m, 2 H, H5, H6), 4.37 (m, 1 H, H6''), 4.30 (m, 1 H, H5''), 4.24-4.22 (m, 3 H, H3', H6, H6''), 3.94-3.92 (m,

2 H, ^{i}Pr, H3), 3.88-3.83 (m, 3 H, H3", H4, H4'), 3.80-3.78 (m, 1 H, H5'), 3.64-3.62 (m, 2 H, H3"', H4"), 3.35 (m, 1 H, H4"'), 3.28 (m, 1 H, H5"'), 2.07 (s, 3 H, Ac), 2.02 (s, 3 H, Ac), 1.24 (d, 3 H, J = 5.6 Hz, ^{i}Pr), 1.15 (d, 3 H, J = 5.6 Hz, ^{i}Pr), 0.97 (d, 3 H, J = 5.6 Hz, H6'), 0.82 (d, 3 H, J = 5.0 Hz, H6"'), 0.72 (s, 9 H, ^{t}Bu), 0.59 (s, 9 H, ^{t}Bu), 0.02- (-0.05) (m, 12 H, CH_3); ^{13}C NMR (150 MHz, $CDCl_3$): δ = 170.6 (C), 170.5 (C), 165.9 (C), 165.8 (C), 165.6 (C), 165.5(C), 137.7 (C), 137.5 (C), 133.5 (CH), 133.4 (CH), 133.3 (CH), 133.2 (CH), 129.8 (CH), 129.7 (CH), 129.6 (CH), 129.57 (CH), 129.5 (CH), 128.7 (CH), 128.6 (CH), 128.4 (CH), 128.37 (CH), 128.3 (CH), 127.8 (CH), 127.77 (CH), 127.71 (CH), 101.2 (CH), 99.7 (CH), 98.6 (CH), 96.5 (CH), 75.9 (CH), 75.8 (CH), 74.9 (CH), 73.4 (CH), 73.0 (CH), 72.7 (CH), 72.5 (CH), 72.3 (CH_2), 72.0 (CH_2), 71.8 (CH), 71.0 (CH), 70.3 (CH), 69.5 (CH), 68.7 (CH), 68.2 (CH), 67.7 (CH), 65.3 (CH), 133.5 (CH), 64.4 (CH), 62.8 (CH_2), 62.2 (CH_2), 25.5 (CH_3), 25.4 (CH_3), 23.2 (CH_3), 21.5 (CH_3), 20.8 (CH_3), 18.2 (CH_3), 17.8 (CH_3), 17.5 (CH_3), 16.8 (CH_3), 25.5 (CH_3), -4.2 (CH_3), -4.6 (CH_3), -4.7 (CH_3); HRMS m/z $(M+Na)^+$ calcd for $C_{85}H_{108}O_{25}Si_2Na^+$ 1607.6610 found 1607.6637.

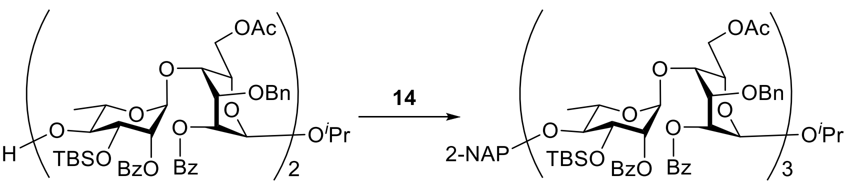

60 **61**

Compound 61. To a solution of donor **14** (770 mg, 688 μmol) and acceptor **60** (1.00 g, 626 μmol) in anhydrous CH_2Cl_2 (10.0 mL), freshly activated AW-500 molecular sieves (0.6 g) was added under nitrogen atmosphere, and the reaction mixture was allowed to stir at room temperature for 0.5 h. Then, the reaction flask was cooled to -40 °C, NIS (169 mg, 751 μmol) and TfOH (5.57 μL, 62.6 μmol) were added, and the reaction mixture was allowed to stir at same temperature for 1 h. Et_3N was added to quench the reaction, whole mixture was filtered through a pad of Celite®, and the solids were washed with CH_2Cl_2. The filtrate was washed with 0.1 N HCl, sat. $NaHCO_{3(aq)}$, 10% $Na_2S_2O_3$, and brine solution. The organic layer was dried over anhydrous $MgSO_4$, filtered, and concentrated *in vacuo*. The resulting crude residue was purified by flash column chromatography to afford the desired product **61** (1.43 g, 92%) as a white solid. $[\alpha]_D^{28}$ = -46.0 (c = 1.0, $CHCl_3$); IR (thin film in KBr) ν 2930, 2857, 1748, 1720, 1601, 1452, 1368, 1316, 1107, 1070, 1028, 979, 858, 838, 777, 751, 711; ^{1}H NMR ($CDCl_3$, 600 MHz): δ = 8.03-8.02 (m, 12 H, Ar-H), 7.84-7.83 (m, 3 H, Ar-H), 7.57-7.55 (m, 6 H, Ar-H),

7.50-7.27 (m, 29 H, Ar-H), 7.24-7.18 (m, 12 H, Ar-H), 5.55 (s, 1 H, H1”), 5.38 (s, 1 H, H1””), 5.18 (s, 1 H, H2), 5.14 (s, 1 H, H2’), 5.08-5.06 (m, 3 H, H2”, H2’”, H2”””), 5.01 (s, 1 H, H1), 4.94 (s, 1 H, H2””), 4.87 (s, 1 H, H1’), 4.84-4.64 (m, 9 H, Ar-CH_2, H1’”, H1”””), 4.58 (ABq, 1 H, J = 11.1 Hz, Ar-CH_2), 4.52-4.48 (m, 3 H, Ar-CH_2, H5, H6), 4.43 (m, 1 H, H6), 4.31-4.28 (m, 2 H, H5”, H6””), 4.23-4.22 (m, 4 H, H3’, H6”, H6””), 4.16 (m, 1 H, H5””), 4.05 (d, 1 H, J = 7.8 Hz, H-3’”), 3.94-3.91 (m, 6 H, H3, H3”, H3””, H3”””, H4’, ^{i}Pr), 3.83 (s, 1 H, H4), 3.79 (m, 1 H, H5’), 3.70 (m, 1 H, H4’”), 3.65-3.62 (m, 2 H, H4”, H4””), 3.51 (s, 1 H, H5’”), 3.43 (s, 1 H, H5”””), 3.26 (m, 1 H, H4”””), 2.07 (s, 3 H, Ac), 2.02-2.00 (m, 6 H, Ac), 1.24 (d, 3 H, J = 6.8 Hz, ^{i}Pr), 1.15 (d, 3 H, J = 5.4 Hz, ^{i}Pr), 0.99 (d, 3 H, J = 5.4 Hz, Rha H6), 0.81 (d, 3 H, J = 6.1 Hz, H6”””), 0.76 (s, 12 H, ^{t}Bu, Rha H6), 0.53 (s, 18 H, ^{t}Bu), 0.03-(-0.14) (m, 18 H, CH_3); ^{13}C NMR ($CDCl_3$, 150 MHz): δ = 170.6 (C), 170.4 (C), 170.3 (C), 165.8 (C), 165.64 (C), 165.6 (C), 165.4 (C), 137.7 (C), 137.6 (C), 137.5 (C), 135.8 (C), 133.4 (CH), 133.2 (CH), 133.1 (CH), 132.8 (CH), 129.9 (CH), 129.8 (CH), 129.77 (CH), 129.74 (CH), 129.7 (CH), 129.6 (CH), 129.5 (CH), 129.2 (CH), 128.5 (CH), 128.4 (CH), 128.38 (CH), 128.3 (CH), 128.2 (CH), 127.9 (CH), 127.86 (CH), 127.8 (CH), 127.79 (CH), 127.73 (CH), 127.7 (CH), 126.3 (CH), 126.1 (CH), 125.7 (CH), 100.9 (CH), 100.4 (CH), 99.7 (CH), 98.6 (CH), 98.5 (CH), 96.5 (CH), 81.0 (CH), 75.8 (CH), 74.9 (CH), 74.7 (CH), 73.5 (CH), 73.4 (CH), 73.2 (CH), 73.0 (CH), 72.3 (CH_2), 72.2 (CH_2), 72.1 (CH_2), 71.3 (CH), 70.7 (CH), 70.3 (CH), 69.4 (CH), 68.4 (CH), 68.2 (CH), 67.8 (CH), 67.7 (CH), 65.2 (CH), 64.3 (CH), 62.8 (CH_2), 62.2 (CH_2), 60.3 (CH_2), 25.6 (CH_3), 25.5 (CH_3), 25.4 (CH_3), 25.3 (CH_3), 23.2 (CH_3), 21.4 (CH_3), 21.0 (CH_3), 20.8 (CH_3), 18.2 (CH_3), 17.9 (CH_3), 17.7 (CH_3), 17.4 (CH_3), 14.1 (CH_3), -4.3 (CH_3), -4.7 (CH_3), -4.9 (CH_3), -5.0 (CH_3); HRMS m/z $(M+Na)^+$ calcd for $C_{137}H_{166}O_{37}Si_3$ Na^+ 2512.0362 found 2512.0316.

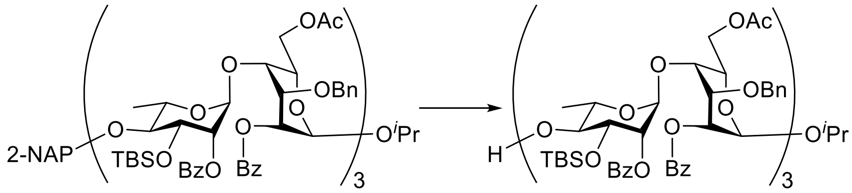

61 **62**

Compound 62. To a solution of compound **61** (319 mg, 128 μmol) in a mixed solvent of CH_2Cl_2/H_2O (3.3 mL, 1/9), DDQ (87 mg, 384 μmol) was added at 0 °C. Then, the reaction mixture was allowed to stir at same temperature for 16 h, and TLC analysis indicated the complete conversion of starting materials into products. The whole reaction mixture was

filtered through a pad of Celite®, solids were washed with CH_2Cl_2, and filtrate was washed with sat. $NaHCO_{3(aq)}$ and brine. Organic layer was dried over anhydrous $MgSO_4$, filtered, and concentrated *in vacuo*. The resulting crude residue was purified by silica gel column chromatography to afford the desired hexasaccharide acceptor **62** (272 mg, 90%) as a colorless syrup. $[\alpha]_D^{28}$ = -20.8 (*c* = 2.6, $CHCl_3$); IR (thin film in KBr) ν 3557, 2930, 2857, 1747, 1720, 1601, 1584, 1493, 1452, 1369, 1316, 1176, 1146, 1109, 1070, 1027, 979, 858, 838, 778, 752, 711; ^{1}H NMR (D_2O, 600 MHz): δ = 8.05-7.94 (m, 12 H, Ar-H), 7.57-7.48 (m, 6 H, Ar-H), 7.46-7.38 (m, 16 H, Ar-H), 7.33-7.26 (m, 9 H, Ar-H), 7.22-7.18 (m, 2 H, Ar-H), 5.55 (s, 1 H, H1”), 5.42 (s, 1 H, H1””), 5.18 (s, 1 H, H2), 5.14 (s, 1 H, H2’), 5.08 (s, 2 H, H2”, H2”’), 5.03-5.02 (m, 2 H, H1, H2”””), 4.94 (s, 1 H, H-2””), 4.88 (s, 1 H, H1’), 4.84-4.81 (m, 2 H, Ar-CH_2), 4.75-4.58 (m, 6 H, H1”’, H1”””, Ar-CH_2), 4.48-4.42 (m, 3 H, H5, H6, H6”), 4.32-4.29 (m, 2 H, H5”, H6””), 4.23-4.18 (m, 5 H, H3’, H5””, H6, H6”, H6””), 4.06 (d, 1 H, *J* = 8.5 Hz, H3”’), 3.95-3.89 (m, 5 H, H3””, H5’, H3”, H3, ^{i}Pr), 3.84-3.78 (m, 2 H, H4, H5’), 3.71 (t, 1 H, *J* = 8.8, H4”’), 3.65-3.62 (m, 3 H, H3”””, H4”, H4””), 3.52 (s, 1 H, H5”’), 3.36 (t, 1 H, J = 7.8 Hz, H4”””), 3.29 (m, 1 H, H5”””), 2.07 (s, 3 H, Ac), 2.02 (s, 3 H, Ac), 2.01 (s, 3 H, Ac), 1.25 (d, 3 H, J = 6.5 Hz, ^{i}Pr), 1.15 (d, 3 H, J = 6.5 Hz, ^{i}Pr), 1.00 (d, 3 H, J = 5.8 Hz, H6’), 0.84 (d, 3 H, J = 5.8 Hz, H6”””), 0.76 (d, 3 H, J = 5.8 Hz, H6”’), 0.71 (s, 9 H, ^{t}Bu), 0.56 (s, 9 H, ^{t}Bu), 0.53 (s, 9 H, ^{t}Bu), 0.00-(-0.12) (m, 18 H, CH_3); ^{13}C NMR ($CDCl_3$, 150 MHz): δ = 170.6 (C), 170.5 (C), 170.4 (C), 165.9 (C), 165.8 (C), 165.6 (C), 165.5 (C), 165.4 (C), 137.8 (C), 137.6 (C), 137.5 (C), 133.49 (CH), 133.4 (CH), 133.3 (CH), 133.2 (CH), 129.9 (CH), 129.8 (CH), 129.78 (CH), 129.74 (CH), 129.7 (CH), 129.6 (CH), 129.5 (CH), 129.3 (CH), 128.6 (CH), 128.5 (CH), 128.4 (CH), 128.38 (CH), 128.33 (CH), 128.0 (CH), 127.9 (CH), 127.8 (CH), 127.77 (CH), 127.7 (CH), 101.2 (CH), 100.4 (CH), 99.7 (CH), 98.7 (CH), 98.6 (CH), 96.5 (CH), 75.9 (CH), 75.8 (CH), 74.9 (CH), 73.5 (CH), 73.2 (CH), 73.0 (CH), 72.5 (CH), 72.3 (CH_2), 72.2 (CH_2), 72.1 (CH_2), 71.8 (CH), 71.4 (CH), 71.0 (CH), 70.3 (CH), 69.4 (CH), 68.7 (CH), 68.2 (CH), 67.9 (CH), 67.7 (CH), 65.3 (CH), 64.4 (CH), 62.9 (CH), 62.8 (CH), 62.2 (CH), 25.5 (CH_3), 25.45 (CH_3), 25.4 (CH_3), 23.2 (CH_3), 21.5 (CH_3), 20.8 (CH_3), 18.2 (CH_3), 18.0 (CH_3), 17.8 (CH_3), 17.5 (CH_3), 16.9 (CH_3), -4.30 (CH_3), -4.33 (CH_3), -4.6 (CH_3), -4.7 (CH_3), -4.92 (CH_3), -4.98 (CH_3).

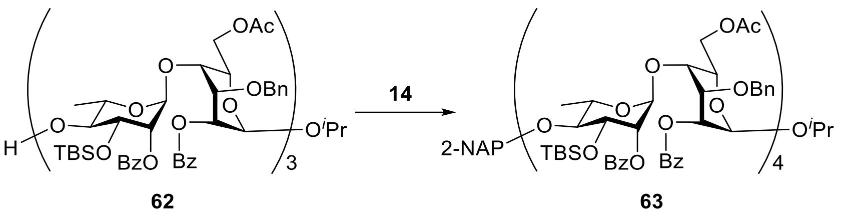

Compound 63. To a solution of donor **14** (403 mg, 393 µmol) and hexasaccharide acceptor **62** **(**838 mg, 357 µmol) in anhydrous CH_2Cl_2 (10.0 mL), freshly activated AW-500 molecular sieves (0.6 g) was added under nitrogen atmosphere, and the reaction mixture was allowed to stir at RT for 0.5 h. Then, the reaction flask was cooled to -40 °C, NIS (88.0 mg, 393 µmol) and TfOH (3.18 µL, 35.7 µmol) were added, and the reaction mixture was allowed to stir at same temperature for 1 h. Et_3N was added to quench the reaction, whole mixture was filtered through a pad of Celite®, solids were washed with CH_2Cl_2. The filtrate was washed with 0.1 N HCl, sat. $NaHCO_3$, 10% $Na_2S_2O_3$, and brine solution. The organic layer was dried over anhydrous $MgSO_4$, filtered, and concentrated *in vacuo*. The crude residue was purified by flash column chromatography to afford the desired product **63** (997 mg, 86%) as a white solid. $[\alpha]_D^{28}$ = -74.2 (*c* = 1.0, $CHCl_3$); IR (thin film in KBr) ν 3064, 2930, 2858, 1747, 1721, 1602, 1585, 1493, 1471, 1452, 1368, 1316, 1176, 1147, 1108, 1070, 1028, 979, 857, 838, 777, 753, 712, 617, 477; ^{1}H NMR ($CDCl_3$, 600 MHz): δ = 8.03-7.93 (m, 16 H, Ar-H), 7.84-7.77 (m, 3 H, Ar-H), 7.57-7.54 (m, 6 H, Ar-H), 7.51-7.47 (m, 4 H, Ar-H), 7.46-7.41 (m, 8 H, Ar-H), 7.40-7.26 (m, 22 H, Ar-H), 7.25-7.14 (m, 8 H, Ar-H), 5.54 (s, 1 H, IdoA H1), 5.41-5.38 (m, 2 H, IdoA H1x2), 5.19 (m, 1 H, H2), 5.14 (m, 1 H, Rha H2), 5.08-5.01 (m, 5 H, H1, Rha H2x3, IdoA H2), 4.96 (s, 1 H, IdoA H2), 4.93 (s, 1 H, IdoA H2), 4.88-4.80 (m, 4 H, Rha H1, Ar-CH_2), 4.79-4.58 (m, 9 H, Rha H1x3, Ar-CH_2), 4.52-4.46 (m, 3 H, H5, H6, Ar-CH_2), 4.44-4.18 (m, 11 H, Rha H3, H6, IdoA H5x3, IdoA H6x3), 4.05-4.01 (m, 2 H, Rha H3x2), 3.95-3.87 (m, 7 H, H3, ^{i}Pr, Rha H3, Rha H4, IdoA H3x3), 3.84 (s, 1 H, H4), 3.79 (m, 1 H, Rha H5), 3.74-3.70 (m, 2 H, Rha H4x2), 3.66-3.62 (m, 3 H, IdoA H4x3), 3.52-3.41 (m, 3H, Rha H5x3), 3.26 (t, 1 H, *J* = 9.2 Hz, Rha H4), 2.07 (s, 3 H, Ac), 2.02 (s, 3 H, Ac), 2.01 (m, 6 H, Ac), 1.25 (d, 3 H, J = 7.2, ^{i}Pr), 1.15 (d, 3 H, J = 5.7, ^{i}Pr), 0.99 (d, 3 H, J = 7.2, Rha H6), 0.80-0.78 (m, 9 H, Rha H6x3), 0.76 (s, 9 H, ^{t}Bu), 0.54 (s, 9 H, ^{t}Bu), 0.53 (s, 9 H, ^{t}Bu), 0.50 (s, 9 H, ^{t}Bu), 0.03-(-0.16) (m, 24, CH_3); ^{13}C NMR ($CDCl_3$, 150 MHz): δ = 170.6 (C), 170.48 (C), 170.4 (C), 165.88 (C), 165.86 (C), 165.8 (C), 165.66 (C), 165.6 (C), 165.5 (C), 165.4 (C), 137.8 (C), 137.7 (C), 137.6 (C), 137.5 (C), 135.9 (C), 133.48 (CH), 133.4 (CH), 133.3 (CH), 133.1 (CH), 132.8 (CH), 129.95 (CH), 129.9 (CH), 129.87 (CH), 129.8 (CH), 129.78 (CH), 129.7 (CH), 129.69 (CH), 129.6 (CH),

129.5 (CH), 129.3 (CH), 129.2 (CH), 128.6 (CH), 128.5 (CH), 128.48 (CH), 128.41 (CH), 128.4 (CH), 128.3 (CH), 128.0 (CH), 127.94 (CH), 127.9 (CH), 127.87 (CH), 127.8 (CH), 127.76 (CH), 127.73 (CH), 127.7 (CH), 126.4 (CH), 126.1 (CH), 126.0 (CH), 125.8 (CH), 101.0 (CH), 100.5 (CH), 100.4 (CH), 99.7 (CH), 98.7 (CH), 96.5 (CH), 81.0 (CH), 75.8 (CH), 74.9 (CH), 74.8 (CH_2), 73.5 (CH), 73.46 (CH), 73.4 (CH), 73.3 (CH), 73.2 (CH), 73.0 (CH), 72.4 (CH_2), 72.3 (CH_2), 72.1 (CH_2), 71.8 (CH), 71.5 (CH), 71.4 (CH), 70.8 (CH), 70.3 (CH), 69.5 (CH), 68.5 (CH), 68.34 (CH), 68.3 (CH), 68.2 (CH), 67.9 (CH), 67.7 (CH), 65.3 (CH), 64.4 (CH), 62.9 (CH_2), 62.8 (CH_2), 62.2 (CH_2), 60.4 (CH_2), 25.6 (CH_3), 25.4 (CH_3), 25.3 (CH_3), 23.2 (CH_3), 21.5 (CH_3), 21.0 (CH_3), 20.8 (CH_3), 18.2 (CH_3), 18.0 (CH_3), 17.7 (CH_3), 17.5 (CH_3), 17.47 (CH_3), 17.4 (CH_3), 14.2 (CH_3), -4.32 (CH_3), -4.35 (CH_3), -4.37 (CH_3), -4.6 (CH_3).

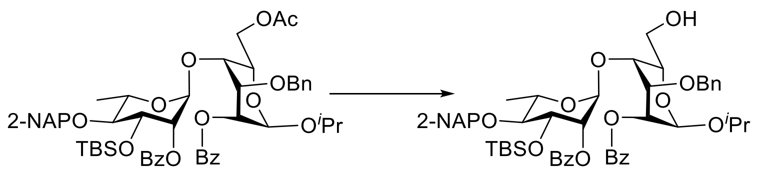

Compound 64. To a solution of compound **57** (1.1 g, 1.38 mmol) in CH_2Cl_2, $Mg(OMe)_2$ (3.52 mL, 7-8% in MeOH) was added at 0 °C under nitrogen atmosphere. Then, the reaction mixture was allowed to stir at 0 °C for 24 h. 2 N HCl was added to quench the reaction, and the resulting solution was washed with H_2O, organic layer was dried over anhydrous $MgSO_4$, filtered, and concentrated *in vacuo*. The resulting crude residue was purified by silica gel column chromatography to afford the desired product **64** (1.01 g, 94%) as a white solid. $[\alpha]_D^{20}$ = -13.8 (*c* = 1.0, $CHCl_3$); IR (thin film in KBr) ν 2927, 1719, 1268, 1108, 1069, 1028, 711 cm^{-1}; ^1H NMR (600 MHz, $CDCl_3$) δ = 8.04-7.18 (m, 22 H, Ar-H), 5.19 (s, 1 H, H2'), 5.14 (s, 1 H, H2), 5.05 (s, 1 H, H1), 4.95-4.91 (m, 2 H, H1', CH_2Ph), 4.82 (d, *J* = 11.8 Hz, 1 H, CH_2Ph), 4.69-4.67 (m, 2 H, $2CH_2Ph$), 4.38 (td, *J* = 4.1 Hz, *J* = 6.3 Hz, 1 H, H5), 4.14 (dd, *J* = 3.4 Hz, *J* = 8.9 Hz, H3'), 3.96-3.87 (m, 5 H, H3, H4, $H6_a$, $H6_b$, $CH(CH_3)_2$), 3.78-3.75 (m, 1 H, H5'), 3.41 (t, *J* = 9.2 Hz, H4'), 1.25 (d, *J* = 6.3 Hz, 3 H, ^{i}Pr), 1.16 (d, *J* = 6.3 Hz, 3 H, ^{i}Pr), 1.00 (d, *J* = 6.2 Hz, 3 H, H6'), 0.80 (s, 9 H, ^{t}Bu), 0.06 (s, 3 H, CH_3), 0.01 (s, 3 H, CH_3); ^{13}C NMR (150 MHz, $CDCl_3$) δ = 166.6 (C), 165.9 (C), 138.0 (C), 136.0 (C), 133.4 (CH), 133.3 (CH), 133.0 (CH), 129.91 (CH), 129.9 (CH), 129.7 (CH), 129.6 (CH), 128.7 (CH), 128.5 (CH), 128.4 (CH), 128.0 (CH), 127.9 (CH), 127.8, (CH), 127.7 (CH), 126.5 (CH), 126.2 (CH), 126.0 (CH), 100.5 (CH), 96.3 (CH), 81.3 (CH), 76.2 (CH), 75.3 (CH_2), 73.9 (CH), 72.2 (CH_2), 71.1 (CH), 69.9 (CH),

69.8 (CH), 68.5 (CH), 67.5 (CH), 61.7 (CH_2), 25.8 (CH_3), 23.4 (CH_3), 21.4 (CH_3), 17.9 (CH_3), 17.8 (CH_3); HRMS m/z $(M+Na)^+$ calcd for $C_{53}H_{64}NaO_{12}Si^+$ 943.4059, found 943.4077.

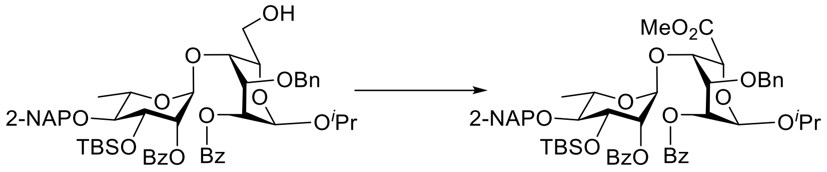

64 **68**

C**ompound 68.** To a solution of compound **64** (428 mg, 258 μmol) in a mixed solvent of CH_2Cl_2/H_2O (12.9 mL, 1/2), TEMPO (12.1 mg, 77.0 μmol) and BAIB (332 mg, 1.03 mmol) were added, and the reaction mixture was allowed to stir at room temperature for 24 h. Then, the reaction mixture was diluted with EtOAc, washed with 10% $Na_2S_2O_3$ and brine. The organic layer was dried over anhydrous $MgSO_4$, filtered, and concentrated *in vacuo* to afford the crude product, which was directly used for the next reaction without further purification. Freshly prepared CH_2N_2 (5.16 mmol in Et_2O) was added to a solution of the crude acid (258 μmol) in THF (4.0 mL) at 0 °C, and the reaction mixture was allowed to stir for 0.5 h at the same temperature. AcOH was added to quench the reaction, then the reaction mixture was diluted with EtOAc and washed with sat. $NaHCO_{3(aq)}$, and brine. Organic layer was dried over anhydrous $MgSO_4$, filtered, and concentrated *in vacuo*. The resulting crude residue was purified by silica gel column chromatography to afford the desired product **68** (355 mg, 79%) as a white solid. $[\alpha]_D^{20}$ = -37.1 (*c* = 1.5, $CHCl_3$); IR (thin film in KBr) ν 2927, 2855, 1722, 1264, 1106, 1069, 710 cm^{-1}; ^1H NMR (600 MHz, $CDCl_3$) δ = 8.04-7.11 (m, 22 H, Ar-H), 5.37 (d, *J* = 3.4 Hz, 1 H, H1), 5.15-5.14 (m, 2 H, H2', H2), 4.93-4.91 (m, 2 H, H5, CH_2Ph), 4.87 (s, 1 H, H1'), 4.84 (ABq, *J* = 11.6 Hz, 1 H, CH_2Ph), 4.71 (ABq, *J* = 11.6 Hz, 1 H, CH_2Ph), 4.65 (d, *J* = 11.3 Hz, 1 H, CH_2Ph), 4.15-4.12 (m, 2 H, H3', H4), 4.06-4.04 (m, 1 H, $CH(CH_3)_2$), 3.94-3.93 (m, 4 H, OCH_3, H3), 3.80-3.77 (m, 1 H, H5'), 3.42 (t, *J* = 9.4 Hz, 1 H, H4'), 1.24 (d, *J* = 6.2 Hz, 3 H, ^{i}Pr), 1.16 (d, *J* = 6.1 Hz, 3 H, ^{i}Pr), 0.95 (d, *J* = 6.2 Hz, 3 H, H6'), 0.81 (s, 9 H, ^{t}Bu), 0.12 (s, 3 H, CH_3), 0.07 (s, 3 H, CH_3); ^{13}C NMR (150 MHz, $CDCl_3$) δ = 170.3 (C), 165.8 (C), 165.8 (C), 137.7 (C), 136.0 (C), 133.2 (CH), 133.0 (CH), 130.1 (CH), 129.9 (CH), 129.8 (CH), 129.4 (CH), 128.6 (CH), 128.5 (CH), 128.4 (CH), 128.0 (CH), 127.9 (CH), 127.8 (CH), 126.7 (CH), 126.4 (CH), 126.1 (CH), 125.9 (CH), 100.6 (CH), 96.7 (CH), 81.1 (CH), 76.5 (CH), 75.6 (CH), 75.5 (CH_2), 73.4 (CH), 72.6 (CH_2), 71.2 (CH), 70.9 (CH), 70.0 (CH), 69.4 (CH), 68.6 (CH), 52.6 (CH), 29.8 (CH_3), 25.8 (CH_3), 23.3 (CH_3), 21.6 (CH_3), 17.8 (CH_3), 17.7

(CH_3); HRMS m/z $(M+Na)^+$ calcd for $C_{54}H_{64}NaO_{13}Si^+$ 971.4008, found 971.4006.

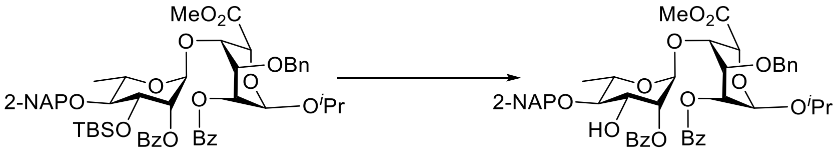

68 72

Compound 72. To a solution of compound **68** (50 mg, 649 μmol) in ACN (0.5 mL), PMA (0.438 mg, 0.24 μmol) was added at room temperature, and the reaction mixture was allowed to stir at room temperature for 3 h. Then, the reaction mixture was diluted with EtOAc, washed with sat. $NaHCO_{3(aq)}$ and brine, organic layer was dried over anhydrous $MgSO_4$, filtered, and concentrated *in vacuo*. The resulting crude residue was purified by silica gel column chromatography to afford the desired product **72** (41 mg, 92%) as a white solid. $[\alpha]_D^{20}$ = -11.2 (*c* = 0.6, $CHCl_3$); IR (thin film in KBr) ν 3505, 2972, 2927, 1761, 1721, 1267, 1110, 1070, 1026, 711 cm^{-1}; ^1H NMR (600 MHz, $CDCl_3$) δ = 8.05-7.21 (m, 22 H, Ar-H), 5.33 (d, *J* = 2.1 Hz, 1 H, H1), 5.21 (dd, *J* = 1.5 Hz, *J* = 3 Hz, 1 H, H2'), 5.14 (t, *J* = 2.5 Hz, 1 H, H2), 4.92 (s, 2 H, H1',H5), 4.86 (ABq, *J* = 11.6 Hz, 1 H, CH_2Ph), 4.76-4.68 (m, 3 H, 3 CH_2Ph), 4.17 (t, *J* = 3.2 Hz, 1 H, H4), 4.05-4.01 (m, 2 H, H3', $CH(CH_3)_2$), 3.92-3.90 (m, 4 H, OCH_3, H3), 3.73-3.71 (m, 1 H, H5'), 3.38 (t, *J* = 9.4 Hz, 1 H, H4'), 1.25 (d, *J* = 6.1 Hz, 3 H, ^{i}Pr), 1.18 (d, *J* = 6.1 Hz, 3 H, ^{i}Pr), 1.10 (d, *J* = 6.2 Hz, 3 H, H'6); ^{13}C NMR (150 MHz, $CDCl_3$) δ = 170.3 (C), 165.9 (C), 165.8 (C), 137.7 (C), 135.7 (C), 133.4 (CH), 133.3 (CH), 133.1 (CH), 130.0 (CH), 129.9 (CH), 128.5 (CH), 128.4 (CH), 128.3 (CH), 128.0 (CH), 127.9 (CH), 126.9 (CH), 126.3 (CH), 126.2 (CH), 126.1 (CH), 99.7 (CH), 96.9 (CH), 81.2 (CH), 75.3 (CH), 75.0 (CH_2), 74.9 (CH), 73.0 (CH_2), 72.4 (CH), 70.9 (CH), 70.2 (CH), 69.2 (CH), 68.4 (CH), 52.7 (CH), 23.4 (CH_3), 21.6 (CH_3), 17.8 (CH_3); HRMS m/z $(M+Na)^+$ calcd for $C_{48}H_{50}NaO_{13}^+$ 857.3144, found 857.3155.

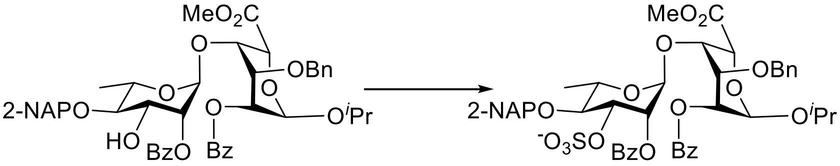

72 76

Compound 76. To a solution of compound **72** (31 mg, 34.4 μmol) in anhydrous DMF (0.6 mL), $SO_3{\cdot}Et_3N$ (47 mg, 240.8 μmol) was added under nitrogen atmosphere at room

temperature. Then, the reaction mixture was heated to 60 °C and allowed to stir at same temperature for 24 h. The reaction flask was cooled down to room temperature, sat. $NaHCO_{3(aq)}$ solution was added, and the mixture was allowed to stir for 12 h. The resulting mixture was extracted with THF, and the combined organic layers were washed with brine, dried over anhydrous $MgSO_4$, filtered, and concentrated *in vacuo*. The crude residue was subjected to AG 50W-X8 cation exchange column (Na^+ form) followed by Sephadex LH-20 column using MeOH as an eluent to afford the desired product **76** (30 mg, 86%) as a white solid. $[\alpha]_D^{20}$ = -53.2 (c = 0.6, MeOH); IR (cm^{-1}): 3845, 2927, 1717, 1267, 1109, 712; ^1H NMR ($CDCl_3$, 600 MHz): 7.95-6.97 (m, 22 H, Ar-H), 5.54 (s, 1 H, H2'), 5.36 (d, *J* = 5.0 Hz, 1 H, H1), 5.02-4.97 (m, 2 H, H2, CH_2Ph), 4.94 (d, *J* = 1.8 Hz, 1 H, H1'), 4.89 (dd, *J* = 3.2 Hz, *J* = 8.6 Hz, 1 H, H3'), 4.82 (d, *J* = 2.4 Hz, 1 H, H5), 4.68-4.63 (m, 3 H, CH_2Ph), 4.08 (t, *J* = 5.3 Hz, 1 H, H4), 3.92-3.89 (m, 1 H, $CH(CH_3)_2$), 3.86-3.84 (m, 1 H, H3), 3.77 (t, *J* = 6.8 Hz, 1 H, H5'), 3.53 (t, *J* = 8.9 Hz, 1 H, H4'), 1.10 (d, *J* = 6.2 Hz, 3 H, ^{i}Pr), 1.03 (d, *J* = 6.1 Hz, 3 H, ^{i}Pr), 0.84 (d, *J* = 6.1 Hz, 3 H, H6'); ^{13}C NMR ($CDCl_3$, 150 MHz): δ = 172.1 (C), 167.2 (C), 167.1 (C), 139.0 (C), 137.6 (C), 134.9 (C), 134.7 (C), 134.6 (CH), 134.5 (CH), 131.5 (CH), 131.0 (CH), 130.9 (CH), 130.7 (CH), 130.0 (CH), 129.7 (CH), 129.5 (CH), 129.4 (CH), 129.2 (CH), 128.9 (CH), 128.9 (CH), 128.8 (CH), 128.4 (CH), 127.1 (CH), 127.0 (CH), 100.0 (CH), 98.3 (CH), 79.7 (CH), 79.6 (CH), 77.6 (CH), 76.3 (CH), 75.6 (CH), 74.7 (CH_2), 73.1 (CH_2), 72.8 (CH), 71.5 (CH), 69.5 (CH), 53.3 (CH_3), 23.7 (CH_3), 22.3 (CH_3), 18.4 (CH_3); HRMS m/z $(M+Na)^+$ calcd for $C_{48}H_{50}NaO_{16}S^+$ 937.2712 found 937.2699.

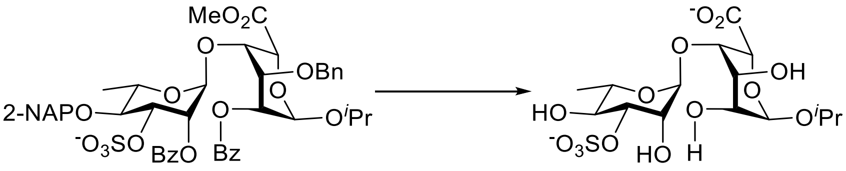

76 5

Compound 5. To a solution of compound **76** (150 mg, 0.15 mmol) in THF/MeOH (2.0 mL, 1/1), LiOH (1.0 mL, 0.5 M) was added at room temperature. Then, the reaction mixture was allowed to stir at room temperature for 24 h. AG 50W-X8 cation exchange resin was added to quench the reaction, and pH was adjusted to 7. The whole mixture was filtered and concentrated *in vacuo*. The crude residue was subjected to Sephadex LH-20 column followed by AG 50W-X8 cation exchange column (Na^+ form) using MeOH as an eluent to afford the desired compound, which was subjected to next reaction. The residue was dissolved in

MeOH/Phosphate buffer (pH = 7) (3.0 mL, 1/1), Pd on activated charcoal (50 % Pd, 200 mg) was added and purged with hydrogen. Then, the reaction was equipped with a hydrogen balloon at room temperature, after stirring for 48 h, the whole mixture was filtered, and the solution was concentrated under reduced pressure. The crude residue was purified by Sephadex G-10 column followed by AG 50W-X8 cation exchange column (Na^+ form) using water as eluent to afford the desired compound **5** (69 mg, 48%) as a white solid. $[\alpha]_D^{20}$ = -6.5 (*c* = 0.5, H_2O); ^1H NMR (D_2O, 600 MHz): 4.90 (d, *J* =4.7 Hz, 1 H, H1), 4.81 (s, 1 H, H1'), 4.40 (s, 1 H, H5), 4.35 (dd, *J* =2.9 Hz, *J* = 9.7 Hz,1 H, H-3'), 4.12 (s, 1 H, H2'), 3.97-3.90 (m, 2 H, H5', $CH(CH_3)_2$), 3.86 (t, *J* =4.4 Hz,1 H, H4), 3.72 (dd, *J* = 6.0 Hz, *J* = 7.9 Hz,1 H, H3), 3.48 (t, *J* =9.7 Hz, 1 H, H4'), 3.35 (dd, *J* = 4.8 Hz, *J* = 7.7 Hz,1 H, H2), 1.21 (d, *J* = 6.1 Hz, 3 H, H6'), 1.11-1.09 (m, 6 H, ^{i}Pr); ^{13}C NMR (D_2O, 150 MHz): δ = 174.8 (C), 100.7 (CH), 99.3 (CH), 78.5 (CH), 71.8 (CH), 71.4 (CH), 71.2 (CH), 70.9 (CH), 69.9 (CH), 69.0 (CH), 68.7 (CH), 22.4 (CH_3), 20.8 (CH_3), 16.6 (CH_3); HRMS *m/z* $(M+H)^-$ calcd for $C_{15}H_{25}O_{14}S^-$ 461.0970 found 461.0962.

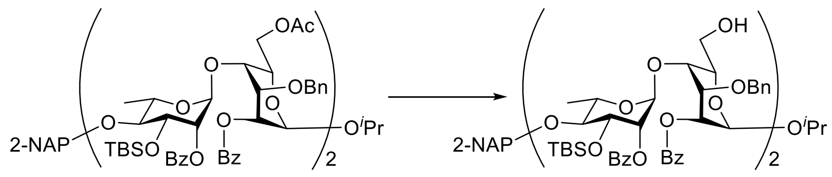

59 **65**

Compound 65. To a solution of compound **59** (1.7 g, 0.676 mmol) in CH_2Cl_2, $Mg(OMe)_2$ (5.0 mL, 7-8% in MeOH) was added at 0 °C under N_2 atmosphere. The reaction mixture was allowed to stir at same temperature for 24 h. Then, 2 N HCl was added to quench the reaction, and the resulting solution was washed with sat $NaHCO_{3(aq)}$ and H_2O. Organic layer was dried over anhydrous $MgSO_4$, filtered, and concentrated *in vacuo*. The resulting crude residue was purified by flash column chromatography to afford the desired product **65** (1.054 g, 94%) as a white solid. $[\alpha]_D^{20}$ = -33.6 (*c* = 0.7, $CHCl_3$); IR (thin film in KBr) ν 3850, 3734, 2928, 2855, 1718, 1268, 1109, 1070, 1026, 710 cm^{-1}; ^1H NMR (600 MHz, $CDCl_3$) δ = 8.02-7.00 (m, 8 H, Ar-H), 7.90-7.78 (m, 3 H, Ar-H), 7.58-7.55 (m, 4 H, Ar-H), 7.50-7.39 (m, 8 H, Ar-H), 7.34-7.32 (m, 2 H, Ar-H), 7.26-7.18 (m, 12 H, Ar-H), 5.53 (s, 1 H, H1''), 5.18 (s, 1 H, H2), 5.16 (s, 1 H, H2'), 5.12 (dd, *J* =1.6 Hz, *J* = 3.2 Hz, 1 H, H2'''), 5.07 (s, 1 H, H2''), 5.04 (s, 1H, H1), 4.92 (s, 1 H, H1'), 4.82-4.78 (m, 3 H, Ar-CH_2), 4.69-4.64 (m, 2 H, Ar-CH_2), 4.58-4.54 (m, 3 H, Ar-CH_2), 4.38 (t, *J* = 6.5 Hz, 1 H, H5), 4.25-4.21 (m, 2 H, H3', H5''), 3.97-3.82 (m, 10 H, H3, H3'', H3''', H4, H4', H6, H6'', ^{i}Pr), 3.78-3.74 (m, 2 H, H4'', H5'), 3.42 (m, 1 H, H5'''), 3.27

(t, J = 3.2 Hz, 1 H, H4'''), 1.26 (d, J = 6.2 Hz, 3 H, ^{i}Pr), 1.16 (d, J = 6.1 Hz, 3 H, ^{i}Pr), 1.03 (d, J = 6.1 Hz, 3 H, H6'), 0.85 (d, J = 6.2 Hz, 3 H, H6'''), 0.76 (s, 9 H, ^{t}Bu), 0.59 (s, 9 H, ^{t}Bu), 0.03 – (-0.05) (3s, 12 H, 4CH3); ^{13}C NMR (150 MHz, $CDCl_3$) δ = 166.6 (C), 165.8 (C), 165.7 (C), 137.9 (C), 137.7 (C), 136.0 (C), 133.6 (CH), 133.5 (CH), 133.4 (CH), 133.4 (CH), 133.2 (CH), 133.0 (CH), 130.0 (CH), 129.9 (CH), 129.8 (CH), 129.7 (CH), 129.6 (CH), 129.5 (CH), 128.7 (CH), 128.6 (CH), 128.6 (CH), 128.5 (CH), 128.4 (CH), 127.9 (CH), 127.9 (CH), 127.8 (CH), 127.8 (CH), 127.7 (CH), 126.4 (CH), 126.2 (CH), 125.9 (CH), 101.1 (CH), 100.0 (CH), 98.5 (CH), 96.5 (CH), 81.0 (CH), 76.2 (CH), 75.6 (CH), 75.2 (CH), 75.0 (CH_2), 73.9 (CH), 73.9 (CH), 73.3 (CH), 72.2 (CH_2), 72.0 (CH_2), 71.0 (CH), 70.0 (CH), 69.8 (CH), 68.4 (CH), 68.3 (CH), 67.6 (CH), 66.6 (CH), 61.6 (CH_2), 61.6 (CH_2), 25.7 (CH_3), 25.5 (CH_3), 23.4 (CH_3), 21.5 (CH_3), 18.3 (CH_3), 17.8 (CH_3), 17.6 (CH_3), 17.5 (CH_3); HRMS m/z $(M+Na)^+$ calcd for $C_{92}H_{112}NaO_{23}Si_2^+$ 1663.7025, found 1663.7065.

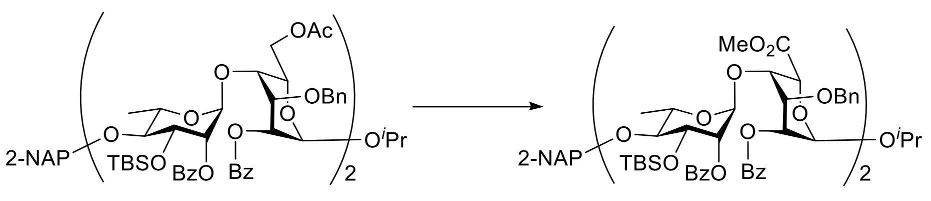

65 **69**

Compound 69. To a solution of compound **65** (875 mg, 533 μmol) in a mixed solvent CH_2Cl_2/H_2O (26.4 mL, 1/2), TEMPO (25 mg, 0.16 mmol) and BAIB (687 mg, 2.13 mmol) were added, and the reaction mixture was allowed to stir at room temperature for 24 h. Then, the reaction mixture was diluted with EtOAc, washed with 10% $Na_2S_2O_3$ and brine. The organic layer was dried over anhydrous $MgSO_4$, filtered, and concentrated *in vacuo* to afford the crude product, which was directly used for the next reaction without further purification. Freshly prepared CH_2N_2 (10.7 mmol in Et_2O) was added to a solution of crude acid (0.533 mmol) in THF (8.8 mL) at 0 °C, and the reaction mixture was allowed to stir for 0.5 h at same temperature. AcOH was added to quench the excess CH_2N_2, then the reaction mixture was diluted with EtOAc and washed with sat. $NaHCO_{3(aq)}$, and brine. Organic layer was dried over anhydrous $MgSO_4$, filtered, and concentrated *in vacuo*. The resulting crude residue was purified by silica gel column chromatography to afford the desired product **69** (686 mg, 76%) as a white solid. $[\alpha]_D^{20}$ = -46.8 (c = 0.7, $CHCl_3$); IR (thin film in KBr) ν 2951, 2927, 2855, 1766, 1721, 1267, 1108, 1069, 1045, 1028, 712 cm^{-1}; ^{1}H NMR (600 MHz, $CDCl_3$) δ = 8.03-7.95 (m, 8 H, Ar-H), 7.84-7.75 (m, 3 H, Ar-H), 7.57-7.52 (m, 3 H, Ar-H), 7.49-7.41 (m, 7 H,

Ar-H), 7.38-7.35 (m, 3 H, Ar-H), 7.28-7.26 (m, 6 H, Ar-H), 7.23-7.19 (m, 3 H, Ar-H), 7.17-7.08 (m, 5 H, Ar-H), 5.57 (s, 1 H, H1”), 5.35 (d, J = 3.1 Hz, 1 H, H1), 5.14 (t, J = 3.8 Hz, 1 H, H2), 5.11 (t, J = 2.3 Hz, 1 H, H2’), 5.06 (s, 1 H, H2”), 5.02 (s, 1 H, H2’”), 4.88 (d, J = 3.8 Hz, 1 H, H5), 4.85 (s, 1 H, H1’), 4.80 (ABq, J = 11.6 Hz, 1 H, Ar-CH_2), 4.74-4.67 (m, 5 H, Ar-CH_2, H1’”, H5”), 4.63 (ABq, J = 11.8 Hz, 1 H, Ar-CH_2, 4.46 (ABq, J = 11.4 Hz, 1 H, Ar-CH_2), 4.17 (dd, J = 3.2 Hz, J = 9.0 Hz, 1 H, H3’), 4.13 (t, J = 4.3 Hz, 1 H, H4), 4.01-3.96 (m, 3 H, H3, H3”, ^{i}Pr), 3.92-3.91 (m, 7 H, CO_2Me, H4”), 3.87-3.82 (m, 2 H, H4’, H3’”), 3.78-3.76 (m, 1 H, H5’), 3.33-3.32 (m, 1 H, H5’”), 3.21 (t, J = 9.1 Hz, 1 H, H4’”), 1.21 (d, J = 6.2 Hz, 3 H, ^{i}Pr), 1.13 (d, J = 6.1 Hz, 3 H, ^{i}Pr), 0.86 (d, J = 6.1 Hz, 3 H, H6’), 0.74-0.73 (m, 12 H, H6’”, ^{t}Bu), 0.52 (s, 9 H, ^{t}Bu), 0.03- (-0.05) (3s, 12 H, 4CH_3); ^{13}C NMR (150 MHz, $CDCl_3$) δ = 170.2 (C), 169.4 (C), 166.0 (C), 165.9 (C), 165.8 (C), 165.7 (C), 137.7 (C), 137.4 (C), 136.1 (C), 133.5 (CH), 133.4 (CH), 133.2 (CH), 132.9 (CH), 130.1 (CH), 130.0 (CH), 129.9 (CH), 129.8 (CH), 129.8 (CH), 129.7 (CH), 129.6 (CH), 129.2 (CH), 128.6 (CH), 128.6 (CH), 128.5 (CH), 128.5 (CH), 128.41 (CH), 128.4 (CH), 128.1 (CH), 128.01 (CH), 128.0 (CH), 127.9 (CH), 127.81 (CH), 127.8 (CH), 127.7 (CH), 126.5 (CH), 126.3 (CH), 126.1 (CH), 125.9 (CH), 101.8 (CH), 100.3 (CH), 99.6 (CH), 97.0 (CH), 80.9 (CH), 76.2 (CH), 75.3 (CH_2), 75.0 (CH_2), 73.5 (CH), 73.2 (CH), 73.1 (CH_2), 72.9 (CH_2), 72.2 (CH), 71.3 (CH), 71.1 (CH), 70.9 (CH), 70.2 (CH), 69.0 (CH), 68.4 (CH), 68.0 (CH), 67.9 (CH), 67.7 (CH), 52.7 (CH), 52.6 (CH), 25.8 (CH_3), 25.5 (CH_3), 23.3 (CH_3), 21.6 (CH_3), 18.2 (CH_3), 17.8 (CH_3) ,17.5 (CH_3), 17.3 (CH_3); HRMS m/z $(M+Na)^+$ calcd for $C_{94}H_{112}NaO_{25}Si_2^+$ 1719.6923, found 1719.6948.

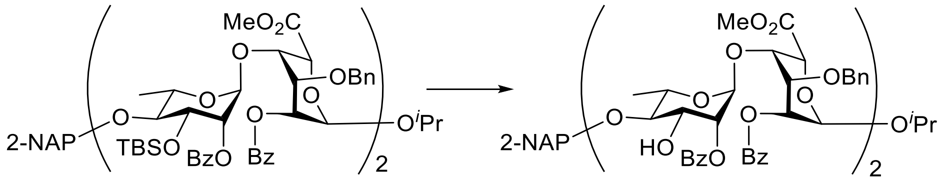

69 **73**

Compound 73. To a solution of compound **69** (100 mg, 58.0 μmol) in ACN (3.0 mL), PMA (150 mg, 174 μmol) was added. Then, the reaction mixture was allowed to stir at room temperature for 24 h, diluted with EtOAc, washed with sat. $NaHCO_{3\ (aq)}$ and brine. The organic layer was dried over $MgSO_4$, filtered, and concentrated *in vacuo*. The resulting crude residue was purified by flash column chromatography to afford the desired product **73** (77 mg, 86%) as a white solid. $[\alpha]_D^{20}$ = -19.8 (c = 1.0, $CHCl_3$); IR (thin film in KBr) ν 3502, 2971, 2929, 1720, 1267, 1111, 1071, 1038, 712 cm^{-1}; ^{1}H NMR (600 MHz, $CDCl_3$) δ = 8.15-8.13 (m, 2 H,

Ar-H), 8.07-8.00 (m, 6 H, Ar-H), 7.88-7.81 (m, 3 H, Ar-H), 7.69 (s, 1 H, Ar-H), 7.62-7.58 (m, 2 H, Ar-H), 7.54-7.43 (m, 7 H, Ar-H), 7.38-7.35 (m, 8 H, Ar-H), 7.25-7.18 (m, 4 H, Ar-H), 5.50 (d, J = 1.7 Hz, 1 H, H1”), 5.33 (s, 1 H, H1), 5.22 (s, 1 H, H2’), 5.18 (m, 2 H, H2, H2’”), 5.02 (s, 1H, H2”), 4.96-4.95 (m, 2 H, H1’, H5), 4.92 (s, 1 H, H1’”), 4.87-4.82 (m, 2 H, Ar-CH_2), 4.75-4.67 (m, 5 H, Ar-CH_2, H5”), 4.19 (t, J = 3.2 Hz, 1 H, H4), 4.15 (s, 1 H, H4”), 3.97 (s, 6 H, CO_2Me), 3.91 (t, J = 3.2 Hz, 1 H, H3), 3.88-3.85 (m, 2 H, H3”, H4’), 3.78-3.76 (m, 1 H, H5’”), 3.65-3.63 (m, 1 H, H5’), 3.42 (t, J = 9.4 Hz, 1 H, H4’”), 3.02 (d, J = 5.7 Hz, 1 H, OH), 1.29 (d, J = 6.9 Hz, 3 H, ^{i}Pr), 1.20 (d, J = 6.0 Hz, 3 H, ^{i}Pr), 1.05 (d, J = 6.2 Hz, 3 H, H6’”), 0.98 (d, J = 6.2 Hz, 3 H, H6’); ^{13}C NMR (150 MHz, $CDCl_3$) δ = 170.2 (C), 169.6 (C), 166.0 (C), 165.9 (C), 165.6 (C), 137.7 (C), 136.9 (C), 135.6 (C), 133.5 (CH), 133.5 (CH), 133.4 (CH), 133.3 (CH), 133.1 (CH), 130.2 (CH), 130.0 (CH), 129.9 (C), 129.4 (C), 129.0 (C), 128.6 (CH), 128.54 (CH), 128.52 (CH), 128.5 (CH), 128.4 (CH), 128.32 (CH), 128.3 (CH), 128.2 (CH), 128.0 (CH), 127.8 (CH), 127.1 (CH), 126.3 (CH), 126.2 (CH), 100.3 (CH), 99.6 (CH), 98.8 (CH), 97.0 (CH), 81.0 (CH), 78.7 (CH), 75.4 (CH), 75.2 (CH_2), 75.0 (CH), 75.0 (CH), 74.7 (CH), 73.2 (CH), 73.0 (CH), 72.7 (CH_2), 72.4 (CH_2), 70.8 (CH), 70.1 (CH), 70.0 (CH), 69.3 (CH), 69.2 (CH), 68.9 (CH), 68.5 (CH), 68.3 (CH), 67.9 (CH), 52.8 (CH), 52.8 (CH), 23.3 (CH_3), 21.6 (CH_3), 17.8 (CH_3), 17.4 (CH_3); HRMS m/z $(M+Na)^+$ calcd for $C_{82}H_{84}NaO_{25}^+$ 1491.5194, found 1491.5205.

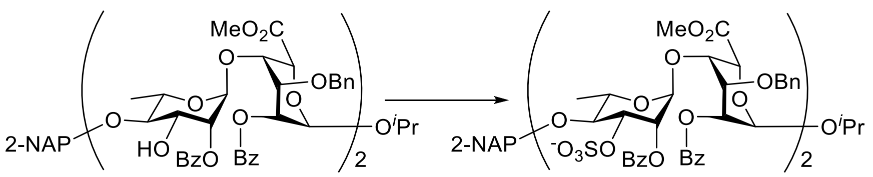

73 **77**

Compound 77. To a solution of compound **73** (124 mg, 84.0 μmol) in anhydrous DMF (2.0 mL), $SO_3 \cdot Et_3N$ (214 mg, 1.18 mmol) was added under nitrogen atmosphere at room temperature Then, the reaction mixture was heated to 60 °C and allowed to stir at same temperature for 72 h. The reaction flask was cooled down to room temperature, sat. $NaHCO_{3(aq)}$ solution was added, and the mixture was allowed to stir for 12 h. The resulting mixture was extracted with THF, and the combined organic layers were washed with brine, dried over anhydrous $MgSO_4$, filtered, and concentrated *in vacuo*. The crude residue was subjected to AG 50W-X8 cation exchange column (Na^+ form) followed by Sephadex LH-20 column using MeOH as an eluent to afford the desired product **77** (118 mg, 86%) as a white solid. ^{1}H NMR

(600 MHz, CD_3OD): δ = 8.00-7.90 (m, 8 H, Ar-H), 7.77-7.70 (m, 3 H, Ar-H), 7.60 (s, 1 H, Ar-H), 7.55-7.45 (m, 3 H, Ar-H), 7.45-7.38 (m, 6 H, Ar-H), 7.34-7.31 (m, 2 H, Ar-H), 7.25 (d, J = 8.1 Hz, 1 H), 7.18-7.06 (m, 12 H, Ar-H), 5.62 (s, 1 H, H1''), 5.51 (s, 1 H, H2'), 5.40 (s, 1 H, H2'''), 5.31 (s, 1 H, H1), 5.17 (s, 1 H, H2''), 5.04 (t, J = 4.4 Hz, 1 H, H2), 4.92 (s, 1 H, H1'), 4.87 (d, J = 8.2 Hz, 1 H, H3'), 4.70 (d, J = 3.6 Hz, 1 H, H3'''), 4.62-4.59 (m, 3 H, Ar-CH_2, H5''), 4.50-4.42 (m, 5 H, Ar-CH_2), 4.16 (t, J = 4.8 Hz, 1 H, H4), 4.06-3.87 (m, 11 H, H3, H3'', H4', H4'', ^{i}Pr, CO_2Me), 3.72 (s, 1 H, H5'), 3.37 (s, 2 H, H4''', H5'''), 1.09 (d, J = 6.2 Hz, 3 H, ^{i}Pr), 1.03 (d, J = 6.1 Hz, 3 H, ^{i}Pr), 0.73 (d, J = 5.9 Hz, 3 H, H6'), 0.67 (d, J = 3.7 Hz, 3 H, H6'''); ^{13}C NMR (150 MHz, CD_3OD): δ = 172.0 (C), 171.5 (C), 167.3 (C), 167.2 (C), 167.2 (C), 167.1 (C), 139.0 (C), 138.9 (C), 137.4 (C), 135.1 (C), 134.8 (C), 134.6 (C), 134.6 (C), 134.5 (CH), 134.4 (CH), 131.4 (CH), 131.3 (CH), 131.2 (CH), 131.1 (CH), 131.1 (CH), 131.0 (CH), 130.8 (CH), 130.7 (CH), 130.2 (CH), 129.9 (CH), 129.7 (CH), 129.6 (CH), 129.51 (CH), 129.5 (CH), 129.3 (CH), 129.2 (CH), 129.0 (CH), 128.8 (CH), 128.7 (CH), 128.3 (CH), 127.1 (CH), 127.0 (CH), 102.1 (CH), 100.1 (CH), 98.3 (CH), 77.9 (CH), 77.3 (CH), 77.3 (CH), 77.1 (CH), 76.4 (CH), 74.9 (CH), 74.4 (CH_2), 73.3 (CH_2), 73.1 (CH), 73.0 (CH), 72.9 (CH), 72.1 (CH), 70.9 (CH), 69.5 (CH), 69.1 (CH), 69.0 (CH), 68.6 (CH), 53.7 (CH), 53.4 (CH), 23.7 (CH_3), 22.3 (CH_3), 18.6 (CH_3), 18.3 (CH_3); HRMS *m/z* $(M+H)^-$ calcd for $C_{82}H_{83}O_{31}S_2^-$ 1633.4466 found 1633.4399.

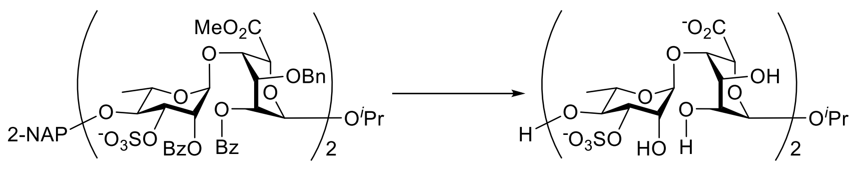

77 **6**

Compound 6. To a solution of compound **77** (240 mg, 143 μmol) in THF/MeOH (2.0 mL, 1/1), LiOH (1.0 mL, 0.5 M) was added at room temperature. Then, the reaction mixture was allowed to stir at room temperature for 72 h, AG 50W-X8 cation exchange resin was added to quench the reaction, and pH was adjusted to 7. The whole mixture was filtered through a pad of Celite® and concentrated *in vacuo*. The crude residue was subjected to Sephadex LH-20 column followed by AG 50W-X8 cation exchange column (Na^+ form) using MeOH as an eluent to afford the desired compound. The obtained compound was further used to next step. The residue was dissolved in MeOH/Phosphate buffer (2.0 mL, pH = 7) and Pd on activated charcoal (50 % Pd, 200 mg) were added, and purged with hydrogen gas. Then, the reaction mixture was equipped with a hydrogen balloon at room temperature and allowed to stir for 72

h. The reaction mixture was filtered through a pad of Celite® and the filtrate was concentrated *in vacuo*. The resulting crude residue was purified by using a Sephadex G-10 column followed by AG 50W-X8 cation exchange column (Na^+ form) using water as an eluent to afford the desired product **6** (89 mg, 74%) as a white solid. ^1H NMR (600 MHz, D_2O): δ = 4.98 (d, 1 H, *J* = 3.8 Hz, H-1”), 4.93 (d, 1 H, *J* = 5.0 Hz, H1), 4.81 (s, 1 H, H1”’), 4.79 (s, 1 H, H1’), 4.52 (dd, 1 H, *J* = 3.3 Hz, *J* = 9.5 Hz, H3”’), 4.50 (d, 1 H, *J* = 3.3 Hz, H5”), 4.33 (d, 1 H, 3.3 Hz, H5), 4.30 (dd, 1 H, *J* = 3.1 Hz, *J* = 9.9 Hz, H3’), 4.11 (s, 2 H, H2’, H2”’), 3.97-3.87 (m, 5 H, H4, H4”, H5’, H5”’, ^{i}Pr), 3.74-3.67 (m, 3 H, H3, H3”, H4”’), 3.57 (dd, 1 H, *J* = 4.1 Hz, *J* = 8.2 Hz, H2”), 3.47 (t, 1 H, *J* = 9.7 Hz, H4’), 3.35 (dd, 1 H, *J* = 5.1 Hz, *J* = 7.9 Hz, H2), 1.27 (d, 3 H, *J* = 6.2 Hz, H6’), 1.14 (d, 3 H, *J* = 6.2 Hz, H6”’), 1.11-1.09 (m, 6 H, ^{i}Pr); ^{13}C NMR (150 MHz, D_2O): δ = 173.8 (C), 173.6 (C), 103.0 (CH), 101.3 (CH), 100.5 (CH), 99.2 (CH), 79.4 (CH), 78.6 (CH), 78.4 (CH), 78.2 (CH), 76.1 (CH), 71.7 (CH), 71.5 (CH), 71.4 (CH), 71.1 (CH), 70.6 (CH), 70.5 (CH), 69.9 (CH), 69.0 (CH), 68.8 (CH), 68.6 (CH), 67.8 (CH), 22.4 (CH_3), 20.8 (CH_3), 16.7 (CH_3), 16.6 (CH_3); HRMS m/z $(M+H)^+$ calcd for $C_{27}H_{43}O_{27}S_2Na^-$ 885.1258 found 885.1254.

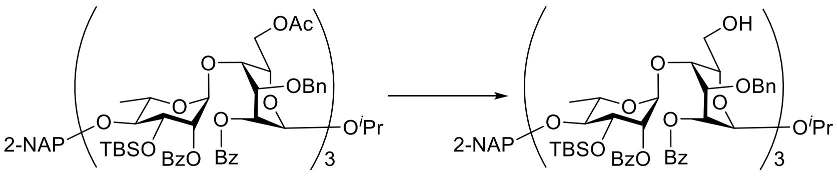

61 **66**

Compound 66. To a solution of compound **61** (711 mg, 0.286 mmol) in CH_2Cl_2, $Mg(OMe)_2$ (15.0 mL, 7-8% in MeOH) was added at 0 °C under nitrogen atmosphere. The reaction mixture was allowed to stir at same temperature for 24 h. Then, 2 N HCl was added to quench the reaction, and the resulting solution was washed with sat. $NaHCO_{3(aq)}$ and H_2O. The organic layer was dried over anhydrous $MgSO_4$, filtered, and concentrated *in vacuo*. The resulting crude residue was purified by flash column chromatography to afford the desired product **66** (633 mg, 93%) as a white solid. $[\alpha]_D^{28}$ = -74.5 (*c* = 3.0, $CHCl_3$); IR (thin film in KBr) ν 3521, 2930, 2858, 1719, 1602, 1584, 1493, 1452, 1385, 1361, 1316, 1176, 1110, 1070, 1027, 977, 898, 858, 838, 777, 754, 711, 620 cm^{-1}; ^1H NMR (600 MHz, $CDCl_3$): δ = 8.06-8.00 (m, 12 H, Ar-H), 7.90-7.82 (m, 3 H, Ar-H), 7.63-7.59 (m, 6 H, Ar-H), 7.54-7.45 (m, 11 H, Ar-H), 7.41-7.37 (m, 9 H, Ar-H), 7.30-7.23 (m, 10 H, Ar-H), 5.58 (s, 1 H, IdoA H1), 5.46 (s, 1 H, IdoA H1), 5.23 (s, 1 H, H2), 5.21 (s, 1 H, Rha H2), 5.14 (m, 3 H, Rha H2 x2, IdoA H2), 5.08 (s, 1

H, H1), 4.97 (s,2 H, Rha H1, IdoA H2), 4.87-4.83 (m, 3 H, Ar-CH_2, Rha H1 x2), 4.78-4.58 (m, 6 H, Ar-CH_2), 4.43 (m, 1 H, H5), 4.30-4.27 (m, 2 H, Rha H3, IdoA H5), 4.19 (m, 1 H, IdoA H5), 4.06 (dd, 1 H, J = 8.8 Hz, J = 2.4 Hz, Rha H3), 4.00-3.88 (m, 14 H, Rha H3, Rha H4, H3, H4, H6, IdoA H3 x2, IdoA H6 x2), 3.85-3.81 (m, 2 H, Rha H5, IdoA H4), 3.78-3.73 (m, 2 H, Rha H4, IdoA H4), 3.53-3.47 (m, 2 H, Rha H5 x2), 3.32 (m, 2 H, Rha H4), 2.26 (s, 3 H, OH), 1.29 (d, 3 H, J = 5.3 Hz, ^{i}Pr), 1.20 (d, 3 H, J = 6.0 Hz, ^{i}Pr), 1.08 (d, 3 H, J = 6.0 Hz, Rha H6), 0.91 (m, 6 H, J = 6.1 Hz, Rha H6 x2), 0.81 (s, 9 H, ^{t}Bu), 0.62 (s, 18 H, ^{t}Bu), 0.07-(-0.07) (m, 18 H, CH_3); ^{13}C NMR (150 MHz, $CDCl_3$): δ = 166.5 (C), 165.64 (C), 165.6 (C), 137.8 (C), 137.7 (C), 137.5 (C), 135.9 (C), 133.6 (CH), 133.5 (CH), 133.4 (CH), 133.3 (CH), 133.2 (CH), 133.1 (CH), 132.8 (CH), 129.85 (CH), 129.8 (CH), 129.79 (CH), 129.7 (CH), 129.6 (CH), 129.55 (CH), 129.5 (CH), 129.42 (CH), 129.4 (CH), 128.6 (CH), 128.5 (CH), 128.4 (CH), 128.35 (CH), 128.3 (CH), 127.88 (CH), 127.8 (CH), 127.77 (CH), 127.7 (CH), 127.6 (CH), 126.3 (CH), 126.1 (CH), 126.07 (CH), 125.8 (CH), 100.9 (CH), 100.3 (CH), 99.9 (CH), 98.5 (CH), 98.3 (CH), 96.3 (CH), 80.9 (CH), 76.1 (CH), 75.4 (CH), 75.3 (CH), 75.2 (CH), 74.8 (CH_2), 73.8 (CH), 73.7 (CH), 73.3 (CH), 73.1 (CH), 72.1 (CH_2), 72.0 (CH), 71.8 (CH_2), 71.6 (CH), 70.8 (CH), 69.8 (CH), 69.7 (CH), 68.4 (CH), 68.33 (CH), 68.3 (CH), 68.2 (CH), 68.1 (CH), 67.5 (CH), 66.5 (CH), 66.4 (CH), 61.5 (CH), 61.46 (CH), 61.4 (CH), 25.6 (CH_3), 25.5 (CH_3), 25.45 (CH_3), 25.4 (CH_3), 23.3 (CH_3), 21.3 (CH_3), 18.2 (CH_3), 17.9 (CH_3), 17.7 (CH_3), 17.54 (CH_3), 17.5 (CH_3), 17.4 (CH_3), -4.1 (CH_3), -4.2 (CH_3), -4.62 (CH_3), -4.67 (CH_3).

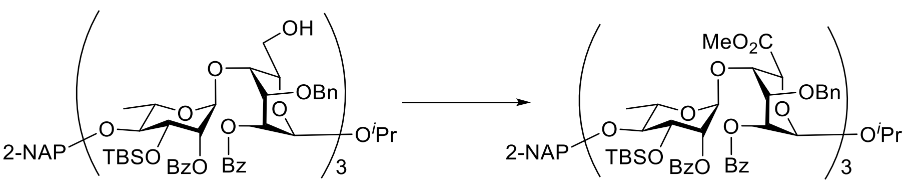

66

70

Compound 70. To a solution of compound **66** (137 mg, 58.0 μmol) in a mixed solvent of CH_2Cl_2/H_2O (4.05 mL, 1/2), TEMPO (3.6 mg, 23.0 μmol) and BAIB (144 mg, 433 μmol) were added at 0 °C, and the reaction mixture was allowed to stir at room temperature for 24 h. Then, the reaction mixture was diluted with EtOAc, washed with 10% $Na_2S_2O_3$ and brine. The organic layer was dried over anhydrous $MgSO_4$, filtered, and concentrated *in vacuo* to afford the crude product, which was directly used for the next reaction without further purification. Freshly prepared CH_2N_2 (1.74 mmol in Et_2O) was added to a solution of crude acid (58 μmol) in THF (1.37 mL) at 0 °C, and the reaction mixture was allowed to stir at room temperature for

0.5 h. AcOH was then added to quench the excess CH_2N_2, reaction mixture was diluted with EtOAc, washed with sat. $NaHCO_{3(aq)}$ and brine. The organic layer was dried over anhydrous $MgSO_4$, filtered, and concentrated *in vacuo*. The crude residue was purified by flash column chromatography to afford the desired product **70** (124 mg, 87%) as a white solid. $[\alpha]_D^{28}$ = -48.1 (*c* = 4.0, $CHCl_3$); IR (thin film in KBr) ν 2930, 2857, 1767, 1721, 1601, 1452, 1385, 1315, 1211, 1109, 1070, 1028, 838, 778, 752, 711; ^{1}H NMR ($CDCl_3$, 600 MHz): δ = 8.03-7.91 (m, 12 H, Ar-H), 7.84-7.75 (m, 3 H, Ar-H), 7.57-7.53 (m, 4 H, Ar-H), 7.49-7.41 (m, 12 H, Ar-H), 7.36-7.34 (m, 4 H, Ar-H), 7.28-7.25 (m, 9 H, Ar-H), 7.23-7.06 (m, 8 H, Ar-H), 5.58 (s, 1 H, H1''), 5.41 (s, 1 H, H1''''), 5.34 (d, 1 H, *J* = 3.3 Hz, H1), 5.14 (s, 1 H, H2), 5.10 (s, 1 H, H2'), 5.07 (s, 1 H, H2''), 5.00 (s, 2 H, H2''', H2'''''), 4.96 (s, 1 H, H2''''), 4.88-4.84 (m, 3 H, H5, H1', Ar-CH_2,), 4.80-4.60 (m, 9 H, Ar-CH_2, H1''', H1''''', H5''), 4.48-4.45 (m, 2 H, H5'''', Ar-CH_2), 4.16 (dd, 1 H, *J* = 9.2 Hz, *J* = 2.9 Hz, H3'), 4.12 (m, 1 H, H4), 4.01-3.88 (m, 17 H, H3, H3'', H3''', H3'''', H4'', H4'''', ^{i}Pr, CO_2Me), 3.81 (m, 1 H, H3'''''), 3.77 (m, 1 H, H5'), 3.66 (m, 1 H, H4'''), 3.43 (s, 1 H, H5'''), 3.32 (s, 1 H, H5'''''), 3.21 (t, 1 H, J = 9.2 Hz, H4'''''), 1.21 (d, 3 H, J = 6.2 Hz, ^{i}Pr), 1.13 (d, 3 H, J = 5.9 Hz, ^{i}Pr), 0.85 (d, 3 H, J = 5.9 Hz, H6'), 0.75 (d, 3 H, J = 5.9 Hz, H6'''''), 0.72 (s, 9 H, ^{t}Bu), 0.53 (d, 3 H, J = 5.2 Hz, H6'''''), 0.49 (s, 9 H, ^{t}Bu), 0.45 (s, 9 H, ^{t}Bu), 0.04-(-0.04) (m, 18 H, CH_3); ^{13}C NMR ($CDCl_3$, 150 MHz): δ = 170.0 (C), 169.2 (C), 169.1 (C), 165.9 (C), 165.8 (C), 165.7 (C), 165.6 (C), 165.5 (C), 137.5 (C), 137.4 (C), 137.2 (C), 135.9 (C), 133.5 (CH), 133.4 (CH), 133.3 (CH), 133.2 (CH), 133.1 (CH), 132.8 (CH), 130.0 (CH), 129.96 (CH), 129.9 (CH), 129.8 (CH), 129.7 (CH), 129.6 (CH), 129.5 (CH), 129.4 (CH), 129.1 (CH), 128.6 (CH), 128.55 (CH), 128.5 (CH), 128.46 (CH), 128.4 (CH), 128.39 (CH), 128.3 (CH), 128.15 (CH), 128.1 (CH), 128.0 (CH), 127.9 (CH), 127.86 (CH), 127.8 (CH), 127.74 (CH), 127.7 (CH), 127.6 (CH), 126.4 (CH), 126.2 (CH), 126.0 (CH), 125.7 (CH), 101.6 (CH), 101.0 (CH), 100.5 (CH), 100.2 (CH), 99.6 (CH), 96.8 (CH), 80.7 (CH), 76.1 (CH), 75.2 (CH), 74.9 (CH_2), 73.4 (CH), 73.1 (CH), 72.9 (CH), 72.7 (CH_2), 72.4 (CH_2), 72.3 (CH_2), 71.3 (CH), 70.9 (CH), 70.8 (CH), 70.5 (CH), 69.9 (CH), 68.8 (CH), 68.3 (CH), 68.1 (CH), 67.9 (CH), 67.8 (CH), 67.6 (CH), 67.4 (CH), 52.6 (CH), 52.5 (CH), 52.4 (CH), 25.6 (CH_3), 25.4 (CH_3), 25.2 (CH_3), 23.2 (CH_3), 21.5 (CH_3), 18.0 (CH_3), 17.8 (CH_3), 17.6 (CH_3), 17.4 (CH_3), 17.3 (CH_3), 17.2 (CH_3), -4.1 (CH_3), -4.2 (CH_3), -4.6 (CH_3), -4.71 (CH_3), -4.77 (CH_3), -4.78 (CH_3); HRMS m/z $(M+H)^+$ calcd for $C_{134}H_{160}O_{37}Si_3^-$ 2446.0019 found 2446.0011.

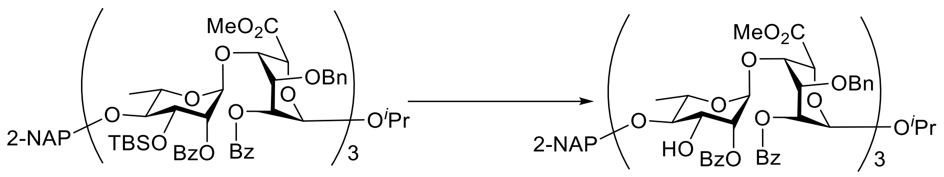

70 74

Compound 74. To a solution of compound **70** (50 mg, 20.0 μmol) in ACN (0.5 mL), PMA (15 mg, 10.0 μmol) was added. Then, the reaction mixture was allowed to stir at room temperature for 24 h, diluted with EtOAc and washed with sat. $NaHCO_{3(aq)}$ and brine. The organic layer was dried over anhydrous $MgSO_4$, filtered, and concentrated *in vacuo*. The crude residue was purified by flash column chromatography to afford the desired product **74** (35 mg, 84%) as a white solid. $[\alpha]_D^{28}$ = -12.1 (c = 1.0, $CHCl_3$); IR (thin film in KBr) ν 3515, 2931, 1720, 1601, 1452, 1316, 1111, 1070, 1028, 979, 795, 750, 711, 476 cm^{-1}; ^1H NMR (600 MHz, $CDCl_3$) δ = 8.10-7.93 (m, 12 H, Ar-H), 7.83-7.75 (m, 3 H, Ar-H), 7.63-7.53 (m, 4 H, Ar-H), 7.48-7.42 (m, 8 H, Ar-H), 7.40-7.29 (m, 9 H, Ar-H), 7.26-7.10 (m, 16 H, Ar-H), 5.45 (d, 1 H, *J* = 2.3 Hz, H-1''), 5.39 (d, 1 H, J = 1.8 Hz, H-1'''''), 5.27 (d, 1 H, J = 1.4 Hz, H-1), 5.15-5.12 (m, 4 H, H2, H2', H2''', H2'''''), 4.98-4.97 (m, 2 H, H2'', H2''''), 4.90-4.86 (m, 4 H, H1', H1''', H1''''', H5), 4.82-4.59 (m, 10 H, H5'', H5'''', Ar-CH_2), 4.13-4.09 (m, 3 H, H4, H4'', H4''''), 4.03-3.97 (m, 4 H, ^iPr, H3', H3''', H3'''''), 3.93-3.91 (m, 9 H, CO_2Me), 3.85-3.8-70 (m, 6 H, H3, H3'', H4', H4''', H4'''', H5'''''), 3.60-3.56 (m, 2 H, H5', H5'''), 3.37 (t, 1 H, *J* = 9.5, H4''''), 3.01 (s, 1 H, OH), 2.93 (s, 1 H, OH), 2.03 (s, 1 H, OH), 1.21 (d, 3 H, *J* = 6.2 Hz, ^iPr), 1.15 (d, 3 H, *J* = 6.1 Hz, ^iPr), 0.99 (d, 3 H, *J* = 6.5 Hz, H6'''''), 0.92-0.90 (m, 6 H, H6', H6'''); ^{13}C NMR ($CDCl_3$, 150 MHz): δ = 165.8 (C), 136.7 (C), 133.4 (CH), 130.0 (CH), 129.9 (CH), 129.8 (CH), 129.7 (CH), 129.5 (CH), 128.5 (CH), 128.45 (CH), 128.4 (CH), 128.3 (CH), 128.2 (CH), 128.18 (CH), 128.1 (CH), 128.0 (CH), 127.8 (CH), 127.7 (CH), 127.0 (CH), 126.21 (CH), 126.2 (CH), 126.0 (CH), 100.1 (CH), 99.9 (CH), 99.4 (CH), 98.8 (CH), 98.7 (CH), 96.8 (CH), 80.9 (CH), 78.4 (CH), 78.3 (CH), 75.3 (CH), 75.1 (CH_2), 75.0 (CH), 74.9 (CH), 74.7 (CH), 74.6 (CH), 74.5 (CH), 73.12 (CH), 73.11 (CH), 73.1 (CH), 72.9 (CH_2), 72.7 (CH_2), 72.5 (CH_2), 72.3 (CH_2), 70.7 (CH), 70.0 (CH), 69.8 (CH), 69.7 (CH), 69.2 (CH), 69.1 (CH), 68.8 (CH), 68.7 (CH), 68.5 (CH), 68.4 (CH), 68.1 (CH), 67.9 (CH), 67.8 (CH), 60.3 (CH), 52.76 (CH), 52.7 (CH), 52.6 (CH), 29.6 (CH_3), 23.2 (CH_3), 21.4 (CH_3), 21.0 (CH_3), 17.6 (CH_3), 17.3 (CH_3), 17.2 (CH_3), 14.1 (CH_3); HRMS m/z $(M+Na)^+$ calcd for $C_{116}H_{118}O_{37}Na^+$ 2125.7244 found 2125.7215.

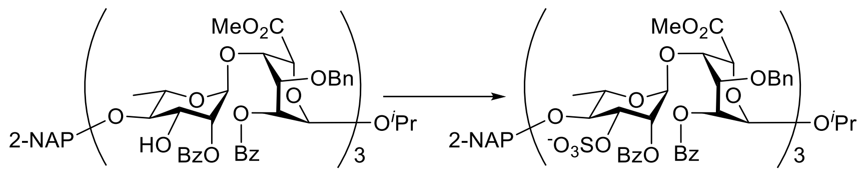

Compound 78. To a solution of compound **74** (24 mg, 11.0 μmol) in anhydrous DMF (0.5 mL), $SO_3 \cdot Et_3N$ (92 mg, 231.0 μmol) was added under nitrogen atmosphere at room temperature Then, the reaction mixture was heated to 60 °C and allowed to stir at the same temperature for 72 h. The reaction flask was cooled down to room temperature, sat. $NaHCO_{3(aq)}$ solution was added, and the mixture was allowed to stir for 12 h. The resulting mixture was extracted with THF, and the combined organic layers were washed with brine, dried over anhydrous $MgSO_4$, filtered, and concentrated *in vacuo*. The crude residue was subjected to AG 50W-X8 cation exchange column (Na^+ form) followed by Sephadex LH-20 column using MeOH as an eluent to afford the desired product **78** (25 mg, 90%) as a white solid. ^{1}H NMR (600 MHz, CD_3OD): δ = 8.09-8.00 (m, 12 H, Ar-H), 7.87-7.80 (m, 3 H, Ar-H), 7.60-7.40 (m, 16 H, Ar-H), 7.37-7.19 (m, 18 H, Ar-H), 7.16-7.02 (m, 3 H, Ar-H), 5.70 (s, 1 H, IdoA H1), 5.61 (s, 1 H, H2'), 5.55 (s, 1 H, IdoA H1), 5.48-5.43 (m, 3 H, Rha H2x2, H1), 5.36 (s, 1 H, IdoA H2), 5.17 (s, 1 H, IdoA H2), 5.11 (m, 1 H, H2), 5.02 (d, 1 H, *J* = 2.1 Hz, H1'), 4.95-4.90 (m, 3 H, H3', H5, Rha H1), 4.80-4.79 (m, 2 H, Rha H3x2), 4.73-4.66 (m, 4 H, Ar-CH_2, IdoA H5), 4.60-4.48 (m, 7H, Ar-CH_2, IdoA H5x2), 4.16 (t, 1 H, *J* = 4.6 Hz, H4), 4.03-4.02 (m, 2 H, IdoA H4x2), 4.00-3.89 (m, 11 H, ^{i}Pr, IdoA H3x3, H4', CO_2Me), 3.83 (s, 4 H, H5', CO_2Me), 3.75 (t, 1 H, *J* = 8.8 Hz, Rha H5), 3.48-3.45 (m, 3 H, Rha H4x2, Rha H5), 1.19 (d, 3 H, *J* = 6.8 Hz, ^{i}Pr), 1.12 (d, 3 H, *J* = 5.8 Hz, ^{i}Pr), 0.84 (d, 3 H, *J* = 6.8 Hz, H6'), 0.80 (d, 3 H, *J* = 5.7 Hz, Rha H6), 0.63 (d, 3 H, *J* = 5.4 Hz, Rha H6); ^{13}C NMR (150 MHz, CD_3OD): δ = 170.5 (C), 170.0 (C), 169.7 (C), 165.69 (C), 165.6 (C), 137.5 (C), 135.9 (C), 133.5 (CH), 133.2 (CH), 133.0 (CH), 132.9 (CH), 129.8 (CH), 129.64 (CH), 129.6 (CH), 129.58 (CH), 129.5 (CH), 129.4 (CH), 129.3 (CH), 128.6 (CH), 128.5 (CH), 128.4 (CH), 128.3 (CH), 128.15 (CH), 128.1 (CH), 128.0 (CH), 127.97 (CH), 127.9 (CH), 127.8 (CH), 127.7 (CH), 127.6 (CH), 127.4 (CH), 127.2 (CH), 127.1 (CH), 126.7 (CH), 125.5 (CH), 125.3 (CH), 96.8 (CH), 75.6 (CH), 71.5 (CH), 67.6 (CH), 67.4 (CH), 51.9 (CH), 51.7 (CH), 22.1 (CH_3), 20.7 (CH_3), 17.0 (CH_3), 16.8 (CH_3); HRMS m/z $(M+H)^+$ calcd for $C_{116}H_{115}O_{46}S_3H_3^-$ 2341.5984 found 2341.5867.

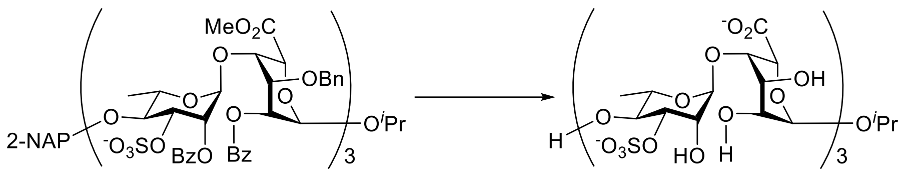

78 **7**

Compound 7. To a solution of compound **78** (92 mg, 50.7 μmol) in THF/MeOH (2.0 mL, 1/1), LiOH (1.0 mL, 0.5 M) was added at room temperature. Then, the reaction mixture was allowed to stir for 72 h. AG 50W-X8 cation exchange resin was added to quench the reaction, and pH was adjusted to 7. The whole mixture was filtered through a pad of Celite® and then concentrated *in vacuo*. The crude residue was subjected to Sephadex LH-20 column followed by AG 50W-X8 cation exchange column (Na^+ form) using MeOH as an eluent to afford the desired compound, which was used for next step. The obtained compound was dissolved in MeOH/Phosphate buffer (pH = 7) (2.0 mL, 1/1), Pd on activated charcoal (50 % Pd, 200 mg) was added, and purged with hydrogen. Then the reaction mixture was equipped with a hydrogen balloon at room temperature, and allowed to stir for 72 h. After that, the reaction mixture was filtered through a pad of Celite® and the filtrate was concentrated *in vacuo*. The crude residue was purified by using a Sephadex G-10 column followed by AG 50W-X8 cation exchange column (Na^+ form) using water as an eluent to obtain the desired product **7** (20 mg, 71%) as a white solid. ^{1}H NMR (D_2O, 600 MHz): δ = 4.96 (s, 2 H, H1”, H1””), 4.89 (d, 1 H, *J* = 4.6 Hz, H1), 4.80-4.77 (m, 3 H, H1’, H1’”, H1””’), 4.51-4.49 (m, 2 H, Rha H3x2), 4.42-4.38 (m, 3 H, H5, H5”, H5””), 4.35 (dd, 1 H, *J* = 9.3 Hz, *J* = 2.3 Hz, Rha H3), 4.12 (s, 3 H, H2’, H2’”, H2””’), 3.97-3.85 (m, 7 H, H4, H4”, H4””, H5’, H5’”, H5””’, ^{i}Pr), 3.73-3.67 (m, 5 H, H3, H3”, H3””, Rha H4x2), 3.57-3.53 (m, 2 H, H2”, H2””), 3.46 (t, 1 H, J = 10.1 Hz, Rha H4), 3.34 (m, 1 H, H2), 1.20 (d, 3 H, J = 6.0 Hz, Rha H6), 1.14-1.09 (m, 12 H, ^{i}Pr, Rha H6 x2); ^{13}C NMR (D_2O, 150 MHz): δ = 174.8 (C), 174.2 (C), 103.0 (CH), 101.2 (CH), 101.1 (CH), 100.5 (CH), 99.2 (CH), 81.6 (CH), 79.5 (CH), 78.7 (CH), 78.5 (CH), 78.4 (CH), 75.7 (CH), 71.8 (CH), 71.7 (CH), 71.6 (CH), 71.3 (CH), 71.2 (CH), 71.0 (CH), 70.9 (CH), 70.7 (CH), 70.6 (CH), 69.9 (CH), 68.8 (CH), 68.6 (CH), 67.7 (CH), 22.4 (CH), 20.8 (CH), 16.7 (CH), 16.6 (CH), 16.5 (CH); HRMS m/z $(M+H)^+$ calcd for $C_{39}H_{56}O_{40}S_3H_6^{2-}$ 632.0917 found 632.0915.

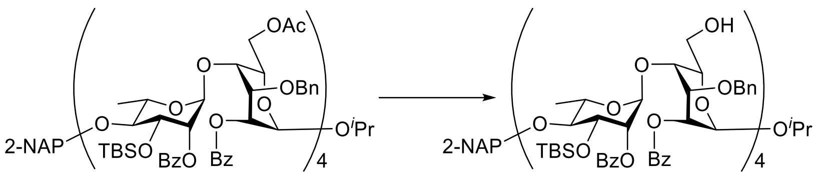

Compound 67. To a solution of compound **63** (300 mg, 66.0 μmol) in CH_2Cl_2, $Mg(OMe)_2$ (1.0 mL, 7-8% in MeOH) was added at 0 °C under nitrogen atmosphere, and the reaction mixture was allowed to stir at same temperature for 24 h. Then, 1 N HCl was added to neutralize the reaction and washed with H_2O. Organic layer was dried over anhydrous $MgSO_4$, filtered, and concentrated *in vacuo*. The resulting crude residue was purified by flash column chromatography to afford the desired product **67** (250 mg, 88%) as a white solid. $[\alpha]_D^{28}$ = -41.5 (*c* = 1.0, $CHCl_3$); IR (thin film in KBr) ν 3529, 2930, 2857, 1719, 1602, 1452, 1385, 1316, 1110, 1070, 1027, 977, 858, 838, 778, 752, 711; ^1H NMR ($CDCl_3$, 600 MHz): δ = 8.06-7.98 (m, 16 H, Ar-H), 7.89-7.82 (m, 3 H, Ar-H), 7.63-7.58 (m, 9 H, Ar-H), 7.54-7.36 (m, 30 H, Ar-H), 7.30-7.22 (m, 15 H, Ar-H), 5.57 (s, 1 H, IdoA H1), 5.45 (s, 2 H, IdoA H1x2), 5.23-5.20 (m, 2 H, H2, Rha H2), 5.15-5.12 (m, 4 H, IdoA H2, Rha H2x3), 5.08 (d, 1 H, *J* = 1.9 Hz, H1), 5.00 (s, 1 H, IdoA H2), 4.98 (s, 2 H, IdoA H2, Rha H1), 4.87-4.82 (m, 5 H, Rha H1 x3, Ar-CH_2), 4.77-4.57 (m, 9 H, Ar-CH_2), 4.42 (m, 1 H, H5), 4.30-4.26 (m, 2 H, IdoA H5, Rha H3), 4.19-4.18 (m, 2 H, IdoA H5x2), 4.06-3.89 (m, 18 H, IdoA H3x4, IdoA H6x4, H4, Rha H3x2, ^{i}Pr), 3.83-3.73 (m, 7 H, IdoA H4x3, Rha H4x3, Rha H5), 3.53 (m, 2 H, Rha, H5x2), 3.46 (m, 1 H, Rha H5), 3.31 (t, 1 H, J = 9.1 Hz, Rha H4), 2.25 (s, 4 H, OHx4), 1.29 (d, 3 H, J = 6.6 Hz, ^{i}Pr), 1.20 (d, 3 H, J = 6.6 Hz, ^{i}Pr), 1.08 (d, 3 H, J = 6.6 Hz, Rha H6), 0.92-0.90 (m, 9 H, Rha H6x3), 0.80 (s, 9 H, ^{t}Bu), 0.62-0.59 (m, 27 H, ^{t}Bu), 0.06-(-0.08) (m, 24 H, CH_3); ^{13}C NMR ($CDCl_3$, 150 MHz): δ = 166.5 (C), 165.63 (C), 165.6 (C), 165.49 (C), 165.4 (C), 137.8 (C), 137.7 (C), 137.6 (C), 137.5 (C), 135.9 (C), 133.6 (CH), 133.56 (CH), 133.5 (CH), 133.4 (CH), 133.3 (CH), 133.2 (CH), 133.1 (CH), 132.8 (CH), 129.8 (CH), 129.7 (CH), 129.6 (CH), 129.5 (CH), 129.4 (CH), 129.3 (CH), 128.6 (CH), 128.49 (CH), 128.46 (CH), 128.44 (CH), 128.4 (CH), 128.34 (CH), 128.3 (CH), 127.9 (CH), 127.87 (CH), 127.8 (CH), 127.76 (CH), 127.7 (CH), 127.6 (CH), 126.2 (CH), 126.0 (CH), 125.8 (CH), 100.9 (CH), 100.3 (CH), 100.2 (CH), 99.9 (CH), 98.5 (CH), 98.4 (CH), 98.32 (CH), 96.3 (CH), 80.9 (CH), 76.1 (CH), 75.4 (CH), 75.3 (CH), 75.2 (CH), 74.8 (CH_2), 73.8 (CH), 73.75 (CH), 73.7 (CH), 73.3 (CH), 72.07 (CH_2), 72.04 (CH_2), 72.0 (CH_2), 71.8 (CH_2), 71.6 (CH), 70.8 (CH), 69.8 (CH), 68.3 (CH), 68.2 (CH), 68.1 (CH), 67.5 (CH), 66.5 (CH), 66.4 (CH), 61.5 (CH), 61.4 (CH), 25.6 (CH_3), 25.45 (CH_3), 25.42 (CH_3),

23.3 (CH_3), 21.3 (CH_3), 18.2 (CH_3), 17.9 (CH_3), 17.7 (CH_3), 17.54 (CH_3), 17.5 (CH_3), 17.4 (CH_3), -4.1 (CH_3), -4.2 (CH_3), -4.62 (CH_3), -4.68 (CH_3), -4.8 (CH_3), -4.96 (CH_3), -4.98 (CH_3); HRMS m/z $(M+Na)^+$ calcd for $C_{170}H_{209}O_{45}Si_4Na^{2+}$ 1552.6515 found 1552.6452.

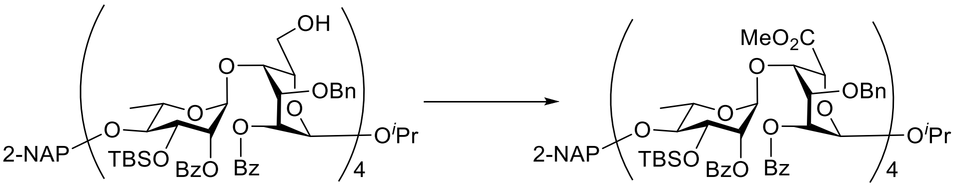

67 **71**

Compound 71. To a solution of compound **67** (1.7 g, 0.564 mmol) in a mixed solvent CH_2Cl_2/H_2O (52.5 mL, 1/2), TEMPO (32 mg, 225 μmol) and BAIB (1.8 g, 5.64 mmol) were added at 0 °C, and the reaction mixture was allowed to stir at room temperature for 24 h. Then, the reaction mixture was diluted with EtOAc, washed with 10% $Na_2S_2O_3$ and brine. The organic layer was dried over anhydrous $MgSO_4$, filtered, and concentrated *in vacuo* to afford the crude product, which was directly used for the next reaction without further purification. Freshly prepared CH_2N_2 (22.56 mmol in Et_2O) was added to solution of crude acid (58.0 μmol) in THF (17.5 mL) at 0 °C, and the reaction mixture was allowed to stir at room temperature for 0.5 h. After that AcOH was added to quench the excess CH_2N_2, reaction mixture was diluted with EtOAc, washed with sat. $NaHCO_{3(aq)}$ and brine. The organic layer was dried over anhydrous $MgSO_4$, filtered, and concentrated *in vacuo*. The crude residue was purified by flash column chromatography to afford the desired product **71** (1.42 g, 78%) as a white solid. $[\alpha]_D^{28.4}$ = -59.5 (c = 1.0, $CHCl_3$); IR (thin film in KBr) ν 2953, 2930, 2857, 1767, 1721, 1602, 1584, 1494, 1452, 1385, 1361, 1316, 1212, 1176, 1146, 1110, 1070, 1028, 980, 933, 838, 778, 753, 711; ^1H NMR ($CDCl_3$, 600 MHz): δ = 8.07-7.93 (m, 16 H, Ar-H), 7.88-7.79 (m, 3 H, Ar-H), 7.62-7.57 (m, 5 H, Ar-H), 7.54-7.49 (m, 18 H, Ar-H), 7.41-7.37 (m, 6 H, Ar-H), 7.31-7.11 (m, 19 H, Ar-H), 5.63 (s, 1 H, IdoA H1), 5.47-5.45 (m, 2 H, IdoA H1 x2), 5.38 (d, 1 H, J = 2.4 Hz, H1), 5.19-5.11 (m, 3 H, IdoA H2 x2, Rha H2), 5.03-5.00 (m, 5 H, IdoA H2 x2, Rha H2 x3), 4.92-0.89 (m, 4 H, H5, Ar-CH_2), 4.84-4.64 (m, 11 H, Rha H1x4, IdoA H5,Ar-CH_2), 4.52-4.49 (m, 3 H, IdoA H5x2, Ar-CH_2), 4.21 (dd, 1 H, *J* = 9.3 Hz, *J* = 2.4 Hz, Rha H3), 4.16 (m, 1 H, H4), 4.05-3.93 (m, 21 H, IdoA H4x3, IdoA H3x3, H3, CO_2Me, Rha H3, ^{i}Pr), 3.86-3.79 (m, 2 H, Rha H3x3), 3.71-3.67 (m, 2 H, Rha H4, Rha H5), 3.46 (m, 2 H, Rha H5x2), 3.35 (m, 1 H, Rha H5), 3.25 (t, 1 H, J = 8.6 Hz, Rha H4), 1.25 (d, 3 H, J = 5.6 Hz, ^{i}Pr), 1.17 (d, 3 H, J = 5.6 Hz, ^{i}Pr), 0.89 (d, 3 H, J = 6.3 Hz, Rha H6), 0.79-0.76 (m, 12 H, Rha H6, ^{t}Bu), 0.49-0.47 (m, 18

H, ^{t}Bu), 0.08-(0.06) (m, 24 H, CH_3); ^{13}C NMR ($CDCl_3$, 150 MHz): δ = 170.0 (C), 169.2 (C), 169.1 (C), 169.0 (C), 165.88 (C), 165.83 (C), 165.8 (C), 165.7 (C), 165.6 (C), 165.5 (C), 165.4 (C), 137.5 (C), 137.4 (C), 137.3 (C), 137.2 (C), 135.9 (C), 133.6 (CH), 133.4 (CH), 133.2 (CH), 133.1 (CH), 132.8 (CH), 130.0 (CH), 129.9 (CH), 129.8 (CH), 129.79 (CH), 129.74 (CH), 129.65 (CH), 129.60 (CH), 129.5 (CH), 129.4 (CH), 129.15 (CH), 129.11 (CH), 128.5 (CH), 128.47 (CH), 128.43 (CH), 128.4 (CH), 128.3 (CH), 128.2 (CH), 128.15 (CH), 128.1 (CH), 128.0 (CH), 127.9 (CH), 127.86 (CH), 127.8 (CH), 127.7 (CH), 127.69 (CH), 127.6 (CH), 126.4 (CH), 126.2 (CH), 126.0 (CH), 125.7 (CH), 101.6 (CH), 101.0 (CH), 100.5 (CH), 100.2 (CH), 99.6 (CH), 96.8 (CH), 80.7 (CH), 76.1 (CH), 75.2 (CH), 74.9 (CH_2), 73.5 (CH), 73.4 (CH), 73.3 (CH), 73.09 (CH), 73.0 (CH), 72.9 (CH), 72.7 (CH_2), 72.6 (CH_2), 72.3 (CH_2), 71.3 (CH), 70.9 (CH), 70.8 (CH), 70.7 (CH), 70.6 (CH), 70.0 (CH), 68.8 (CH), 68.3 (CH), 68.1 (CH), 67.9 (CH), 67.8 (CH), 67.5 (CH), 67.4 (CH), 52.6 (CH), 52.5 (CH), 52.4 (CH), 29.7 (CH_3), 25.6 (CH_3), 25.4 (CH_3), 25.3 (CH_3), 25.2 (CH_3), 23.2 (CH_3), 21.5 (CH_3), 18.0 (CH_3), 17.8 (CH_3), 17.7 (CH_3), 17.6 (CH_3), 17.4 (CH_3), 17.34 (CH_3), 17.3 (CH_3), 17.2 (CH_3), -4.1 (CH_3), -4.2 (CH_3), -4.6 (CH_3); HRMS m/z $(M+H)^+$ calcd for $C_{174}H_{209}O_{49}Si_4^+$ 3196.2989 found 3196.2975.

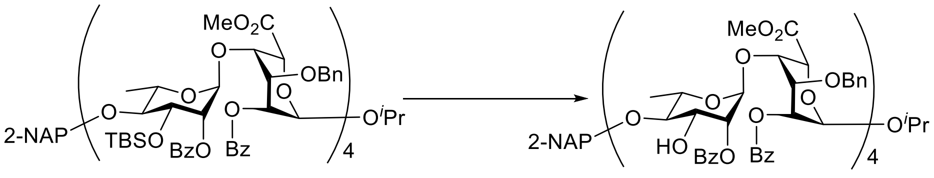

71 **75**

Compound 75. To a solution of compound **71** (89 mg, 27.0 μmol) in ACN (1.0 mL), PMA (8.1 mg, 5.4 μmol) was added. Then, the reaction mixture was allowed to stir at room temperature for 24 h, after that the reaction was diluted with EtOAc and washed with sat. $NaHCO_{3(aq)}$ and brine. The organic layer was dried over anhydrous $MgSO_4$, filtered, and concentrated *in vacuo*. The crude residue was purified by flash column chromatography to afford the desired product **75** (47 mg, 61%) as a white solid. $[\alpha]_D^{28}$ = -42.6 (*c* = 0.5, $CHCl_3$); IR (thin film in KBr) ν 3517, 2933, 1720, 1601, 1452, 1111, 1070, 1028, 711 cm^{-1}; ^{1}H NMR (600 MHz, $CDCl_3$) δ = 8.14-7.97 (m, 16 H, Ar-H), 7.87-7.79 (m, 3 H, Ar-H), 7.67 (s, 1 H, Ar-H), 7.61-7.59 (m, 4 H, Ar-H), 7.53-7.42 (m, 13 H, Ar-H), 7.38-7.33 (m, 9 H, Ar-H), 7.30-7.16 (m, 21 H, Ar-H), 5.50 (d, 1 H, *J* = 3.1 Hz, IdoA H1), 5.44 (m, 2 H, IdoA H1x2), 5.32 (d, 1 H, J = 2.3 Hz, IdoA H1), 5.19-5.15 (m, 5 H, IdoA H2, Rha H2x4), 5.04-5.02 (m, 3 H, IdoA H2x3), 4.94-4.90 (m, 5 H, IdoA H5,

Rha H1x4), 4.87-4.80 (m, 2 H, Ar-CH_2), 4.74-4.62 (m, 11 H, IdoA H5 x 3, Ar-CH_2), 4.17 (s, 1 H, IdoA H4), 4.14-4.12 (m, 3 H, IdoA H4x3), 4.08-4.03 (m, 5 H, Rha H3x4, *ⁱ*Pr), 3.97-3.96 (m, 12 H, CO_2Me), 3.89-3.86 (m, 2 H, IdoA H3, Rha H4), 3.84-3.83 (m, 3 H, IdoA H3x3), 3.81-3.73 (m, 3 H, Rha H4x2, Rha H5), 3.66-3.62 (m, 3 H, Rha H5x3), 3.41 (t, 1 H, J = 9.5 Hz, Rha H4), 3.10 (d, 1 H, J = 6.5 Hz, OH), 3.04 (d, 1 H, J = 6.5 Hz, OH), 3.03 (d, 1 H, J = 5.2 Hz, OH), 2.11 (d, 1 H, J = 5.2 Hz, OH), 1.26 (d, 3 H, J = 5.6 Hz, ^{i}Pr), 1.19 (d, 3 H, J = 6.2 Hz, ^{i}Pr), 1.04 (d, 3 H, J = 5.6 Hz, Rha H6), 0.96-0.93 (m, 9 H, Rha H6x3); ^{13}C NMR ($CDCl_3$, 150 MHz): δ = 170.1 (C), 169.5 (C), 169.4 (C), 169.3 (C), 165.9 (C), 165.8 (C), 165.65 (C), 165.6 (C), 165.5 (C), 137.6 (C), 136.7 (C), 135.4 (C), 133.6 (CH), 133.5 (CH), 133.4 (CH), 133.3 (CH), 133.1 (CH), 133.0 (CH), 130.0 (CH), 129.9 (CH), 129.8 (CH), 129.7 (CH), 129.54 (CH), 129.50 (CH), 129.3 (CH), 129.05 (CH), 129.01 (CH), 128.8 (CH), 128.56 (CH), 128.5 (CH), 128.4 (CH), 128.3 (CH), 128.2 (CH), 128.19 (CH), 128.1 (CH), 128.0 (CH), 127.8 (CH), 127.7 (CH), 127.0 (CH), 126.2(CH), 126.1 (CH), 100.2 (CH), 100.0 (CH), 99.9 (CH), 99.5 (CH), 98.9 (CH), 98.8 (CH), 98.7 (CH), 96.8 (CH), 80.9 (CH), 78.4 (CH), 78.3 (CH), 78.2 (CH), 75.3 (CH), 75.1 (CH_2), 75.0 (CH), 74.9 (CH), 74.8 (CH), 74.7 (CH), 74.6 (CH), 74.5 (CH), 73.2 (CH), 73.1 (CH), 72.9 (CH), 72.7 (CH_2), 72.6 (CH_2), 72.3 (CH_2), 70.7 (CH), 70.0 (CH), 69.8 (CH), 69.7 (CH), 69.2 (CH), 68.8 (CH), 68.7 (CH), 68.5 (CH), 68.4 (CH), 68.1 (CH), 67.9 (CH), 67.8 (CH), 52.7 (CH), 52.6 (CH), 23.2 (CH_3), 21.4 (CH_3), 17.6 (CH_3), 17.2 (CH_3); HRMS m/z $(M+H)^+$ calcd for $C_{150}H_{153}O_{49}^+$ 2738.9509 found 2738.9477.

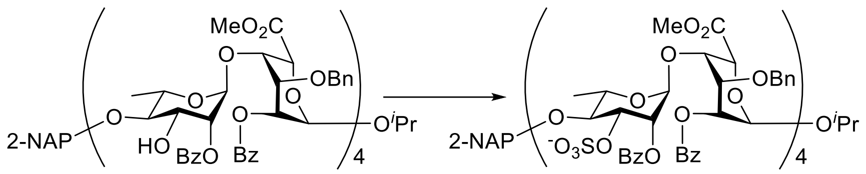

75 79

Compound 79. To a solution of compound **75** (221 mg, 77.0 μmol) in anhydrous DMF (4.2 mL), $SO_3{\cdot}Et_3N$ (539 mg, 3.08 mmol) was added under nitrogen atmosphere at room temperature. Then, the reaction mixture was heated to 60 °C and allowed to stir at same temperature for 72 h. The reaction flask was cooled down to room temperature, sat. $NaHCO_{3(aq)}$ solution was added, and the mixture was allowed to stir for 12 h. The resulting mixture was extracted with THF, and the combined organic layers were washed with brine, dried over anhydrous $MgSO_4$, filtered, and concentrated *in vacuo*. The crude residue was subjected to AG 50W-X8 cation exchange column (Na^+ form) followed by Sephadex LH-20 column using

MeOH as an eluent to afford the desired product **79** (71%), which was directly used for next step.

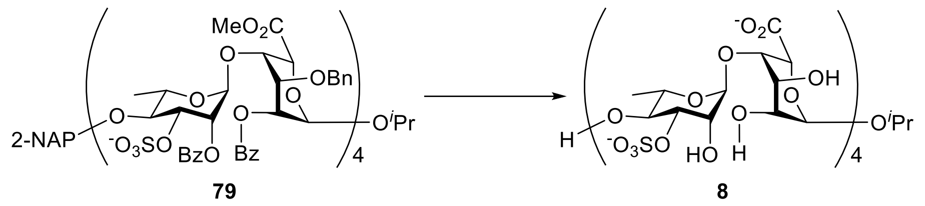

Compound 8. The compound **79** (89 mg, 49.0 µmol) was dissolved in THF/MeOH (2.0 mL, 1/1), LiOH (1.0 mL, 0.5 M) was added. The reaction mixture was allowed to stir at room temperature for 72 h. Then, the reaction was concentrated *in vacuo*. The crude residue was subjected to AG 50W-X8 cation exchange column (Na^+ form) followed by Sephadex LH-20 column using MeOH as an eluent to afford the desired product **79a** as a white solid. ^{1}H NMR (600 MHz, D_2O) δ = 7.87 (m, 4 H, Ar-H), 7.52-7.48 (m, 3 H, Ar-H), 7.33-7.24 (m, 20 H, Ar-H), 5.02 (m, 4 H, IdoA H1x4), 4.90 (ABq, 1 H, *J* = 10.8 Hz, Ar-CH_2), 4.79-4.76 (m, 5 H, Rha H1x4, Ar-CH_2), 4.57-4.46 (m, 12 H, Rha H3x4, IdoA H5x4), 4.18 (m, 4 H, Rha H2x2), 4.06-4.05 (m, 3 H, IdoA H4x3), 3.99 (s, 1 H, IdoA H4), 3.97-3.90 (m, 4 H, IdoA H2x3, ^{i}Pr), 3.77-3.66 (m, 5 H, Rha H5 x 4, IdoA H3), 3.61-3.58 (m, 6 H, IdoA H3x3, Rha H4x3), 3.53-3.48 (m, 2 H, IdoA H2, Rha H4); ^{13}C NMR (D_2O, 150 MHz): δ = 174.6 (C), 174.4 (C), 157.9 (C), 137.1 (C), 137.06 (C), 137.0 (C), 134.9 (CH), 132.9 (CH), 132.8 (CH), 130.4 (CH), 128.6 (CH), 128.4 (CH), 128.2 (CH), 128.1 (CH), 128.0 (CH), 127.9 (CH), 127.6 (CH), 127.5 (CH), 127.2 (CH), 127.1 (CH), 126.5 (CH), 126.3 (CH), 102.8 (CH), 102.6 (CH), 101.6 (CH), 101.5 (CH), 100.9 (CH), 100.1 (CH), 99.9 (CH), 98.9 (CH), 98.7 (CH), 78.6 (CH), 78.1 (CH), 77.7 (CH), 77.5 (CH), 77.4 (CH), 76.2 (CH), 75.7 (CH), 75.5 (CH), 75.2 (CH), 75.0 (CH), 74.6 (CH), 74.4 (CH), 74.2 (CH_2), 73.3 (CH), 72.1 (CH_2), 71.7 (CH), 71.5 (CH), 69.9 (CH), 69.0 (CH), 68.8 (CH), 68.7 (CH), 68.6 (CH), 68.5 (CH), 68.2 (CH), 67.9 (CH), 67.8 (CH), 66.4 (CH), 66.2 (CH), 22.3 (CH_3), 22.2 (CH_3), 20.8 (CH_3), 20.7 (CH_3), 20.6 (CH_3), 16.94 (CH_3), 16.9 (CH_3), 16.7 (CH_3); HRMS m/z $(M+2Na)^-$ calcd for $C_{90}H_{108}O_{53}S_4Na_2^{2-}$ 1105.2222 found 1105.2226. To a solution of deacylated compound (11 mg, 1.9 µmol) in MeOH/Phosphate buffer (pH = 7) (2.0 mL, 1/1), Pd on activated charcoal (50 % Pd, 22 mg) was added. The reaction mixture was degassed several times with hydrogen gas, and then allowed to stir under hydrogen atmosphere for 72 h. Then, the reaction mixture was filtered through a pad of Celite®, filtrate was concentrated under reduced pressure. The crude residue was subjected to Sephadex G-10

column followed by AG 50W-X8 cation exchange column (Na^+ form) using water as an eluent to afford the desired product **8** (5.5 mg, 60%) as a white solid. ^1H NMR (D_2O, 600 MHz): δ = 4.96-4.95 (m, 3 H, IdoA H1 x 3), 4.89 (d, 1 H, *J* = 5.1 Hz, IdoA H1), 4.51-4.49 (m, 3 H, Rha H3x3), 4.42-4.40 (m, 3 H, IdoA H5x3), 4.38 (d, 1 H, *J* = 3.9 Hz, IdoA H5), 4.35 (dd, 1 H, *J* = 9.4, *J* = 3.0 Hz, Rha H3), 4.12 (m, 4 H, Rha H2x4), 3.97-3.92 (m, 5 H, Rha H5x4, ^{i}Pr), 3.90-3.84 (m, 4 H, IdoA H4x4), 3.73-3.67 (m, 7 H, IdoA H3x4, Rha H4x3), 3.57-3.53 (m, 3 H, IdoA H2x2), 3.46 (t, 1 H, J = 10.1 Hz, Rha H4), 3.34 (m, 1 H, IdoA H2), 1.20 (d, 3 H, *J* = 5.9 Hz, Rha H6), 1.14-1.09 (m, 12 H, Rha H6x3, ^{i}Pr); ^{13}C NMR (D_2O, 150 MHz): δ = 174.8 (C), 174.3 (C), 103.08 (CH), 103.03 (CH), 101.2 (CH), 101.1 (CH), 101.0 (CH), 100.5 (CH), 99.2 (CH), 79.6 (CH), 79.5 (CH), 79.4 (CH), 78.7 (CH), 78.5 (CH), 78.4 (CH), 75.6 (CH), 75.5 (CH), 71.8 (CH), 71.78 (CH), 71.70 (CH), 71.3 (CH), 71.2 (CH), 71.19 (CH), 71.12 (CH), 71.0 (CH), 70.79 (CH), 70.70 (CH), 70.0 (CH), 68.8 (CH), 68.6 (CH), 67.77 (CH), 67.71 (CH), 22.4 (CH_3), 20.8 (CH_3), 16.7 (CH_3), 16.6 (CH_3); HRMS m/z $(M+2Na)^-$ calcd $C_{51}H_{76}O_{53}S_4Na_2^{2-}$ 855.0970 found 855.0977.

4. Synthetic Procedures and Characterization data for U_3's.

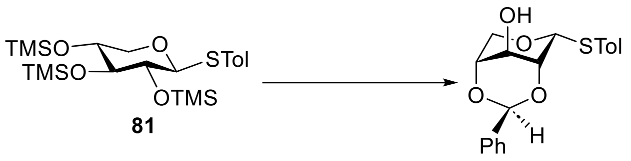

Compound 82. To a solution of compound **81** (0.82 g, 1.73 mmol) and PhCHO (184.5 μL, 1.81 mmol) in anhydrous CH_2Cl_2 (8.0 ml), freshly activated 3 Å molecular sieves (0.8 g) were added and allowed to stir at RT for 0.5 h under a nitrogen atmosphere. The reaction mixture was placed in an ice bath, then TMSOTf (46.9 μL, 0.259 mmol, 0.15 equiv) was added drop by drop. After 4 h of stirring in an ice bath, TBAF (1 M solution in THF, 1.73 mL, 1.73 mmol, 1.0 equiv) was added to the mixture, and the reaction flask was gradually warmed up to room temperature, then the solution was kept stirring 1 h. The whole mixture was filtered through a pad of Celite®, the solvent was removed under reduced pressure and the residue was extracted with EtOAc (100 mL × 2), and the combined organic layers were washed with brine, dried over $MgSO_4$, filtered, and concentrated *in vacuo*. The residue was purified by flash column chromatography (EtOAc/Hex = 1/7) on silica gel to get the compound **82** (0.482 g, 81%) as white solid. Crystals of compound **82** were used for X-ray crystallography analysis (Figure S1, CCDC 2363767). $[\alpha]_D^{26}$ = -67.0 (*c* = 0.88, $CHCl_3$); IR (thin film in KBr) ν 3367, 2920, 1725,

1492, 1264, 1102, 1046, 802, 711 ; ^{1}H NMR ($CDCl_3$, 600 MHz): 7.45-7.44 (m, 2 H, Ar-H), 7.41 (d, J = 7.8 Hz, 2H, Ar-H), 7.35-7.34 (m, 3H, Ar-H), 7.13 (d, J = 7.7 Hz, 2H, Ar-H), 6.98 (s, 1H, CHPh), 5.74 (s, 1H, H-1), 4.65, 4.15 (ABq, J = 12.8 Hz, 2 H, H-5), 4.37 (s, 1 H, H-3), 4.33 (s, 1H, H-2), 3.96 (s, 1 H, H-4), 2.74 (s, 1 H, OH), 2.33 (s, 3 H, Ar-CH_3) ; ^{13}C NMR ($CDCl_3$, 150 MHz): 138.7 (C), 138.3 (C), 132.7 (2C, CH), 131.7 (C), 130.1 (2C, CH), 129.4 (CH), 128.6 (2C, CH), 126.5 (2C, CH), 96.4 (CH), 87.2 (CH), 72.7 (CH), 70.4 (CH), 64.1 (CH), 61.0 (CH_2), 21.3 (CH_3); HRMS: *m/z* calcd for $C_{19}H_{20}O_4SH^+$ 345.1155, found 345.1164.

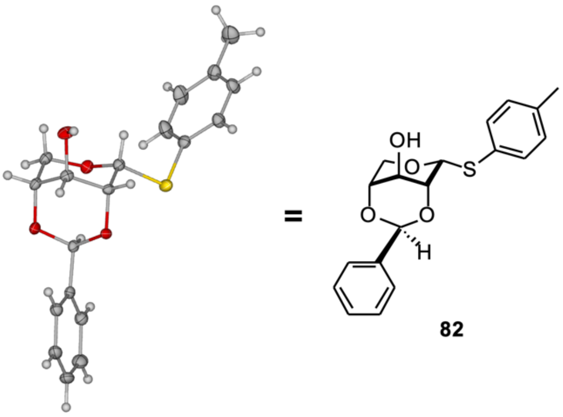

Figure S1. Crystal structure of compound **82** (CCDC 2363767).

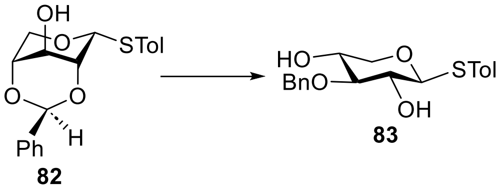

Compound 83. To a solution of compound **82** (57.9 mg, 0.168 mmol) and BnBr (26.1 mg, 0.218 mmol) in anhydrous DMF (0.6 mL), NaH (5.0 mg, 0.336 mmol) was added under ice bath. After stirring for 1 h, the reaction was quenched with MeOH. CSA was to bring the *p*H near 2. The whole mixture was then stirred for 1 h. The solvents were removed under reduced pressure and the residue was extracted with EtOAc (2 x 3 mL), and the combined organic layers were washed with brine, dried over $MgSO_4$, filtered, and concentrated *in vacuo*. The residue was purified by flash column chromatography (EtOAc/Hex = 1/4) on silica gel to afford the desired compound **83** (48.3 mg, 83%) as white solid.

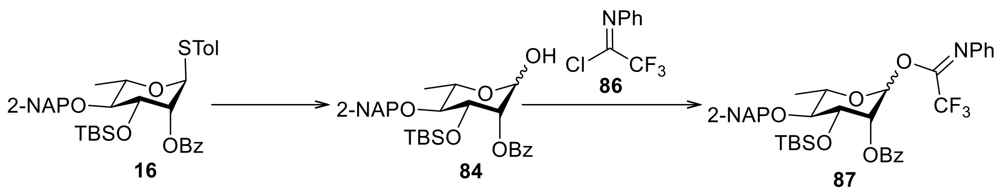

Compound 87. To a solution of compound **16** (20.8 g, 33.0 mmol) in a mixed solvent of acetone/H_2O (200.0 mL, 9/1), *N*-bromosuccinimide (17.6 g, 99.1 mmol) was added at 0 °C, and the reaction mixture was allowed to stir for 1 h, MeOH was added to quench the reaction and concentrated *in vacuo*. The resulting residue was diluted with CH_2Cl_2 and washed with saturated $NaHCO_{3(aq)}$. The organic layer was dried over anhydrous $MgSO_4$, filtered, and concentrated *in vacuo*. The resulting crude residue was purified by flash column chromatography (EtOAc/Hex =1/4) on silica gel to afford the desired compound (16.6 g, 96%) as yellow syrup. Anomeric lactol (16.6 g, 31.66 mmol) was dissolved in acetone (170.0 mL), 2,2,2-trifluoro-*N*-phenylacetimidoyl chloride (15.4 mL, 94.99 mmol) and cesium carbonate (30.9 g, 94.99 mmol) were added. Then, the reaction mixture was allowed to stir for 0.5 h and filtered through a pad of Celite®. Filtrate was concentrated *in vacuo* to obtain the crude compound **87** as a yellow syrup, which was directly used for next reactions without further purification. HRMS *m/z* $(M+Na)^+$ calcd for $C_{38}H_{42}F_3NO_6SiNa^+$ 716.2626, found 716.2602.

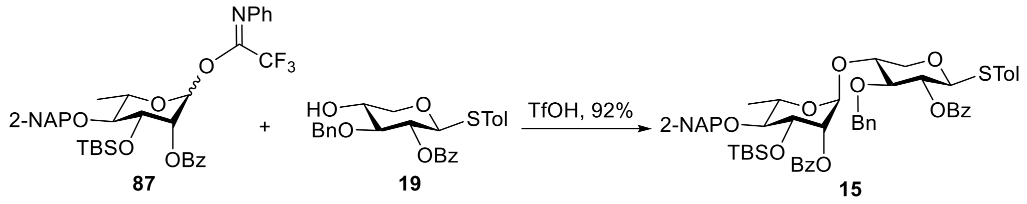

Compound 15. To a solution of donor **87** (1.4 g, 2.03 mmol), and acceptor **19** (830 mg, 1.85 mmol) in anhydrous CH_2Cl_2 (25mL), freshly activated 3 Å molecular sieves (2.2 g) were added and allowed to stir at room temperature for 0.5 h under a nitrogen atmosphere. Then, the reaction mixture was cooled to 0 °C, TfOH (24.5 μL, 0.278 mmol) was added, and allowed to stir at same temperature for 3 h. After completion of the reaction as indicated by TLC, Et_3N was added to quench the reaction. The whole mixture was filtered through a pad of Celite® and solids were washed with CH_2Cl_2. The filtrate was sequentially washed with 10% $Na_2S_2O_3$, saturated $NaHCO_{3(aq)}$ and H_2O. The organic layer was dried over anhydrous $MgSO_4$, filtered, and concentrated *in vacuo*. The crude residue was purified by flash column chromatography (EtOAc/Hex = 1/9) on silica gel to afford the desired product **15** (1.83 g, 92%) as a white solid.

$[\alpha]_D^{27}$ = -32.8 (*c* = 0.4, $CHCl_3$); IR (thin film in KBr) ν 2929, 2857, 1725, 1601, 1493, 1451, 1315, 1268, 1108, 1069, 977, 838, 753, 710, 476 cm^{-1}; ^1H NMR ($CDCl_3$, 600 MHz) δ = 8.07-8.04 (m, 4 H, Ar-H), 7.83-7.73 (m, 4 H, Ar-H), 7.60-7.54 (m, 2 H, Ar-H), 7.48-7.40 (m, 7 H, Ar-H), 7.35-7.34 (m, 2 H, Ar-H), 7.16-7.15 (m, 2 H, Ar-H), 7.08-6.99 (m, 5 H, Ar-H), 5.25 (s, 1 H, H-2'), 5.23-5.20 (m, 1H, H-2), 5.04 (ABq, *J* = 11.5 Hz, 1 H, $ArCH_2$), 4.95 (s, 1 H, H-1'), 4.81-4.78 (m, 3 H, H-1, $ArCH_2$), 4.64 (ABq, *J* = 10.6 Hz, 1 H, $ArCH_2$), 4.33-4.31 (m, 1 H, H-5a), 4.27-4.25 (m, 1 H, H-3'), 3.97-3.95 (m, 2 H, H-4, H-5'), 3.80-3.77 (m, 1 H, H-3), 3.52 (t, *J* = 9.2 Hz, 1 H, H-4'), 3.44 (t, *J* = 9.6 Hz, 1 H, H-5b), 2.30 (s, 3H, Ar-CH_3), 1.21 (d, *J* = 4.4 Hz, 3 H, H-6'), 0.79 (s, 9 H, TBS-*t*Bu), 0.06 (s, 3H, TBS-CH_3), 0.01 (s, 3H, TBS-CH_3); ^{13}C NMR ($CDCl_3$, 150 MHz) δ = 166.2 (C), 165.3 (C), 138.4 (C), 137.3 (C), 136.1 (C), 133.5 (2C, CH), 133.4 (3C, C/2×CH), 133.0 (C), 130.1 (C), 130.07 (2C, CH), 130.00 (2C, CH), 129.8 (2C, CH), 129.1 (C), 128.7 (2C, CH), 128.6 (2C, CH), 128.5 (3C, CH), 128.4 (2C, C/CH), 128.1 (CH), 128.0 (CH), 127.9 (CH), 127.8 (CH), 126.3 (CH), 126.2 (CH), 125.9 (2C, CH), 95.0 (CH), 87.3 (CH), 81.4 (CH), 80.8 (CH), 75.5 (2C, CH_2), 73.7 (CH), 72.3 (CH), 71.9 (CH), 71.4 (CH), 68.6 (CH), 65.6 (CH_2), 25.9 (3C, CH_3), 21.3 (CH_3), 18.3 (CH_3), 17.9 (C), -4.4 (CH_3), -4.5 (CH_3); HRMS *m/z* $(M+H)^+$ calcd for $C_{19}H_{20}O_4SH^+$ 345.1155, found 345.1164.

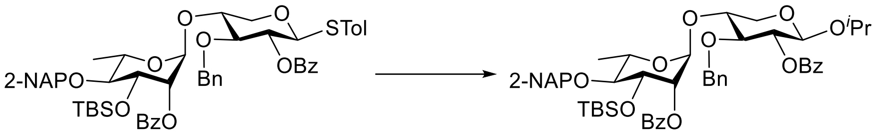

Compound 88. To a solution of donor **15** (538 mg, 563.0 μmol), and acceptor (anhydrous ^{i}PrOH, 129 μL, 1.7 mmol) in CH_2Cl_2 (5.0 mL), freshly activated AW-500 molecular sieves (540 mg) were added and allowed to stir at room temperature for 0.5 h under a nitrogen atmosphere. Then, the reaction flask was cooled to 0 °C, NIS (150 mg, 68.0 μmol), TfOH (7.5 *μ*L, 85.0 μmol) were added, and the reaction mixture was allowed to stir at the same temperature for 3 h. Et_3N was added to quench the reaction, the whole mixture was filtered through a pad of Celite® and the solids were washed with CH_2Cl_2. The filtrate was washed with 10% $Na_2S_2O_3$ and sat. $NaHCO_{3(aq)}$, organic layer was dried over anhydrous $MgSO_4$, filtered, and concentrated *in vacuo*. The crude residue was purified by flash column chromatography (EtOAc/Hex = 1/13) on silica gel to afford the desired compound **88** (328 mg, 85%) as a white solid. $[\alpha]_D^{26}$ = -6.8 (*c* = 1.9, $CHCl_3$); IR (thin film in KBr) ν 2927, 2855, 1726, 1451, 1267, 1106, 1071, 871, 710 cm^{-1}; ^1H NMR ($CDCl_3$, 600 MHz) δ = 8.07-8.05 (m,

2 H, Ar-H), 8.00-7.98 (m, 2 H, Ar-H), 7.83-7.76 (m, 3 H, Ar-H), 7.73 (s, 1 H, Ar-H), 7.60-7.53 (m, 2 H, Ar-H), 7.48-7.40 (m, 7 H, Ar-H), 7.15-7.13 (m, 2 H, Ar-H), 7.02-7.00 (m, 2 H, Ar-H), 6.97-6.95 (m, 2 H, Ar-H), 5.28 (s, 1 H, H-2'), 5.17-5.14 (m, 1H, H-2), 5.04 (ABq, *J* = 11.6 Hz, 1 H, $ArCH_2$), 4.95 (s, 1 H, H-1'), 4.81-4.78 (m, 2 H, $ArCH_2$), 4.61 (ABq, *J* = 10.8 Hz, 1 H, $ArCH_2$), 4.55 (d, *J* = 7.5 Hz, 1 H, H-1), 4.30-4.28 (dd, *J* = 3.3 Hz, *J* = 9.1 Hz, 1 H, H-3'), 4.16-4.13 (dd, *J* = 4.9 Hz, *J* = 11.6 Hz, 1 H, H-5a), 4.04-4.00 (m, 2 H, H-5', H-4), 3.89-3.85 (m, 1 H, IPA-CH), 3.74 (t, *J* = 8.9 Hz, 1 H, H-3), 3.53 (t, *J* = 9.3 Hz, 1 H, H-4'), 3.35-3.32 (t, *J* = 11.1 Hz, *J* = 10.2 Hz, 1 H, H-5b), 1.22 (d, *J* = 6.2 Hz, 3 H, H-6'), 1.15 (d, *J* = 6.2, 3 H, IPA-CH_3), 0.99 (d, *J* = 6.1, 3 H, IPA-CH_3), 0.80 (s, 9 H, TBS-*t*Bu), 0.09 (s, 3H, TBS-CH_3), 0.02 (s, 3H, TBS-CH_3); ^{13}C NMR ($CDCl_3$, 150 MHz) δ = 166.4 (C), 165.5 (C), 137.8 (C), 136.4 (C), 133.6 (CH), 133.4 (CH), 133.2 (C), 130.5 (C), 130.4 (C), 130.2 (2C, CH), 130.1 (2C, CH), 128.8 (CH), 128.7 (CH), 128.6 (CH), 128.3 (2C, CH), 128.1 (CH), 128.0 (CH), 126.5 (CH), 126.4 (CH), 126.1 (CH), 100.8 (CH), 95.1 (CH), 81.7 (CH), 80.6 (CH), 75.7 (CH_2), 75.6 (CH_2), 73.9 (CH), 73.7 (CH), 72.8 (CH), 72.6 (CH), 71.6 (CH), 68.6 (CH), 62.8 (CH_2), 30.1 (C), 26.1 (3C, CH_3), 23.7 (CH_3), 22.3 (CH_3), 18.5 (CH_3), 18.2 (C), -4.2 (CH_3), -4.3 (CH_3); HRMS *m/z* $(M+Na)^+$ calcd for $C_{52}H_{62}O_{11}SiNa^+$ 913.3954, found 913.3954.

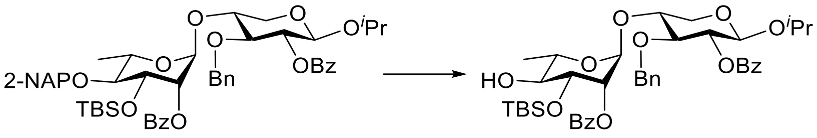

Compound 89. To a solution of compound **88** (469 mg, 527.0 μmol) in a co-solvent CH_2Cl_2/H_2O (9.4 mL, 19/1), DDQ (359 mg, 1.6 mmol) was added at RT. After stirring for 1 h, the whole mixture was filtered through a pad of Celite® and solids were washed with CH_2Cl_2, the filtrate was washed with 10% $Na_2S_2O_3$, sat. $NaHCO_{3(aq)}$. The organic layer was dried over anhydrous $MgSO_4$, filtered and concentrated *in vacuo*. The resulting crude residue was purified by flash column chromatography (EtOAc/Hex = 1/6) on silica gel to afford the desired compound **89** (0.34 g, 86%) as a white solid. $[\alpha]_D^{27}$ = -15.3 (*c* = 0.7, $CHCl_3$); IR (thin film in KBr) ν 3544, 2929, 1725, 1451, 1315, 1269, 1108, 1070, 1028, 838, 779, 754, 710 cm^{-1}; ^1H NMR ($CDCl_3$, 600 MHz) δ = 8.02-8.01 (d, *J* = 7.8 Hz, 2 H, Ar-H), 7.97-7.96 (d, *J* = 7.8 Hz, 2 H, Ar-H), 7.55 (m, 2 H, Ar-H), 7.44-7.40 (m, 4 H, Ar-H), 7.16-7.15 (m, 2 H, Ar-H), 7.09-7.08 (m, 3 H, Ar-H), 5.22 (s, 1 H, H-2'), 5.15 (t, *J* = 8.4 Hz, *J* = 7.9 Hz, 1H, H-2), 4.95 (s, 1 H, H-1'), 4.77-4.62 (ABq, *J* = 11.1 Hz, 2 H, $ArCH_2$), 4.55 (d, *J* = 7.3 Hz, 1 H, H-1), 4.15-4.12 (dd,

J = 4.6 Hz, J = 11.7 Hz, 1 H, H-5a), 4.03-4.00 (m, 2 H, H-4, H-3'), 3.92-3.85 (m, 2 H, H-5', IPA-CH), 3.73 (t, J = 8.8 Hz, 1 H, H-3), 3.63 (t, J = 9.2 Hz, 1 H, H-4'), 3.33 (t, J = 10.8 Hz, J = 10.4 Hz, 1 H, H-5b), 1.24 (d, J = 4.3 Hz, 3 H, H-6'), 1.15 (d, J = 6.1, 3 H, IPA-CH_3), 0.99 (d, J = 6.0, 3 H, IPA-CH_3), 0.77 (s, 9 H, TBS-*t*Bu), 0.06 (s, 3H, TBS-CH_3), 0.04 (s, 3H, TBS-CH_3); ^{13}C NMR ($CDCl_3$, 150 MHz) δ = 166.2 (C), 165.3 (C), 137.8 (C), 133.4 (CH), 133.2 (CH), 130.2 (C), 130.0 (2C, CH), 129.9 (2C, CH), 128.6 (2C, CH), 128.5 (2C, CH), 128.4 (2C, CH), 128.3 (2C, CH), 127.8 (CH), 100.5 (CH), 95.1 (CH), 80.2 (CH), 75.0 (CH_2), 73.6 (CH), 73.5 (CH), 73.0 (CH), 72.6 (CH), 72.4 (CH), 71.6 (CH), 68.7 (CH), 62.5 (CH_2), 29.9 (C), 25.8 (3C, CH_3), 23.5 (CH_3), 22.0 (CH_3), 18.1 (C), 17.9 (CH_3), -4.4 (CH_3), -4.6 (CH_3); HRMS m/z $(M+Na)^+$ calcd for $C_{41}H_{54}O_{11}SiNa^+$ 773.3328, found 773.3325.

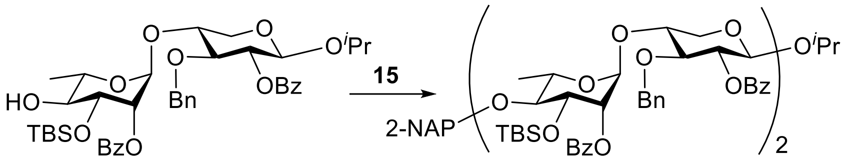

89 **90**

Compound 90. To a solution of donor **15** (394 mg, 412.0 μmol) and acceptor **89** (258 mg, 344.0 μmol) in anhydrous CH_2Cl_2 (6.5 mL), freshly activated AW-500 molecular sieves (550 mg) was added under a nitrogen atmosphere, and reaction mixture was allowed to stir at RT for 0.5 h. Then, the reaction flask was cooled to -40 °C, NIS (92.8 mg, 412.0 μmol) and TfOH (4.5 μL, 51.0 μmol) were added, and the solution was allowed to stir at the same temperature for 3 h. Et_3N was added to quench the reaction, the whole mixture was filtered through a pad of Celite®, solids were washed with CH_2Cl_2. The filtrate was washed with 10% $Na_2S_2O_3$ and sat. $NaHCO_{3(aq)}$, and then, the organic layer was dried over anhydrous $MgSO_4$, filtered, and concentrated *in vacuo*. The crude residue was purified by flash column chromatography (EtOAc/Hex = 1/6) on silica gel to afford the compound **90** (476 mg, 87%) as white solid. $[\alpha]_D^{27}$ = -34.1 (c = 0.4, $CHCl_3$); IR (thin film in KBr) ν 2929, 1724, 1267, 1105, 1070, 711 cm^{-1}; ^{1}H NMR ($CDCl_3$, 600 MHz) δ = 8.07 (d, J = 7.7 Hz, 2 H, Ar-H), 8.01 (d, J = 7.7 Hz, 2 H, Ar-H), 7.90 (d, J = 7.7 Hz, 2 H, Ar-H), 7.86 (d, J = 7.7 Hz, 2 H, Ar-H), 7.82-7.75 (m, 3 H, Ar-H), 7.71 (s, 1 H, Ar-H), 7.60-7.52 (m, 3 H, Ar-H), 7.48-7.45 (m, 6 H, Ar-H), 7.40-7.38 (m, 4 H, Ar-H), 7.23-7.21 (m, 2 H, Ar-H), 7.11 (d, J = 7.4 Hz, 2 H, Ar-H), 7.03-6.97 (m, 8 H, Ar-H), 5.24 (s, 1 H, H-2'''), 5.21 (t, J = 6.7 Hz, J = 6.9 Hz, 1 H, H-2''), 5.15 (d, J = 5.8 Hz, 1 H, H-1''), 5.11-5.08 (m, 2 H, H-2, H-2'), 5.02 (ABq, J = 11.5 Hz, 1 H, $ArCH_2$), 4.97 (s, 1 H, H-1'''), 4.93 (s, 1 H, H-1'), 4.77-4.73 (m, 2 H, $ArCH_2$), 4.67-4.63 (m, 2 H, $ArCH_2$), 4.49-4.47 (m, 2 H, H-1, Ar-CH_2), 4.22-4.17 (m, 3 H, H-3', H-5a'', H-3'''), 4.09-4.07 (dd, J = 4.7 Hz, J = 11.7 Hz, 1 H, H-5a),

3.98-3.87 (m, 5 H, H-4, H-4', H-5', H-4'', H-5'''), 3.84-3.80 (m, 1 H, IPA-CH), 3.70 (t, *J* = 7.5 Hz, 1 H, H-3''), 3.64 (t, *J* = 8.8 Hz, 1 H, H-3), 3.50 (t, *J* = 9.2 Hz, 1 H, H-4'''), 3.46-3.43 (dd, *J* = 8.4 Hz, *J* = 11.7 Hz, 1 H, H-5b''), 3.27 (t, *J* = 10.8 Hz, *J* = 10.6 Hz, 1 H, H-5), 1.23 (d, *J* = 5.8 Hz, 3 H, H-6'), 1.19 (d, *J* = 6.1 Hz, 3 H, H-6'''), 1.12 (d, *J* = 6.8 Hz, 3 H, IPA-CH_3), 0.95 (d, *J* = 6.0 Hz, 3 H, IPA-CH_3), 0.77 (s, 9 H, TBS-*t*Bu), 0.74 (s, 9 H, TBS-*t*Bu), 0.009 (s, 3 H, TBS-CH_3), -0.02 (s, 3 H, TBS-CH_3), -0.03 (s, 3 H, TBS-CH_3), -0.07 (s, 3 H, TBS-CH_3); ^{13}C NMR ($CDCl_3$, 150 MHz) δ = 166.3 (C), 166.2 (2C, C), 165.3 (2C, C), 165.2 (2C, C), 137.7 (C), 137.5 (C), 136.1 (C), 133.47 (CH), 133.4 (CH), 133.0 (CH), 130.3 (C), 130.2 (C), 130.0 (CH), 129.96 (C), 129.9 (CH), 128.7 (CH), 128.65 (CH), 128.6 (CH), 128.5 (CH), 128.4 (CH), 128.3 (CH), 128.1 (CH), 128.0 (CH), 127.9 (CH), 127.8 (CH), 127.7 (CH), 126.3 (CH), 126.2 (CH), 126.0 (CH), 100.5 (CH), 100.1 (CH), 95.1 (CH), 94.8 (CH), 81.4 (CH), 79.4 (CH), 79.2 (CH), 75.5 (CH_2), 74.9 (CH_2), 74.7 (CH_2), 73.8 (CH), 73.7 (CH), 73.5 (CH), 73.4 (CH), 72.7 (CH), 72.6 (CH), 72.3 (CH), 71.5 (CH), 71.3 (CH), 68.6 (CH), 68.0 (CH), 62.6 (CH_2), 61.4 (CH_2), 26.0 (3C, CH_3), 25.9 (3C, CH_3), 23.4 (CH_3), 22.0 (CH_3), 18.3 (CH_3), 18.2 (CH_3), 18.0 (C), 17.9 (C), -4.08 (CH_3), -4.4 (CH_3), -4.5 (CH_3), -4.6 (CH_3); HRMS m/z $(M+Na)^+$ calcd for $C_{90}H_{108}O_{21}Si_2Na^+$ 1604.6844, found 1604.6845.

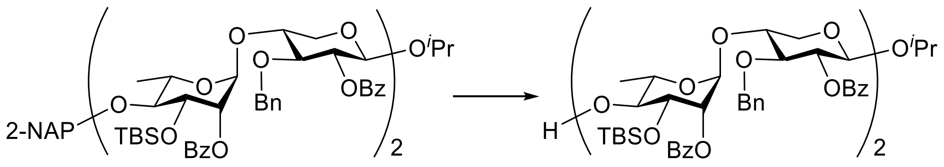

90 **91**

Compound 91. To a solution of compound **90** (409 mg, 259.0 μmol) in a mixed solvent of CH_2Cl_2/H_2O (8.0 mL, 19/1), DDQ (176 mg, 777.0 μmol) was added at room temperature. After stirring for 1 h, the whole mixture was filtered through a pad of Celite® and solids were washed with CH_2Cl_2. The filtrate was washed with 10% $Na_2S_2O_3$ and sat. $NaHCO_{3(aq)}$. The organic layer was dried over anhydrous $MgSO_4$, filtered, and concentrated *in vacuo*. The resulting crude residue was purified by flash column chromatography (EtOAc/Hex = 1/4) on silica gel to afford the desired compound **91** (0.317 g, 85%) as a white solid. $[\alpha]_D^{27}$ = -29.5 (*c* = 0.4, $CHCl_3$); IR (thin film in KBr) ν 2929, 2857, 1723, 1602, 1451, 1316, 1267, 1096, 1069, 1028, 837, 779, 710 cm^{-1}; ^{1}H NMR ($CDCl_3$, 600 MHz) δ = 8.02-8.00 (m, 4 H, Ar-H), 7.90 (d, *J* = 7.7 Hz, 2 H, Ar-H), 7.86 (d, *J* = 7.7 Hz, 2 H, Ar-H), 7.86 (d, *J* = 7.7 Hz, 2 H, Ar-H), 7.57-7.52 (m, 3 H, Ar-H), 7.47-7.38 (m, 7 H, Ar-H), 7.25-7.24 (m, 3 H, Ar-H), 7.13 (s, 4 H, Ar-H), 7.04-7.00 (m, 5 H, Ar-H), 5.22-5.16 (m, 3 H, H-1'', H-2'', H-2'''), 5.11-5.08 (m, 2 H, H-2, H-1'), 4.98 (s, 1 H, H-1'''), 4.93 (s, 1 H, H-1'), 4.71-4.65 (m, 2 H, $ArCH_2$), 4.49-4.47 (m, 2 H, H-1, $ArCH_2$), 4.23-

4.18 (m, 2 H, H-3', H-5a''), 4.10-4.07 (m, 1 H, H-5a), 3.98-3.89 (m, 5 H, H-4, H-4', H-5', H-4'', H-3'''), 3.84-3.80 (m, 1 H, IPA-CH), 3.76-3.73 (m, 1 H, H-5'''), 3.70 (t, J = 7.3 Hz, 1 H, H-3''), 3.64 (t, J = 8.8 Hz, 1 H, H-3), 3.58 (t, J = 9.3 Hz, 1 H, H-4'''), 3.48-3.45 (dd, J = 7.9 Hz, J = 12.0 Hz, 1 H, H-5b''), 3.29-3.26 (m, 1 H, H-5b), 1.24 (d, 3 H, H-6'), 1.12 (d, J = 6.1 Hz, 3 H, H-6'''), 1.12 (d, J = 6.1 Hz, 3 H, IPA-CH_3), 0.95 (d, J = 6.0 Hz, 3 H, IPA-CH_3), 0.74 (s, 18 H, TBS-*t*Bu), -0.01 (s, 3 H, TBS-CH_3), -0.03 (s, 3 H, TBS-CH_3), -0.04 (s, 3 H, TBS-CH_3), -0.07 (s, 3 H, TBS-CH_3); ^{13}C NMR ($CDCl_3$, 150 MHz) δ = 166.3 (C), 166.2 (C), 165.3 (C), 165.2 (C), 137.7 (2C, C), 133.5 (CH), 133.4 (CH), 133.1 (CH), 133.0 (CH), 130.3 (C), 130.0 (CH), 129.96 (C), 129.9 (CH), 129.8 (C), 128.7 (CH), 128.6 (CH), 128.5 (CH), 128.46 (CH), 128.4 (CH), 128.1 (CH), 127.9 (CH), 127.7 (CH), 100.5 (CH), 100.0 (CH), 95.3 (CH), 94.8 (CH), 79.4 (CH), 78.7 (CH), 77.4 (CH), 76.4 (CH), 74.7 (CH_2), 74.5 (CH_2), 73.7 (CH), 73.6 (CH), 73.5 (CH), 73.4 (CH), 73.0 (CH), 72.5 (CH), 72.46 (CH), 72.4 (CH), 71.5 (CH), 68.7 (CH), 68.0 (CH), 62.6 (CH_2), 61.1 (CH_2), 25.9 (3C, CH_3), 25.7 (3C, CH_3), 23.4 (CH_3), 22.0 (CH_3), 18.4 (CH_3), 18.1 (C), 17.9 (C), 17.8 (CH_3), -4.0 (CH_3), -4.4 (CH_3), -4.5 (CH_3), -4.6 (CH_3); HRMS m/z $(M+H)^+$ calcd for $C_{79}H_{100}O_{21}Si_2H^+$ 1441.6368, found 1441.6368.

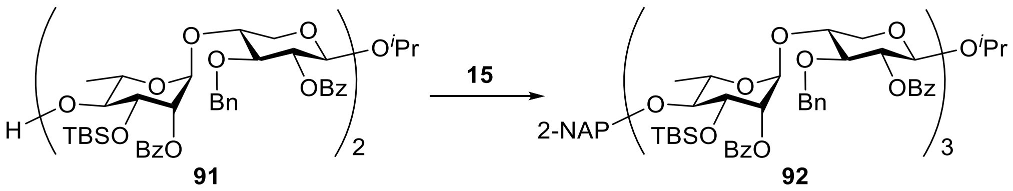

Compound 92. To a solution of donor **15** (245 mg, 256.0 μmol) and acceptor **91** (308 mg, 213.0 μmol) in anhydrous CH_2Cl_2 (5.5 mL), freshly activated AW-500 molecular sieves (550 mg) was added under a nitrogen atmosphere, and reaction mixture was allowed to stir at room temperature for 0.5 h. Then, the reaction flask was cooled to -40 °C, NIS (57.6 mg, 256 μmol) and TfOH (2.8 μL, 32.0 μmol) were added, and the solution was allowed to stir at the same temperature for 3 h. Et_3N was added to quench the reaction, the whole mixture was filtered through a pad of Celite®, solids were washed with CH_2Cl_2. The filtrate was washed with 10% $Na_2S_2O_3$ and sat. $NaHCO_{3(aq)}$, and then, the organic layer was dried over anhydrous $MgSO_4$, filtered, and concentrated *in vacuo*. The resulting crude residue was purified by flash column chromatography (EtOAc/Hex = 1/5) on silica gel to afford the desired compound **92** (419 mg, 86%) as a white solid. $[\alpha]_D^{27}$ = -34.7 (*c* = 0.6, $CHCl_3$); IR (thin film in KBr) ν 2929, 1723, 1266, 1096, 1069, 838, 710 cm^{-1}; ^{1}H NMR ($CDCl_3$, 600 MHz) δ = 8.06 (d, J = 7.9 Hz, 2 H, Ar-H), 8.02-7.99 (m, 4 H, Ar-H), 7.92-7.90 (m, 4 H, Ar-H), 7.82-7.75 (m, 5 H, Ar-H), 7.71 (s, 1 H, Ar-H), 7.60-7.52 (m, 4 H, Ar-H), 7.48-7.42 (m, 9 H,

Ar-H), 7.40-7.36 (m, 4 H, Ar-H), 7.29 (t, J = 7.3 Hz, J = 7.4 Hz, 2 H, Ar-H), 7.19 (t, J = 7.3 Hz, J = 7.4 Hz, 2 H, Ar-H), 7.11-6.97 (m, 15 H, Ar-H), 5.23 (s, 1 H, H-2’’’), 5.20-5.15 (m, 2 H, H-2’’, H-2’’’’), 5.13 (d, J = 5.7 Hz, 1 H, H-1’’), 5.10-5.06 (m, 4 H, H-2, H-2’, H-2’’’ , H-1’’’’), 5.02 (ABq, J = 11.5 Hz, 1 H, ArCH2), 4.96 (s, 1 H, H-1’’’’’), 4.94 (s, 1 H, H-1’’’), 4.91 (s, 1 H, H-1’), 4.77-4.73 (m, 2 H, ArCH2), 4.64-4.61 (m, 3 H, ArCH2), 4.57 (ABq, J = 11.0 Hz, 1 H, ArCH2), 4.47-4.45 (m, 2 H, H-1, ArCH2), 4.21-4.15 (m, 4 H, H-3’, H-5a’’, H-5a’’’’, H-3’’’’’), 4.10-4.05 (m, 2 H, H-5a, H-3’’’), 3.97-3.86 (m, 7 H, H-4, H-4’, H-5’, H-4’’, H-5’’’, H-4’’’’, H-5’’’’’), 3.83-3.79 (m, 1 H, IPA-CH), 3.78-3.74 (m, 1 H, H-4’’’), 3.69 (t, J = 7.4 Hz, J = 7.5 Hz, 1 H, H-3’’), 3.65-3.61 (m, 2 H, H-3, H-3’’’’), 3.49 (t, J = 9.2 Hz, 1 H, H-4’’’’’), 3.45-3.38 (m, 2 H, H-5b’’, H-5b’’’’), 3.26 (t, J = 10.6 Hz, J = 10.8 Hz, 1 H, H-5b), 1.20-1.14 (m, 9 H, H-6’, H-6’’’, H-6’’’’’), 1.12 (d, J = 6.0 Hz, 3 H, IPA-CH3), 0.94 (d, J = 5.8 Hz, 3 H, IPA-CH3), 0.77, 0.71, 0.70 (s, 27 H, TBS-*t*Bu), -0.004, -0.02, -0.06, -0.10, -0.13 (s, 18 H, TBS-CH3); ^{13}C NMR ($CDCl_3$, 150 MHz) δ = 166.37 (C), 166.3 (2C, C), 165.3 (C), 165.29 (C), 165.26 (C), 137.7 (C), 137.6 (C), 137.5 (C), 136.1 (C), 133.4 (2C, CH), 133.1 (CH), 133.09 (2C, C, CH), 133.0 (CH), 130.3 (C), 130.2 (C), 130.1 (CH), 130.03 (CH), 130.01 (2C, C, CH), 129.9 (CH), 129.8 (CH), 128.7 (CH), 128.6 (CH), 128.5 (CH), 128.46 (CH), 128.4 (CH), 128.3 (CH), 128.13 (CH), 128.1 (CH), 127.9 (CH), 127.8 (CH), 127.7 (CH), 126.3 (CH), 126.2 (CH), 126.0 (CH), 100.5 (CH), 100.1 (CH), 100.04 (CH), 95.0 (2C, CH), 94.9 (CH), 81.4 (CH), 79.4 (CH), 79.3 (CH), 78.4 (CH), 77.4 (CH), 75.5 (CH2), 75.0 (CH2), 74.7 (CH2), 74.4 (CH2), 73.8 (CH), 73.7 (CH), 73.5 (CH), 73.4 (2C, CH), 72.8 (CH), 72.5 (CH), 72.3 (CH), 71.5 (CH), 71.4 (CH), 71.4 (CH), 68.6 (CH), 68.0 (2C, CH), 62.6 (CH2), 61.4 (2C, CH2), 29.9 (C), 26.0 (6C, CH3), 25.9 (3C, CH3), 23.4 (CH3), 22.0 (CH3), 18.3 (CH3), 18.2 (2C, CH3), 17.9 (C), 17.8 (C), -4.0 (CH3), -4.1 (CH3), -4.5 (2C, CH3), -4.54 (CH3), -4.6 (CH3); HRMS m/z ($M+Na^{+}+NH4^{+}$) calcd for $C_{128}H_{154}O_{31}Si_3Na_1NH_4$ 1156.5021, found 1156.5011.

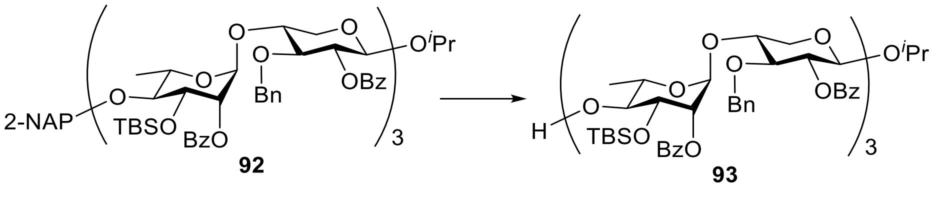

Compound 93. To a solution of compound **92** (205 mg, 90.0 μmol) in a mixed solvent of CH_2Cl_2/H_2O (4.0 mL, 19/1), DDQ (61.6 g, 271.0 μmol) was added at room temperature. After stirring for 1 h, the whole mixture was filtered through a pad of Celite® and solids were washed with CH_2Cl_2. The filtrate was then washed with 10% $Na_2S_2O_3$ and sat. $NaHCO_{3(aq)}$. The organic

layer was dried over anhydrous $MgSO_4$, filtered, and concentrated *in vacuo*. The crude residue was purified by flash column chromatography (EtOAc/Hex = 1/4) on silica gel to afford the desired compound **93** (152 mg, 80%) as a white solid. $[\alpha]_D^{29}$ = -48.5 (c = 1.1, $CHCl_3$); IR (thin film in KBr) ν 2929, 1723, 1451, 1267, 1097, 1028, 837, 710 cm^{-1}; ^1H NMR ($CDCl_3$, 600 MHz) δ = 8.02-7.99 (m, 6 H, Ar-H), 7.90 (t, J = 7.9 Hz, 4 H, Ar-H), 7.79 (d, J = 7.7 Hz, 2 H, Ar-H), 7.58-7.52 (m, 4 H, Ar-H), 7.48-7.42 (m, 7 H, Ar-H), 7.40-7.37 (m, 3 H, Ar-H), 7.31 (t, J = 7.5 Hz, J = 7.7 Hz, 2 H, Ar-H), 7.20 (t, J = 7.5 Hz, J = 7.7 Hz, 2 H, Ar-H), 7.13 (s, 5 H, Ar-H), 7.08-7.00 (m, 10 H, Ar-H), 5.21-5.15 (m, 4 H, H-2'', H-1'''', H-2'''', H-2'''''), 5.10-5.07 (m, 4 H, H-2, H-2', H-1'', H-2'''), 4.98 (s, 1 H, H-1'''''), 4.95 (s, 1 H, H-1'), 4.92 (s, 1 H, H-1'''), 4.72-4.55 (m, 5 H, $ArCH_2$), 4.47-4.46 (m, 2 H, H-1, $ArCH_2$), 4.21-4.14 (m, 3 H, H-5a'', H-3''', H-5a''''), 4.11-4.06 (m, 2 H, H-5a, H-3'), 3.96-3.38 (m, 7 H, H-4, H-4', H-4'', H-4''', H-5''', H-4'''', H-3'''''), 3.83-3.73 (m, 3 H, H-5', H-5''''', IPA-CH), 3.70 (t, J = 7.2 Hz, 1 H, H-3''''), 3.66-3.62 (m, 2 H, H-3, H-3''), 3.60-3.57 (m, 1 H, H-4'''''), 3.47-3.44 (dd, J = 11.9 Hz, J = 7.9 Hz, 1 H, H-5b''''), 3.42-3.39 (dd, J = 8.3 Hz, J = 11.8 Hz, 1 H, H-5b''), 3.27 (t, J = 10.8 Hz, J = 10.7 Hz, 1 H, H-5b), 1.79 (1 H, OH), 1.21-1.16 (m, 9 H, H-6', H-6''', H-6'''''), 1.12 (d, J = 6.2 Hz, 3 H, IPA-CH_3), 0.95 (d, J = 6.1 Hz, 3 H, IPA-CH_3), 0.74, 0.71 (s, 27 H, TBS-*t*Bu), -0.01, -0.04, -0.06, -0.10, -0.12, -0.13 (s, 18 H, TBS-CH_3); ^{13}C NMR ($CDCl_3$, 150 MHz) δ = 166.4 (C), 166.3 (C), 166.2 (C), 165.3 (C), 165.29 (C), 165.2 (C), 137.7 (2C, C), 137.5 (C), 133.4 (CH), 133.2 (CH), 133.07 (CH), 133.0 (CH), 130.3 (C), 130.1 (CH), 130.0 (CH), 129.96 (C), 129.9 (CH), 129.8 (C), 128.7 (CH), 128.68 (CH), 128.6 (CH), 128.5 (CH), 128.45 (CH), 128.44 (CH), 128.4 (CH), 128.3 (CH), 128.2 (CH), 127.9 (CH), 127.8 (CH), 127.7 (CH), 100.5 (CH), 100.0 (CH), 99.9 (CH), 95.3 (CH), 95.1 (CH), 94.8 (CH), 79.4 (CH), 78.8 (CH), 78.4 (CH), 74.7 (CH_2), 74.6 (CH_2), 74.4 (CH_2), 73.8 (CH), 73.7 (CH), 73.6 (CH), 73.5 (CH), 73.4 (CH), 73.3 (CH), 73.0 (CH), 72.6 (CH), 72.5 (CH), 72.4 (CH), 72.3 (CH), 71.6 (CH), 71.5 (CH), 71.4 (CH), 68.7 (CH), 68.1 (CH), 68.0 (CH), 62.6 (CH_2), 61.4 (CH_2), 61.0 (CH_2), 25.9 (6C, CH_3), 25.7 (3C, CH_3), 23.4 (CH_3), 22.0 (CH_3), 18.4 (CH_3), 18.3 (CH_3), 18.1 (C), 17.9 (CH_3), 17.8 (2C, C), -4.0 (CH_3), -4.1 (CH_3), -4.4 (CH_3), -4.5 (CH_3), -4.56 (CH_3), -4.6 (CH_3); HRMS m/z $(M+H)^+$ calcd for $C_{117}H_{146}O_{31}Si_3H^+$ 2132.9262, found 2132.9259.

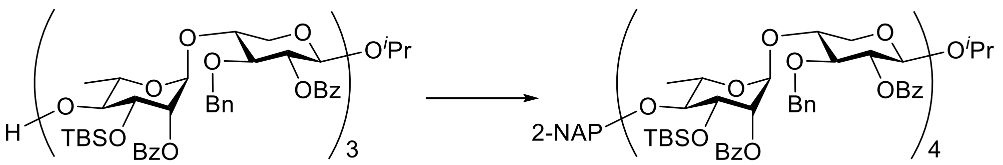

93 **94**

Compound 94. To a solution of donor **15** (60.1 mg, 63.0 μmol), and acceptor **93** (112 mg, 52.0 μmol) in anhydrous CH_2Cl_2 (2.0 ml), freshly activated AW-500 molecular sieves were added under a nitrogen atmosphere. The reaction mixture was allowed to stir at room temperature for 0.5 h. Then, the reaction flask was cooled to -40 °C, NIS (14.2 mg, 63.0 μmol) and TfOH (0.7 μL, 7.9 μmol) were added to the reaction mixture, and the reaction was allowed to stir at same temperature for 3 h. Et_3N was added to quench the reaction. The whole mixture was filtered through a pad of Celite®, and the solids were washed with CH_2Cl_2. The filtrate was washed with 10% $Na_2S_2O_3$ and sat. $NaHCO_{3(aq)}$. The organic layer was dried over anhydrous $MgSO_4$, filtered, and concentrated *in vacuo*. The resulting crude residue was purified by flash column chromatography (EtOAc/Hex = 1/4) on silica gel to afford the desired compound **94** (135 mg, 87%) as a white solid. $[\alpha]_D^{29}$ = -42.4 (c = 0.9, $CHCl_3$); IR (thin film in KBr) *v* 2928, 1725, 1265, 1096, 1069, 837, 710 cm^{-1}; ^{1}H NMR ($CDCl_3$, 600 MHz) δ = 8.02 (d, *J* = 7.7 Hz, 2 H, Ar-H), 8.02-7.99 (m, 6 H, Ar-H), 7.90 (t, *J* = 8.9 Hz, *J* = 8.5 Hz, 4 H, Ar-H), 7.84-7.57 (m, 7 H, Ar-H), 7.71 (s, 1 H, Ar-H), 7.59-7.52 (m, 5 H, Ar-H), 7.48-7.42 (m, 16 H, Ar-H), 7.30-7.25 (m, 4 H, Ar-H), 7.19 (t, *J* = 7.6 Hz, *J* = 7.7 Hz, 2 H, Ar-H), 7.11-6.97 (m, 20 H, Ar-H), 5.23-5.00 (m, 12 H, H-2, H-2’, H-1’’, H-2’’, H-2’’’, H-1’’’’, H-2’’’’, H-2’’’’’, H-1’’’’’’, H-2’’’’’’, H-2’’’’’’’, $ArCH_2$), 4.96-4.91 (m, 4 H, H-1’, H-1’’’, H-1’’’’’, H-1’’’’’’’), 4.77-4.73 (m, 2 H, $ArCH_2$), 4.64-4.54 (m, 6 H, $ArCH_2$), 4.47-4.45 (m, 2 H, H-1, $ArCH_2$), 4.22-4.05 (m, 8 H, H-5a, H-3’, H-5a’’, H-3’’’, H-5a’’’’, H-3’’’’’, H-5a’’’’’’, H-3’’’’’’’), 3.98-3.85 (m, 9 H, H-4, H-4’, H-5’, H-4’’, H-5’’’, H-4’’’’, H-5’’’’’, H-4’’’’’’, H-5’’’’’’’), 3.83-3.79 (m, 1 H, IPA-CH), 3.76-3.72 (m, 2 H, H-4’’’, H-4’’’’’), 3.69 (t, *J* = 7.6 Hz, *J* = 7.7 Hz, 1 H, H-3’’’’’’), 3.64-3.62 (m, 3 H, H-3, H-3’’, H-3’’’’), 3.49 (t, *J* = 9.2 Hz, *J* = 9.4 Hz, 1 H, H-4’’’’’’’), 3.45-3.38 (m, 3 H, H-5b’’, H-5b’’’’, H-5b’’’’’’), 1.20-1.18 (m, 6 H, H-6’, H-6’’’’’’’), 1.15-1.11 (m, 9 H, H-6’’’, H-6’’’’’, IPA-CH_3), 0.95 (d, *J* = 6.0 Hz, 3 H, IPA-CH_3), 0.77, 0.72, 0.70, 0.68 (s, 36 H, TBS-*t*Bu), -0.008, -0.02, -0.06, -0.10, -0.131, -0.136, -0.15, -0.16 (s, 24 H, TBS-CH_3); ^{13}C NMR ($CDCl_3$, 150 MHz) δ = 166.35 (2C, C), 166.3 (C), 165.37 (C), 165.3 (C), 165.27 (C), 165.2 (C), 137.7 (C), 137.6 (2C, C), 137.5 (C), 136.1 (C), 133.4 (CH), 133.1 (CH), 133.08 (CH), 133.07 (CH), 132.9 (CH), 130.3 (C), 130.2 (C), 130.1 (CH), 130.0 (CH), 129.9 (CH), 129.8 (C), 128.67 (CH), 128.6 (CH), 128.5 (CH), 128.45 (CH), 128.43 (CH), 128.4 (CH), 128.3 (CH), 128.1 (CH), 128.0 (CH), 127.9 (CH), 127.8 (CH), 127.7

(CH), 126.3 (CH), 126.2 (CH), 126.0 (CH), 100.5 (CH), 100.1 (CH), 100.0 (CH), 99.9 (CH), 95.2 (CH), 95.1 (CH), 94.9 (CH), 81.4 (CH), 79.4 (CH), 79.3 (CH), 78.4 (CH), 78.3 (CH), 76.3 (CH), 75.5 (CH_2), 75.0 (CH_2), 74.7 (CH_2), 74.4 (CH_2), 74.3 (CH_2), 73.8 (CH), 73.7 (CH), 73.6 (CH), 73.5 (CH), 73.4 (CH), 73.3 (CH), 72.8 (CH), 72.5 (CH), 72.3 (CH), 71.5 (CH), 71.42 (CH), 71.4 (CH), 68.6 (CH), 68.1 (CH), 68.0 (CH), 62.6 (CH_2), 61.4 (2C, CH_2), 61.3 (CH_2), 25.94 (6C, CH_3), 25.92 (3C, CH_3), 25.9 (3C, CH_3), 23.4 (CH_3), 22.0 (CH_3), 18.3 (CH_3), 18.2 (3C, CH_3), 17.9 (C), 17.87 (2C, C), 17.8 (C), -4.1 (CH_3), -4.2 (2C, CH_3), -4.5 (2C, CH_3), -4.55 (CH_3), -4.58 (2C, CH_3); HRMS m/z $(M+H)^+$ calcd for $C_{166}H_{200}O_{41}Si_4N_2H_8$ 1499.6686, found 1499.6689.

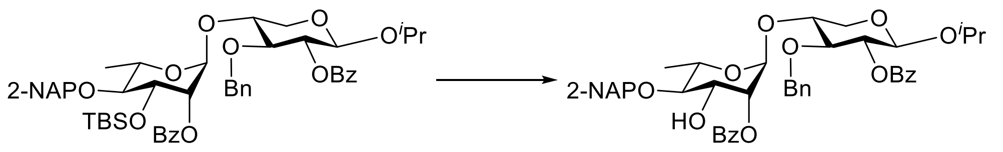

88 **95**

Compound 95. To a solution of compound **88** (859 mg, 964.0 μmol) in CH_3CN (86.0 mL), $BF_3 \cdot Et_2O$ (357 μL, 1.4 mmol) was added. The reaction mixture was allowed to stir at room temperature for 1 h. Then, sat. $NaHCO_{3(aq)}$ was added to quench the reaction and extracted with EtOAc (2 x 100 mL). The combined organic layers were washed with brine, dried over anhydrous $MgSO_4$, filtered, and concentrated *in vacuo*. The crude residue was purified by flash column chromatography (EtOAc/Hex =1/4) on silica gel to afford the desired compound **95** (672 mg, 90%) as a white solid. $[\alpha]_D^{26}$ = -48.3 (*c* = 1.4, $CHCl_3$); IR (thin film in KBr) *v* 3476, 2972, 2918, 1724, 1601, 1451, 1315, 1268, 1177, 1097, 1069, 1028, 978, 818, 751, 711, 476 cm^{-1}; ^1H NMR ($CDCl_3$, 600 MHz) δ = 8.00-7.99 (m, 4 H, Ar-H), 7.81-7.74 (m, 4 H, Ar-H), 7.57-7.52 (m, 2 H, Ar-H), 7.46-7.40 (m, 7 H, Ar-H), 7.16-7.15 (m, 2 H, Ar-H), 7.03-6.99 (m, 3 H, Ar-H), 5.32 (s, 1 H, H-2'), 5.17-5.14 (m, 1H, H-2), 4.98 (s, 1 H, H-1'), 4.91-4.84 (ABq, J = 11.4 Hz, 2 H, $ArCH_2$), 4.71-4.59 (ABq, J = 10.9 Hz, 2 H, $ArCH_2$), 4.55 (d, J = 7.02 Hz, 1 H, H-1), 4.19-4.17 (m, 1 H, H-3'), 4.14-4.11 (dd, J = 4.6 Hz, J = 11.8 Hz, 1 H, H-5a), 4.0-3.96 (m, 2 H, H-5', H-4), 3.88-3.86 (m, 1H, IPA-CH), 3.70 (t, J = 8.4 Hz, 1 H, H-3), 3.48 (t, J = 9.4 Hz, 1 H, H-4'), 3.35-3.32 (m, 1 H, H-5b), 2.12 (s, 1 H, OH), 1.27 (d, J = 5.8 Hz, 3 H, H-6'), 1.15 (d, J = 6.1, 3 H, IPA-CH_3), 1.00 (d, J = 6.1, 3 H, IPA-CH_3); ^{13}C NMR ($CDCl_3$, 150 MHz) δ = 166.5 (C), 165.3 (C), 137.6 (C), 135.8 (C), 133.6 (CH), 133.4 (C), 133.3 (CH), 133.2 (C), 130.2 (C), 130.0 (2C, CH), 129.9 (2C, CH), 129.7 (C), 128.7 (2C, CH), 128.6 (2C, CH), 128.55 (2C, CH), 128.5 (CH), 128.4 (2C, CH), 128.1 (CH), 127.9 (CH), 127.8 (CH), 126.8 (CH), 126.4 (CH), 126.2 (CH), 126.1 (CH), 100.2 (CH), 94.9 (CH), 81.7 (CH), 79.9 (CH), 75.2 (CH_2), 75.1 (CH_2), 73.4 (CH), 73.3 (CH), 72.6 (CH), 72.3 (CH), 70.5 (CH), 68.2 (CH), 62.2

(CH_2), 23.5 (CH_3), 22.0 (CH_3), 18.3 (CH_3); HRMS m/z $(M+Na)^+$ calcd for $C_{46}H_{48}O_{11}Na^+$ 799.3089, found 799.3088.

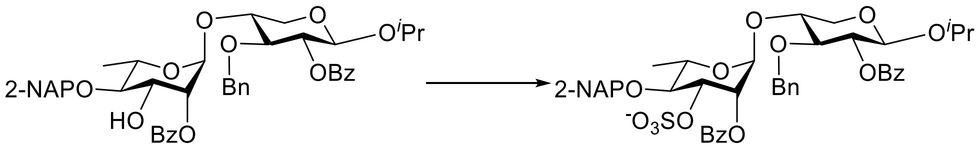

95 **99**

Compound 99. To a solution compound **95** (234 mg, 301.0 μmol) in anhydrous DMF (3.0 mL), $SO_3 \cdot Et_3N$ (304 mg, 3.0 mmol) was added under a nitrogen atmosphere at RT. Then, the reaction mixture was heated to 60 °C and allowed to stir at same temperature for 12 h. The reaction flask was cooled down to room temperature, sat. $NaHCO_{3(aq)}$ solution was added, and the mixture was allowed to stir for 1h. The resulting mixture was extracted with EtOAc, and the combined organic layers were washed with brine, dried over anhydrous $MgSO_4$, filtered, and concentrated *in vacuo*. The crude residue was purified by AG 50W-X8 cation exchange column (Na^+ form) followed by Sephadex LH-20 column using MeOH as an eluent to afford the desired product **99** (0.243 g, 89%) as a white solid. ^{1}H NMR ($CDCl_3$, 600 MHz) δ = 7.95-7.89 (m, 4 H, Ar-H), 7.67-7.63 (m, 4 H, Ar-H), 7.40-7.26 (m, 7 H, Ar-H), 7.17-7.14 (m, 3 H, Ar-H), 7.06-7.05 (m, 1 H, Ar-H), 6.89-6.85 (m, 3 H, Ar-H), 5.89 (s, 1 H, H-2'), 5.06-5.01 (m, 3 H, H-3', H-2, ArCH_2), 4.77 (s, 1 H, H-1'), 4.63-4.56 (ABq, J = 11.2 Hz, J = 10.7 Hz, 2 H, ArCH_2), 4.03-4.01 (m, 1 H, H-5'), 3.91-3.88 (dd, J = 4.5 Hz, J = 11.7 Hz, 1 H, H-5a), 3.82-3.79 (m, 1 H, H-4), 3.61-3.56 (m, 1 H, IPA-CH), 3.53-3.49 (m, 2 H, H-3, H-4'), 3.21-3.17 (m, 1 H, H-5b), 1.08 (d, J = 6.1 Hz, 3 H, H-6'), 0.98 (d, J = 6.1, 3 H, IPA-CH_3), 0.79 (d, J = 6.1, 3 H, IPA-CH_3); ^{13}C NMR ($CDCl_3$, 150 MHz) δ = 166.7 (C), 165.3 (C), 137.6 (C), 135.8 (C), 133.7 (CH), 133.3 (C), 133.2 (CH), 133.1 (C), 130.2 (CH), 130.0 (C), 129.8 (CH), 129.4 (CH), 129.3 (CH), 128.7 (CH), 128.5 (CH), 128.4 (CH), 128.2 (CH), 128.1 (CH), 128.0 (CH), 127.7 (CH), 127.6 (CH), 127.4 (CH), 126.9 (CH), 126.1 (CH), 126.0 (CH), 120.8 (CH), 100.0 (CH), 96.3 (CH), 79.6 (CH), 78.6 (CH), 77.6 (CH), 77.4 (CH), 74.9 (CH_2), 74.7 (CH_2), 73.7 (CH), 73.4 (CH), 72.2 (CH), 71.8 (CH), 68.3 (CH), 62.2 (CH_2), 23.3 (CH_3), 21.9 (CH_3), 18.2 (CH_3); HRMS m/z $(M+Na)^+$ calcd for $C_{46}H_{47}O_{14}SHNa^+$ 879.2657, found 879.2659.

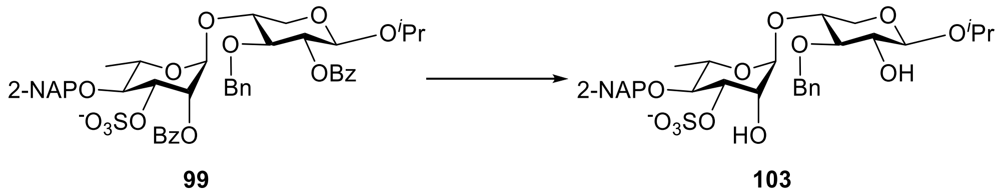

Compound 103. To a solution of compound **99** (118 mg, 134.0 μmol) in a co-solvent ($CHCl_3/MeOH/H_2O$ =1.2/5.1/0.8 mL), 5.0 M $NaOH_{(aq)}$ (0.8 mL, 0.5 M final concentration) was added, and the mixture was allowed to stir for 48 h at room temperature. AG 50W-X8 cation exchange resin was added to quench the reaction, and pH was adjusted to 7. The whole mixture was filtered through a pad of Celite®, washed with MeOH, and concentrated *in vacuo*. The crude residue was purified by Sephadex LH-20 column followed by AG 50W-X8 cation exchange column (Na^+ form) using MeOH as an eluent to afford the desired compound **103** (62.9 mg, 70%) as a white solid. ^{1}H NMR (CD_3OD, 600 MHz) δ = 7.83-7.79 (m, 4 H, Ar-H), 7.55-7.54 (m, 1 H, Ar-H), 7.44-7.42 (m, 2 H, Ar-H), 7.37-7.35 (m, 2 H, Ar-H), 6.98-6.96 (m, 3 H, Ar-H), 5.15-5.14 (ABq, *J* = 10.9 Hz,, 1 H, $ArCH_2$), 4.83-4.76 (m, 4 H, H-1', H-3', $ArCH_2$), 4.69-4.67 (ABq, *J* = 10.9 Hz, 1 H, $ArCH_2$), 4.32-4.29 (m, 2 H, H-1, H-2'), 4.06-4.00 (m, 2 H, H-5a, H-5'), 3.96-3.91 (m, 1 H, IPA-CH), 3.55 (t, *J* = 9.4 Hz, 1 H, H-4'), 3.41 (t, *J* = 9.0 Hz, 1 H, H-3), 3.27-3.26 (m, 1 H, H-2), 3.21 (t, *J* = 10.9 Hz, H-5b), 1.08 (dd, *J* = 5.8 Hz, *J* = 6.0 Hz, 6 H, IPA-CH_3), 1.07 (d, *J* = 6.1, 3 H, H-6'); ^{13}C NMR (CD_3OD, 150 MHz) δ = 139.5 (C), 137.7 (C), 134.7 (C), 134.4 (C), 131.9 (CH), 129.7 (CH), 129.0 (CH), 128.9 (CH), 128.8 (CH), 128.7 (CH), 128.5 (CH), 128.3 (CH), 128.0 (CH), 127.9 (CH), 126.8 (CH), 126.7 (CH), 103.6 (CH), 98.7 (CH), 84.0 (CH), 80.6 (CH), 79.7 (CH), 76.7 (CH_2), 75.6 (CH_2), 75.5 (CH), 73.8 (CH), 72.9 (CH), 70.5 (CH), 68.9 (CH), 63.5 (CH_2), 23.8 (CH_3), 22.1 (CH_3), 18.2 (CH_3); HRMS m/z $(M+Na)^+$ calcd for $C_{32}H_{39}O_{12}SHNa^+$ 671.2133, found 671.2128.

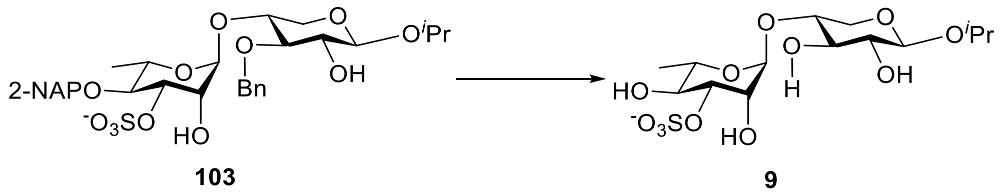

Compound 9. A mixture of compound **103** (50.1 mg, 75.0 μmol) and $Pd(OH)_2$ on activated charcoal (20% Pd, wet, Degussa type, 150 mg) in a mixed solvent $MeOH/H_2O$ (1.0 mL, 1/1) was purged with hydrogen for 10 min at room temperature. The reaction flask was then

equipped with a hydrogen balloon. After stirring for 48 h, the whole mixture was filtered, and the solution was concentrated under reduced pressure. The residue was purified by Sephadex G-10 column followed by AG 50W-X8 cation exchange column (Na^+ form) using water as eluent to afford the desired compound **9** (23.8 mg, 72%) as a white solid. ^1H NMR (D_2O, 600 MHz) δ = 4.99 (s, 1 H, H-1'), 4.58 (d, *J* = 7.9 Hz, 1 H, H-1), 4.55-4.53 (dd, *J* = 3.12 Hz, *J* = 9.7 Hz, 1 H, H-3'), 4.33 (s, 1 H, H-2'), 4.17-4.11 (m, 3 H, H-5a, H-5', IPA-CH), 3.75-3.71 (m, 1 H, H-4), 3.69-3.63 (m, 2 H, H-3, H-4'), 3.42 (t, *J* = 10.9 Hz, *J* = 11.3 Hz, 1 H, H-5b), 3.29 (t, *J* = 8.2 Hz, *J* = 9.0 Hz, 1 H, H-2), 1.39 (d, *J* = 6.3 Hz, 1 H, H-6'), 1.30 (d, *J* = 6.3, 3 H, IPA-CH_3), 1.28 (d, *J* = 6.1 Hz, 3H, IPA-CH_3); ^{13}C NMR (D_2O, 150 MHz) δ = 101.1 (CH), 97.5 (CH), 78.4 (CH), 73.9 (CH), 73.6 (CH), 73.2 (CH), 73.0 (CH), 69.7 (CH), 68.8 (CH), 68.6 (CH), 62.3 (CH_2), 22.2 (CH_3), 20.9 (CH_3), 16.6 (CH_3); HRMS m/z $(M+Na)^+$ calcd for $C_{14}H_{25}NaO_{12}SH^+$ 441.1037, found 441.1034.

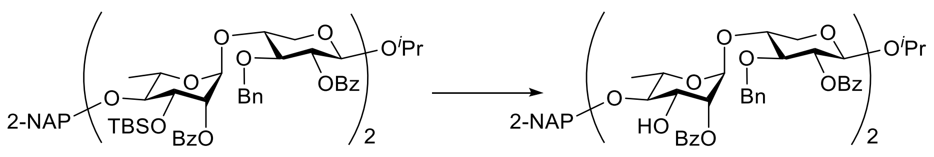

90 **96**

Compound 96. To a solution of compound **90** (206 mg, 130.0 μmol) in CH_3CN (20.6 mL), $BF_3{\cdot}Et_2O$ (48.1 μL, 195.0 μmol) was added at room temperature. The reaction mixture was allowed to stir at room temperature for 1 h. Then, sat. $NaHCO_{3(aq)}$ was added to quench the reaction, pH was adjusted to 7, and the whole mixture was directly extracted with EtOAc (100 mL × 2). The combined organic layers were washed with brine, dried over anhydrous $MgSO_4$, filtered, and concentrated *in vacuo*. The crude residue was purified by flash column chromatography (EtOAc/Hex =1/3) on silica gel to afford the desired compound **96** (156 mg, 88%) as white solid. $[\alpha]_D^{27}$ = -12.1 (*c* = 0.7, $CHCl_3$); IR (thin film in KBr) ν 3490, 2930, 1723, 1601, 1451, 1316, 1267, 1097, 1069, 1028, 725, 711 cm^{-1}; ^1H NMR ($CDCl_3$, 600 MHz) δ = 8.02 (d, *J* = 7.8 Hz, 2 H, Ar-H), 7.99 (d, *J* = 7.8 Hz, 2 H, Ar-H), 7.95 (d, *J* = 7.8 Hz, 2 H, Ar-H), 7.92 (d, *J* = 7.8 Hz, 2 H, Ar-H), 7.81-7.74 (m, 4 H, Ar-H), 7.57 (t, *J* = 7.4 Hz, 2 H, Ar-H), 7.53-7.48 (m, 2 H, Ar-H), 7.46-7.38 (m, 9 H, Ar-H), 7.34 (t, *J* = 7.4 Hz, 2 H, Ar-H), 7.11-7.08 (m, 7 H, Ar-H), 7.03-6.96 (m, 3 H, Ar-H), 5.29 (s, 1 H, H-2'''), 5.21-5.17 (m, 2 H, H-2', H-2''), 5.12 (t, *J* = 7.8 Hz, *J* = 7.9 Hz, 1 H, H-2), 4.97 (s, 1 H, H-1'''), 4.92-4.85 (m, 4 H, H-1', H-1'', $ArCH_2$), 4.67 (ABq, *J* = 10.9 Hz, 1 H, $ArCH_2$), 4.61 (ABq, *J* = 11.6 Hz, 1 H, $ArCH_2$), 4.59-4.50 (m, 3 H, H-1, $ArCH_2$), 4.18-4.16 (m, 1 H, H-2'''), 4.11-4.04 (m, 2 H, H-5a, H-5a''),

3.98-3.95 (m, 3 H, H-3’, H-4’’, H-5’’’), 3.93-3.81 (m, 3 H, H-4, H-5’, IPA-CH), 3.67-3.58 (m, 3 H, H-3, H-4’, H-3’’), 3.48 (t, *J* = 9.4 Hz, 1 H, H-4’’’), 3.35-3.32 (dd, *J* = 9.0 Hz, *J* = 11.5 Hz, 1 H, H-5b’’), 3.26 (t, *J* = 10.6 Hz, *J* = 10.5 Hz, 1 H, H-5), 2.34 (d, *J* = 4.6 Hz, 1 H, OH), 2.11 (d, *J* = 4.9 Hz, 1 H, OH), 1.27 (d, *J* = 6.0 Hz, 3 H, H-6’’’), 1.15-1.12 (m, 6 H, H-6’, IPA-CH_3), 0.98 (d, *J* = 6.0 Hz, 3 H, IPA-CH_3); ^{13}C NMR ($CDCl_3$, 150 MHz) δ = 166.53 (C), 166.5 (C), 165.3 (2C, C), 137.6 (C), 137.3 (C), 135.8 (C), 133.7 (2C, CH), 133.5 (CH), 133.4 (C), 133.22 (C), 133.2 (CH), 130.2 (C), 130.1 (CH), 130.0 (CH), 129.8 (CH), 129.7 (CH), 129.69 (CH), 129.6 (CH), 128.8 (CH), 128.7 (CH), 128.6 (CH), 128.5 (CH), 128.46 (CH), 128.4 (CH), 128.1 (CH), 127.94 (CH), 127.9 (CH), 127.8 (CH), 126.9 (CH), 126.4 (CH), 126.2 (CH), 126.1 (CH), 101.6 (CH), 100.3 (CH), 95.0 (CH), 94.9 (CH), 81.6 (CH), 80.6 (CH), 79.65 (CH), 79.6 (CH), 77.4 (CH), 75.3 (CH_2), 75.2 (CH_2), 74.9 (CH_2), 73.4 (CH), 73.34 (CH), 73.3 (CH), 73.1 (CH), 72.9 (CH), 72.7 (CH), 72.3 (CH), 70.5 (CH), 70.3 (CH), 68.2 (CH), 67.2 (CH), 62.2 (CH_2), 62.1 (CH_2), 23.4 (CH_3), 22.0 (CH_3), 18.3 (CH_3), 17.7 (CH_3); HRMS m/z $(M+Na)^+$ calcd for $C_{78}H_{80}O_{21}Na^+$ 1375.5084, found 1375.5092.

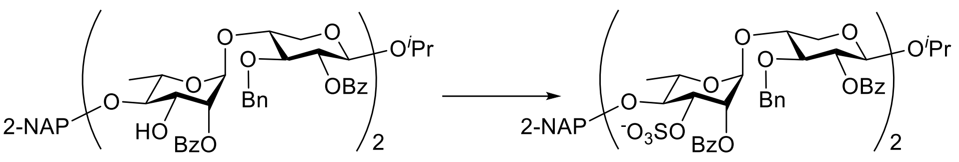

96 100

Compound 100. To a solution of compound **96** (156 mg, 115.0 μmol) in anhydrous DMF (3.1 mL), $SO_3·Et_3N$ (233 mg, 2.3 mmol) was added under a nitrogen atmosphere at room temperature. Then, the reaction mixture was heated to 60 °C and allowed to stir at same temperature for 12 h. The reaction flask was cooled down to room temperature, sat. $NaHCO_{3(aq)}$ solution was added, and the mixture was allowed to stir for 24 h. The resulting mixture was extracted with EtOAc, and the combined organic layers were washed with brine, dried over anhydrous $MgSO_4$, filtered, and concentrated *in vacuo*. The crude residue was purified by AG 50W-X8 cation exchange column (Na^+ form) followed by Sephadex LH-20 column using MeOH as an eluent to afford the desired product **100** (134 mg, 75%) as white solid. ^1H NMR (600 MHz, CD_3OD) δ = 8.05-8.03 (m, 6 H, Ar-H), 7.96 (s, 1 H, Ar-H), 7.92-7.90 (m, 3 H, Ar-H), 7.85-7.82 (m, 2 H, Ar-H), 7.64-7.59 (m, 4 H, Ar-H), 7.52-7.50 (m, 2 H, Ar-H), 7.47-7.45 (m, 6 H, Ar-H), 7.39 (t, *J* = 7.4 Hz, *J* = 7.2 Hz, 1 H, Ar-H), 7.28 (t, *J* = 7.5 Hz, *J* = 7.6 Hz, 2 H, Ar-H), 7.06 (d, *J* = 7.5 Hz, 2 H, Ar-H), 6.98-6.94 (m, 5 H, Ar-H), 6.88 (t, *J* = 7.3 Hz, 1 H, Ar-H), 6.77 (t, *J* = 7.5 Hz, 1 H, Ar-H),

5.72 (s, 1 H, H-2’’’), 5.62 (s, 1 H, H-2’), 5.38 (d, 1 H, J = 7.6 Hz, 1 H, H-1’’), 5.24 (ABq, J = 10.9 Hz, 1 H, $ArCH_2$), 5.12-5.09 (m, 3 H, H-2’’, H-1’’’, $ArCH_2$), 5.05 (s, 1 H, H-1’), 4.94-4.91 (m, 2 H, H-3’, H-2), 4.85-4.83 (m, 1 H, H-3’’’), 4.78 (ABq, J = 10.1 Hz, 1 H, $ArCH_2$), 4.57 (d, J = 7.8 Hz, 1 H, H-1), 4.50-4.47 (m, 2 H, $ArCH_2$), 4.37-4.36 (ABq, J = 11.2 Hz, 1 H, $ArCH_2$), 4.26-4.23 (dd, J = 4.8 Hz, J = 11.7 Hz, 1 H, H-5a’’), 4.12-4.06 (m, 3 H, H-4’, H-5’, H-5’’’), 3.99-3.93 (m, 2 H, H-4’’, H-5a), 3.90-3.81 (m, 3 H, H-3, H-4, IPA-CH), 3.73-3.66 (m, 2 H, H-5b, H-4’’’), 3.53 (t, J = 10.9 Hz, J = 10.5 Hz, 1 H, H-3’’), 3.35-3.31 (m, 1 H, H-5b’’), 1.17 (t, J = 6.4 Hz, 6 H, H-6’, H-6’’’), 1.09 (d, J = 6.1 Hz, 3 H, IPA-CH_3), 0.93 (d, J = 6.1 Hz, 3 H, IPA-CH_3); ^{13}C NMR (150 MHz, CD_3OD) δ = 167.7 (C), 167.3 (C), 167.2 (C), 166.8 (C), 138.8 (C), 138.7 (C), 137.5 (C), 134.7 (C), 134.5 (C), 134.3 (CH), 133.9 (CH), 131.4 (CH), 131.3 (C), 131.2 (C), 131.1 (C), 130.8 (CH), 130.7 (CH), 130.4 (CH), 129.8 (CH), 129.7 (CH), 129.6 (CH), 129.5 (CH), 129.3 (CH), 129.1 (CH), 129.0 (CH), 128.9 (CH), 128.7 (CH), 128.6 (CH), 128.5 (CH), 128.4 (CH), 128.3 (CH), 128.2 (CH), 126.9 (CH), 126.8 (CH), 121.4 (CH), 101.7 (CH), 101.3 (CH), 96.2 (CH), 96.0 (CH), 82.0 (CH), 81.1 (CH), 79.6 (CH), 79.5 (CH), 79.2 (CH), 77.8 (CH), 77.3 (CH), 77.1 (CH_2), 76.3 (CH_2), 75.7 (CH_2), 75.4 (CH), 75.3 (CH), 75.1 (CH), 74.8 (CH), 73.6 (CH), 73.3 (CH), 72.7 (CH), 69.1 (CH), 68.7 (CH), 63.7 (CH_2), 63.6 (CH_2), 23.6 (CH_3), 22.3 (CH_3), 18.48 (CH_3), 18.40 (CH_3); HRMS m/z $(M+H)^+$ calcd for $C_{78}H_{78}O_{27}S_2H_2$ 1513.4401, found 1513.4423.

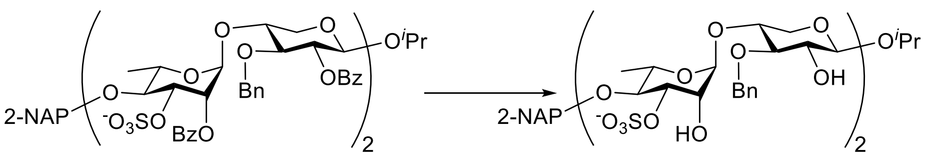

100 **104**

Compound 104. 5.0 M $NaOH_{(aq)}$ (1.4 mL) was added to a solution of compound **100** (176 mg, 113.0 μmol) in a co-solvent of $CHCl_3$/MeOH/H_2O (1.9/8.8/1.4 mL), and the reaction mixture was allowed to stir for 48 h at room temperature. AG 50W-X8 cation exchange resin was added to quench the reaction, and pH was adjusted to 7. The whole mixture was filtered through a pad of Celite®, solids were washed with MeOH, and concentrated *in vacuo*. The crude residue was purified by Sephadex LH-20 column followed by AG 50W-X8 cation exchange column (Na^+ form) using MeOH as an eluent to afford the desired compound **104** (0.113 mg, 88%) as a white solid. ^1H NMR (CD_3OD, 600 MHz) δ = 7.88-7.82 (m, 4 H, Ar-H), 7.60-7.58 (m, 1 H, Ar-H), 7.48-7.41 (m, 6 H, Ar-H), 7.32-7.30 (m, 2 H, Ar-H), 7.24-7.23 (m, 1 H, Ar-H), 6.99-6.98 (m, 3 H, Ar-H), 5.20 (ABq, J = 10.9 Hz, 1 H, $ArCH_2$), 4.93-4.70 (m, 9 H, H-1’, H-3’, H-

1''', H-3''', 5 x $ArCH_2$), 4.61 (d, *J* = 7.3 Hz, 1 H, H-1''), 4.33-4.31 (m, 2 H, H-1, H-2'), 4.20-4.19 (m, 1 H, H-2'''), 4.10-3.95 (m, 5 H, H-5a, H-5', H-5a'', H-5''', IPA-CH), 3.73-3.66 (m, 3 H, H-4, H-4'', H-4'''), 3.58-3.51 (m, 2 H, H-4', H-3''), 3.46-3.40 (m, 2 H, H-3, H-2''), 3.29-3.28 (m, 1 H, H-2), 3.25-3.21 (m, 2 H, H-5b, H-5b''), 1.20 (t, *J* = 5.8 Hz, 6 H, IPA-CH_3), 1.10-1.07 (dd, *J* = 6.3 Hz, *J* = 8.2 Hz, 6 H, H-6', H-6'''); ^{13}C NMR (CD_3OD, 150 MHz) δ = 139.6 (2C, C), 137.9 (C), 134.8 (C), 134.4 (C), 130.0 (2C, CH), 129.8 (2C, CH), 129.2 (2C, CH), 129.1 (2C, CH), 129.0 (CH), 128.7 (CH), 128.6 (2C, CH), 128.3 (CH), 128.1 (CH), 128.0 (CH), 126.8 (CH), 126.7 (CH), 105.7 (CH), 103.6 (CH), 98.7 (2C, CH), 84.2 (CH), 84.0 (CH), 80.7 (CH), 79.9 (CH), 79.6 (CH), 78.5 (CH), 76.8 (CH_2), 76.7 (CH_2), 76.4 (CH), 75.7 (CH_2), 75.6 (CH), 73.8 (CH), 73.7 (CH), 72.9 (CH), 70.8 (CH), 70.6 (CH), 69.0 (CH), 68.9 (CH), 63.6 (CH_2), 63.4 (CH_2), 23.8 (CH_3), 22.1 (CH_3), 18.2 (CH_3), 18.1 (CH_3); HRMS m/z $(M+H)^+$ calcd for $C_{50}H_{62}Na_2O_{23}S_2H^+$ 1141.2991, found 1141.2970.

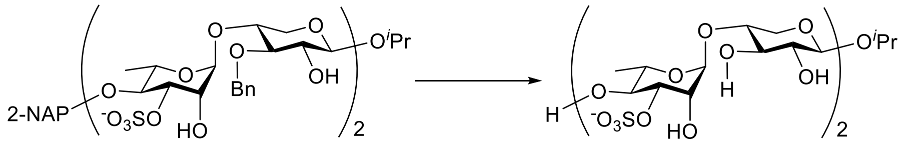

104 **10**

Compound 10. A mixture of compound **104** (10.2 mg, 9.0 μmol) and $Pd(OH)_2$ on activated charcoal (20% Pd, wet, Degussa type, 30.6 mg) in a mixed solvent of MeOH/H_2O (0.5 mL, 1/1) was purged with hydrogen for 10 min at room temperature. The reaction flask was then equipped with a hydrogen balloon. After stirring for 48 h, the whole mixture was filtered, and the filtrate was concentrated under reduced pressure. The crude residue was purified by Sephadex G-10 column followed by AG 50W-X8 cation exchange column (Na^+ form) using water as eluent to furnish the desired compound **10** (5.12 mg, 70%) as a white solid. ^{1}H NMR (D_2O, 600 MHz) δ = 4.90 (s, 2 H, H-1', H-1'''), 4.67 (d, *J* = 7.6 Hz, 1 H, H-1''), 4.64-4.62 (dd, *J* = 3.1 Hz, *J* = 9.3 Hz, 1 H, H-3'), 4.49 (d, *J* = 7.9 Hz, 1 H, H-1), 4.47-4.45 (dd, *J* = 3.3 Hz, *J* = 9.7 Hz, 1 H, H-3'''), 4.26-4.24 (m, 2 H, H-2', H-2'''), 4.11-4.03 (m, 5 H, H-5a, H-5', H-5a'', H-5''', IPA-CH), 3.82 (t, *J* = 9.4 Hz, 1 H, H-4'), 3.68-3.55 (m, 5 H, H-4, H-3, H-4'', H-3'', H-4'''), 3.29-3.29 (m, 3 H, H-5b, H-5b'', H-2''), 3.32-3.31 (dd, *J* = 8.1 Hz, *J* = 9.1 Hz, 1 H, H-2), 1.32 (d, *J* = 6.3 Hz, 3 H, H-6'), 1.30 (d, *J* = 6.2 Hz, 3 H, H-6'''), 1.21-1.18 (m, 6 H, IPA-CH_3); ^{13}C NMR (D_2O, 150 MHz) δ = 103.5 (CH), 101.1 (CH), 97.5 (CH), 97.4 (CH), 78.4 (CH), 78.2 (CH), 77.1 (CH), 73.9 (CH), 73.8 (2C, CH), 73.5 (CH), 73.3 (CH), 73.2 (CH), 73.0 (CH), 69.8 (CH), 68.8 (CH), 68.7 (CH), 68.6 (CH), 67.7 (CH), 62.3 (2C, CH_2), 22.2 (CH_3), 20.9 (CH_3), 16.8 (CH_3), 16.6 (CH_3); HRMS m/z $(M+H)^+$ calcd for $C_{25}H_{42}O_{23}S_2H_2$ 775.1642,

found 775.1633.

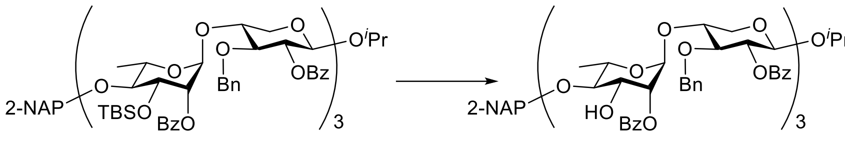

Compound 97. To a solution of compound **92** (143 mg, 63.0 μmol) in CH_3CN (14.0 mL, 100 mL/g**)**, BF_3/Et_2O (23.3 μL, 189.0 μmol) was added at ambient temperature. Then, the reaction mixture was allowed to stir at room temperature for 1 h, sat. $NaHCO_{3(aq)}$ was added to quench the reaction, pH was adjusted to neutral, and the whole mixture was directly extracted with EtOAc (2 x 100 mL). The combined organic layers were washed with brine, dried over anhydrous $MgSO_4$, filtered, and concentrated *in vacuo*. The crude residue was purified by flash column chromatography (EtOAc/Hex =1/2) on silica gel to afford the desired compound **97** (101 mg, 83%) as a white solid. $[\alpha]_D^{27.1}$ = -5.8041 (c = 0.449, $CHCl_3$); IR (thin film in KBr) ν 3493, 2925, 1724, 1602, 1452, 1316, 1268, 1107, 1028, 754, 711 cm^{-1}; ^{1}H NMR ($CDCl_3$, 600 MHz) δ = 8.02-7.98 (m, 6 H, Ar-H), 7.95-7.86 (m, 6 H, Ar-H), 7.81-7.74 (m, 4 H, Ar-H), 7.59-7.55 (m, 3 H, Ar-H), 7.53-7.37 (m, 15 H, Ar-H), 7.34-7.31 (m, 4 H, Ar-H), 7.10-7.04 (m, 11 H, Ar-H), 7.02-6.95 (m, 3 H, Ar-H), 5.28 (s, 1 H, H-2’’’’’), 5.19-5.10 (m, 5 H, H-2, H-2’, H-2’’, H-2’’’, H-2’’’’), 4.97 (s, 1 H, H-1’’’’’), 4.92-4.83 (m, 6 H, H-1’, H-1’’, H-1’’’, H-1’’’’, 2 x $ArCH_2$), 4.67 (ABq, *J* = 11.0 Hz, 1 H, $ArCH_2$), 4.60-4.49 (m, 5 H, H-1, 4 x $ArCH_2$), 4.45 (ABq, *J* = 11.1 Hz, 1 H, $ArCH_2$), 4.18-4.15 (m, 1 H, H-3’’’’’), 4.11-4.08 (dd, *J* = 4.6 Hz, *J* = 11.9 Hz, 1 H, H-5a’’’’), 4.06-4.01 (m, 2 H, H-5a, H-5a’’), 3.98-3.88 (m, 6 H, H-4, H-3’, H-4’’, H-3’’’, H-4’’’’, H-5’’’’’), 3.86-3.80 (m, 3 H, H-5’, H-5’’’, IPA-CH), 3.66 (t, *J* = 8.3 Hz, 1 H, H-3’’’’), 3.63-3.54 (m, 4 H, H-3, H-4’, H-3’’, H-4’’’), 3.48 (t, *J* = 9.4 Hz, *J* = 9.5 Hz, 1 H, H-4’’’’’), 3.36-3.32 (dd, *J* = 8.9 Hz, *J* = 11.8 Hz, 1 H, H-5b’’’’), 3.28-3.24 (m, 2 H, H-5b, H-5b’’), 2.33-2.30 (m, 2 H, OH), 2.11 (d, *J* = 5.2 Hz, 1 H, OH), 1.26 (d, *J* = 6.1 Hz, 3 H, H-6’’’’’), 1.15-1.11 (m, 9 H, H-6’, H-6’’’, IPA-CH_3), 0.97 (d, *J* = 6.1 Hz, 3 H, IPA-CH_3); ^{13}C NMR ($CDCl_3$, 150 MHz) δ = 166.52 (C), 166.5 (2C, C), 165.3 (2C, C), 165.2 (C), 137.5 (2C, C), 137.4 (C), 137.3 (C), 135.7 (C), 133.7 (CH), 133.6 (CH), 133.5 (CH), 133.4 (CH), 133.22 (C), 133.2 (CH), 130.2 (C), 130.1 (CH), 130.0 (CH), 129.9 (CH), 129.8 (CH), 129.7 (2C, C), 129.7 (C), 129.63 (C), 129.6 (C), 128.8 (CH), 128.7 (CH), 128.54 (CH), 128.53 (CH), 128.5 (CH), 128.45 (CH), 128.4 (CH), 128.1 (CH), 127.94 (CH), 127.9 (CH), 127.8 (CH), 126.9 (CH), 126.4 (CH), 126.2 (CH), 126.1 (CH), 101.7 (CH), 101.6 (CH), 100.3 (CH), 95.0 (CH), 94.9

(2C, CH), 81.6 (CH), 80.6 (CH), 80.5 (CH), 79.63 (CH), 79.6 (CH), 79.3 (CH), 77.4 (CH), 75.3 (CH_2), 75.2 (CH_2), 75.0 (CH_2), 74.9 (CH_2), 73.4 (CH), 73.3 (CH), 73.2 (CH), 73.1 (CH), 73.0 (CH), 72.9 (CH), 72.7 (CH), 72.3 (CH), 70.5 (CH), 70.33 (CH), 70.3 (CH), 68.2 (CH), 67.3 (CH), 67.2 (CH), 62.27 (CH_2), 62.2 (CH_2), 62.1 (CH_2), 23.4 (CH_3), 22.0 (CH_3), 18.3 (CH_3), 17.8 (CH_3), 17.7 (CH_3); HRMS m/z $(M+H)^+$ calcd for $C_{110}H_{112}O_{31}H^+$ 1930.7294, found 1930.7281.

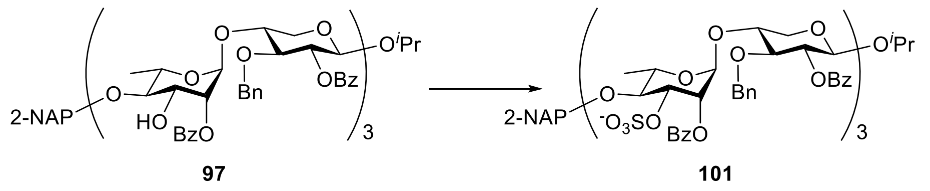

Compound 101. To a solution of compound **97** (90.8 mg, 47.0 μmol) in anhydrous DMF (1.8 mL), $SO_3{\cdot}Et_3N$ (143 mg, 1.4 mmol) was added under a nitrogen atmosphere at room temperature. Then, the reaction mixture was heated to 60 °C and allowed to stir at same temperature for 24 h. The reaction flask was cooled down to room temperature, sat. $NaHCO_{3(aq)}$ solution was added, and the mixture was allowed to stir for 24 h. The resulting mixture was extracted with EtOAc, and the combined organic layers were washed with brine, dried over anhydrous $MgSO_4$, filtered and concentrated *in vacuo*. The crude residue was purified by AG 50W-X8 cation exchange column (Na^+ form) followed by Sephadex LH-20 column using MeOH as an eluent to afford the desired product **101** (85.4 mg, 81%) as a white solid. ^{1}H NMR (CD_3OD, 600 MHz) δ = 8.07-8.02 (m, 8 H, Ar-H), 7.98-7.95 (m, 2 H, Ar-H), 7.92-7.89 (m, 3 H, Ar-H), 7.85-7.82 (m, 3 H, Ar-H), 7.64-7.59 (m, 5 H, Ar-H), 7.53-7.45 (m, 10 H, Ar-H), 7.40-7.36 (m, 2 H, Ar-H), 7.30-7.24 (m, 4 H, Ar-H), 7.07 (d, *J* = 7.4 Hz, 1 H, Ar-H), 6.99-6.86 (m, 11 H, Ar-H), 6.77 (t, *J* = 7.6 Hz, 1 H, Ar-H), 5.71 (s, 1 H, H-2’’’’’), 5.63 (s, 1 H, H-2’’’), 5.59 (s, 1 H, H-2’), 5.35 (d, *J* = 7.6 Hz, 1 H, H-1’’’’), 5.25-5.22 (m, 2 H, H-1’’, $ArCH_2$), 5.10-5.07 (m, 3 H, H-2’’’’, H-1’’’’’, H-3’’’’’), 5.04-5.00 (m, 3 H, H-1’, H-2’’, H-1’’’), 4.93-4.76 (m, 5 H, H-2, H-3’, H-3’’’, 2 x $ArCH_2$), 4.56 (d, *J* = 7.9 Hz, 1 H, H-1), 4.50-4.42 (m, 3 H, $ArCH_2$), 4.35-4.31 (m, 2 H, $ArCH_2$), 4.24-4.21 (dd, *J* = 4.7 Hz, *J* = 11.8 Hz, 1 H, H-5a’’’’), 4.17-4.14 (dd, *J* = 5.1 Hz, *J* = 11.7 Hz, 1 H, H-5a’’), 4.11-4.03 (m, 4 H, H-5a, H-4’, H-4’’’, H-5’’’’’), 3.95-3.79 (m, 7 H, H-4, H-5’, H-4’’, H-5’’’, H-3’’’’, H-4’’’’, IPA-CH), 3.71-3.65 (m, 3 H, H-3, H-3’’, H-4’’’’’), 3.49 (t, *J* = 10.9 Hz, *J* = 10.5 Hz, 1 H, H-5b’’’’), 3.39 (t, *J* = 10.7 Hz, *J* = 11.0 Hz, 1 H, H-5b’’), 3.32-3.29 (m, 1 H, H-5b), 1.17-1.14 (m, 9 H, H-6’, H-6’’’, H-6’’’’’), 1.09 (d, *J* = 6.2 Hz, 3 H, IPA-CH_3), 0.93 (d, *J* = 6.1 Hz, 3 H, IPA-CH_3); ^{13}C NMR (CD_3OD, 150 MHz) δ = 167.7 (2C, C), 167.6 (C), 167.24 (C), 167.2 (C),

166.8 (C), 138.8 (C), 138.73 (C), 138.7 (C), 137.5 (C), 134.7 (C), 134.5 (C), 134.43 (CH), 134.4 (CH), 134.3 (CH), 134.0 (CH), 133.8 (CH), 131.3 (CH), 131.27 (3C, C), 131.2 (C), 131.15 (C), 131.1 (C), 130.9 (CH), 130.86 (CH), 130.8 (CH), 130.7 (CH), 129.8 (CH), 129.6 (CH), 129.5 (CH), 129.3 (CH), 129.2 (CH), 129.1 (CH), 129.0 (CH), 128.9 (CH), 128.7 (CH), 128.6 (CH), 128.5 (CH), 128.4 (CH), 128.3 (CH), 128.1 (CH), 126.8 (CH), 126.7 (CH), 101.7 (CH), 101.1 (CH), 101.0 (CH), 96.3 (CH), 96.2 (CH), 96.0 (CH), 82.0 (CH), 81.3 (CH), 81.0 (CH), 79.7 (CH), 77.8 (CH), 77.4 (CH), 77.3 (CH), 77.0 (CH_2), 76.4 (CH_2), 76.2 (CH_2), 75.7 (CH_2), 75.6 (CH), 75.4 (CH), 75.3 (CH), 75.2 (CH), 75.1 (CH), 74.7 (CH), 73.6 (CH), 73.3 (CH), 72.7 (CH), 72.6 (CH), 69.1 (CH), 68.7 (CH), 63.7 (2C, CH_2), 63.6 (CH_2), 23.5 (CH_3), 22.3 (CH_3), 18.5 (CH_3), 18.41 (CH_3), 18.4 (CH_3); HRMS m/z $(M+H)^+$ calcd for $C_{110}H_{109}O_{40}S_3H_3H^+$ 1083.7890, found 1083.7832.

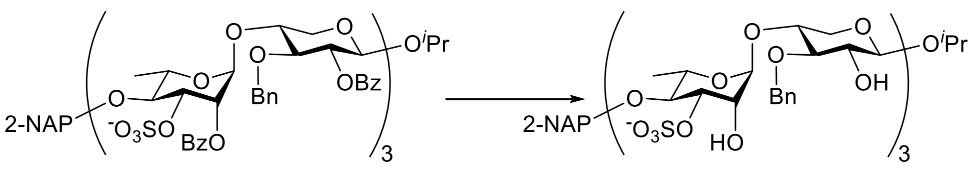

101 **105**

Compound 105. 5.0 M $NaOH_{(aq)}$ (374.0 μL) was added to a solution of compound **101** (46.5 mg, 21.0 μmol) in a co-solvent of $CHCl_3/MeOH/H_2O$ (0.3/2.4/0.4 mL) and allowed to stir for 48 h at room temperature. AG 50W-X8 cation exchange resin was added to quench the reaction, and pH was adjusted to 7. The whole mixture was filtered through a pad of Celite®, washed with MeOH, and concentrated *in vacuo*. The crude residue was purified by Sephadex LH-20 column followed by AG 50W-X8 cation exchange column (Na^+ form) using MeOH as an eluent to afford the desired compound **105** (30.5 mg, 91%) as a white solid. ^1H NMR (600 MHz, CD_3OD) δ = 7.87 (s, 1 H, Ar-H), 7.85-7.82 (m, 3 H, Ar-H), 7.60-7.58 (m, 1 H, Ar-H), 7.51-7.44 (m, 6 H, Ar-H), 7.42-7.41 (m, 2 H, Ar-H), 7.32 (t, *J* = 7.5 Hz, *J* = 7.7 Hz, 4 H, Ar-H), 7.25-7.23 (m, 2 H, Ar-H), 7.00-6.98 (m, 3 H, Ar-H), 5.20 (ABq, *J* = 10.9 Hz, 1 H, $ArCH_2$), 4.97-4.69 (m, 11 H, H-1', H-3', H-1''', H-3''', H-1''''', H-3''''', 5 x $ArCH_2$), 4.62-4.59 (m, 4 H, H-1'', H-1'''', 2 x $ArCH_2$), 4.33-4.31 (m, 2 H, H-1, H-2'''''), 4.20-4.19 (m, 2 H, H-2', H-2'''), 4.10-3.95 (m, 7 H, H-5a, H-5', H-5a'', H-5''', H-5a'''', H-5''''', IPA-CH), 3.73-3.66 (m, 5 H, H-4, H-4', H-4'', H-4''', H-4''''), 3.58-3.52 (m, 3 H, H-3'', H-3'''', H-4'''''), 3.46-3.41 (m, 3 H, H-3, H-2'', H-2''''), 3.34-3.28 (m, 1 H, H-2), 3.25-3.20 (m, 3 H, H-5b, H-5b'', H-5b''''), 1.20 (t, *J* = 5.7 Hz, 6 H, IPA- CH_3), 1.10-1.07 (m, 9 H, H-6', H-6''', H-6'''''); ^{13}C NMR (150 MHz, CD_3OD) δ = 139.7 (C), 139.6 (2C, C), 137.9 (C), 134.8 (C), 134.4 (C), 130.0

(CH), 129.8 (CH), 129.2 (CH), 129.1 (CH), 129.0 (CH), 128.7 (CH), 128.6 (CH), 128.3 (CH), 128.1 (CH), 128.0 (CH), 126.8 (CH), 126.6 (CH), 105.8 (CH), 105.7 (CH), 103.7 (CH), 98.8 (CH), 98.7 (CH), 84.3 (CH), 84.1 (CH), 84.0 (CH), 80.7 (CH), 79.9 (CH), 79.6 (CH), 78.6 (CH), 78.5 (CH), 76.9 (CH_2), 76.7 (CH_2), 76.6 (CH_2), 76.44 (CH), 76.4 (CH), 75.6 (2C, CH, CH_2), 73.9 (CH), 73.8 (CH), 73.7 (CH), 72.9 (CH), 70.8 (CH), 70.6 (CH), 69.1 (CH), 69.0 (CH), 68.9 (CH), 63.6 (CH_2), 63.4 (CH_2), 23.8 (CH_3), 22.1 (CH_3), 18.2 (CH_3), 18.17 (CH_3), 18.16 (CH_3); HRMS m/z $(M+H)^+$ calcd for $C_{68}H_{85}O_{34}S_3H_3H^+$ 513.8034, found 513.8038.

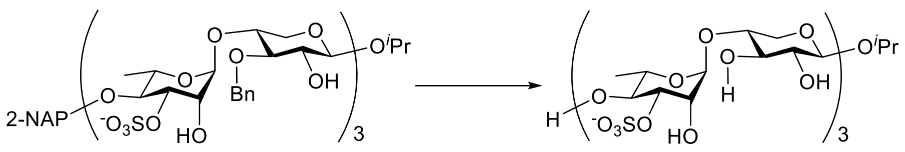

105 **11**

Compound 11. A mixture of hexa-saccharide **105** (39.1 mg, 0.024 mmol) and $Pd(OH)_2$ on activated charcoal (20% Pd, wet, Degussa type, 117 mg) in a mixed solvent of MeOH/H_2O (0.5 mL, 1/1) was purged with hydrogen for 10 minutes at room temperature. The reaction flask was then equipped with a hydrogen balloon, after stirring for 48 h, the whole mixture was filtered, and the solution was concentrated under reduced pressure. The crude residue was purified by Sephadex G-10 column followed by AG 50W-X8 cation exchange column (Na^+ form) using water as eluent to furnish the desired compound **11** (21.6 mg, 74%) as a white solid. ^1H NMR (600 MHz, D_2O) δ = 4.90 (m, 3 H, H-1', H-1''', H-1'''''), 4.66-4.62 (m, 4 H, H-3', H-1'', H-3''', H-1''''), 4.49-4.55 (m, 2 H, H-1, H-3'''''), 4.25 (s, 3 H, H-2', H-2''', H-2'''''), 4.10-4.02 (m, 7 H, H-5a, H-5', H-5a'', H-5''', H-5a'''', H-5''''', IPA-CH), 3.83-3.80 (m, 2 H, H-4', H-4'''), 3.68-3.54 (m, 7 H, H-3, H-4, H-3'', H-4'', H-3'''', H-4'''', H-4'''''), 3.38-3.28 (m, 5 H, H-5b, H-2'', H-5b'', H-2'''', H-5b''''), 3.22 (t, J = 8.9 Hz, J = 8.3 Hz, 1 H, H-2), 1.32-1.29 (m, 9 H, H-6', H-6''', H-6'''''), 1.21-1.18 (m, 6 H, IPA- CH_3); ^{13}C NMR (150 MHz, D_2O) δ = 103.5 (2C, CH), 101.1 (CH), 97.5 (CH), 97.4 (2C, CH), 78.4 (CH), 78.2 (2C, CH), 77.2 (CH), 77.1 (CH), 74.0 (CH), 73.9 (CH), 73.84 (CH), 73.8 (CH), 73.5 (2C, CH), 73.3 (2C, CH), 73.2 (CH), 72.9 (CH), 69.8 (CH), 68.8 (CH), 68.7 (2C, CH), 68.6 (CH), 67.7 (2C, CH), 62.3 (3C, CH_2), 22.2 (CH_3), 20.9 (CH_3), 16.9 (2C, CH_3), 16.6 (CH_3); HRMS m/z $(M+3Na)^+$ calcd for $C_{36}H_{59}O_{34}S_3Na_3H^+$ 1201.1816, found 1201.1840.

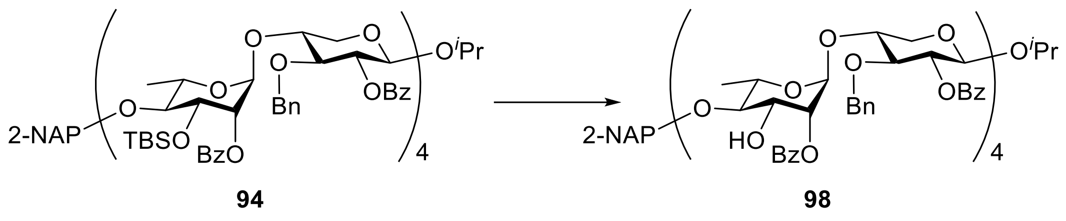

Compound 98. To a solution of compound **94** (102 mg, 34.0 μmol) in CH_3CN (10.2 mL), $BF_3·Et_2O$ (12.7 μL, 51.0 μmol) was added at room temperature. The reaction mixture was allowed to stir at room temperature for 7 h. Then, sat. $NaHCO_{3(aq)}$ was added to quench the reaction, pH was adjusted to neutral, and the whole mixture was directly extracted with EtOAc (2 × 100 mL). The combined organic layers were washed with brine, dried over anhydrous $MgSO_4$, filtered, and concentrated *in vacuo*. The resulting crude residue was purified by flash column chromatography (EtOAc/Hex =1/2) on silica gel to afford the desired compound **98** (67.8 mg, 80%) as a white solid. $[\alpha]_D^{30}$ = -15.4 (*c* = 0.5, $CHCl_3$); IR (thin film in KBr) ν 2923, 1723, 1451, 1267, 1097, 1069, 1028, 711 cm^{-1}; ^{1}H NMR ($CDCl_3$, 600 MHz) δ = 8.02-7.97 (m, 8 H, Ar-H), 7.95 (d, *J* = 7.5 Hz, 2 H, Ar-H), 7.91 (d, *J* = 7.5 Hz, 2 H, Ar-H), 7.86 (t, *J* = 7.5 Hz, *J* = 7.6 Hz, 4 H, Ar-H), 7.80-7.74 (m, 4 H, Ar-H), 7.58-7.55 (m, 4 H, Ar-H), 7.53-7.37 (m, 17 H, Ar-H), 5.28 (s, 1 H, H-2’’’’’’’), 5.19-5.09 (m, 7 H, H-2, H-2’, H-2’’, H-2’’’, H-2’’’’, H-2’’’’’, H-2’’’’’’), 4.97 (s, 1 H, H-1’’’’’’’), 4.92-4.82 (m, 8 H, H-1, H-1’, H-1’’’, H-1’’’’’, 4 x $ArCH_2$), 4.67 (d, *J* = 10.9 Hz, 1 H, $ArCH_2$), 4.59-4.43 (m, 8 H, H-1’’, H-1’’’’, H-1’’’’’’, 5 x $ArCH_2$), 4.17-4.16 (m, 1 H, H-3’’’’’’’), 4.11-4.08 (dd, *J* = 4.3 Hz, *J* = 12.2 Hz, 1 H, H-5a), 4.06-4.00 (m, 3 H, H-5a’’, H-5a’’’’, H-5a’’’’’’), 3.98-3.77 (m, 12 H, H-4, H-3’, H-5’, H-4’’, H-3’’’, H-5’’’, H-4’’’’, H-3’’’’’, H-5’’’’’, H-4’’’’’’, H-5’’’’’’’, IPA-CH), 3.66 (t, *J* = 8.3 Hz, 1 H, H-3), 3.63-3.54 (m, 6 H, H-4’, H-3’’, H-4’’’, H-3’’’’, H-4’’’’’, H-3’’’’’’), 3.48 (t, *J* = 9.5 Hz, *J* = 9.4 Hz, 1 H, H-4’’’’’’’), 3.36-3.32 (dd, *J* = 9.1 Hz, *J* = 11.6 Hz, 1 H, H-5b), 3.29-3.24 (m, 3 H, H-5b’’, H-5b’’’’, H-5b’’’’’’), 2.34-2.30 (3 H, OH), 2.12 (1 H, OH), 1.26 (d, *J* = 6.1 Hz, 3 H, H-6’’’’’’’), 1.15-1.10 (m, 12 H, H-6’, H-6’’’, H-6’’’’’, IPA-CH_3), 0.97 (d, *J* = 6.1 Hz, 3 H, IPA-CH_3); ^{13}C NMR ($CDCl_3$, 150 MHz) δ = 166.5 (3C, C), 165.3 (C), 165.2 (C), 137.5 (C), 137.4 (C), 137.3 (2C, C), 135.7 (C), 133.7 (CH), 133.5 (CH), 133.4 (CH), 133.22 (2C, C), 133.2 (CH), 130.2 (CH), 130.1 (CH), 130.0 (C), 129.9 (CH), 129.8 (CH), 129.75 (2C, C), 129.7 (C), 129.68 (C), 129.63 (C), 129.6 (2C, C), 128.8 (CH), 128.7 (CH), 128.5 (CH), 128.49 (CH), 128.45 (CH), 128.4 (CH), 128.0 (CH), 127.95 (CH), 127.9 (CH), 127.8 (CH), 126.9 (CH), 126.4 (CH), 126.2 (CH), 126.1 (CH), 101.8 (CH), 101.7 (CH), 101.6 (CH), 100.3 (CH), 95.0 (CH), 94.9 (3C, CH), 81.6 (CH), 80.6 (CH), 80.5 (CH), 79.63 (CH), 79.6 (CH), 79.4 (CH), 79.3 (CH), 77.4 (CH),

75.3 (CH_2), 75.2 (CH_2), 75.0 (2C, CH_2), 74.9 (CH_2), 73.4 (CH), 73.3 (CH), 73.2 (CH), 73.14 (CH), 73.1 (CH), 73.0 (CH), 72.9 (CH), 72.7 (CH), 72.3 (CH), 70.5 (CH), 70.3 (CH), 70.2 (CH), 68.2 (CH), 67.3 (CH), 67.2 (CH), 62.3 (2C, CH_2), 62.2 (2C, CH_2), 23.4 (CH_3), 22.0 (CH_3), 18.3 (CH_3), 17.8 (CH_3), 17.76 (CH_3), 17.7 (CH_3); HRMS m/z $(M+Na)^+$ calcd for $C_{142}H_{144}O_{41}Na^+$ 2528.9109, found 2528.9109.

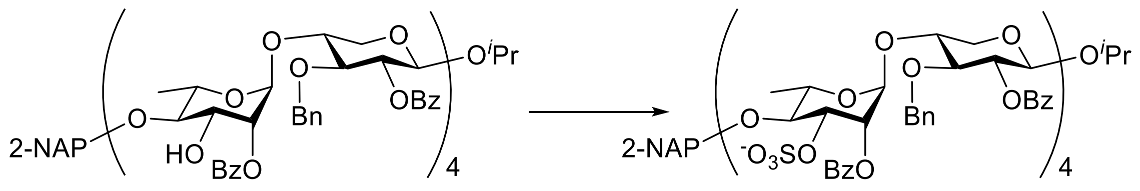

98 102

Compound 102. To a solution of compound **98** (67.8 mg, 27.0 μmol) in anhydrous DMF (1.3 mL), $SO_3{\cdot}Et_3N$ (109 mg, 1.08 mmol) was added under a nitrogen atmosphere at room temperature. Then, the reaction mixture was heated to 60 °C and allowed to stir at same temperature for 12 h. The reaction flask was cooled down to room temperature, sat. $NaHCO_{3(aq)}$ solution was added, and the mixture was allowed to stir for 24 h. The resulting mixture was extracted with EtOAc, and the combined organic layers were washed with brine, dried over anhydrous $MgSO_4$, filtered, and concentrated *in vacuo* to afford the desired compound **102**, which was directly used for next reaction without further purification. HRMS m/z $(M+H)^+$ calcd for $C_{142}H_{140}O_{53}S_4H_4H^+$ 2826.7562, found 2826.7565.

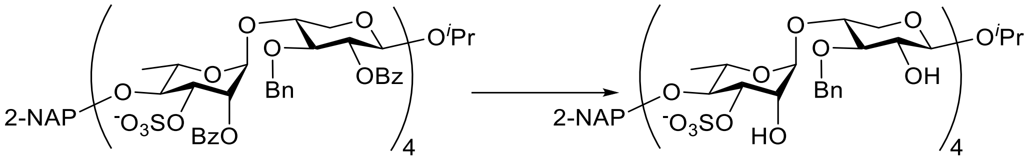

102 106

Compound 106. To a solution of compound **102** (30.2 mg, 10.0 μmol) in a co-solvent $CHCl_3/MeOH/H_2O$ (0.35/1.5/0.24 mL), 5.0 M $NaOH_{(aq)}$ (247 μL) was added, and the reaction mixture was allowed to stir for 48 h at room temperature. AG 50W-X8 cation exchange resin was added to quench the reaction, and pH was adjusted to 7. The whole mixture was filtered through a pad of Celite®, solids were washed with MeOH, and concentrated *in vacuo*. The crude residue was purified by Sephadex LH-20 column, followed by AG 50W-X8 cation exchange column (Na^+ form) using MeOH as an eluent, to afford the desired compound **106** (18.9 mg, 67% in two

steps) as a white solid. ^{1}H NMR (600 MHz, CD_3OD) δ = 7.88 (s, 1 H, Ar-H), 7.85-7.82 (m, 3 H, Ar-H), 7.60-7.58 (m, 1 H, Ar-H), 7.51-7.44 (m, 8 H, Ar-H),7.42-7.41 (m, 2 H, Ar-H), 7.34-7.31 (m, 6 H, Ar-H), 7.25-7.22 (m, 3 H, Ar-H), 6.99-6.98 (m, 3 H, Ar-H), 5.20 (d, J = 10.7 Hz, 1 H, $ArCH_2$), 4.97 (d, J = 10.2 Hz, 1 H, $ArCH_2$), 4.93-4.69 (m, 15 H, H-1', H-3', H-1''', H-3''', H-1''''', H-3''''', H-1''''''', H-3''''''', 7 x $ArCH_2$), 4.62-4.60 (m, 3 H, H-1'', H-1'''', H-1''''''), 4.33-4.31 (m, 2 H, H-1, H-2'''''''), 4.20-4.19 (m, 3 H, H-2', H-2''', H-2'''''), 4.11-4.06 (m, 1 H, H-5'''''''), 4.04-3.94 (m, 8 H, H-5a, H-5', H-5a'', H-5''', H-5a'''', H-5''''', H-5a'''''', IPA-CH), 3.75-3.66 (m, 7 H, H-4, H-4', H-4'', H-4''', H-4'''', H-4''''', H-4''''''), 3.58-3.52 (m, 4 H, H-3'', H-3'''', H-3'''''', H-4'''''''), 3.46-3.41 (m, 4 H, H-3, H-2'', H-2'''', H-2''''''), 3.29-3.28 (m, 1 H, H-2), 3.25-3.21 (m, 4 H, H-5b, H-5b'', H-5b'''', H-5b''''''), 1.20 (t, J = 5.8 Hz, 6 H, H-6''''''', IPA-CH_3), 1.10-1.07 (m, 12 H, H-6', H-6''', H-6''''', IPA-CH_3); ^{13}C NMR (150 MHz, CD_3OD) δ = 139.7 (2C, C), 139.6 (2C, C), 137.9 (C), 134.8 (C), 134.4 (C), 130.2 (CH), 130.0 (CH), 129.8 (CH), 129.2 (CH), 129.1 (CH), 129.0 (CH), 128.7 (CH), 128.8 (CH), 128.6 (CH), 128.3 (CH), 128.1 (CH), 128.03 (CH), 126.8 (CH), 126.6 (CH), 105.7 (CH), 103.7 (CH), 98.8 (CH), 98.7 (CH), 84.3 (CH), 84.1 (CH), 84.0 (CH), 80.7 (CH), 79.9 (CH), 79.6 (CH), 78.6 (CH), 78.5 (CH), 76.9 (CH_2), 76.7 (CH_2), 76.6 (CH_2), 76.4 (CH_2), 75.6 (2C, CH_2, CH), 73.8 (CH), 73.7 (CH), 72.9 (CH), 70.8 (CH), 70.6 (CH), 69.01 (CH), 69.0 (CH), 68.9 (CH), 63.6 (CH_2), 63.4 (3C, CH_2), 23.8 (CH_3), 22.1 (CH_3), 18.2 (CH_3), 18.1 (3C, CH_3); HRMS m/z $(M+H)^+$ calcd for $C_{86}H_{108}O_{45}S_4H_4H^+$ 995.2606, found 995.2602.

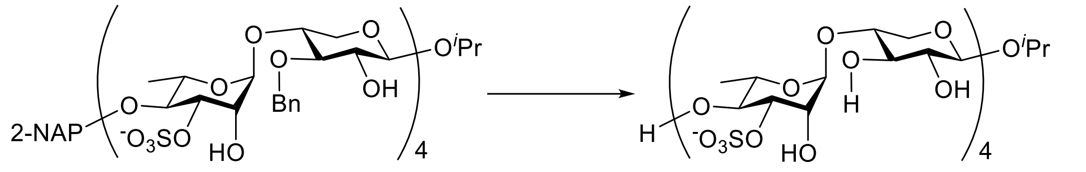

106 **12**

Compound 12. A mixture of the octasaccharide **106** (17.6 mg, 8.45 μmol) and $Pd(OH)_2$ on activated charcoal (20% Pd, wet, Degussa type, 52.8 mg) in a mixed solvent of $MeOH/H_2O$ (0.5 mL, 1/1) was purged with hydrogen for 10 min at room temperature. The reaction flask was then equipped with a hydrogen balloon, after stirring for 48 h, the whole mixture was filtered, and the solution was concentrated under reduced pressure. The crude residue was purified by Sephadex G-10 column followed by AG 50W-X8 cation exchange column (Na^+ form) using water as an eluent to furnish the desired compound **12** (9.1 mg, 68%) as a white

solid. ^{1}H NMR (600 MHz, D_2O) δ = 4.91-4.90 (m, 4 H, H-1', H-1''', H-1''''', H-1'''''''), 4.66-4.62 (m, 6 H, H-3', H-1'', H-3''', H-1'''', H-3''''', H-1''''''), 4.49-4.45 (m, 2 H, H-1, H-3'''''''), 4.25 (s, 4 H, H-2', H-2''', H-2''''', H-2'''''''), 4.10-4.03 (m, 9 H, H-5a, H-5', H-5a'', H-5''', H-5a'''', H-5''''', H-5a'''''', H-5''''''', IPA-CH), 3.82 (t, *J* = 9.3 Hz, *J* = 9.4 Hz, 3 H, H-4', H-4''', H-4'''''), 3.67-3.61 (m, 7 H, H-4, H-3'', H-4'', H-3'''', H-4'''', H-3'''''', H-4''''''), 3.59-3.55 (m, 2 H, H-3, H-4'''''''), 3.38-3.29 (m, 7 H, H-5b, H-2'', H-5b'', H-2'''', H-5b'''', H-2'''''', H-5b''''''), 3.21 (t, *J* = 8.6 Hz, *J* = 8.7 Hz, 1 H, H-2), 1.32-1.29 (m, 12 H, H-6', H-6''', H-6''''', H-6'''''''), 1.22 (d, *J* = 6.2 Hz, 3 H, IPA-CH_3), 1.19 (d, *J* = 6.1 Hz, 3 H, IPA-CH_3); ^{13}C NMR (150 MHz, D_2O) δ = 103.5 (3C, CH), 101.1 (CH), 97.5 (CH), 97.4 (3C, CH), 78.4 (CH), 78.2 (3C, CH), 77.1 (3C, CH), 74.0 (CH), 73.98 (CH), 73.9 (CH), 73.84 (CH), 73.8 (CH), 73.5 (3C, CH), 73.3 (CH), 73.2 (CH), 73.0 (CH), 69.8 (CH), 68.8 (CH), 68.7 (2C, CH), 68.6 (CH), 67.7 (4C, CH), 62.3 (4C, CH_2), 22.3 (CH_3), 20.9 (CH_3), 16.9 (3C, CH_3), 16.6 (CH_3); HRMS m/z $(M+H)^+$ calcd for $C_{47}H_{76}Na_4O_{45}S_4H^+$ 1581.2205, found 1581.2243.

5. Crystallographic Data for Compound 82.

Table S1. Crystal data and structure refinement for i18010.

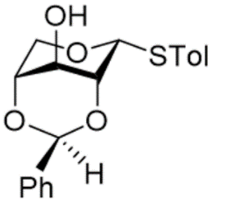

82
CCDC 2363767

Identification code	i18010	
Empirical formula	$C_{19}H_{20}O_4S$	
Formula weight	344.41	
Temperature	100.0(2) K	
Wavelength	0.71073 Å	
Crystal system	Monoclinic	
Space group	P 21	
Unit cell dimensions	a = 9.1283(3) Å	α = 90°.
	b = 7.5794(2) Å	β = 96.608(2)°.
	c = 12.2259(4) Å	γ = 90°.
Volume	840.25(4) $Å^3$	
Z	2	
Density (calculated)	1.361 Mg/m^3	
Absorption coefficient	0.213 mm^{-1}	
F(000)	364	
Crystal size	0.122 x 0.079 x 0.076 mm^3	
Theta range for data collection	2.954 to 30.494°.	
Index ranges	-12<=h<=13, -10<=k<=10, -17<=l<=17	
Reflections collected	33471	
Independent reflections	5125 [R(int) = 0.0947]	
Completeness to theta = 25.242°	99.8 %	
Absorption correction	Numerical	
Max. and min. transmission	1 and 0.955	
Refinement method	Full-matrix least-squares on F^2	
Data / restraints / parameters	5125 / 1 / 222	
Goodness-of-fit on F^2	1.056	
Final R indices [I>2sigma(I)]	R1 = 0.0427, wR2 = 0.0775	
R indices (all data)	R1 = 0.0755, wR2 = 0.0905	
Absolute structure parameter	-0.06(4)	
Extinction coefficient	n/a	
Largest diff. peak and hole	0.311 and -0.371 e.$Å^{-3}$	

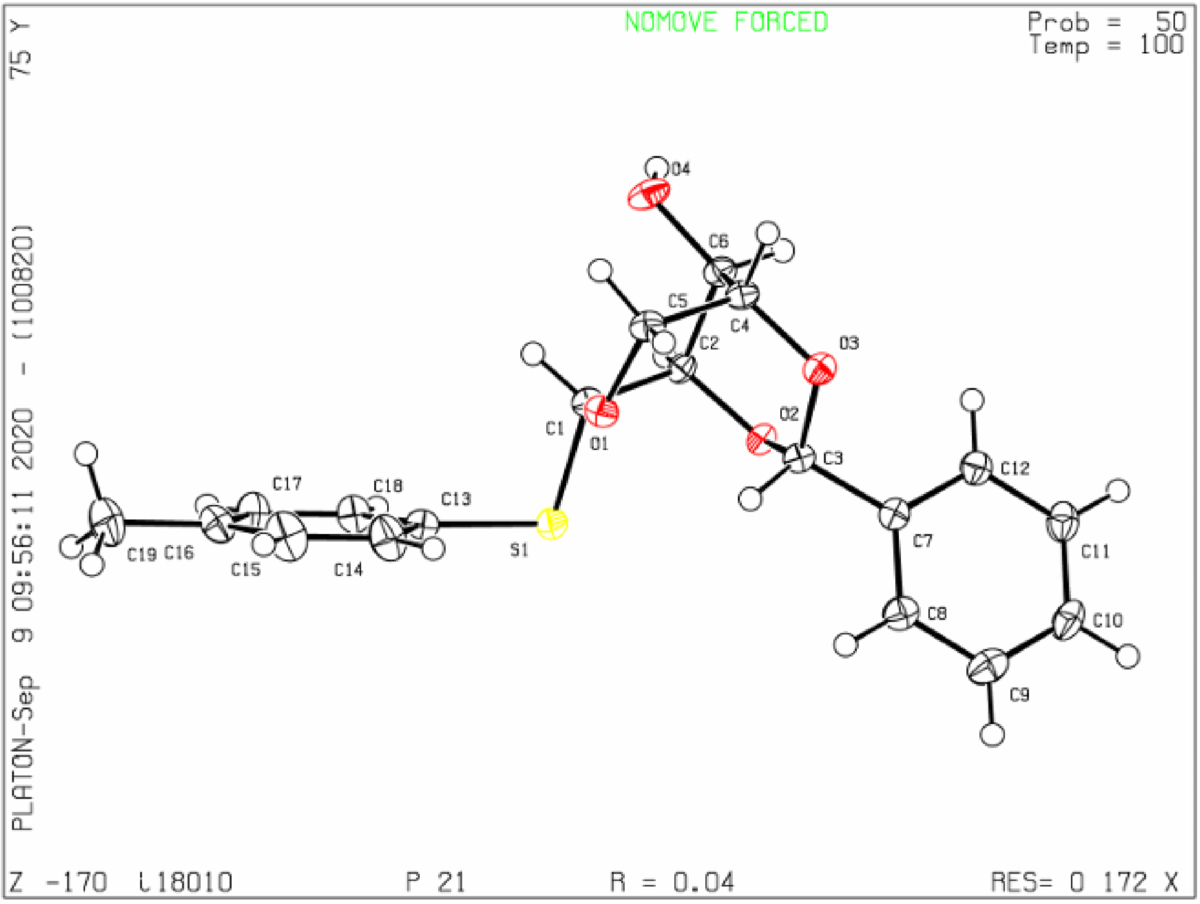

Figure S2. ORTEP drawing of **82** (CCDC 2363767, 50% thermal probability).

6. Biological Procedures.

Evaluation of immunomodulatory effects. The mouse macrophage cell line RAW264.7 (ATCC TIB-71) was maintained at 37 °C with 5% CO_2 in DMEM supplemented with 10% fetal bovine serum, 100 U of penicillin/mL, and 100 µg of streptomycin/mL (Sigma). RAW cells were plated at 4×10^5 cells in wells of 24-well plates (Greiner Bio-One) in supplemented DMEM one day prior to treatment. The cells were treated with **1-12** at 10, 50 and 250 µM for 24 h. Following the treatment, the medium was collected for G-CSF quantitation using the Quantikine mouse ELISA kits (R&D Systems) according to the manufacturer's protocol. Alternatively, the cells were collected, and the total RNAs of each sample were prepared by using GeneJet RNA purification (Thermo Scientific). The total RNAs of each sample were then reverse-transcribed into cDNA by using (oligodT) 20 primers and reverse transcriptase (Thermo Scientific), and the gene-specific expression was monitored using polymerase chain reaction. The amplified gene fragments were analyzed on a 1% agarose gel. The results shown are representative of three independent experiments.

(A)

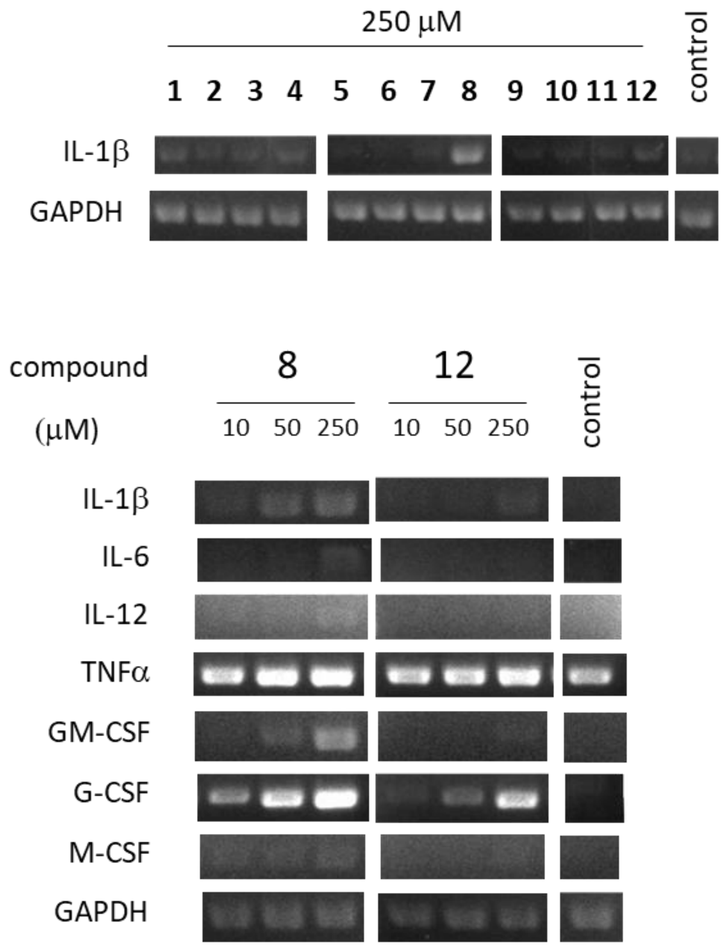

Figure S3. (A) Gene expression of IL-1β in the cells treated with 250 μM of **1**–**12**. **(B)** Gene expression of cytokines in the cells treated with various concentration of **8** and **12.**

7. NMR Spectra for the Synthesis of $A_{3'S}$ Libraries

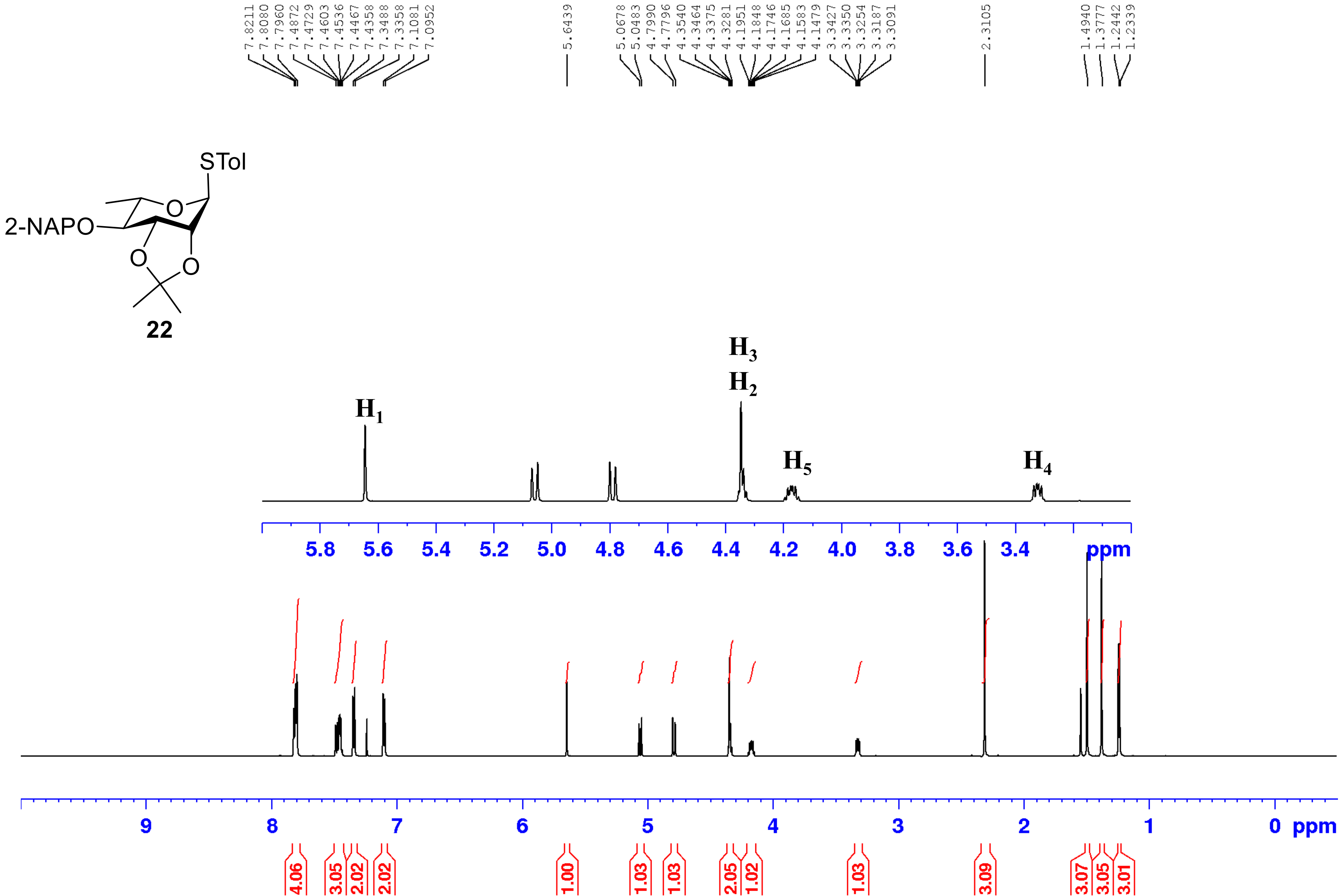

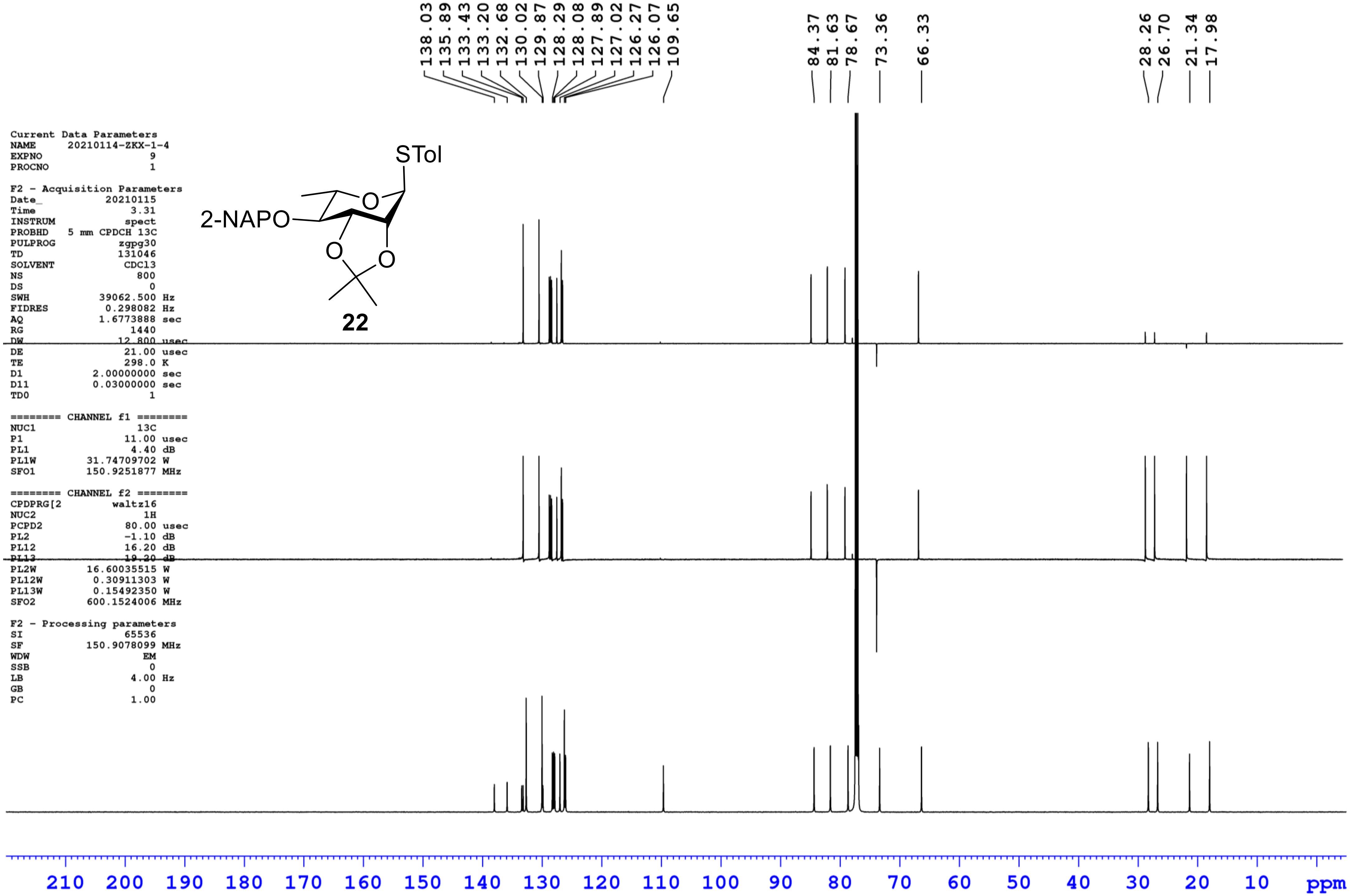
138.03
135.89
133.43
133.20
132.68
130.02
129.87
128.29
128.08
127.89
127.02
126.27
126.07
109.65
84.37
81.63
78.67
73.36
66.33
28.26
26.70
21.34
17.98
Current Data Parameters
NAME 20210114-ZKX-1-4
EXPNO 9
PROCNO 1
F2 - Acquisition Parameters
Date_ 20210115
Time 3.31
INSTRUM spect
PROBHD 5 mm CPDCH 13C
PULPROG zgpg30
TD 131046
SOLVENT CDCl3
NS 800
DS 0
SWH 39062.500 Hz
FIDRES 0.298082 Hz
AQ 1.6773888 sec
RG 1440
DW 12.800 usec
DE 21.00 usec
TE 298.0 K
D1 2.00000000 sec
D11 0.03000000 sec
TD0 1
======== CHANNEL f1 ========
NUC1 13C
P1 11.00 usec
PL1 4.40 dB
PL1W 31.74709702 W
SFO1 150.9251877 MHz
======== CHANNEL f2 ========
CPDPRG[2 waltz16
NUC2 1H
PCPD2 80.00 usec
PL2 -1.10 dB
PL12 16.20 dB
PL13 19.20 dB
PL2W 16.60035515 W
PL12W 0.30911303 W
PL13W 0.15492350 W
SFO2 600.1524006 MHz
F2 - Processing parameters
SI 65536
SF 150.9078099 MHz
WDW EM
SSB 0
LB 4.00 Hz
GB 0
PC 1.00
STol
2-NAPO
O
O
O
22
210 200 190 180 170 160 150 140 130 120 110 100 90 80 70 60 50 40 30 20 10 ppm

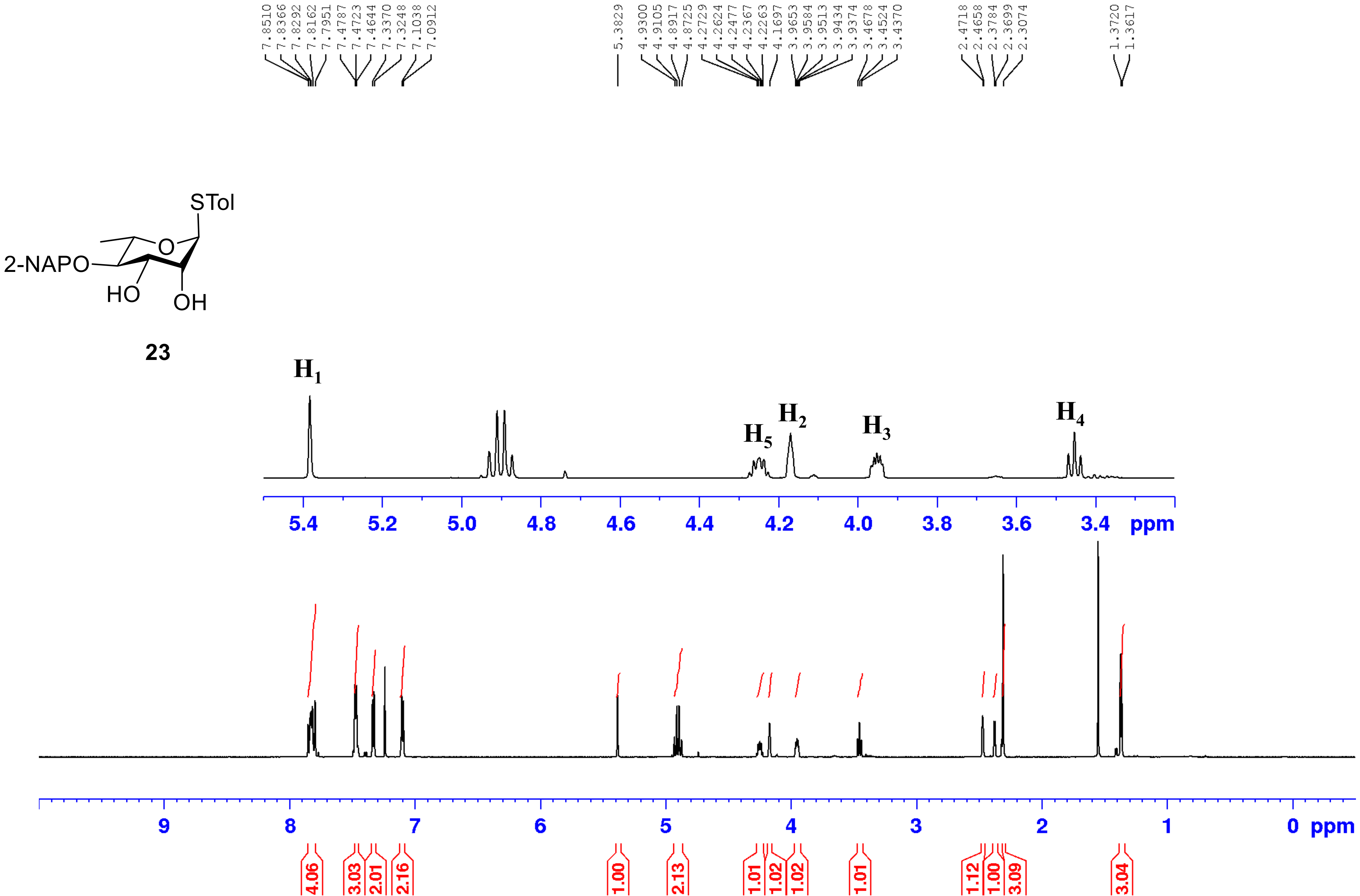
7.8510
7.8366
7.8292
7.8162
7.7951
7.4787
7.4723
7.4644
7.3370
7.3248
7.1038
7.0912
5.3829
4.9300
4.9105
4.8917
4.8725
4.2729
4.2624
4.2477
4.2367
4.2263
4.1697
3.9653
3.9584
3.9513
3.9434
3.9374
3.4678
3.4524
3.4370
2.4718
2.4658
2.3784
2.3699
2.3074
1.3720
1.3617
STol
O
2-NAPO
HO
OH
H1
H5
H2
H3
H4
5.4
5.2
5.0
4.8
4.6
4.4
4.2
4.0
3.8
3.6
3.4
ppm
9
8
7
6
5
4
3
2
1
0
ppm
4.06
3.03
2.01
2.16
1.00
2.13
1.01
1.02
1.02
1.01
1.12
1.00
3.09
3.04

23

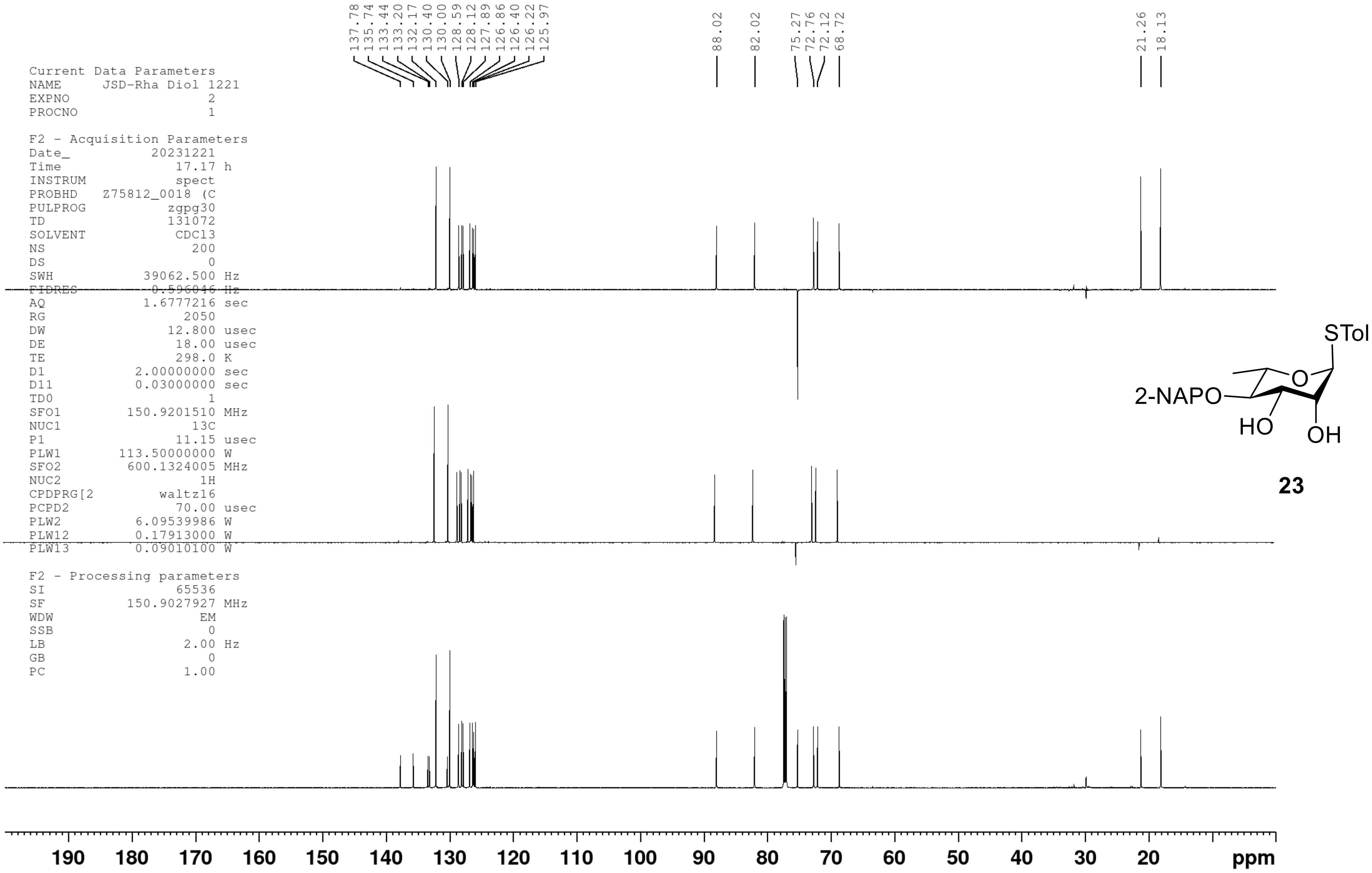
137.78
135.74
133.44
133.20
132.17
130.40
130.00
128.59
128.12
127.89
126.86
126.40
126.22
125.97
88.02
82.02
75.27
72.76
72.12
68.72
21.26
18.13
Current Data Parameters
NAME JSD-Rha Diol 1221
EXPNO 2
PROCNO 1
F2 - Acquisition Parameters
Date_ 20231221
Time 17.17 h
INSTRUM spect
PROBHD Z75812_0018 (C
PULPROG zgpg30
TD 131072
SOLVENT CDCl3
NS 200
DS 0
SWH 39062.500 Hz
FIDRES 0.596046 Hz
AQ 1.6777216 sec
RG 2050
DW 12.800 usec
DE 18.00 usec
TE 298.0 K
D1 2.00000000 sec
D11 0.03000000 sec
TD0 1
SFO1 150.9201510 MHz
NUC1 13C
P1 11.15 usec
PLW1 113.50000000 W
SFO2 600.1324005 MHz
NUC2 1H
CPDPRG[2 waltz16
PCPD2 70.00 usec
PLW2 6.09539986 W
PLW12 0.17913000 W
PLW13 0.09010100 W
F2 - Processing parameters
SI 65536
SF 150.9027927 MHz
WDW EM
SSB 0
LB 2.00 Hz
GB 0
PC 1.00
190 180 170 160 150 140 130 120 110 100 90 80 70 60 50 40 30 20 ppm
STol
O
2-NAPO
HO
OH

23

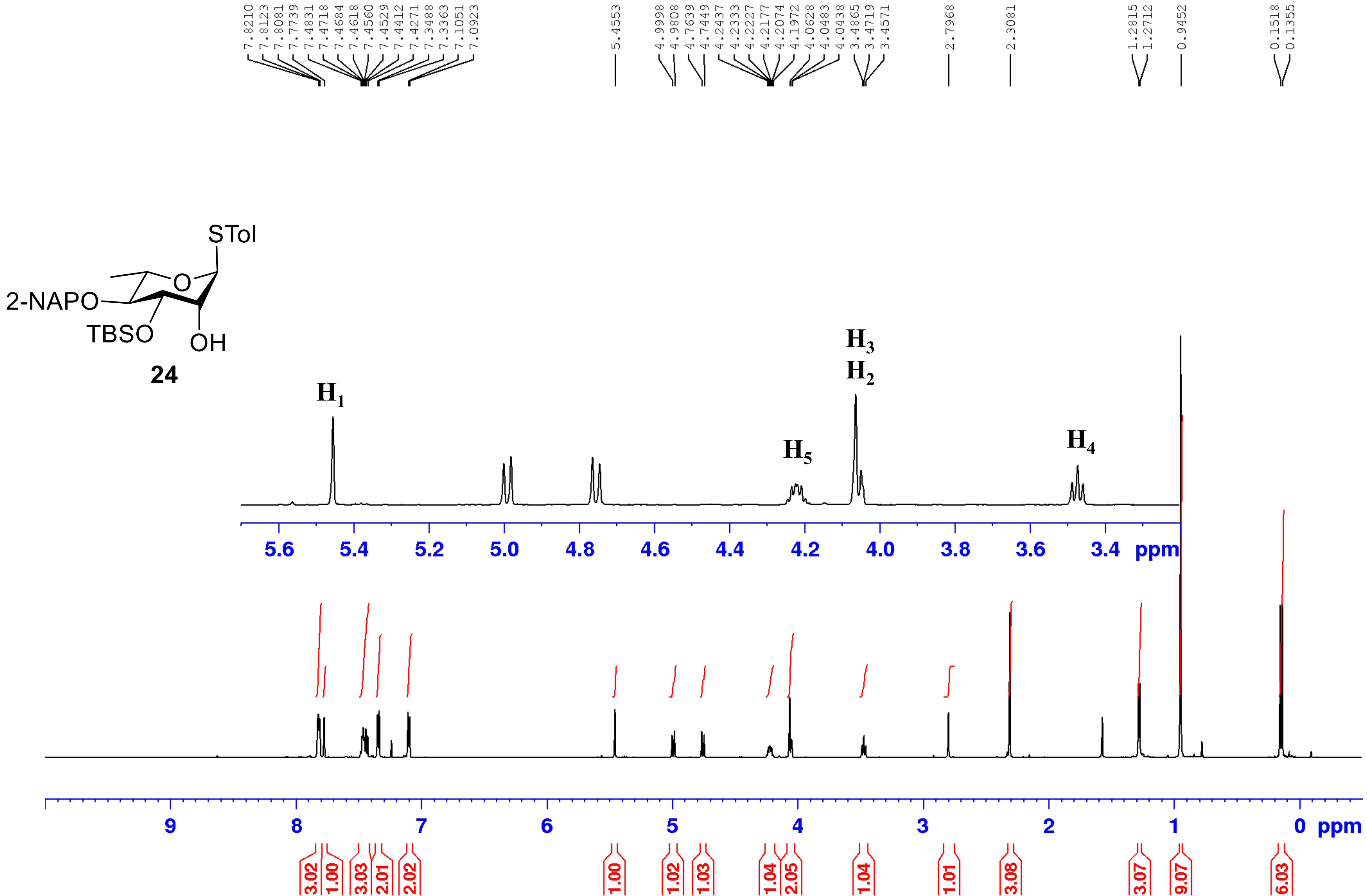

STol
2-NAPO
TBSO
OH
O
24
H1
H3
H2
H5
H4
ppm

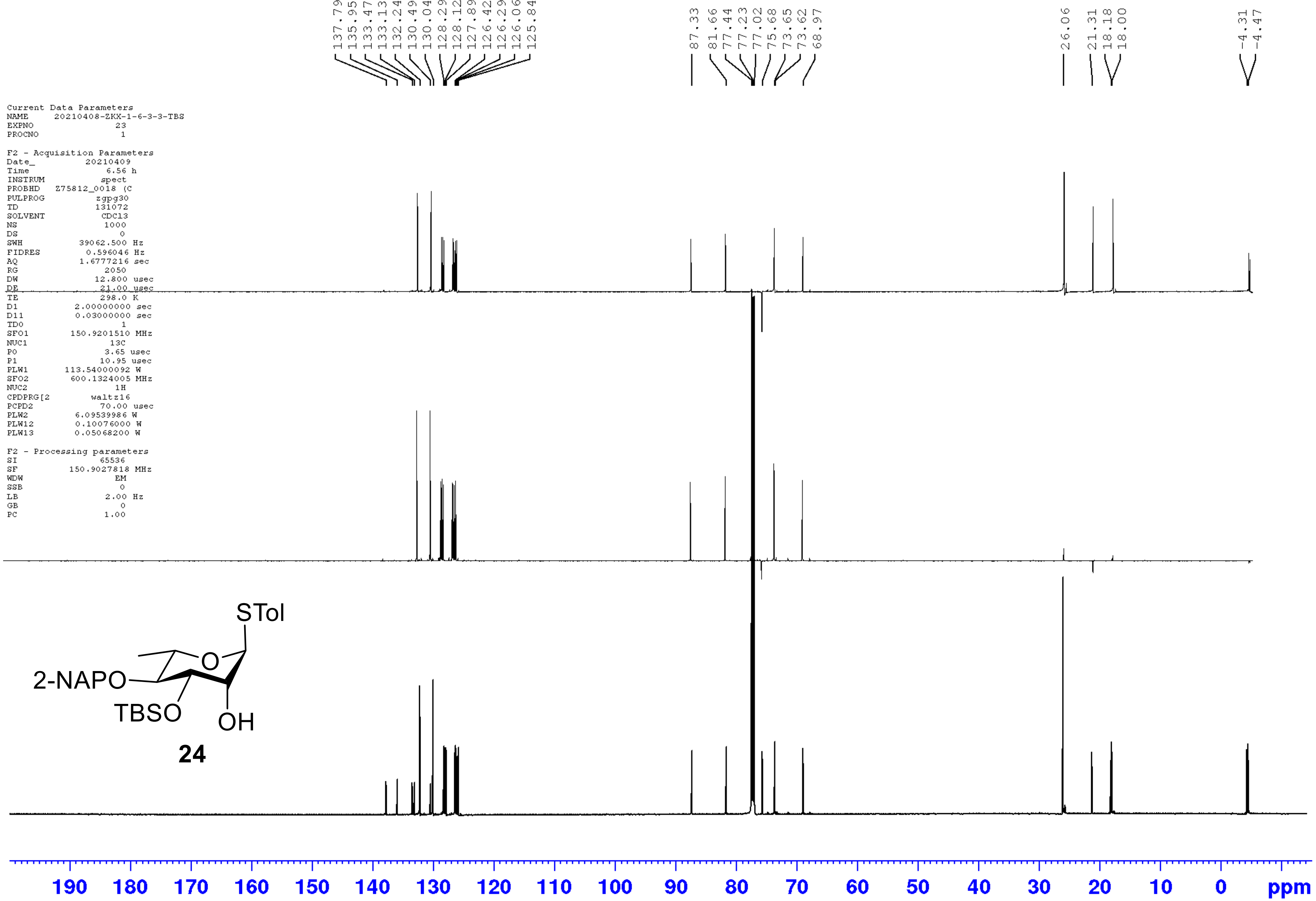
137.79
135.95
133.47
133.13
132.24
130.49
130.04
128.29
128.12
127.89
126.42
126.29
126.06
125.84
87.33
81.66
77.44
77.23
77.02
75.68
73.65
73.62
68.97
26.06
21.31
18.18
18.00
-4.31
-4.47
Current Data Parameters
NAME 20210408-ZKX-1-6-3-3-TBS
EXPNO 23
PROCNO 1
F2 - Acquisition Parameters
Date_ 20210409
Time 6.56 h
INSTRUM spect
PROBHD Z75812_0018 (C
PULPROG zgpg30
TD 131072
SOLVENT CDCl3
NS 1000
DS 0
SWH 39062.500 Hz
FIDRES 0.596046 Hz
AQ 1.6777216 sec
RG 2050
DW 12.800 usec
DE 21.00 usec
TE 298.0 K
D1 2.00000000 sec
D11 0.03000000 sec
TD0 1
SFO1 150.9201510 MHz
NUC1 13C
P0 3.65 usec
P1 10.95 usec
PLW1 113.54000092 W
SFO2 600.1324005 MHz
NUC2 1H
CPDPRG[2 waltz16
PCPD2 70.00 usec
PLW2 6.09539986 W
PLW12 0.10076000 W
PLW13 0.05068200 W
F2 - Processing parameters
SI 65536
SF 150.9027818 MHz
WDW EM
SSB 0
LB 2.00 Hz
GB 0
PC 1.00
STol
2-NAPO
O
TBSO
OH
24
190 180 170 160 150 140 130 120 110 100 90 80 70 60 50 40 30 20 10 0 ppm

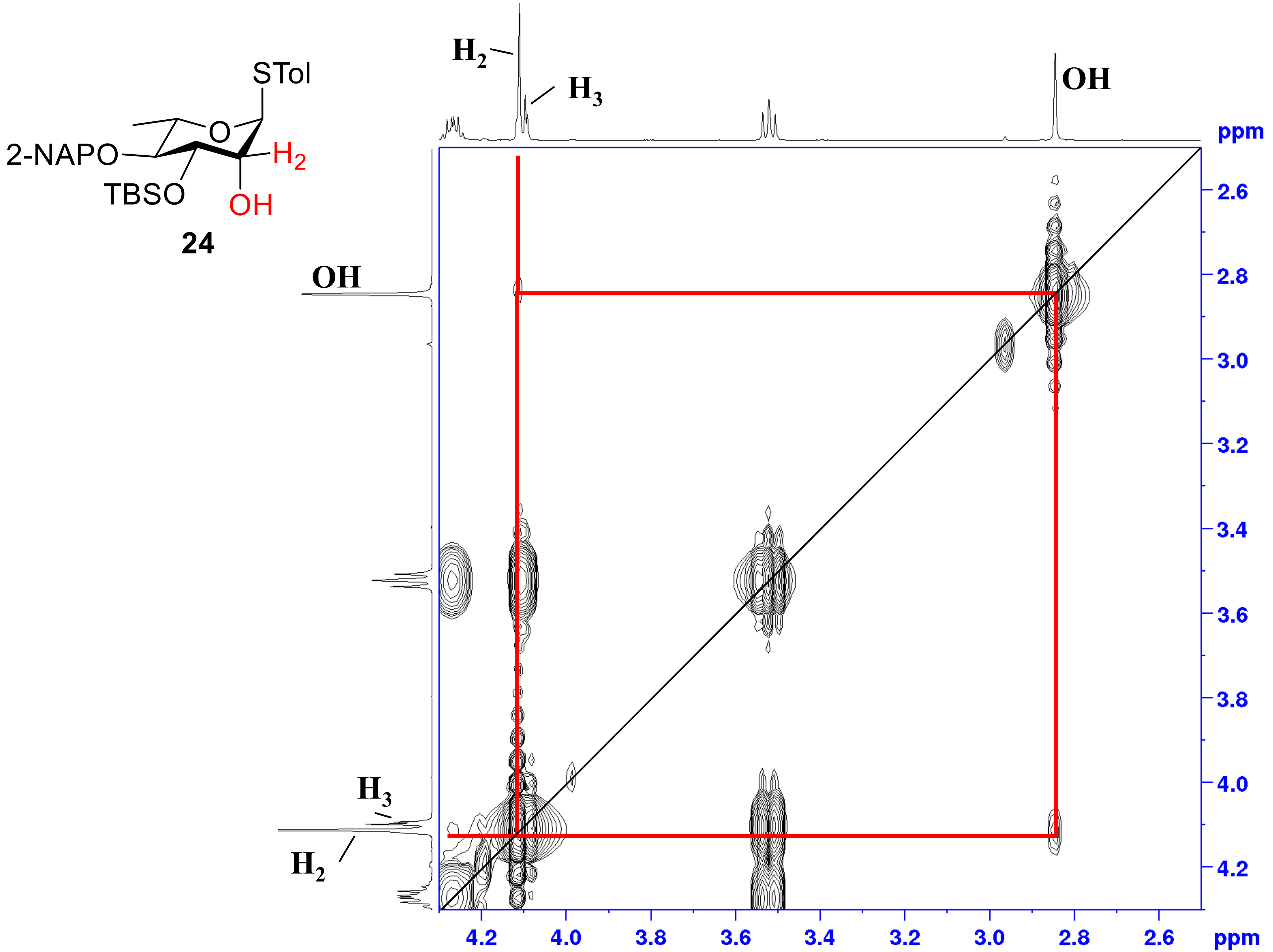

STol
O
2-NAPO
H2
TBSO
OH
24
H2
H3
OH
ppm
OH
H3
H2
2.6
2.8
3.0
3.2
3.4
3.6
3.8
4.0
4.2
ppm
4.2
4.0
3.8
3.6
3.4
3.2
3.0
2.8
2.6
ppm

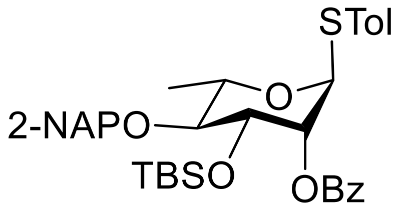
STol
O
2-NAPO
TBSO
OBz

16

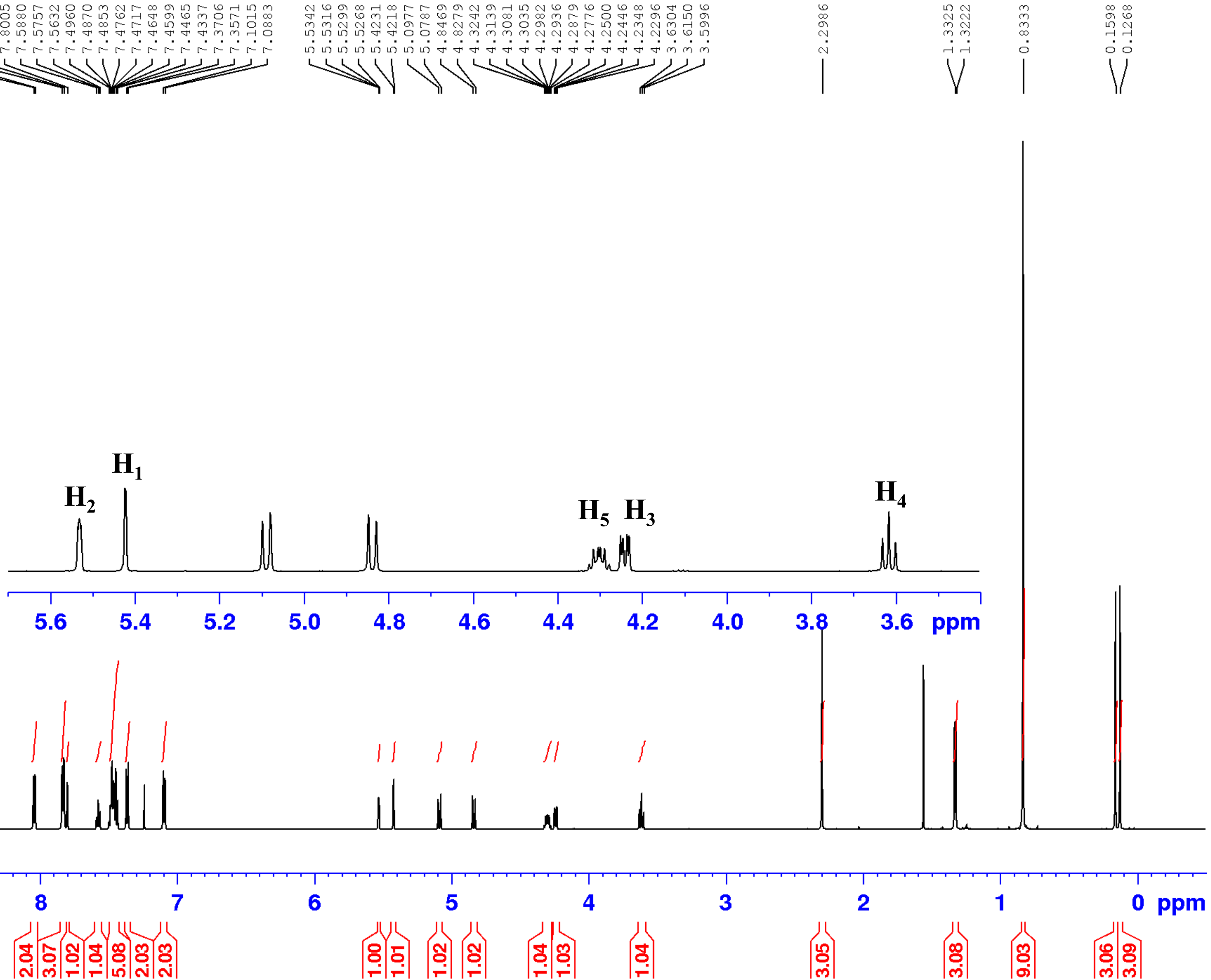
8.0470 8.0348 7.8389 7.8252 7.8005 7.5880 7.5757 7.5632 7.4960 7.4870 7.4853 7.4762 7.4717 7.4648 7.4599 7.4465 7.4337 7.3706 7.3571 7.1015 7.0883
5.5342 5.5316 5.5299 5.5268 5.4231 5.4218 5.0977 5.0787 4.8469 4.8279 4.3242 4.3139 4.3081 4.3035 4.2982 4.2936 4.2879 4.2776 4.2500 4.2446 4.2348 4.2296 3.6304 3.6150 3.5996
2.2986
1.3325 1.3222
0.8333
0.1598 0.1268
H_2 H_1 H_5 H_3 H_4
5.6 5.4 5.2 5.0 4.8 4.6 4.4 4.2 4.0 3.8 3.6 ppm
9 8 7 6 5 4 3 2 1 0 ppm
2.04 3.07 1.02 1.04 5.08 2.03 2.03 1.00 1.01 1.02 1.02 1.04 1.03 1.04 3.05 3.08 9.03 3.06 3.09

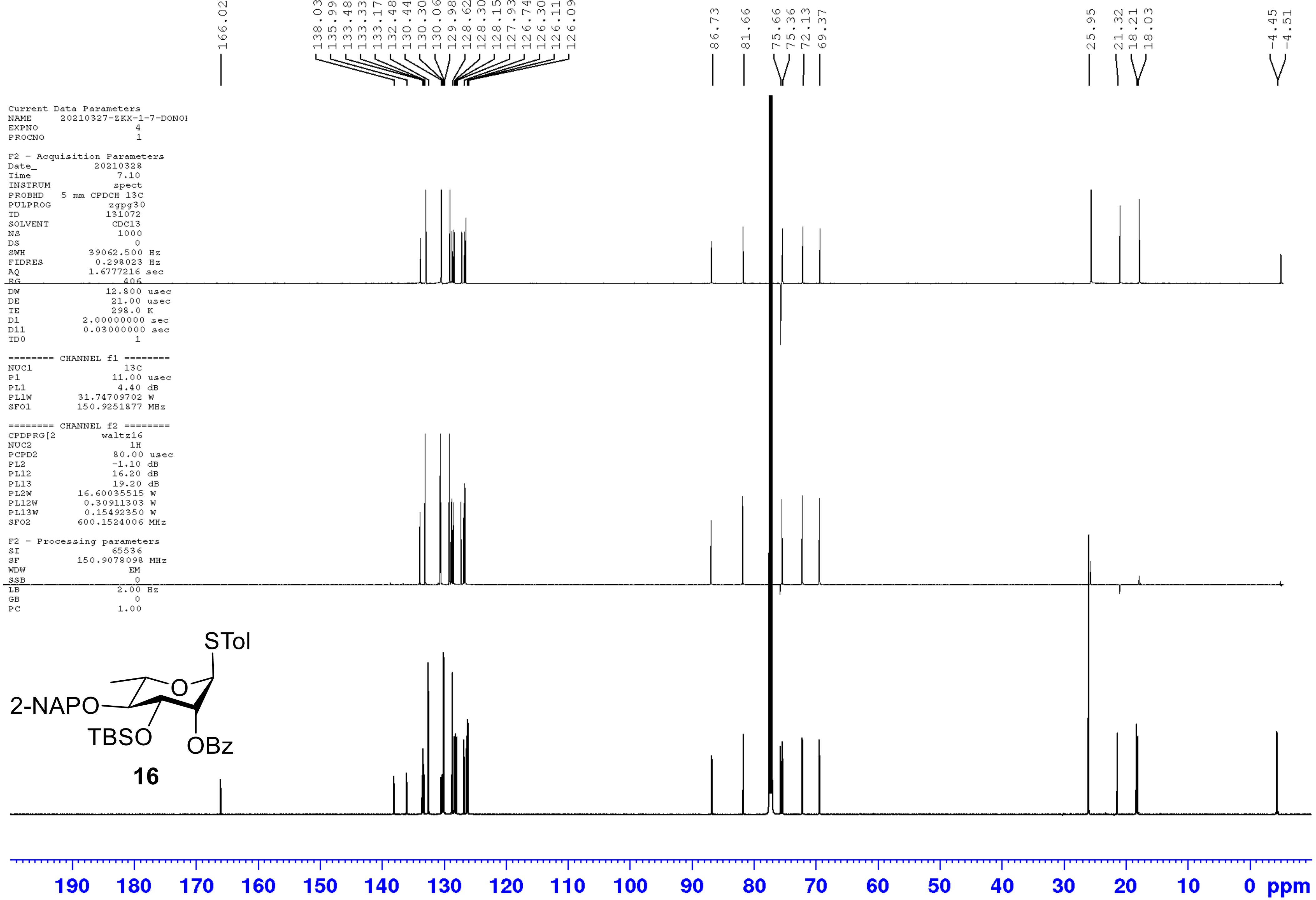
166.02
138.03
135.99
133.48
133.33
133.17
132.48
130.44
130.30
130.06
129.98
128.62
128.30
128.15
127.93
126.74
126.30
126.11
126.09
86.73
81.66
75.66
75.36
72.13
69.37
25.95
21.32
18.21
18.03
-4.45
-4.51
Current Data Parameters
NAME 20210327-ZKX-1-7-DONOI
EXPNO 4
PROCNO 1
F2 - Acquisition Parameters
Date_ 20210328
Time 7.10
INSTRUM spect
PROBHD 5 mm CPDCH 13C
PULPROG zgpg30
TD 131072
SOLVENT CDCl3
NS 1000
DS 0
SWH 39062.500 Hz
FIDRES 0.298023 Hz
AQ 1.6777216 sec
RG 406
DW 12.800 usec
DE 21.00 usec
TE 298.0 K
D1 2.00000000 sec
D11 0.03000000 sec
TD0 1
======== CHANNEL f1 ========
NUC1 13C
P1 11.00 usec
PL1 4.40 dB
PL1W 31.74709702 W
SFO1 150.9251877 MHz
======== CHANNEL f2 ========
CPDPRG[2 waltz16
NUC2 1H
PCPD2 80.00 usec
PL2 -1.10 dB
PL12 16.20 dB
PL13 19.20 dB
PL2W 16.60035515 W
PL12W 0.30911303 W
PL13W 0.15492350 W
SFO2 600.1524006 MHz
F2 - Processing parameters
SI 65536
SF 150.9078098 MHz
WDW EM
SSB 0
LB 2.00 Hz
GB 0
PC 1.00
STol
O
2-NAPO
TBSO
OBz
190 180 170 160 150 140 130 120 110 100 90 80 70 60 50 40 30 20 10 0 ppm

16

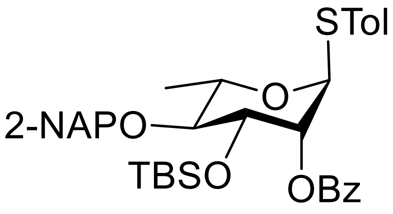
STol
O
2-NAPO
TBSO
OBz

16

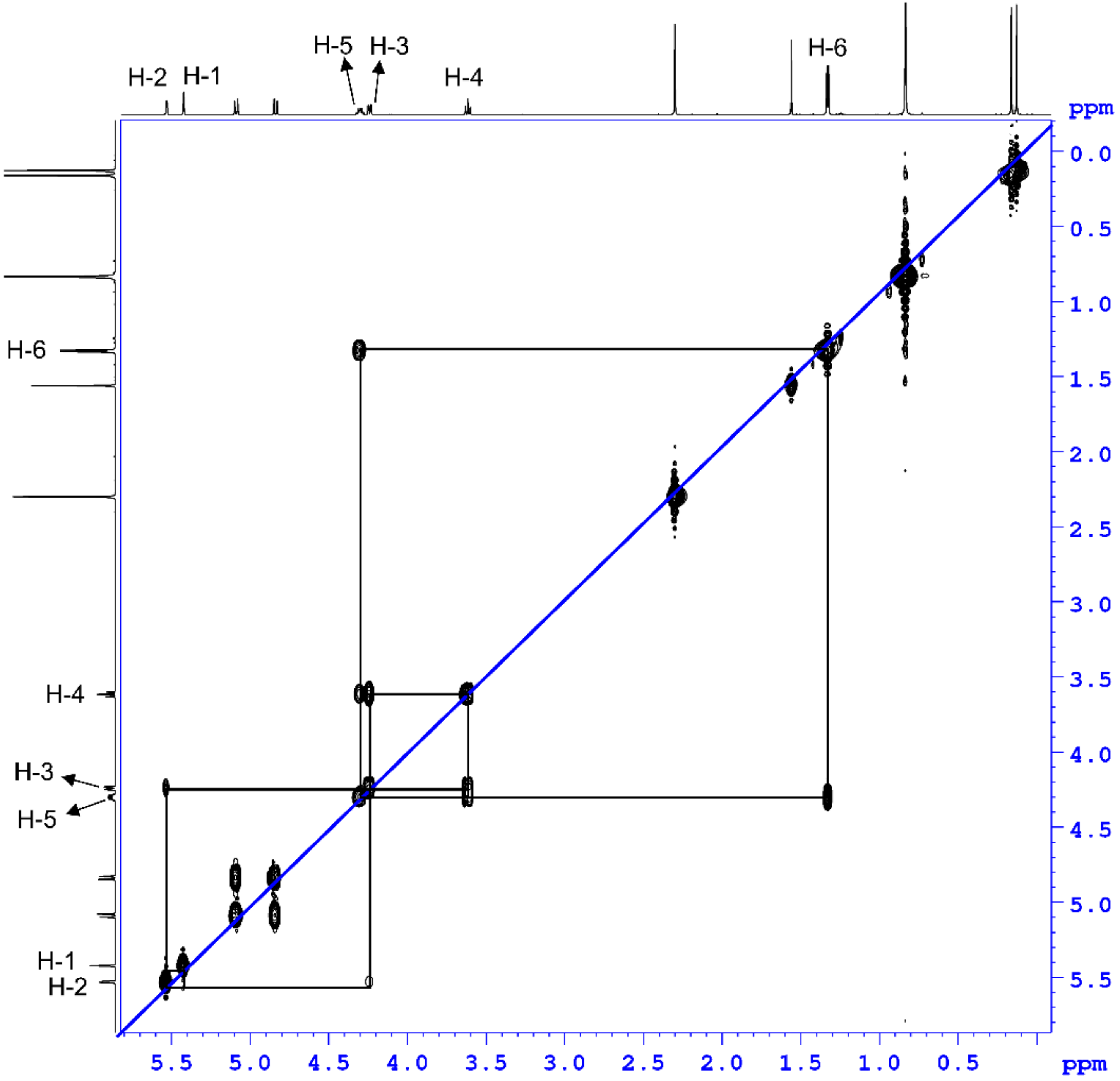
H-2
H-1
H-5
H-3
H-4
H-6
ppm
H-6
H-4
H-3
H-5
H-1
H-2
0.0
0.5
1.0
1.5
2.0
2.5
3.0
3.5
4.0
4.5
5.0
5.5
5.5 5.0 4.5 4.0 3.5 3.0 2.5 2.0 1.5 1.0 0.5
ppm

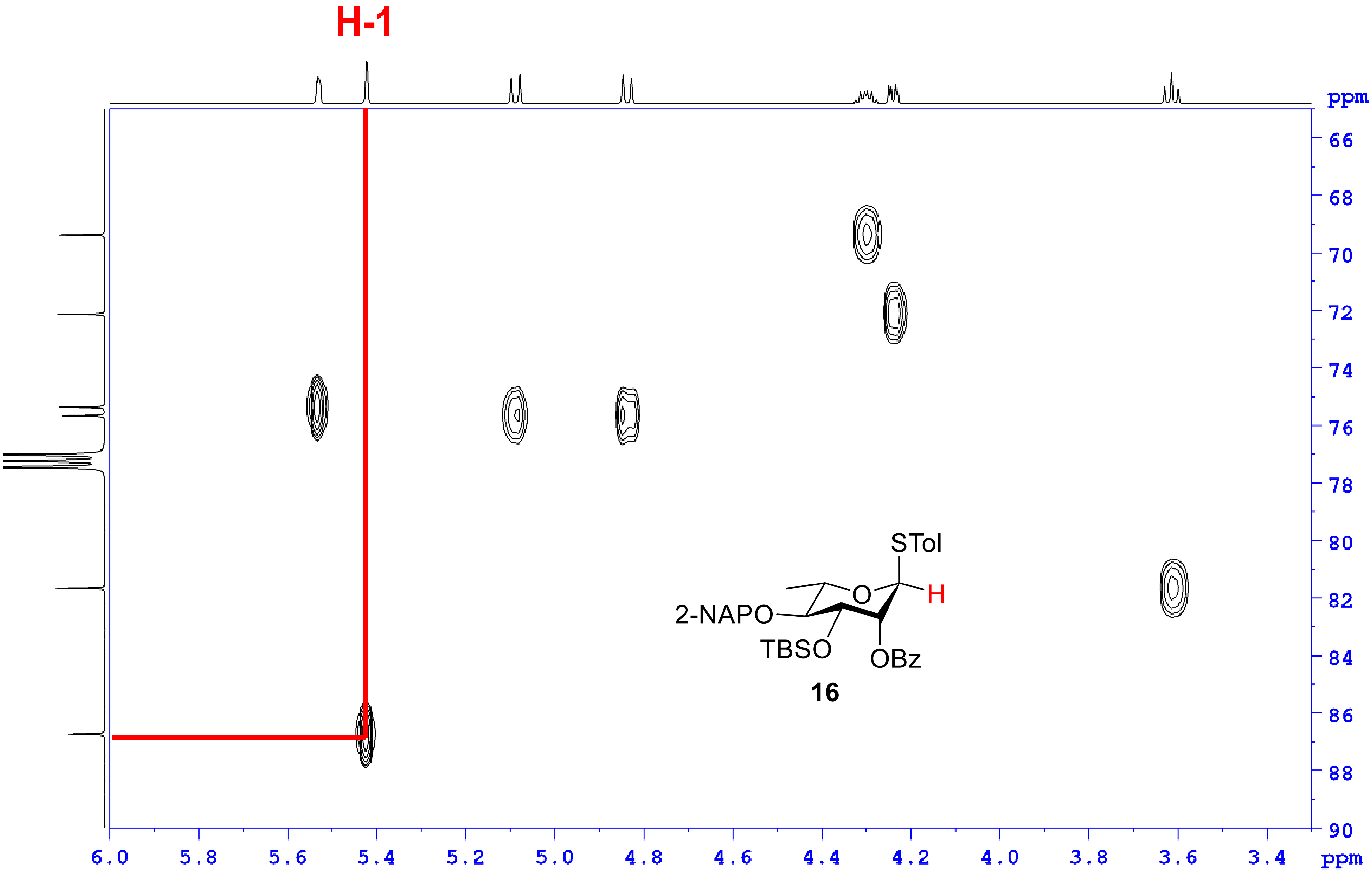

H-1
ppm
66
68
70
72
74
76
78
80
82
84
86
88
90
6.0
5.8
5.6
5.4
5.2
5.0
4.8
4.6
4.4
4.2
4.0
3.8
3.6
3.4
ppm
STol
O
H
2-NAPO
TBSO
OBz
16

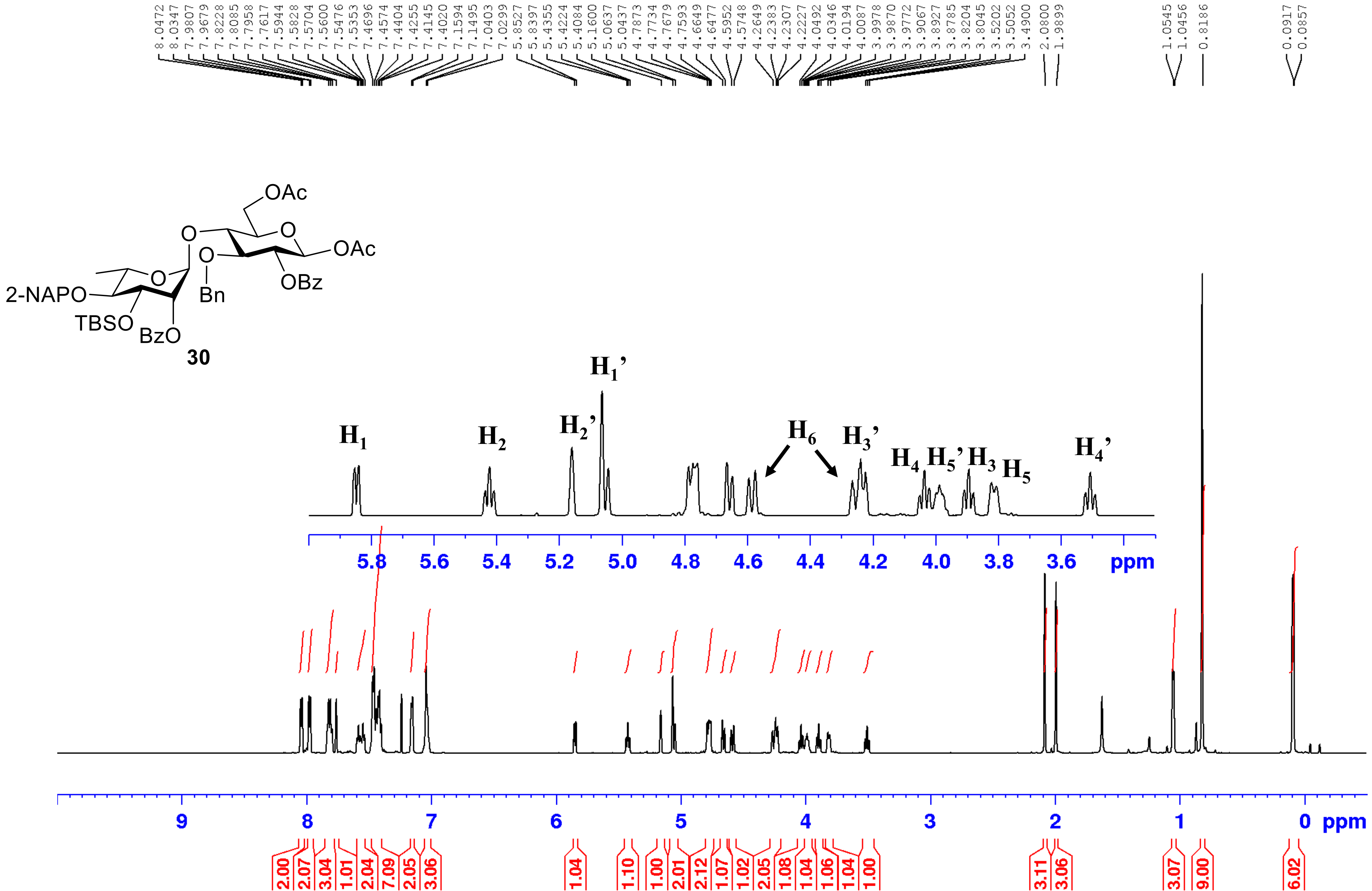

OAc
O
O
O
OAc
2-NAPO
O
Bn
OBz
TBSO
BzO
H1
H2
H2'
H1'
H6
H3'
H4
H5'
H3
H5
H4'
ppm

30

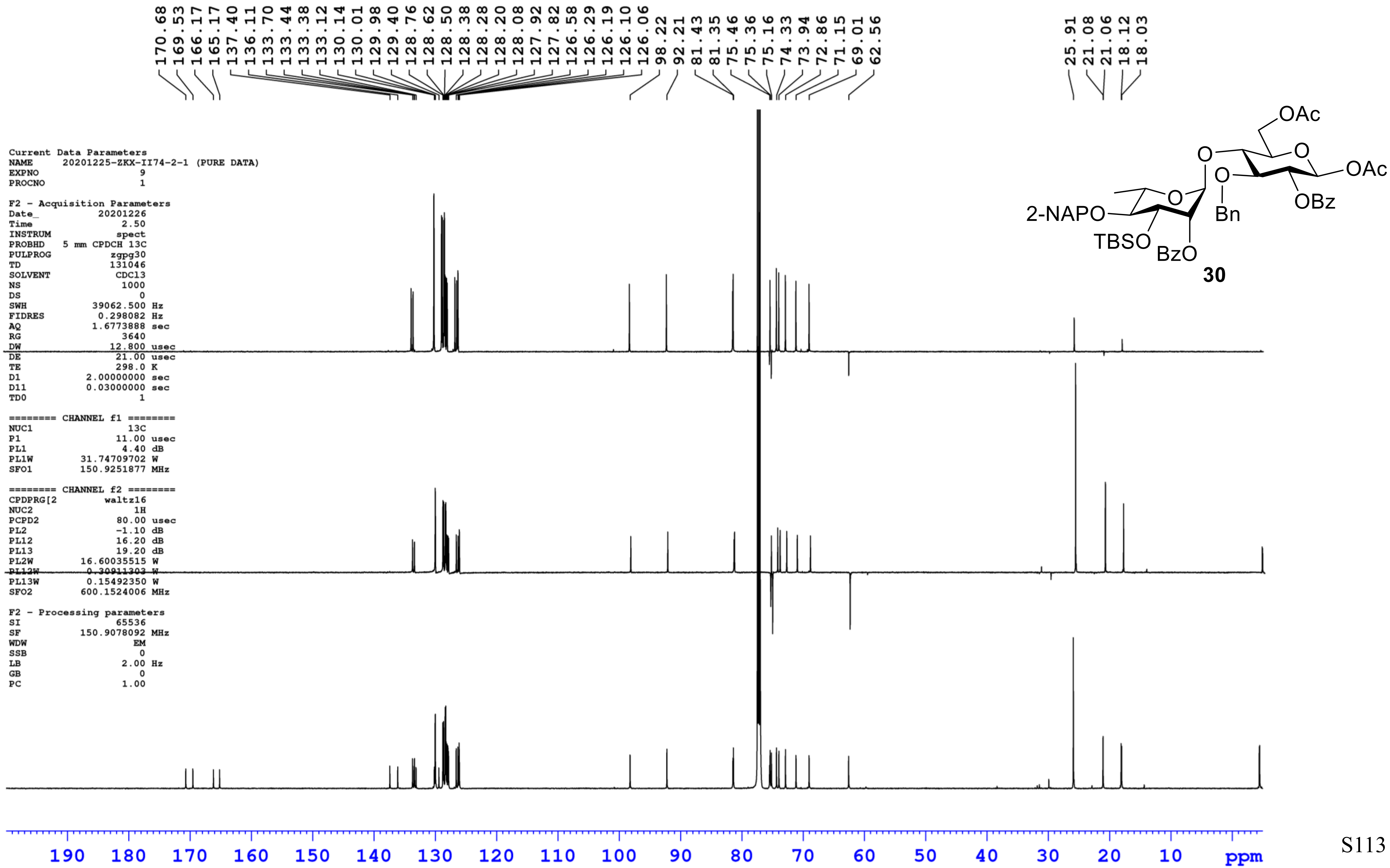
170.68
169.53
166.17
165.17
137.40
136.11
133.70
133.44
133.38
133.12
130.14
130.01
129.98
129.40
128.76
128.62
128.50
128.38
128.28
128.20
128.08
127.92
127.82
126.58
126.29
126.19
126.10
126.06
98.22
92.21
81.43
81.35
75.46
75.36
75.16
74.33
73.94
72.86
71.15
69.01
62.56
25.91
21.08
21.06
18.12
18.03
Current Data Parameters
NAME 20201225-ZKX-II74-2-1 (PURE DATA)
EXPNO 9
PROCNO 1
F2 - Acquisition Parameters
Date_ 20201226
Time 2.50
INSTRUM spect
PROBHD 5 mm CPDCH 13C
PULPROG zgpg30
TD 131046
SOLVENT CDCl3
NS 1000
DS 0
SWH 39062.500 Hz
FIDRES 0.298082 Hz
AQ 1.6773888 sec
RG 3640
DW 12.800 usec
DE 21.00 usec
TE 298.0 K
D1 2.00000000 sec
D11 0.03000000 sec
TD0 1
======== CHANNEL f1 ========
NUC1 13C
P1 11.00 usec
PL1 4.40 dB
PL1W 31.74709702 W
SFO1 150.9251877 MHz
======== CHANNEL f2 ========
CPDPRG[2 waltz16
NUC2 1H
PCPD2 80.00 usec
PL2 -1.10 dB
PL12 16.20 dB
PL13 19.20 dB
PL2W 16.60035515 W
PL12W 0.30911303 W
PL13W 0.15492350 W
SFO2 600.1524006 MHz
F2 - Processing parameters
SI 65536
SF 150.9078092 MHz
WDW EM
SSB 0
LB 2.00 Hz
GB 0
PC 1.00
190 180 170 160 150 140 130 120 110 100 90 80 70 60 50 40 30 20 10 ppm
OAc
O
O
OAc
2-NAPO
O
O
Bn
OBz
TBSO
BzO

30

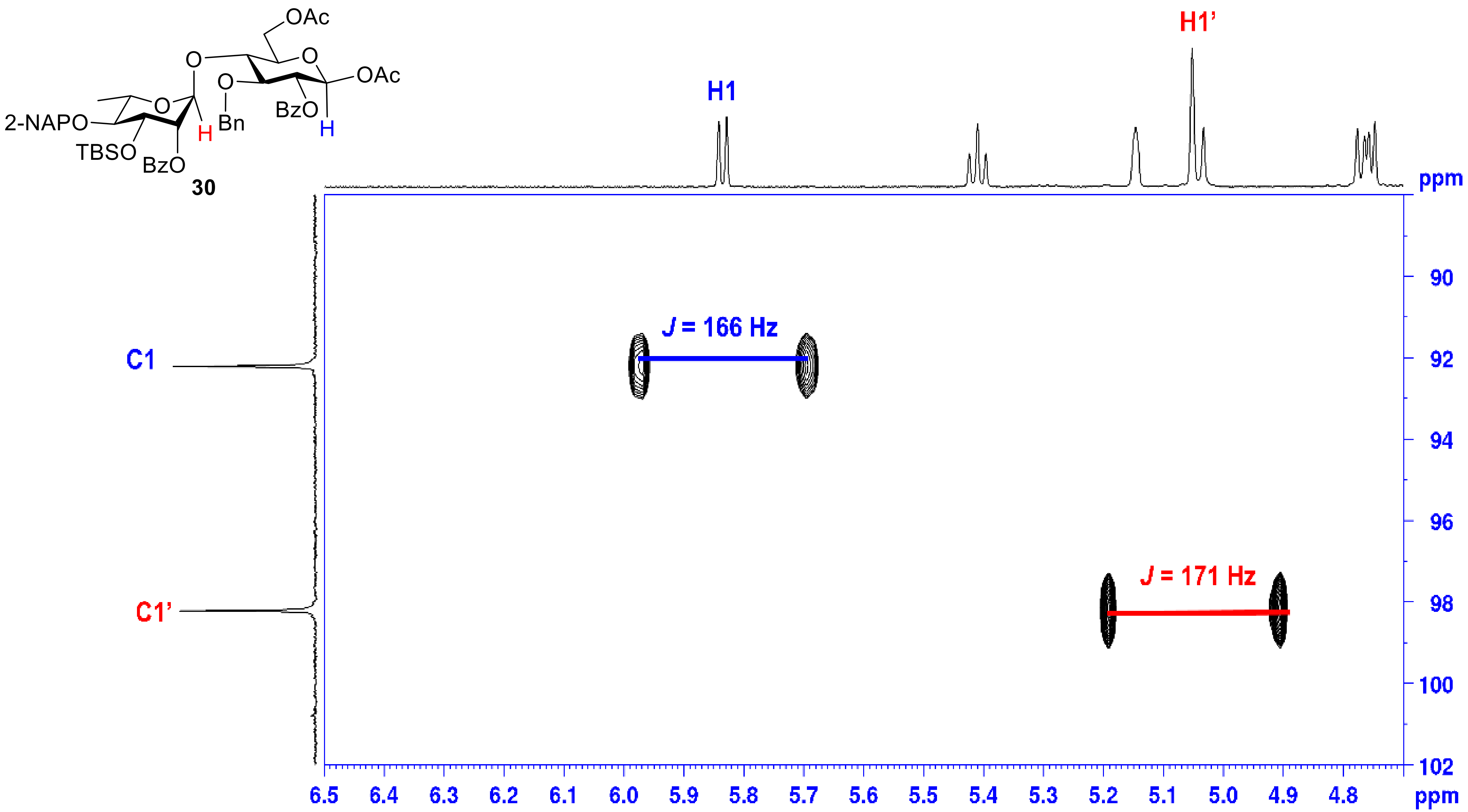
OAc
O
O
O
OAc
2-NAPO
O
H
Bn
BzO
H
TBSO
BzO
30
H1
H1'
ppm
J = 166 Hz
J = 171 Hz
C1
C1'
90
92
94
96
98
100
102
6.5
6.4
6.3
6.2
6.1
6.0
5.9
5.8
5.7
5.6
5.5
5.4
5.3
5.2
5.1
5.0
4.9
4.8
ppm

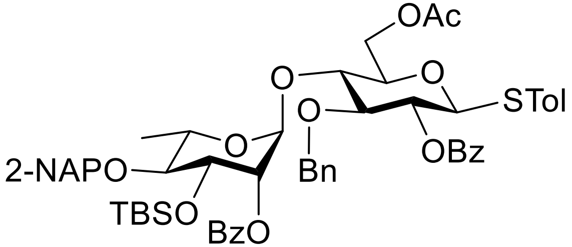
OAc
O
O
O
STol
O
2-NAPO
Bn
OBz
TBSO
BzO

13

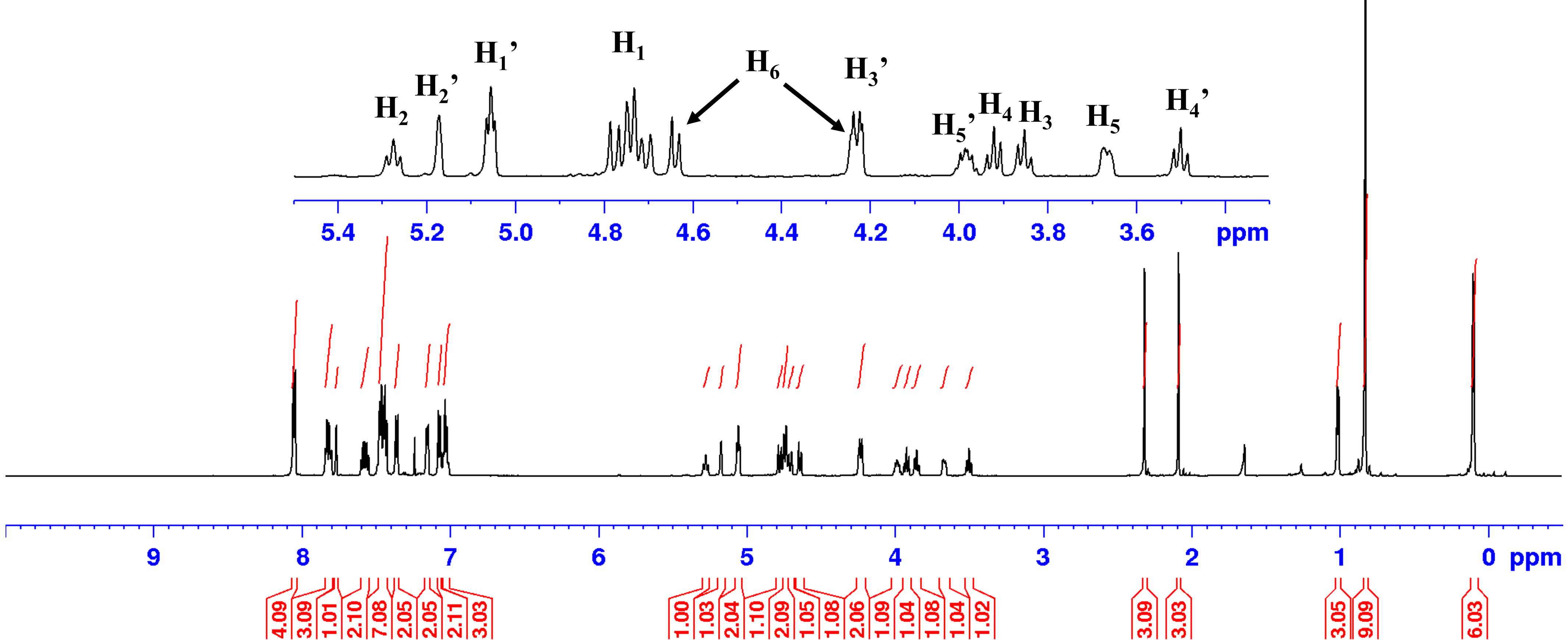
H2
H2'
H1'
H1
H6
H3'
H5'
H4
H3
H5
H4'
5.4
5.2
5.0
4.8
4.6
4.4
4.2
4.0
3.8
3.6
ppm
9
8
7
6
5
4
3
2
1
0 ppm

20201228-ZKX-II78-2-2-C

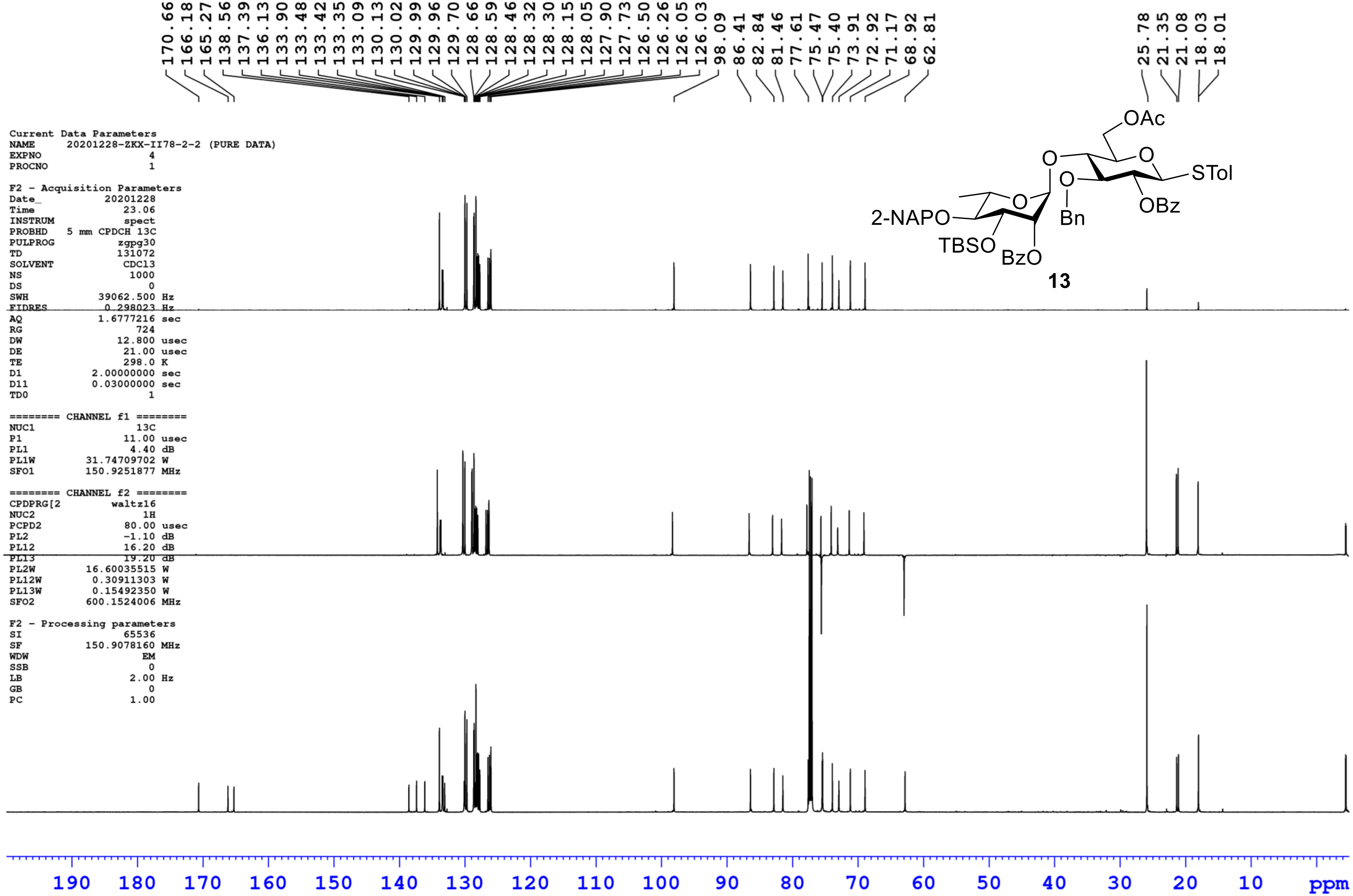

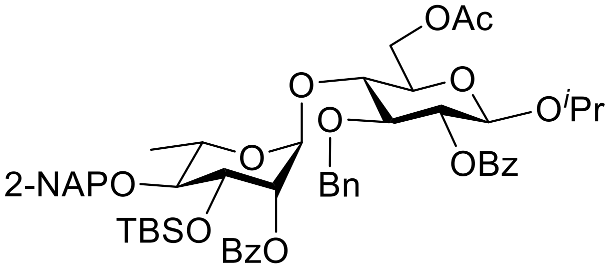

31

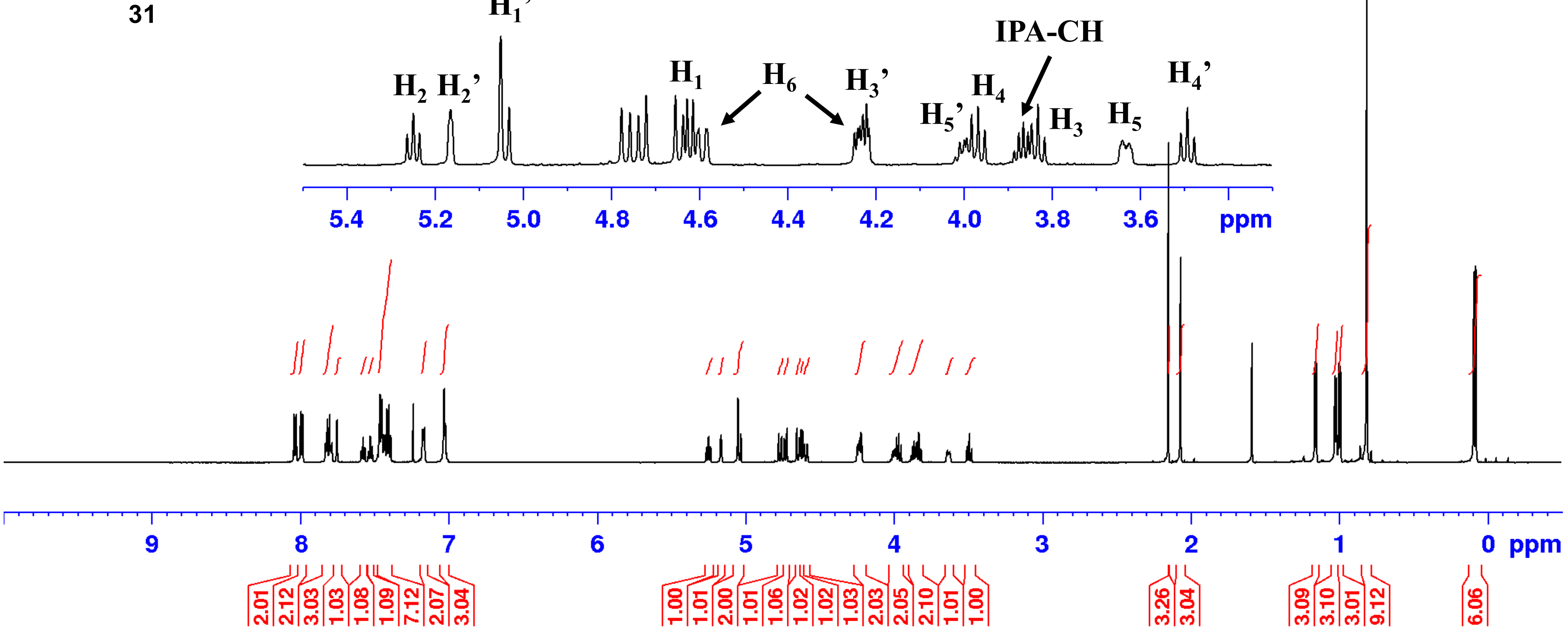

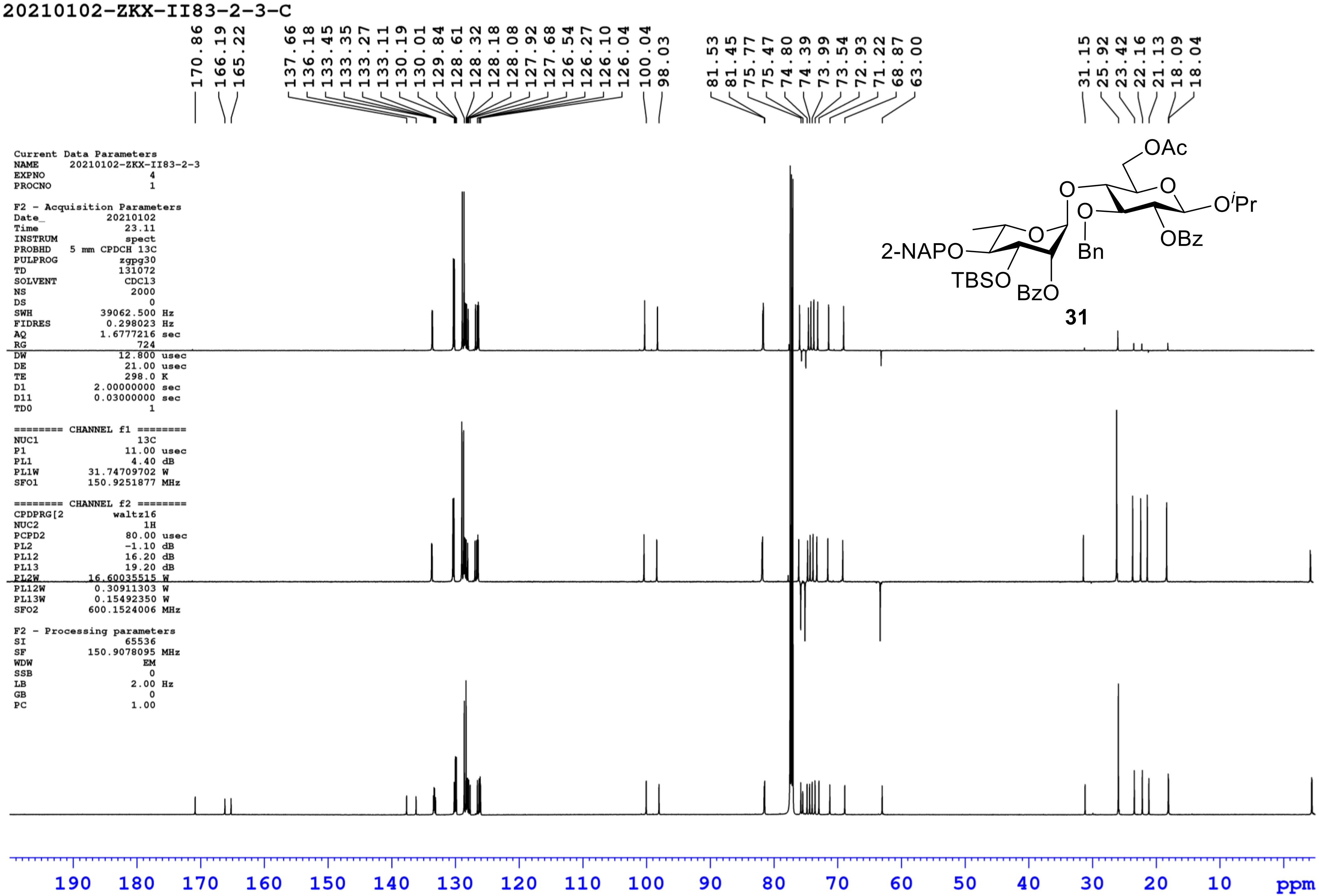

20210102-ZKX-II83-2-3-C
170.86
166.19
165.22
137.66
136.18
133.45
133.35
133.27
133.11
130.19
130.01
129.84
128.61
128.32
128.18
128.08
127.92
127.68
126.54
126.27
126.10
126.04
100.04
98.03
81.53
81.45
75.77
75.47
74.80
74.39
73.99
73.54
72.93
71.22
68.87
63.00
31.15
25.92
23.42
22.16
21.13
18.09
18.04
Current Data Parameters
NAME 20210102-ZKX-II83-2-3
EXPNO 4
PROCNO 1
F2 - Acquisition Parameters
Date_ 20210102
Time 23.11
INSTRUM spect
PROBHD 5 mm CPDCH 13C
PULPROG zgpg30
TD 131072
SOLVENT CDCl3
NS 2000
DS 0
SWH 39062.500 Hz
FIDRES 0.298023 Hz
AQ 1.6777216 sec
RG 724
DW 12.800 usec
DE 21.00 usec
TE 298.0 K
D1 2.00000000 sec
D11 0.03000000 sec
TD0 1
======== CHANNEL f1 ========
NUC1 13C
P1 11.00 usec
PL1 4.40 dB
PL1W 31.74709702 W
SFO1 150.9251877 MHz
======== CHANNEL f2 ========
CPDPRG[2 waltz16
NUC2 1H
PCPD2 80.00 usec
PL2 -1.10 dB
PL12 16.20 dB
PL13 19.20 dB
PL2W 16.60035515 W
PL12W 0.30911303 W
PL13W 0.15492350 W
SFO2 600.1524006 MHz
F2 - Processing parameters
SI 65536
SF 150.9078095 MHz
WDW EM
SSB 0
LB 2.00 Hz
GB 0
PC 1.00
OAc
O
O
O
O^{i}Pr
O
2-NAPO
Bn
OBz
TBSO
BzO
31
190 180 170 160 150 140 130 120 110 100 90 80 70 60 50 40 30 20 10 ppm

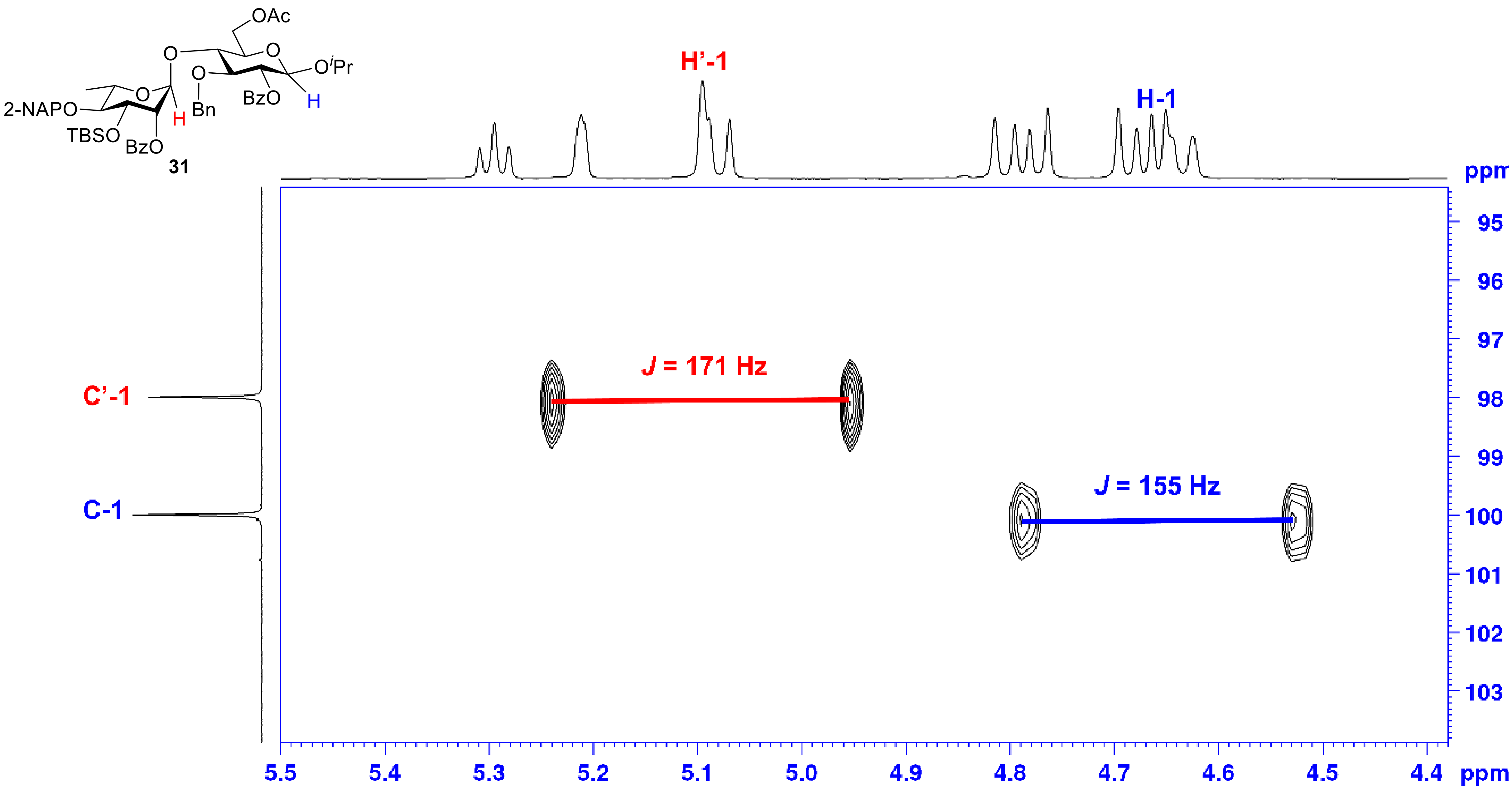
OAc
O
O
O^{i}Pr
O
2-NAPO
O
Bn
BzO
H
H
TBSO
BzO
31
H'-1
H-1
ppm
95
96
97
98
99
100
101
102
103
J = 171 Hz
J = 155 Hz
C'-1
C-1
5.5
5.4
5.3
5.2
5.1
5.0
4.9
4.8
4.7
4.6
4.5
4.4
ppm

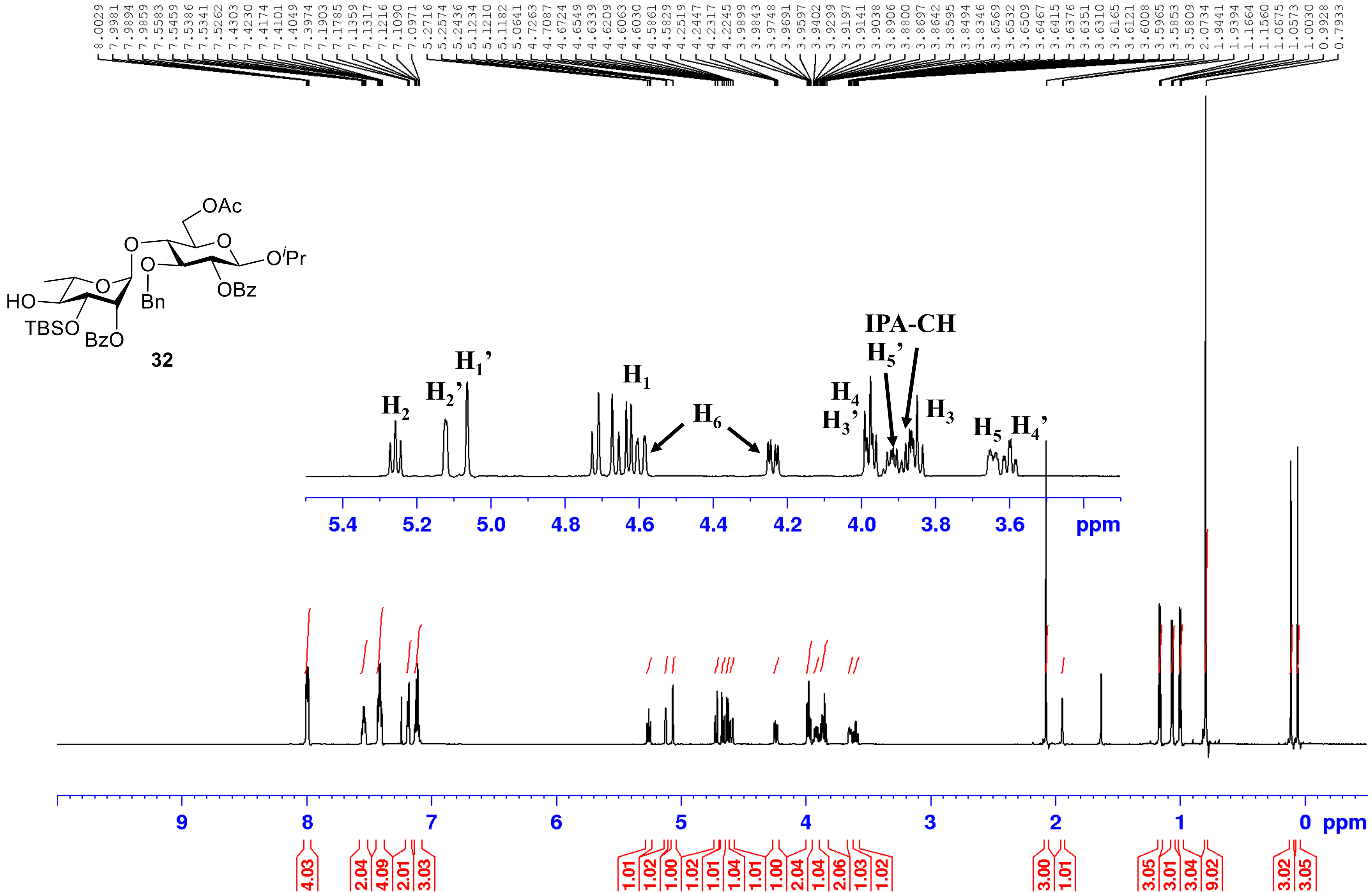
OAc
O
O
OⁱPr
HO
Bn
OBz
TBSO
BzO
32
IPA-CH
H2
H2'
H1'
H1
H6
H4
H3'
H5'
H3
H5
H4'
ppm

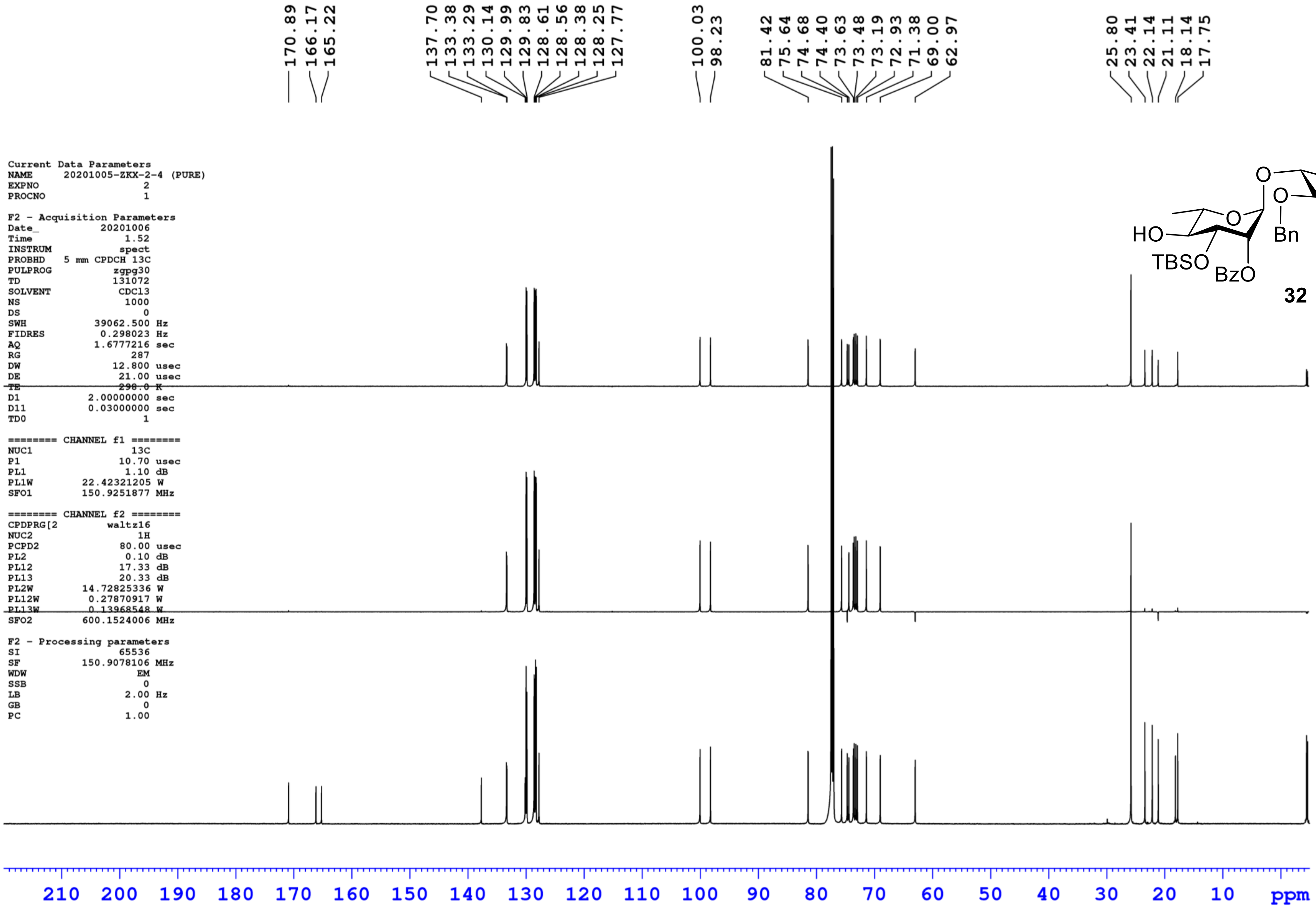
170.89
166.17
165.22
137.70
133.38
133.29
130.14
129.99
129.83
128.61
128.56
128.38
128.25
127.77
100.03
98.23
81.42
75.64
74.68
74.40
73.63
73.48
73.19
72.93
71.38
69.00
62.97
25.80
23.41
22.14
21.11
18.14
17.75
Current Data Parameters
NAME 20201005-ZKX-2-4 (PURE)
EXPNO 2
PROCNO 1
F2 - Acquisition Parameters
Date_ 20201006
Time 1.52
INSTRUM spect
PROBHD 5 mm CPDCH 13C
PULPROG zgpg30
TD 131072
SOLVENT CDCl3
NS 1000
DS 0
SWH 39062.500 Hz
FIDRES 0.298023 Hz
AQ 1.6777216 sec
RG 287
DW 12.800 usec
DE 21.00 usec
TE 298.0 K
D1 2.00000000 sec
D11 0.03000000 sec
TD0 1
======== CHANNEL f1 ========
NUC1 13C
P1 10.70 usec
PL1 1.10 dB
PL1W 22.42321205 W
SFO1 150.9251877 MHz
======== CHANNEL f2 ========
CPDPRG[2 waltz16
NUC2 1H
PCPD2 80.00 usec
PL2 0.10 dB
PL12 17.33 dB
PL13 20.33 dB
PL2W 14.72825336 W
PL12W 0.27870917 W
PL13W 0.13968548 W
SFO2 600.1524006 MHz
F2 - Processing parameters
SI 65536
SF 150.9078106 MHz
WDW EM
SSB 0
LB 2.00 Hz
GB 0
PC 1.00
210 200 190 180 170 160 150 140 130 120 110 100 90 80 70 60 50 40 30 20 10 ppm

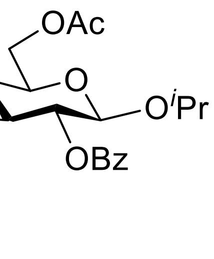
OAc
O
O
O^{i}Pr
O
HO
Bn
OBz
TBSO
BzO

32

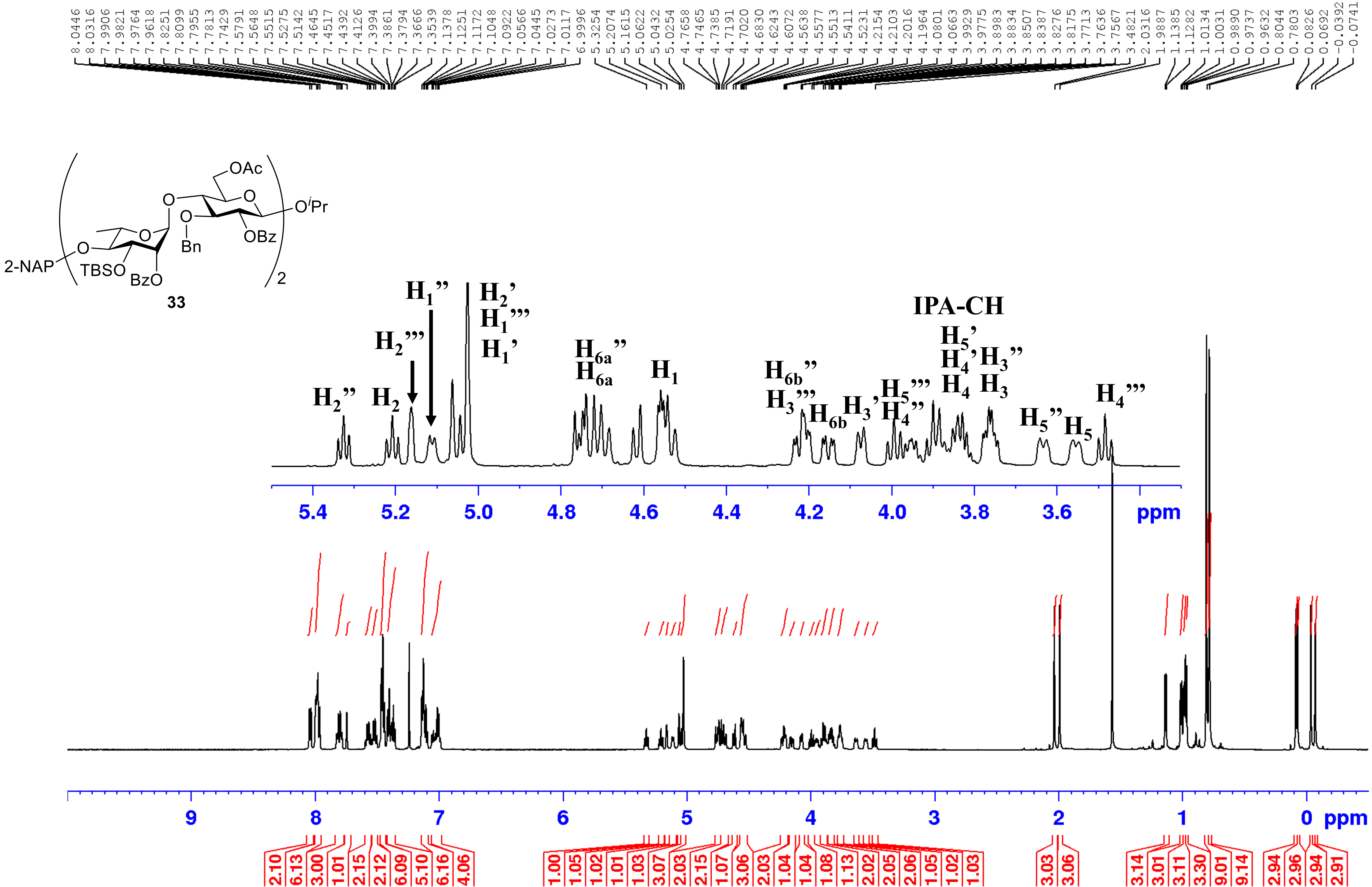

OAc
O
O
OⁱPr
2-NAP
TBSO
BzO
Bn
OBz
2
33
H1''
H2'
H1'''
H1'
H2'''
H2''
H2
H6a''
H6a
H1
H6b''
H3'''
H6b
H3'
H5'''
H4''
IPA-CH
H5'
H4'
H3''
H4
H3
H5''
H5
H4'''
ppm

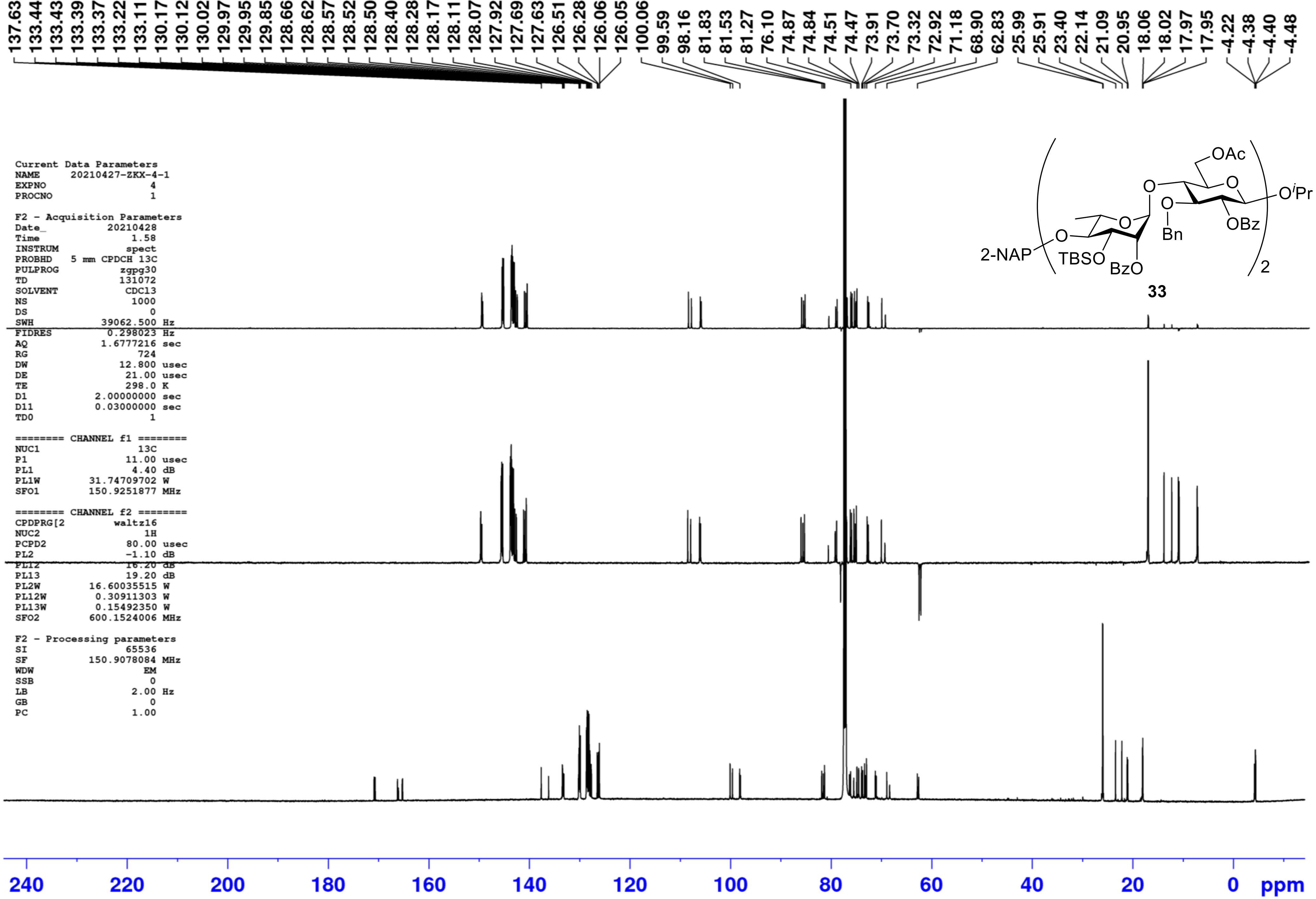
137.63
133.44
133.43
133.39
133.37
133.22
133.11
130.17
130.12
130.02
129.97
129.95
129.85
128.66
128.62
128.57
128.52
128.50
128.40
128.28
128.17
128.11
128.07
127.92
127.69
127.63
126.51
126.28
126.06
126.05
100.06
99.59
98.16
81.83
81.53
81.27
76.10
74.87
74.84
74.51
74.47
73.91
73.70
73.32
72.92
71.18
68.90
62.83
25.99
25.91
23.40
22.14
21.09
20.95
18.06
18.02
17.97
17.95
-4.22
-4.38
-4.40
-4.48
Current Data Parameters
NAME 20210427-ZKX-4-1
EXPNO 4
PROCNO 1
F2 - Acquisition Parameters
Date_ 20210428
Time 1.58
INSTRUM spect
PROBHD 5 mm CPDCH 13C
PULPROG zgpg30
TD 131072
SOLVENT CDCl3
NS 1000
DS 0
SWH 39062.500 Hz
FIDRES 0.298023 Hz
AQ 1.6777216 sec
RG 724
DW 12.800 usec
DE 21.00 usec
TE 298.0 K
D1 2.00000000 sec
D11 0.03000000 sec
TD0 1
======== CHANNEL f1 ========
NUC1 13C
P1 11.00 usec
PL1 4.40 dB
PL1W 31.74709702 W
SFO1 150.9251877 MHz
======== CHANNEL f2 ========
CPDPRG[2 waltz16
NUC2 1H
PCPD2 80.00 usec
PL2 -1.10 dB
PL12 16.20 dB
PL13 19.20 dB
PL2W 16.60035515 W
PL12W 0.30911303 W
PL13W 0.15492350 W
SFO2 600.1524006 MHz
F2 - Processing parameters
SI 65536
SF 150.9078084 MHz
WDW EM
SSB 0
LB 2.00 Hz
GB 0
PC 1.00
OAc
O
O
OⁱPr
O
OBz
Bn
O
2-NAP
O
TBSO
BzO
2
33
240
220
200
180
160
140
120
100
80
60
40
20
0
ppm

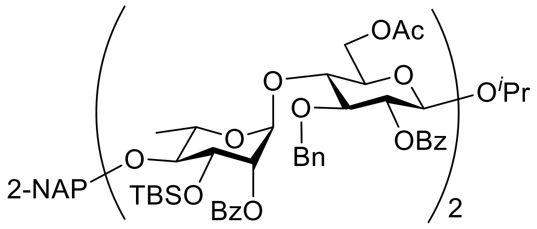

OAc
O
O
O'Pr
O
O
Bn
OBz
2-NAP
O
TBSO
BzO
2

33

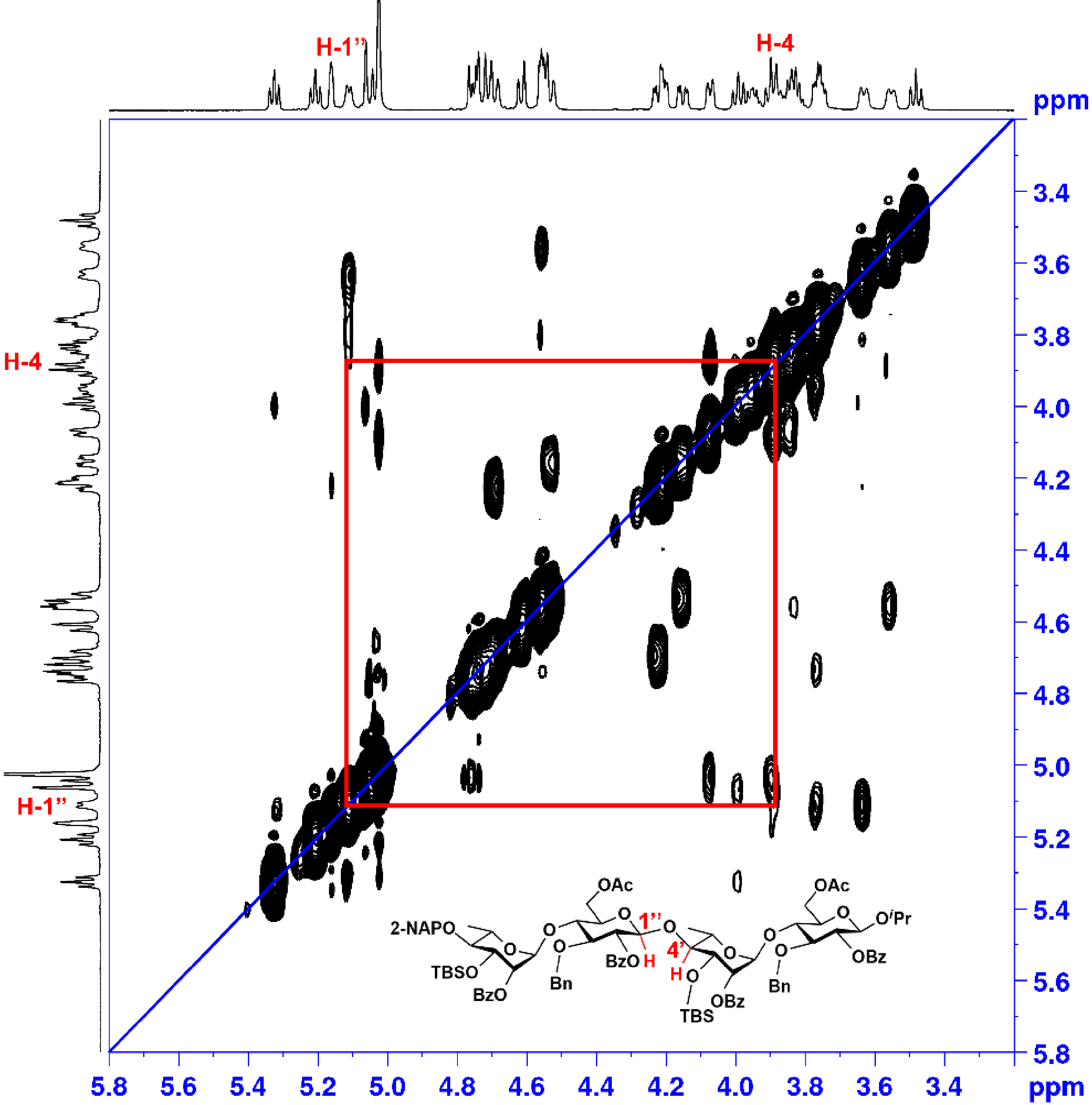

H-1''
H-4
ppm
H-4
H-1''
3.4
3.6
3.8
4.0
4.2
4.4
4.6
4.8
5.0
5.2
5.4
5.6
5.8
ppm
5.8
5.6
5.4
5.2
5.0
4.8
4.6
4.4
4.2
4.0
3.8
3.6
3.4
ppm
OAc
OAc
2-NAPO
1''
O'Pr
4'
TBSO
BzO H
H O
OBz
BzO
Bn
OBz
Bn
TBS

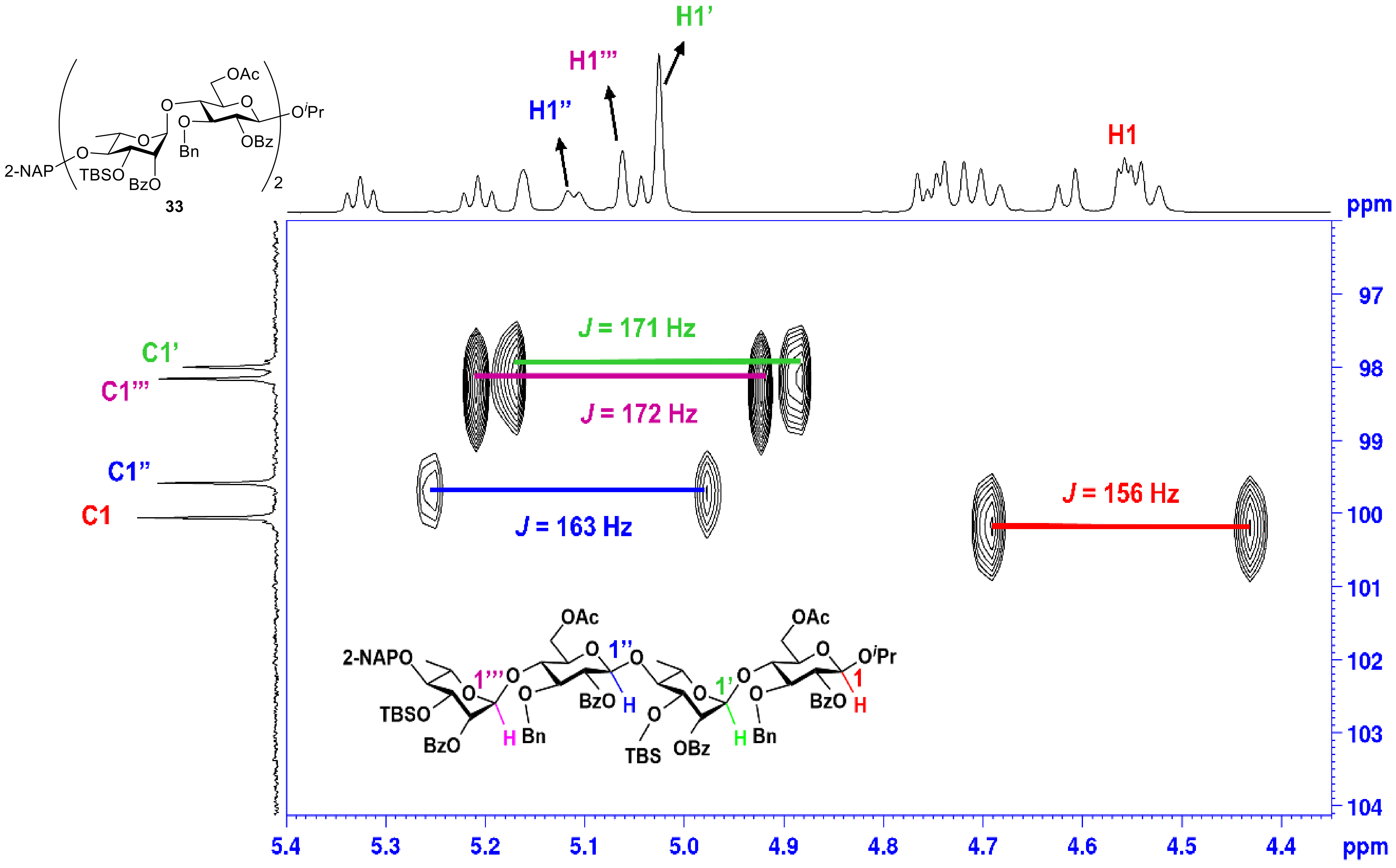

OAc
O
O
O
O
Bn
OBz
O^{i}Pr
2-NAP
TBSO
BzO
2
33
H1'
H1'''
H1''
H1
ppm
C1'
C1'''
C1''
C1
J = 171 Hz
J = 172 Hz
J = 163 Hz
J = 156 Hz
97
98
99
100
101
102
103
104
2-NAPO
1'''
1''
1'
1
H
Bn
TBS
OBz
5.4
5.3
5.2
5.1
5.0
4.9
4.8
4.7
4.6
4.5
4.4
ppm

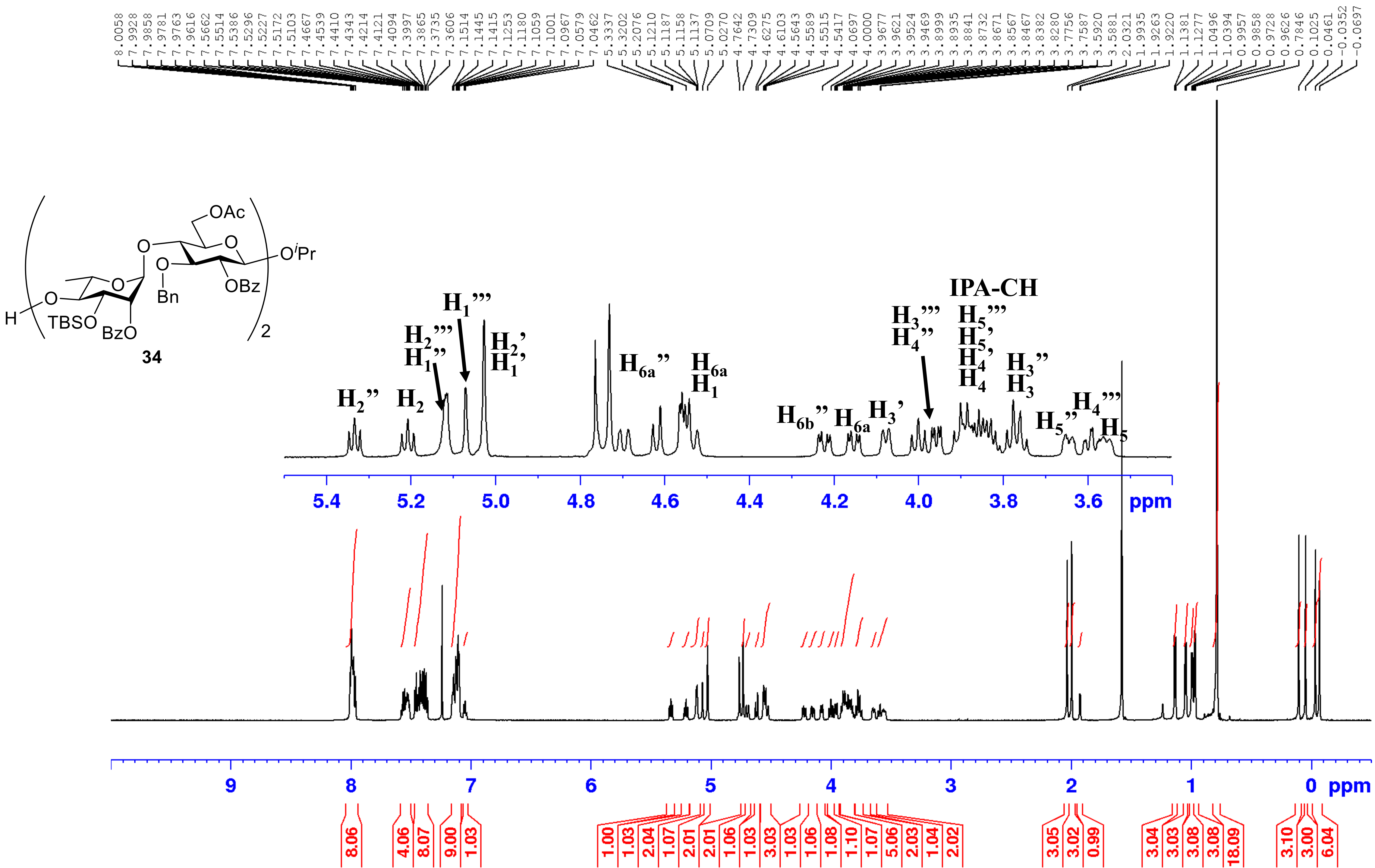
OAc
O
O
O
O
O^{i}Pr
OBz
Bn
H
O
O
TBSO
BzO
2
34
IPA-CH
ppm

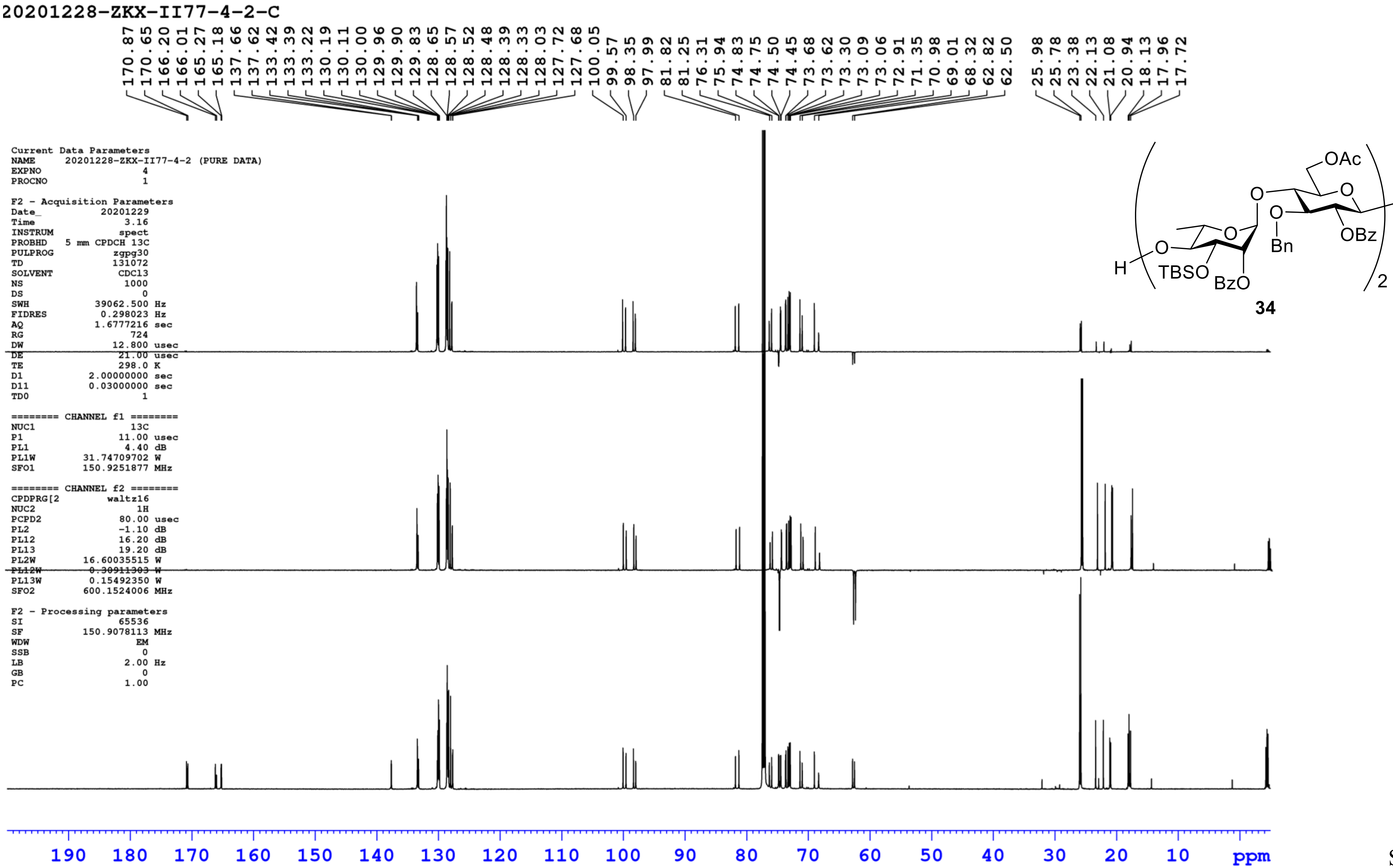
20201228-ZKX-II77-4-2-C
170.87
170.65
166.20
166.01
165.27
165.18
137.66
137.62
133.42
133.39
133.22
130.19
130.11
130.00
129.96
129.90
129.83
128.65
128.57
128.52
128.48
128.39
128.33
128.03
127.72
127.68
100.05
99.57
98.35
97.99
81.82
81.25
76.31
75.94
74.83
74.75
74.50
74.45
73.68
73.62
73.30
73.09
73.06
72.91
71.35
70.98
69.01
68.32
62.82
62.50
25.98
25.78
23.38
22.13
21.08
20.94
18.13
17.96
17.72
Current Data Parameters
NAME 20201228-ZKX-II77-4-2 (PURE DATA)
EXPNO 4
PROCNO 1
F2 - Acquisition Parameters
Date_ 20201229
Time 3.16
INSTRUM spect
PROBHD 5 mm CPDCH 13C
PULPROG zgpg30
TD 131072
SOLVENT CDCl3
NS 1000
DS 0
SWH 39062.500 Hz
FIDRES 0.298023 Hz
AQ 1.6777216 sec
RG 724
DW 12.800 usec
DE 21.00 usec
TE 298.0 K
D1 2.00000000 sec
D11 0.03000000 sec
TD0 1
======== CHANNEL f1 ========
NUC1 13C
P1 11.00 usec
PL1 4.40 dB
PL1W 31.74709702 W
SFO1 150.9251877 MHz
======== CHANNEL f2 ========
CPDPRG[2 waltz16
NUC2 1H
PCPD2 80.00 usec
PL2 -1.10 dB
PL12 16.20 dB
PL13 19.20 dB
PL2W 16.60035515 W
PL12W 0.30911303 W
PL13W 0.15492350 W
SFO2 600.1524006 MHz
F2 - Processing parameters
SI 65536
SF 150.9078113 MHz
WDW EM
SSB 0
LB 2.00 Hz
GB 0
PC 1.00
OAc
O
O
O
O^{i}Pr
Bn
OBz
O
O
H
TBSO
BzO
2
34
190
180
170
160
150
140
130
120
110
100
90
80
70
60
50
40
30
20
10
ppm

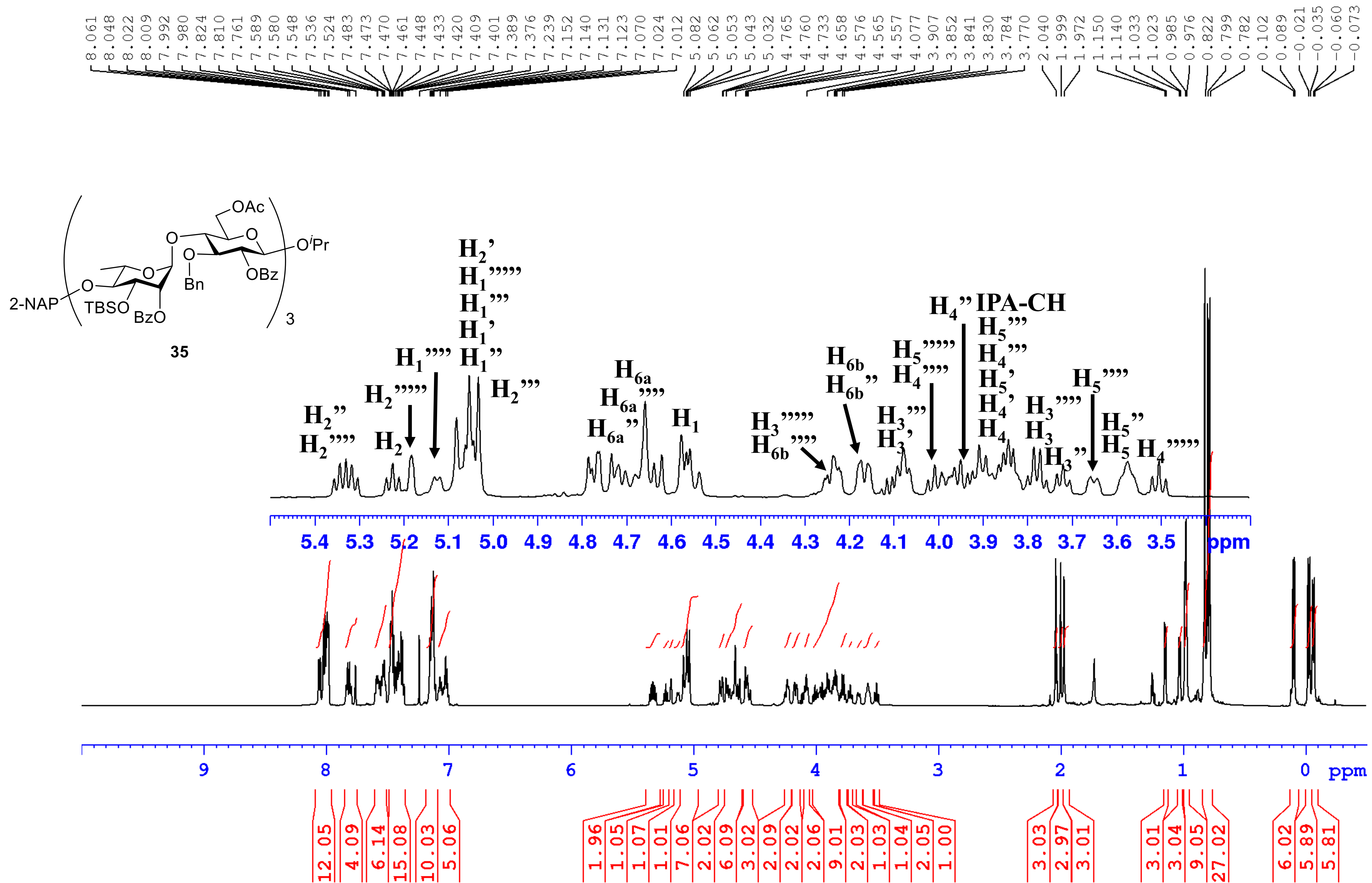

8.061 8.048 8.022 8.009 7.992 7.980 7.824 7.810 7.761 7.589 7.580 7.548 7.536 7.524 7.483 7.473 7.470 7.461 7.448 7.433 7.420 7.409 7.401 7.389 7.376 7.239 7.152 7.140 7.131 7.123 7.070 7.024 7.012 5.082 5.062 5.053 5.043 5.032 4.765 4.760 4.733 4.658 4.576 4.565 4.557 4.077 3.907 3.852 3.841 3.830 3.784 3.770 2.040 1.999 1.972 1.150 1.140 1.033 1.023 0.985 0.976 0.822 0.799 0.782 0.102 0.089 -0.021 -0.035 -0.060 -0.073
OAc
O
O
O
OBz
O^iPr
Bn
2-NAP
TBSO
BzO
3
35
ppm
ppm
12.05 4.09 6.14 15.08 10.03 5.06 1.96 1.05 1.07 1.01 7.06 2.02 6.09 3.02 2.09 2.02 2.06 9.01 2.03 1.03 1.04 2.05 1.00 3.03 2.97 3.01 3.01 3.04 9.05 27.02 6.02 5.89 5.81

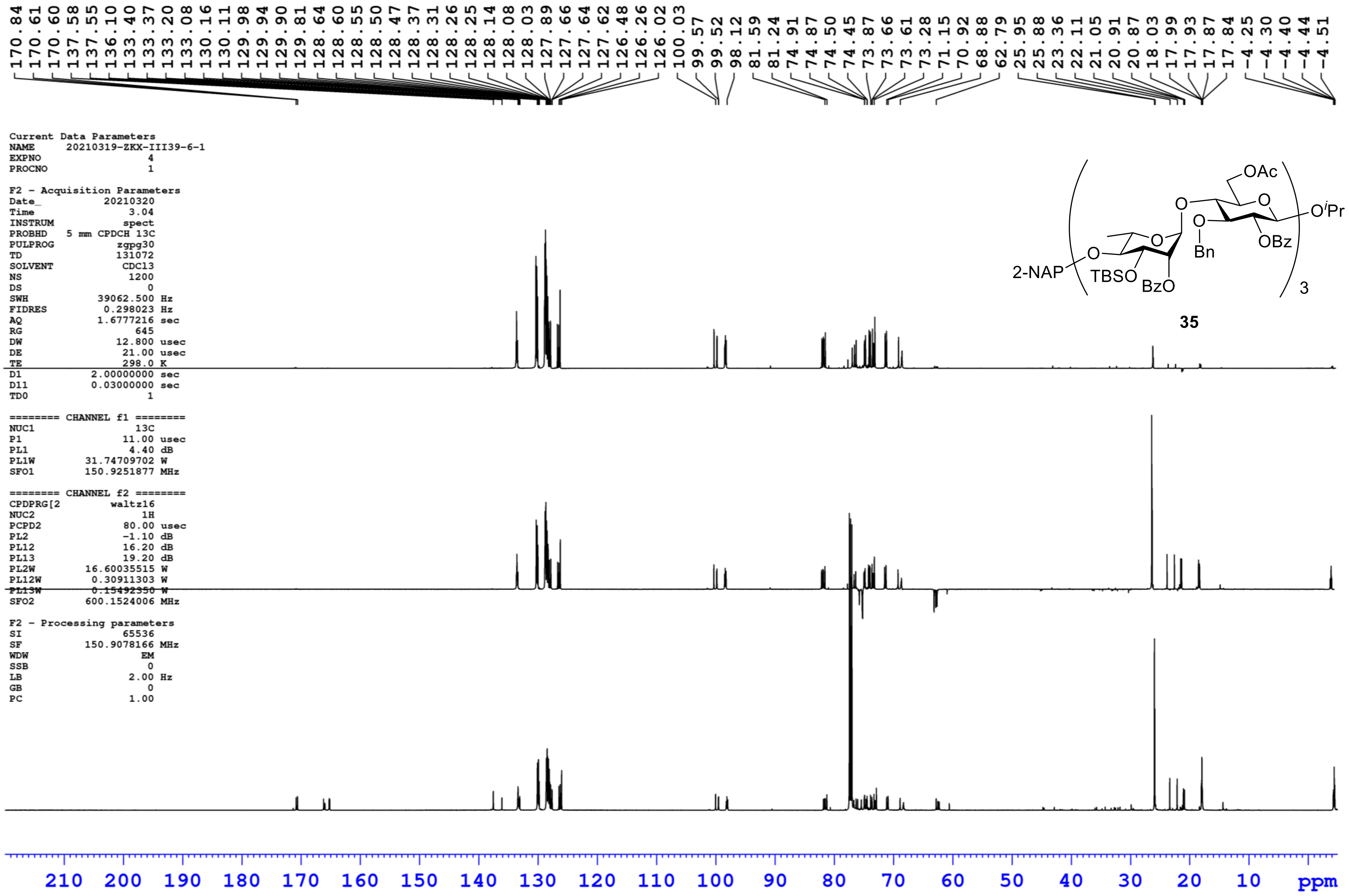

170.84
170.61
170.60
137.58
137.55
136.10
133.40
133.37
133.20
133.08
130.16
130.11
129.98
129.94
129.90
129.81
128.64
128.60
128.55
128.50
128.47
128.37
128.31
128.26
128.25
128.14
128.08
128.03
127.89
127.66
127.64
127.62
126.48
126.26
126.02
100.03
99.57
99.52
98.12
81.59
81.24
74.91
74.87
74.50
74.45
73.87
73.66
73.61
73.28
71.15
70.92
68.88
62.79
25.95
25.88
23.36
22.11
21.05
20.91
20.87
18.03
17.99
17.93
17.87
17.84
-4.25
-4.30
-4.40
-4.44
-4.51
Current Data Parameters
NAME 20210319-ZKX-III39-6-1
EXPNO 4
PROCNO 1
F2 - Acquisition Parameters
Date_ 20210320
Time 3.04
INSTRUM spect
PROBHD 5 mm CPDCH 13C
PULPROG zgpg30
TD 131072
SOLVENT CDCl3
NS 1200
DS 0
SWH 39062.500 Hz
FIDRES 0.298023 Hz
AQ 1.6777216 sec
RG 645
DW 12.800 usec
DE 21.00 usec
TE 298.0 K
D1 2.00000000 sec
D11 0.03000000 sec
TD0 1
======== CHANNEL f1 ========
NUC1 13C
P1 11.00 usec
PL1 4.40 dB
PL1W 31.74709702 W
SFO1 150.9251877 MHz
======== CHANNEL f2 ========
CPDPRG[2 waltz16
NUC2 1H
PCPD2 80.00 usec
PL2 -1.10 dB
PL12 16.20 dB
PL13 19.20 dB
PL2W 16.60035515 W
PL12W 0.30911303 W
PL13W 0.15492350 W
SFO2 600.1524006 MHz
F2 - Processing parameters
SI 65536
SF 150.9078166 MHz
WDW EM
SSB 0
LB 2.00 Hz
GB 0
PC 1.00
OAc
O
O
O^{i}Pr
O
O
Bn
OBz
2-NAP
O
TBSO
BzO
3
35
210 200 190 180 170 160 150 140 130 120 110 100 90 80 70 60 50 40 30 20 10 ppm

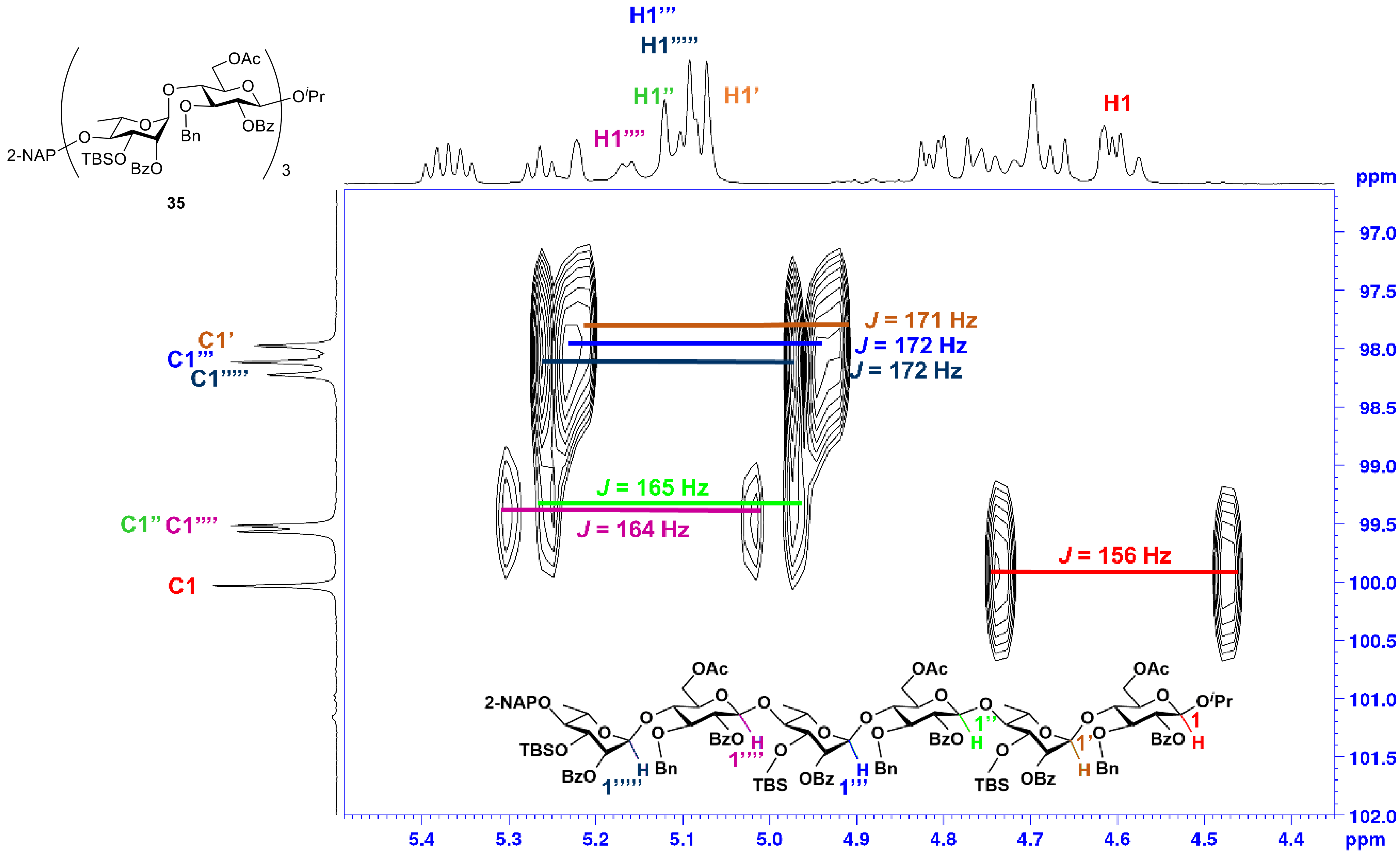

2-NAP
TBSO
BzO
Bn
OAc
OBz
O^{i}Pr
3
35
H1'''
H1'''''
H1''
H1'
H1''''
H1
ppm
C1'
C1'''
C1'''''
C1''
C1''''
C1
J = 171 Hz
J = 172 Hz
J = 172 Hz
J = 165 Hz
J = 164 Hz
J = 156 Hz
97.0
97.5
98.0
98.5
99.0
99.5
100.0
100.5
101.0
101.5
102.0
5.4
5.3
5.2
5.1
5.0
4.9
4.8
4.7
4.6
4.5
4.4
ppm
2-NAPO
TBS

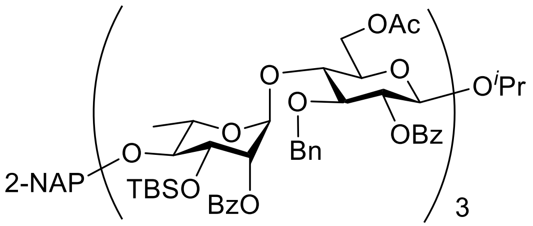
OAc
O
O
O
O
O^iPr
2-NAP
TBSO
BzO
Bn
OBz
3

35

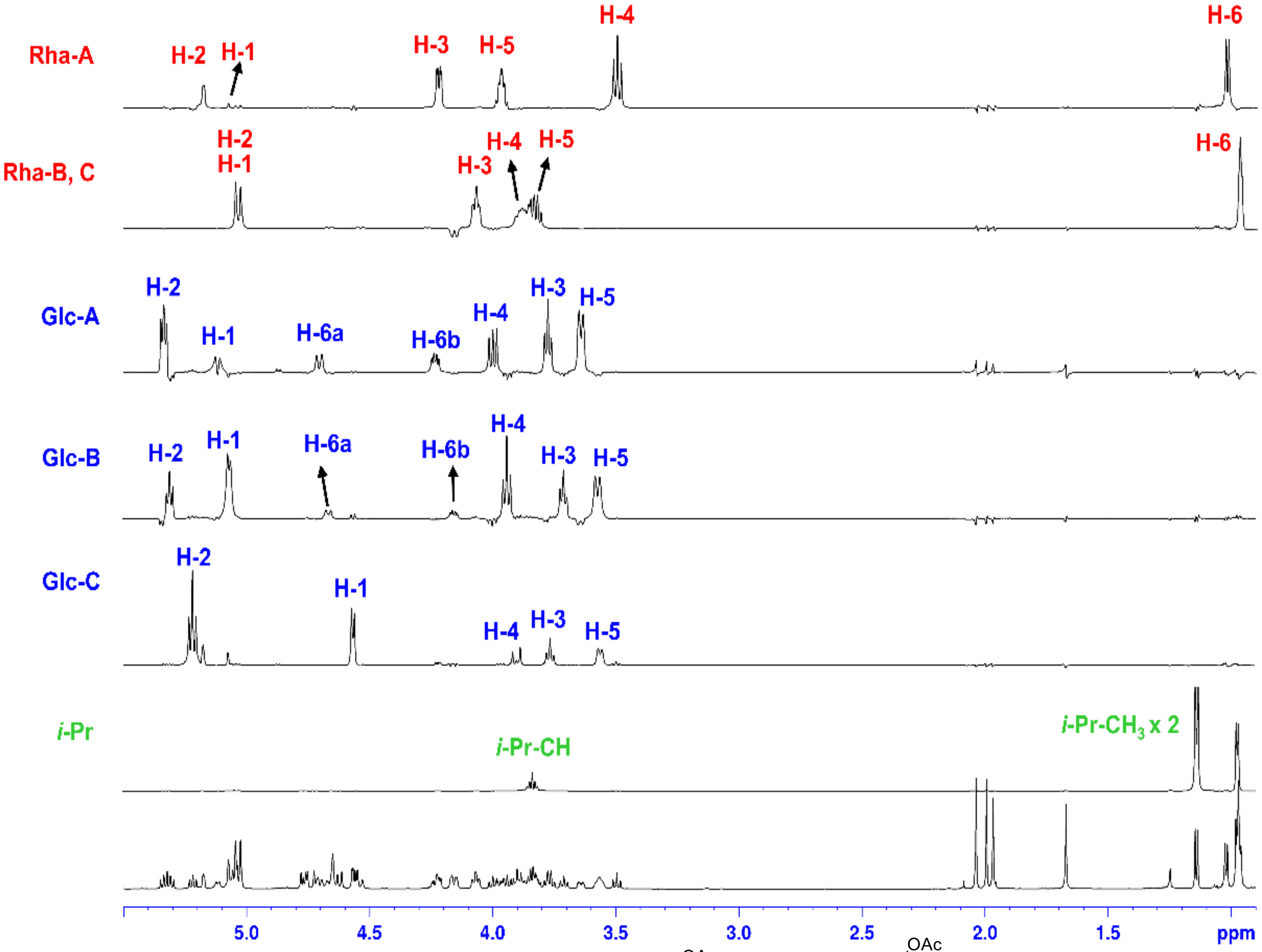
Rha-A
H-2
H-1
H-3
H-5
H-4
H-6
Rha-B, C
H-2
H-1
H-3
H-4
H-5
H-6
Glc-A
H-2
H-1
H-6a
H-6b
H-4
H-3
H-5
Glc-B
H-2
H-1
H-6a
H-6b
H-4
H-3
H-5
Glc-C
H-2
H-1
H-4
H-3
H-5
i-Pr
i-Pr-CH
i-Pr-CH3 x 2
5.0
4.5
4.0
3.5
3.0
2.5
2.0
1.5
ppm

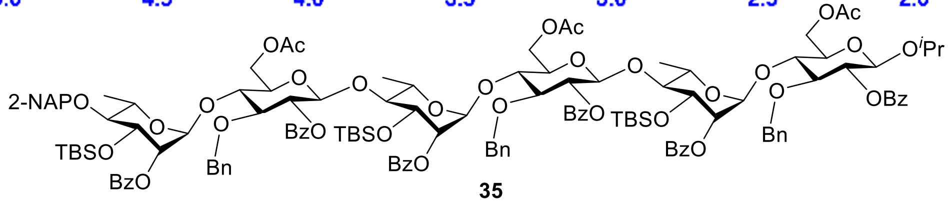
2-NAPO
TBSO
BzO
OAc
Bn
BzO
TBSO
BzO
OAc
Bn
BzO
TBSO
BzO
OAc
Bn
OBz
O^iPr

35

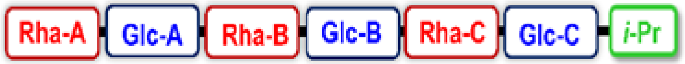
Rha-A
Glc-A
Rha-B
Glc-B
Rha-C
Glc-C
i-Pr

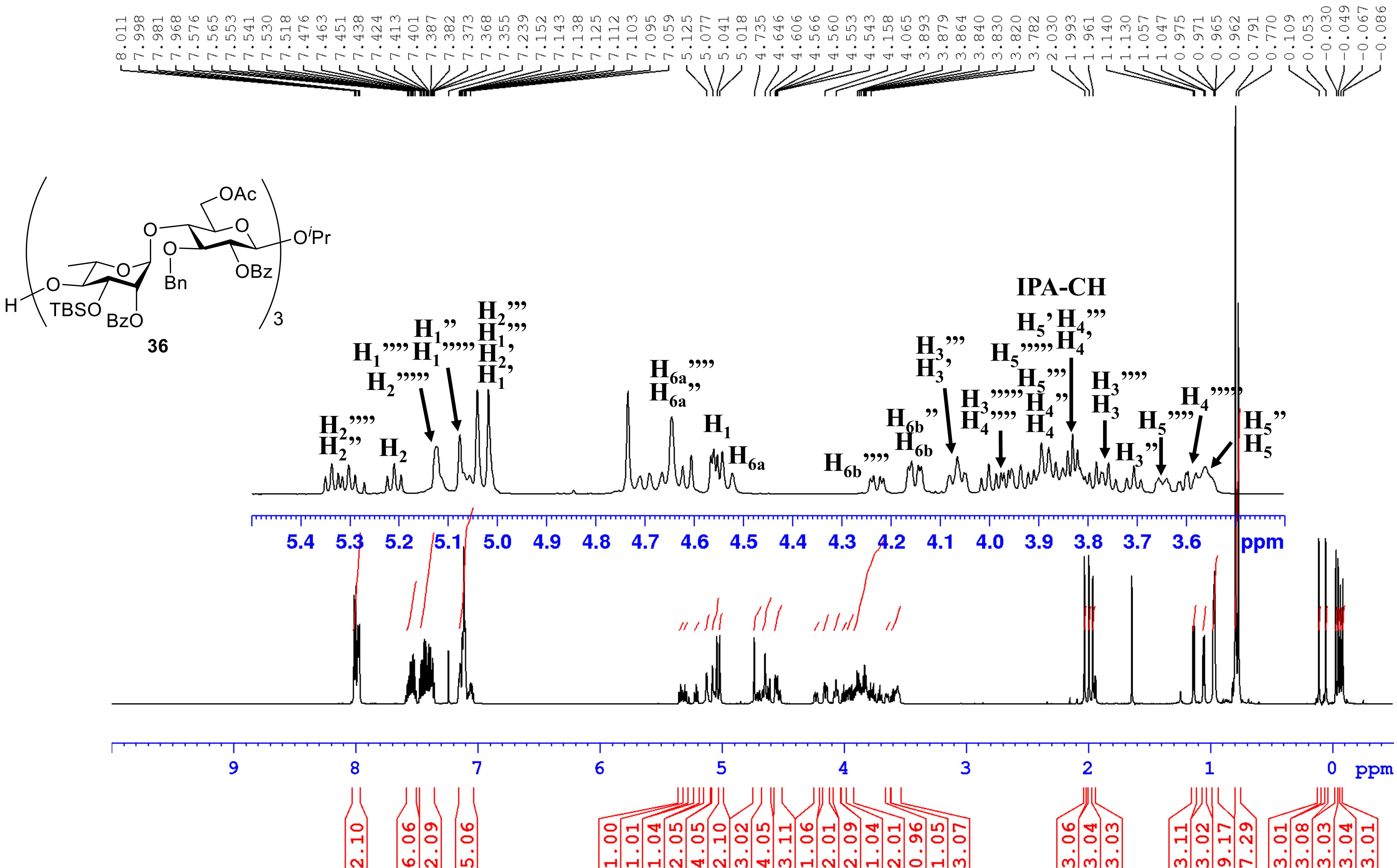

OAc
O'Pr
OBz
O
Bn
TBSO
BzO
H
3
36
IPA-CH
H2'''' H2''
H2
H1'''' H2'''''
H1'' H1'''''
H2''' H1''' H2' H1
H6a'''' H6a''
H1
H6a
H6b''''
H6b'' H6b
H3''' H3'
H3''''' H4''''
H5' H4''' H4' H4
H5''''' H5''' H4'' H4
H3'''' H3
H3''
H5''''
H4'''''
H5'' H5
ppm
ppm

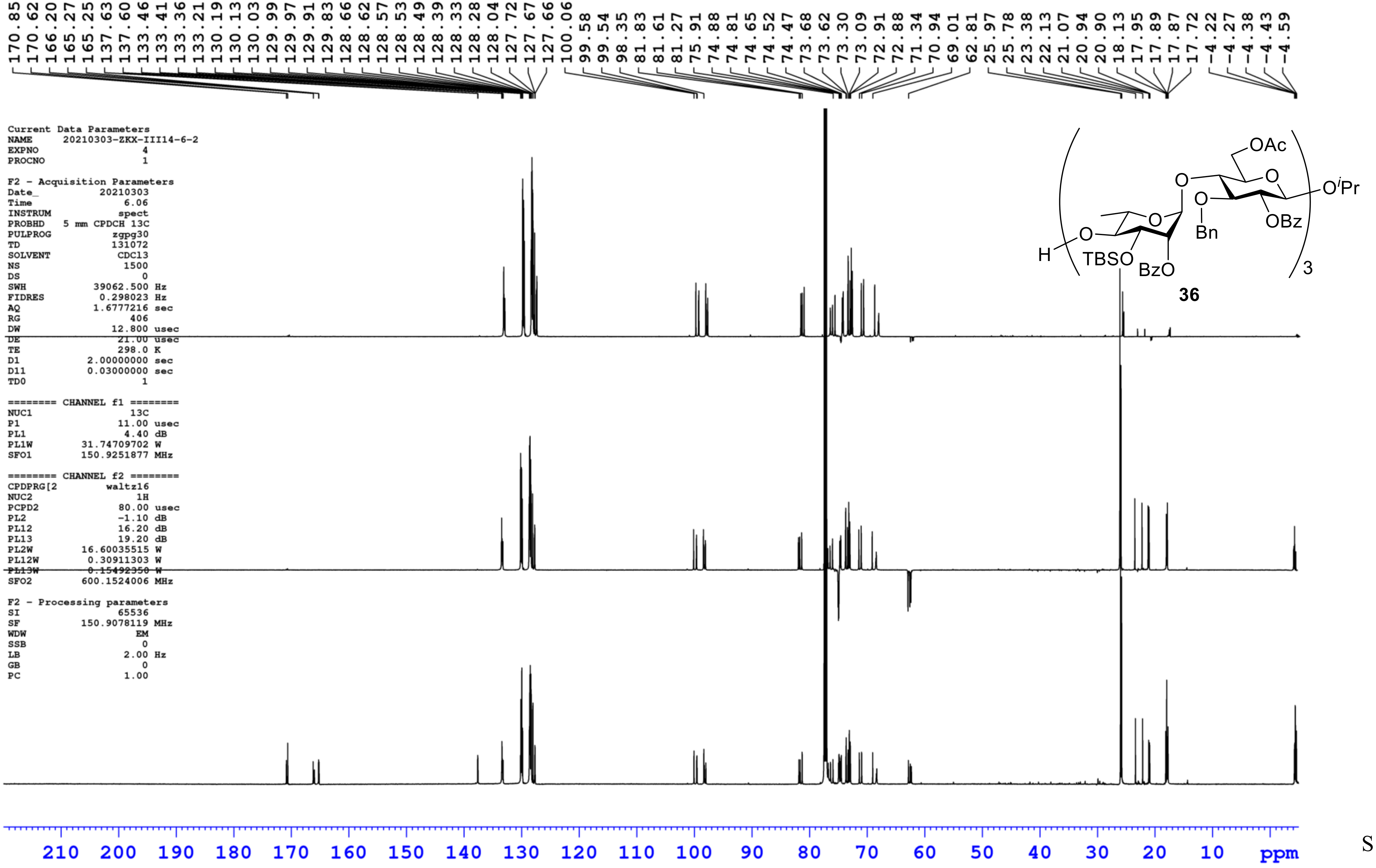
170.85
170.62
166.20
165.27
165.25
137.63
137.60
133.46
133.41
133.36
133.21
130.19
130.13
130.03
129.99
129.97
129.91
129.83
128.66
128.62
128.57
128.53
128.49
128.39
128.33
128.28
128.04
127.72
127.67
127.66
100.06
99.58
99.54
98.35
81.83
81.61
81.27
75.91
74.88
74.81
74.65
74.52
74.47
73.68
73.62
73.30
73.09
72.91
72.88
71.34
70.94
69.01
62.81
25.97
25.78
23.38
22.13
21.07
20.94
20.90
18.13
17.95
17.89
17.87
17.72
-4.22
-4.27
-4.38
-4.43
-4.59
Current Data Parameters
NAME 20210303-ZKX-III14-6-2
EXPNO 4
PROCNO 1
F2 - Acquisition Parameters
Date_ 20210303
Time 6.06
INSTRUM spect
PROBHD 5 mm CPDCH 13C
PULPROG zgpg30
TD 131072
SOLVENT CDCl3
NS 1500
DS 0
SWH 39062.500 Hz
FIDRES 0.298023 Hz
AQ 1.6777216 sec
RG 406
DW 12.800 usec
DE 21.00 usec
TE 298.0 K
D1 2.00000000 sec
D11 0.03000000 sec
TD0 1
======== CHANNEL f1 ========
NUC1 13C
P1 11.00 usec
PL1 4.40 dB
PL1W 31.74709702 W
SFO1 150.9251877 MHz
======== CHANNEL f2 ========
CPDPRG[2 waltz16
NUC2 1H
PCPD2 80.00 usec
PL2 -1.10 dB
PL12 16.20 dB
PL13 19.20 dB
PL2W 16.60035515 W
PL12W 0.30911303 W
PL13W 0.15492350 W
SFO2 600.1524006 MHz
F2 - Processing parameters
SI 65536
SF 150.9078119 MHz
WDW EM
SSB 0
LB 2.00 Hz
GB 0
PC 1.00
OAc
O
O
OⁱPr
O
O
Bn
OBz
H
O
TBSO
BzO
3
36
210 200 190 180 170 160 150 140 130 120 110 100 90 80 70 60 50 40 30 20 10 ppm

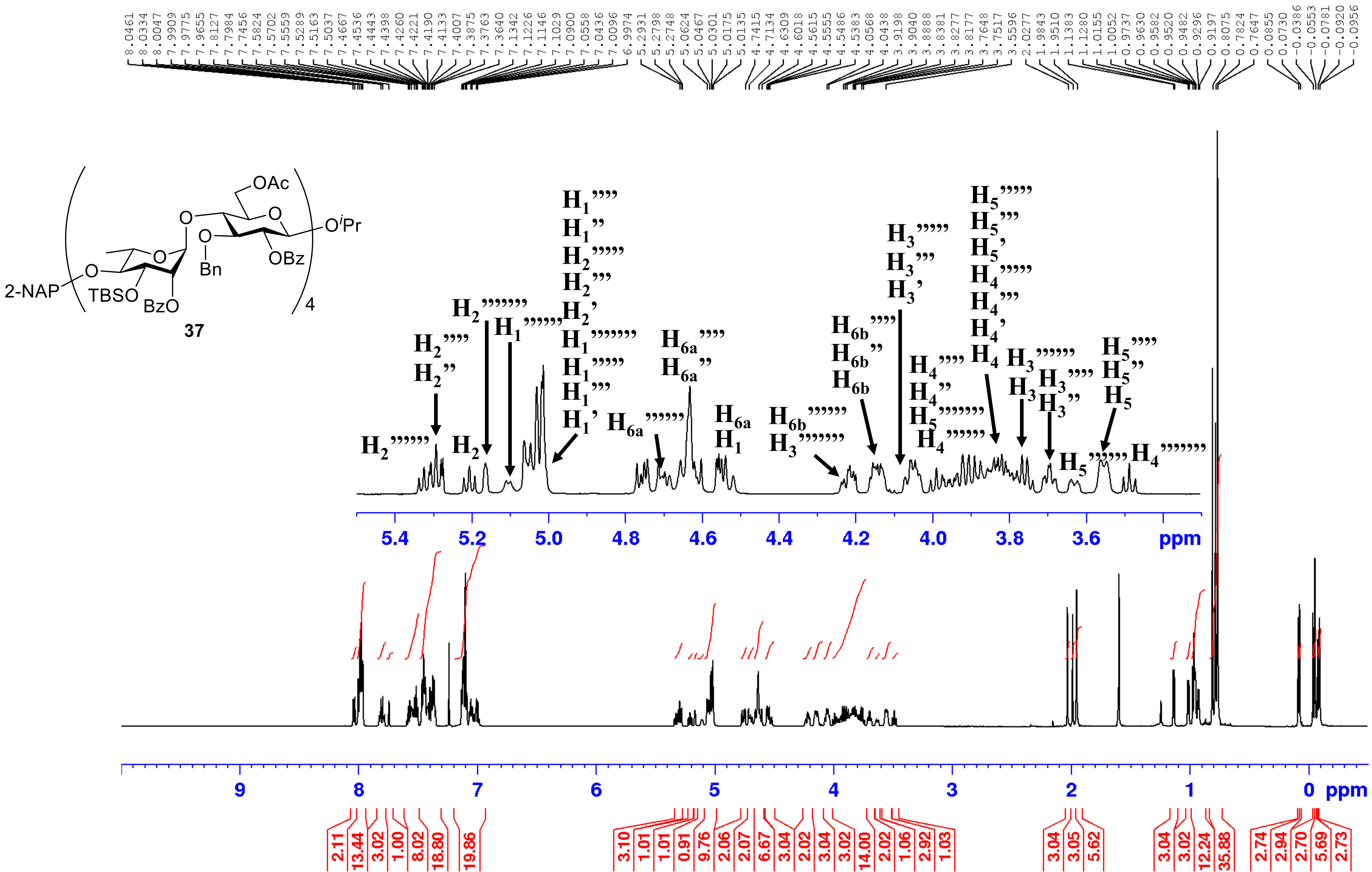

OAc
O
O
O
OiPr
OBz
Bn
2-NAP
TBSO
BzO
4
37
ppm

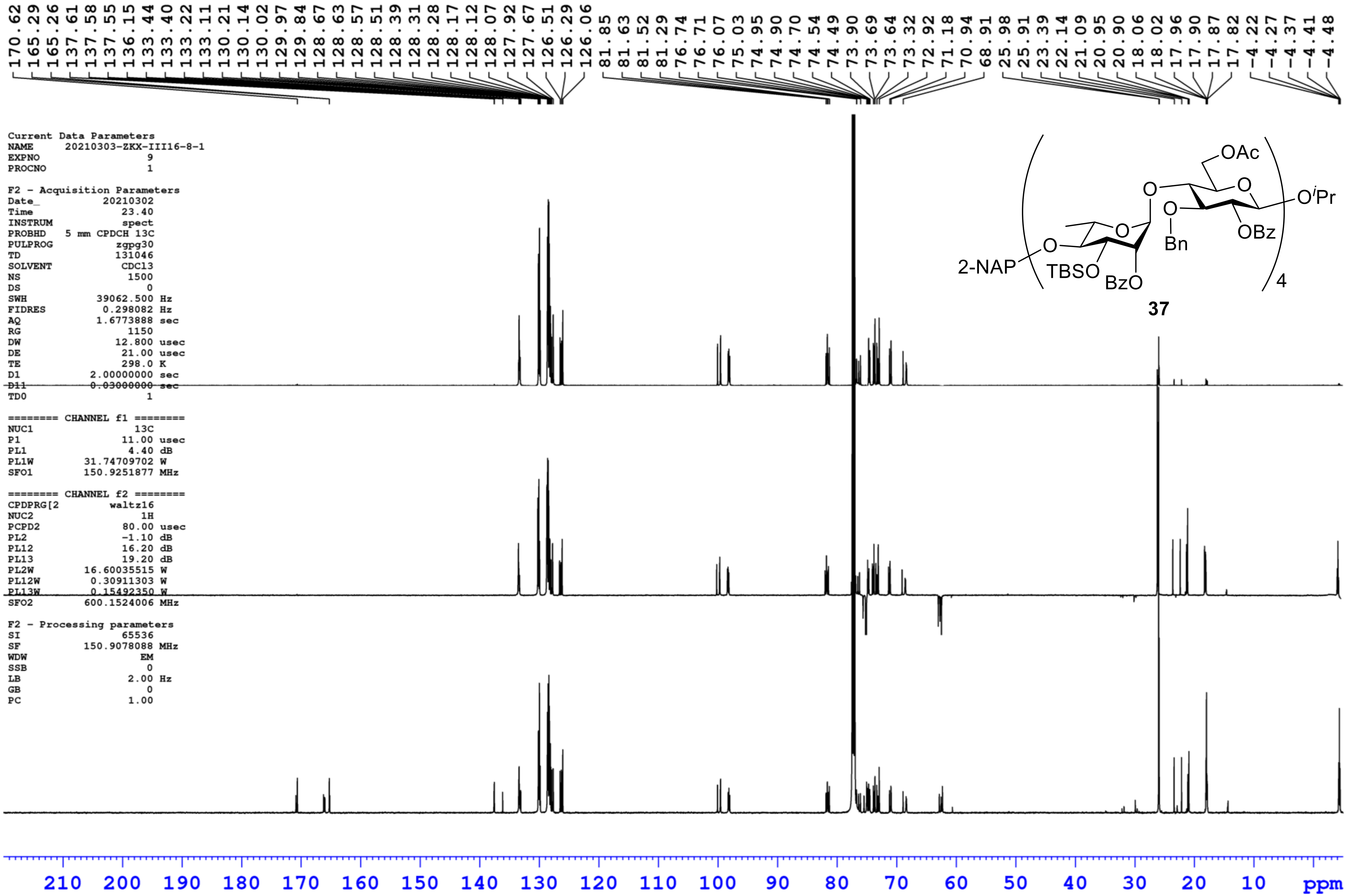
170.62
165.29
165.26
137.61
137.58
137.55
136.15
133.44
133.40
133.22
133.11
130.21
130.14
130.02
129.97
129.84
128.67
128.63
128.57
128.51
128.39
128.31
128.28
128.17
128.12
128.07
127.92
127.67
126.51
126.29
126.06
81.85
81.63
81.52
81.29
76.74
76.71
76.07
75.03
74.95
74.90
74.70
74.54
74.49
73.90
73.69
73.64
73.32
72.92
71.18
70.94
68.91
25.98
25.91
23.39
22.14
21.09
20.95
20.90
18.06
18.02
17.96
17.90
17.87
17.82
-4.22
-4.27
-4.37
-4.41
-4.48
Current Data Parameters
NAME 20210303-ZKX-III16-8-1
EXPNO 9
PROCNO 1
F2 - Acquisition Parameters
Date_ 20210302
Time 23.40
INSTRUM spect
PROBHD 5 mm CPDCH 13C
PULPROG zgpg30
TD 131046
SOLVENT CDCl3
NS 1500
DS 0
SWH 39062.500 Hz
FIDRES 0.298082 Hz
AQ 1.6773888 sec
RG 1150
DW 12.800 usec
DE 21.00 usec
TE 298.0 K
D1 2.00000000 sec
D11 0.03000000 sec
TD0 1
======== CHANNEL f1 ========
NUC1 13C
P1 11.00 usec
PL1 4.40 dB
PL1W 31.74709702 W
SFO1 150.9251877 MHz
======== CHANNEL f2 ========
CPDPRG[2 waltz16
NUC2 1H
PCPD2 80.00 usec
PL2 -1.10 dB
PL12 16.20 dB
PL13 19.20 dB
PL2W 16.60035515 W
PL12W 0.30911303 W
PL13W 0.15492350 W
SFO2 600.1524006 MHz
F2 - Processing parameters
SI 65536
SF 150.9078088 MHz
WDW EM
SSB 0
LB 2.00 Hz
GB 0
PC 1.00
OAc
O
O
O
O^{i}Pr
O
Bn
OBz
O
2-NAP
TBSO
BzO
4
37
210 200 190 180 170 160 150 140 130 120 110 100 90 80 70 60 50 40 30 20 10 ppm

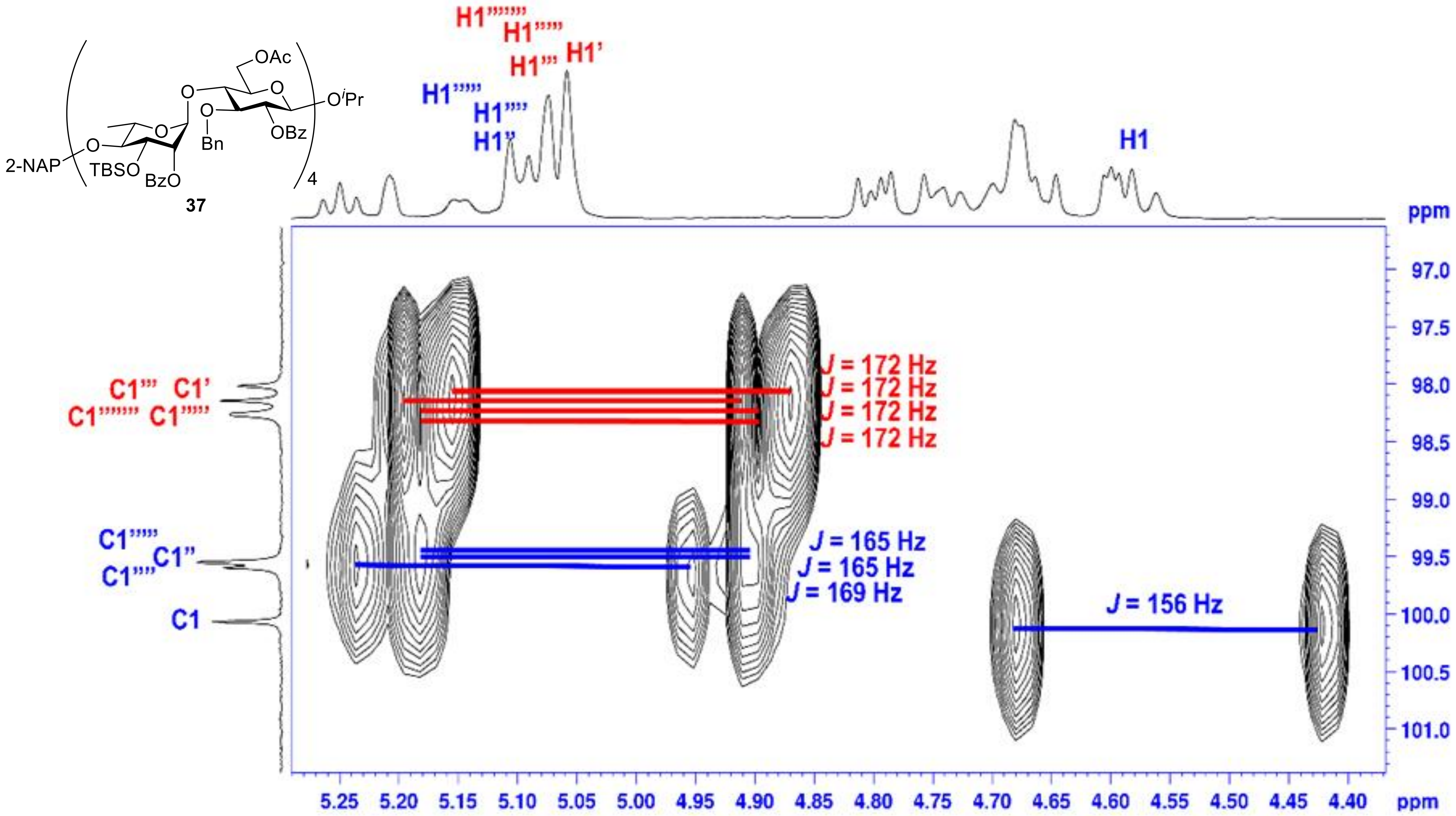
2-NAP
TBSO
BzO
OAc
O
Bn
OBz
O^{i}Pr
4
37
H1'
H1'''
H1'''''
H1'''''''
H1''
H1''''
H1''''''
H1
ppm
C1''' C1'
C1''''''' C1'''''
C1''''''
C1''
C1''''
C1
J = 172 Hz
J = 172 Hz
J = 172 Hz
J = 172 Hz
J = 165 Hz
J = 165 Hz
J = 169 Hz
J = 156 Hz
97.0
97.5
98.0
98.5
99.0
99.5
100.0
100.5
101.0
5.25 5.20 5.15 5.10 5.05 5.00 4.95 4.90 4.85 4.80 4.75 4.70 4.65 4.60 4.55 4.50 4.45 4.40

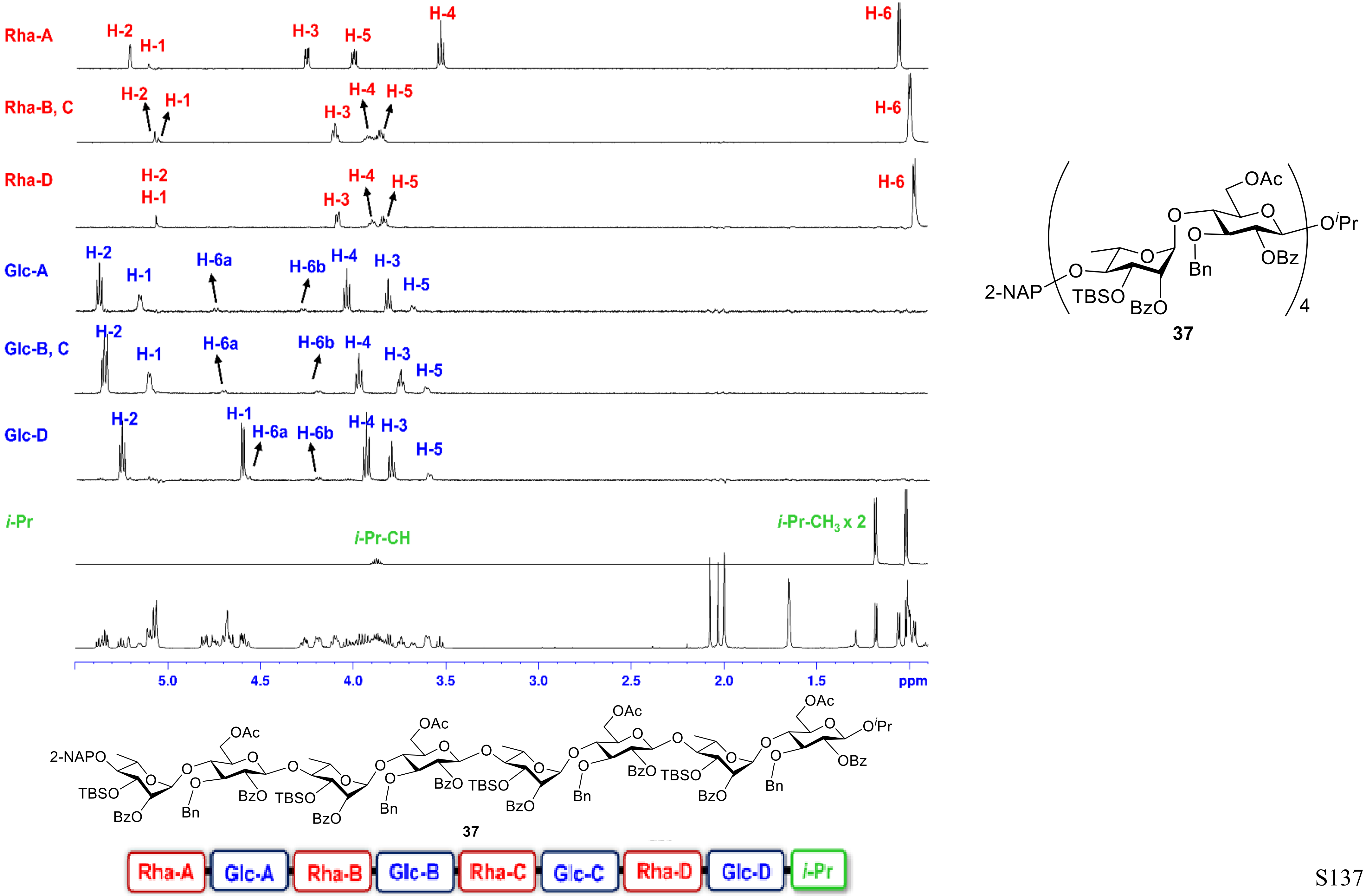

Rha-A
H-2
H-1
H-3
H-5
H-4
H-6
Rha-B, C
H-2
H-1
H-3
H-4
H-5
H-6
Rha-D
H-2
H-1
H-3
H-4
H-5
H-6
Glc-A
H-2
H-1
H-6a
H-6b
H-4
H-3
H-5
Glc-B, C
H-2
H-1
H-6a
H-6b
H-4
H-3
H-5
Glc-D
H-2
H-1
H-6a
H-6b
H-4
H-3
H-5
i-Pr
i-Pr-CH
i-Pr-CH3 x 2
5.0
4.5
4.0
3.5
3.0
2.5
2.0
1.5
ppm
2-NAP
TBSO
BzO
OAc
O
Bn
OBz
O^iPr
4
37
2-NAPO
37
Rha-A
Glc-A
Rha-B
Glc-B
Rha-C
Glc-C
Rha-D
Glc-D
i-Pr

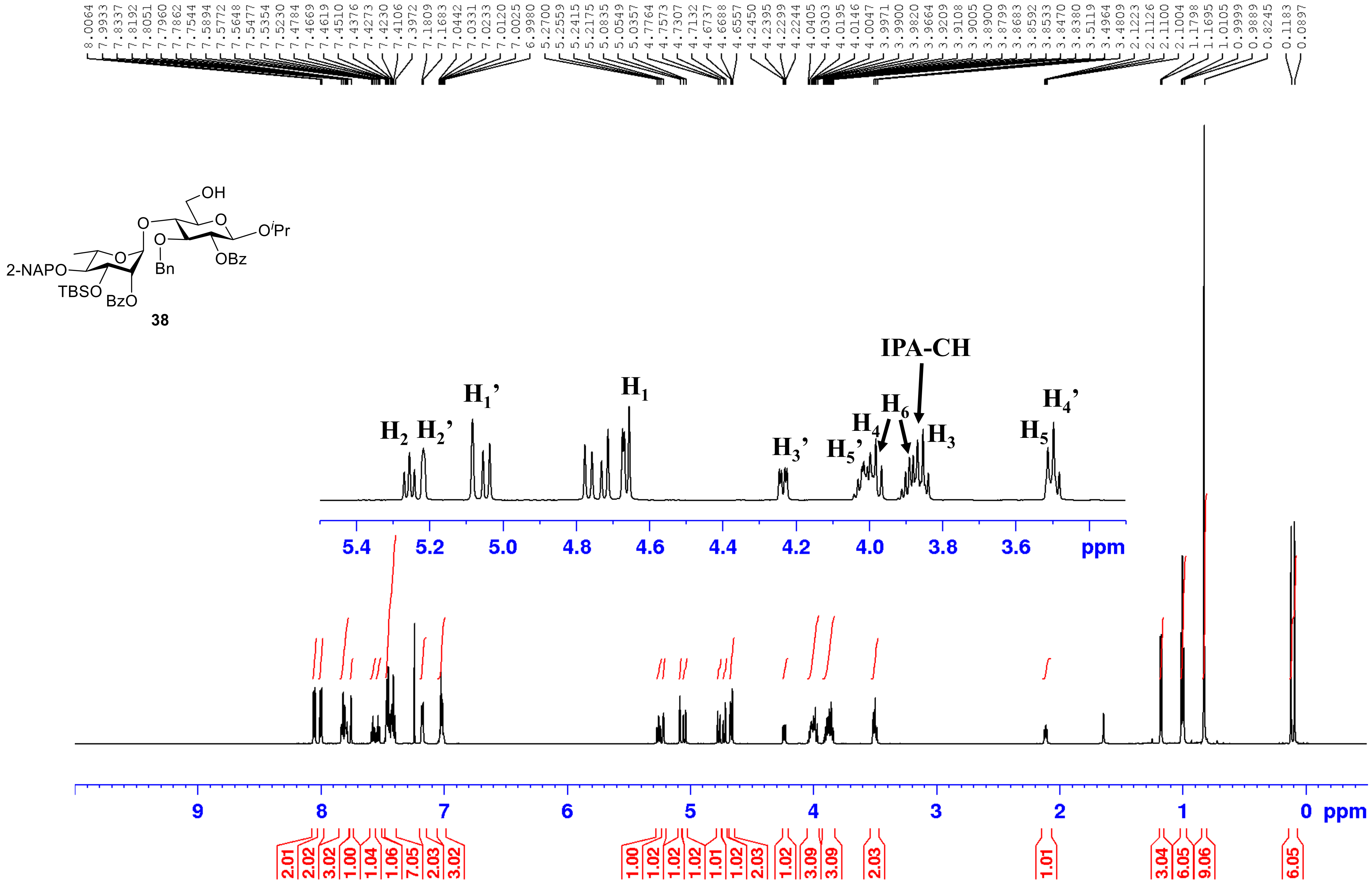

2-NAPO
TBSO
BzO
O
Bn
OBz
OH
O^{i}Pr
38
IPA-CH
H$_1$
H$_1$'
H$_2$
H$_2$'
H$_3$'
H$_5$'
H$_4$
H$_6$
H$_3$
H$_5$
H$_4$'
ppm

20210219-ZKX-2-3-2-C

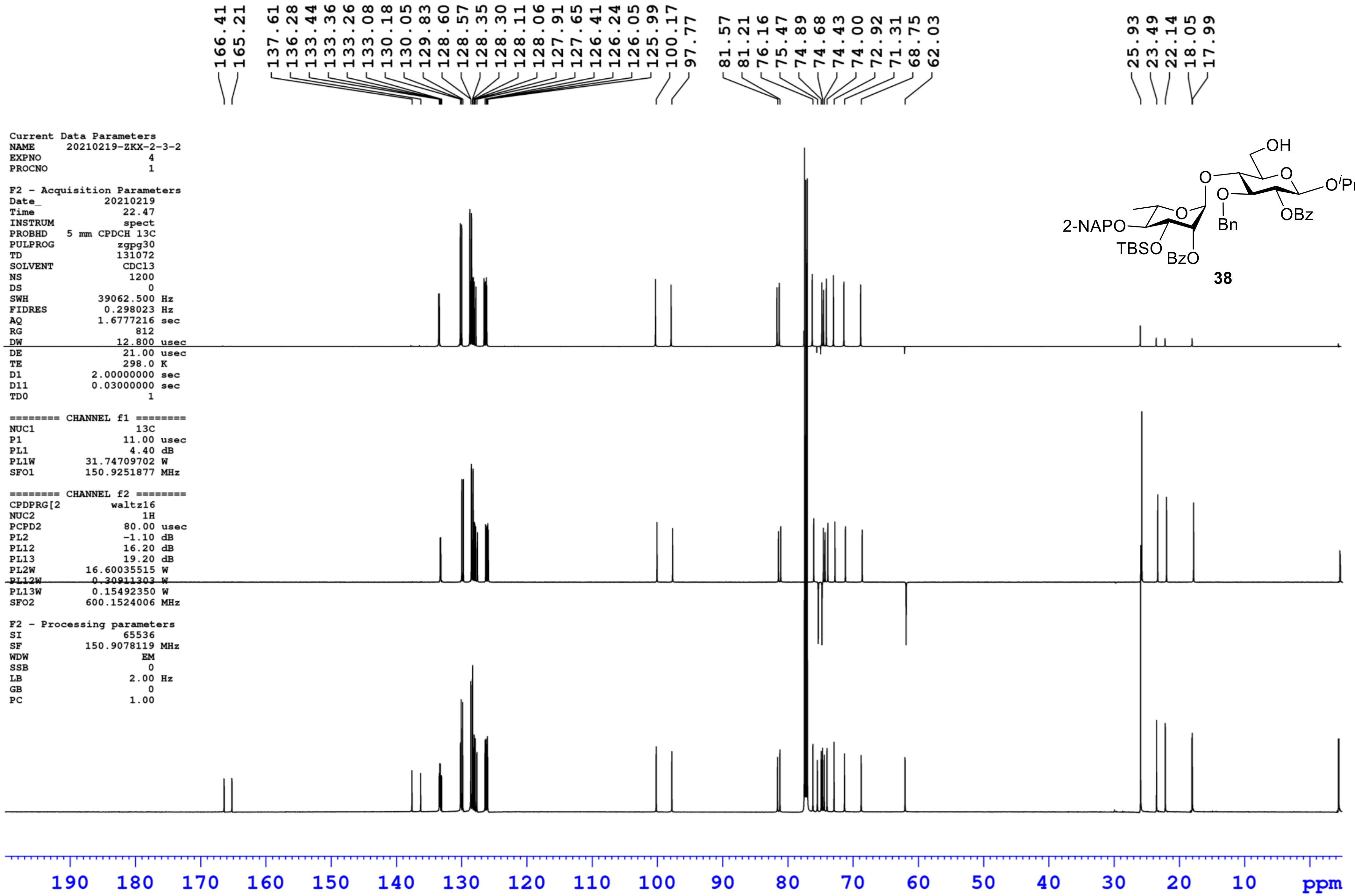

38

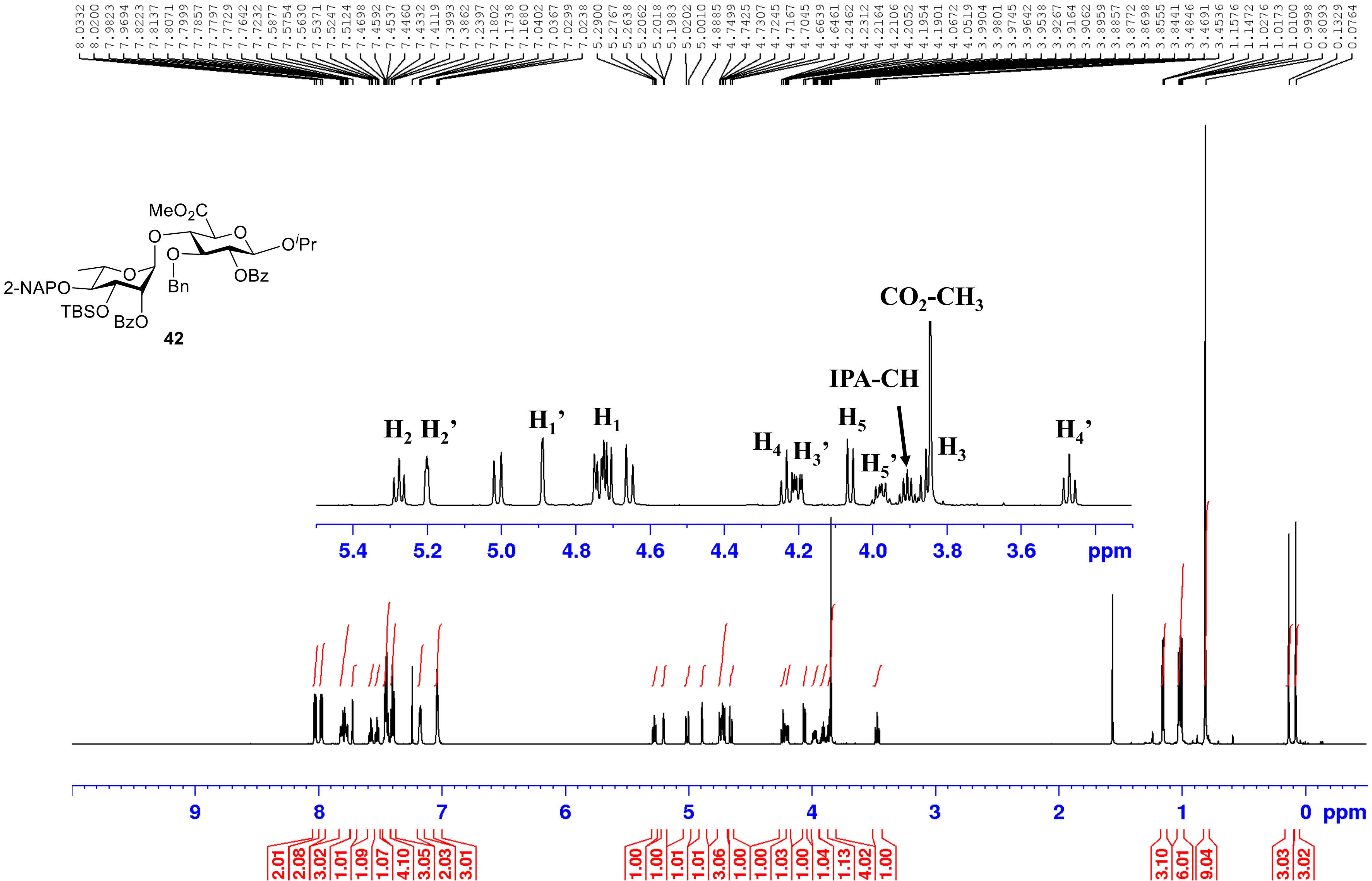
MeO2C
O
O
OiPr
O
2-NAPO
O
Bn
OBz
TBSO
BzO
42
CO2-CH3
IPA-CH
H2 H2'
H1'
H1
H4
H3'
H5
H5'
H3
H4'
ppm

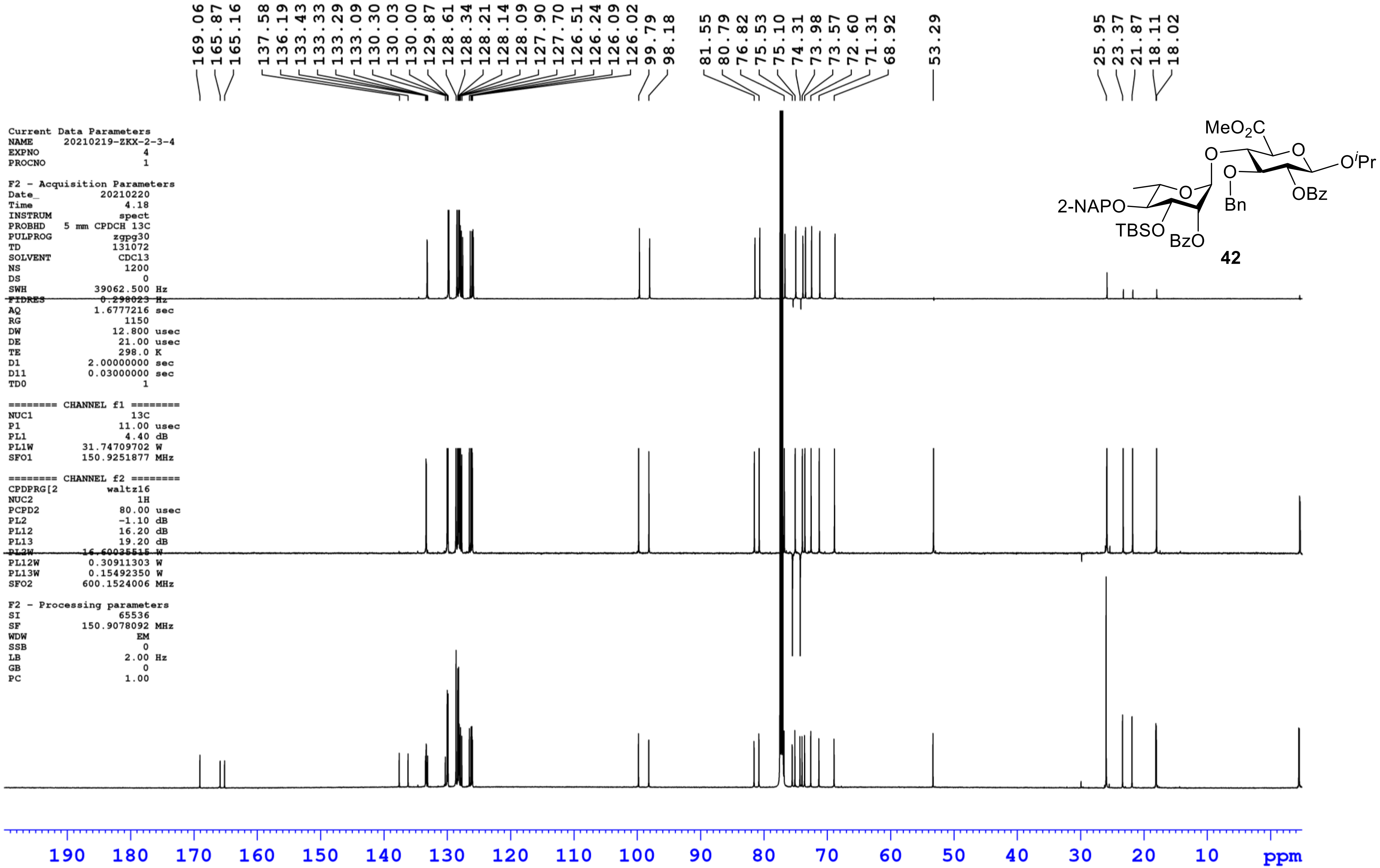
169.06
165.87
165.16
137.58
136.19
133.43
133.33
133.29
133.09
130.30
130.03
130.00
129.87
128.61
128.34
128.21
128.14
128.09
127.90
127.70
126.51
126.24
126.09
126.02
99.79
98.18
81.55
80.79
76.82
75.53
75.10
74.31
73.98
73.57
72.60
71.31
68.92
53.29
25.95
23.37
21.87
18.11
18.02
Current Data Parameters
NAME 20210219-ZKX-2-3-4
EXPNO 4
PROCNO 1
F2 - Acquisition Parameters
Date_ 20210220
Time 4.18
INSTRUM spect
PROBHD 5 mm CPDCH 13C
PULPROG zgpg30
TD 131072
SOLVENT CDCl3
NS 1200
DS 0
SWH 39062.500 Hz
FIDRES 0.298023 Hz
AQ 1.6777216 sec
RG 1150
DW 12.800 usec
DE 21.00 usec
TE 298.0 K
D1 2.00000000 sec
D11 0.03000000 sec
TD0 1
======== CHANNEL f1 ========
NUC1 13C
P1 11.00 usec
PL1 4.40 dB
PL1W 31.74709702 W
SFO1 150.9251877 MHz
======== CHANNEL f2 ========
CPDPRG[2 waltz16
NUC2 1H
PCPD2 80.00 usec
PL2 -1.10 dB
PL12 16.20 dB
PL13 19.20 dB
PL2W 16.60035515 W
PL12W 0.30911303 W
PL13W 0.15492350 W
SFO2 600.1524006 MHz
F2 - Processing parameters
SI 65536
SF 150.9078092 MHz
WDW EM
SSB 0
LB 2.00 Hz
GB 0
PC 1.00
190
180
170
160
150
140
130
120
110
100
90
80
70
60
50
40
30
20
10
ppm
MeO2C
O
O
OiPr
O
2-NAPO
O
Bn
OBz
TBSO
BzO

42

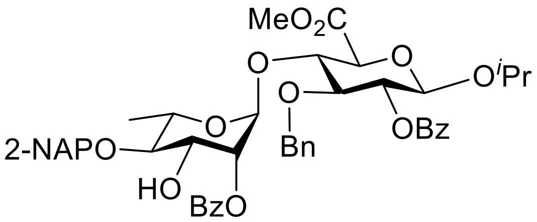
MeO2C
O
O
O^{i}Pr
O
Bn
OBz
2-NAPO
HO
BzO

46

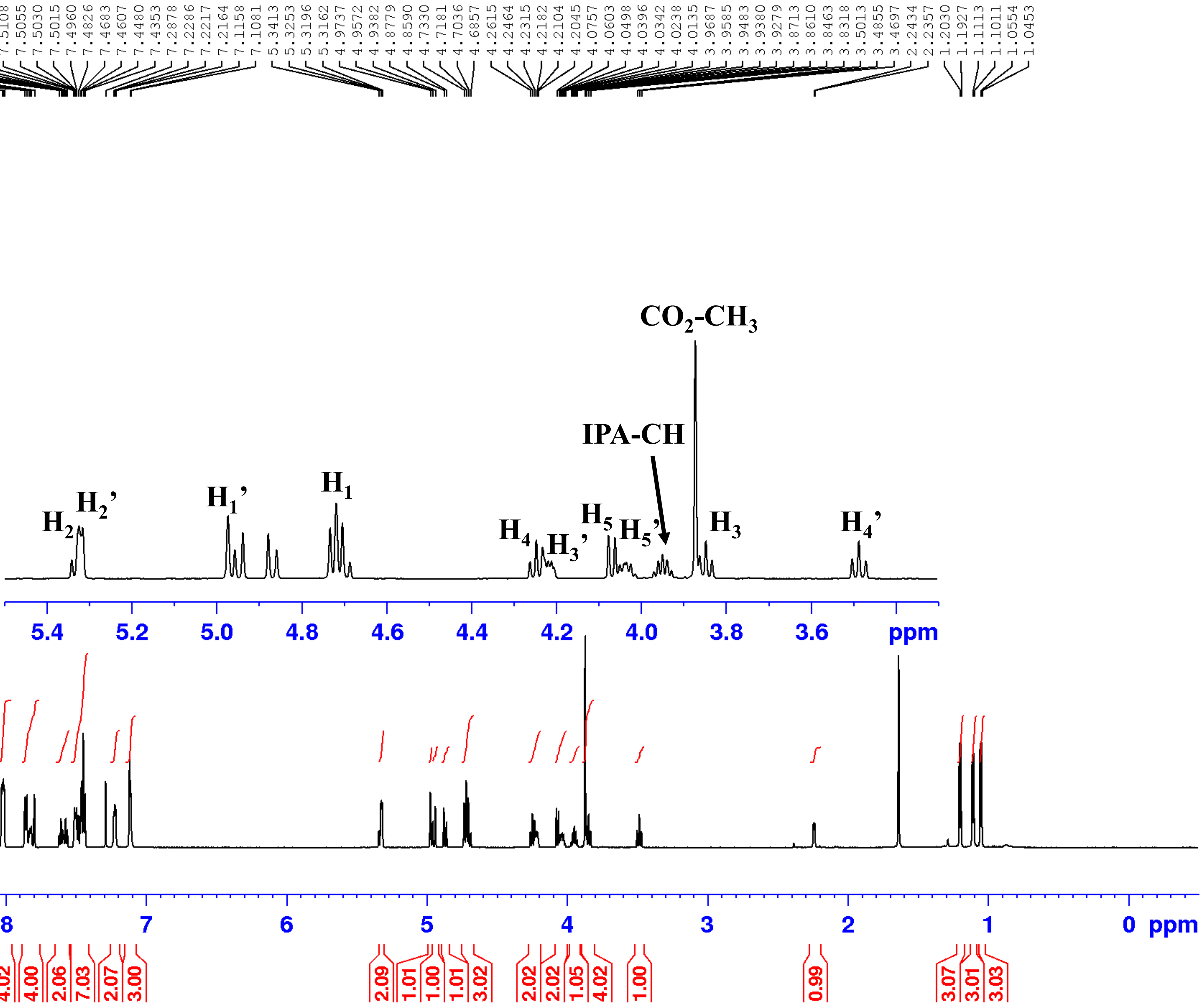
8.0240
8.0175
8.0111
7.8642
7.8501
7.8329
7.8265
7.8179
7.7965
7.6195
7.6072
7.5948
7.5862
7.5739
7.5616
7.5167
7.5108
7.5055
7.5030
7.5015
7.4960
7.4826
7.4683
7.4607
7.4480
7.4353
7.2878
7.2286
7.2217
7.2164
7.1158
7.1081
5.3413
5.3253
5.3196
5.3162
4.9737
4.9572
4.9382
4.8779
4.8590
4.7330
4.7181
4.7036
4.6857
4.2615
4.2464
4.2315
4.2182
4.2104
4.2045
4.0757
4.0603
4.0498
4.0396
4.0342
4.0238
4.0135
3.9687
3.9585
3.9483
3.9380
3.9279
3.8713
3.8610
3.8463
3.8318
3.5013
3.4855
3.4697
2.2434
2.2357
1.2030
1.1927
1.1113
1.1011
1.0554
1.0453
CO2-CH3
IPA-CH
H2
H2'
H1'
H1
H4
H3'
H5
H5'
H3
H4'
5.4
5.2
5.0
4.8
4.6
4.4
4.2
4.0
3.8
3.6
ppm
9
8
7
6
5
4
3
2
1
0 ppm
4.02
4.00
2.06
7.03
2.07
3.00
2.09
1.01
1.00
1.01
3.02
2.02
2.02
1.05
4.02
1.00
0.99
3.07
3.01
3.03

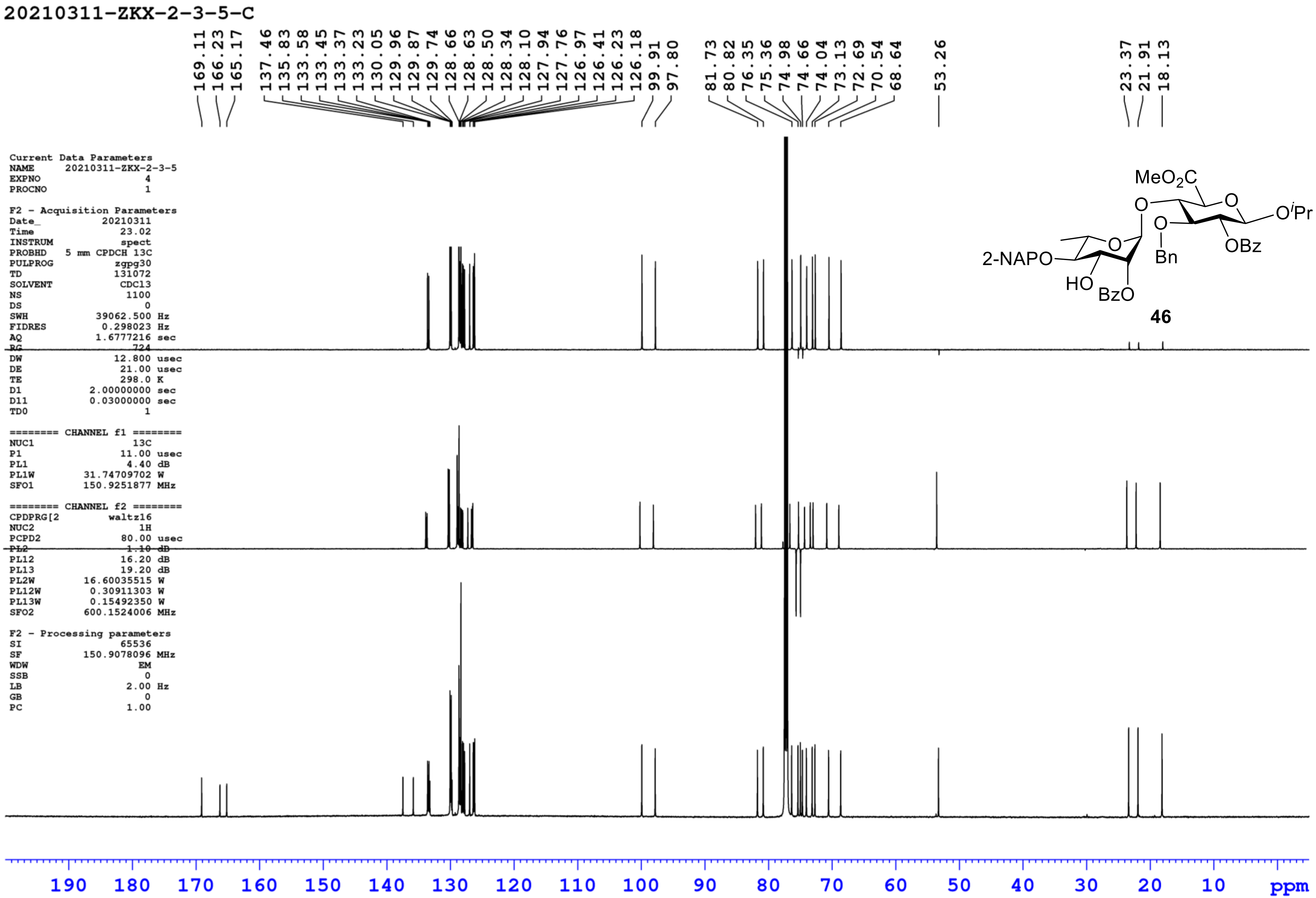
20210311-ZKX-2-3-5-C
169.11
166.23
165.17
137.46
135.83
133.58
133.45
133.37
133.23
130.05
129.96
129.87
129.74
128.66
128.63
128.50
128.34
128.10
127.94
127.76
126.97
126.41
126.23
126.18
99.91
97.80
81.73
80.82
76.35
75.36
74.98
74.66
74.04
73.13
72.69
70.54
68.64
53.26
23.37
21.91
18.13
Current Data Parameters
NAME 20210311-ZKX-2-3-5
EXPNO 4
PROCNO 1
F2 - Acquisition Parameters
Date_ 20210311
Time 23.02
INSTRUM spect
PROBHD 5 mm CPDCH 13C
PULPROG zgpg30
TD 131072
SOLVENT CDCl3
NS 1100
DS 0
SWH 39062.500 Hz
FIDRES 0.298023 Hz
AQ 1.6777216 sec
RG 724
DW 12.800 usec
DE 21.00 usec
TE 298.0 K
D1 2.00000000 sec
D11 0.03000000 sec
TD0 1
======== CHANNEL f1 ========
NUC1 13C
P1 11.00 usec
PL1 4.40 dB
PL1W 31.74709702 W
SFO1 150.9251877 MHz
======== CHANNEL f2 ========
CPDPRG[2 waltz16
NUC2 1H
PCPD2 80.00 usec
PL2 1.10 dB
PL12 16.20 dB
PL13 19.20 dB
PL2W 16.60035515 W
PL12W 0.30911303 W
PL13W 0.15492350 W
SFO2 600.1524006 MHz
F2 - Processing parameters
SI 65536
SF 150.9078096 MHz
WDW EM
SSB 0
LB 2.00 Hz
GB 0
PC 1.00
MeO2C
O
O
OiPr
O
2-NAPO
O
Bn
OBz
HO
BzO
46
190
180
170
160
150
140
130
120
110
100
90
80
70
60
50
40
30
20
10
ppm

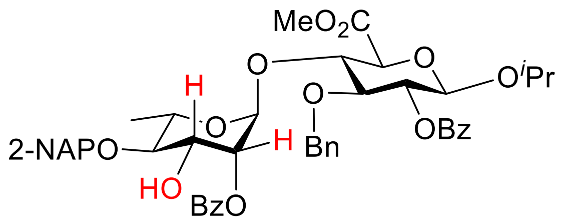
MeO2C
O
O
OiPr
H
O
O
H
Bn
OBz
2-NAPO
HO
BzO
46

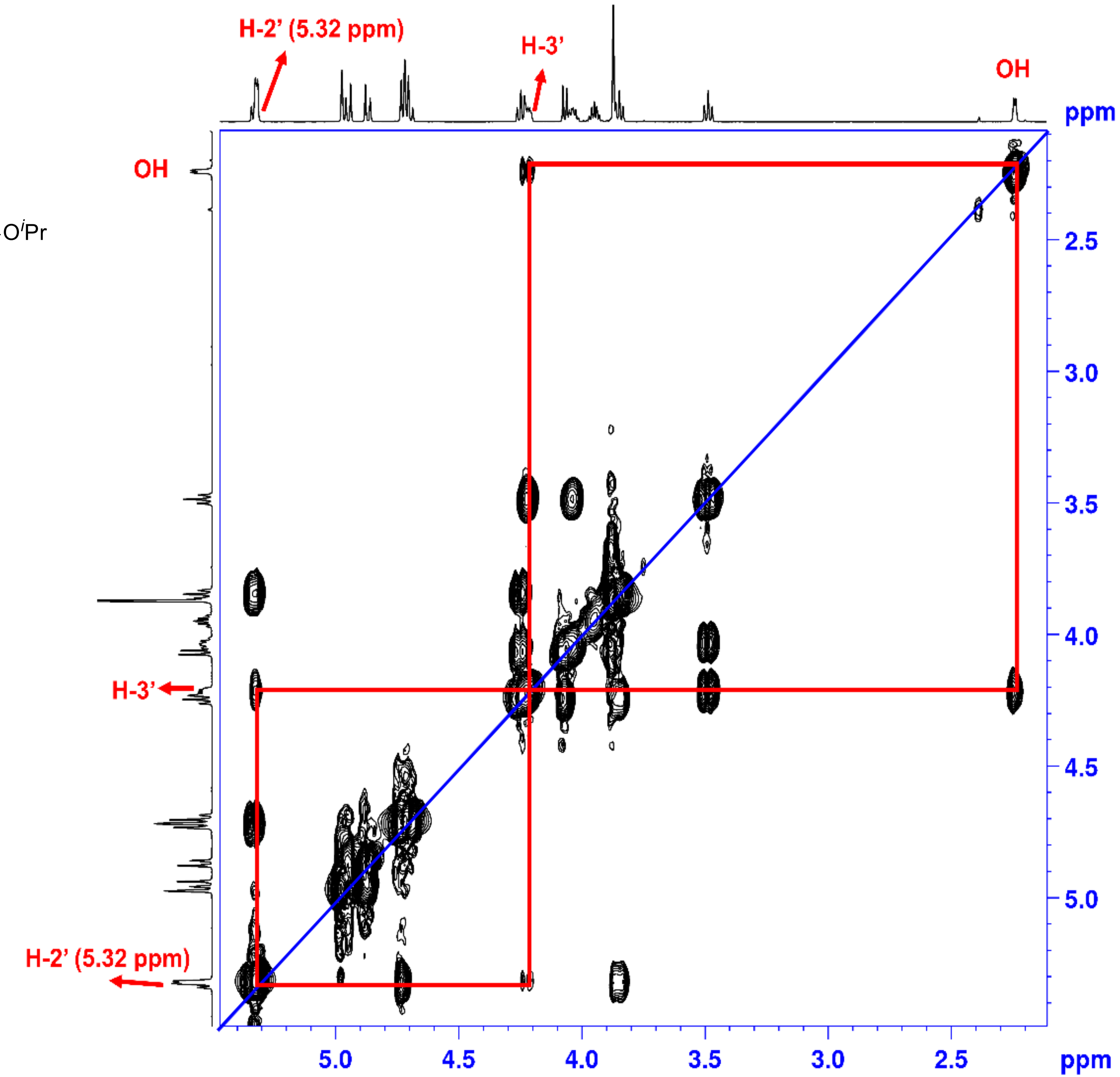
H-2' (5.32 ppm)
H-3'
OH
ppm
OH
H-3'
H-2' (5.32 ppm)
2.5
3.0
3.5
4.0
4.5
5.0
5.0
4.5
4.0
3.5
3.0
2.5
ppm

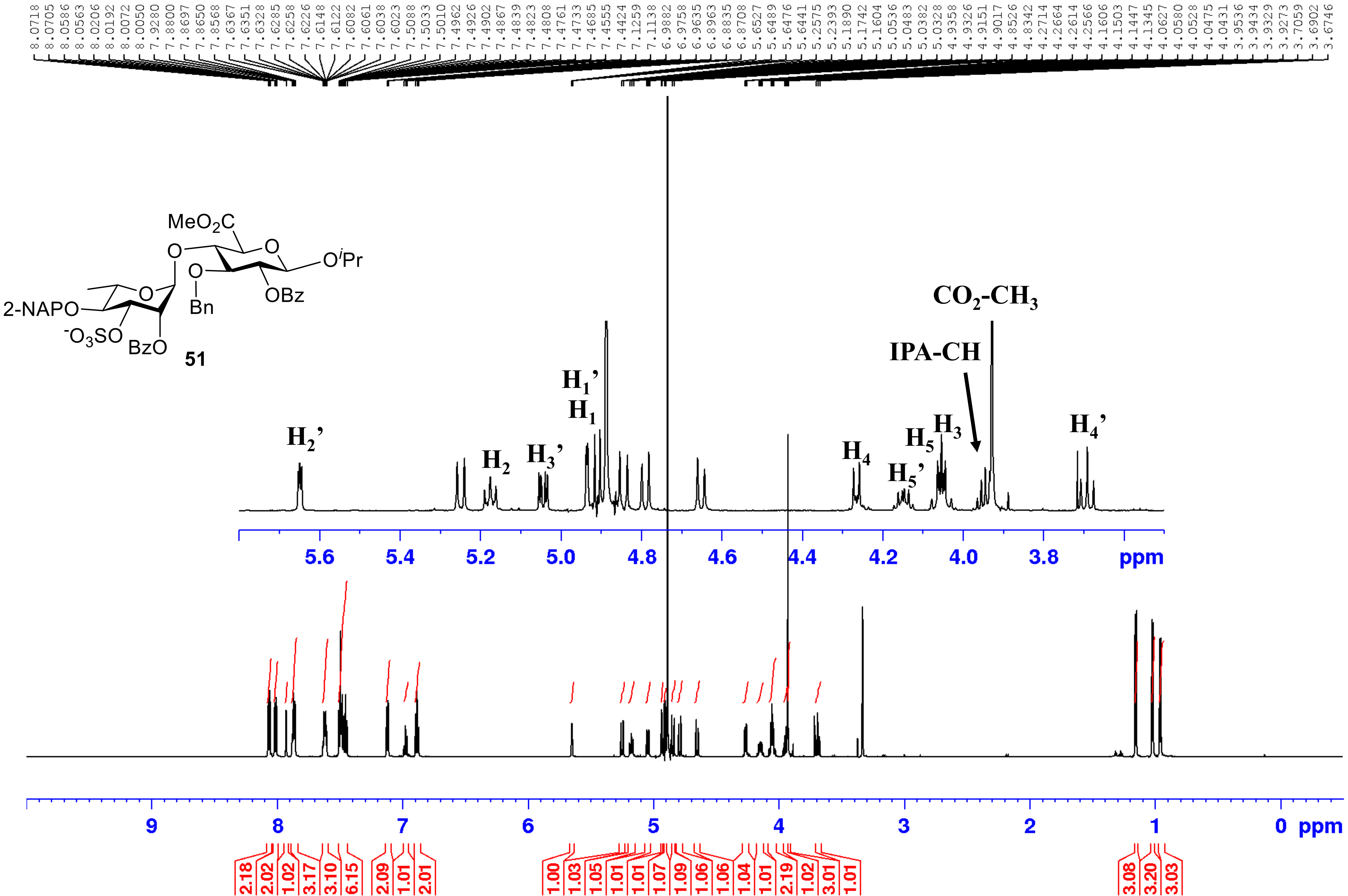

MeO2C
O
O
O^iPr
O
Bn
OBz
2-NAPO
O
-O3SO
BzO
51
CO2-CH3
IPA-CH
H1'
H1
H2'
H2
H3'
H4
H5
H3
H5'
H4'
ppm

20201203-ZKX-II54-2-3-6-C

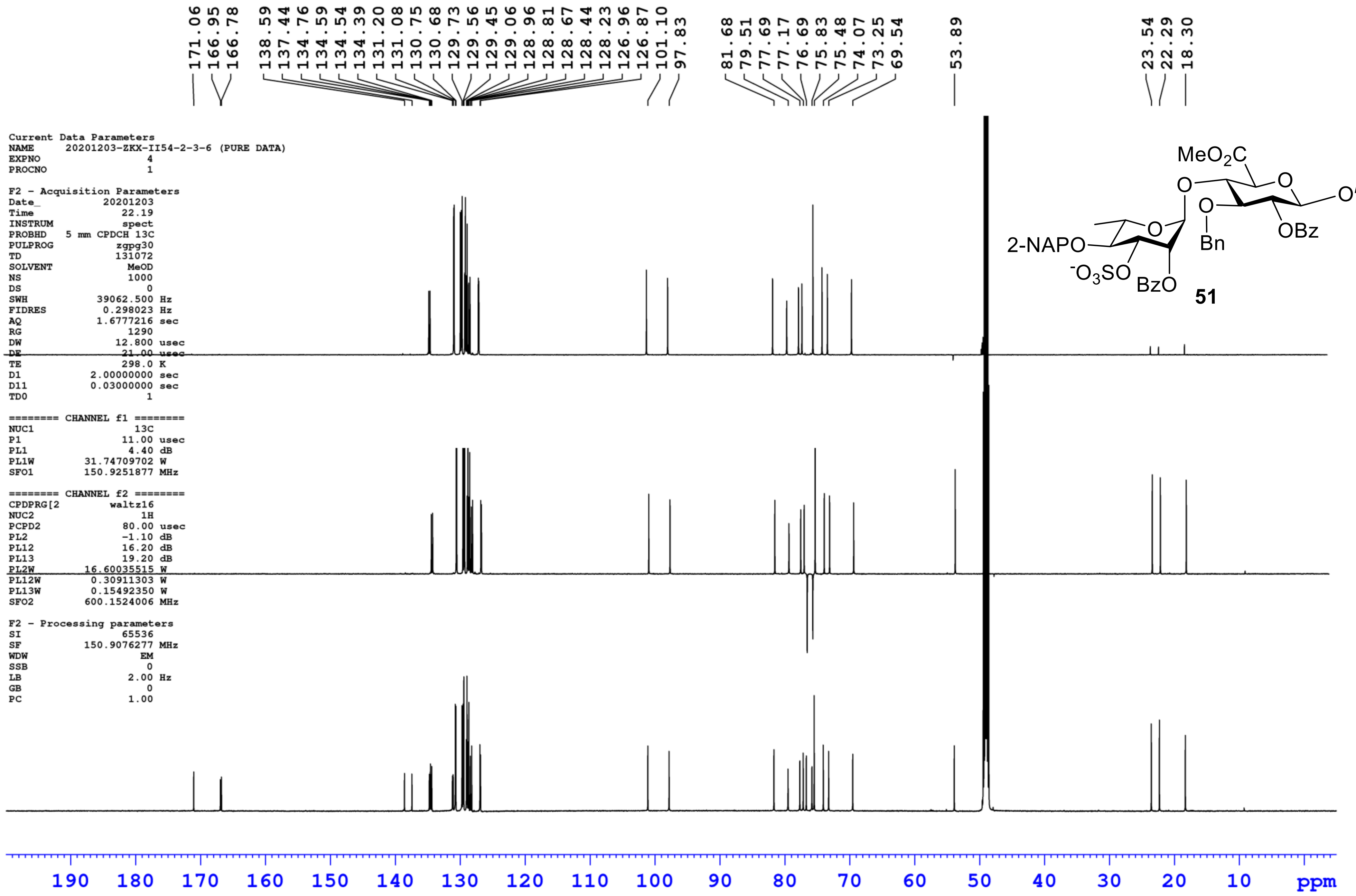

51

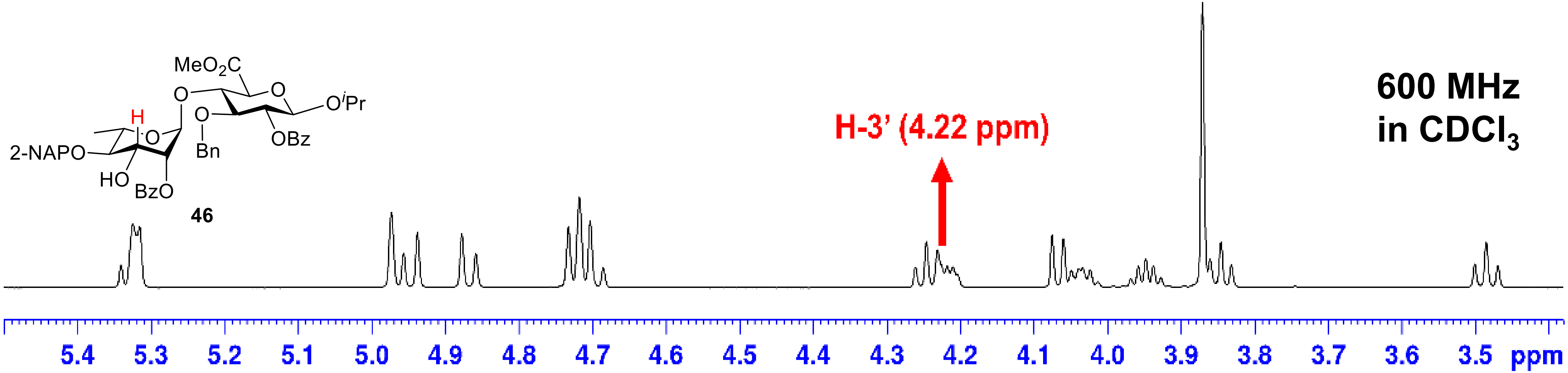

MeO2C
O
O
OiPr
H
O
O
2-NAPO
Bn
OBz
HO
BzO
46
600 MHz
in CDCl3
H-3' (4.22 ppm)
5.4 5.3 5.2 5.1 5.0 4.9 4.8 4.7 4.6 4.5 4.4 4.3 4.2 4.1 4.0 3.9 3.8 3.7 3.6 3.5 ppm

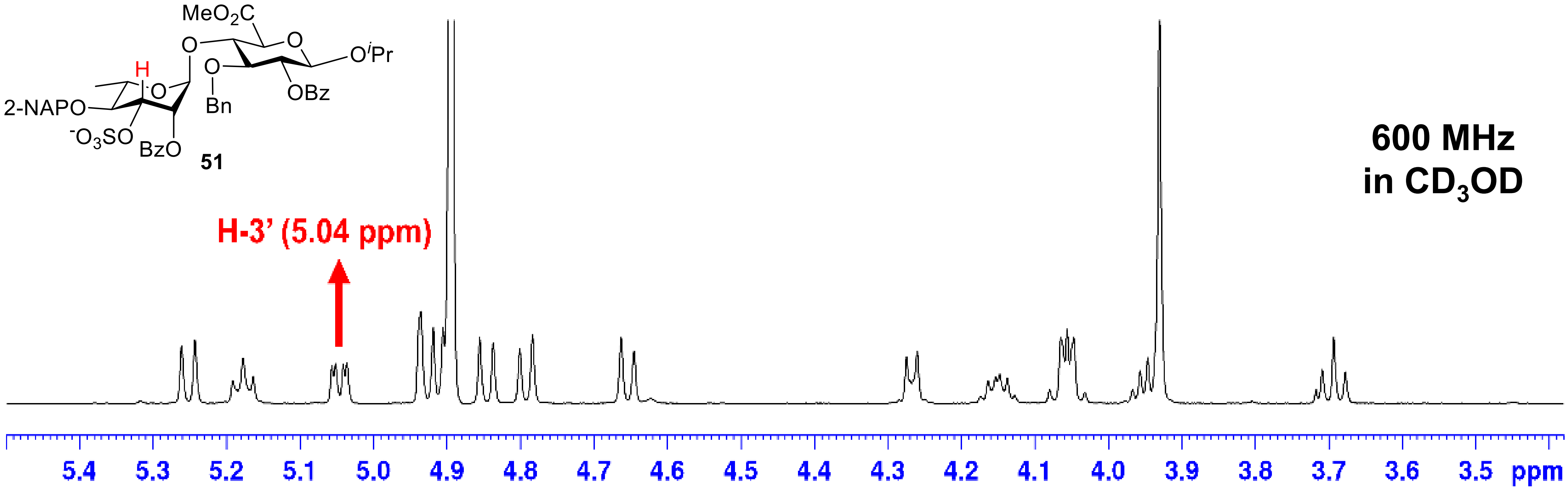

MeO2C
O
O
OiPr
H
O
O
2-NAPO
Bn
OBz
-O3SO
BzO
51
600 MHz
in CD3OD
H-3' (5.04 ppm)
5.4 5.3 5.2 5.1 5.0 4.9 4.8 4.7 4.6 4.5 4.4 4.3 4.2 4.1 4.0 3.9 3.8 3.7 3.6 3.5 ppm

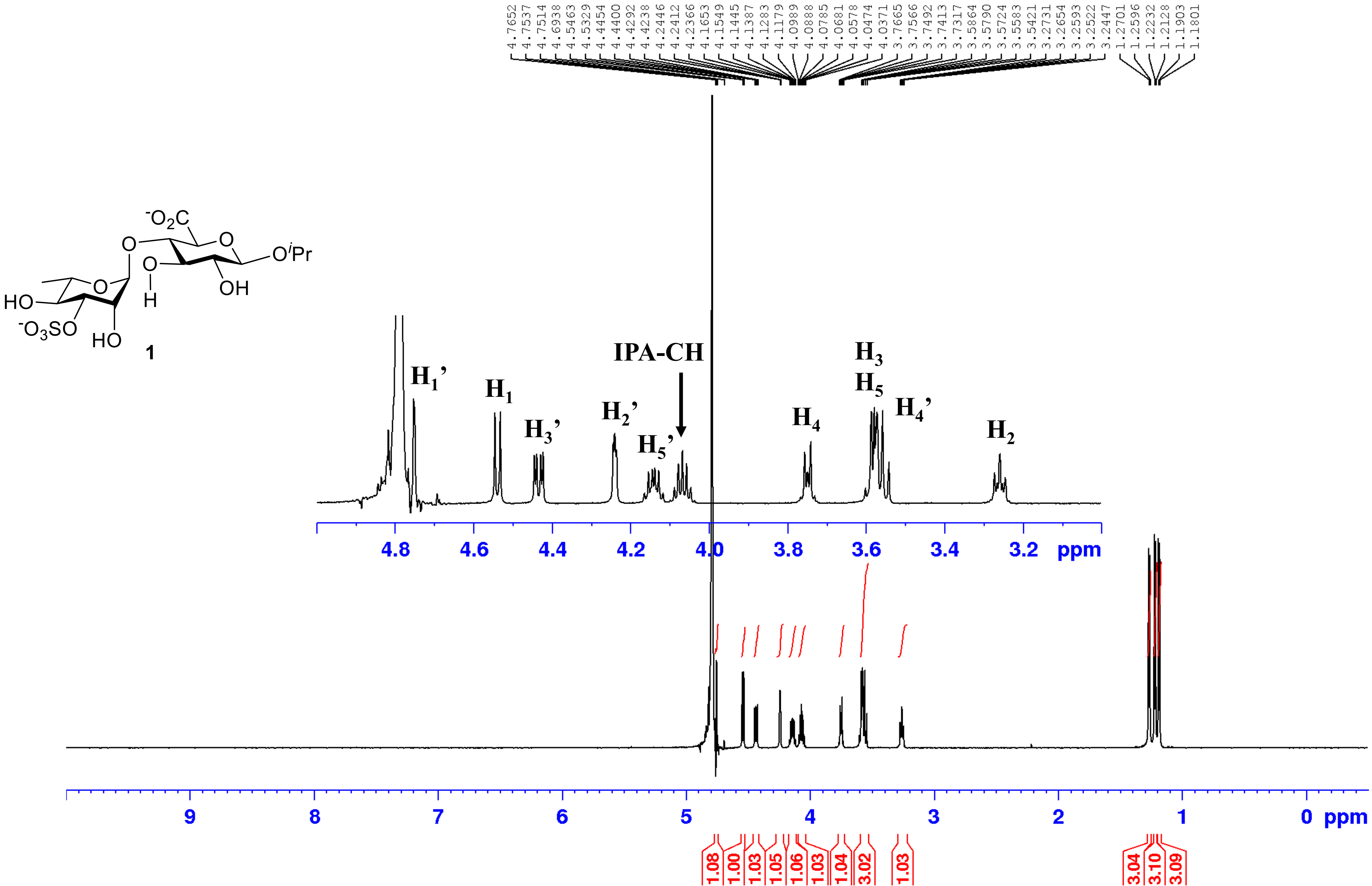

IPA-CH
H1'
H1
H3'
H2'
H5'
H4
H3
H5
H4'
H2
1
ppm

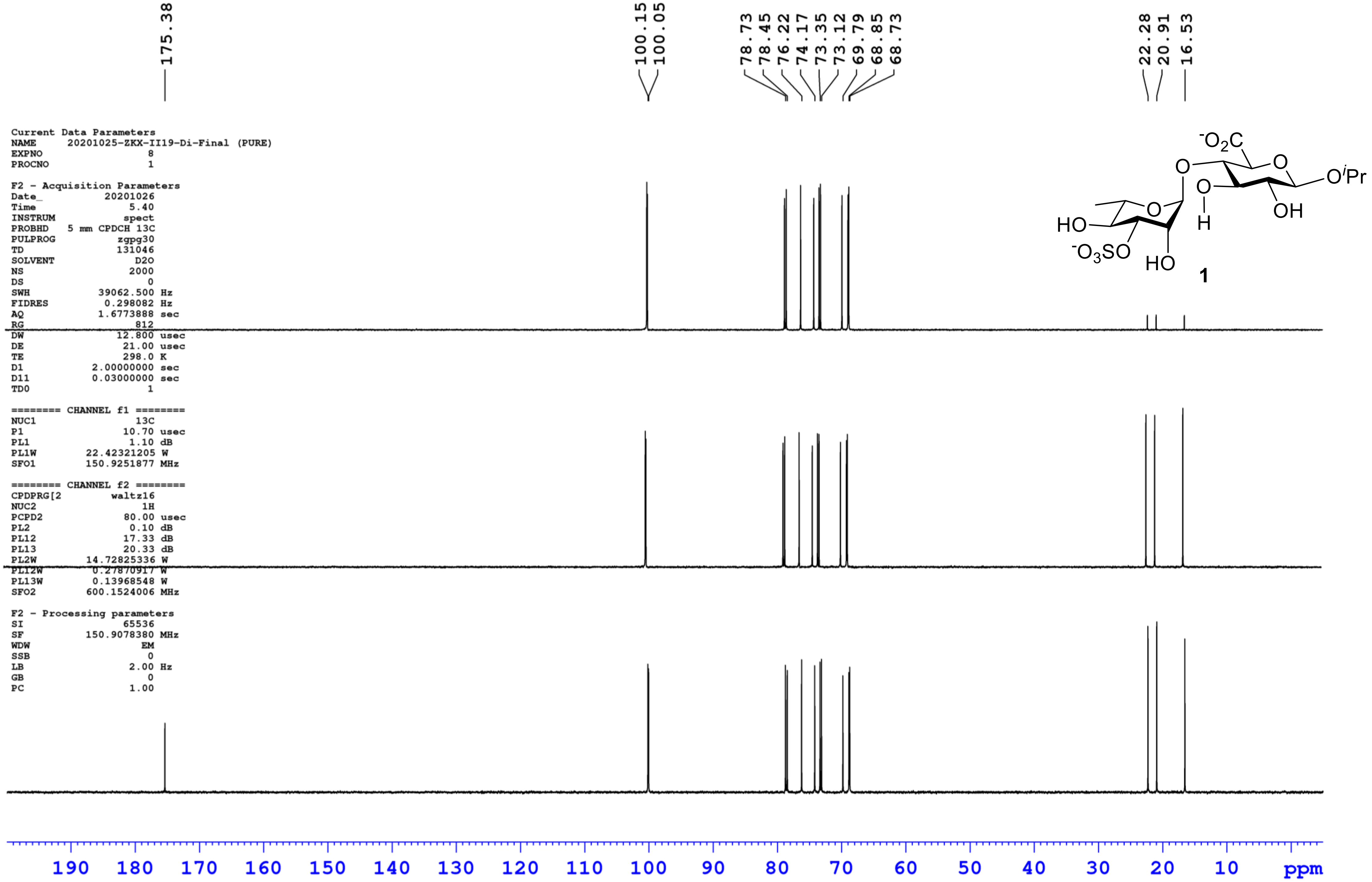
175.38
100.15
100.05
78.73
78.45
76.22
74.17
73.35
73.12
69.79
68.85
68.73
22.28
20.91
16.53
Current Data Parameters
NAME 20201025-ZKX-II19-Di-Final (PURE)
EXPNO 8
PROCNO 1
F2 - Acquisition Parameters
Date_ 20201026
Time 5.40
INSTRUM spect
PROBHD 5 mm CPDCH 13C
PULPROG zgpg30
TD 131046
SOLVENT D2O
NS 2000
DS 0
SWH 39062.500 Hz
FIDRES 0.298082 Hz
AQ 1.6773888 sec
RG 812
DW 12.800 usec
DE 21.00 usec
TE 298.0 K
D1 2.00000000 sec
D11 0.03000000 sec
TD0 1
======== CHANNEL f1 ========
NUC1 13C
P1 10.70 usec
PL1 1.10 dB
PL1W 22.42321205 W
SFO1 150.9251877 MHz
======== CHANNEL f2 ========
CPDPRG[2 waltz16
NUC2 1H
PCPD2 80.00 usec
PL2 0.10 dB
PL12 17.33 dB
PL13 20.33 dB
PL2W 14.72825336 W
PL12W 0.27870917 W
PL13W 0.13968548 W
SFO2 600.1524006 MHz
F2 - Processing parameters
SI 65536
SF 150.9078380 MHz
WDW EM
SSB 0
LB 2.00 Hz
GB 0
PC 1.00
1
190 180 170 160 150 140 130 120 110 100 90 80 70 60 50 40 30 20 10 ppm

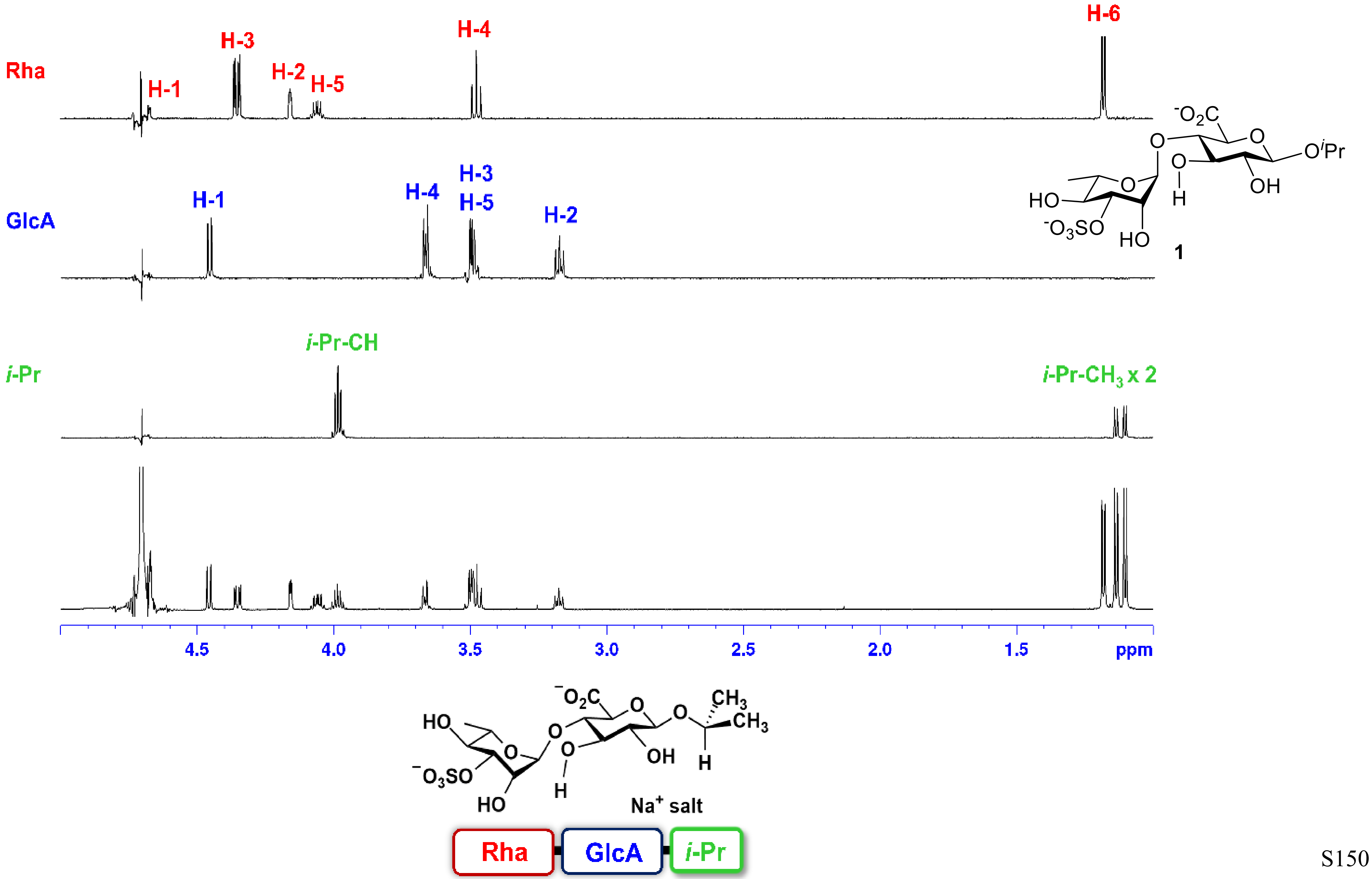
Rha
H-1
H-3
H-2
H-5
H-4
H-6
GlcA
H-1
H-4
H-3
H-5
H-2
i-Pr
i-Pr-CH
i-Pr-CH3 x 2
4.5
4.0
3.5
3.0
2.5
2.0
1.5
ppm
1
Na+ salt
Rha
GlcA
i-Pr

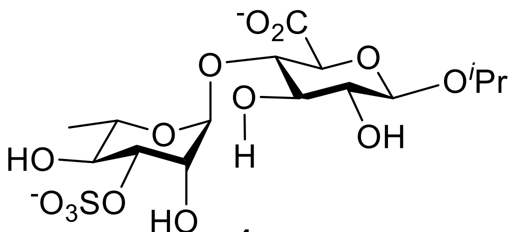

1

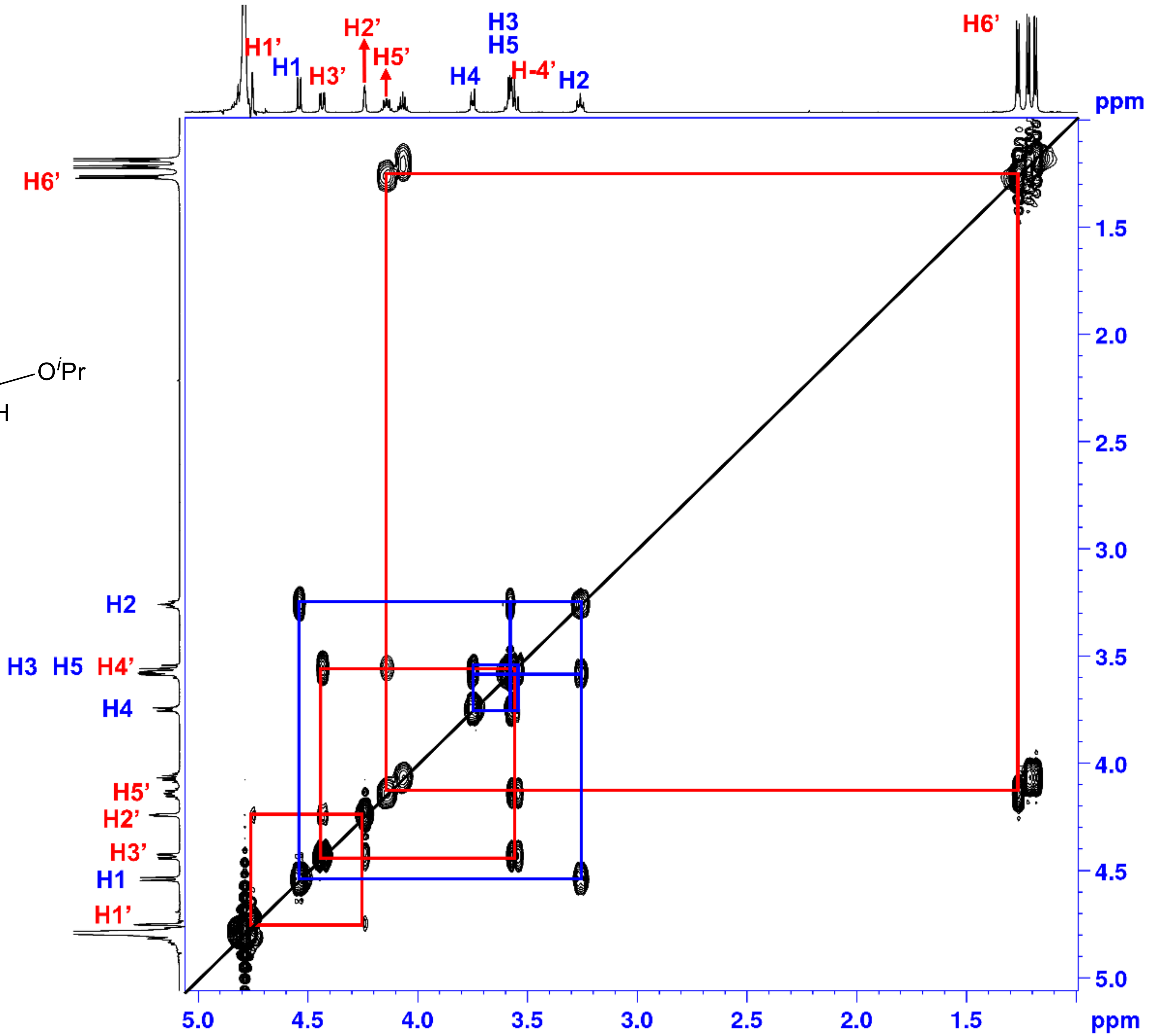

H1'
H1
H3'
H2'
H5'
H4
H3
H5
H-4'
H2
H6'
ppm
H2
H3 H5 H4'
H4
H5'
H2'
H3'
H1
H1'
1.5
2.0
2.5
3.0
3.5
4.0
4.5
5.0
5.0
4.5
4.0
3.5
3.0
2.5
2.0
1.5
ppm

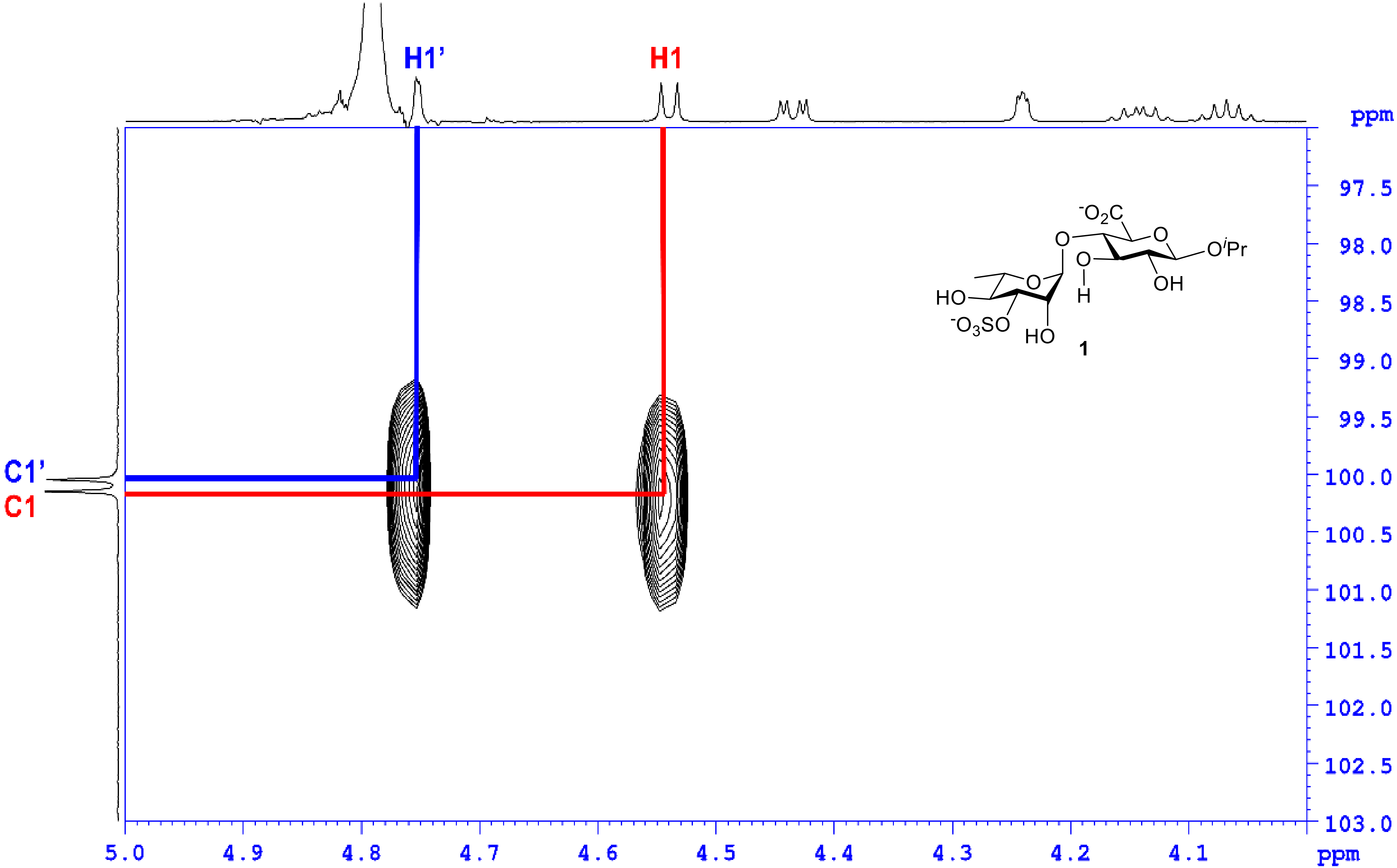
H1'
H1
ppm
97.5
98.0
98.5
99.0
99.5
100.0
100.5
101.0
101.5
102.0
102.5
103.0
C1'
C1
5.0
4.9
4.8
4.7
4.6
4.5
4.4
4.3
4.2
4.1
ppm
⁻O₂C
O
O
O
OⁱPr
H
OH
HO
⁻O₃SO
HO
1

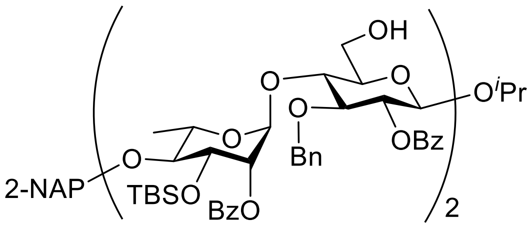
OH
O
O
O
O'Pr
OBz
Bn
2-NAP
TBSO
BzO
2

39

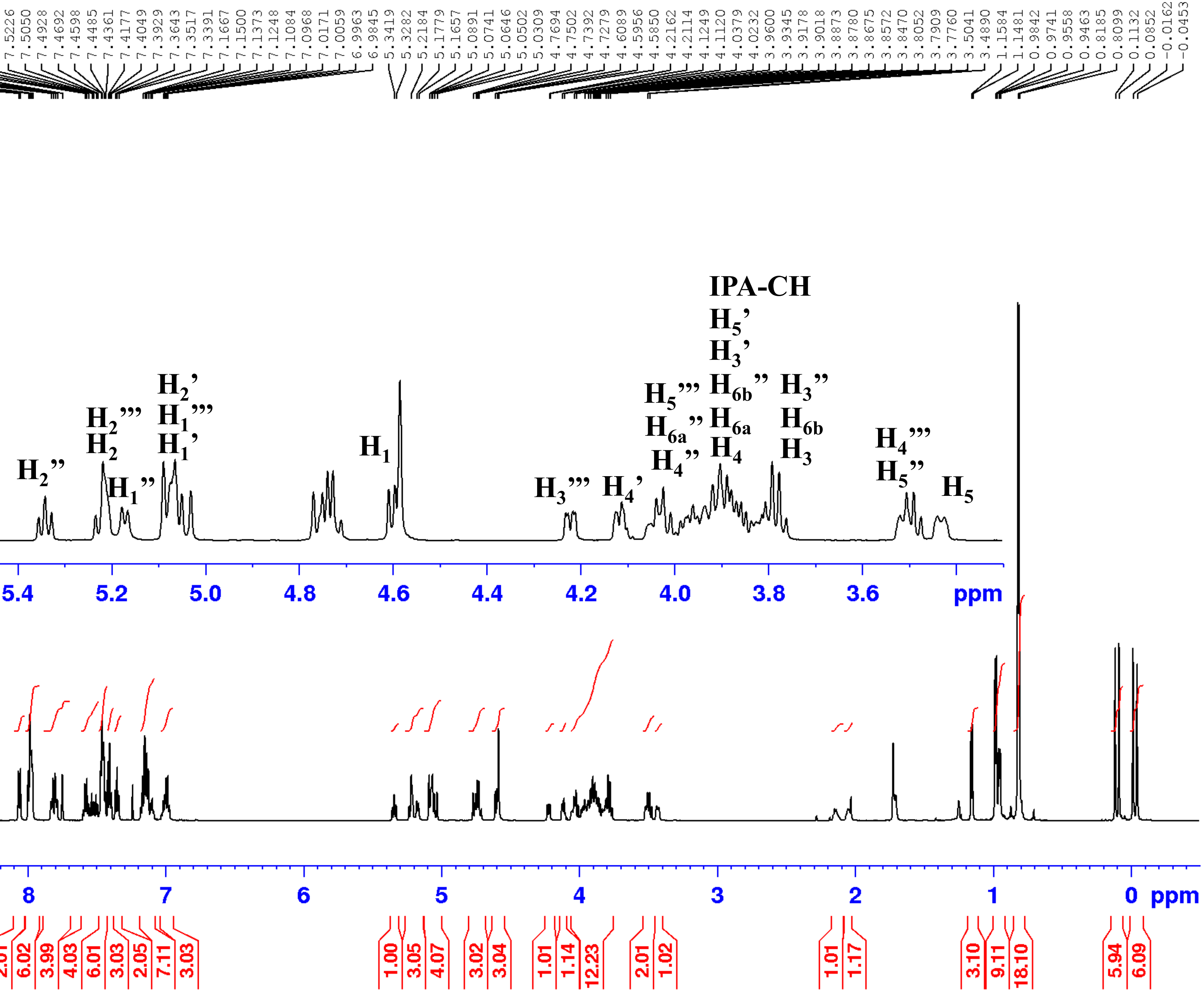
8.0663 8.0532 7.9963 7.9837 7.9721 7.9648 7.8318 7.8157 7.8008 7.7858 7.7486 7.5843 7.5718 7.5593 7.5355 7.5226 7.5050 7.4928 7.4692 7.4598 7.4485 7.4361 7.4177 7.4049 7.3929 7.3643 7.3517 7.3391 7.1667 7.1500 7.1373 7.1248 7.1084 7.0968 7.0171 7.0059 6.9963 6.9845 5.3419 5.3282 5.2184 5.1779 5.1657 5.0891 5.0741 5.0646 5.0502 5.0309 4.7694 4.7502 4.7392 4.7279 4.6089 4.5956 4.5850 4.2162 4.2114 4.1249 4.1120 4.0379 4.0232 3.9600 3.9345 3.9178 3.9018 3.8873 3.8780 3.8675 3.8572 3.8470 3.8052 3.7909 3.7760 3.5041 3.4890 1.1584 1.1481 0.9842 0.9741 0.9558 0.9463 0.8185 0.8099 0.1132 0.0852 -0.0162 -0.0453
H2''
H2''' H2
H1''
H2' H1''' H1'
H1
H3'''
H4'
H5''' H6a'' H4''
IPA-CH H5' H3' H6b'' H6a H4
H3'' H6b H3
H4''' H5''
H5
5.4 5.2 5.0 4.8 4.6 4.4 4.2 4.0 3.8 3.6 ppm
9 8 7 6 5 4 3 2 1 0 ppm
2.01 6.02 3.99 4.03 6.01 3.03 2.05 7.11 3.03 1.00 3.05 4.07 3.02 3.04 1.01 1.14 12.23 2.01 1.02 1.01 1.17 3.10 9.11 18.10 5.94 6.09

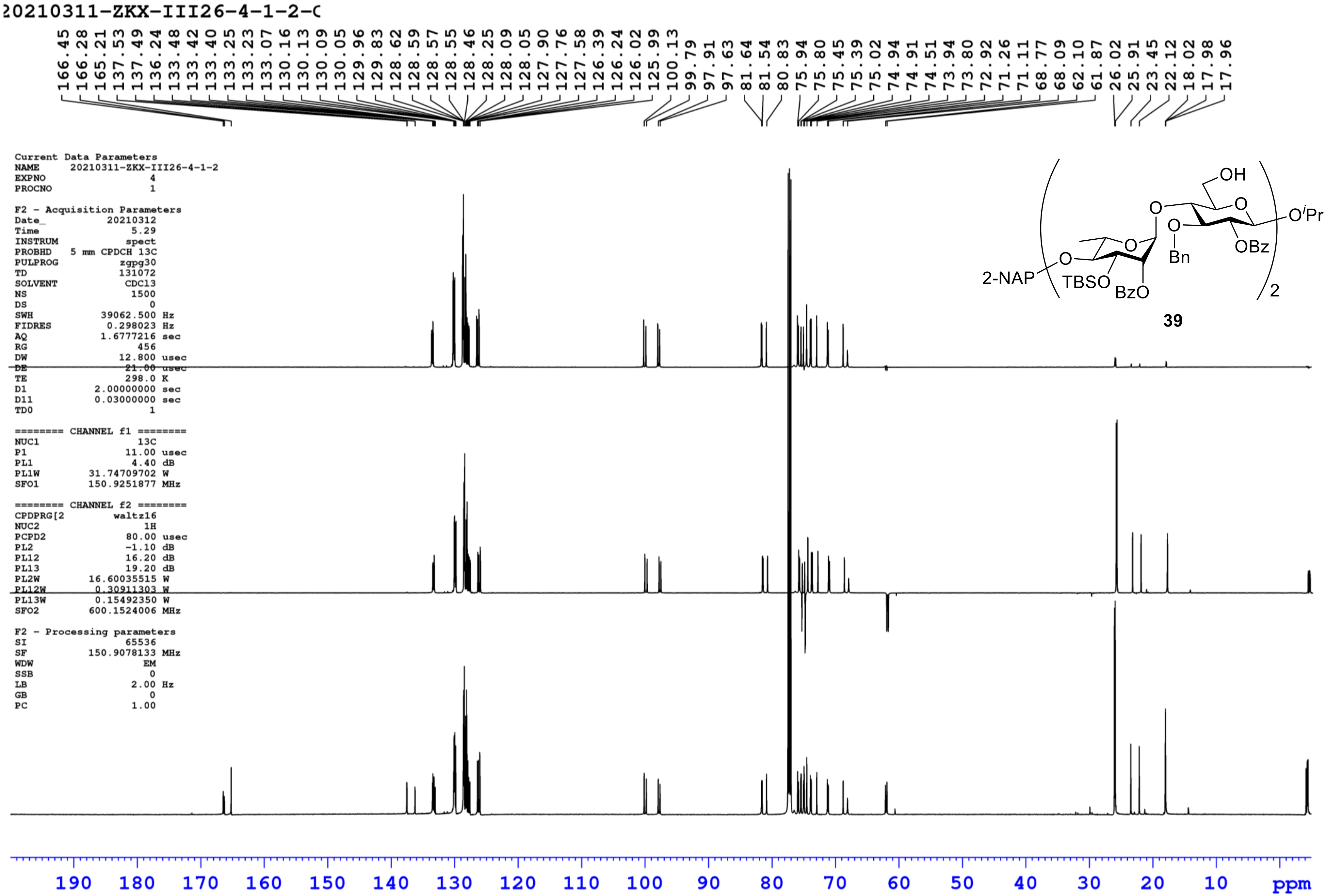
20210311-ZKX-III26-4-1-2-C
166.45 166.28 165.21 137.53 137.49 136.24 133.48 133.42 133.40 133.25 133.23 133.07 130.16 130.13 130.09 130.05 129.96 129.83 128.62 128.59 128.57 128.55 128.46 128.25 128.09 128.05 127.90 127.76 127.58 126.39 126.24 126.02 125.99 100.13 99.79 97.91 97.63 81.64 81.54 80.83 75.94 75.80 75.45 75.39 75.02 74.94 74.91 74.51 73.94 73.80 72.92 71.26 71.11 68.77 68.09 62.10 61.87 26.02 25.91 23.45 22.12 18.02 17.98 17.96
Current Data Parameters
NAME 20210311-ZKX-III26-4-1-2
EXPNO 4
PROCNO 1
F2 - Acquisition Parameters
Date_ 20210312
Time 5.29
INSTRUM spect
PROBHD 5 mm CPDCH 13C
PULPROG zgpg30
TD 131072
SOLVENT CDCl3
NS 1500
DS 0
SWH 39062.500 Hz
FIDRES 0.298023 Hz
AQ 1.6777216 sec
RG 456
DW 12.800 usec
DE 21.00 usec
TE 298.0 K
D1 2.00000000 sec
D11 0.03000000 sec
TD0 1
======== CHANNEL f1 ========
NUC1 13C
P1 11.00 usec
PL1 4.40 dB
PL1W 31.74709702 W
SFO1 150.9251877 MHz
======== CHANNEL f2 ========
CPDPRG[2 waltz16
NUC2 1H
PCPD2 80.00 usec
PL2 -1.10 dB
PL12 16.20 dB
PL13 19.20 dB
PL2W 16.60035515 W
PL12W 0.30911303 W
PL13W 0.15492350 W
SFO2 600.1524006 MHz
F2 - Processing parameters
SI 65536
SF 150.9078133 MHz
WDW EM
SSB 0
LB 2.00 Hz
GB 0
PC 1.00
OH
O
O
O
OⁱPr
O
O
Bn
OBz
2-NAP
TBSO
BzO
2
39
190 180 170 160 150 140 130 120 110 100 90 80 70 60 50 40 30 20 10 ppm

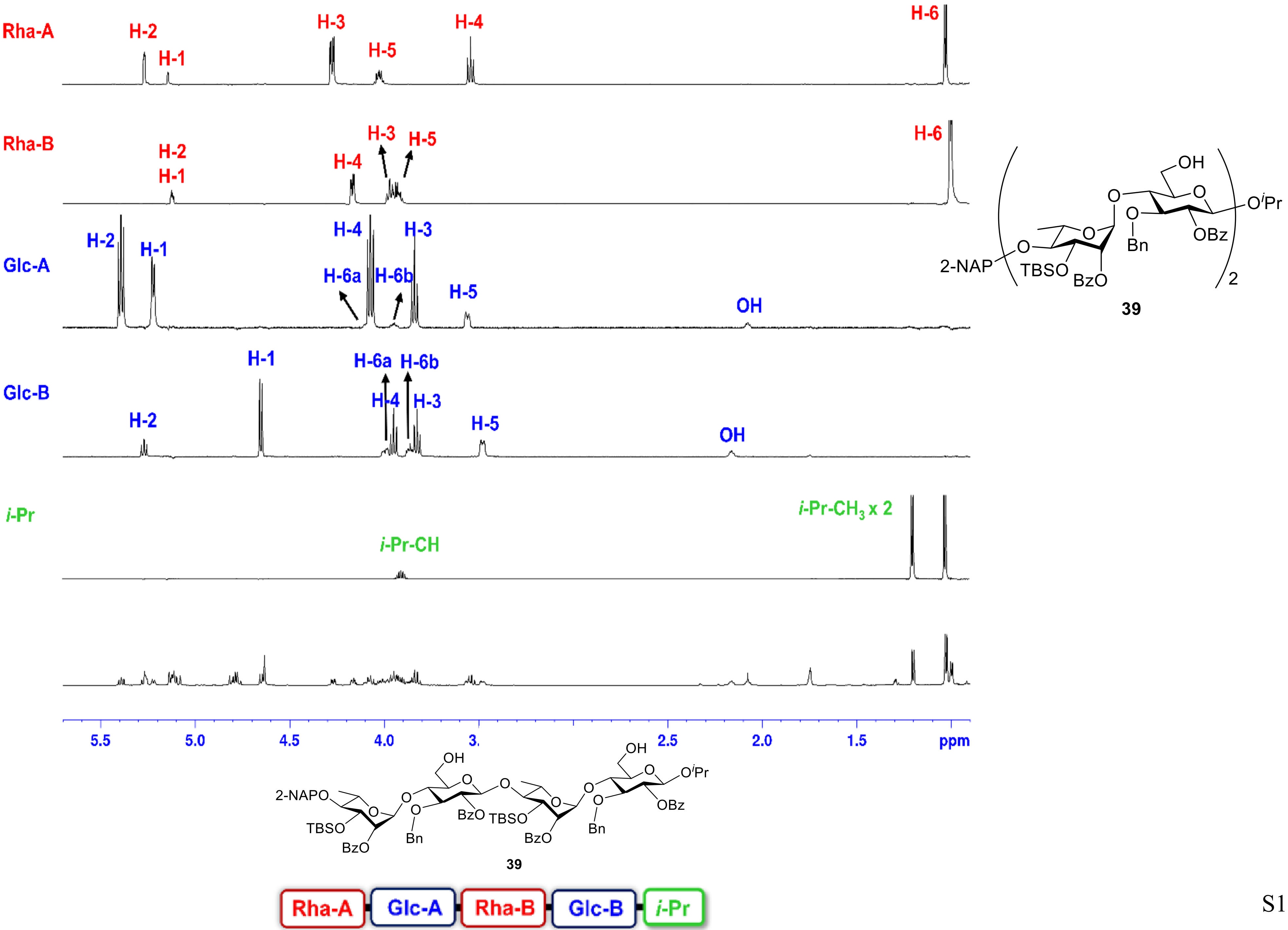
Rha-A
H-2
H-1
H-3
H-5
H-4
H-6
Rha-B
H-2
H-1
H-4
H-3
H-5
H-6
Glc-A
H-2
H-1
H-4
H-6a
H-6b
H-3
H-5
OH
Glc-B
H-1
H-2
H-6a
H-6b
H-4
H-3
H-5
OH
i-Pr
i-Pr-CH
i-Pr-CH3 x 2
5.5
5.0
4.5
4.0
3.
2.5
2.0
1.5
ppm
OH
O
O
OiPr
O
Bn
OBz
2-NAP
O
TBSO
BzO
2
39
2-NAPO
TBSO
BzO
Bn
BzO
TBSO
BzO
Bn
OBz
OiPr
39
Rha-A
Glc-A
Rha-B
Glc-B
i-Pr

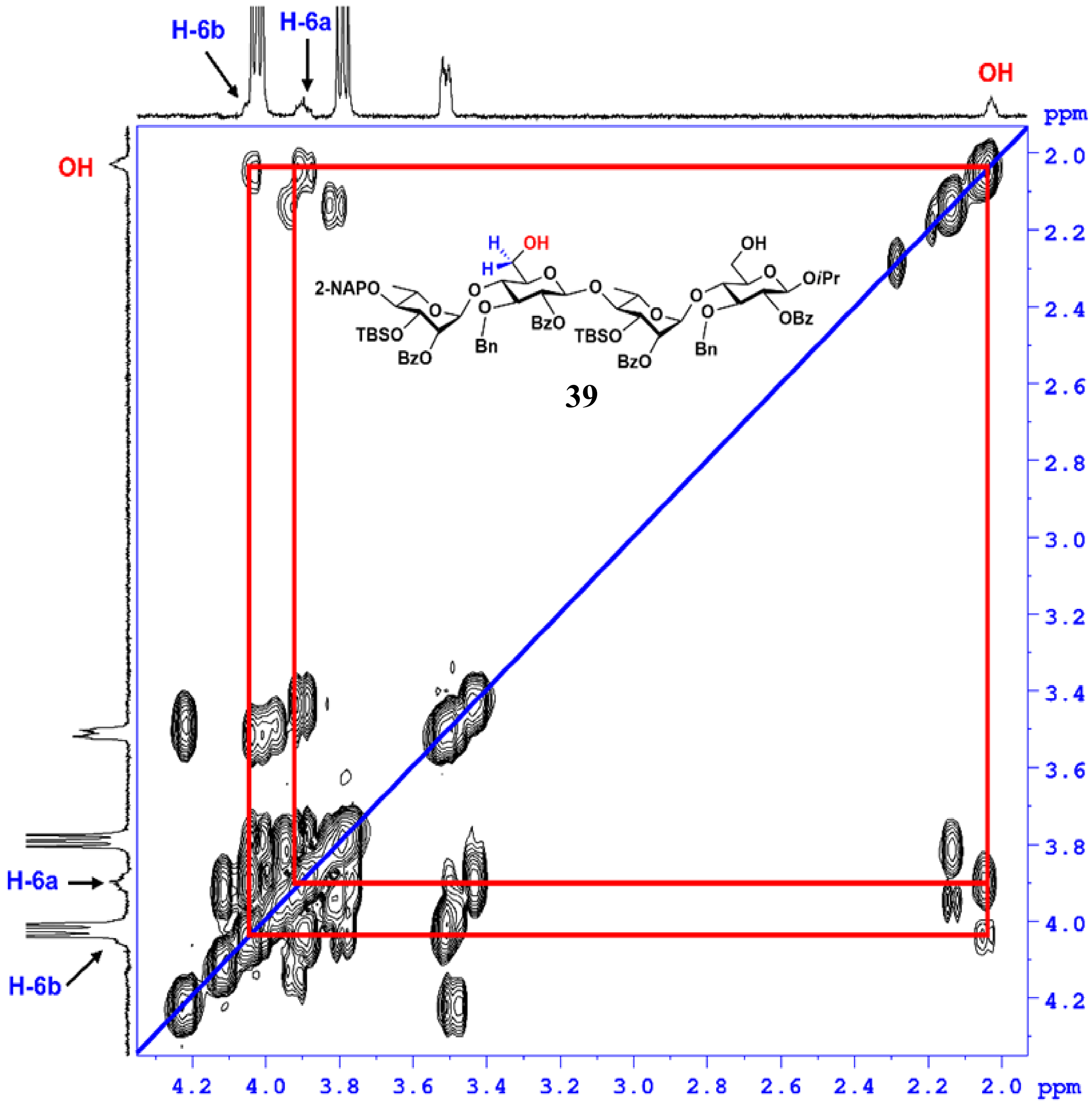
H-6b
H-6a
OH
ppm
2-NAPO
TBSO
BzO
Bn
BzO
TBSO
BzO
Bn
OBz
OiPr
4.2 4.0 3.8 3.6 3.4 3.2 3.0 2.8 2.6 2.4 2.2 2.0 ppm

39

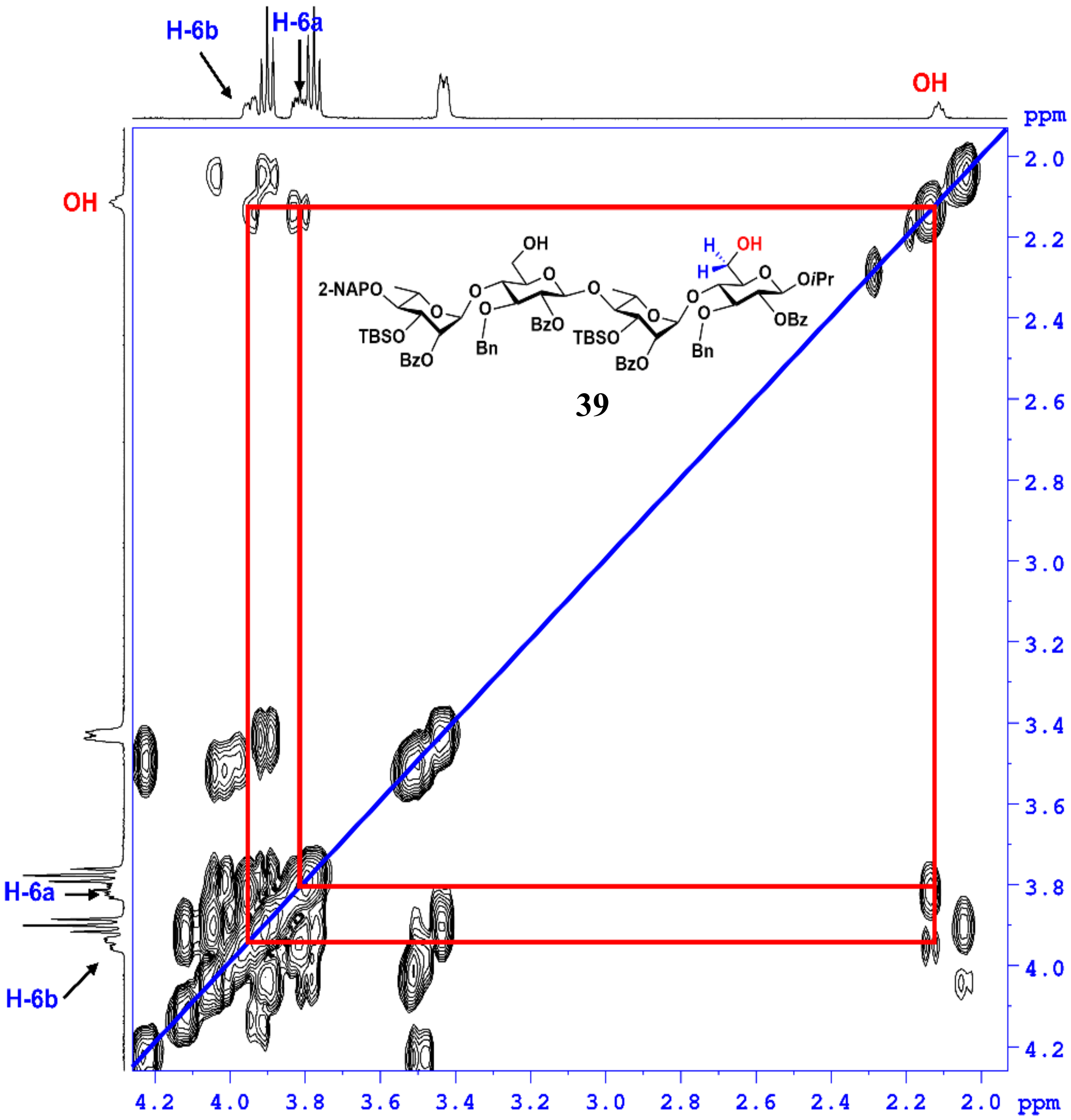
H-6b
H-6a
OH
ppm
2-NAPO
TBSO
BzO
Bn
BzO
TBSO
BzO
Bn
OBz
OiPr
4.2 4.0 3.8 3.6 3.4 3.2 3.0 2.8 2.6 2.4 2.2 2.0 ppm

39

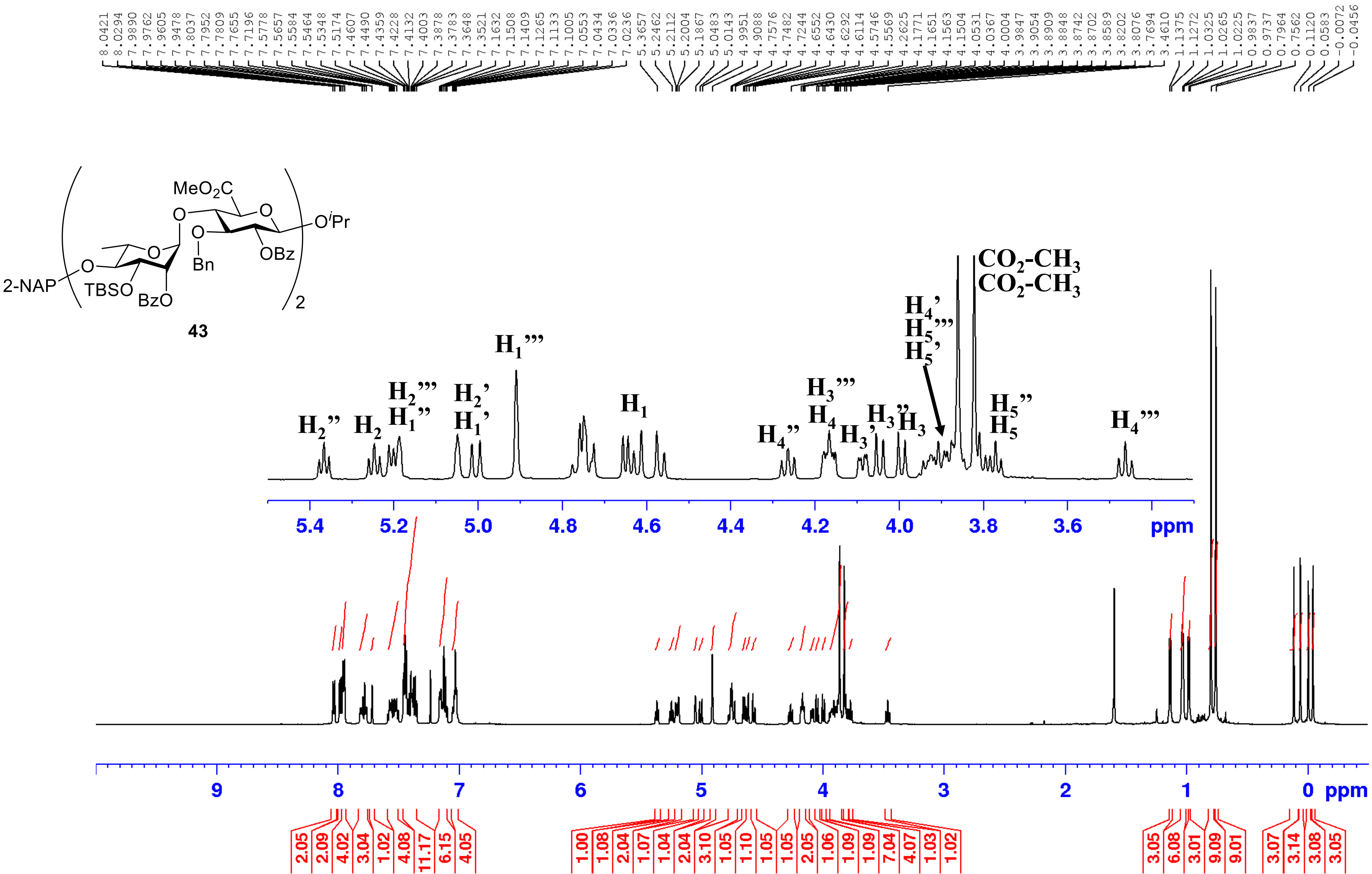
MeO2C
O
O
OiPr
O
Bn
OBz
O
O
2-NAP
TBSO
BzO
2
43
8.0421 8.0294 7.9890 7.9762 7.9605 7.9478 7.8037 7.7952 7.7809 7.7655 7.7196 7.5778 7.5657 7.5584 7.5464 7.5348 7.5174 7.4607 7.4490 7.4359 7.4228 7.4132 7.4003 7.3878 7.3783 7.3648 7.3521 7.1632 7.1508 7.1409 7.1265 7.1133 7.1005 7.0553 7.0434 7.0336 7.0236 5.3657 5.2462 5.2112 5.2004 5.1867 5.0483 5.0143 4.9951 4.9088 4.7576 4.7482 4.7244 4.6552 4.6430 4.6292 4.6114 4.5746 4.5569 4.2625 4.1771 4.1651 4.1563 4.1504 4.0531 4.0367 4.0004 3.9847 3.9054 3.8909 3.8848 3.8742 3.8702 3.8589 3.8202 3.8076 3.7694 3.4610 1.1375 1.1272 1.0325 1.0265 1.0225 0.9837 0.9737 0.7964 0.7562 0.1120 0.0583 -0.0072 -0.0456
H2''
H2
H2''' H1''
H2' H1'
H1'''
H1
H4''
H3''' H4
H3'
H3''
H3
H4' H5''' H5'
CO2-CH3
CO2-CH3
H5'' H5
H4'''
5.4 5.2 5.0 4.8 4.6 4.4 4.2 4.0 3.8 3.6 ppm
9 8 7 6 5 4 3 2 1 0 ppm
2.05 2.09 4.02 3.04 1.02 4.08 11.17 6.15 4.05
1.00 1.08 2.04 1.07 1.04 2.04 3.10 1.05 1.10 1.05 1.05 2.05 1.06 1.09 1.09 7.04 4.07 1.03 1.02
3.05 6.08 3.01 9.09 9.01
3.07 3.14 3.08 3.05

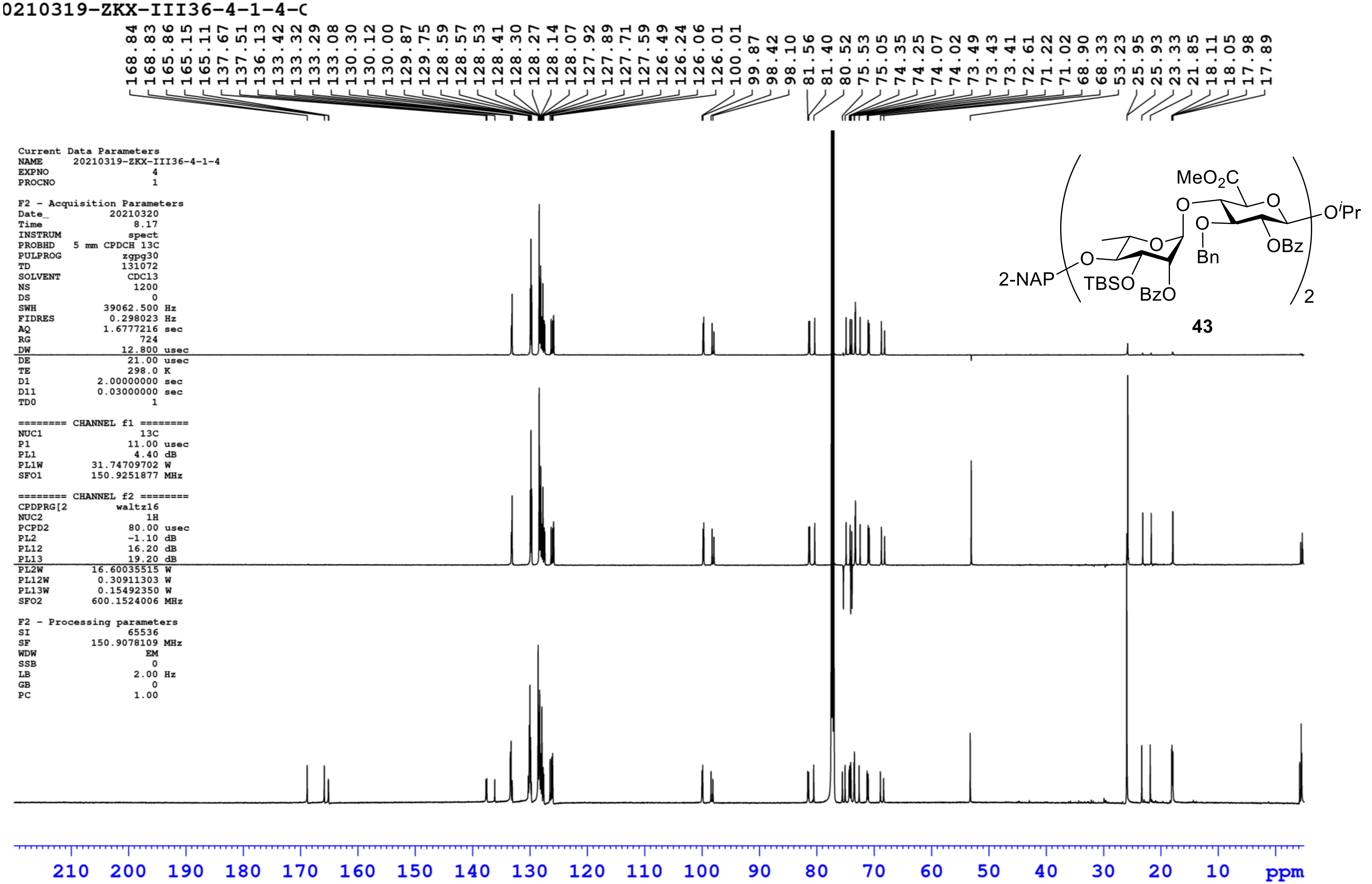
0210319-ZKX-III36-4-1-4-C
168.84
168.83
165.86
165.15
165.11
137.67
137.51
136.13
133.42
133.32
133.29
133.08
130.30
130.12
130.00
129.87
129.75
128.59
128.57
128.53
128.41
128.30
128.27
128.14
128.07
127.92
127.89
127.71
127.59
126.49
126.24
126.06
126.01
100.01
99.87
98.42
98.10
81.56
81.40
80.52
75.53
75.05
74.35
74.25
74.07
74.02
73.49
73.43
73.41
72.61
71.22
71.02
68.90
68.33
53.23
25.95
25.93
23.33
21.85
18.11
18.05
17.98
17.89
Current Data Parameters
NAME 20210319-ZKX-III36-4-1-4
EXPNO 4
PROCNO 1
F2 - Acquisition Parameters
Date_ 20210320
Time 8.17
INSTRUM spect
PROBHD 5 mm CPDCH 13C
PULPROG zgpg30
TD 131072
SOLVENT CDCl3
NS 1200
DS 0
SWH 39062.500 Hz
FIDRES 0.298023 Hz
AQ 1.6777216 sec
RG 724
DW 12.800 usec
DE 21.00 usec
TE 298.0 K
D1 2.00000000 sec
D11 0.03000000 sec
TD0 1
======== CHANNEL f1 ========
NUC1 13C
P1 11.00 usec
PL1 4.40 dB
PL1W 31.74709702 W
SFO1 150.9251877 MHz
======== CHANNEL f2 ========
CPDPRG[2 waltz16
NUC2 1H
PCPD2 80.00 usec
PL2 -1.10 dB
PL12 16.20 dB
PL13 19.20 dB
PL2W 16.60035515 W
PL12W 0.30911303 W
PL13W 0.15492350 W
SFO2 600.1524006 MHz
F2 - Processing parameters
SI 65536
SF 150.9078109 MHz
WDW EM
SSB 0
LB 2.00 Hz
GB 0
PC 1.00
MeO2C
O
O
OiPr
O
Bn
OBz
2-NAP
O
O
TBSO
BzO
2
43
210 200 190 180 170 160 150 140 130 120 110 100 90 80 70 60 50 40 30 20 10 ppm

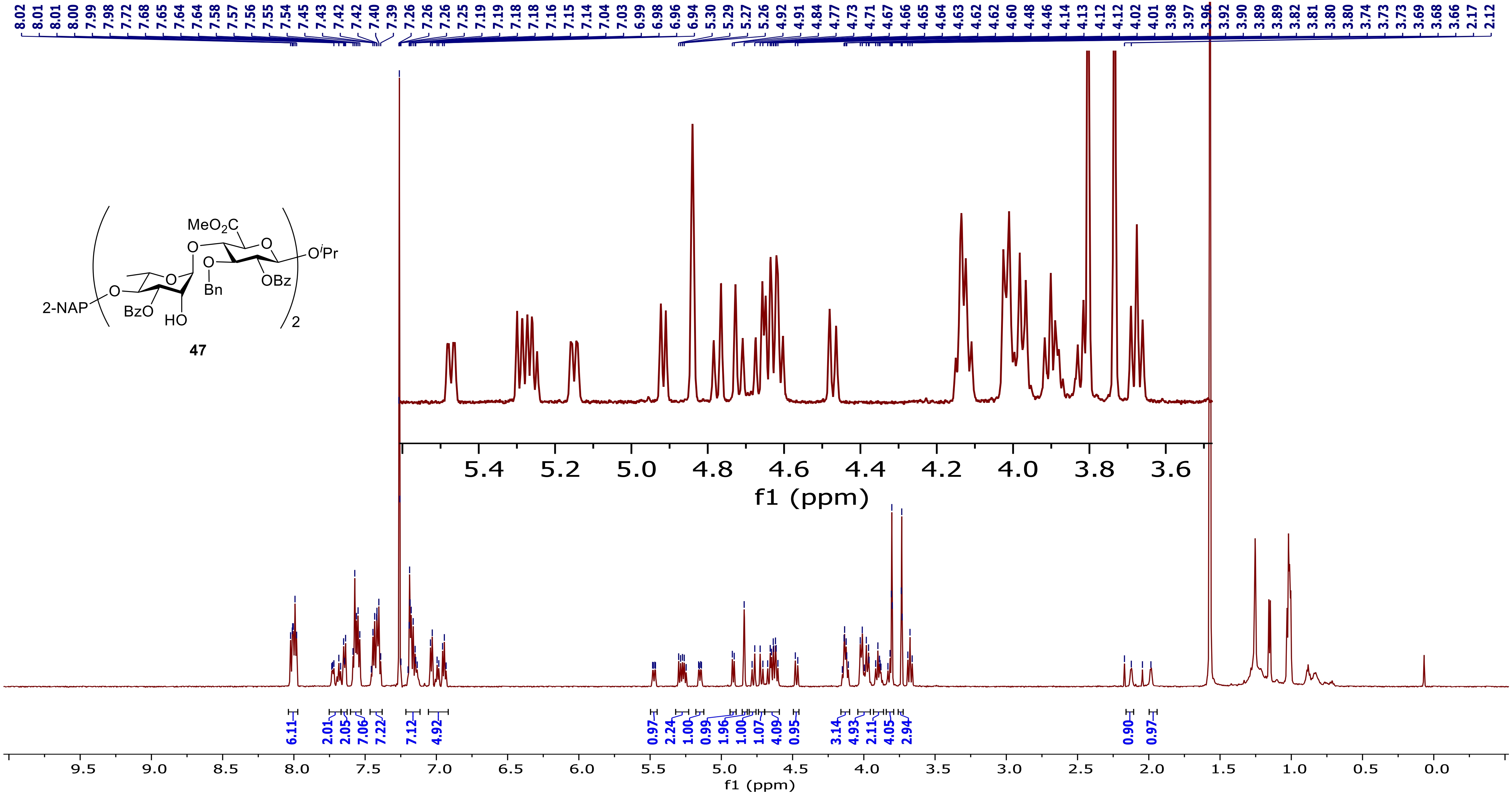

8.02 8.01 8.01 8.00 7.99 7.98 7.72 7.68 7.65 7.64 7.64 7.58 7.57 7.56 7.55 7.54 7.45 7.43 7.42 7.42 7.40 7.39 7.26 7.26 7.26 7.25 7.19 7.19 7.18 7.18 7.16 7.15 7.14 7.04 7.03 6.99 6.98 6.96 6.94 5.30 5.29 5.27 5.26 4.92 4.91 4.84 4.77 4.73 4.71 4.67 4.66 4.65 4.64 4.63 4.62 4.62 4.60 4.48 4.46 4.14 4.13 4.12 4.12 4.02 4.01 3.98 3.97 3.96 3.92 3.90 3.89 3.89 3.82 3.81 3.80 3.80 3.74 3.73 3.73 3.69 3.68 3.66 2.17 2.12
MeO2C
O
OiPr
OBz
O
Bn
O
2-NAP
O
BzO
HO
2
47
5.4 5.2 5.0 4.8 4.6 4.4 4.2 4.0 3.8 3.6
f1 (ppm)
6.11 2.01 2.05 7.06 7.22 7.12 4.92 0.97 2.24 1.00 0.99 1.96 1.00 1.07 4.09 0.95 3.14 4.93 2.11 4.05 2.94 0.90 0.97
9.5 9.0 8.5 8.0 7.5 7.0 6.5 6.0 5.5 5.0 4.5 4.0 3.5 3.0 2.5 2.0 1.5 1.0 0.5 0.0
f1 (ppm)

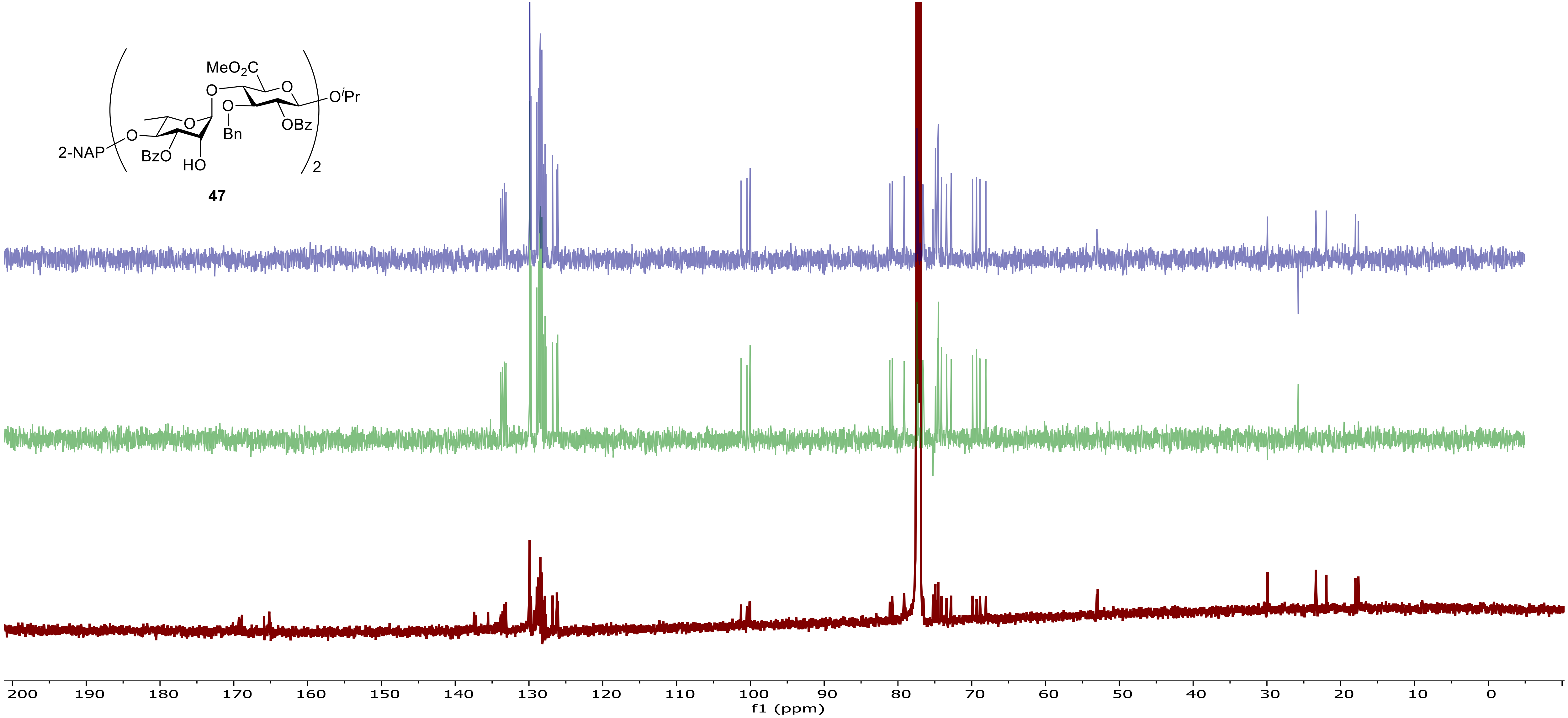
MeO2C
O
O
O
O
OiPr
2-NAP
O
BzO
HO
Bn
OBz
2
47
200 190 180 170 160 150 140 130 120 110 100 90 80 70 60 50 40 30 20 10 0
f1 (ppm)

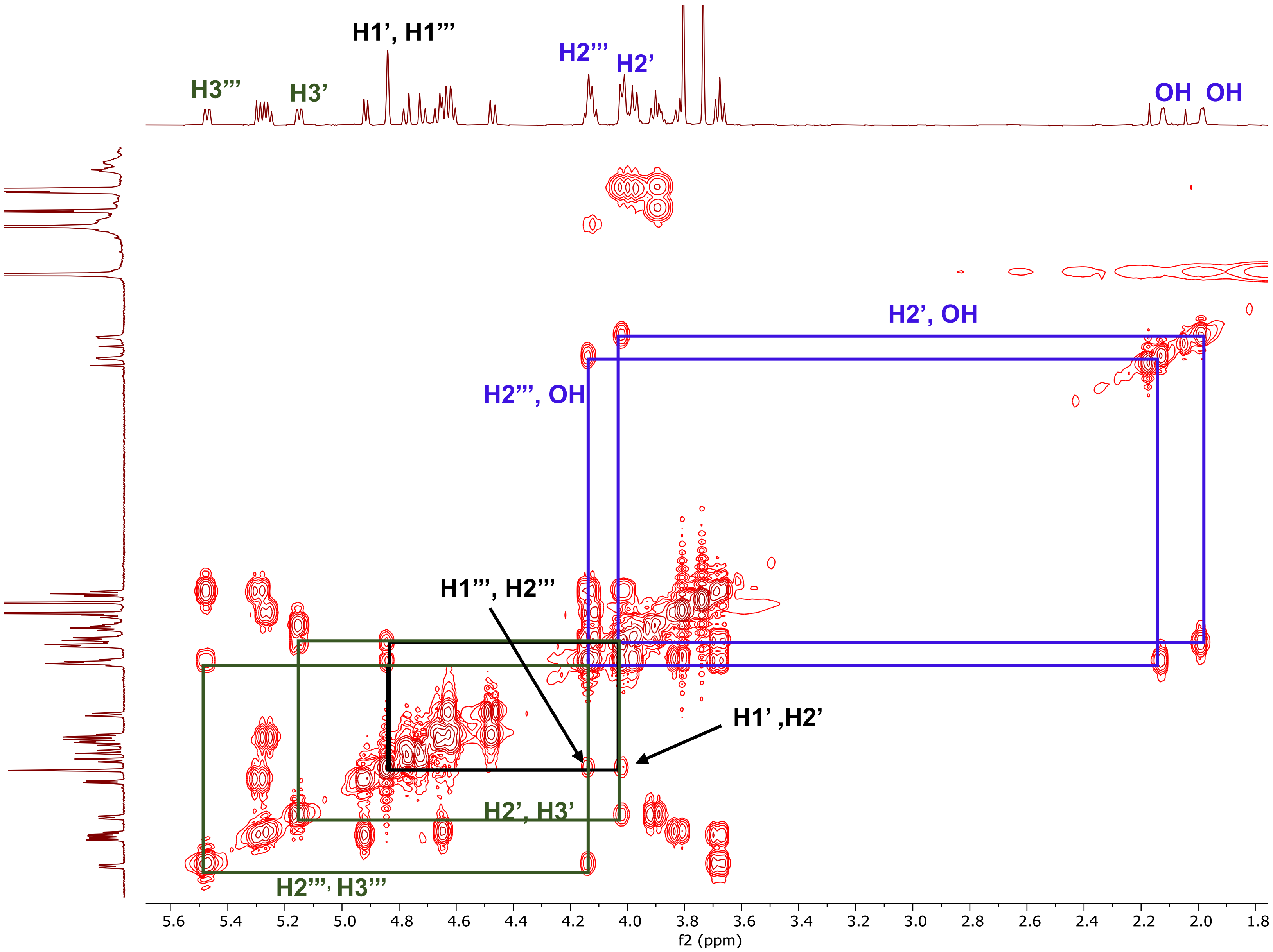

H1', H1'''
H2'''
H2'
H3'''
H3'
OH OH
H2', OH
H2''', OH
H1''', H2'''
H1' ,H2'
H2', H3'
H2''', H3'''
f2 (ppm)
f1 (ppm)

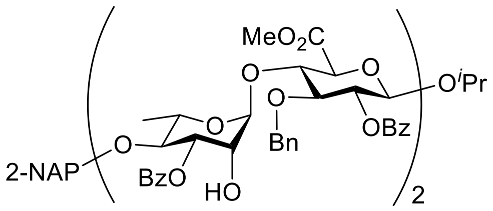

MeO2C
O
O
O
O
OiPr
Bn
OBz
2-NAP
O
BzO
HO
2

47

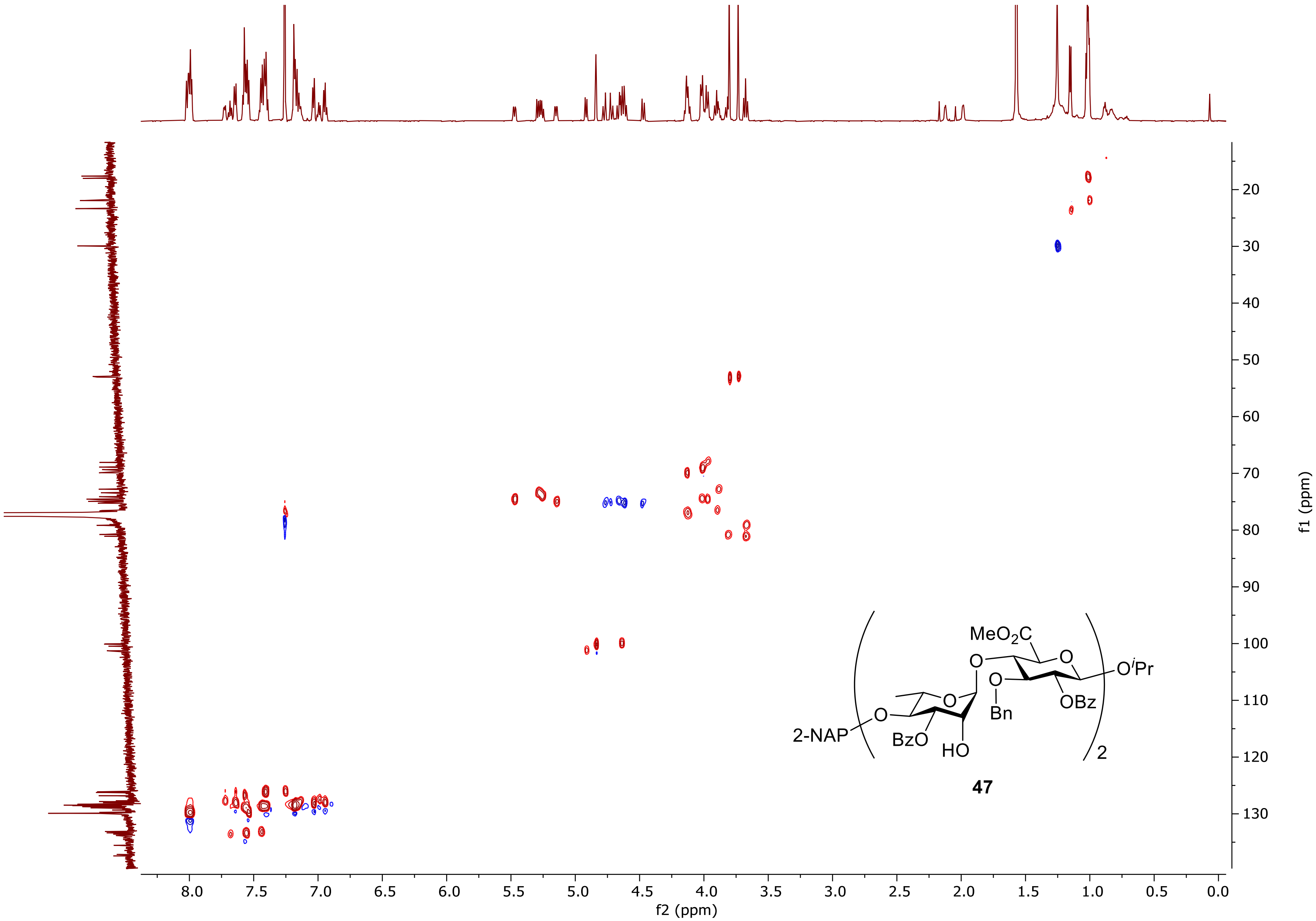

f1 (ppm)
f2 (ppm)
MeO2C
O
O
O
O
O'Pr
O
Bn
OBz
2-NAP
BzO
HO
2
47

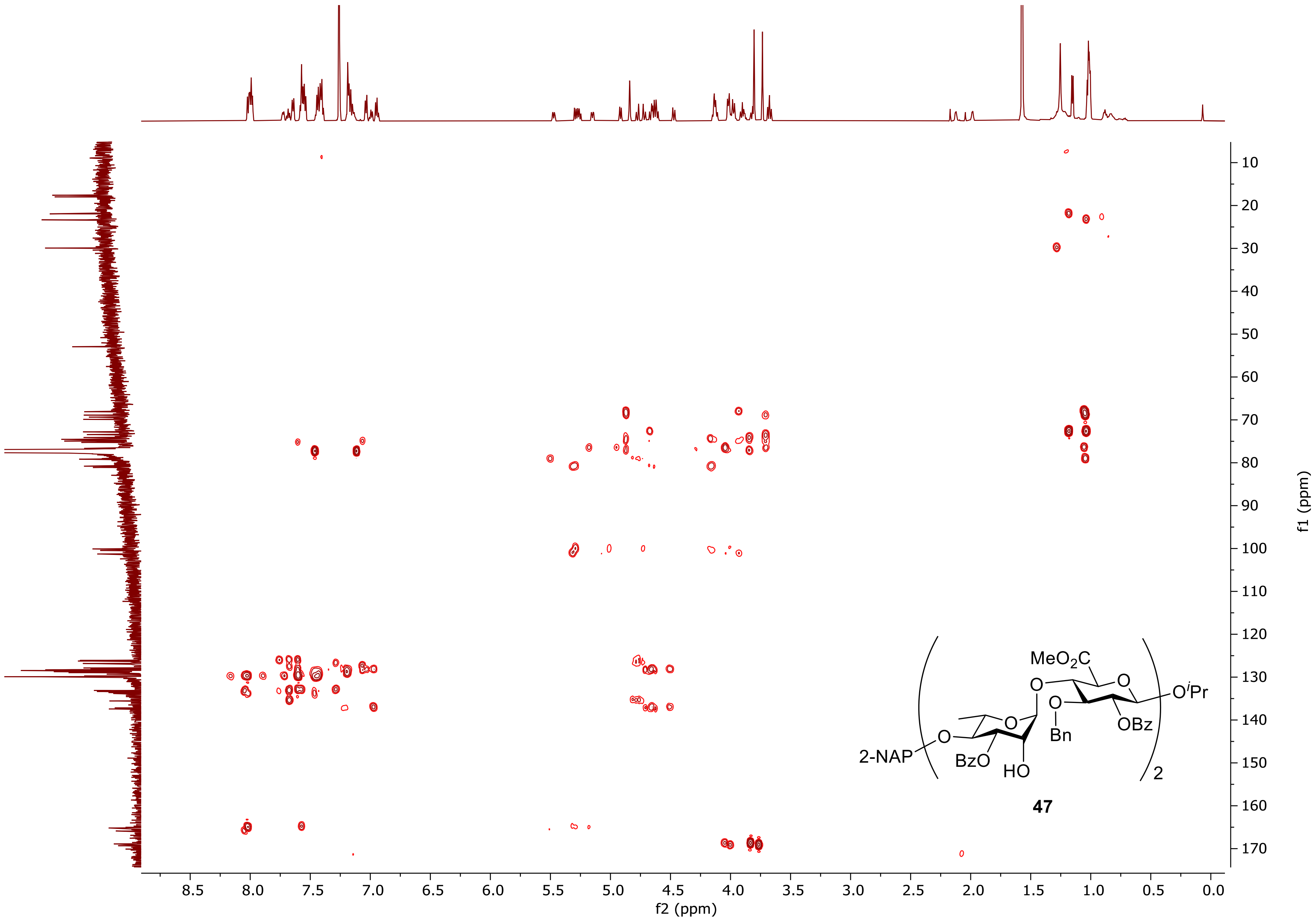

f1 (ppm)
f2 (ppm)
MeO2C
O
O
O
OiPr
O
Bn
OBz
2-NAP
O
BzO
HO
2
47

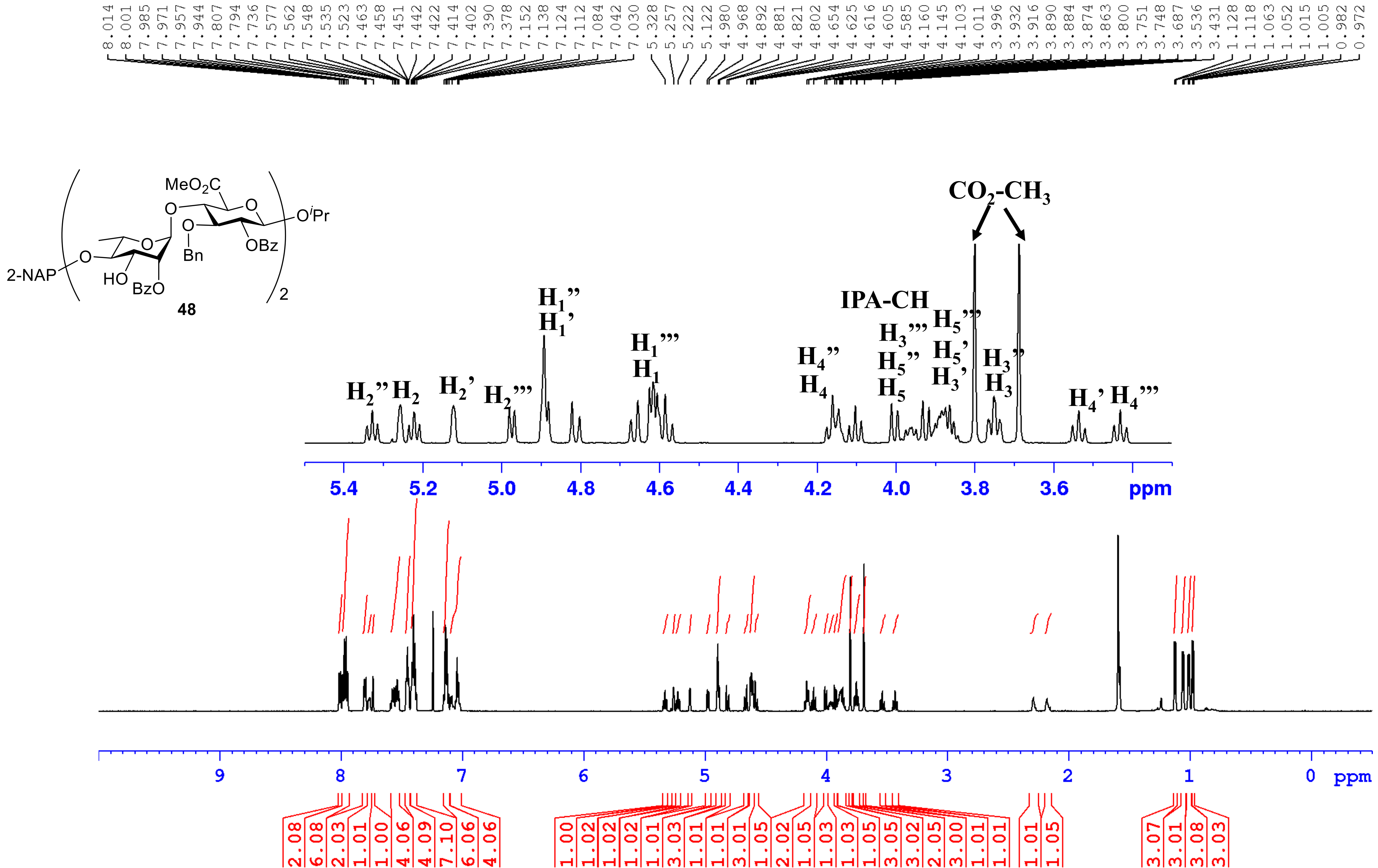
8.014 8.001 7.985 7.971 7.957 7.944 7.807 7.794 7.736 7.577 7.562 7.548 7.535 7.523 7.463 7.458 7.451 7.442 7.422 7.414 7.402 7.390 7.378 7.152 7.138 7.124 7.112 7.084 7.042 7.030 5.328 5.257 5.222 5.122 4.980 4.968 4.892 4.881 4.821 4.802 4.654 4.625 4.616 4.605 4.585 4.160 4.145 4.103 4.011 3.996 3.932 3.916 3.890 3.884 3.874 3.863 3.800 3.751 3.748 3.687 3.536 3.431 1.128 1.118 1.063 1.052 1.015 1.005 0.982 0.972
MeO2C
O
O
OiPr
O
Bn
OBz
2-NAP
O
HO
BzO
2
48
CO2-CH3
IPA-CH
H1''
H1'
H1'''
H1
H2'' H2 H2' H2'''
H4''
H4
H3'''
H5''
H5
H5'''
H5'
H3'
H3''
H3
H4' H4'''
5.4 5.2 5.0 4.8 4.6 4.4 4.2 4.0 3.8 3.6 ppm
9 8 7 6 5 4 3 2 1 0 ppm
2.08 6.08 2.03 1.01 1.00 4.06 4.09 7.10 6.06 4.06 1.00 1.02 1.02 1.02 1.01 3.03 1.01 1.01 3.01 1.05 2.02 1.05 1.03 1.03 1.05 3.05 3.02 2.05 3.00 1.01 1.01 1.01 1.05 3.07 3.01 3.08 3.03

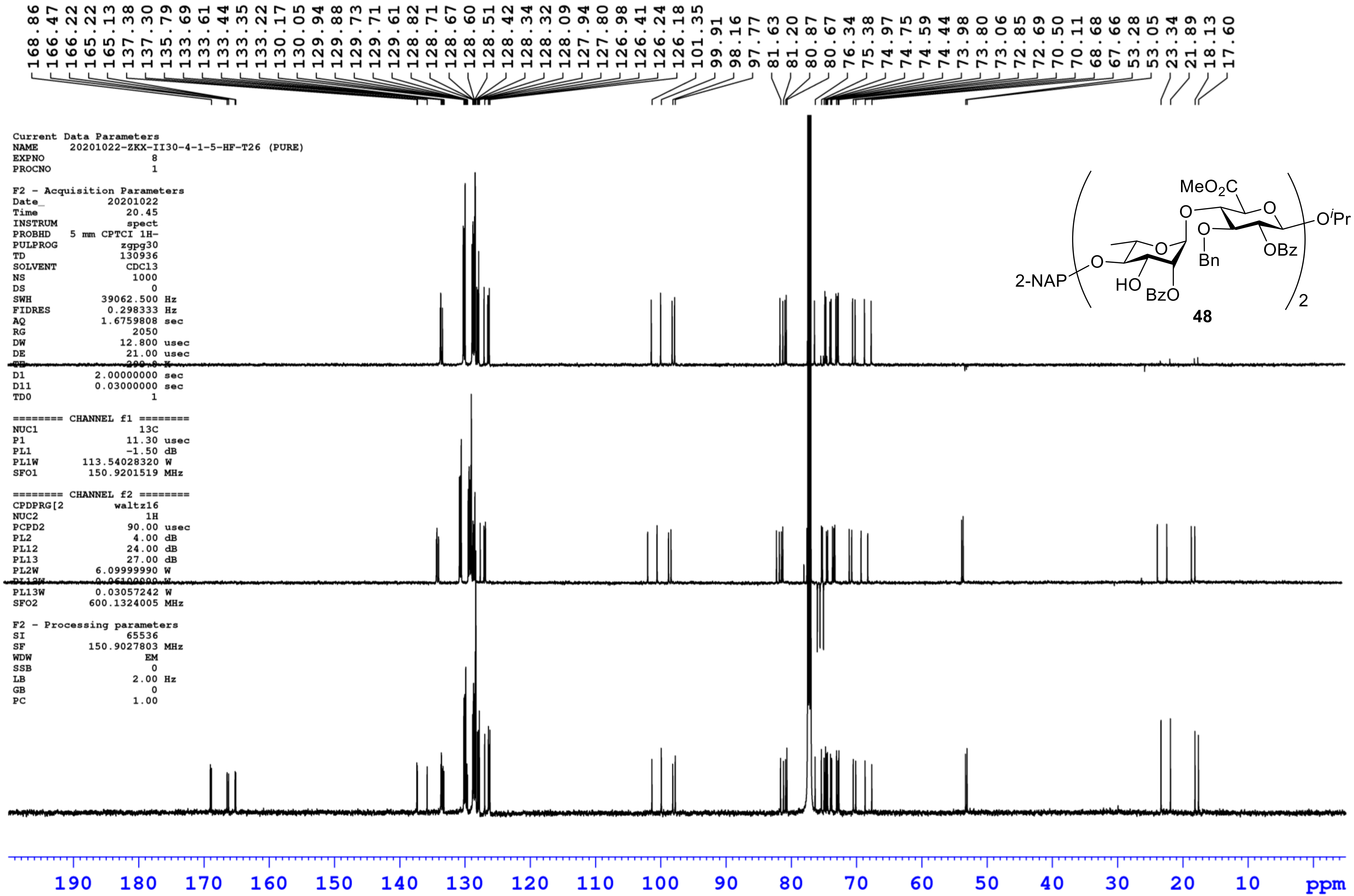

168.86
166.47
166.22
165.22
165.13
137.38
137.30
135.79
133.69
133.61
133.44
133.35
133.22
130.17
130.05
129.94
129.88
129.73
129.71
129.61
128.82
128.71
128.67
128.60
128.51
128.42
128.34
128.32
128.09
127.94
127.80
126.98
126.41
126.24
126.18
101.35
99.91
98.16
97.77
81.63
81.20
80.87
80.67
76.34
75.38
74.97
74.75
74.59
74.44
73.98
73.80
73.06
72.85
72.69
70.50
70.11
68.68
67.66
53.28
53.05
23.34
21.89
18.13
17.60
Current Data Parameters
NAME 20201022-ZKX-II30-4-1-5-HF-T26 (PURE)
EXPNO 8
PROCNO 1
F2 - Acquisition Parameters
Date_ 20201022
Time 20.45
INSTRUM spect
PROBHD 5 mm CPTCI 1H-
PULPROG zgpg30
TD 130936
SOLVENT CDCl3
NS 1000
DS 0
SWH 39062.500 Hz
FIDRES 0.298333 Hz
AQ 1.6759808 sec
RG 2050
DW 12.800 usec
DE 21.00 usec
TE [illegible]
D1 2.00000000 sec
D11 0.03000000 sec
TD0 1
======== CHANNEL f1 ========
NUC1 13C
P1 11.30 usec
PL1 -1.50 dB
PL1W 113.54028320 W
SFO1 150.9201519 MHz
======== CHANNEL f2 ========
CPDPRG[2 waltz16
NUC2 1H
PCPD2 90.00 usec
PL2 4.00 dB
PL12 24.00 dB
PL13 27.00 dB
PL2W 6.09999990 W
PL12W [illegible]
PL13W 0.03057242 W
SFO2 600.1324005 MHz
F2 - Processing parameters
SI 65536
SF 150.9027803 MHz
WDW EM
SSB 0
LB 2.00 Hz
GB 0
PC 1.00
MeO2C
O
O
O
OiPr
O
OBz
Bn
2-NAP
O
HO
BzO
2
48
190 180 170 160 150 140 130 120 110 100 90 80 70 60 50 40 30 20 10 ppm

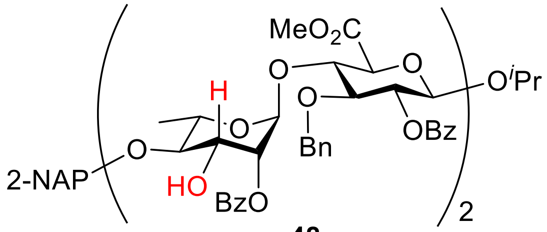
MeO2C
O
O
OiPr
H
O
O
Bn
OBz
2-NAP
O
HO
BzO
2

48

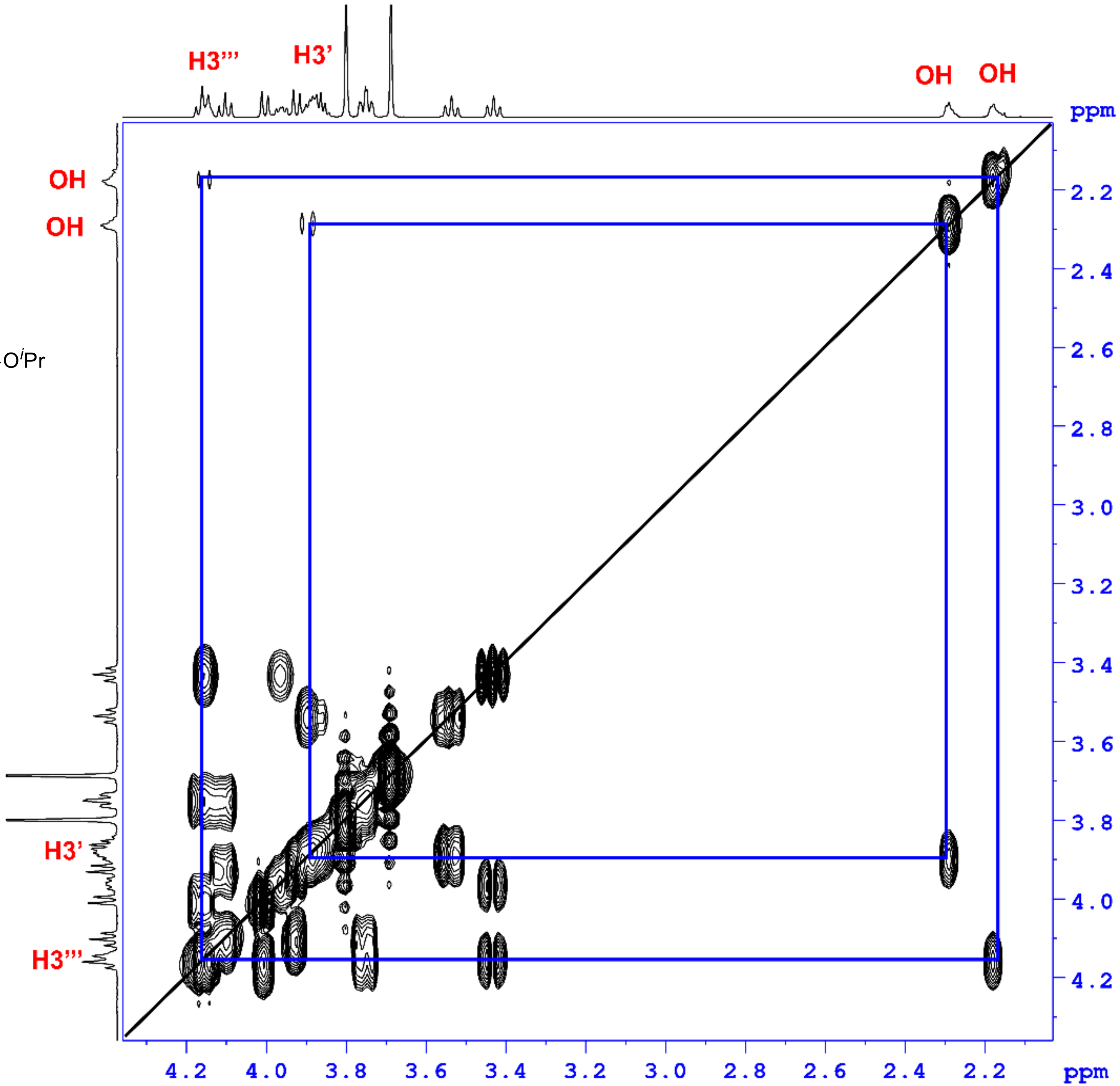
H3'''
H3'
OH
OH
ppm
OH
OH
H3'
H3'''
2.2
2.4
2.6
2.8
3.0
3.2
3.4
3.6
3.8
4.0
4.2
4.2
4.0
3.8
3.6
3.4
3.2
3.0
2.8
2.6
2.4
2.2
ppm

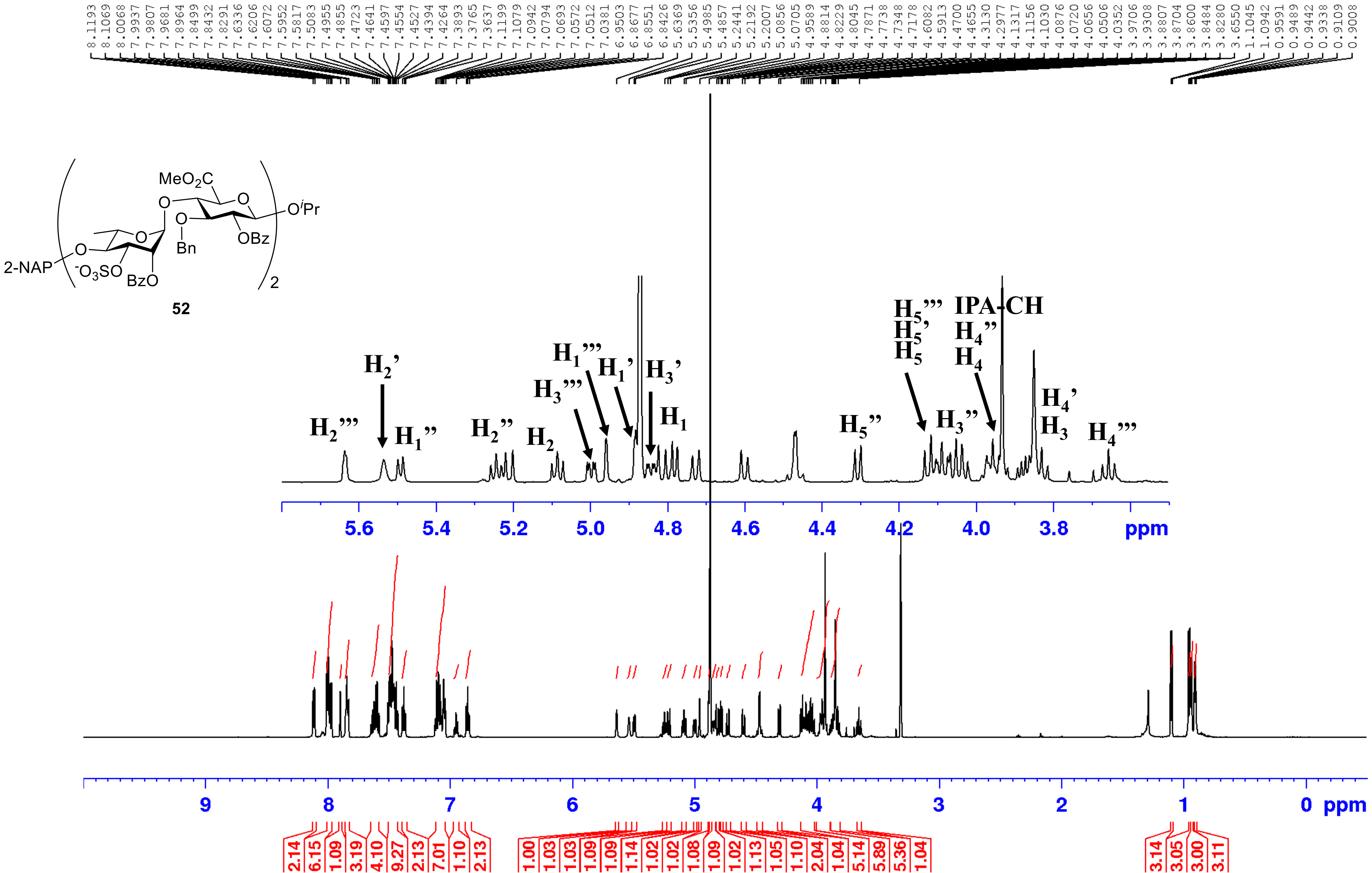

2-NAP
MeO2C
OiPr
OBz
Bn
-O3SO
BzO
52
H2'''
H2'
H1''
H2''
H2
H3'''
H1'''
H1'
H3'
H1
H5''
H5'''
H5'
H5
H3''
IPA-CH
H4''
H4
H4'
H3
H4'''
ppm

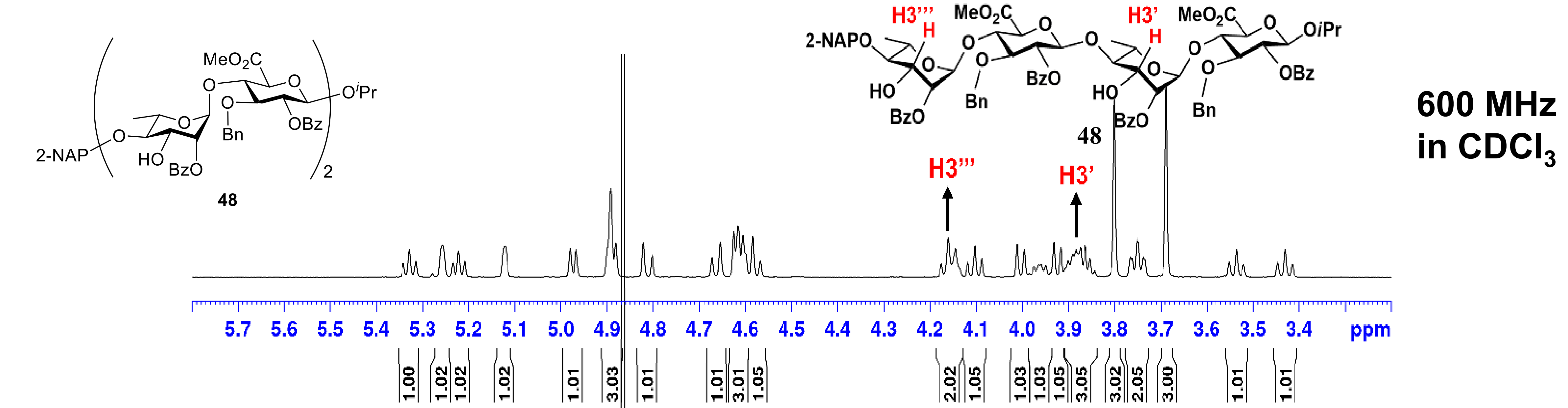
48
H3'''
H3'
600 MHz in $CDCl_3$
ppm

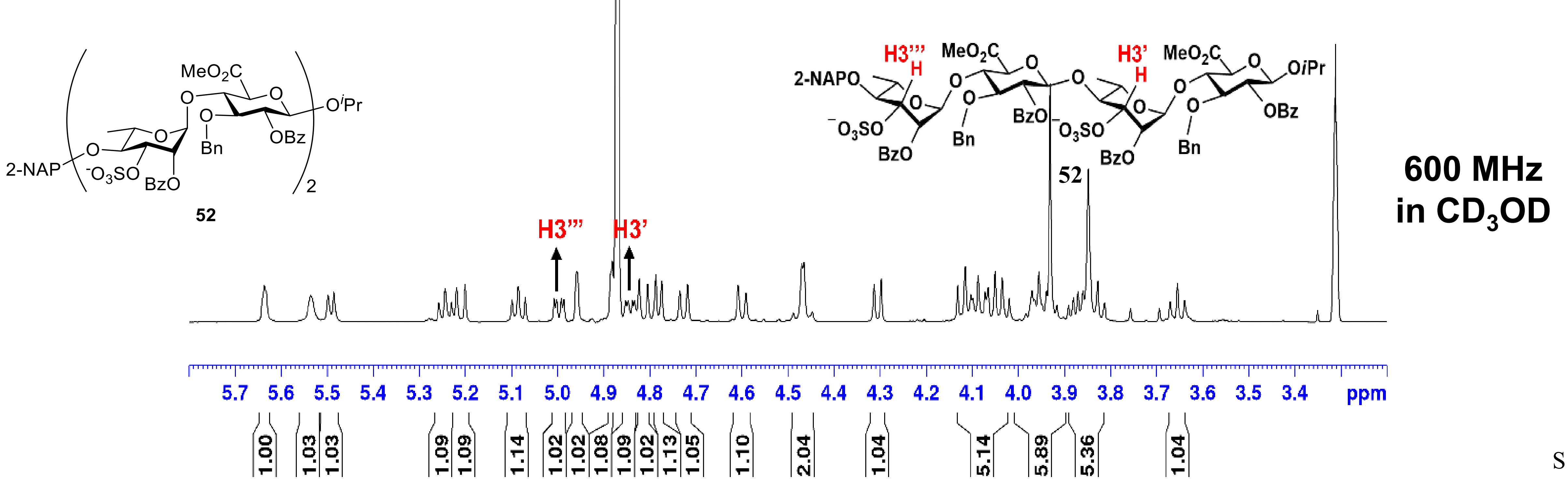
52
H3'''
H3'
600 MHz in CD_3OD
ppm

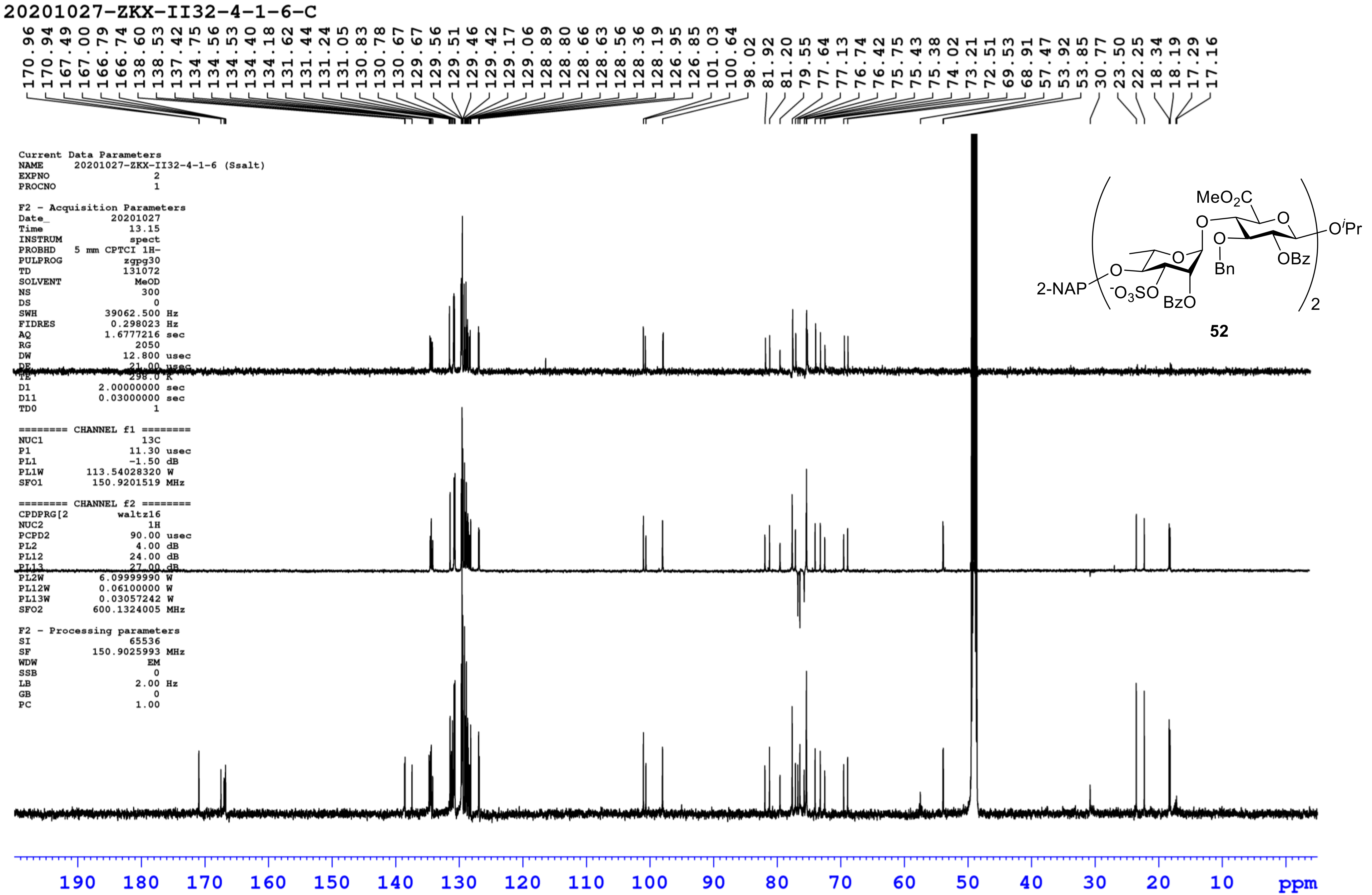
20201027-ZKX-II32-4-1-6-C
170.96
170.94
167.49
167.00
166.79
166.74
138.60
138.53
137.42
134.75
134.56
134.53
134.40
134.18
131.62
131.44
131.24
131.05
130.83
130.78
130.67
129.67
129.56
129.51
129.46
129.42
129.17
129.06
128.89
128.80
128.66
128.63
128.56
128.36
128.19
126.95
126.85
101.03
100.64
98.02
81.92
81.20
79.55
77.64
77.13
76.74
76.42
75.75
75.43
75.38
74.02
73.21
72.51
69.53
68.91
57.47
53.92
53.85
30.77
23.50
22.25
18.34
18.19
17.29
17.16
Current Data Parameters
NAME 20201027-ZKX-II32-4-1-6 (Ssalt)
EXPNO 2
PROCNO 1
F2 - Acquisition Parameters
Date_ 20201027
Time 13.15
INSTRUM spect
PROBHD 5 mm CPTCI 1H-
PULPROG zgpg30
TD 131072
SOLVENT MeOD
NS 300
DS 0
SWH 39062.500 Hz
FIDRES 0.298023 Hz
AQ 1.6777216 sec
RG 2050
DW 12.800 usec
DE 21.00 usec
TE 298.0 K
D1 2.00000000 sec
D11 0.03000000 sec
TD0 1
======== CHANNEL f1 ========
NUC1 13C
P1 11.30 usec
PL1 -1.50 dB
PL1W 113.54028320 W
SFO1 150.9201519 MHz
======== CHANNEL f2 ========
CPDPRG[2 waltz16
NUC2 1H
PCPD2 90.00 usec
PL2 4.00 dB
PL12 24.00 dB
PL13 27.00 dB
PL2W 6.09999990 W
PL12W 0.06100000 W
PL13W 0.03057242 W
SFO2 600.1324005 MHz
F2 - Processing parameters
SI 65536
SF 150.9025993 MHz
WDW EM
SSB 0
LB 2.00 Hz
GB 0
PC 1.00
MeO2C
O
O
OiPr
O
Bn
OBz
O
2-NAP
-O3SO
BzO
2
52
190 180 170 160 150 140 130 120 110 100 90 80 70 60 50 40 30 20 10 ppm

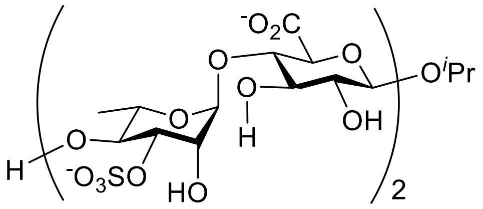

2

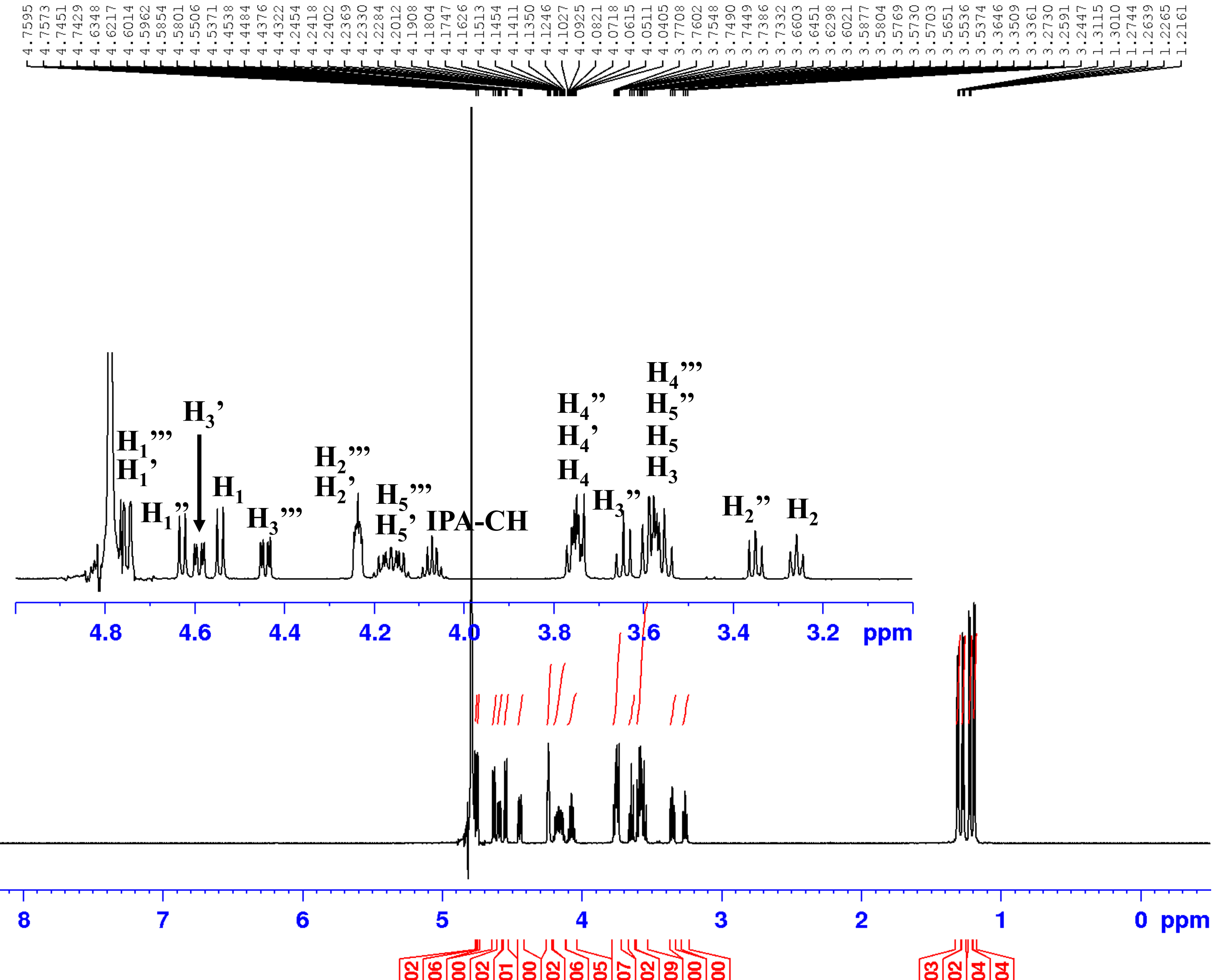

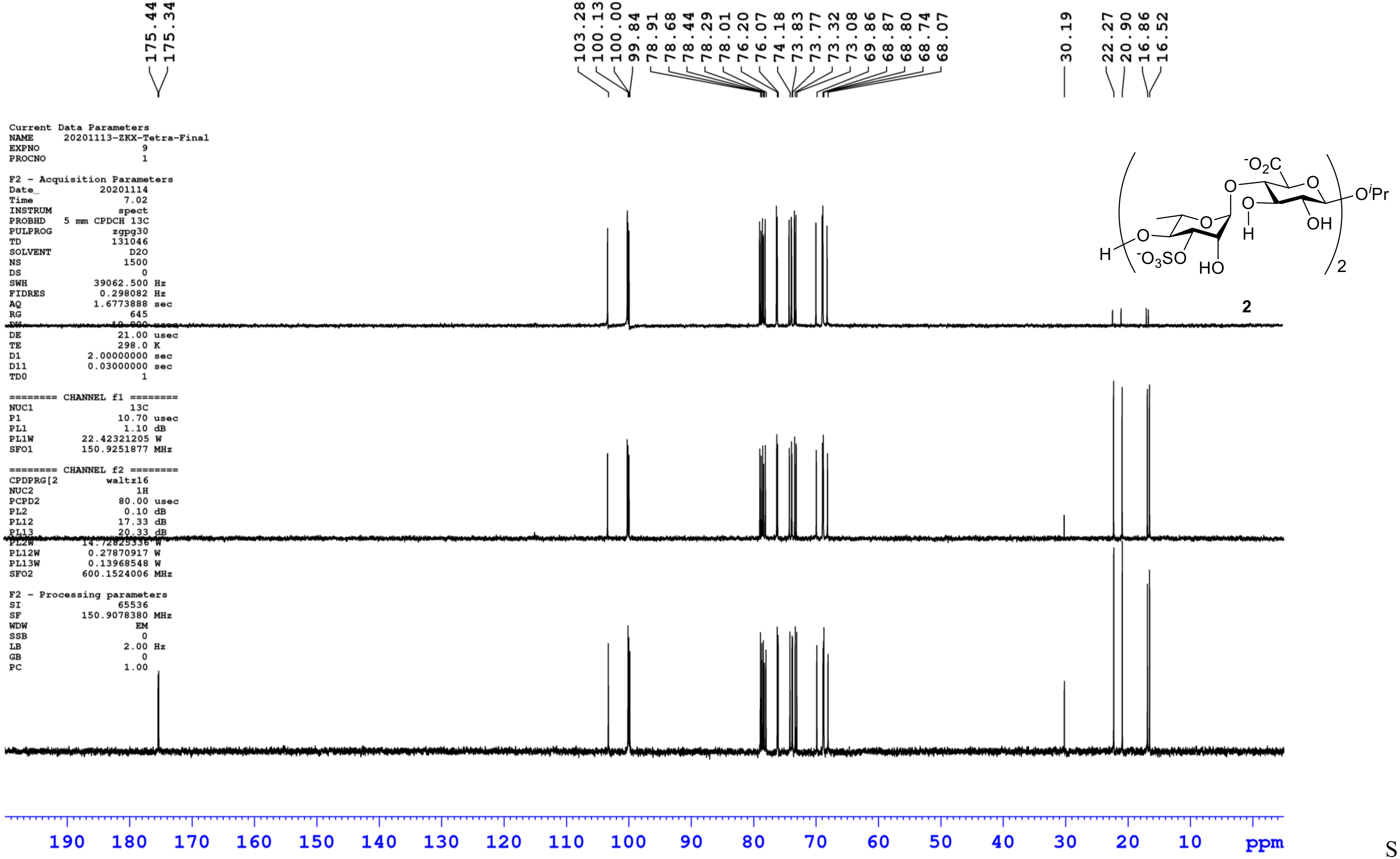
175.44
175.34
103.28
100.13
100.00
99.84
78.91
78.68
78.44
78.29
78.01
76.20
76.07
74.18
73.83
73.77
73.32
73.08
69.86
68.87
68.80
68.74
68.07
30.19
22.27
20.90
16.86
16.52
Current Data Parameters
NAME 20201113-ZKX-Tetra-Final
EXPNO 9
PROCNO 1
F2 - Acquisition Parameters
Date_ 20201114
Time 7.02
INSTRUM spect
PROBHD 5 mm CPDCH 13C
PULPROG zgpg30
TD 131046
SOLVENT D2O
NS 1500
DS 0
SWH 39062.500 Hz
FIDRES 0.298082 Hz
AQ 1.6773888 sec
RG 645
DW 10.000 usec
DE 21.00 usec
TE 298.0 K
D1 2.00000000 sec
D11 0.03000000 sec
TD0 1
======== CHANNEL f1 ========
NUC1 13C
P1 10.70 usec
PL1 1.10 dB
PL1W 22.42321205 W
SFO1 150.9251877 MHz
======== CHANNEL f2 ========
CPDPRG[2 waltz16
NUC2 1H
PCPD2 80.00 usec
PL2 0.10 dB
PL12 17.33 dB
PL13 20.33 dB
PL2W 14.72825336 W
PL12W 0.27870917 W
PL13W 0.13968548 W
SFO2 600.1524006 MHz
F2 - Processing parameters
SI 65536
SF 150.9078380 MHz
WDW EM
SSB 0
LB 2.00 Hz
GB 0
PC 1.00
190 180 170 160 150 140 130 120 110 100 90 80 70 60 50 40 30 20 10 ppm

2

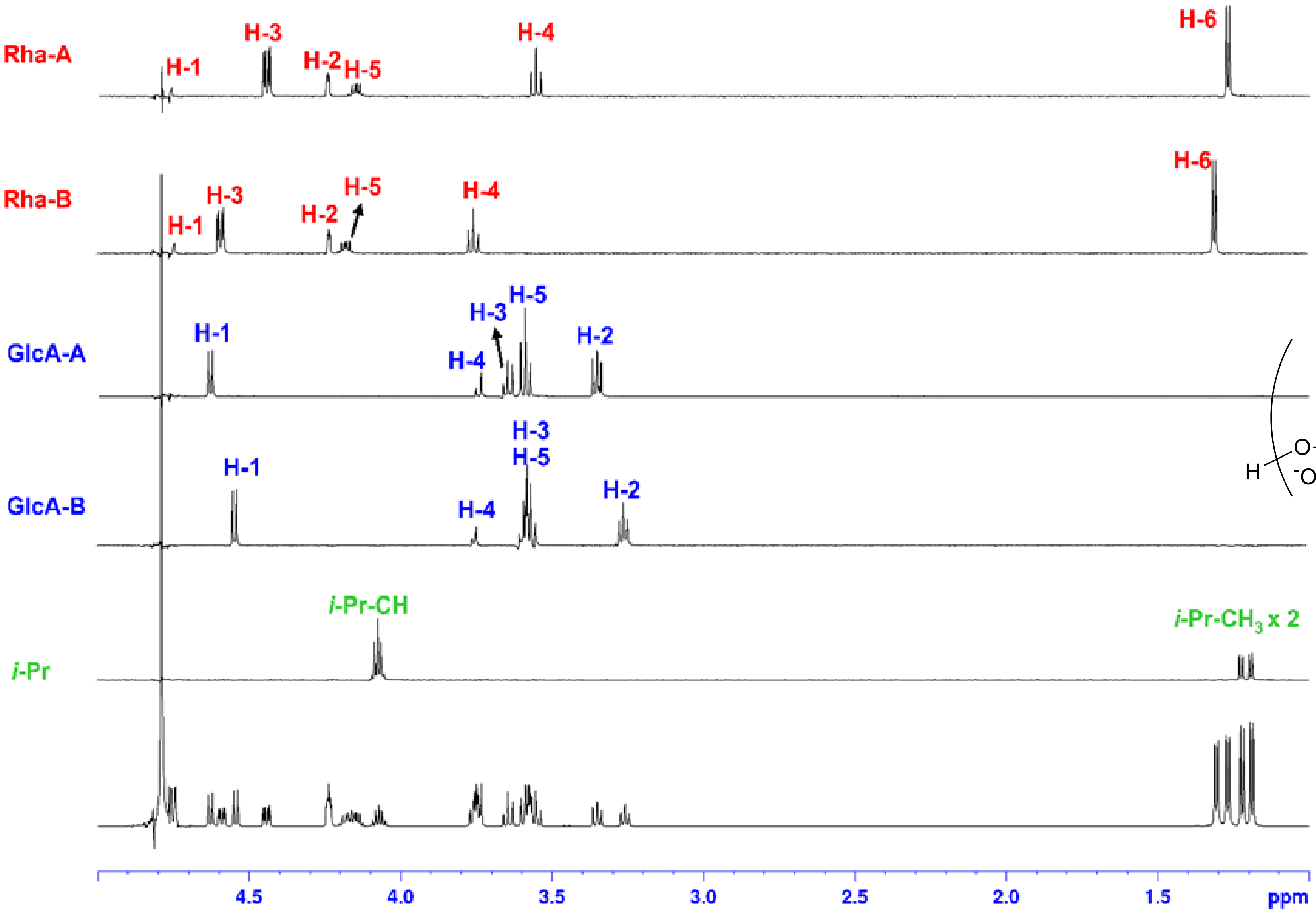
Rha-A
H-1
H-3
H-2
H-5
H-4
H-6
Rha-B
H-1
H-3
H-2
H-5
H-4
H-6
GlcA-A
H-1
H-4
H-3
H-5
H-2
GlcA-B
H-1
H-4
H-3
H-5
H-2
i-Pr
i-Pr-CH
i-Pr-CH3 x 2
4.5
4.0
3.5
3.0
2.5
2.0
1.5
ppm

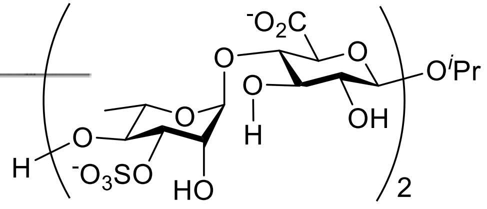

2

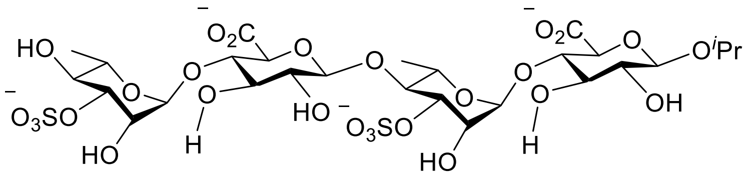

2

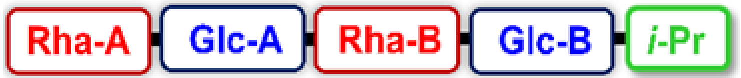
Rha-A
Glc-A
Rha-B
Glc-B
i-Pr

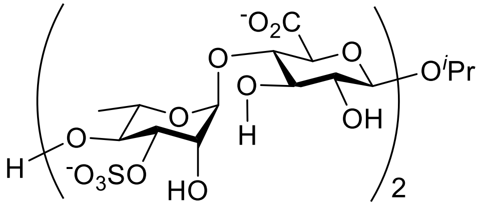

2

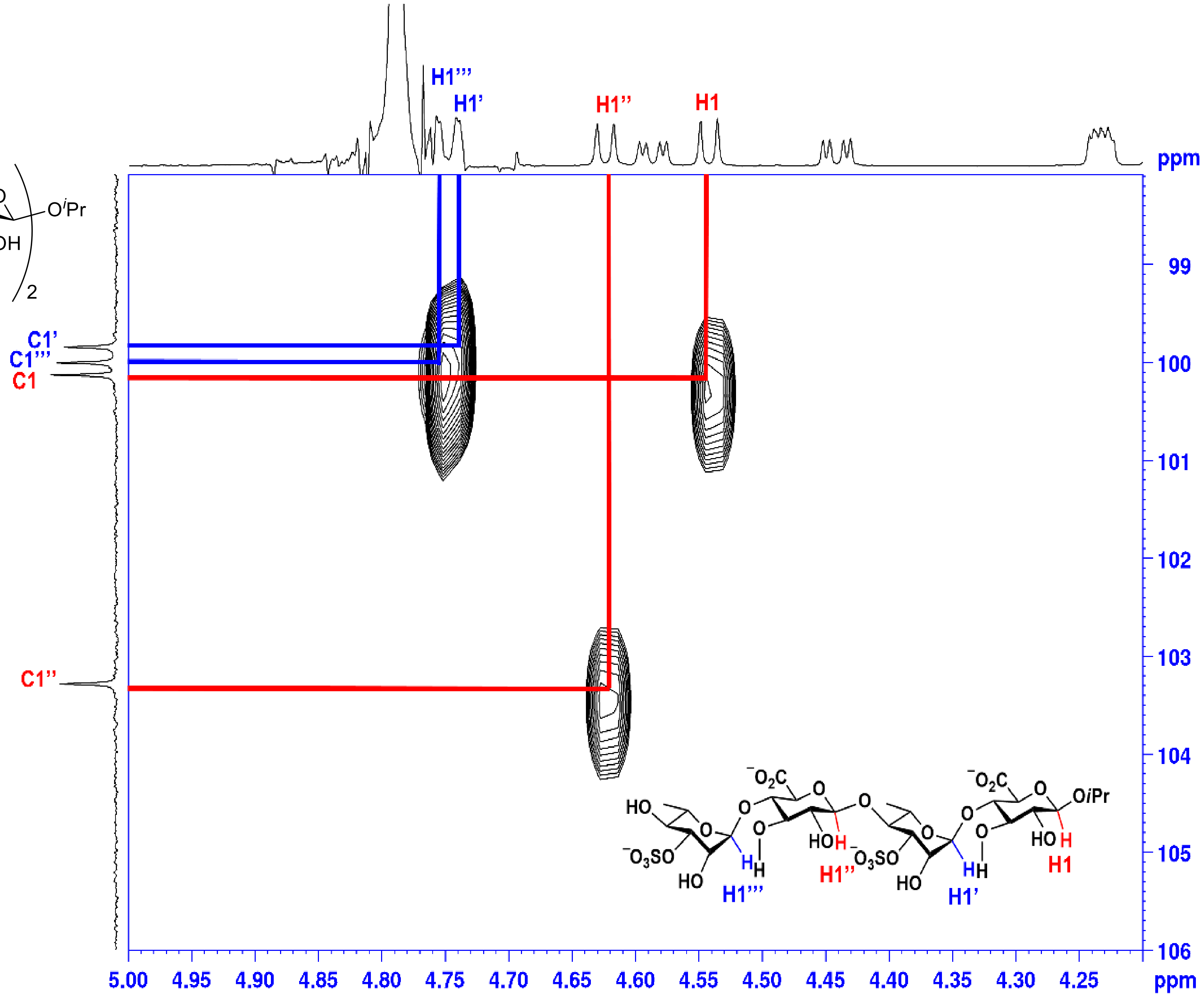

H1'''
H1'
H1''
H1
ppm
C1'
C1'''
C1
C1''
99
100
101
102
103
104
105
106
5.00
4.95
4.90
4.85
4.80
4.75
4.70
4.65
4.60
4.55
4.50
4.45
4.40
4.35
4.30
4.25
ppm

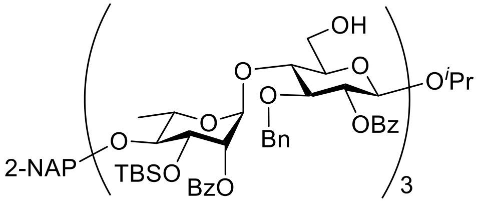
OH
O
O^{i}Pr
OBz
Bn
2-NAP
TBSO
BzO
3

40

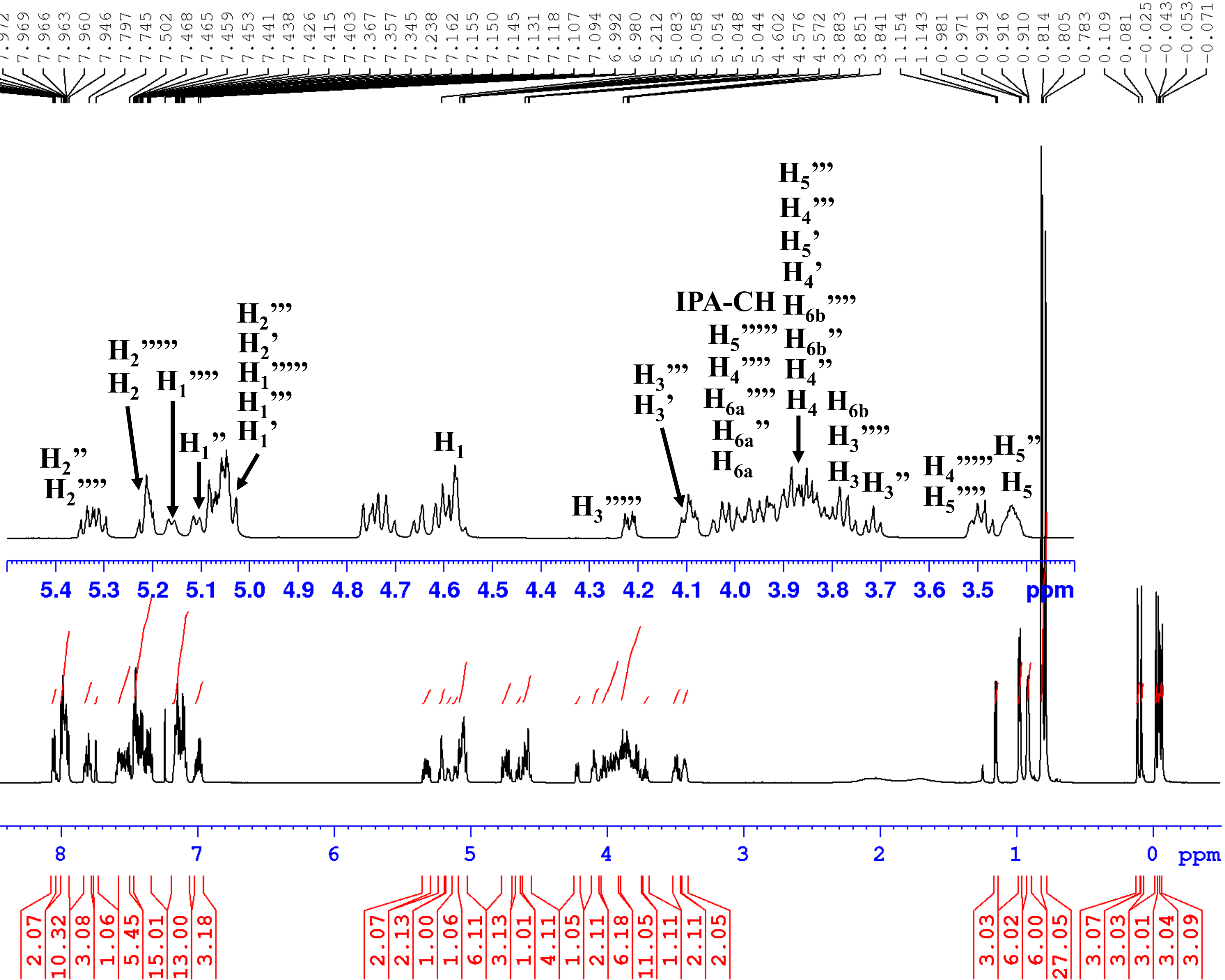
8.062 8.061 8.049 8.046 7.999 7.986 7.983 7.981 7.976 7.975 7.972 7.969 7.966 7.963 7.960 7.946 7.797 7.745 7.502 7.468 7.465 7.459 7.453 7.441 7.438 7.426 7.415 7.403 7.367 7.357 7.345 7.238 7.162 7.155 7.150 7.145 7.131 7.118 7.107 7.094 6.992 6.980 5.212 5.083 5.058 5.054 5.048 5.044 4.602 4.576 4.572 3.883 3.851 3.841 1.154 1.143 0.981 0.971 0.919 0.916 0.910 0.814 0.805 0.783 0.109 0.081 −0.025 −0.043 −0.053 −0.071
H2'' H2''''
H2''''' H2
H1''''
H1''
H2''' H2' H1''''' H1''' H1'
H1
H3'''''
H3''' H3'
IPA-CH
H5''''' H4'''' H6a'''' H6a'' H6a
H5''' H4''' H5' H4' H6b'''' H6b'' H4'' H4
H6b H3'''' H3
H3''
H4''''' H5''''
H5'' H5
5.4 5.3 5.2 5.1 5.0 4.9 4.8 4.7 4.6 4.5 4.4 4.3 4.2 4.1 4.0 3.9 3.8 3.7 3.6 3.5 ppm
9 8 7 6 5 4 3 2 1 0 ppm
2.07 10.32 3.08 1.06 5.45 15.01 13.00 3.18
2.07 2.13 1.00 1.06 6.11 3.13 1.01 4.11 1.05 2.11 6.18 11.05 1.11 2.11 2.05
3.03 6.02 6.00 27.05 3.07 3.03 3.01 3.04 3.09

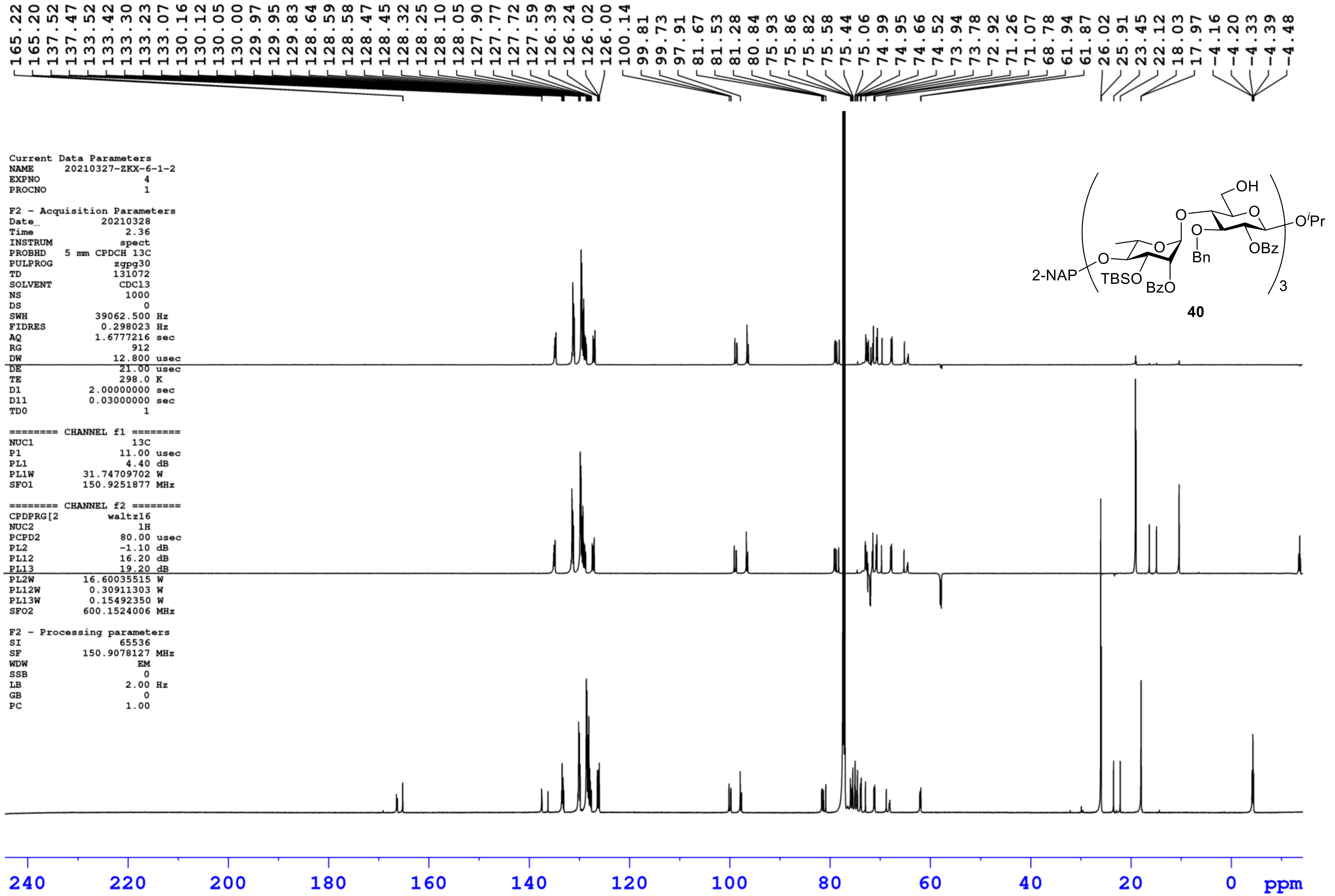
165.22
165.20
137.52
137.47
133.52
133.42
133.30
133.23
133.07
130.16
130.12
130.05
130.00
129.97
129.95
129.83
128.64
128.59
128.58
128.47
128.45
128.32
128.25
128.10
128.05
127.90
127.77
127.72
127.59
126.39
126.24
126.02
126.00
100.14
99.81
99.73
97.91
81.67
81.53
81.28
80.84
75.93
75.86
75.82
75.58
75.44
75.06
74.99
74.95
74.66
74.52
73.94
73.78
72.92
71.26
71.07
68.78
61.94
61.87
26.02
25.91
23.45
22.12
18.03
17.97
-4.16
-4.20
-4.33
-4.39
-4.48
Current Data Parameters
NAME 20210327-ZKX-6-1-2
EXPNO 4
PROCNO 1
F2 - Acquisition Parameters
Date_ 20210328
Time 2.36
INSTRUM spect
PROBHD 5 mm CPDCH 13C
PULPROG zgpg30
TD 131072
SOLVENT CDCl3
NS 1000
DS 0
SWH 39062.500 Hz
FIDRES 0.298023 Hz
AQ 1.6777216 sec
RG 912
DW 12.800 usec
DE 21.00 usec
TE 298.0 K
D1 2.00000000 sec
D11 0.03000000 sec
TD0 1
======== CHANNEL f1 ========
NUC1 13C
P1 11.00 usec
PL1 4.40 dB
PL1W 31.74709702 W
SFO1 150.9251877 MHz
======== CHANNEL f2 ========
CPDPRG[2 waltz16
NUC2 1H
PCPD2 80.00 usec
PL2 -1.10 dB
PL12 16.20 dB
PL13 19.20 dB
PL2W 16.60035515 W
PL12W 0.30911303 W
PL13W 0.15492350 W
SFO2 600.1524006 MHz
F2 - Processing parameters
SI 65536
SF 150.9078127 MHz
WDW EM
SSB 0
LB 2.00 Hz
GB 0
PC 1.00
OH
O
O
O
O^iPr
O
Bn
OBz
2-NAP
O
TBSO
BzO
3
40
240
220
200
180
160
140
120
100
80
60
40
20
0
ppm

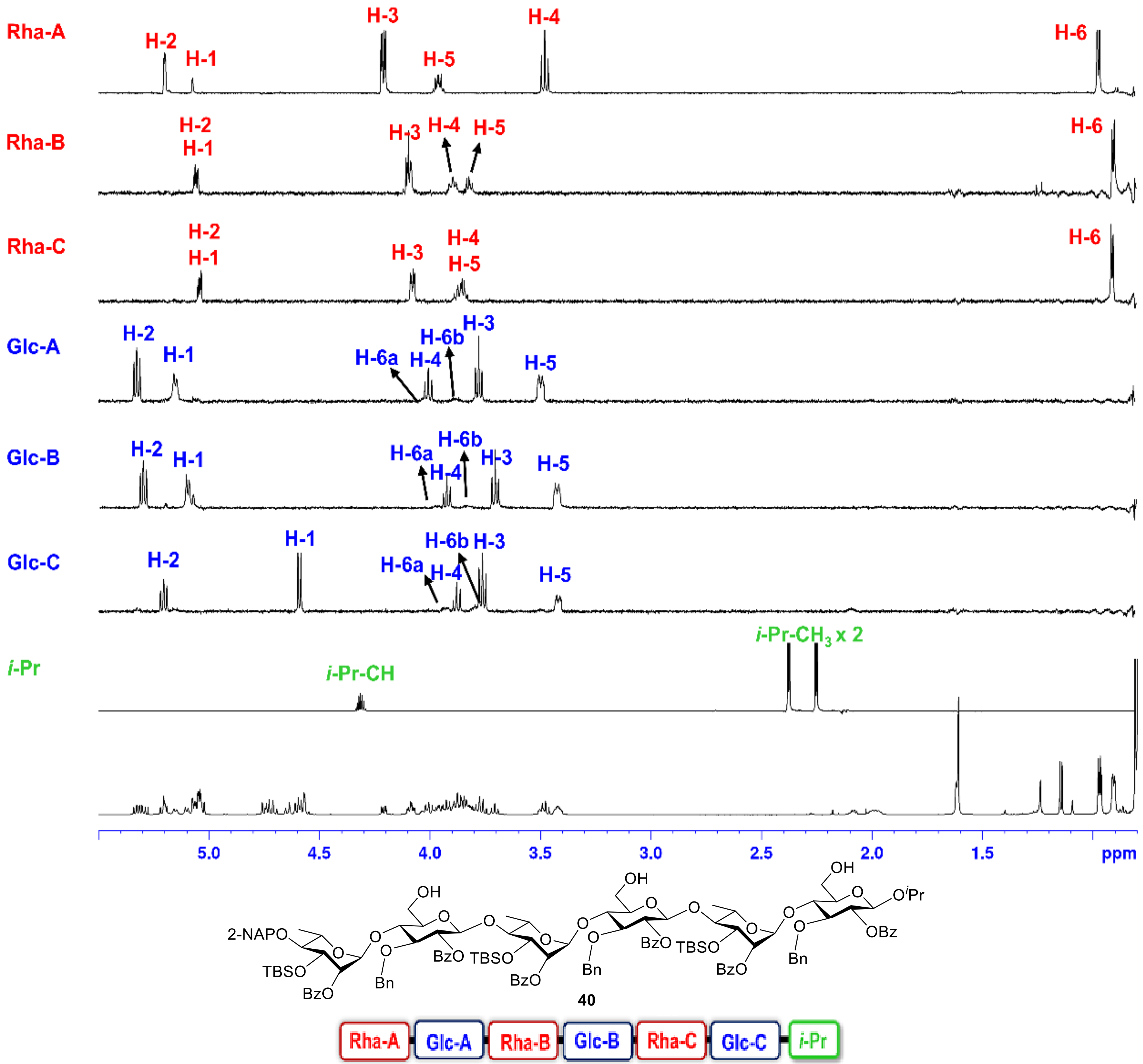

Rha-A
H-2
H-1
H-3
H-5
H-4
H-6
Rha-B
H-2
H-1
H-3
H-4
H-5
H-6
Rha-C
H-2
H-1
H-3
H-4
H-5
H-6
Glc-A
H-2
H-1
H-6a
H-6b
H-4
H-3
H-5
Glc-B
H-2
H-1
H-6a
H-6b
H-4
H-3
H-5
Glc-C
H-2
H-1
H-6a
H-6b
H-4
H-3
H-5
i-Pr
i-Pr-CH
i-Pr-CH3 x 2
5.0
4.5
4.0
3.5
3.0
2.5
2.0
1.5
ppm
2-NAPO
TBSO
BzO
OH
Bn
BzO
TBSO
BzO
OH
Bn
BzO
TBSO
BzO
OH
O^iPr
OBz
Bn
40
Rha-A
Glc-A
Rha-B
Glc-B
Rha-C
Glc-C
i-Pr

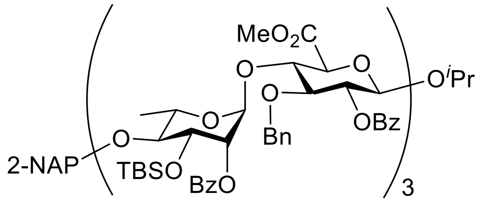
MeO2C
O
O
O
OiPr
O
Bn
OBz
2-NAP
TBSO
BzO
3

44

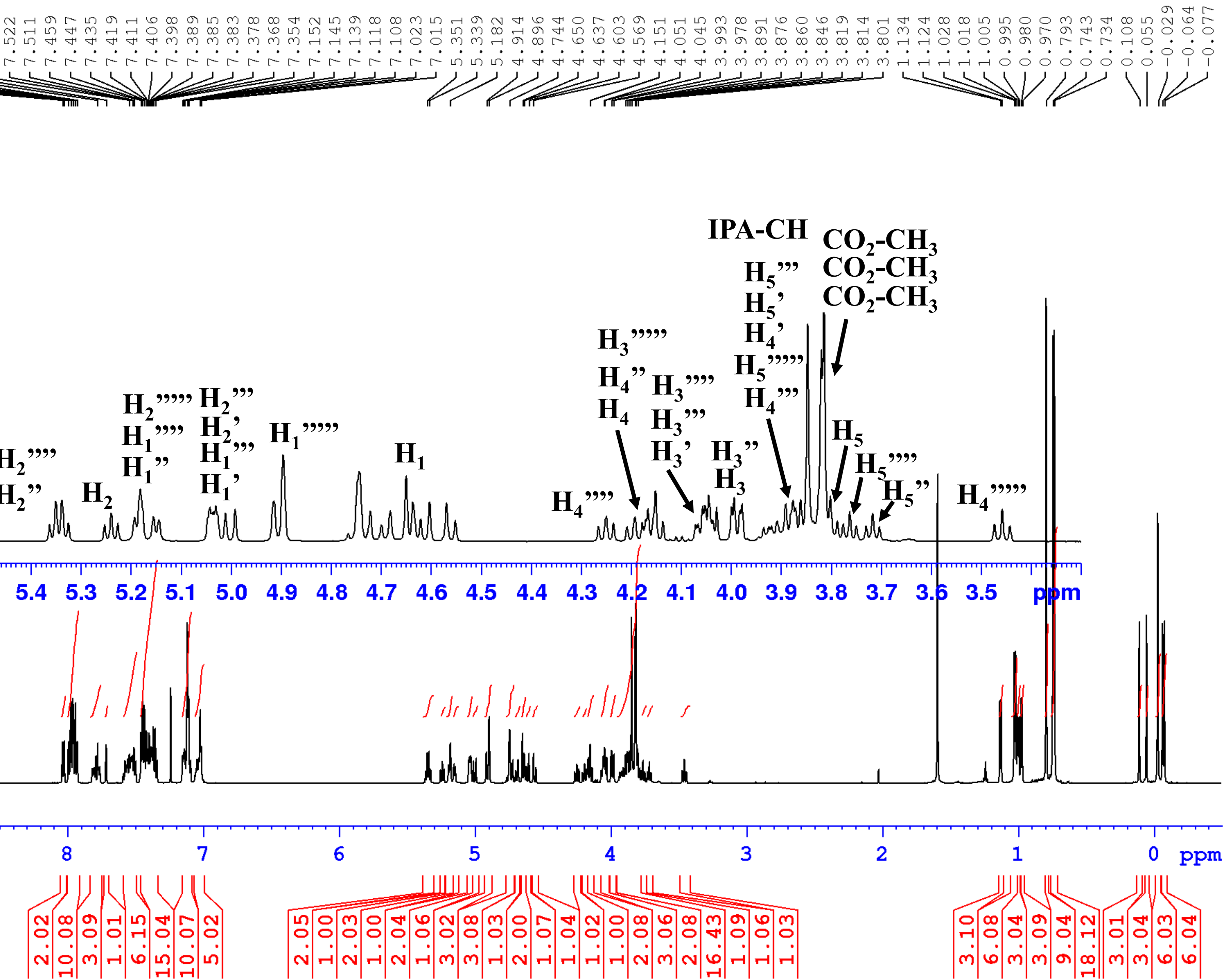
8.038 8.025 7.993 7.975 7.961 7.941 7.928 7.779 7.716 7.544 7.522 7.511 7.459 7.447 7.435 7.419 7.411 7.406 7.398 7.389 7.385 7.383 7.378 7.368 7.354 7.152 7.145 7.139 7.118 7.108 7.023 7.015 5.351 5.339 5.182 4.914 4.896 4.744 4.650 4.637 4.603 4.569 4.151 4.051 4.045 3.993 3.978 3.891 3.876 3.860 3.846 3.819 3.814 3.801 1.134 1.124 1.028 1.018 1.005 0.995 0.980 0.970 0.793 0.743 0.734 0.108 0.055 -0.029 -0.064 -0.077
IPA-CH
CO2-CH3
CO2-CH3
CO2-CH3
H5'''
H5'
H4'
H5'''''
H4'''
H3'''''
H4''
H4
H3''''
H3'''
H3'
H3''
H3
H5
H5''''
H5''
H4''''
H4'''''
H2''''
H2''
H2
H2'''''
H1''''
H1''
H2'''
H2'
H1'''
H1'
H1'''''
H1
5.4 5.3 5.2 5.1 5.0 4.9 4.8 4.7 4.6 4.5 4.4 4.3 4.2 4.1 4.0 3.9 3.8 3.7 3.6 3.5 ppm
9 8 7 6 5 4 3 2 1 0 ppm
2.02 10.08 3.09 1.01 6.15 15.04 10.07 5.02
2.05 1.00 2.03 1.00 2.04 1.06 3.02 3.08 1.03 2.00 1.07 1.04 1.02 1.00 2.08 3.06 2.08 16.43 1.09 1.06 1.03
3.10 6.08 3.04 3.09 9.04 18.12 3.01 3.04 6.03 6.04

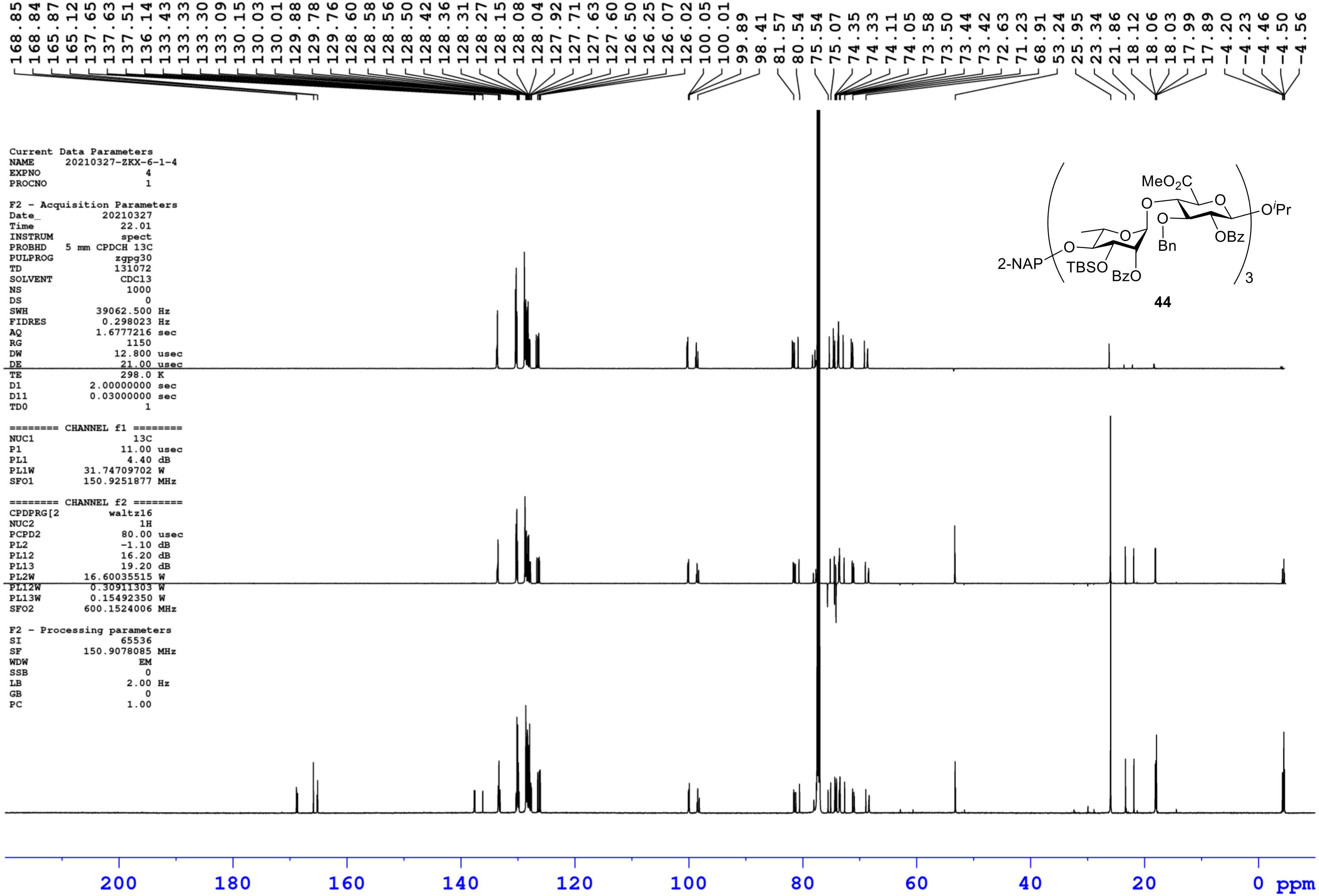


```
Current Data Parameters
NAME      20210327-ZKX-6-1-4
EXPNO                   4
PROCNO                  1

F2 - Acquisition Parameters
Date_            20210327
Time                22.01
INSTRUM             spect
PROBHD   5 mm CPDCH 13C
PULPROG           zgpg30
TD                 131072
SOLVENT             CDCl3
NS                   1000
DS                      0
SWH             39062.500 Hz
FIDRES           0.298023 Hz
AQ              1.6777216 sec
RG                   1150
DW                 12.800 usec
DE                  21.00 usec
TE                  298.0 K
D1             2.00000000 sec
D11            0.03000000 sec
TD0                     1

======== CHANNEL f1 ========
NUC1                  13C
P1                  11.00 usec
PL1                  4.40 dB
PL1W          31.74709702 W
SFO1          150.9251877 MHz

======== CHANNEL f2 ========
CPDPRG[2          waltz16
NUC2                   1H
PCPD2               80.00 usec
PL2                 -1.10 dB
PL12                16.20 dB
PL13                19.20 dB
PL2W          16.60035515 W
PL12W          0.30911303 W
PL13W          0.15492350 W
SFO2          600.1524006 MHz

F2 - Processing parameters
SI                  65536
SF            150.9078085 MHz
WDW                    EM
SSB                     0
LB                   2.00 Hz
GB                      0
PC                   1.00
```

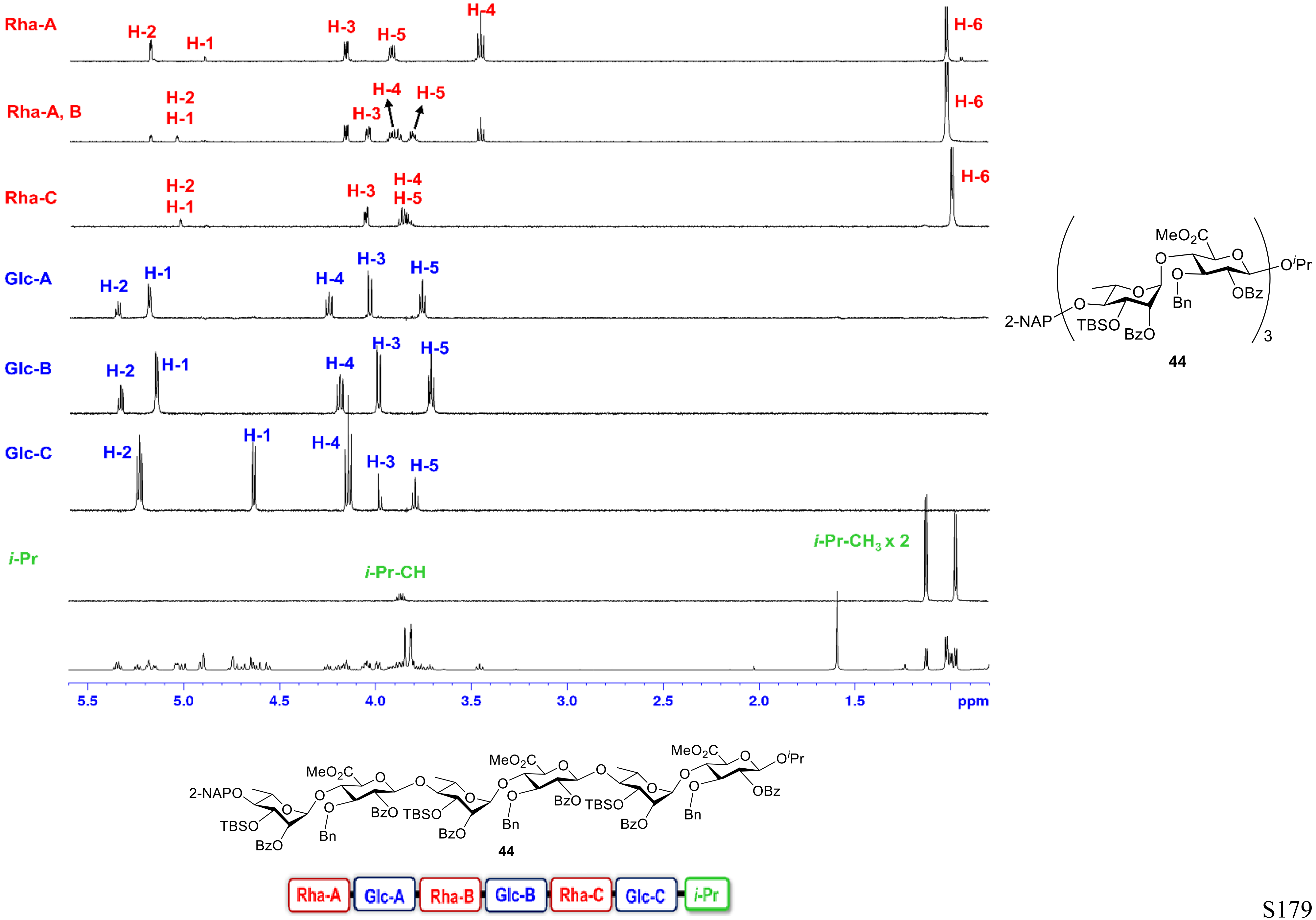
Rha-A
H-2
H-1
H-3
H-5
H-4
H-6
Rha-A, B
Rha-C
Glc-A
Glc-B
Glc-C
i-Pr
i-Pr-CH
i-Pr-CH3 x 2
5.5
5.0
4.5
4.0
3.5
3.0
2.5
2.0
1.5
ppm
MeO2C
2-NAP
TBSO
BzO
Bn
OBz
O^{i}Pr
44
2-NAPO
Rha-A
Glc-A
Rha-B
Glc-B
Rha-C
Glc-C
i-Pr

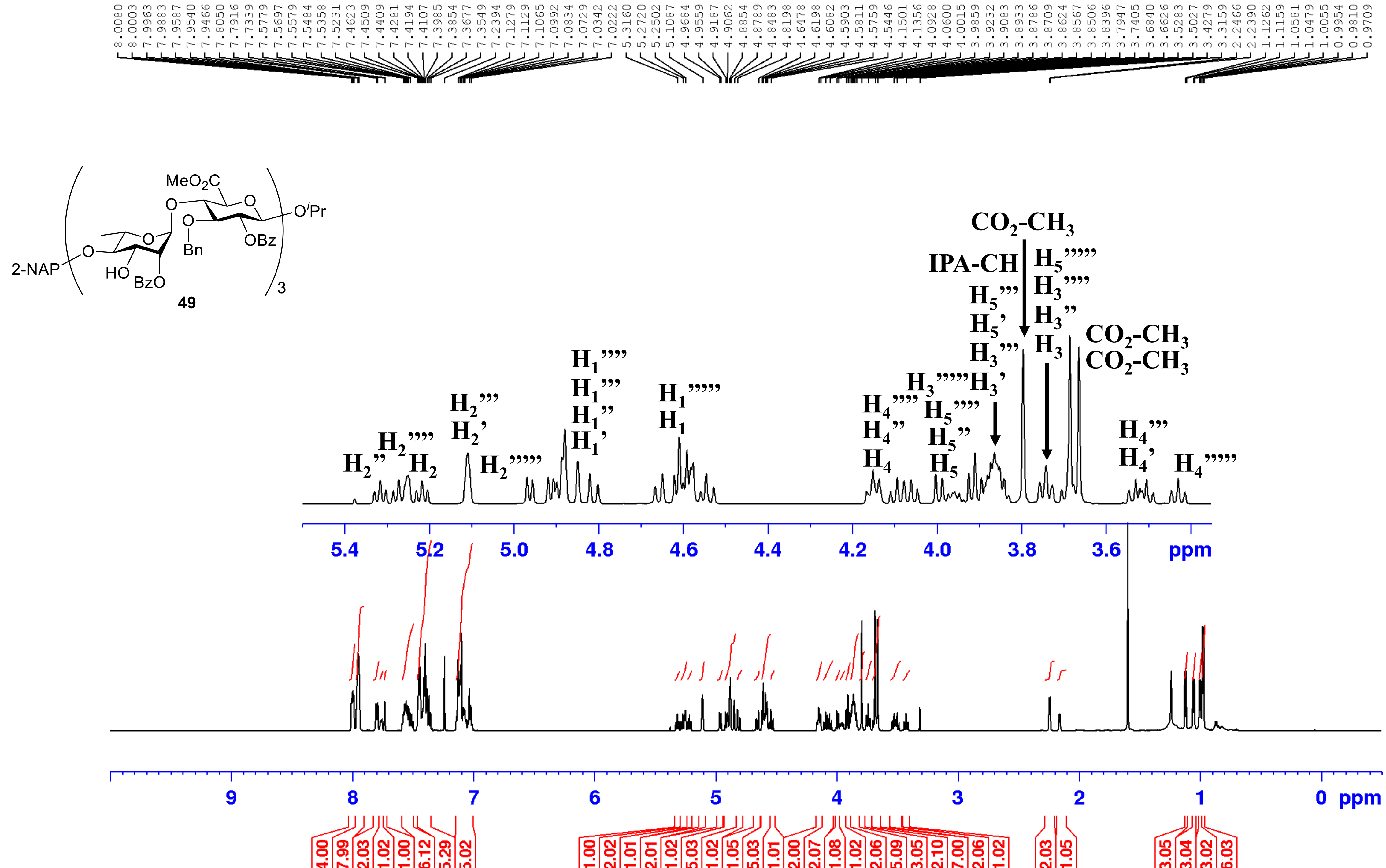

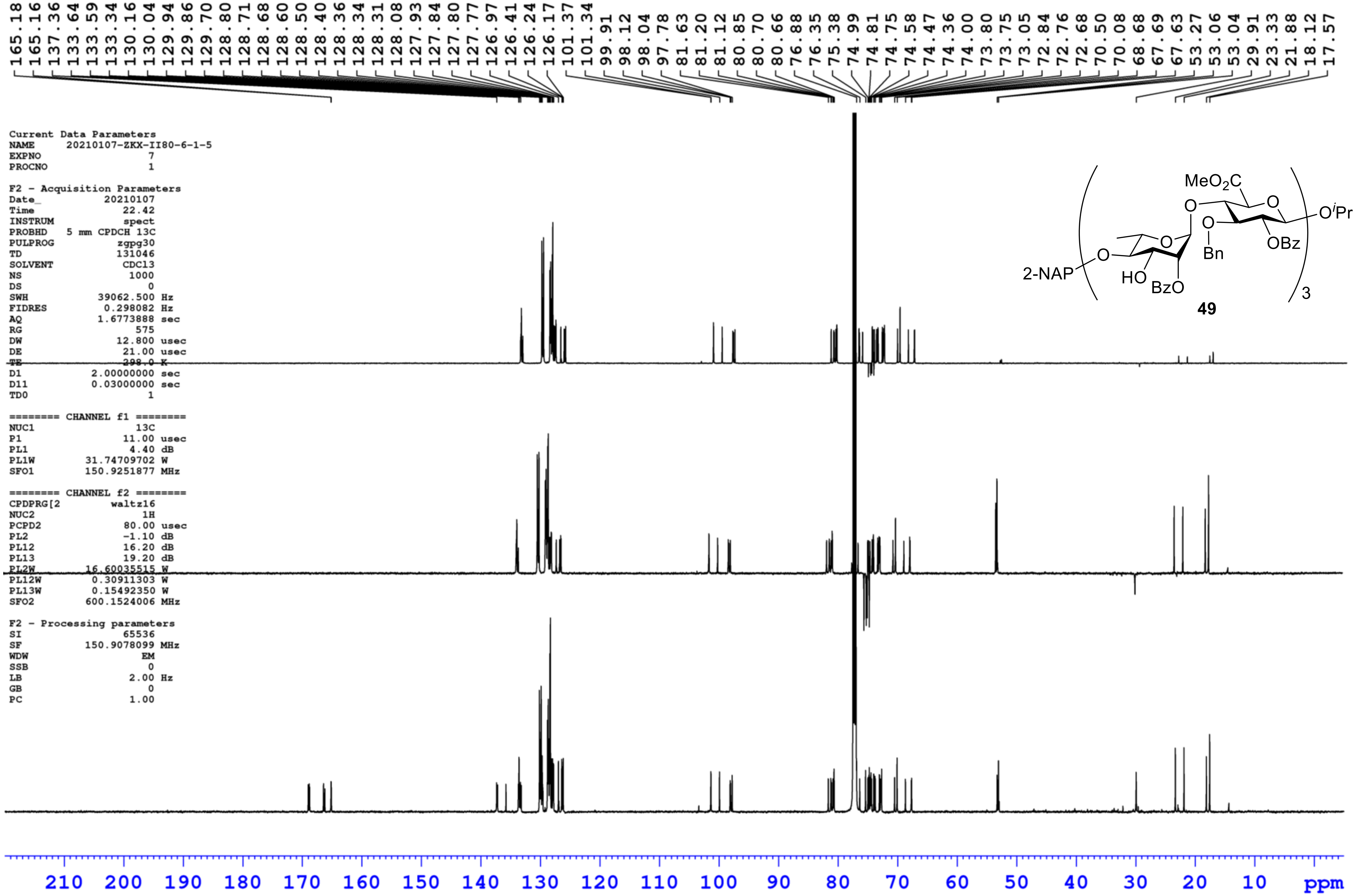
165.18
165.16
137.36
133.64
133.59
133.34
130.16
130.04
129.94
129.86
129.70
128.80
128.71
128.68
128.60
128.50
128.40
128.36
128.34
128.31
128.08
127.93
127.84
127.80
127.77
126.97
126.41
126.24
126.17
101.37
101.34
99.91
98.12
98.04
97.78
81.63
81.20
81.12
80.85
80.70
80.66
76.88
76.35
75.38
74.99
74.81
74.75
74.58
74.47
74.36
74.00
73.80
73.75
73.05
72.84
72.76
72.68
70.50
70.08
68.68
67.69
67.63
53.27
53.06
53.04
29.91
23.33
21.88
18.12
17.57
Current Data Parameters
NAME 20210107-ZKX-II80-6-1-5
EXPNO 7
PROCNO 1
F2 - Acquisition Parameters
Date_ 20210107
Time 22.42
INSTRUM spect
PROBHD 5 mm CPDCH 13C
PULPROG zgpg30
TD 131046
SOLVENT CDCl3
NS 1000
DS 0
SWH 39062.500 Hz
FIDRES 0.298082 Hz
AQ 1.6773888 sec
RG 575
DW 12.800 usec
DE 21.00 usec
TE 298.0 K
D1 2.00000000 sec
D11 0.03000000 sec
TD0 1
======== CHANNEL f1 ========
NUC1 13C
P1 11.00 usec
PL1 4.40 dB
PL1W 31.74709702 W
SFO1 150.9251877 MHz
======== CHANNEL f2 ========
CPDPRG[2 waltz16
NUC2 1H
PCPD2 80.00 usec
PL2 -1.10 dB
PL12 16.20 dB
PL13 19.20 dB
PL2W 16.60035515 W
PL12W 0.30911303 W
PL13W 0.15492350 W
SFO2 600.1524006 MHz
F2 - Processing parameters
SI 65536
SF 150.9078099 MHz
WDW EM
SSB 0
LB 2.00 Hz
GB 0
PC 1.00
MeO2C
O
O
O
OiPr
O
Bn
OBz
2-NAP
O
HO
BzO
3
49
210 200 190 180 170 160 150 140 130 120 110 100 90 80 70 60 50 40 30 20 10 ppm

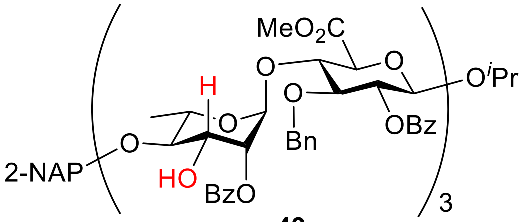
MeO2C
O
H
O
O
OiPr
Bn
OBz
2-NAP
O
HO
BzO
3

49

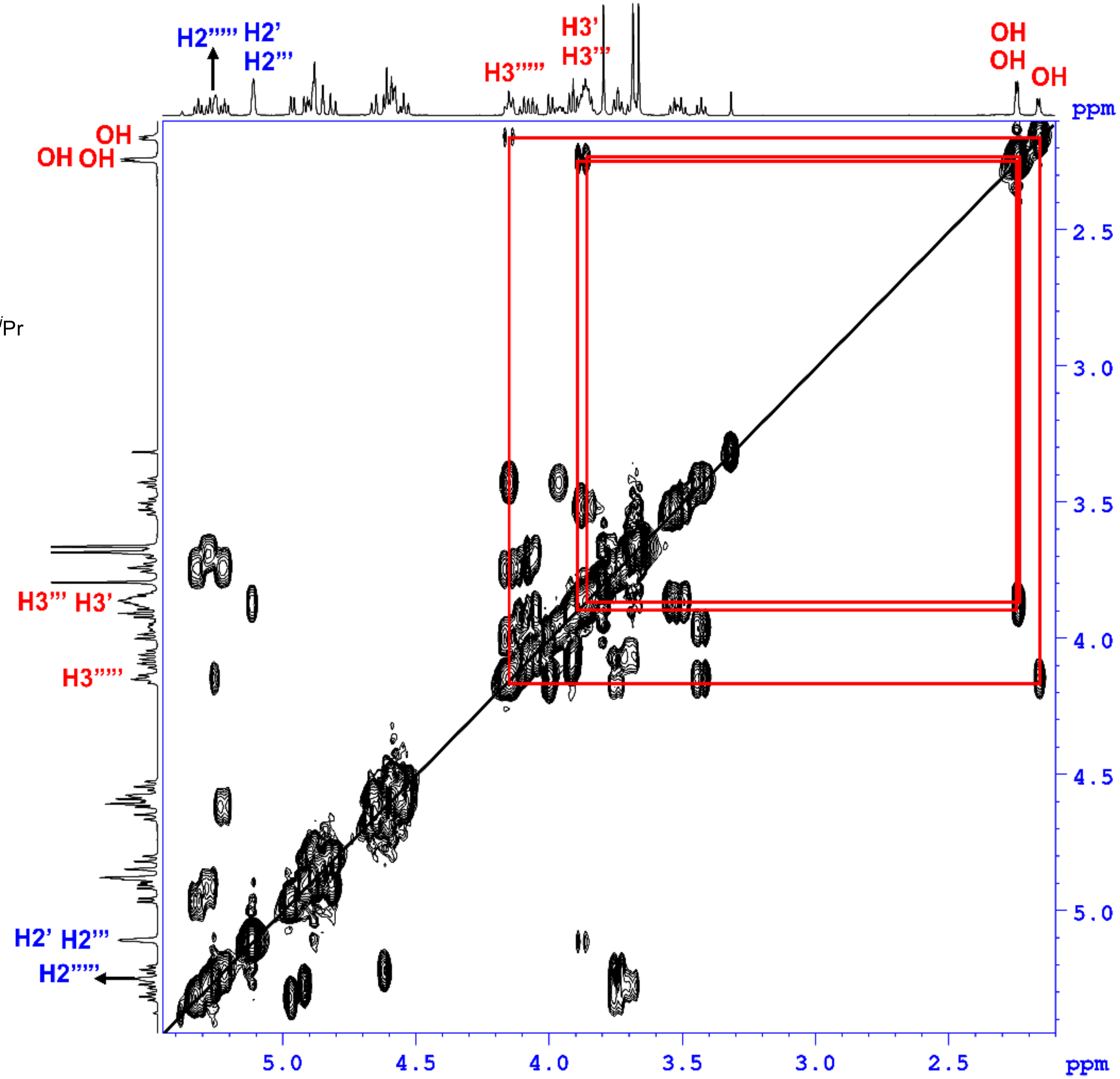
H2''''' H2'
H2'''
H3'
H3'''
H3'''''
OH
OH
OH
ppm
OH
OH OH
H3''' H3'
H3'''''
H2' H2'''
H2'''''
2.5
3.0
3.5
4.0
4.5
5.0
5.0
4.5
4.0
3.5
3.0
2.5
ppm

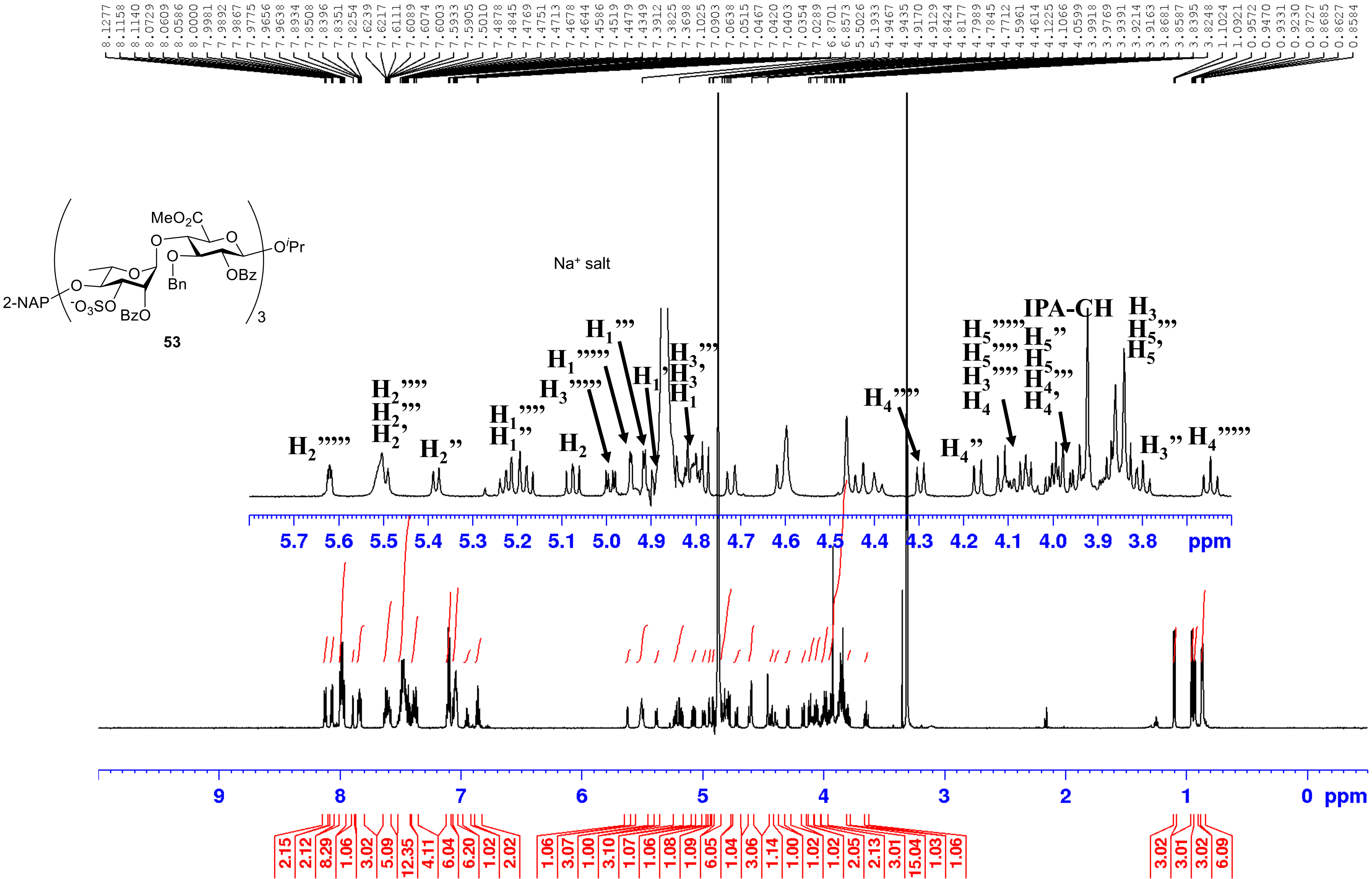

MeO2C
OiPr
OBz
Bn
2-NAP
-O3SO
BzO
3
53
Na+ salt
IPA-CH
ppm

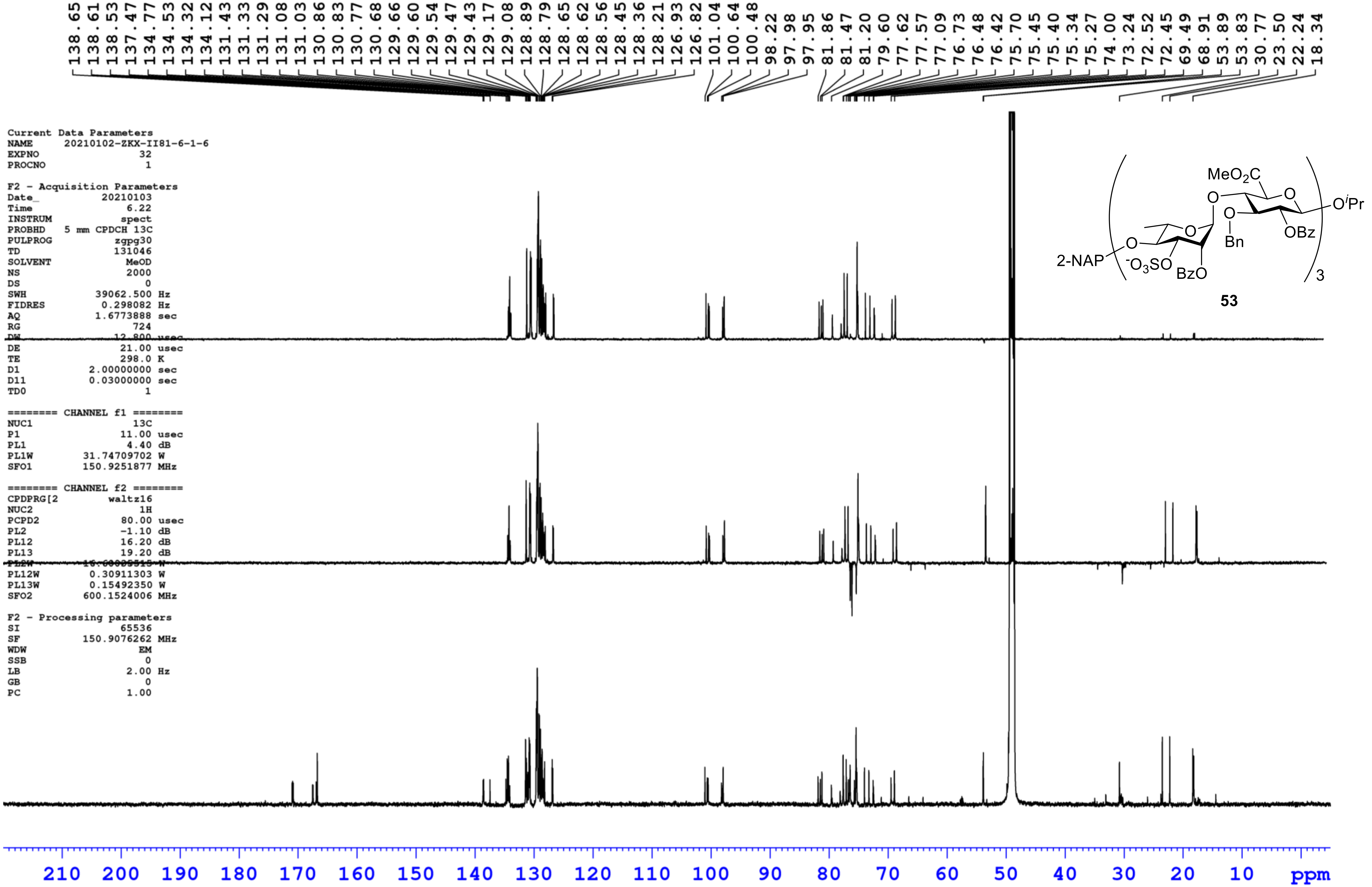
138.65
138.61
138.53
137.47
134.77
134.53
134.32
134.12
131.43
131.33
131.29
131.08
131.03
130.86
130.83
130.77
130.68
130.66
129.60
129.54
129.47
129.43
129.17
129.08
128.89
128.79
128.65
128.62
128.56
128.45
128.36
128.21
126.93
126.82
101.04
100.64
100.48
98.22
97.98
97.95
81.86
81.47
81.20
79.60
77.62
77.57
77.09
76.73
76.48
76.42
75.70
75.45
75.40
75.34
75.27
74.00
73.24
72.52
72.45
69.49
68.91
53.89
53.83
30.77
23.50
22.24
18.34
Current Data Parameters
NAME 20210102-ZKX-II81-6-1-6
EXPNO 32
PROCNO 1
F2 - Acquisition Parameters
Date_ 20210103
Time 6.22
INSTRUM spect
PROBHD 5 mm CPDCH 13C
PULPROG zgpg30
TD 131046
SOLVENT MeOD
NS 2000
DS 0
SWH 39062.500 Hz
FIDRES 0.298082 Hz
AQ 1.6773888 sec
RG 724
DW 12.800 usec
DE 21.00 usec
TE 298.0 K
D1 2.00000000 sec
D11 0.03000000 sec
TD0 1
======== CHANNEL f1 ========
NUC1 13C
P1 11.00 usec
PL1 4.40 dB
PL1W 31.74709702 W
SFO1 150.9251877 MHz
======== CHANNEL f2 ========
CPDPRG[2 waltz16
NUC2 1H
PCPD2 80.00 usec
PL2 -1.10 dB
PL12 16.20 dB
PL13 19.20 dB
PL2W 16.60035515 W
PL12W 0.30911303 W
PL13W 0.15492350 W
SFO2 600.1524006 MHz
F2 - Processing parameters
SI 65536
SF 150.9076262 MHz
WDW EM
SSB 0
LB 2.00 Hz
GB 0
PC 1.00
210 200 190 180 170 160 150 140 130 120 110 100 90 80 70 60 50 40 30 20 10 ppm
MeO2C
O
O
OiPr
O
O
Bn
OBz
2-NAP
O
-O3SO
BzO
3

53

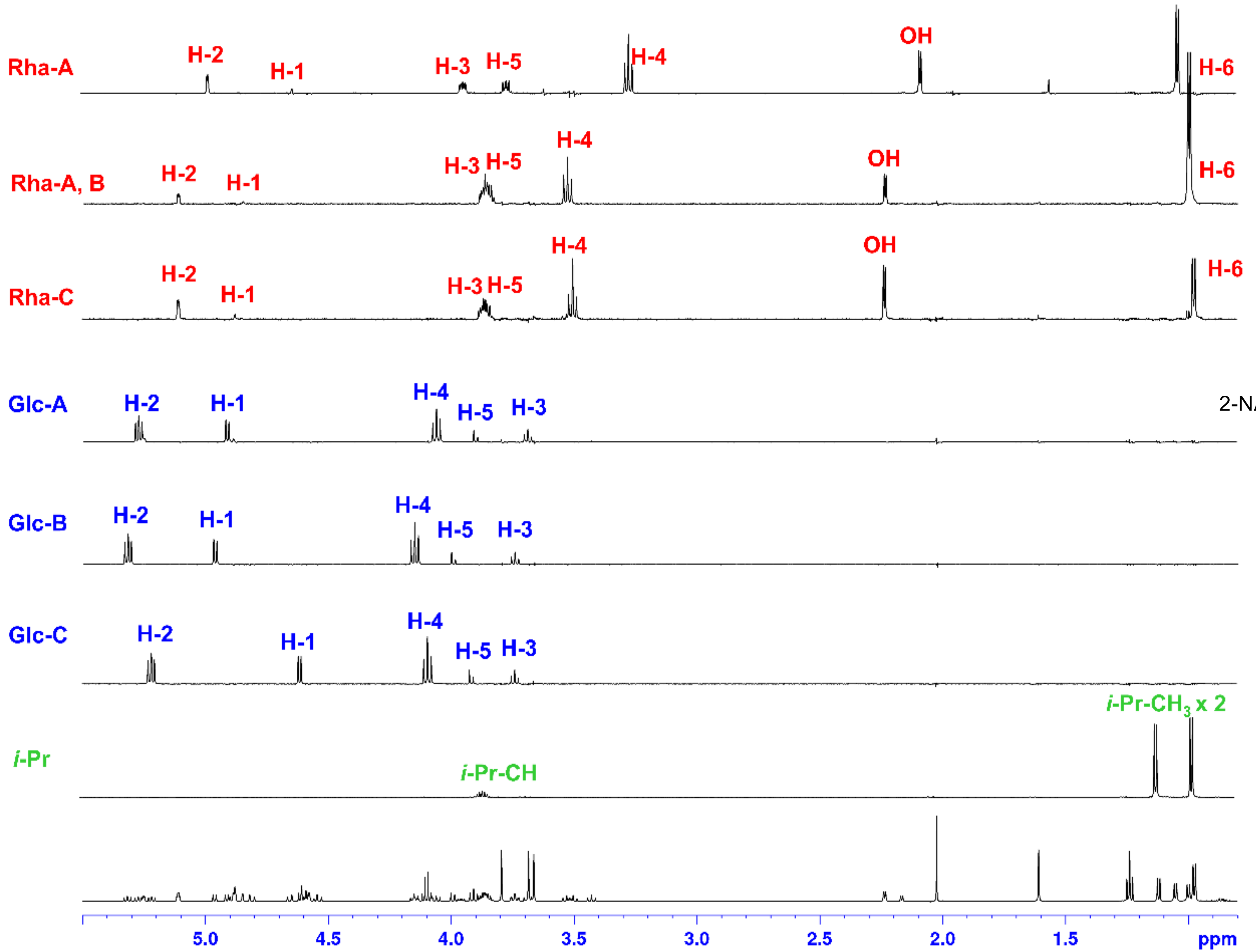

Rha-A
H-2
H-1
H-3
H-5
H-4
OH
H-6
Rha-A, B
H-2
H-1
H-3
H-5
H-4
OH
H-6
Rha-C
H-2
H-1
H-3
H-5
H-4
OH
H-6
Glc-A
H-2
H-1
H-4
H-5
H-3
Glc-B
H-2
H-1
H-4
H-5
H-3
Glc-C
H-2
H-1
H-4
H-5
H-3
i-Pr
i-Pr-CH
i-Pr-CH3 x 2
5.0
4.5
4.0
3.5
3.0
2.5
2.0
1.5
ppm

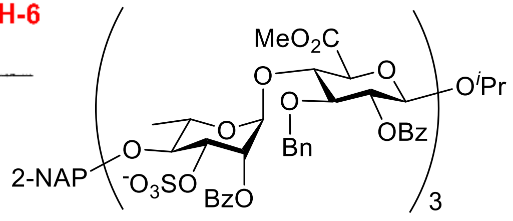

MeO2C
O
O
OiPr
O
Bn
OBz
O
O
2-NAP
-O3SO
BzO
3

53

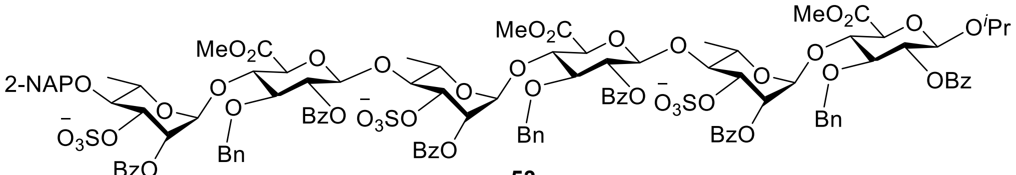

2-NAPO
MeO2C
O
O
O
O
O3SO
BzO
Bn
BzO
O3SO
BzO
MeO2C
Bn
BzO
O3SO
BzO
MeO2C
OiPr
OBz
Bn

53

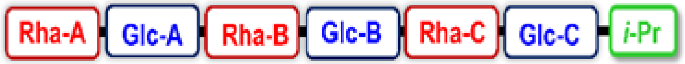

Rha-A
Glc-A
Rha-B
Glc-B
Rha-C
Glc-C
i-Pr

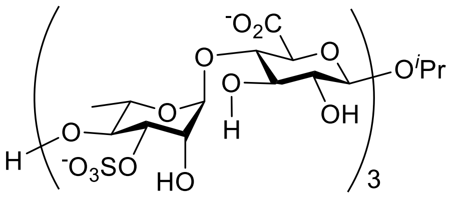

3

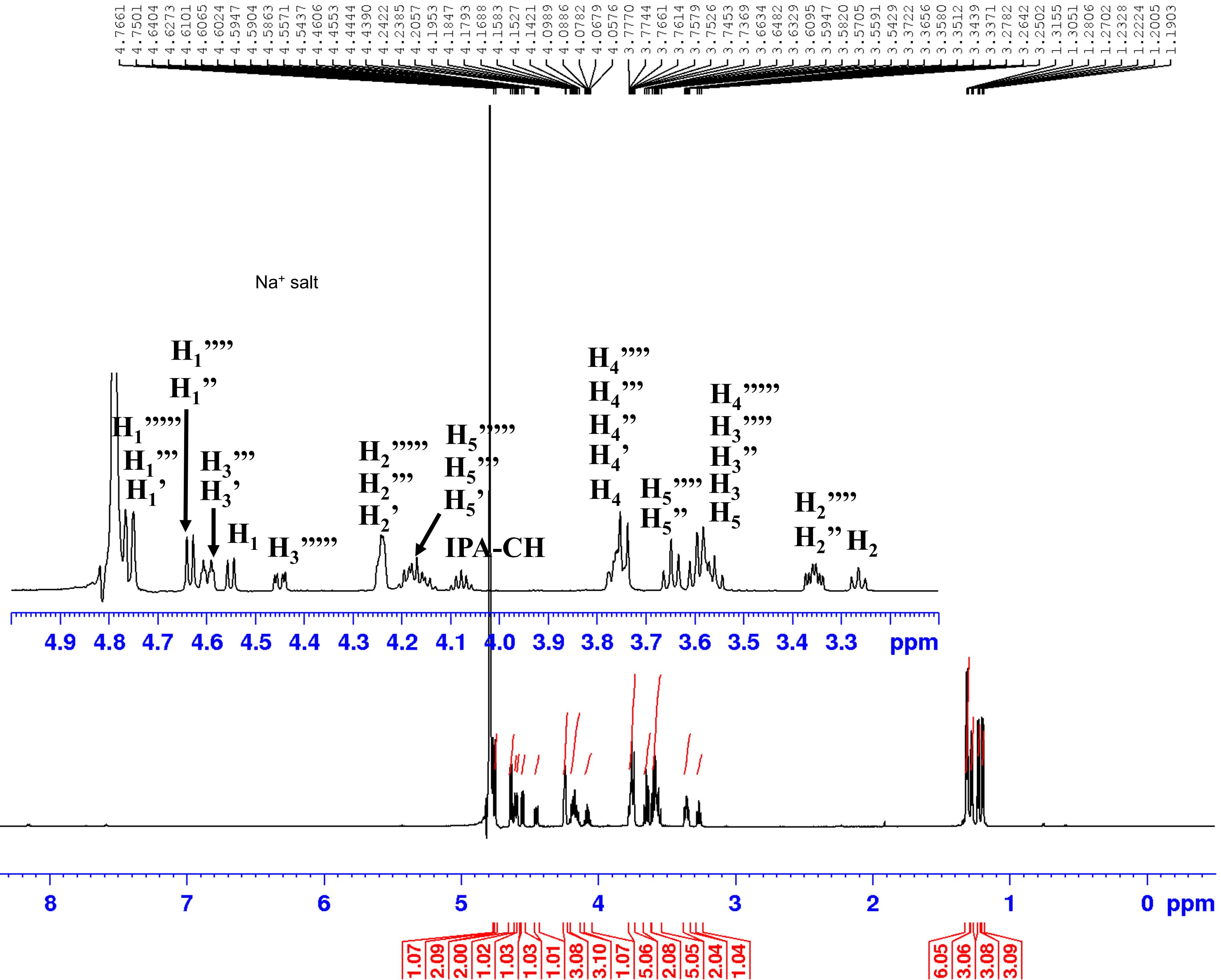

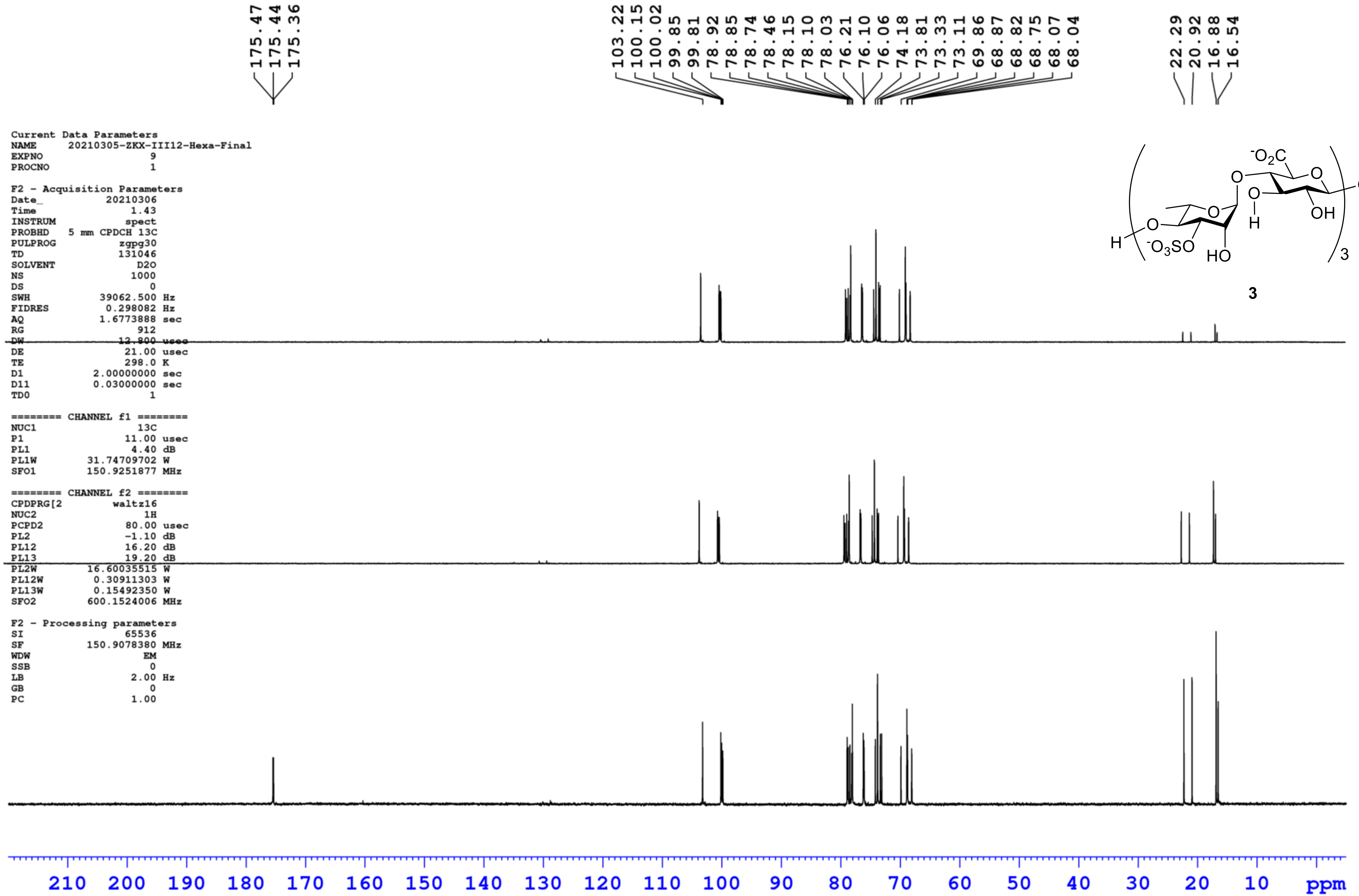
175.47
175.44
175.36
103.22
100.15
100.02
99.85
99.81
78.92
78.85
78.74
78.46
78.15
78.10
78.03
76.21
76.10
76.06
74.18
73.81
73.33
73.11
69.86
68.87
68.82
68.75
68.07
68.04
22.29
20.92
16.88
16.54
Current Data Parameters
NAME 20210305-ZKX-III12-Hexa-Final
EXPNO 9
PROCNO 1
F2 - Acquisition Parameters
Date_ 20210306
Time 1.43
INSTRUM spect
PROBHD 5 mm CPDCH 13C
PULPROG zgpg30
TD 131046
SOLVENT D2O
NS 1000
DS 0
SWH 39062.500 Hz
FIDRES 0.298082 Hz
AQ 1.6773888 sec
RG 912
DW 12.800 usec
DE 21.00 usec
TE 298.0 K
D1 2.00000000 sec
D11 0.03000000 sec
TD0 1
======== CHANNEL f1 ========
NUC1 13C
P1 11.00 usec
PL1 4.40 dB
PL1W 31.74709702 W
SFO1 150.9251877 MHz
======== CHANNEL f2 ========
CPDPRG[2 waltz16
NUC2 1H
PCPD2 80.00 usec
PL2 -1.10 dB
PL12 16.20 dB
PL13 19.20 dB
PL2W 16.60035515 W
PL12W 0.30911303 W
PL13W 0.15492350 W
SFO2 600.1524006 MHz
F2 - Processing parameters
SI 65536
SF 150.9078380 MHz
WDW EM
SSB 0
LB 2.00 Hz
GB 0
PC 1.00
-O2C
O
O
OiPr
O
H
OH
O
H
-O3SO
HO
3
3
210 200 190 180 170 160 150 140 130 120 110 100 90 80 70 60 50 40 30 20 10 ppm

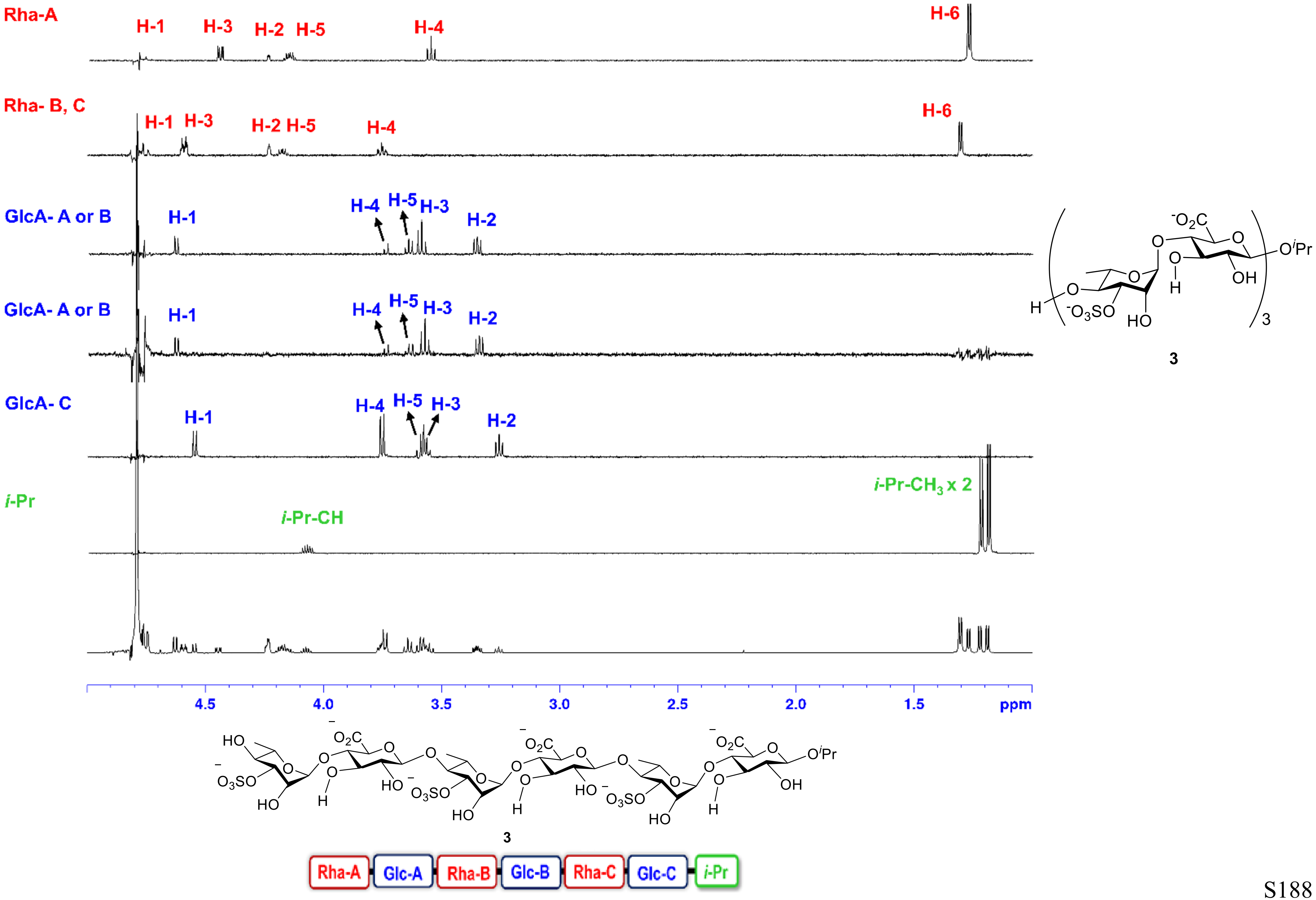

Rha-A
H-1
H-3
H-2
H-5
H-4
H-6
Rha- B, C
H-1
H-3
H-2 H-5
H-4
H-6
GlcA- A or B
H-1
H-4
H-5
H-3
H-2
GlcA- A or B
H-1
H-4
H-5
H-3
H-2
GlcA- C
H-1
H-4
H-5
H-3
H-2
i-Pr
i-Pr-CH
i-Pr-CH3 x 2
4.5
4.0
3.5
3.0
2.5
2.0
1.5
ppm
3
3
Rha-A
Glc-A
Rha-B
Glc-B
Rha-C
Glc-C
i-Pr

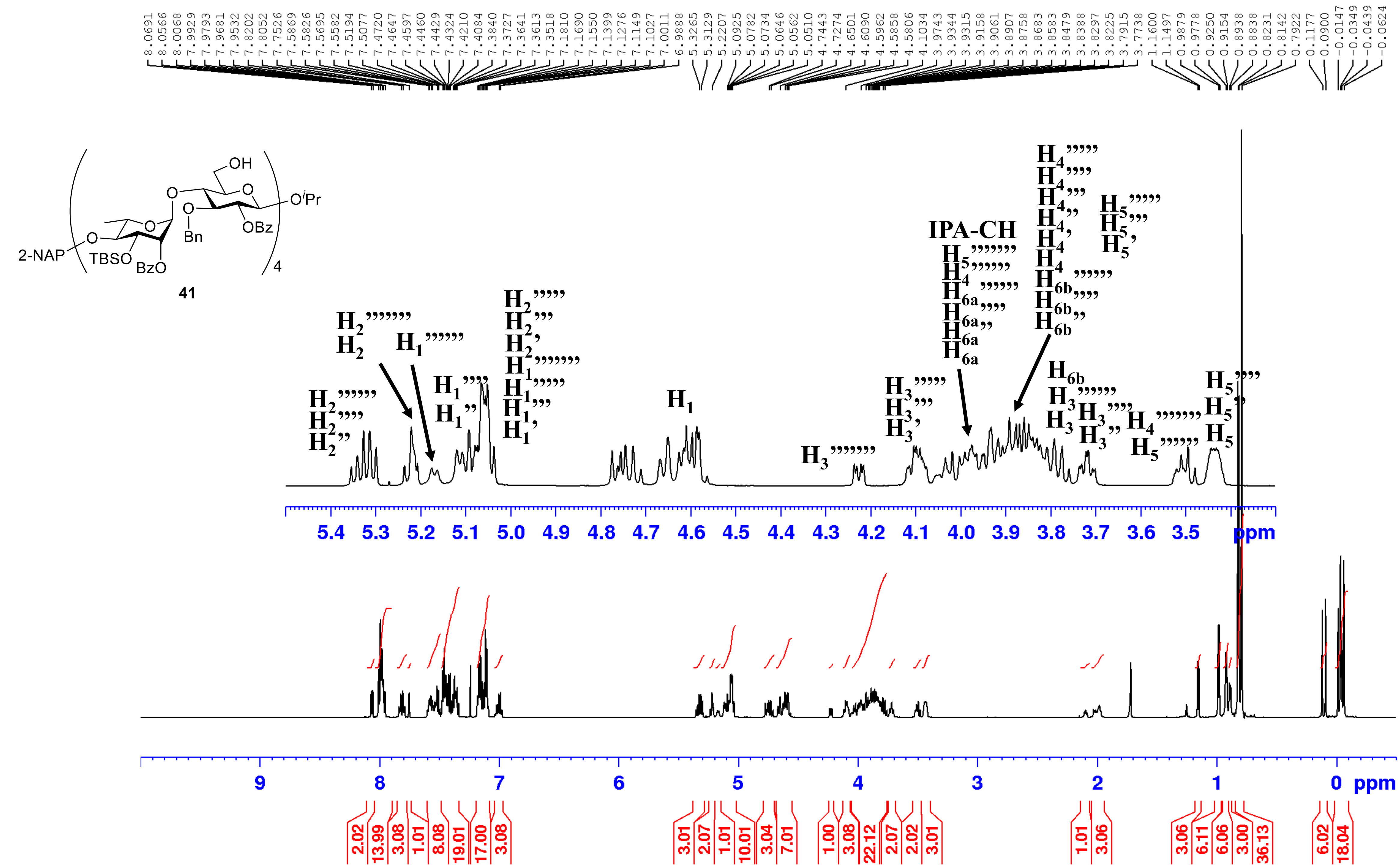
OH
O
O
O
O
OⁱPr
2-NAP
TBSO
BzO
Bn
OBz
4
41
IPA-CH
ppm
9
8
7
6
5
4
3
2
1
0
ppm

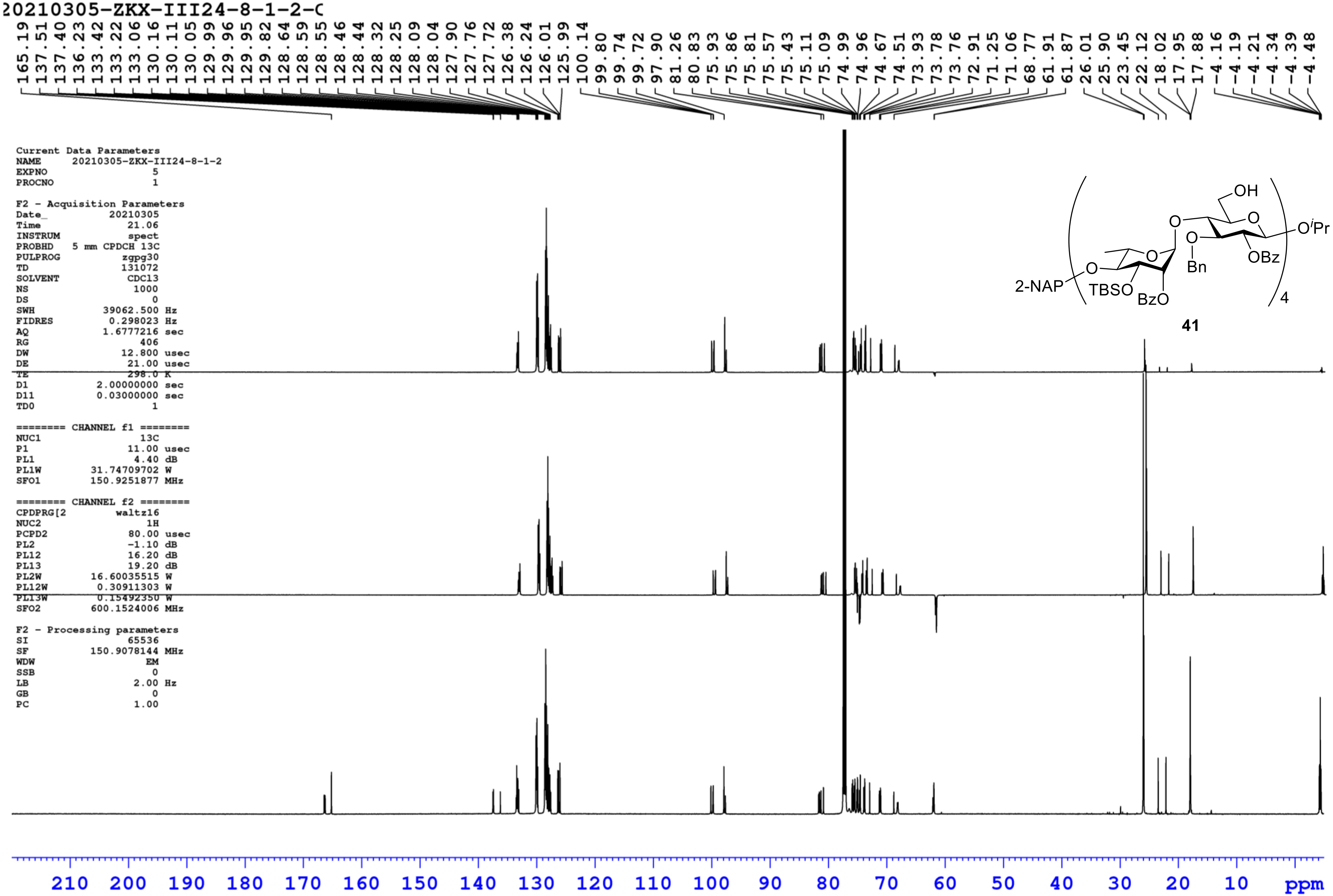
20210305-ZKX-III24-8-1-2-C
165.19
137.51
137.40
136.23
133.42
133.22
133.06
130.16
130.11
130.05
129.99
129.96
129.95
129.82
128.64
128.59
128.55
128.46
128.44
128.32
128.25
128.09
128.04
127.90
127.76
127.72
126.38
126.24
126.01
125.99
100.14
99.80
99.74
99.72
97.90
81.26
80.83
75.93
75.86
75.81
75.57
75.43
75.11
75.09
74.99
74.96
74.67
74.51
73.93
73.78
73.76
72.91
71.25
71.06
68.77
61.91
61.87
26.01
25.90
23.45
22.12
18.02
17.95
17.88
-4.16
-4.19
-4.21
-4.34
-4.39
-4.48
Current Data Parameters
NAME 20210305-ZKX-III24-8-1-2
EXPNO 5
PROCNO 1
F2 - Acquisition Parameters
Date_ 20210305
Time 21.06
INSTRUM spect
PROBHD 5 mm CPDCH 13C
PULPROG zgpg30
TD 131072
SOLVENT CDCl3
NS 1000
DS 0
SWH 39062.500 Hz
FIDRES 0.298023 Hz
AQ 1.6777216 sec
RG 406
DW 12.800 usec
DE 21.00 usec
TE 298.0 K
D1 2.00000000 sec
D11 0.03000000 sec
TD0 1
======== CHANNEL f1 ========
NUC1 13C
P1 11.00 usec
PL1 4.40 dB
PL1W 31.74709702 W
SFO1 150.9251877 MHz
======== CHANNEL f2 ========
CPDPRG[2 waltz16
NUC2 1H
PCPD2 80.00 usec
PL2 -1.10 dB
PL12 16.20 dB
PL13 19.20 dB
PL2W 16.60035515 W
PL12W 0.30911303 W
PL13W 0.15492350 W
SFO2 600.1524006 MHz
F2 - Processing parameters
SI 65536
SF 150.9078144 MHz
WDW EM
SSB 0
LB 2.00 Hz
GB 0
PC 1.00
OH
O
O
O
O^{i}Pr
O
Bn
OBz
2-NAP
O
TBSO
BzO
4
41
210 200 190 180 170 160 150 140 130 120 110 100 90 80 70 60 50 40 30 20 10 ppm

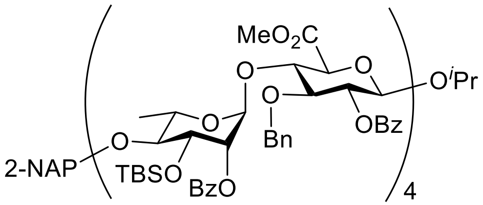
MeO2C
O^iPr
OBz
Bn
2-NAP
TBSO
BzO
4

45

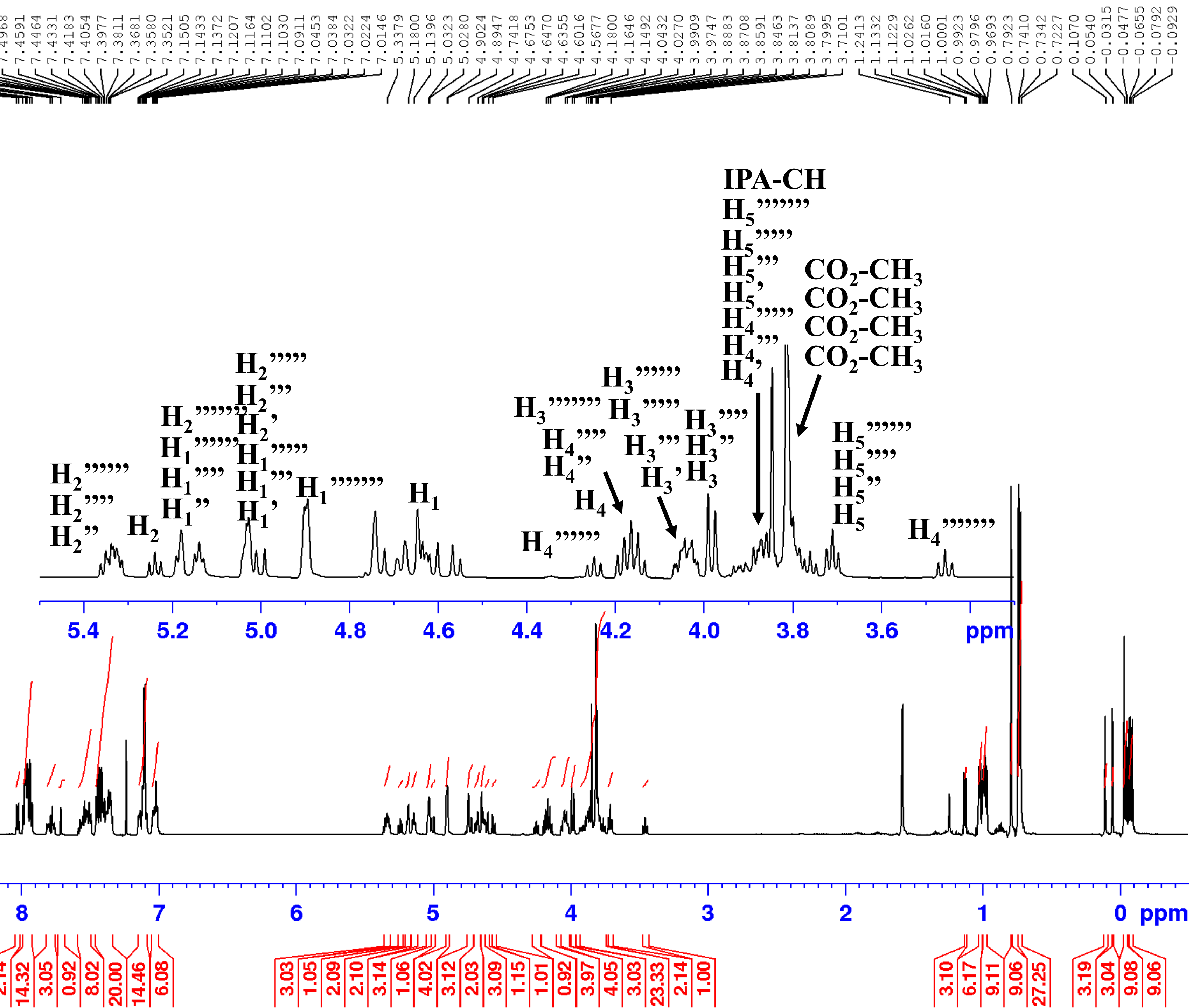
IPA-CH
CO2-CH3
CO2-CH3
CO2-CH3
CO2-CH3
H1
H2
H4
H5
ppm
5.4
5.2
5.0
4.8
4.6
4.4
4.2
4.0
3.8
3.6
9
8
7
6
5
4
3
2
1
0 ppm

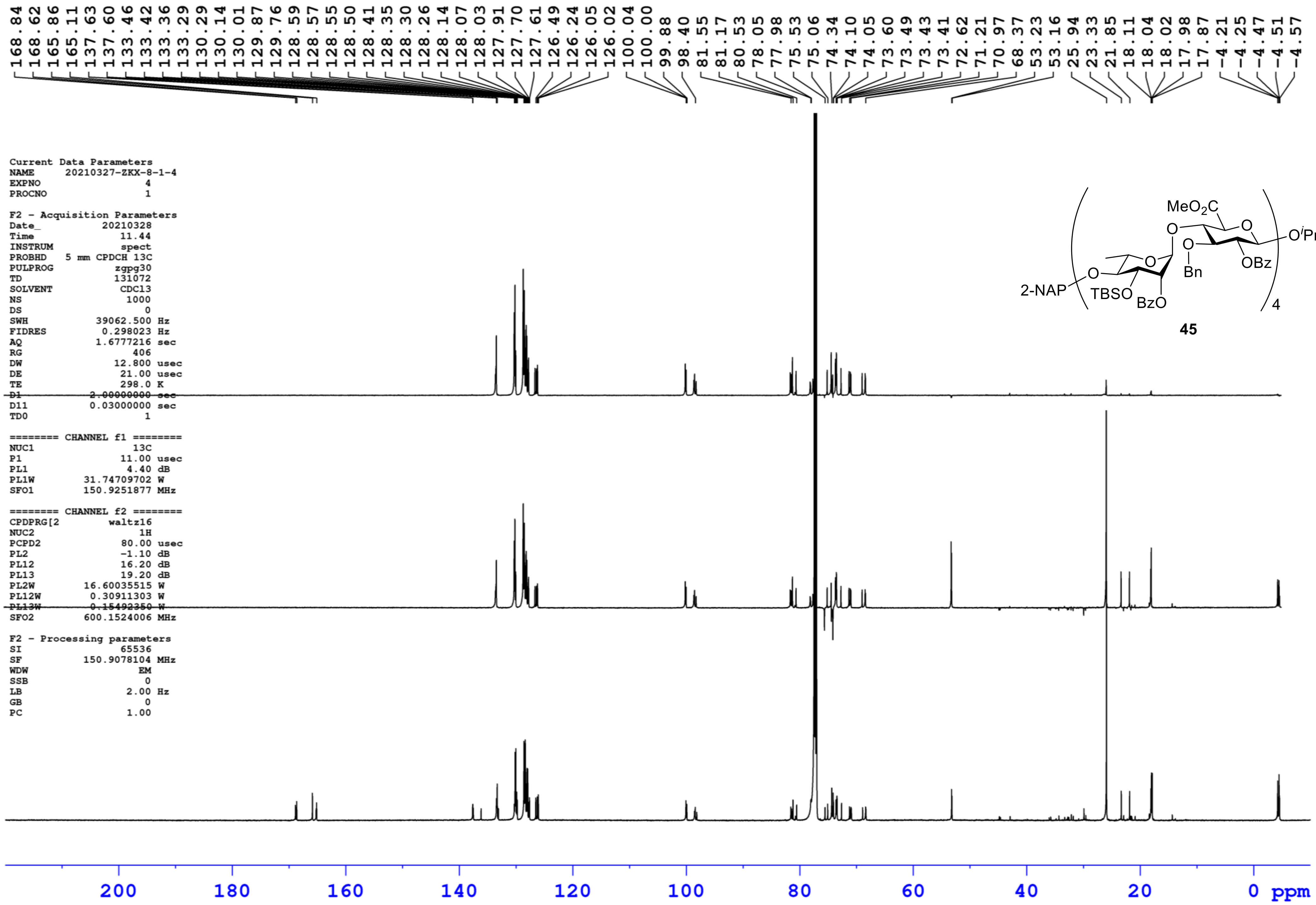
168.84
168.62
165.86
165.11
137.63
137.60
133.46
133.42
133.36
133.29
130.29
130.14
130.01
129.87
129.76
128.59
128.57
128.55
128.50
128.41
128.35
128.30
128.26
128.14
128.07
128.03
127.91
127.70
127.61
126.49
126.24
126.05
126.02
100.04
100.00
99.88
98.40
81.55
81.17
80.53
78.05
77.98
75.53
75.06
74.34
74.10
74.05
73.60
73.49
73.43
73.41
72.62
71.21
70.97
68.37
53.23
53.16
25.94
23.33
21.85
18.11
18.04
18.02
17.98
17.87
-4.21
-4.25
-4.47
-4.51
-4.57
Current Data Parameters
NAME 20210327-ZKX-8-1-4
EXPNO 4
PROCNO 1
F2 - Acquisition Parameters
Date_ 20210328
Time 11.44
INSTRUM spect
PROBHD 5 mm CPDCH 13C
PULPROG zgpg30
TD 131072
SOLVENT CDCl3
NS 1000
DS 0
SWH 39062.500 Hz
FIDRES 0.298023 Hz
AQ 1.6777216 sec
RG 406
DW 12.800 usec
DE 21.00 usec
TE 298.0 K
D1 2.00000000 sec
D11 0.03000000 sec
TD0 1
======== CHANNEL f1 ========
NUC1 13C
P1 11.00 usec
PL1 4.40 dB
PL1W 31.74709702 W
SFO1 150.9251877 MHz
======== CHANNEL f2 ========
CPDPRG[2 waltz16
NUC2 1H
PCPD2 80.00 usec
PL2 -1.10 dB
PL12 16.20 dB
PL13 19.20 dB
PL2W 16.60035515 W
PL12W 0.30911303 W
PL13W 0.15492350 W
SFO2 600.1524006 MHz
F2 - Processing parameters
SI 65536
SF 150.9078104 MHz
WDW EM
SSB 0
LB 2.00 Hz
GB 0
PC 1.00
MeO2C
O
O
OiPr
O
Bn
OBz
2-NAP
O
TBSO
BzO
4
45
200
180
160
140
120
100
80
60
40
20
0 ppm

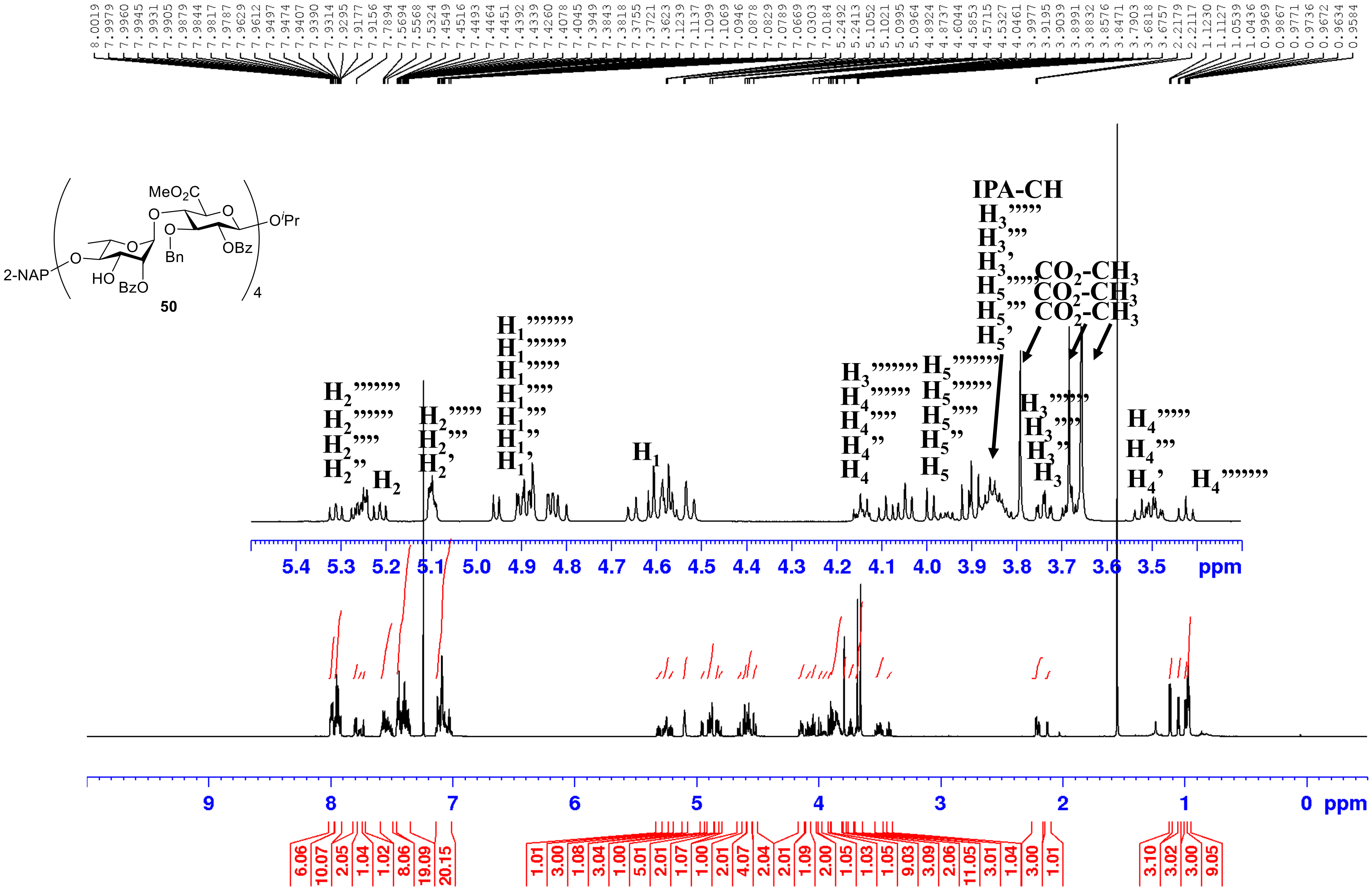

8.0019 7.9979 7.9960 7.9945 7.9931 7.9905 7.9879 7.9844 7.9817 7.9787 7.9629 7.9612 7.9497 7.9474 7.9407 7.9390 7.9314 7.9295 7.9177 7.9156 7.7894 7.5694 7.5568 7.5324 7.4549 7.4516 7.4493 7.4464 7.4451 7.4392 7.4339 7.4260 7.4078 7.4045 7.3949 7.3843 7.3818 7.3755 7.3721 7.3623 7.1239 7.1137 7.1099 7.1069 7.0946 7.0878 7.0829 7.0789 7.0669 7.0303 7.0184 5.2492 5.2413 5.1052 5.1021 5.0995 5.0964 4.8924 4.8737 4.6044 4.5853 4.5715 4.5327 4.0461 3.9977 3.9195 3.9039 3.8991 3.8832 3.8576 3.8471 3.7903 3.6818 3.6757 2.2179 2.2117 1.1230 1.1127 1.0539 1.0436 0.9969 0.9867 0.9771 0.9736 0.9672 0.9634 0.9584
MeO2C
O
O
OiPr
2-NAP
O
HO
BzO
Bn
OBz
4
50
IPA-CH
H3''''', H3''', H3', H5''''', H5''', H5'
CO2-CH3
CO2-CH3
CO2-CH3
H2''''''', H2'''''', H2'''', H2''
H2
H2''''', H2''', H2'
H1''''''', H1'''''', H1''''', H1'''', H1''', H1'', H1', H1
H1
H3''''''', H4'''''', H4'''', H4'', H4
H5''''''', H5'''''', H5'''', H5'', H5
H3''''', H3'''', H3'', H3
H4''''', H4''', H4'
H4''''''
5.4 5.3 5.2 5.1 5.0 4.9 4.8 4.7 4.6 4.5 4.4 4.3 4.2 4.1 4.0 3.9 3.8 3.7 3.6 3.5 ppm
9 8 7 6 5 4 3 2 1 0 ppm
6.06 10.07 2.05 1.04 1.02 8.06 19.09 20.15 1.01 3.00 1.08 3.04 1.00 5.01 2.01 1.07 1.00 2.01 4.07 2.04 2.01 1.09 2.00 1.05 1.03 1.05 9.03 3.09 2.06 11.05 3.01 1.04 3.00 1.01 3.10 3.02 3.00 9.05

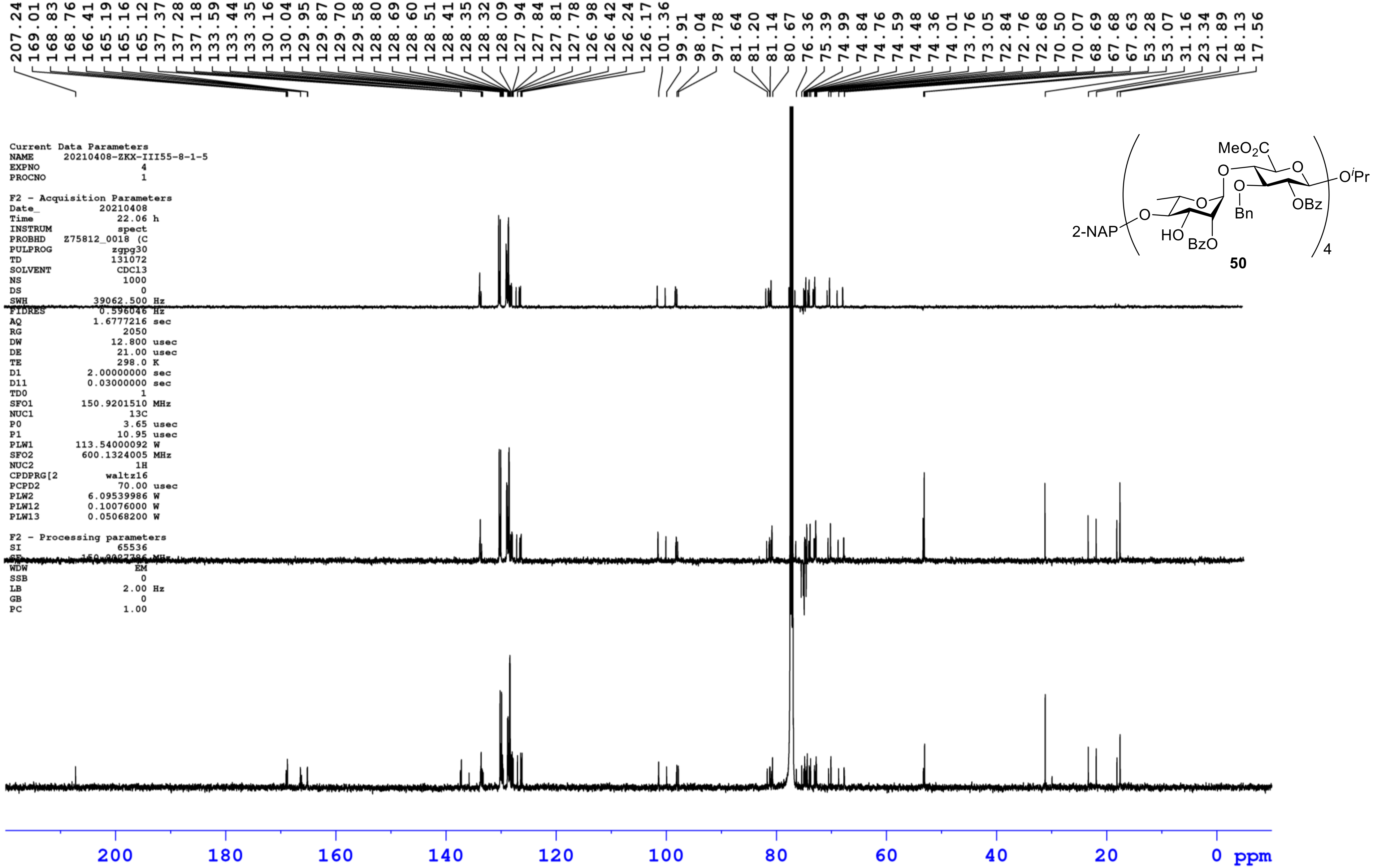
207.24 169.01 168.83 168.76 166.41 165.19 165.16 165.12 137.37 137.28 137.18 133.59 133.44 133.35 130.16 130.04 129.95 129.87 129.70 129.58 128.80 128.69 128.60 128.51 128.41 128.35 128.32 128.09 127.94 127.84 127.81 127.78 126.98 126.42 126.24 126.17 101.36 99.91 98.04 97.78 81.64 81.20 81.14 80.67 76.36 75.39 74.99 74.84 74.76 74.59 74.48 74.36 74.01 73.76 73.05 72.84 72.76 72.68 70.50 70.07 68.69 67.68 67.63 53.28 53.07 31.16 23.34 21.89 18.13 17.56
Current Data Parameters
NAME 20210408-ZKX-III55-8-1-5
EXPNO 4
PROCNO 1
F2 - Acquisition Parameters
Date_ 20210408
Time 22.06 h
INSTRUM spect
PROBHD Z75812_0018 (C
PULPROG zgpg30
TD 131072
SOLVENT CDCl3
NS 1000
DS 0
SWH 39062.500 Hz
FIDRES 0.596046 Hz
AQ 1.6777216 sec
RG 2050
DW 12.800 usec
DE 21.00 usec
TE 298.0 K
D1 2.00000000 sec
D11 0.03000000 sec
TD0 1
SFO1 150.9201510 MHz
NUC1 13C
P0 3.65 usec
P1 10.95 usec
PLW1 113.54000092 W
SFO2 600.1324005 MHz
NUC2 1H
CPDPRG[2 waltz16
PCPD2 70.00 usec
PLW2 6.09539986 W
PLW12 0.10076000 W
PLW13 0.05068200 W
F2 - Processing parameters
SI 65536
SF 150.9027786 MHz
WDW EM
SSB 0
LB 2.00 Hz
GB 0
PC 1.00
200 180 160 140 120 100 80 60 40 20 0 ppm
MeO2C
O
O
OiPr
O
Bn
OBz
2-NAP
O
HO
BzO
4

50

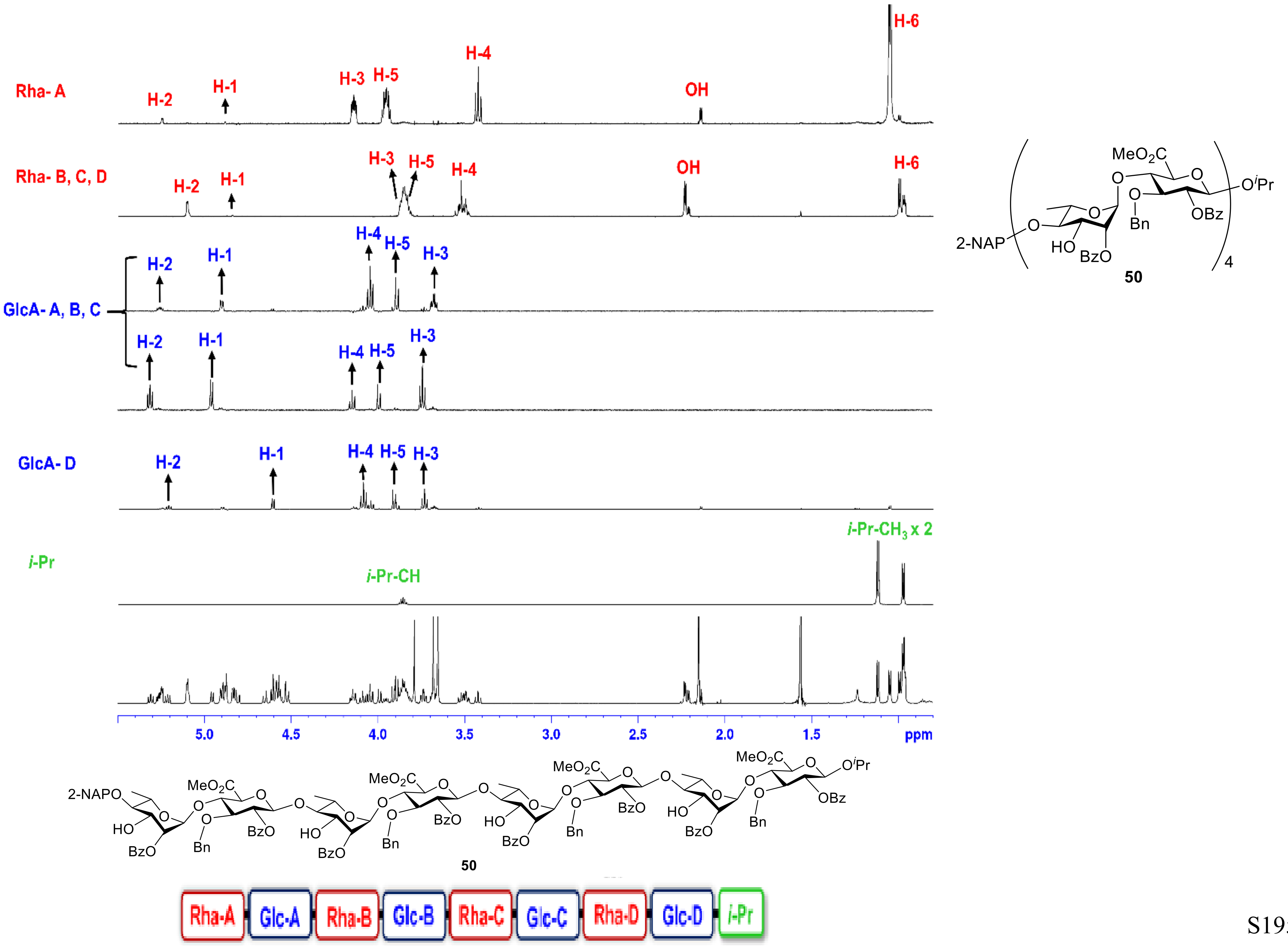

Rha- A
H-2
H-1
H-3
H-5
H-4
OH
H-6
Rha- B, C, D
H-2
H-1
H-3
H-5
H-4
OH
H-6
GlcA- A, B, C
H-2
H-1
H-4
H-5
H-3
H-2
H-1
H-4
H-5
H-3
GlcA- D
H-2
H-1
H-4
H-5
H-3
i-Pr
i-Pr-CH
i-Pr-CH$_3$ x 2
5.0
4.5
4.0
3.5
3.0
2.5
2.0
1.5
ppm
MeO_2C
O^{i}Pr
2-NAP
HO
BzO
Bn
OBz
4
50
Rha-A
Glc-A
Rha-B
Glc-B
Rha-C
Glc-C
Rha-D
Glc-D
i-Pr

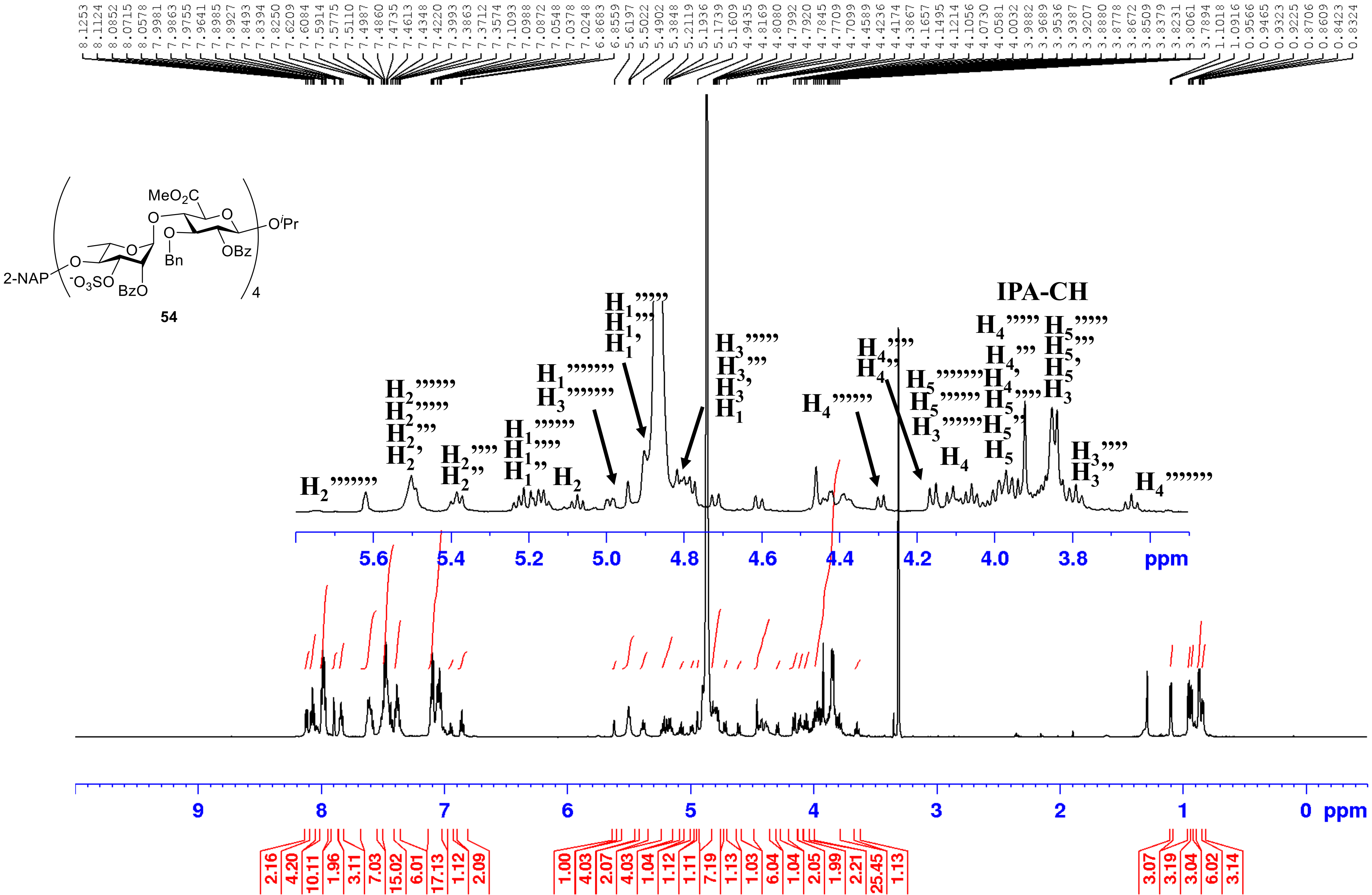

54

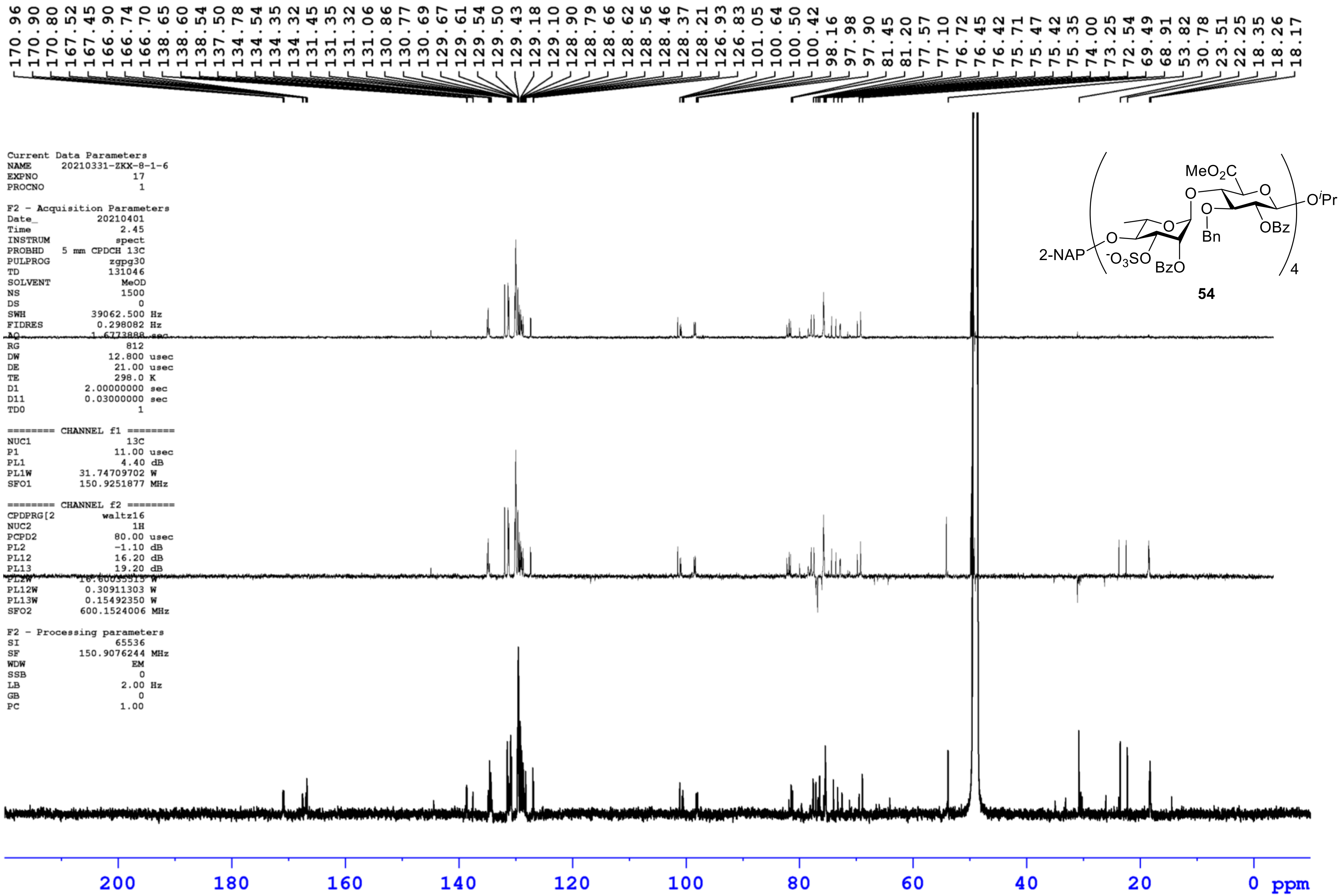
170.96
170.90
170.80
167.52
167.45
166.90
166.74
166.70
138.65
138.60
138.54
137.50
134.78
134.54
134.35
134.32
131.45
131.35
131.32
131.06
130.86
130.77
130.69
129.67
129.61
129.54
129.50
129.43
129.18
129.10
128.90
128.79
128.66
128.62
128.56
128.46
128.37
128.21
126.93
126.83
101.05
100.64
100.50
100.42
98.16
97.98
97.90
81.45
81.20
77.57
77.10
76.72
76.45
76.42
75.71
75.47
75.42
75.35
74.00
73.25
72.54
69.49
68.91
53.82
30.78
23.51
22.25
18.35
18.26
18.17
Current Data Parameters
NAME 20210331-ZKX-8-1-6
EXPNO 17
PROCNO 1
F2 - Acquisition Parameters
Date_ 20210401
Time 2.45
INSTRUM spect
PROBHD 5 mm CPDCH 13C
PULPROG zgpg30
TD 131046
SOLVENT MeOD
NS 1500
DS 0
SWH 39062.500 Hz
FIDRES 0.298082 Hz
AQ 1.6773888 sec
RG 812
DW 12.800 usec
DE 21.00 usec
TE 298.0 K
D1 2.00000000 sec
D11 0.03000000 sec
TD0 1
======== CHANNEL f1 ========
NUC1 13C
P1 11.00 usec
PL1 4.40 dB
PL1W 31.74709702 W
SFO1 150.9251877 MHz
======== CHANNEL f2 ========
CPDPRG[2 waltz16
NUC2 1H
PCPD2 80.00 usec
PL2 -1.10 dB
PL12 16.20 dB
PL13 19.20 dB
PL2W 16.60035515 W
PL12W 0.30911303 W
PL13W 0.15492350 W
SFO2 600.1524006 MHz
F2 - Processing parameters
SI 65536
SF 150.9076244 MHz
WDW EM
SSB 0
LB 2.00 Hz
GB 0
PC 1.00
200
180
160
140
120
100
80
60
40
20
0 ppm
MeO2C
O
O
O
OiPr
O
Bn
OBz
2-NAP
O
-O3SO
BzO
4

54

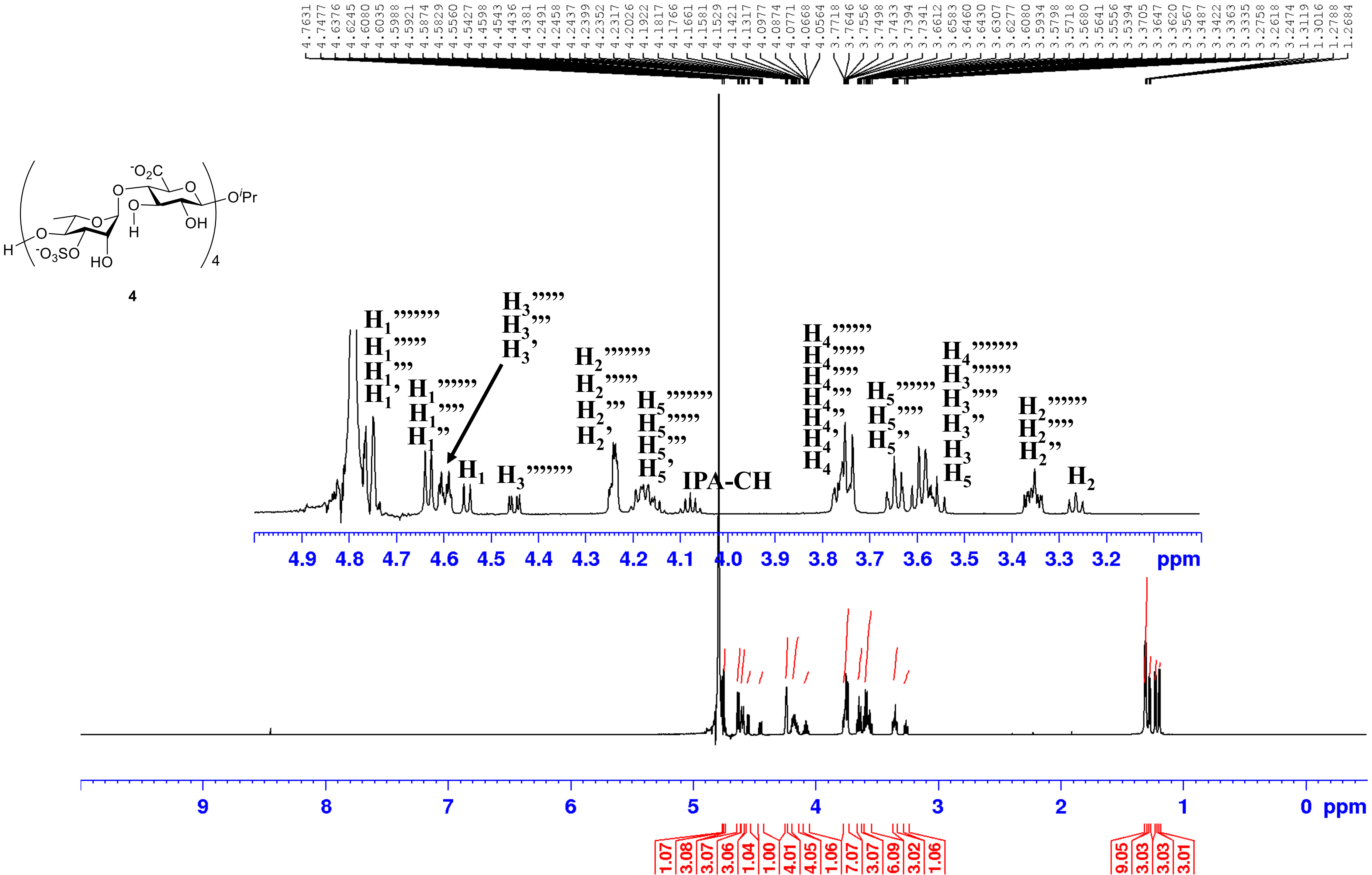

4

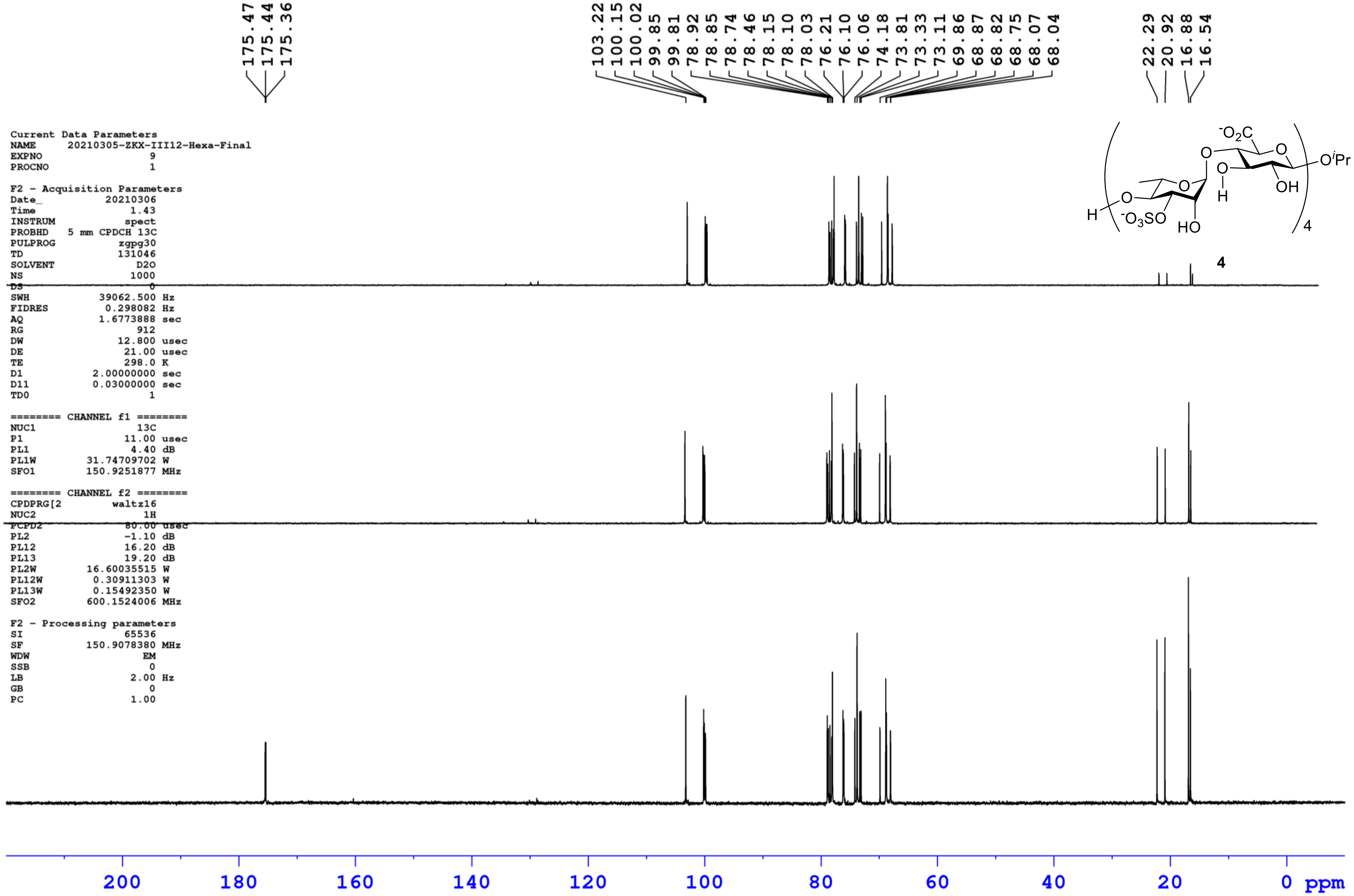
175.47
175.44
175.36
103.22
100.15
100.02
99.85
99.81
78.92
78.85
78.74
78.46
78.15
78.10
78.03
76.21
76.10
76.06
74.18
73.81
73.33
73.11
69.86
68.87
68.82
68.75
68.07
68.04
22.29
20.92
16.88
16.54
Current Data Parameters
NAME 20210305-ZKX-III12-Hexa-Final
EXPNO 9
PROCNO 1
F2 - Acquisition Parameters
Date_ 20210306
Time 1.43
INSTRUM spect
PROBHD 5 mm CPDCH 13C
PULPROG zgpg30
TD 131046
SOLVENT D2O
NS 1000
DS 0
SWH 39062.500 Hz
FIDRES 0.298082 Hz
AQ 1.6773888 sec
RG 912
DW 12.800 usec
DE 21.00 usec
TE 298.0 K
D1 2.00000000 sec
D11 0.03000000 sec
TD0 1
======== CHANNEL f1 ========
NUC1 13C
P1 11.00 usec
PL1 4.40 dB
PL1W 31.74709702 W
SFO1 150.9251877 MHz
======== CHANNEL f2 ========
CPDPRG[2 waltz16
NUC2 1H
PCPD2 80.00 usec
PL2 -1.10 dB
PL12 16.20 dB
PL13 19.20 dB
PL2W 16.60035515 W
PL12W 0.30911303 W
PL13W 0.15492350 W
SFO2 600.1524006 MHz
F2 - Processing parameters
SI 65536
SF 150.9078380 MHz
WDW EM
SSB 0
LB 2.00 Hz
GB 0
PC 1.00
⁻O2C
O
O
OⁱPr
O
H
OH
O
H
O
⁻O3SO
HO
4
4
200
180
160
140
120
100
80
60
40
20
0 ppm

8. NMR Spectra for the Synthesis of $B_{3'S}$ Libraries

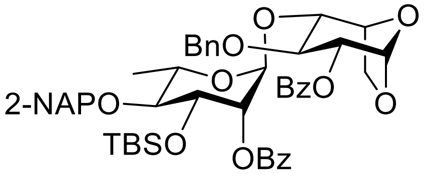

BnO
O
O
2-NAPO
O
BzO
O
TBSO
OBz

55

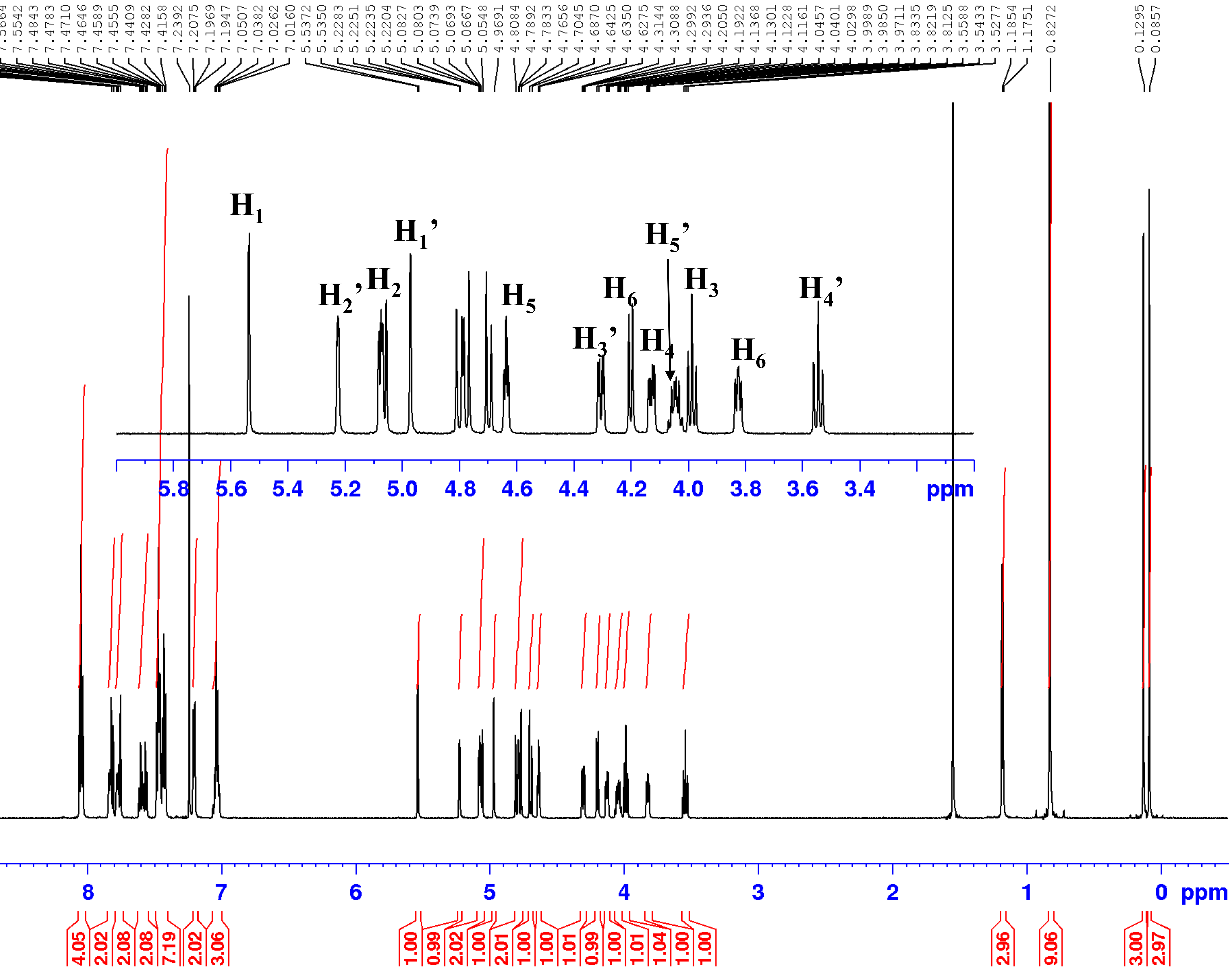

7.8211 7.8065 7.7824 7.7761 7.7668 7.7520 7.6129 7.6004 7.5881 7.5790 7.5664 7.5542 7.4843 7.4783 7.4710 7.4646 7.4589 7.4555 7.4409 7.4282 7.4158 7.2392 7.2075 7.1969 7.1947 7.0507 7.0382 7.0262 7.0160 5.5372 5.5350 5.2283 5.2251 5.2235 5.2204 5.0827 5.0803 5.0739 5.0693 5.0667 5.0548 4.9691 4.8084 4.7892 4.7833 4.7656 4.7045 4.6870 4.6425 4.6350 4.6275 4.3144 4.3088 4.2992 4.2936 4.2050 4.1922 4.1368 4.1301 4.1228 4.1161 4.0457 4.0401 4.0298 3.9989 3.9850 3.9711 3.8335 3.8219 3.8125 3.5588 3.5433 3.5277 1.1854 1.1751 0.8272 0.1295 0.0857
H_1
H_1'
H_2'
H_2
H_5
H_5'
H_6
H_3
H_3'
H_4
H_6
H_4'
5.8 5.6 5.4 5.2 5.0 4.8 4.6 4.4 4.2 4.0 3.8 3.6 3.4 ppm
9 8 7 6 5 4 3 2 1 0 ppm
4.05 2.02 2.08 2.08 7.19 2.02 3.06 1.00 0.99 2.02 1.00 2.01 1.00 1.00 1.01 0.99 1.00 1.01 1.04 1.00 1.00 2.96 9.06 3.00 2.97

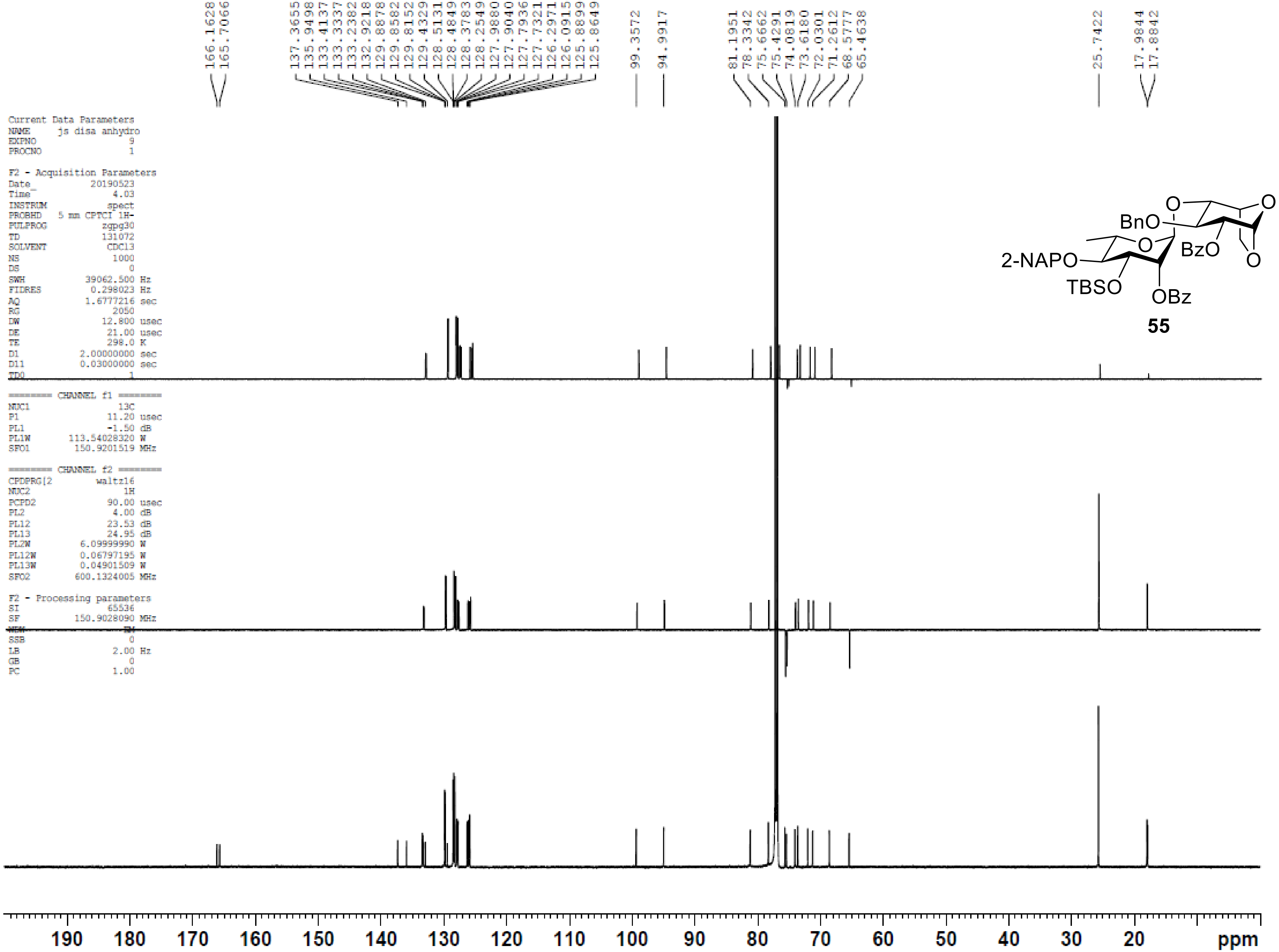
166.1628
165.7066
137.3655
135.9498
133.4137
133.3337
133.2382
132.9218
129.8878
129.8582
129.8152
129.4329
128.5131
128.4849
128.3783
128.2549
127.9880
127.9040
127.7936
127.7321
126.2971
126.0915
125.8899
125.8649
99.3572
94.9917
81.1951
78.3342
75.6662
75.4291
74.0819
73.6180
72.0301
71.2612
68.5777
65.4638
25.7422
17.9844
17.8842
Current Data Parameters
NAME js disa anhydro
EXPNO 9
PROCNO 1
F2 - Acquisition Parameters
Date_ 20190523
Time 4.03
INSTRUM spect
PROBHD 5 mm CPTCI 1H-
PULPROG zgpg30
TD 131072
SOLVENT CDCl3
NS 1000
DS 0
SWH 39062.500 Hz
FIDRES 0.298023 Hz
AQ 1.6777216 sec
RG 2050
DW 12.800 usec
DE 21.00 usec
TE 298.0 K
D1 2.00000000 sec
D11 0.03000000 sec
TD0 1
======== CHANNEL f1 ========
NUC1 13C
P1 11.20 usec
PL1 -1.50 dB
PL1W 113.54028320 W
SFO1 150.9201519 MHz
======== CHANNEL f2 ========
CPDPRG[2 waltz16
NUC2 1H
PCPD2 90.00 usec
PL2 4.00 dB
PL12 23.53 dB
PL13 24.95 dB
PL2W 6.09999990 W
PL12W 0.06797195 W
PL13W 0.04901509 W
SFO2 600.1324005 MHz
F2 - Processing parameters
SI 65536
SF 150.9028090 MHz
SSB 0
LB 2.00 Hz
GB 0
PC 1.00
BnO
BzO
2-NAPO
TBSO
OBz
55
190 180 170 160 150 140 130 120 110 100 90 80 70 60 50 40 30 20 ppm

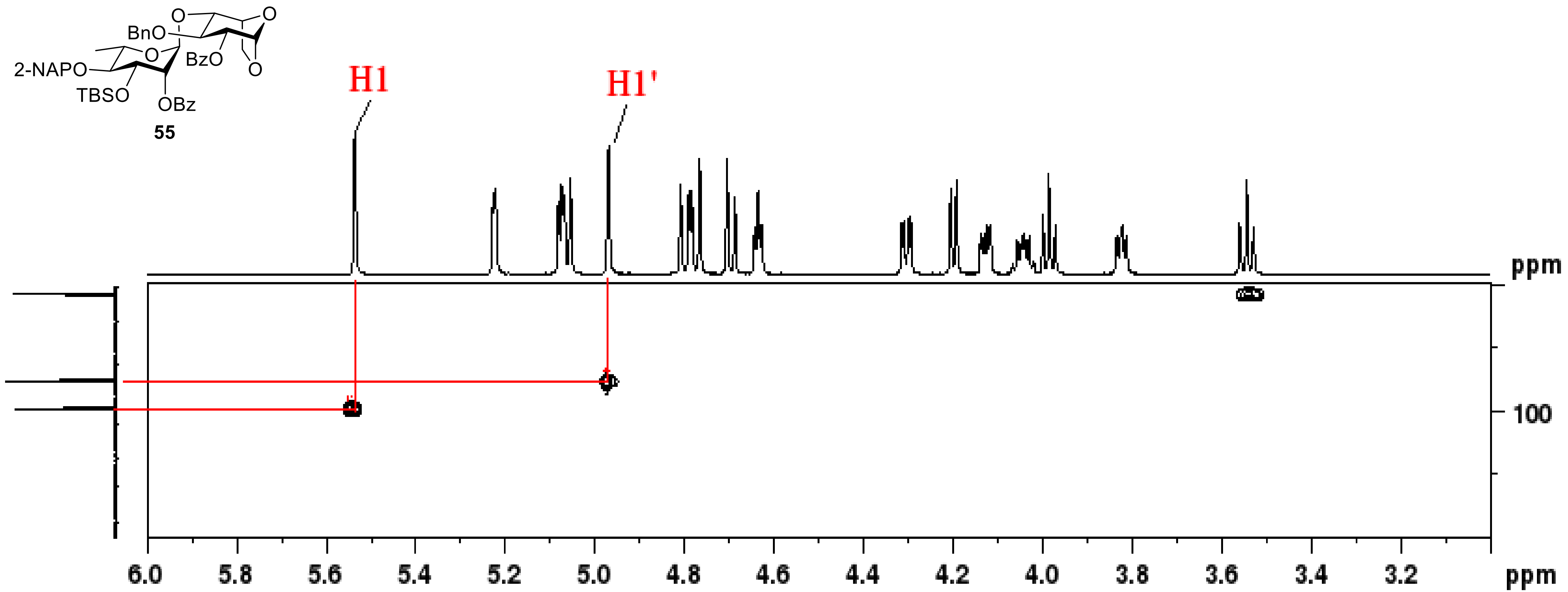
2-NAPO
BnO
BzO
TBSO
OBz
55
H1
H1'
ppm
100
6.0
5.8
5.6
5.4
5.2
5.0
4.8
4.6
4.4
4.2
4.0
3.8
3.6
3.4
3.2
ppm

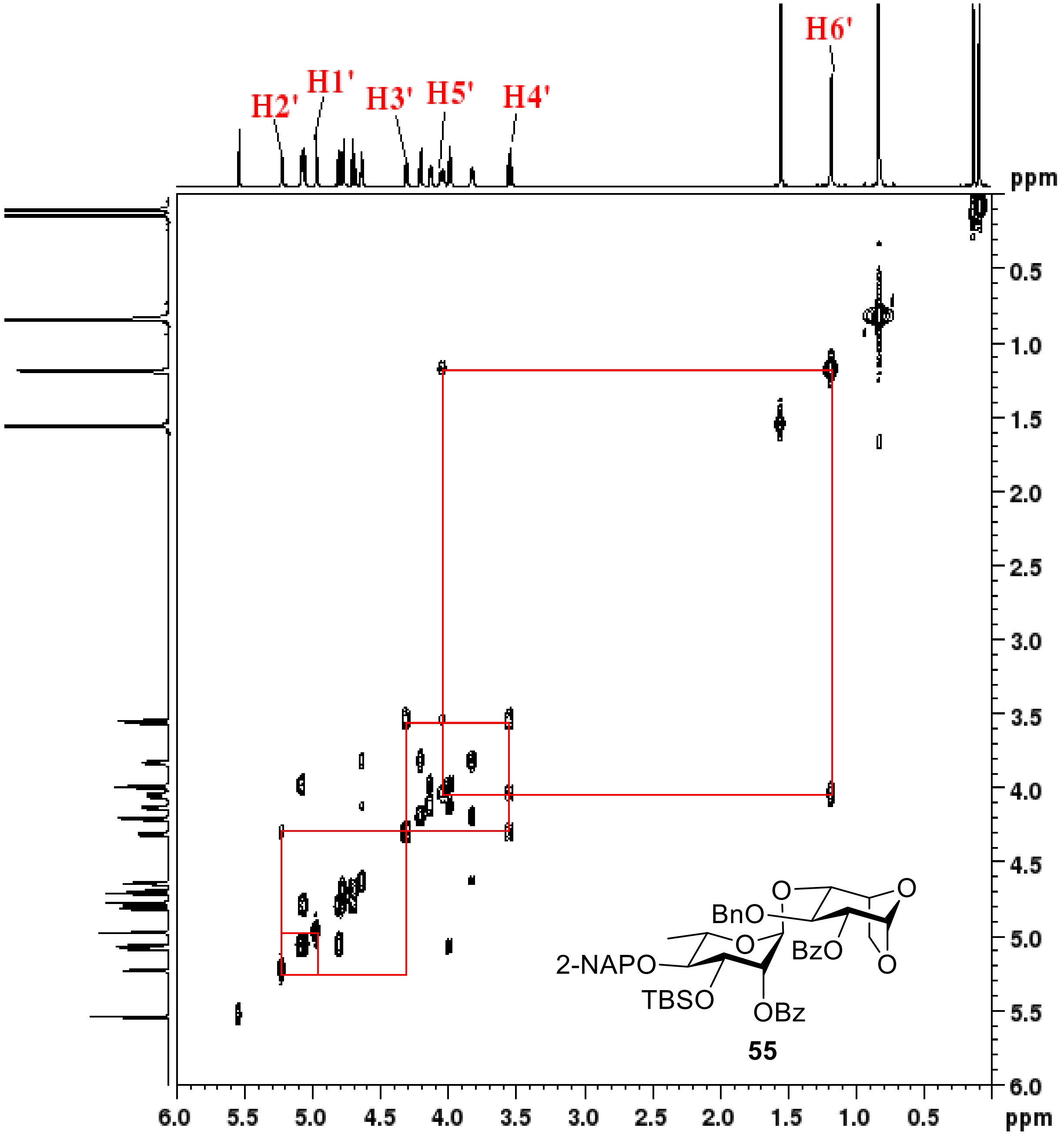
H2'
H1'
H3'
H5'
H4'
H6'
ppm
BnO
2-NAPO
TBSO
BzO
OBz
55

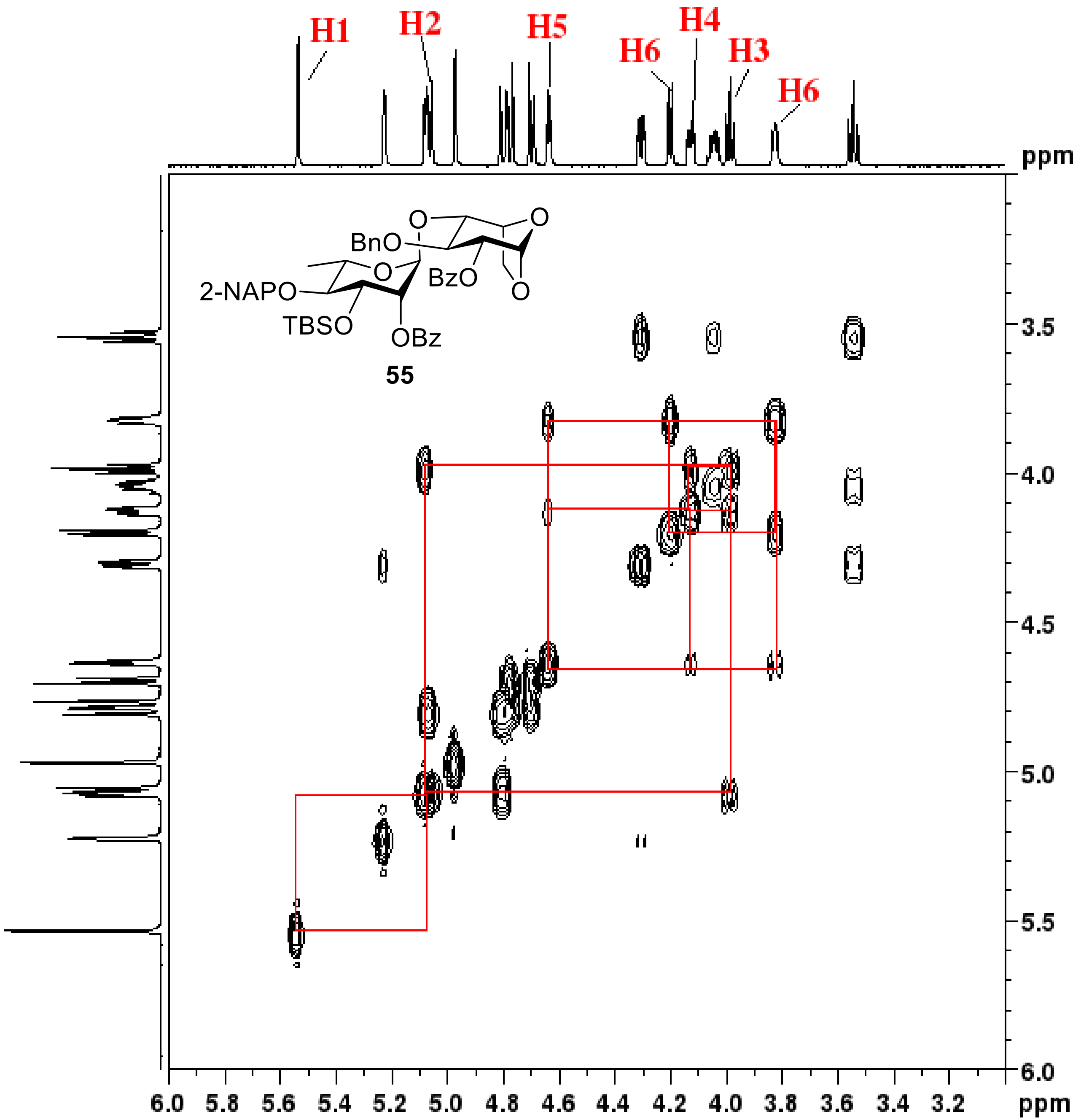
H1
H2
H5
H6
H4
H3
H6
ppm
BnO
2-NAPO
TBSO
BzO
OBz
55

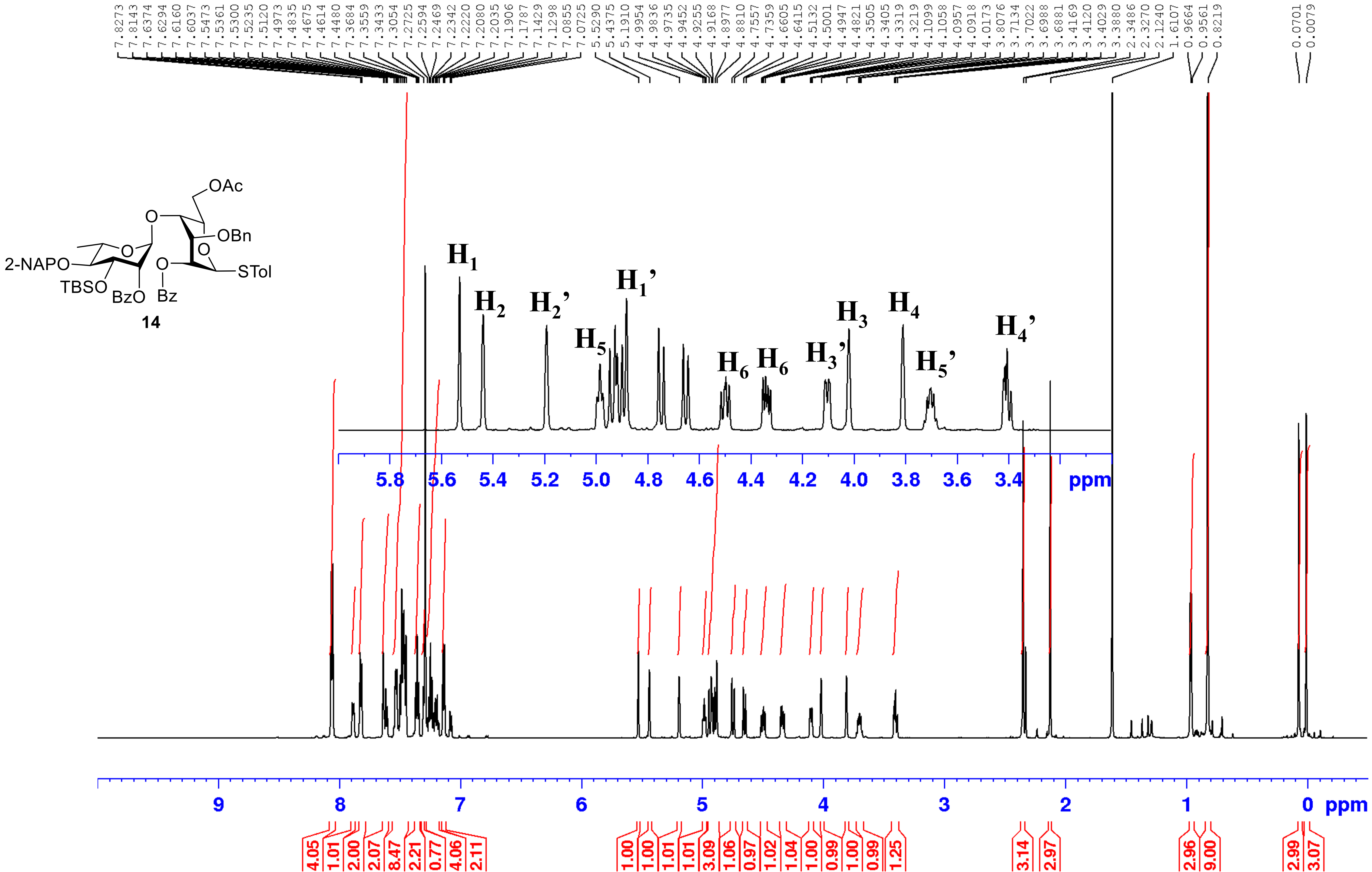
OAc
OBn
2-NAPO
TBSO
BzO
Bz
STol
14
H1
H2
H2'
H5
H1'
H6
H6
H3'
H3
H4
H5'
H4'
ppm

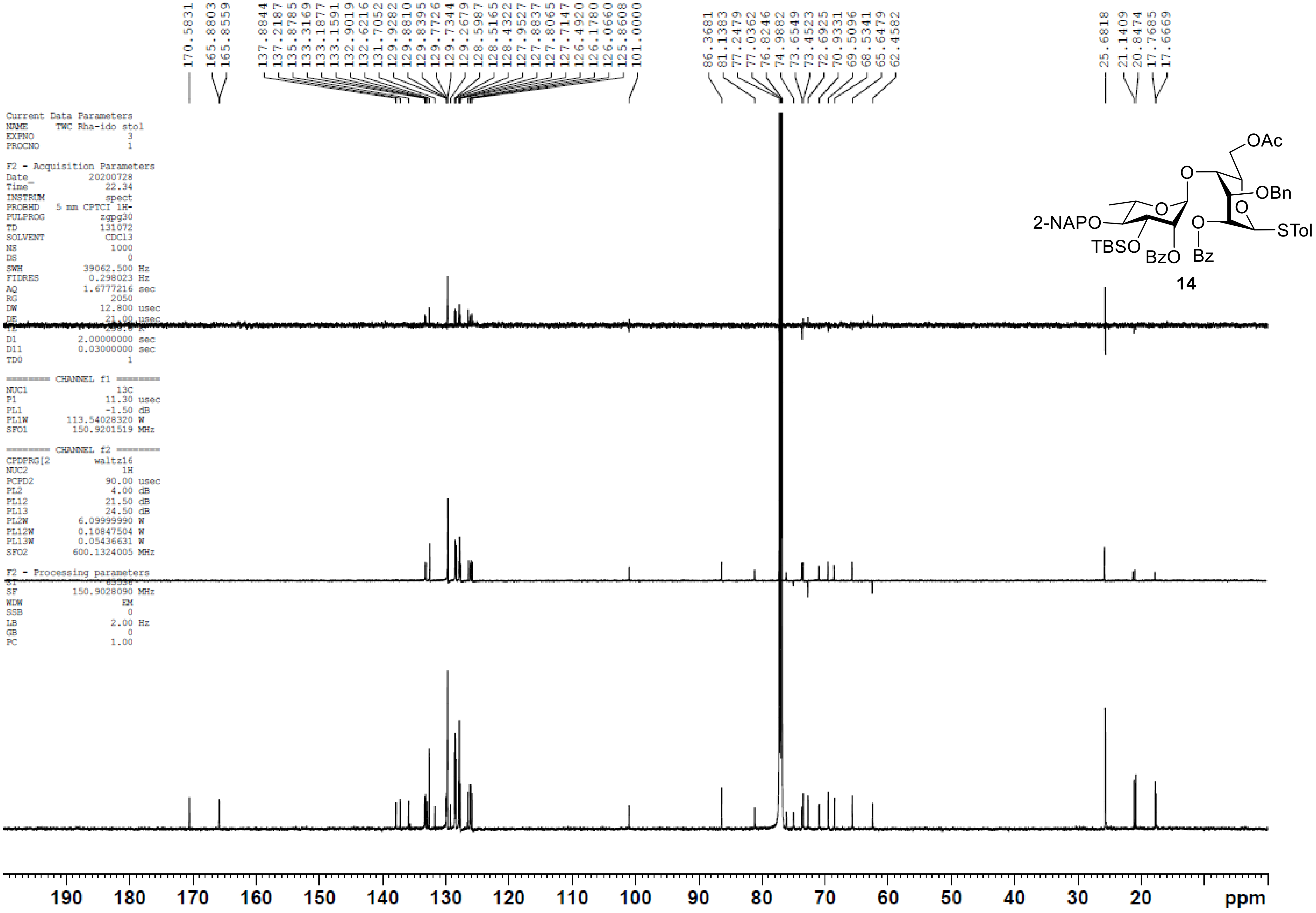
170.5831
165.8803
165.8559
137.8844
137.2187
135.8785
133.3169
133.1877
133.1591
132.9019
132.6216
131.7052
129.9282
129.8810
129.8395
129.7726
129.7344
129.2679
128.5987
128.5165
128.4322
127.9527
127.8837
127.8065
127.7147
126.4920
126.1780
126.0660
125.8608
101.0000
86.3681
81.1383
77.2479
77.0362
76.8246
74.9882
73.6549
73.4523
72.6925
70.9331
69.5096
68.5341
65.6479
62.4582
25.6818
21.1409
20.8474
17.7685
17.6669
Current Data Parameters
NAME TWC Rha-ido stol
EXPNO 3
PROCNO 1
F2 - Acquisition Parameters
Date_ 20200728
Time 22.34
INSTRUM spect
PROBHD 5 mm CPTCI 1H-
PULPROG zgpg30
TD 131072
SOLVENT CDCl3
NS 1000
DS 0
SWH 39062.500 Hz
FIDRES 0.298023 Hz
AQ 1.6777216 sec
RG 2050
DW 12.800 usec
DE 21.00 usec
D1 2.00000000 sec
D11 0.03000000 sec
TD0 1
======== CHANNEL f1 ========
NUC1 13C
P1 11.30 usec
PL1 -1.50 dB
PL1W 113.54028320 W
SFO1 150.9201519 MHz
======== CHANNEL f2 ========
CPDPRG[2 waltz16
NUC2 1H
PCPD2 90.00 usec
PL2 4.00 dB
PL12 21.50 dB
PL13 24.50 dB
PL2W 6.09999990 W
PL12W 0.10847504 W
PL13W 0.05436631 W
SFO2 600.1324005 MHz
F2 - Processing parameters
SF 150.9028090 MHz
WDW EM
SSB 0
LB 2.00 Hz
GB 0
PC 1.00
190 180 170 160 150 140 130 120 110 100 90 80 70 60 50 40 30 20 ppm
OAc
OBn
2-NAPO
STol
TBSO
BzO
Bz

14

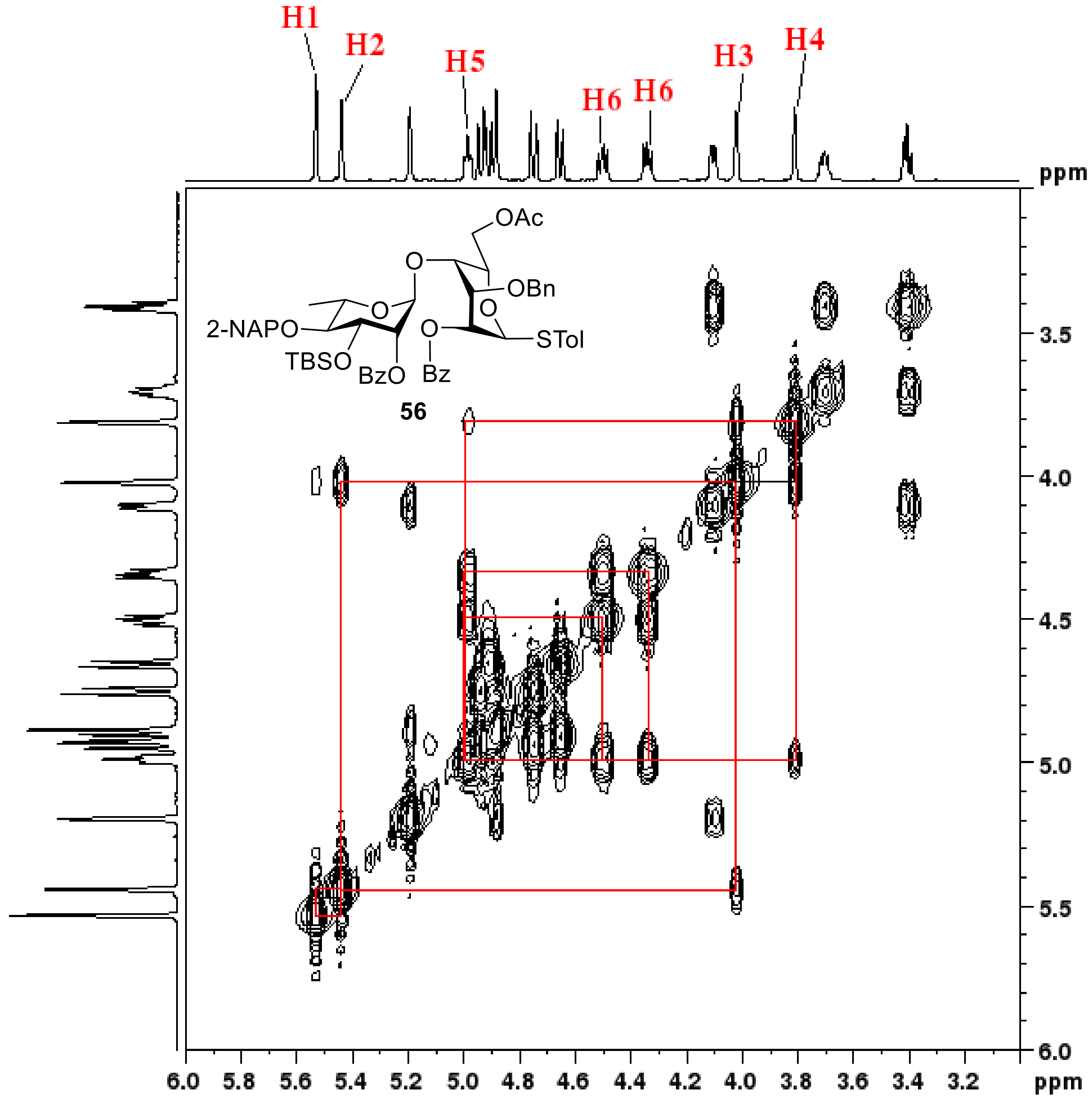
H1
H2
H5
H6
H6
H3
H4
OAc
OBn
2-NAPO
O
O
O
O
STol
TBSO
BzO
Bz
56
ppm

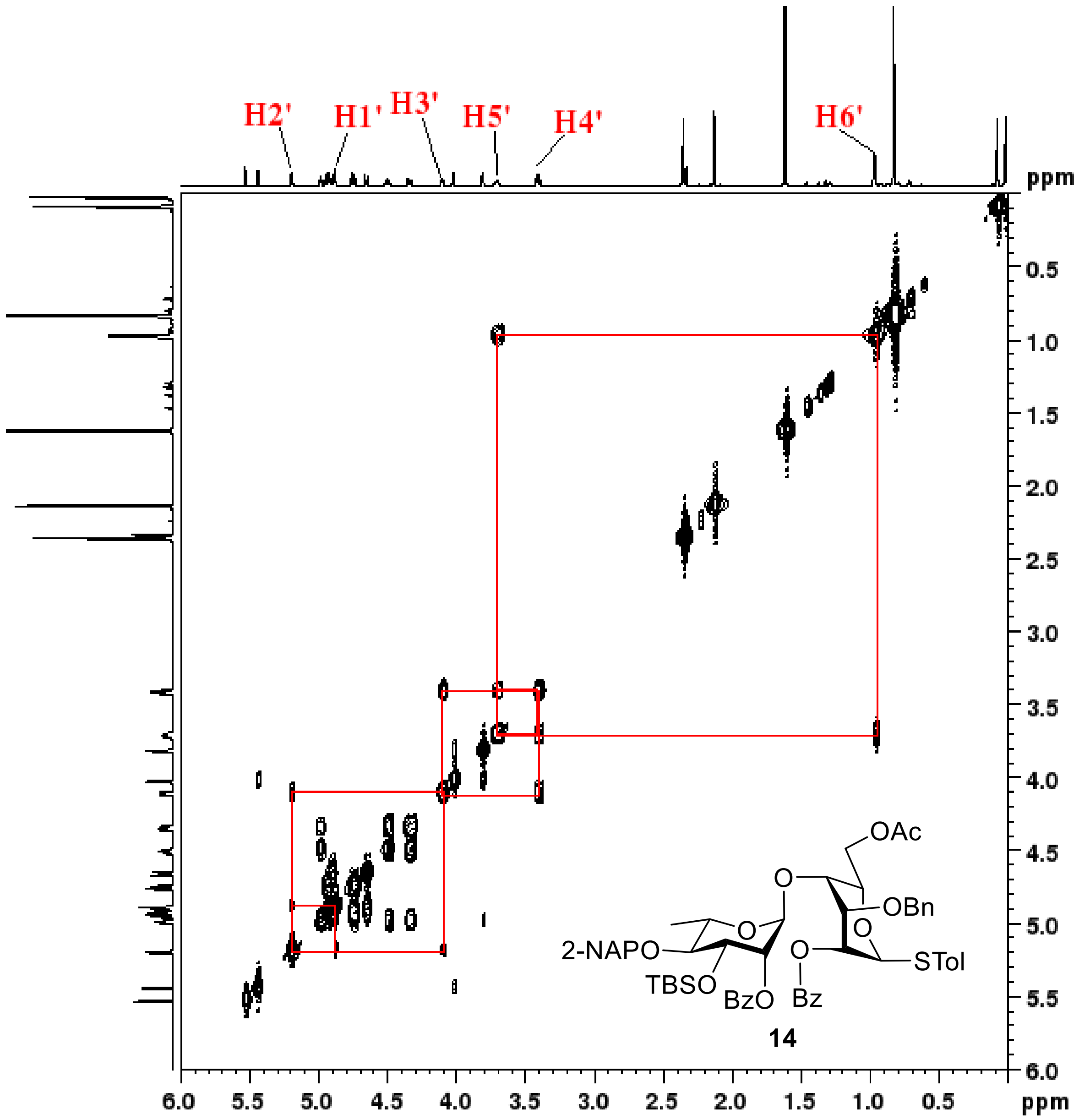
H2'
H1'
H3'
H5'
H4'
H6'
OAc
OBn
2-NAPO
O
O
O
O
STol
TBSO
BzO
Bz
14
ppm

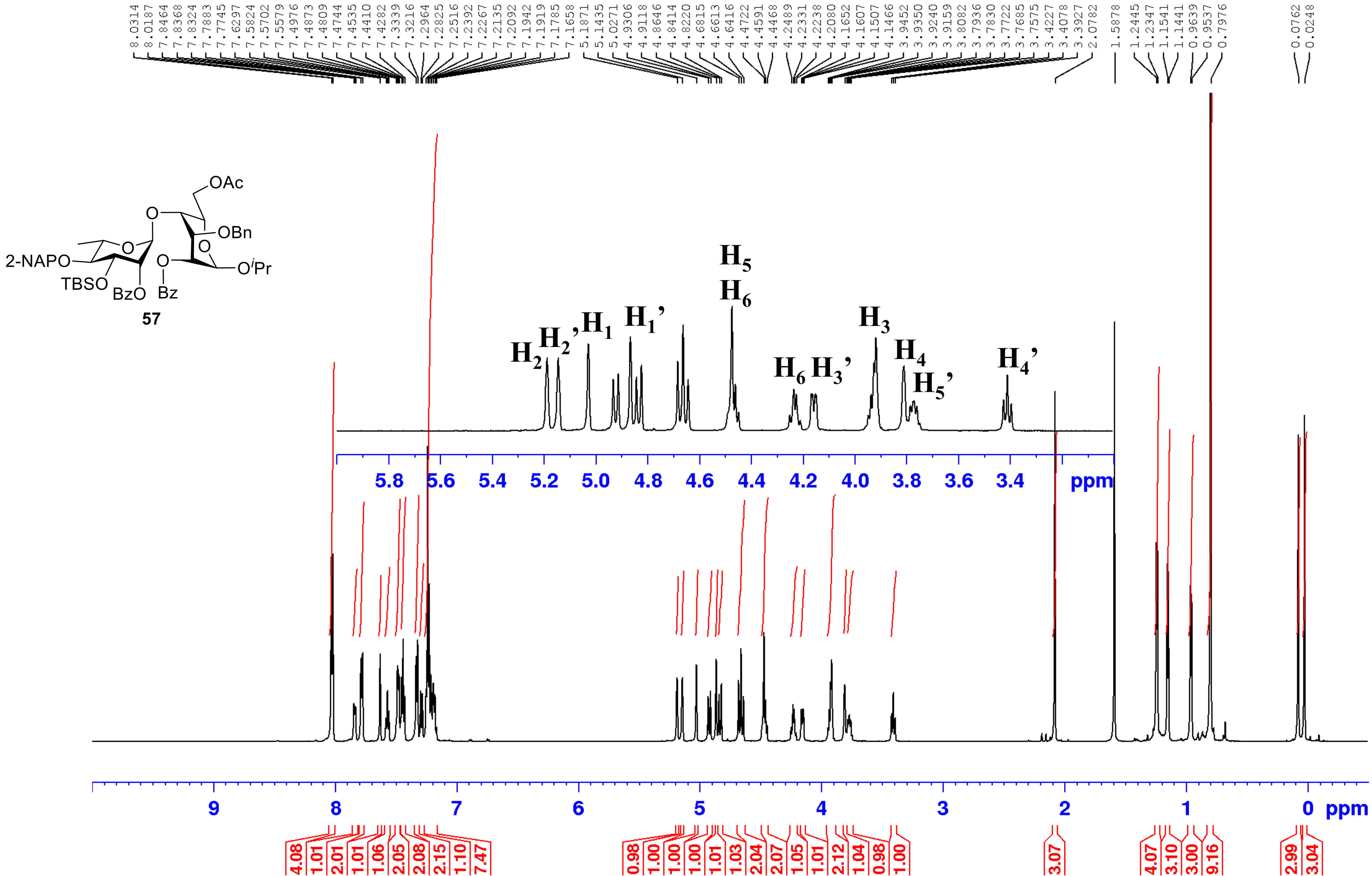

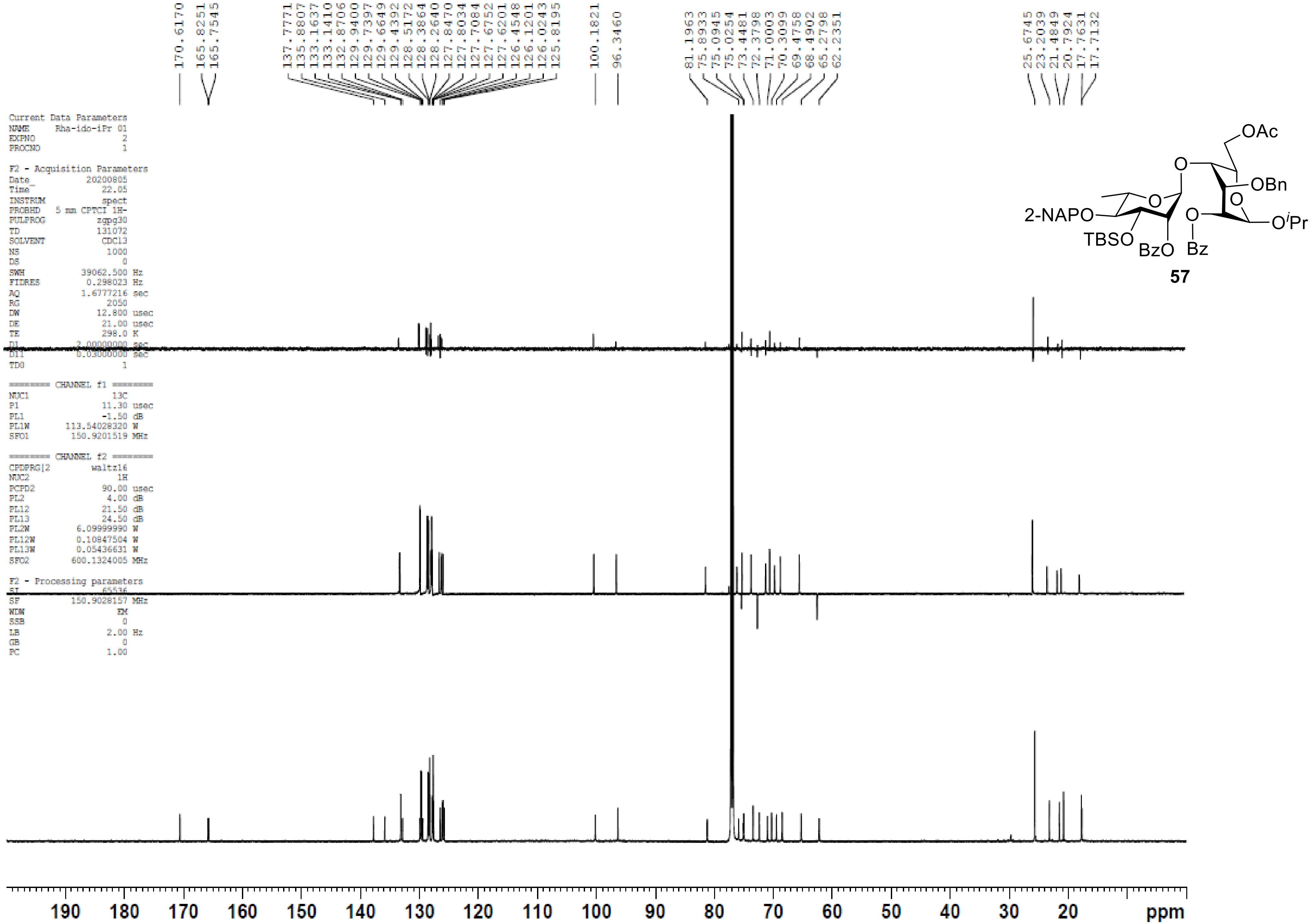
170.6170
165.8251
165.7545
137.7771
135.8807
133.1637
133.1410
132.8706
129.9400
129.7397
129.6649
129.4392
128.5172
128.3864
128.2640
127.8470
127.8034
127.7084
127.6752
127.6201
126.4548
126.1201
126.0243
125.8195
100.1821
96.3460
81.1963
75.8933
75.0945
75.0254
73.4481
72.3798
71.0003
70.3099
69.4758
68.4902
65.2798
62.2351
25.6745
23.2039
21.4849
20.7924
17.7631
17.7132
Current Data Parameters
NAME Rha-ido-iPr 01
EXPNO 2
PROCNO 1
F2 - Acquisition Parameters
Date_ 20200805
Time 22.05
INSTRUM spect
PROBHD 5 mm CPTCI 1H-
PULPROG zgpg30
TD 131072
SOLVENT CDCl3
NS 1000
DS 0
SWH 39062.500 Hz
FIDRES 0.298023 Hz
AQ 1.6777216 sec
RG 2050
DW 12.800 usec
DE 21.00 usec
TE 298.0 K
D1 2.00000000 sec
D11 0.03000000 sec
TD0 1
======== CHANNEL f1 ========
NUC1 13C
P1 11.30 usec
PL1 -1.50 dB
PL1W 113.54028320 W
SFO1 150.9201519 MHz
======== CHANNEL f2 ========
CPDPRG[2 waltz16
NUC2 1H
PCPD2 90.00 usec
PL2 4.00 dB
PL12 21.50 dB
PL13 24.50 dB
PL2W 6.09999990 W
PL12W 0.10847504 W
PL13W 0.05436631 W
SFO2 600.1324005 MHz
F2 - Processing parameters
SI 65536
SF 150.9028157 MHz
WDW EM
SSB 0
LB 2.00 Hz
GB 0
PC 1.00
190 180 170 160 150 140 130 120 110 100 90 80 70 60 50 40 30 20 ppm
OAc
O
OBn
O
2-NAPO
O
O
O^{i}Pr
TBSO
BzO
Bz

57

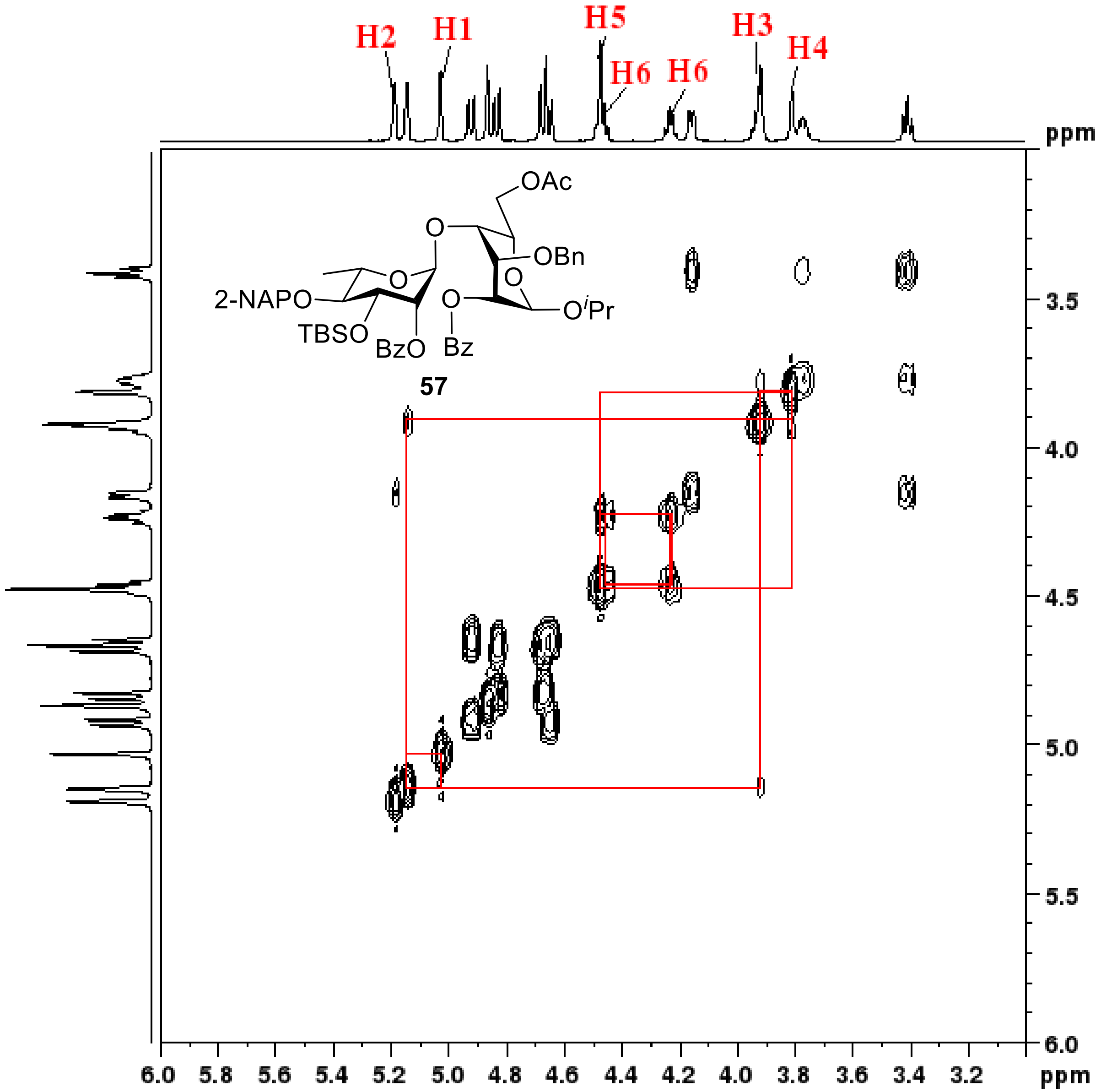
H2
H1
H5
H6
H6
H3
H4
ppm
OAc
OBn
O
2-NAPO
TBSO
BzO
Bz
O^iPr
57
3.5
4.0
4.5
5.0
5.5
6.0
6.0 5.8 5.6 5.4 5.2 5.0 4.8 4.6 4.4 4.2 4.0 3.8 3.6 3.4 3.2
ppm

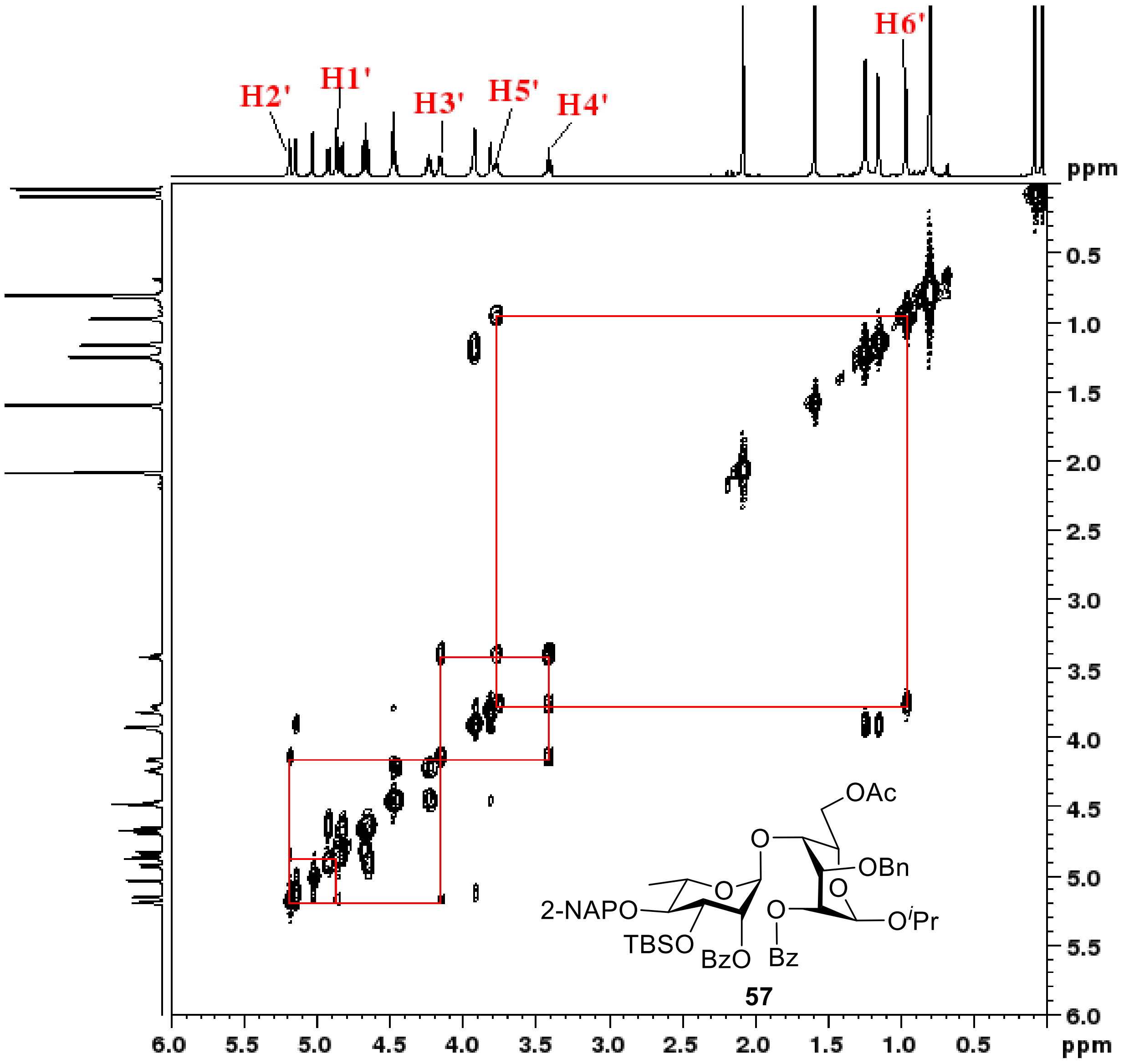
H2'
H1'
H3'
H5'
H4'
H6'
ppm
0.5
1.0
1.5
2.0
2.5
3.0
3.5
4.0
4.5
5.0
5.5
6.0
OAc
OBn
O
2-NAPO
TBSO
BzO
Bz
O^iPr
57
6.0 5.5 5.0 4.5 4.0 3.5 3.0 2.5 2.0 1.5 1.0 0.5
ppm

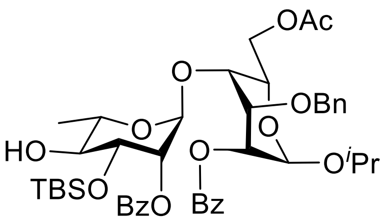
OAc
O
OBn
O
HO
O
O
O^{i}Pr
TBSO
BzO
Bz

58

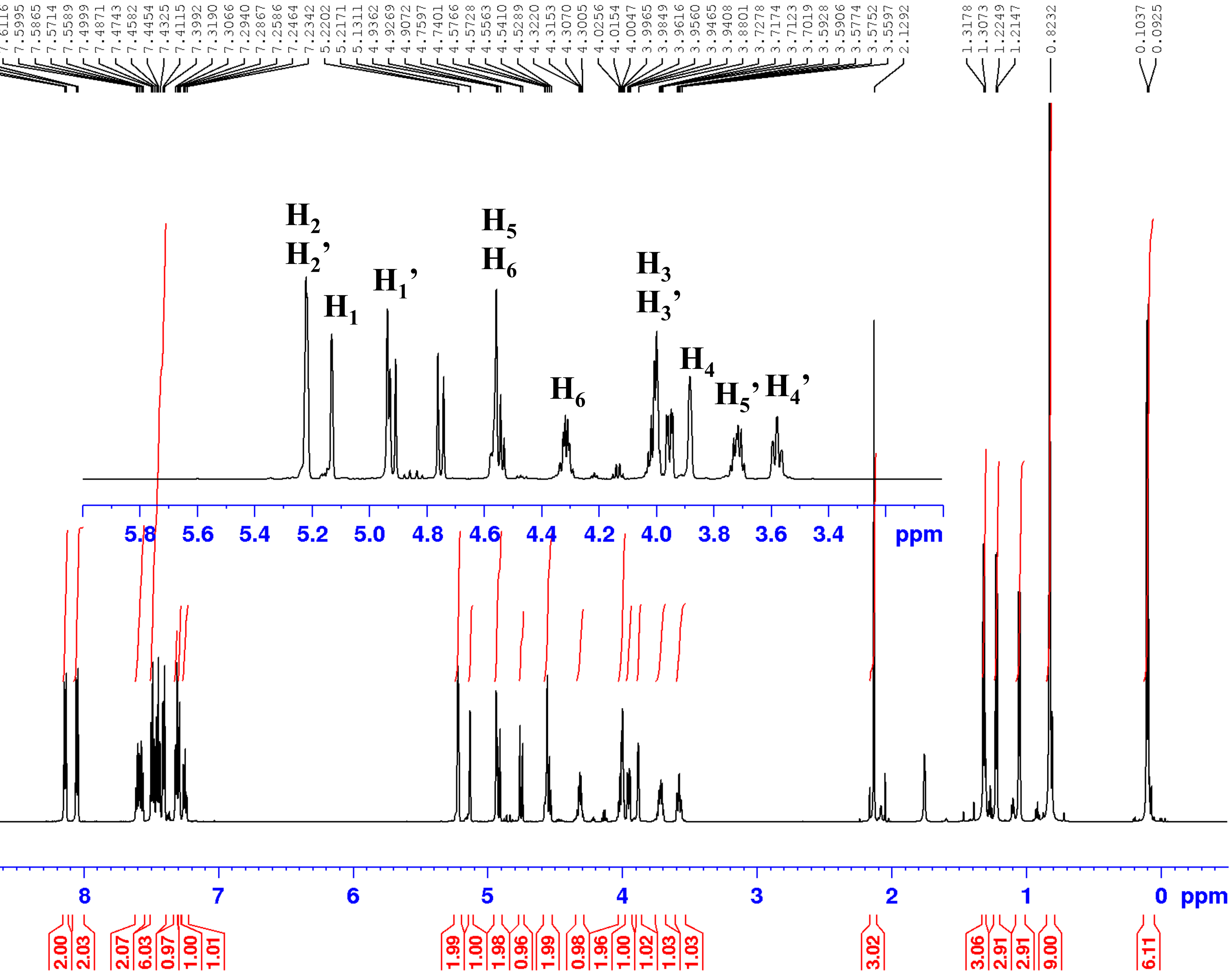
8.1438
8.1307
8.0562
8.0433
7.6116
7.5995
7.5865
7.5714
7.5589
7.4999
7.4871
7.4743
7.4582
7.4454
7.4325
7.4115
7.3992
7.3190
7.3066
7.2940
7.2867
7.2586
7.2464
7.2342
5.2202
5.2171
5.1311
4.9362
4.9269
4.9072
4.7597
4.7401
4.5766
4.5728
4.5563
4.5410
4.5289
4.3220
4.3153
4.3070
4.3005
4.0256
4.0154
4.0047
3.9965
3.9849
3.9616
3.9560
3.9465
3.9408
3.8801
3.7278
3.7174
3.7123
3.7019
3.5928
3.5906
3.5774
3.5752
3.5597
2.1292
1.3178
1.3073
1.2249
1.2147
0.8232
0.1037
0.0925
H_2
H_2'
H_1
H_1'
H_5
H_6
H_6
H_3
H_3'
H_4
H_5'
H_4'
5.8 5.6 5.4 5.2 5.0 4.8 4.6 4.4 4.2 4.0 3.8 3.6 3.4 ppm
9 8 7 6 5 4 3 2 1 0 ppm
2.00
2.03
2.07
6.03
0.97
1.00
1.01
1.99
1.00
1.98
0.96
1.99
0.98
1.96
1.00
1.02
1.03
1.03
3.02
3.06
2.91
2.91
9.00
6.11

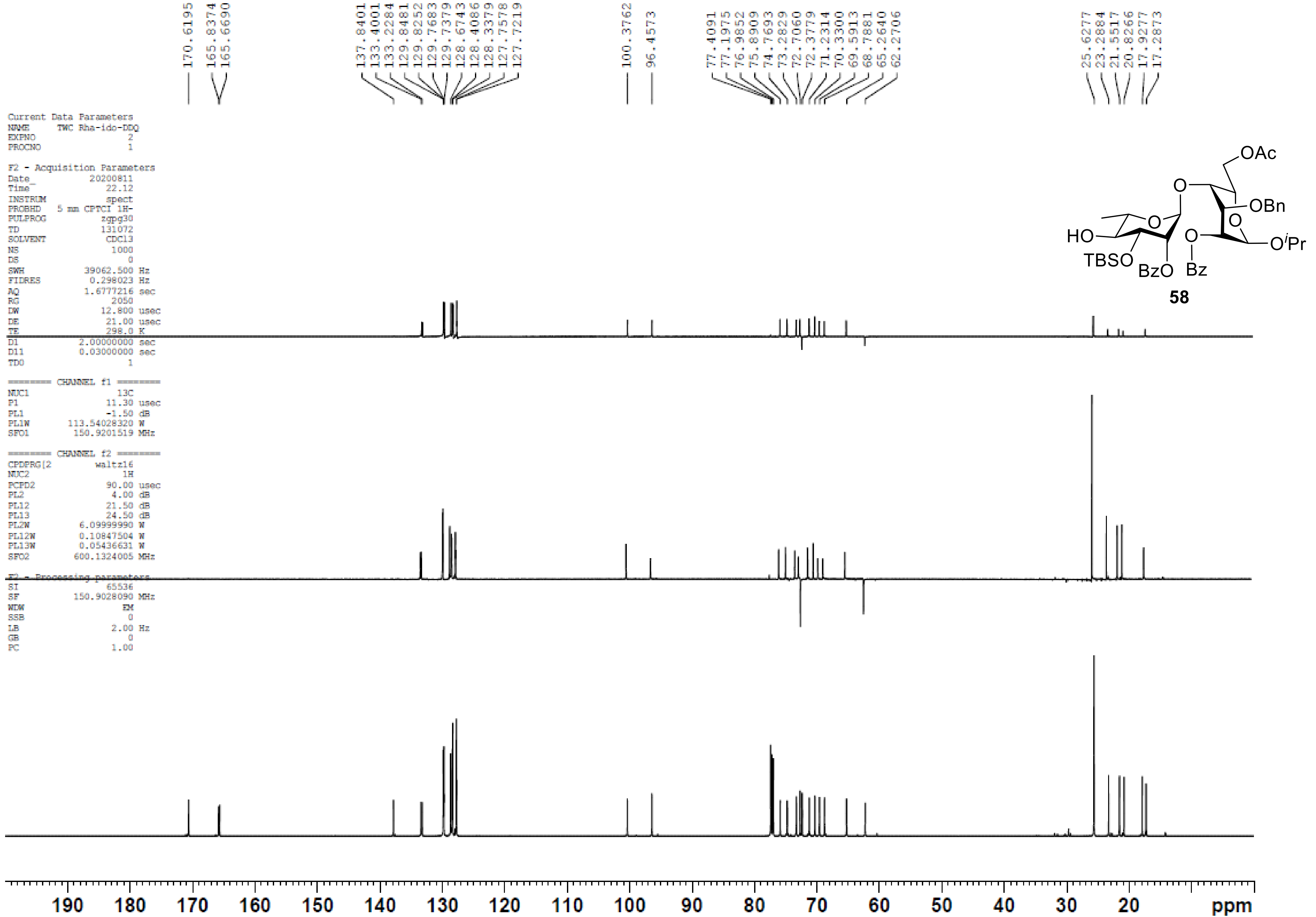
170.6195
165.8374
165.6690
137.8401
133.4001
133.2284
129.8481
129.8252
129.7683
129.7379
128.6743
128.4086
128.3379
127.7578
127.7219
100.3762
96.4573
77.4091
77.1975
76.9852
75.8909
74.7693
73.2829
72.7060
72.3779
71.2314
70.3300
69.5913
68.7881
65.2640
62.2706
25.6277
23.2884
21.5517
20.8266
17.9277
17.2873
Current Data Parameters
NAME TWC Rha-ido-DDQ
EXPNO 2
PROCNO 1
F2 - Acquisition Parameters
Date_ 20200811
Time 22.12
INSTRUM spect
PROBHD 5 mm CPTCI 1H-
PULPROG zgpg30
TD 131072
SOLVENT CDCl3
NS 1000
DS 0
SWH 39062.500 Hz
FIDRES 0.298023 Hz
AQ 1.6777216 sec
RG 2050
DW 12.800 usec
DE 21.00 usec
TE 298.0 K
D1 2.00000000 sec
D11 0.03000000 sec
TD0 1
======== CHANNEL f1 ========
NUC1 13C
P1 11.30 usec
PL1 -1.50 dB
PL1W 113.54028320 W
SFO1 150.9201519 MHz
======== CHANNEL f2 ========
CPDPRG[2 waltz16
NUC2 1H
PCPD2 90.00 usec
PL2 4.00 dB
PL12 21.50 dB
PL13 24.50 dB
PL2W 6.09999990 W
PL12W 0.10847504 W
PL13W 0.05436631 W
SFO2 600.1324005 MHz
F2 - Processing parameters
SI 65536
SF 150.9028090 MHz
WDW EM
SSB 0
LB 2.00 Hz
GB 0
PC 1.00
OAc
O
OBn
O
HO
O
O
O^iPr
TBSO
BzO
Bz
58
190 180 170 160 150 140 130 120 110 100 90 80 70 60 50 40 30 20 ppm

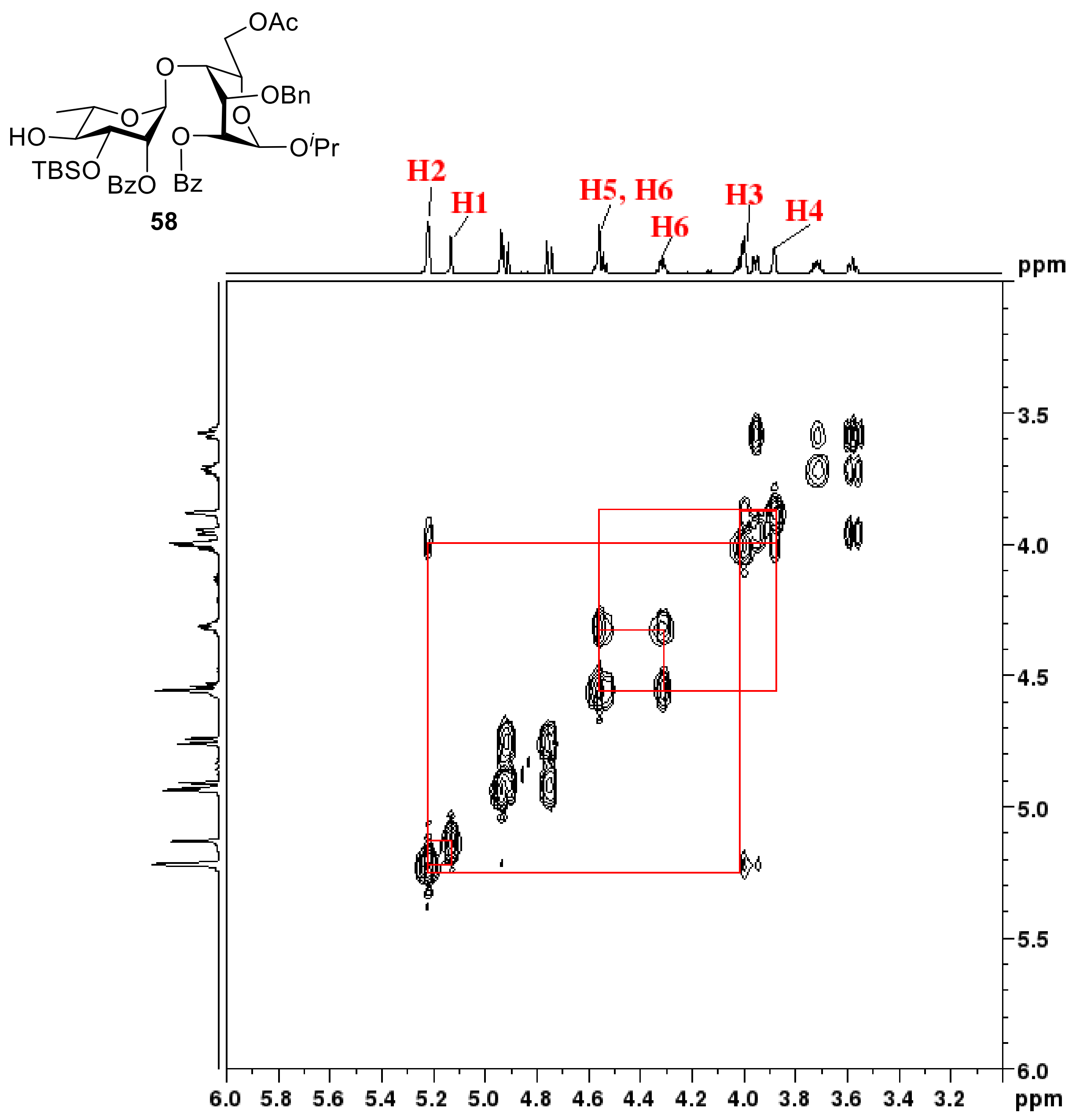
OAc
OBn
HO
TBSO
BzO
Bz
O^{i}Pr
58
H2
H1
H5, H6
H6
H3
H4
ppm
3.5
4.0
4.5
5.0
5.5
6.0
6.0
5.8
5.6
5.4
5.2
5.0
4.8
4.6
4.4
4.2
4.0
3.8
3.6
3.4
3.2

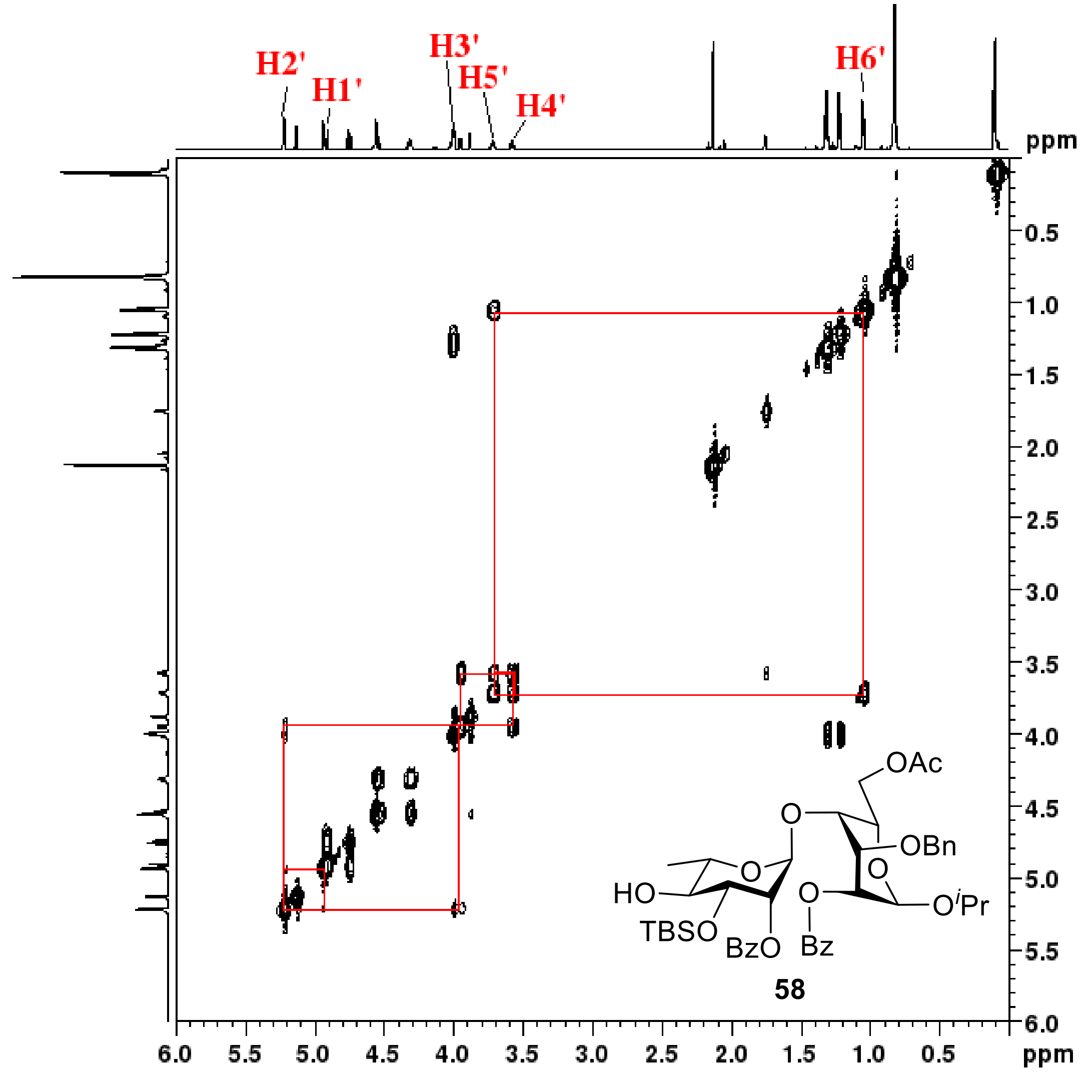
H2'
H1'
H3'
H5'
H4'
H6'
ppm
0.5
1.0
1.5
2.0
2.5
3.0
3.5
4.0
4.5
5.0
5.5
6.0
OAc
OBn
HO
TBSO
BzO
Bz
O^{i}Pr
58
6.0
5.5
5.0
4.5
4.0
3.5
3.0
2.5
2.0
1.5
1.0
0.5

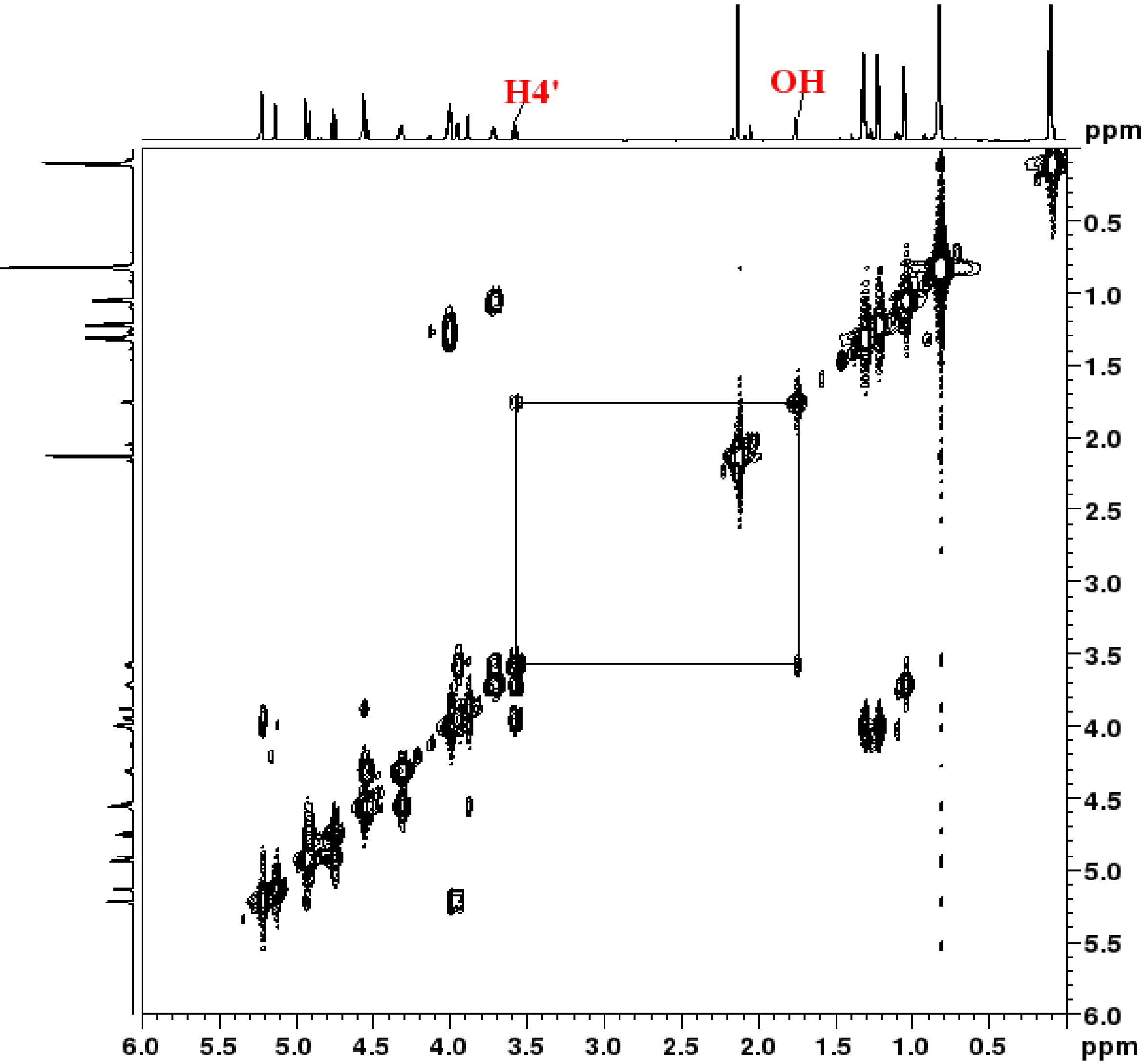

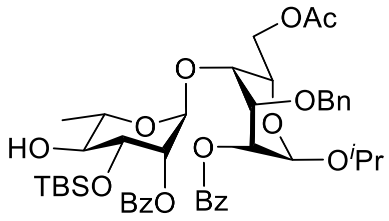

58

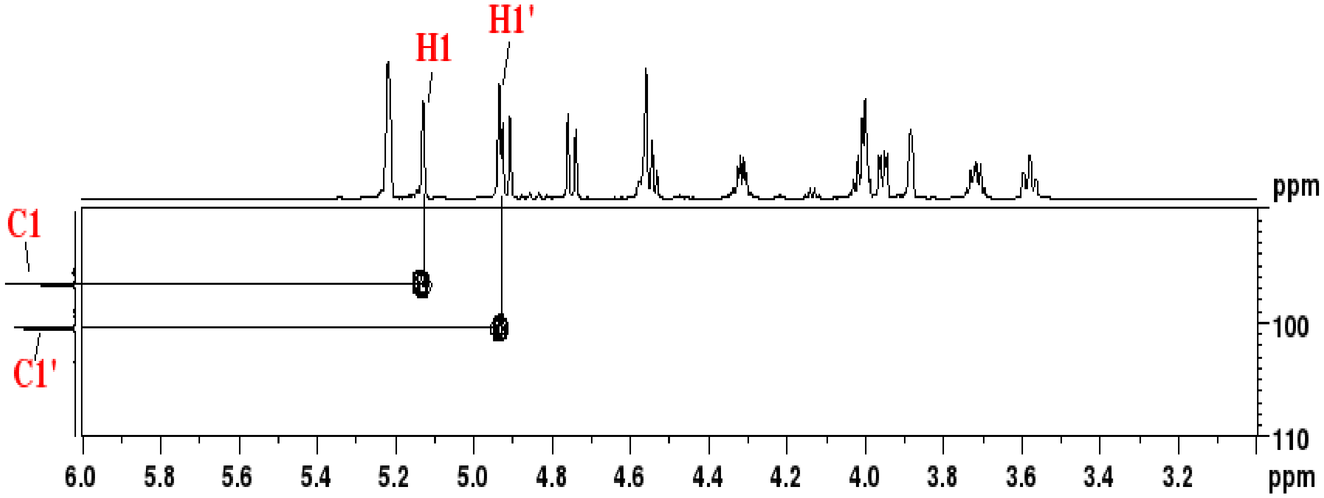

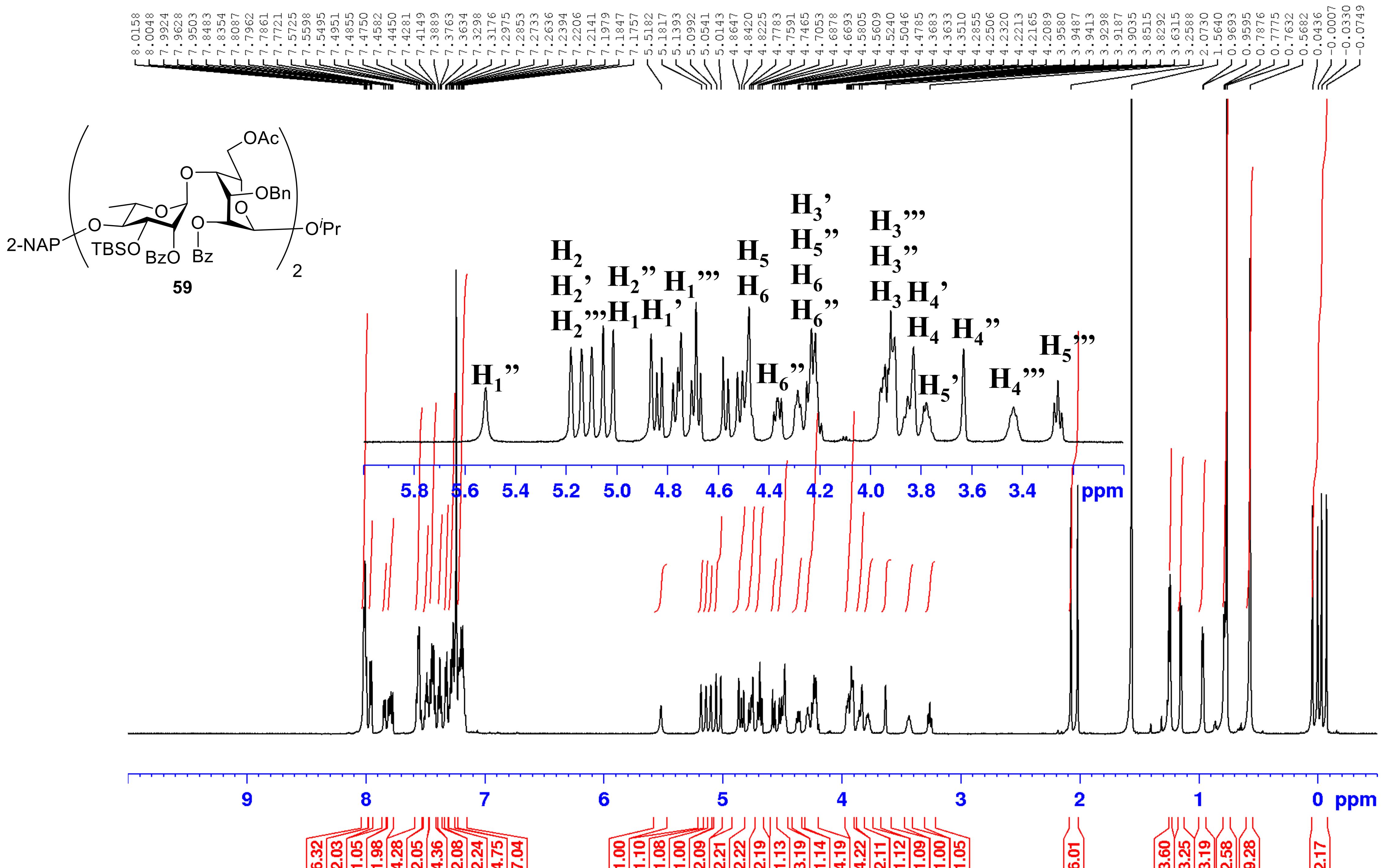

OAc
OBn
O
O
O
O
O
2-NAP
TBSO
BzO
Bz
O^{i}Pr
2
59
H$_1$”
H$_2$ H$_2$’ H$_2$’’’
H$_2$” H$_1$
H$_1$’’’ H$_1$’
H$_5$ H$_6$
H$_6$”
H$_3$’ H$_5$” H$_6$ H$_6$”
H$_3$’’’ H$_3$” H$_3$
H$_4$’ H$_4$
H$_5$’
H$_4$”
H$_4$’’’
H$_5$’’’
ppm

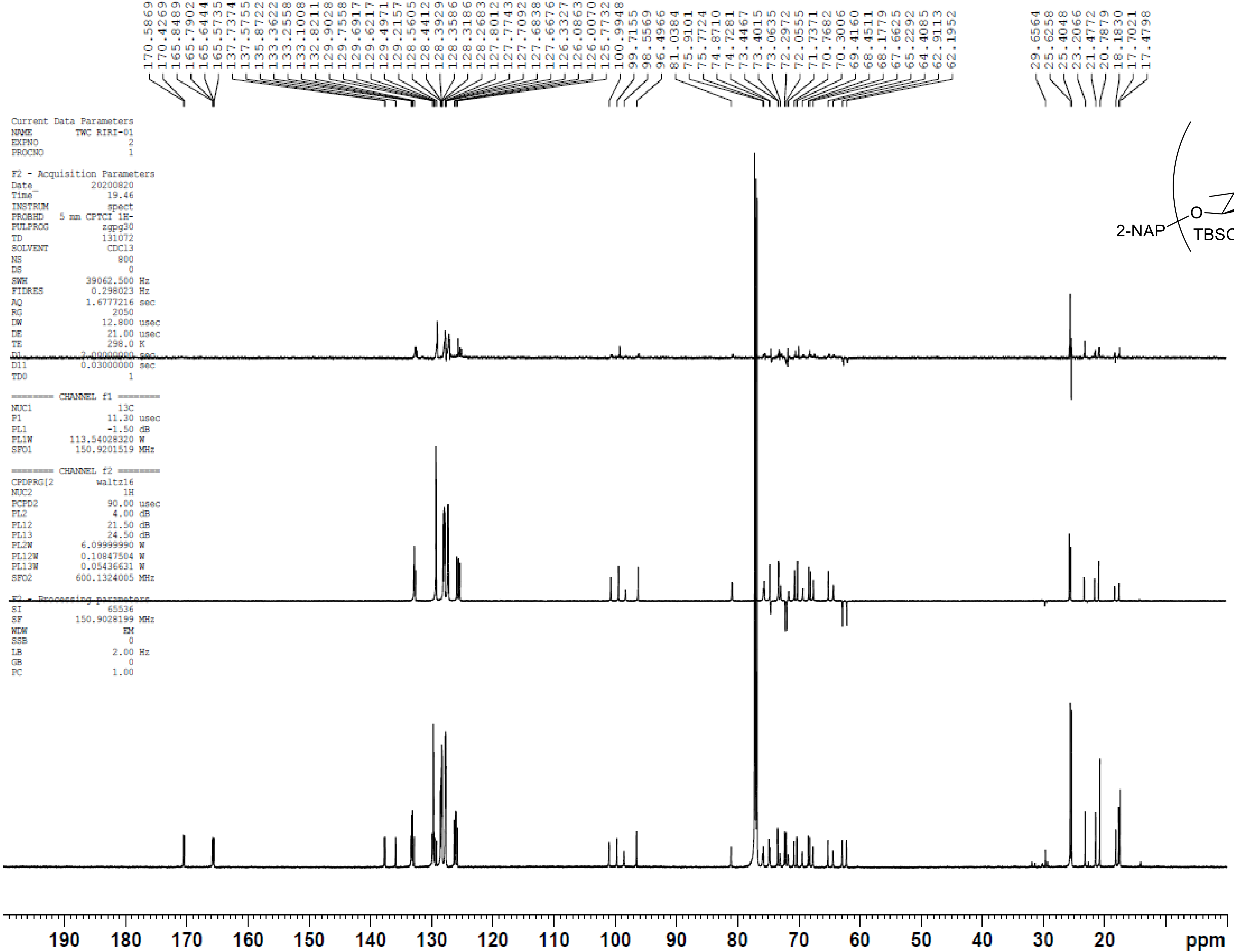
170.5869
170.4269
165.8489
165.7902
165.6444
165.5735
137.7374
135.8722
133.3622
133.2558
133.1008
132.8211
129.9028
129.7558
129.6917
129.6217
129.4971
129.2157
128.5605
128.4412
128.3929
128.3586
128.3186
128.2683
127.8012
127.7743
127.7092
127.6838
127.6676
126.3327
126.0863
126.0070
125.7732
100.9948
99.7155
98.5569
96.4966
81.0384
75.9101
75.7724
74.8710
74.7281
73.4467
73.4015
73.0635
72.2972
72.0555
71.7371
70.7682
70.3006
69.4160
68.4511
68.1779
67.6625
65.2292
64.4085
62.9113
62.1952
29.6564
25.6258
25.4048
23.2066
21.4772
20.7879
18.1830
17.7021
17.4798
Current Data Parameters
NAME TWC RIRI-01
EXPNO 2
PROCNO 1
F2 - Acquisition Parameters
Date_ 20200820
Time_ 19.46
INSTRUM spect
PROBHD 5 mm CPTCI 1H-
PULPROG zgpg30
TD 131072
SOLVENT CDCl3
NS 800
DS 0
SWH 39062.500 Hz
FIDRES 0.298023 Hz
AQ 1.6777216 sec
RG 2050
DW 12.800 usec
DE 21.00 usec
TE 298.0 K
D1 2.00000000 sec
D11 0.03000000 sec
TD0 1
======== CHANNEL f1 ========
NUC1 13C
P1 11.30 usec
PL1 -1.50 dB
PL1W 113.54028320 W
SFO1 150.9201519 MHz
======== CHANNEL f2 ========
CPDPRG[2 waltz16
NUC2 1H
PCPD2 90.00 usec
PL2 4.00 dB
PL12 21.50 dB
PL13 24.50 dB
PL2W 6.09999990 W
PL12W 0.10847504 W
PL13W 0.05436631 W
SFO2 600.1324005 MHz
F2 - Processing parameters
SI 65536
SF 150.9028199 MHz
WDW EM
SSB 0
LB 2.00 Hz
GB 0
PC 1.00
190 180 170 160 150 140 130 120 110 100 90 80 70 60 50 40 30 20 ppm

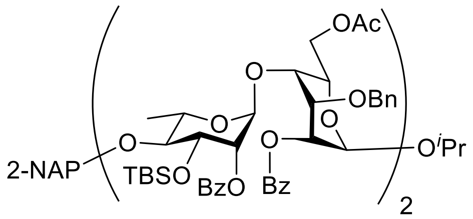
OAc
O
OBn
O
O
O
O
O^{i}Pr
2-NAP
TBSO
BzO
Bz
2

59

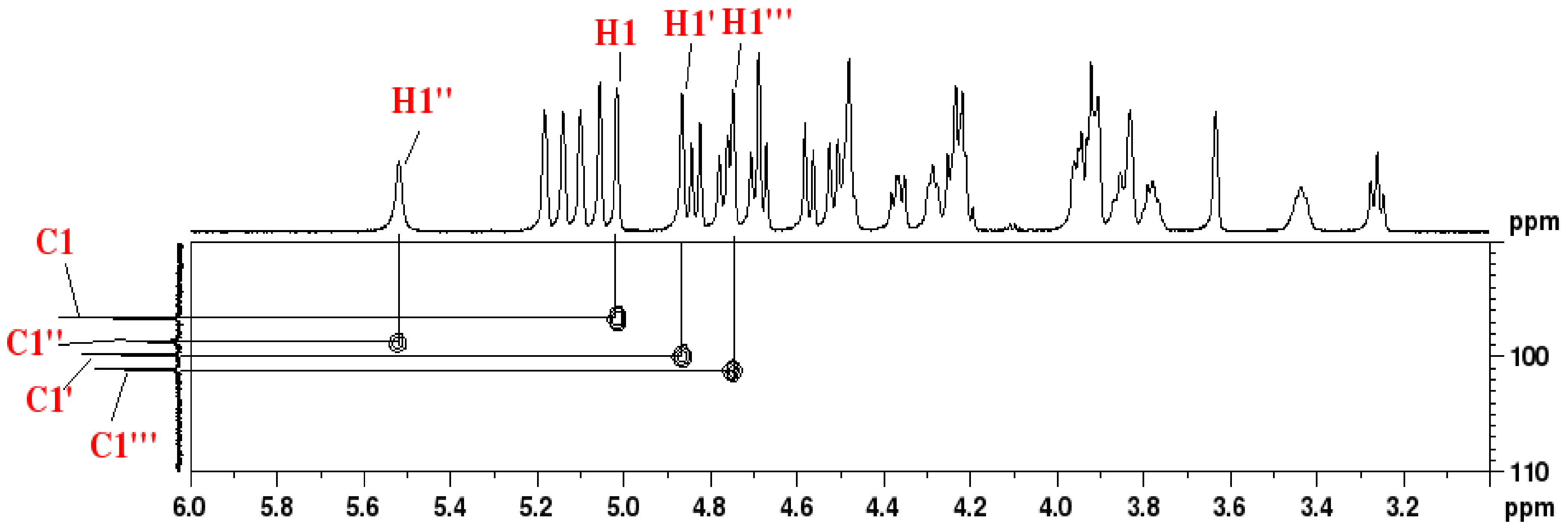
H1''
H1
H1'
H1'''
C1
C1''
C1'
C1'''
ppm
100
110
6.0 5.8 5.6 5.4 5.2 5.0 4.8 4.6 4.4 4.2 4.0 3.8 3.6 3.4 3.2
ppm

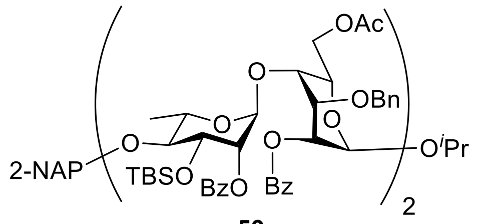
OAc
O
OBn
O
O
O
O
2-NAP
TBSO
BzO
Bz
O^{i}Pr
2

59

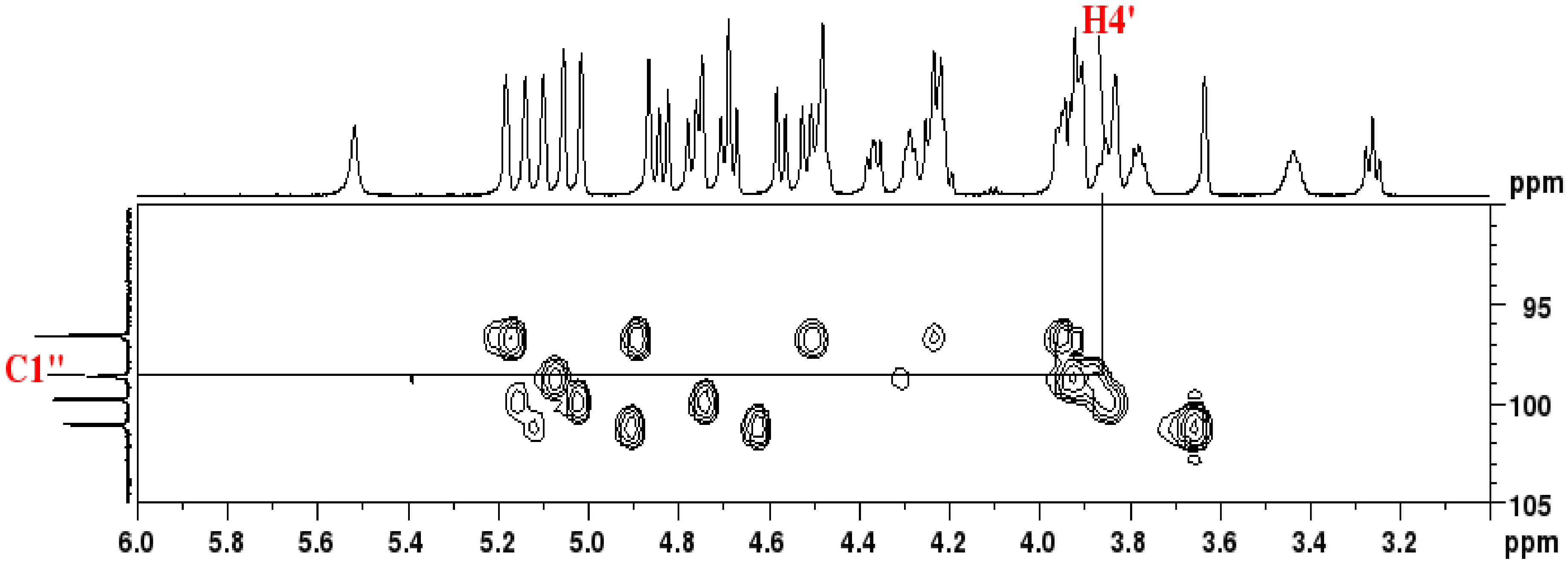
H4'
ppm
C1''
95
100
105
6.0 5.8 5.6 5.4 5.2 5.0 4.8 4.6 4.4 4.2 4.0 3.8 3.6 3.4 3.2
ppm

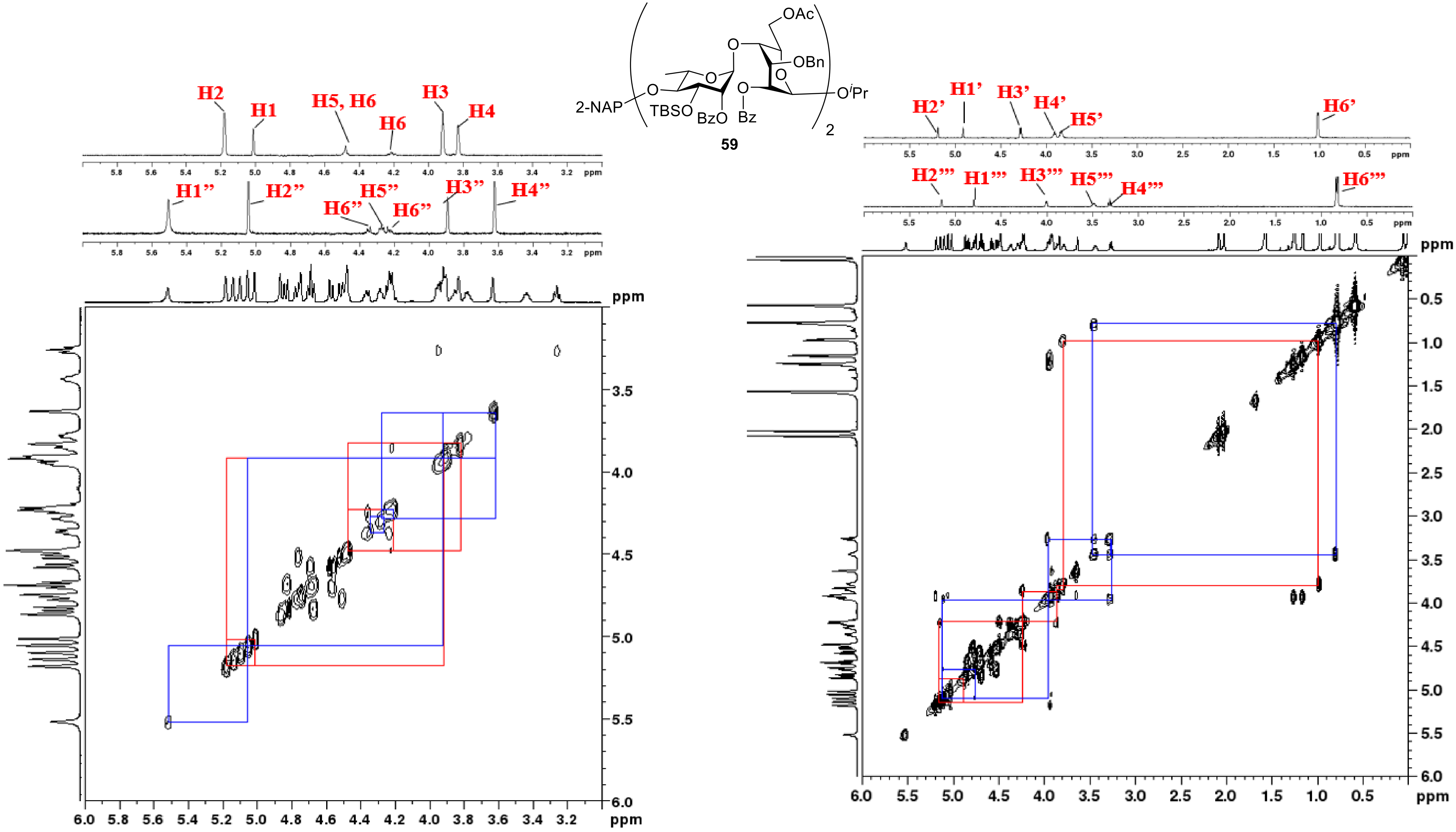

2-NAP
TBSO
BzO
OAc
OBn
Bz
O^{i}Pr
59
H2
H1
H5, H6
H6
H3
H4
H1”
H2”
H5”
H6”
H6”
H3”
H4”
H2’
H1’
H3’
H4’
H5’
H6’
H2’’’
H1’’’
H3’’’
H5’’’
H4’’’
H6’’’
ppm

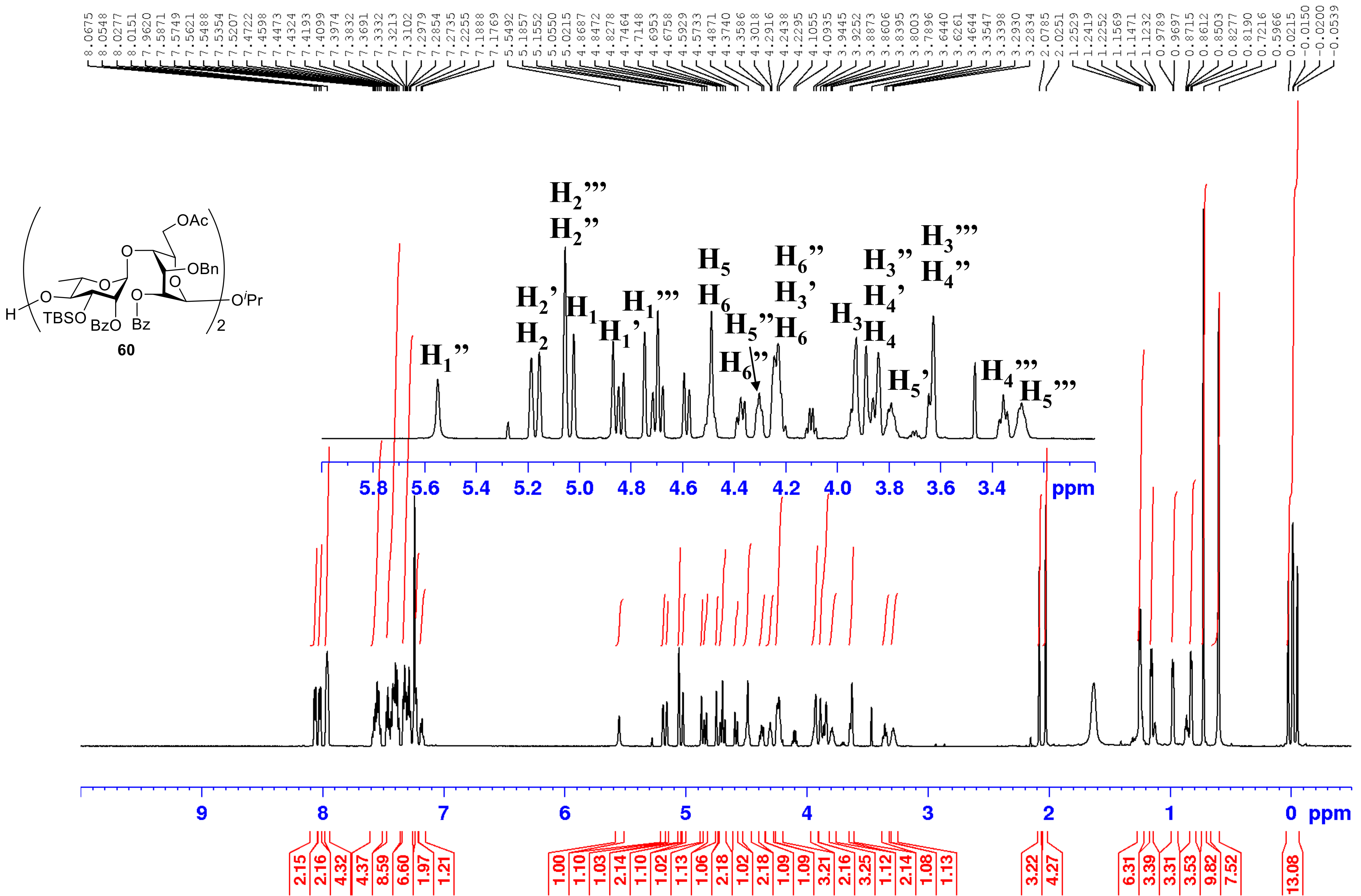
60
ppm
ppm

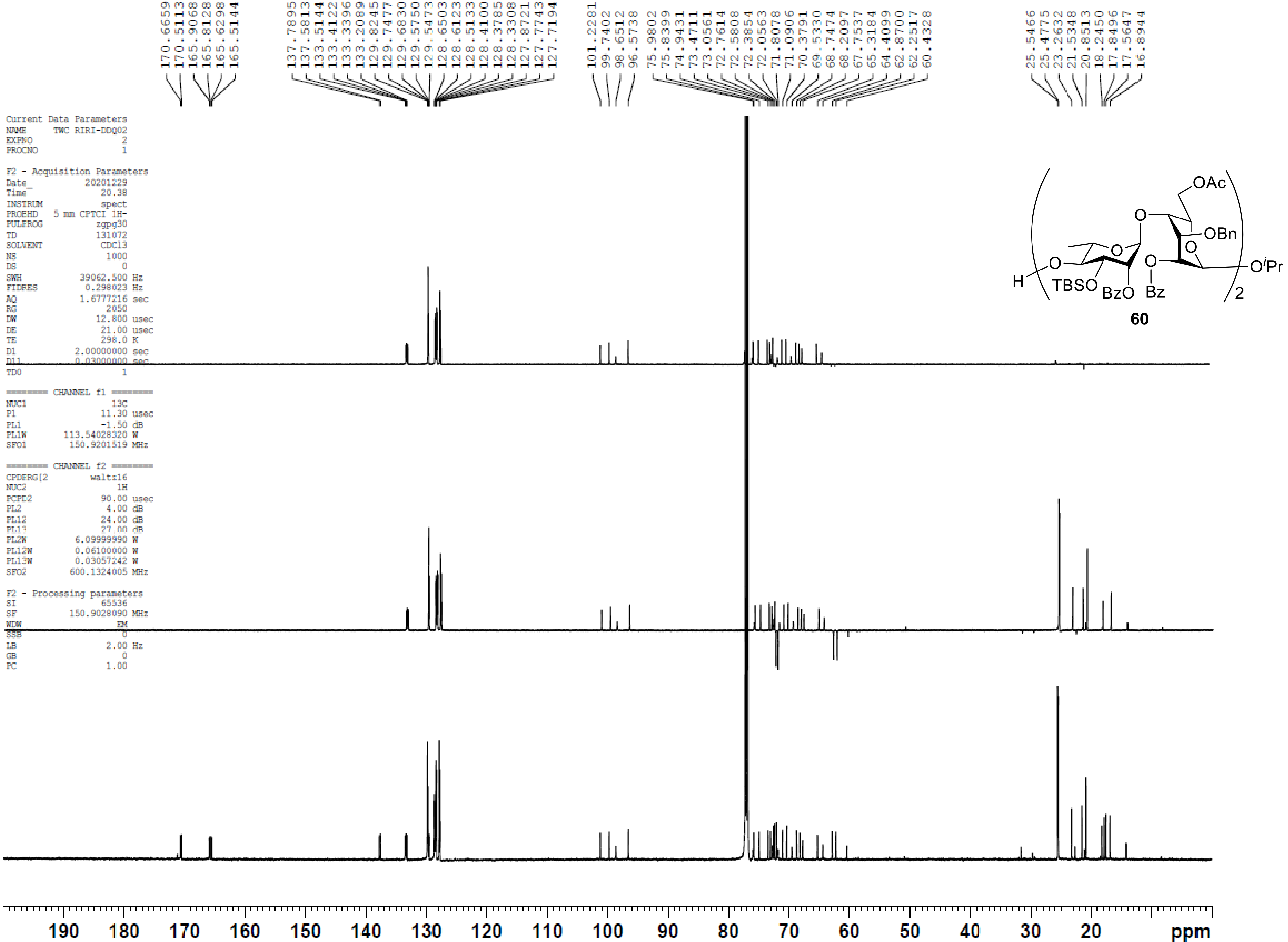
170.6659
170.5113
165.9068
165.8128
165.6298
165.5144
137.7895
137.5813
133.5144
133.4122
133.3396
133.2089
129.8245
129.7477
129.6830
129.5750
129.5473
128.6503
128.6123
128.5133
128.4100
128.3785
128.3308
127.8721
127.7743
127.7194
101.2281
99.7402
98.6512
96.5738
75.9802
75.8399
74.9431
73.4711
73.0561
72.7614
72.5808
72.3854
72.0563
71.8078
71.0906
70.3791
69.5330
68.7474
68.2097
67.7537
65.3184
64.4099
62.8700
62.2517
60.4328
25.5466
25.4775
23.2632
21.5348
20.8513
18.2450
17.8496
17.5647
16.8944
Current Data Parameters
NAME TWC RIRI-DDQ02
EXPNO 2
PROCNO 1
F2 - Acquisition Parameters
Date_ 20201229
Time 20.38
INSTRUM spect
PROBHD 5 mm CPTCI 1H-
PULPROG zgpg30
TD 131072
SOLVENT CDCl3
NS 1000
DS 0
SWH 39062.500 Hz
FIDRES 0.298023 Hz
AQ 1.6777216 sec
RG 2050
DW 12.800 usec
DE 21.00 usec
TE 298.0 K
D1 2.00000000 sec
D11 0.03000000 sec
TD0 1
======== CHANNEL f1 ========
NUC1 13C
P1 11.30 usec
PL1 -1.50 dB
PL1W 113.54028320 W
SFO1 150.9201519 MHz
======== CHANNEL f2 ========
CPDPRG[2 waltz16
NUC2 1H
PCPD2 90.00 usec
PL2 4.00 dB
PL12 24.00 dB
PL13 27.00 dB
PL2W 6.09999990 W
PL12W 0.06100000 W
PL13W 0.03057242 W
SFO2 600.1324005 MHz
F2 - Processing parameters
SI 65536
SF 150.9028090 MHz
WDW EM
SSB 0
LB 2.00 Hz
GB 0
PC 1.00
190 180 170 160 150 140 130 120 110 100 90 80 70 60 50 40 30 20 ppm
OAc
O
OBn
O
O
O
O
OiPr
H
TBSO
BzO
Bz
2

60

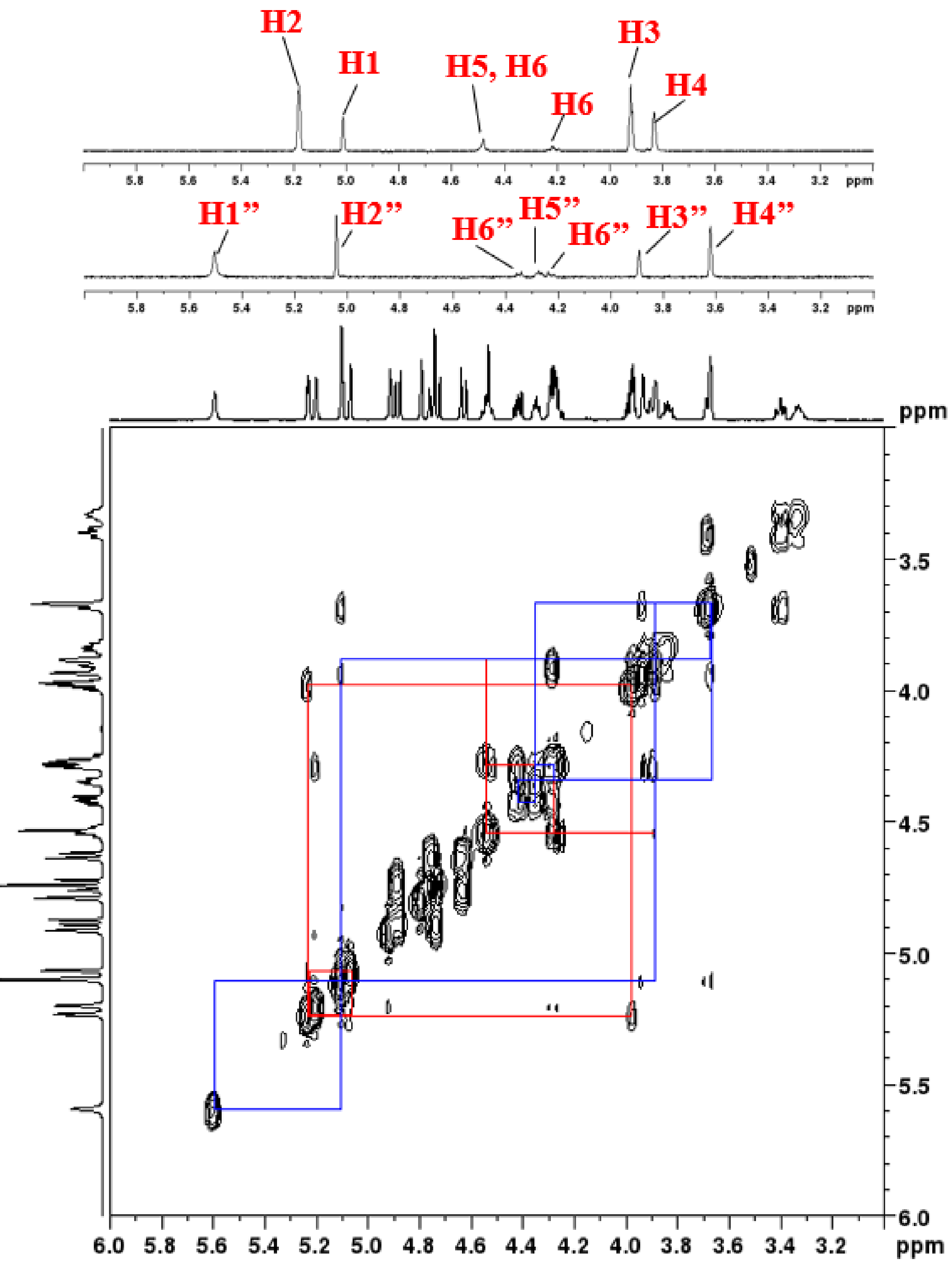
H2
H1
H5, H6
H6
H3
H4
H1”
H2”
H6”
H5”
H6”
H3”
H4”
ppm

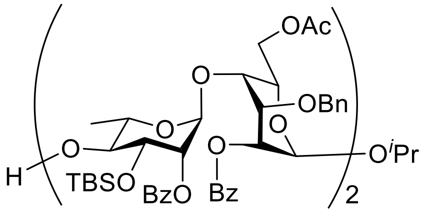
OAc
OBn
O
H
TBSO
BzO
Bz
O^{i}Pr
2

60

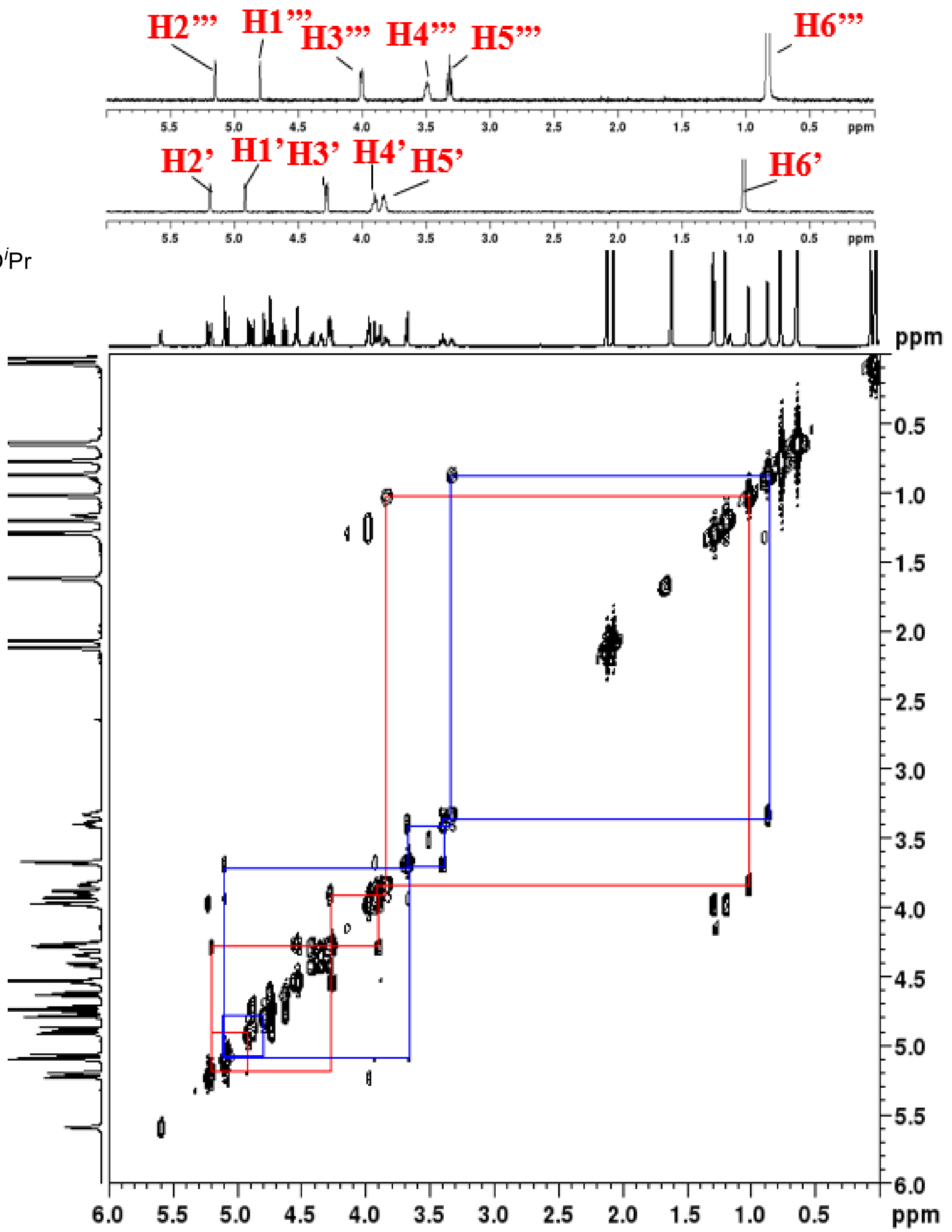
H2’’’
H1’’’
H3’’’
H4’’’
H5’’’
H6’’’
H2’
H1’
H3’
H4’
H5’
H6’
ppm

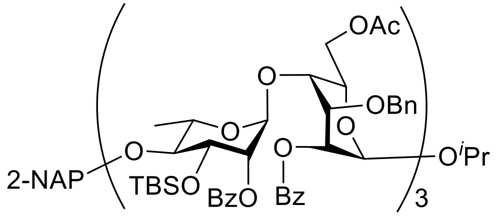
OAc
OBn
O^iPr
2-NAP
TBSO
BzO
Bz
3
61

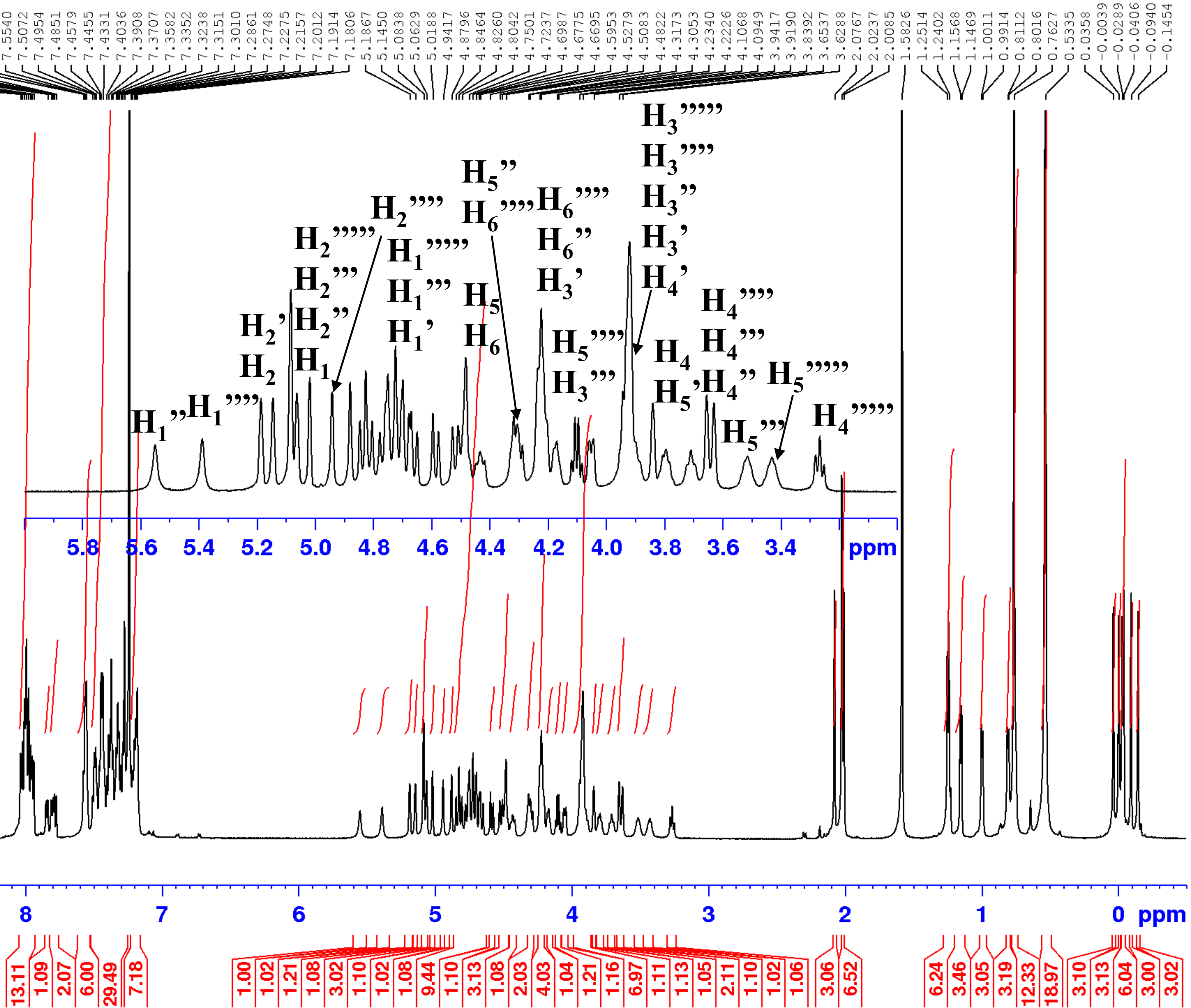
ppm
9
8
7
6
5
4
3
2
1
0
5.8
5.6
5.4
5.2
5.0
4.8
4.6
4.4
4.2
4.0
3.8
3.6
3.4

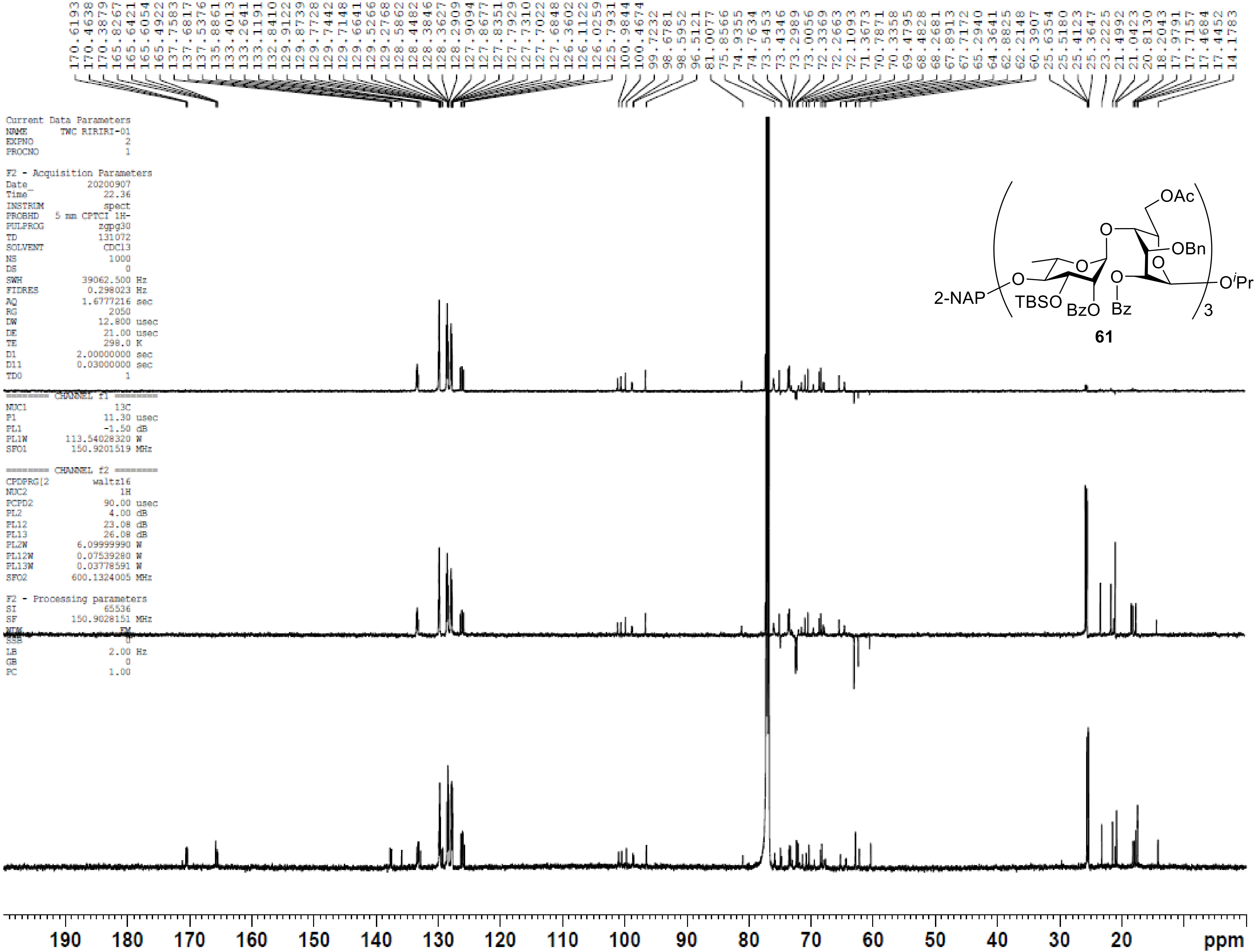
170.6193
170.4638
170.3879
165.8267
165.6421
165.6054
165.4922
137.7583
137.6817
137.5376
135.8861
133.4013
133.2641
133.1191
132.8410
129.9122
129.8739
129.7728
129.7442
129.7148
129.6641
129.5266
129.2768
128.5862
128.4482
128.3846
128.3627
128.2909
127.9094
127.8677
127.8351
127.7929
127.7310
127.7022
127.6848
126.3602
126.1122
126.0259
125.7931
100.9844
100.4674
99.7232
98.6781
98.5952
96.5121
81.0077
75.8566
74.9355
74.7634
73.5453
73.4346
73.2989
73.0056
72.3369
72.2663
72.1093
71.3673
70.7871
70.3358
69.4795
68.4828
68.2681
67.8913
67.7172
65.2940
64.3641
62.8825
62.2148
60.3907
25.6354
25.5180
25.4123
25.3647
23.2225
21.4992
21.0423
20.8130
18.2043
17.9791
17.7157
17.4684
17.4452
14.1783
Current Data Parameters
NAME TWC RIRIRI-01
EXPNO 2
PROCNO 1
F2 - Acquisition Parameters
Date_ 20200907
Time 22.36
INSTRUM spect
PROBHD 5 mm CPTCI 1H-
PULPROG zgpg30
TD 131072
SOLVENT CDCl3
NS 1000
DS 0
SWH 39062.500 Hz
FIDRES 0.298023 Hz
AQ 1.6777216 sec
RG 2050
DW 12.800 usec
DE 21.00 usec
TE 298.0 K
D1 2.00000000 sec
D11 0.03000000 sec
TD0 1
======== CHANNEL f1 ========
NUC1 13C
P1 11.30 usec
PL1 -1.50 dB
PL1W 113.54028320 W
SFO1 150.9201519 MHz
======== CHANNEL f2 ========
CPDPRG[2 waltz16
NUC2 1H
PCPD2 90.00 usec
PL2 4.00 dB
PL12 23.08 dB
PL13 26.08 dB
PL2W 6.09999990 W
PL12W 0.07539280 W
PL13W 0.03778591 W
SFO2 600.1324005 MHz
F2 - Processing parameters
SI 65536
SF 150.9028151 MHz
WDW EM
SSB 0
LB 2.00 Hz
GB 0
PC 1.00
OAc
OBn
2-NAP
TBSO
BzO
Bz
O^iPr
3
61
190
180
170
160
150
140
130
120
110
100
90
80
70
60
50
40
30
20
ppm

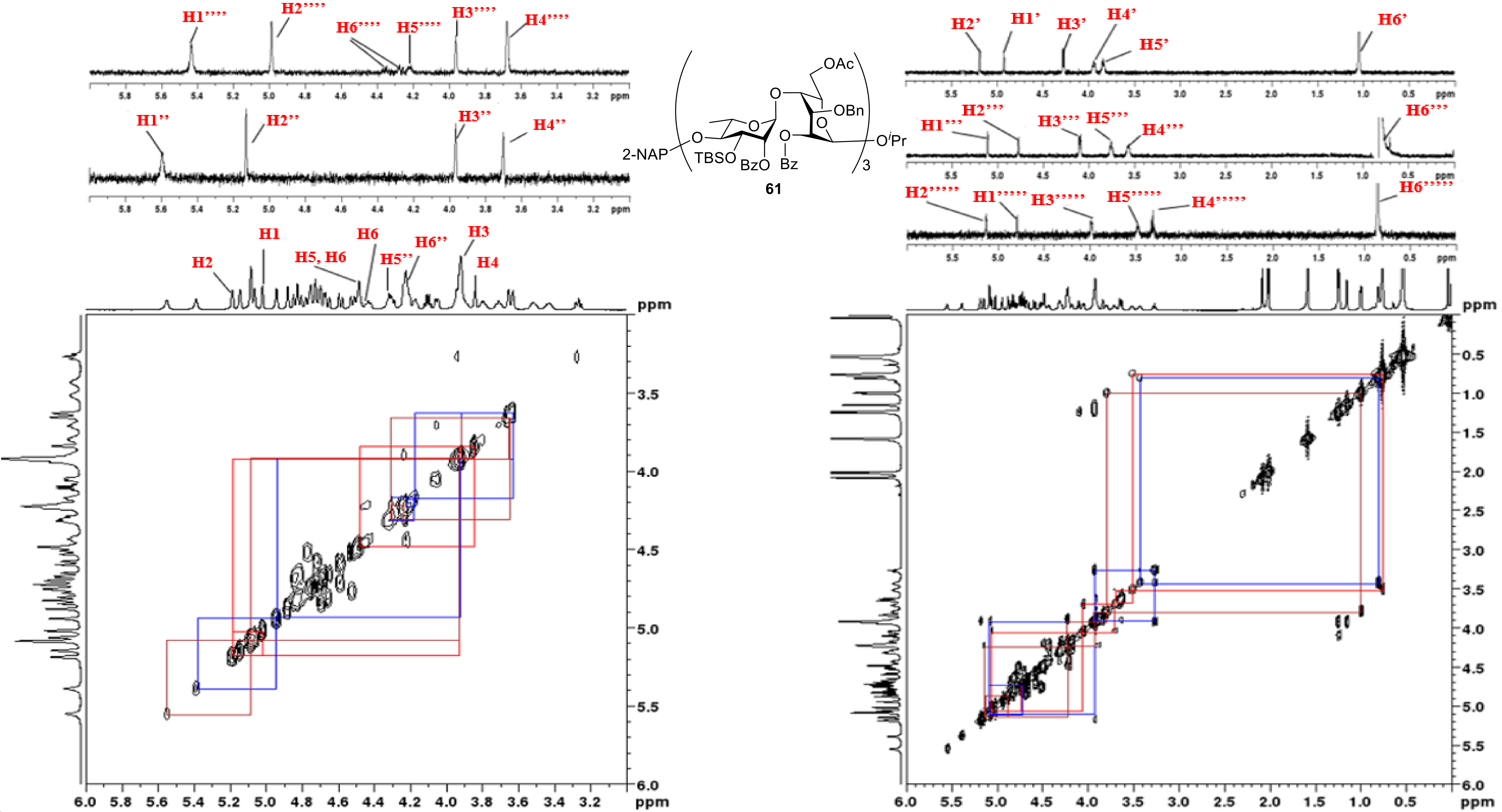
H1''''
H2''''
H6''''
H5''''
H3''''
H4''''
H1''
H2''
H3''
H4''
H2
H1
H5, H6
H6
H5''
H6''
H3
H4
OAc
OBn
O^{i}Pr
2-NAP
TBSO
BzO
Bz
3
61
H2'
H1'
H3'
H4'
H5'
H6'
H1'''
H2'''
H3'''
H5'''
H4'''
H6'''
H2'''''
H1'''''
H3'''''
H5'''''
H4'''''
H6'''''
ppm

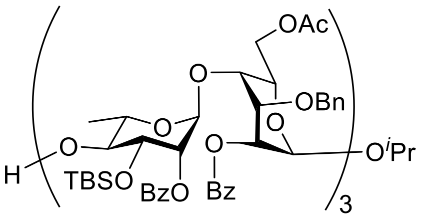

OAc
OBn
O
H
TBSO
BzO
Bz
O^{i}Pr
3

62

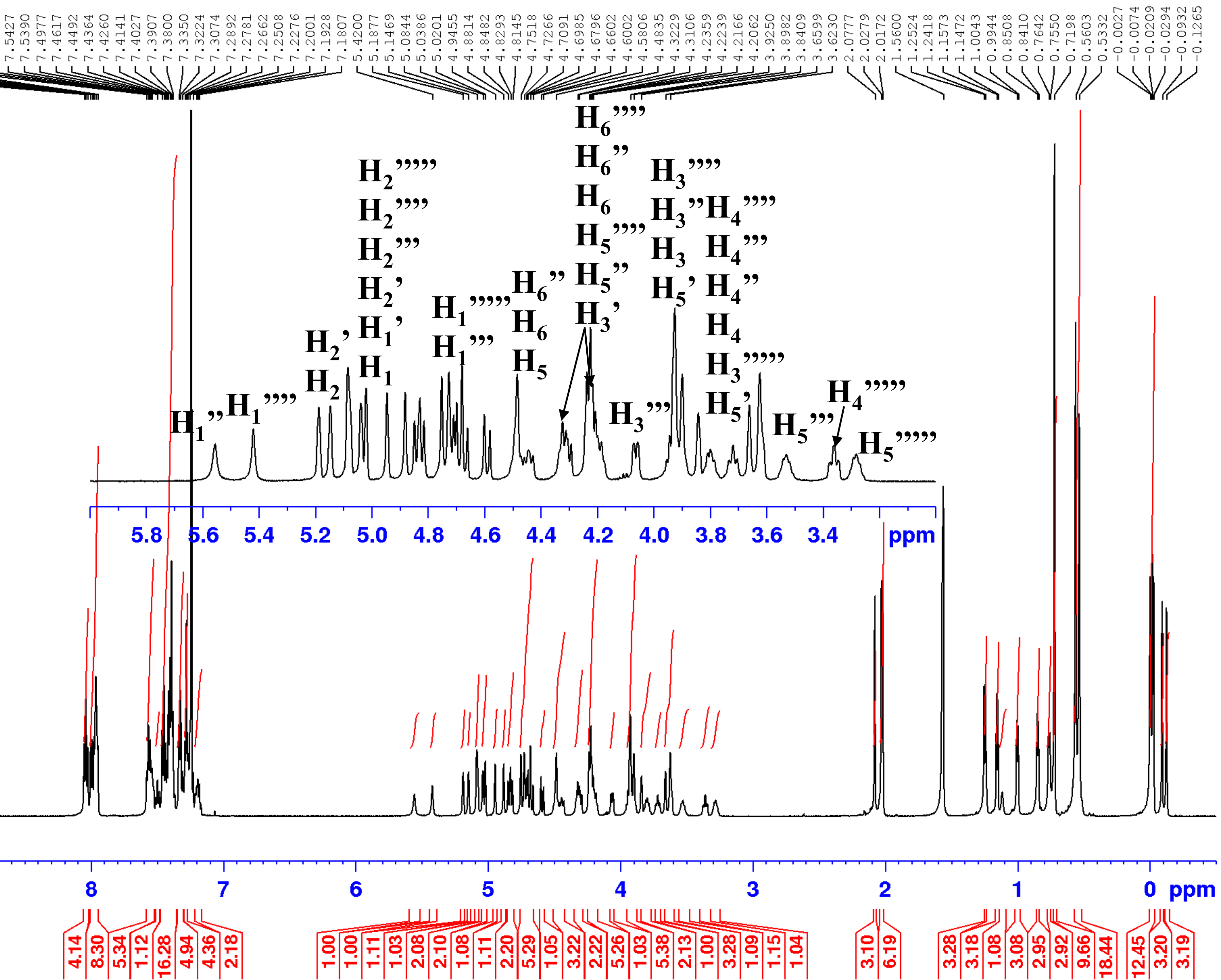

8.0513 8.0378 8.0243 8.0024 7.9893 7.9758 7.9627 7.9476 7.5772 7.5654 7.5530 7.5427 7.5390 7.4977 7.4617 7.4492 7.4364 7.4260 7.4141 7.4027 7.3907 7.3800 7.3350 7.3224 7.3074 7.2892 7.2781 7.2662 7.2508 7.2276 7.2001 7.1928 7.1807 5.4200 5.1877 5.1469 5.0844 5.0386 5.0201 4.9455 4.8814 4.8482 4.8293 4.8145 4.7518 4.7266 4.7091 4.6985 4.6796 4.6602 4.6002 4.5806 4.4835 4.3229 4.3106 4.2359 4.2239 4.2166 4.2062 3.9250 3.8982 3.8409 3.6599 3.6230 2.0777 2.0279 2.0172 1.5600 1.2524 1.2418 1.1573 1.1472 1.0043 0.9944 0.8508 0.8410 0.7642 0.7550 0.7198 0.5603 0.5332 -0.0027 -0.0074 -0.0209 -0.0294 -0.0932 -0.1265
H1'' H1'''' H2' H2 H2''''' H2'''' H2''' H2' H1' H1 H1''''' H1''' H6'' H6 H5 H6'''' H6'' H6 H5'''' H5'' H3' H3''' H3'''' H3'' H3 H5' H4'''' H4''' H4'' H4 H3''''' H5' H5''' H4''''' H5'''''
5.8 5.6 5.4 5.2 5.0 4.8 4.6 4.4 4.2 4.0 3.8 3.6 3.4 ppm
9 8 7 6 5 4 3 2 1 0 ppm
4.14 8.30 5.34 1.12 16.28 4.94 4.36 2.18 1.00 1.00 1.11 1.03 2.08 2.10 1.08 1.11 2.20 5.29 1.05 3.22 2.22 5.26 1.03 5.38 2.13 1.00 3.28 1.09 1.15 1.04 3.10 6.19 3.28 3.18 1.08 3.08 2.95 2.92 9.66 18.44 12.45 3.20 3.19

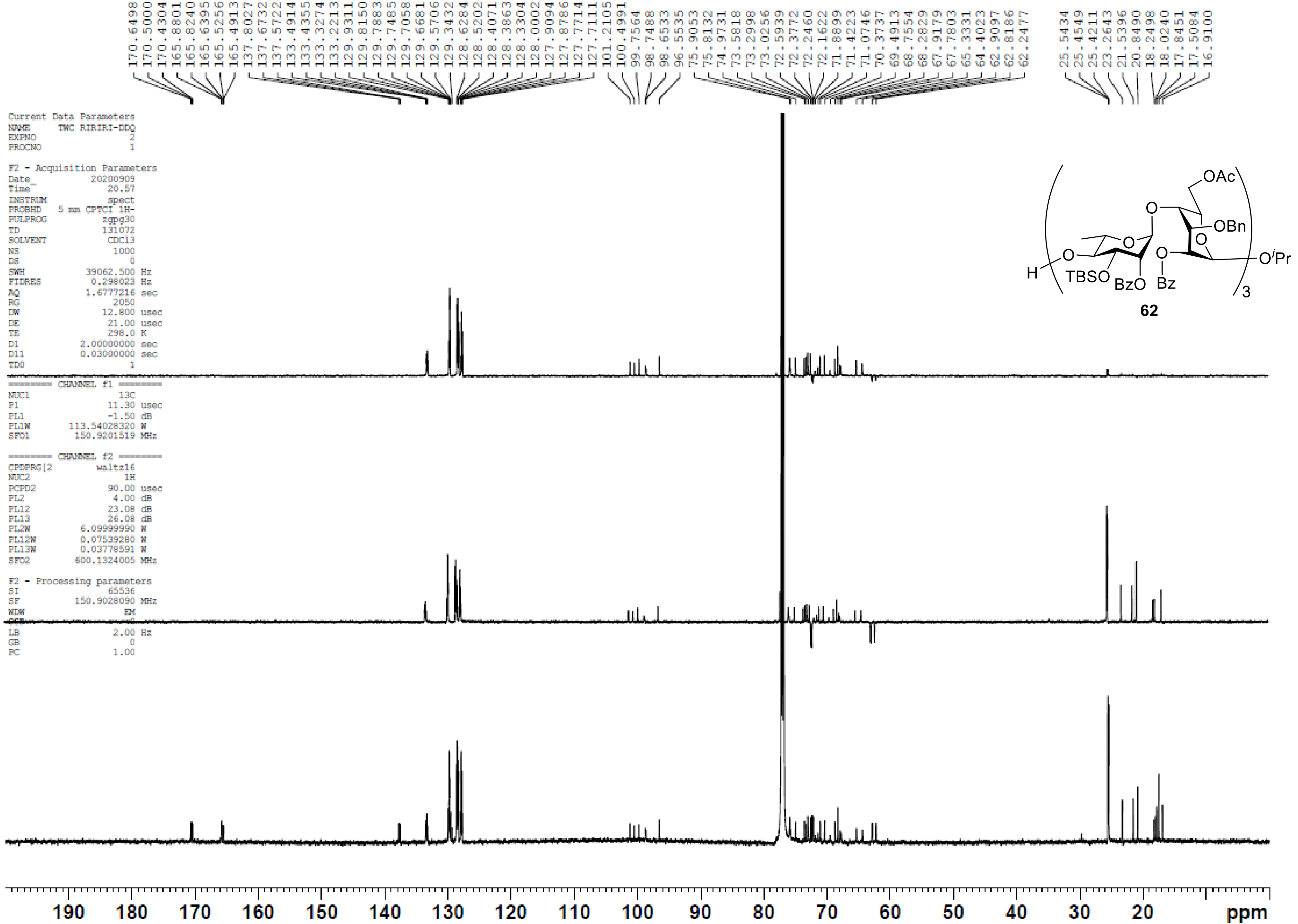
170.6498
170.5000
170.4304
165.8801
165.8240
165.6395
165.5256
165.4913
137.8027
137.6732
137.5722
133.4914
133.4355
133.3274
133.2213
129.9311
129.8150
129.7883
129.7485
129.7058
129.6681
129.5706
129.3432
128.6284
128.5202
128.4071
128.3863
128.3304
128.0002
127.9094
127.8786
127.7714
127.7111
101.2105
100.4991
99.7564
98.7488
98.6533
96.5535
75.9053
75.8132
74.9731
73.5818
73.2998
73.0256
72.5939
72.3772
72.2460
72.1622
71.8899
71.4223
71.0746
70.3737
69.4913
68.7554
68.2829
67.9179
67.7803
65.3331
64.4023
62.9097
62.8186
62.2477
25.5434
25.4549
25.4211
23.2643
21.5396
20.8490
18.2498
18.0240
17.8451
17.5084
16.9100
Current Data Parameters
NAME TWC RIRIRI-DDQ
EXPNO 2
PROCNO 1
F2 - Acquisition Parameters
Date_ 20200909
Time 20.57
INSTRUM spect
PROBHD 5 mm CPTCI 1H-
PULPROG zgpg30
TD 131072
SOLVENT CDCl3
NS 1000
DS 0
SWH 39062.500 Hz
FIDRES 0.298023 Hz
AQ 1.6777216 sec
RG 2050
DW 12.800 usec
DE 21.00 usec
TE 298.0 K
D1 2.00000000 sec
D11 0.03000000 sec
TD0 1
======== CHANNEL f1 ========
NUC1 13C
P1 11.30 usec
PL1 -1.50 dB
PL1W 113.54028320 W
SFO1 150.9201519 MHz
======== CHANNEL f2 ========
CPDPRG[2 waltz16
NUC2 1H
PCPD2 90.00 usec
PL2 4.00 dB
PL12 23.08 dB
PL13 26.08 dB
PL2W 6.09999990 W
PL12W 0.07539280 W
PL13W 0.03778591 W
SFO2 600.1324005 MHz
F2 - Processing parameters
SI 65536
SF 150.9028090 MHz
WDW EM
LB 2.00 Hz
GB 0
PC 1.00
OAc
O
OBn
O
O
O
O
O^{i}Pr
H
TBSO
BzO
Bz
3
62
190 180 170 160 150 140 130 120 110 100 90 80 70 60 50 40 30 20 ppm

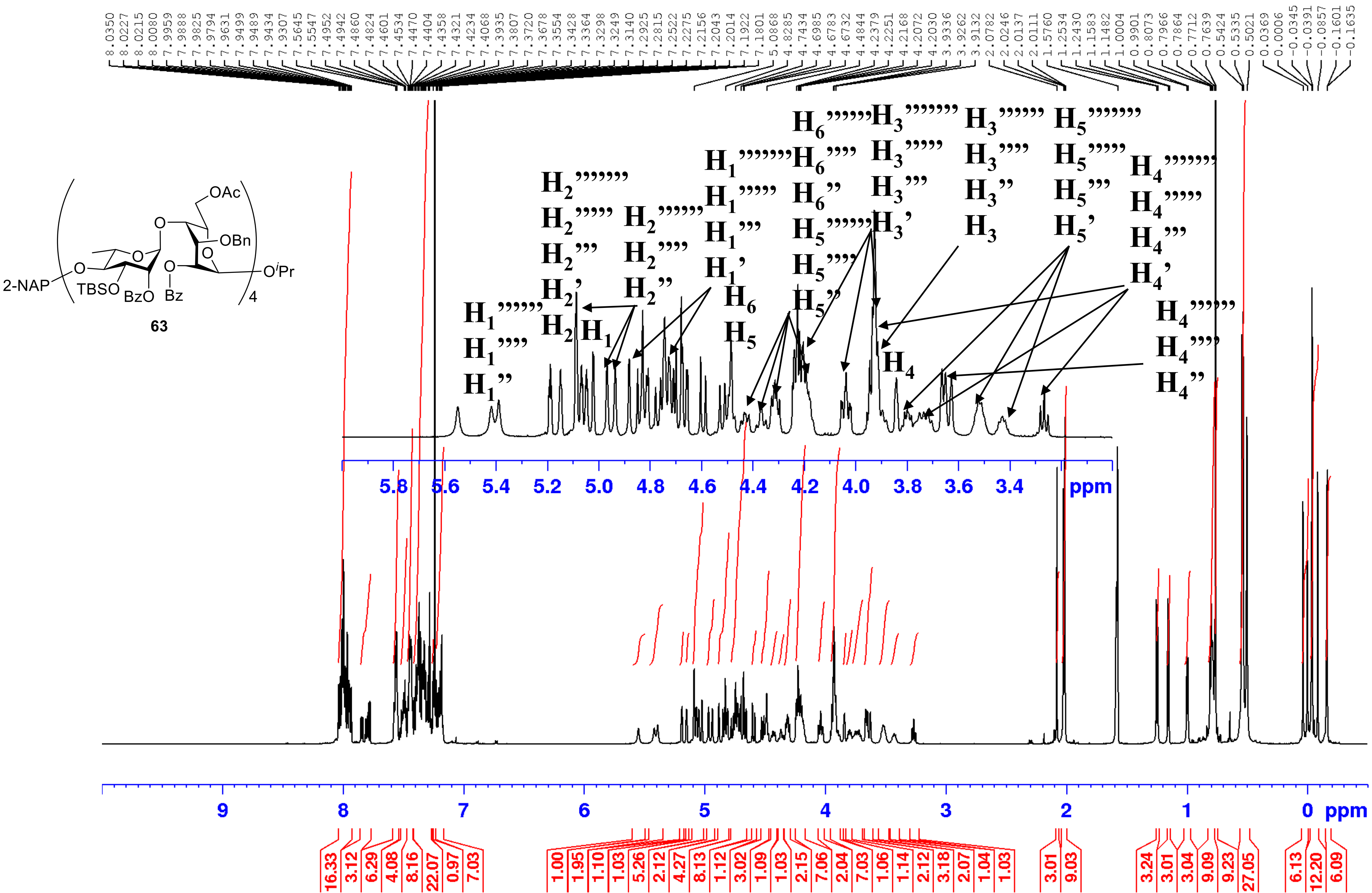

63

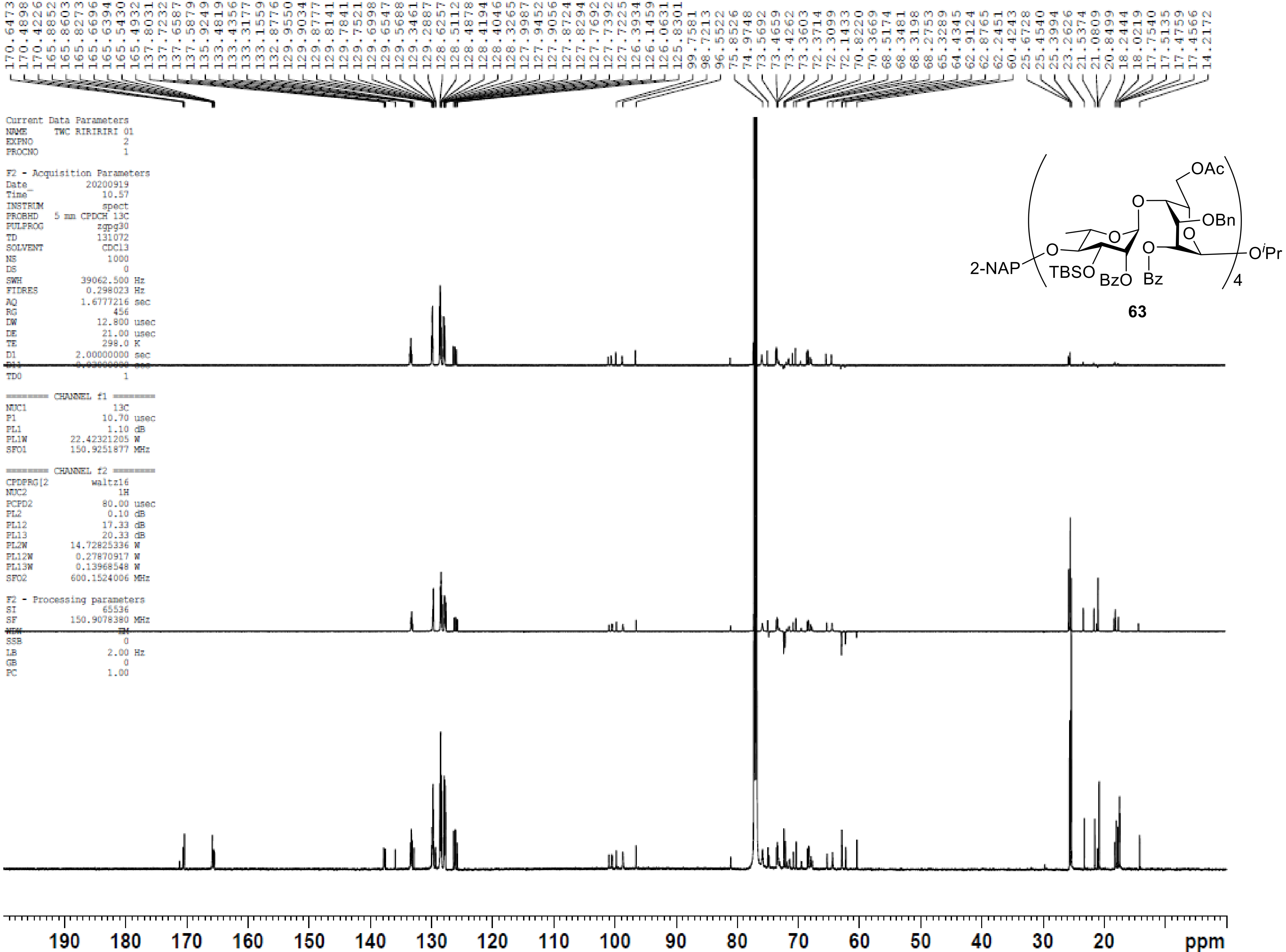
170.6473
170.4898
170.4226
165.8852
165.8603
165.8273
165.6696
165.6394
165.5430
165.4932
137.8031
137.7232
137.6587
137.5879
135.9249
133.4819
133.4356
133.3177
133.1559
132.8776
129.9550
129.9034
129.8777
129.8141
129.7841
129.7521
129.6998
129.6547
129.5688
129.3461
129.2887
128.6257
128.5112
128.4878
128.4194
128.4046
128.3265
127.9987
127.9452
127.9056
127.8724
127.8294
127.7692
127.7392
127.7225
126.3934
126.1459
126.0631
125.8301
99.7581
98.7213
96.5522
75.8526
74.9748
73.5692
73.4659
73.4262
73.3603
72.3714
72.3099
72.1433
70.8220
70.3669
68.5174
68.3481
68.3198
68.2753
65.3289
64.4345
62.9124
62.8765
62.2451
60.4243
25.4540
25.3994
23.2626
21.5374
21.0809
20.8499
18.2444
18.0219
17.7540
17.5135
17.4759
17.4566
14.2172
Current Data Parameters
NAME TWC RIRIRIRI 01
EXPNO 2
PROCNO 1
F2 - Acquisition Parameters
Date_ 20200919
Time 10.57
INSTRUM spect
PROBHD 5 mm CPDCH 13C
PULPROG zgpg30
TD 131072
SOLVENT CDCl3
NS 1000
DS 0
SWH 39062.500 Hz
FIDRES 0.298023 Hz
AQ 1.6777216 sec
RG 456
DW 12.800 usec
DE 21.00 usec
TE 298.0 K
D1 2.00000000 sec
TD0 1
======== CHANNEL f1 ========
NUC1 13C
P1 10.70 usec
PL1 1.10 dB
PL1W 22.42321205 W
SFO1 150.9251877 MHz
======== CHANNEL f2 ========
CPDPRG[2 waltz16
NUC2 1H
PCPD2 80.00 usec
PL2 0.10 dB
PL12 17.33 dB
PL13 20.33 dB
PL2W 14.72825336 W
PL12W 0.27870917 W
PL13W 0.13968548 W
SFO2 600.1524006 MHz
F2 - Processing parameters
SI 65536
SF 150.9078380 MHz
SSB 0
LB 2.00 Hz
GB 0
PC 1.00
OAc
O
OBn
O
O
O
O
O^{i}Pr
2-NAP
TBSO
BzO
Bz
4
63
190 180 170 160 150 140 130 120 110 100 90 80 70 60 50 40 30 20 ppm

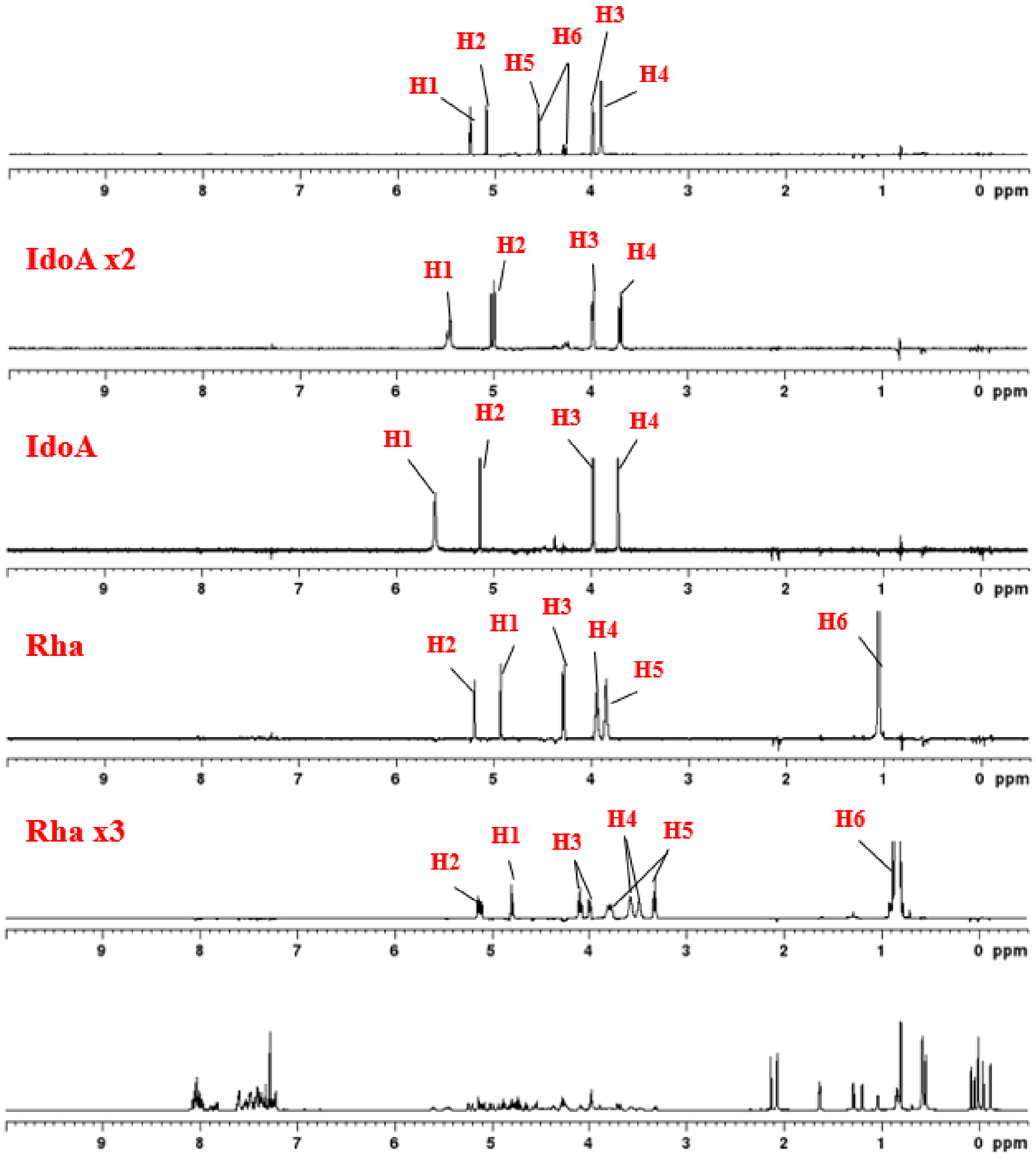
H1
H2
H5
H6
H3
H4
IdoA x2
IdoA
Rha
Rha x3
ppm

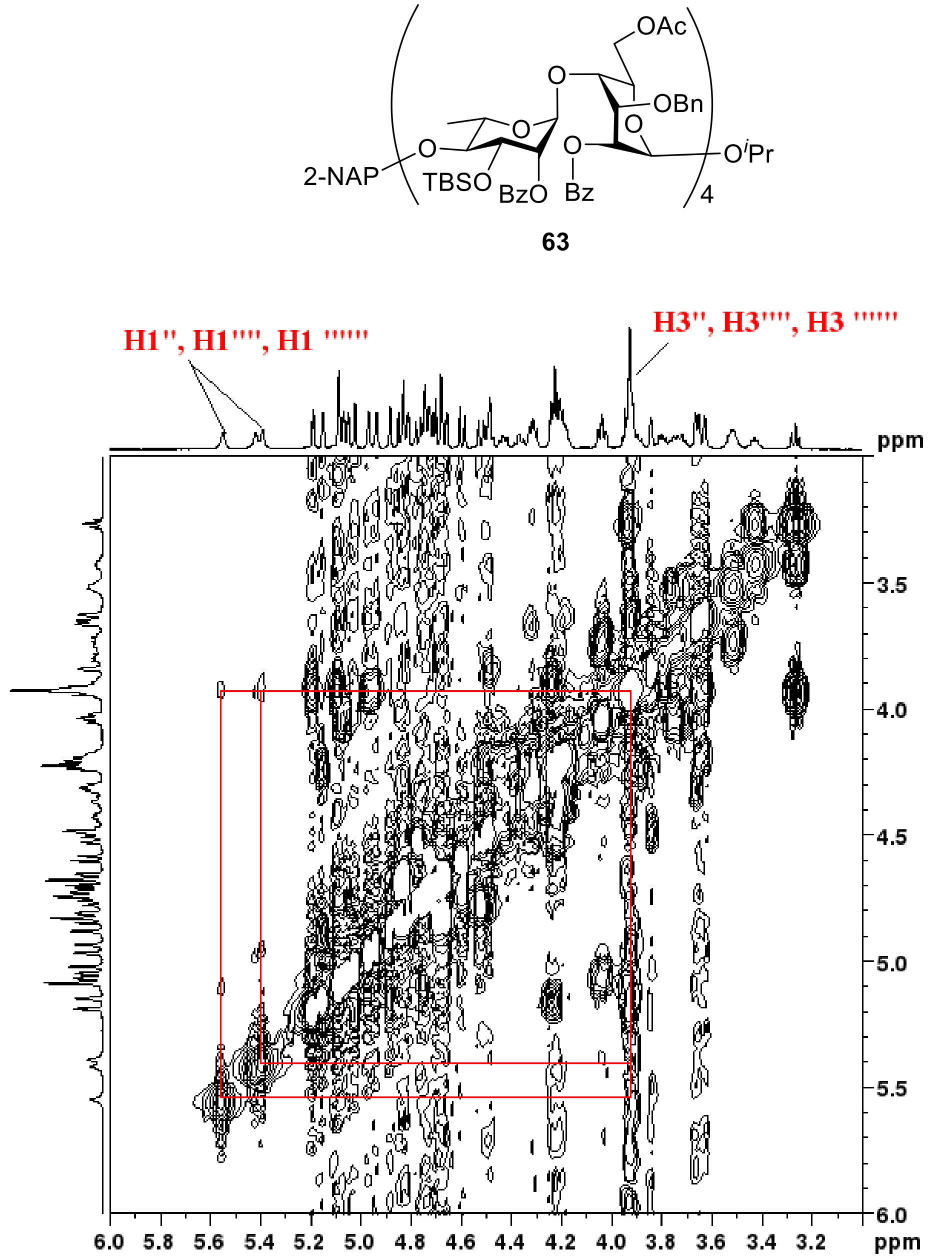
OAc
OBn
O^{i}Pr
2-NAP
TBSO
BzO
Bz
4
H1", H1"", H1 """
H3", H3"", H3 """
ppm

63

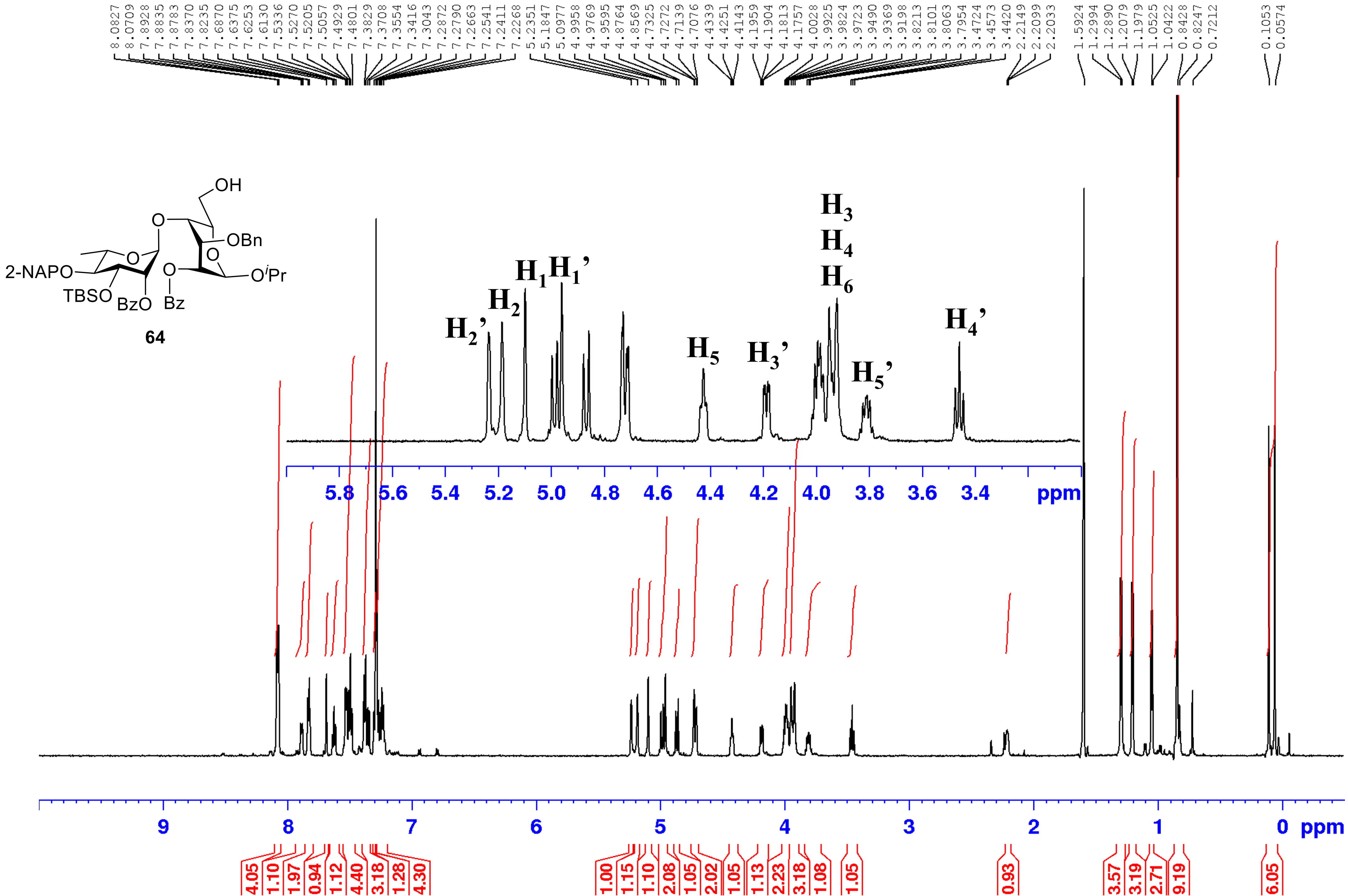

H2'
H2
H1
H1'
H5
H3'
H3
H4
H6
H5'
H4'
ppm

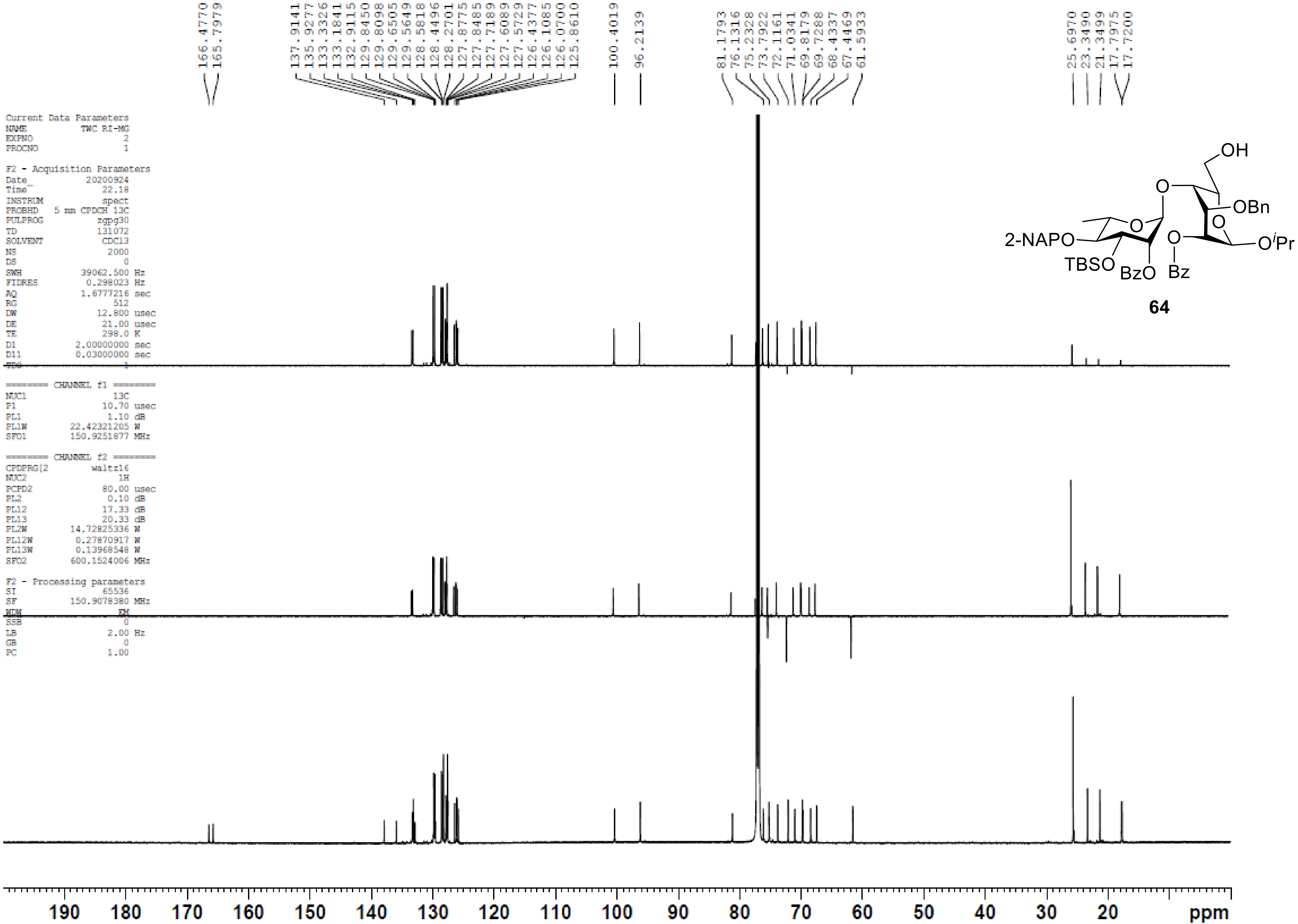

166.4770
165.7979
137.9141
135.9277
133.3326
133.1841
132.9115
129.8450
129.8098
129.6505
129.5649
128.5818
128.4496
128.2701
127.8775
127.8485
127.7189
127.6089
127.5729
126.4377
126.1085
126.0700
125.8610
100.4019
96.2139
81.1793
76.1316
75.2328
73.7922
72.1161
71.0341
69.8179
69.7288
68.4337
67.4469
61.5933
25.6970
23.3490
21.3499
17.7975
17.7200
Current Data Parameters
NAME TWC RI-MG
EXPNO 2
PROCNO 1
F2 - Acquisition Parameters
Date_ 20200924
Time 22.18
INSTRUM spect
PROBHD 5 mm CPDCH 13C
PULPROG zgpg30
TD 131072
SOLVENT CDCl3
NS 2000
DS 0
SWH 39062.500 Hz
FIDRES 0.298023 Hz
AQ 1.6777216 sec
RG 512
DW 12.800 usec
DE 21.00 usec
TE 298.0 K
D1 2.00000000 sec
D11 0.03000000 sec
TD0 1
======== CHANNEL f1 ========
NUC1 13C
P1 10.70 usec
PL1 1.10 dB
PL1W 22.42321205 W
SFO1 150.9251877 MHz
======== CHANNEL f2 ========
CPDPRG[2 waltz16
NUC2 1H
PCPD2 80.00 usec
PL2 0.10 dB
PL12 17.33 dB
PL13 20.33 dB
PL2W 14.72825336 W
PL12W 0.27870917 W
PL13W 0.13968548 W
SFO2 600.1524006 MHz
F2 - Processing parameters
SI 65536
SF 150.9078380 MHz
WDW EM
SSB 0
LB 2.00 Hz
GB 0
PC 1.00
OH
O
OBn
O
O
2-NAPO
O
O^{i}Pr
TBSO
BzO
Bz
64
190
180
170
160
150
140
130
120
110
100
90
80
70
60
50
40
30
20
ppm

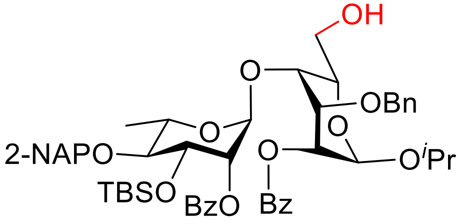
OH
O
OBn
O
2-NAPO
O
O
O'Pr
TBSO
BzO
Bz

64

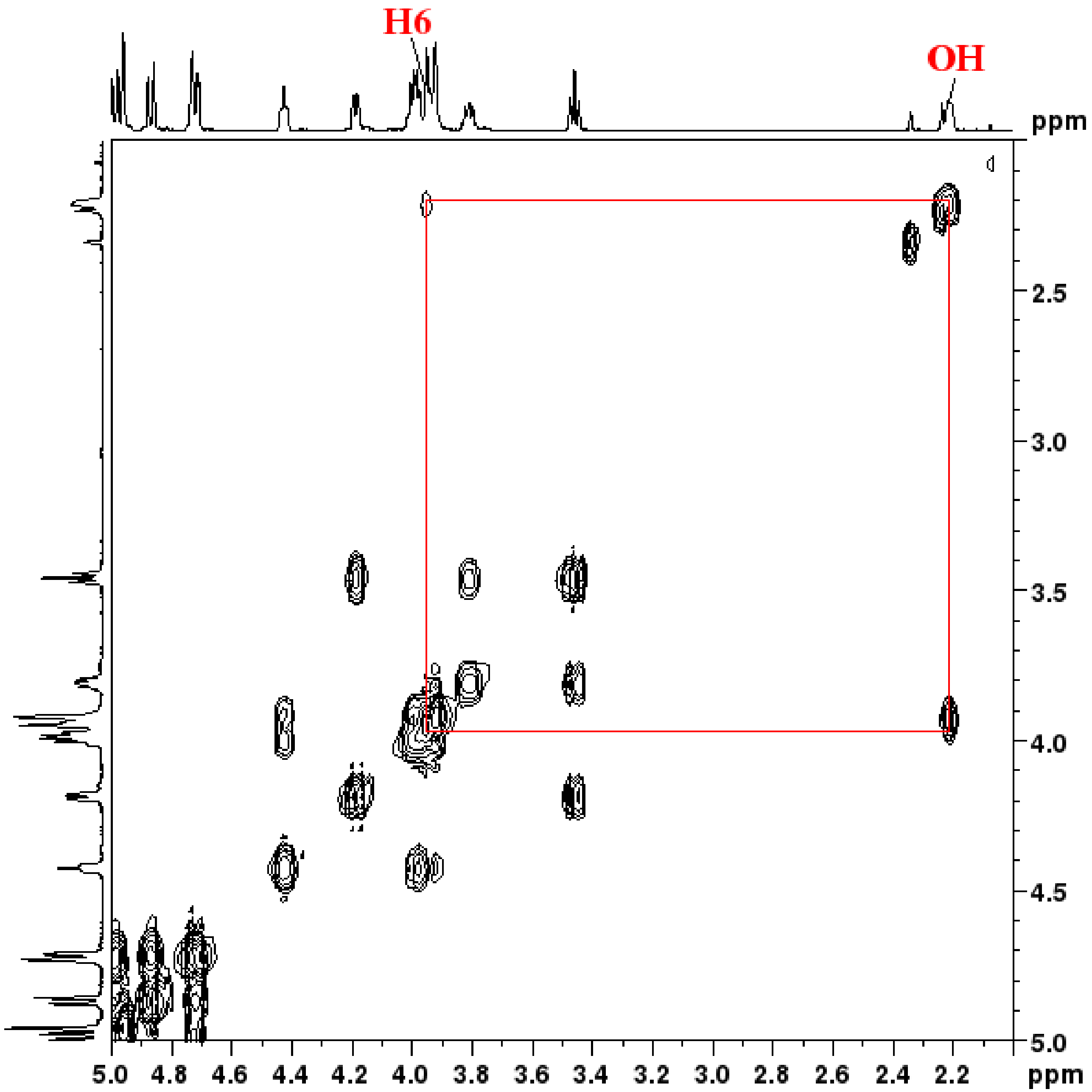
H6
OH
ppm
2.5
3.0
3.5
4.0
4.5
5.0
5.0 4.8 4.6 4.4 4.2 4.0 3.8 3.6 3.4 3.2 3.0 2.8 2.6 2.4 2.2
ppm

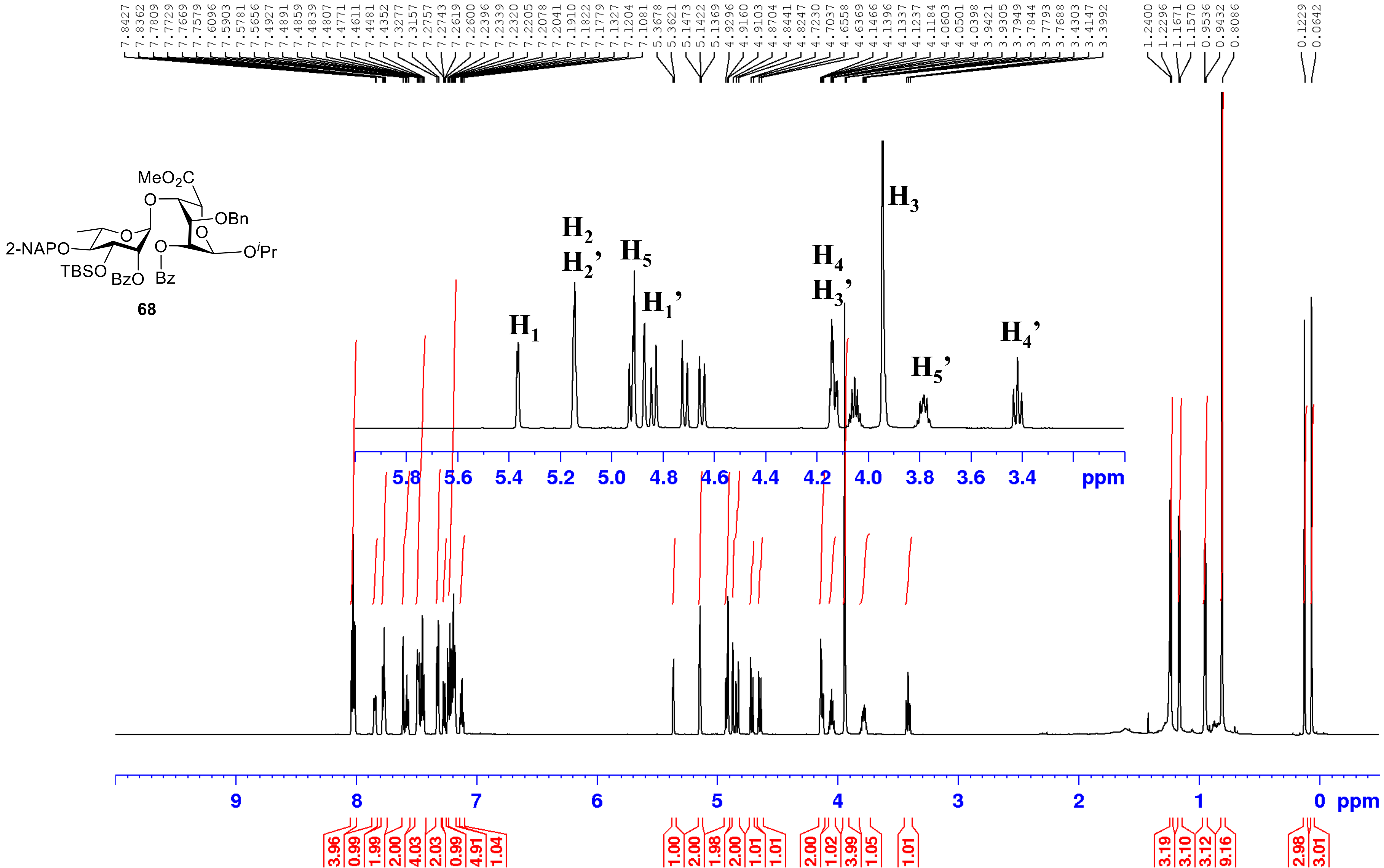
MeO2C
OBn
2-NAPO
TBSO
BzO
Bz
O^iPr
68
H1
H2
H2'
H5
H1'
H4
H3'
H3
H5'
H4'
ppm

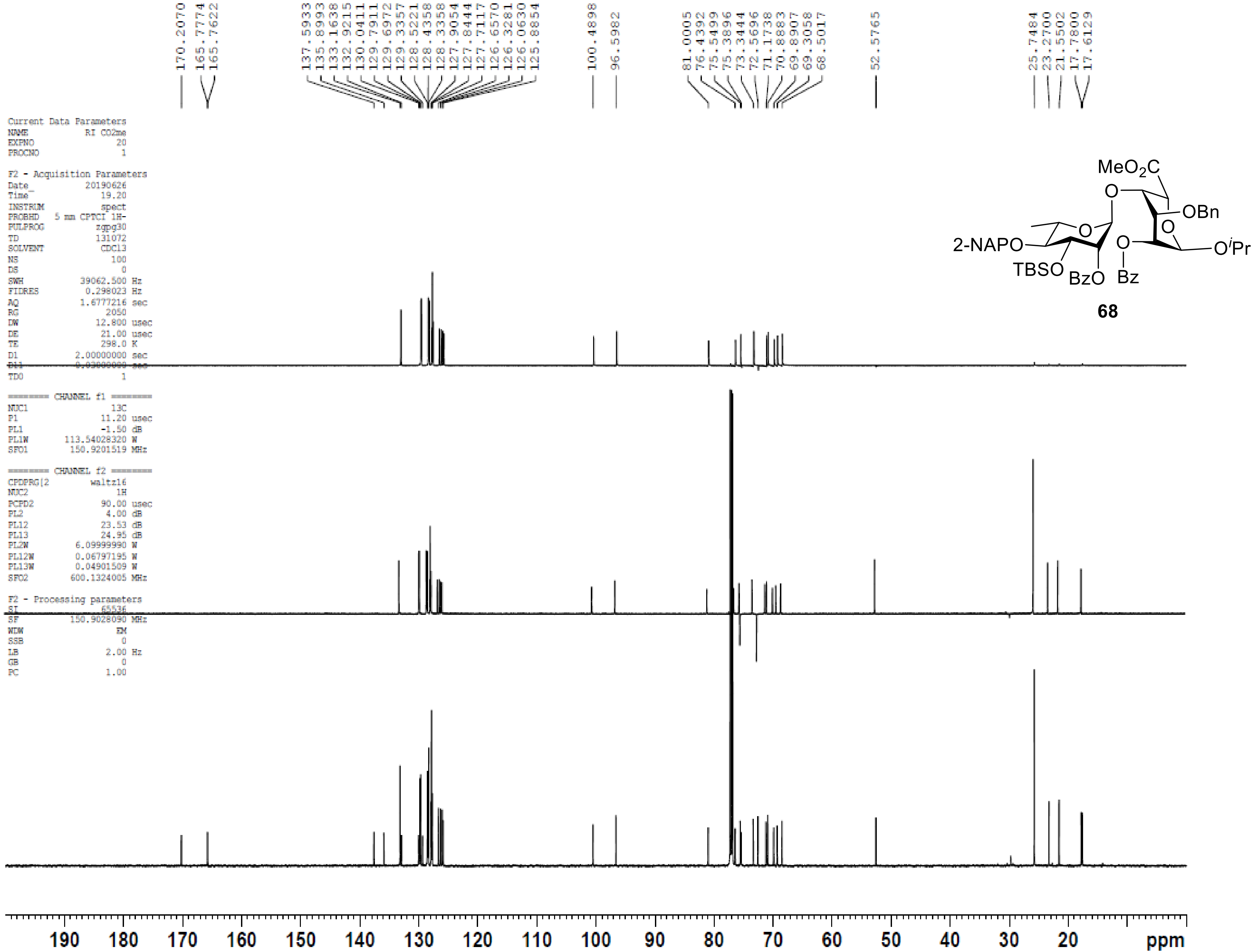
170.2070
165.7774
165.7622
137.5933
135.8993
133.1638
132.9215
130.0411
129.7911
129.6972
129.3357
128.5221
128.4358
128.3358
127.9054
127.8444
127.7117
126.6570
126.3281
126.0630
125.8854
100.4898
96.5982
81.0005
76.4392
75.5499
75.3896
73.3444
72.5696
71.1738
70.8883
69.8907
69.3058
68.5017
52.5765
25.7484
23.2700
21.5502
17.7800
17.6129
Current Data Parameters
NAME RI CO2me
EXPNO 20
PROCNO 1
F2 - Acquisition Parameters
Date_ 20190626
Time 19.20
INSTRUM spect
PROBHD 5 mm CPTCI 1H-
PULPROG zgpg30
TD 131072
SOLVENT CDCl3
NS 100
DS 0
SWH 39062.500 Hz
FIDRES 0.298023 Hz
AQ 1.6777216 sec
RG 2050
DW 12.800 usec
DE 21.00 usec
TE 298.0 K
D1 2.00000000 sec
TD0 1
======== CHANNEL f1 ========
NUC1 13C
P1 11.20 usec
PL1 -1.50 dB
PL1W 113.54028320 W
SFO1 150.9201519 MHz
======== CHANNEL f2 ========
CPDPRG[2 waltz16
NUC2 1H
PCPD2 90.00 usec
PL2 4.00 dB
PL12 23.53 dB
PL13 24.95 dB
PL2W 6.09999990 W
PL12W 0.06797195 W
PL13W 0.04901509 W
SFO2 600.1324005 MHz
F2 - Processing parameters
SI 65536
SF 150.9028090 MHz
WDW EM
SSB 0
LB 2.00 Hz
GB 0
PC 1.00
190 180 170 160 150 140 130 120 110 100 90 80 70 60 50 40 30 20 ppm
MeO2C
OBn
2-NAPO
O
O
O
OiPr
TBSO
BzO
Bz

68

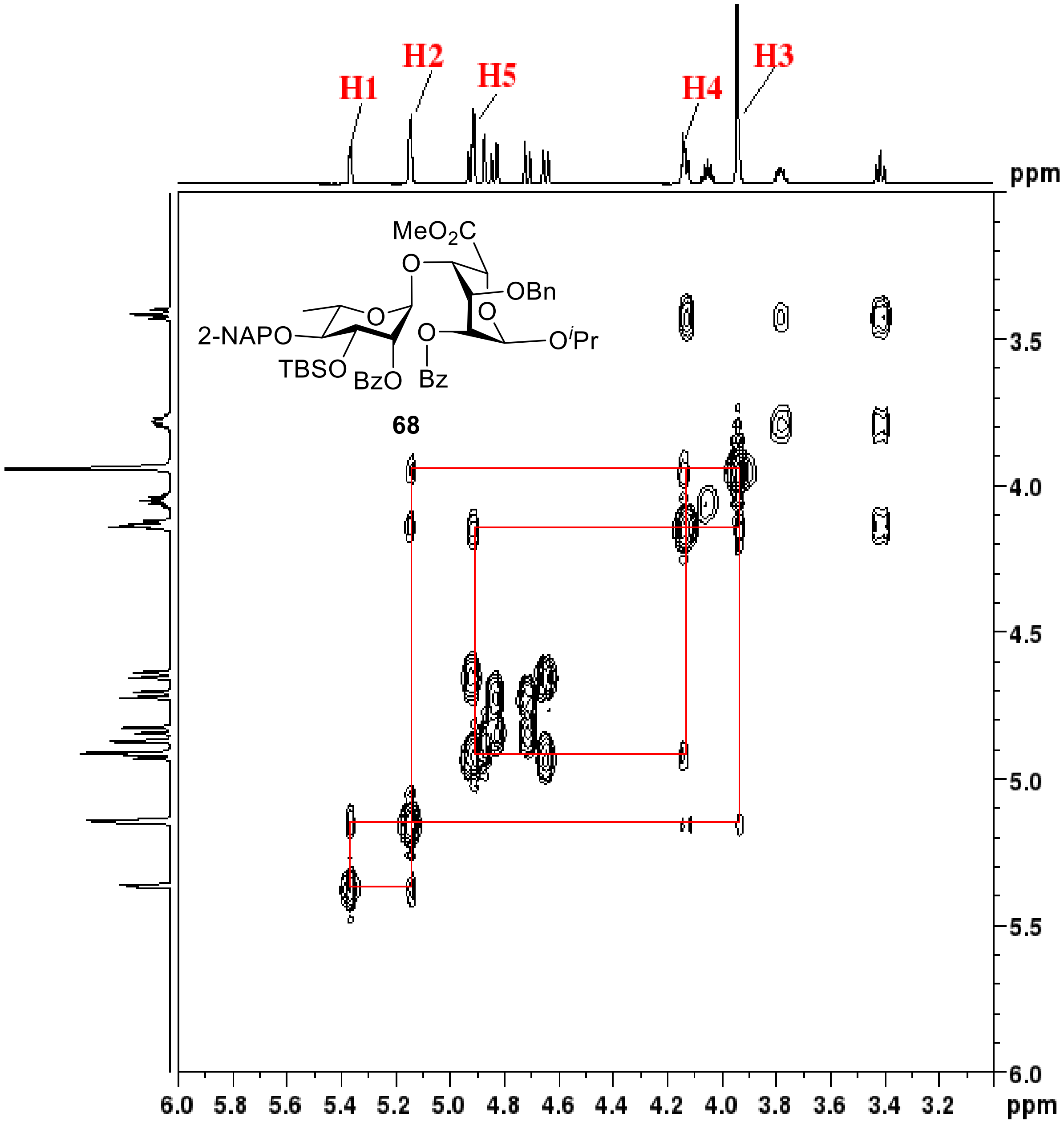
H1
H2
H5
H4
H3
ppm
MeO2C
O
OBn
O
O
2-NAPO
O
O
OiPr
TBSO
BzO
Bz
68
3.5
4.0
4.5
5.0
5.5
6.0
6.0 5.8 5.6 5.4 5.2 5.0 4.8 4.6 4.4 4.2 4.0 3.8 3.6 3.4 3.2
ppm

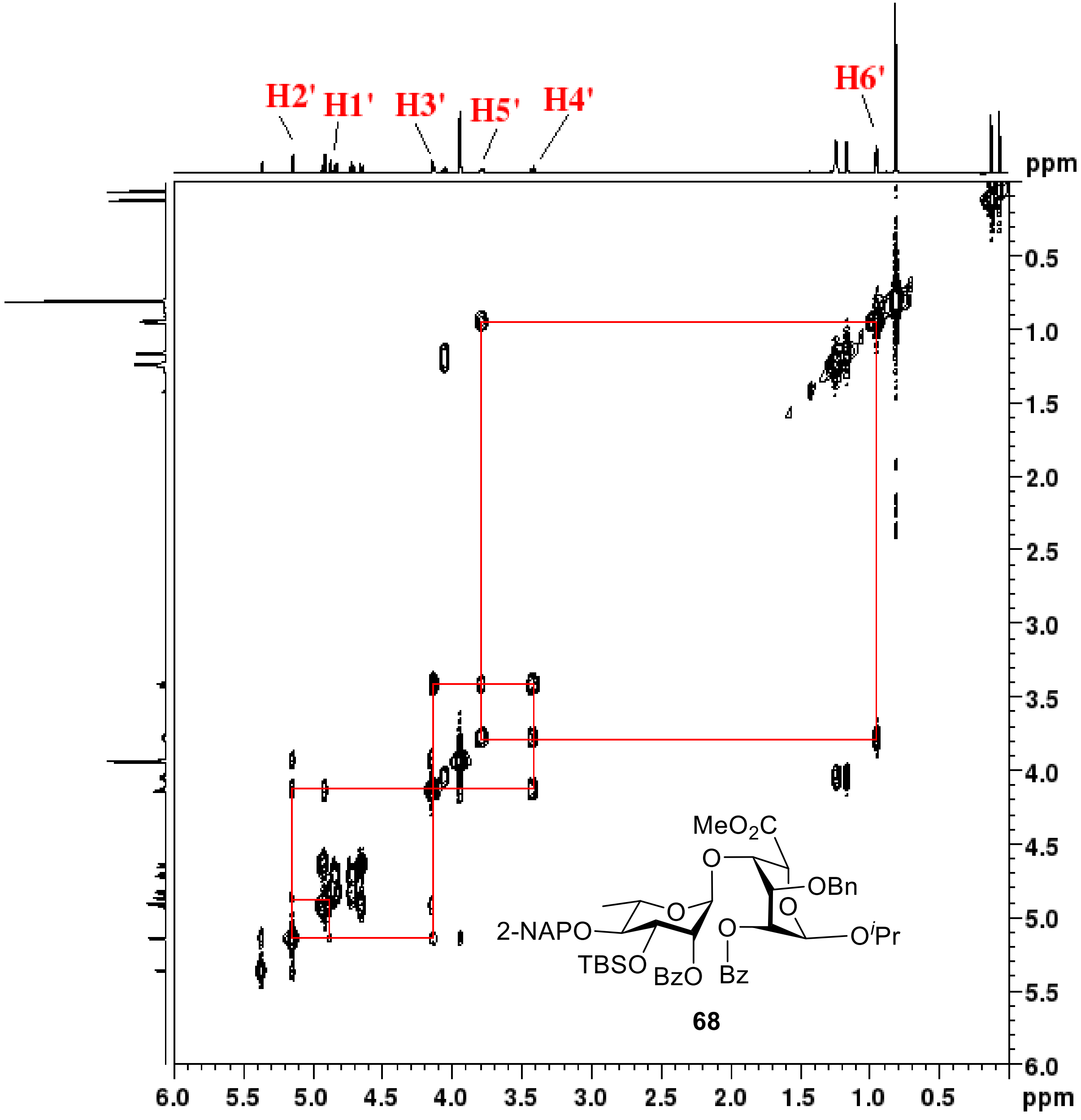
H2'
H1'
H3'
H5'
H4'
H6'
ppm
0.5
1.0
1.5
2.0
2.5
3.0
3.5
4.0
4.5
5.0
5.5
6.0
MeO2C
O
OBn
O
O
2-NAPO
O
O
OiPr
TBSO
BzO
Bz
68
6.0 5.5 5.0 4.5 4.0 3.5 3.0 2.5 2.0 1.5 1.0 0.5
ppm

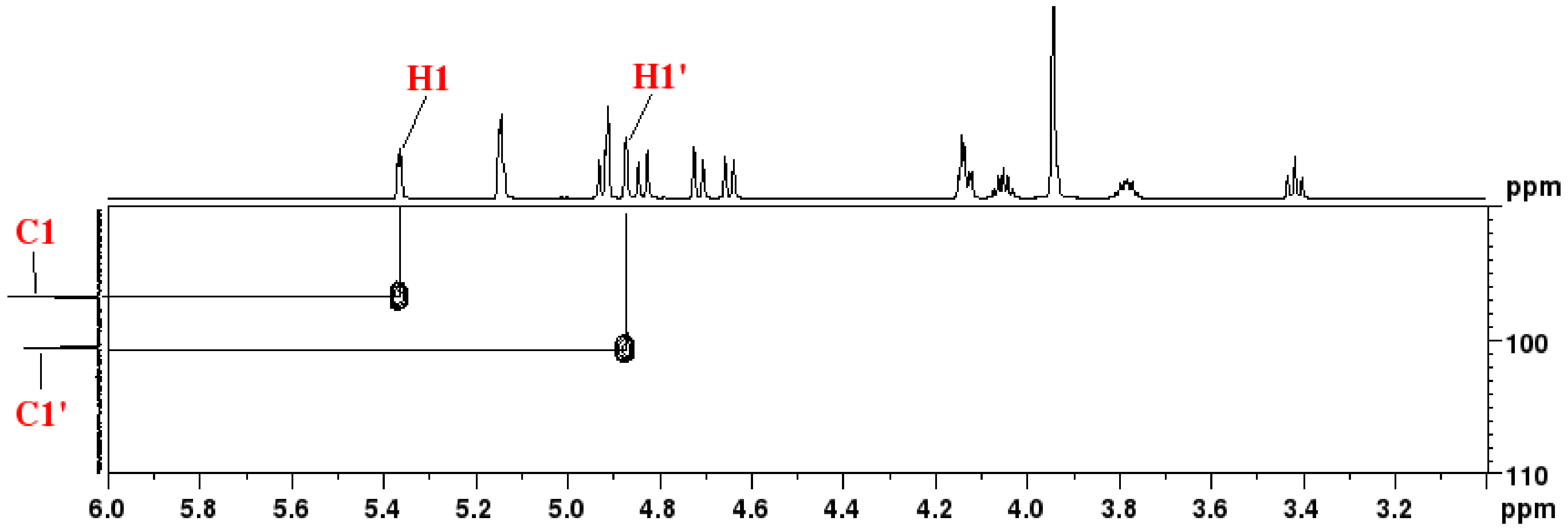
H1
H1'
C1
C1'
ppm
100
110
6.0
5.8
5.6
5.4
5.2
5.0
4.8
4.6
4.4
4.2
4.0
3.8
3.6
3.4
3.2
ppm

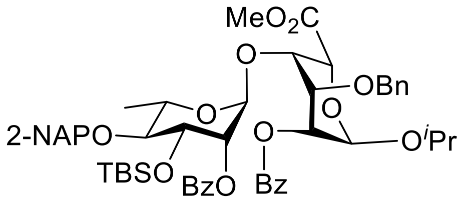
MeO2C
O
OBn
O
2-NAPO
O
O
OⁱPr
TBSO
BzO
Bz

68

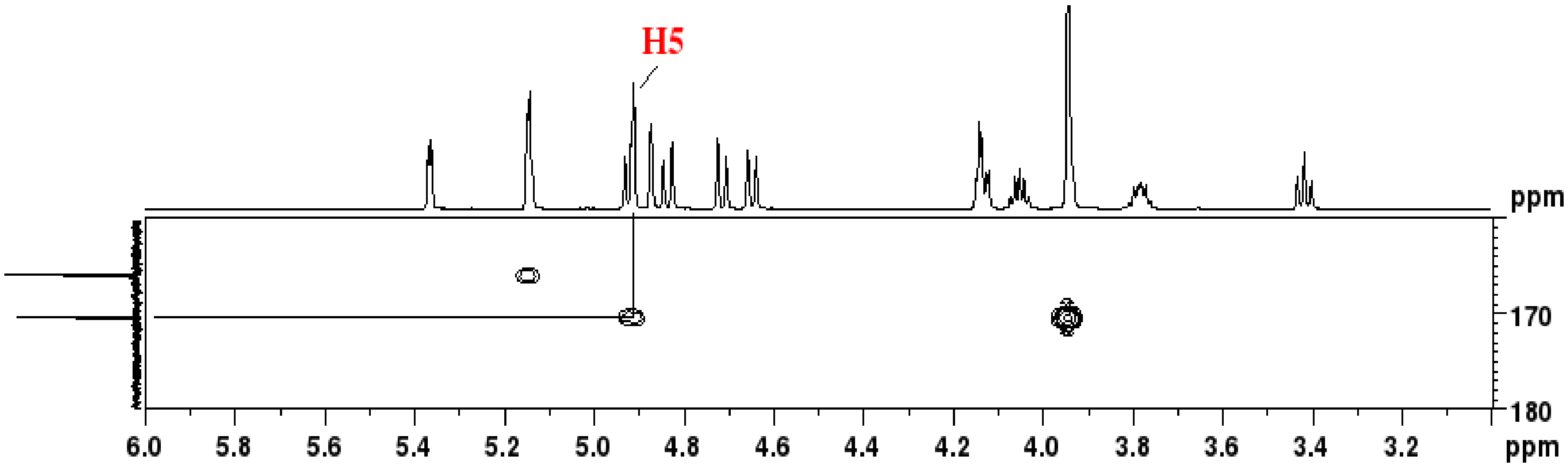
H5
ppm
170
180
6.0
5.8
5.6
5.4
5.2
5.0
4.8
4.6
4.4
4.2
4.0
3.8
3.6
3.4
3.2
ppm

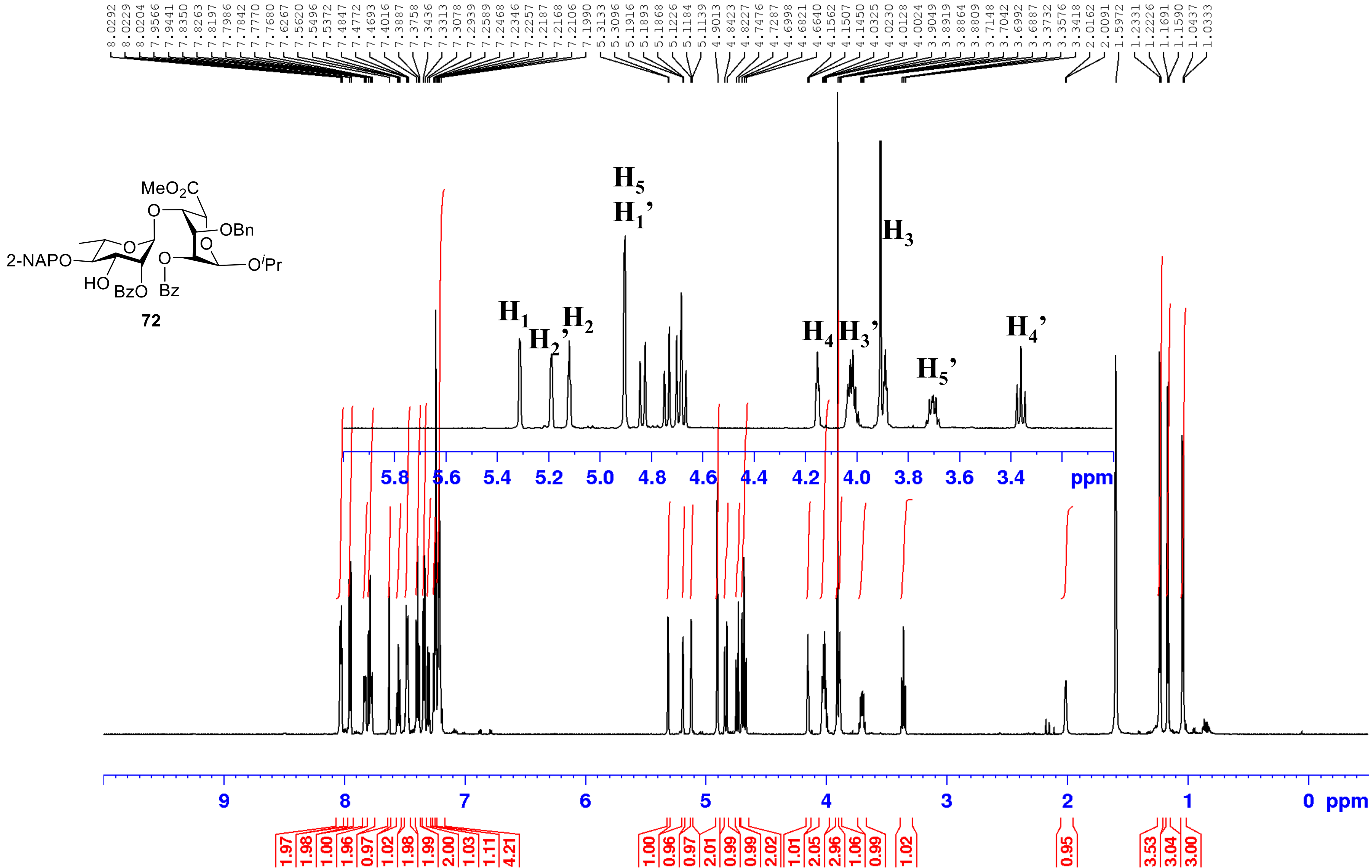
MeO2C
OBn
2-NAPO
HO
BzO
Bz
O^iPr
72
H5
H1'
H1
H2'
H2
H3
H4
H3'
H5'
H4'
ppm

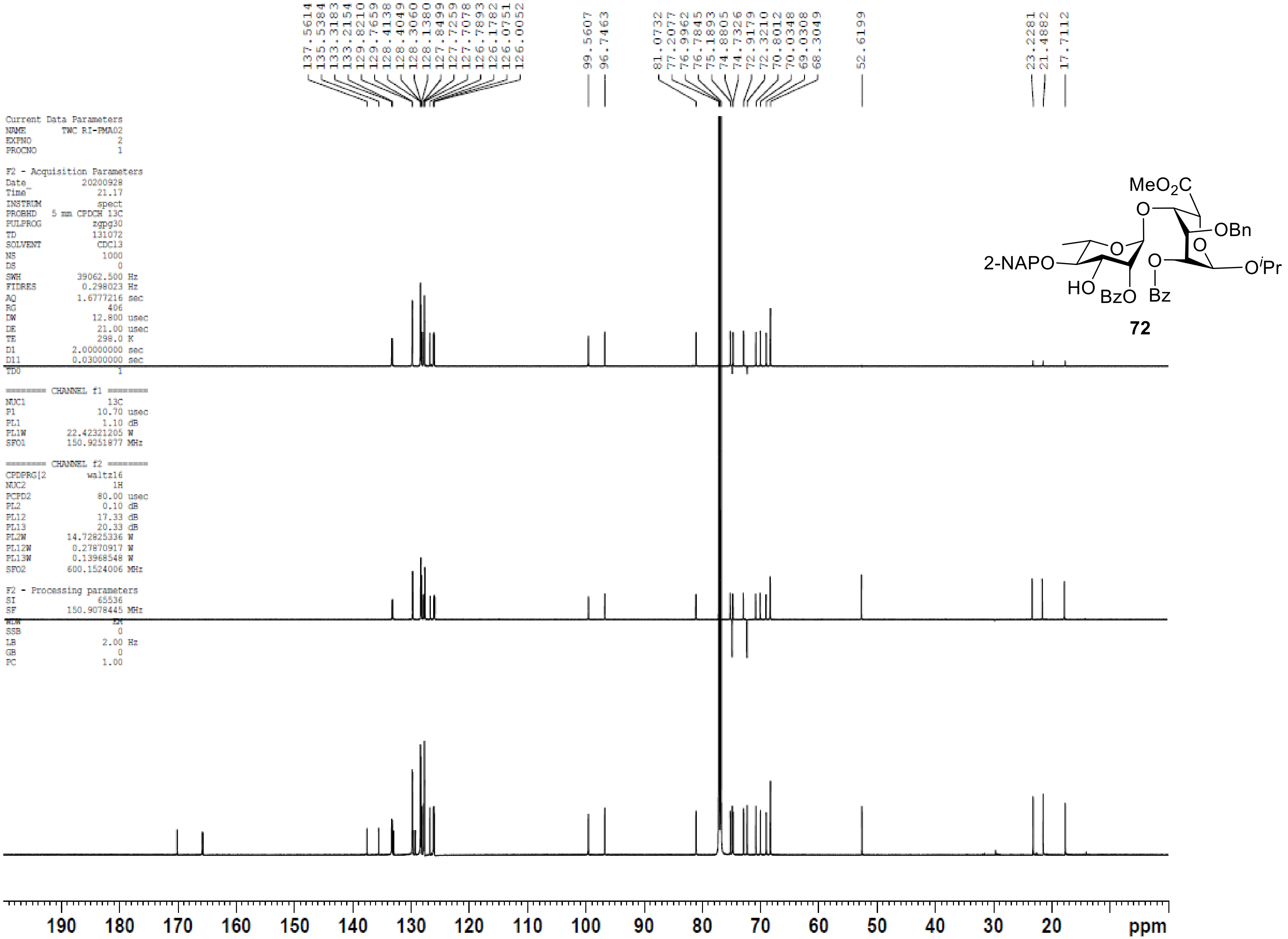
137.5614
135.5384
133.3183
133.2154
129.8210
129.7659
128.4138
128.4049
128.3060
128.1380
127.8499
127.7259
127.7078
126.7893
126.1782
126.0751
126.0052
99.5607
96.7463
81.0732
77.2077
76.9962
76.7845
75.1893
74.8805
74.7326
72.9179
72.3210
70.8012
70.0348
69.0308
68.3049
52.6199
23.2281
21.4882
17.7112
Current Data Parameters
NAME TWC RI-PMA02
EXPNO 2
PROCNO 1
F2 - Acquisition Parameters
Date_ 20200928
Time 21.17
INSTRUM spect
PROBHD 5 mm CPDCH 13C
PULPROG zgpg30
TD 131072
SOLVENT CDCl3
NS 1000
DS 0
SWH 39062.500 Hz
FIDRES 0.298023 Hz
AQ 1.6777216 sec
RG 406
DW 12.800 usec
DE 21.00 usec
TE 298.0 K
D1 2.00000000 sec
D11 0.03000000 sec
TD0 1
======== CHANNEL f1 ========
NUC1 13C
P1 10.70 usec
PL1 1.10 dB
PL1W 22.42321205 W
SFO1 150.9251877 MHz
======== CHANNEL f2 ========
CPDPRG[2 waltz16
NUC2 1H
PCPD2 80.00 usec
PL2 0.10 dB
PL12 17.33 dB
PL13 20.33 dB
PL2W 14.72825336 W
PL12W 0.27870917 W
PL13W 0.13968548 W
SFO2 600.1524006 MHz
F2 - Processing parameters
SI 65536
SF 150.9078445 MHz
WDW EM
SSB 0
LB 2.00 Hz
GB 0
PC 1.00
MeO2C
O
OBn
O
2-NAPO
O
O
O-iPr
HO
BzO
Bz
190 180 170 160 150 140 130 120 110 100 90 80 70 60 50 40 30 20 ppm

72

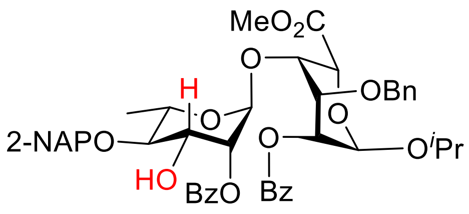

MeO2C
OBn
H
O
2-NAPO
O
O
OiPr
HO
BzO
Bz

72

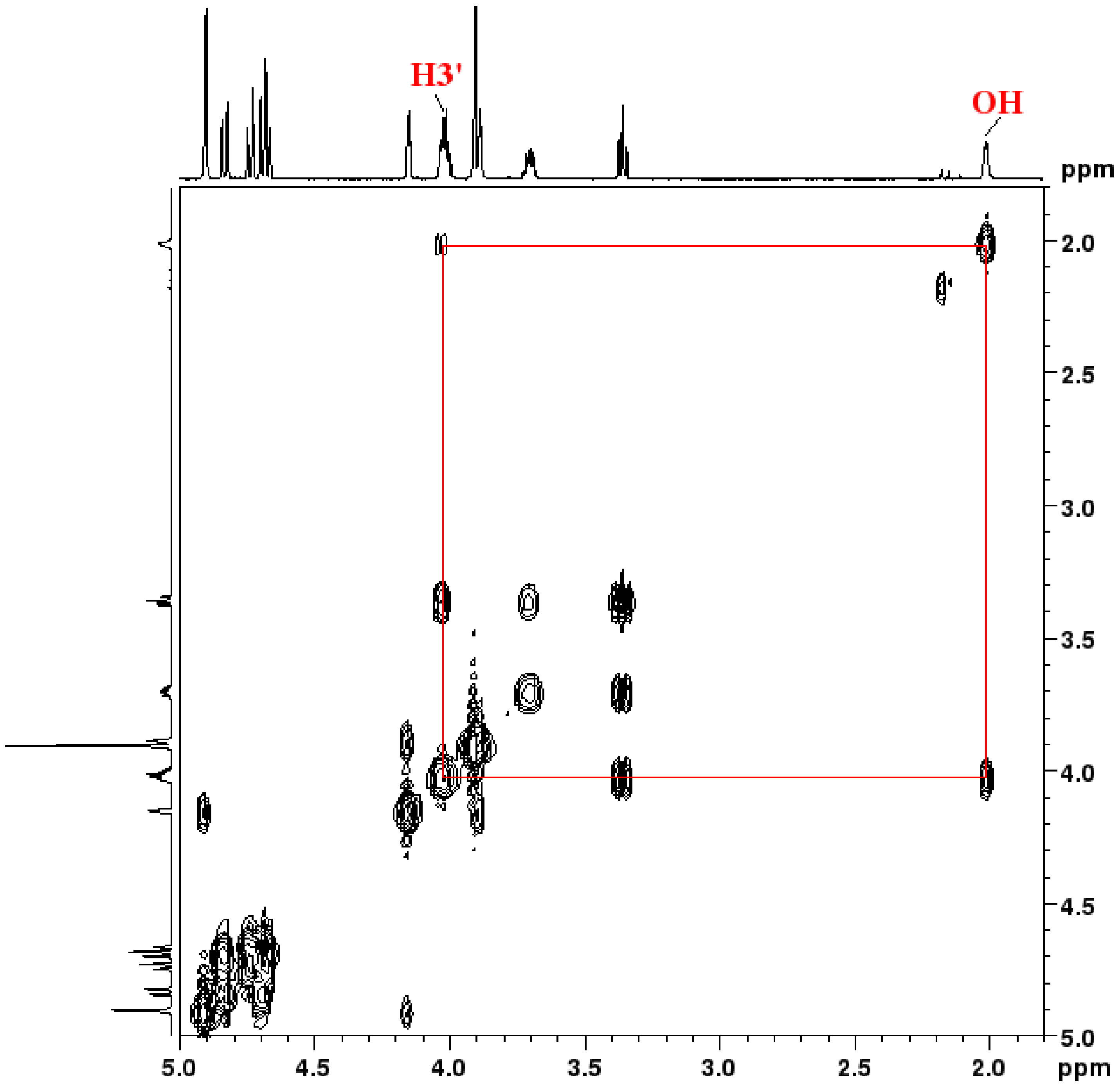

H3'
OH
ppm
2.0
2.5
3.0
3.5
4.0
4.5
5.0
5.0
4.5
4.0
3.5
3.0
2.5
2.0
ppm

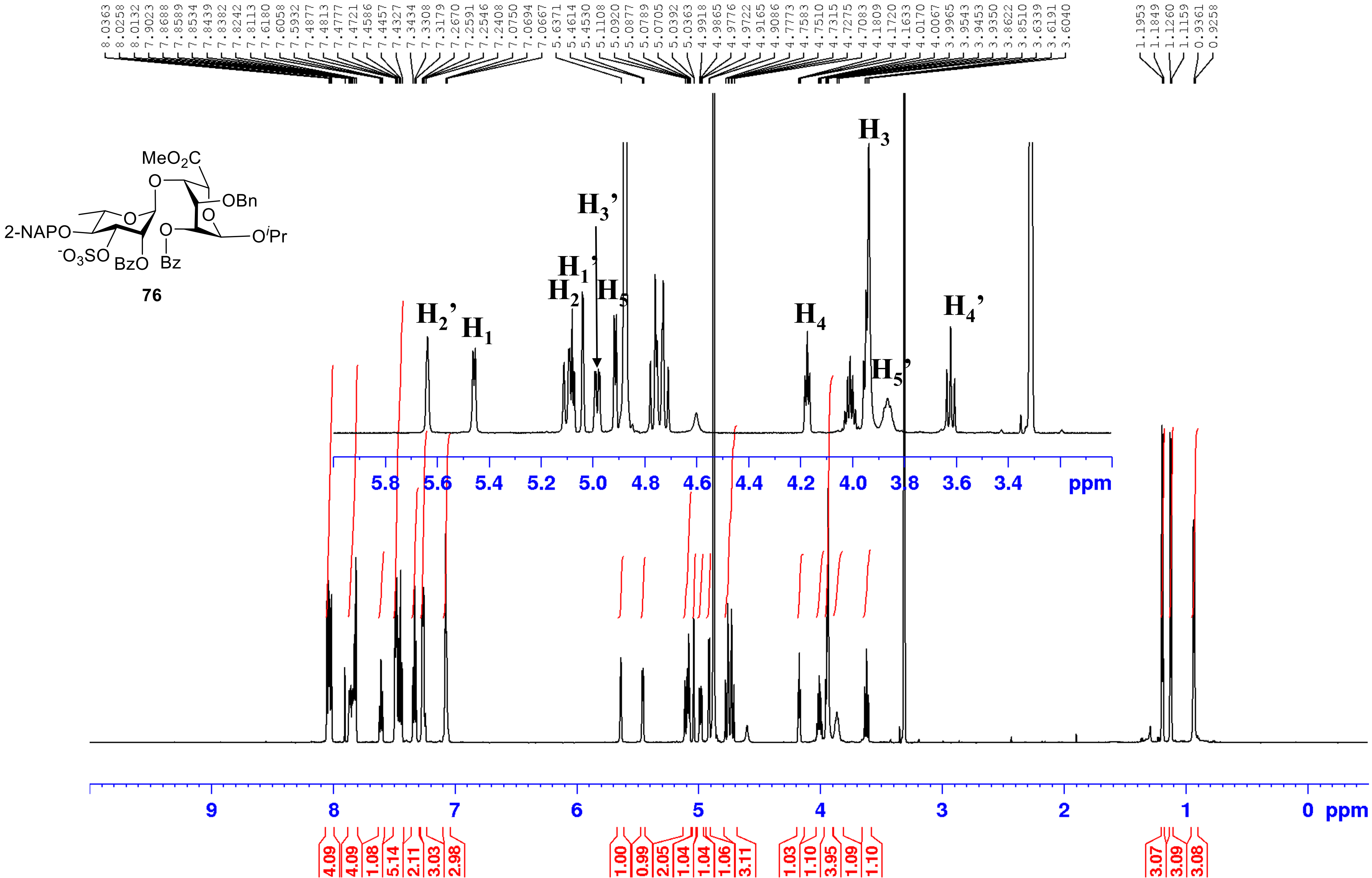

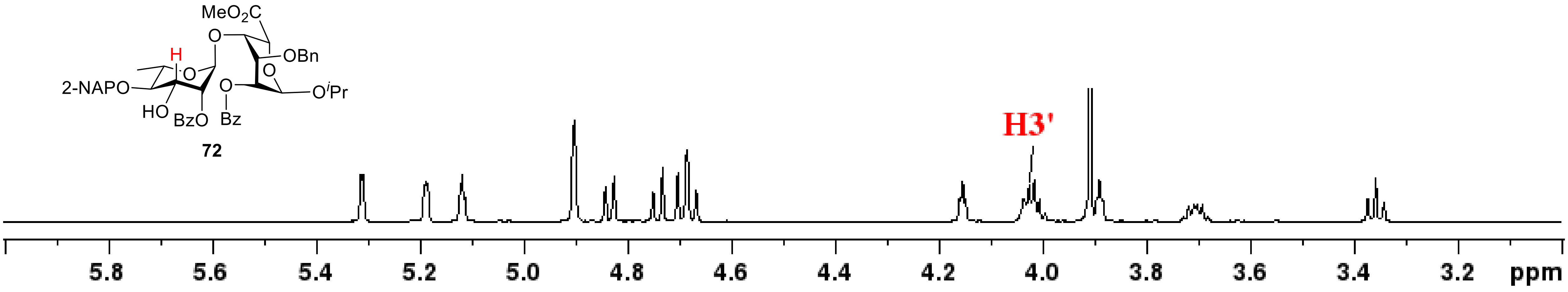
MeO2C
OBn
O
H
O
2-NAPO
O
OiPr
HO
BzO
Bz
72
H3'
5.8
5.6
5.4
5.2
5.0
4.8
4.6
4.4
4.2
4.0
3.8
3.6
3.4
3.2
ppm

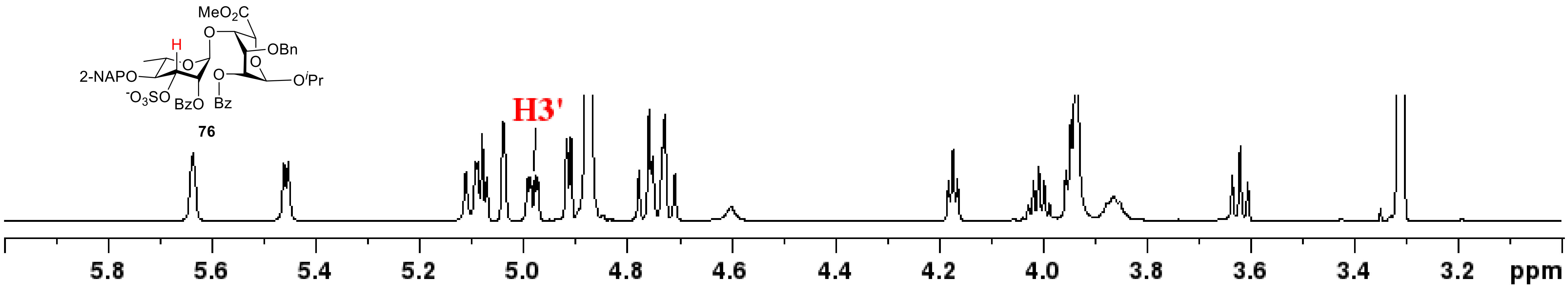
MeO2C
OBn
H
2-NAPO
OiPr
-O3SO
BzO
Bz
76
H3'
5.8
5.6
5.4
5.2
5.0
4.8
4.6
4.4
4.2
4.0
3.8
3.6
3.4
3.2
ppm

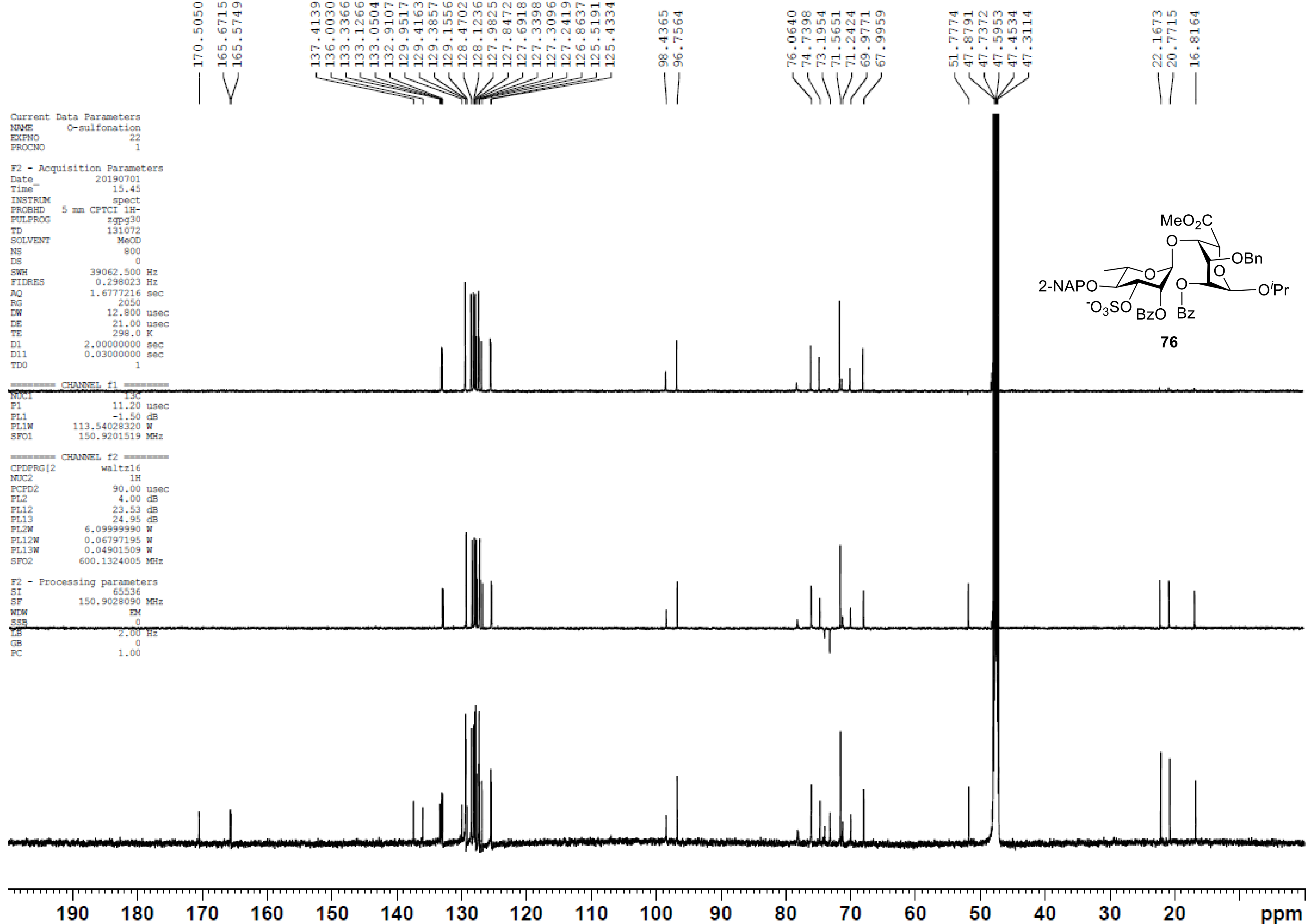

170.5050
165.6715
165.5749
137.4139
136.0030
133.3366
133.1266
133.0504
132.9107
129.9517
129.4163
129.3857
129.1556
128.4702
128.1236
127.9825
127.8472
127.6918
127.3398
127.3096
127.2419
126.8637
125.5191
125.4334
98.4365
96.7564
76.0640
74.7398
73.1954
71.5651
71.2424
69.9771
67.9959
51.7774
47.8791
47.7372
47.5953
47.4534
47.3114
22.1673
20.7715
16.8164
Current Data Parameters
NAME O-sulfonation
EXPNO 22
PROCNO 1
F2 - Acquisition Parameters
Date_ 20190701
Time 15.45
INSTRUM spect
PROBHD 5 mm CPTCI 1H-
PULPROG zgpg30
TD 131072
SOLVENT MeOD
NS 800
DS 0
SWH 39062.500 Hz
FIDRES 0.298023 Hz
AQ 1.6777216 sec
RG 2050
DW 12.800 usec
DE 21.00 usec
TE 298.0 K
D1 2.00000000 sec
D11 0.03000000 sec
TD0 1
======== CHANNEL f1 ========
NUC1 13C
P1 11.20 usec
PL1 -1.50 dB
PL1W 113.54028320 W
SFO1 150.9201519 MHz
======== CHANNEL f2 ========
CPDPRG[2 waltz16
NUC2 1H
PCPD2 90.00 usec
PL2 4.00 dB
PL12 23.53 dB
PL13 24.95 dB
PL2W 6.09999990 W
PL12W 0.06797195 W
PL13W 0.04901509 W
SFO2 600.1324005 MHz
F2 - Processing parameters
SI 65536
SF 150.9028090 MHz
WDW EM
SSB 0
LB 2.00 Hz
GB 0
PC 1.00
MeO2C
OBn
2-NAPO
O
-O3SO
BzO
Bz
O^{i}Pr
76
190 180 170 160 150 140 130 120 110 100 90 80 70 60 50 40 30 20 ppm

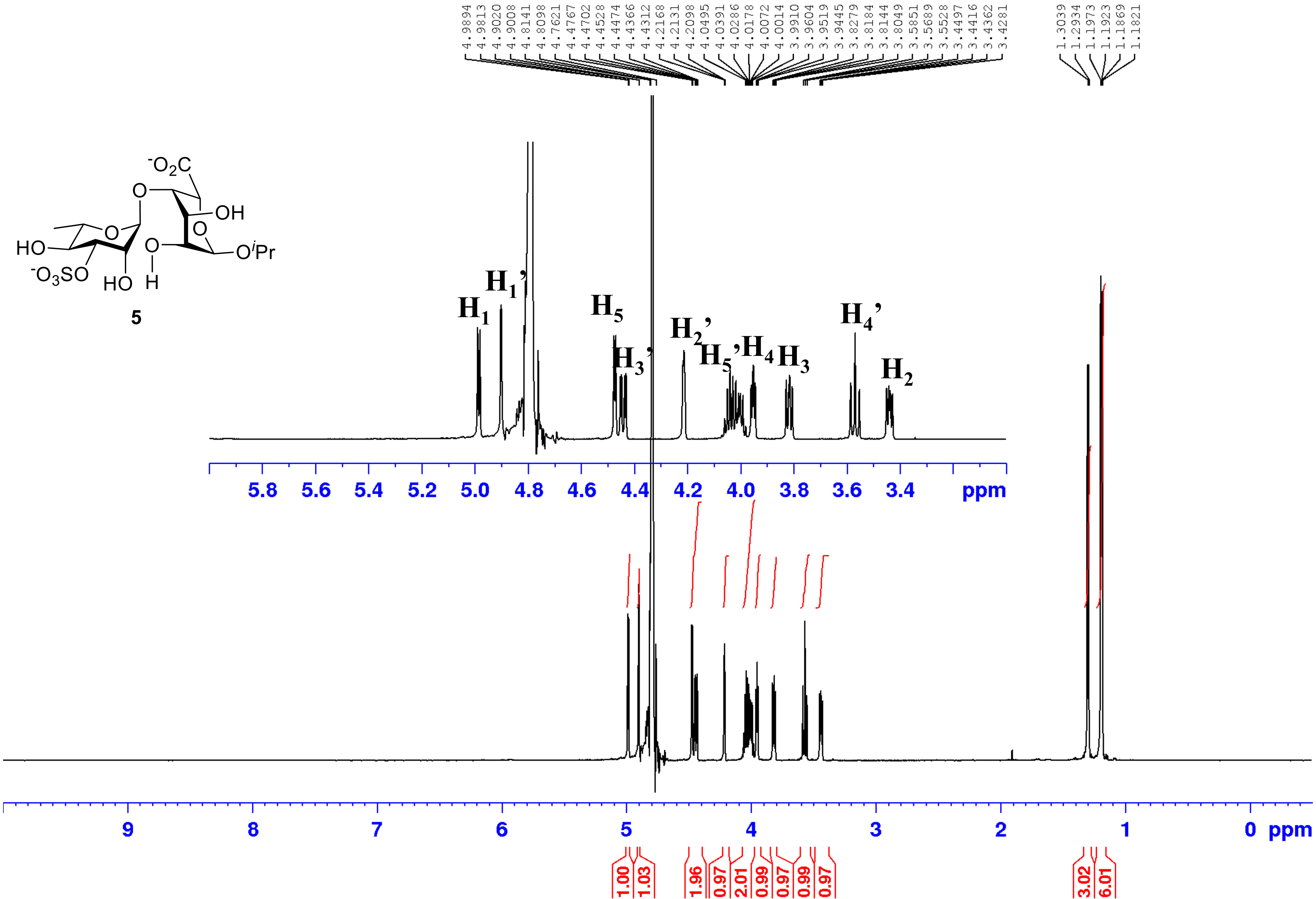

4.9894 4.9813 4.9020 4.9008 4.8141 4.8098 4.7621 4.4767 4.4702 4.4528 4.4474 4.4366 4.4312 4.2168 4.2131 4.2098 4.0495 4.0391 4.0286 4.0178 4.0072 4.0014 3.9910 3.9604 3.9519 3.9445 3.8279 3.8184 3.8144 3.8049 3.5851 3.5689 3.5528 3.4497 3.4416 3.4362 3.4281
1.3039 1.2934 1.1973 1.1923 1.1869 1.1821
-O2C
OH
O
HO
-O3SO
HO
H
OiPr
5
H1
H1'
H5
H3'
H2'
H5'
H4
H3
H4'
H2
5.8 5.6 5.4 5.2 5.0 4.8 4.6 4.4 4.2 4.0 3.8 3.6 3.4 ppm
9 8 7 6 5 4 3 2 1 0 ppm
1.00 1.03 1.96 0.97 2.01 0.99 0.97 0.99 0.97 3.02 6.01

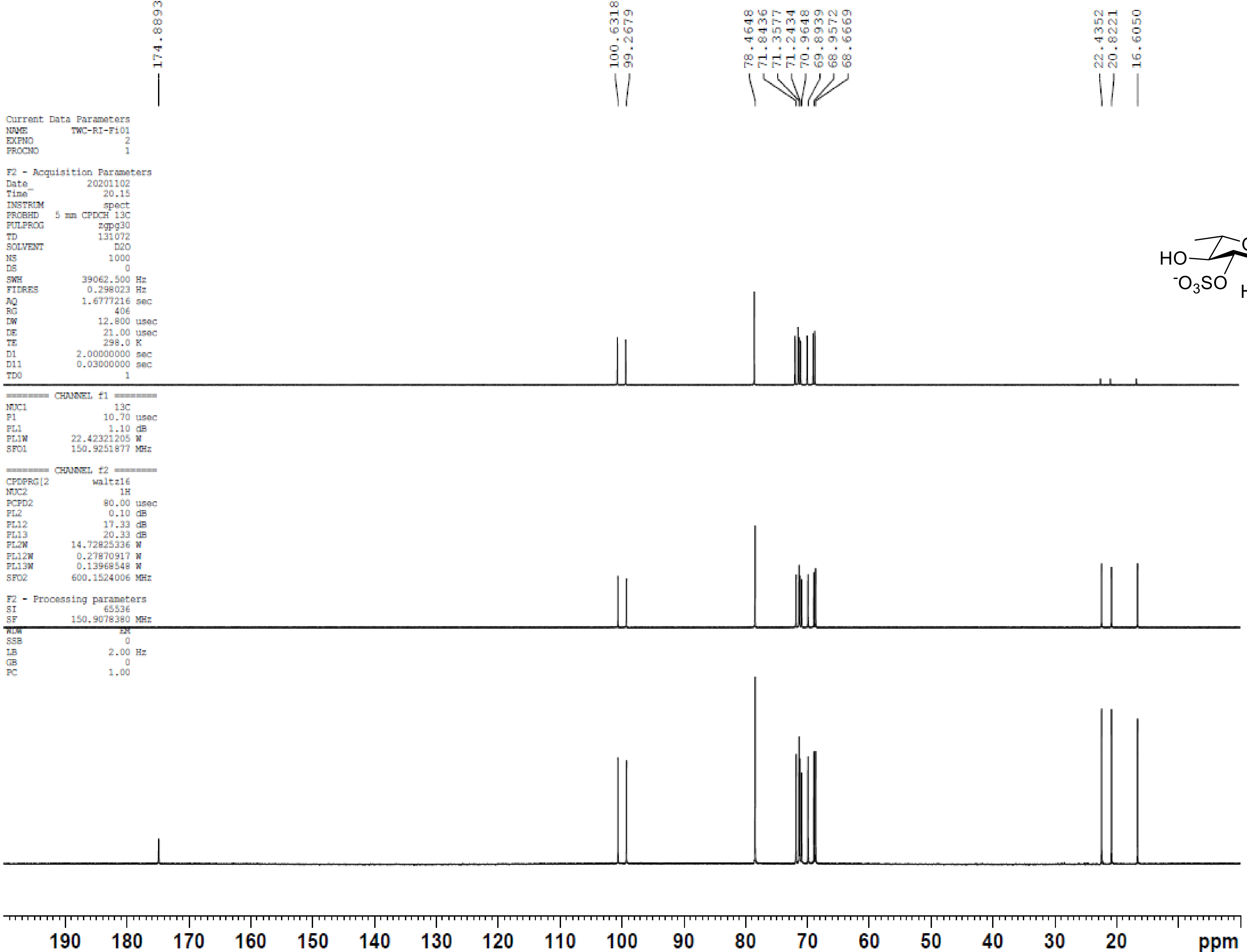
174.8893
100.6318
99.2679
78.4648
71.8436
71.3577
71.2434
70.9648
69.8939
68.9572
68.6669
22.4352
20.8221
16.6050
Current Data Parameters
NAME TWC-RI-F101
EXPNO 2
PROCNO 1
F2 - Acquisition Parameters
Date_ 20201102
Time 20.15
INSTRUM spect
PROBHD 5 mm CPDCH 13C
PULPROG zgpg30
TD 131072
SOLVENT D2O
NS 1000
DS 0
SWH 39062.500 Hz
FIDRES 0.298023 Hz
AQ 1.6777216 sec
RG 406
DW 12.800 usec
DE 21.00 usec
TE 298.0 K
D1 2.00000000 sec
D11 0.03000000 sec
TD0 1
======== CHANNEL f1 ========
NUC1 13C
P1 10.70 usec
PL1 1.10 dB
PL1W 22.42321205 W
SFO1 150.9251877 MHz
======== CHANNEL f2 ========
CPDPRG[2 waltz16
NUC2 1H
PCPD2 80.00 usec
PL2 0.10 dB
PL12 17.33 dB
PL13 20.33 dB
PL2W 14.72825336 W
PL12W 0.27870917 W
PL13W 0.13968548 W
SFO2 600.1524006 MHz
F2 - Processing parameters
SI 65536
SF 150.9078380 MHz
WDW EM
SSB 0
LB 2.00 Hz
GB 0
PC 1.00
190 180 170 160 150 140 130 120 110 100 90 80 70 60 50 40 30 20 ppm

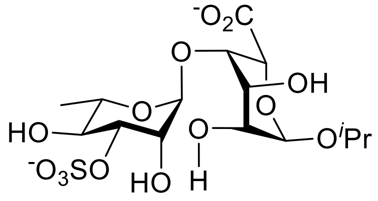
-O2C
O
OH
O
HO
O
O
OiPr
-O3SO
HO
H

5

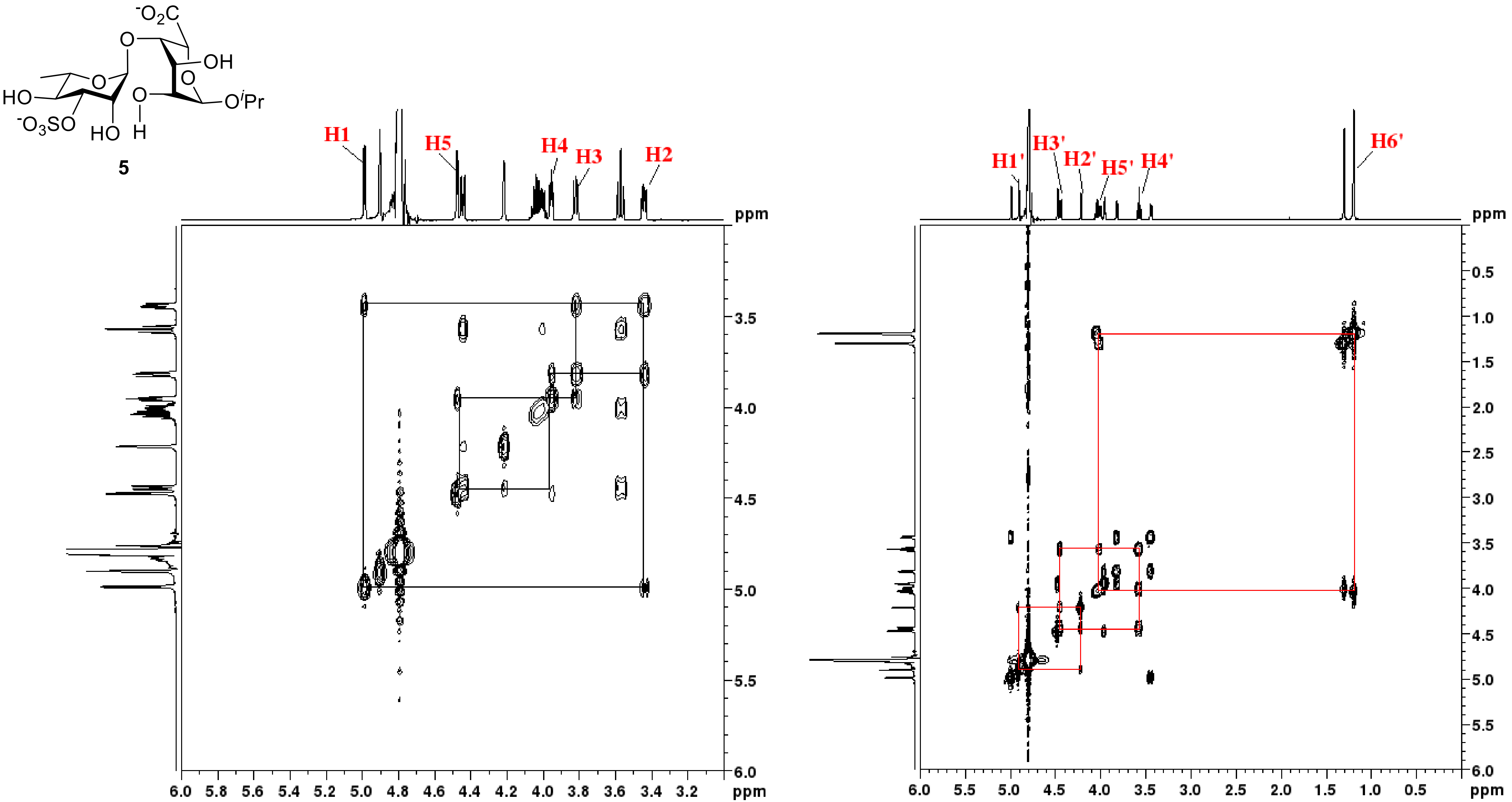

5

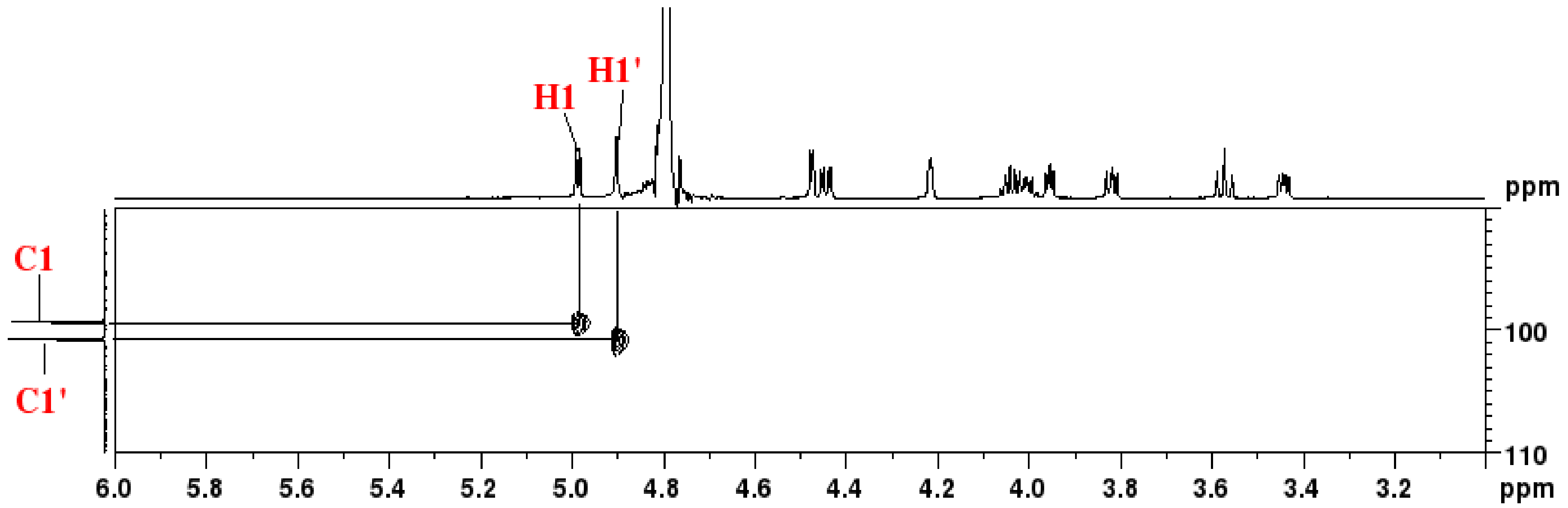
H1
H1'
C1
C1'
ppm
100
110
6.0
5.8
5.6
5.4
5.2
5.0
4.8
4.6
4.4
4.2
4.0
3.8
3.6
3.4
3.2
ppm

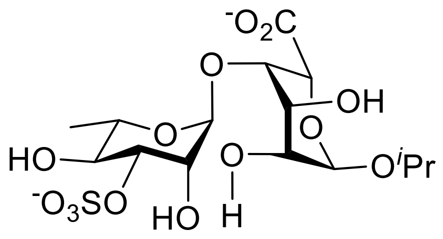
-O2C
O
OH
O
O
HO
O
OiPr
-O3SO
HO
H

5

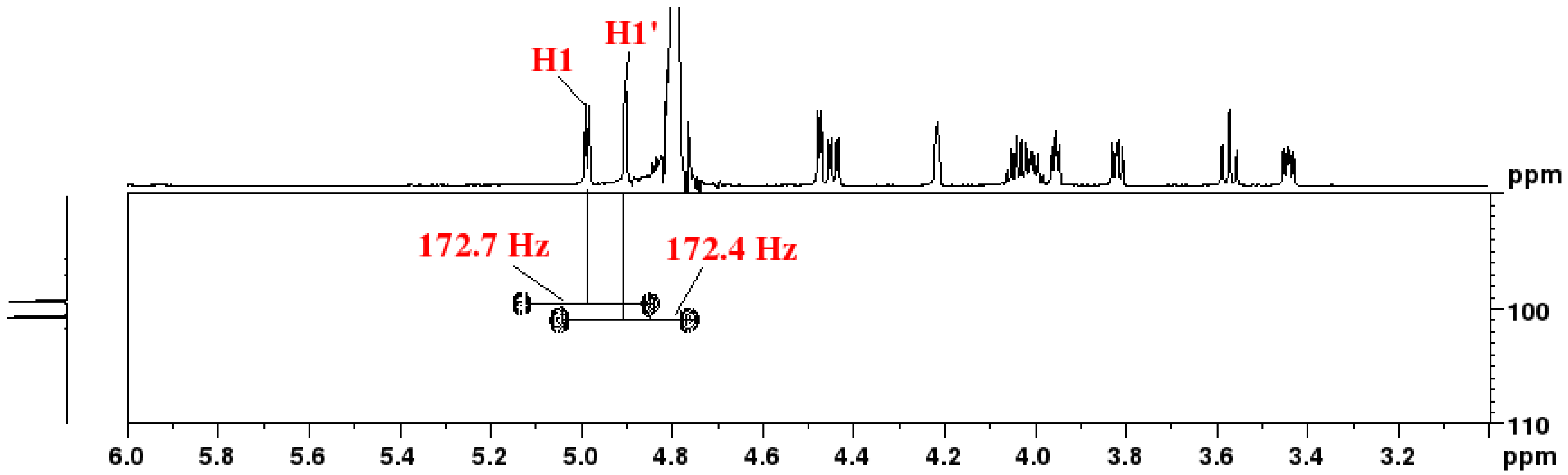
H1
H1'
ppm
172.7 Hz
172.4 Hz
100
110
6.0
5.8
5.6
5.4
5.2
5.0
4.8
4.6
4.4
4.2
4.0
3.8
3.6
3.4
3.2
ppm

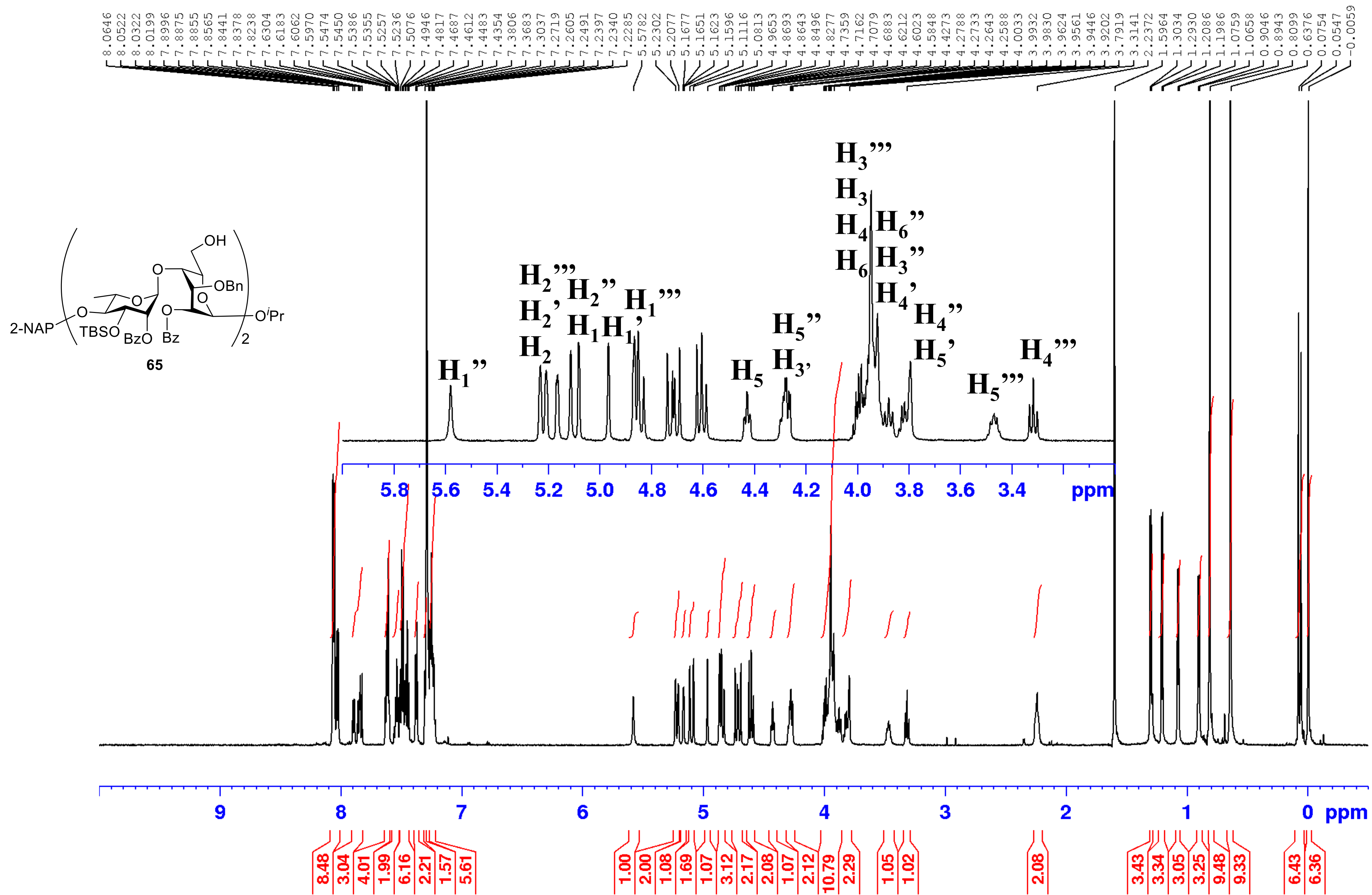

2-NAP
TBSO
BzO
Bz
OBn
OH
O^{i}Pr
65
H$_1$''
H$_2$'''
H$_2$'
H$_2$
H$_2$''
H$_1$
H$_1$'
H$_1$'''
H$_5$
H$_5$''
H$_3$'
H$_3$'''
H$_3$
H$_4$
H$_6$
H$_6$''
H$_3$''
H$_4$'
H$_4$''
H$_5$'
H$_5$'''
H$_4$'''
ppm

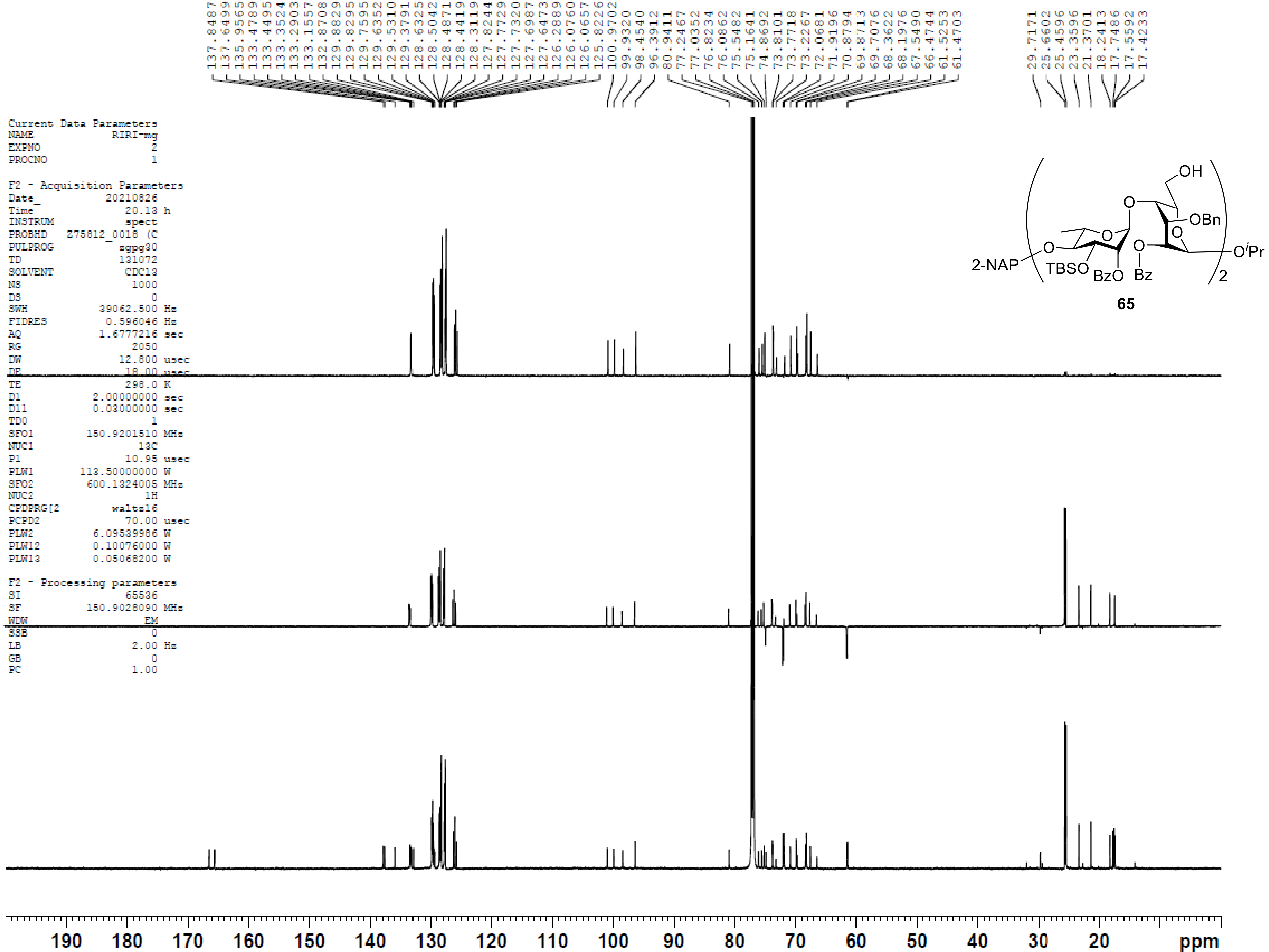
137.8487
137.6499
135.9565
133.4789
133.4495
133.3524
133.2903
133.1557
132.8708
129.8829
129.8295
129.7595
129.6352
129.5310
129.3791
128.6325
128.5042
128.4871
128.4419
128.3119
127.8244
127.7729
127.7320
127.6987
127.6473
126.2889
126.0760
126.0657
125.8226
100.9702
99.9320
98.4540
96.3912
80.9411
77.2467
77.0352
76.8234
76.0862
75.5482
75.1641
74.8692
73.8101
73.7718
73.2267
72.0681
71.9196
70.8794
69.8713
69.7076
68.3622
68.1976
67.5490
66.4744
61.5253
61.4703
29.7171
25.6602
25.4596
23.3596
21.3701
18.2413
17.7486
17.5592
17.4233
Current Data Parameters
NAME RIRI-mg
EXPNO 2
PROCNO 1
F2 - Acquisition Parameters
Date_ 20210826
Time 20.13 h
INSTRUM spect
PROBHD Z75812_0018 (C
PULPROG zgpg30
TD 131072
SOLVENT CDCl3
NS 1000
DS 0
SWH 39062.500 Hz
FIDRES 0.596046 Hz
AQ 1.6777216 sec
RG 2050
DW 12.800 usec
DE 18.00 usec
TE 298.0 K
D1 2.00000000 sec
D11 0.03000000 sec
TD0 1
SFO1 150.9201510 MHz
NUC1 13C
P1 10.95 usec
PLW1 113.50000000 W
SFO2 600.1324005 MHz
NUC2 1H
CPDPRG[2 waltz16
PCPD2 70.00 usec
PLW2 6.09539986 W
PLW12 0.10076000 W
PLW13 0.05068200 W
F2 - Processing parameters
SI 65536
SF 150.9028090 MHz
WDW EM
SSB 0
LB 2.00 Hz
GB 0
PC 1.00
OH
O
OBn
O
O
O^iPr
O
2-NAP
TBSO
BzO
Bz
2
65
190
180
170
160
150
140
130
120
110
100
90
80
70
60
50
40
30
20
ppm

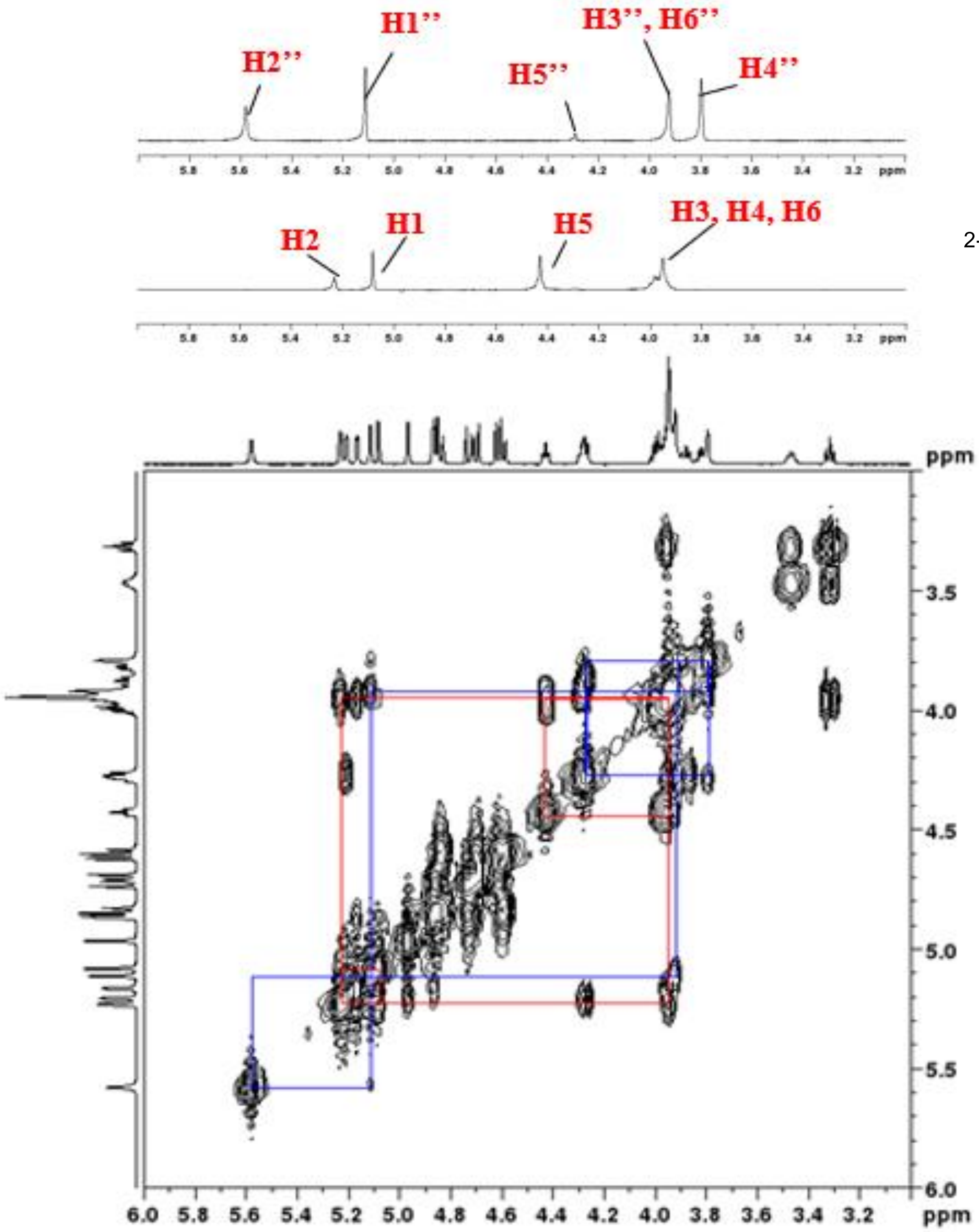
H2''
H1''
H5''
H3'', H6''
H4''
H2
H1
H5
H3, H4, H6
ppm

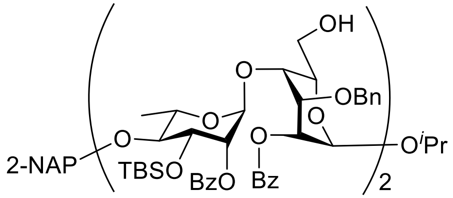
OH
OBn
2-NAP
TBSO
BzO
Bz
O^{i}Pr
2

65

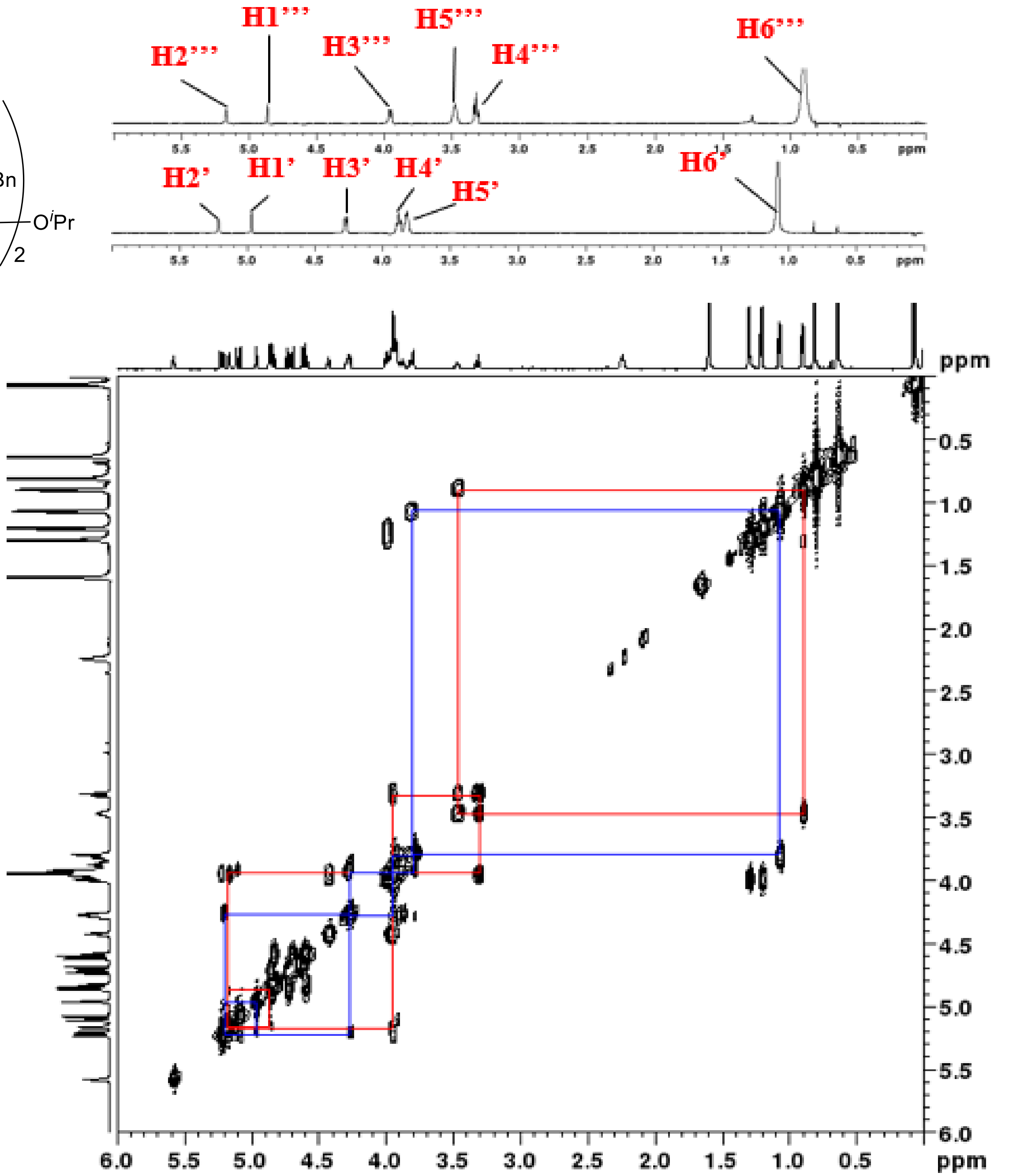
H1'''
H5'''
H6'''
H2'''
H3'''
H4'''
H2'
H1'
H3'
H4'
H6'
H5'
ppm

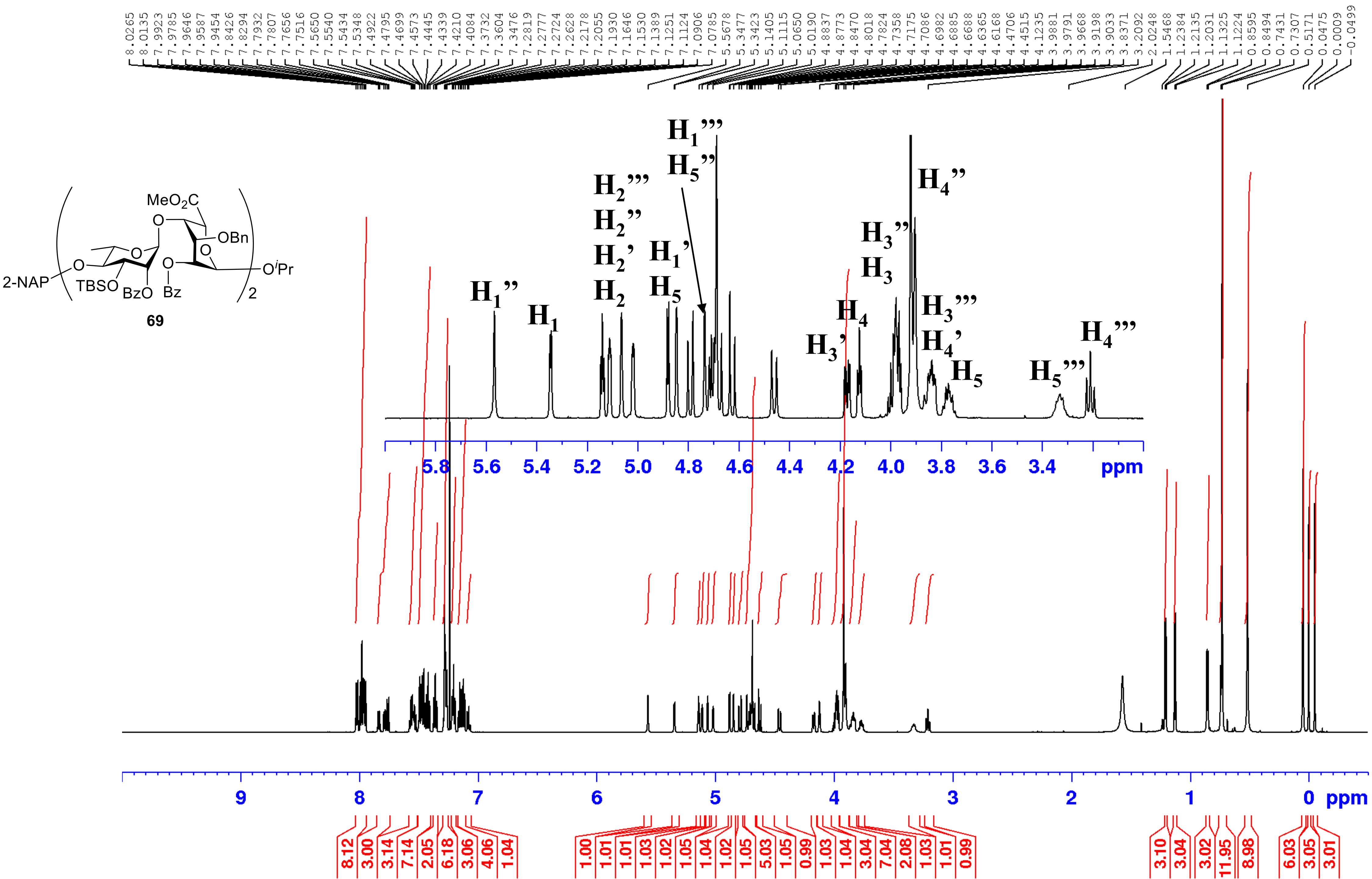

MeO2C
OBn
O
O
O
O
O
2-NAP
TBSO
BzO
Bz
O^{i}Pr
2

69

8.0265 8.0135 7.9923 7.9785 7.9646 7.9587 7.9454 7.8426 7.8294 7.7932 7.7807 7.7656 7.7516 7.5650 7.5540 7.5434 7.5348 7.4922 7.4795 7.4699 7.4573 7.4445 7.4339 7.4210 7.4084 7.3732 7.3604 7.3476 7.2819 7.2777 7.2724 7.2628 7.2178 7.2055 7.1930 7.1646 7.1530 7.1389 7.1251 7.1124 7.0906 7.0785 5.5678 5.3477 5.3423 5.1405 5.1115 5.0650 5.0190 4.8837 4.8773 4.8470 4.8018 4.7824 4.7358 4.7175 4.7086 4.6982 4.6885 4.6688 4.6365 4.6168 4.4706 4.4515 4.1235 3.9881 3.9791 3.9668 3.9198 3.9033 3.8371 3.2092 2.0248 1.5468 1.2384 1.2135 1.2031 1.1325 1.1224 0.8595 0.8494 0.7431 0.7307 0.5171 0.0475 0.0009 -0.0499
H1''
H1
H2''' H2'' H2' H2
H1' H5
H1''' H5''
H3'
H4
H3'' H3
H4''
H3''' H4'
H5
H5'''
H4'''
5.8 5.6 5.4 5.2 5.0 4.8 4.6 4.4 4.2 4.0 3.8 3.6 3.4 ppm
9 8 7 6 5 4 3 2 1 0 ppm
8.12 3.00 3.14 7.14 2.05 6.18 3.06 4.06 1.04 1.00 1.01 1.01 1.03 1.02 1.05 1.04 1.02 1.05 5.03 1.05 0.99 1.03 1.04 3.04 7.04 2.08 1.03 1.01 0.99 3.10 3.04 3.02 11.95 8.98 6.03 3.05 3.01

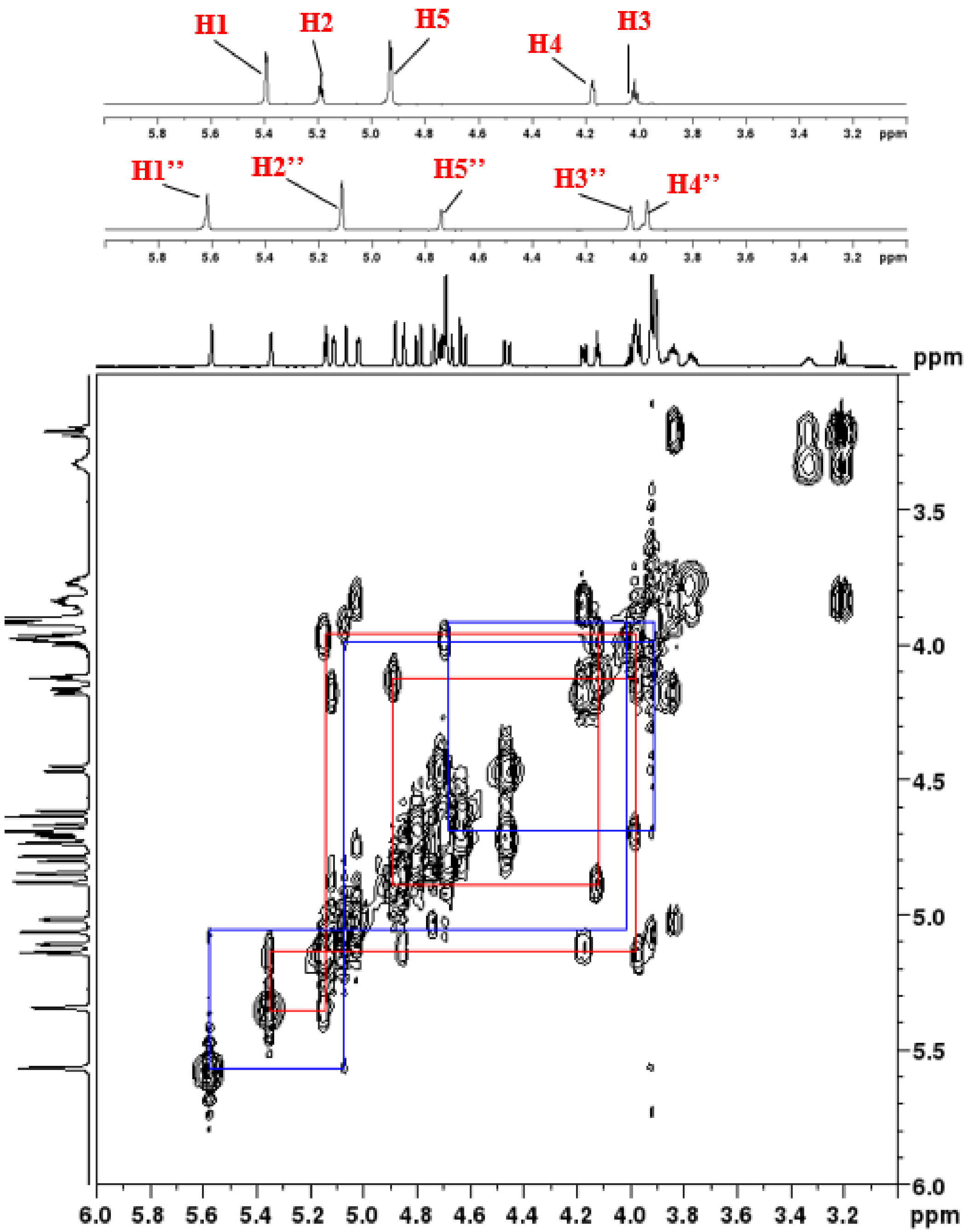
H1
H2
H5
H4
H3
H1''
H2''
H5''
H3''
H4''
ppm

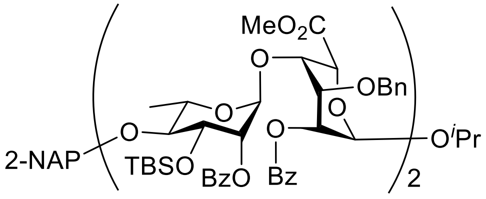
MeO2C
O
OBn
2-NAP
TBSO
BzO
Bz
O^{i}Pr
2

69

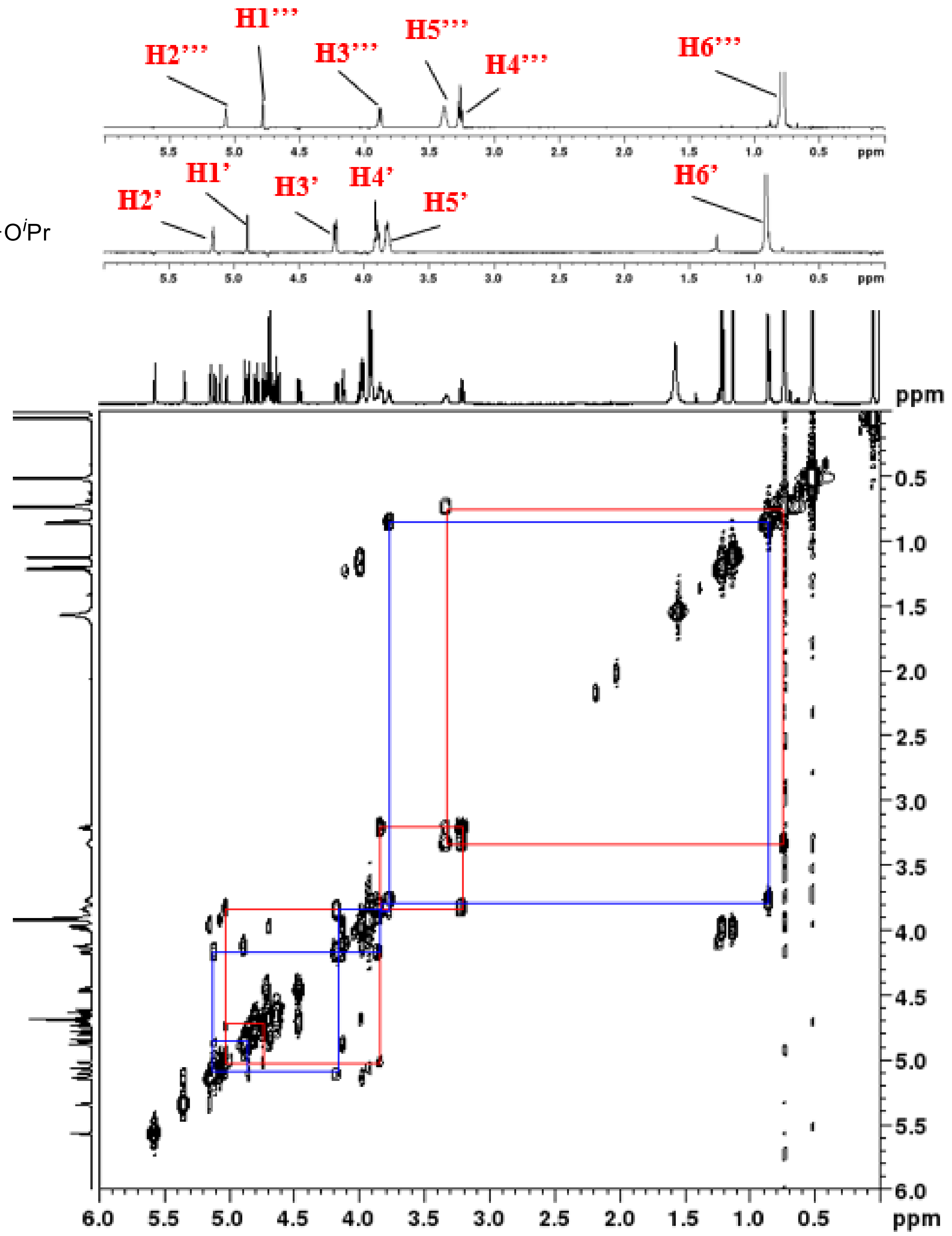
H1'''
H2'''
H3'''
H5'''
H4'''
H6'''
H1'
H2'
H3'
H4'
H5'
H6'
ppm

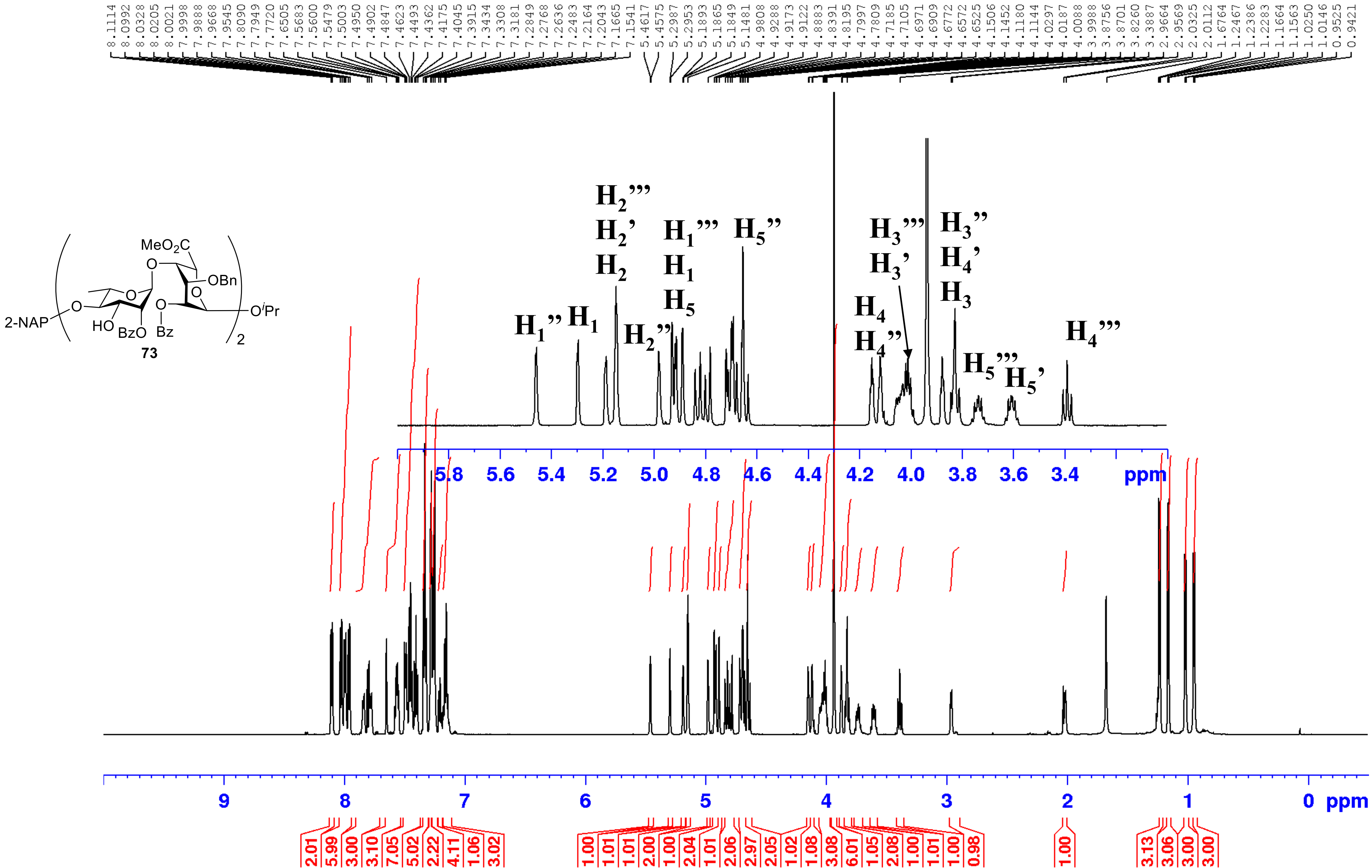

MeO2C
OBn
O
O
O
2-NAP
HO
BzO
Bz
O^iPr
2
73
H1''
H1
H2'''
H2'
H2
H2''
H1'''
H1
H5
H5''
H3'''
H3'
H4
H4''
H3''
H4'
H3
H5'''
H5'
H4'''
8.1114 8.0992 8.0328 8.0205 8.0021 7.9998 7.9888 7.9668 7.9545 7.8090 7.7949 7.7720 7.6505 7.5683 7.5600 7.5479 7.5003 7.4950 7.4902 7.4847 7.4623 7.4493 7.4362 7.4175 7.4045 7.3915 7.3434 7.3308 7.3181 7.2849 7.2768 7.2636 7.2483 7.2164 7.2043 7.1665 7.1541 5.4617 5.4575 5.2987 5.2953 5.1893 5.1865 5.1849 5.1481 4.9808 4.9288 4.9173 4.9122 4.8883 4.8391 4.8195 4.7997 4.7809 4.7185 4.7105 4.6971 4.6909 4.6772 4.6572 4.6525 4.1506 4.1452 4.1180 4.1144 4.0297 4.0187 4.0088 3.9988 3.8756 3.8701 3.8260 3.3887 2.9664 2.9569 2.0325 2.0112 1.6764 1.2467 1.2386 1.2283 1.1664 1.1563 1.0250 1.0146 0.9525 0.9421
5.8 5.6 5.4 5.2 5.0 4.8 4.6 4.4 4.2 4.0 3.8 3.6 3.4 ppm
9 8 7 6 5 4 3 2 1 0 ppm
2.01 5.99 3.00 3.10 7.05 5.02 2.22 4.11 1.06 3.02 1.00 1.01 1.01 2.00 1.00 2.04 1.01 2.06 2.97 2.05 1.02 1.08 3.08 6.01 1.05 2.08 1.00 1.01 1.00 0.98 1.00 3.13 3.06 3.00 3.00

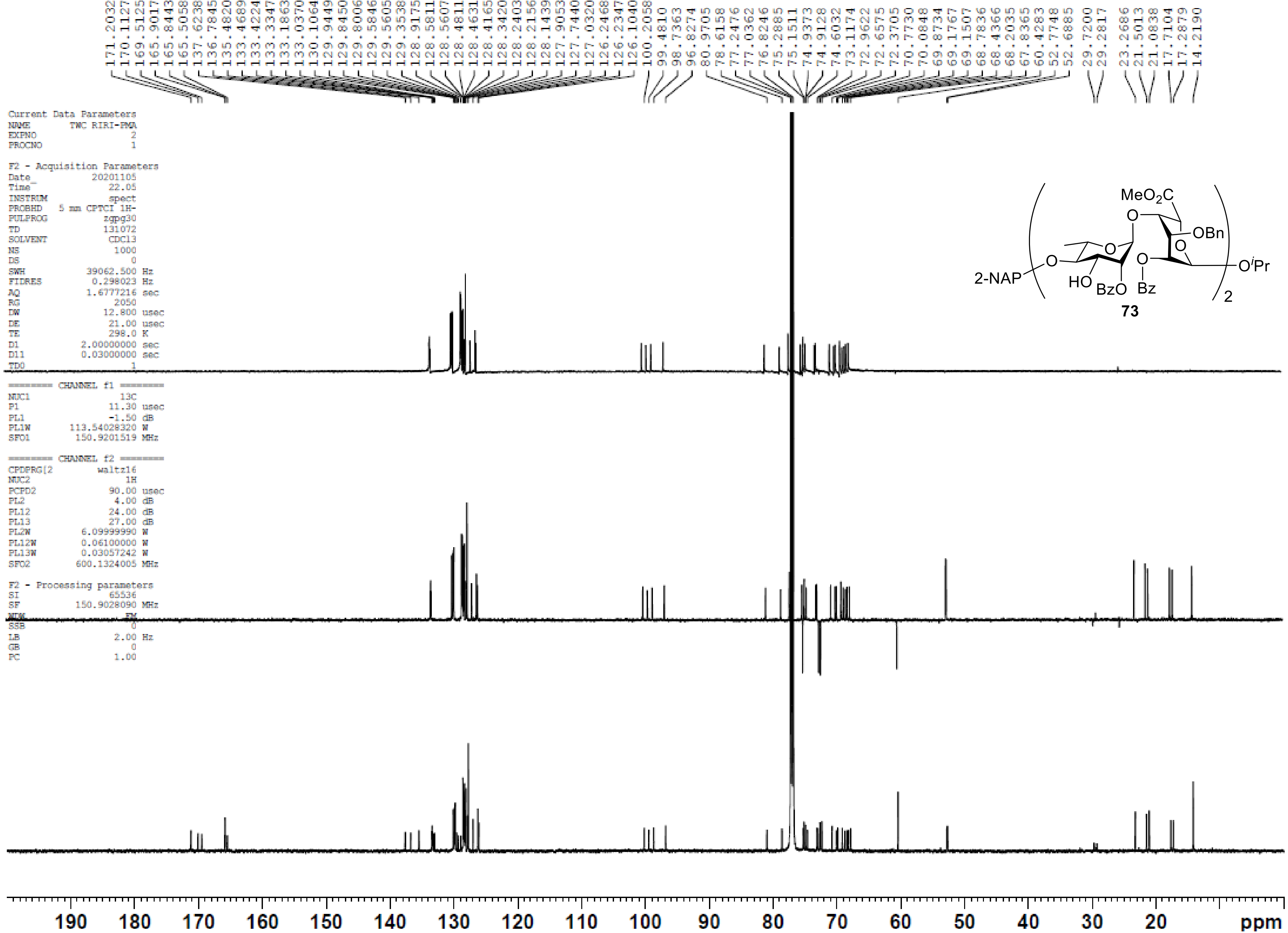
171.2032
170.1127
169.5125
165.9017
165.8443
165.5058
137.6238
136.7845
135.4820
133.4689
133.4224
133.3347
133.1863
133.0370
130.1064
129.9449
129.8450
129.8006
129.5846
129.5605
129.3538
128.9175
128.5811
128.5607
128.4811
128.4631
128.4165
128.3420
128.2403
128.2156
128.1439
127.9053
127.7440
127.0320
126.2468
126.2347
126.1040
100.2058
99.4810
98.7363
96.8274
80.9705
78.6158
77.2476
77.0362
76.8246
75.2885
75.1511
74.9373
74.9128
74.6032
73.1174
72.9622
72.6575
72.3705
70.7730
70.0848
69.8734
69.1767
69.1507
68.7836
68.4366
68.2035
67.8365
60.4283
52.7748
52.6885
29.7200
29.2817
23.2686
21.5013
21.0838
17.7104
17.2879
14.2190
Current Data Parameters
NAME TWC RIRI-PMA
EXPNO 2
PROCNO 1
F2 - Acquisition Parameters
Date_ 20201105
Time 22.05
INSTRUM spect
PROBHD 5 mm CPTCI 1H-
PULPROG zgpg30
TD 131072
SOLVENT CDCl3
NS 1000
DS 0
SWH 39062.500 Hz
FIDRES 0.298023 Hz
AQ 1.6777216 sec
RG 2050
DW 12.800 usec
DE 21.00 usec
TE 298.0 K
D1 2.00000000 sec
D11 0.03000000 sec
TD0 1
======== CHANNEL f1 ========
NUC1 13C
P1 11.30 usec
PL1 -1.50 dB
PL1W 113.54028320 W
SFO1 150.9201519 MHz
======== CHANNEL f2 ========
CPDPRG[2 waltz16
NUC2 1H
PCPD2 90.00 usec
PL2 4.00 dB
PL12 24.00 dB
PL13 27.00 dB
PL2W 6.09999990 W
PL12W 0.06100000 W
PL13W 0.03057242 W
SFO2 600.1324005 MHz
F2 - Processing parameters
SI 65536
SF 150.9028090 MHz
WDW EM
SSB 0
LB 2.00 Hz
GB 0
PC 1.00
MeO2C
OBn
O
2-NAP
HO
BzO
Bz
OiPr
2
73
190 180 170 160 150 140 130 120 110 100 90 80 70 60 50 40 30 20 ppm

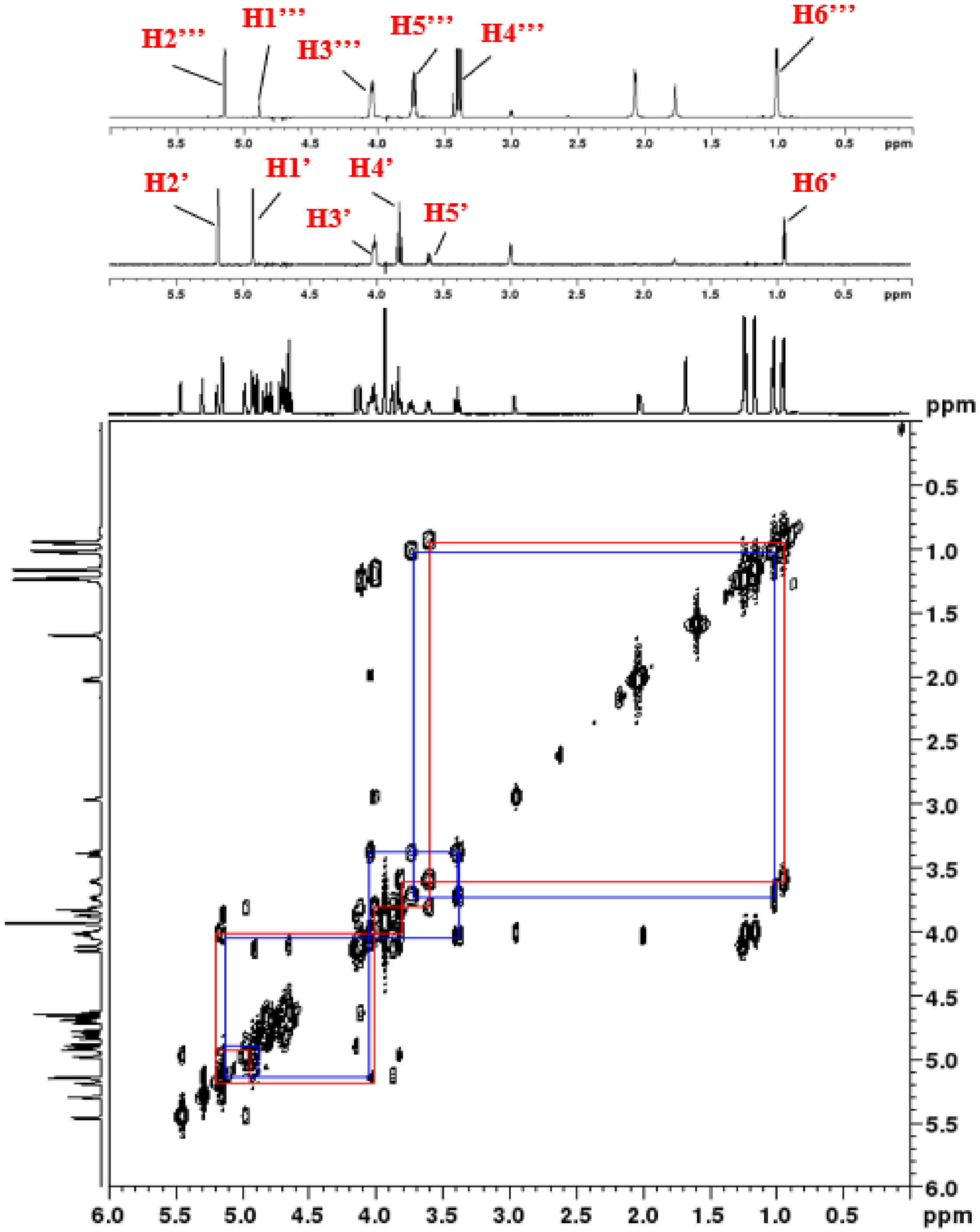
H2'''
H1'''
H3'''
H5'''
H4'''
H6'''
H2'
H1'
H4'
H3'
H5'
H6'
ppm

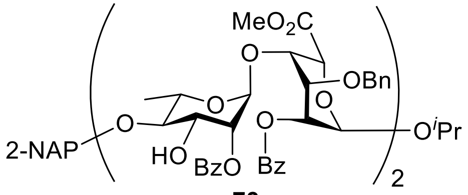
MeO2C
OBn
2-NAP
HO
BzO
Bz
O^{i}Pr
2

73

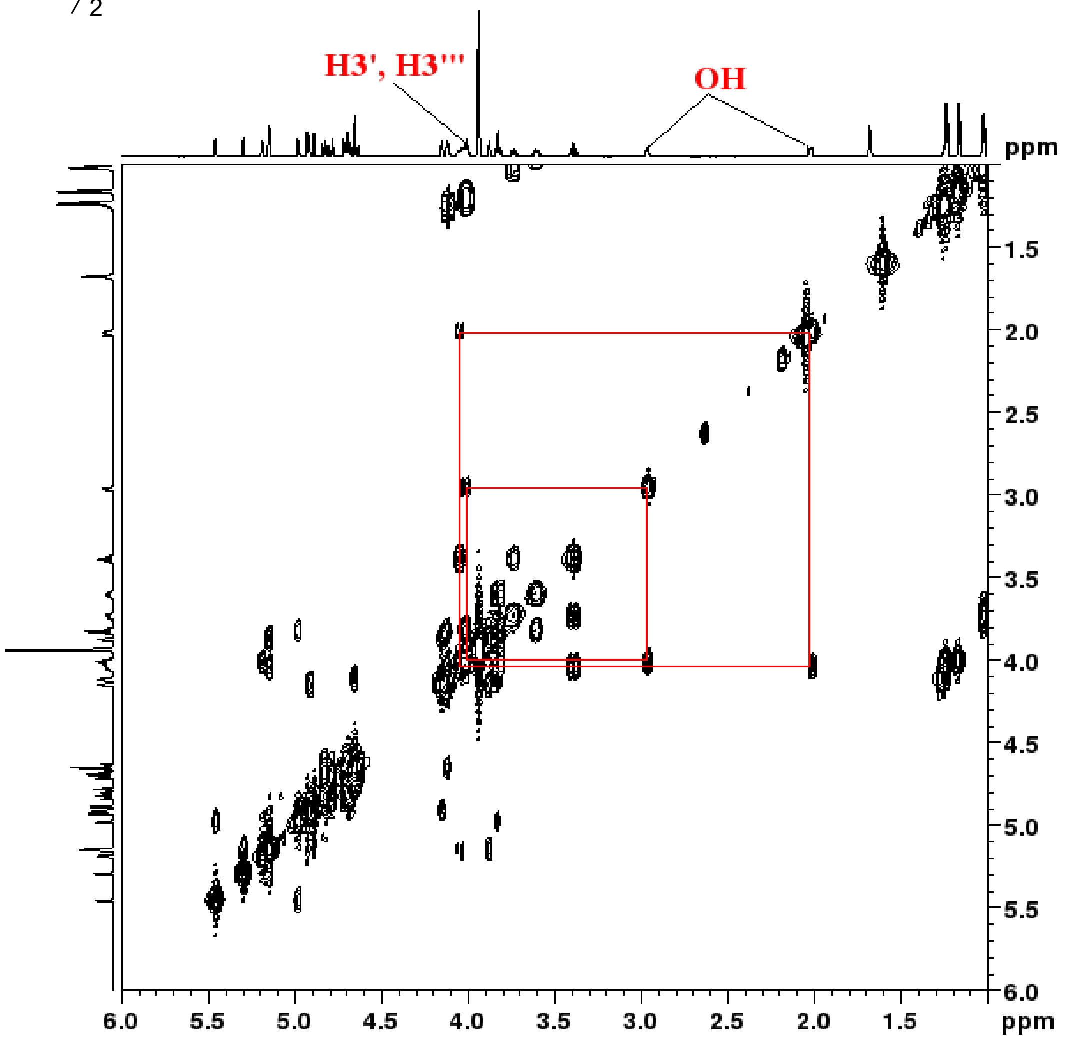
H3', H3'''
OH
ppm

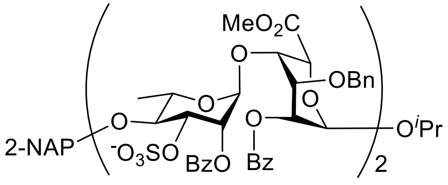

77

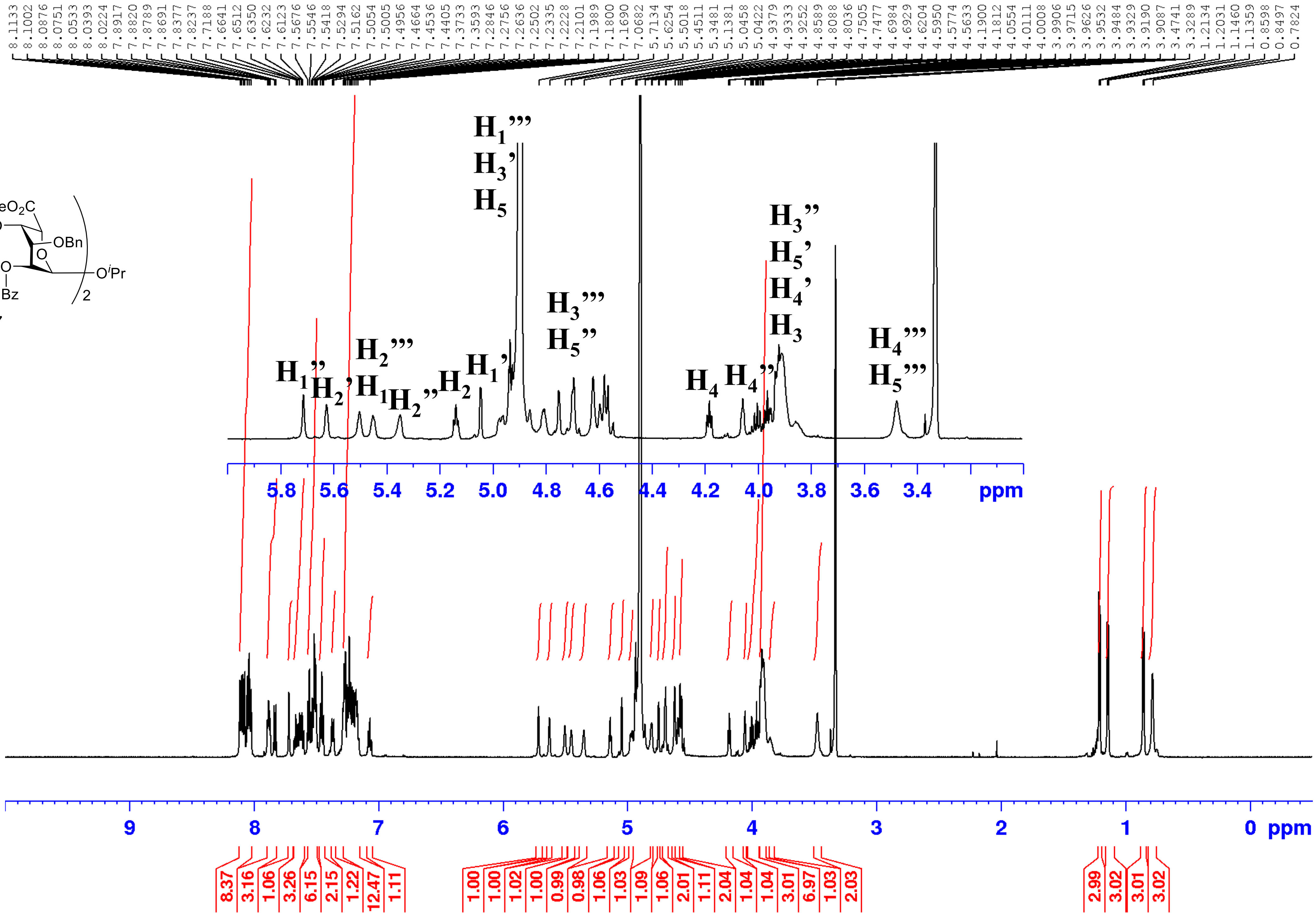

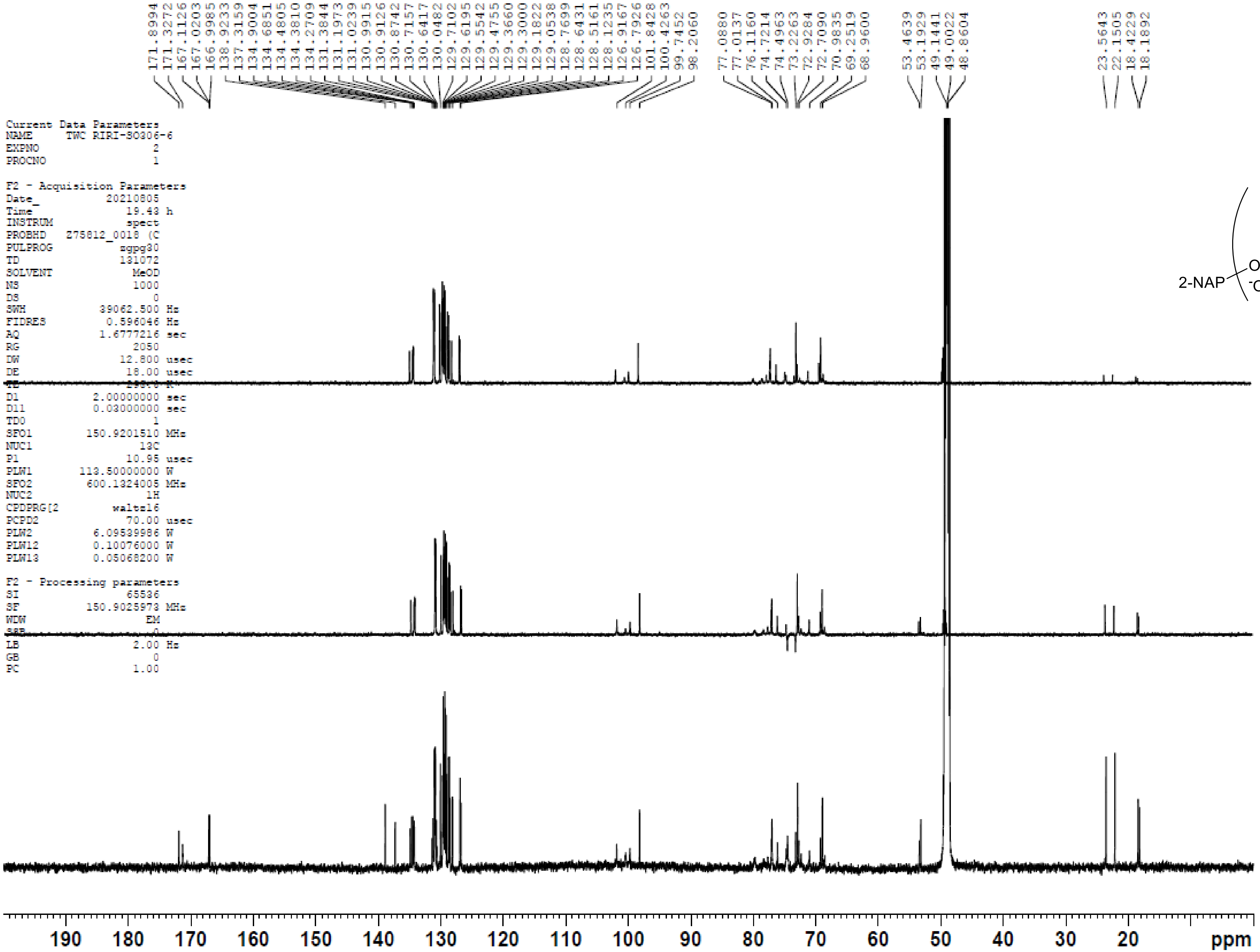
171.8994
171.3272
167.1126
167.0203
166.9985
138.9233
137.3159
134.9004
134.6851
134.4805
134.3810
134.2709
131.3844
131.1973
131.0239
130.9915
130.9126
130.8742
130.7157
130.6417
130.0482
129.7102
129.6195
129.5542
129.4755
129.3660
129.3000
129.1822
129.0538
128.7699
128.6431
128.5161
128.1235
126.9167
126.7926
101.8428
100.4263
99.7452
98.2060
77.0880
77.0137
76.1160
74.7214
74.4963
73.2263
72.9284
72.7090
70.9835
69.2519
68.9600
53.4639
53.1929
49.1441
49.0022
48.8604
23.5643
22.1505
18.4229
18.1892
Current Data Parameters
NAME TWC RIRI-SO306-6
EXPNO 2
PROCNO 1
F2 - Acquisition Parameters
Date_ 20210805
Time 19.43 h
INSTRUM spect
PROBHD Z75812_0018 (C
PULPROG zgpg30
TD 131072
SOLVENT MeOD
NS 1000
DS 0
SWH 39062.500 Hz
FIDRES 0.596046 Hz
AQ 1.6777216 sec
RG 2050
DW 12.800 usec
DE 18.00 usec
D1 2.00000000 sec
D11 0.03000000 sec
TD0 1
SFO1 150.9201510 MHz
NUC1 13C
P1 10.95 usec
PLW1 113.50000000 W
SFO2 600.1324005 MHz
NUC2 1H
CPDPRG[2 waltz16
PCPD2 70.00 usec
PLW2 6.09539986 W
PLW12 0.10076000 W
PLW13 0.05068200 W
F2 - Processing parameters
SI 65536
SF 150.9025973 MHz
WDW EM
LB 2.00 Hz
GB 0
PC 1.00
190 180 170 160 150 140 130 120 110 100 90 80 70 60 50 40 30 20 ppm

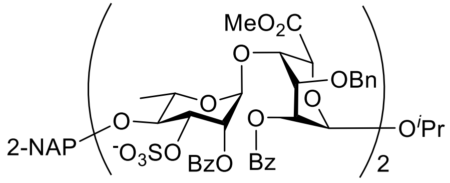
MeO2C
O
OBn
O
O
O-iPr
2-NAP
-O3SO
BzO
O
Bz
2

77

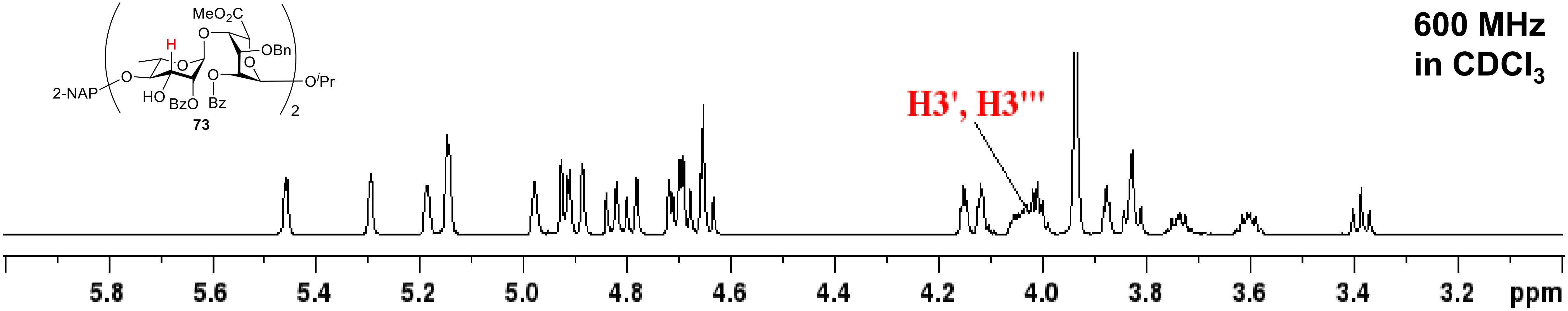
MeO2C
H
OBn
O
O
O
O
O
O
OiPr
2-NAP
HO
BzO
Bz
2
73
600 MHz
in CDCl3
H3', H3'''
5.8
5.6
5.4
5.2
5.0
4.8
4.6
4.4
4.2
4.0
3.8
3.6
3.4
3.2
ppm

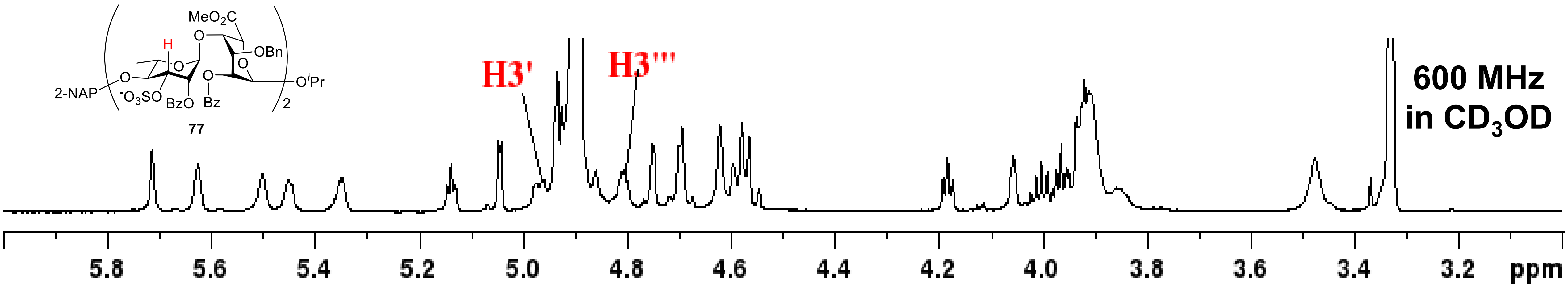
MeO2C
H
OBn
O
O
O
O
O
O
OiPr
2-NAP
-O3SO
BzO
Bz
2
77
H3'
H3'''
600 MHz
in CD3OD
5.8
5.6
5.4
5.2
5.0
4.8
4.6
4.4
4.2
4.0
3.8
3.6
3.4
3.2
ppm

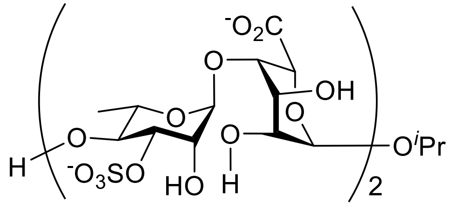

-O2C
OH
O
O
OiPr
H
-O3SO
HO
2

6

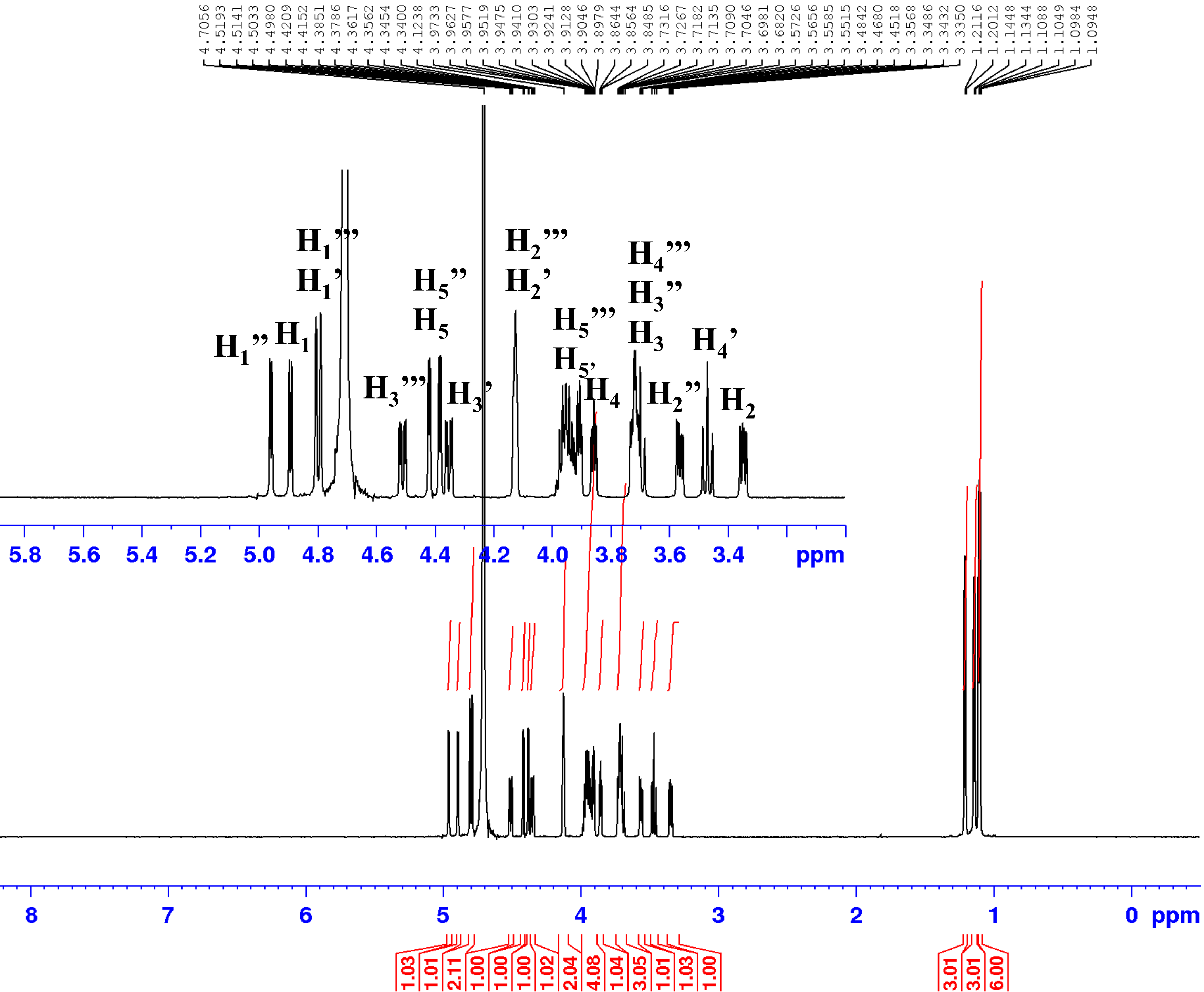

4.7056 4.5193 4.5141 4.5033 4.4980 4.4209 4.4152 4.3851 4.3786 4.3617 4.3562 4.3454 4.3400 4.1238 3.9733 3.9627 3.9577 3.9519 3.9475 3.9410 3.9303 3.9241 3.9128 3.9046 3.8979 3.8644 3.8564 3.8485 3.7316 3.7267 3.7182 3.7135 3.7090 3.7046 3.6981 3.6820 3.5726 3.5656 3.5585 3.5515 3.4842 3.4680 3.4518 3.3568 3.3486 3.3432 3.3350 1.2116 1.2012 1.1448 1.1344 1.1088 1.1049 1.0984 1.0948
H1''
H1
H1''' H1'
H3'''
H5'' H5
H3'
H2''' H2'
H5''' H5'
H4
H4''' H3'' H3
H2''
H4'
H2
5.8 5.6 5.4 5.2 5.0 4.8 4.6 4.4 4.2 4.0 3.8 3.6 3.4 ppm
9 8 7 6 5 4 3 2 1 0 ppm
1.03 1.01 2.11 1.00 1.00 1.00 1.02 2.04 4.08 1.04 3.05 1.01 1.03 1.00 3.01 3.01 6.00

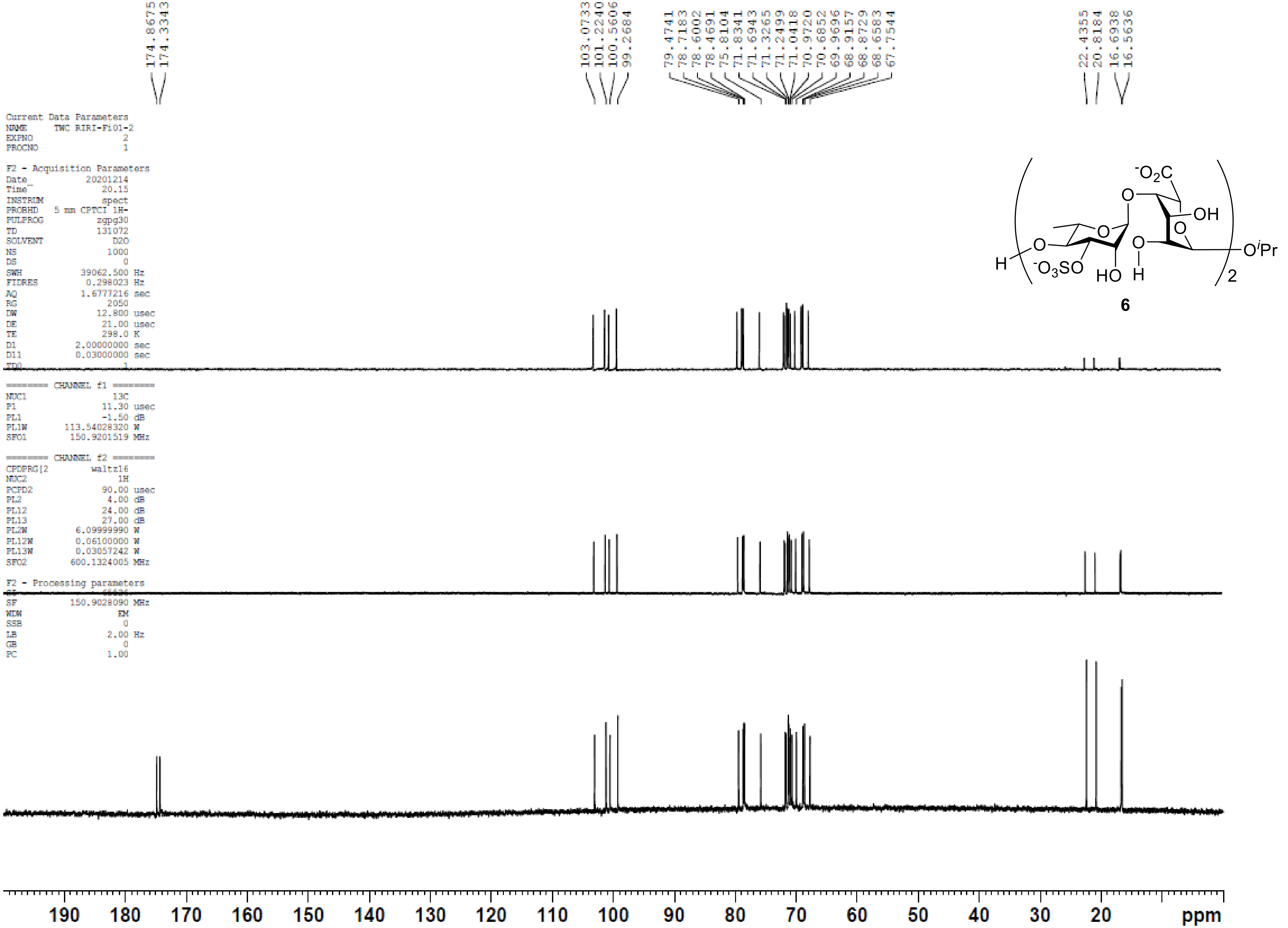
174.8675
174.3343
103.0733
101.2240
100.5606
99.2684
79.4741
78.7183
78.6002
78.4691
75.8104
71.8341
71.6943
71.3265
71.2499
71.0418
70.9720
70.6852
69.9696
68.9157
68.8729
68.6583
67.7544
22.4355
20.8184
16.6938
16.5636
Current Data Parameters
NAME TWC RIRI-F101-2
EXPNO 2
PROCNO 1
F2 - Acquisition Parameters
Date_ 20201214
Time 20.15
INSTRUM spect
PROBHD 5 mm CPTCI 1H-
PULPROG zgpg30
TD 131072
SOLVENT D2O
NS 1000
DS 0
SWH 39062.500 Hz
FIDRES 0.298023 Hz
AQ 1.6777216 sec
RG 2050
DW 12.800 usec
DE 21.00 usec
TE 298.0 K
D1 2.00000000 sec
D11 0.03000000 sec
TD0 1
======== CHANNEL f1 ========
NUC1 13C
P1 11.30 usec
PL1 -1.50 dB
PL1W 113.54028320 W
SFO1 150.9201519 MHz
======== CHANNEL f2 ========
CPDPRG[2 waltz16
NUC2 1H
PCPD2 90.00 usec
PL2 4.00 dB
PL12 24.00 dB
PL13 27.00 dB
PL2W 6.09999990 W
PL12W 0.06100000 W
PL13W 0.03057242 W
SFO2 600.1324005 MHz
F2 - Processing parameters
SF 150.9028090 MHz
WDW EM
SSB 0
LB 2.00 Hz
GB 0
PC 1.00
^-O_2C
OH
O
O
O
O
O
H
^-O_3SO
HO
H
O^iPr
2
6
190 180 170 160 150 140 130 120 110 100 90 80 70 60 50 40 30 20 ppm

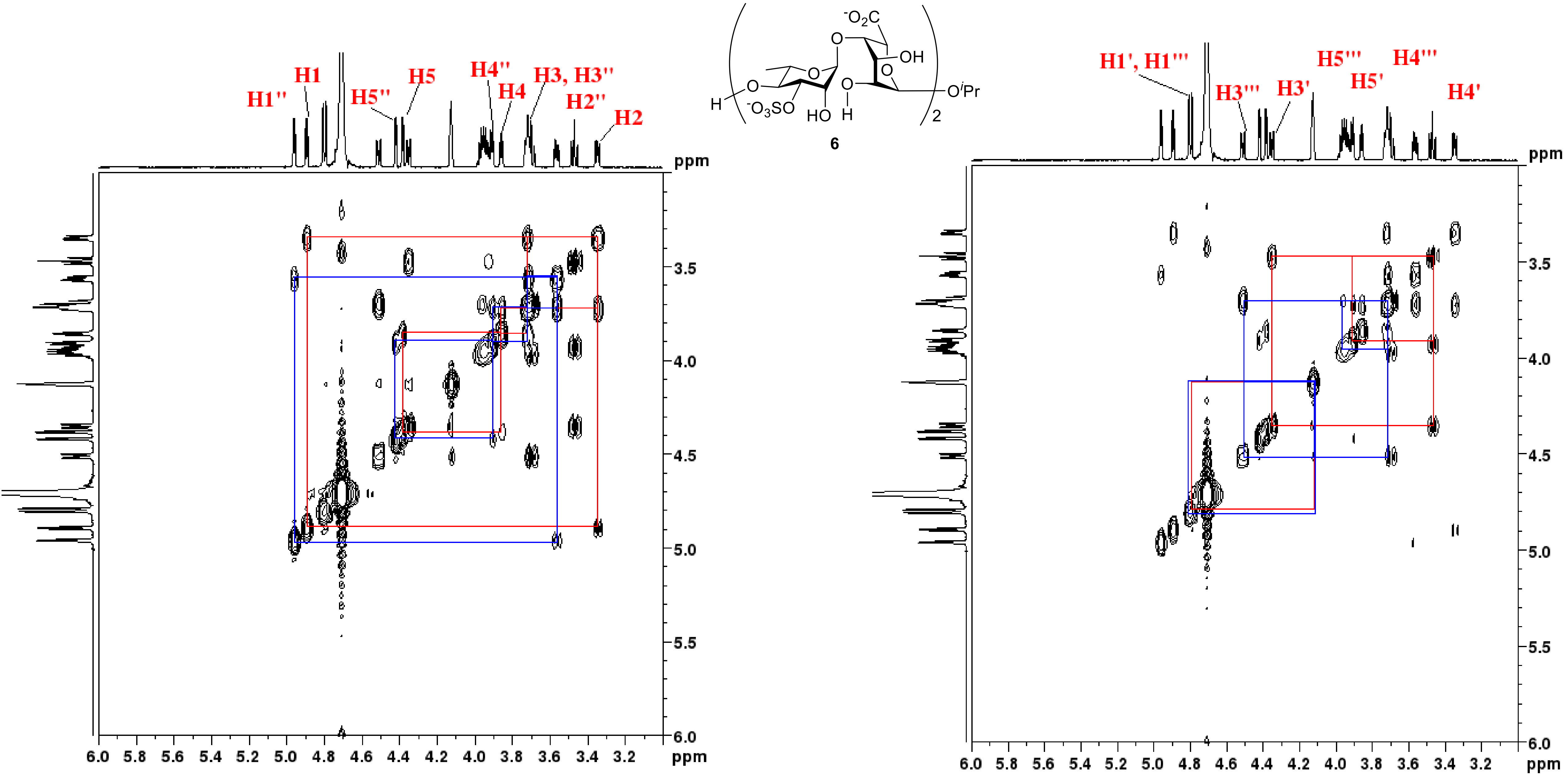
H1''
H1
H5''
H5
H4''
H4
H3, H3''
H2''
H2
ppm
6
H1', H1'''
H3'''
H3'
H5'''
H5'
H4'''
H4'

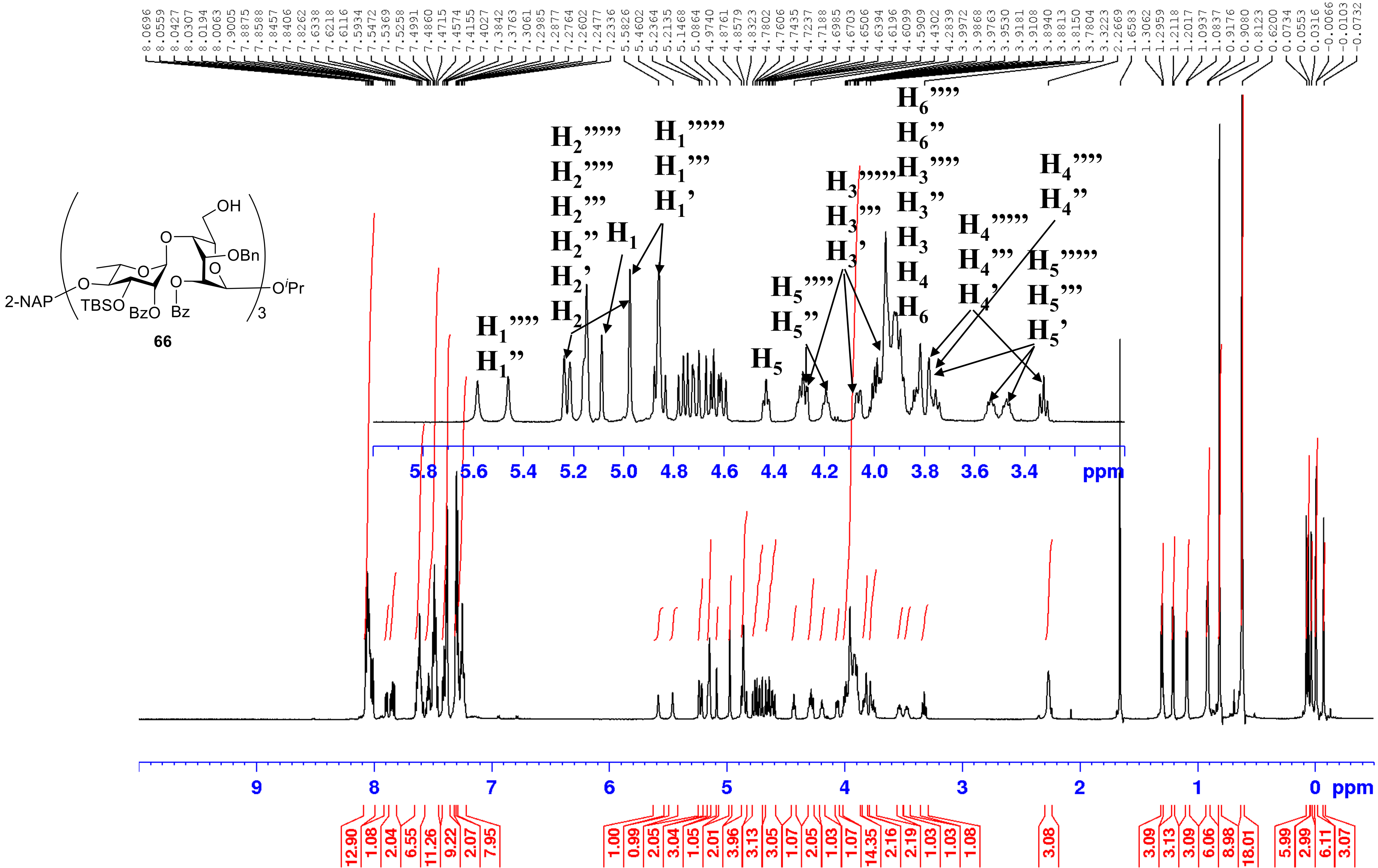
66

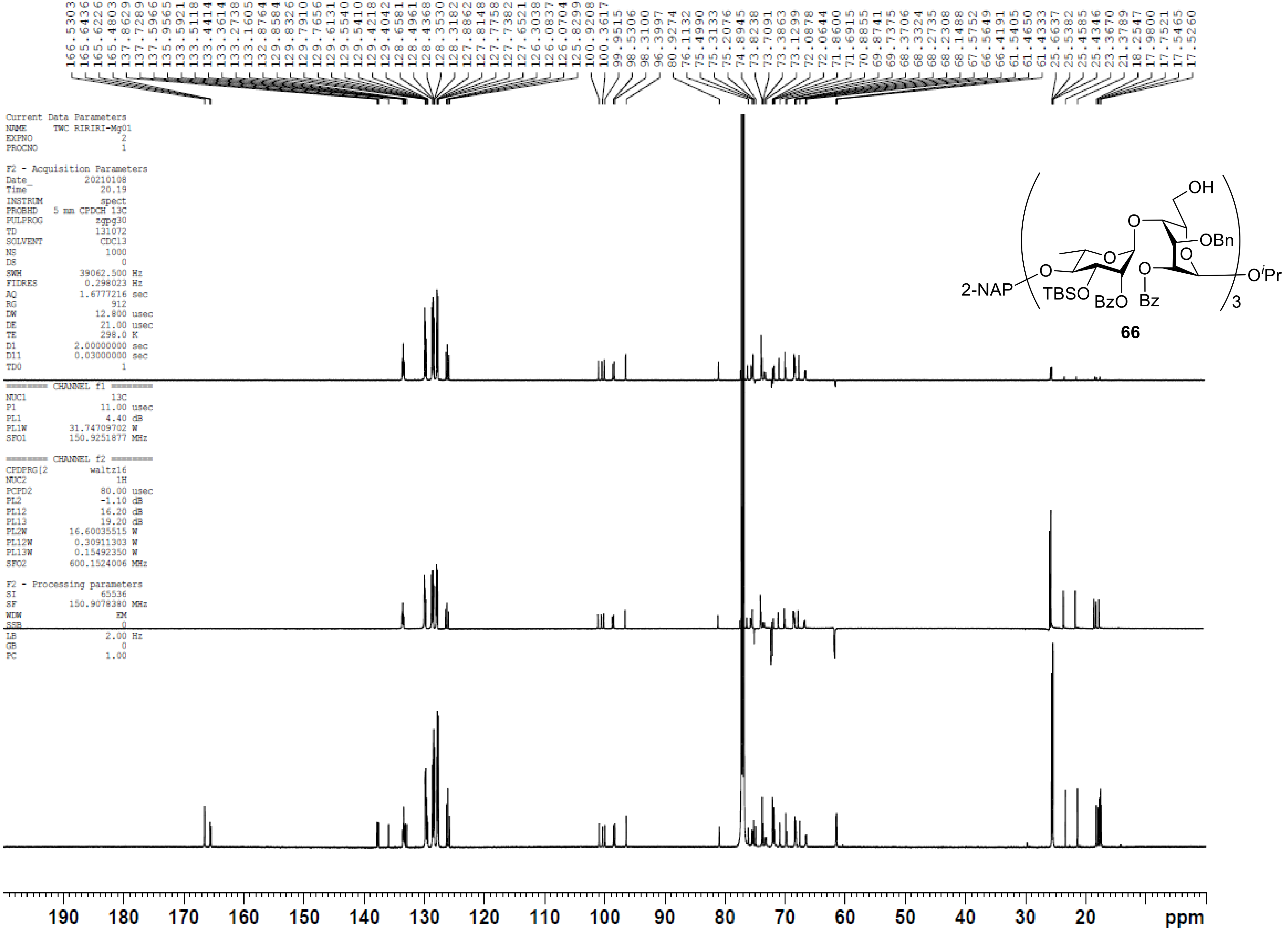
166.5303
165.6436
165.6226
165.4803
137.8629
137.7289
137.5966
135.9565
133.5921
133.5118
133.4414
133.3614
133.2738
133.1605
132.8764
129.8584
129.8326
129.7910
129.7656
129.6131
129.5540
129.5410
129.4218
129.4042
128.6581
128.4961
128.4368
128.3530
128.3182
127.8862
127.8148
127.7758
127.7382
127.6521
126.3038
126.0837
126.0704
125.8299
100.9208
100.3617
99.9515
98.5306
98.3100
96.3997
80.9274
76.1132
75.4990
75.3133
75.2076
74.8945
73.8238
73.7091
73.3863
73.1299
72.0878
72.0644
71.8600
71.6915
70.8855
69.8741
69.7375
68.3706
68.3324
68.2735
68.2308
68.1488
67.5752
66.5649
66.4191
61.5405
61.4650
61.4333
25.6637
25.5382
25.4585
25.4346
23.3670
21.3789
18.2547
17.9800
17.7521
17.5465
17.5260
Current Data Parameters
NAME TWC RIRIRI-Mg01
EXPNO 2
PROCNO 1
F2 - Acquisition Parameters
Date_ 20210108
Time 20.19
INSTRUM spect
PROBHD 5 mm CPDCH 13C
PULPROG zgpg30
TD 131072
SOLVENT CDCl3
NS 1000
DS 0
SWH 39062.500 Hz
FIDRES 0.298023 Hz
AQ 1.6777216 sec
RG 912
DW 12.800 usec
DE 21.00 usec
TE 298.0 K
D1 2.00000000 sec
D11 0.03000000 sec
TD0 1
======== CHANNEL f1 ========
NUC1 13C
P1 11.00 usec
PL1 4.40 dB
PL1W 31.74709702 W
SFO1 150.9251877 MHz
======== CHANNEL f2 ========
CPDPRG[2 waltz16
NUC2 1H
PCPD2 80.00 usec
PL2 -1.10 dB
PL12 16.20 dB
PL13 19.20 dB
PL2W 16.60035515 W
PL12W 0.30911303 W
PL13W 0.15492350 W
SFO2 600.1524006 MHz
F2 - Processing parameters
SI 65536
SF 150.9078380 MHz
WDW EM
SSB 0
LB 2.00 Hz
GB 0
PC 1.00
190 180 170 160 150 140 130 120 110 100 90 80 70 60 50 40 30 20 ppm
OH
O
OBn
O
O
2-NAP
TBSO
BzO
O
Bz
O^{i}Pr
3

66

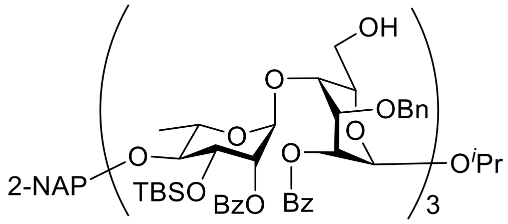
OH
O
OBn
O
O
O
2-NAP
TBSO
BzO
O
Bz
O^{i}Pr
3

66

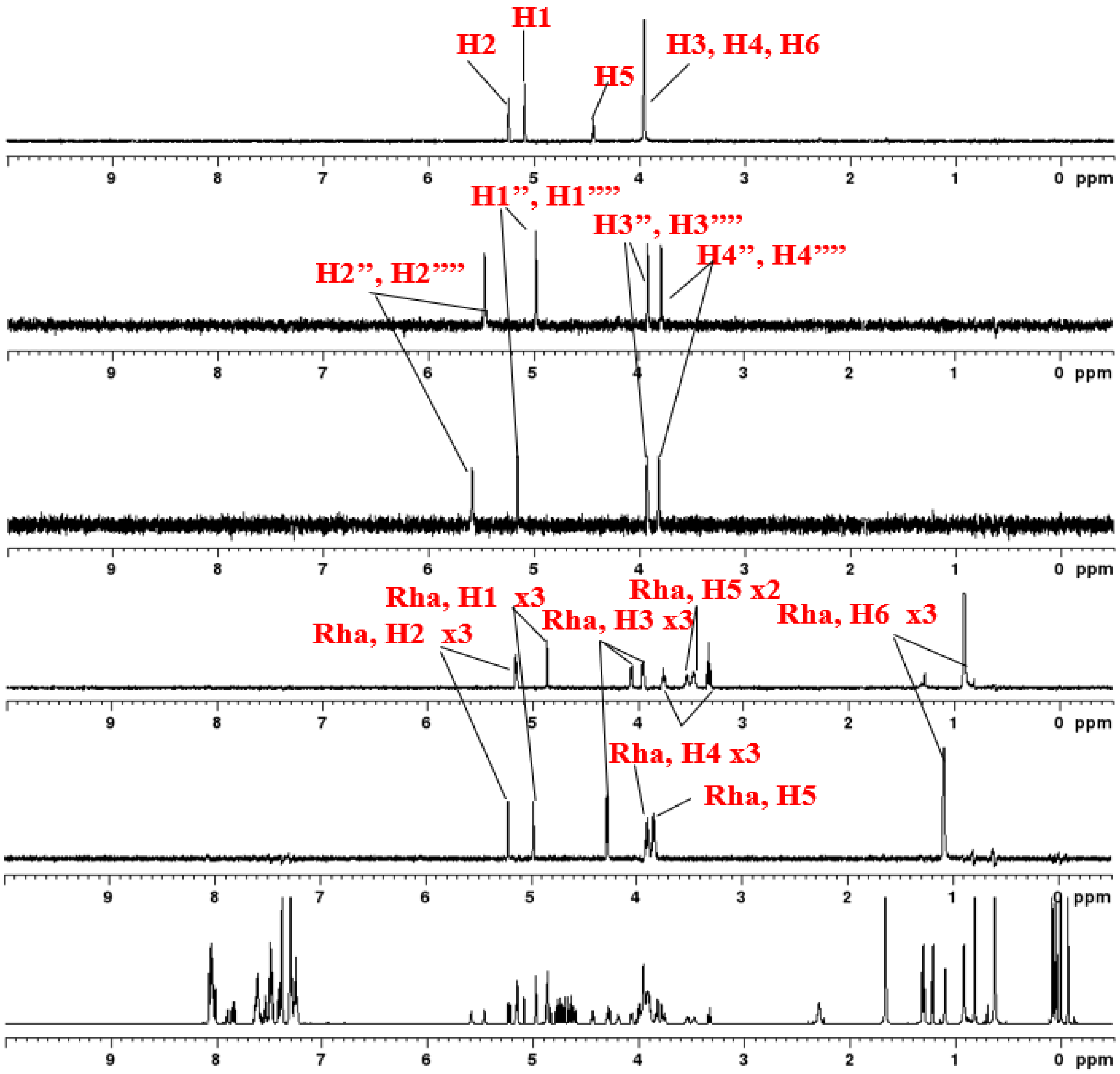
H1
H2
H5
H3, H4, H6
H1", H1"""
H3", H3"""
H4", H4"""
H2", H2"""
Rha, H1 x3
Rha, H5 x2
Rha, H3 x3
Rha, H6 x3
Rha, H2 x3
Rha, H4 x3
Rha, H5
9
8
7
6
5
4
3
2
1
0
ppm

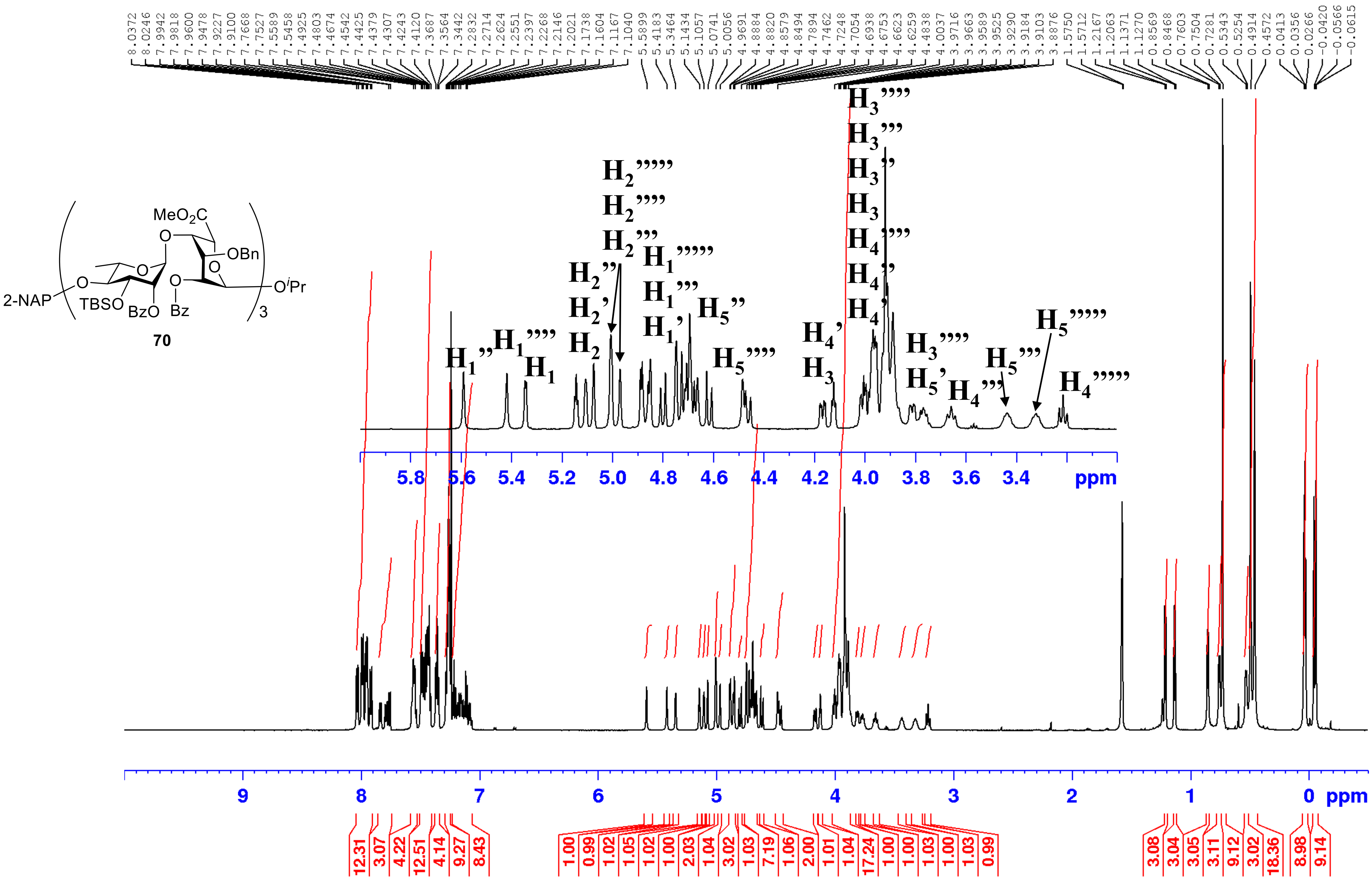

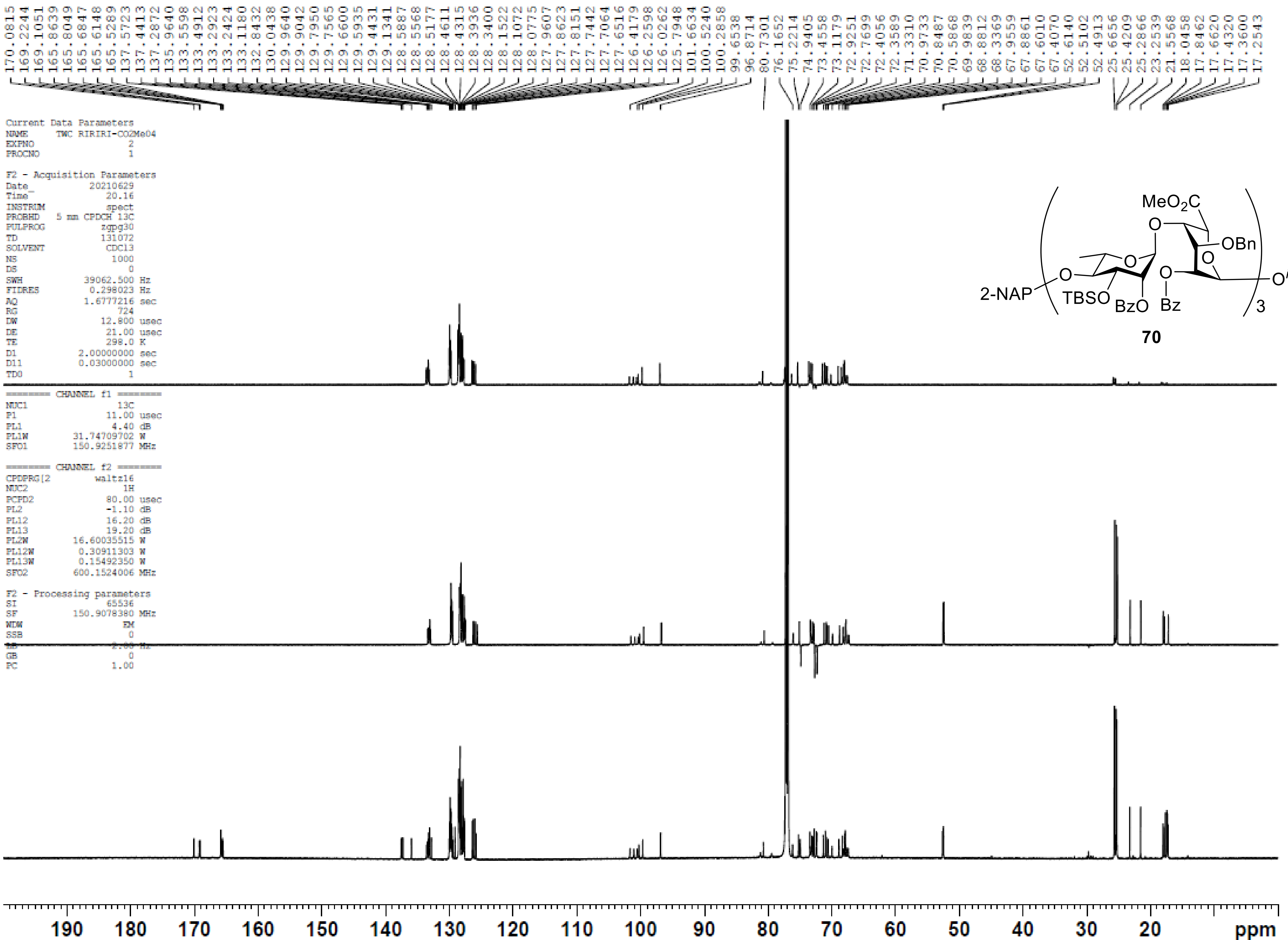
170.0815
169.2244
169.1051
165.8639
165.8049
165.6847
165.6148
165.5289
137.5723
137.4413
137.2872
135.9640
133.5598
133.4912
133.2923
133.2424
133.1180
132.8432
130.0438
129.9640
129.9042
129.7950
129.7565
129.6600
129.5935
129.4431
129.1341
128.5887
128.5568
128.5177
128.4611
128.4315
128.3936
128.3400
128.1522
128.1072
128.0775
127.9607
127.8623
127.8151
127.7442
127.7064
127.6516
126.4179
126.2598
126.0262
125.7948
101.6634
100.5240
100.2858
99.6538
96.8714
80.7301
76.1652
75.2214
74.9405
73.4558
73.1179
72.9251
72.7699
72.4056
72.3589
71.3310
70.9733
70.8487
70.5868
69.9839
68.8812
68.3369
67.9559
67.8861
67.6010
67.4070
67.6140
52.5102
52.4913
25.6656
25.4209
25.2866
23.2539
21.5568
18.0458
17.6620
17.4320
17.3600
17.2543
Current Data Parameters
NAME TWC RIRIRI-CO2Me04
EXPNO 2
PROCNO 1
F2 - Acquisition Parameters
Date_ 20210629
Time 20.16
INSTRUM spect
PROBHD 5 mm CPDCH 13C
PULPROG zgpg30
TD 131072
SOLVENT CDCl3
NS 1000
DS 0
SWH 39062.500 Hz
FIDRES 0.298023 Hz
AQ 1.6777216 sec
RG 724
DW 12.800 usec
DE 21.00 usec
TE 298.0 K
D1 2.00000000 sec
D11 0.03000000 sec
TD0 1
======== CHANNEL f1 ========
NUC1 13C
P1 11.00 usec
PL1 4.40 dB
PL1W 31.74709702 W
SFO1 150.9251877 MHz
======== CHANNEL f2 ========
CPDPRG[2 waltz16
NUC2 1H
PCPD2 80.00 usec
PL2 -1.10 dB
PL12 16.20 dB
PL13 19.20 dB
PL2W 16.60035515 W
PL12W 0.30911303 W
PL13W 0.15492350 W
SFO2 600.1524006 MHz
F2 - Processing parameters
SI 65536
SF 150.9078380 MHz
WDW EM
SSB 0
LB 2.00 Hz
GB 0
PC 1.00
190
180
170
160
150
140
130
120
110
100
90
80
70
60
50
40
30
20
ppm
MeO2C
O
OBn
O
O
O
O
O-iPr
2-NAP
TBSO
BzO
Bz
3

70

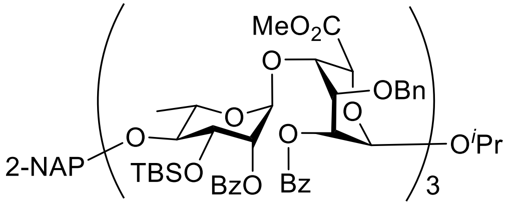
MeO2C
OBn
O
2-NAP
TBSO
BzO
Bz
O^{i}Pr
3

70

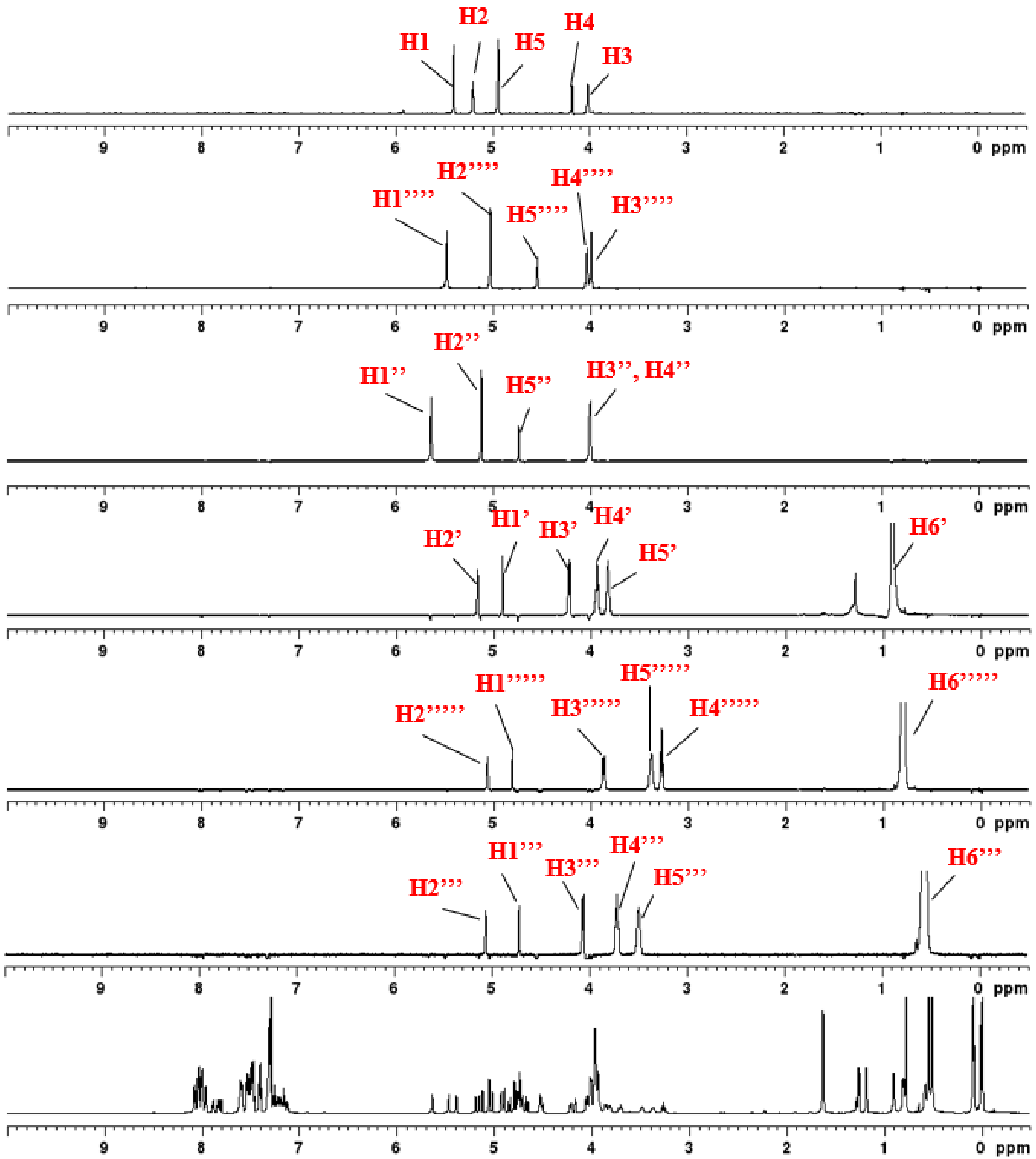
H1
H2
H5
H4
H3
H1''''
H2''''
H5''''
H4''''
H3''''
H1''
H2''
H5''
H3'', H4''
H2'
H1'
H3'
H4'
H5'
H6'
H2'''''
H1'''''
H3'''''
H5'''''
H4'''''
H6'''''
H2'''
H1'''
H3'''
H4'''
H5'''
H6'''
9 8 7 6 5 4 3 2 1 0 ppm

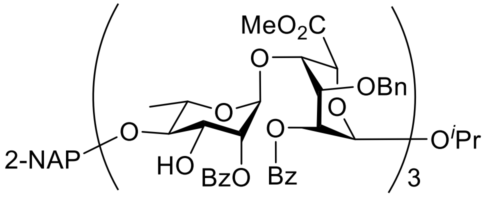
MeO2C
O
OBn
O
O
O
2-NAP
HO
BzO
Bz
OiPr
3

74

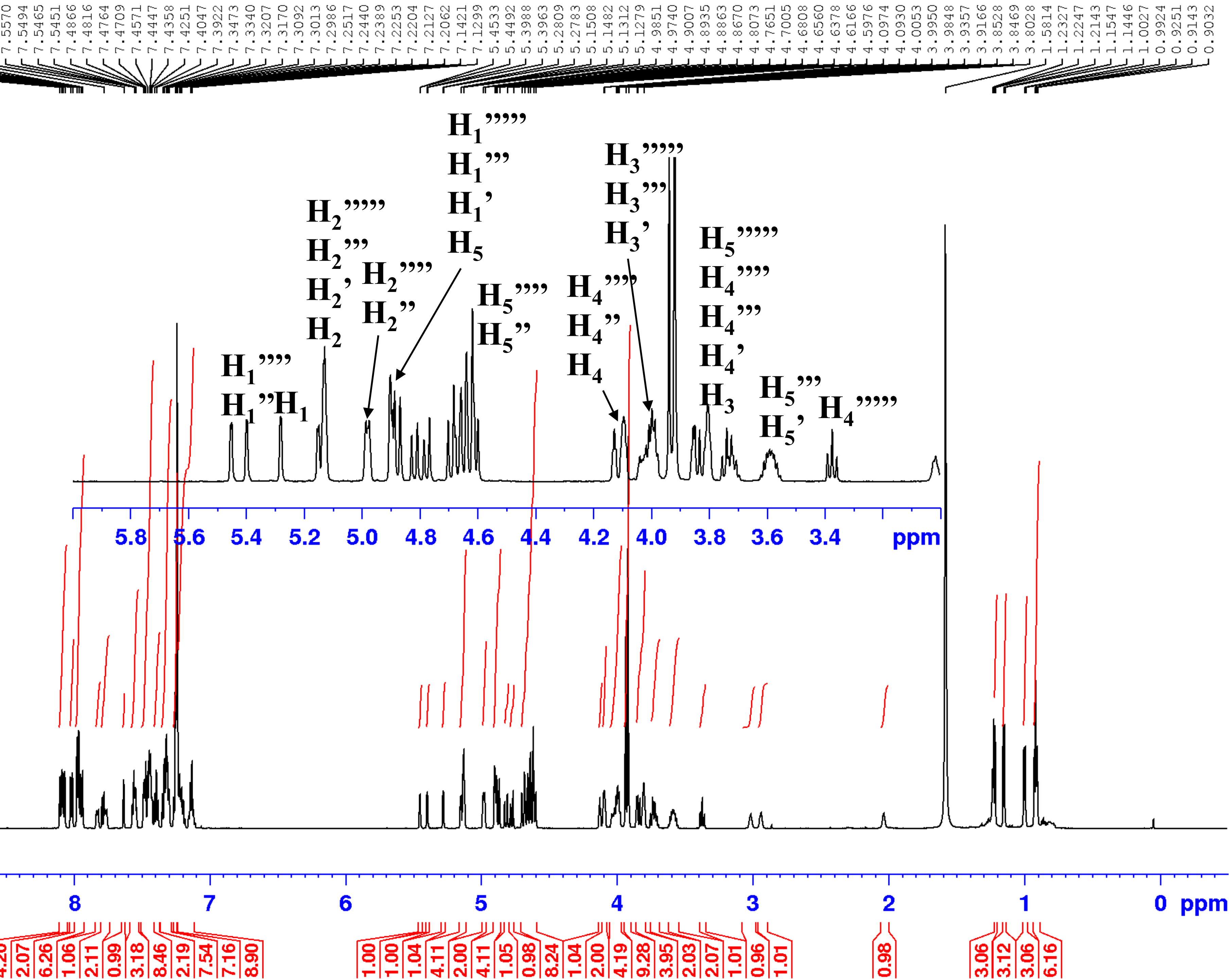
8.1037 8.0904 8.0811 8.0678 8.0249 8.0116 7.9764 7.9642 7.9498 7.9364 7.7787 7.6336 7.5570 7.5494 7.5465 7.5451 7.4866 7.4816 7.4764 7.4709 7.4571 7.4447 7.4358 7.4251 7.4047 7.3922 7.3473 7.3340 7.3207 7.3170 7.3092 7.3013 7.2986 7.2517 7.2440 7.2389 7.2253 7.2204 7.2127 7.2062 7.1421 7.1299 5.4533 5.4492 5.3988 5.3963 5.2809 5.2783 5.1508 5.1482 5.1312 5.1279 4.9851 4.9740 4.9007 4.8935 4.8863 4.8670 4.8073 4.7651 4.7005 4.6808 4.6560 4.6378 4.6166 4.5976 4.0974 4.0930 4.0053 3.9950 3.9848 3.9357 3.9166 3.8528 3.8469 3.8028 1.5814 1.2327 1.2247 1.2143 1.1547 1.1446 1.0027 0.9924 0.9251 0.9143 0.9032
H1'''' H1'' H1
H2''''' H2''' H2' H2
H2'''' H2''
H1''''' H1''' H1' H5
H5'''' H5''
H4'''' H4'' H4
H3''''' H3''' H3'
H5''''' H4'''' H4''' H4' H3
H5''' H5'
H4'''''
5.8 5.6 5.4 5.2 5.0 4.8 4.6 4.4 4.2 4.0 3.8 3.6 3.4 ppm
9 8 7 6 5 4 3 2 1 0 ppm
4.20 2.07 6.26 1.06 2.11 0.99 3.18 8.46 2.19 7.54 7.16 8.90 1.00 1.00 1.04 4.11 2.00 4.11 1.05 0.98 8.24 1.04 2.00 4.19 9.28 3.95 2.03 2.07 1.01 0.96 1.01 0.98 3.06 3.12 3.06 6.16

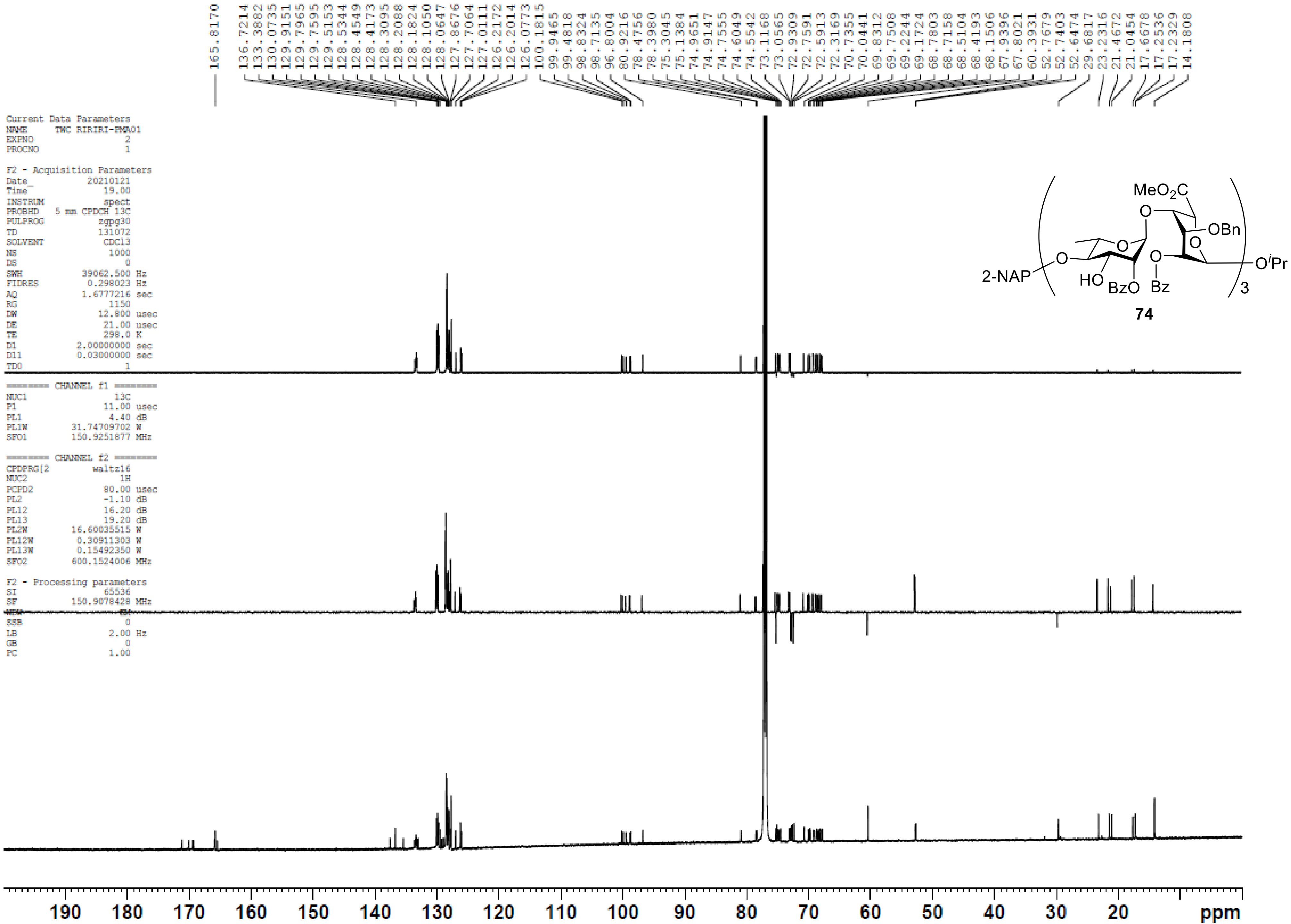
165.8170
136.7214
133.3882
130.0735
129.9151
129.7965
129.7595
129.5153
128.5344
128.4549
128.4173
128.3095
128.2088
128.1824
128.1050
128.0647
127.8676
127.7064
127.0111
126.2172
126.2014
126.0773
100.1815
99.9465
99.4818
98.8324
98.7135
96.8004
80.9216
78.4756
78.3980
75.3045
75.1384
74.9651
74.9147
74.7555
74.6049
74.5542
73.1168
73.0565
72.9309
72.7591
72.5913
72.3169
70.7355
70.0441
69.8312
69.7508
69.2244
69.1724
68.7803
68.7158
68.5104
68.4193
68.1506
67.9396
67.8021
60.3931
52.7679
52.7403
52.6474
29.6817
23.2316
21.4672
21.0454
17.6678
17.2536
17.2329
14.1808
Current Data Parameters
NAME TWC RIRIRI-PMA01
EXPNO 2
PROCNO 1
F2 - Acquisition Parameters
Date_ 20210121
Time 19.00
INSTRUM spect
PROBHD 5 mm CPDCH 13C
PULPROG zgpg30
TD 131072
SOLVENT CDCl3
NS 1000
DS 0
SWH 39062.500 Hz
FIDRES 0.298023 Hz
AQ 1.6777216 sec
RG 1150
DW 12.800 usec
DE 21.00 usec
TE 298.0 K
D1 2.00000000 sec
D11 0.03000000 sec
TD0 1
======== CHANNEL f1 ========
NUC1 13C
P1 11.00 usec
PL1 4.40 dB
PL1W 31.74709702 W
SFO1 150.9251877 MHz
======== CHANNEL f2 ========
CPDPRG[2 waltz16
NUC2 1H
PCPD2 80.00 usec
PL2 -1.10 dB
PL12 16.20 dB
PL13 19.20 dB
PL2W 16.60035515 W
PL12W 0.30911303 W
PL13W 0.15492350 W
SFO2 600.1524006 MHz
F2 - Processing parameters
SI 65536
SF 150.9078428 MHz
SSB 0
LB 2.00 Hz
GB 0
PC 1.00
190 180 170 160 150 140 130 120 110 100 90 80 70 60 50 40 30 20 ppm
MeO2C
O
OBn
O
O
O
O
O
2-NAP
HO
BzO
Bz
O^iPr
3

74

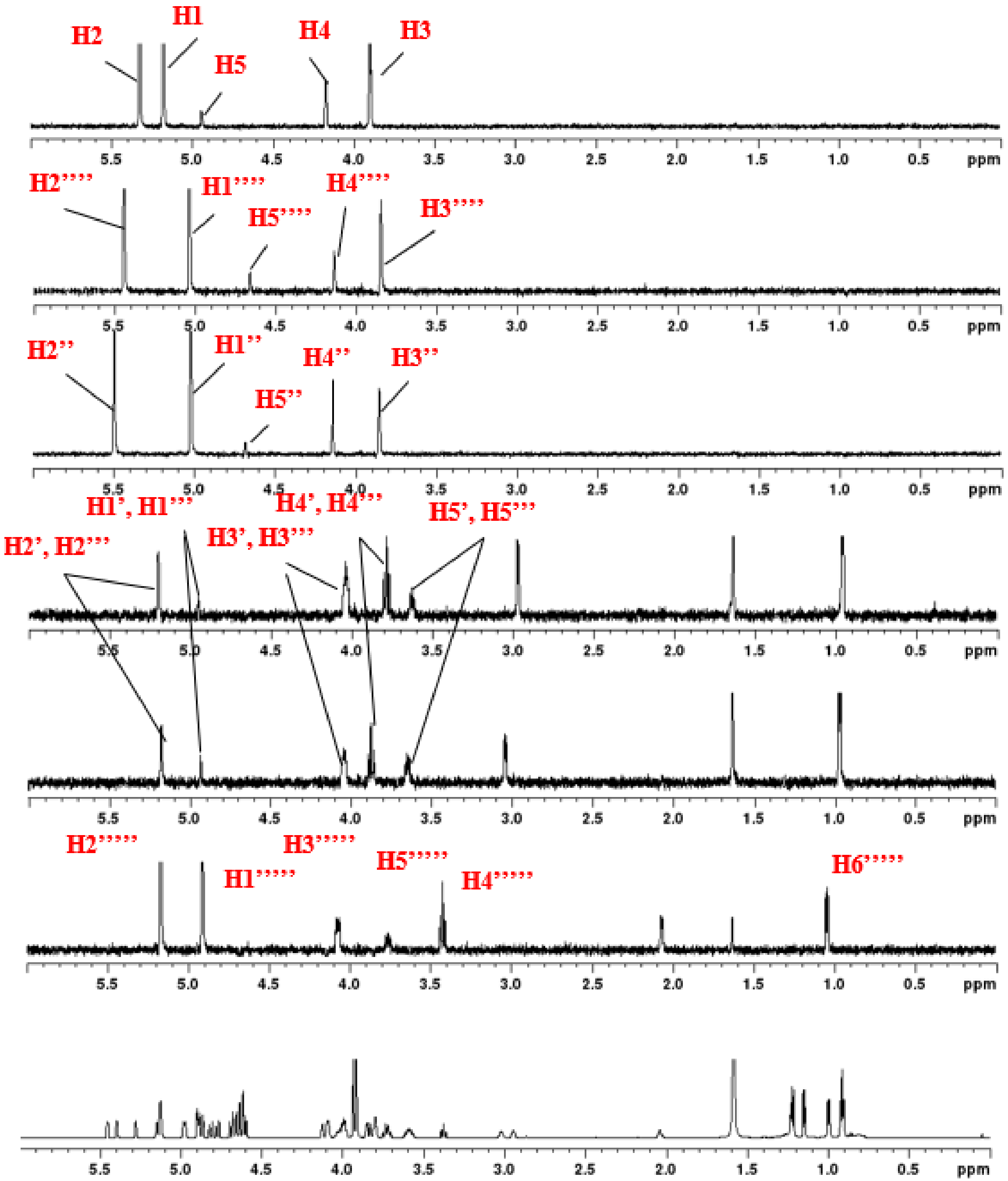
H2
H1
H5
H4
H3
H2''''
H1''''
H5''''
H4''''
H3''''
H2''
H1''
H5''
H4''
H3''
H1', H1'''
H4', H4'''
H5', H5'''
H2', H2'''
H3', H3'''
H2'''''
H3'''''
H1'''''
H5'''''
H4'''''
H6'''''
ppm

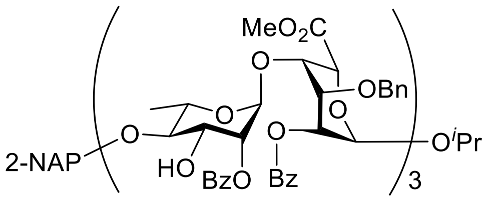
MeO2C
OBn
2-NAP
HO
BzO
Bz
OiPr
3

74

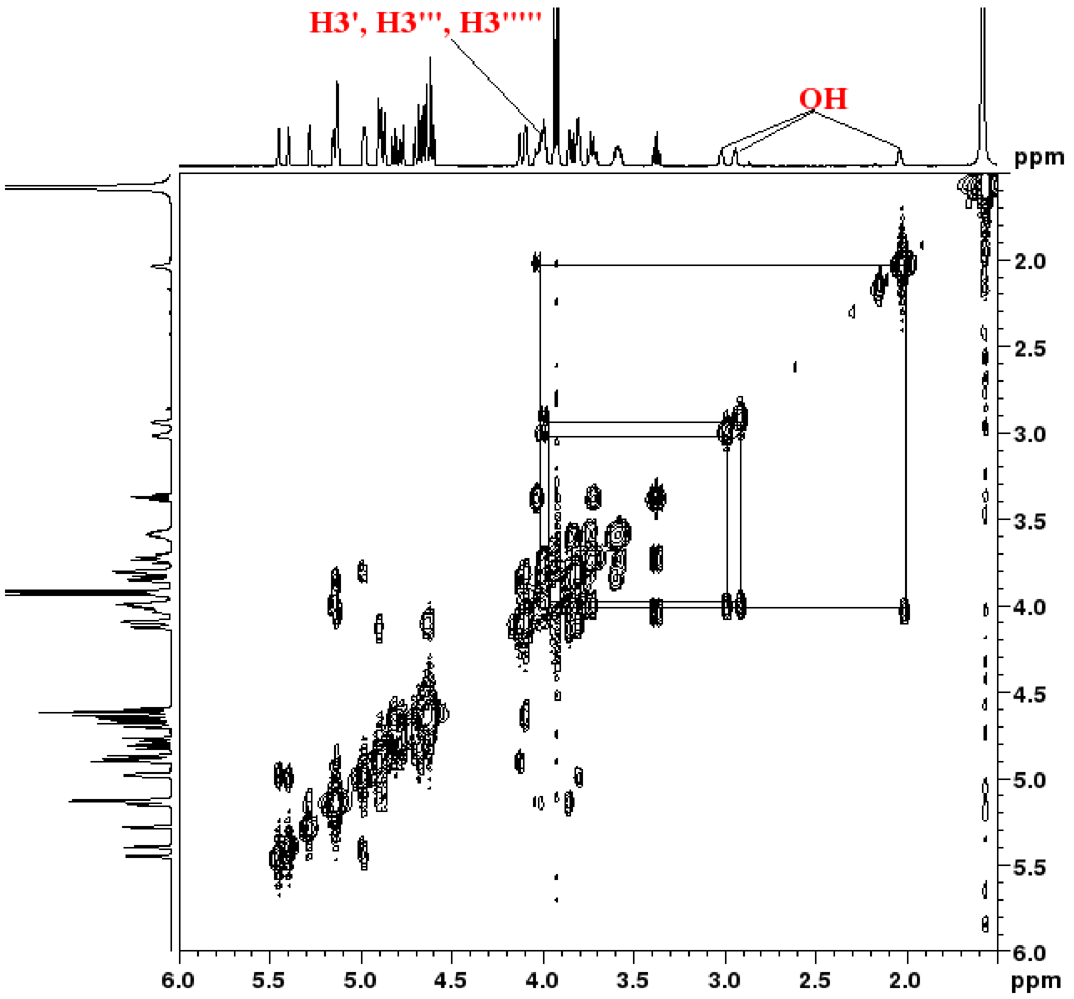
H3', H3''', H3'''''
OH
ppm

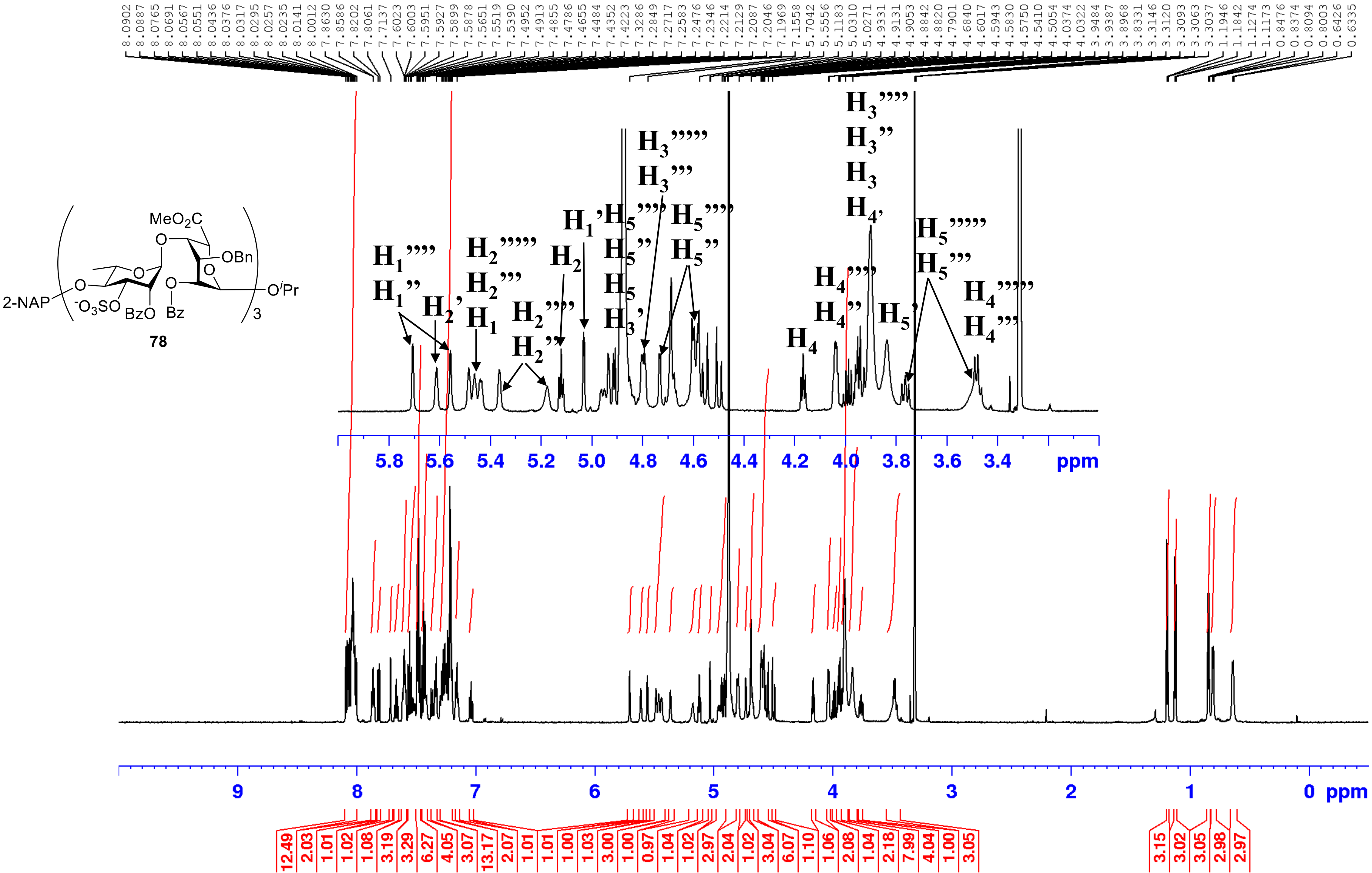

H1'''' H1''
H2'
H2''''' H2''' H1
H2'''' H2''
H2
H1' H5'''' H5'' H5 H3'
H3''''' H3'''
H5'''' H5''
H4
H4'''' H4''
H3'''' H3'' H3 H4'
H5'
H5''''' H5'''
H4''''' H4'''
ppm

78

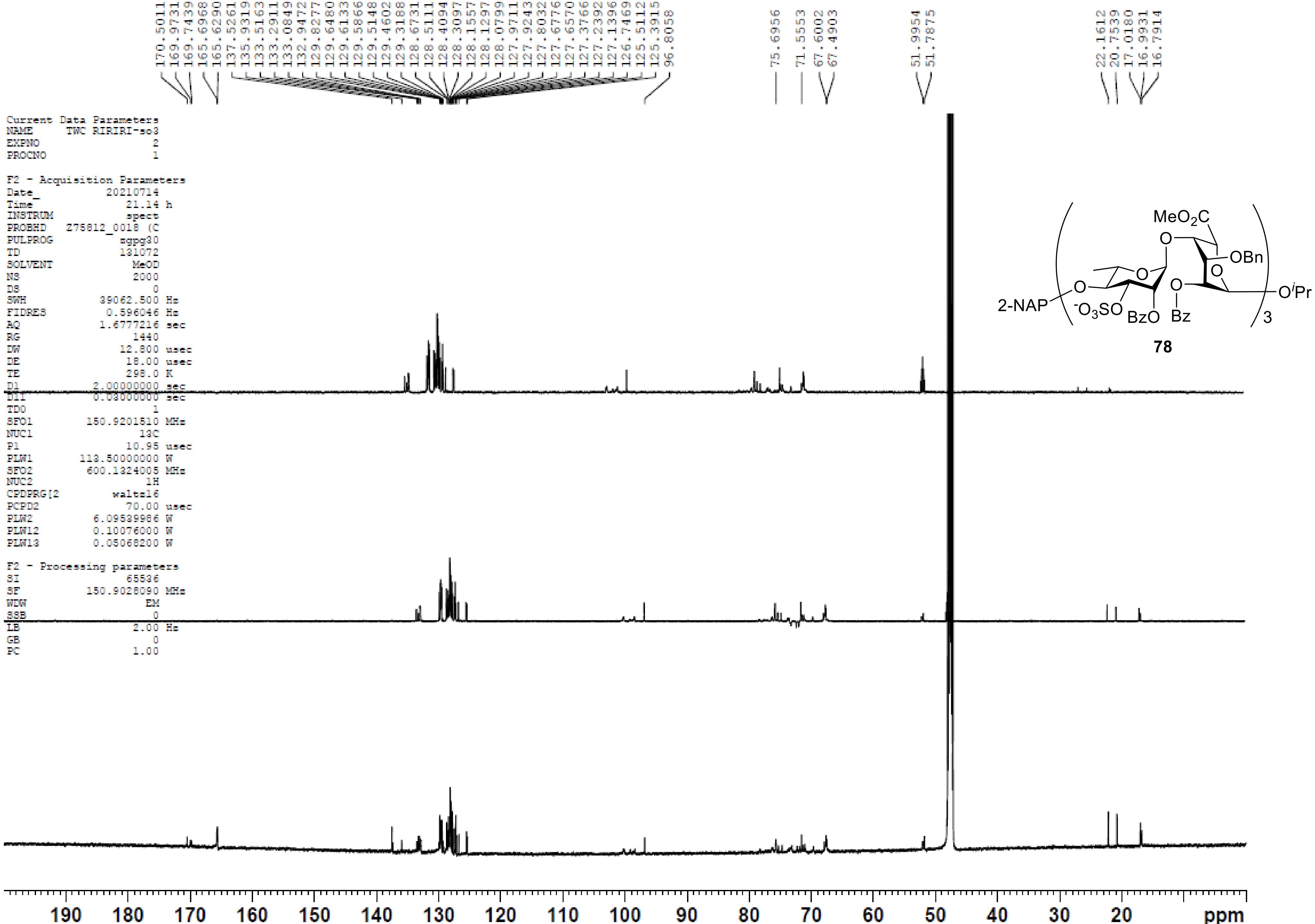
170.5011
169.9731
169.7439
165.6968
165.6290
137.5261
135.9319
133.5163
133.2911
133.0849
132.9472
129.8277
129.6480
129.6133
129.5866
129.5148
129.4602
129.3188
128.6731
128.5111
128.4094
128.3097
128.1557
128.1297
128.0799
127.9711
127.9243
127.8032
127.6776
127.6570
127.3766
127.2392
127.1396
126.7469
125.5112
125.3915
96.8058
75.6956
71.5553
67.6002
67.4903
51.9954
51.7875
22.1612
20.7539
17.0180
16.9931
16.7914
Current Data Parameters
NAME TWC RIRIRI-so3
EXPNO 2
PROCNO 1
F2 - Acquisition Parameters
Date_ 20210714
Time 21.14 h
INSTRUM spect
PROBHD Z75812_0018 (C
PULPROG zgpg30
TD 131072
SOLVENT MeOD
NS 2000
DS 0
SWH 39062.500 Hz
FIDRES 0.596046 Hz
AQ 1.6777216 sec
RG 1440
DW 12.800 usec
DE 18.00 usec
TE 298.0 K
D1 2.00000000 sec
D11 0.03000000 sec
TD0 1
SFO1 150.9201510 MHz
NUC1 13C
P1 10.95 usec
PLW1 113.50000000 W
SFO2 600.1324005 MHz
NUC2 1H
CPDPRG[2 waltz16
PCPD2 70.00 usec
PLW2 6.09539986 W
PLW12 0.10076000 W
PLW13 0.05068200 W
F2 - Processing parameters
SI 65536
SF 150.9028090 MHz
WDW EM
SSB 0
LB 2.00 Hz
GB 0
PC 1.00
MeO2C
O
OBn
O
O
O
2-NAP
O
-O3SO
BzO
Bz
OiPr
3
78
190 180 170 160 150 140 130 120 110 100 90 80 70 60 50 40 30 20 ppm

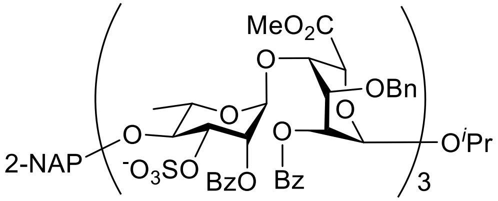
MeO2C
OBn
O
O
O
O
O
2-NAP
-O3SO
BzO
Bz
OiPr
3

78

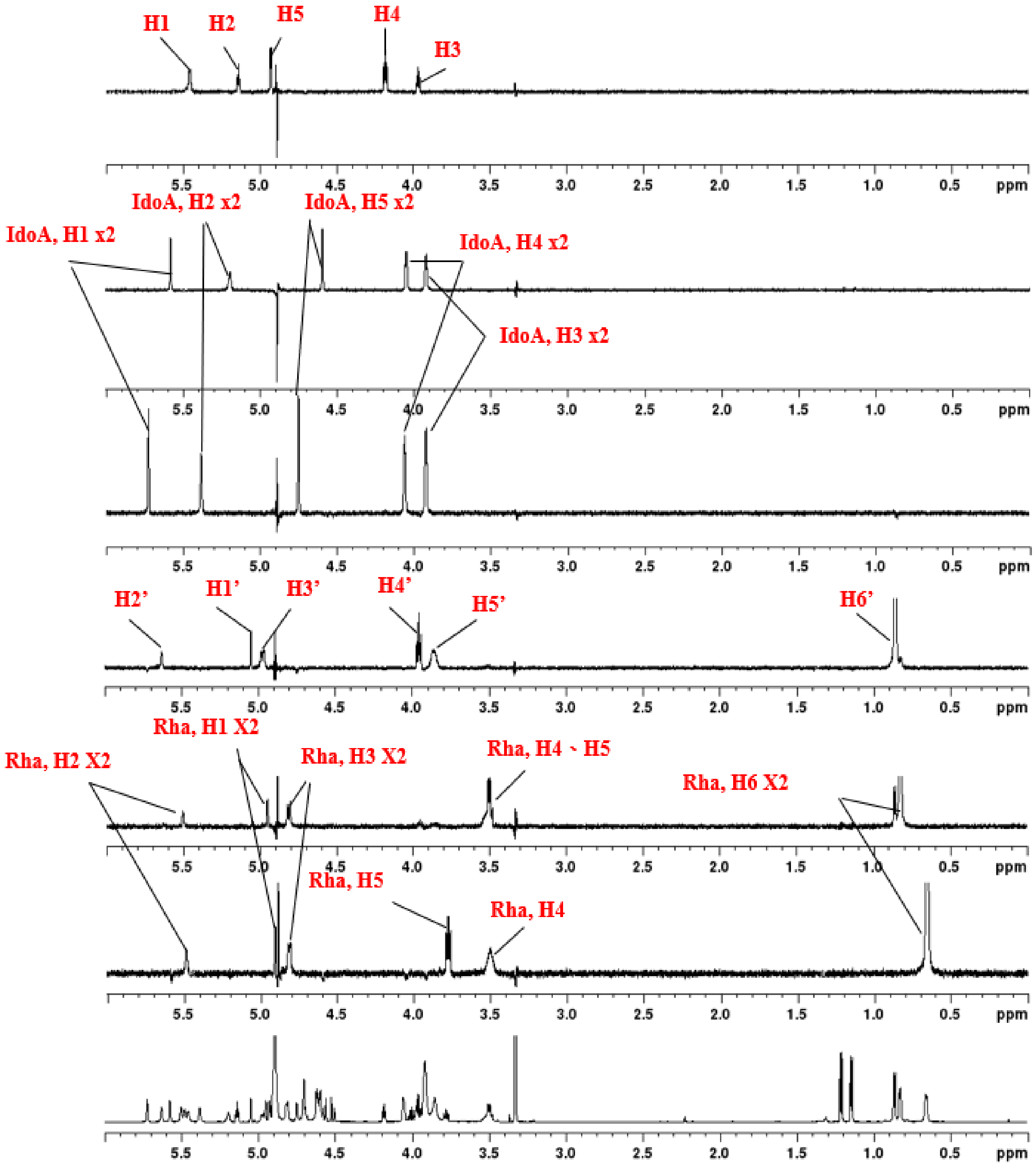
H1
H2
H5
H4
H3
IdoA, H2 x2
IdoA, H5 x2
IdoA, H1 x2
IdoA, H4 x2
IdoA, H3 x2
H2'
H1'
H3'
H4'
H5'
H6'
Rha, H1 X2
Rha, H2 X2
Rha, H3 X2
Rha, H4、H5
Rha, H6 X2
Rha, H5
Rha, H4
5.5
5.0
4.5
4.0
3.5
3.0
2.5
2.0
1.5
1.0
0.5
ppm

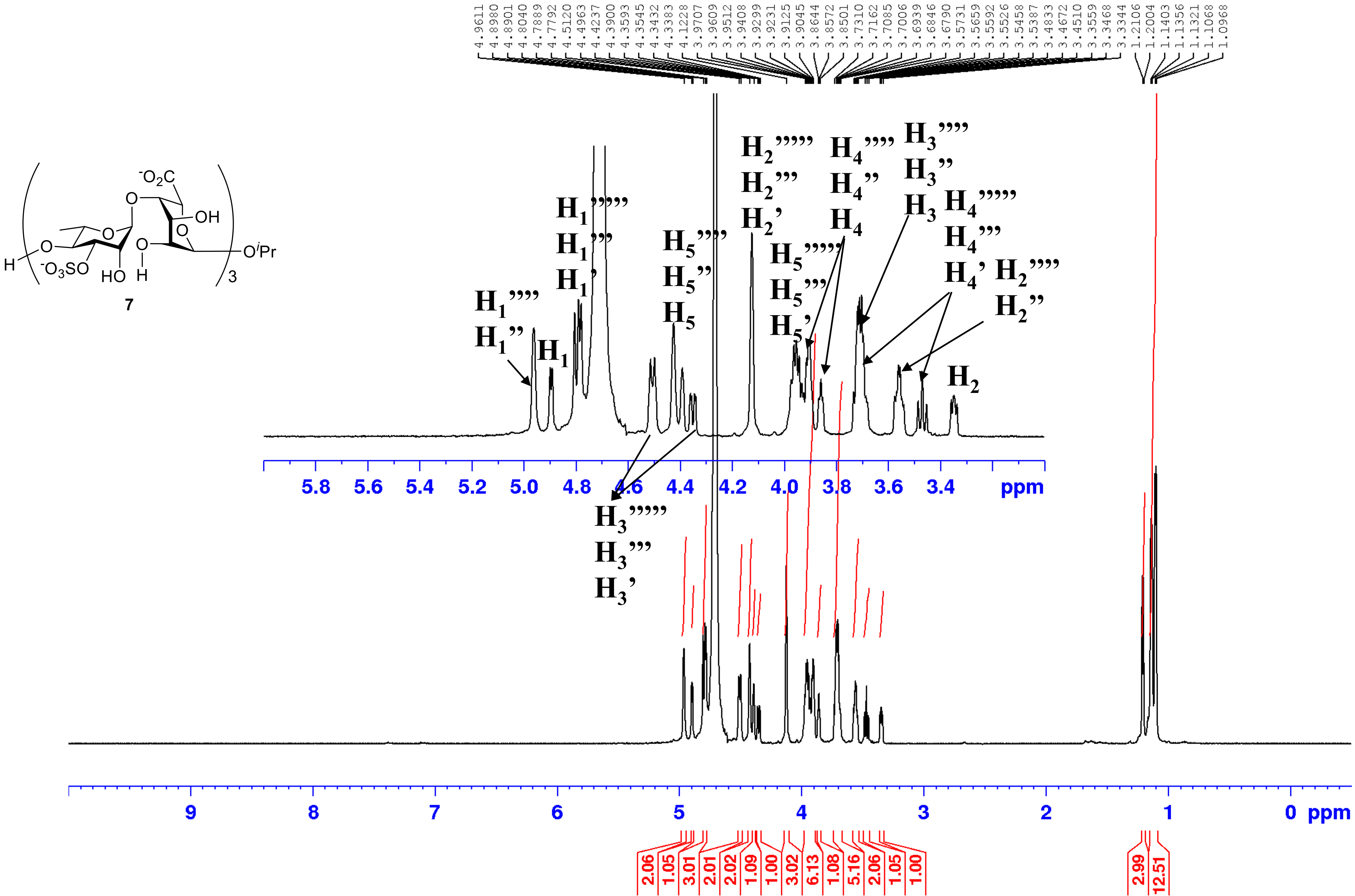

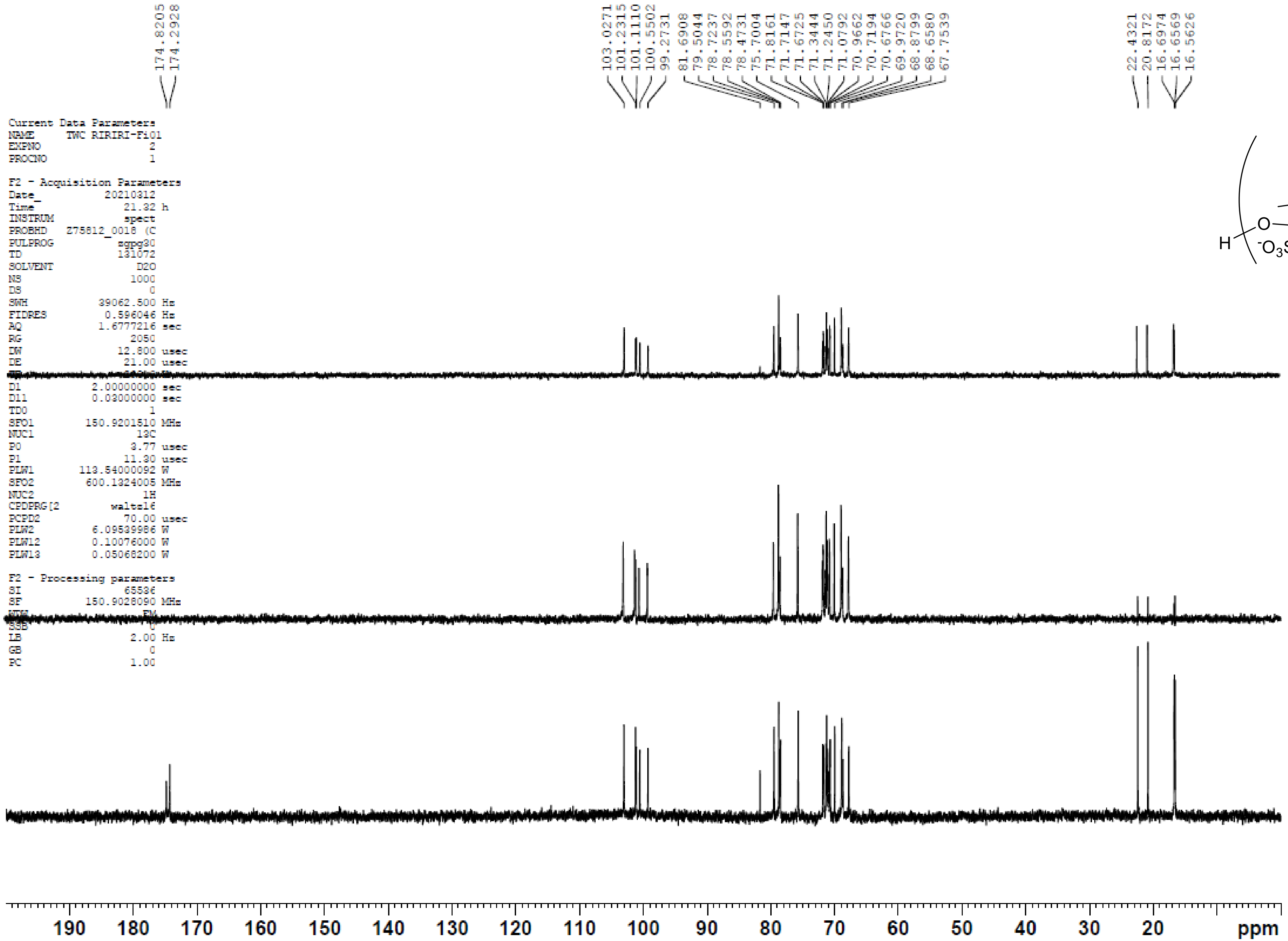
174.8205
174.2928
103.0271
101.2315
101.1110
100.5502
99.2731
81.6908
79.5044
78.7237
78.5592
78.4731
75.7004
71.8161
71.7147
71.6725
71.3444
71.2450
71.0792
70.9662
70.7194
70.6766
69.9720
68.8799
68.6580
67.7539
22.4321
20.8172
16.6974
16.6569
16.5626
Current Data Parameters
NAME TWC RIRIRI-Fi01
EXPNO 2
PROCNO 1
F2 - Acquisition Parameters
Date_ 20210312
Time 21.32 h
INSTRUM spect
PROBHD Z75812_0018 (C
PULPROG zgpg30
TD 131072
SOLVENT D2O
NS 1000
DS 0
SWH 39062.500 Hz
FIDRES 0.596046 Hz
AQ 1.6777216 sec
RG 2050
DW 12.800 usec
DE 21.00 usec
D1 2.00000000 sec
D11 0.03000000 sec
TD0 1
SFO1 150.9201510 MHz
NUC1 13C
P0 3.77 usec
P1 11.30 usec
PLW1 113.54000092 W
SFO2 600.1324005 MHz
NUC2 1H
CPDPRG[2 waltz16
PCPD2 70.00 usec
PLW2 6.09539986 W
PLW12 0.10076000 W
PLW13 0.05068200 W
F2 - Processing parameters
SI 65536
SF 150.9028090 MHz
LB 2.00 Hz
GB 0
PC 1.00
190 180 170 160 150 140 130 120 110 100 90 80 70 60 50 40 30 20 ppm

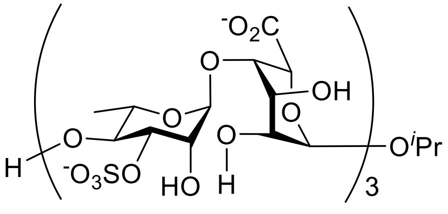
^-O_2C
OH
O
O
H
^-O_3SO
HO
H
O^iPr
3

7

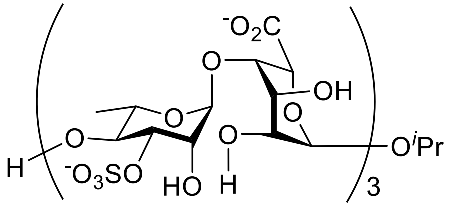

-O2C
O
OH
O
O
O
O
H
-O3SO
HO
H
OiPr
3

7

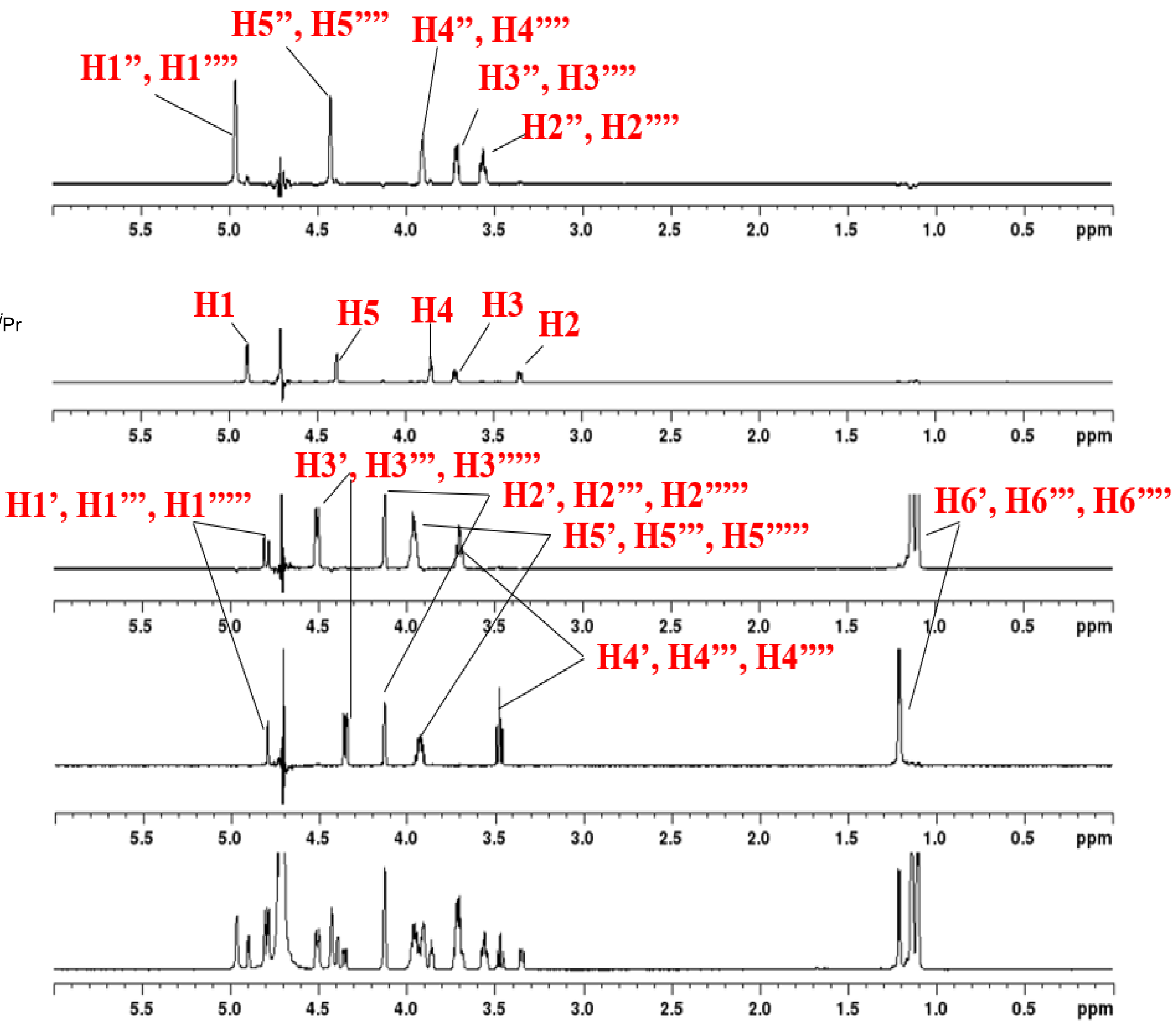

H1”, H1””’
H5”, H5””’
H4”, H4””’
H3”, H3””’
H2”, H2””’
H1
H5
H4
H3
H2
H3’, H3’”, H3’””
H2’, H2’”, H2’””
H1’, H1’”, H1’””
H5’, H5’”, H5’””
H6’, H6’”, H6’””
H4’, H4’”, H4’””
5.5
5.0
4.5
4.0
3.5
3.0
2.5
2.0
1.5
1.0
0.5
ppm

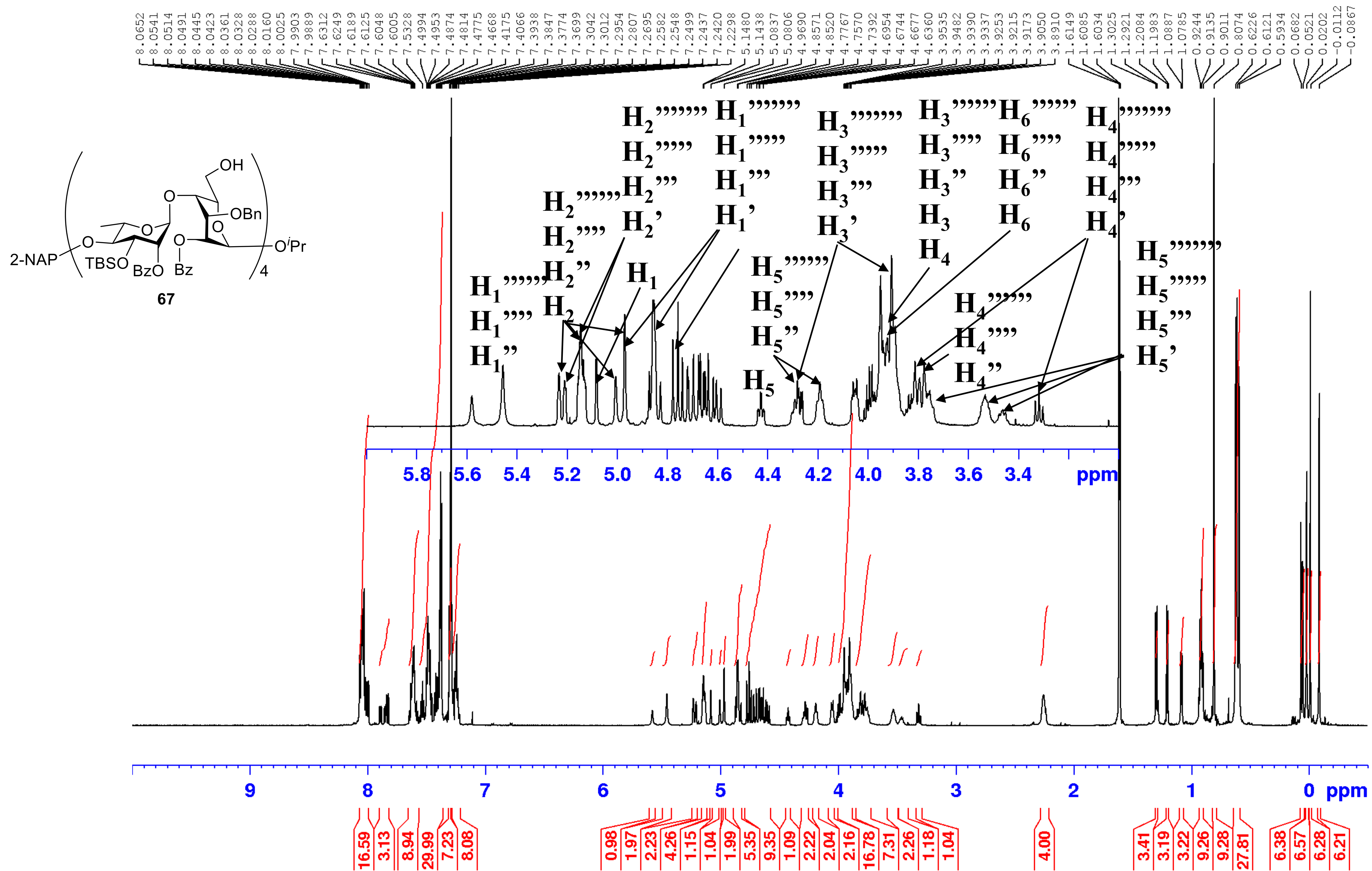

OH
OBn
2-NAP
TBSO
BzO
Bz
O^{i}Pr
4
67
ppm

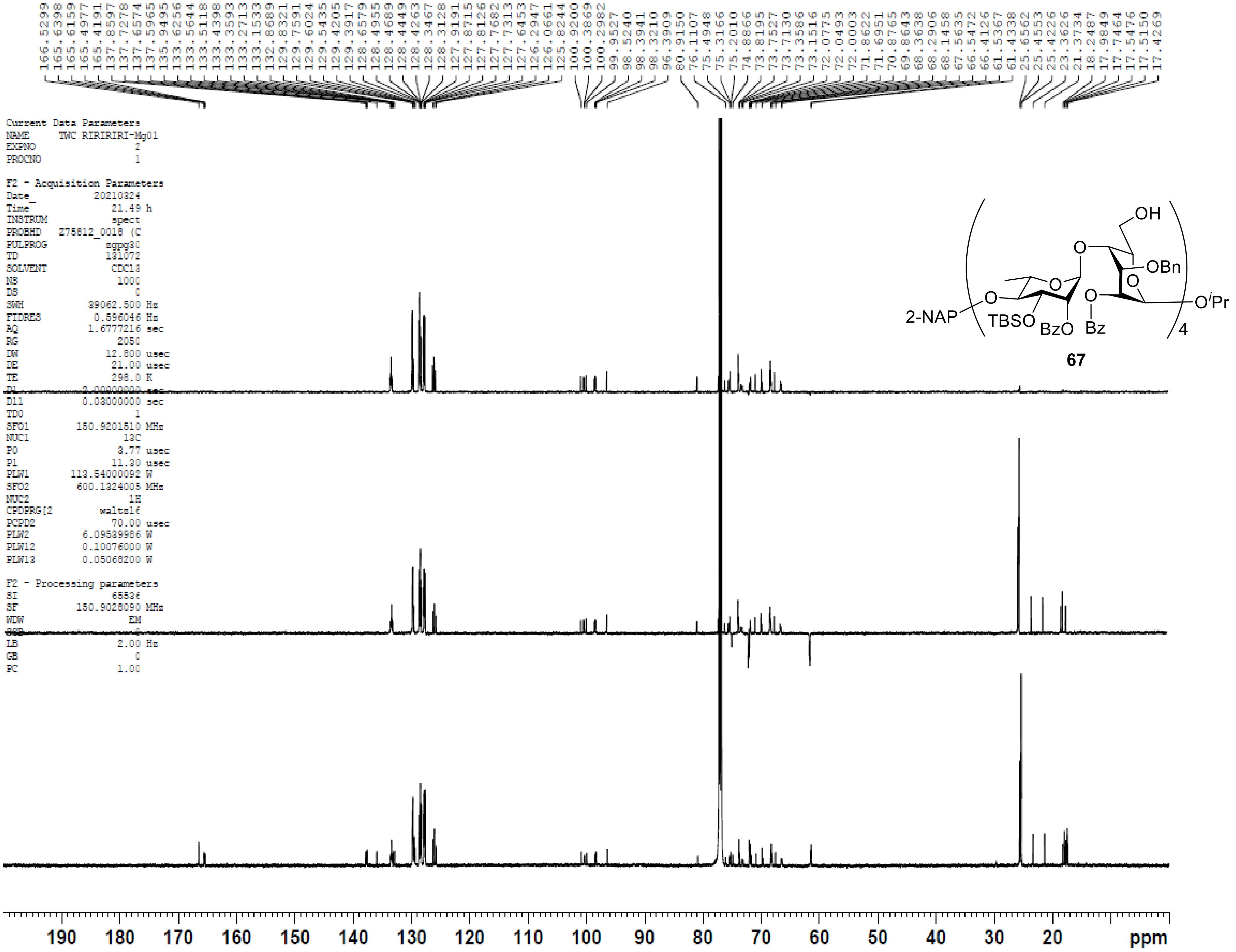

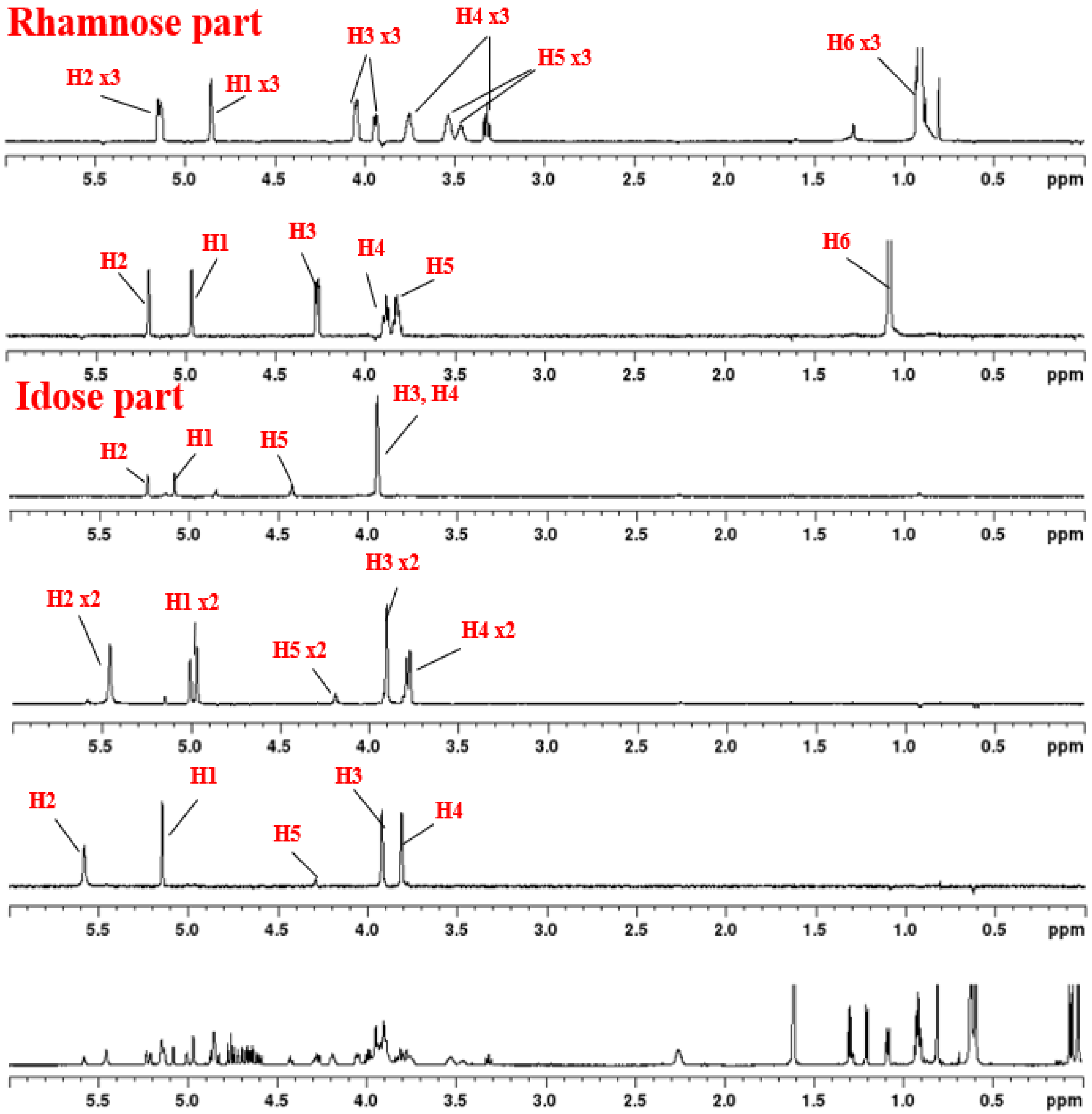
Rhamnose part
H2 x3
H1 x3
H3 x3
H4 x3
H5 x3
H6 x3
H2
H1
H3
H4
H5
H6
Idose part
H2
H1
H5
H3, H4
H2 x2
H1 x2
H5 x2
H3 x2
H4 x2
H2
H1
H5
H3
H4
ppm

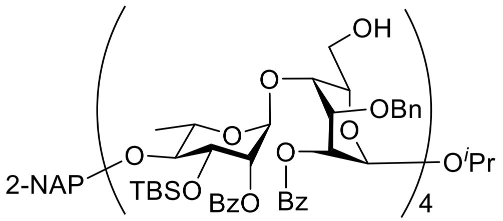
OH
O
OBn
O
O
O
O
O^{i}Pr
2-NAP
TBSO
BzO
Bz
4

67

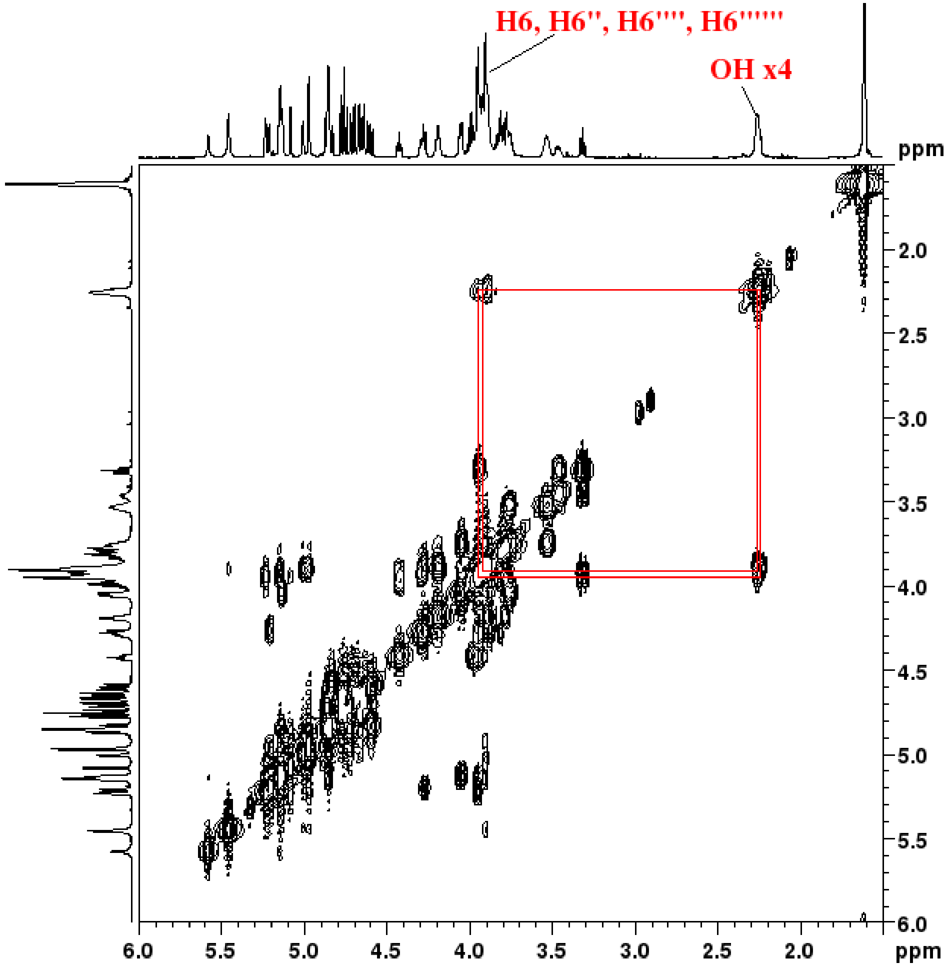
H6, H6'', H6'''', H6''''''
OH x4
ppm

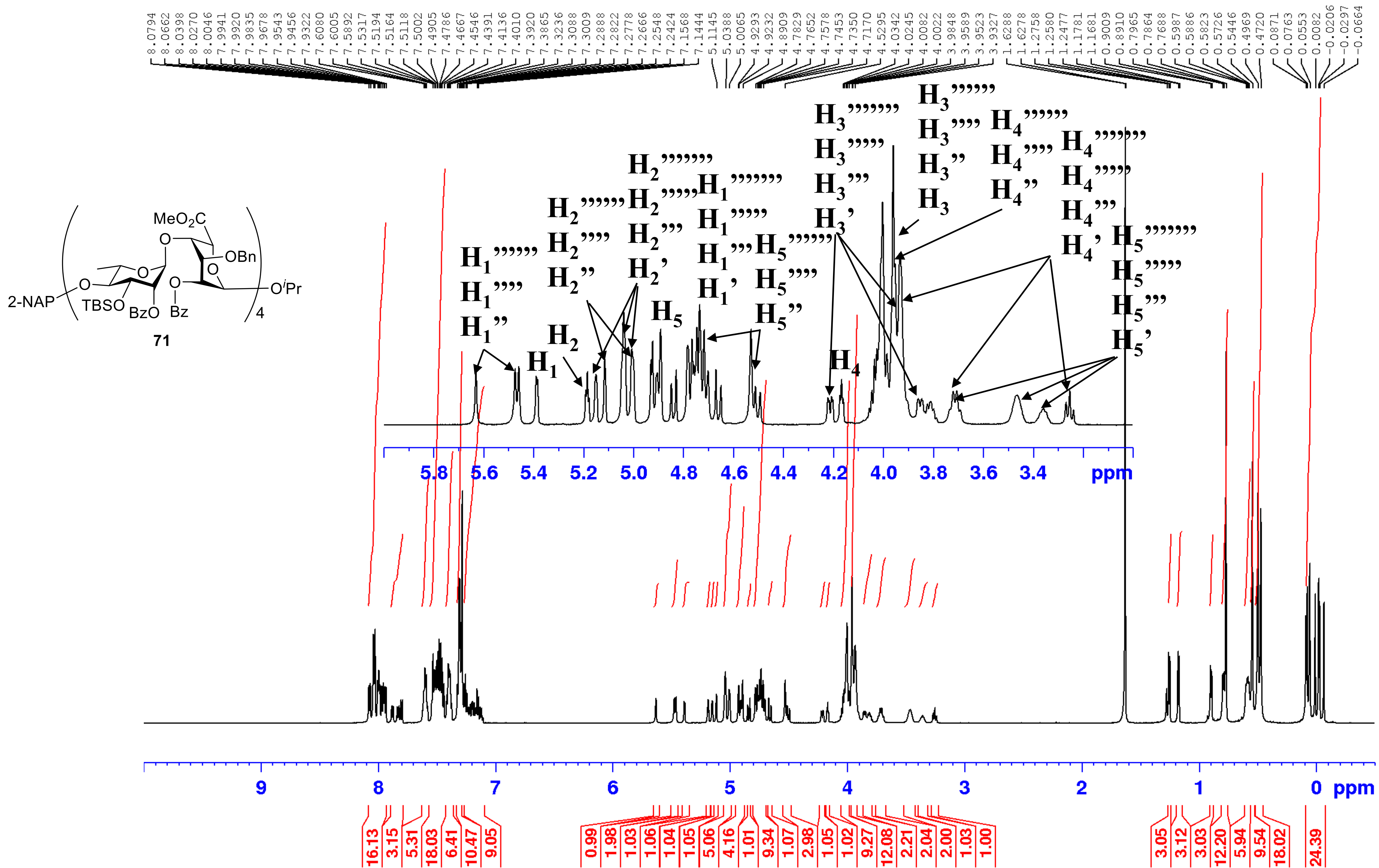
MeO2C
OBn
2-NAP
TBSO
BzO
Bz
O^{i}Pr
4
71
ppm

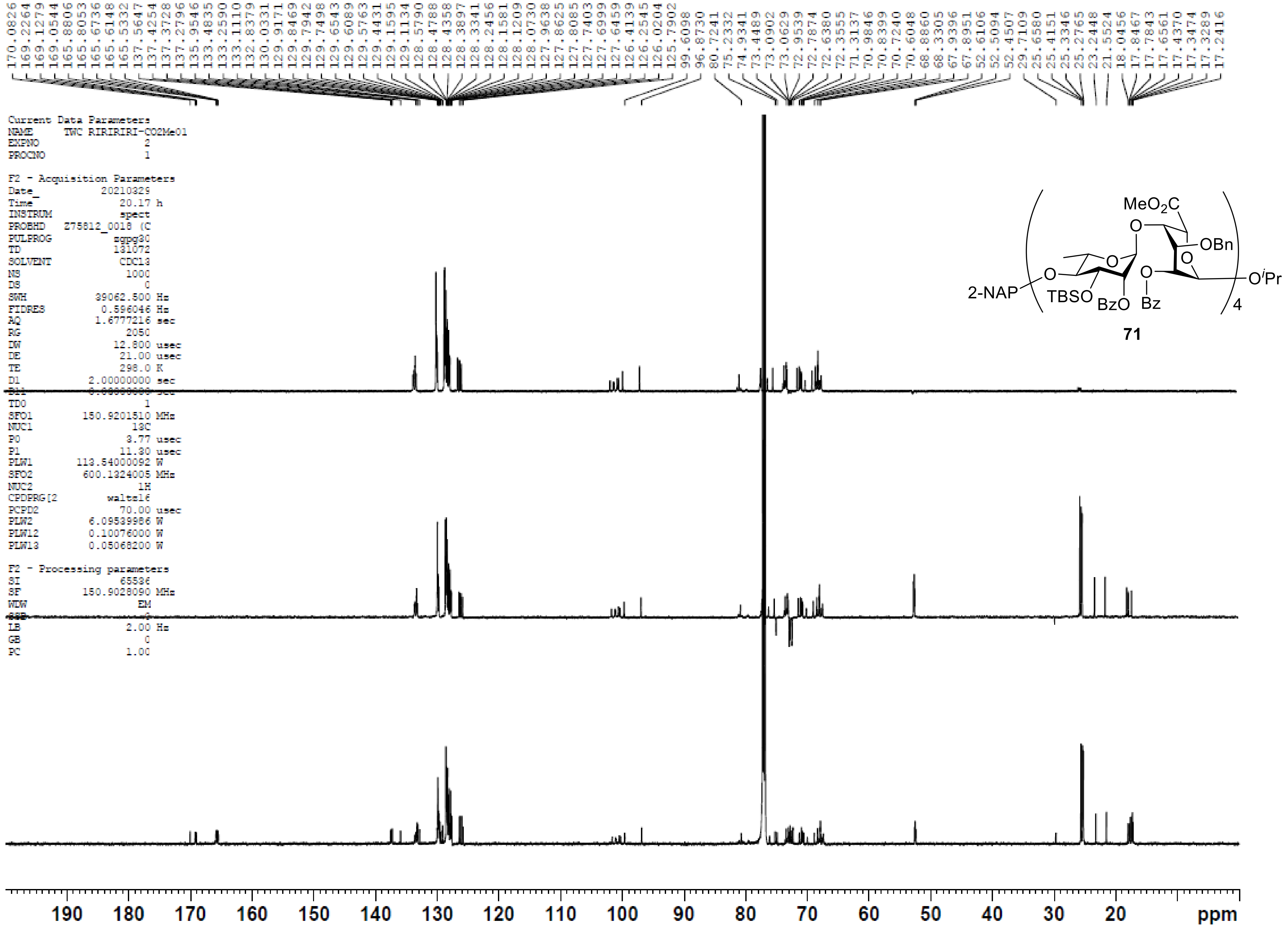
170.0826
169.2264
169.1279
169.0544
165.8806
165.8053
165.6736
165.6148
165.5332
137.5647
137.4254
137.3728
137.2796
135.9546
133.4835
133.2590
133.1110
132.8379
130.0331
129.9171
129.8469
129.7942
129.7498
129.6543
129.6089
129.5763
129.4431
129.1595
129.1134
128.5790
128.4788
128.4358
128.3897
128.3341
128.2456
128.1581
128.1209
128.0730
127.9638
127.8625
127.8085
127.7403
127.6999
127.6459
126.4139
126.2545
126.0204
125.7902
99.6098
96.8730
80.7241
75.2332
74.9341
73.4489
73.0902
73.0629
72.9539
72.7874
72.6380
72.3555
71.3137
70.9846
70.8399
70.7240
70.6048
68.8860
68.3305
67.9396
67.8951
52.6106
52.5094
52.4507
29.7109
25.6580
25.4151
25.3346
25.2765
23.2448
21.5524
18.0456
17.8467
17.7843
17.6561
17.4370
17.3474
17.3289
17.2416
Current Data Parameters
NAME TWC RIRIRIRI-CO2Me01
EXPNO 2
PROCNO 1
F2 - Acquisition Parameters
Date_ 20210329
Time 20.17 h
INSTRUM spect
PROBHD Z75812_0018 (C
PULPROG zgpg30
TD 131072
SOLVENT CDCl3
NS 1000
DS 0
SWH 39062.500 Hz
FIDRES 0.596046 Hz
AQ 1.6777216 sec
RG 2050
DW 12.800 usec
DE 21.00 usec
TE 298.0 K
D1 2.00000000 sec
TD0 1
SFO1 150.9201510 MHz
NUC1 13C
P0 3.77 usec
P1 11.30 usec
PLW1 113.54000092 W
SFO2 600.1324005 MHz
NUC2 1H
CPDPRG[2 waltz16
PCPD2 70.00 usec
PLW2 6.09539986 W
PLW12 0.10076000 W
PLW13 0.05068200 W
F2 - Processing parameters
SI 65536
SF 150.9028090 MHz
WDW EM
LB 2.00 Hz
GB 0
PC 1.00
190 180 170 160 150 140 130 120 110 100 90 80 70 60 50 40 30 20 ppm
MeO2C
O
OBn
O
O
2-NAP
O
O
OiPr
TBSO
BzO
Bz
4
71

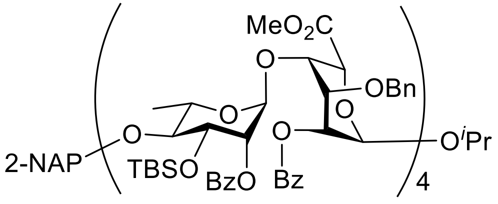
MeO2C
O
OBn
O
O
O
2-NAP
O
OiPr
TBSO
BzO
Bz
4

71

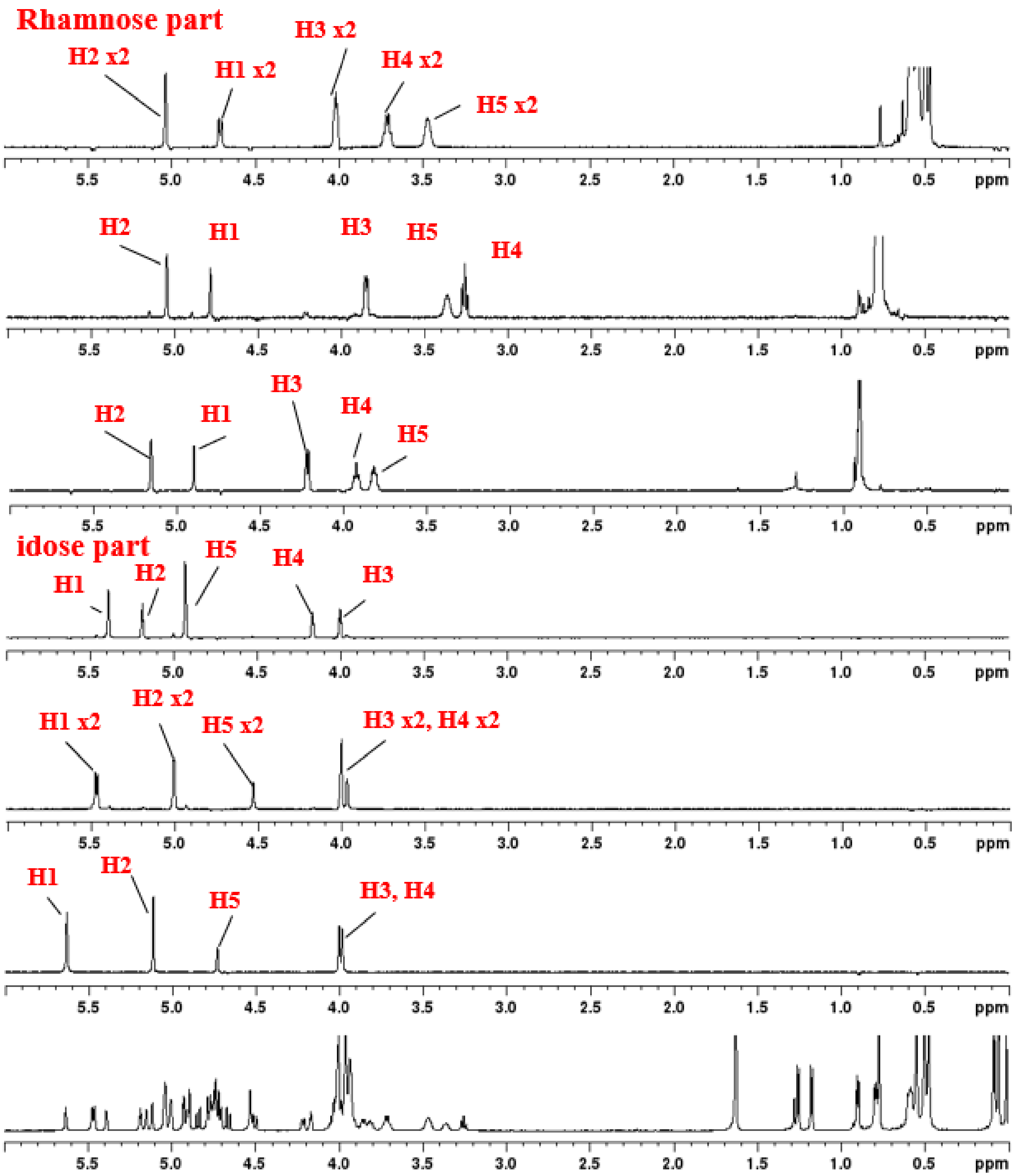
Rhamnose part
H2 x2
H1 x2
H3 x2
H4 x2
H5 x2
H2
H1
H3
H5
H4
H2
H1
H3
H4
H5
idose part
H1
H2
H5
H4
H3
H1 x2
H2 x2
H5 x2
H3 x2, H4 x2
H1
H2
H5
H3, H4
5.5 5.0 4.5 4.0 3.5 3.0 2.5 2.0 1.5 1.0 0.5 ppm

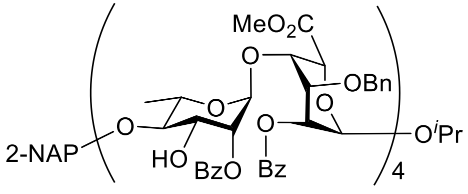
MeO2C
O
OBn
O
2-NAP
O
HO
BzO
O
Bz
OiPr
4

75

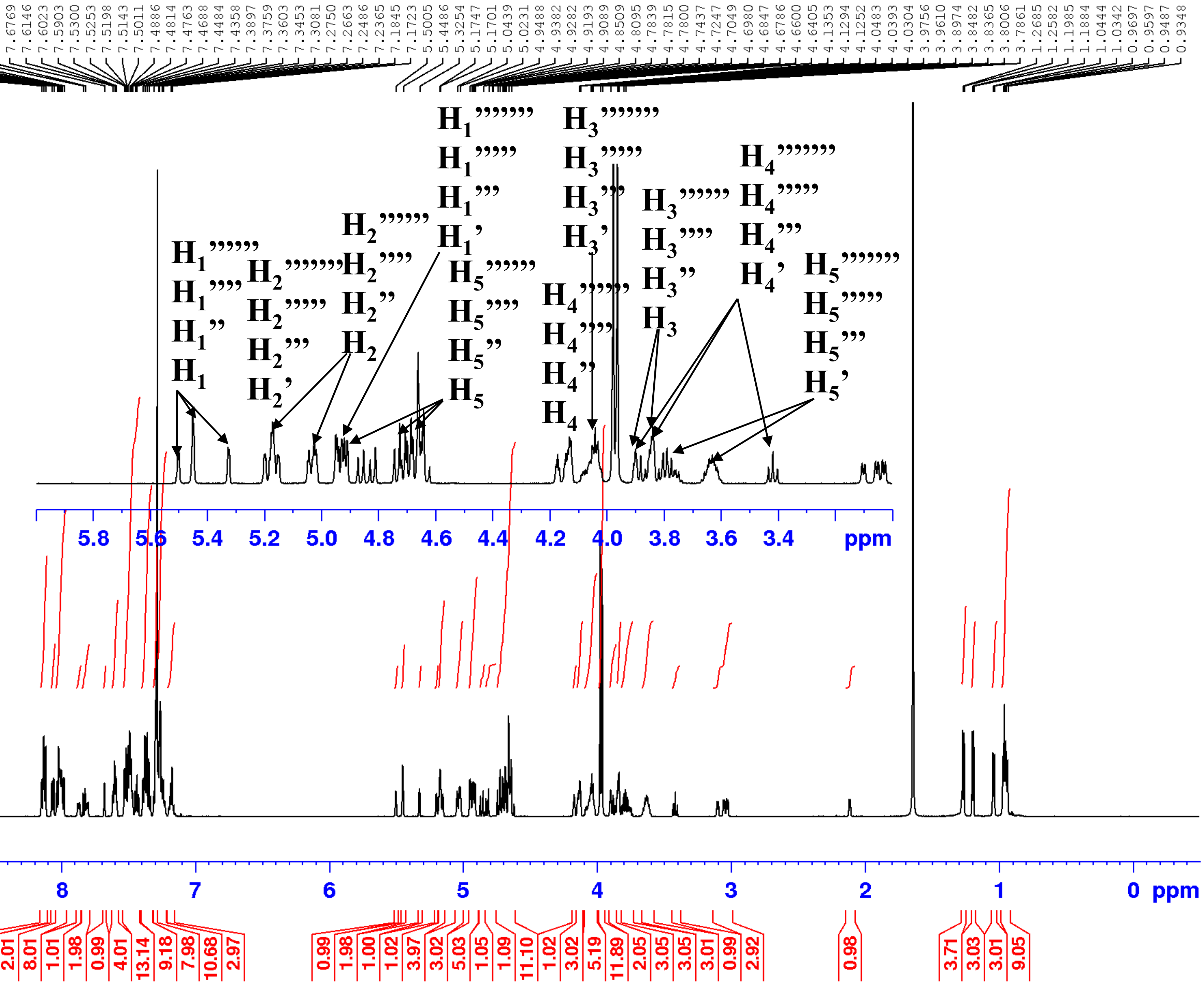
8.1467 8.1307 8.1167 8.0690 8.0558 8.0317 8.0181 8.0112 7.9975 7.9932 7.9793 7.8361 7.8219 7.6769 7.6146 7.6023 7.5903 7.5300 7.5253 7.5198 7.5143 7.5011 7.4886 7.4814 7.4763 7.4688 7.4484 7.4358 7.3897 7.3759 7.3603 7.3453 7.3081 7.2750 7.2663 7.2486 7.2365 7.1845 7.1723 5.5005 5.4486 5.3254 5.1747 5.1701 5.0439 5.0231 4.9488 4.9382 4.9282 4.9193 4.9089 4.8509 4.8095 4.7839 4.7815 4.7800 4.7437 4.7247 4.7049 4.6980 4.6847 4.6786 4.6600 4.6405 4.1353 4.1294 4.1252 4.0483 4.0393 4.0304 3.9756 3.9610 3.8974 3.8482 3.8365 3.8006 3.7861 1.2685 1.2582 1.1985 1.1884 1.0444 1.0342 0.9697 0.9597 0.9487 0.9348
H1’’’’’’, H1’’’’, H1’’, H1
H2’’’’’’’, H2’’’’’, H2’’’, H2’
H2’’’’’’, H2’’’’, H2’’, H2
H1’’’’’’’, H1’’’’’, H1’’’, H1’
H5’’’’’’, H5’’’’, H5’’, H5
H4’’’’’’, H4’’’’, H4’’, H4
H3’’’’’’’, H3’’’’’, H3’’’, H3’
H3’’’’’’, H3’’’’, H3’’, H3
H4’’’’’’’, H4’’’’’, H4’’’, H4’
H5’’’’’’’, H5’’’’’, H5’’’, H5’
5.8 5.6 5.4 5.2 5.0 4.8 4.6 4.4 4.2 4.0 3.8 3.6 3.4 ppm
9 8 7 6 5 4 3 2 1 0 ppm
5.98 2.01 8.01 1.01 1.98 0.99 4.01 13.14 9.18 7.98 10.68 2.97 0.99 1.98 1.00 1.02 3.97 3.02 5.03 1.05 1.09 11.10 1.02 3.02 5.19 11.89 2.05 3.05 3.05 3.01 0.99 2.92 0.98 3.71 3.03 3.01 9.05

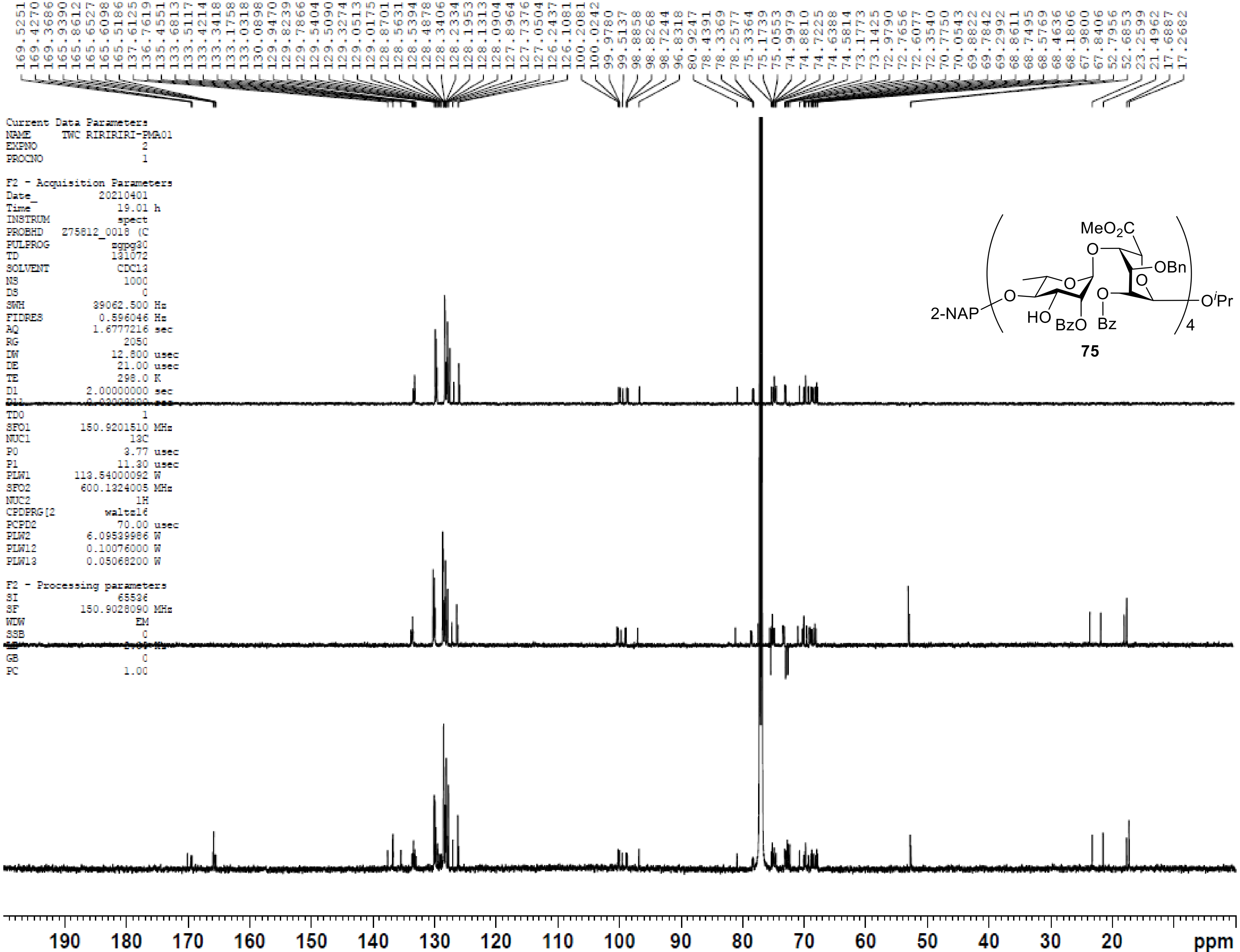
169.5251
169.4270
169.3686
165.9390
165.8612
165.6527
165.6098
165.5186
137.6125
136.7619
135.4551
133.6813
133.5117
133.4214
133.3418
133.1758
133.0318
130.0898
129.9470
129.8239
129.7866
129.5404
129.5090
129.3274
129.0513
129.0175
128.8701
128.5631
128.5394
128.4878
128.3406
128.2334
128.1953
128.1313
128.0904
127.8964
127.7376
127.0504
126.2437
126.1081
100.2081
100.0242
99.9780
99.5137
98.8858
98.8268
98.7244
96.8318
80.9247
78.4391
78.3369
78.2577
75.3364
75.1739
75.0553
74.9979
74.8810
74.7225
74.6388
74.5814
73.1773
73.1425
72.9790
72.7656
72.6077
72.3540
70.7750
70.0543
69.8822
69.7842
69.2992
68.8611
68.7495
68.5769
68.4636
68.1806
67.9800
67.8406
52.7956
52.6853
23.2599
21.4962
17.6887
17.2682
Current Data Parameters
NAME TWC RIRIRIRI-PMA01
EXPNO 2
PROCNO 1
F2 - Acquisition Parameters
Date_ 20210401
Time 19.01 h
INSTRUM spect
PROBHD Z75812_0018 (C
PULPROG zgpg30
TD 131072
SOLVENT CDCl3
NS 1000
DS 0
SWH 39062.500 Hz
FIDRES 0.596046 Hz
AQ 1.6777216 sec
RG 2050
DW 12.800 usec
DE 21.00 usec
TE 298.0 K
D1 2.00000000 sec
TD0 1
SFO1 150.9201510 MHz
NUC1 13C
P0 3.77 usec
P1 11.30 usec
PLW1 113.54000092 W
SFO2 600.1324005 MHz
NUC2 1H
CPDPRG[2 waltz16
PCPD2 70.00 usec
PLW2 6.09539986 W
PLW12 0.10076000 W
PLW13 0.05068200 W
F2 - Processing parameters
SI 65536
SF 150.9028090 MHz
WDW EM
SSB 0
GB 0
PC 1.00
MeO2C
O
OBn
O
O
O
2-NAP
HO
BzO
O
Bz
OiPr
4
75
190
180
170
160
150
140
130
120
110
100
90
80
70
60
50
40
30
20
ppm

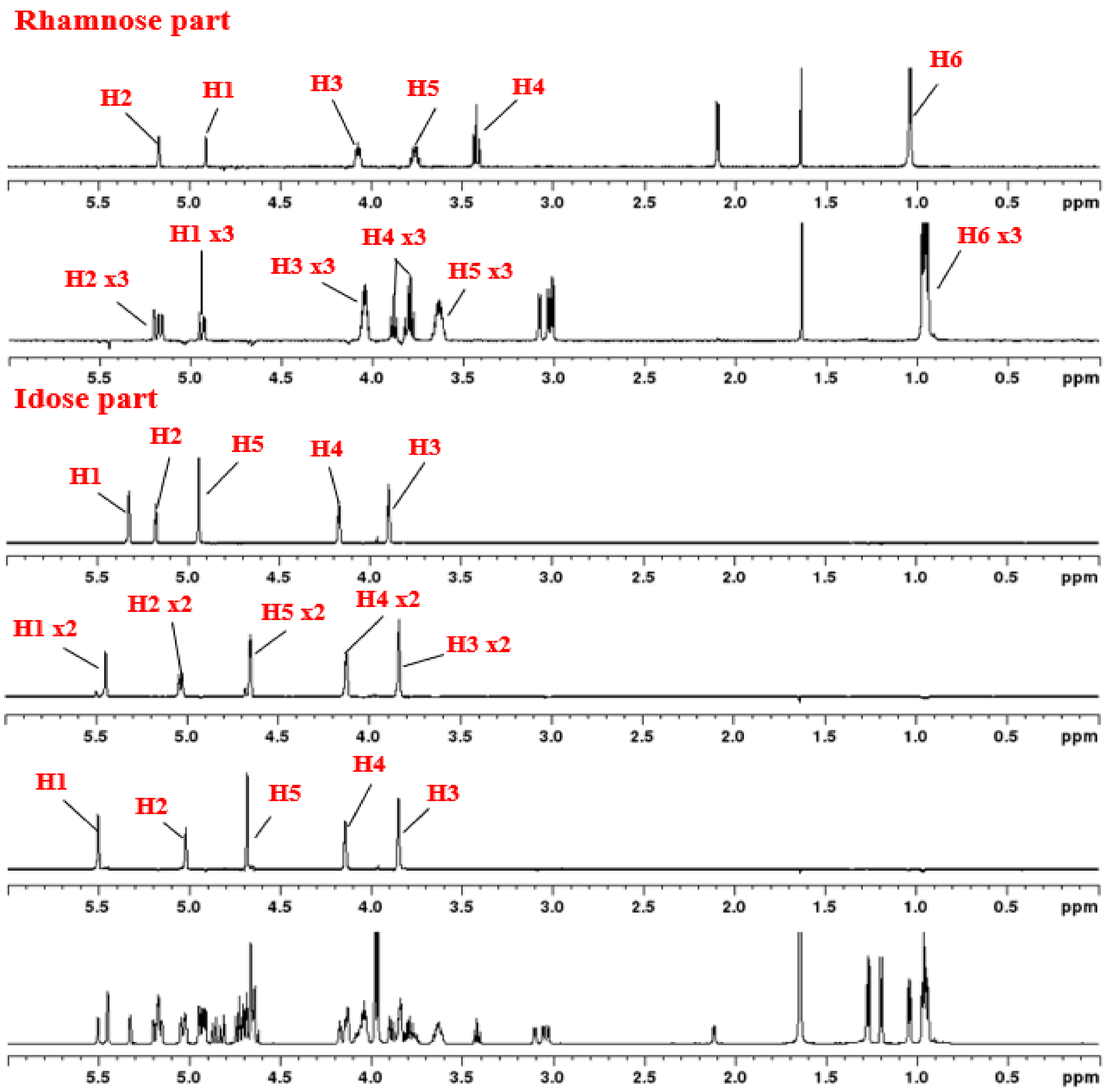
Rhamnose part
H2
H1
H3
H5
H4
H6
H2 x3
H1 x3
H3 x3
H4 x3
H5 x3
H6 x3
Idose part
H1
H2
H5
H4
H3
H1 x2
H2 x2
H5 x2
H4 x2
H3 x2
H1
H2
H5
H4
H3
ppm

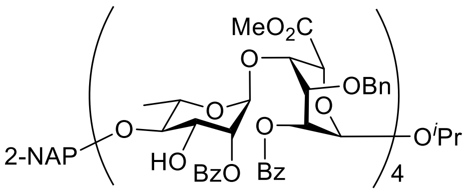
MeO2C
OBn
O
2-NAP
HO
BzO
Bz
O^iPr
4

75

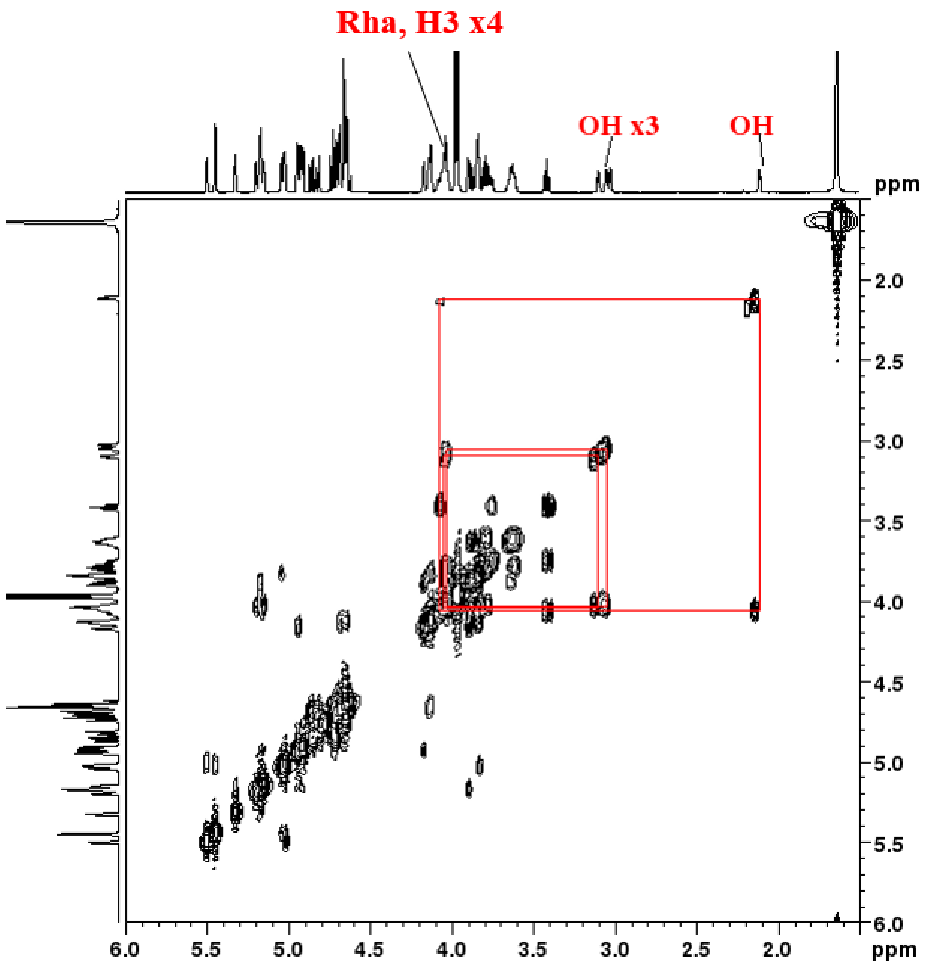
Rha, H3 x4
OH x3
OH
ppm

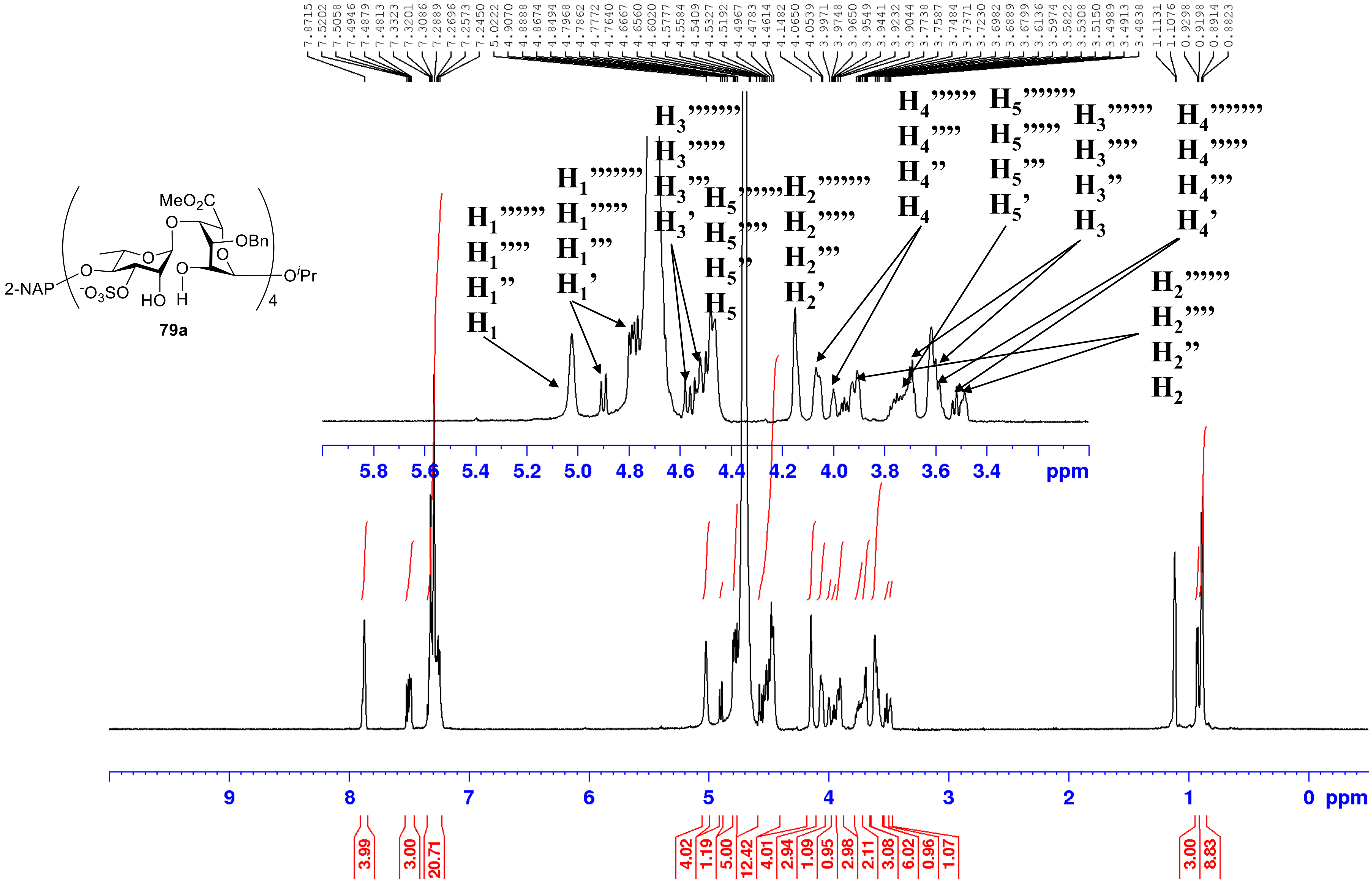
7.8715 7.5202 7.5058 7.4946 7.4879 7.4813 7.3323 7.3201 7.3086 7.2889 7.2696 7.2573 7.2450
5.0222 4.9070 4.8888 4.8674 4.8494 4.7968 4.7862 4.7772 4.7640 4.6667 4.6560 4.6020 4.5777 4.5584 4.5409 4.5327 4.5192 4.4967 4.4783 4.4614
4.1482 4.0650 4.0539 3.9971 3.9748 3.9650 3.9549 3.9441 3.9232 3.9044 3.7738 3.7587 3.7484 3.7371 3.7230 3.6982 3.6889 3.6799 3.6136 3.5974 3.5822 3.5308 3.5150 3.4989 3.4913 3.4838
1.1131 1.1076 0.9298 0.9198 0.8914 0.8823
MeO2C
OBn
O
O
O
2-NAP
-O3SO
HO
H
OiPr
4
5.8 5.6 5.4 5.2 5.0 4.8 4.6 4.4 4.2 4.0 3.8 3.6 3.4 ppm
9 8 7 6 5 4 3 2 1 0 ppm
3.99 3.00 20.71 4.02 1.19 5.00 12.42 4.01 2.94 1.09 0.95 2.98 2.11 3.08 6.02 0.96 1.07 3.00 8.83

79a

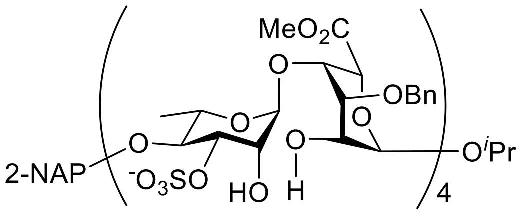
MeO2C
OBn
2-NAP
-O3SO
HO
H
O'Pr
4

79a

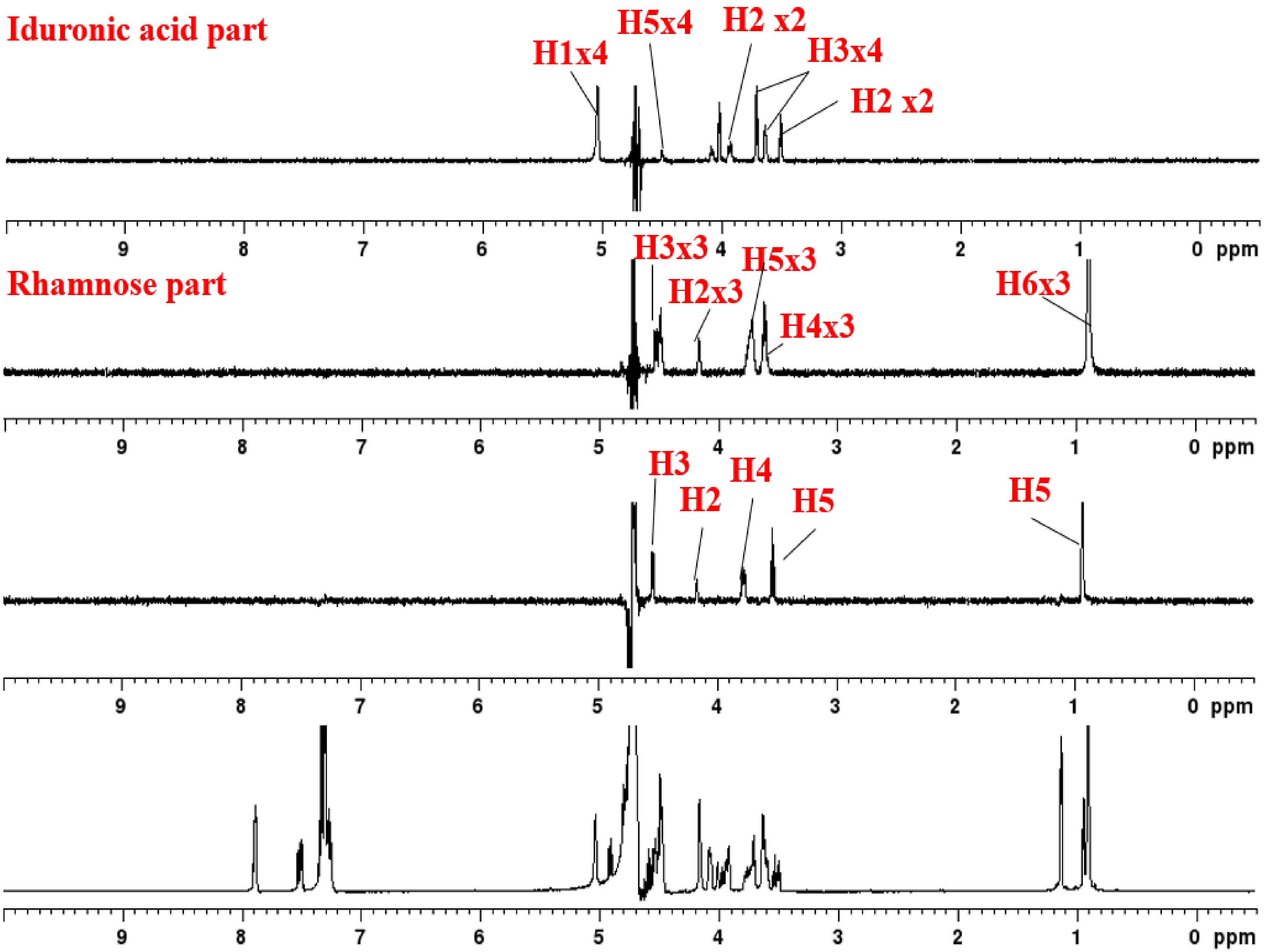
Iduronic acid part
H1x4
H5x4
H2 x2
H3x4
H2 x2
Rhamnose part
H3x3
H5x3
H2x3
H4x3
H6x3
H3
H2
H4
H5
H5
9 8 7 6 5 4 3 2 1 0 ppm

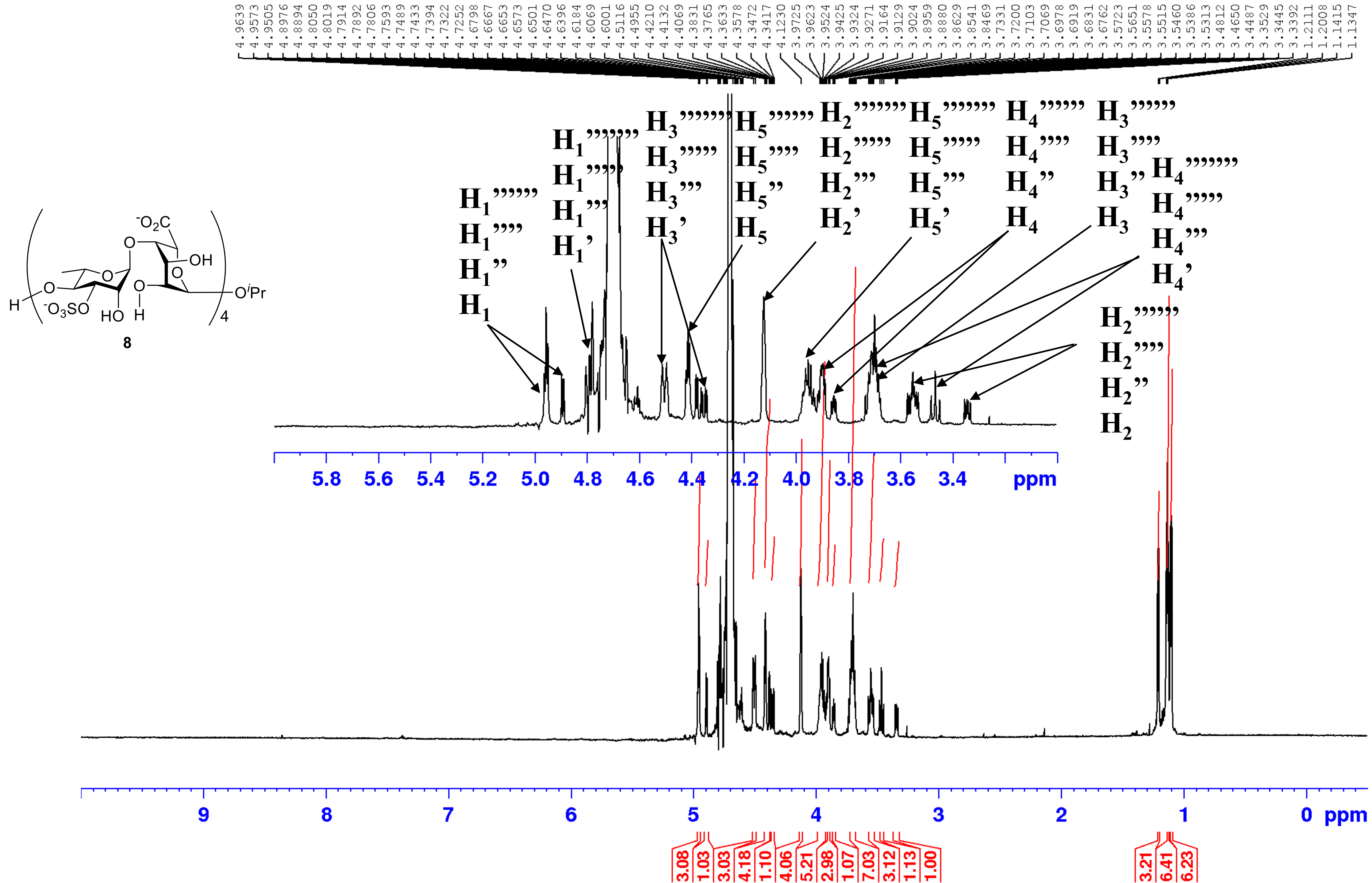
8
ppm

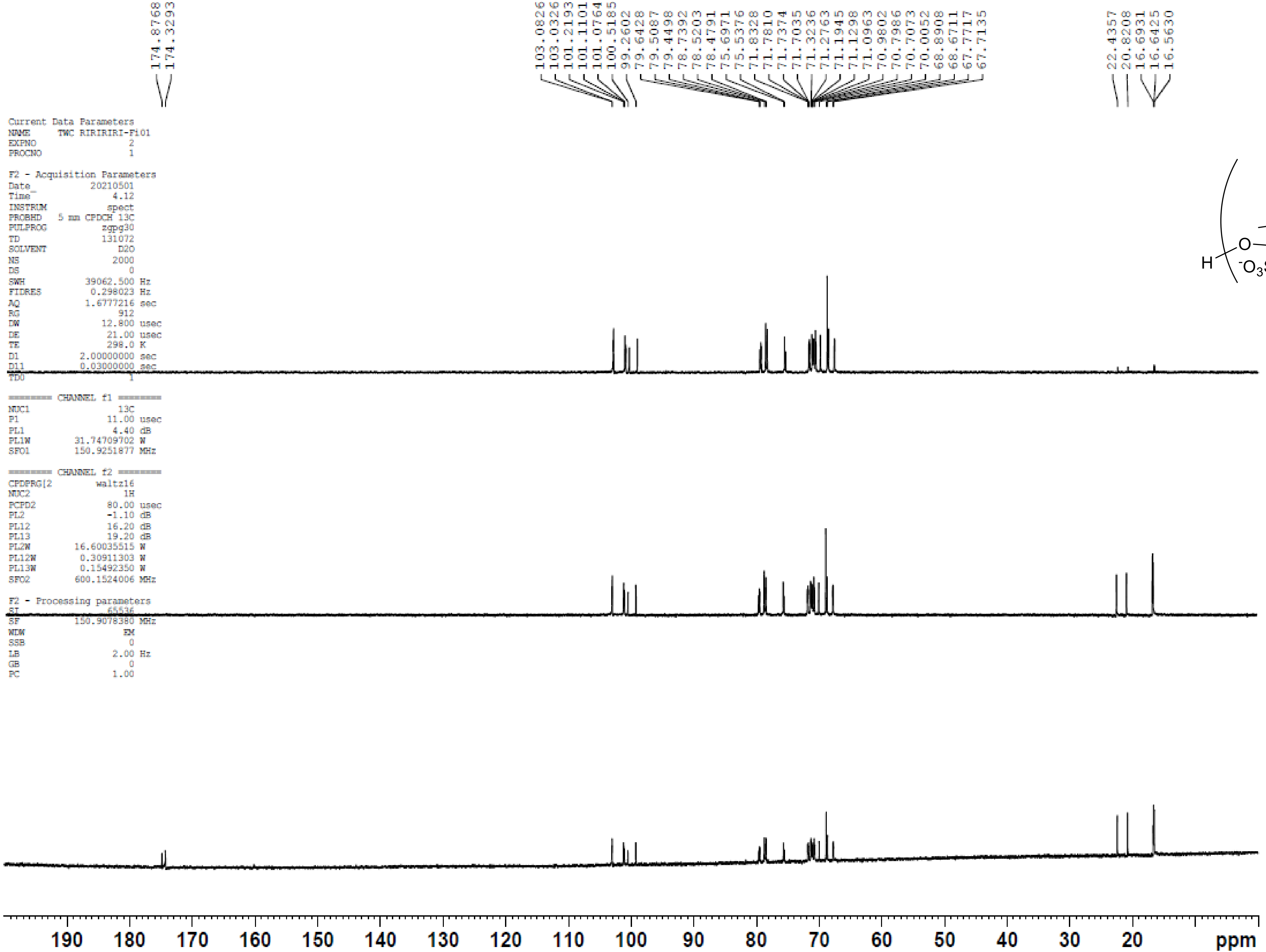
174.8768
174.3293
103.0826
103.0326
101.2193
101.1101
101.0764
100.5185
99.2602
79.6428
79.5087
79.4498
78.7392
78.5203
78.4791
75.6971
75.5376
71.8328
71.7810
71.7374
71.7035
71.3236
71.2763
71.1945
71.1298
71.0963
70.9802
70.7986
70.7073
70.0052
68.8908
68.6711
67.7717
67.7135
22.4357
20.8208
16.6931
16.6425
16.5630
Current Data Parameters
NAME TWC RIRIRIRI-Fi01
EXPNO 2
PROCNO 1
F2 - Acquisition Parameters
Date_ 20210501
Time 4.12
INSTRUM spect
PROBHD 5 mm CPDCH 13C
PULPROG zgpg30
TD 131072
SOLVENT D2O
NS 2000
DS 0
SWH 39062.500 Hz
FIDRES 0.298023 Hz
AQ 1.6777216 sec
RG 912
DW 12.800 usec
DE 21.00 usec
TE 298.0 K
D1 2.00000000 sec
D11 0.03000000 sec
TD0 1
======== CHANNEL f1 ========
NUC1 13C
P1 11.00 usec
PL1 4.40 dB
PL1W 31.74709702 W
SFO1 150.9251877 MHz
======== CHANNEL f2 ========
CPDPRG[2 waltz16
NUC2 1H
PCPD2 80.00 usec
PL2 -1.10 dB
PL12 16.20 dB
PL13 19.20 dB
PL2W 16.60035515 W
PL12W 0.30911303 W
PL13W 0.15492350 W
SFO2 600.1524006 MHz
F2 - Processing parameters
SI 65536
SF 150.9078380 MHz
WDW EM
SSB 0
LB 2.00 Hz
GB 0
PC 1.00
190 180 170 160 150 140 130 120 110 100 90 80 70 60 50 40 30 20 ppm

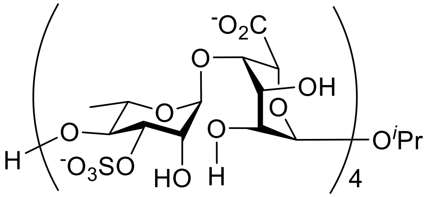
-O2C
OH
O
O
H
O
-O3SO
HO
H
O
OiPr
4

8

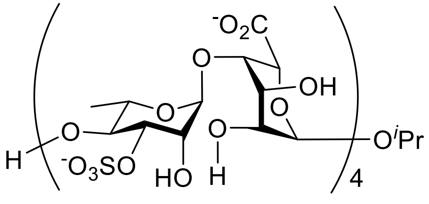

^-O_2C
OH
O
H
^-O_3SO
HO
H
O^iPr
4

8

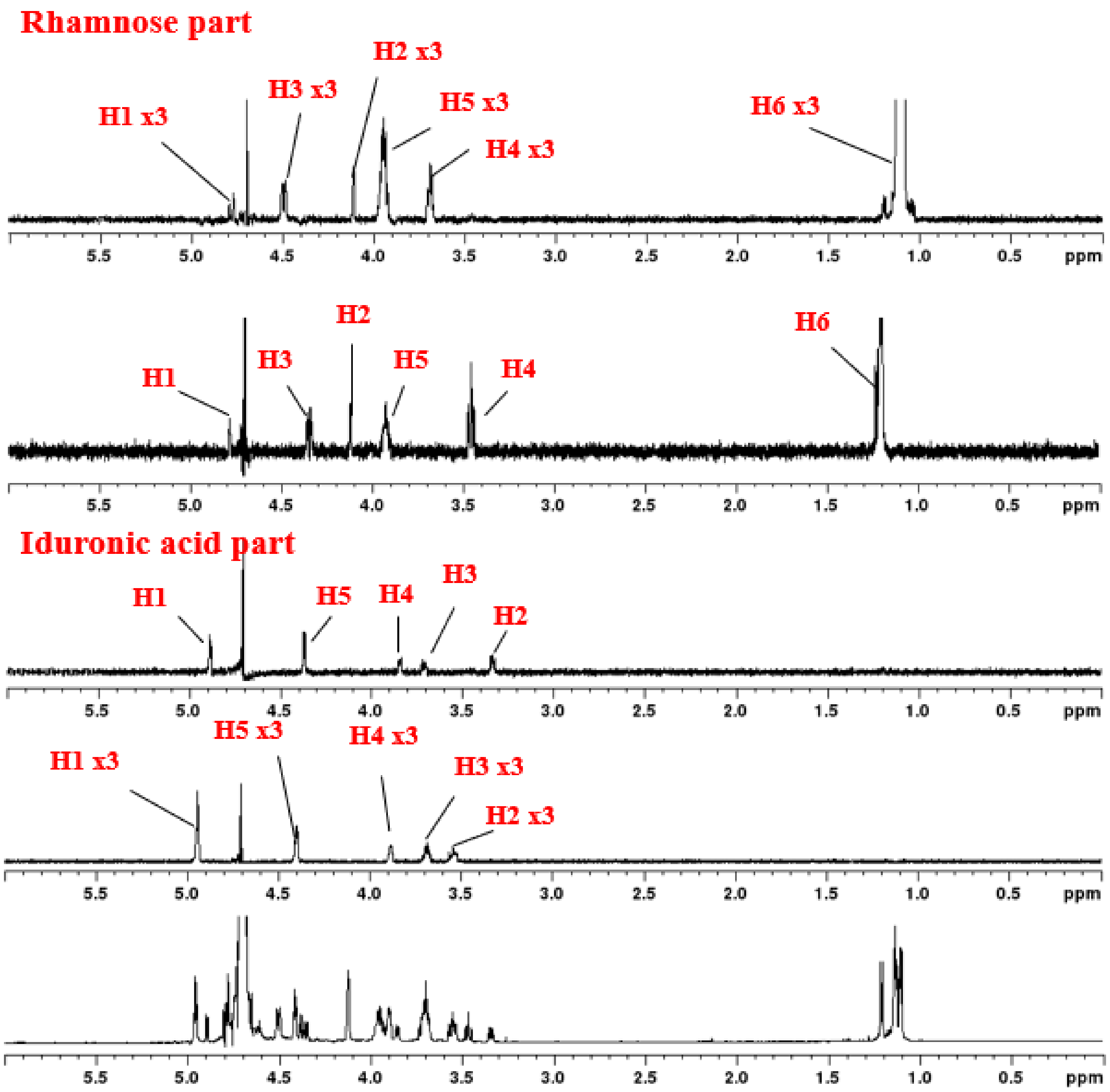

Rhamnose part
H1 x3
H3 x3
H2 x3
H5 x3
H4 x3
H6 x3
H1
H3
H2
H5
H4
H6
Iduronic acid part
H1
H5
H4
H3
H2
H1 x3
H5 x3
H4 x3
H3 x3
H2 x3
5.5
5.0
4.5
4.0
3.5
3.0
2.5
2.0
1.5
1.0
0.5
ppm

9. NMR Spectra for the Synthesis of $U_{3'S}$ Libraries

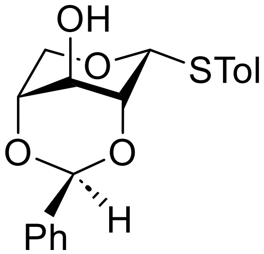
OH
O
STol
O
O
Ph
H

82

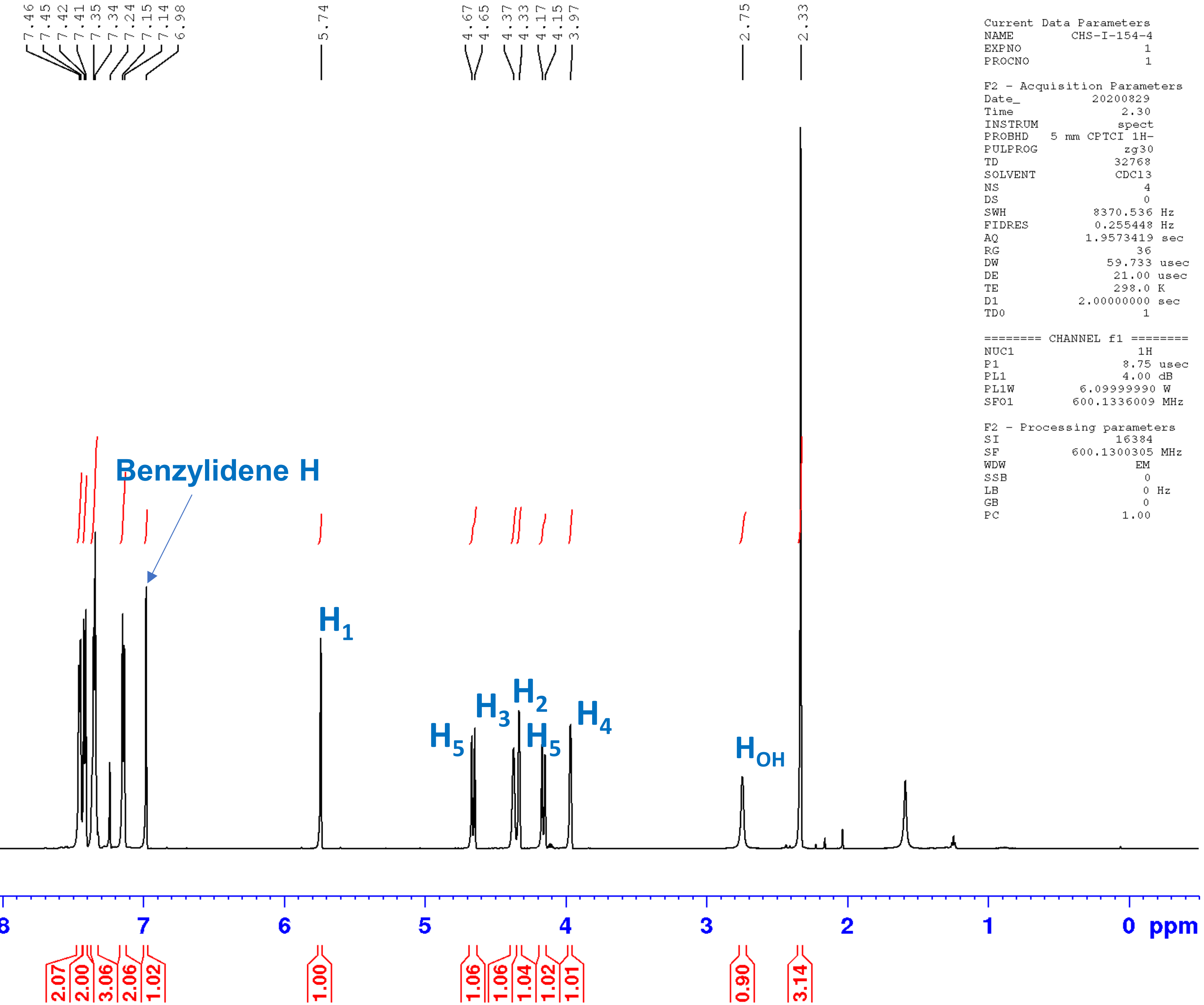
7.46
7.45
7.42
7.41
7.35
7.34
7.24
7.15
7.14
6.98
5.74
4.67
4.65
4.37
4.33
4.17
4.15
3.97
2.75
2.33
Benzylidene H
H_1
H_5
H_3
H_2
H_5
H_4
H_{OH}
9
8
7
6
5
4
3
2
1
0 ppm
2.07
2.00
3.06
2.06
1.02
1.00
1.06
1.06
1.04
1.02
1.01
0.90
3.14
Current Data Parameters
NAME CHS-I-154-4
EXPNO 1
PROCNO 1
F2 - Acquisition Parameters
Date_ 20200829
Time 2.30
INSTRUM spect
PROBHD 5 mm CPTCI 1H-
PULPROG zg30
TD 32768
SOLVENT CDCl3
NS 4
DS 0
SWH 8370.536 Hz
FIDRES 0.255448 Hz
AQ 1.9573419 sec
RG 36
DW 59.733 usec
DE 21.00 usec
TE 298.0 K
D1 2.00000000 sec
TD0 1
======== CHANNEL f1 ========
NUC1 1H
P1 8.75 usec
PL1 4.00 dB
PL1W 6.09999990 W
SFO1 600.1336009 MHz
F2 - Processing parameters
SI 16384
SF 600.1300305 MHz
WDW EM
SSB 0
LB 0 Hz
GB 0
PC 1.00

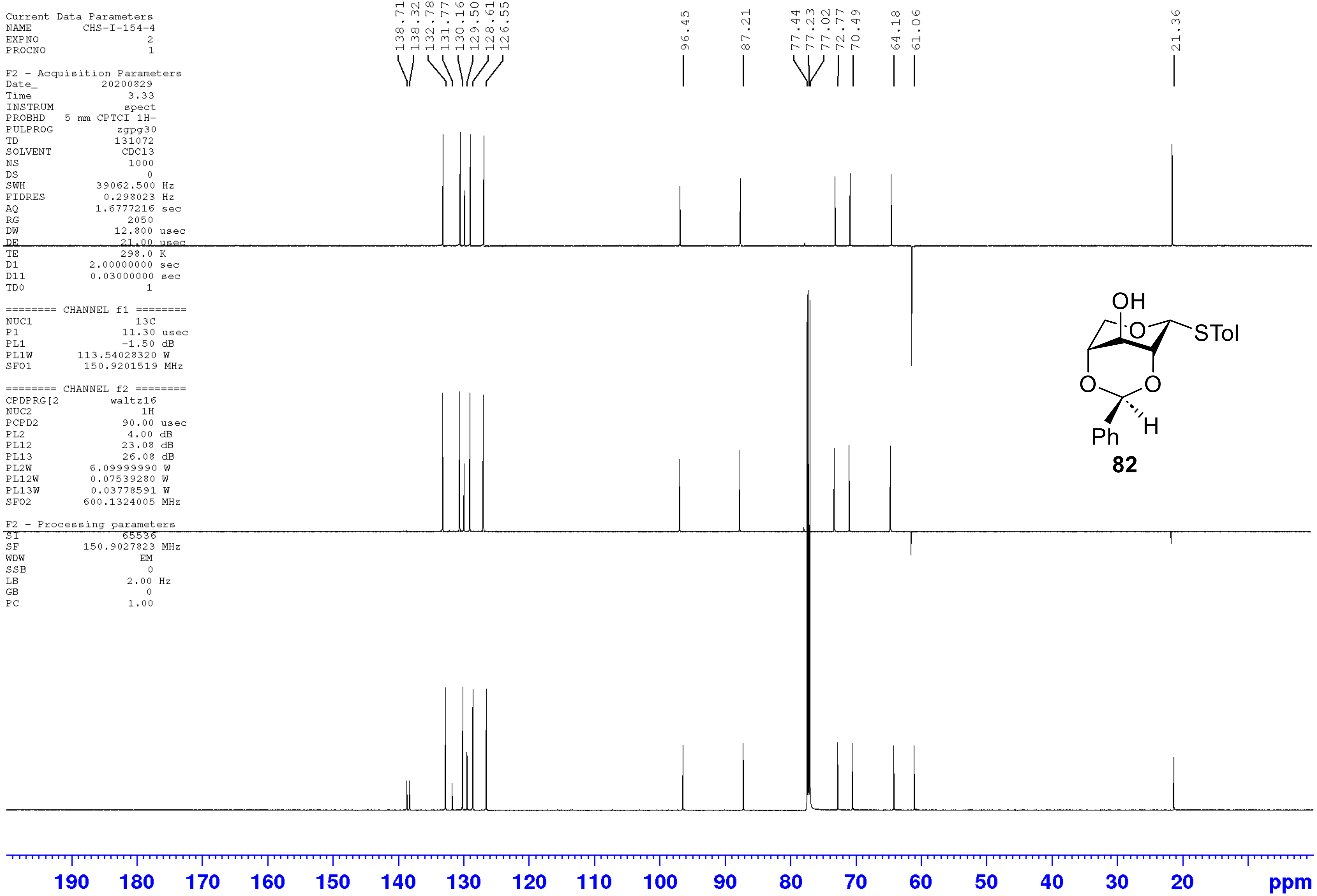
Current Data Parameters
NAME CHS-I-154-4
EXPNO 2
PROCNO 1
F2 - Acquisition Parameters
Date_ 20200829
Time 3.33
INSTRUM spect
PROBHD 5 mm CPTCI 1H-
PULPROG zgpg30
TD 131072
SOLVENT CDCl3
NS 1000
DS 0
SWH 39062.500 Hz
FIDRES 0.298023 Hz
AQ 1.6777216 sec
RG 2050
DW 12.800 usec
DE 21.00 usec
TE 298.0 K
D1 2.00000000 sec
D11 0.03000000 sec
TD0 1
======== CHANNEL f1 ========
NUC1 13C
P1 11.30 usec
PL1 -1.50 dB
PL1W 113.54028320 W
SFO1 150.9201519 MHz
======== CHANNEL f2 ========
CPDPRG[2 waltz16
NUC2 1H
PCPD2 90.00 usec
PL2 4.00 dB
PL12 23.08 dB
PL13 26.08 dB
PL2W 6.09999990 W
PL12W 0.07539280 W
PL13W 0.03778591 W
SFO2 600.1324005 MHz
F2 - Processing parameters
SI 65536
SF 150.9027823 MHz
WDW EM
SSB 0
LB 2.00 Hz
GB 0
PC 1.00
138.71
138.32
132.78
131.77
130.16
129.50
128.61
126.55
96.45
87.21
77.44
77.23
77.02
72.77
70.49
64.18
61.06
21.36
OH
O
STol
O
O
Ph
H
82
190 180 170 160 150 140 130 120 110 100 90 80 70 60 50 40 30 20 ppm

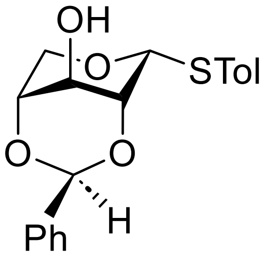
OH
STol
O
O
O
Ph
H

82

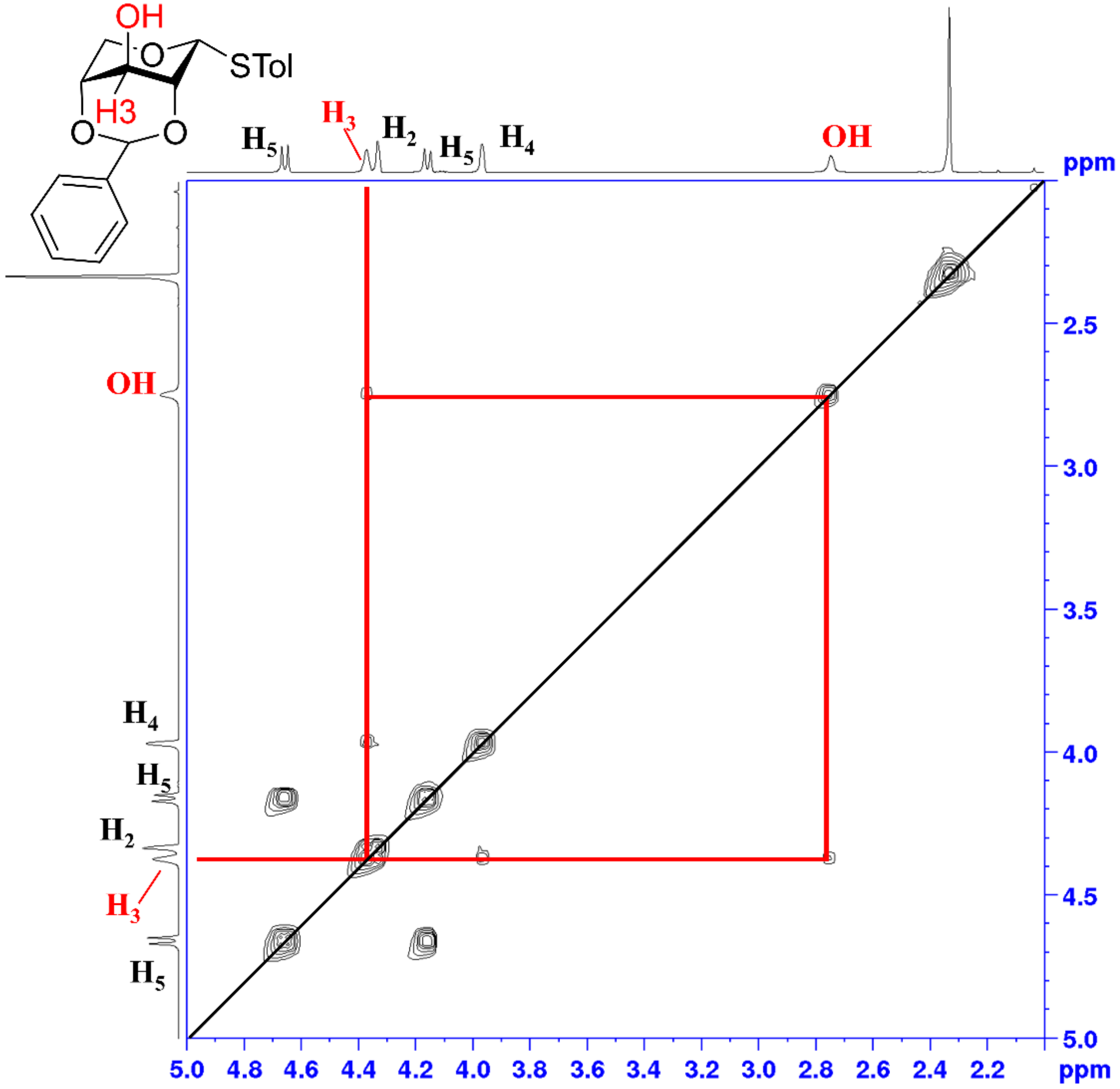
OH
STol
H3
H5
H3
H2
H5
H4
OH
ppm
H4
H5
H2
H3
H5
5.0 4.8 4.6 4.4 4.2 4.0 3.8 3.6 3.4 3.2 3.0 2.8 2.6 2.4 2.2
2.5 3.0 3.5 4.0 4.5 5.0

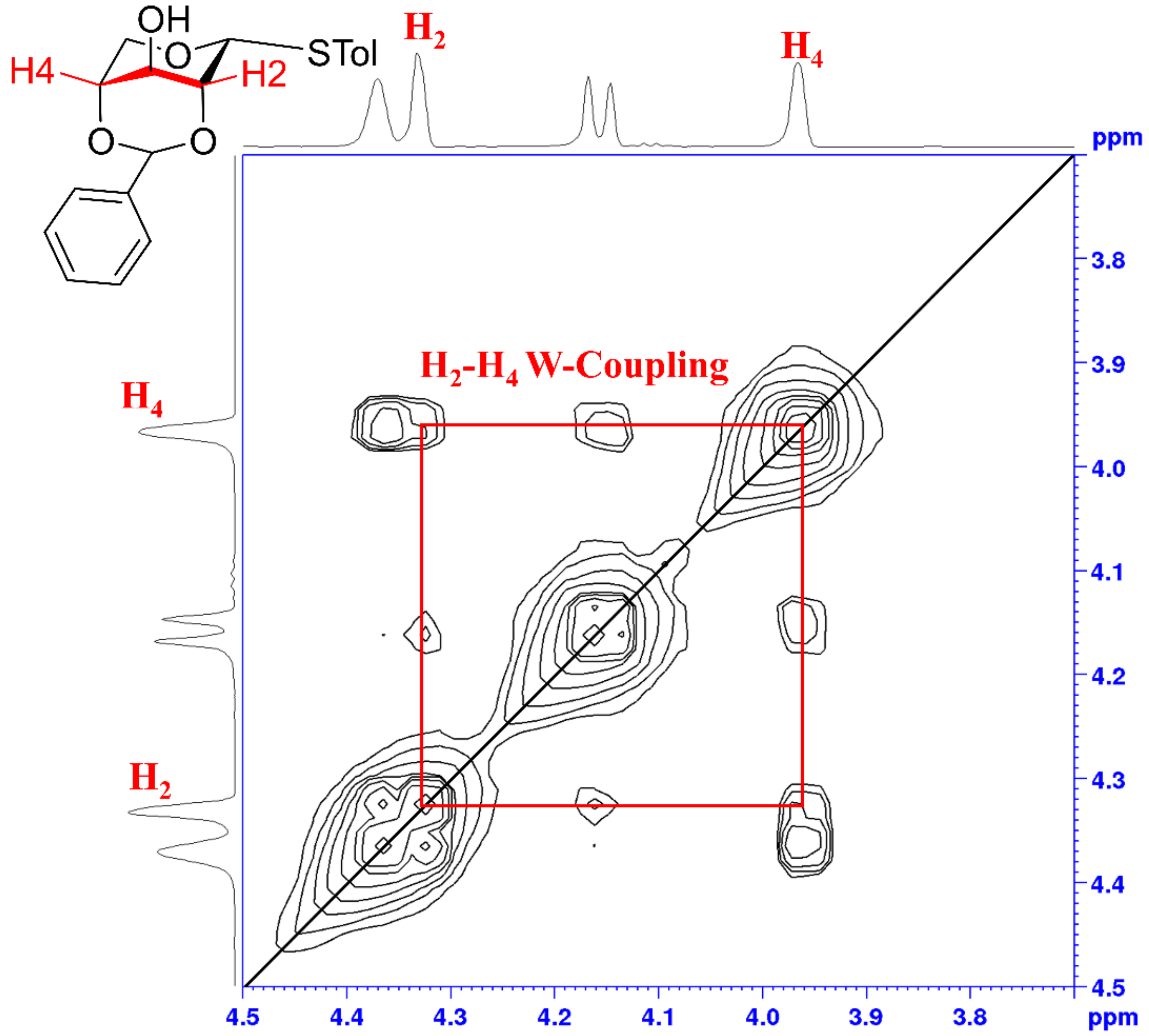
OH
STol
H4
H2
H2
H4
ppm
H2-H4 W-Coupling
H4
H2
4.5 4.4 4.3 4.2 4.1 4.0 3.9 3.8
3.8 3.9 4.0 4.1 4.2 4.3 4.4 4.5

```
Current Data Parameters
NAME            CHS-n-ph
EXPNO                  1
PROCNO                 1

F2 - Acquisition Parameters
Date_           20210427
Time               18.41
INSTRUM            spect
PROBHD   5 mm CPDCH 13C
PULPROG             zg30
TD                 32768
SOLVENT            CDCl3
NS                     8
DS                     0
SWH             8389.262 Hz
FIDRES          0.256020 Hz
AQ             1.9529728 sec
RG                     9
DW                59.600 usec
DE                 21.00 usec
TE                 298.0 K
D1            2.00000000 sec
TD0                    1

======== CHANNEL f1 ========
NUC1                  1H
P1                 10.20 usec
PL1                -0.90 dB
PL1W         15.85321522 W
SFO1         600.1536010 MHz

F2 - Processing parameters
SI                 16384
SF           600.1500000 MHz
WDW                   EM
SSB                    0
LB                     0 Hz
GB                     0
PC                  1.00
```

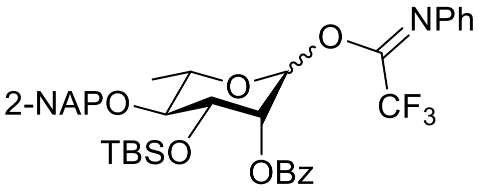

87

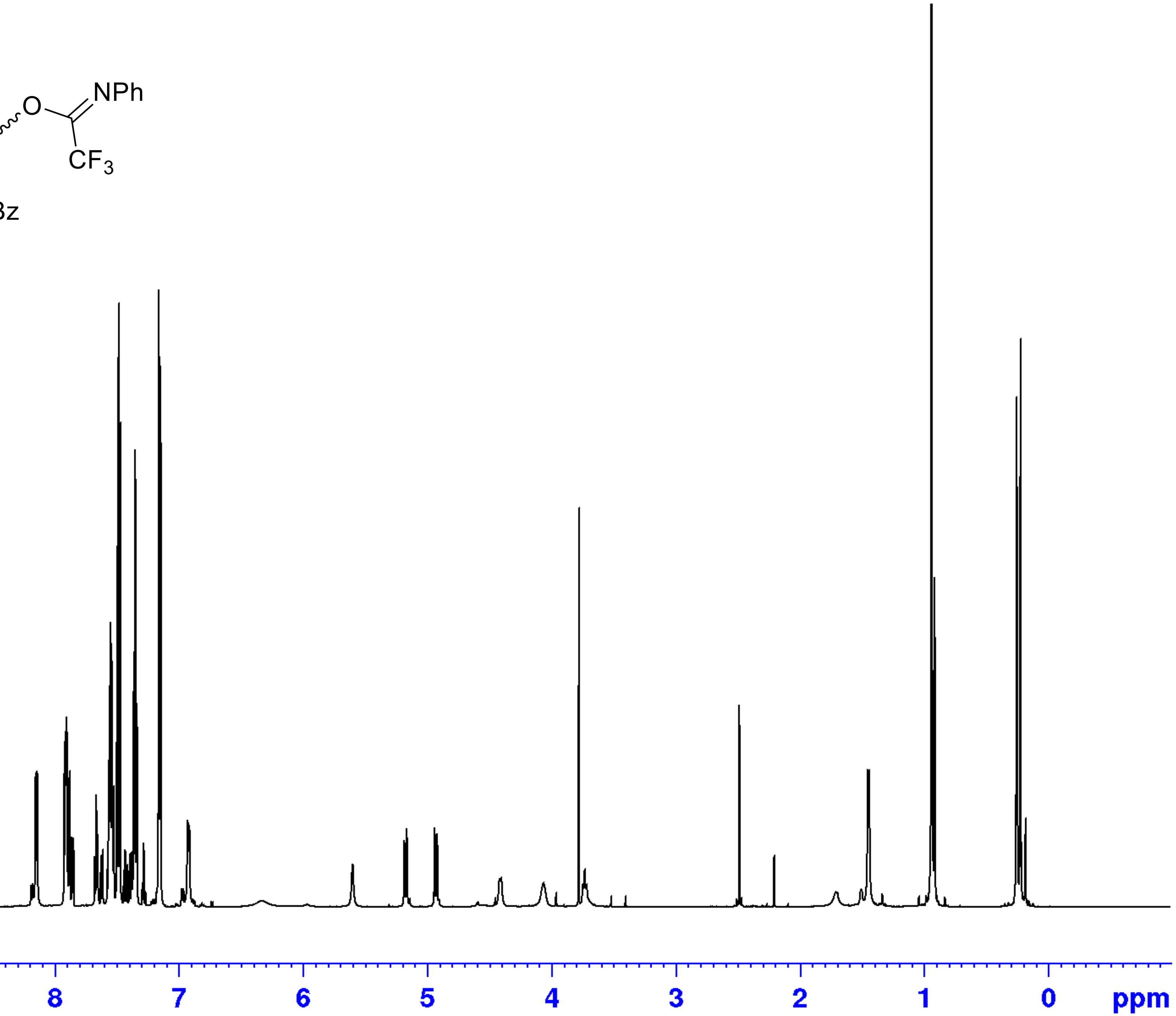

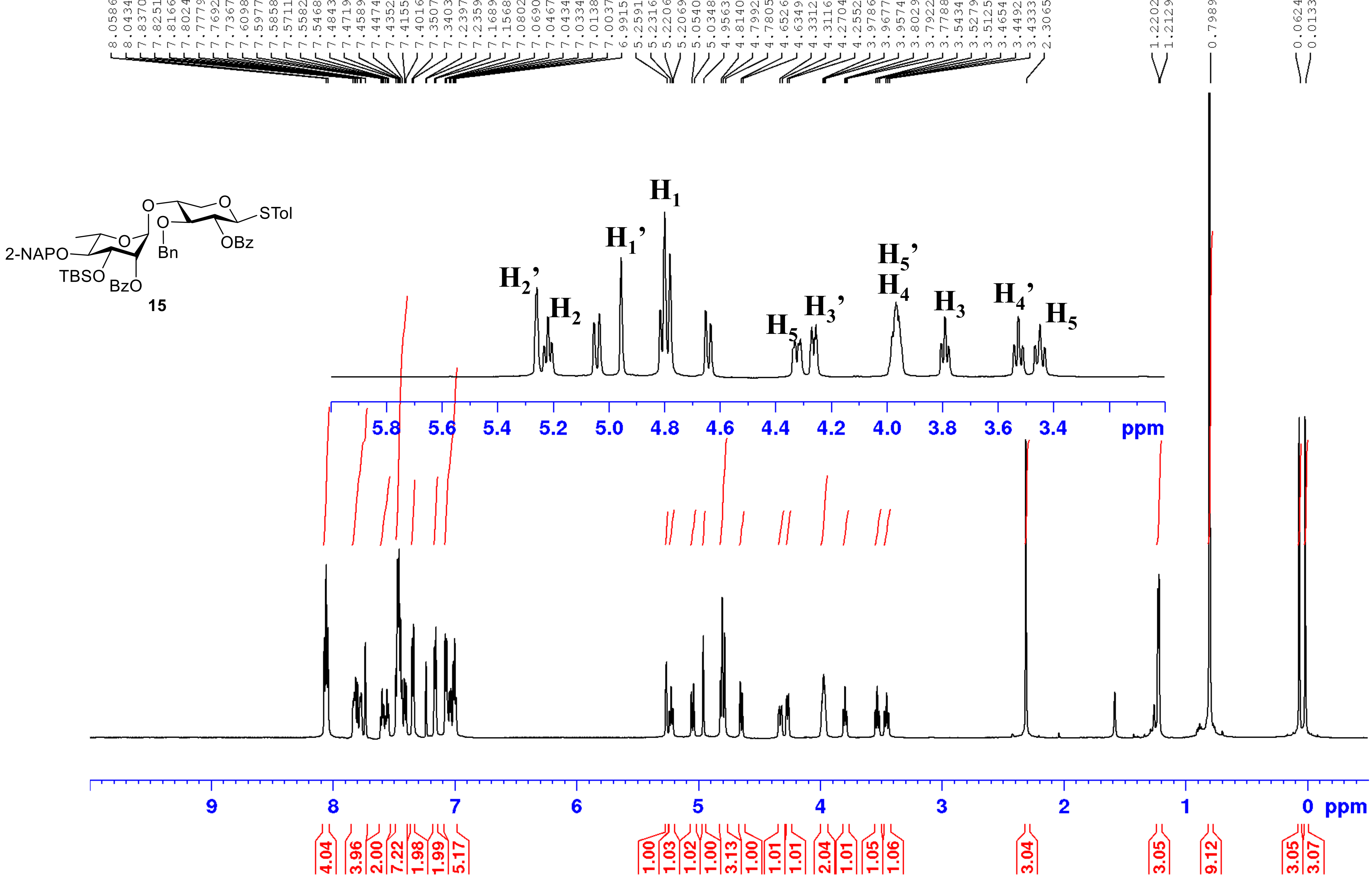

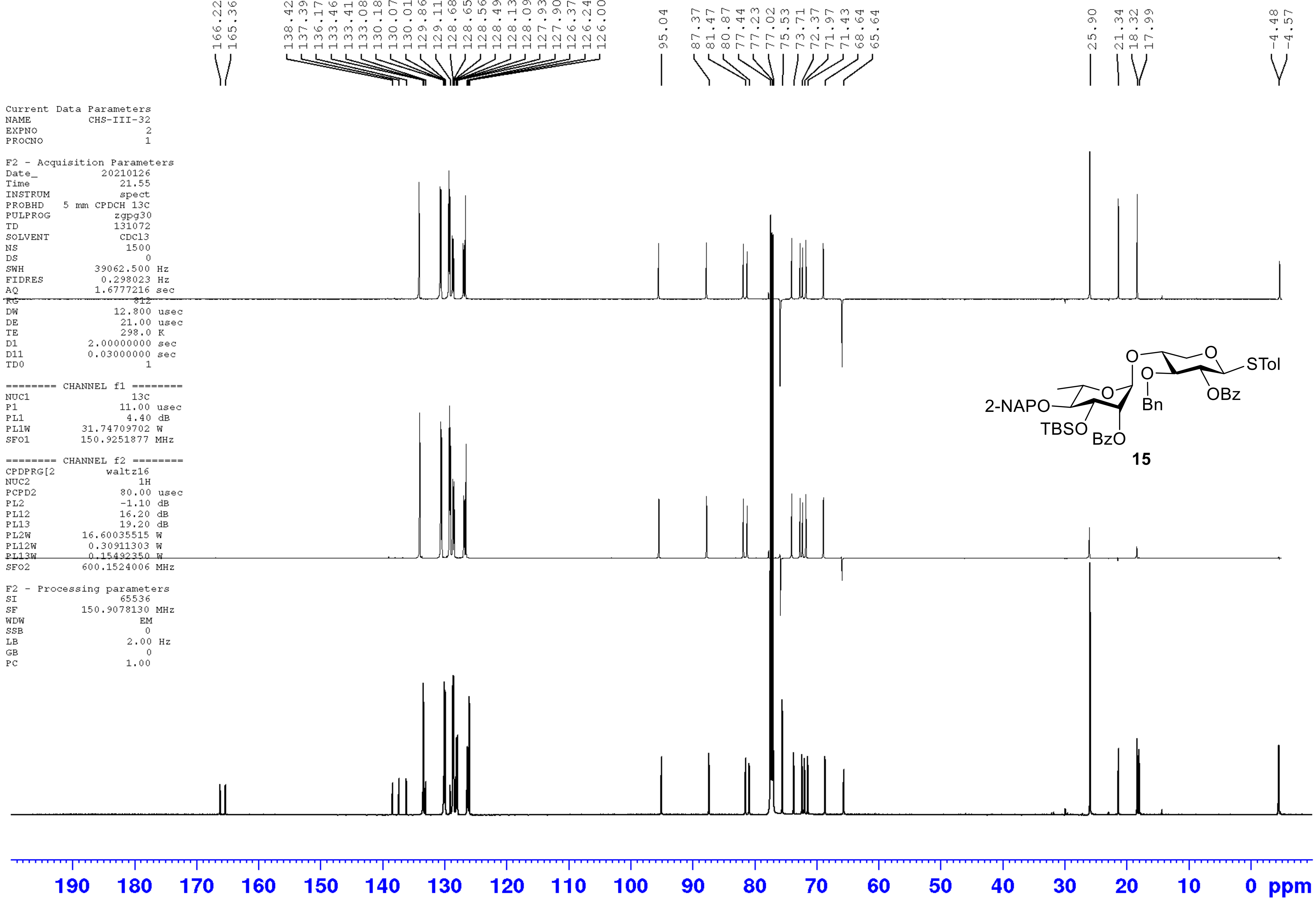
166.22
165.36
138.42
137.39
136.17
133.46
133.41
133.08
130.18
130.07
130.01
129.86
129.11
128.68
128.65
128.56
128.49
128.13
128.09
127.93
127.90
126.37
126.24
126.00
95.04
87.37
81.47
80.87
77.44
77.23
77.02
75.53
73.71
72.37
71.97
71.43
68.64
65.64
25.90
21.34
18.32
17.99
-4.48
-4.57
Current Data Parameters
NAME CHS-III-32
EXPNO 2
PROCNO 1
F2 - Acquisition Parameters
Date_ 20210126
Time 21.55
INSTRUM spect
PROBHD 5 mm CPDCH 13C
PULPROG zgpg30
TD 131072
SOLVENT CDCl3
NS 1500
DS 0
SWH 39062.500 Hz
FIDRES 0.298023 Hz
AQ 1.6777216 sec
RG 812
DW 12.800 usec
DE 21.00 usec
TE 298.0 K
D1 2.00000000 sec
D11 0.03000000 sec
TD0 1
======== CHANNEL f1 ========
NUC1 13C
P1 11.00 usec
PL1 4.40 dB
PL1W 31.74709702 W
SFO1 150.9251877 MHz
======== CHANNEL f2 ========
CPDPRG[2 waltz16
NUC2 1H
PCPD2 80.00 usec
PL2 -1.10 dB
PL12 16.20 dB
PL13 19.20 dB
PL2W 16.60035515 W
PL12W 0.30911303 W
PL13W 0.15492350 W
SFO2 600.1524006 MHz
F2 - Processing parameters
SI 65536
SF 150.9078130 MHz
WDW EM
SSB 0
LB 2.00 Hz
GB 0
PC 1.00
2-NAPO
TBSO
BzO
O
Bn
OBz
STol
15
190 180 170 160 150 140 130 120 110 100 90 80 70 60 50 40 30 20 10 0 ppm

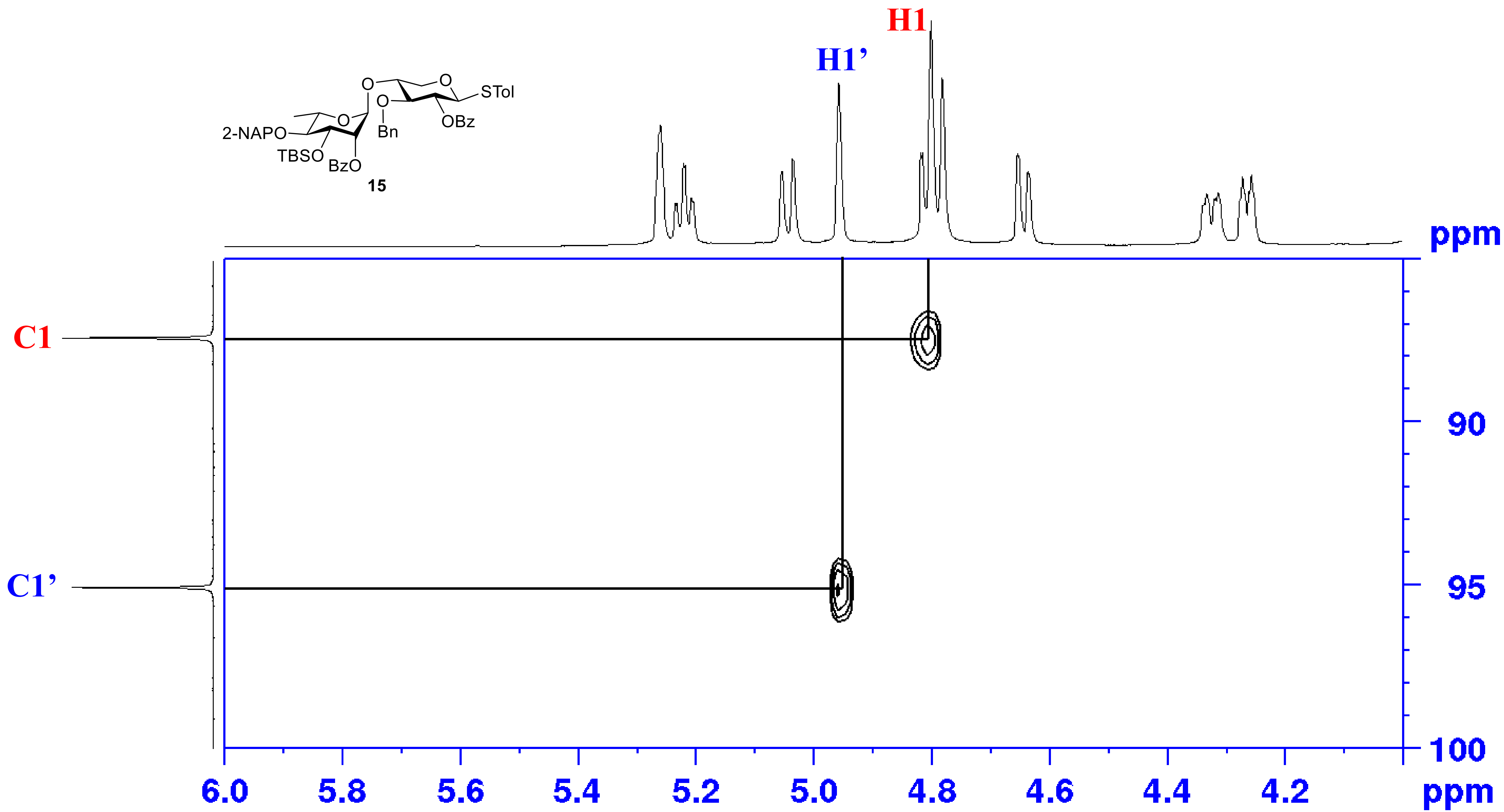

2-NAPO
TBSO
BzO
O
Bn
OBz
STol
15
H1'
H1
C1
C1'
ppm
90
95
100
6.0
5.8
5.6
5.4
5.2
5.0
4.8
4.6
4.4
4.2
ppm

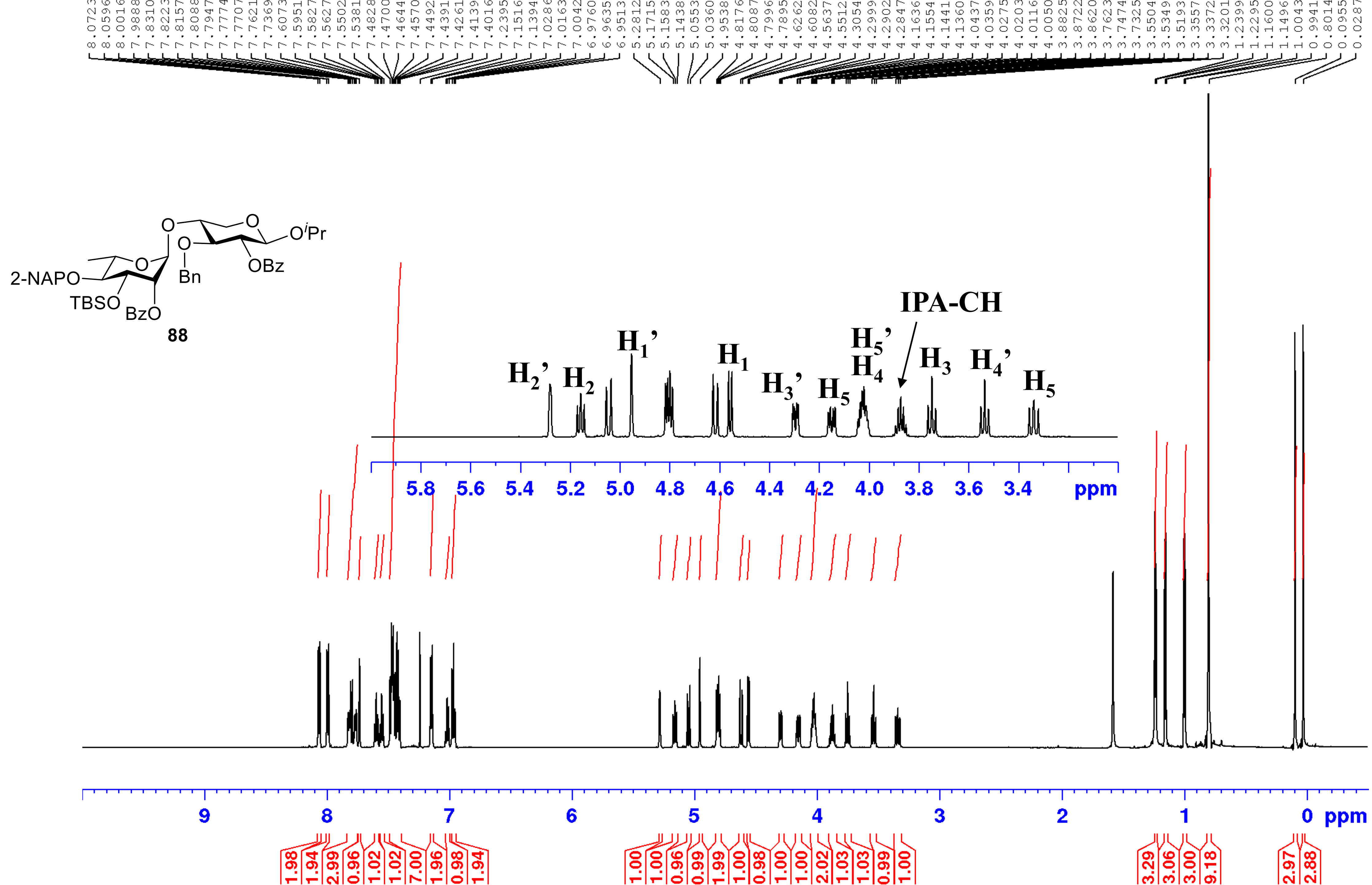

8.0723 8.0596 8.0016 7.9888 7.8310 7.8223 7.8157 7.8088 7.7947 7.7774 7.7707 7.7621 7.7369 7.6073 7.5951 7.5827 7.5627 7.5502 7.5381 7.4828 7.4700 7.4644 7.4570 7.4492 7.4391 7.4261 7.4139 7.4016 7.2395 7.1516 7.1394 7.0286 7.0163 7.0042 6.9760 6.9635 6.9513 5.2812 5.1715 5.1583 5.1438 5.0553 5.0360 4.9538 4.8176 4.8087 4.7996 4.7895 4.6262 4.6082 4.5637 4.5512 4.3054 4.2999 4.2902 4.2847 4.1636 4.1554 4.1441 4.1360 4.0437 4.0359 4.0275 4.0203 4.0116 4.0050 3.8825 3.8722 3.8620 3.7623 3.7474 3.7325 3.5504 3.5349 3.5193 3.3557 3.3372 3.3201 1.2399 1.2295 1.1600 1.1496 1.0043 0.9941 0.8014 0.0955 0.0287
O
O
O^{i}Pr
O
Bn
OBz
2-NAPO
O
TBSO
BzO
88
IPA-CH
H$_2$'
H$_2$
H$_1$'
H$_1$
H$_3$'
H$_5$
H$_5$'
H$_4$
H$_3$
H$_4$'
H$_5$
5.8 5.6 5.4 5.2 5.0 4.8 4.6 4.4 4.2 4.0 3.8 3.6 3.4 ppm
9 8 7 6 5 4 3 2 1 0 ppm
1.98 1.94 2.99 0.96 1.02 1.02 7.00 1.96 0.98 1.94
1.00 1.00 0.96 0.99 1.99 1.00 0.98 1.00 1.00 2.02 1.03 1.03 0.99 1.00
3.29 3.06 3.00 9.18
2.97 2.88

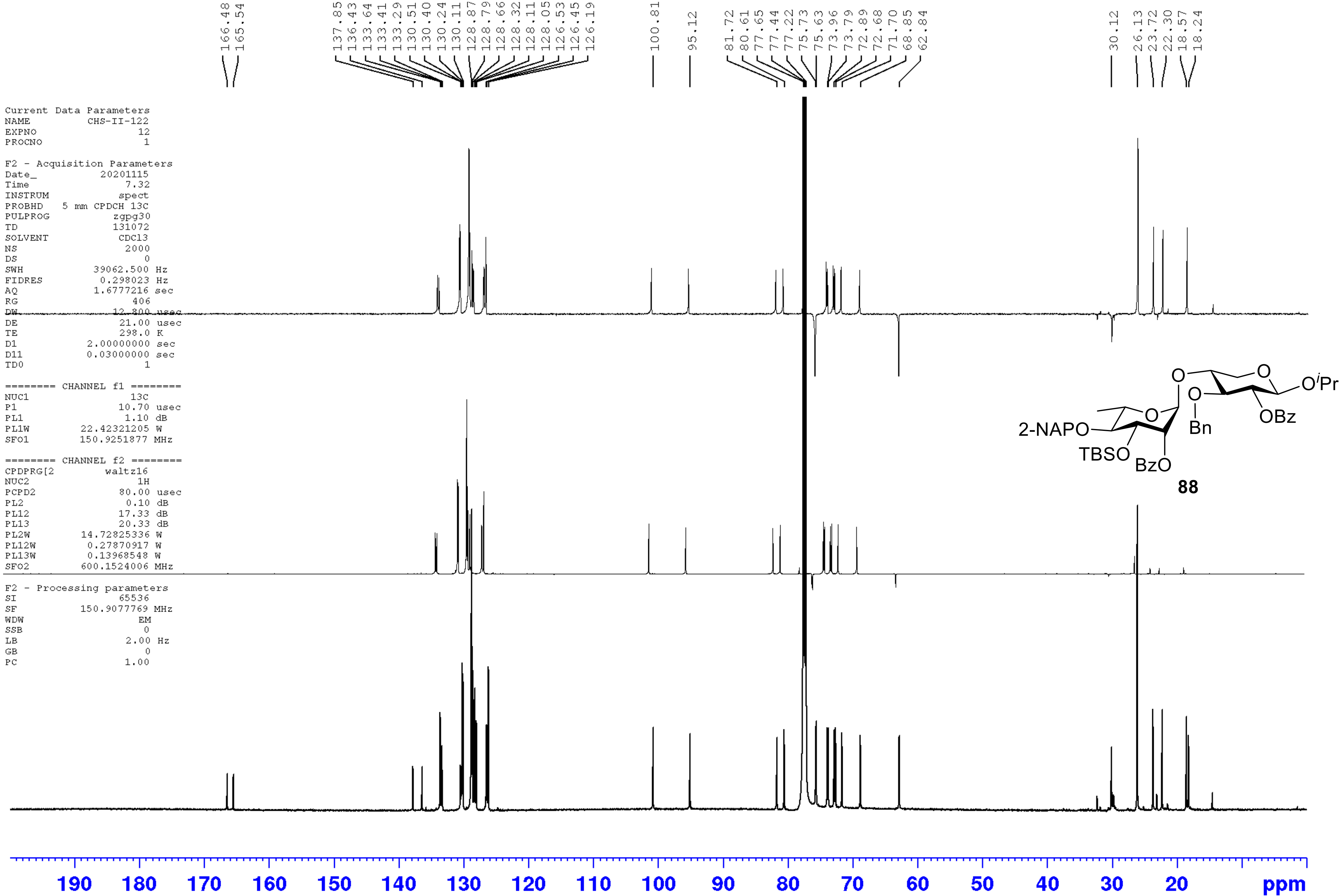

166.48
165.54
137.85
136.43
133.64
133.41
133.29
130.51
130.40
130.24
130.11
128.87
128.79
128.66
128.32
128.11
128.05
126.53
126.45
126.19
100.81
95.12
81.72
80.61
77.65
77.44
77.22
75.73
75.63
73.96
73.79
72.89
72.68
71.70
68.85
62.84
30.12
26.13
23.72
22.30
18.57
18.24
Current Data Parameters
NAME CHS-II-122
EXPNO 12
PROCNO 1
F2 - Acquisition Parameters
Date_ 20201115
Time 7.32
INSTRUM spect
PROBHD 5 mm CPDCH 13C
PULPROG zgpg30
TD 131072
SOLVENT CDCl3
NS 2000
DS 0
SWH 39062.500 Hz
FIDRES 0.298023 Hz
AQ 1.6777216 sec
RG 406
DW 12.800 usec
DE 21.00 usec
TE 298.0 K
D1 2.00000000 sec
D11 0.03000000 sec
TD0 1
======== CHANNEL f1 ========
NUC1 13C
P1 10.70 usec
PL1 1.10 dB
PL1W 22.42321205 W
SFO1 150.9251877 MHz
======== CHANNEL f2 ========
CPDPRG[2 waltz16
NUC2 1H
PCPD2 80.00 usec
PL2 0.10 dB
PL12 17.33 dB
PL13 20.33 dB
PL2W 14.72825336 W
PL12W 0.27870917 W
PL13W 0.13968548 W
SFO2 600.1524006 MHz
F2 - Processing parameters
SI 65536
SF 150.9077769 MHz
WDW EM
SSB 0
LB 2.00 Hz
GB 0
PC 1.00
2-NAPO
TBSO
BzO
Bn
OBz
O^{i}Pr
88
190 180 170 160 150 140 130 120 110 100 90 80 70 60 50 40 30 20 ppm

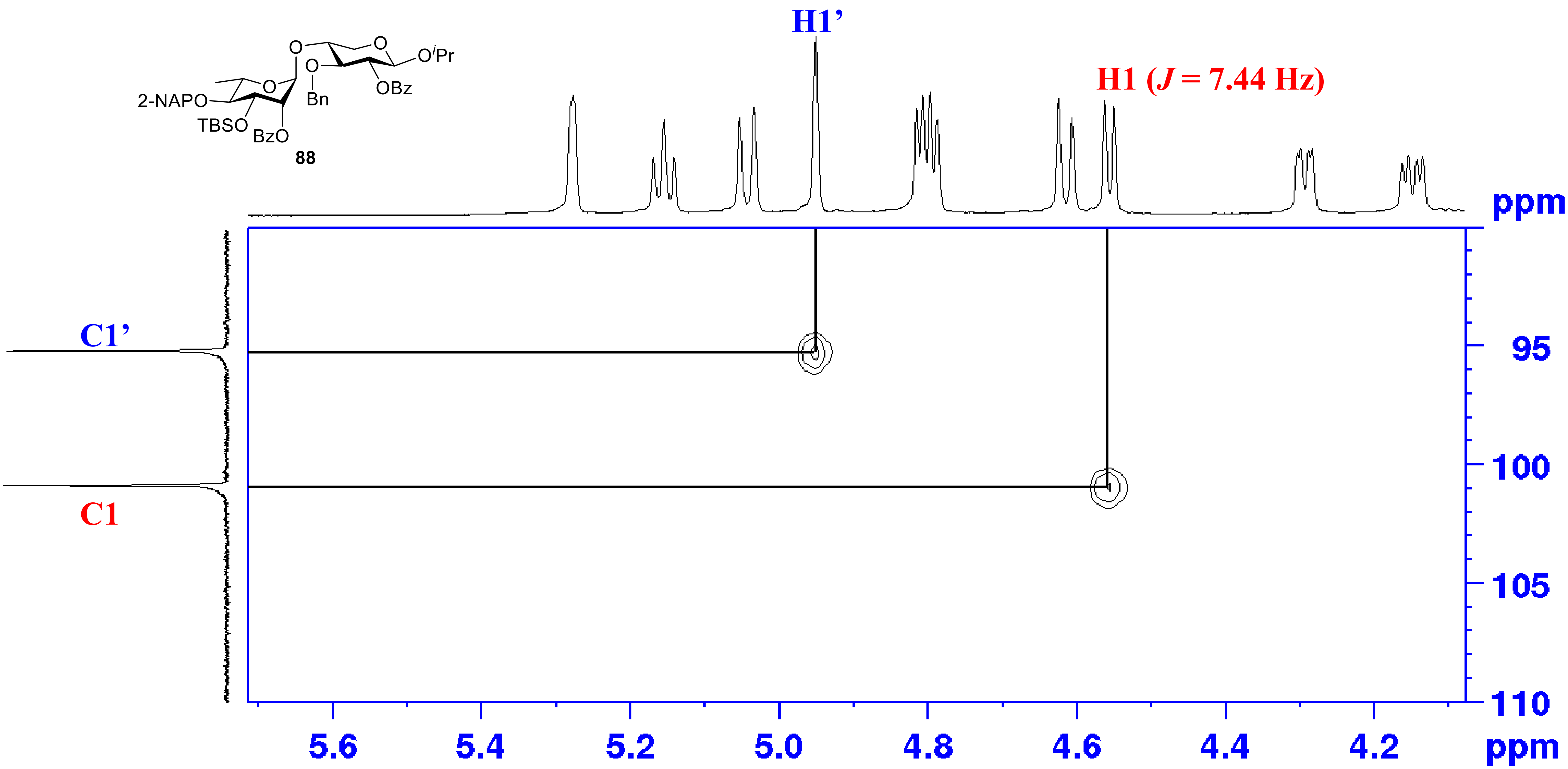
2-NAPO
TBSO
BzO
O
Bn
OBz
O^{i}Pr
88
H1’
H1 (J = 7.44 Hz)
ppm
C1’
C1
95
100
105
110
5.6
5.4
5.2
5.0
4.8
4.6
4.4
4.2
ppm

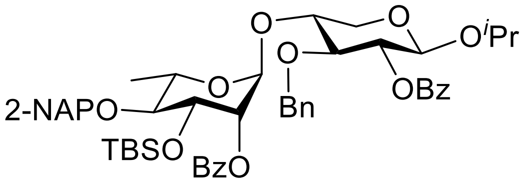
O
O
O
O^{i}Pr
2-NAPO
TBSO
BzO
Bn
OBz

88

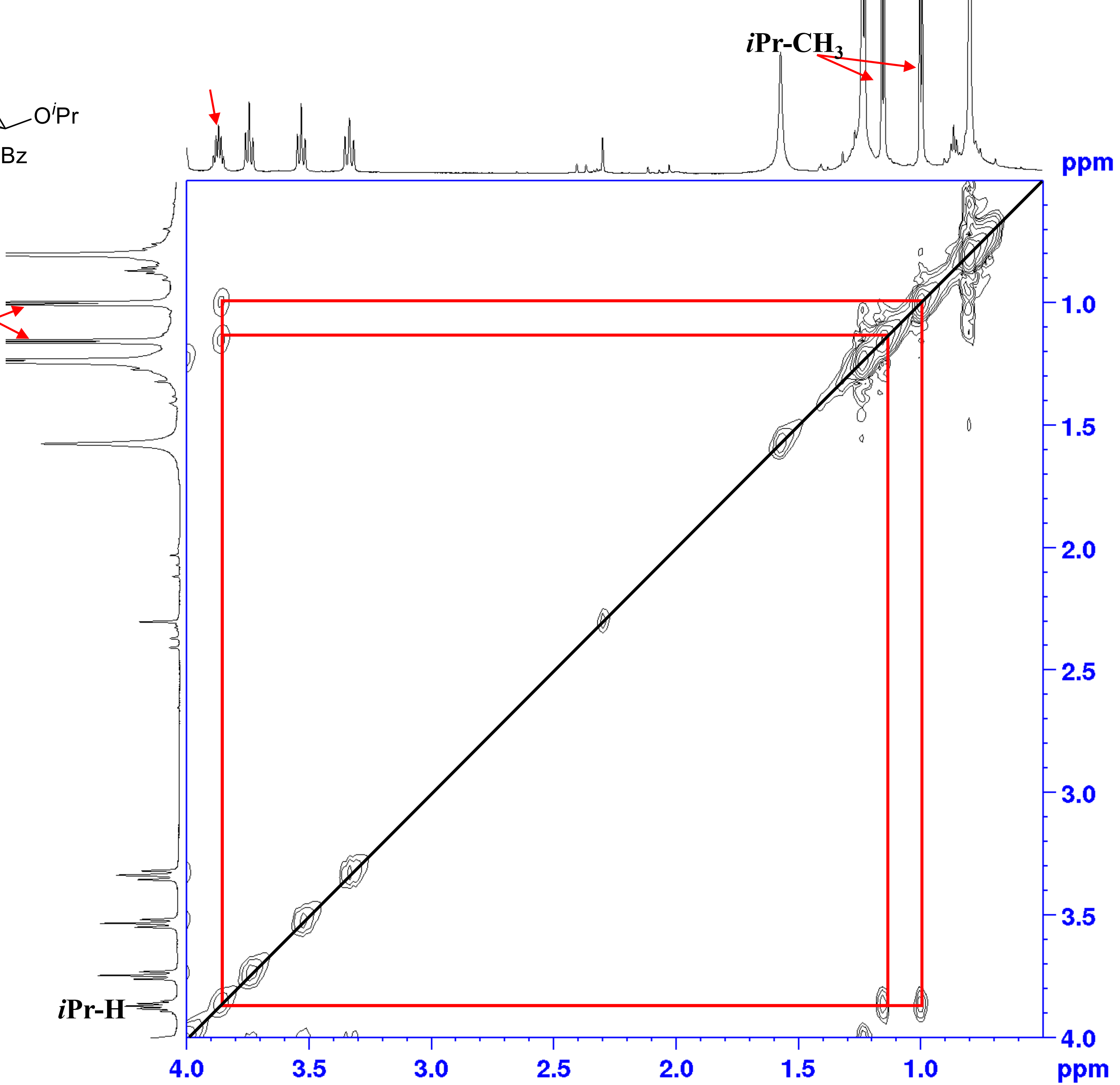
*i*Pr-CH$_3$
*i*Pr-CH$_3$
*i*Pr-H
ppm
1.0
1.5
2.0
2.5
3.0
3.5
4.0
4.0
3.5
3.0
2.5
2.0
1.5
1.0
ppm

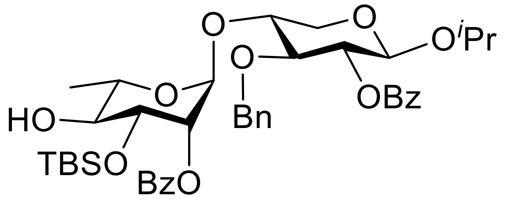
O
O
O
O^{i}Pr
O
Bn
OBz
HO
TBSO
BzO

89

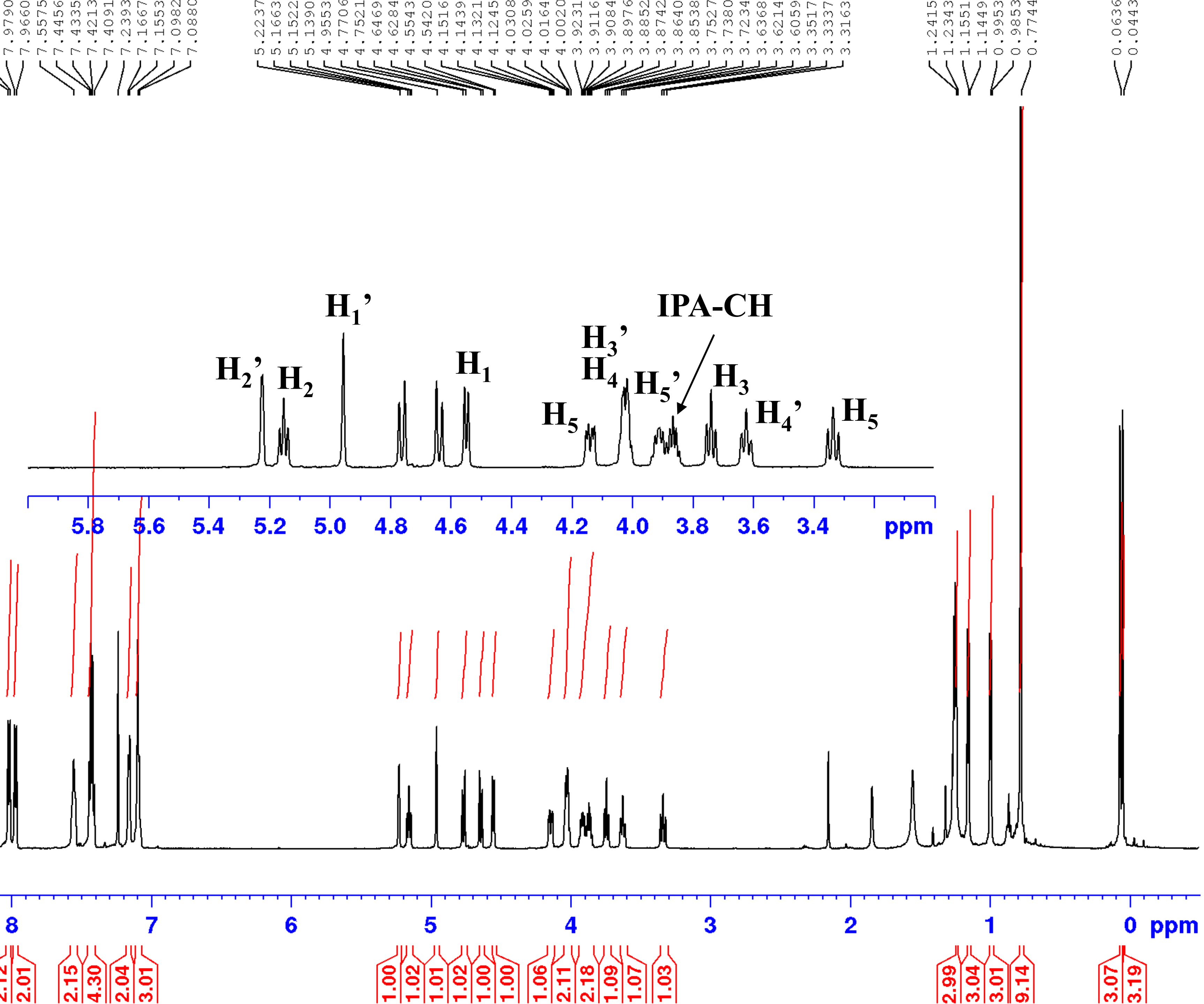
8.0261
8.0131
7.9790
7.9660
7.5575
7.4456
7.4335
7.4213
7.4091
7.2393
7.1667
7.1553
7.0982
7.0880
5.2237
5.1663
5.1522
5.1390
4.9553
4.7706
4.7521
4.6469
4.6284
4.5543
4.5420
4.1516
4.1439
4.1321
4.1245
4.0308
4.0259
4.0164
4.0020
3.9231
3.9116
3.9084
3.8976
3.8852
3.8742
3.8640
3.8538
3.7527
3.7380
3.7234
3.6368
3.6214
3.6059
3.3517
3.3337
3.3163
1.2415
1.2343
1.1551
1.1449
0.9953
0.9853
0.7744
0.0636
0.0443
H2'
H2
H1'
H1
H5
H3'
H4
H5'
IPA-CH
H3
H4'
H5
5.8 5.6 5.4 5.2 5.0 4.8 4.6 4.4 4.2 4.0 3.8 3.6 3.4 ppm
9 8 7 6 5 4 3 2 1 0 ppm
2.12
2.01
2.15
4.30
2.04
3.01
1.00
1.02
1.01
1.02
1.00
1.00
1.06
2.11
2.18
1.09
1.07
1.03
2.99
3.04
3.01
9.14
3.07
3.19

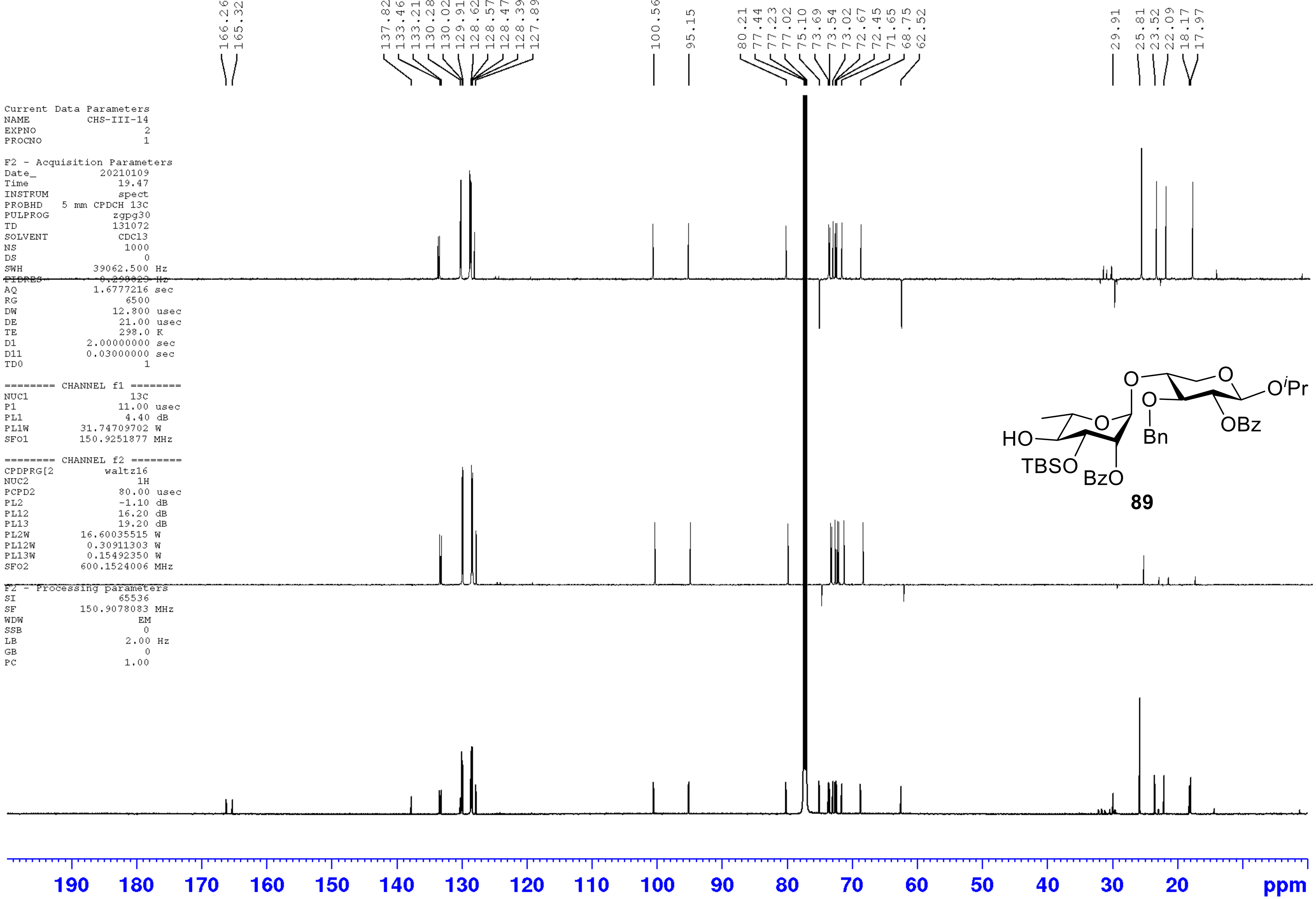
166.26
165.32
137.82
133.46
133.21
130.28
130.02
129.91
128.62
128.57
128.47
128.39
127.89
100.56
95.15
80.21
77.44
77.23
77.02
75.10
73.69
73.54
73.02
72.67
72.45
71.65
68.75
62.52
29.91
25.81
23.52
22.09
18.17
17.97
Current Data Parameters
NAME CHS-III-14
EXPNO 2
PROCNO 1
F2 - Acquisition Parameters
Date_ 20210109
Time 19.47
INSTRUM spect
PROBHD 5 mm CPDCH 13C
PULPROG zgpg30
TD 131072
SOLVENT CDCl3
NS 1000
DS 0
SWH 39062.500 Hz
FIDRES 0.298023 Hz
AQ 1.6777216 sec
RG 6500
DW 12.800 usec
DE 21.00 usec
TE 298.0 K
D1 2.00000000 sec
D11 0.03000000 sec
TD0 1
======== CHANNEL f1 ========
NUC1 13C
P1 11.00 usec
PL1 4.40 dB
PL1W 31.74709702 W
SFO1 150.9251877 MHz
======== CHANNEL f2 ========
CPDPRG[2 waltz16
NUC2 1H
PCPD2 80.00 usec
PL2 -1.10 dB
PL12 16.20 dB
PL13 19.20 dB
PL2W 16.60035515 W
PL12W 0.30911303 W
PL13W 0.15492350 W
SFO2 600.1524006 MHz
F2 - Processing parameters
SI 65536
SF 150.9078083 MHz
WDW EM
SSB 0
LB 2.00 Hz
GB 0
PC 1.00
190 180 170 160 150 140 130 120 110 100 90 80 70 60 50 40 30 20 ppm
O
O
O^{i}Pr
O
Bn
OBz
HO
O
TBSO
BzO

89

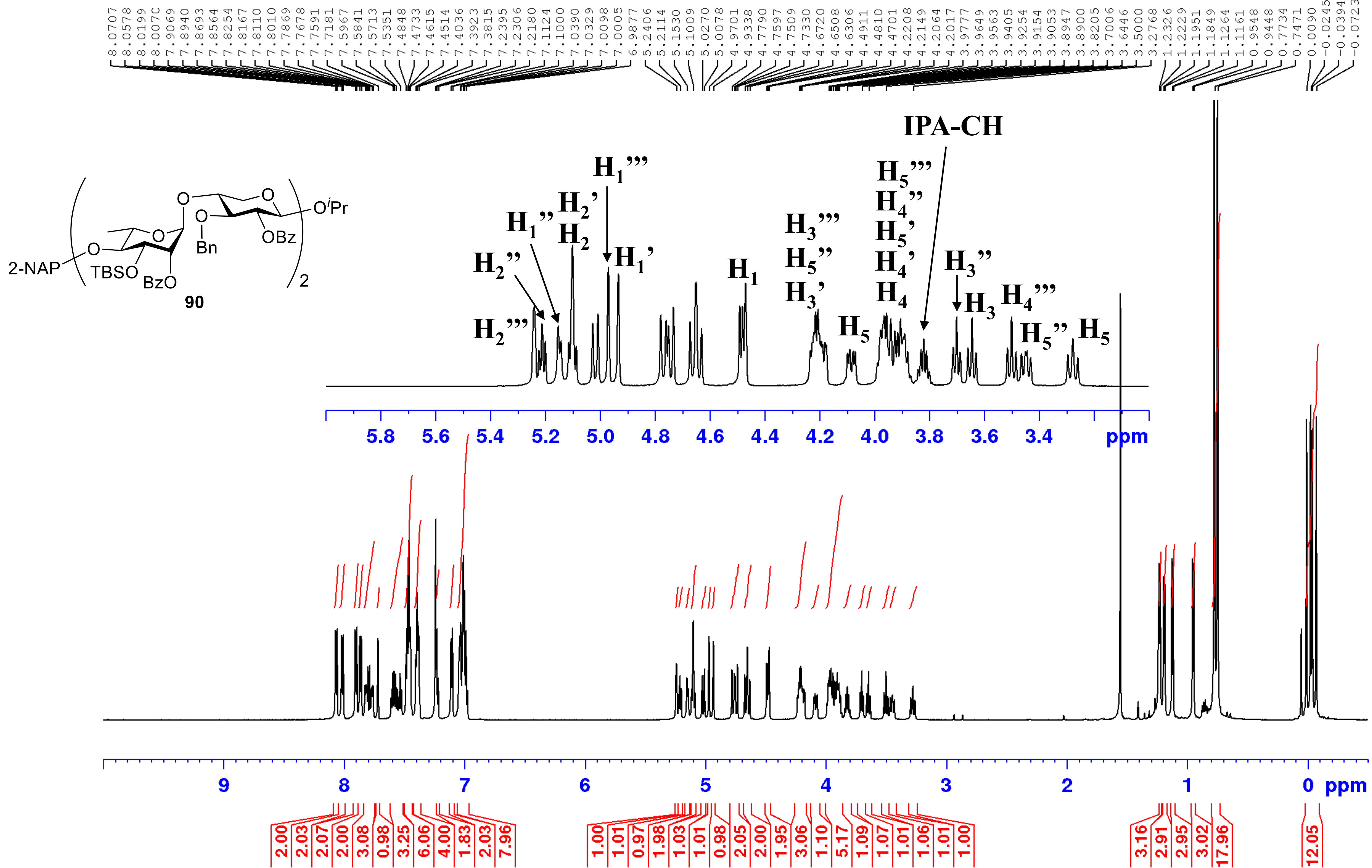

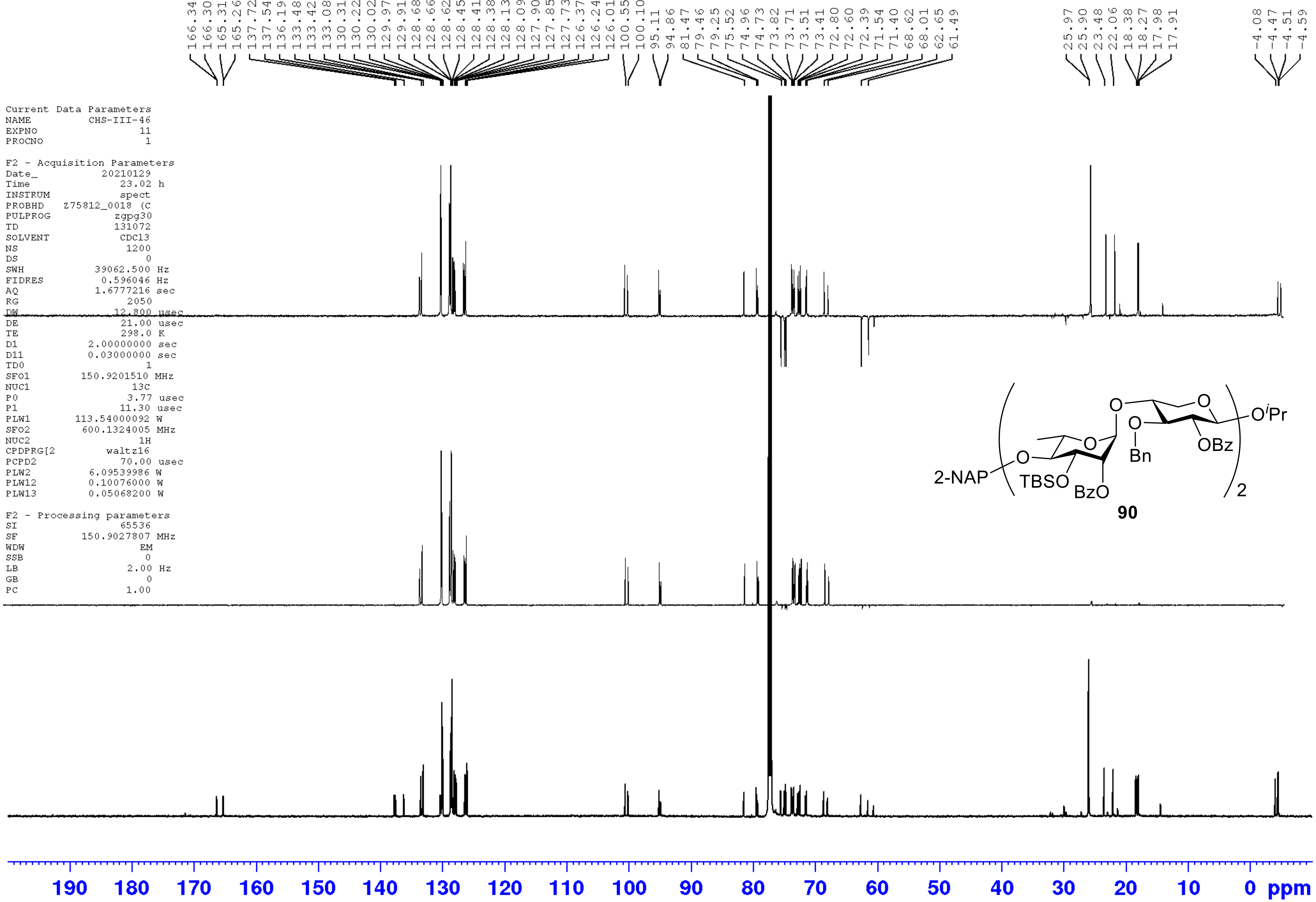

166.34
166.30
165.31
165.26
137.72
137.54
136.19
133.48
133.42
133.08
130.31
130.22
130.02
129.97
129.91
128.68
128.66
128.62
128.45
128.41
128.38
128.13
128.09
127.90
127.85
127.73
126.37
126.24
126.01
100.55
100.10
95.11
94.86
81.47
79.46
79.25
75.52
74.96
74.73
73.82
73.71
73.51
73.41
72.80
72.60
72.39
71.54
71.40
68.62
68.01
62.65
61.49
25.97
25.90
23.48
22.06
18.38
18.27
17.98
17.91
-4.08
-4.47
-4.51
-4.59
Current Data Parameters
NAME CHS-III-46
EXPNO 11
PROCNO 1
F2 - Acquisition Parameters
Date_ 20210129
Time 23.02 h
INSTRUM spect
PROBHD Z75812_0018 (C
PULPROG zgpg30
TD 131072
SOLVENT CDCl3
NS 1200
DS 0
SWH 39062.500 Hz
FIDRES 0.596046 Hz
AQ 1.6777216 sec
RG 2050
DW 12.800 usec
DE 21.00 usec
TE 298.0 K
D1 2.00000000 sec
D11 0.03000000 sec
TD0 1
SFO1 150.9201510 MHz
NUC1 13C
P0 3.77 usec
P1 11.30 usec
PLW1 113.54000092 W
SFO2 600.1324005 MHz
NUC2 1H
CPDPRG[2 waltz16
PCPD2 70.00 usec
PLW2 6.09539986 W
PLW12 0.10076000 W
PLW13 0.05068200 W
F2 - Processing parameters
SI 65536
SF 150.9027807 MHz
WDW EM
SSB 0
LB 2.00 Hz
GB 0
PC 1.00
O
O
O
O^iPr
O
Bn
OBz
O
2-NAP
O
TBSO
BzO
2
90
190 180 170 160 150 140 130 120 110 100 90 80 70 60 50 40 30 20 10 0 ppm

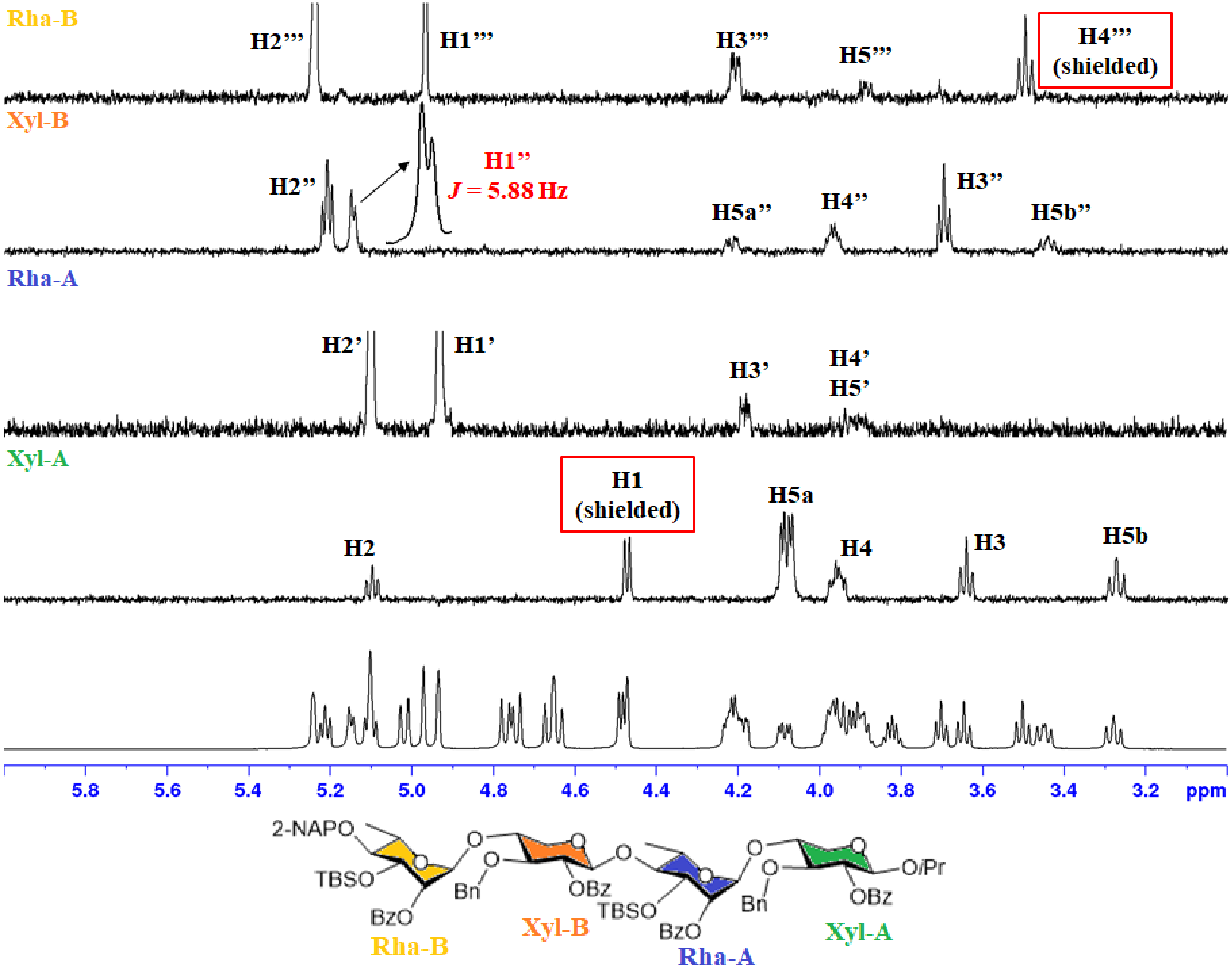
Rha-B
H2'''
H1'''
H3'''
H5'''
H4''' (shielded)
Xyl-B
H2''
H1''
J = 5.88 Hz
H5a''
H4''
H3''
H5b''
Rha-A
H2'
H1'
H3'
H4'
H5'
Xyl-A
H1 (shielded)
H2
H5a
H4
H3
H5b
5.8
5.6
5.4
5.2
5.0
4.8
4.6
4.4
4.2
4.0
3.8
3.6
3.4
3.2
ppm
2-NAPO
TBSO
BzO
Bn
OBz
TBSO
BzO
Bn
OBz
OiPr
Rha-B
Xyl-B
Rha-A
Xyl-A

90

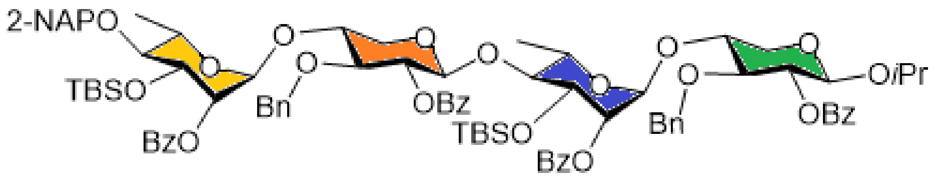
2-NAPO
TBSO
BzO
Bn
OBz
TBSO
BzO
Bn
OBz
O*i*Pr

90

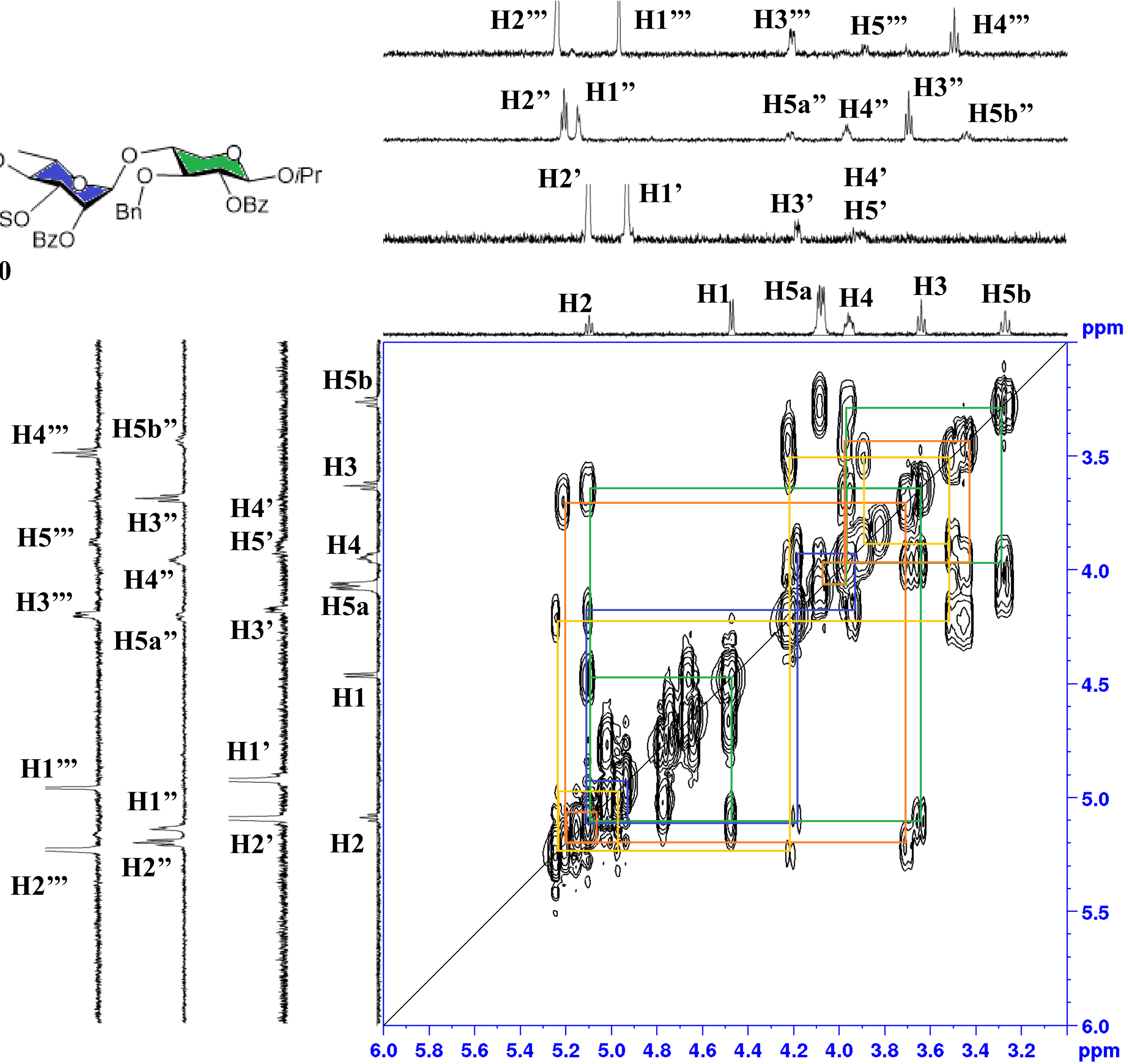
H2''' H1''' H3''' H5''' H4'''
H2'' H1'' H5a'' H4'' H3'' H5b''
H2' H1' H3' H4' H5'
H2 H1 H5a H4 H3 H5b
ppm
6.0 5.8 5.6 5.4 5.2 5.0 4.8 4.6 4.4 4.2 4.0 3.8 3.6 3.4 3.2
3.5 4.0 4.5 5.0 5.5 6.0

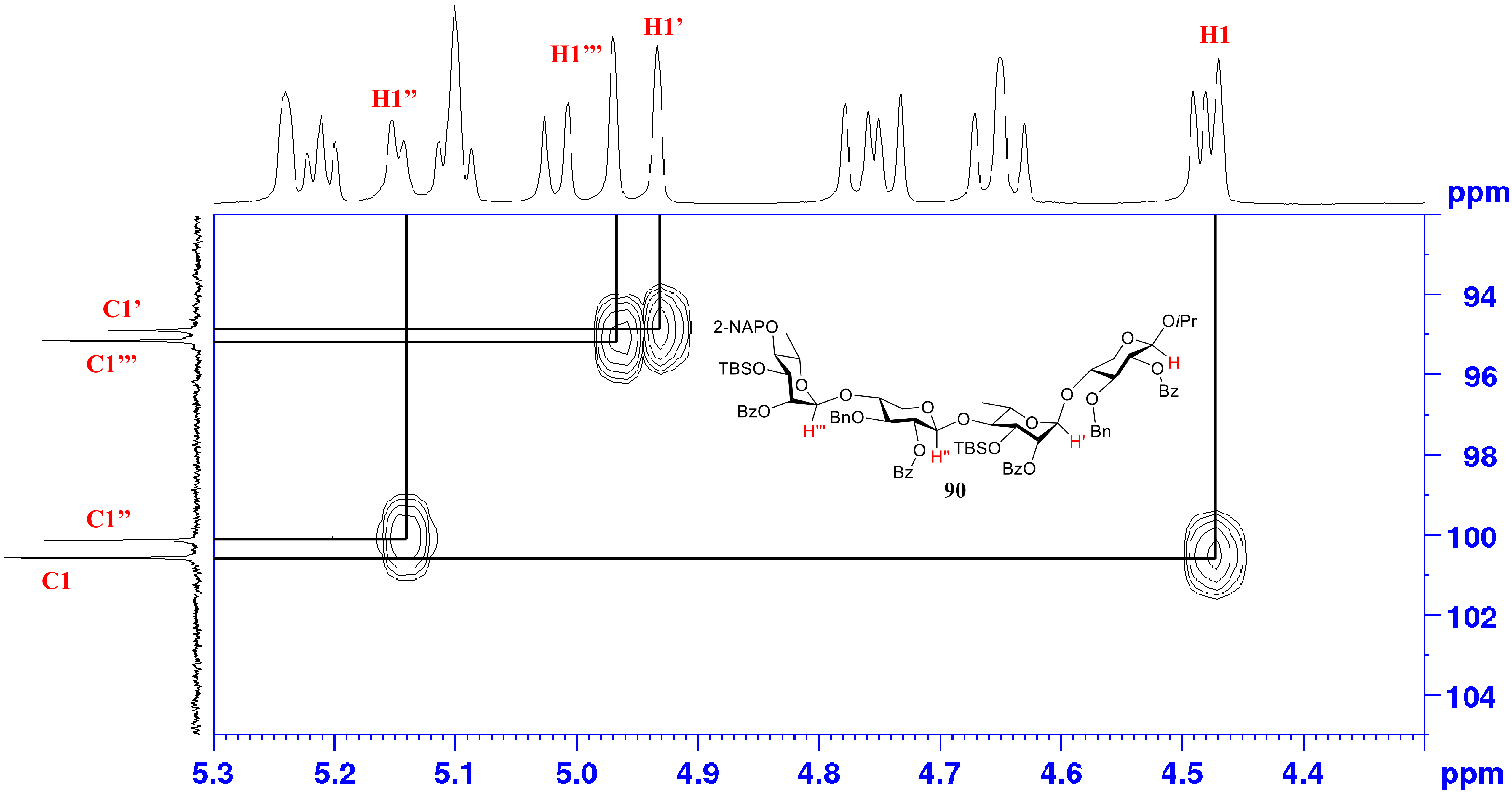
H1"
H1'''
H1'
H1
ppm
C1'
C1'''
C1"
C1
94
96
98
100
102
104
5.3
5.2
5.1
5.0
4.9
4.8
4.7
4.6
4.5
4.4
ppm
2-NAPO
TBSO
BzO
H'''
BnO
O
Bz
H"
TBSO
BzO
H'
Bn
OiPr
H
Bz
90

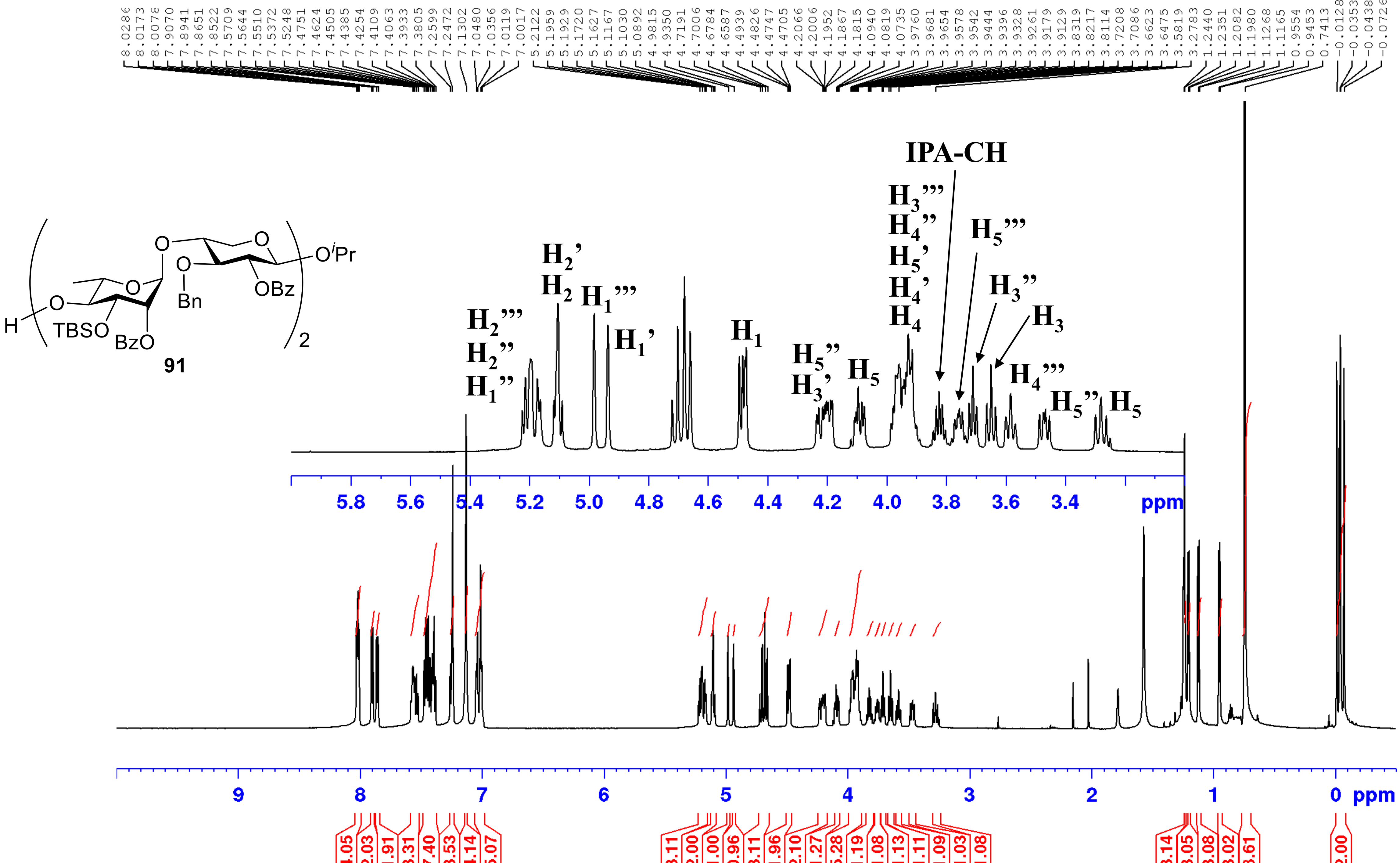

H
O
TBSO
BzO
O
Bn
O
OBz
O
O^iPr
2
91
IPA-CH
H2'''
H2''
H1''
H2'
H2
H1'''
H1'
H1
H5''
H3'
H5
H3'''
H4''
H5'
H4'
H4
H5'''
H3''
H3
H4'''
H5''
H5
ppm

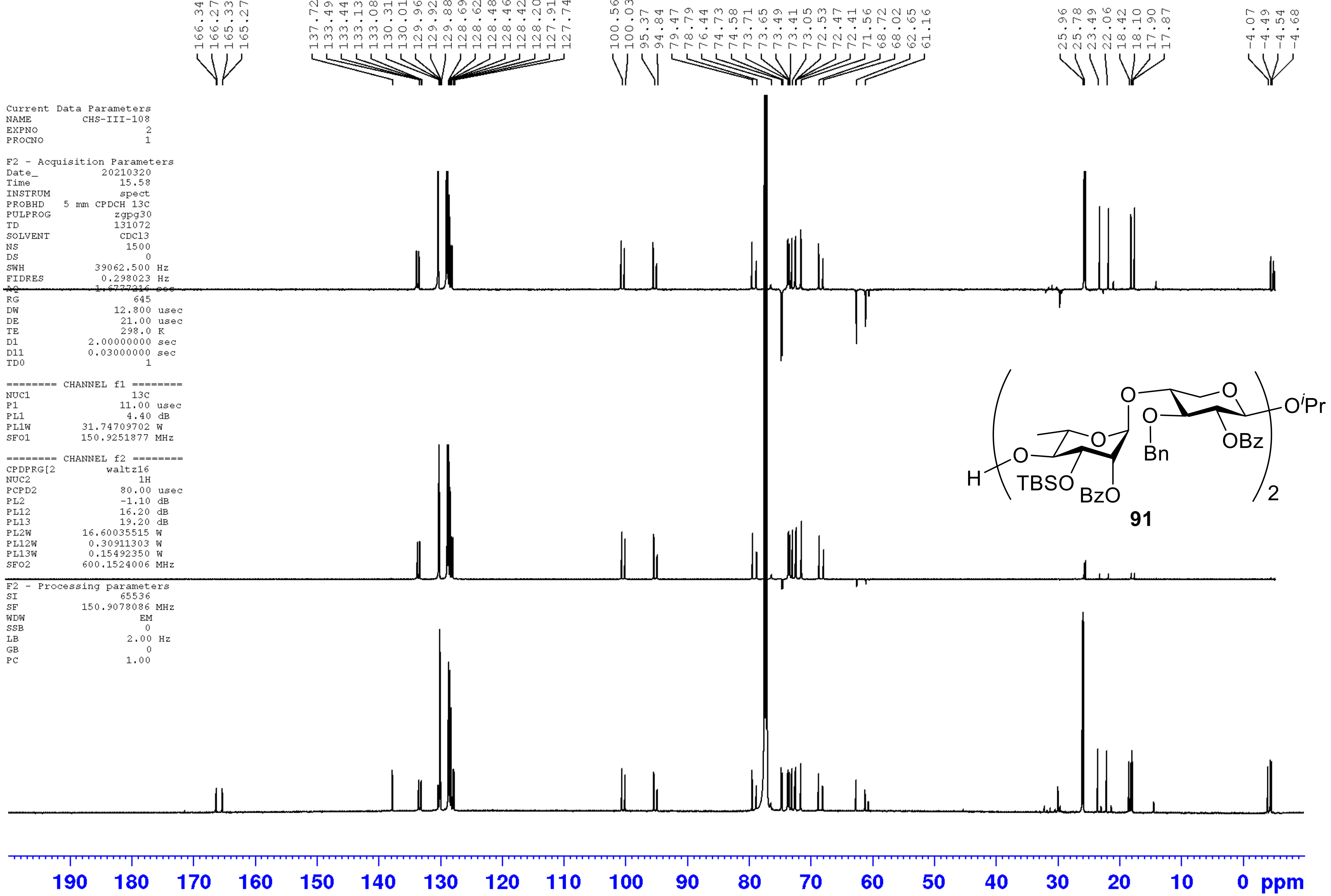
166.34
166.27
165.33
165.27
137.72
133.49
133.44
133.13
133.08
130.31
130.01
129.96
129.92
129.88
128.69
128.62
128.48
128.46
128.42
128.20
127.91
127.74
100.56
100.03
95.37
94.84
79.47
78.79
76.44
74.73
74.58
73.71
73.65
73.49
73.41
73.05
72.53
72.47
72.41
71.56
68.72
68.02
62.65
61.16
25.96
25.78
23.49
22.06
18.42
18.10
17.90
17.87
-4.07
-4.49
-4.54
-4.68
Current Data Parameters
NAME CHS-III-108
EXPNO 2
PROCNO 1
F2 - Acquisition Parameters
Date_ 20210320
Time 15.58
INSTRUM spect
PROBHD 5 mm CPDCH 13C
PULPROG zgpg30
TD 131072
SOLVENT CDCl3
NS 1500
DS 0
SWH 39062.500 Hz
FIDRES 0.298023 Hz
AQ 1.6777216 sec
RG 645
DW 12.800 usec
DE 21.00 usec
TE 298.0 K
D1 2.00000000 sec
D11 0.03000000 sec
TD0 1
======== CHANNEL f1 ========
NUC1 13C
P1 11.00 usec
PL1 4.40 dB
PL1W 31.74709702 W
SFO1 150.9251877 MHz
======== CHANNEL f2 ========
CPDPRG[2 waltz16
NUC2 1H
PCPD2 80.00 usec
PL2 -1.10 dB
PL12 16.20 dB
PL13 19.20 dB
PL2W 16.60035515 W
PL12W 0.30911303 W
PL13W 0.15492350 W
SFO2 600.1524006 MHz
F2 - Processing parameters
SI 65536
SF 150.9078086 MHz
WDW EM
SSB 0
LB 2.00 Hz
GB 0
PC 1.00
O
O
O
O
O
O-iPr
OBz
Bn
H
TBSO
BzO
2
91
190 180 170 160 150 140 130 120 110 100 90 80 70 60 50 40 30 20 10 0 ppm

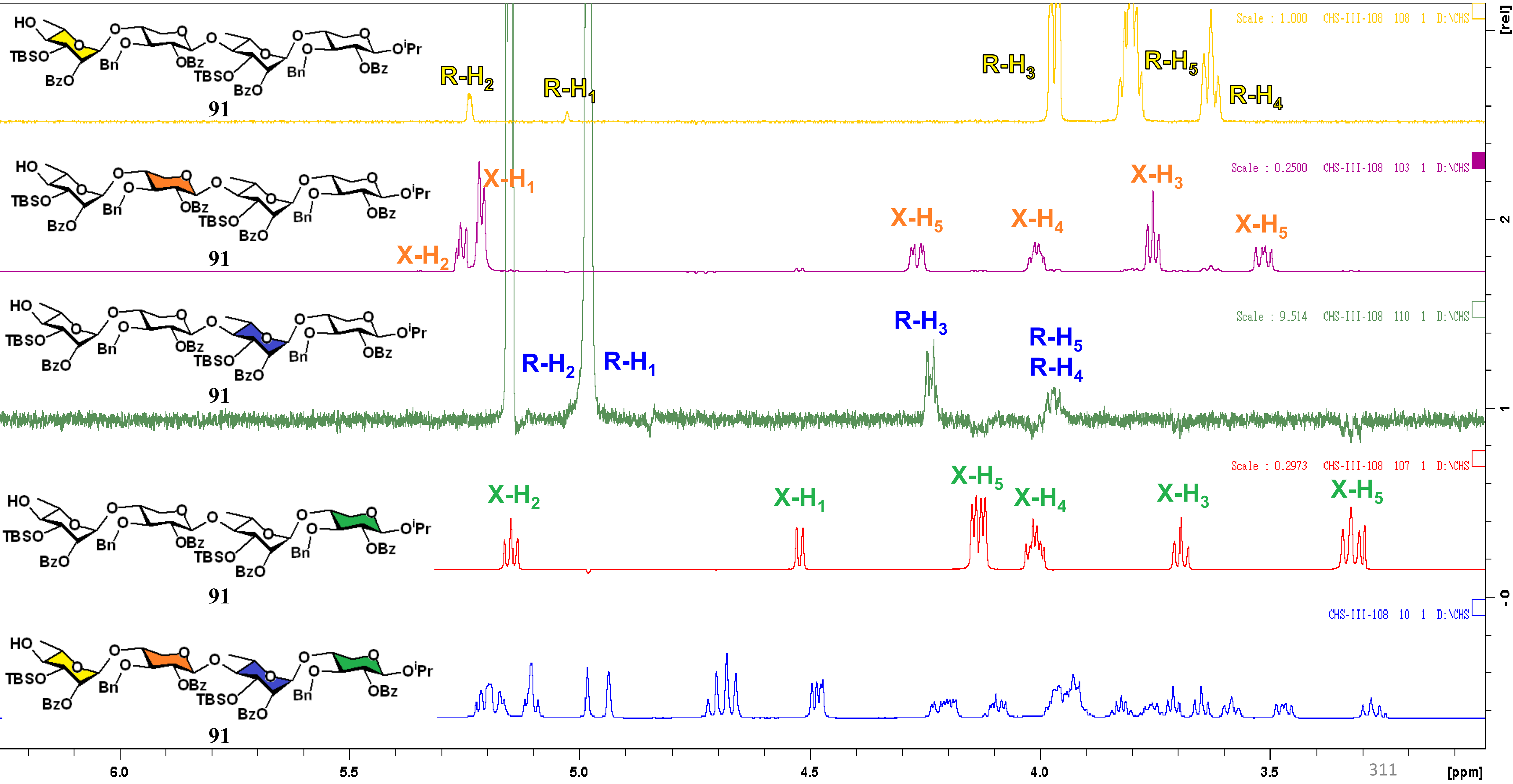

R-H2
R-H1
R-H3
R-H5
R-H4
Scale : 1.000 CHS-III-108 108 1 D:\CHS
X-H1
X-H2
X-H5
X-H4
X-H3
X-H5
Scale : 0.2500 CHS-III-108 103 1 D:\CHS
R-H2
R-H1
R-H3
R-H5
R-H4
Scale : 9.514 CHS-III-108 110 1 D:\CHS
X-H2
X-H1
X-H5
X-H4
X-H3
X-H5
Scale : 0.2973 CHS-III-108 107 1 D:\CHS
CHS-III-108 10 1 D:\CHS
91
91
91
91
91
6.0
5.5
5.0
4.5
4.0
3.5
[ppm]
[rel]
2
1
0

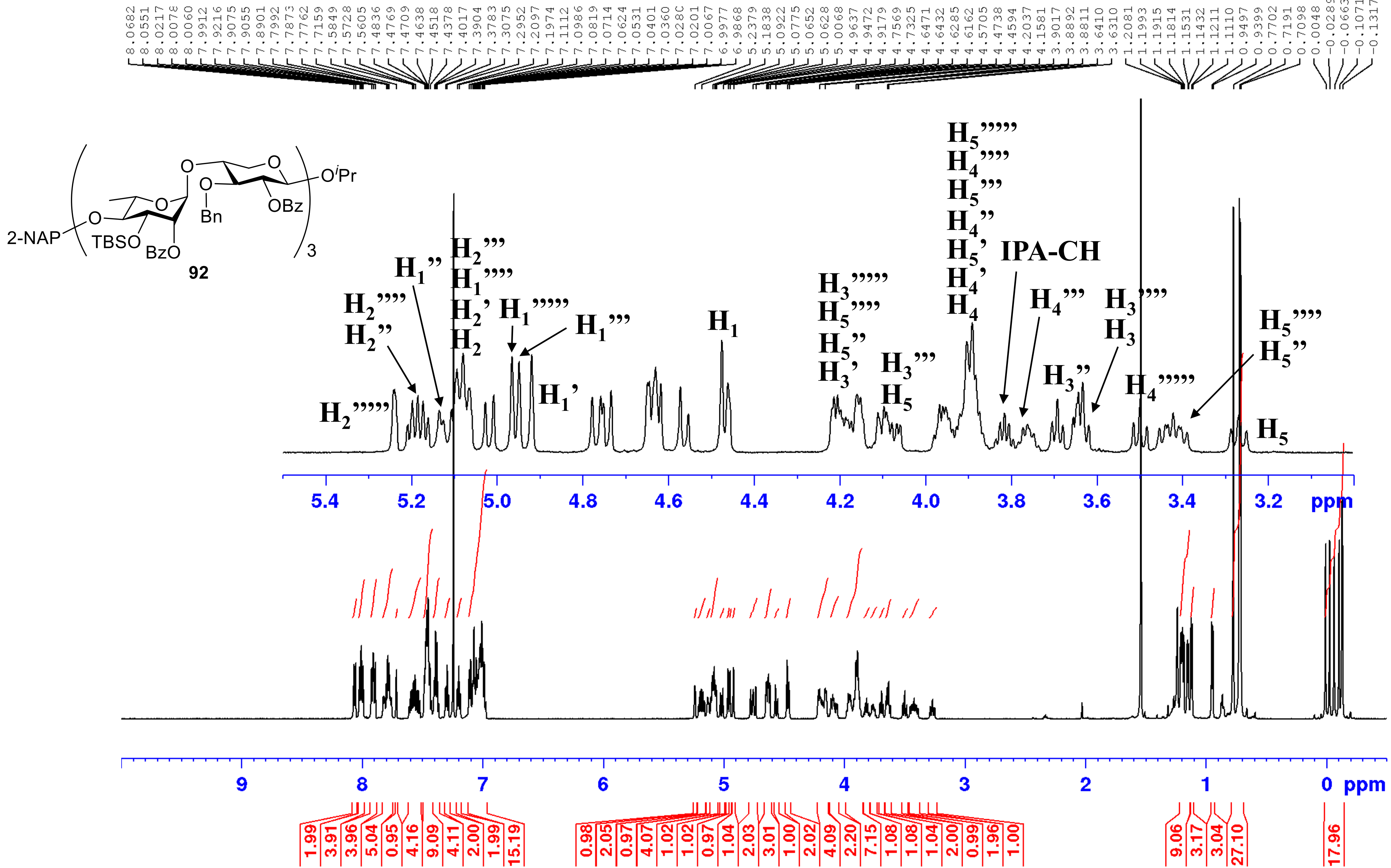

2-NAP
TBSO
BzO
O
Bn
OBz
O^{i}Pr
3
92
H$_2$'''''
H$_2$''''
H$_2$''
H$_1$''
H$_2$'''
H$_1$''''
H$_2$'
H$_2$
H$_1$'''''
H$_1$'''
H$_1$'
H$_1$
H$_3$'''''
H$_5$''''
H$_5$''
H$_3$'
H$_3$'''
H$_5$
H$_5$'''''
H$_4$''''
H$_5$'''
H$_4$''
H$_5$'
H$_4$'
H$_4$
IPA-CH
H$_4$'''
H$_3$''
H$_3$''''
H$_3$
H$_4$'''''
H$_5$''''
H$_5$''
H$_5$
ppm

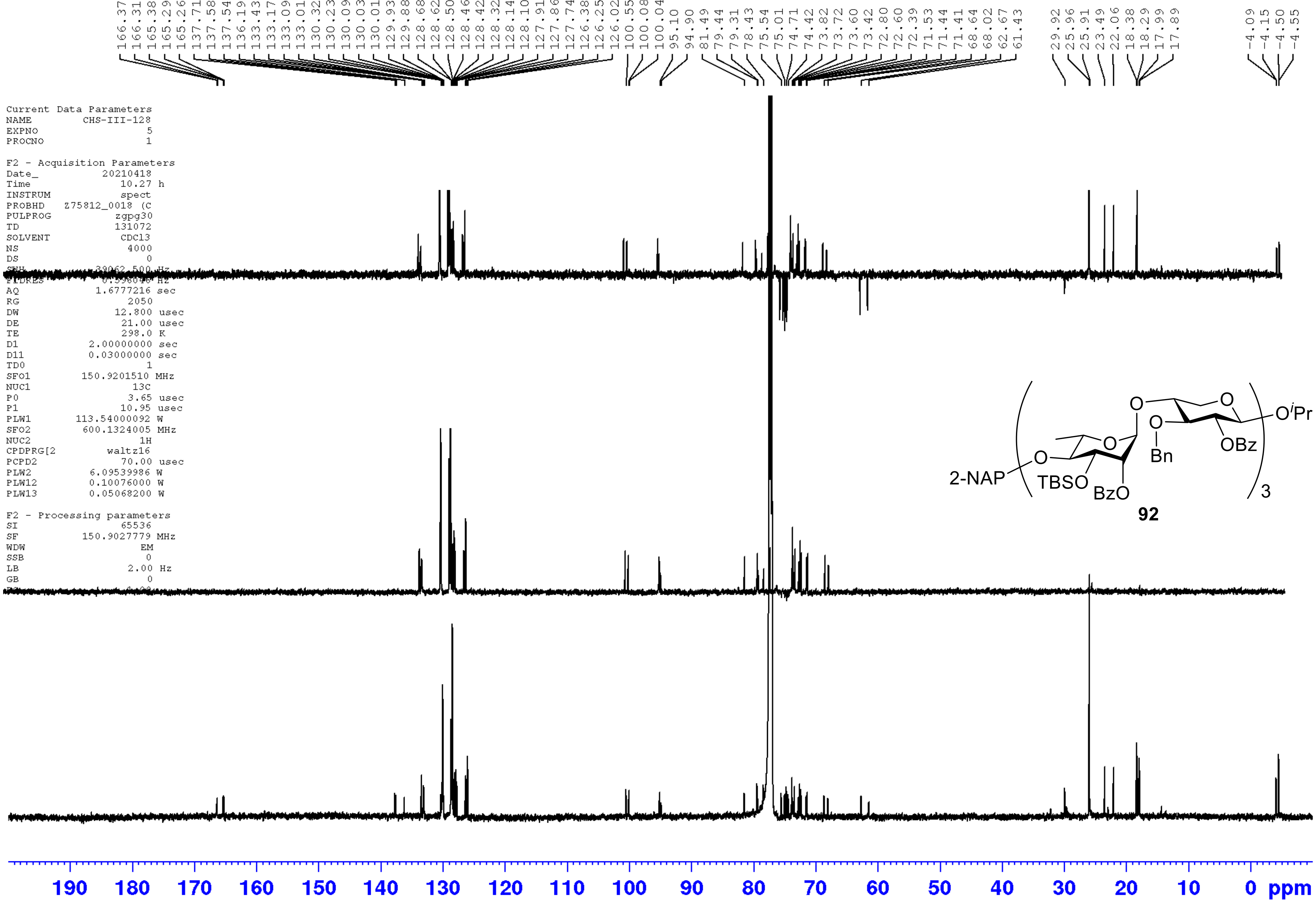
166.37 166.31 165.38 165.29 165.26 137.71 137.58 137.54 136.19 133.43 133.17 133.09 133.01 130.32 130.23 130.09 130.03 130.01 129.93 129.88 128.68 128.62 128.50 128.46 128.42 128.32 128.14 128.10 127.91 127.86 127.74 126.38 126.25 126.02 100.55 100.08 100.04 95.10 94.90 81.49 79.44 79.31 78.43 75.54 75.01 74.71 74.42 73.82 73.72 73.60 73.42 72.80 72.60 72.39 71.53 71.44 71.41 68.64 68.02 62.67 61.43 29.92 25.96 25.91 23.49 22.06 18.38 18.29 17.99 17.89 -4.09 -4.15 -4.50 -4.55
Current Data Parameters
NAME CHS-III-128
EXPNO 5
PROCNO 1
F2 - Acquisition Parameters
Date_ 20210418
Time 10.27 h
INSTRUM spect
PROBHD Z75812_0018 (C
PULPROG zgpg30
TD 131072
SOLVENT CDCl3
NS 4000
DS 0
SWH 39062.500 Hz
FIDRES 0.596046 Hz
AQ 1.6777216 sec
RG 2050
DW 12.800 usec
DE 21.00 usec
TE 298.0 K
D1 2.00000000 sec
D11 0.03000000 sec
TD0 1
SFO1 150.9201510 MHz
NUC1 13C
P0 3.65 usec
P1 10.95 usec
PLW1 113.54000092 W
SFO2 600.1324005 MHz
NUC2 1H
CPDPRG[2 waltz16
PCPD2 70.00 usec
PLW2 6.09539986 W
PLW12 0.10076000 W
PLW13 0.05068200 W
F2 - Processing parameters
SI 65536
SF 150.9027779 MHz
WDW EM
SSB 0
LB 2.00 Hz
GB 0
2-NAP
TBSO
BzO
O
Bn
OBz
O^{i}Pr
3
92
190 180 170 160 150 140 130 120 110 100 90 80 70 60 50 40 30 20 10 0 ppm

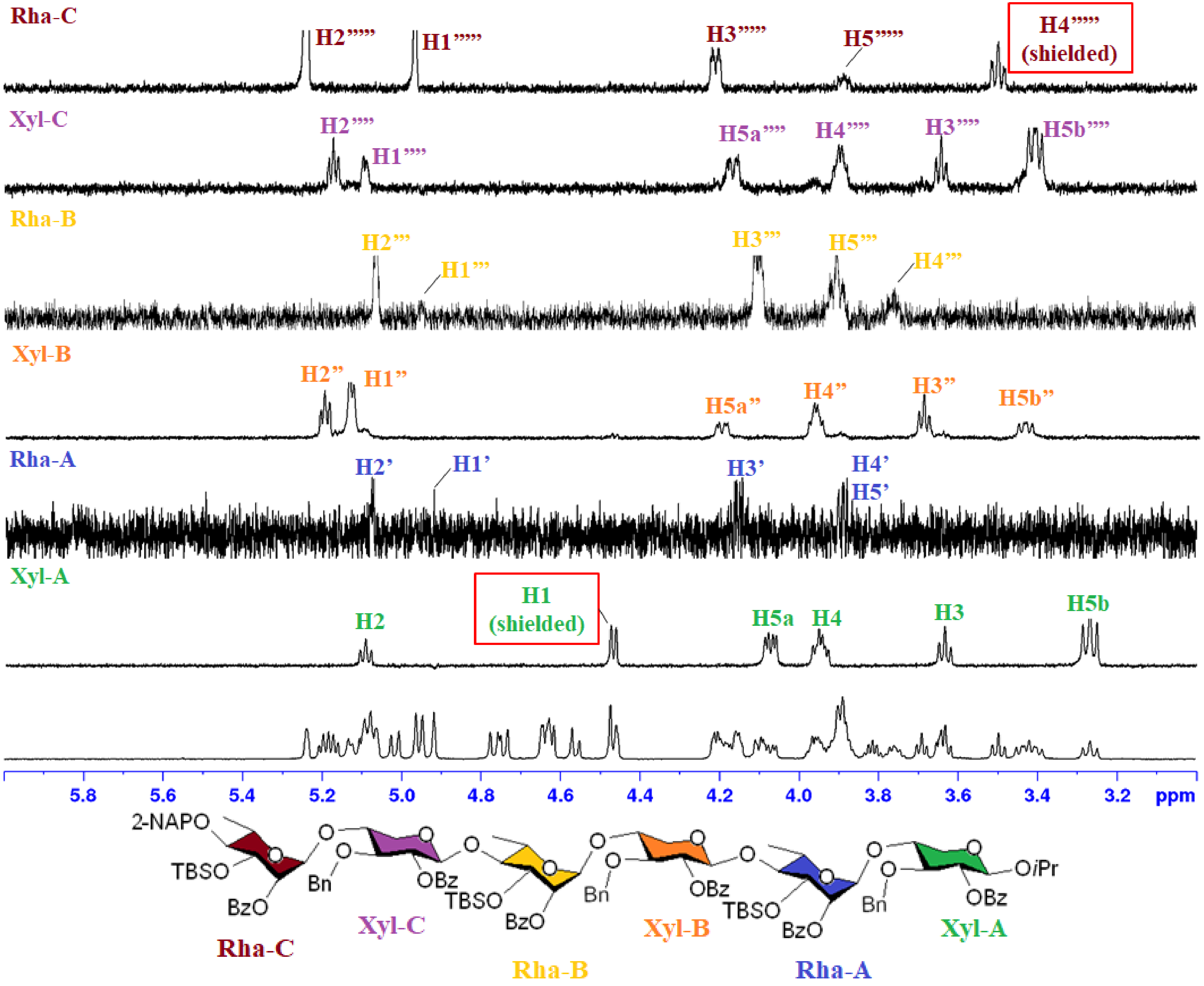
Rha-C
H2''''''
H1''''''
H3''''''
H5''''''
H4''''''
(shielded)
Xyl-C
H2'''''
H1'''''
H5a'''''
H4'''''
H3'''''
H5b'''''
Rha-B
H2''''
H1''''
H3''''
H5''''
H4''''
Xyl-B
H2''
H1''
H5a''
H4''
H3''
H5b''
Rha-A
H2'
H1'
H3'
H4'
H5'
Xyl-A
H2
H1
(shielded)
H5a
H4
H3
H5b
5.8
5.6
5.4
5.2
5.0
4.8
4.6
4.4
4.2
4.0
3.8
3.6
3.4
3.2
ppm
2-NAPO
TBSO
BzO
Bn
OBz
TBSO
BzO
Bn
OBz
TBSO
BzO
Bn
OBz
OiPr
Rha-C
Xyl-C
Rha-B
Xyl-B
Rha-A
Xyl-A

92

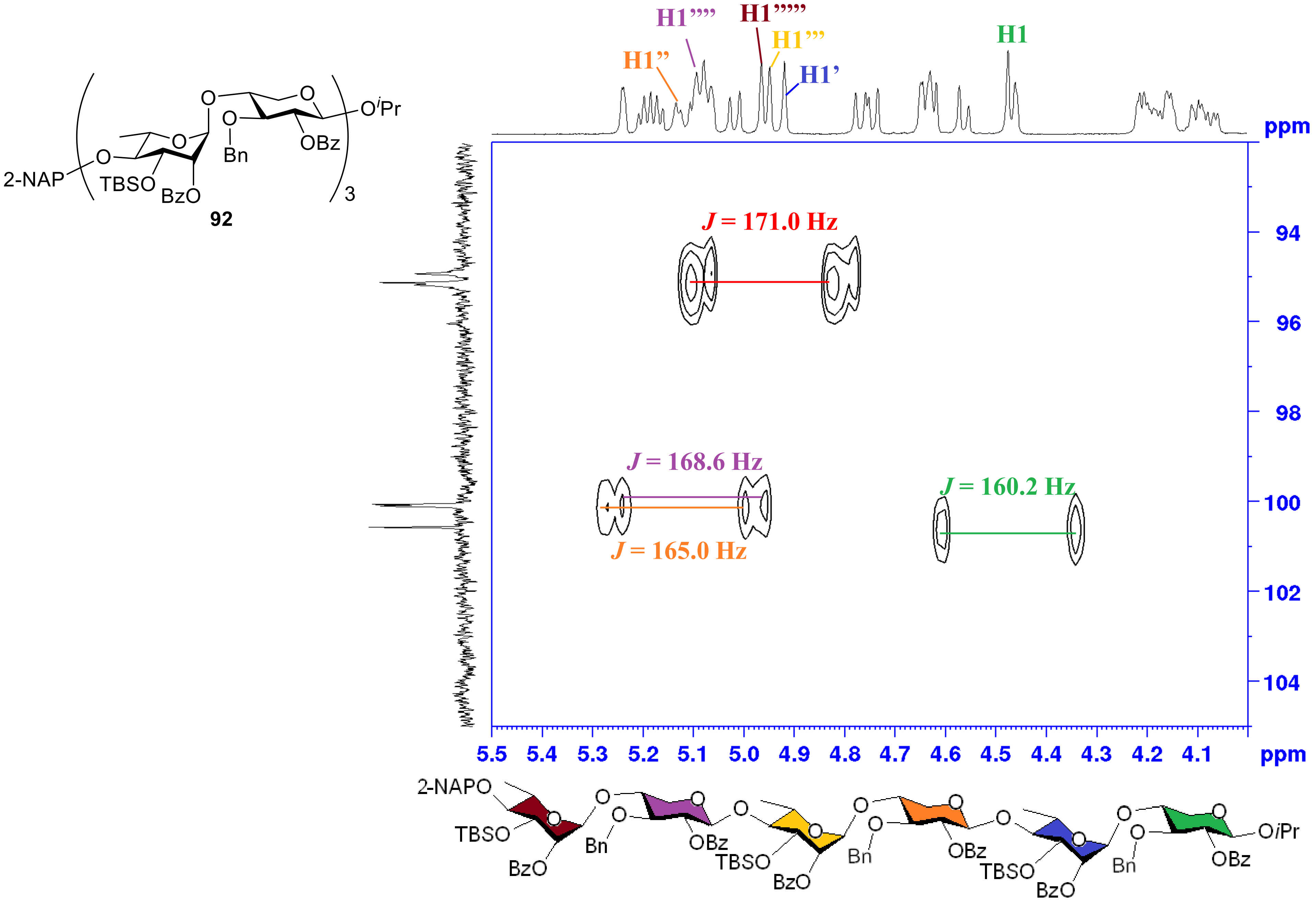

H1''''
H1'''''
H1'''
H1''
H1'
H1
ppm
J = 171.0 Hz
J = 168.6 Hz
J = 165.0 Hz
J = 160.2 Hz
94
96
98
100
102
104
5.5 5.4 5.3 5.2 5.1 5.0 4.9 4.8 4.7 4.6 4.5 4.4 4.3 4.2 4.1
2-NAP
TBSO
BzO
Bn
OBz
O^{i}Pr
2-NAPO
OiPr

92

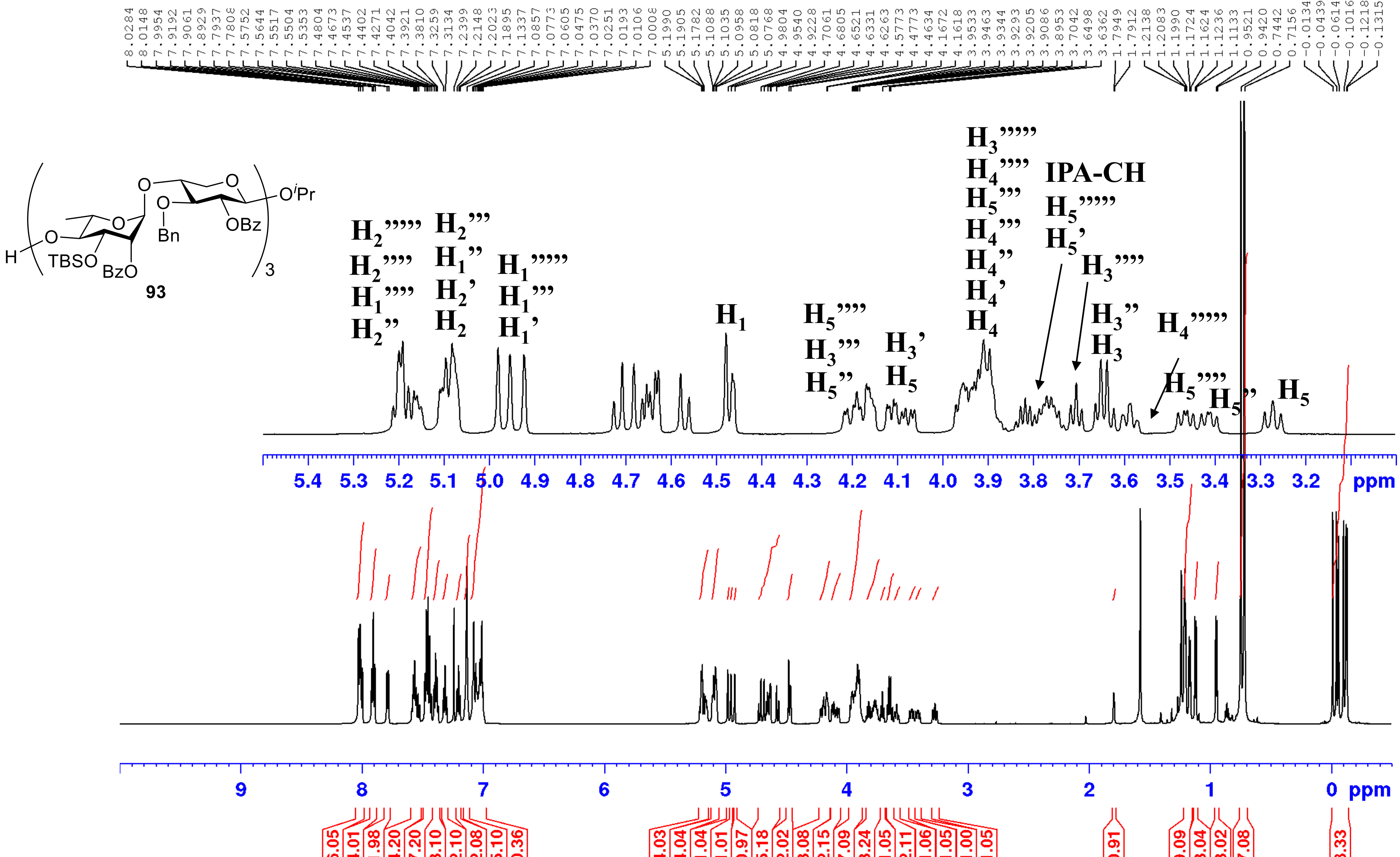
93
H2''''' H2'''' H1'''' H2''
H2''' H1'' H2' H2
H1''''' H1''' H1'
H1
H5'''' H3''' H5''
H3' H5
H3''''' H4'''' H5''' H4''' H4'' H4' H4
IPA-CH H5''''' H5'
H3''''
H3'' H3
H4'''''
H5''''
H5''
H5
ppm

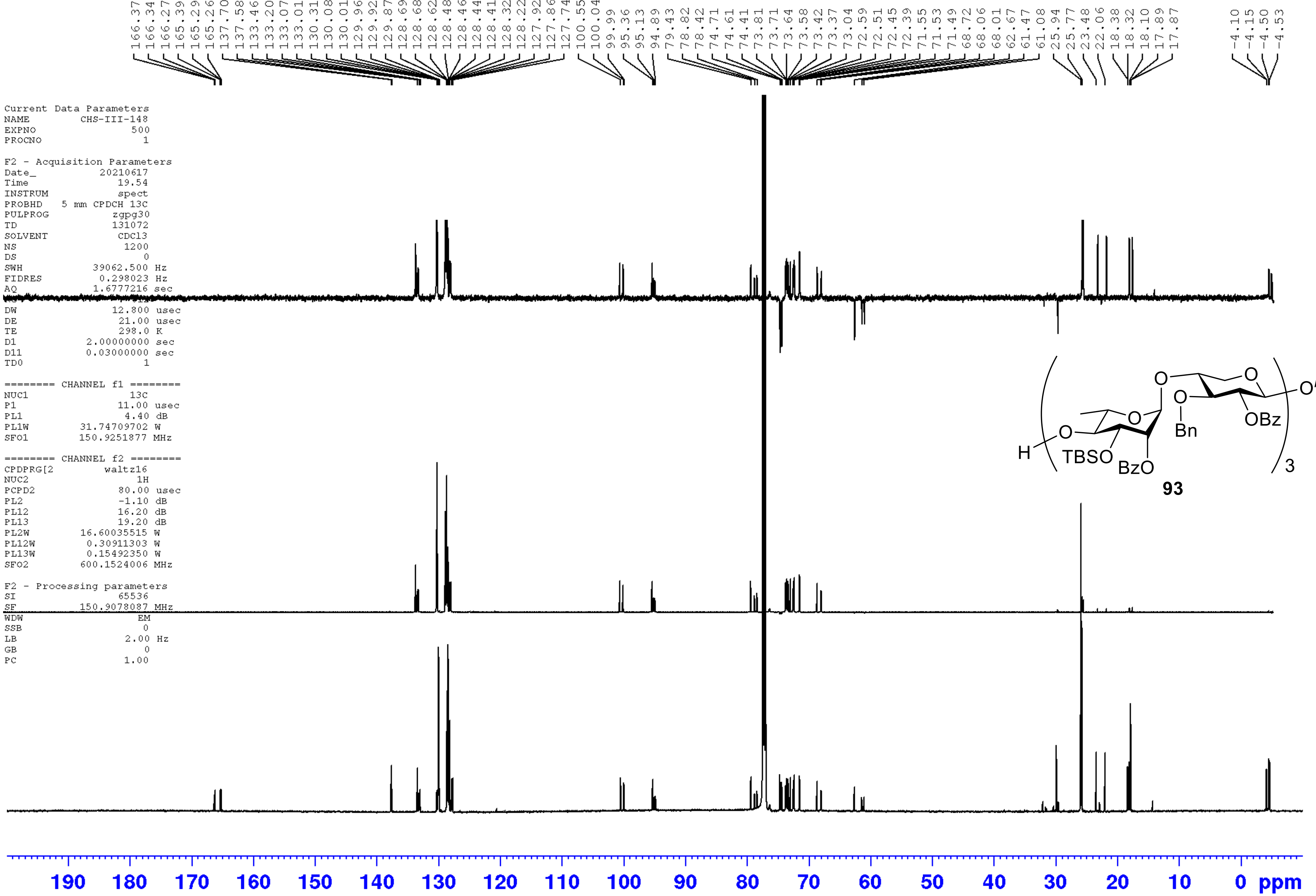
166.37
166.34
166.27
165.39
165.29
165.26
137.70
137.58
133.46
133.20
133.07
133.01
130.31
130.08
130.01
129.96
129.92
129.87
128.69
128.68
128.62
128.48
128.46
128.44
128.41
128.32
128.22
127.92
127.86
127.74
100.55
100.04
99.99
95.36
95.13
94.89
79.43
78.82
78.42
74.71
74.61
74.41
73.81
73.71
73.64
73.58
73.42
73.37
73.04
72.59
72.51
72.45
72.39
71.55
71.53
71.49
68.72
68.06
68.01
62.67
61.47
61.08
25.94
25.77
23.48
22.06
18.38
18.32
18.10
17.89
17.87
-4.10
-4.15
-4.50
-4.53
Current Data Parameters
NAME CHS-III-148
EXPNO 500
PROCNO 1
F2 - Acquisition Parameters
Date_ 20210617
Time 19.54
INSTRUM spect
PROBHD 5 mm CPDCH 13C
PULPROG zgpg30
TD 131072
SOLVENT CDCl3
NS 1200
DS 0
SWH 39062.500 Hz
FIDRES 0.298023 Hz
AQ 1.6777216 sec
DW 12.800 usec
DE 21.00 usec
TE 298.0 K
D1 2.00000000 sec
D11 0.03000000 sec
TD0 1
======== CHANNEL f1 ========
NUC1 13C
P1 11.00 usec
PL1 4.40 dB
PL1W 31.74709702 W
SFO1 150.9251877 MHz
======== CHANNEL f2 ========
CPDPRG[2 waltz16
NUC2 1H
PCPD2 80.00 usec
PL2 -1.10 dB
PL12 16.20 dB
PL13 19.20 dB
PL2W 16.60035515 W
PL12W 0.30911303 W
PL13W 0.15492350 W
SFO2 600.1524006 MHz
F2 - Processing parameters
SI 65536
SF 150.9078087 MHz
WDW EM
SSB 0
LB 2.00 Hz
GB 0
PC 1.00
O
O
O
O
O
OiPr
Bn
OBz
H
TBSO
BzO
3
93
190 180 170 160 150 140 130 120 110 100 90 80 70 60 50 40 30 20 10 0 ppm

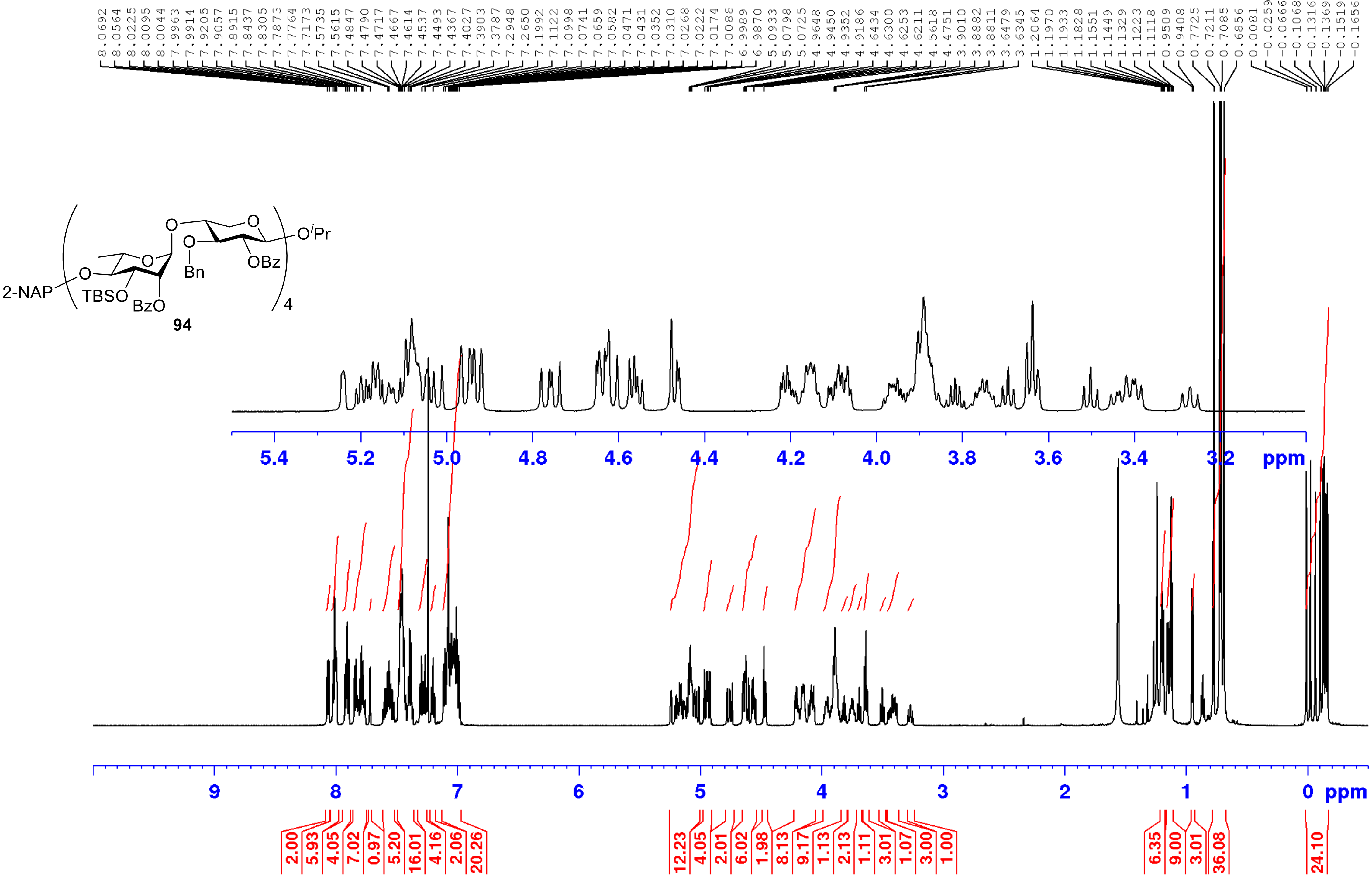

O
O
O
OiPr
O
Bn
OBz
2-NAP
O
TBSO
BzO
4
94
5.4
5.2
5.0
4.8
4.6
4.4
4.2
4.0
3.8
3.6
3.4
3.2
ppm
9
8
7
6
5
4
3
2
1
0
ppm
2.00
5.93
4.05
7.02
0.97
5.20
16.01
4.16
2.06
20.26
12.23
4.05
2.01
6.02
1.98
8.13
9.17
1.13
2.13
1.11
3.01
1.07
3.00
1.00
6.35
9.00
3.01
36.08
24.10

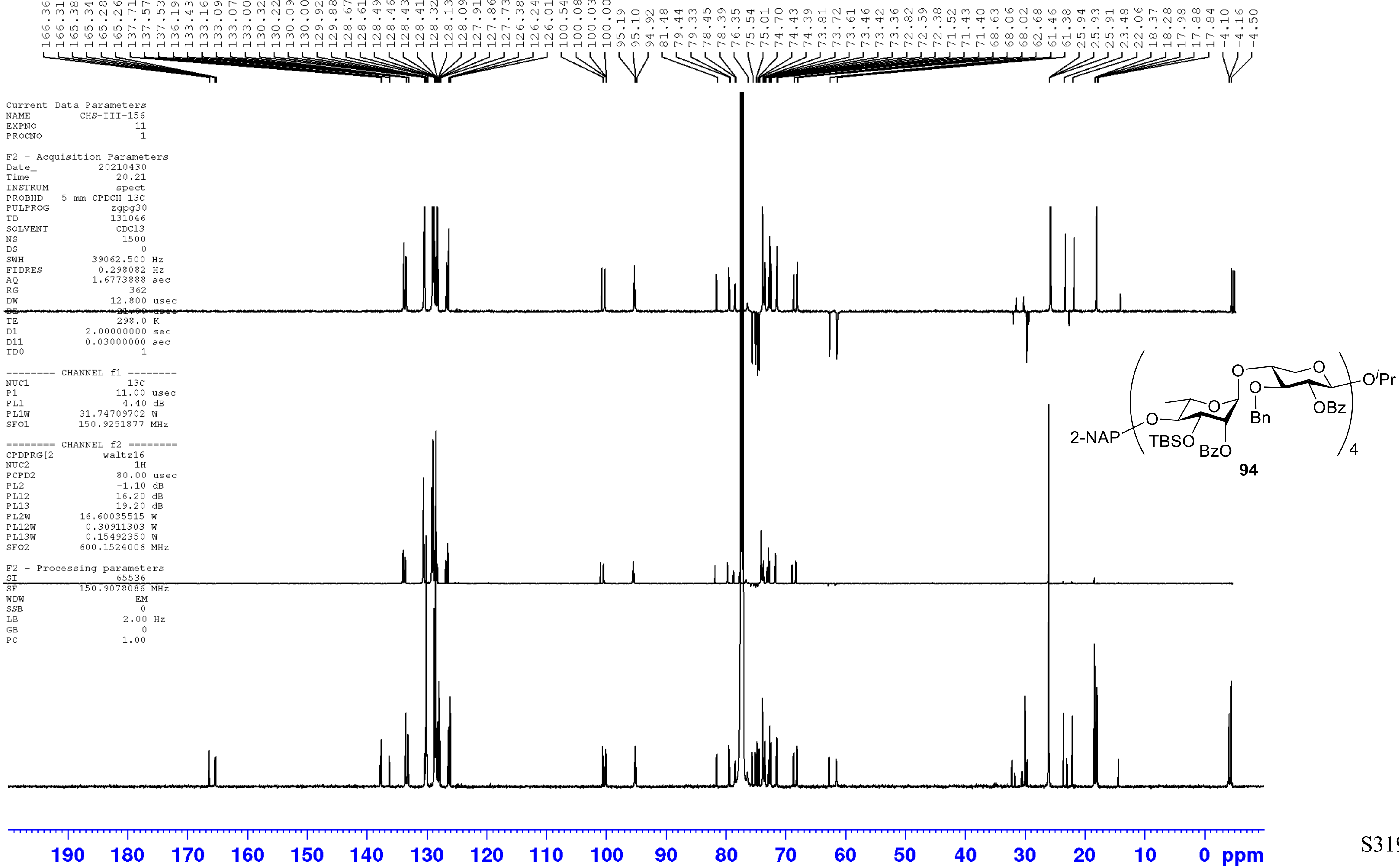
166.36 166.31 165.38 165.34 165.28 165.26 137.71 137.57 137.53 136.19 133.43 133.16 133.09 133.07 133.00 130.32 130.22 130.09 130.00 129.92 129.88 128.67 128.61 128.49 128.46 128.43 128.41 128.32 128.13 128.09 127.91 127.86 127.73 126.38 126.24 126.01 100.54 100.08 100.03 100.00 95.19 95.10 94.92 81.48 79.44 79.33 78.45 78.39 76.35 75.54 75.01 74.70 74.43 74.39 73.81 73.72 73.61 73.46 73.42 73.36 72.82 72.59 72.38 71.52 71.43 71.40 68.63 68.06 68.02 62.68 61.46 61.38 25.94 25.93 25.91 23.48 22.06 18.37 18.28 17.98 17.88 17.84 -4.10 -4.16 -4.50
Current Data Parameters
NAME CHS-III-156
EXPNO 11
PROCNO 1
F2 - Acquisition Parameters
Date_ 20210430
Time 20.21
INSTRUM spect
PROBHD 5 mm CPDCH 13C
PULPROG zgpg30
TD 131046
SOLVENT CDCl3
NS 1500
DS 0
SWH 39062.500 Hz
FIDRES 0.298082 Hz
AQ 1.6773888 sec
RG 362
DW 12.800 usec
TE 298.0 K
D1 2.00000000 sec
D11 0.03000000 sec
TD0 1
======== CHANNEL f1 ========
NUC1 13C
P1 11.00 usec
PL1 4.40 dB
PL1W 31.74709702 W
SFO1 150.9251877 MHz
======== CHANNEL f2 ========
CPDPRG[2 waltz16
NUC2 1H
PCPD2 80.00 usec
PL2 -1.10 dB
PL12 16.20 dB
PL13 19.20 dB
PL2W 16.60035515 W
PL12W 0.30911303 W
PL13W 0.15492350 W
SFO2 600.1524006 MHz
F2 - Processing parameters
SI 65536
SF 150.9078086 MHz
WDW EM
SSB 0
LB 2.00 Hz
GB 0
PC 1.00
190 180 170 160 150 140 130 120 110 100 90 80 70 60 50 40 30 20 10 0 ppm
O^{i}Pr
2-NAP
TBSO
BzO
Bn
OBz
4

94

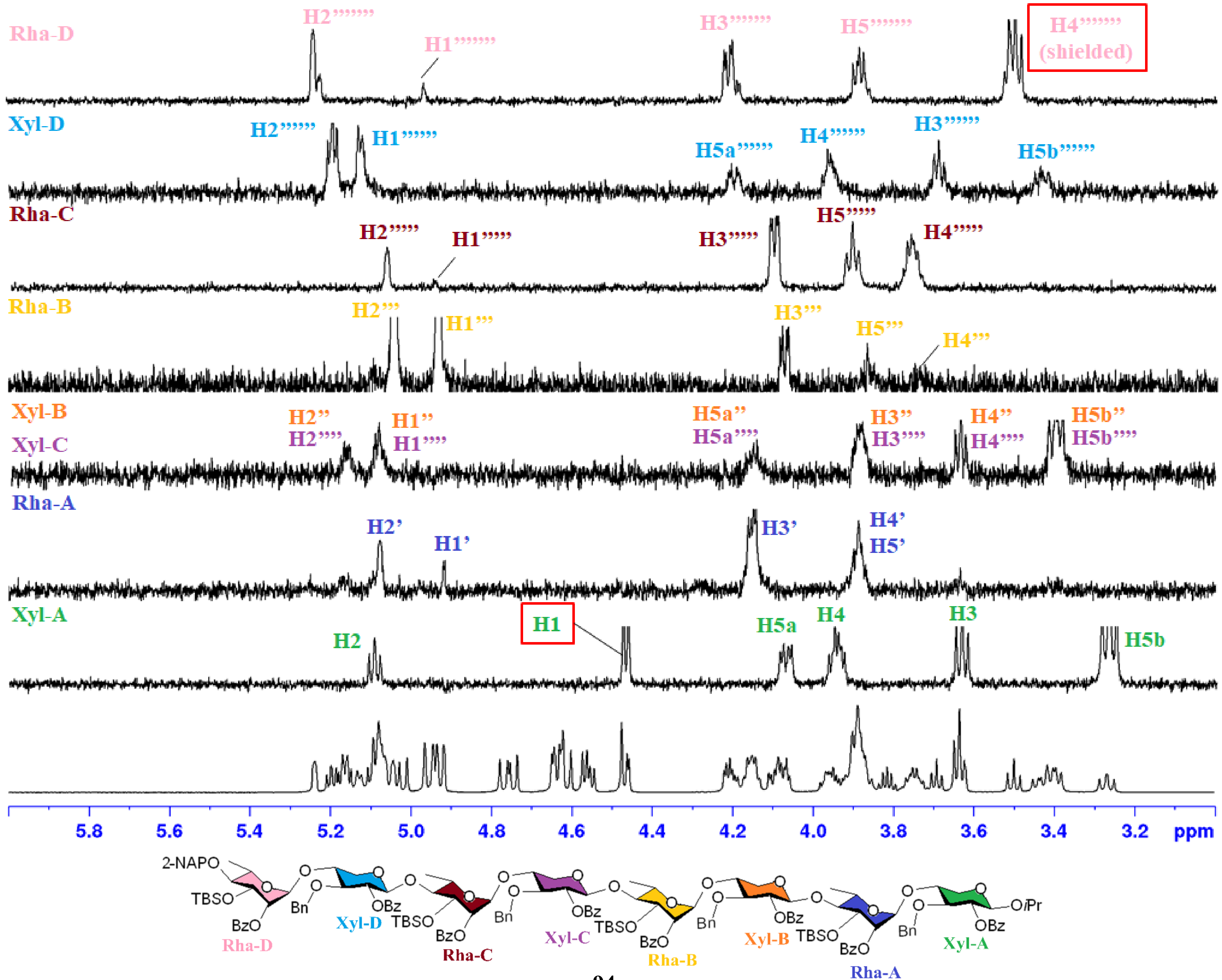

Rha-D
H2'''''''
H1'''''''
H3'''''''
H5'''''''
H4'''''''
(shielded)
Xyl-D
H2''''''
H1''''''
H5a''''''
H4''''''
H3''''''
H5b''''''
Rha-C
H2'''''
H1'''''
H3'''''
H5'''''
H4'''''
Rha-B
H2'''
H1'''
H3'''
H5'''
H4'''
Xyl-B
Xyl-C
H2''
H2''''
H1''
H1''''
H5a''
H5a''''
H3''
H3''''
H4''
H4''''
H5b''
H5b''''
Rha-A
H2'
H1'
H3'
H4'
H5'
Xyl-A
H2
H1
H5a
H4
H3
H5b
5.8
5.6
5.4
5.2
5.0
4.8
4.6
4.4
4.2
4.0
3.8
3.6
3.4
3.2
ppm
2-NAPO
TBSO
BzO
Rha-D
Bn
OBz
Xyl-D
TBSO
BzO
Rha-C
Bn
OBz
Xyl-C
TBSO
BzO
Rha-B
Bn
OBz
Xyl-B
TBSO
BzO
Rha-A
Bn
OBz
Xyl-A
OiPr

94

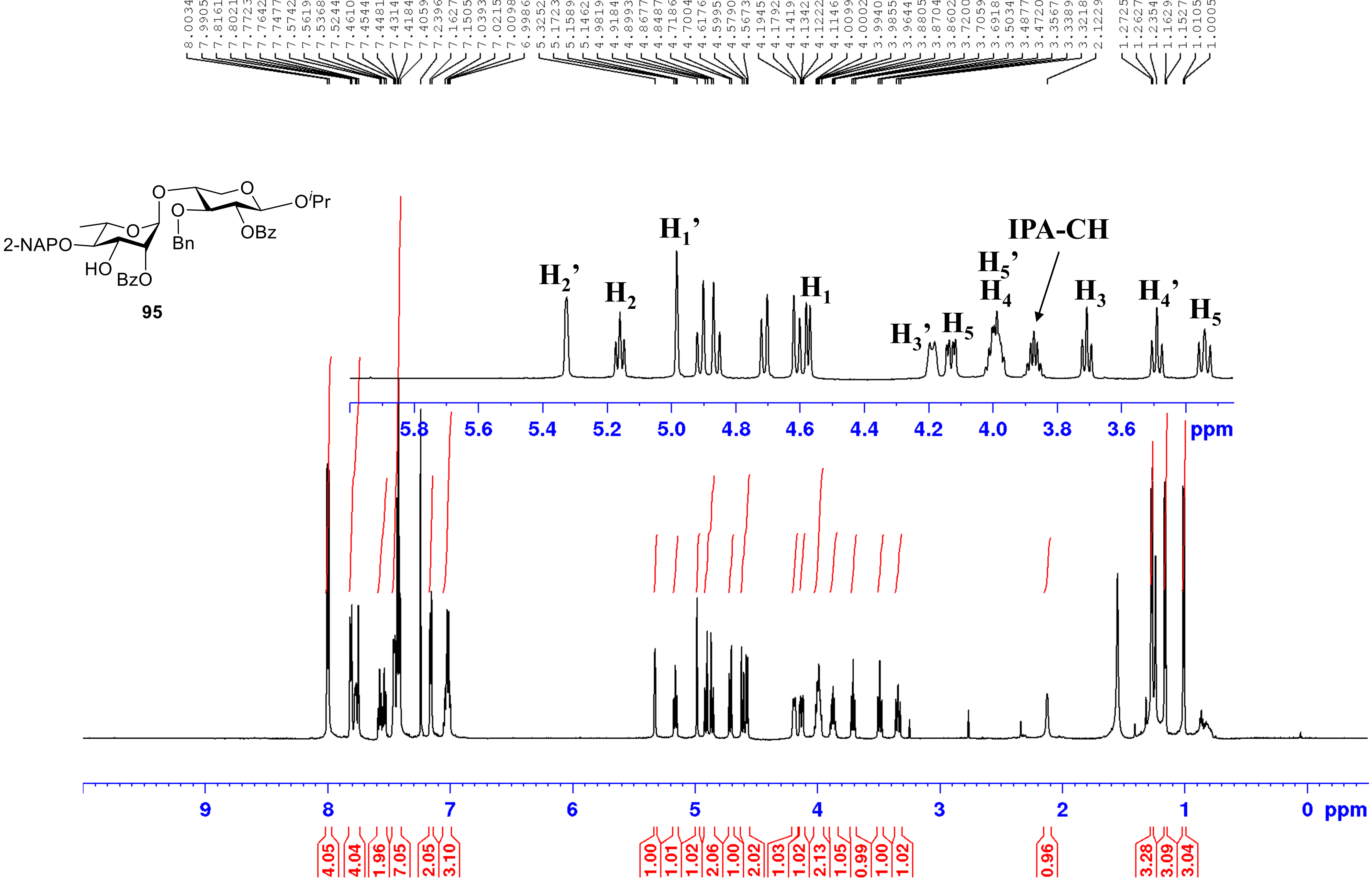

2-NAPO
HO
BzO
O
Bn
OBz
O^{i}Pr
95
H1'
H2'
H2
H1
H3'
H5
H5'
H4
IPA-CH
H3
H4'
H5
ppm

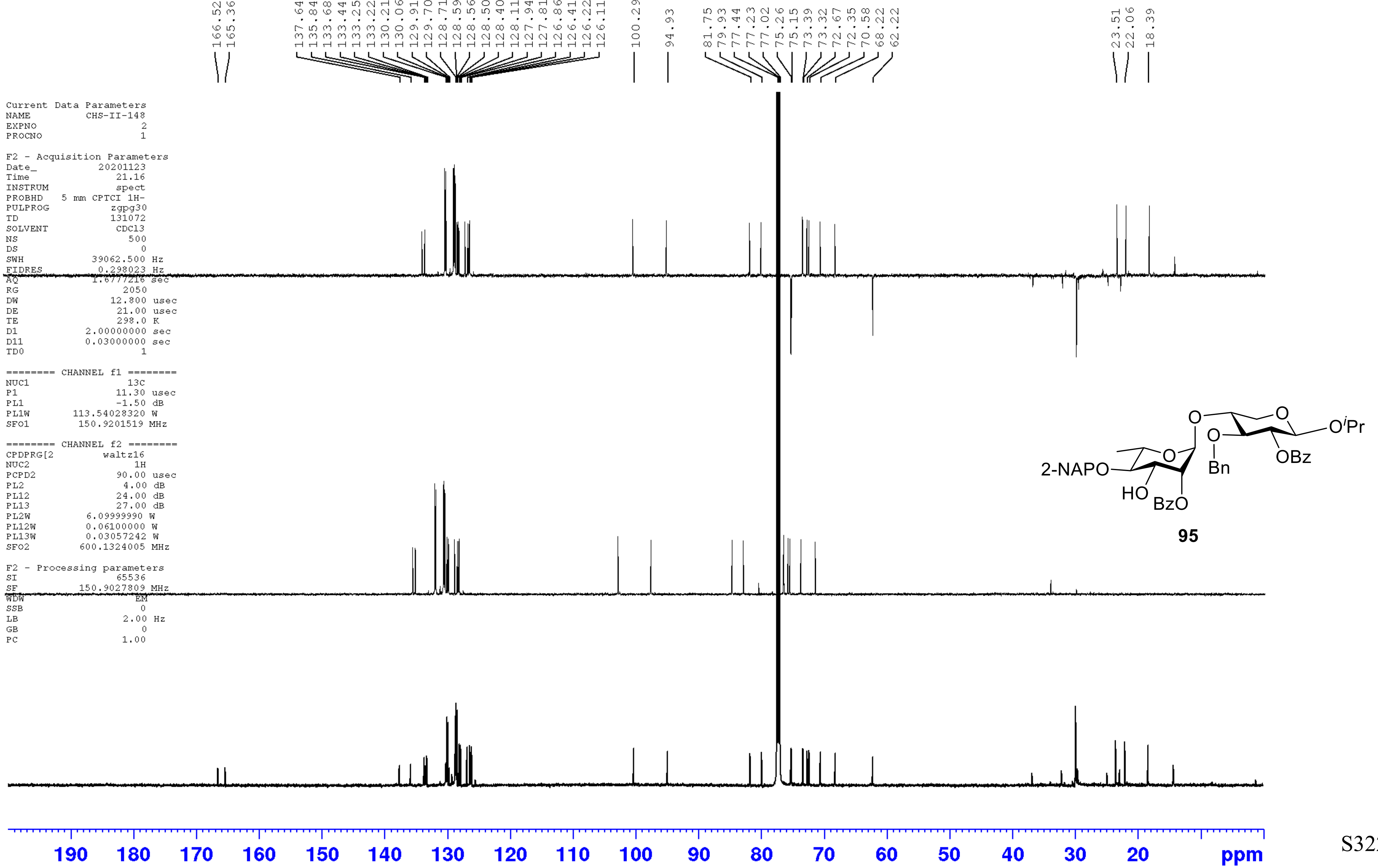
166.52
165.36
137.64
135.84
133.68
133.44
133.25
133.22
130.21
130.06
129.91
129.70
128.71
128.59
128.56
128.50
128.40
128.11
127.94
127.81
126.86
126.41
126.22
126.11
100.29
94.93
81.75
79.93
77.44
77.23
77.02
75.26
75.15
73.39
73.32
72.67
72.35
70.58
68.22
62.22
23.51
22.06
18.39
Current Data Parameters
NAME CHS-II-148
EXPNO 2
PROCNO 1
F2 - Acquisition Parameters
Date_ 20201123
Time 21.16
INSTRUM spect
PROBHD 5 mm CPTCI 1H-
PULPROG zgpg30
TD 131072
SOLVENT CDCl3
NS 500
DS 0
SWH 39062.500 Hz
FIDRES 0.298023 Hz
AQ 1.6777216 sec
RG 2050
DW 12.800 usec
DE 21.00 usec
TE 298.0 K
D1 2.00000000 sec
D11 0.03000000 sec
TD0 1
======== CHANNEL f1 ========
NUC1 13C
P1 11.30 usec
PL1 -1.50 dB
PL1W 113.54028320 W
SFO1 150.9201519 MHz
======== CHANNEL f2 ========
CPDPRG[2 waltz16
NUC2 1H
PCPD2 90.00 usec
PL2 4.00 dB
PL12 24.00 dB
PL13 27.00 dB
PL2W 6.09999990 W
PL12W 0.06100000 W
PL13W 0.03057242 W
SFO2 600.1324005 MHz
F2 - Processing parameters
SI 65536
SF 150.9027809 MHz
WDW EM
SSB 0
LB 2.00 Hz
GB 0
PC 1.00
190
180
170
160
150
140
130
120
110
100
90
80
70
60
50
40
30
20
ppm
2-NAPO
HO
BzO
O
O
Bn
OBz
O^{i}Pr

95

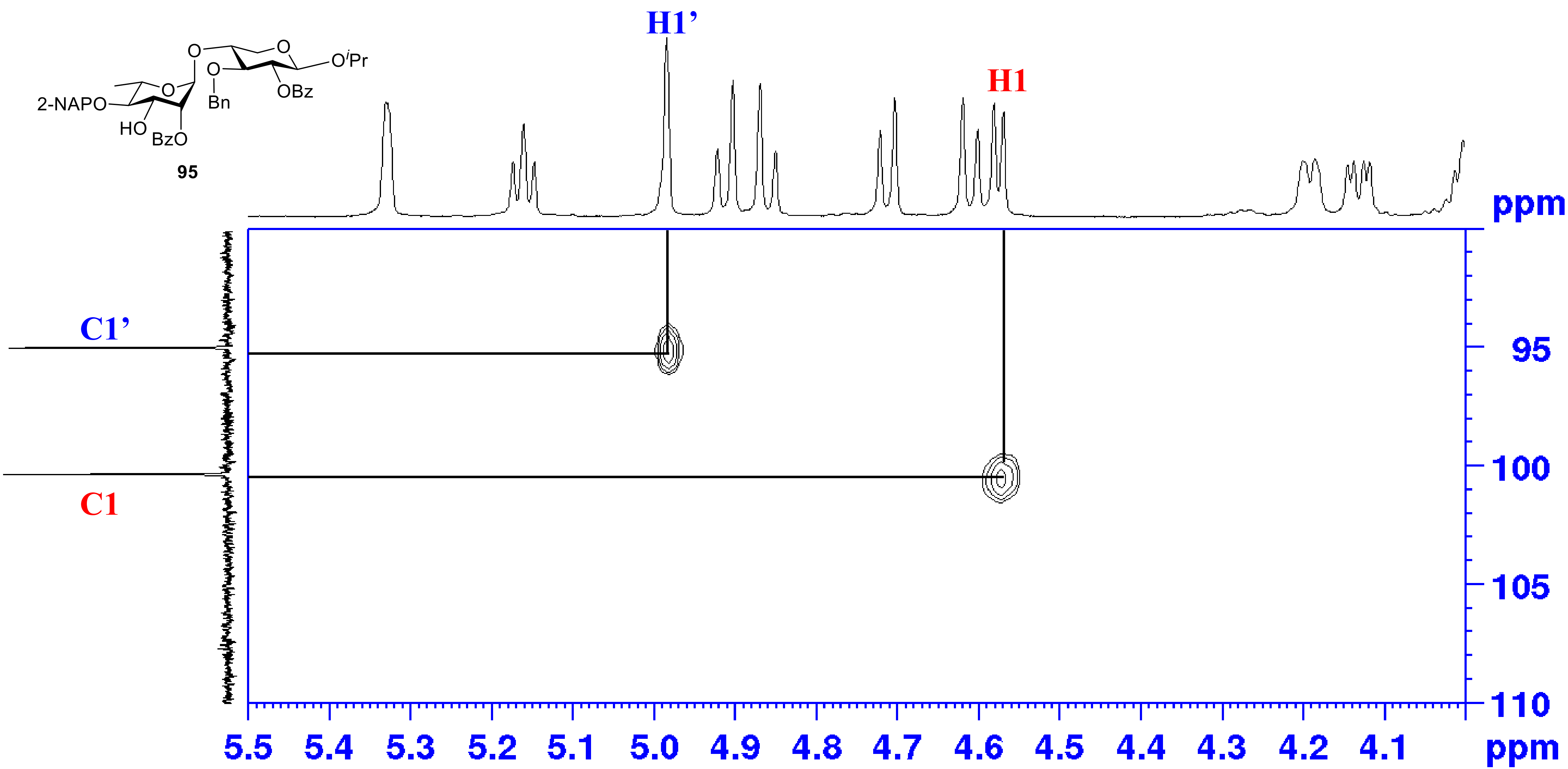

2-NAPO
HO
BzO
O
O
O
Bn
O
OBz
O^{i}Pr
95
H1'
H1
C1'
C1
ppm
95
100
105
110
5.5
5.4
5.3
5.2
5.1
5.0
4.9
4.8
4.7
4.6
4.5
4.4
4.3
4.2
4.1
ppm

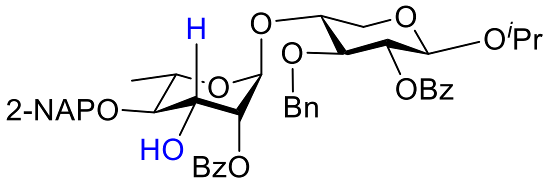
H
O
O
O
O^{i}Pr
2-NAPO
HO
BzO
Bn
OBz

95

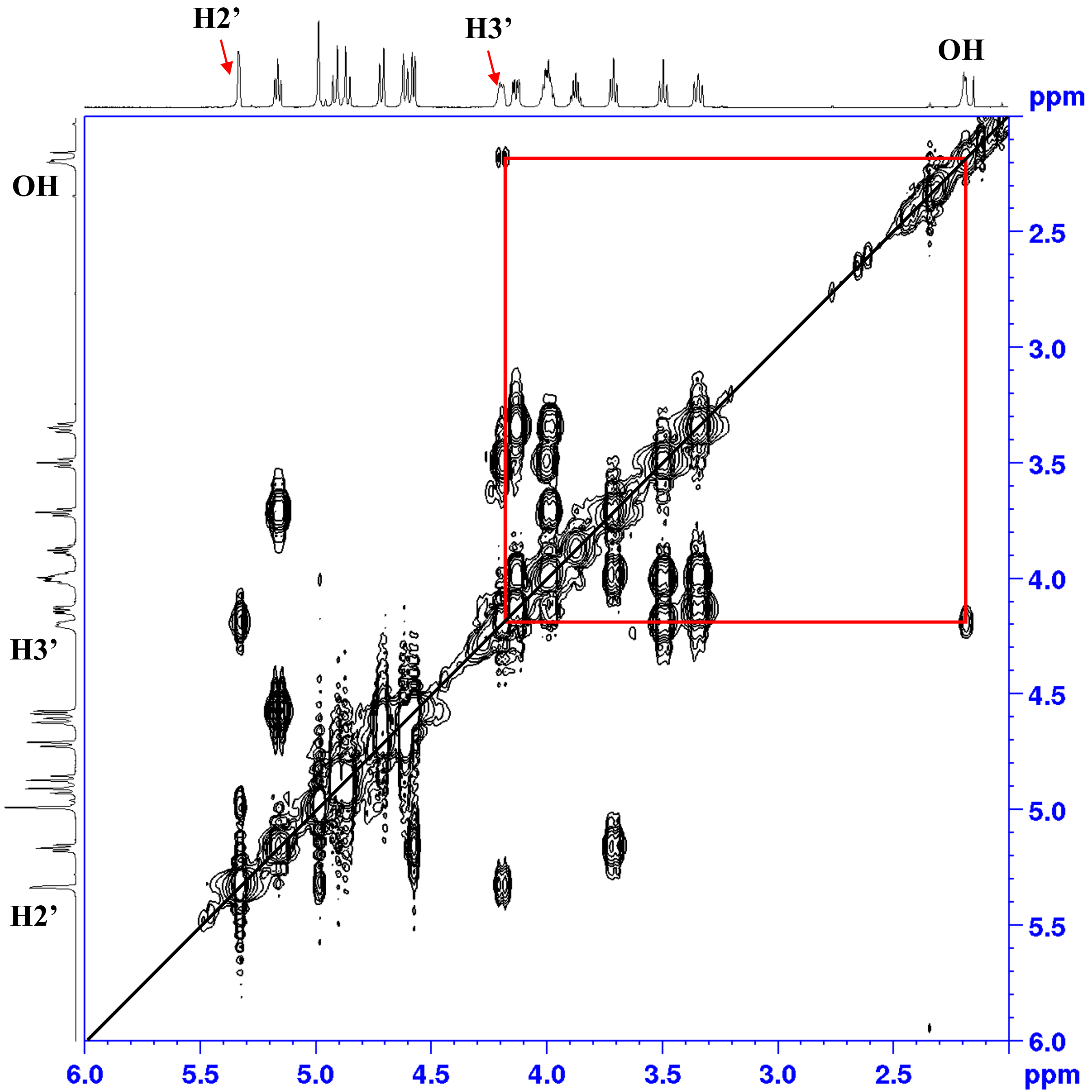
H2'
H3'
OH
ppm
OH
H3'
H2'
2.5
3.0
3.5
4.0
4.5
5.0
5.5
6.0
6.0
5.5
5.0
4.5
4.0
3.5
3.0
2.5
ppm

Current Data Parameters
NAME CHS-III-72
EXPNO 3
PROCNO 1

F2 - Acquisition Parameters
Date_ 20210305
Time 18.44 h
INSTRUM spect
PROBHD Z75812_0018 (C
PULPROG zg30
TD 32768
SOLVENT CDCl3
NS 8
DS 0
SWH 8370.536 Hz
FIDRES 0.510897 Hz
AQ 1.9573419 sec
RG 22.6
DW 59.733 usec
DE 21.00 usec
TE 298.0 K
D1 2.00000000 sec
TD0 1
SFO1 600.1336009 MHz
NUC1 1H
P0 2.78 usec
P1 8.35 usec
PLW1 6.09539986 W

F2 - Processing parameters
SI 16384
SF 600.1300269 MHz
WDW EM
SSB 0
LB 0 Hz
GB 0
PC 1.00

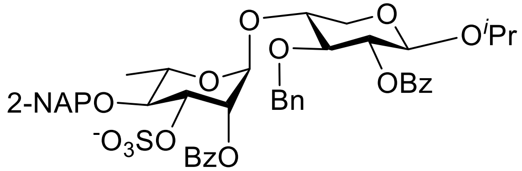

99

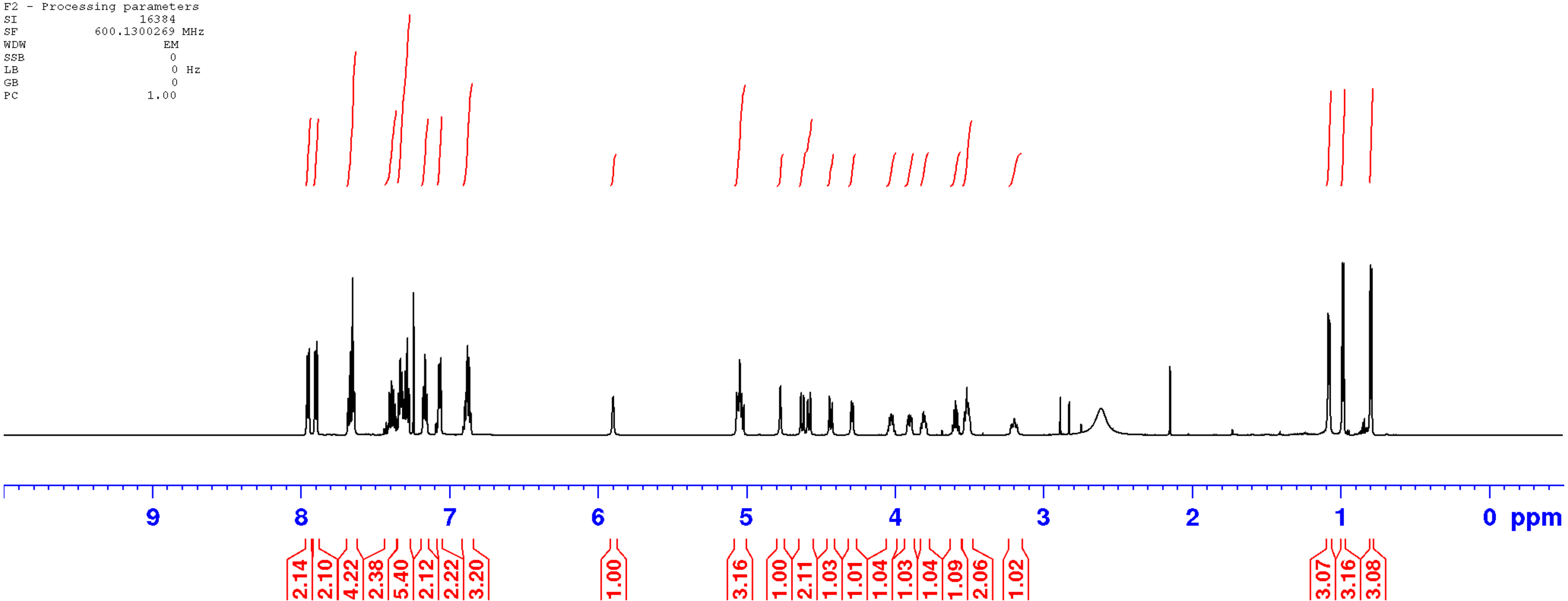

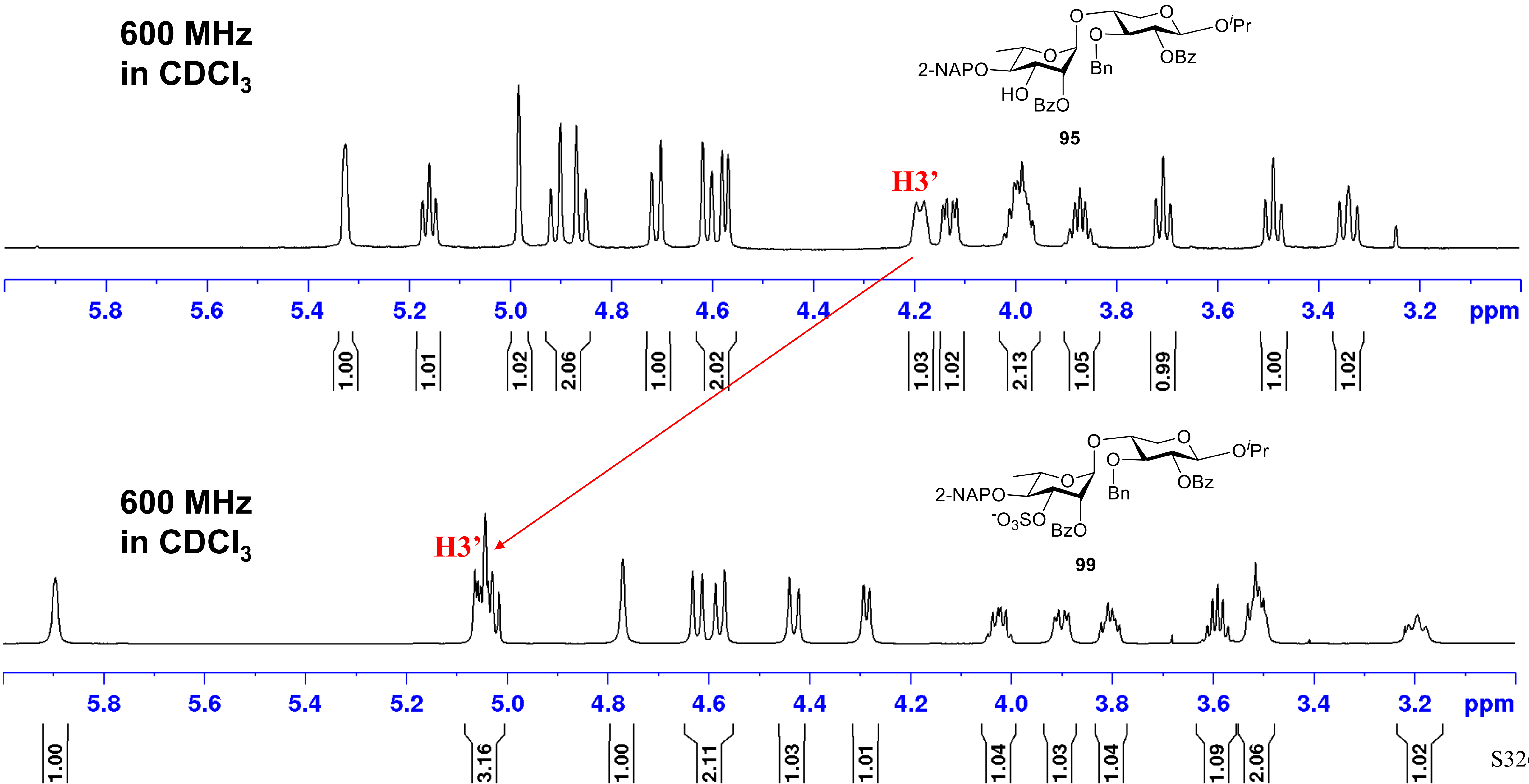

600 MHz
in CDCl3
2-NAPO
HO
BzO
O
Bn
OBz
O^iPr
95
H3'
5.8
5.6
5.4
5.2
5.0
4.8
4.6
4.4
4.2
4.0
3.8
3.6
3.4
3.2
ppm
1.00
1.01
1.02
2.06
1.00
2.02
1.03
1.02
2.13
1.05
0.99
1.00
1.02
600 MHz
in CDCl3
2-NAPO
-O3SO
BzO
Bn
OBz
O^iPr
99
H3'
1.00
3.16
1.00
2.11
1.03
1.01
1.04
1.03
1.04
1.09
2.06
1.02

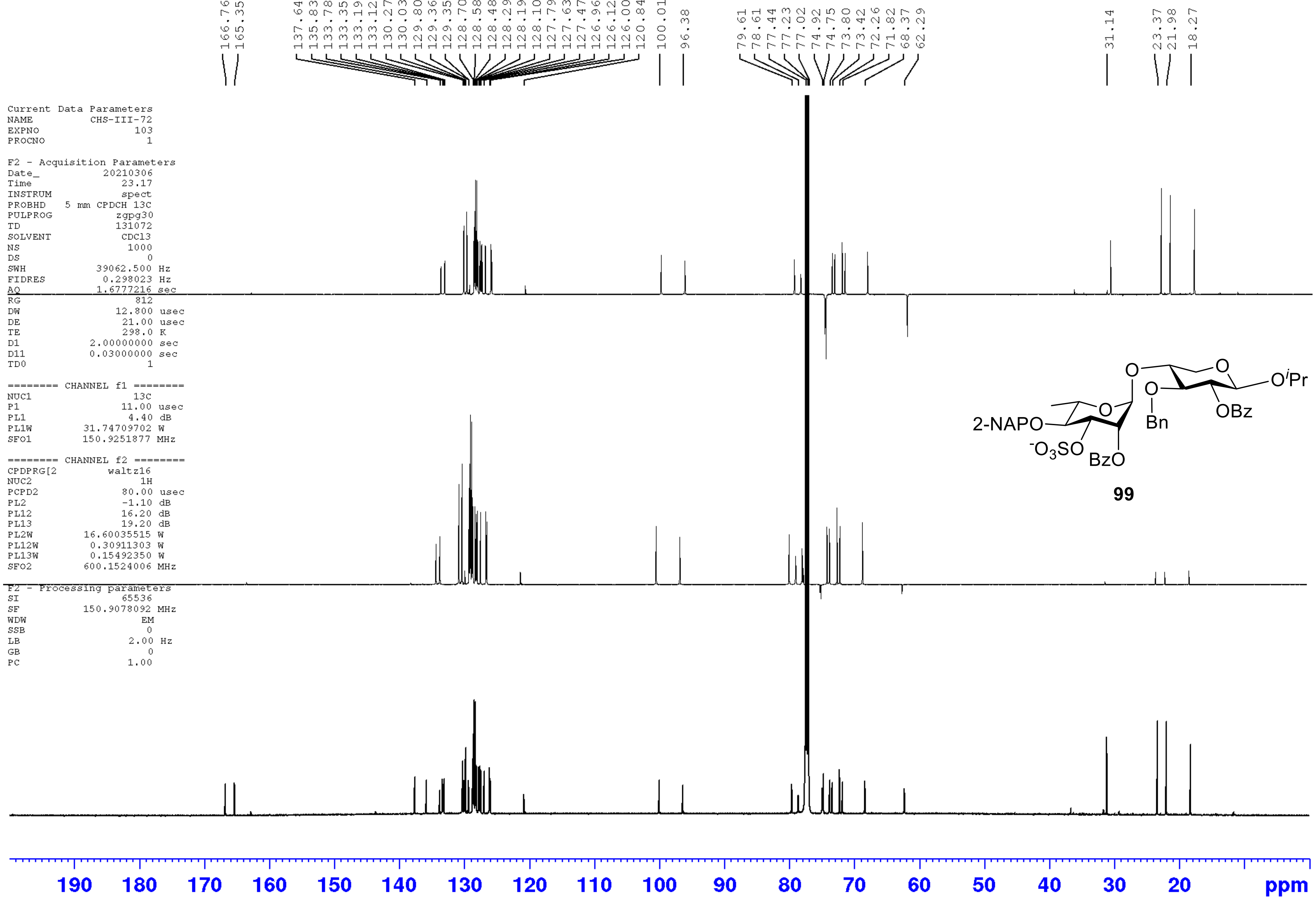
166.76
165.35
137.64
135.83
133.78
133.35
133.19
133.12
130.27
130.03
129.80
129.36
129.35
128.70
128.58
128.48
128.29
128.19
128.10
127.79
127.63
127.47
126.96
126.12
126.00
120.84
100.01
96.38
79.61
78.61
77.44
77.23
77.02
74.92
74.75
73.80
73.42
72.26
71.82
68.37
62.29
31.14
23.37
21.98
18.27
Current Data Parameters
NAME CHS-III-72
EXPNO 103
PROCNO 1
F2 - Acquisition Parameters
Date_ 20210306
Time 23.17
INSTRUM spect
PROBHD 5 mm CPDCH 13C
PULPROG zgpg30
TD 131072
SOLVENT CDCl3
NS 1000
DS 0
SWH 39062.500 Hz
FIDRES 0.298023 Hz
AQ 1.6777216 sec
RG 812
DW 12.800 usec
DE 21.00 usec
TE 298.0 K
D1 2.00000000 sec
D11 0.03000000 sec
TD0 1
======== CHANNEL f1 ========
NUC1 13C
P1 11.00 usec
PL1 4.40 dB
PL1W 31.74709702 W
SFO1 150.9251877 MHz
======== CHANNEL f2 ========
CPDPRG[2 waltz16
NUC2 1H
PCPD2 80.00 usec
PL2 -1.10 dB
PL12 16.20 dB
PL13 19.20 dB
PL2W 16.60035515 W
PL12W 0.30911303 W
PL13W 0.15492350 W
SFO2 600.1524006 MHz
F2 - Processing parameters
SI 65536
SF 150.9078092 MHz
WDW EM
SSB 0
LB 2.00 Hz
GB 0
PC 1.00
O
O
O
O^{i}Pr
O
Bn
OBz
2-NAPO
O
^-O_3SO
BzO
99
190
180
170
160
150
140
130
120
110
100
90
80
70
60
50
40
30
20
ppm

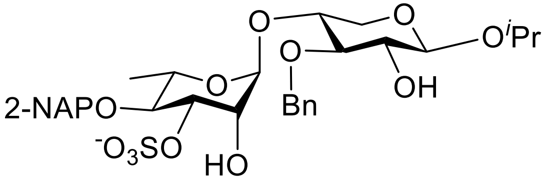
O
O
OⁱPr
O
O
2-NAPO
Bn
OH
-O3SO
HO

103

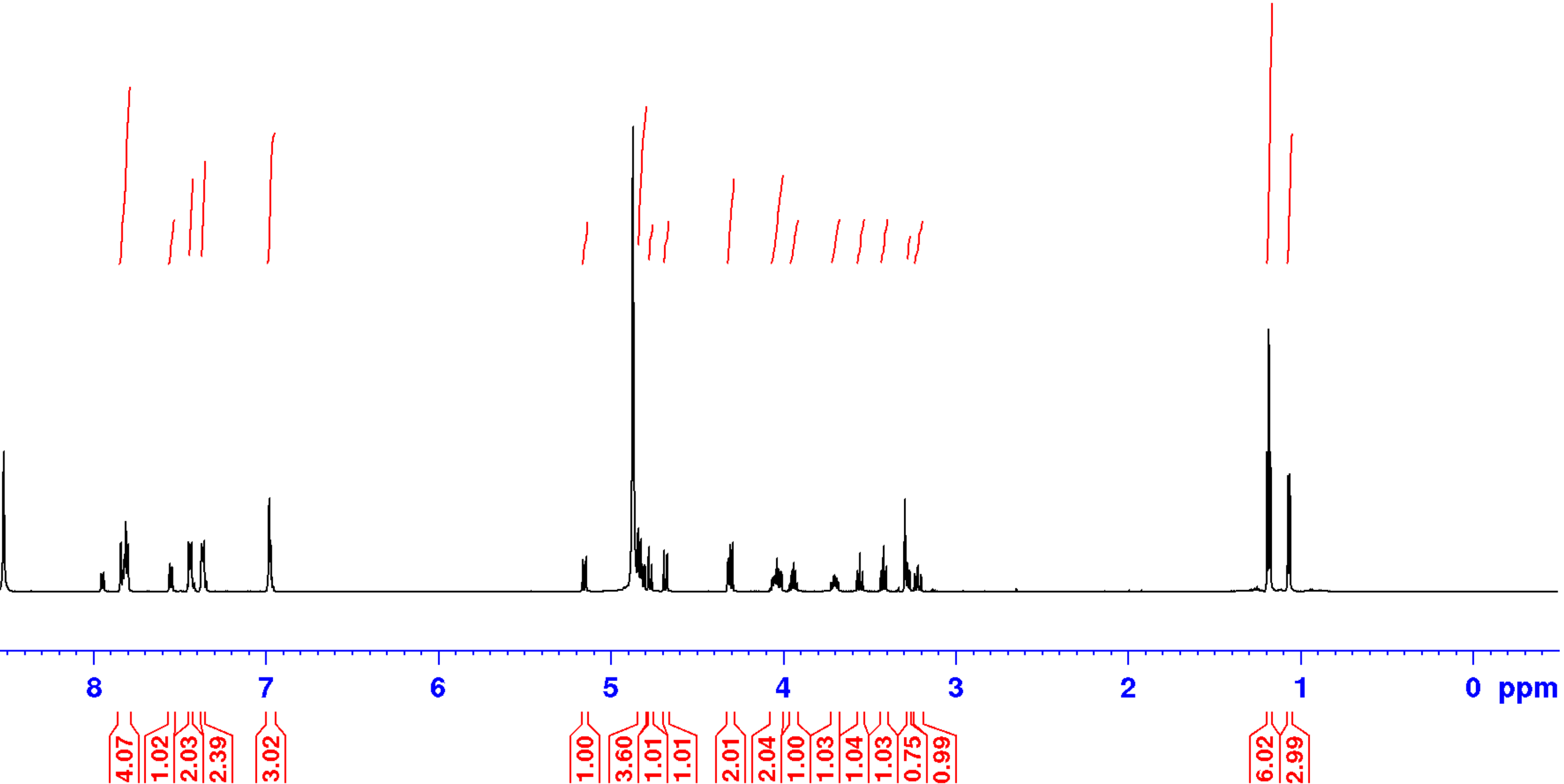
Current Data Parameters
NAME CHS-III-80
EXPNO 3
PROCNO 1
F2 - Acquisition Parameters
Date_ 20210305
Time 14.03
INSTRUM spect
PROBHD 5 mm CPDCH 13C
PULPROG zg30
TD 32768
SOLVENT MeOD
NS 4
DS 0
SWH 8389.262 Hz
FIDRES 0.256020 Hz
AQ 1.9529728 sec
RG 25.4
DW 59.600 usec
DE 21.00 usec
TE 298.0 K
D1 2.00000000 sec
TD0 1
======== CHANNEL f1 ========
NUC1 1H
P1 10.20 usec
PL1 -0.90 dB
PL1W 15.85321522 W
SFO1 600.1536010 MHz
F2 - Processing parameters
SI 16384
SF 600.1500239 MHz
WDW EM
SSB 0
LB 0 Hz
GB 0
PC 1.00
7.8396 7.8283 7.8181 7.8110 7.8022 7.7968 7.5570 7.5548 7.5430 7.5410 7.4460 7.4427 7.4384 7.4338 7.4303 7.4258 7.3734 7.3703 7.3643 7.3579 6.9820 6.9791 6.9735 6.9709 6.9638 5.1597 5.1415 4.8696 4.8394 4.8362 4.8242 4.8210 4.8153 4.8051 4.7998 4.7777 4.7608 4.6902 4.6719 4.3219 4.3180 4.3170 4.3135 4.3044 4.2917 4.0624 4.0521 4.0465 4.0360 4.0272 4.0167 4.0081 3.9601 3.9498 3.9395 3.9292 3.9189 3.7204 3.7118 3.7041 3.6961 3.6886 3.6799 3.5700 3.5542 3.5385 3.4322 3.4172 3.4021 3.2798 3.2778 3.2648 3.2355 3.2174 3.1991 1.1914 1.1813 1.1716 1.0706 1.0603
9 8 7 6 5 4 3 2 1 0 ppm
4.07 1.02 2.03 2.39 3.02 1.00 3.60 1.01 1.01 2.01 2.04 1.00 1.03 1.04 1.03 0.75 0.99 6.02 2.99

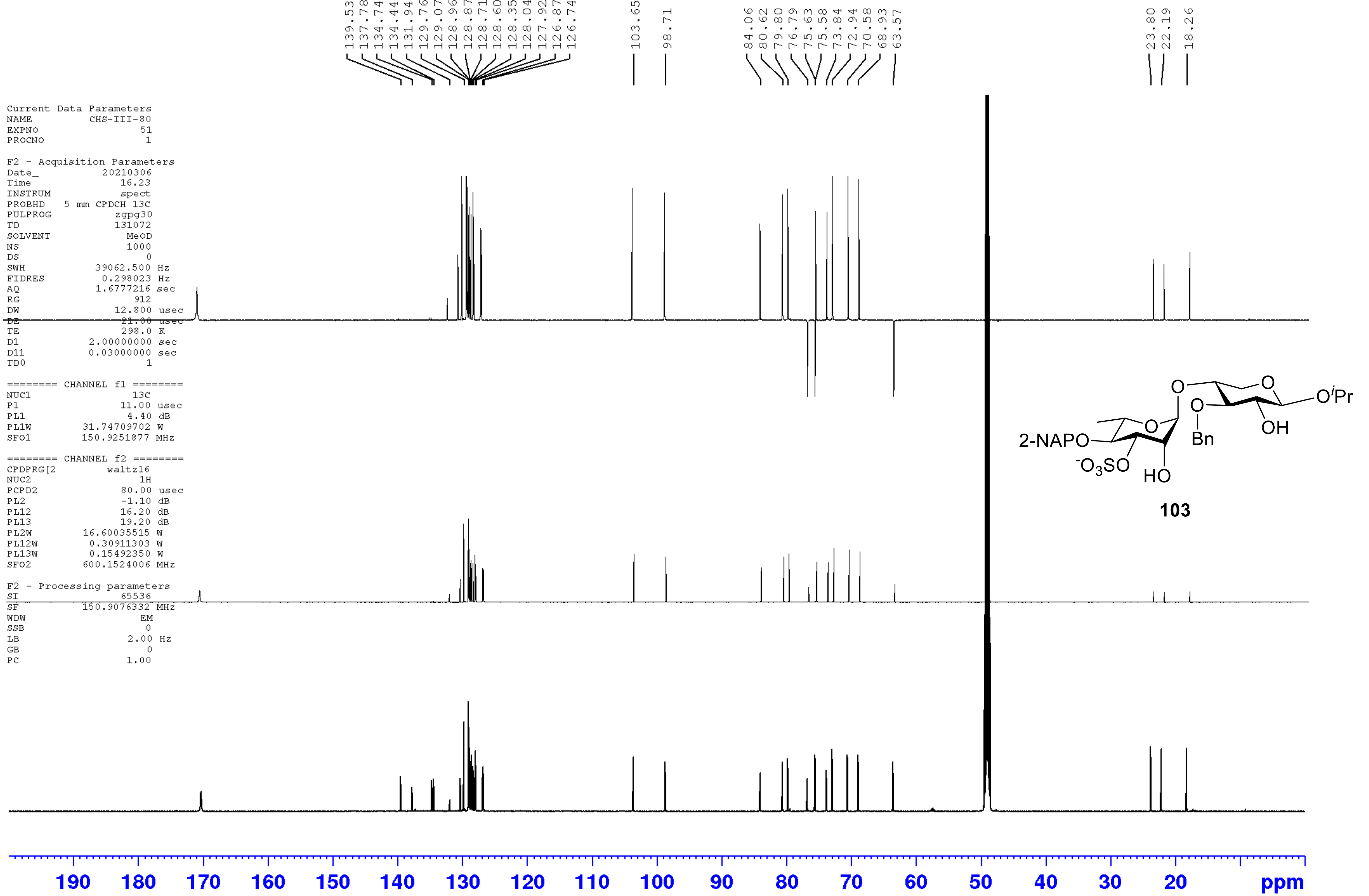

139.53
137.78
134.74
134.44
131.94
129.76
129.07
128.96
128.87
128.71
128.60
128.35
128.04
127.92
126.87
126.74
103.65
98.71
84.06
80.62
79.80
76.79
75.63
75.58
73.84
72.94
70.58
68.93
63.57
23.80
22.19
18.26
Current Data Parameters
NAME CHS-III-80
EXPNO 51
PROCNO 1
F2 - Acquisition Parameters
Date_ 20210306
Time 16.23
INSTRUM spect
PROBHD 5 mm CPDCH 13C
PULPROG zgpg30
TD 131072
SOLVENT MeOD
NS 1000
DS 0
SWH 39062.500 Hz
FIDRES 0.298023 Hz
AQ 1.6777216 sec
RG 912
DW 12.800 usec
DE 21.00 usec
TE 298.0 K
D1 2.00000000 sec
D11 0.03000000 sec
TD0 1
======== CHANNEL f1 ========
NUC1 13C
P1 11.00 usec
PL1 4.40 dB
PL1W 31.74709702 W
SFO1 150.9251877 MHz
======== CHANNEL f2 ========
CPDPRG[2 waltz16
NUC2 1H
PCPD2 80.00 usec
PL2 -1.10 dB
PL12 16.20 dB
PL13 19.20 dB
PL2W 16.60035515 W
PL12W 0.30911303 W
PL13W 0.15492350 W
SFO2 600.1524006 MHz
F2 - Processing parameters
SI 65536
SF 150.9076332 MHz
WDW EM
SSB 0
LB 2.00 Hz
GB 0
PC 1.00
2-NAPO
$^{-}O_3SO$
HO
O
Bn
OH
O^iPr
103
190 180 170 160 150 140 130 120 110 100 90 80 70 60 50 40 30 20 ppm

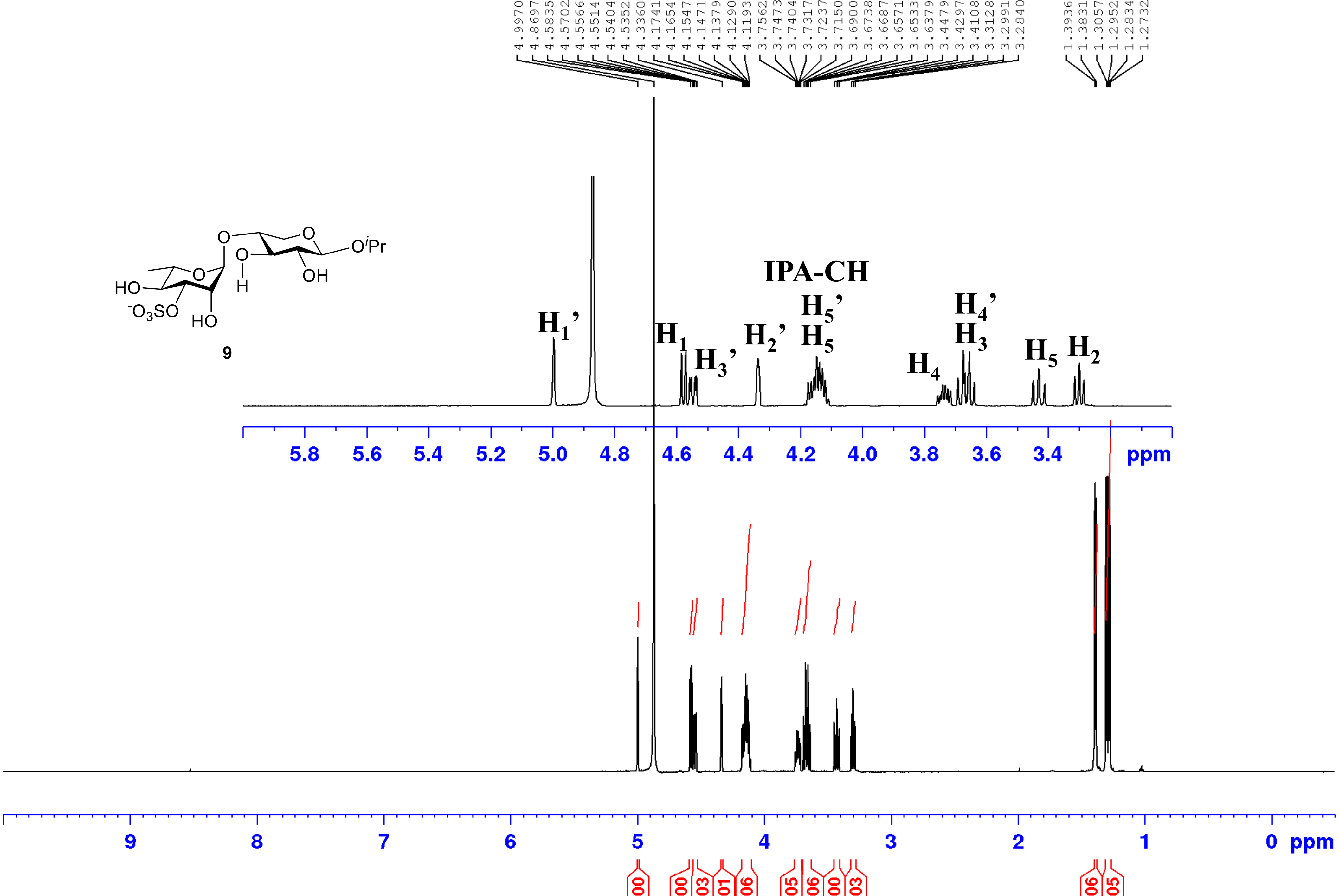
9
IPA-CH
H1'
H1
H3'
H2'
H5'
H5
H4
H4'
H3
H5
H2
ppm
1.00
1.00
1.03
1.01
3.06
1.05
2.06
1.00
1.03
3.06
6.05

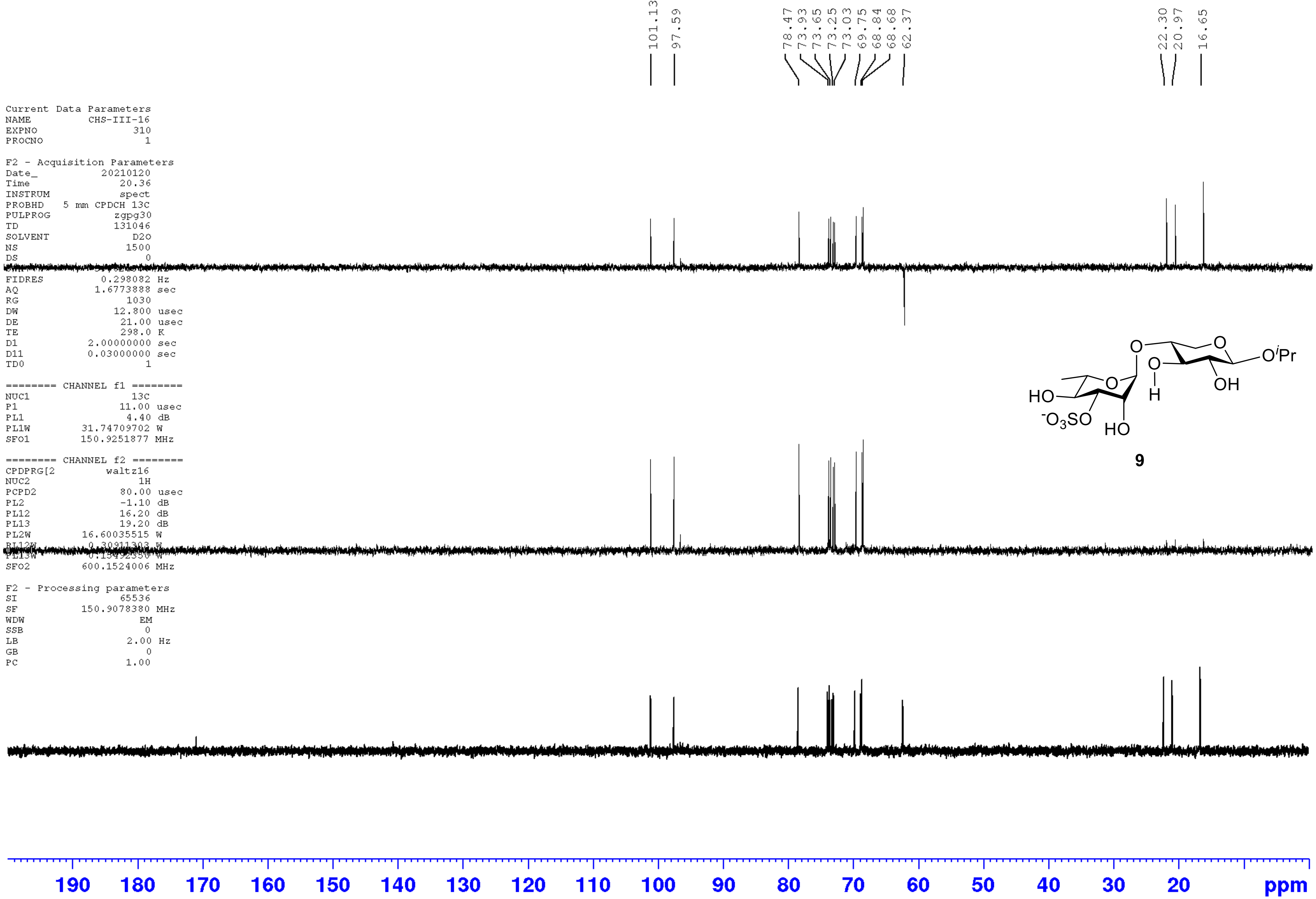
101.13
97.59
78.47
73.93
73.65
73.25
73.03
69.75
68.84
68.68
62.37
22.30
20.97
16.65
Current Data Parameters
NAME CHS-III-16
EXPNO 310
PROCNO 1
F2 - Acquisition Parameters
Date_ 20210120
Time 20.36
INSTRUM spect
PROBHD 5 mm CPDCH 13C
PULPROG zgpg30
TD 131046
SOLVENT D2O
NS 1500
DS 0
FIDRES 0.298082 Hz
AQ 1.6773888 sec
RG 1030
DW 12.800 usec
DE 21.00 usec
TE 298.0 K
D1 2.00000000 sec
D11 0.03000000 sec
TD0 1
======== CHANNEL f1 ========
NUC1 13C
P1 11.00 usec
PL1 4.40 dB
PL1W 31.74709702 W
SFO1 150.9251877 MHz
======== CHANNEL f2 ========
CPDPRG[2 waltz16
NUC2 1H
PCPD2 80.00 usec
PL2 -1.10 dB
PL12 16.20 dB
PL13 19.20 dB
PL2W 16.60035515 W
PL12W 0.30911303 W
PL13W 0.15492350 W
SFO2 600.1524006 MHz
F2 - Processing parameters
SI 65536
SF 150.9078380 MHz
WDW EM
SSB 0
LB 2.00 Hz
GB 0
PC 1.00
190 180 170 160 150 140 130 120 110 100 90 80 70 60 50 40 30 20 ppm
HO
-O3SO
HO
O
O
H
OH
OiPr

9

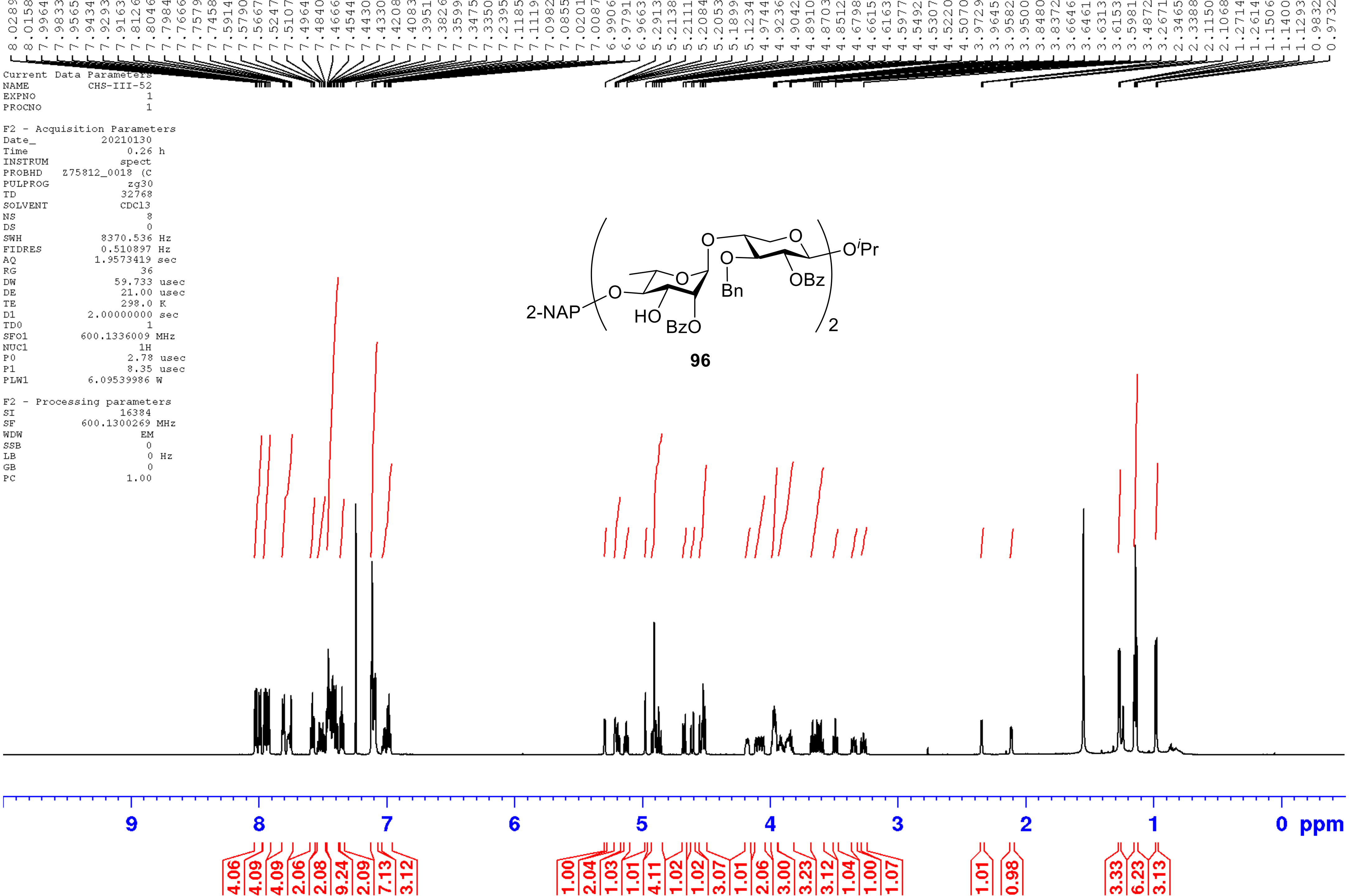


```
Current Data Parameters
NAME          CHS-III-52
EXPNO                  1
PROCNO                 1

F2 - Acquisition Parameters
Date_           20210130
Time              0.26 h
INSTRUM            spect
PROBHD   Z75812_0018 (C
PULPROG             zg30
TD                 32768
SOLVENT            CDCl3
NS                     8
DS                     0
SWH             8370.536 Hz
FIDRES          0.510897 Hz
AQ             1.9573419 sec
RG                    36
DW                59.733 usec
DE                 21.00 usec
TE                 298.0 K
D1            2.00000000 sec
TD0                    1
SFO1        600.1336009 MHz
NUC1                  1H
P0                  2.78 usec
P1                  8.35 usec
PLW1          6.09539986 W

F2 - Processing parameters
SI                 16384
SF           600.1300269 MHz
WDW                   EM
SSB                    0
LB                     0 Hz
GB                     0
PC                  1.00
```


96

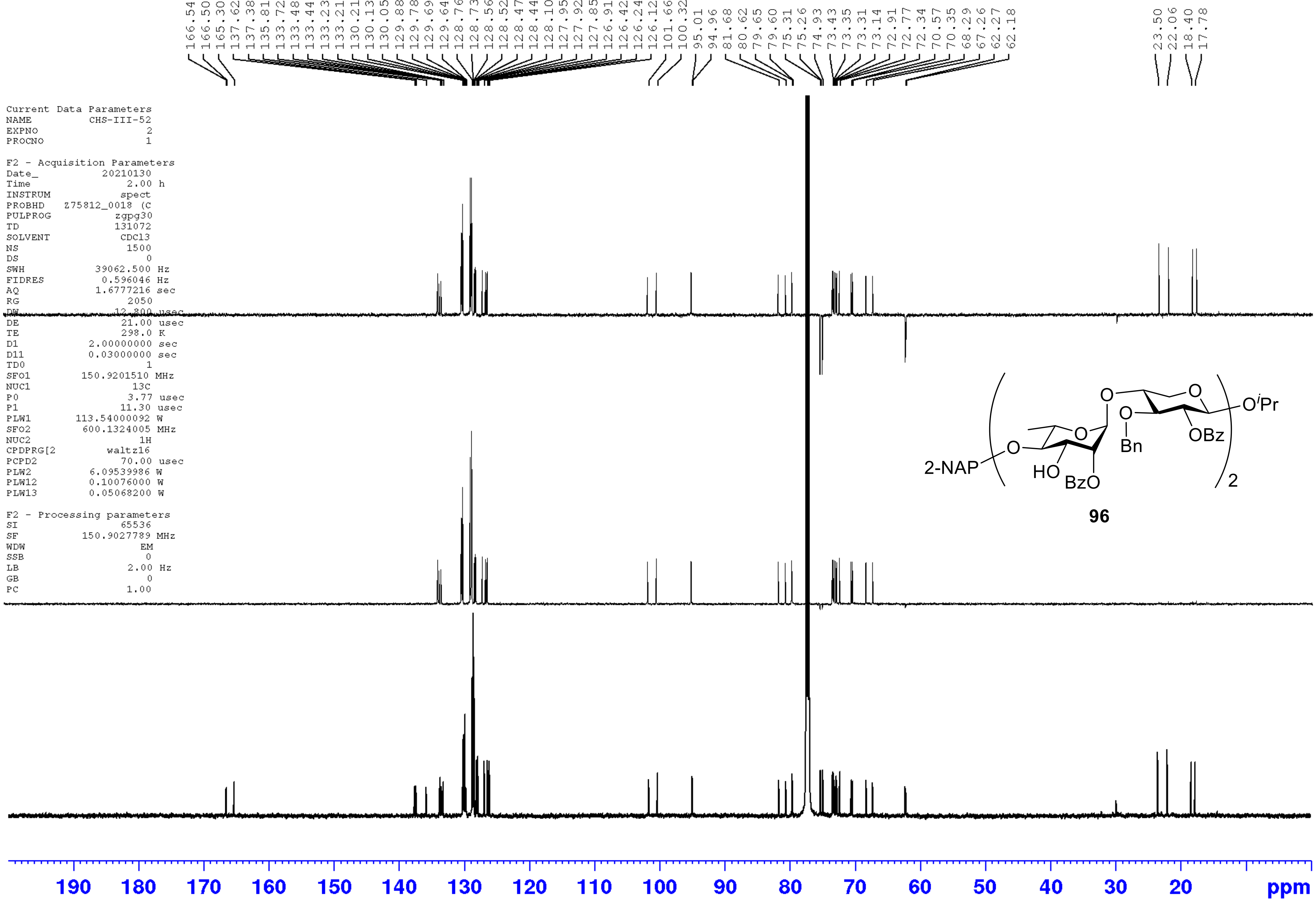
166.54
166.50
165.30
137.62
137.38
135.81
133.72
133.48
133.44
133.23
133.21
130.21
130.13
130.05
129.88
129.78
129.69
129.64
128.76
128.73
128.56
128.52
128.47
128.44
128.10
127.95
127.92
127.85
126.91
126.42
126.24
126.12
101.66
100.32
95.01
94.96
81.68
80.62
79.65
79.60
75.31
75.26
74.93
73.43
73.35
73.31
73.14
72.91
72.77
72.34
70.57
70.35
68.29
67.26
62.27
62.18
23.50
22.06
18.40
17.78
Current Data Parameters
NAME CHS-III-52
EXPNO 2
PROCNO 1
F2 - Acquisition Parameters
Date_ 20210130
Time 2.00 h
INSTRUM spect
PROBHD Z75812_0018 (C
PULPROG zgpg30
TD 131072
SOLVENT CDCl3
NS 1500
DS 0
SWH 39062.500 Hz
FIDRES 0.596046 Hz
AQ 1.6777216 sec
RG 2050
DW 12.800 usec
DE 21.00 usec
TE 298.0 K
D1 2.00000000 sec
D11 0.03000000 sec
TD0 1
SFO1 150.9201510 MHz
NUC1 13C
P0 3.77 usec
P1 11.30 usec
PLW1 113.54000092 W
SFO2 600.1324005 MHz
NUC2 1H
CPDPRG[2 waltz16
PCPD2 70.00 usec
PLW2 6.09539986 W
PLW12 0.10076000 W
PLW13 0.05068200 W
F2 - Processing parameters
SI 65536
SF 150.9027789 MHz
WDW EM
SSB 0
LB 2.00 Hz
GB 0
PC 1.00
190 180 170 160 150 140 130 120 110 100 90 80 70 60 50 40 30 20 ppm
O
O
O^{i}Pr
O
O
Bn
OBz
2-NAP
O
HO
BzO
2

96

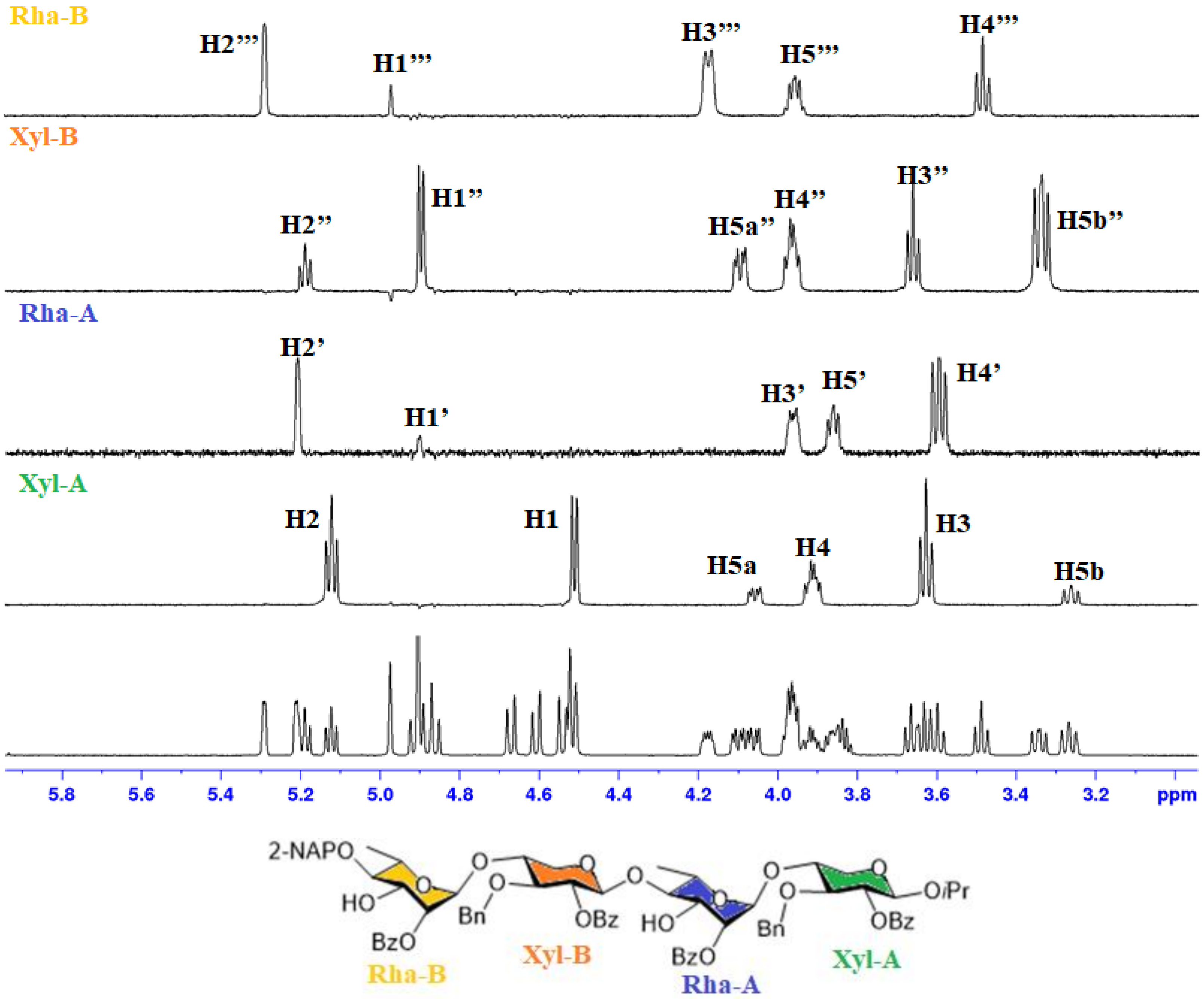
Rha-B
H2'''
H1'''
H3'''
H5'''
H4'''
Xyl-B
H2''
H1''
H5a''
H4''
H3''
H5b''
Rha-A
H2'
H1'
H3'
H5'
H4'
Xyl-A
H2
H1
H5a
H4
H3
H5b
5.8
5.6
5.4
5.2
5.0
4.8
4.6
4.4
4.2
4.0
3.8
3.6
3.4
3.2
ppm
2-NAPO
HO
BzO
Bn
OBz
HO
BzO
Bn
OBz
OiPr
Rha-B
Xyl-B
Rha-A
Xyl-A

96

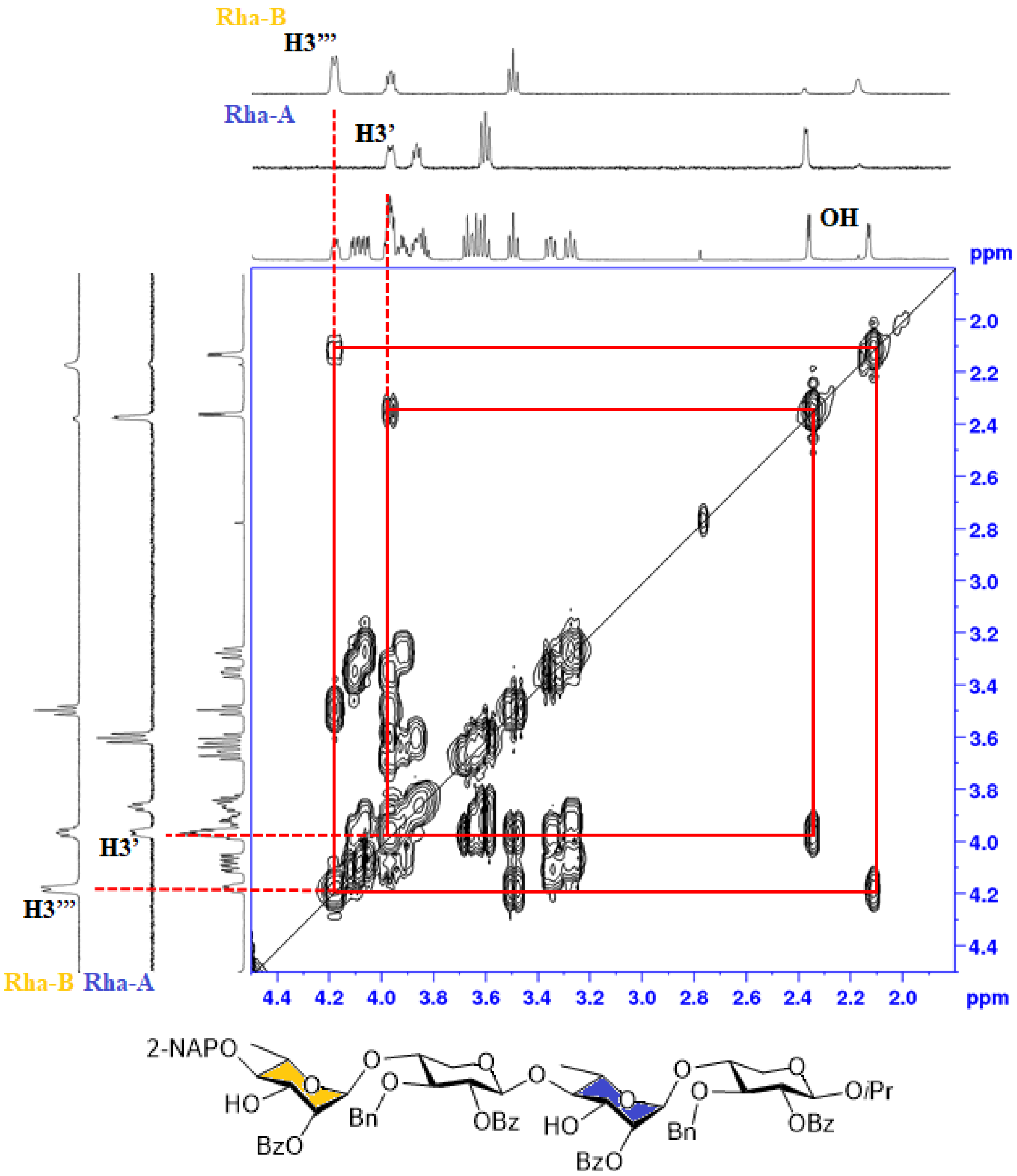
Rha-B
H3'''
Rha-A
H3'
OH
ppm
2.0
2.2
2.4
2.6
2.8
3.0
3.2
3.4
3.6
3.8
4.0
4.2
4.4
H3'
H3'''
Rha-B Rha-A
4.4 4.2 4.0 3.8 3.6 3.4 3.2 3.0 2.8 2.6 2.4 2.2 2.0
ppm
2-NAPO
HO
BzO
Bn
OBz
HO
BzO
Bn
OBz
O*i*Pr

96

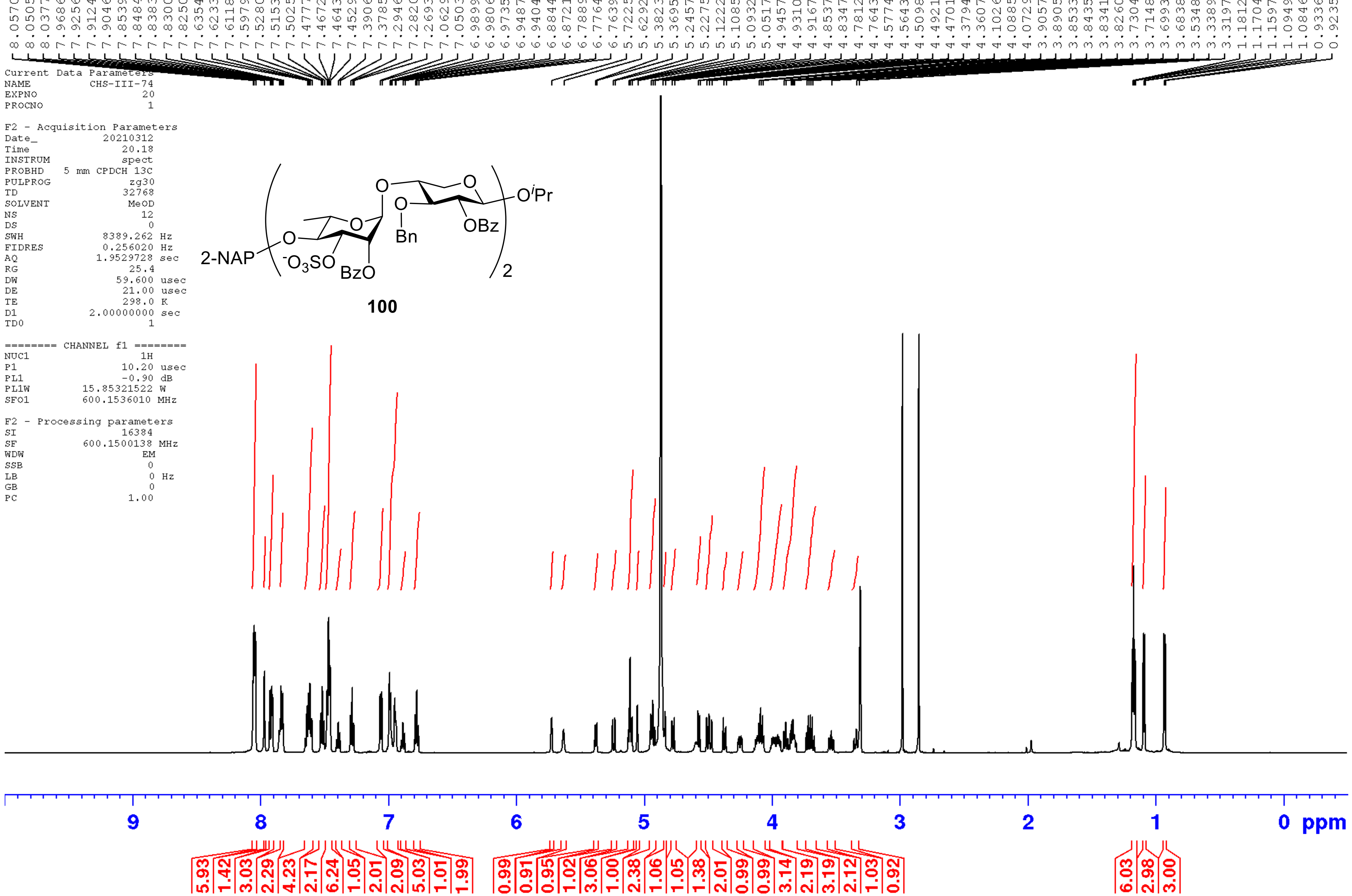

Current Data Parameters
NAME CHS-III-74
EXPNO 20
PROCNO 1
F2 - Acquisition Parameters
Date_ 20210312
Time 20.18
INSTRUM spect
PROBHD 5 mm CPDCH 13C
PULPROG zg30
TD 32768
SOLVENT MeOD
NS 12
DS 0
SWH 8389.262 Hz
FIDRES 0.256020 Hz
AQ 1.9529728 sec
RG 25.4
DW 59.600 usec
DE 21.00 usec
TE 298.0 K
D1 2.00000000 sec
TD0 1
======== CHANNEL f1 ========
NUC1 1H
P1 10.20 usec
PL1 -0.90 dB
PL1W 15.85321522 W
SFO1 600.1536010 MHz
F2 - Processing parameters
SI 16384
SF 600.1500138 MHz
WDW EM
SSB 0
LB 0 Hz
GB 0
PC 1.00
2-NAP
O^{i}Pr
OBz
Bn
BzO
^-O_3SO
100
ppm

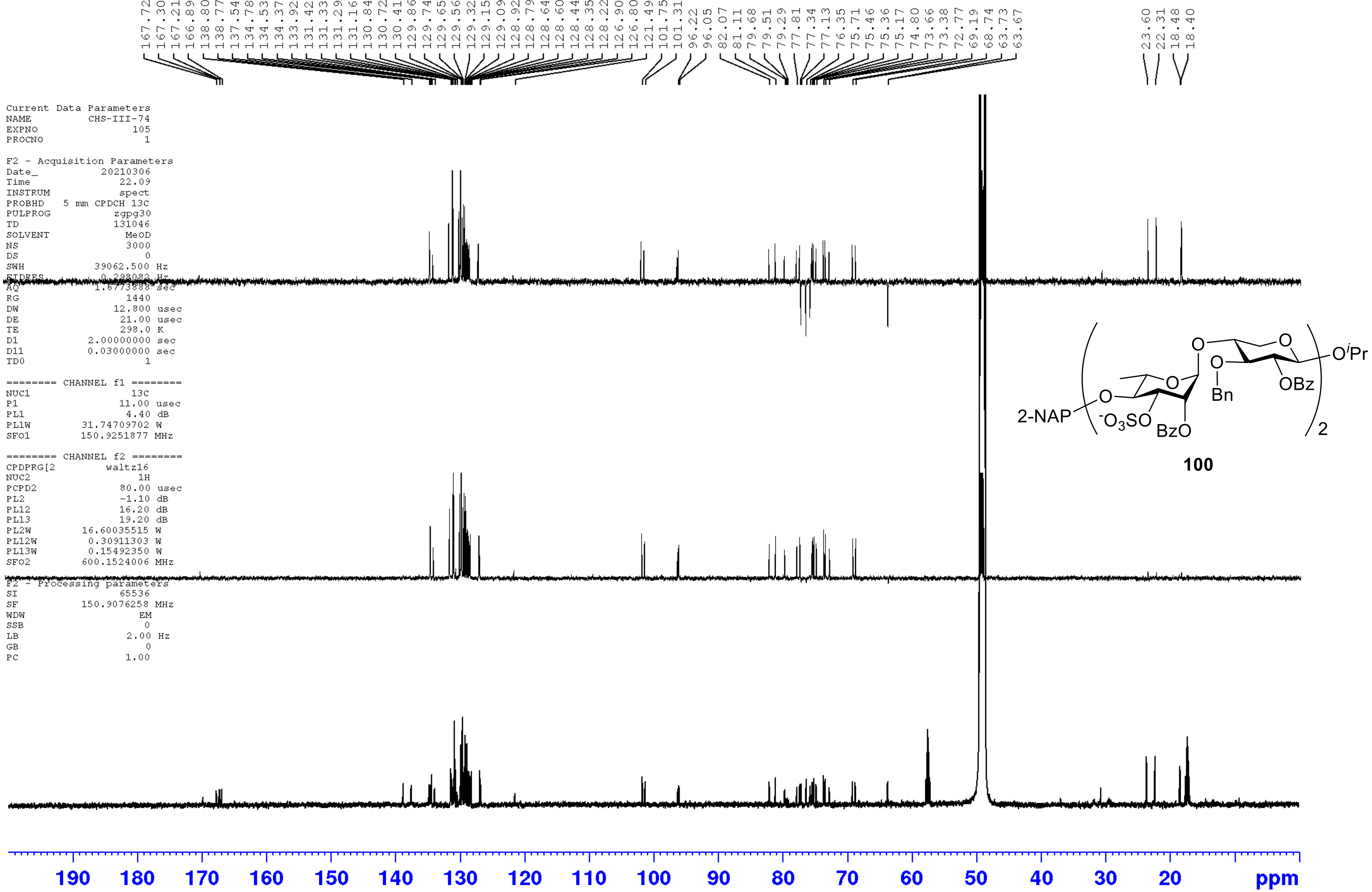
167.72
167.30
167.21
166.89
138.80
138.77
137.54
134.78
134.53
134.37
133.92
131.42
131.33
131.29
131.16
130.84
130.72
130.41
129.86
129.74
129.65
129.56
129.32
129.15
129.09
128.92
128.79
128.64
128.60
128.44
128.35
128.22
126.90
126.80
121.49
101.75
101.31
96.22
96.05
82.07
81.11
79.68
79.51
79.29
77.81
77.34
77.13
76.35
75.71
75.46
75.36
75.17
74.80
73.66
73.38
72.77
69.19
68.74
63.73
63.67
23.60
22.31
18.48
18.40
Current Data Parameters
NAME CHS-III-74
EXPNO 105
PROCNO 1
F2 - Acquisition Parameters
Date_ 20210306
Time 22.09
INSTRUM spect
PROBHD 5 mm CPDCH 13C
PULPROG zgpg30
TD 131046
SOLVENT MeOD
NS 3000
DS 0
SWH 39062.500 Hz
FIDRES 0.298082 Hz
AQ 1.6773888 sec
RG 1440
DW 12.800 usec
DE 21.00 usec
TE 298.0 K
D1 2.00000000 sec
D11 0.03000000 sec
TD0 1
======== CHANNEL f1 ========
NUC1 13C
P1 11.00 usec
PL1 4.40 dB
PL1W 31.74709702 W
SFO1 150.9251877 MHz
======== CHANNEL f2 ========
CPDPRG[2 waltz16
NUC2 1H
PCPD2 80.00 usec
PL2 -1.10 dB
PL12 16.20 dB
PL13 19.20 dB
PL2W 16.60035515 W
PL12W 0.30911303 W
PL13W 0.15492350 W
SFO2 600.1524006 MHz
F2 - Processing parameters
SI 65536
SF 150.9076258 MHz
WDW EM
SSB 0
LB 2.00 Hz
GB 0
PC 1.00
190 180 170 160 150 140 130 120 110 100 90 80 70 60 50 40 30 20 ppm
O
O
O^iPr
O
O
Bn
OBz
O
2-NAP
-O3SO
BzO
2

100

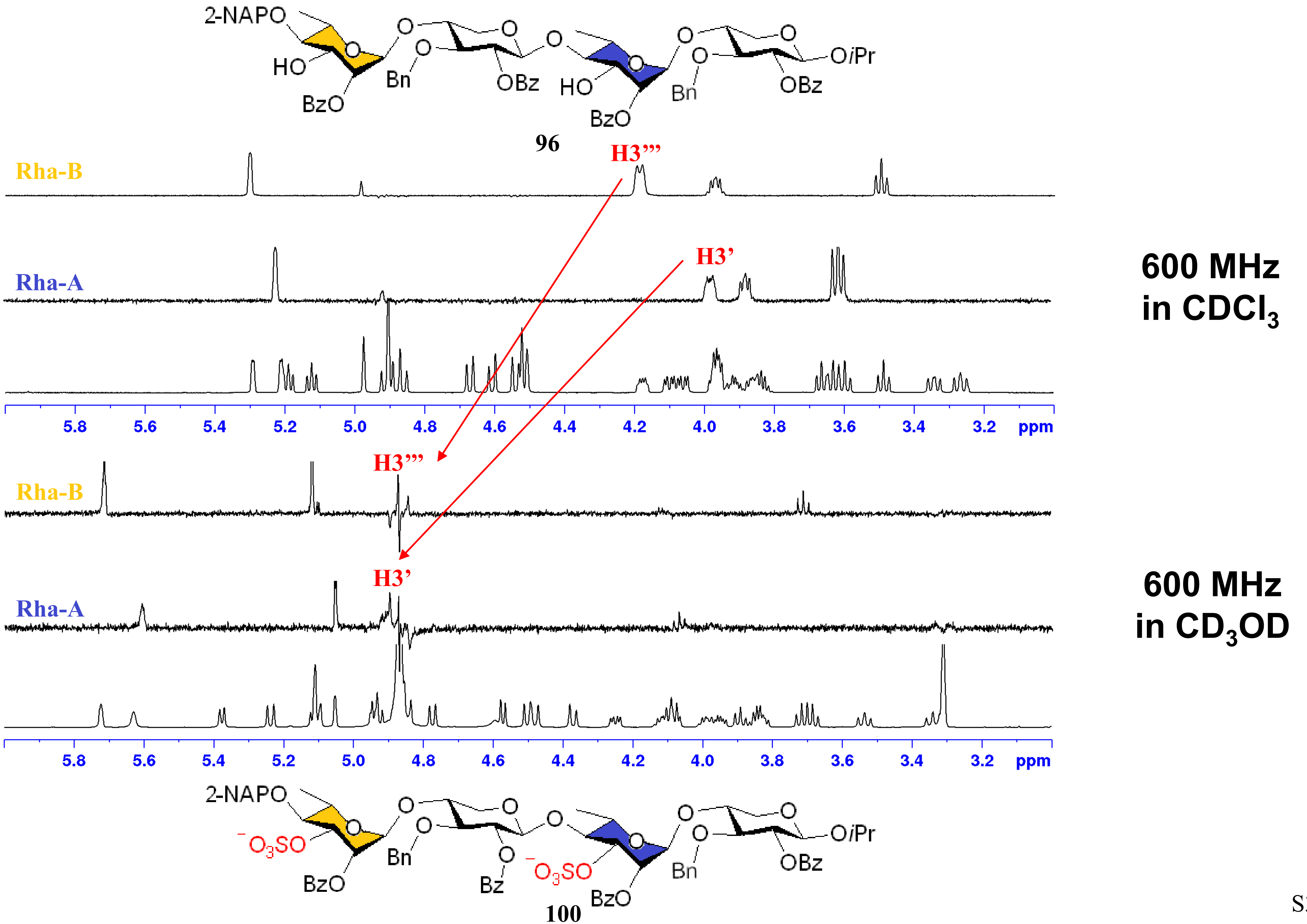
2-NAPO
HO
BzO
Bn
OBz
HO
BzO
Bn
OBz
OiPr
96
H3'''
Rha-B
Rha-A
H3'
600 MHz in $CDCl_3$
5.8 5.6 5.4 5.2 5.0 4.8 4.6 4.4 4.2 4.0 3.8 3.6 3.4 3.2 ppm
H3'''
Rha-B
H3'
Rha-A
600 MHz in CD_3OD
5.8 5.6 5.4 5.2 5.0 4.8 4.6 4.4 4.2 4.0 3.8 3.6 3.4 3.2 ppm
2-NAPO
$^{-}O_3SO$
BzO
Bn
Bz
$^{-}O_3SO$
BzO
Bn
OBz
OiPr
100

Current Data Parameters
NAME CHS-III-76
EXPNO 2
PROCNO 1

F2 - Acquisition Parameters
Date_ 20210304
Time 14.07
INSTRUM spect
PROBHD 5 mm CPDCH 13C
PULPROG zg30
TD 32768
SOLVENT MeOD
NS 8
DS 0
SWH 8389.262 Hz
FIDRES 0.256020 Hz
AQ 1.9529728 sec
RG 32
DW 59.600 usec
DE 21.00 usec
TE 298.0 K
D1 2.00000000 sec
TD0 1

======== CHANNEL f1 ========
NUC1 1H
P1 10.20 usec
PL1 -0.90 dB
PL1W 15.85321522 W
SFO1 600.1536010 MHz

F2 - Processing parameters
SI 16384
SF 600.1500121 MHz
WDW EM
SSB 0
LB 0 Hz
GB 0
PC 1.00

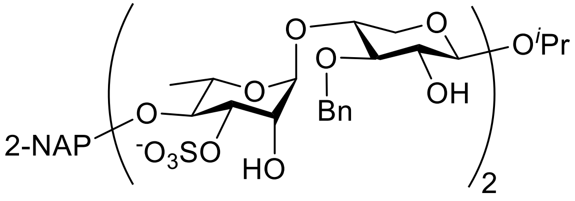

104

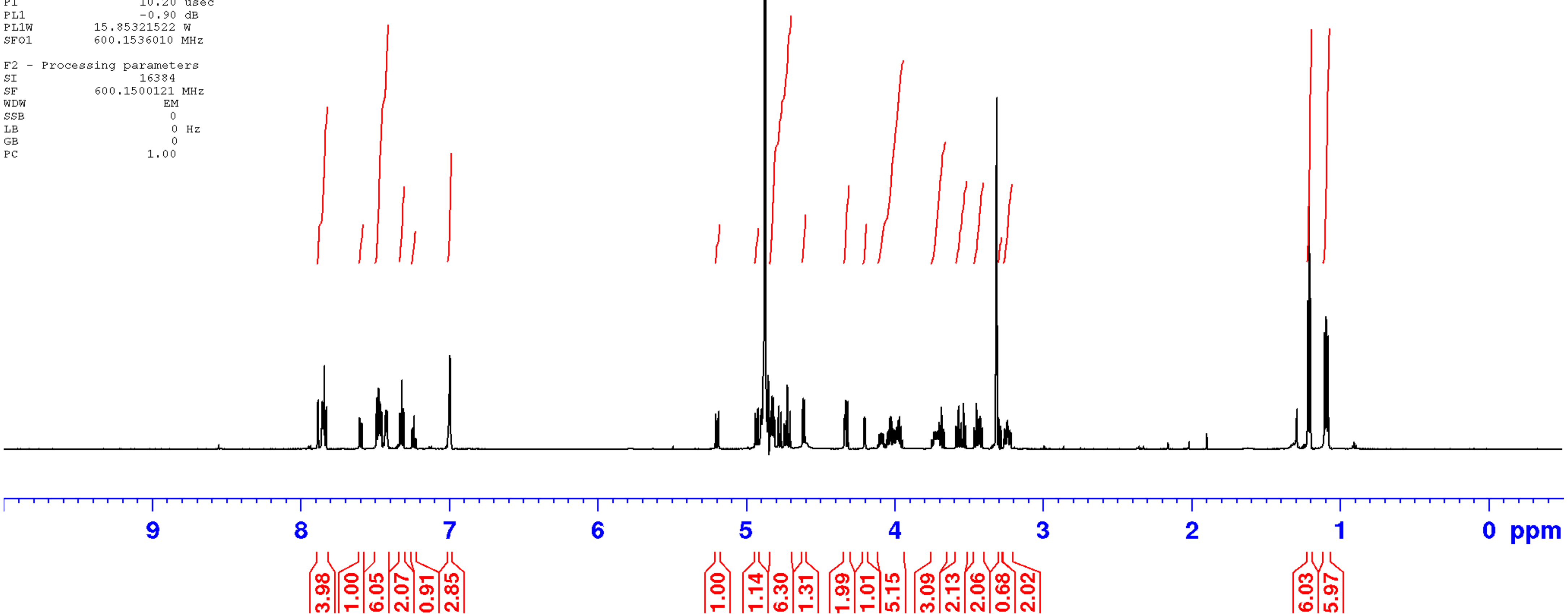

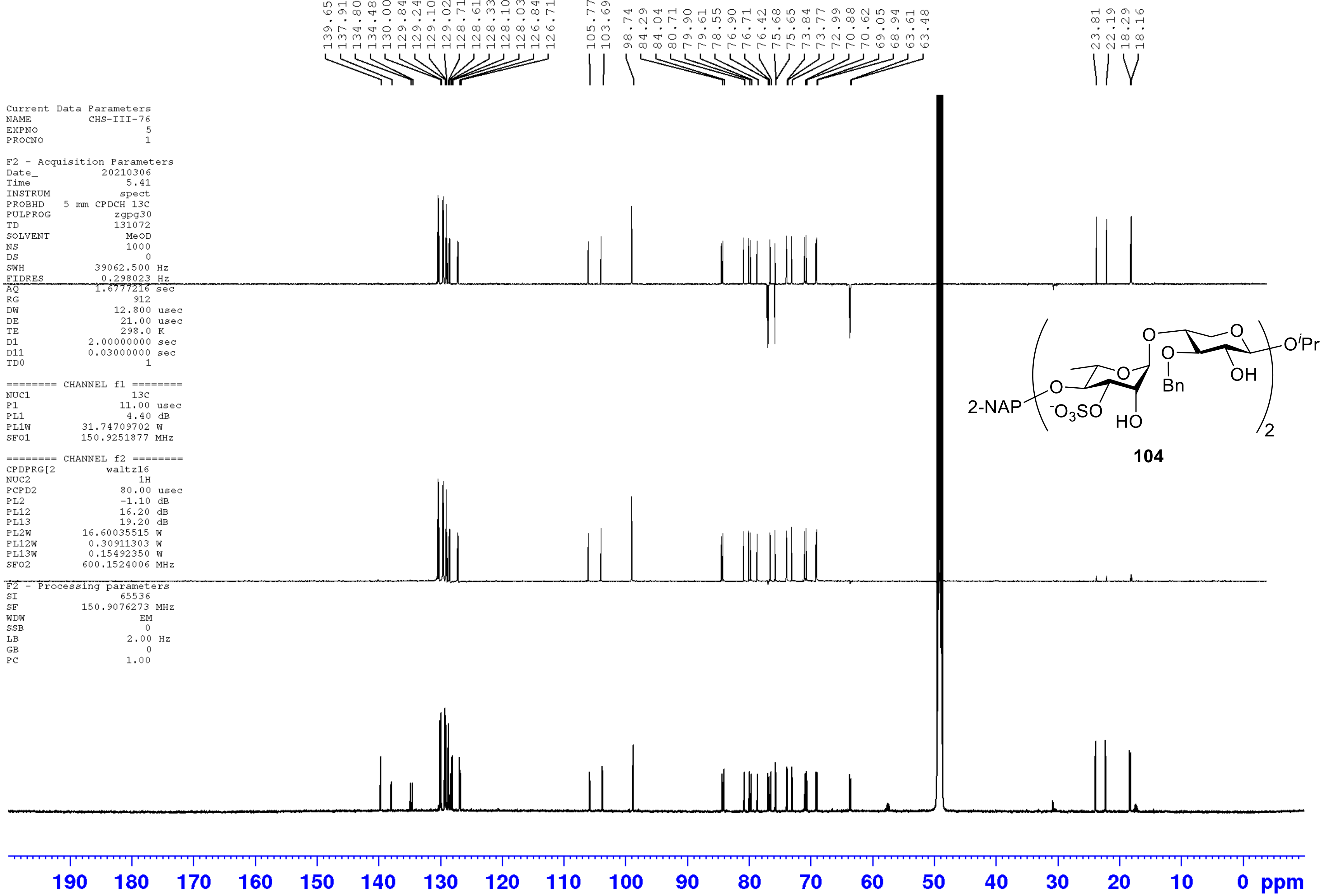
139.65
137.91
134.80
134.48
130.00
129.84
129.24
129.10
129.02
128.71
128.61
128.33
128.10
128.03
126.84
126.71
105.77
103.69
98.74
84.29
84.04
80.71
79.90
79.61
78.55
76.90
76.71
76.42
75.68
75.65
73.84
73.77
72.99
70.88
70.62
69.05
68.94
63.61
63.48
23.81
22.19
18.29
18.16
Current Data Parameters
NAME CHS-III-76
EXPNO 5
PROCNO 1
F2 - Acquisition Parameters
Date_ 20210306
Time 5.41
INSTRUM spect
PROBHD 5 mm CPDCH 13C
PULPROG zgpg30
TD 131072
SOLVENT MeOD
NS 1000
DS 0
SWH 39062.500 Hz
FIDRES 0.298023 Hz
AQ 1.6777216 sec
RG 912
DW 12.800 usec
DE 21.00 usec
TE 298.0 K
D1 2.00000000 sec
D11 0.03000000 sec
TD0 1
======== CHANNEL f1 ========
NUC1 13C
P1 11.00 usec
PL1 4.40 dB
PL1W 31.74709702 W
SFO1 150.9251877 MHz
======== CHANNEL f2 ========
CPDPRG[2 waltz16
NUC2 1H
PCPD2 80.00 usec
PL2 -1.10 dB
PL12 16.20 dB
PL13 19.20 dB
PL2W 16.60035515 W
PL12W 0.30911303 W
PL13W 0.15492350 W
SFO2 600.1524006 MHz
F2 - Processing parameters
SI 65536
SF 150.9076273 MHz
WDW EM
SSB 0
LB 2.00 Hz
GB 0
PC 1.00
O
O
O
O
OiPr
O
Bn
OH
2-NAP
-O3SO
HO
2
104
190 180 170 160 150 140 130 120 110 100 90 80 70 60 50 40 30 20 10 0 ppm

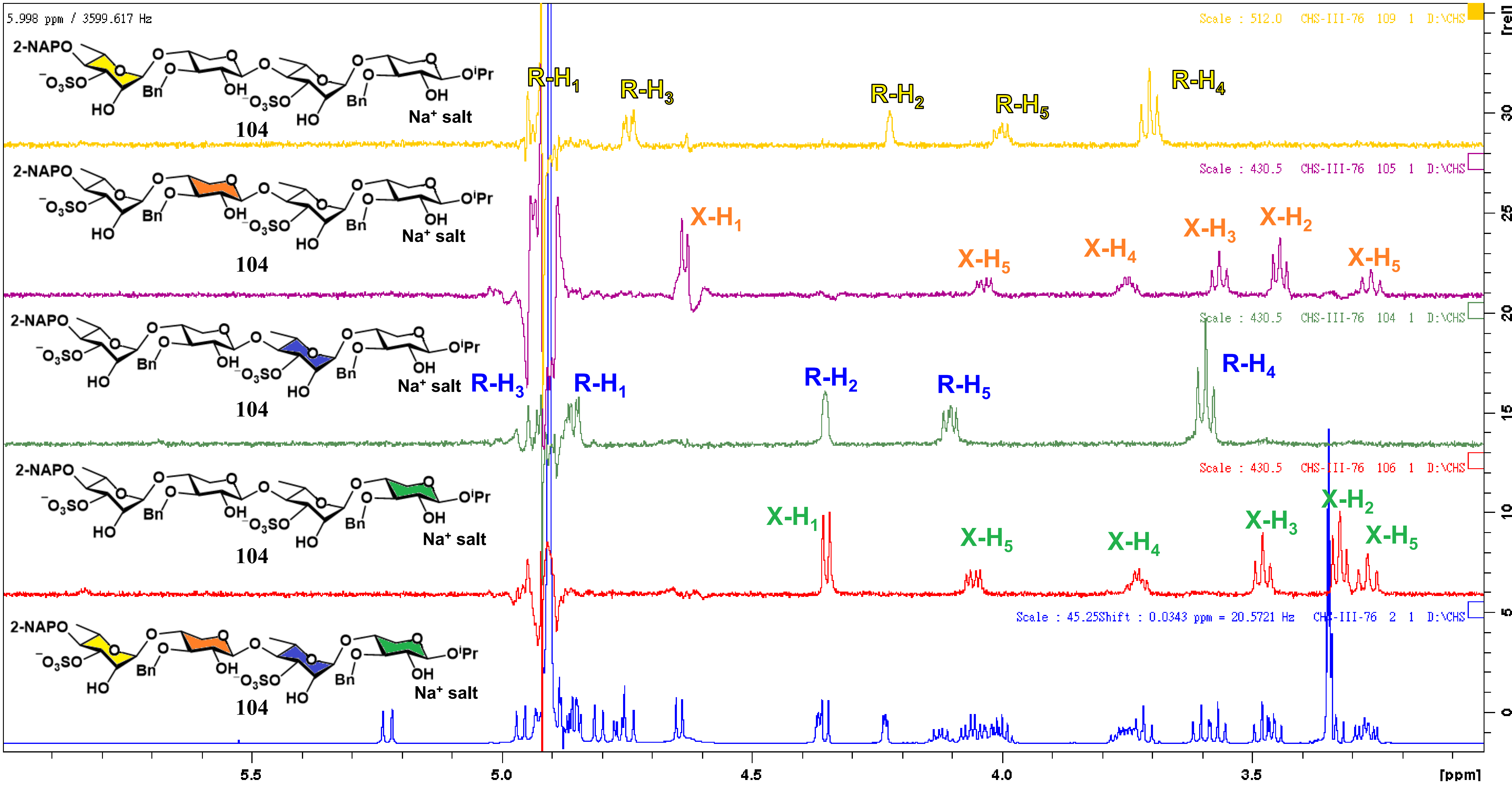

5.998 ppm / 3599.617 Hz
2-NAPO
$^{-}O_3SO$
HO
Bn
OH
$^{-}O_3SO$
HO
Bn
OH
O^iPr
Na^+ salt
104
R-H1
R-H3
R-H2
R-H5
R-H4
X-H1
X-H5
X-H4
X-H3
X-H2
X-H5
Scale : 512.0 CHS-III-76 109 1 D:\CHS
Scale : 430.5 CHS-III-76 105 1 D:\CHS
Scale : 430.5 CHS-III-76 104 1 D:\CHS
Scale : 430.5 CHS-III-76 106 1 D:\CHS
Scale : 45.25Shift : 0.0343 ppm = 20.5721 Hz CHS-III-76 2 1 D:\CHS
5.5
5.0
4.5
4.0
3.5
[ppm]
[rel]
30
25
20
15
10
5
0

Current Data Parameters
NAME CHS-RXRX
EXPNO 10
PROCNO 1

F2 - Acquisition Parameters
Date_ 20210603
Time 18.47 h
INSTRUM spect
PROBHD Z75812_0018 (C
PULPROG zg30
TD 32768
SOLVENT D2O
NS 8
DS 0
SWH 8370.536 Hz
FIDRES 0.510897 Hz
AQ 1.9573419 sec
RG 9
DW 59.733 usec
DE 10.00 usec
TE 298.0 K
D1 2.00000000 sec
TD0 1
SFO1 600.1336009 MHz
NUC1 1H
P1 8.50 usec
PLW1 6.09539986 W

F2 - Processing parameters
SI 16384
SF 600.1299466 MHz
WDW EM
SSB 0
LB 0 Hz
GB 0
PC 1.00

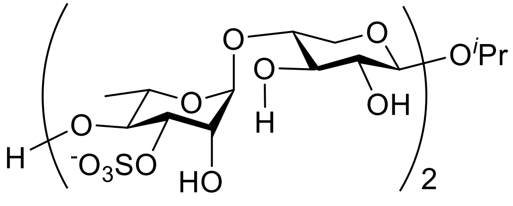

10

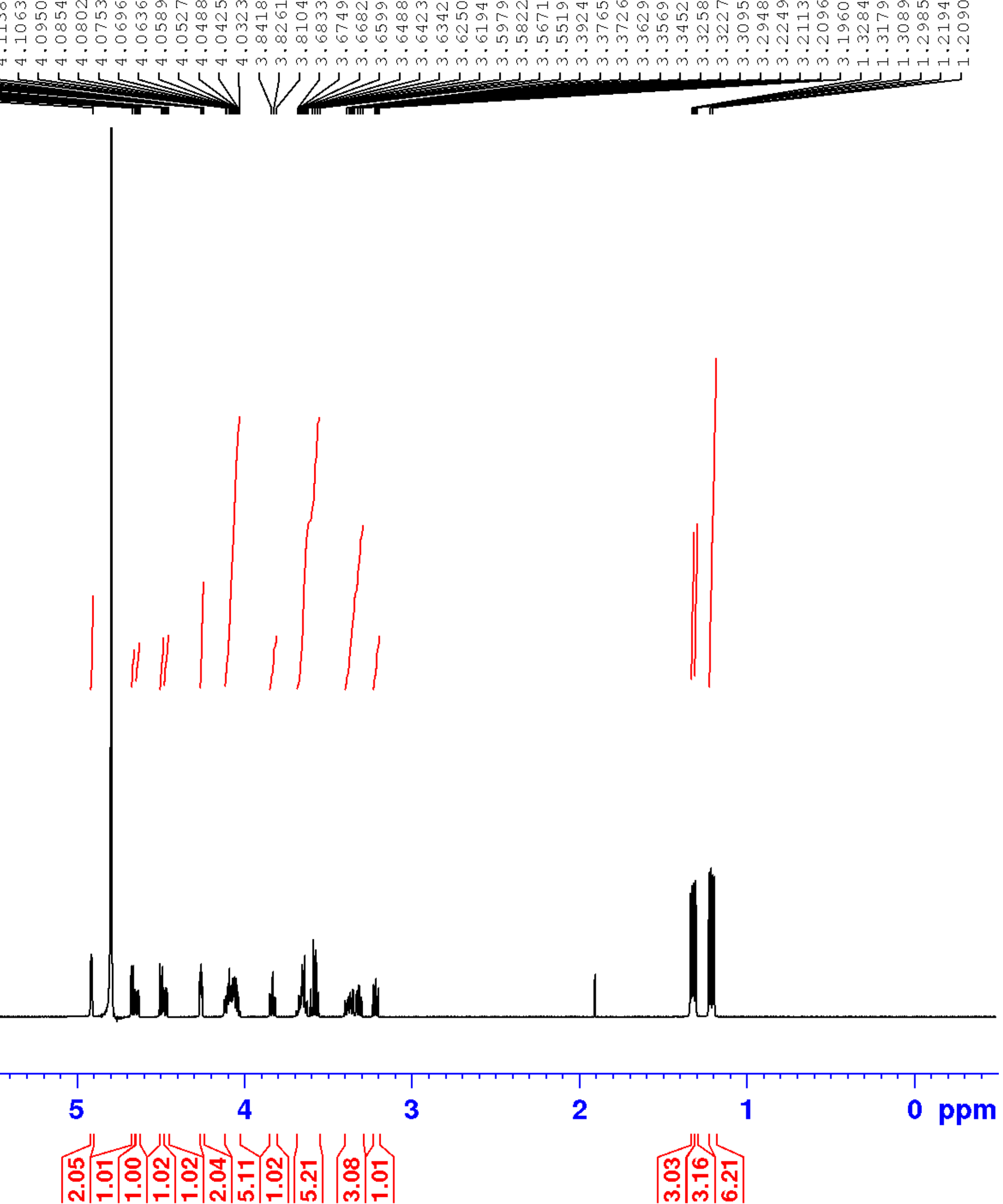

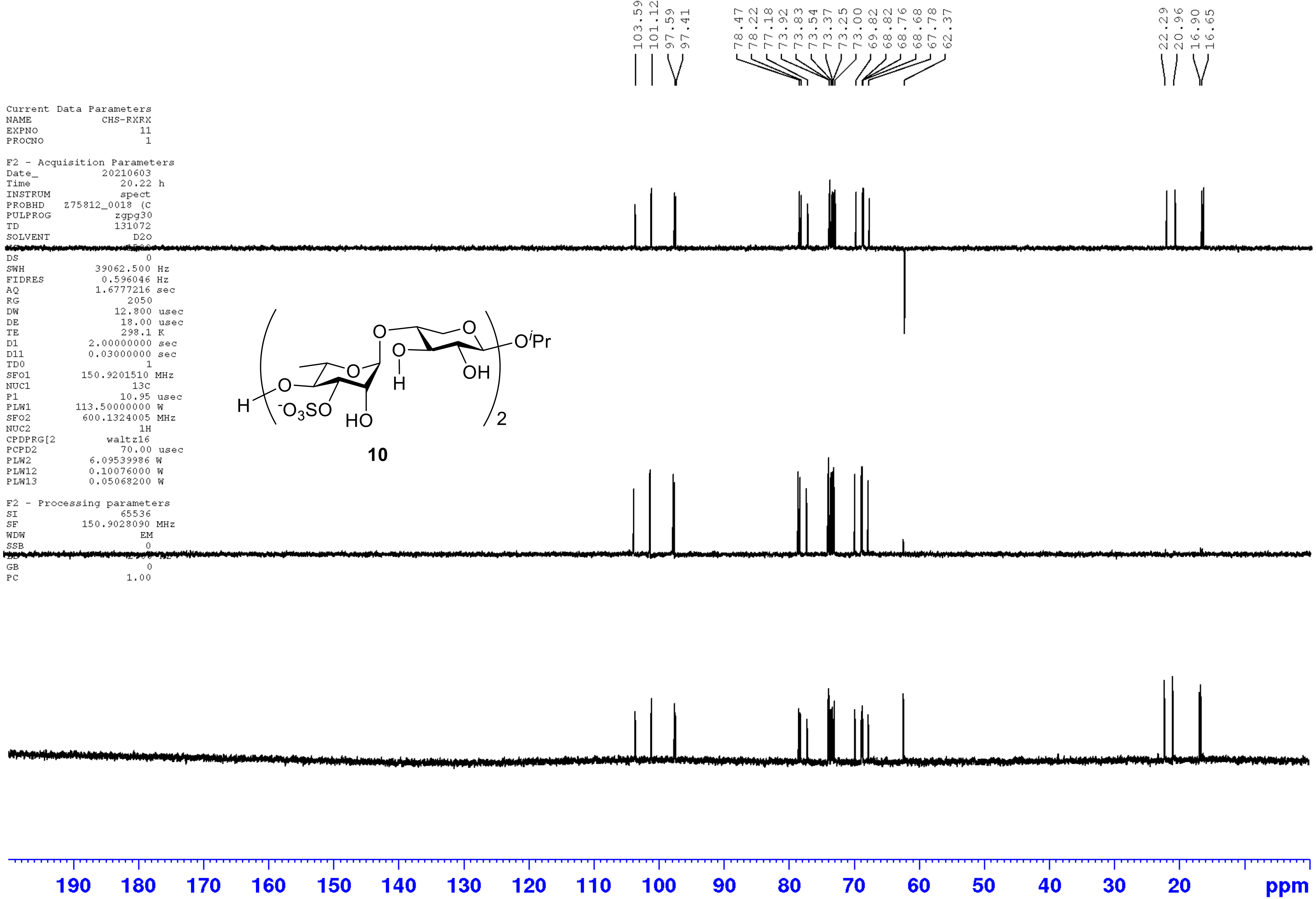
103.59
101.12
97.59
97.41
78.47
78.22
77.18
73.92
73.83
73.54
73.37
73.25
73.00
69.82
68.82
68.76
68.68
67.78
62.37
22.29
20.96
16.90
16.65
Current Data Parameters
NAME CHS-RXRX
EXPNO 11
PROCNO 1
F2 - Acquisition Parameters
Date_ 20210603
Time 20.22 h
INSTRUM spect
PROBHD Z75812_0018 (C
PULPROG zgpg30
TD 131072
SOLVENT D2O
DS 0
SWH 39062.500 Hz
FIDRES 0.596046 Hz
AQ 1.6777216 sec
RG 2050
DW 12.800 usec
DE 18.00 usec
TE 298.1 K
D1 2.00000000 sec
D11 0.03000000 sec
TD0 1
SFO1 150.9201510 MHz
NUC1 13C
P1 10.95 usec
PLW1 113.50000000 W
SFO2 600.1324005 MHz
NUC2 1H
CPDPRG[2 waltz16
PCPD2 70.00 usec
PLW2 6.09539986 W
PLW12 0.10076000 W
PLW13 0.05068200 W
F2 - Processing parameters
SI 65536
SF 150.9028090 MHz
WDW EM
SSB 0
GB 0
PC 1.00
O^iPr
OH
H
O
-O3SO
HO
2
10
190 180 170 160 150 140 130 120 110 100 90 80 70 60 50 40 30 20 ppm

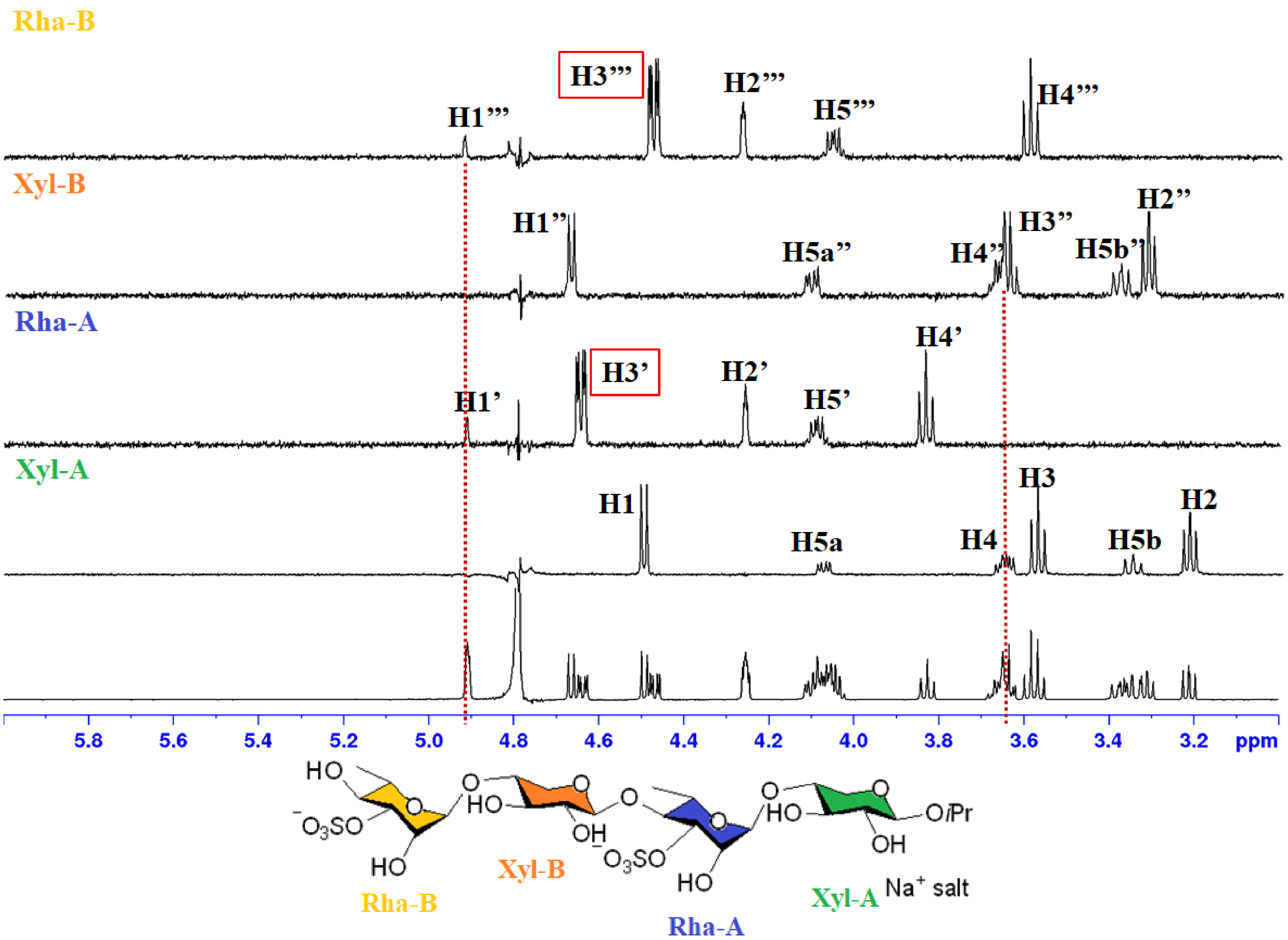
Rha-B
H3'''
H2'''
H1'''
H5'''
H4'''
Xyl-B
H1''
H5a''
H4''
H3''
H5b''
H2''
Rha-A
H3'
H2'
H1'
H5'
H4'
Xyl-A
H1
H5a
H4
H3
H5b
H2
5.8
5.6
5.4
5.2
5.0
4.8
4.6
4.4
4.2
4.0
3.8
3.6
3.4
3.2
ppm
HO
O
HO
$^{-}O_3SO$
OH
HO
$^{-}O_3SO$
HO
OiPr
OH
Rha-B
Xyl-B
Rha-A
Xyl-A
Na^+ salt

10

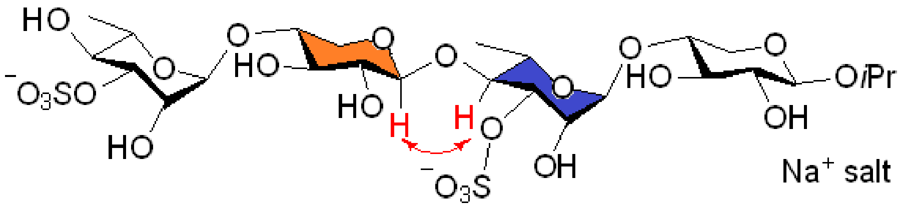
HO
^-O_3SO
HO
O
HO
HO
H
H
O
O
^-O_3S
OH
O
HO
O
OiPr
OH
Na⁺ salt

10

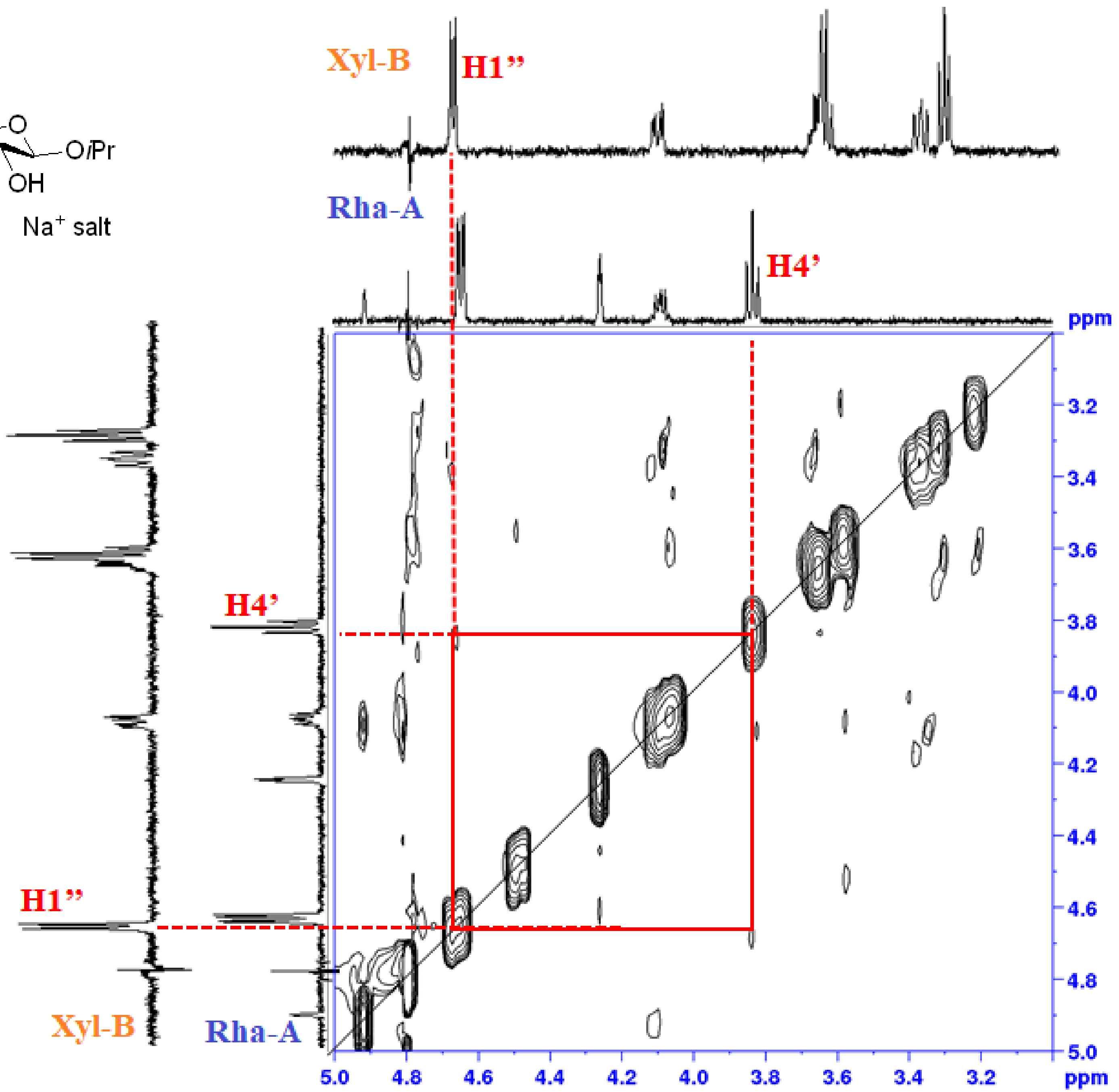
Xyl-B
H1''
Rha-A
H4'
ppm
H4'
H1''
Xyl-B
Rha-A
5.0
4.8
4.6
4.4
4.2
4.0
3.8
3.6
3.4
3.2
ppm

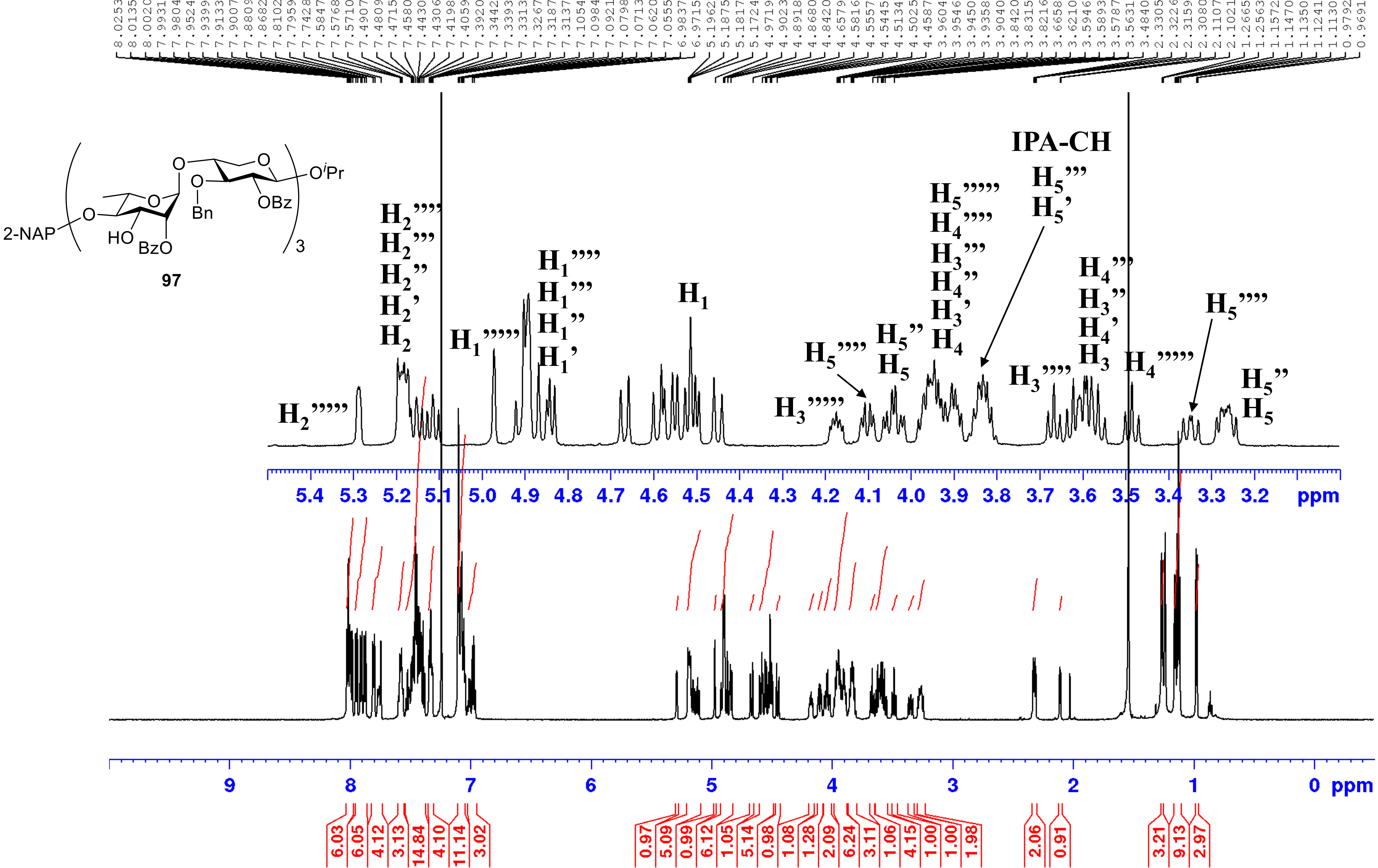
2-NAP
HO
BzO
O
Bn
OBz
O^{i}Pr
3
97
IPA-CH
ppm

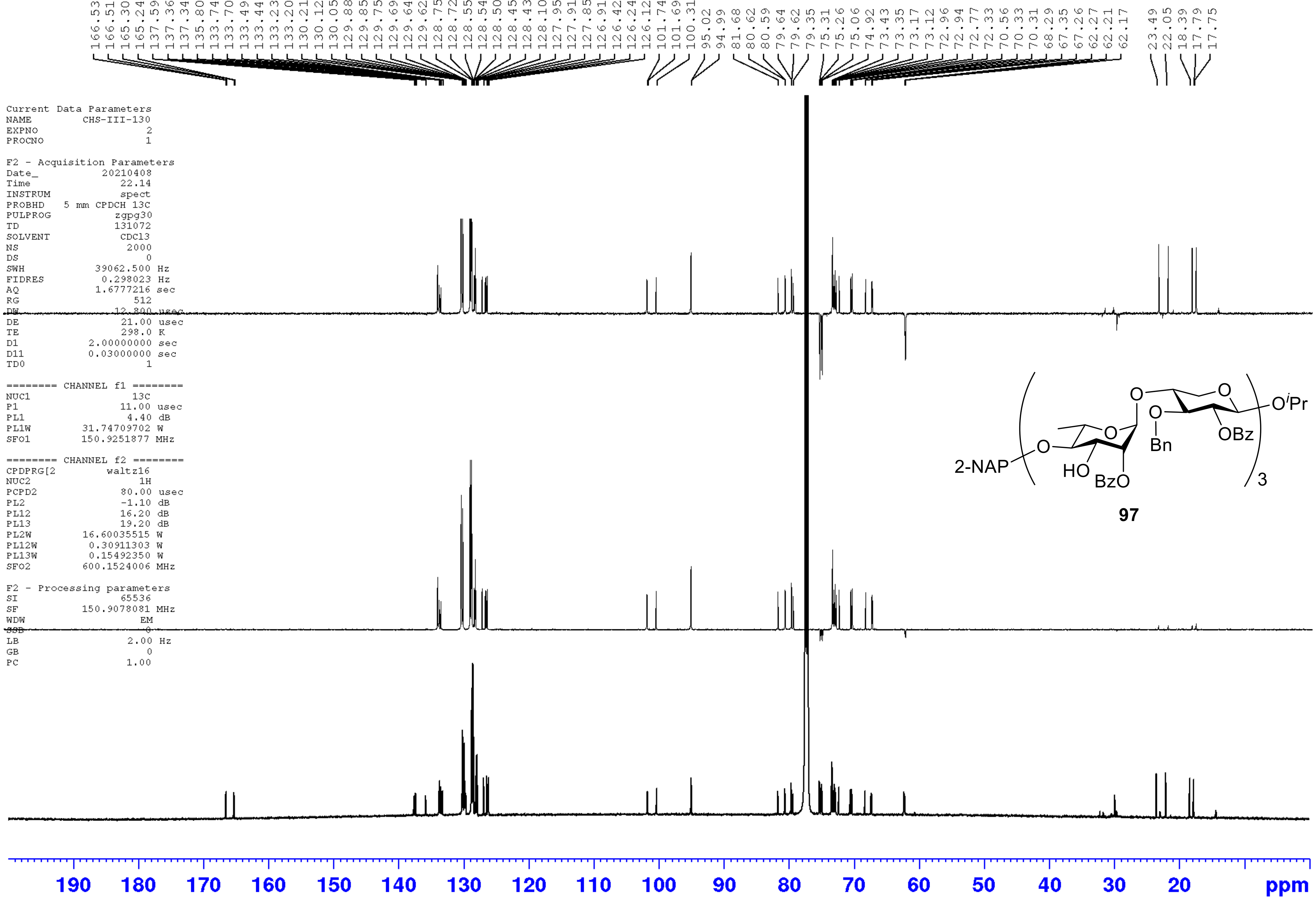
166.53
166.51
165.30
165.24
137.59
137.36
137.34
135.80
133.74
133.70
133.49
133.44
133.23
133.20
130.21
130.12
130.05
129.88
129.85
129.75
129.69
129.64
129.62
128.75
128.72
128.55
128.54
128.50
128.45
128.43
128.10
127.95
127.91
127.85
126.91
126.42
126.24
126.12
101.74
101.69
100.31
95.02
94.99
81.68
80.62
80.59
79.64
79.62
79.35
75.31
75.26
75.06
74.92
73.43
73.35
73.17
73.12
72.96
72.94
72.77
72.33
70.56
70.33
70.31
68.29
67.35
67.26
62.27
62.21
62.17
23.49
22.05
18.39
17.79
17.75
Current Data Parameters
NAME CHS-III-130
EXPNO 2
PROCNO 1
F2 - Acquisition Parameters
Date_ 20210408
Time 22.14
INSTRUM spect
PROBHD 5 mm CPDCH 13C
PULPROG zgpg30
TD 131072
SOLVENT CDCl3
NS 2000
DS 0
SWH 39062.500 Hz
FIDRES 0.298023 Hz
AQ 1.6777216 sec
RG 512
DW 12.800 usec
DE 21.00 usec
TE 298.0 K
D1 2.00000000 sec
D11 0.03000000 sec
TD0 1
======== CHANNEL f1 ========
NUC1 13C
P1 11.00 usec
PL1 4.40 dB
PL1W 31.74709702 W
SFO1 150.9251877 MHz
======== CHANNEL f2 ========
CPDPRG[2 waltz16
NUC2 1H
PCPD2 80.00 usec
PL2 -1.10 dB
PL12 16.20 dB
PL13 19.20 dB
PL2W 16.60035515 W
PL12W 0.30911303 W
PL13W 0.15492350 W
SFO2 600.1524006 MHz
F2 - Processing parameters
SI 65536
SF 150.9078081 MHz
WDW EM
SSB 0
LB 2.00 Hz
GB 0
PC 1.00
190
180
170
160
150
140
130
120
110
100
90
80
70
60
50
40
30
20
ppm
O
O
O
OiPr
OBz
Bn
2-NAP
O
HO
BzO
O
3

97

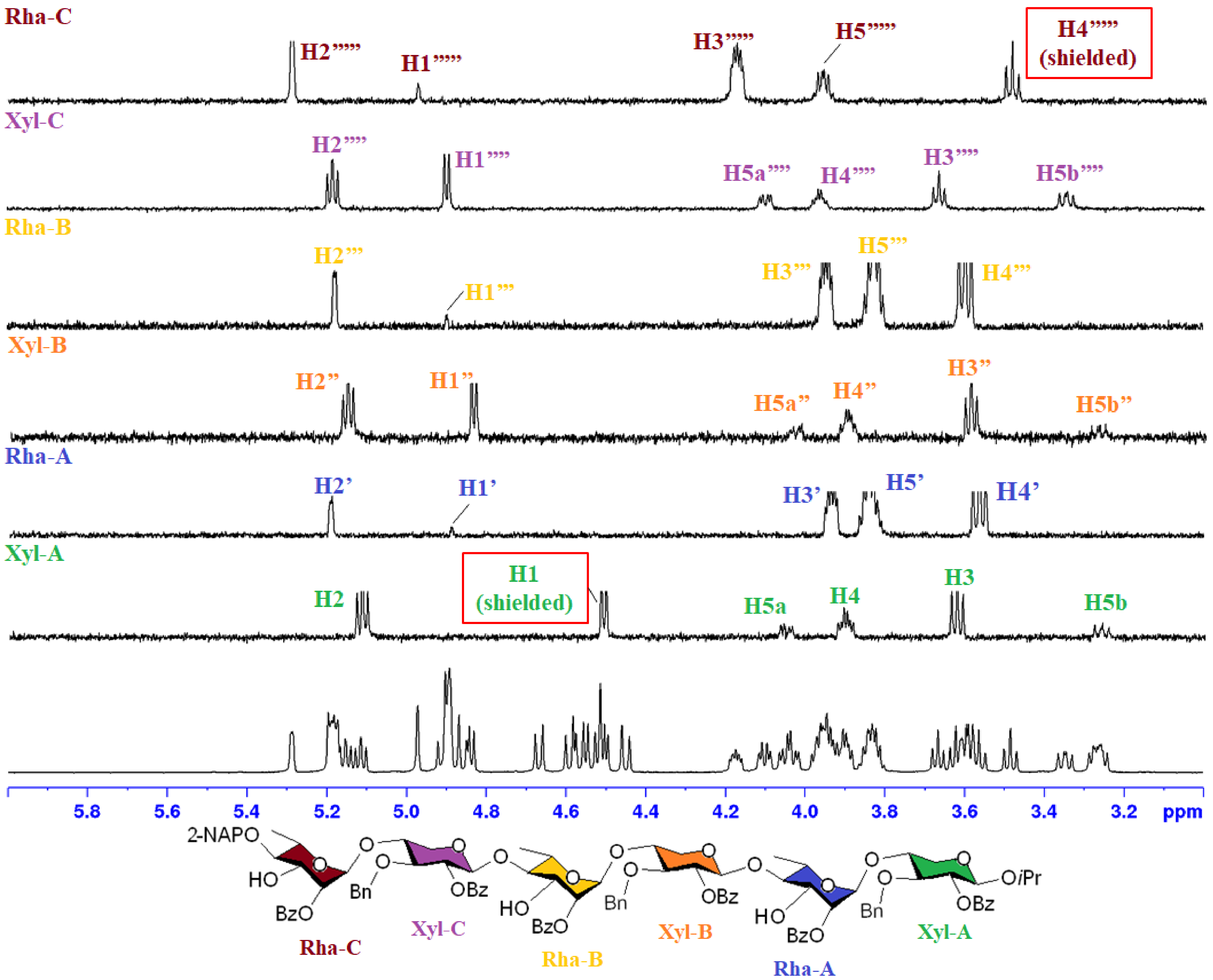

Rha-C
H2'''''
H1'''''
H3'''''
H5'''''
H4'''''
(shielded)
Xyl-C
H2''''
H1''''
H5a''''
H4''''
H3''''
H5b''''
Rha-B
H2'''
H1'''
H3'''
H5'''
H4'''
Xyl-B
H2''
H1''
H5a''
H4''
H3''
H5b''
Rha-A
H2'
H1'
H3'
H5'
H4'
Xyl-A
H2
H1
(shielded)
H5a
H4
H3
H5b
5.8
5.6
5.4
5.2
5.0
4.8
4.6
4.4
4.2
4.0
3.8
3.6
3.4
3.2
ppm
2-NAPO
HO
BzO
Bn
OBz
HO
BzO
Bn
OBz
HO
BzO
Bn
OBz
OiPr
Rha-C
Xyl-C
Rha-B
Xyl-B
Rha-A
Xyl-A

97

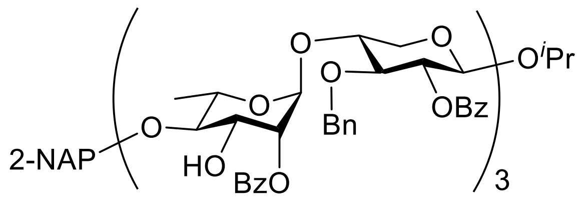

97

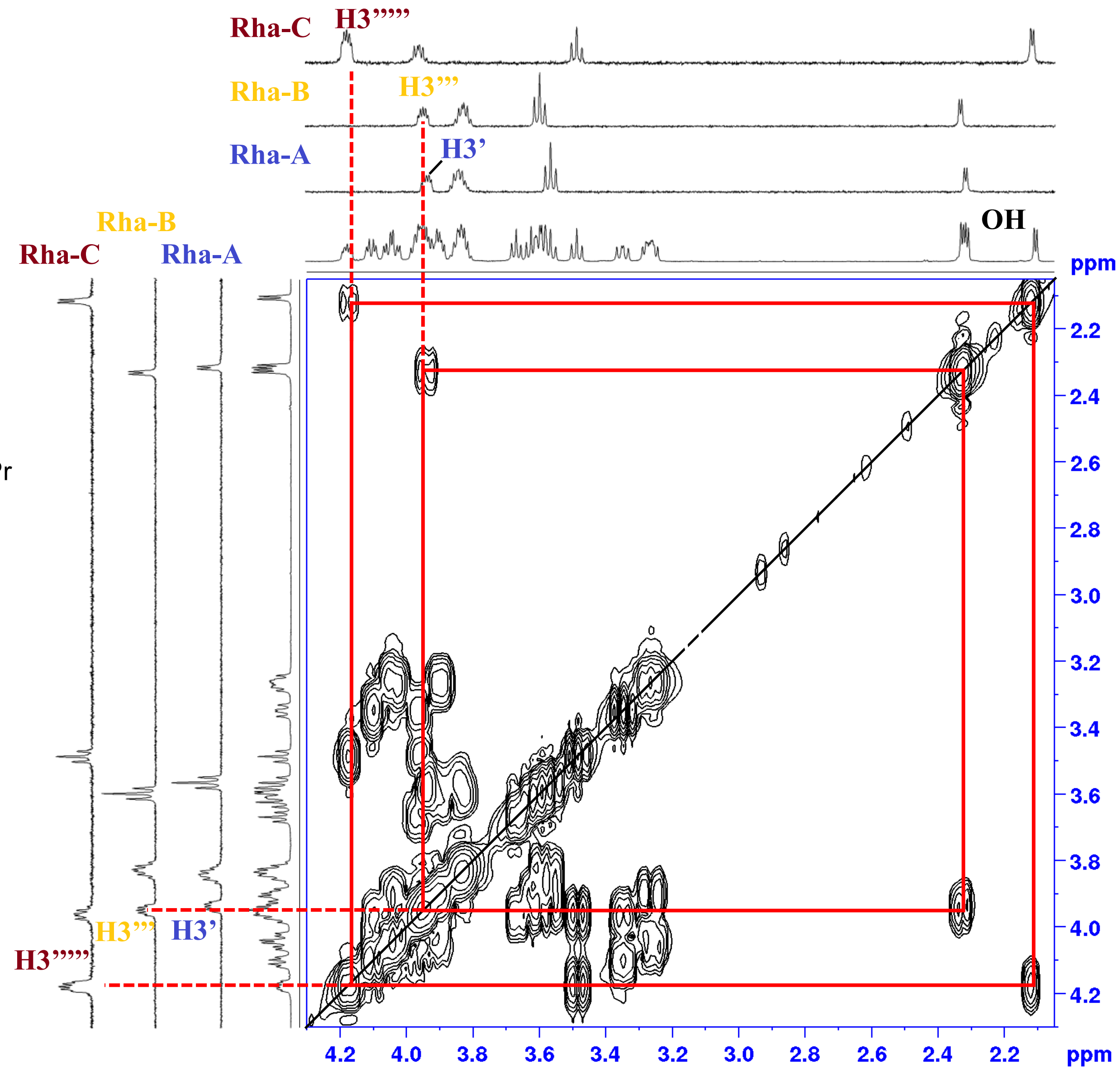

Rha-C
H3'''''
Rha-B
H3'''
Rha-A
H3'
OH
ppm
2.2
2.4
2.6
2.8
3.0
3.2
3.4
3.6
3.8
4.0
4.2
4.2
4.0
3.8
3.6
3.4
3.2
3.0
2.8
2.6
2.4
2.2
ppm

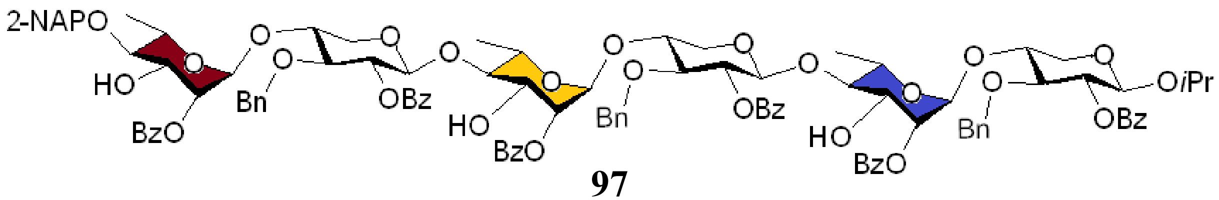

97

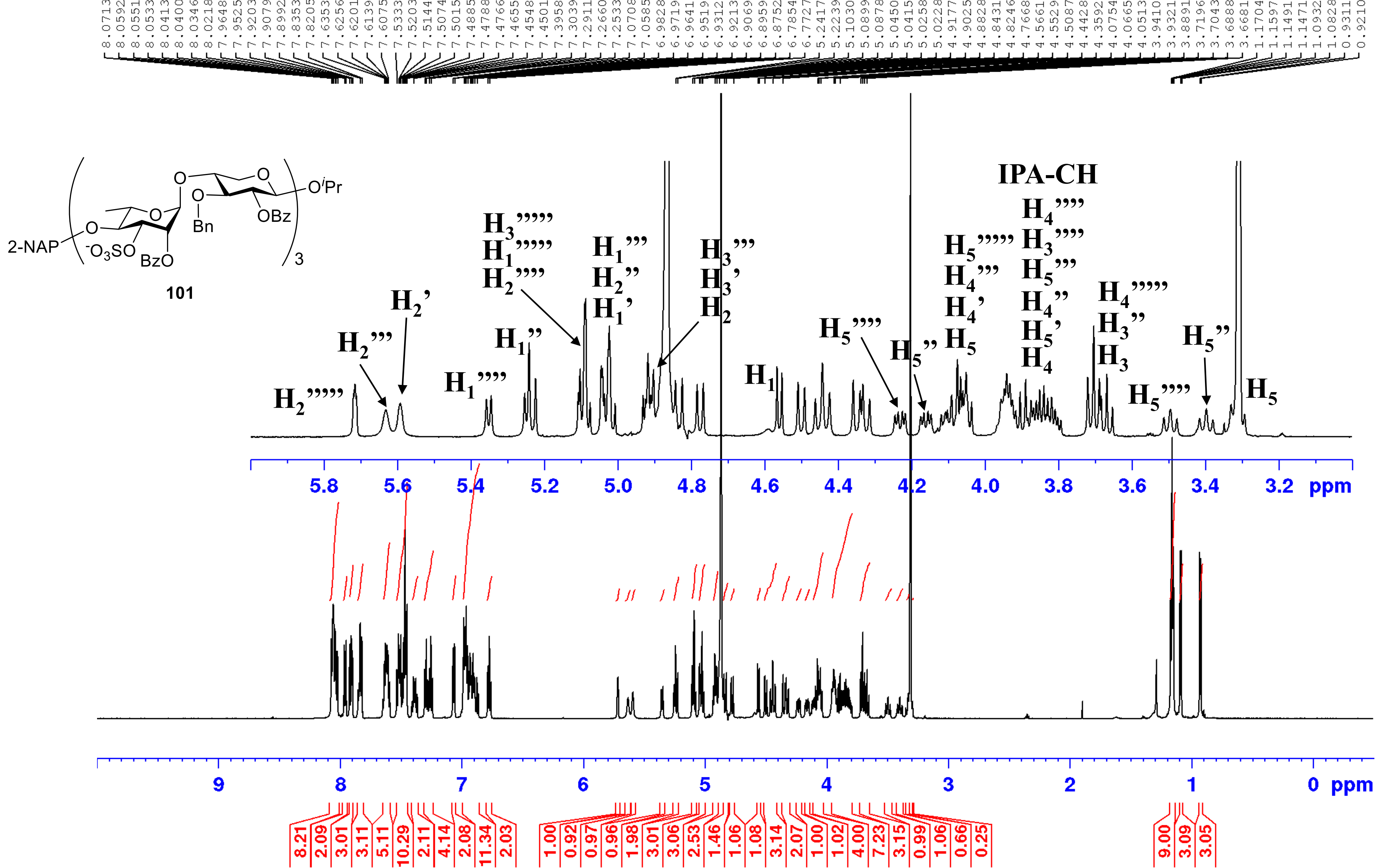
2-NAP
OiPr
OBz
Bn
BzO
101
IPA-CH
ppm

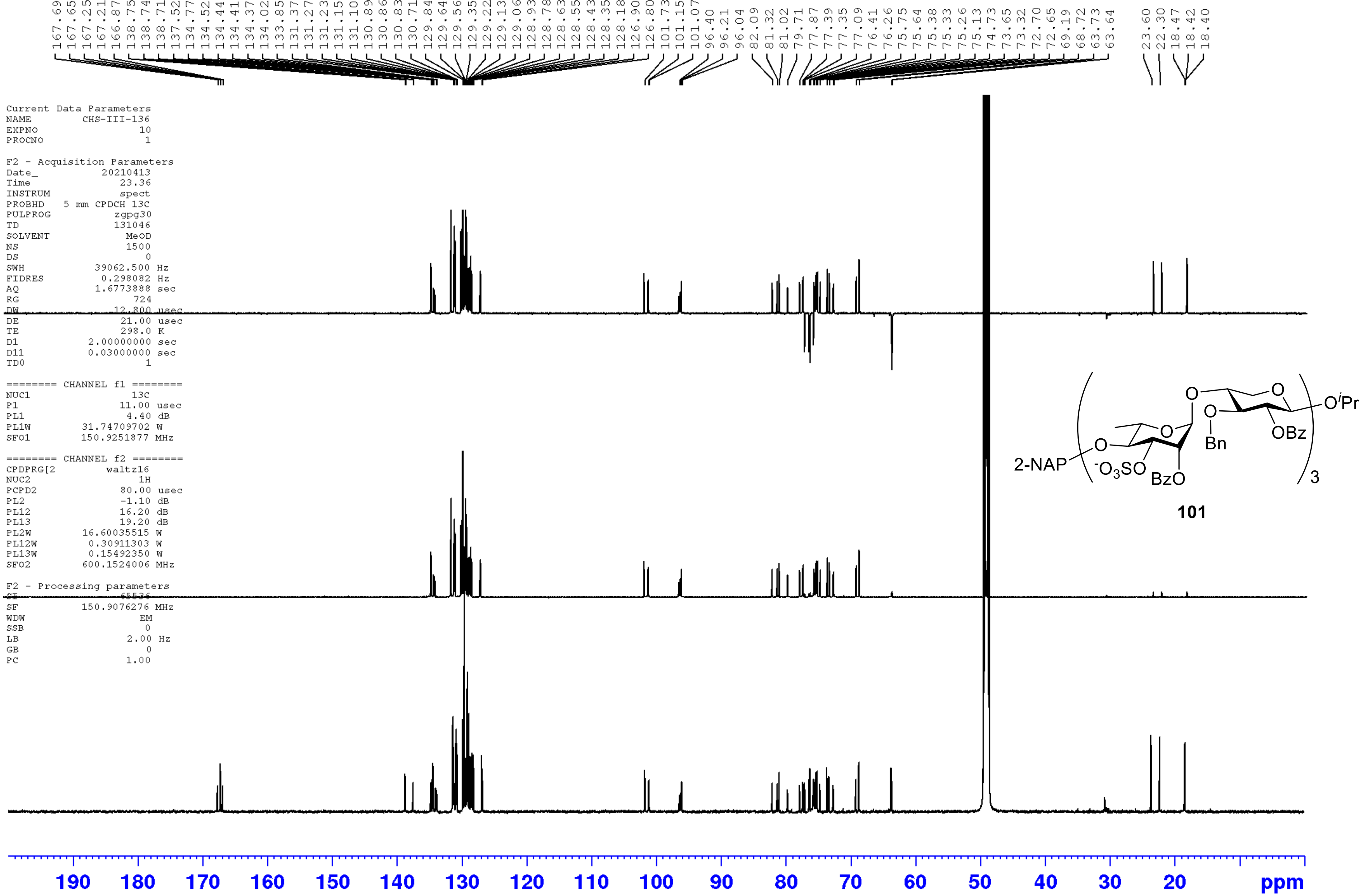
167.69
167.65
167.25
167.21
166.87
138.75
138.74
138.71
137.52
134.77
134.52
134.44
134.41
134.37
134.02
133.85
131.37
131.27
131.23
131.15
131.10
130.89
130.86
130.83
130.71
129.84
129.64
129.56
129.35
129.22
129.13
129.06
128.93
128.78
128.63
128.55
128.43
128.35
128.18
126.90
126.80
101.73
101.15
101.07
96.40
96.21
96.04
82.09
81.32
81.02
79.71
77.87
77.39
77.35
77.09
76.41
76.26
75.75
75.64
75.38
75.33
75.26
75.13
74.73
73.65
73.32
72.70
72.65
69.19
68.72
63.73
63.64
23.60
22.30
18.47
18.42
18.40
Current Data Parameters
NAME CHS-III-136
EXPNO 10
PROCNO 1
F2 - Acquisition Parameters
Date_ 20210413
Time 23.36
INSTRUM spect
PROBHD 5 mm CPDCH 13C
PULPROG zgpg30
TD 131046
SOLVENT MeOD
NS 1500
DS 0
SWH 39062.500 Hz
FIDRES 0.298082 Hz
AQ 1.6773888 sec
RG 724
DW 12.800 usec
DE 21.00 usec
TE 298.0 K
D1 2.00000000 sec
D11 0.03000000 sec
TD0 1
======== CHANNEL f1 ========
NUC1 13C
P1 11.00 usec
PL1 4.40 dB
PL1W 31.74709702 W
SFO1 150.9251877 MHz
======== CHANNEL f2 ========
CPDPRG[2 waltz16
NUC2 1H
PCPD2 80.00 usec
PL2 -1.10 dB
PL12 16.20 dB
PL13 19.20 dB
PL2W 16.60035515 W
PL12W 0.30911303 W
PL13W 0.15492350 W
SFO2 600.1524006 MHz
F2 - Processing parameters
SI 65536
SF 150.9076276 MHz
WDW EM
SSB 0
LB 2.00 Hz
GB 0
PC 1.00
2-NAP
O
-O3SO
BzO
O
O
O
Bn
O
OBz
OiPr
3
101
190 180 170 160 150 140 130 120 110 100 90 80 70 60 50 40 30 20 ppm

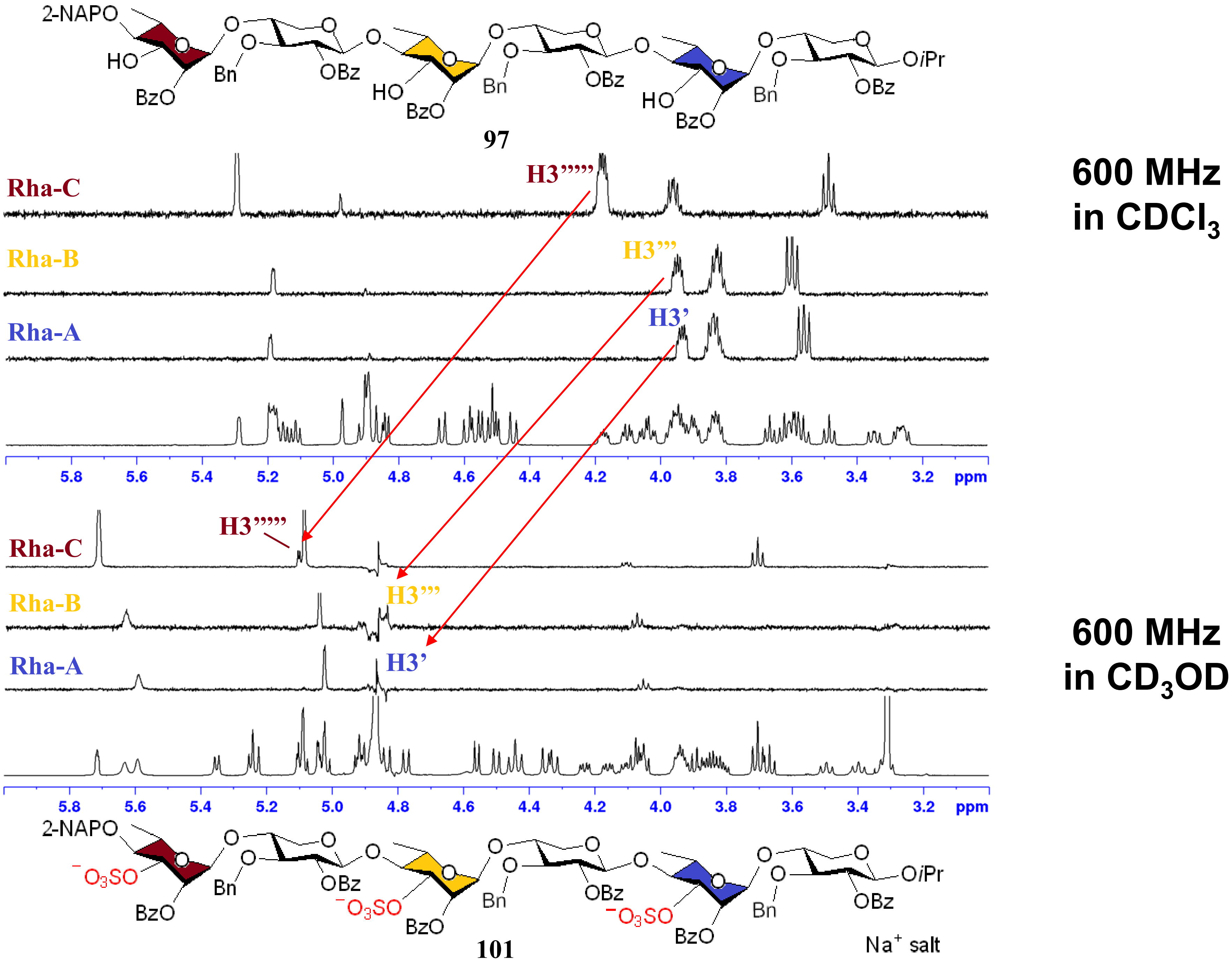

2-NAPO
HO
BzO
Bn
OBz
HO
BzO
Bn
OBz
HO
BzO
Bn
OBz
OiPr
97
600 MHz in $CDCl_3$
Rha-C
H3”””
Rha-B
H3’’’
Rha-A
H3’
5.8
5.6
5.4
5.2
5.0
4.8
4.6
4.4
4.2
4.0
3.8
3.6
3.4
3.2
ppm
600 MHz in CD_3OD
Rha-C
H3”””
Rha-B
H3’’’
Rha-A
H3’
2-NAPO
$^{-}O_3SO$
BzO
Bn
OBz
$^{-}O_3SO$
BzO
Bn
OBz
$^{-}O_3SO$
BzO
Bn
OBz
OiPr
Na^+ salt
101

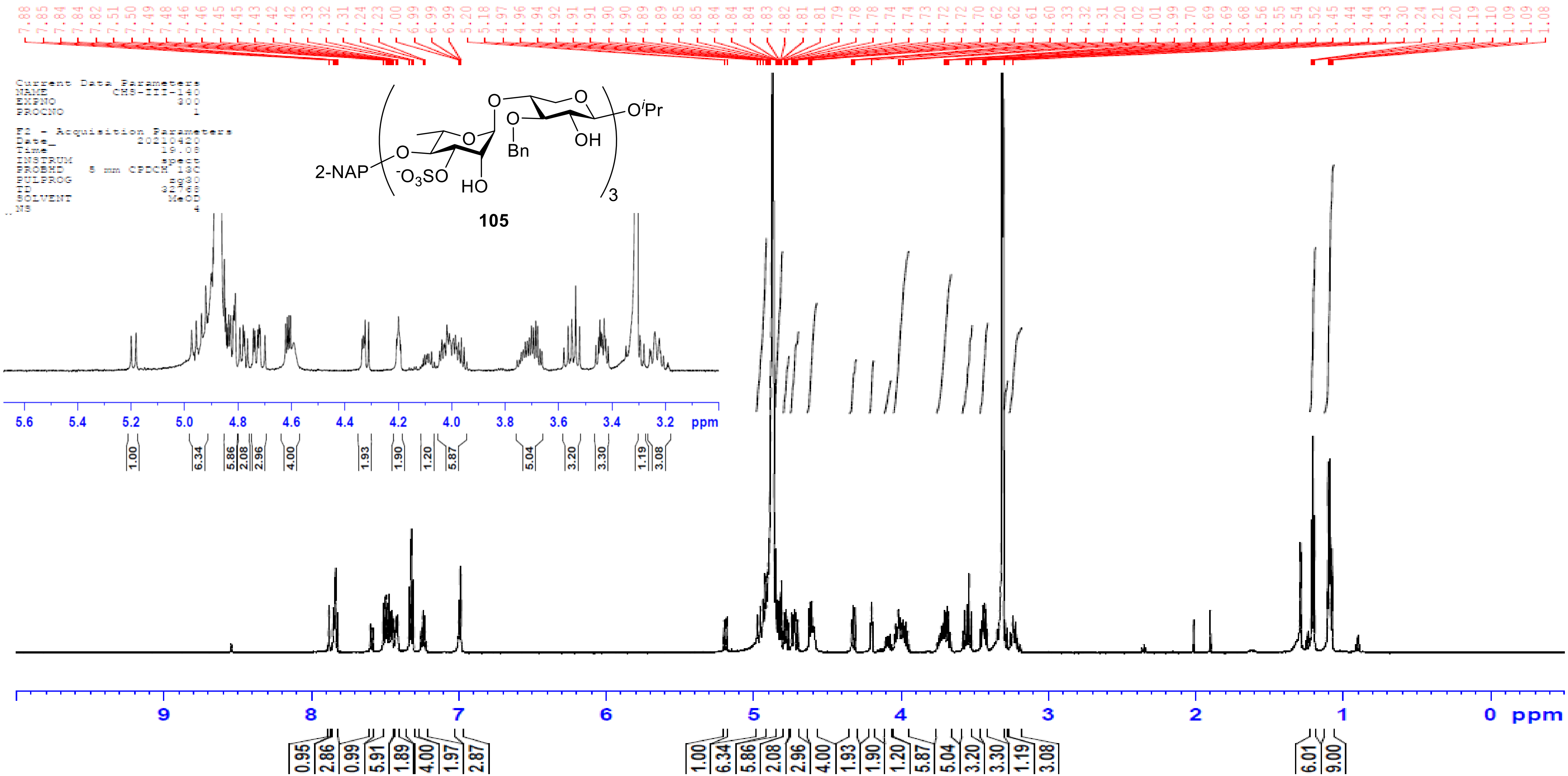
Current Data Parameters
NAME CHS-III-140
EXPNO 300
PROCNO 1
F2 - Acquisition Parameters
Date_ 20210420
Time 19.08
INSTRUM spect
PROBHD 5 mm CPDCH 13C
PULPROG zg30
TD 32768
SOLVENT MeOD
NS 4
2-NAP
$^{-}O_3SO$
HO
Bn
OH
O^iPr
3
105
ppm

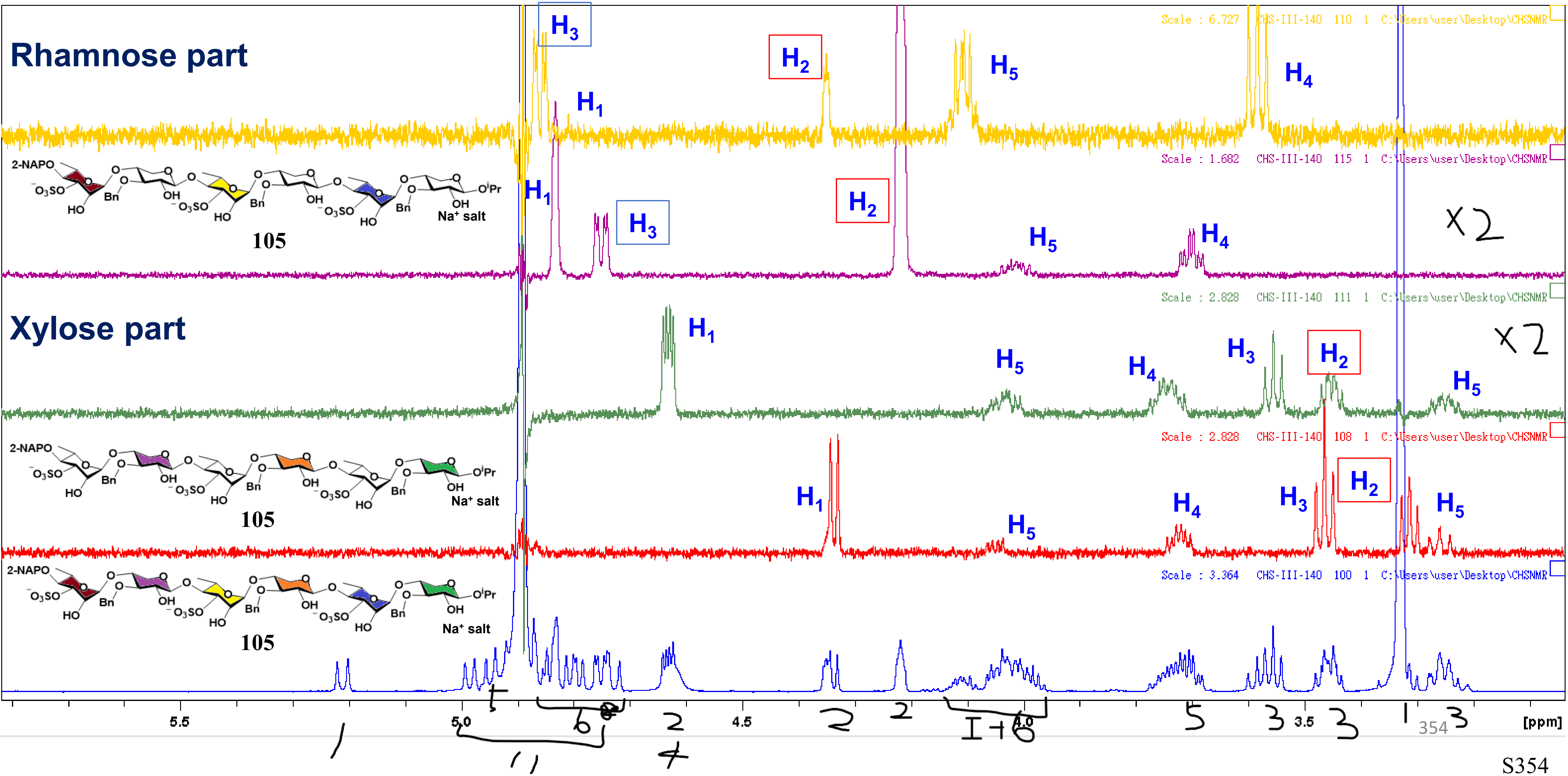

Rhamnose part
Xylose part
2-NAPO
105
Na+ salt
Scale : 6.727 CHS-III-140 110 1
Scale : 1.682 CHS-III-140 115 1
Scale : 2.828 CHS-III-140 111 1
Scale : 2.828 CHS-III-140 108 1
Scale : 3.364 CHS-III-140 100 1
x2
5.5
5.0
4.5
4.0
3.5
[ppm]

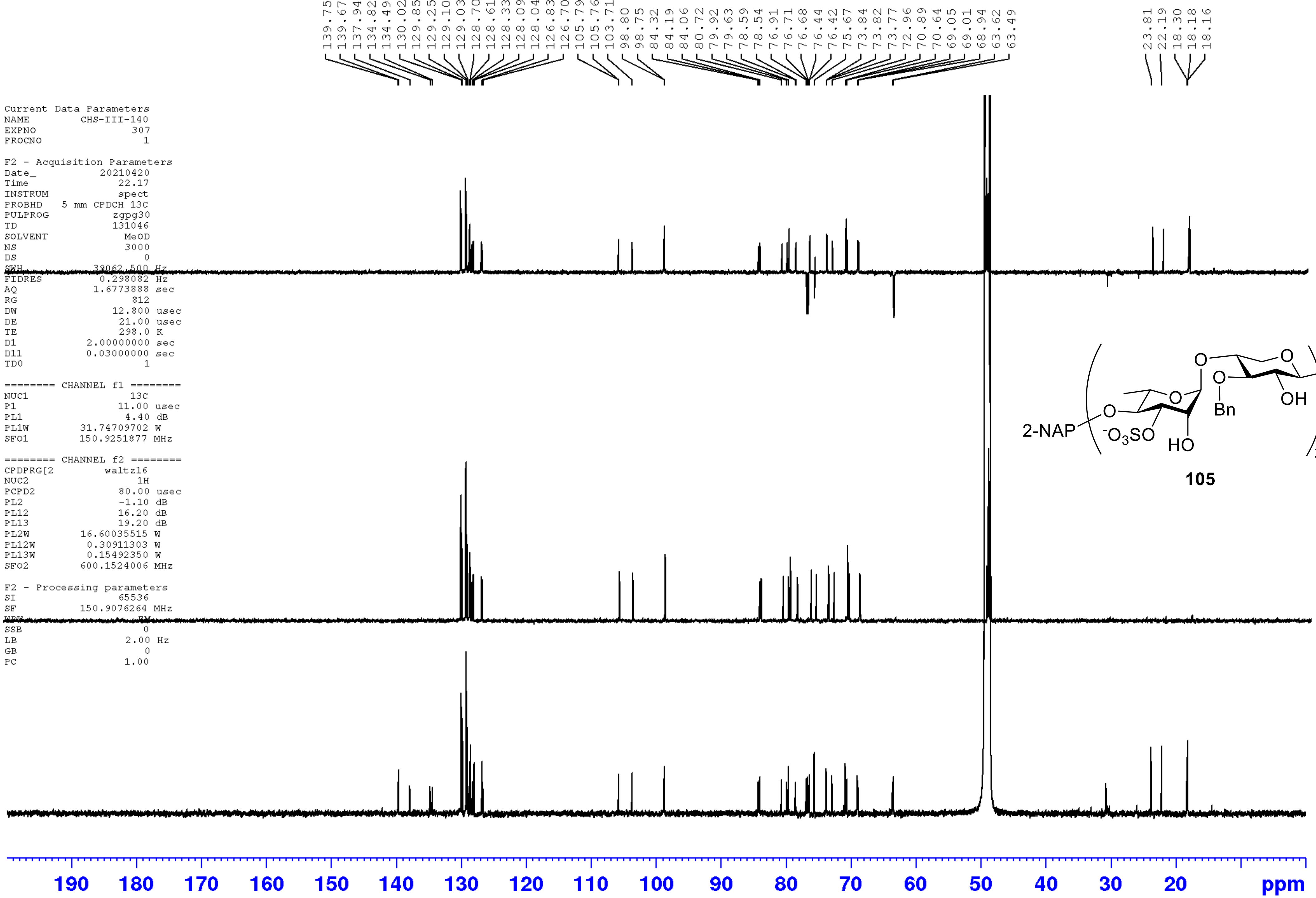
139.75
139.67
137.94
134.82
134.49
130.02
129.85
129.25
129.10
129.03
128.70
128.61
128.33
128.09
128.04
126.83
126.70
105.79
105.76
103.71
98.80
98.75
84.32
84.19
84.06
80.72
79.92
79.63
78.59
78.54
76.91
76.71
76.68
76.44
76.42
75.67
73.84
73.82
73.77
72.96
70.89
70.64
69.05
69.01
68.94
63.62
63.49
23.81
22.19
18.30
18.18
18.16
Current Data Parameters
NAME CHS-III-140
EXPNO 307
PROCNO 1
F2 - Acquisition Parameters
Date_ 20210420
Time 22.17
INSTRUM spect
PROBHD 5 mm CPDCH 13C
PULPROG zgpg30
TD 131046
SOLVENT MeOD
NS 3000
DS 0
SWH 39062.500 Hz
FIDRES 0.298082 Hz
AQ 1.6773888 sec
RG 812
DW 12.800 usec
DE 21.00 usec
TE 298.0 K
D1 2.00000000 sec
D11 0.03000000 sec
TD0 1
======== CHANNEL f1 ========
NUC1 13C
P1 11.00 usec
PL1 4.40 dB
PL1W 31.74709702 W
SFO1 150.9251877 MHz
======== CHANNEL f2 ========
CPDPRG[2 waltz16
NUC2 1H
PCPD2 80.00 usec
PL2 -1.10 dB
PL12 16.20 dB
PL13 19.20 dB
PL2W 16.60035515 W
PL12W 0.30911303 W
PL13W 0.15492350 W
SFO2 600.1524006 MHz
F2 - Processing parameters
SI 65536
SF 150.9076264 MHz
SSB 0
LB 2.00 Hz
GB 0
PC 1.00
190 180 170 160 150 140 130 120 110 100 90 80 70 60 50 40 30 20 ppm
O
O
O
O^iPr
O
Bn
OH
2-NAP
O
^-O_3SO
HO
3

105

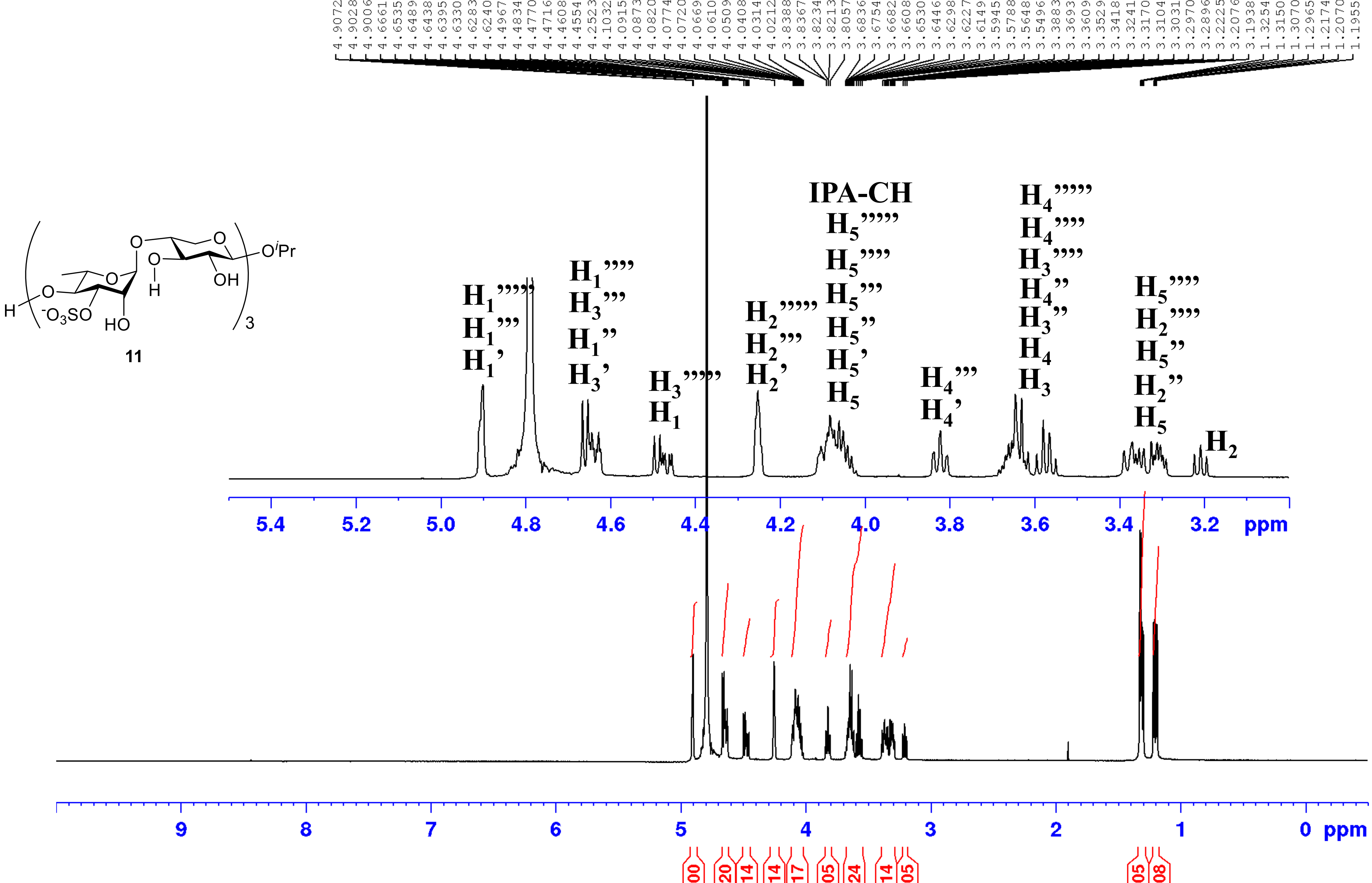
11
IPA-CH
ppm

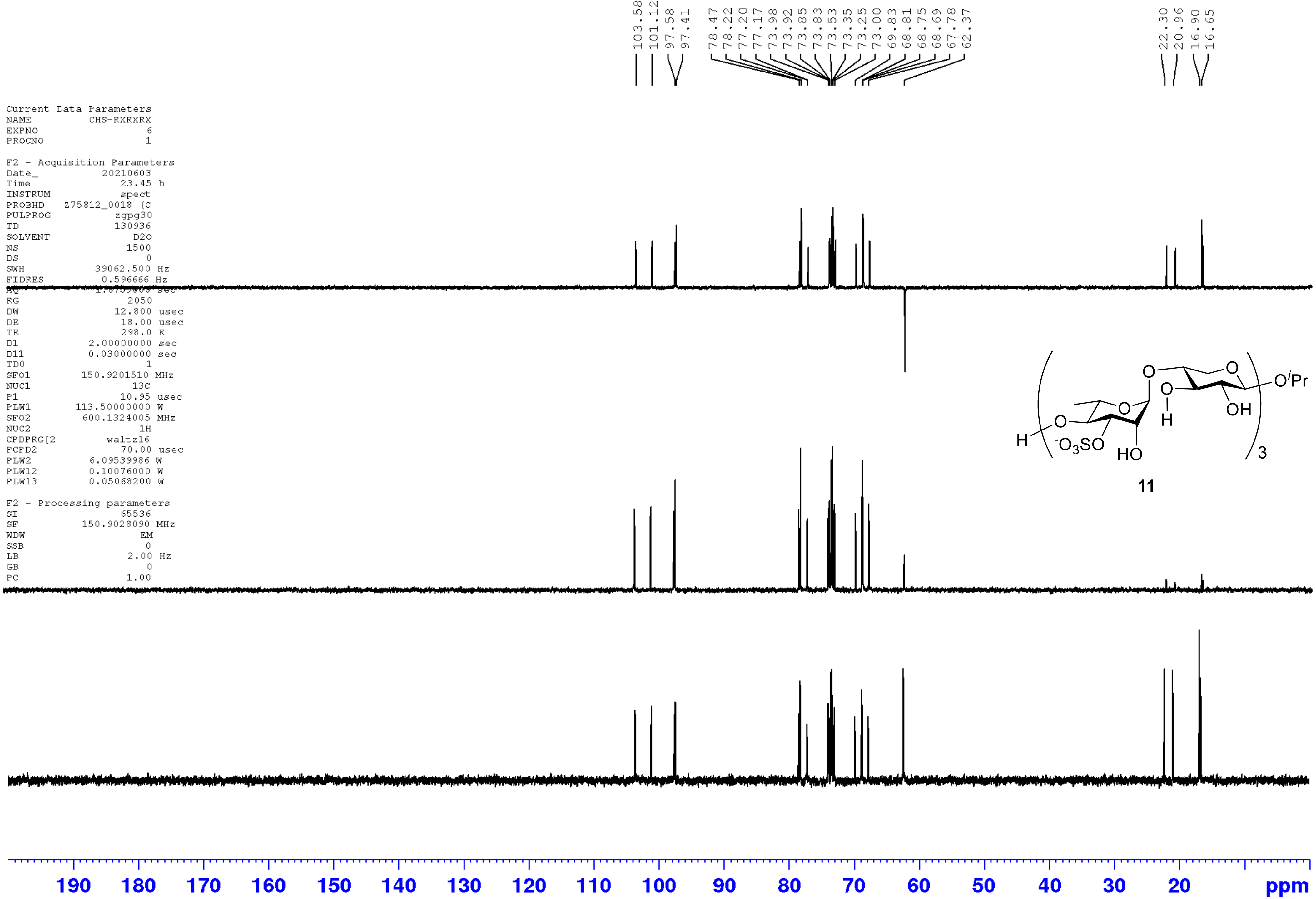
103.58
101.12
97.58
97.41
78.47
78.22
77.20
77.17
73.98
73.92
73.85
73.83
73.53
73.35
73.25
73.00
69.83
68.81
68.75
68.69
67.78
62.37
22.30
20.96
16.90
16.65
Current Data Parameters
NAME CHS-RXRXRX
EXPNO 6
PROCNO 1
F2 - Acquisition Parameters
Date_ 20210603
Time 23.45 h
INSTRUM spect
PROBHD Z75812_0018 (C
PULPROG zgpg30
TD 130936
SOLVENT D2O
NS 1500
DS 0
SWH 39062.500 Hz
FIDRES 0.596666 Hz
RG 2050
DW 12.800 usec
DE 18.00 usec
TE 298.0 K
D1 2.00000000 sec
D11 0.03000000 sec
TD0 1
SFO1 150.9201510 MHz
NUC1 13C
P1 10.95 usec
PLW1 113.50000000 W
SFO2 600.1324005 MHz
NUC2 1H
CPDPRG[2 waltz16
PCPD2 70.00 usec
PLW2 6.09539986 W
PLW12 0.10076000 W
PLW13 0.05068200 W
F2 - Processing parameters
SI 65536
SF 150.9028090 MHz
WDW EM
SSB 0
LB 2.00 Hz
GB 0
PC 1.00
OiPr
OH
H
-O3SO
HO
3
190 180 170 160 150 140 130 120 110 100 90 80 70 60 50 40 30 20 ppm

11

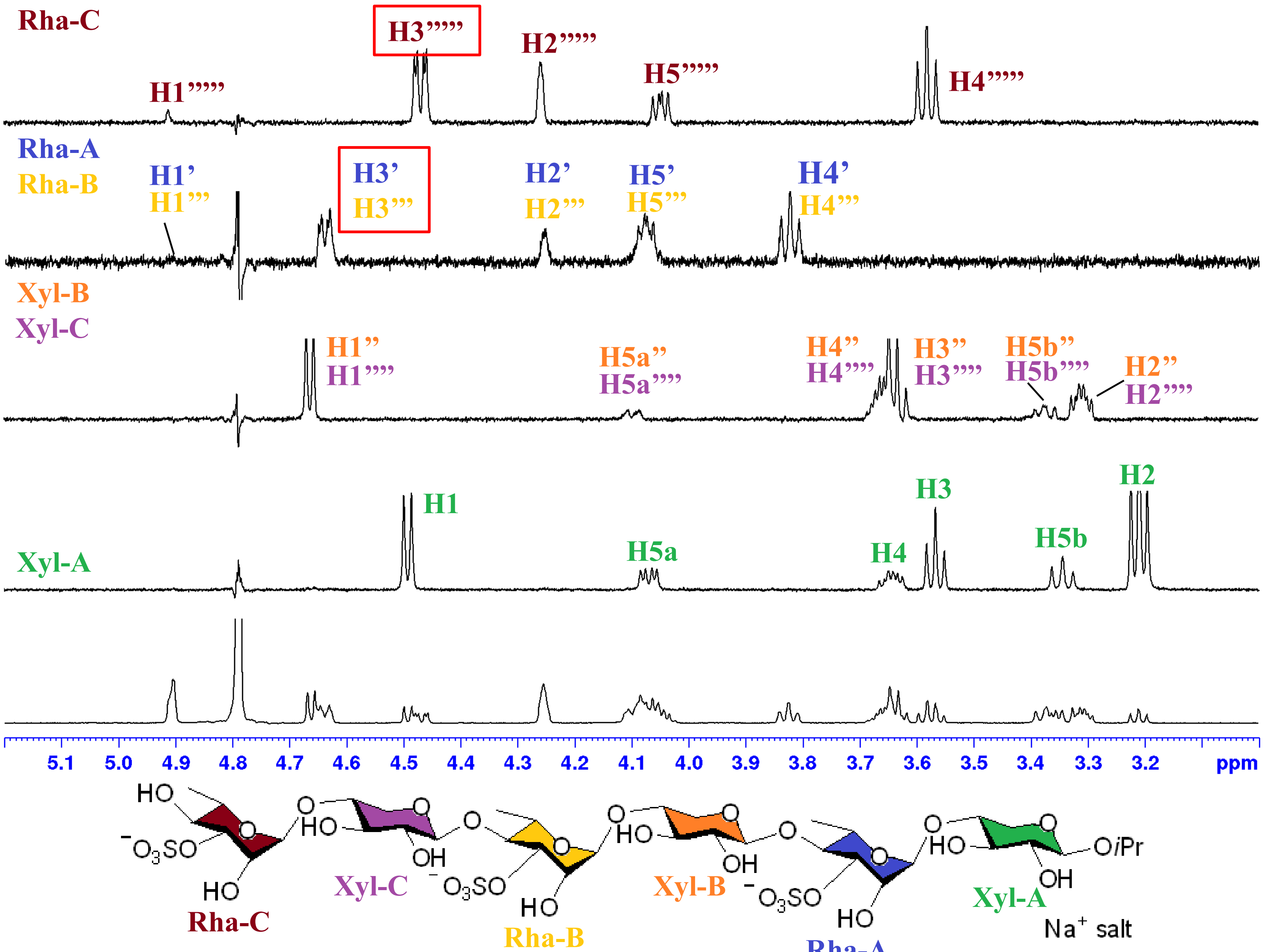

Rha-C
H3'''''
H2'''''
H1'''''
H5'''''
H4'''''
Rha-A
Rha-B
H1'
H1'''
H3'
H3'''
H2'
H2'''
H5'
H5'''
H4'
H4'''
Xyl-B
Xyl-C
H1''
H1''''
H5a''
H5a''''
H4''
H4''''
H3''
H3''''
H5b''
H5b''''
H2''
H2''''
H1
H5a
H4
H3
H5b
H2
Xyl-A
5.1 5.0 4.9 4.8 4.7 4.6 4.5 4.4 4.3 4.2 4.1 4.0 3.9 3.8 3.7 3.6 3.5 3.4 3.3 3.2 ppm
Rha-C
Xyl-C
Rha-B
Xyl-B
Rha-A
Xyl-A
OiPr
Na+ salt

11

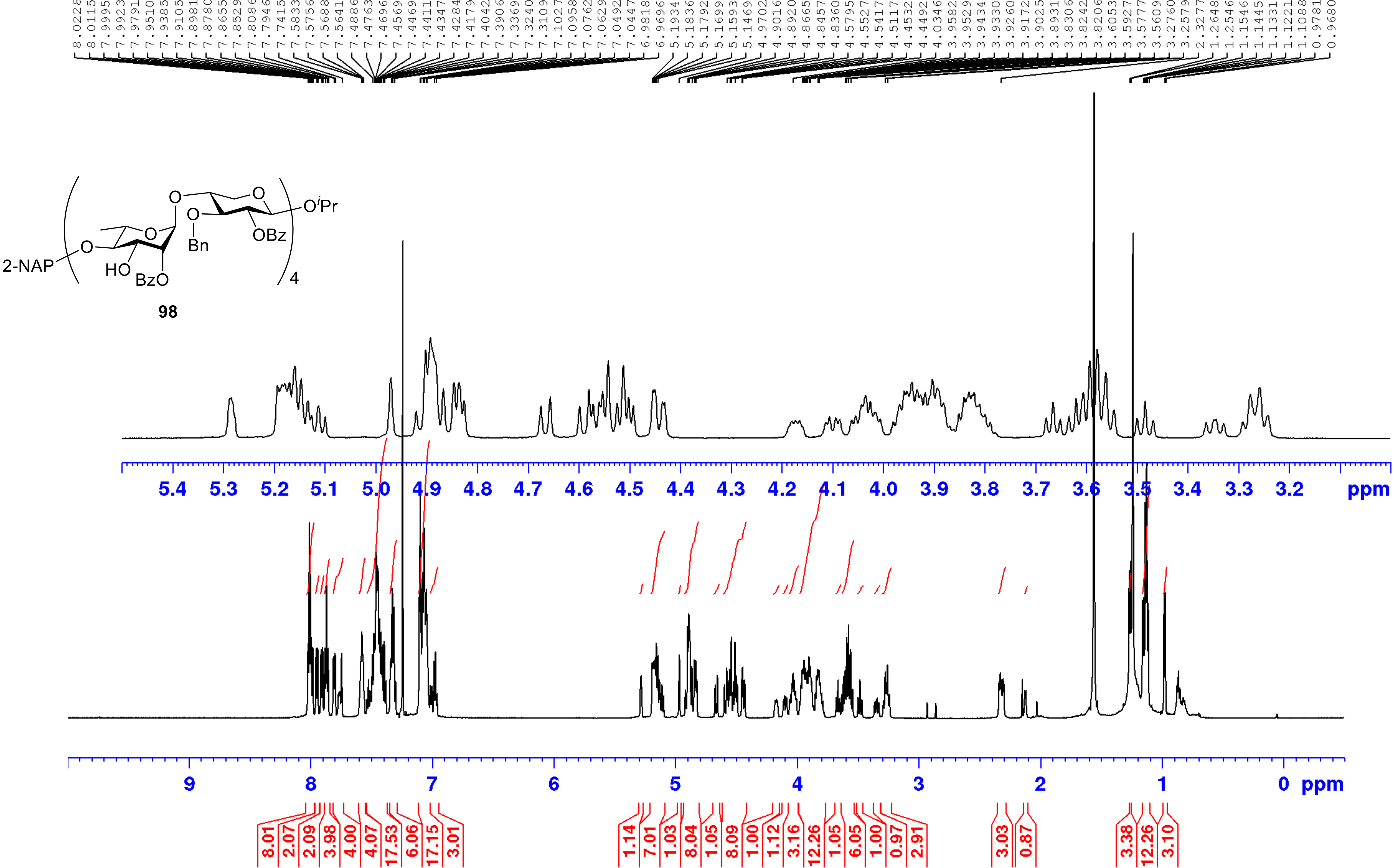

98

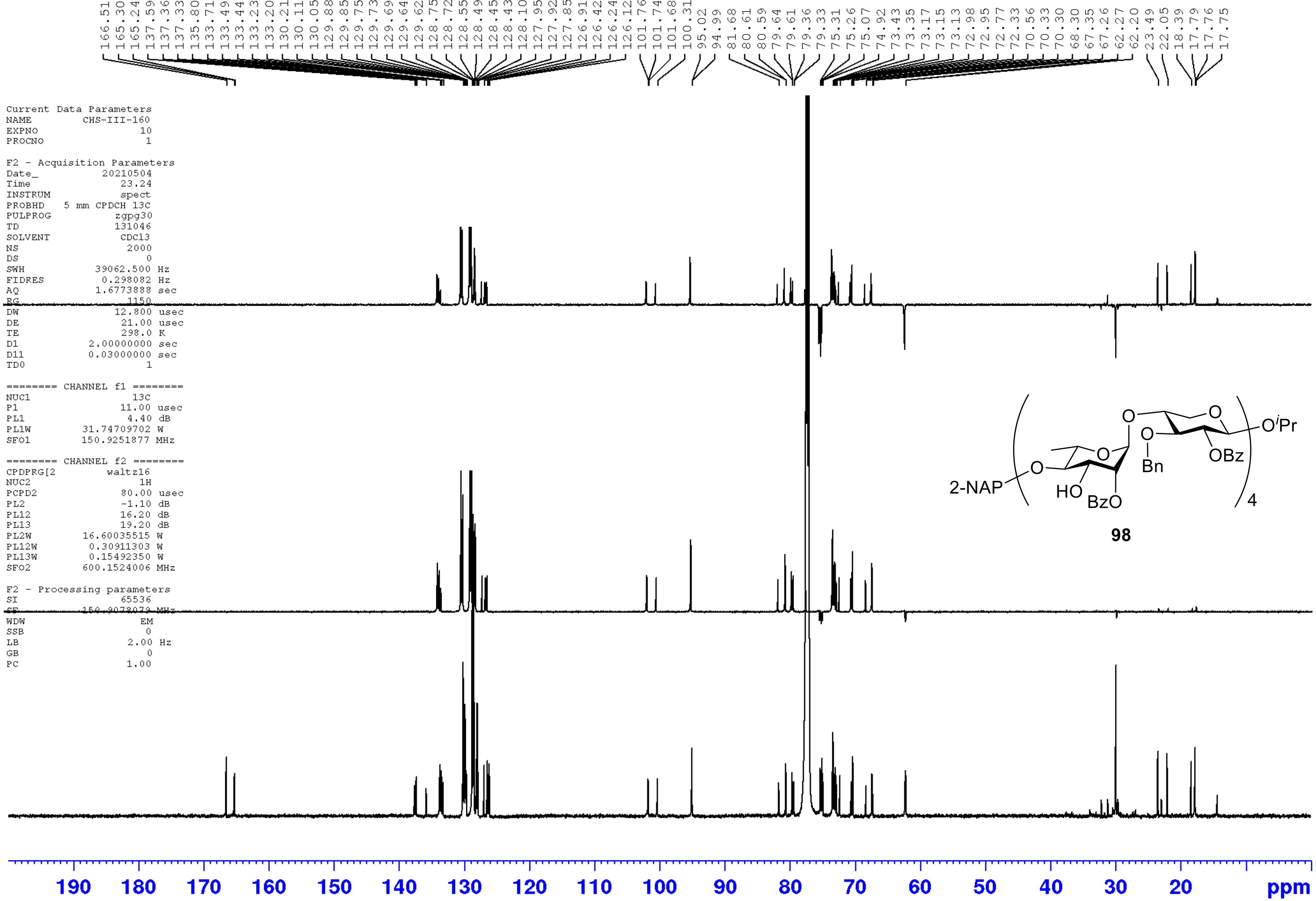

166.51
165.30
165.24
137.59
137.36
137.33
135.80
133.71
133.49
133.44
133.23
133.20
130.21
130.11
130.05
129.88
129.85
129.75
129.73
129.69
129.64
129.62
128.75
128.72
128.55
128.49
128.45
128.43
128.10
127.95
127.92
127.85
126.91
126.42
126.24
126.12
101.76
101.74
101.68
100.31
95.02
94.99
81.68
80.61
80.59
79.64
79.61
79.36
79.33
75.31
75.26
75.07
74.92
73.43
73.35
73.17
73.15
73.13
72.98
72.95
72.77
72.33
70.56
70.33
70.30
68.35
67.35
67.26
62.27
62.20
23.49
22.05
18.39
17.79
17.76
17.75
Current Data Parameters
NAME CHS-III-160
EXPNO 10
PROCNO 1
F2 - Acquisition Parameters
Date_ 20210504
Time 23.24
INSTRUM spect
PROBHD 5 mm CPDCH 13C
PULPROG zgpg30
TD 131046
SOLVENT CDCl3
NS 2000
DS 0
SWH 39062.500 Hz
FIDRES 0.298082 Hz
AQ 1.6773888 sec
RG 1150
DW 12.800 usec
DE 21.00 usec
TE 298.0 K
D1 2.00000000 sec
D11 0.03000000 sec
TD0 1
======== CHANNEL f1 ========
NUC1 13C
P1 11.00 usec
PL1 4.40 dB
PL1W 31.74709702 W
SFO1 150.9251877 MHz
======== CHANNEL f2 ========
CPDPRG[2 waltz16
NUC2 1H
PCPD2 80.00 usec
PL2 -1.10 dB
PL12 16.20 dB
PL13 19.20 dB
PL2W 16.60035515 W
PL12W 0.30911303 W
PL13W 0.15492350 W
SFO2 600.1524006 MHz
F2 - Processing parameters
SI 65536
SF 150.9078079 MHz
WDW EM
SSB 0
LB 2.00 Hz
GB 0
PC 1.00
2-NAP
O
HO
BzO
O
O
O
Bn
O
OBz
O^{i}Pr
4
98
190 180 170 160 150 140 130 120 110 100 90 80 70 60 50 40 30 20 ppm

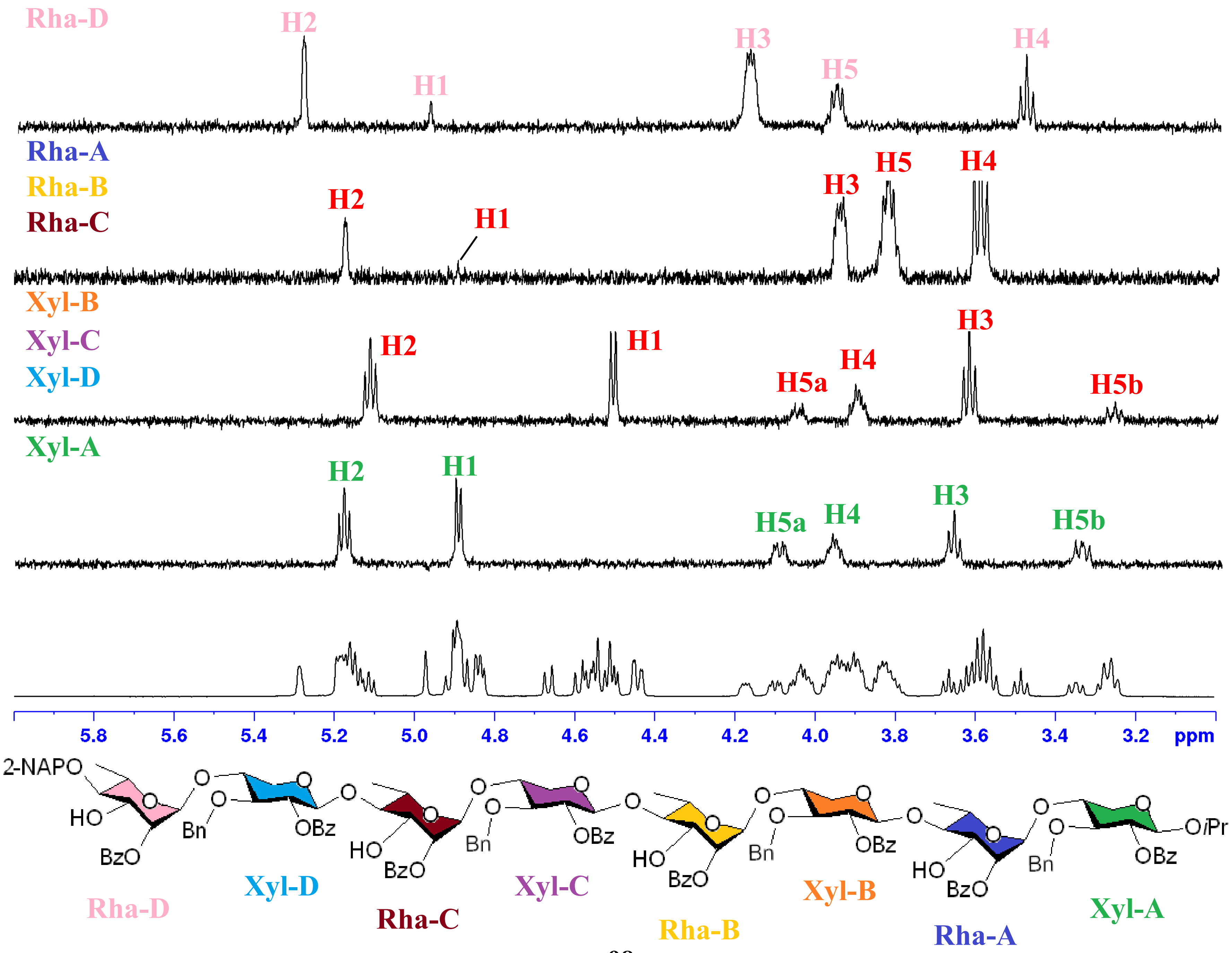
Rha-D
H2
H1
H3
H5
H4
Rha-A
Rha-B
Rha-C
H2
H1
H3
H5
H4
Xyl-B
Xyl-C
Xyl-D
H2
H1
H5a
H4
H3
H5b
Xyl-A
H2
H1
H5a
H4
H3
H5b
5.8
5.6
5.4
5.2
5.0
4.8
4.6
4.4
4.2
4.0
3.8
3.6
3.4
3.2
ppm
2-NAPO
HO
BzO
Bn
OBz
HO
BzO
Bn
OBz
HO
BzO
Bn
OBz
HO
BzO
Bn
OBz
O*i*Pr
Rha-D
Xyl-D
Rha-C
Xyl-C
Rha-B
Xyl-B
Rha-A
Xyl-A

98

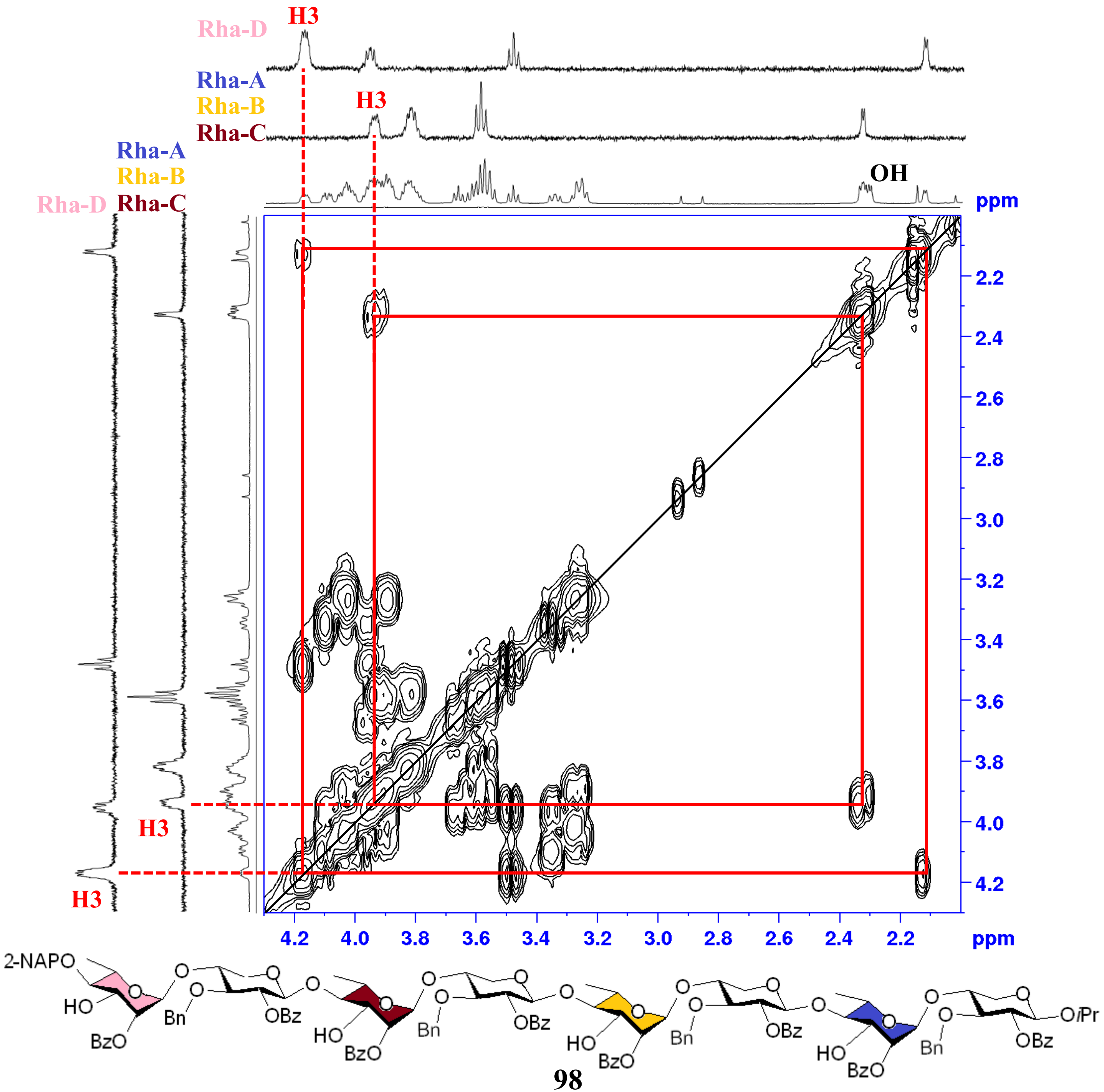

Rha-D
H3
Rha-A
Rha-B
Rha-C
H3
Rha-A
Rha-B
Rha-D Rha-C
OH
ppm
H3
H3
ppm
2-NAPO
HO
BzO
Bn
OBz
HO
BzO
Bn
OBz
HO
BzO
Bn
OBz
HO
BzO
Bn
OBz
OiPr

98

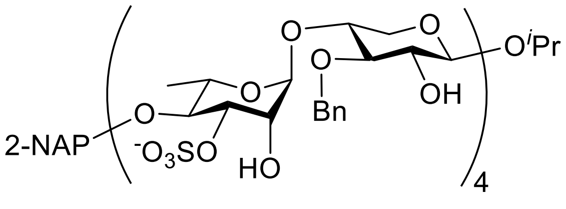

2-NAP
O
$^{-}$O$_3$SO
HO
O
O
O
Bn
O
OH
O^{i}Pr
4

106

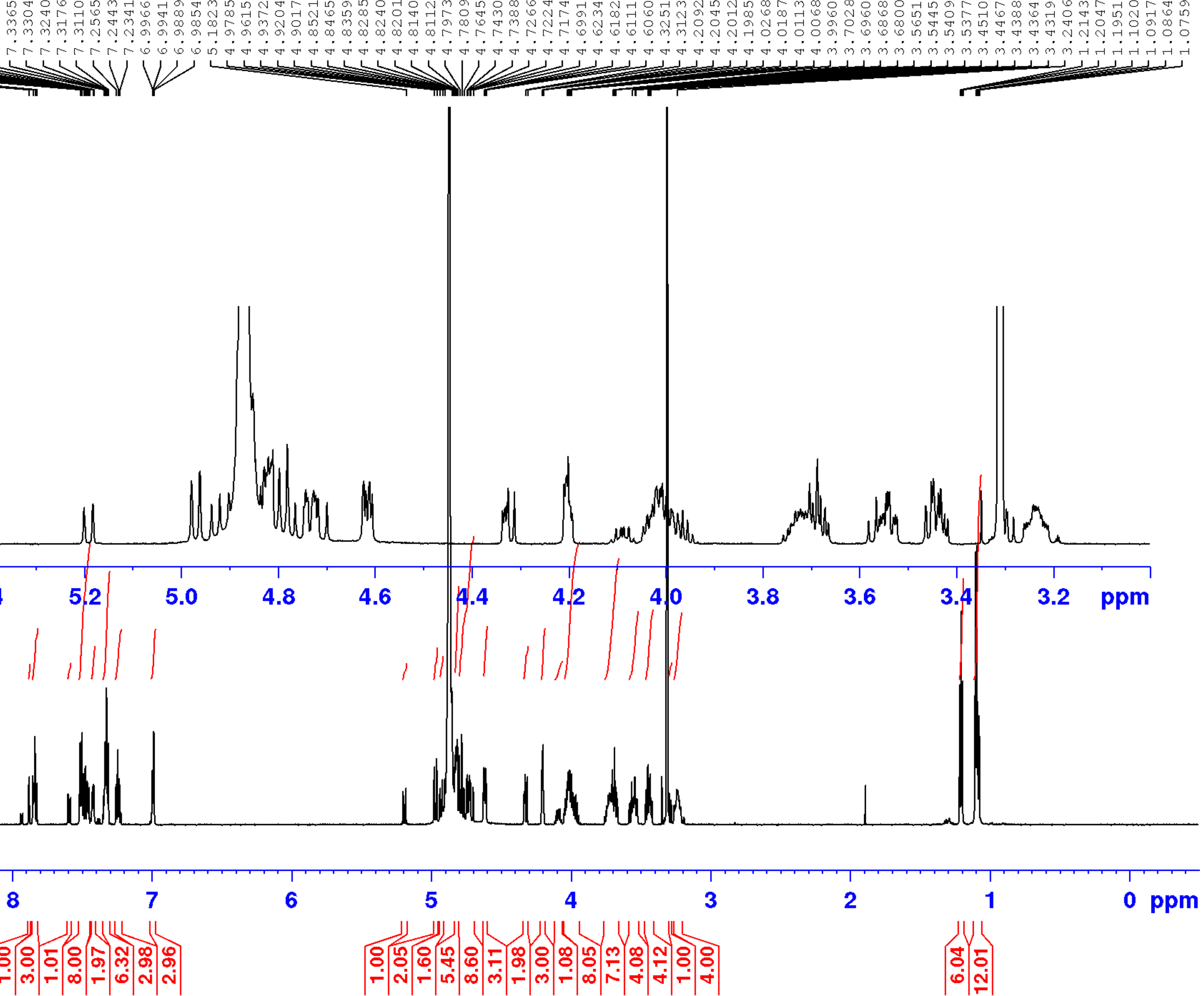

7.8801
7.8530
7.8373
7.8237
7.5148
7.5016
7.4889
7.4766
7.4664
7.4647
7.4577
7.4505
7.4490
7.4227
7.4170
7.3430
7.3365
7.3304
7.3240
7.3176
7.3110
7.2565
7.2443
7.2341
6.9966
6.9941
6.9889
6.9854
5.1823
4.9785
4.9615
4.9372
4.9204
4.9017
4.8521
4.8465
4.8359
4.8285
4.8240
4.8201
4.8140
4.8112
4.7973
4.7809
4.7645
4.7430
4.7388
4.7266
4.7224
4.7174
4.6991
4.6234
4.6182
4.6111
4.6060
4.3251
4.3123
4.2092
4.2045
4.2012
4.1985
4.0268
4.0187
4.0113
4.0068
3.9960
3.7028
3.6960
3.6868
3.6800
3.5651
3.5445
3.5409
3.5377
3.4510
3.4467
3.4388
3.4364
3.4319
3.2406
1.2143
1.2047
1.1951
1.1020
1.0917
1.0864
1.0759
5.4
5.2
5.0
4.8
4.6
4.4
4.2
4.0
3.8
3.6
3.4
3.2
ppm
9
8
7
6
5
4
3
2
1
0
ppm
1.00
3.00
1.01
8.00
1.97
6.32
2.98
2.96
1.00
2.05
1.60
5.45
8.60
3.11
1.98
3.00
1.08
8.05
7.13
4.08
4.12
1.00
4.00
6.04
12.01

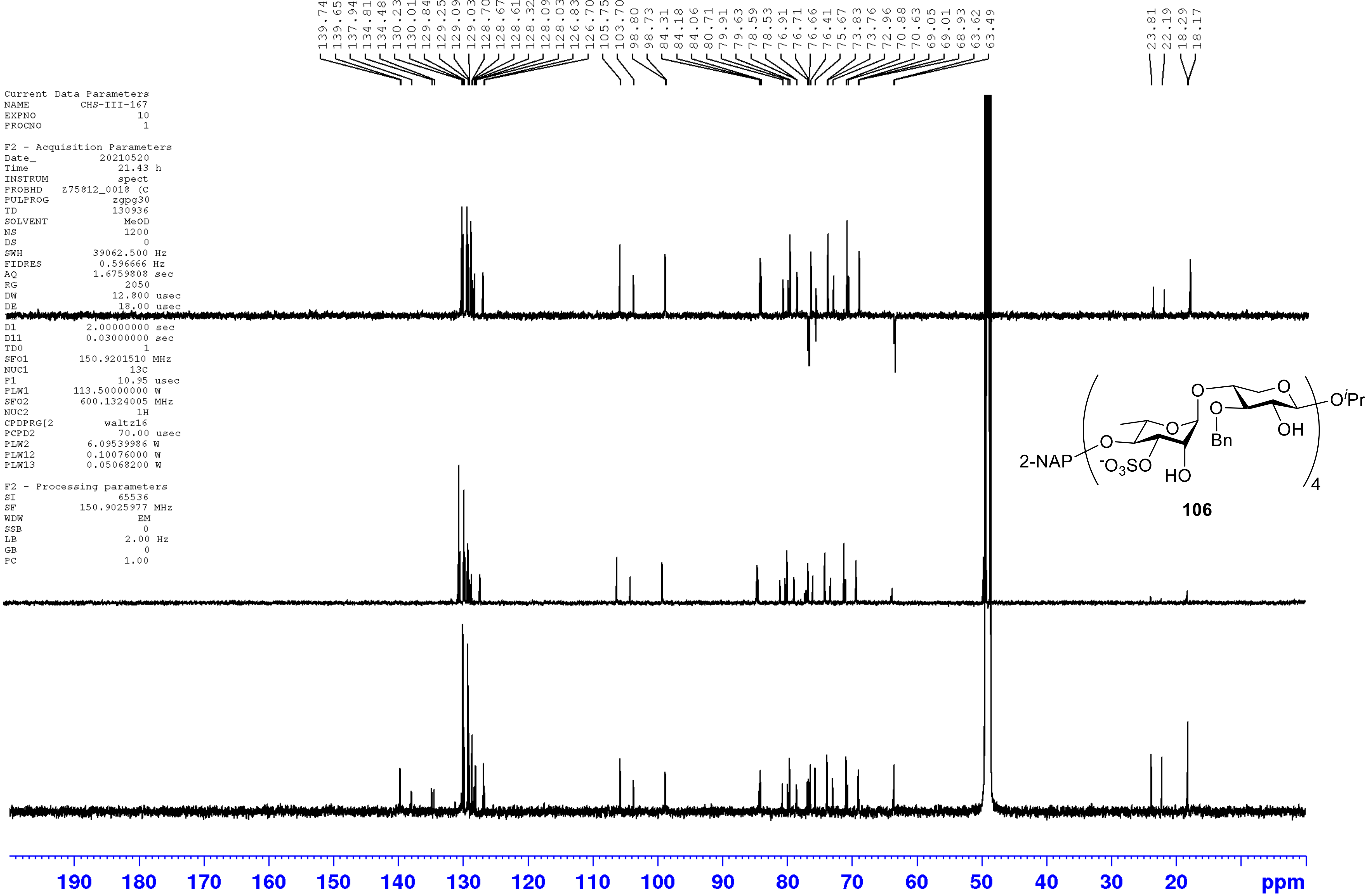
139.74
139.65
137.94
134.81
134.48
130.23
130.01
129.84
129.25
129.09
129.03
128.70
128.67
128.61
128.32
128.09
128.03
126.83
126.70
105.75
103.70
98.80
98.73
84.31
84.18
84.06
80.71
79.91
79.63
78.59
78.53
76.91
76.71
76.66
76.41
75.67
73.83
73.76
72.96
70.88
70.63
69.05
69.01
68.93
63.62
63.49
23.81
22.19
18.29
18.17
Current Data Parameters
NAME CHS-III-167
EXPNO 10
PROCNO 1
F2 - Acquisition Parameters
Date_ 20210520
Time 21.43 h
INSTRUM spect
PROBHD Z75812_0018 (C
PULPROG zgpg30
TD 130936
SOLVENT MeOD
NS 1200
DS 0
SWH 39062.500 Hz
FIDRES 0.596666 Hz
AQ 1.6759808 sec
RG 2050
DW 12.800 usec
DE 18.00 usec
D1 2.00000000 sec
D11 0.03000000 sec
TD0 1
SFO1 150.9201510 MHz
NUC1 13C
P1 10.95 usec
PLW1 113.50000000 W
SFO2 600.1324005 MHz
NUC2 1H
CPDPRG[2 waltz16
PCPD2 70.00 usec
PLW2 6.09539986 W
PLW12 0.10076000 W
PLW13 0.05068200 W
F2 - Processing parameters
SI 65536
SF 150.9025977 MHz
WDW EM
SSB 0
LB 2.00 Hz
GB 0
PC 1.00
2-NAP
O
-O3SO
HO
O
O
O
Bn
O
OH
O
OiPr
4
106
190 180 170 160 150 140 130 120 110 100 90 80 70 60 50 40 30 20 ppm

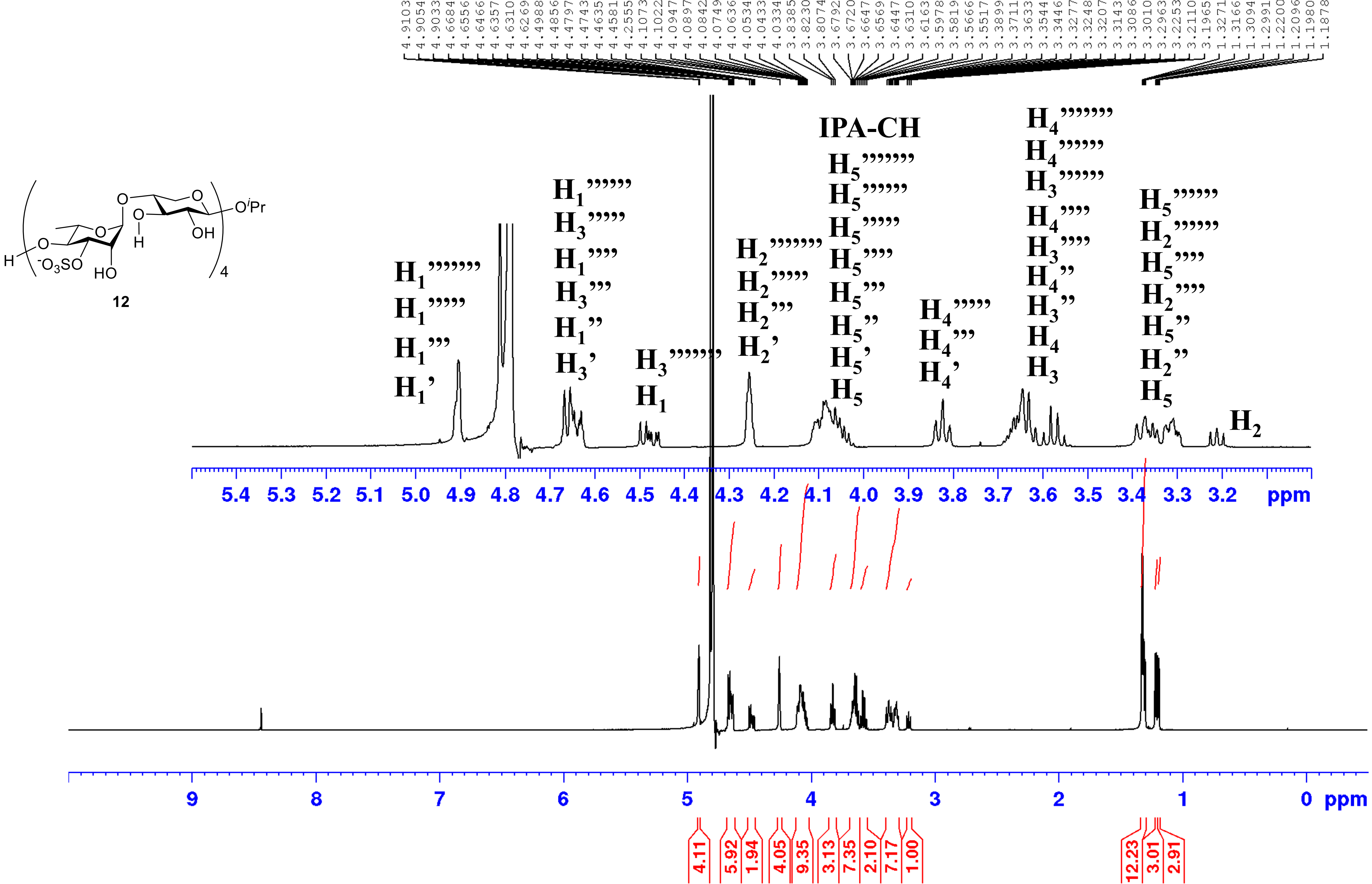
4.9103 4.9054 4.9033 4.6684 4.6556 4.6466 4.6357 4.6310 4.6269 4.4988 4.4856 4.4797 4.4743 4.4635 4.4581 4.2555 4.1073 4.1022 4.0947 4.0897 4.0842 4.0749 4.0636 4.0534 4.0433 4.0334 3.8385 3.8230 3.8074 3.6792 3.6720 3.6647 3.6569 3.6447 3.6310 3.6163 3.5978 3.5819 3.5666 3.5517 3.3899 3.3711 3.3633 3.3544 3.3446 3.3277 3.3248 3.3207 3.3143 3.3086 3.3010 3.2963 3.2253 3.2110 3.1965 1.3271 1.3166 1.3094 1.2991 1.2200 1.2096 1.1980 1.1878
O^iPr
OH
H
HO
-O3SO
4
12
H1''''''' H1''''' H1''' H1'
H1'''''' H3''''' H1'''' H3''' H1'' H3'
H3''''''' H1
H2''''''' H2''''' H2''' H2'
IPA-CH H5''''''' H5'''''' H5''''' H5'''' H5''' H5'' H5' H5
H4''''' H4''' H4'
H4''''''' H4'''''' H3'''''' H4'''' H3'''' H4'' H3'' H4 H3
H5'''''' H2'''''' H5'''' H2'''' H5'' H2'' H5
H2
ppm
4.11 5.92 1.94 4.05 9.35 3.13 7.35 2.10 7.17 1.00 12.23 3.01 2.91

Current Data Parameters
NAME CHS-III-180-2
EXPNO 6
PROCNO 1

F2 - Acquisition Parameters
Date_ 20210601
Time 20.44
INSTRUM spect
PROBHD 5 mm CPDCH 13C
PULPROG zgpg30
TD 131046
SOLVENT D2O
NS 1500
DS 0
SWH 39062.500 Hz
FIDRES 0.298082 Hz
AQ 1.6773888 sec
RG 912
DW 12.800 usec
DE 21.00 usec
TE 298.0 K
D1 2.00000000 sec
D11 0.03000000 sec
TD0 1

======== CHANNEL f1 ========
NUC1 13C
P1 11.00 usec
PL1 4.40 dB
PL1W 31.74709702 W
SFO1 150.9251877 MHz

======== CHANNEL f2 ========
CPDPRG[2 waltz16
NUC2 1H
PCPD2 80.00 usec
PL2 -1.10 dB
PL12 16.20 dB
PL13 19.20 dB
PL2W 16.60035515 W
PL12W 0.30911303 W
PL13W 0.15492350 W
SFO2 600.1524006 MHz

F2 - Processing parameters
SI 65536
SF 150.9078380 MHz
WDW EM
SSB 0
LB 2.00 Hz
GB 0
PC 1.00

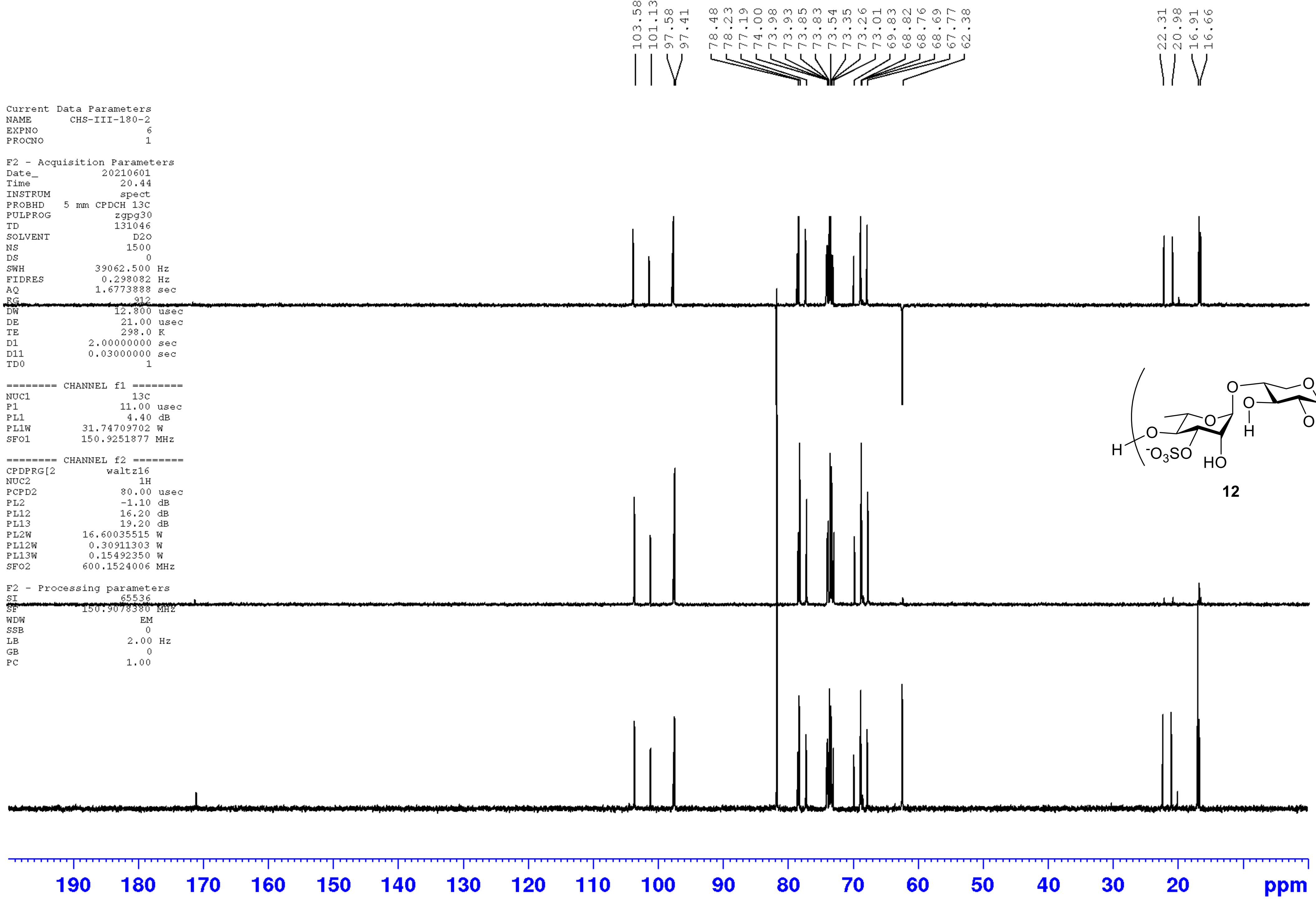

12

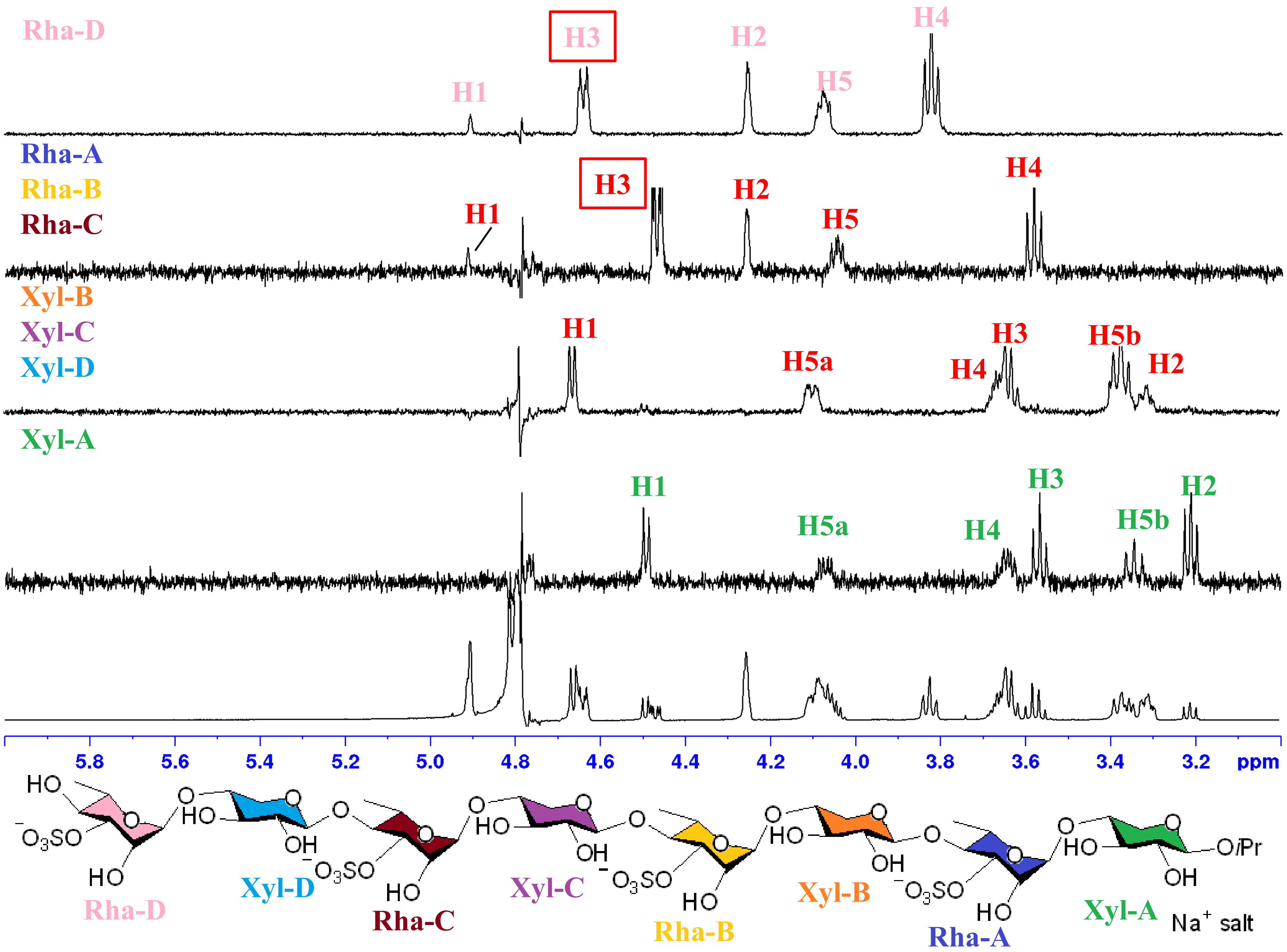
Rha-D
H1
H3
H2
H5
H4
Rha-A
Rha-B
Rha-C
H1
H3
H2
H5
H4
Xyl-B
Xyl-C
Xyl-D
H1
H5a
H4
H3
H5b
H2
Xyl-A
H1
H5a
H4
H3
H5b
H2
5.8
5.6
5.4
5.2
5.0
4.8
4.6
4.4
4.2
4.0
3.8
3.6
3.4
3.2
ppm
Rha-D
Xyl-D
Rha-C
Xyl-C
Rha-B
Xyl-B
Rha-A
Xyl-A
Na+ salt

12
